# Effects of *feruloyl-CoA 6′-hydroxylase 1* overexpression on lignin and cell wall characteristics in transgenic hybrid aspen

**DOI:** 10.3389/fpls.2025.1543168

**Published:** 2025-03-28

**Authors:** Naning Wang, Masatsugu Takada, Shingo Sakamoto, Ruben Vanholme, Geert Goeminne, Hoon Kim, Soichiro Nagano, Naoki Takata, Naofumi Kamimura, Mikiko Uesugi, Akiko Izumi-Nakagawa, Eiji Masai, Nobutaka Mitsuda, Wout Boerjan, John Ralph, Shinya Kajita

**Affiliations:** ^1^ Graduate School of Bio-Applications and Systems Engineering, Tokyo University of Agriculture and Technology, Koganei, Tokyo, Japan; ^2^ Bioproduction Research Institute, National Institute of Advanced Industrial Science and Technology (AIST), Tsukuba, Ibaraki, Japan; ^3^ Global Zero Emission Research Center, National Institute of Advanced Industrial Science and Technology (AIST), Tsukuba, Ibaraki, Japan; ^4^ Department of Plant Biotechnology and Bioinformatics, Ghent University, Ghent, Belgium; ^5^ VIB Center for Plant Systems Biology, Ghent, Belgium; ^6^ VIB Metabolomics Core Ghent, VIB, Ghent, Belgium; ^7^ United States (US) Department of Energy, Great Lakes Bioenergy Research Center, and The Department of Biochemistry, Wisconsin Energy Institute, Madison, WI, United States; ^8^ Forest Tree Breeding Center, Forestry and Forest Products Research Institute, Forest Research and Management Organization, Hitachi, Ibaraki, Japan; ^9^ Forest Bio-Research Center, Forestry and Forest Products Research Institute, Forest Research and Management Organization, Hitachi, Ibaraki, Japan; ^10^ Department of Materials Science and Bioengineering, Nagaoka University of Technology, Nagaoka, Niigata, Japan; ^11^ Graduate School of Science and Technology, University of Tsukuba, Tsukuba, Ibaraki, Japan

**Keywords:** *Arabidopsis thaliana*, coumarin, fluorescence, saccharification efficiency, scopoletin

## Abstract

In plant cell walls, lignin, cellulose, and the hemicelluloses form intricate three-dimensional structures. Owing to its complexity, lignin often acts as a bottleneck for the efficient utilization of polysaccharide components as biochemicals and functional materials. A promising approach to mitigate and/or overcome lignin recalcitrance is the qualitative and quantitative modification of lignin by genetic engineering. Feruloyl-CoA 6′-hydroxylase (F6′H1) is a 2-oxoglutarate-dependent dioxygenase that catalyzes the conversion of feruloyl-CoA, one of the intermediates of the lignin biosynthetic pathway, into 6′-hydroxyferuloyl-CoA, the precursor of scopoletin (7-hydroxy-6-methoxycoumarin). In a previous study with *Arabidopsis thaliana*, we demonstrated that overexpression of *F6′H1* under a xylem-preferential promoter led to scopoletin incorporation into the cell wall. This altered the chemical structure of lignin without affecting lignin content or saccharification efficiency. In the present study, the same *F6′H1* construct was introduced into hybrid aspen (*Populus tremula × tremuloides* T89), a model woody plant, and its effects on plant morphology, lignin chemical structure, global gene expression, and phenolic metabolism were examined. The transgenic plants successfully overproduced scopoletin while exhibiting severe growth retardation, a phenotype not previously observed in *Arabidopsis*. Scopoletin accumulation was most pronounced in the secondary walls of tracheary elements and the compound middle lamella, with low levels in the fiber cell walls. Overexpression of *F6′H1* also affected the metabolism of aromatics, including lignin precursors. Heteronuclear single-quantum coherence (HSQC) NMR spectroscopy revealed that scopoletin in cell walls was bound to lignin, leading to a reduction in lignin content and changes in its monomeric composition and molar mass distribution. Furthermore, the enzymatic saccharification efficiency of the transgenic cell walls was more than three times higher than that of the wild-type plants, even without pretreatment. Although addressing growth inhibition remains a priority, incorporating scopoletin into lignin demonstrates significant potential for improving woody biomass utilization.

## 1 Introduction

The increasing urgency of global warming and depletion of fossil fuels have drawn substantial attention toward renewable lignocellulosic resources (Adewuyi, 2022). Lignocellulose is composed of cellulose, hemicelluloses, and lignin, which form an intricate and complex three-dimensional structure. Polysaccharides (cellulose and hemicelluloses) are commercially used in various chemicals and raw materials. For instance, polysaccharide-derived glucose and xylose can be converted into biofuels, chemicals, and food additives (Ning et al., 2021). However, owing to its complex structure and recalcitrant nature, lignin often acts as a bottleneck for efficient conversion of polysaccharides into primary sugars (Jatoi et al., 2023).

To achieve effective deconstruction, most lignocellulosic substrates require pretreatments such as hydrothermal, chemical, or chemo-thermomechanical treatments to loosen the cell wall structure and recover polysaccharides and subsequent monosaccharides (Takada et al., 2020; Yu et al., 2022). However, these processes require high energy and/or chemical consumption under harsh conditions, leading to excessive polysaccharide degradation and the formation of undesirable byproducts such as furfural, 5-hydroxymethylfurfural, and organic acids (Jönsson et al., 2013; Galbe and Wallberg, 2019).

An alternative strategy for overcoming this bottleneck in bioconversion is the qualitative and quantitative modification of *in planta* lignin via genetic engineering. Lignin is formed primarily by the oxidative coupling of three monolignols—*p*-coumaryl, coniferyl, and sinapyl alcohols (Ralph et al., 2004), which are produced via the general phenylpropanoid (cinnamate/monolignol) pathway (Boerjan et al., 2003; Umezawa, 2018). Upon polymerization (lignification) these alcohols form *p*-hydroxyphenyl (H), guaiacyl (G), and syringyl (S) units in lignin. The overexpression or suppression of genes involved in this pathway can reduce lignin content and modify its chemical structure, thereby making the cell walls more chemically and/or biochemically labile (Jackson et al., 2008; Li et al., 2010; Van Acker et al., 2013).

In addition to the artificial regulation of endogenous gene expression, the expression of heterologous genes is also effective in modifying lignin by bypassing the pathway to the canonical monolignols. Various monolignol precursors can serve as branching points for the bypass, among which *p*-hydroxycinnamoyl-CoA esters, notably *p*-coumaroyl- and feruloyl-CoAs, are promising targets. In transgenic *Arabidopsis* plants, two curcumin biosynthetic genes originating from turmeric for the bioconversion of feruloyl-CoA to curcumin, have been expressed (Oyarce et al., 2019). The accumulated curcumin in the plants was incorporated into lignin, improving the degradability of lignin and the saccharification efficiency of cell wall polysaccharides. Similarly, Mahon et al. (2022) generated transgenic poplars with the apple gene for chalcone synthase 3, which uses *p*-coumaroyl-CoA as a substrate to form naringenin chalcone. The transgenic poplars accumulated naringenin, modifying the lignin and again enhancing saccharification efficiency. The heterologous production of bacterial hydroxycinnamoyl-CoA reductase (HCHL), which can convert both *p*-coumaroyl- and feruloyl-CoAs to the corresponding C6-C1 benzaldehydes, reduced the molar mass of lignin while maintaining its content in transgenic *A. thaliana* (Eudes et al., 2012). Similarly to the two examples mentioned earlier, *HCHL* expression also enhanced the saccharification efficiency of the cell wall biomass. These results indicate that lignin modification via CoA ester bioconversion has potential to increase the availability of lignocellulose.

In a previous study, we overexpressed a variety of genes encoding enzymes capable of bypassing the lignin biosynthetic pathway in *A. thaliana*, under the control of a xylem-preferential promoter (Sakamoto et al., 2020). Among these genes, overexpression of *FERULOYL-CoA 6′-HYDROXYLASE 1* (*F6′H1*) led to the accumulation of scopoletin in the cell wall without significantly affecting lignin content or plant growth. The F6′H1 enzyme, a 2-oxoglutarate-dependent dioxygenase, catalyzes the conversion of feruloyl-CoA, one of the intermediates of the lignin biosynthetic pathway, to 6′-hydroxyferuloyl-CoA. The latter compound undergoes spontaneous reactions (isomerization and lactonization) to form scopoletin (He et al., 2022). Recently, Hoengenaert et al. (2022) demonstrated that simultaneous overexpression of *F6′H1* and coumarin synthase (*COSY*) genes in *A. thaliana* significantly increased scopoletin production, its incorporation into lignin, and the saccharification efficiency of cell walls following alkaline pretreatment. In this study, we examined the effects of *F6′H1* expression in hybrid aspen (*Populus tremula × tremuloides* T89), using the same gene construct as in our previous study in *A. thaliana* (Sakamoto et al., 2020). The transgenic plants were analyzed using metabolomic and transcriptomic approaches in addition to various spectroscopic and wet chemical analyses of lignin and polysaccharides in the cell wall.

## 2 Materials and methods

### 2.1 Genetic transformation of hybrid aspen

Genetic transformation was performed with the wild-type (WT) hybrid aspen (*Populus tremula* × *tremuloides* T89) as described previously (Sakamoto et al., 2020). A gene construct containing the *F6'H1* coding sequence under the control of the poplar cinnamate 4-hydroxylase (*C4H*) promoter was introduced into *Agrobacterium tumefaciens* strain GV3101 and the transformed bacterium was inoculated onto hybrid aspen explants (Sakamoto et al., 2016). Transgenic calli were selected on kanamycin-containing medium and the regenerated transgenic plants were propagated and maintained under sterile conditions on a modified Murashige and Skoog (MS) medium. To prepare wood powder and thin sections, the plants were cultured in MS medium for 4 weeks before being transferred to pots containing soil. The plants were grown for over 10 weeks at 25°C under a 16/8 h light/dark cycle with fluorescent lighting in a climate-controlled room. After 13 weeks of growth, the plants were harvested. Stem segments were stored in fixing solution (50% ethanol, 10% formaldehyde, and 5% acetic acid in water) for future sectioning. The remaining stems were debarked, air-dried in the dark, and stored for further analysis.

### 2.2 Reverse-transcription polymerase chain reaction analysis

Approximately 50 mg of non-debarked stem tissue from harvested *in vitro*-cultured plants was frozen in liquid nitrogen and crushed into powder. TRIzol (Thermo Fisher Scientific, Waltham, MA, USA) was added to lyse the cells, total RNA was extracted, and first-strand cDNA was synthesized by reverse transcription and subjected to PCR. PCR products were subjected to agarose gel electrophoresis and stained with ethidium bromide. Gene expression was quantified based on the fluorescence intensity of the amplified DNA. Amplified DNA from transcripts of *ubiquitin* was used as a control.

### 2.3 Detection of cell-wall-bound scopoletin in sterile cultured plants using pyrolysis gas chromatography-mass spectrometry

To select transgenic plants that accumulated scopoletin, extractive-free cell walls were analyzed using Py-GC/MS (Sakamoto et al., 2020). The stems of the plants that grew in the culture media were washed with methanol to remove methanol-soluble metabolites until the color of the stem became white. The extractive-free stems were pulverized using a Multi-beads Shocker (MB1200C; Yasui Kikai Corporation, Osaka, Japan). In brief, the samples were thermally cleaved using an EGA/PY-3030D multi-shot pyrolyzer (Frontier Laboratories Inc., Fukushima, Japan) at 500°C for 1 s. The samples were then separated and analyzed on a GC-MS system (GCMS-QP2020; Shimadzu Inc., Kyoto, Japan). The inlet temperature was set at 270°C and the interface temperature at 250°C. A Frontier Ultra ALLOY Capillary Column (30 m × 0.25 mm i.d., 0.25 µm) was used for chromatography. The programmed heating conditions were an initial temperature of 70°C for 2 min, followed by heating to 270°C at a rate of 6°C/min and held at 270°C for 10 min. Helium was used as the carrier gas and mass spectrometric detection was performed using electron ionization at 70 eV.

### 2.4 Metabolomic analyses

Plants grown under sterile culture conditions were transferred to pots containing soil and cultivated for 9–10 weeks under natural light in a conditioned greenhouse. The xylem with pith part from the plants were harvested and immediately frozen in liquid nitrogen for preservation. Subsequently, the samples were stored at -80°C until further processing. For metabolomic analysis, frozen stems were pulverized using a Multi-beads Shocker. Approximately 100 mg (19–30 mg dry weight) of frozen stem powder was mixed with 1 mL of methanol. The mixture was then shaken at 70°C for 15 min at 15,000 rpm (Thermo-Shaker MS-100; Hangzhou Allsheng Instruments Co., Ltd., Edison, Zhejiang, China). Subsequently, the sample was centrifuged at 15,000 rpm for 3 min. The methanol supernatant was transferred to a new microcentrifuge tube and freeze-dried. The precipitate remaining at the bottom of each tube was weighed after drying to normalize metabolite detection.

The dried methanol extracts were redissolved in 1 mL methanol. An 800 µL aliquot of the methanol extract was passed through a reversed phase SPE cartridge (Oasis HLB 96-well plate, 60 mg sorbent, 60 µm particle size; Waters), and the eluate was collected in a new tube. The cartridge was then washed twice with 200 µL methanol that was combined with the initial eluate. Next, the methanol extracts were evaporated to dryness under vacuum conditions, dissolved in 100 µL Milli-Q water, and subsequently filtered under centrifugation force (Pall 8019 AcroPrep advance 96-well filter plate, 350 µL, 0.2 µm supor membrane; Tisch Scientific). Samples were subjected to ultra-performance liquid chromatography coupled with high-resolution mass spectrometry (UPLC-HRMS) at the VIB Metabolomics Core Ghent, essentially as in Van Acker et al. (2017) with minor modifications specified in the **Supplementary Material**.

### 2.5 Microscopic observations

For optical microscopy, 20-µm-thick stem cross-sections were prepared using a microtome (MTH-1; Nippon Medical & Chemical Instruments, Osaka, Japan), stained with phloroglucinol-HCl, and immediately observed under a microscope (BZ-X810; Keyence Corporation, Osaka, Japan). For scanning electron microscopy (SEM), stem sections were freeze-dried, gold-coated, and examined using an SEM (S-4500; Hitachi Ltd., Tokyo, Japan) at an accelerating voltage of 5 kV. Ultraviolet microspectrophotometry was performed by embedding the stem sections in epoxy resin, cutting them into 0.5 µm slices, and measuring absorbance at a wavelength of 280 ± 5 nm using an MSP-800 microspectrophotometer (Carl Zeiss, Oberkochen, Germany). UV spectra at each morphological region were analyzed via UV microspectrophotometry using point-by-point photometric measurements (measurement spot size: 1 × 1 μm^2^). Fluorescence microscopy was performed using an optical microscope (BZ-X810n, KEYENCE Corporation, Osaka, Japan) at an excitation wavelength of 360 nm.

### 2.6 Lignin quantification and structural analysis

Debarked stems were ground using a milling machine (Multi-beads Shocker MB1200C; Yasui Kikai, Osaka, Japan) and sieved to obtain particles smaller than 150 µm. To prepare extractive-free cell walls, the milled powder was soaked in 50 mM NaCl overnight and washed three times with water. Then they were extracted with methanol, acetone, ethanol (twice), ethanol/toluene (1:2, v/v, twice), and acetone by ultrasonic extraction for 30 min each, followed by air drying at room temperature. The lignin content was determined using the acetyl bromide method (Yamamura et al., 2012).

Lignin structural analysis was conducted using previously described methods. Briefly, enzyme lignins (ELs) were prepared and analyzed using ^1^H-^13^C 2D heteronuclear single-quantum coherence (HSQC) NMR spectroscopy with dimethyl sulfoxide (DMSO)-*d_6_*/pyridine-*d_5_* (4:1, v/v) as the solvent, as previously described (Kim and Ralph, 2010; Mansfield et al., 2012). The analysis was performed using a Bruker Avance NEO 700 MHz spectrometer (Bruker Biospin, Billerica, MA) equipped with a 5-mm QCI ^1^H/^31^P/^13^C/^15^N cryoprobe (Kim and Ralph, 2010). An adiabatic HSQC experiment was used to collect the data (Kupce and Freeman, 2007). Thioacidolysis monomer analysis was conducted following the method of Yamamura et al. (2012), based on the original method (Lapierre, 1993). Briefly, extractive-free wood powder (approximately 7 mg) was heated at 100°C in a mixture of 1,4-dioxane, ethanethiol, and boron trifluoride diethyl etherate, and analyzed by GC equipped with a flame-ionization detector (see **Supplementary Materials and Methods**).

For Py-GC/MS analysis, samples prepared from *in vitro*-cultured plants were pyrolyzed at 500°C for 4 s using a JHP-5 Pyrolyzer (Japan Analytical Industry Co., Ltd., Tokyo, Japan) and analyzed using a GCMS-QP5050A GC-MS/MS with a DB-5MS (30 m × 0.25 mm × 0.5 µm) column. Size exclusion chromatography (SEC) analysis was performed using an HPLC system with two Agilent Polar Gel M columns and a guard column (Agilent Technologies, Santa Clara, CA, USA). The mobile phase consisted of 10 mM LiBr/DMSO, and the detection wavelength was 280 nm. The detected compounds were identified as previously described (Ralph and Hatfield, 1991; Nakagawa-Izumi et al., 2017).

### 2.7 RNA sequencing

Total RNA was extracted and assessed using an Agilent 5400 Fragment Analyzer System (Agilent Technologies) to confirm that the RNA integrity number (RIN) exceeded 6.7. Subsequently, mRNA-seq libraries were prepared using the NEBNext^®^ Ultra™ Directional RNA Library Prep Kit for Illumina (New England Biolabs, Ipswich, MA, USA). Sequencing was performed with 150-bp paired-end reads on a DNBSEQ-T7 platform (MGI Tech Co., Ltd., Shenzhen, China). The sequence data were deposited in the DDBJ Sequence Read Archive (DRA) under accession number PRJDB19630. The raw sequencing reads were trimmed with EARRINGS [version 1.0.3; (Wang et al., 2021)] using the following parameters: –sensitive -a AGATCGGAAGAGCGTCGTGTAGGGAAAGAGTGTAGATCTCGGTGGTCGCCGTATCATT, and -A GATCGGAAGAGCACACGTCTGAACTCCAGTCACGGATGACTATCTCGTATGCCGTCTTCTGCTTG. Trimmed reads were mapped to the *Populus tremula* version 2.2 reference genome (Potra02_genome.fasta) using HISAT2 [version 2.2.1; (Kim et al., 2015)] with the “no-discordant” and “no-mixed” options. The resulting Sequence Alignment Map was converted into Binary Alignment Map files with SAMtools (version 0.3.3; (Li et al., 2009)). Aligned reads were quantified using FeatureCounts [version 2.0.2; (Liao et al., 2014)] based on the Potra02_genes.gff.gz annotation file. Raw counts were processed using R software (version 4.4.0). DESeq2 [version 1.24.0; (Love et al., 2014)] was employed to identify differentially expressed genes (DEGs) between the WT and the transgenic lines. The number of DEGs, average counts per million reads, fold-change (FC), and Benjamin–Hochberg-adjusted *p*-value (Padj) were recorded. Additionally, trimmed reads were pseudo-aligned to the introduced *F6'H1* sequence using Kallisto (Bray et al., 2016). Counts were normalized by the total number of trimmed reads to obtain counts per million reads.

### 2.8 Enzymatic saccharification

The saccharification assay was performed as described previously (Hu et al., 2022). Briefly, extractive-free wood powder was mixed with 62.5 mM NaOH and autoclaved at 120°C for 1 h. The sample was then subjected to enzymatic hydrolysis at 50°C for 24 h using cellobiase (C6105; Sigma-Aldrich, St. Louis, MO, USA) and Celluclast^®^ (C2730; Sigma-Aldrich). The amount of sugar released was quantified using the 3,5-dinitrosalicylic acid (DNS) method (Miller, 1959). Glucose was used for preparation of calibration curve. Saccharification efficiency was calculated based on the amounts of the released sugars (mainly glucose) per cell wall residue without any pretreatment.

## 3 Results

### 3.1 Overexpression of *F6′H1* in hybrid aspen

**Figure 1:**
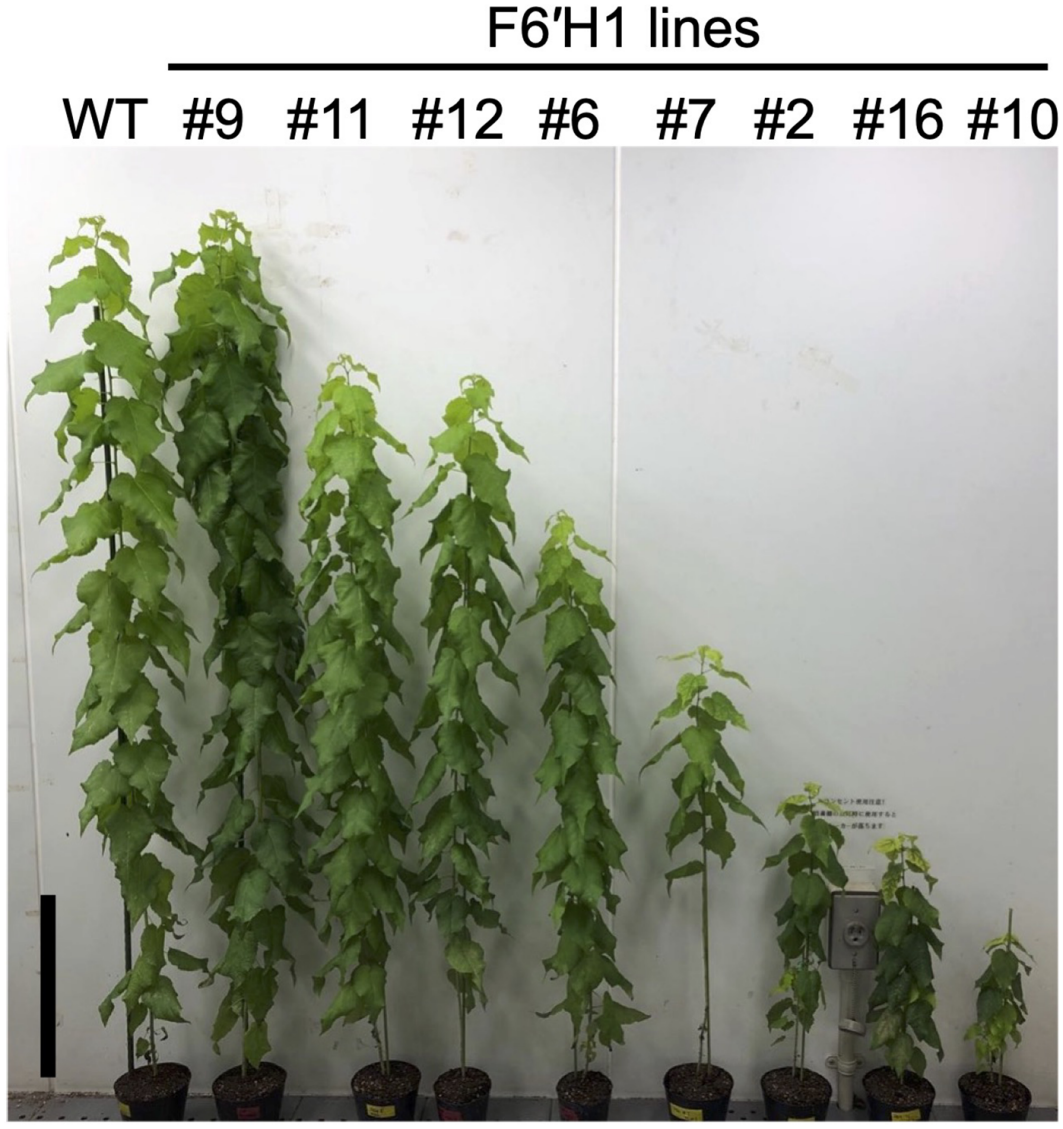
Phenotypic representation of WT and transgenic hybrid aspen overexpressing *F6*′*H1* (15 weeks old). Black bar in represents 20 cm.

Nine independent transgenic lines were generated and maintained on modified MS medium under sterile conditions. Two of the nine lines (#10 and #16) had difficulty rooting compared with the others on the medium. RT-PCR analysis of RNA extracted from the sterile plants confirmed that the *F6'H1* gene was expressed in all transgenic lines (**Supplementary Figure 1**). After transferring the plants to pots with soil, line #9 exhibited normal growth, while the other lines showed significantly shorter heights than the WT plants (**Figure 1**, line #13 is not pictured). As shown in **Supplementary Figure 2**, the debarked stems of the transgenic lines, with the exception of line #9, had smaller diameters than that of the WT. Additionally, the debarked stems of the transgenic lines immediately after peeling the bark were brown or pale brown, in contrast to the whitish-yellow color of the WT stems (**Supplementary Figure 2**)

### 3.2 Scopoletin accumulation in the cell wall of transgenic lines

To confirm scopoletin accumulation in the cell wall and select lines for further in-depth analysis, extractive-free powders from juvenile plants cultured under sterile conditions were analyzed by Py-GC/MS. The scopoletin accumulation in the cell wall was quantified and expressed using a scopoletin index, which represents the ratio of scopoletin (peak area in the chromatograms) to coniferyl alcohol detected in each line (**Supplementary Figure 3**). Scopoletin was clearly detected in all lines except line #9, with varying levels of accumulation across the transgenic lines. Based on scopoletin levels, the transgenic lines were categorized into three groups: low (#9, #12, and #13), medium (#6, #7, and #11), and high (#10 and #16) production lines (**Supplementary Figure 3**). The last two lines, exhibiting severe dwarfism (**Figure 1**), were excluded from most of the subsequent analyses.

### 3.3 Histochemical analysis of the transgenic lines

**Figure 2:**
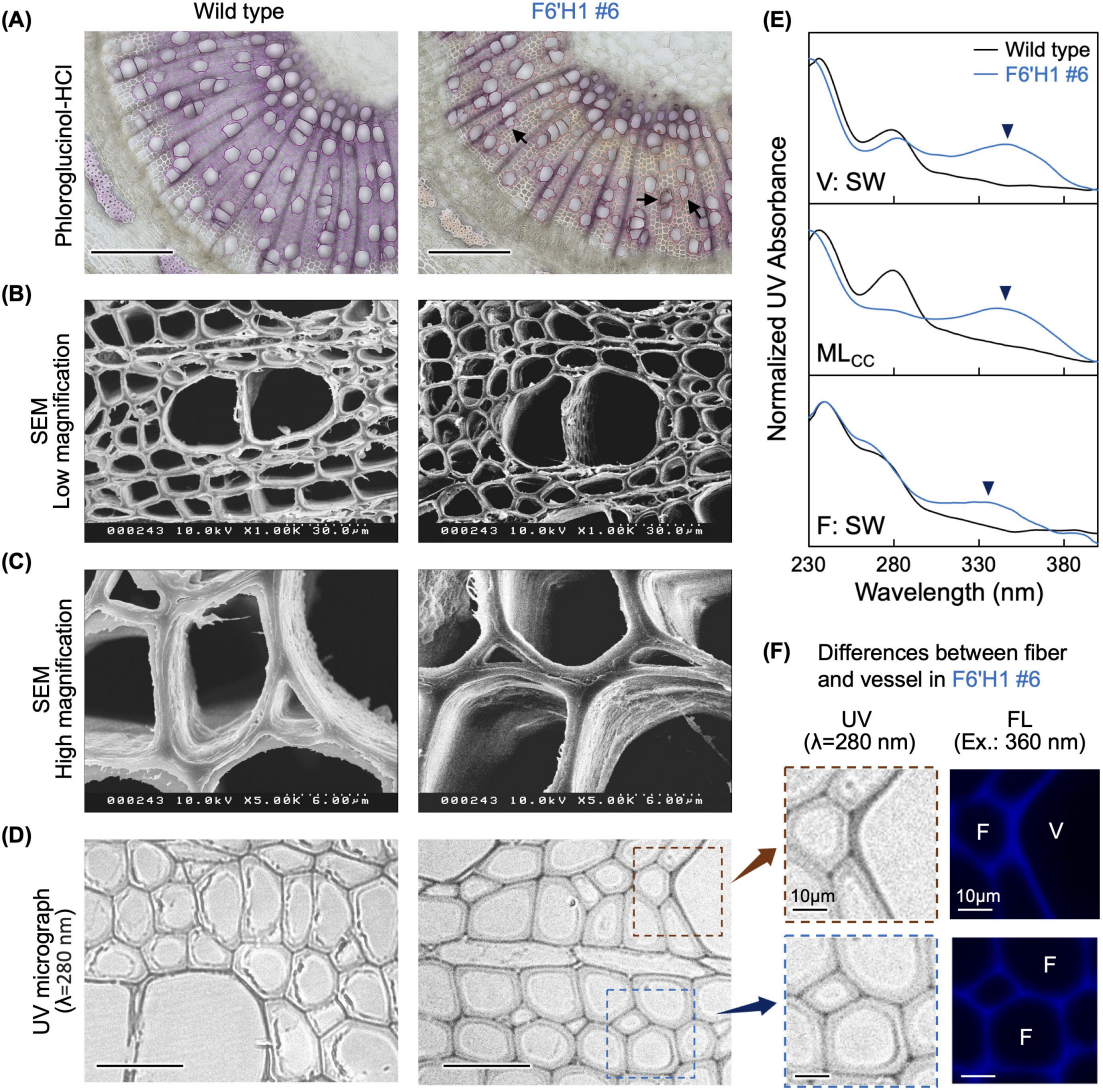
Histochemical analysis of stem sections. **(A)** Phloroglucinol-HCl staining. **(B)** Scanning electron microscopy images of stem sections. **(C)** High magnification views. **(D)** Stem thin sections under an ultraviolet (UV) microscope. **(E)** Normalized UV absorbance of cell walls in secondary wall of vessel (V:SW), middle lamella of cell corners (MLcc), and secondary cell wall of fiber (F:SW). **(F)** High magnification views of thin sections prepared from transgenic line #6 (left panels) and autofluorescence of the same regions (right panels). Black bars in **(A)** and **(D)** represent 200 µm and 50 µm, respectively.

To examine the histochemical characteristics of the transgenic lines, transverse thin sections of stems from each line grown in soil were stained with phloroglucinol-HCl, which produces a red color when reacting with the cinnamaldehyde end-groups in lignin. **Figure 2A** shows representative results of the analysis. The sections prepared from the WT plant exhibited a typical reddish-purple color after the reaction. In contrast, the section from the F6′H1 transgenic line (#6) was clearly lighter in color, particularly in the fibers of xylem and phloem tissues. Although cross-sections of most WT vessels had smooth contours, many of those in the transgenic plants were distorted in shape (arrows in **Figure 2A**). This shape anomaly was more pronounced in the vessels of the secondary xylem tissue than in those of the primary xylem tissue.

To examine the anatomical structure in more detail, the cell walls in secondary xylem tissue were observed under a transmission microscope (**Figures 2B, C**). No significant differences were observed in the shape of the secondary cell walls or their layered structures between the WT and line #6. Unlike the staining analysis with phloroglucinol-HCl, SEM did not reveal any abnormally shaped vessels. This suggests that the shape anomaly of the vessels in the transgenic line may have occurred during sectioning and staining of the fresh stem samples.

### 3.4 Scopoletin distribution in cell walls

The distribution of lignin and scopoletin within the cell walls was examined using a UV microscope. Under UV light at 280 nm, no significant difference in the appearance of xylem cell walls was observed between the WT and line #6 (**Figure 2D**). Absorption spectra of distinct parts of xylem cells (i.e., secondary wall of vessels and fibers, and the middle lamella at the cell corner region) were also analyzed and data after normalization are shown in **Figure 2E**. For the WT, a gentle shoulder peak was observed at 280 nm in all three regions analyzed. Conversely, an additional absorbance peak at 340 nm appeared in the F6′H1 line. This additional peak was especially pronounced in the vessel secondary wall and middle lamella of the cell corner. Given that scopoletin has a recorded absorbance at 340 nm, this peak was attributed to scopoletin bound to the cell walls. Although all three regions clearly absorbed at 340 nm, the fiber cell wall showed a lower absorbance compared with that of the others, suggesting that scopoletin was heterogeneously distributed within the cell walls of line #6.

Given that scopoletin can be excited under irradiation at 360 nm, its distribution was examined using fluorescence microscopy (Patel et al., 2021). In contrast to the weak fluorescence in the WT (**Supplementary Figure 4**), strong fluorescence was detected in the F6′H1 line under the same conditions. Fluorescence micrographs with the F6′H1 line indicate that scopoletin accumulated in the cell walls of both fibers and vessels (**Figure 2F**). Compared with the secondary cell wall of the fibers, the fluorescence intensity appeared relatively stronger in the secondary wall of vessels and middle lamella, including the cell corner region (arrow and arrowhead in **Supplementary Figure 4**). This heterogeneous distribution of scopoletin, as revealed by fluorescence, aligns with the observations made using UV microscopy.

### 3.5 Chemical compositional analysis

**Figure 3:**
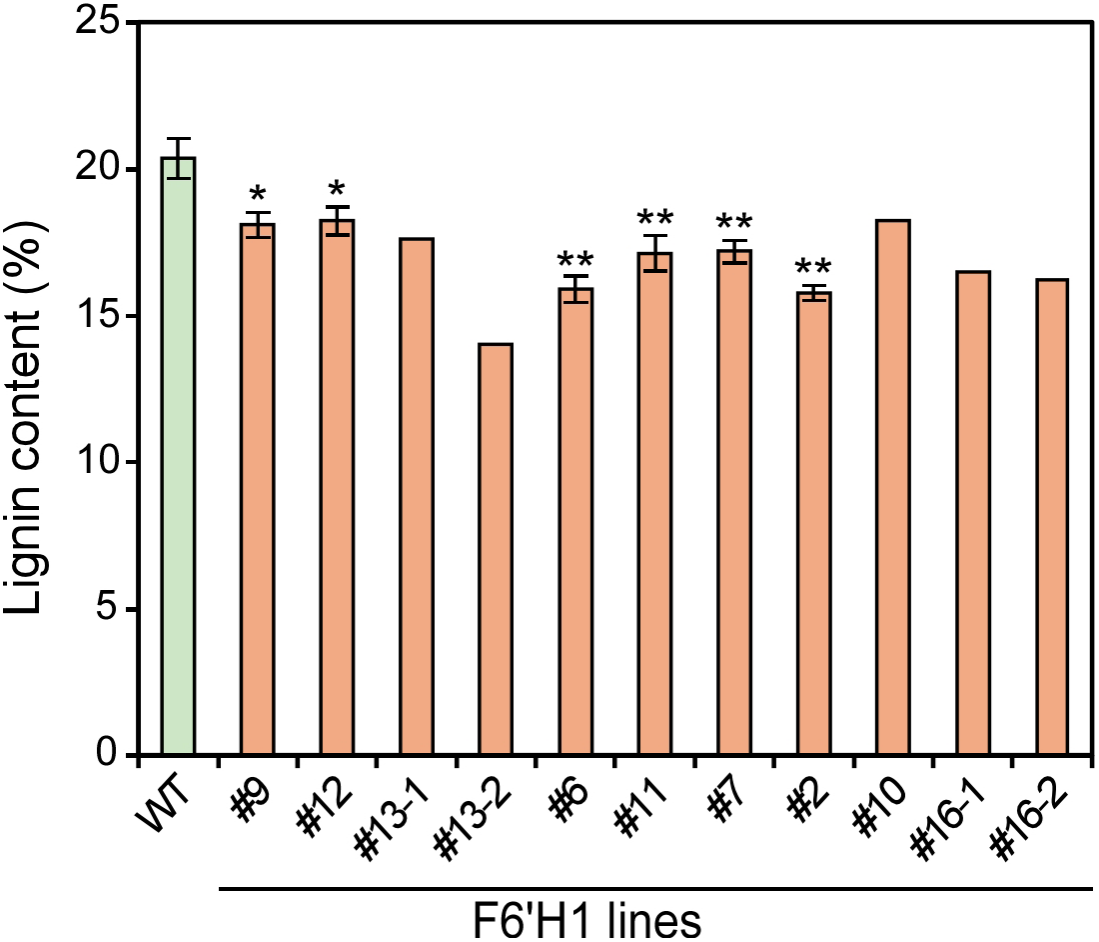
Lignin content in cell wall residue prepared from stems of F6′H1 transgenic and wild-type (WT) plants. Values represent means with standard deviation from three to four independent biological replicates, except for line #10 (n =1), #13 (n = 2), and #16 (n =2). Asterisks indicate significant differences from the WT using the Student’s *t*-test (*0.01 < *p* < 0.05; **p <0.01).

The effect of scopoletin accumulation on the chemical composition of cell walls was evaluated using extractive-free wood powder. The lignin content, determined using the acetyl bromide method, is shown in **Figure 3**. The lignin content in the F6′H1 lines (16.3–18.1%) was significantly lower than that in the WT (20.4%). This decrease in lignin content was not observed in our previous study on *F6'H1*-expressing *A. thaliana* plants (Sakamoto et al., 2020).

In addition to lignin content, α-cellulose and hemicellulose contents were determined using wood powders prepared from the WT and two representative *F6′H1*-expressing lines (#6 and #12). The lines highly accumulating scopoletin were excluded from the analysis owing to their limited growth. No significant differences in α-cellulose or hemicellulose contents were observed between the WT and F6′H1 lines (**Supplementary Figure 5A**). Similarly, the monosaccharide composition was nearly identical between the WT and F6′H1 lines (**Supplementary Figure 5B**). These findings indicate that scopoletin accumulation does not significantly affect polysaccharide biosynthesis, although expression of xylan biosynthetic genes was suppressed, as discussed later.

### 3.6 Effect of scopoletin incorporation on lignin structure

**Figure 4:**
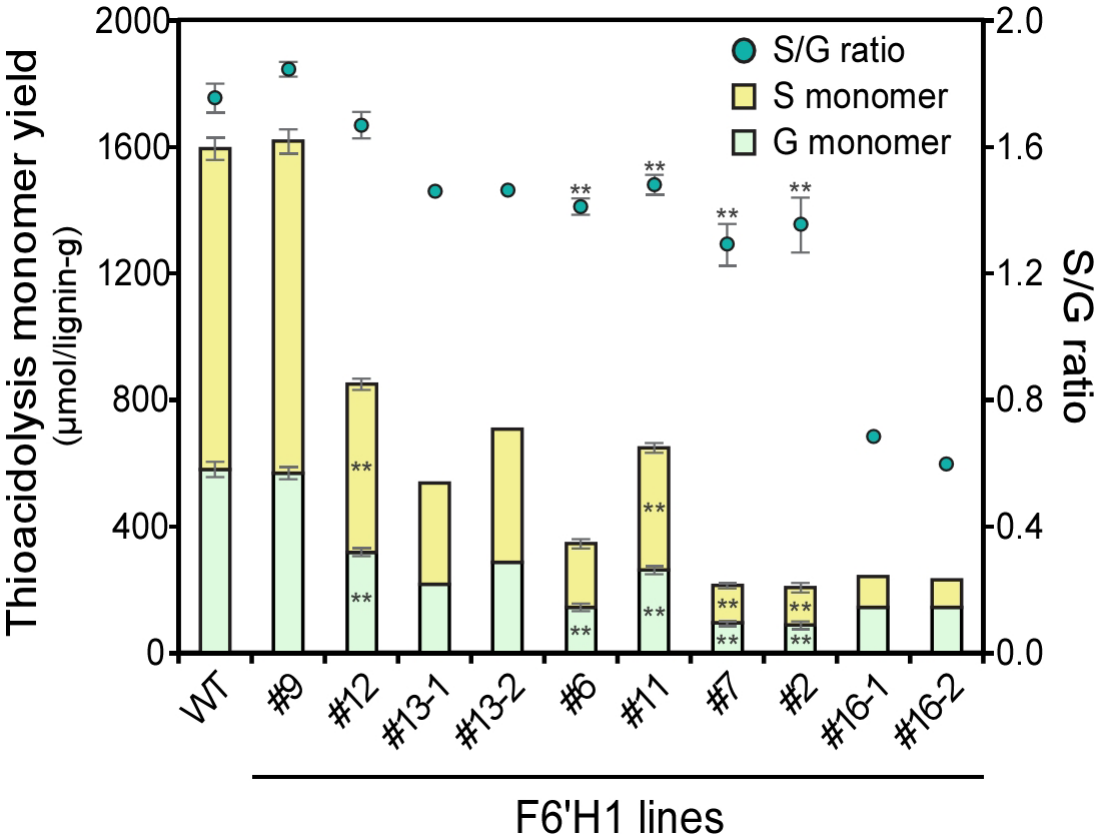
Thioacidolysis monomer yield and its monomeric composition. Values represent means with standard deviation from three to four independent biological replicates, except for #13 and #16 (n =2). Asterisks indicate significant differences from the wild type using the Student’s *t*-test (*0.01 < *p* < 0.05; **p <0.01).

The lignin structure of the plants was characterized using thioacidolysis (**Figure 4**), a method that selectively cleaves 8–O–4 (β–*O*–4) linkages in lignin to yield trithioethylated monomers. These monomers are often used as indicators to estimate the relative abundance of the linkages and the aromatic units involved. The total yields of the monomers with guaiacyl (G) or syringyl (S) residues were considerably reduced in the F6′H1 lines compared with those in the WT plants. The highest yield reduction, up to approximately 13% residual yield, was observed in line #7 (212 μmol/g lignin) compared with the WT (1,593 μmol/g), indicating a decrease in releasable β-ether units in the F6′H1 lines. Additionally, the S/G monomer ratio was lower in most F6′H1 lines (0.6–1.8) compared with that in the WT (1.8).

Thioacidolytic dimers were also quantified as their desulfurization products using GC/MS for the WT and two F6′H1 lines (#6 and #12). Ten distinct dimers containing the substructures 8–5, 8–1, 8–8, 4–*O*–5, and 5–5 were identified by their mass spectra (Lapierre et al., 1991; Kishimoto et al., 2010). Among these, two compounds contained two S units (SS), five contained both S and G units (SG), and three contained two G units (GG). Similarly to the monomer analysis, total dimer yields decreased from 234 μmol/g lignin in the WT to 43 and 72 μmol/g lignin in lines #6 and #12, respectively (**Supplementary Figure 6**). The ratios of SS/SG/GG dimers in the WT and lines #6 and #12 were 27/16/57, 2/21/77, and 15/18/67, respectively. These findings indicate that *F6′H1* expression reduces the proportion of S subunits, as also indicated by the results of the thioacidolytic monomer analysis (**Figure 4**) and NMR (see below).

**Figure 5:**
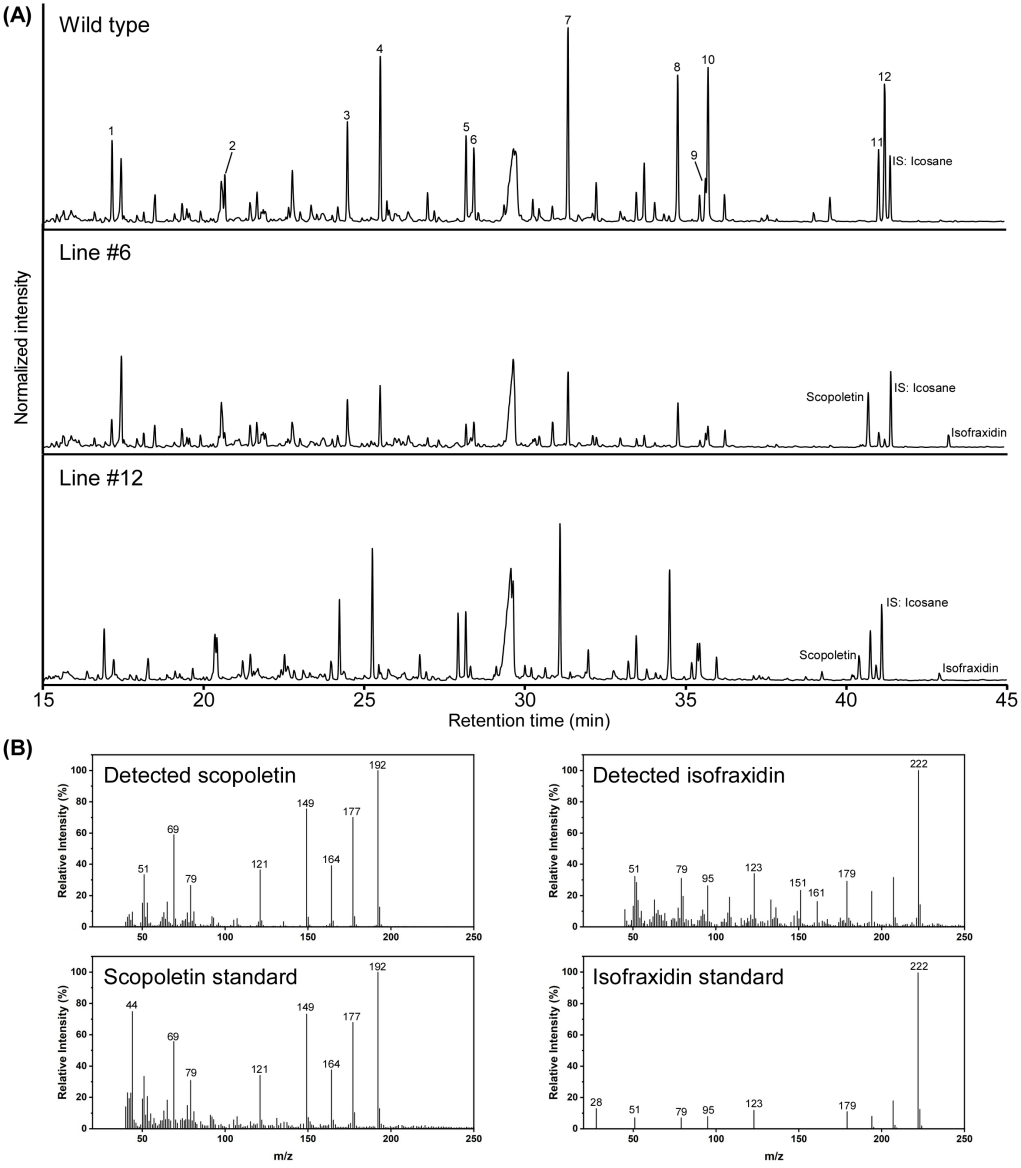
Pyrolysis gas chromatography-mass spectrometry (Py-GC/MS) analysis of cell wall residues prepared from stems of two F6′H1 lines (#6 and #12) and the wild-type (WT) plants. **(A)** Typical pyrogram obtained for each sample. Peak numbers correspond to compounds listed in **Supplementary Table 1**. **(B)** Typical mass spectra of scopoletin and isofraxidine detected in pyrolysates from the cell wall residue of lines #6 and #12. Scopoletin standard was also analyzed under the same condition used for the cell wall residue. Mass spectrum of isofraxidin standard analyzed by GC-MS was obtained from a database (https://spectrabase.com/spectrum/DDJMGSwvIWk).

To further confirm the relationship between the change in lignin structure and scopoletin accumulation, Py-GC/MS analysis was conducted again on the same wood powder samples as used for thioacidolysis, on lines #6, #11, #12, #16, and the WT. In addition to scopoletin, twelve compounds with G and S units, such as guaiacol and syringol, were identified in the F6′H1 lines (**Figure 5**; **Supplementary Table 1**). Notably, isofraxidin (6,8-dimethoxyumbelliferone), which is a structural analog of scopoletin, was detected exclusively in the transgenic lines and not in the WT (**Figure 5**). The peak area of isofraxidin on the pyrogram was significantly smaller than that of scopoletin in all transgenic lines analyzed. The S/G ratios calculated from the detected pyrolytic products with G and S units correlated well with those obtained from thioacidolysis. Furthermore, the scopoletin index, calculated as the ratio of scopoletin to coniferyl alcohol, was negatively correlated with the S/G ratio (**Supplementary Figure 7**).

To assess the impact of *F6′H1* expression on the molar mass distribution of lignin, cellulolytic enzyme lignins (CELs) (Chang et al., 1975) isolated from WT, line #12, and line #6 were subjected to size-exclusion chromatography (SEC) as shown in **Supplementary Figure 8**. A significant reduction in the large molar mass fraction was observed in the F6′H1 lines compared with that in the WT. The weight-average molar mass of lignin was calculated using polystyrene sulfonate standards (**Supplementary Figure 8**). The molar mass of the WT was approximately 8,790, whereas that of lines #6 and #12 decreased to approximately 3,460 and 5,680, respectively. The molar mass distribution of lignin therefore decreased significantly with the increase in the amount of scopoletin incorporated into the lignin molecule. 

### 3.7 2D nuclear magnetic resonance (NMR) analysis of ELs

ELs isolated from the WT and an F6′H1 line (line #11) were subjected to semi-quantitative HSQC-NMR profiling to characterize the lignin structures. This technique provides information on the S/G/H composition of the lignin from the aromatic region and the relative occurrence of each type of structural unit, characterized by their inter-unit linkage types, in the aliphatic region. The scopoletin peaks were assigned based on a standard reference compound. The spectral data revealed that approximately 5.7% of the lignin polymer in F6′H1 line #11 was derived from scopoletin (**Supplementary Figure 9**). The data also suggested that scopoletin was predominantly incorporated into lignin through its 4–O-position because the peaks corresponding to correlations from the C/H pairs labeled Sc_2_, Sc_5_, Sc_7_, and Sc_8_ all closely matched those of the authentic scopoletin compound that lacks additional C–C bonds at these positions. No signals corresponding to scopoletin units were detected in the WT plant. In the F6′H1 line, lignin compositional analysis revealed a relative increase in G units and a concomitant decrease in S units compared with that in the WT, as indicated by thioacidolytic analysis. H units, which represented 0.1% in the WT, increased to 0.9% in the F6′H1 line, although these measures are less reliable due to the need to derive them following subtraction of the overlapping phenylalanine peak.

The distribution of the linkages over the lignin polymer may be changed in the transgenic lines, as reported previously (Hu et al., 2022). In the aliphatic region, the relative level of β-aryl ether (8–O–4) units appeared to be slightly increased in the F6′H1 line, whereas the relative level of phenylcoumaran (8–5) and, especially, the resinol (8–8) were reduced in the F6′H1 line compared with those in the WT.

### 3.8 Transcriptomic analysis using RNA-seq

**Figure 6:**
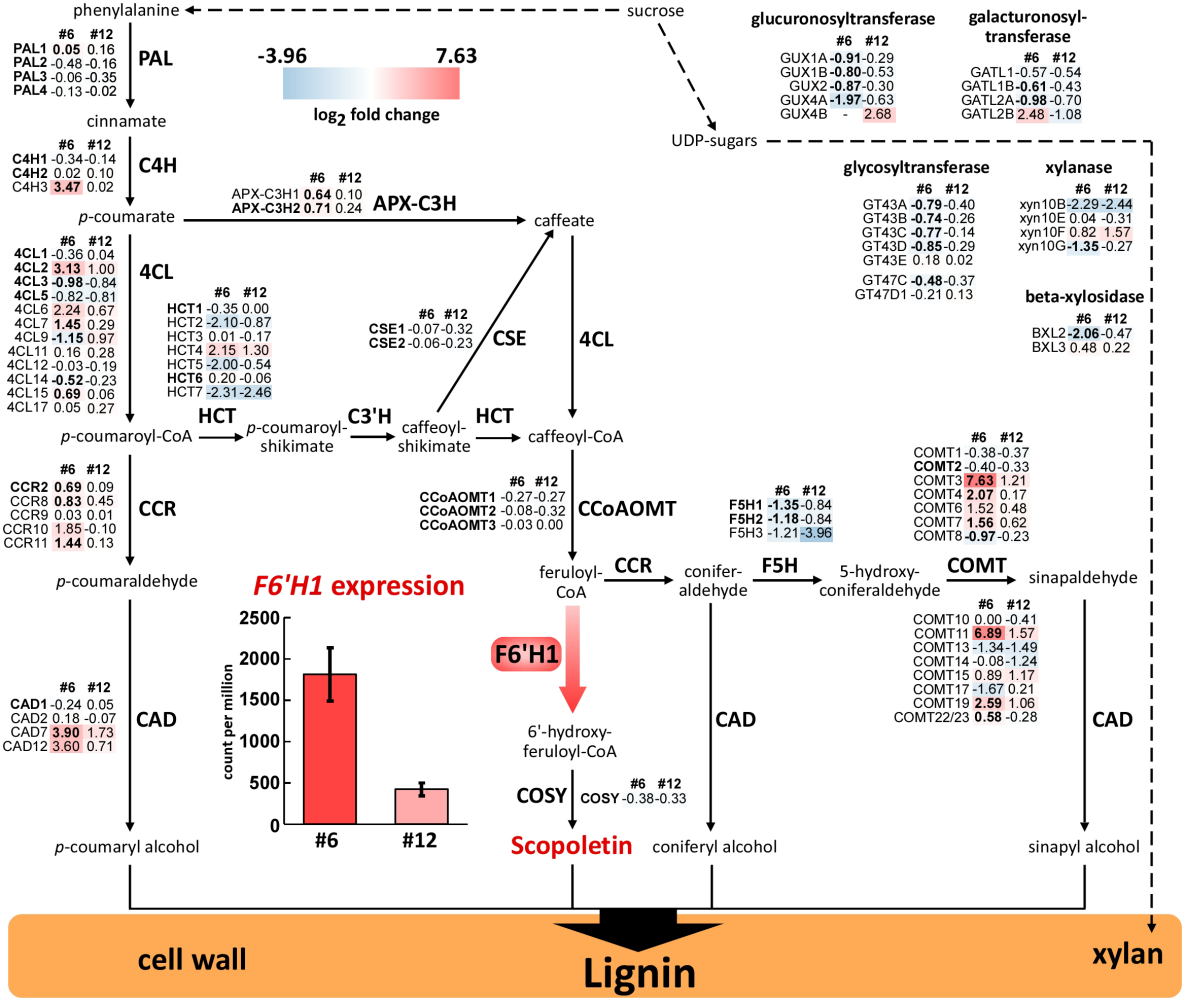
Expression profiles of lignin biosynthetic genes and gene family members in stems of F6′H1 transgenic plants. The analysis was performed with five (#6) or four (#12) biological replicates of each line as well as the WT (n = 4). Values indicate the logarithm of fold-change (log2FC) of gene expression in each transgenic line compared with the WT (obtained from biological quadruplicates). Abbreviated gene names and values in bold font indicate genes involved in monolignol biosynthesis and significant differences compared with the WT, respectively. A bar graph shows expression levels of *F6′H1* in different transgenic lines (lines #6, left, and #12, right).

To examine the global changes in gene expression induced by *F6'H1* expression, RNA-seq was performed using total RNA isolated from the developing xylem tissues of the WT and two transgenic lines (#6 and #12). Consistently with the RT-PCR results from sterile plants grown on MS medium, RNA-seq revealed higher *F6'H1* expression in line #6 (1,813 ± 322 counts per million; CPM) than in line #12 (422 ± 78 CPM; **Figure 6**). No *F6'H1* expression was detected in the WT plant.

DEG analysis using DESeq2 [version 1.24.0; (Love et al., 2014)] identified 9,255 DEGs with significant changes (adjusted *p* < 0.01) in #6 and 180 DEGs in #12 compared to the WT (**Supplementary Figure 10**, **Supplementary Table 2**). In line #6, 5,133 and 4,122 genes were upregulated and downregulated, respectively, and in line #12, 151 and 29 genes were upregulated and downregulated, respectively. Gene ontology (GO) category enrichment analysis was conducted on the DEGs to evaluate the transcriptional impact on both transgenic lines. In line #6, the 5,133 upregulated DEGs were primarily associated with cytoplasmic translation and amino acid and peptide metabolism. The 4,122 downregulated DEGs were enriched in the biosynthetic processes of macromolecules, including xylan and actin.

A comparison of the DEGs revealed that 146 upregulated and 24 downregulated genes were shared between lines #6 and #12. The upregulated DEGs were enriched in pathways related to systemic acquired resistance and biotic stress responses. In contrast, no significant GO enrichment was observed for the downregulated genes. Notably, *F6'H1* expression did not significantly affect the expression of most genes involved in lignin biosynthesis (**Figure 6**).

### 3.9 Expression of lignin biosynthetic genes

RNA-seq provided insights into the global expression of lignin biosynthetic genes in the F6′H1 lines (**Figure 6**). In both transgenic lines, most genes primarily involved in monolignol biosynthesis were expressed at levels similar to or lower than those in the WT. The expression of genes encoding caffeoyl CoA 3-*O*-methyltransferase (CCoAOMT), which catalyzes the formation of feruloyl-CoA, the substrate for F6′H1, was slightly decreased. Conversely, genes encoding cinnamoyl-CoA reductase (CCR), which is responsible for converting *p*-hydroxycinnamoyl-CoAs into its corresponding *p*-hydroxycinnamaldehydes (*p*-coumaraldehyde and coniferaldehyde), were slightly increased. Additionally, some homologous genes of cinnamyl alcohol dehydrogenase (CAD) and caffeic acid 3-*O*-methyltransferase (COMT) were upregulated. Notably, results from the GO analysis indicated that several genes involved in xylan biosynthesis exhibited decreased expression in both lines #6 and #12. Despite these transcriptional changes, the xylan content in the wood of the transgenic lines was comparable to that of the WT, suggesting that the reduced expression of these genes did not have a measurable impact on xylan accumulation.

### 3.10 Untargeted metabolomic analysis

**Figure 7:**
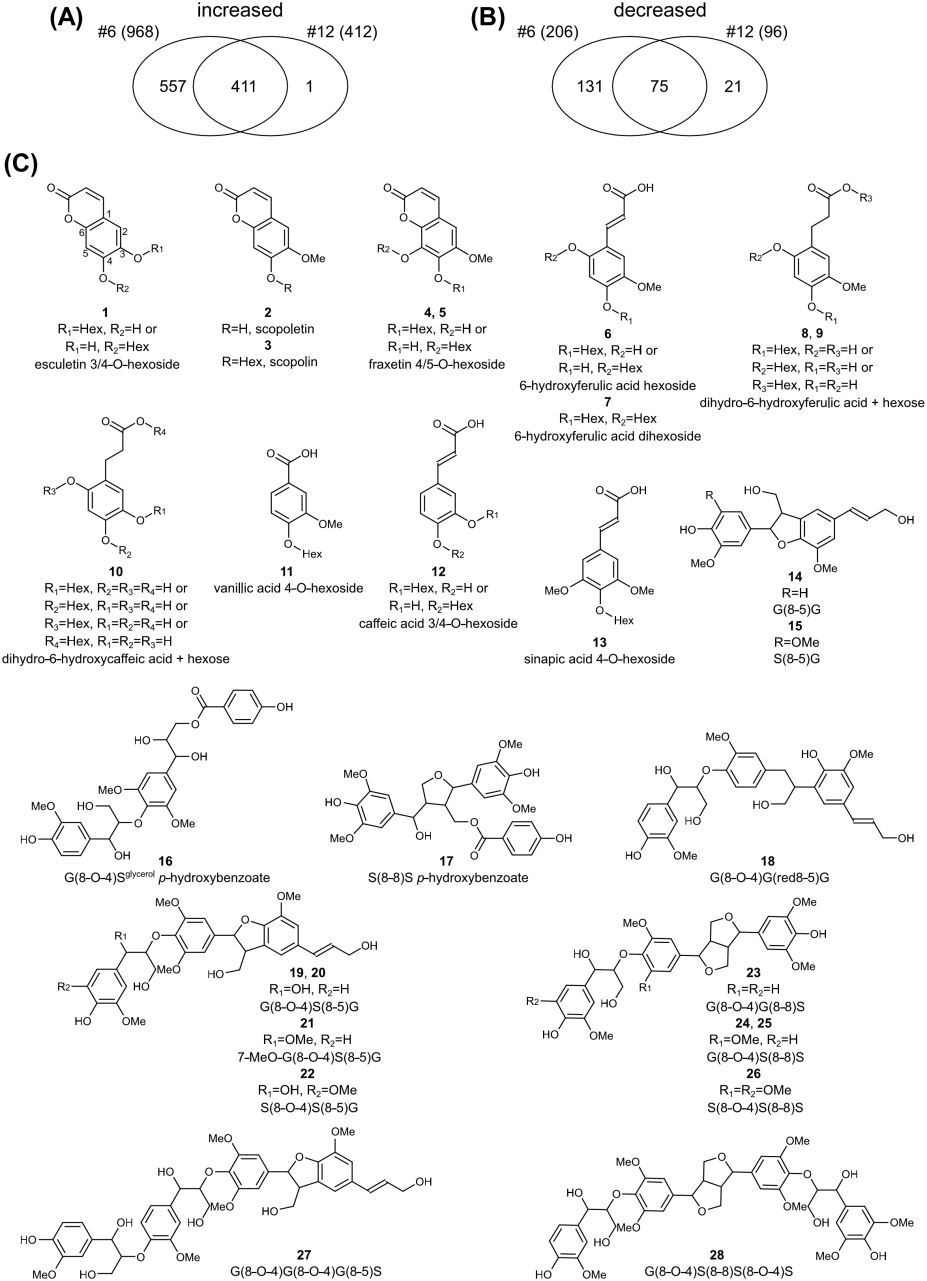
Untargeted metabolomic profiling of *F6’H1* overexpression lines. **(A)** Venn-diagram of the number of *m/z* signals with higher intensity in lines #6 and #12. **(B)** Venn-diagram of the number of *m/z* signals with lower intensity in lines #6 and #12. **(C)** Structures of the differential compounds between the transgenic lines and the wild-type plant.

To investigate the relationship between the altered lignin structure and soluble phenolic metabolites in the transgenic plants, the xylem extracts from lines #6 and #12 and the WT were subjected to UPLC-MS analysis. An untargeted metabolomic approach was used to identify metabolic alterations associated with *F6′H1* overexpression. It revealed a total of 3,405 *m/z* signals across the samples. Lines #6 and #12 exhibited 968 and 412 *m/z* signals, respectively, with a higher intensity compared to the WT. Notably, 411 signals showed higher intensities in both transgenic lines (**Figure 7A**). For structural characterization, the top twenty *m/z* signals with the highest average intensities across both transgenic lines were selected from this subset of 411 signals. These twenty signals were assigned to thirteen metabolites (**Table 1**, **Figure 7C**). Owing to the nature of the MS-based analysis, several metabolites were detected as adducts and fragments in addition to their molecular ions. Structural annotation based on MS/MS fragmentation spectra (**Supplementary Figure 11**) revealed the presence of five coumarins (1–5), four 6-hydroxylated phenylpropanoids (6–10), one benzenoid (11), and two phenylpropanoids without 6-hydroxy functionality (12, 13). The coumarins included scopoletin and its glucoside, scopolin, as well as two fraxetin derivatives (hydroxylated scopoletins).

**Table 1:** Differential compounds in the *F6’H1* overexpression lines #6 and #12.

		m/z	Rt(min)	WT		#6		ratio	#12		ratio
				average	s.d.	average	s.d.	#6/WT	average	s.d.	#12/WT
increased in #6 and #12											
**1**	esculetin 3/4-O-hexoside	339.0719	6.12	335	165	360000	27900	>100	103000	24500	>100
**2**	scopoletin^1^	176.0106	10.77	0	0	116000	22400	>100	54900	3740	>100
**3**	scopolin^1^	191.0345	7.37	535	162	226000	51600	>100	145000	41500	>100
**4**	fraxetin 4/5-O-hexoside 1	369.0823	6.56	33	17	101000	25000	>100	40700	9150	>100
**5**	fraxetin 4/5-O-hexoside 2	369.0823	8.05	11	13	117000	32900	>100	19900	5180	>100
**6**	6-hydroxyferulic acid hexoside	371.0977	7.34	0	0	127000	12800	>100	34500	9880	>100
**7**	6-hydroxyferulic acid dihexoside^2^	1067.308	5.45	0	0	242000	109000	>100	104000	48200	>100
**8**	dihydro-6-hydroxyferulic acid + hexose 1	373.1132	6.32	371	193	161000	32200	>100	72000	10900	>100
**9**	dihydro-6-hydroxyferulic acid + hexose 2	373.1129	6.70	462	180	782000	79600	>100	553000	122000	>100
**10**	dihydro-6-hydroxycaffeic acid + hexose	359.0978	4.81	8	11	137000	8460	>100	61100	11300	>100
**11**	vanilic acid 4-O-hexoside^2^	659.1824	4.63	19700	4040	116000	8940	6	40100	6720	2
**12**	caffeic acid 3/4-O-hexoside	341.0878	6.88	11300	1810	320000	88700	28	68000	8670	6
**13**	sinapic acid 4-O-hexoside	385.1122	7.28	6940	1280	151000	22100	22	63700	10100	9
decreased in #6 and #12											
**14**	G(8-5)G^1^	339.1233	15.62	9720	3470	1080	93	0.11	820	389	0.08
**15**	S(8-5)G^1^	369.1338	15.40	8510	3000	2020	512	0.24	1430	538	0.17
**16**	G(8-O-4)S^glycerol^ *p*-hydroxybenzoate	559.1816	13.63	5580	4380	147	86	0.03	314	175	0.06
**17**	S(8-8)S *p*-hydroxybenzoate	555.1859	17.15	75800	52900	3500	1740	0.05	5490	3400	0.07
**18**	G(8-O-4)G(red8-5)G	555.2229	15.24	11700	6000	1710	1110	0.15	1150	1010	0.10
**19**	G(8-O-4)S(8-5)G 1	583.2174	17.05	112000	39200	6160	1790	0.05	4680	2610	0.04
**20**	G(8-O-4)S(8-5)G 2	583.218	17.81	28400	10500	1310	487	0.05	804	534	0.03
**21**	7-MeO-G(8-O-4)S(8-5)G	597.2334	20.24	7900	2880	961	519	0.12	1940	1020	0.25
**22**	S(8-O-4)S(8-5)G	613.2281	16.81	14200	6420	1800	1070	0.13	1780	667	0.13
**23**	G(8-O-4)G(8-8)S	583.2179	19.91	10100	4340	718	230	0.07	1250	718	0.12
**24**	G(8-O-4)S(8-8)S	613.2283	18.82	38200	14600	2690	1090	0.07	7340	3260	0.19
**25**	G(8-O-4)S(8-8)S	613.2286	19.60	7900	3620	484	170	0.06	1230	677	0.16
**26**	S(8-O-4)S(8-8)S	643.2385	18.53	25800	11100	1170	432	0.05	5460	2080	0.21
**27**	G(8-O-4)G(8-O-4)S(8-5)G	779.2912	17.20	5770	2170	85	62	0.01	117	94	0.02
**28**	G(8-O-4)S(8-8)S(4-O-8)S	839.3128	19.66	13300	6340	216	152	0.02	1670	1150	0.13
**29**	unknown 1	319.1393	8.40	6910	1860	812	164	0.12	2790	611	0.40
**30**	unknown 2	375.1444	9.16	6790	1600	296	112	0.04	705	288	0.10

For each compound the observed *m/z* and retention time (Rt) are given. The average and standard deviation (s.d.) of the signal intensity for WT (n = 4), #6 (n = 4) and #12 (n = 4) is given. ^1^ Detected as an in-source fragment. ^2^ Detected as homodimer. See **Figure 7** for molecular structures and **Supplementary Figure 11** for the structural elucidation of the compounds.

Additionally, 206 and 96 *m/z* signals exhibited significantly lower intensities in lines #6 and #12, respectively, compared with the WT. Among these, 75 *m/z* signals were lower in both transgenic lines (**Figure 7B**). Using a similar approach as for the higher-intensity *m/z* peaks, we analyzed the top twenty signals with the highest intensities in the WT line. These twenty signals were associated with 17 compounds, 15 of which were structurally annotated based on their MS/MS fragmentation spectra (**Table 1**, **Figure 7C**, **Supplementary Figure 11**). The annotated compounds included four dilignols (14–17), nine trilignols (18–26), and two tetralignols (27–28).

### 3.11 Saccharification efficiency of F6′H1 transgenic lines

**Figure 8:**
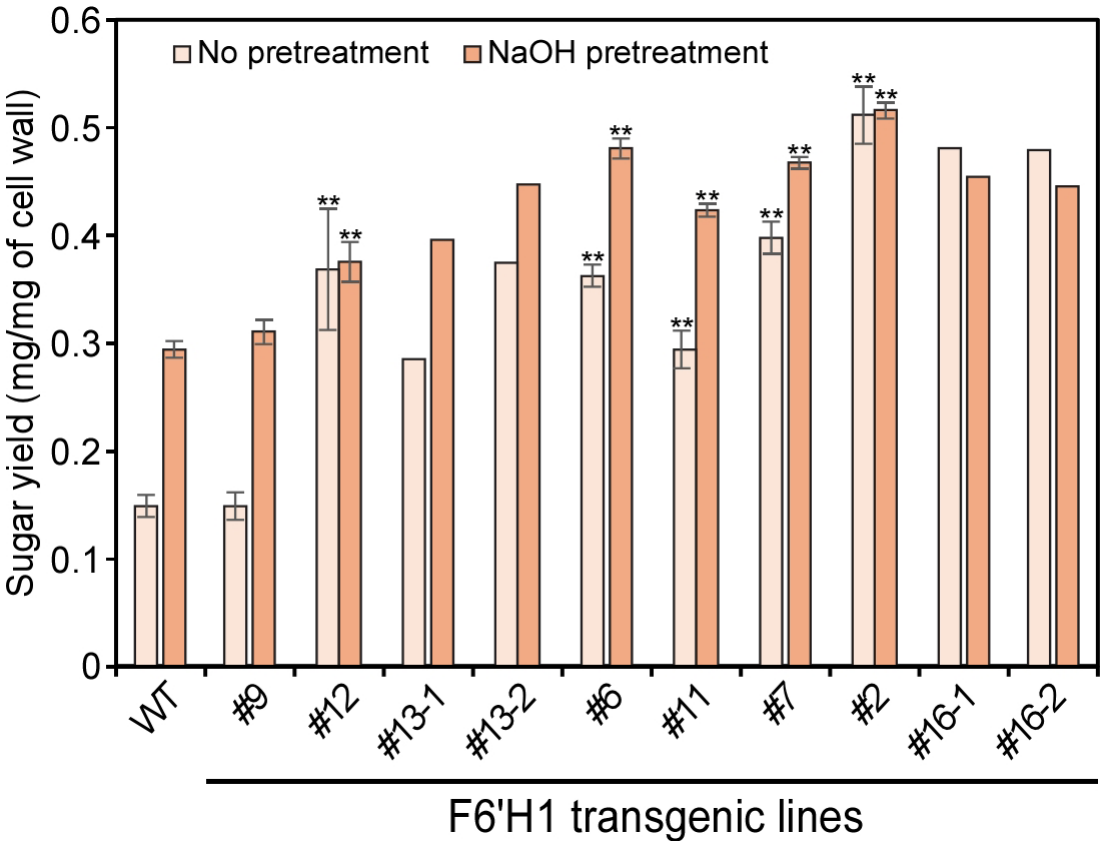
Saccharification efficiency of cell wall residue (CWR) assessed by enzymatic hydrolysis with and without alkaline pretreatments. The analysis was performed with two (#13 and #16) to six biological replicates of each line.

Enzymatic hydrolysis was conducted on wood powders from the WT and F6′H1 transgenic lines using a cellulase cocktail, and glucose yields were measured (**Figure 8**). Although there was no significant difference in cellulose content between the WT and transgenic lines, the saccharification efficiency of the F6′H1 lines was significantly increased, even without pretreatment, except for line #9 that displayed growth characteristics similar to that of the WT. The glucose yields of F6′H1 lines were up to three times higher (0.48 mg/mg of wood powder in line #16, but only two biological replicates were available due to growth inhibition of this line) than that of the WT (0.15 mg/mg). In some cases, the saccharification efficiency was further improved by the alkaline pretreatment of wood powder. This improvement is likely attributable to the decreased lignin content and reduced molar mass distribution of lignin in the F6′H1 lines, both of which enhance cellulase accessibility to cellulose within the cell walls.

## 4 Discussion

In this study, successful overproduction of scopoletin and its incorporation into lignin was achieved in transgenic hybrid aspens, similarly to results observed in transgenic *A. thaliana* plants (Sakamoto et al., 2020; Hoengenaert et al., 2022). Hoengenaert et al. (2022) attempted simultaneous overexpression of *COSY* and *F6'H1*; however, scopoletin accumulation in the present study, as in the study by Sakamoto et al. (2020), was achieved by overexpressing *F6'H1* alone as the last step in the biosynthesis of scopoletin, presumably because the isomerization and lactonization can occur spontaneously in light. Recently, scopoletin overaccumulation has also been reported in transgenic soybeans and tobacco BY-2 cells, as well as in transgenic *Arabidopsis* plants overexpressing *F6'H1* under the control of the Cauliflower Mosaic virus (CaMV) 35S promoter (Beesley et al., 2023).

Co-overexpression of *F6'H1* and *COSY* had no negative impact on the growth of *Arabidopsis* plants (Hoengenaert et al., 2022). Additionally, no obvious phenotypic differences were observed in transgenic *Arabidopsis* and soybean plants overproducing scopoletin and scopolin via constitutive expression of *F6'H1* controlled by the CaMV35S promoter (Beesley et al., 2023). In contrast, all transgenic hybrid aspens with scopoletin overaccumulation in the present study exhibited growth retardation. Growth inhibition is often observed in transgenic plants in which lignin is quantitatively and/or qualitatively altered (El Houari et al., 2021; Ha et al., 2021; De Meester et al., 2022). Such dwarfism, collectively referred to as lignin modification-induced dwarfism (De Meester et al., 2022), is thought to be caused by a loss of mechanical strength (collapsed xylem) or hydrophobicity in the xylem, excessive stress responses, and/or abnormal accumulation of phenolic intermediates.

Coumarins such as scopoletin and esculetin are known as phytoalexins with antimicrobial activity. They are abundantly secreted in the rhizosphere where they regulate root microflora and contribute to iron uptake (Stassen et al., 2021). Beneficial rhizomicrobes activate induced systemic resistance (ISR) (Pieterse et al., 2014), a conserved plant defense mechanism that enhances defensive capacity against a broad spectrum of plant pathogens. Another inducible defense mechanism, systemic acquired resistance (SAR), is activated after pathogen infection. SAR is associated with local and systemic increases in endogenously synthesized salicylic acid (SA), whose synthesis is regulated by isochorismate synthase 1 (ICS1). The expression of SAR requires the coordinate expression of genes encoding pathogenesis-related (PR) protein. Unlike SAR, ISR functions independently of SA and PR gene activation, but requires jasmonic acid and ethylene signaling (van Wees et al., 2000). In *Arabidopsis*, SAR and ISR both require a positive key regulatory protein NPR1 that is encoded by nonexpressor of pathogenesis-related genes 1 (*NPR1)* (Ding et al., 2018).

Expression of the aspen homologue (Potri.006G148100) of *Arabidopsis NPR1* was significantly elevated in line #6 (1.6-fold, *p <*0.01) compared to its expression in the WT. In addition, three (Potri.012G118500, Potri.015G117200, and Potri.002G056500) and two (first two of the three) poplar homologues of *NPR3*, a negative regulator of SAR, were significantly upregulated in lines #6 and #12, respectively. A poplar homologue (Potri.015G045300) of *Arabidopsis*, *SAR Deficient 1* (*SARD1*, AT1G73805), a key regulatory gene of *iCS1* induction and SA biosynthesis, was also significantly upregulated in both line #6 (264-fold, *p* < 0.01) and #12 (35.7-fold, *p* < 0.01) compared with the WT. The simultaneous activation of both activators and repressors suggests that disruption of the SA-induced immune system has occurred in the F6′H1 lines. Other stress-response-related genes, such as those encoding glutathione S-transferase and ethylene response factor, were also commonly upregulated in the two F6′H1 lines. Although deformed vessels were observed in the histochemical analysis of F6′H1 lines (**Figure 2A**), they were not as collapsed as to impede water conduction, as previously reported (Kajita et al., 1997; Jones et al., 2001; Voelker et al., 2011; De Meester et al., 2018). These results suggest that the growth retardation observed in F6′H1 lines may be attributed to an abnormal stress response rather than an inhibition of water conduction, although the molecular mechanism behind the induction of these genes remains unclear.

UV spectrophotometric and fluorescence microscopic analyses (**Figures 2D–F**) revealed that scopoletin was heterogeneously distributed within the cell walls. UV-Vis spectra of different parts of the xylem tissue indicated that scopoletin incorporation into the secondary cell wall of fibers was markedly lower than that in the vessel cell walls and compound middle lamellae. However, the mechanism underlying heterogeneous scopoletin incorporation remains unknown. One hypothesis is that scopoletin is more readily incorporated into G lignin, which is more abundant in vessels and the compound middle lamellae (Fergus and Goring, 1970; Saka and Goring, 1988), than into S lignin, which is more abundant in fiber cells. Another possibility is that the promoter of *Populus C4H* (Sakamoto et al., 2020), used to control the expression of *F6′H1* in the present study, drives stronger expression in vessels than in fibers, or more predominantly at early stages of xylem cell lignification. In fact, it was reported that expression of the two *C4H* genes in an aspen (*Populus tremula*) tends to be higher in the early stage of xylem differentiation than in the later stage (Sundell et al., 2017).

Metabolomic analysis revealed that, in addition to scopoletin, its glucoside, scopolin, was significantly overproduced in the methanol-soluble fraction of stems from F6′H1 lines. Derivatives of other coumarins, such esculetin and fraxetin, were also increased. Given that recombinant F6′H1 and its homologue F6′H2 exhibited a trace activity toward *p*-coumaroyl-CoA, in addition to their substantial activity toward feruloyl-CoA (Kai et al., 2008), esculetin in F6′H1 lines is expected to be biosynthesized via 6-hydroxylation of *p*-coumaroyl-CoA, followed by lactonization and subsequent 7-hydroxylation of the coumarin ring. Additionally, isofraxidin, the syringyl analog of scopoletin, was detected in pyrolysates of cell wall residues from F6′H1 lines. These findings suggest that isofraxidin, like scopoletin, may bind to the cell wall, and possibly to lignin.

In addition to coumarins and their derivatives, four derivatives of 6-hydroxyferulic acid and one derivative of 6-hydroxycaffeic acid were significantly accumulated in F6′H1 lines (**Figure 7**, **Table 1**). Derivatives of these compounds also overaccumulated in the *Arabidopsis* mutants deficient in *COSY*. These *o*-hydroxycinnamic acid derivatives, at least 6-hydroxyferulic acid derivatives, should be metabolized from 6-hydroxycinnamoyl-CoAs, the expected products of the reaction catalyzed by F6′H1. Although 6′-hydroxyferuloyl-CoA can spontaneously convert to scopoletin via *trans*–*cis* isomerization and subsequent lactonization (Kai et al., 2008), COSY can convert 6′-hydroxyferuloyl-CoA into scopoletin more efficiently (Vanholme et al., 2019). A homolog (Potri.004G053500) of *Arabidopsis COSY* (AT1G28680) exists in the poplar genome. RNA-seq data revealed that its expression tended to decrease in both F6′H1 lines, although this difference was not significant compared to the WT (**Figure 6**). These results suggest that overexpression of *F6'H1* alone in F6′H1 lines did not produce coumarins from *o*-hydroxycinnamoyl-CoAs as efficiently as when it was co-expressed with *COSY*. The retained *o*-hydroxycinnamoyl-CoAs were therefore likely metabolized into related compounds such as 6-hydroxyferulic and 6-hydroxycaffeic acid derivatives, which may be potential contributors to the growth inhibition observed in F6′H1 lines.

In lines #6 and #12, the lignin content was reduced by 22% and 10%, respectively, compared with the WT (**Figure 3**). This decrease tended to negatively correlate with the expression of F6′H1 in those lines and the level of scopoletin incorporation into their cell wall (**Figures 6** and **Supplementary Table 1**). Although several of the genes primarily involved in lignin biosynthesis (gene names in bold in **Figure 6**) were up- or down-regulated, significant differences in their expression could be detected only in line #6, not in line #12, compared to those in the WT (**Figure 6**). In addition, none of the 170 genes that showed significant change in the expression both in lines #6 and #12 (**Supplementary Figure 10** and **Supplementary Table 2**) included the genes for enzymes or transcription factors primarily involved in lignin biosynthesis. These results suggest that the reduction in lignin content in F6′H1 lines was not due to a downregulation of the lignin biosynthetic genes. Based on the present data, it is difficult to conclude whether the reduction was due to an indirect effect attributable to the stress response discussed above or to an excessive consumption of feruloyl-CoA by F6′H1, which increases metabolic flux to scopoletin, its precursor and derivatives.

As noted above, the NMR shows that the major scopoletin peaks retain the intact unsaturated sidechain. This implies that the major incorporation is via 4–O–8-coupling (with perhaps some 5–8-coupling) to a monolignol which in turn implies that scopoletin units are primarily involved in starting a lignin chain and are not incorporated into a growing polymer (Hoengenaert et al., 2022). The overproduction of non-canonical initiators is often accompanied by structural changes in lignin in genetically modified plants and mutants. In transgenic *Arabidopsis* expressing a bacterial hydroxycinnamoyl-CoA hydratase-lyase, C6-C1 aromatics such as vanillin and syringaldehyde accumulate and are incorporated into lignin mainly as initiators (Eudes et al., 2012). Similarly, dihydrocinnamyl alcohol and its homo-coupled structures have been detected in milled wood lignin prepared from a pine mutant deficient in the cinnamyl alcohol dehydrogenase gene (Ralph et al., 1997). These structures with C6-C1 and dihydrocinnamyl alcohol units exist only at the initial ends of elongating chains because they lack linkage sites in their sidechains to allow coupling into the growing polymer. In both the transgenic *Arabidopsis* and the pine mutant, the molar masses of the isolated lignins were lower than those of the WT plants (Dimmel et al., 2001; Eudes et al., 2012), as also observed in F6′H1 lines (**Supplementary Figure 8**). An excessive supply of such non-canonical initiators for lignin polymerization may lead to a higher number of molecules elongating in cell walls, resulting in a decrease in the molar mass of lignin produced.

The S/G ratios, calculated based on data from thioacidolytic monomers and pyrolytic products, showed a significant negative correlation with the scopoletin level detected by Py-GC/MS in each line (**Supplementary Figure 7**). Although only one line (#11) was analyzed, 2D NMR spectra also revealed that the S/G ratio of a F6′H1 line (#11) was lower than that of the WT (**Supplementary Figure 9**). A possible cause for the decrease in the S/G ratio is a change in the proportion of monolignols owing to the perturbation of phenolic metabolism. These results suggest that *F6'H1* expression has a greater impact on the biosynthesis of S lignin, which requires additional metabolic steps for precursor biosynthesis than G lignin. Conversely, co-overexpression of *F6'H1* and *COSY* under the control of the *CesA4* promoter resulted in an increased S/G ratio in transgenic *Arabidopsis* compared with the WT (Hoengenaert et al., 2022). In the transgenic *Arabidopsis*, the expression level of *F5H1*, the gene responsible for syringyl lignin biosynthesis, was unchanged compared with the WT. In contrast, the expression of three homologs of *F5H1* in the present F6′H1 lines tended to be lower than the WT (**Figure 6**), and the S/G ratio decreased with increasing scopoletin deposition in the cell wall in F6′H1 lines (**Supplementary Figure 7**). The presence or absence of *COSY* overexpression may be responsible for the different results between the two plant species.

A reduction in the relative level of resinol units is expected when the S/G is lower, but the degree of reduction noted here in the NMR spectrum (**Supplementary Figure 9F** vs **Supplementary Figure 9E**) was striking. The simple explanation likely relates to the scopoletin incorporation. Resinol units in dicot lignins are largely S–S and can only arise at the monomer level, i.e., these structures cannot be produced within a growing chain but only from chain initiation, the starting end(s) of the polymer, by monomer dimerization, and there can be only one in any linear chain (Ralph et al., 2008). In the F6′H1 line, the scopoletin units were noted to only be chain-starters. Evidently, the cross-coupling of scopoletin monomers with monolignols is successfully competing with monolignol dimerization as a chain-starting event. Thioacidolysis showed a significant decrease in the ether units (8–O–4-linkages) in F6′H1 lines, but only a fairly mild reduction in line #11 (**Figure 4** and **Supplementary Figure 9**). The NMR data would nevertheless predict a higher thioacidolytic monomer yield, on a lignin basis, from F6′H1 line #11. The logical conclusion is that lignin-(8–O–4)-scopoletin ethers are not cleaved efficiently, as perhaps evidenced by the absence of any notable monomeric scopoletin-derived peak in the chromatograms of thioacidolysis products.

The decrease in lignin content and the S/G ratio associated with the introduction of scopoletin resulted in increased glucose release from the cell wall during enzymatic saccharification, although the major sugar composition of the cell wall did not change significantly. Line #16 showed more than three times the saccharification efficiency of the WT, even without alkaline pretreatment (**Figure 8**). Generally, saccharification efficiency of lignocellulosic materials depends on various factors such as cell wall porosity, cellulose crystallinity, and the binding properties of lignin on hydrolytic enzymes. Based on a result of X-ray diffraction measurement of cell wall residues, no apparent change in cellulose crystallinity could be detected between the WT and F6′H1 lines (**Supplementary Figure 12**). One potential cause for the increased saccharification efficiency in F6′H1 lines may be their lower lignin content. Woody material with lower initial lignin content is more accessible to cellulolytic enzymes, even without pretreatment, resulting in more cellulose being converted to glucose compared with the WT. Additionally, the molar mass of lignin in the F6′H1 line is smaller than that of the WT, suggesting that the lignin is easier to remove, especially with alkaline pretreatment due to the higher phenolic content, and exerts less inhibition against enzymatic hydrolysis. *In vitro* studies have shown that lignin with smaller molar masses has a lesser negative effect on enzymatic cellulose hydrolysis compared with larger lignin (Li et al., 2018).

In conclusion, overexpression of *F6'H1* alone led to the successful accumulation of scopoletin and its subsequent incorporation into lignin in transgenic hybrid aspen. Scopoletin was heterogeneously distributed across different xylem cells and cell wall layers. The incorporation led to reductions in lignin content, S/G ratio, and molar mass distribution. Although the cellulose and hemicellulose contents in the F6′H1 lines remained unchanged, the reduction in lignin resulted in a significant increase in saccharification efficiency in F6′H1 lines, even without pretreatment. If a successful strategy can be developed to generate F6′H1 transgenic hybrid aspens without growth penalties, these plants could serve as a promising feedstock for renewable chemicals and fuels.

## Data Availability

The datasets presented in this study can be found in online repositories. The names of the repository/repositories and accession number(s) can be found in the article/**Supplementary Material**.
