# Supplementary material for: Effects of feruloyl-CoA 6′-hydroxylase 1 overexpression on lignin and cell wall characteristics in transgenic hybrid aspen
Source: Front Plant Sci. 2025 Mar 28;16:1543168. doi: 10.3389/fpls.2025.1543168 (PMC11985793; doi:10.3389/fpls.2025.1543168)
Supplement: Supplementary file 1 [file DataSheet1.pdf]

Supplementary Material

Effects of *FERULOYL-CoA 6'-HYDROXYLASE 1* overexpression on lignin and cell wall characteristics in transgenic hybrid aspen

Naning Wang, Masatsugu Takada, Shingo Sakamoto, Ruben Vanholme, Geert Goeminne, Hoon Kim, Soichiro Nagano, Naoki Takata, Naofumi Kamimura, Mikiko Uesugi, Akiko Izumi-Nakagawa, Eiji Masai, Nobutaka Mitsuda, Wout Boerjan, John Ralph, Shinya Kajita

Supplementary Materials and Methods

RT-PCR

Total RNA was isolated from *in vitro*-cultured plants and then used (1 μg) for synthesis of first strand complementary DNA. PCR was performed using GoTaq® DNA Polymerase (Promega K.K., Tokyo) under following reaction conditions: an initial denaturation at 98°C for 3 min, followed by 30 cycles of denaturation at 95°C for 30 s, annealing at the specific annealing temperature (56.9°C for *F6'H1*, 56°C for *UBIQUITIN*) for 30 s, and extension at 72°C for 1 min. A final extension step was performed at 72 °C for 5 minutes. Nucleotide sequences of primers are follows:

F6H1-Fw1	GATGCTGCTGAAAAGTGGGG
F6H1-Rv1	CTGTTCAGCCTCAGCTTCAGA
Pt×tUBQ.for	GGTTGATTTTTGCTGGGAAGC
Pt×tUBQ.rev	GATCTTGGCCTTCACGTTGT

Preparation of holocellulose

Holocellulose was prepared as previously described (Liu et al., 2021), with some modifications. Briefly, 40 g of extractive-free wood powder was placed in a test tube, mixed with 1.24 mL of water, 75 μL of 20% $NaClO_2$, and 10 μL of acetic acid, and stirred at 75 °C for 1 h. The mixture was purified by adding 75 μL of 20% $NaClO_2$ and 10 μL of acetic acid. Then, 75 μL of 20% $NaClO_2$ and 10 μL of acetic acid were added and stirring for 1 h. These steps were repeated until the wood powder turned white. After washing with distilled water, the residue was collected via filtration, dried, and weighed.

Preparation of α-cellulose

α-Cellulose was prepared as previously described (Mahon et al., 2022). Briefly, approximately 30 mg of holocellulose was mixed with 17.5% NaOH (2.5 mL) and stirred

for 30 min. The reaction was continued for 30 min after adding 2.5 mL water. Then, the residue was filtered, rinsed with distilled water, and immersed in acetic acid for 5 min. After rinsing with distilled water, the residue was collected, dried, and weighed.

Determination of monosaccharide content

Sulfuric acid solution (72%, 250 μL) was added to 20 mg of extractive-free samples for pre-hydrolysis. The reaction mixture was stirred with a glass rod every 10 min for 45 min. Subsequently, 2.7 mL of distilled water was added to dilute the sulfuric acid concentration to 6%. The test tubes were covered with aluminum foil and autoclaved at 121 °C for 1 h. To plot the standard curve, various monosaccharide mixtures were subjected to the same hydrolysis treatment with dilute acid. The samples were cooled and mixed with 120 μL of inositol (10 mg/mL) as an internal standard. The supernatant was recovered via centrifugation and neutralized with an appropriate amount of $NaHCO_3$. Then, monosaccharide content of the sample was determined as their alditol acetate derivatives via gas chromatography (GC) with a flame ionization detector (GC-2010 Plus Shimadzu, Kyoto, Japan).

Thioacidolysis for monomer analysis

Thioacidolysis for monomer analysis was performed as previously described by Yamamura et al. (2012). Briefly, extractive-free wood powder (approximately 7 mg) in a screw-caped vial was mixed with 800 μL of a mixture of 1,4-dioxane, ethanethiol, and boron trifluoride diethyl etherate (87.5:10:2.5 v/v) and heated at 100 °C for 4 h. After heating, the vials were cooled on ice. Docosan (50 μg) was used as an internal standard. The reaction was stopped by adding $NaHCO_3$, and pH was adjusted to 3–4 using 1 M HCl. Diethyl ether was added to extract the degradation product, and the organic layer was recovered. Then, ether was dehydrated by adding saturated Na_2SO_4 and dried under nitrogen gas. The product was silylated with *N*,*O*-bis(trimethylsilyl) acetamide and analyzed via GC with a flame ionization detector.

Thioacidolysis for dimer analysis

Thioacidolysis product obtained for the monomer analysis was dissolved in dioxane, transferred to a test tube with a Teflon screw cap containing a skeletal nickel catalyst slurry (TCI K0073; TOKYO CHEMICAL INDUSTRY Co., LTD, Tokyo) for desulfurization, and incubated at 50 °C for 4 h, as described by Kishimoto et al. (2010). Then, 5 mL of water was added, and pH was adjusted to 3–4 using 1 M HCl. Subsequently, 5 mL of dichloromethane was added. The organic layer was collected, dehydrated using

Na_2SO_4, and concentrated to approximately 300 μL. Then, 10 μL of the solution was mixed with 5 μL of pyridine and 10 μL of *N,O*-bis(trimethylsilyl)trifluoroacetamide and subjected to trimethylsilylation at 60 °C for 20 min. The derivatized sample was analyzed using the GC-2010 system (Shimadzu), injected in splitless mode at an injector temperature of 250 °C. Separation was performed using a column (0.25 mm × 30 m; GL Sciences Inc., Tokyo). The temperature program was set as follows: initial temperature of 180 °C for 1 min, followed by a ramp to 280 °C at 2 °C/min, with a hold time of 9 min at 280 °C. The ion source temperature was set at 260 °C, and carrier gas was He. A response factor of 1.5 (docosane) was used for quantification of all detected dimers (Kishimoto et al. 2010).

Pyrolysis-GC/MS analysis (for data shown in Supplementary Figures 6 and 7, and Supplementary Table 1)

Samples were thermally cleaved using the JHP-3 Pyrolyzer (Japan Analytical Industry, Tokyo), pyrolyzed at 500ºC for 4 s, and separated and analyzed using the GCMS-QP5050A system (SHIMADZU CORPORATION, Kyoto). The cleavage products were further separated and analyzed using the GCMS-QP5050A system. The inlet temperature was set at 280ºC and interface temperature at 250ºC. DB-5MS column (30 m × 0.25 mm × 0.5 μm, Agilent Technologies Japan, Ltd., Tokyo) was used for chromatography. The heating conditions for GC were set as follows: initial temperature of 50 ºC held for 1 min, followed by heating up to 280ºC at 5ºC/min and holding at 280ºC for 13 min. Carrier gas was He.

Size exclusion chromatography

Cellulolytic enzyme lignin (CEL) was prepared as described previously (Madigal *et al.* 2025). It dissolved in 10 mM LiBr in dimethyl sulfoxide solution, and then filtered. The filtered solution was analyzed via size exclusion chromatography using an HPLC system equipped with two Agilent PolarGel M columns (7.5 × 300 mm; 5 μm particle size; Agilent Technologies, Inc. Santa Clara, CA, USA) and a guard column (7.5 × 50 mm). The analytical conditions were set as follows: mobile phase flow rate of 0.5 mL/min, column temperature of 40°C, sample injection volume of 10 μL, and UV detection wavelength of 280 nm, with polystyrene sulfonate standards as references.

Metabolomic analysis

Three microliters of each sample were injected into a Waters Acquity UHPLC (Waters, Milford, MA, USA) device connected to a Synapt XS high-definition mass spectrometer

(Waters). Chromatographic separation was carried out on an ACQUITY UPLC BEH C18 (150 × 2.1 mm; 1.7 μm) column (Waters) maintained at 40°C. The mobile phase consisted of buffer A (water + 0.1% formic acid, pH 3) and buffer B (acetonitrile + 0.1% formic acid, pH 3) under the following gradient: 99% of buffer A, decreased to 50% over 30 min, then to 30% from 30–35 min, and finally to 0% from 35–37 min. The flow rate was set at 0.35 mL/min. Electrospray Ionization (ESI) was applied using a LockSpray ion source in negative ionization mode under the following conditions: capillary voltage, 2 kV; reference capillary voltage, 2.5 kV; source temperature, 120°C; desolvation gas temperature, 550°C; desolvation gas flow, 800 L/h; and cone gas flow, 50 L/h. Full MS scans were performed with a collision energy of 4 eV, whereas high-energy scans (HDMSe) used a ramped collision energy from 20–70 eV. For the DDA-MS/MS, the low-mass ramp was between 6–20 eV, and the high-mass ramp was between 20–70 eV. The mass range was set to 50–1200 Da, with a scan time of 0.15 s. Nitrogen (≥99.5%) was employed for desolvation and cone gas. Leucine-enkephalin (100 pg/μL solubilized in a 1:1 [v/v] water:acetonitrile mixture containing 0.1% formic acid) was used as a lock mass-calibration standard, scanned every 1 min for 0.5 s. Profile data were recorded using Masslynx software (Waters). Data processing was performed using Progenesis QI v3.0 (Waters) for chromatogram alignment and compound ion detection. The detection limit was set at medium sensitivity with a minimum peak width of 0.04 min.

X-ray diffraction (XRD)

Crystallinity index of cellulose in extractive-free wood powder was calculated based on XRD measurement (X-ray diffractometer, RINT 2100 VPC/N, Rigaku, Japan). The measurement parameters were as follows: Scanning angle range, 5°–50°; accelerating voltage, 40 kV; current, 30 mA; scanning speed, 3°/min. The relative index was calculated according to Segal *et al.* (1959).

References

Kishimoto T., Chiba W., Saito K., Fukushima K., Uraki Y., Ubukata M. (2010). Influence of Syringyl to Guaiacyl Ratio on the Structure of Natural and Synthetic Lignins. Journal of Agricultural and Food Chemistry. 58, 895–901.

Liu Y., Wu S., Zhang H., Xiao R. (2021) Fast pyrolysis of holocellulose for the preparation of long-chain ether fuel precursors: Effect of holocellulose types. Bioresource Technology, 338, 125519.

Madigal J.P.T., Terasaki M., Takada M., Kajita S. (2025) Synergetic effect of fungal pretreatment and lignin modification on delignification and saccharification: A case study of a natural lignin mutant in mulberry. Biotechnology for Biofuels and Bioproducts. 18, 13.

Mahon E. L., de Vries L., Jang S.-K., Middar S., Kim H., Unda F., Ralph J., Mansfield S. D. (2022) Exogenous chalcone synthase expression in developing poplar xylem incorporates naringenin into lignins. Plant Physiology, 188, 984–996.

Segal, L., Creely, J.J., Martin, A.E., Conrad, C.M. (1959). An empirical method for estimating the degree of crystallinity of native cellulose using the X-ray diffractometer. Textile Research Journal, 29, 786–794.

Yamamura M., Hattori T., Suzuki S., Shibata D., Umezawa T. (2012) Microscale thioacidolysis method for the rapid analysis of β-O-4 substructures in lignin. Plant Biotechnology. 29, 419-423.

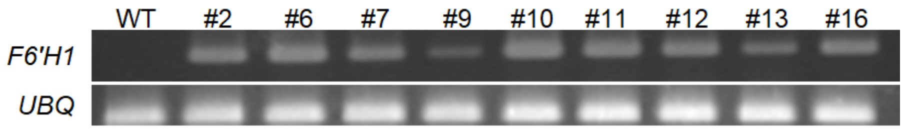

Supplementary Figure 1. Expression analysis of *F6'H1* transgene in *in vitro*-cultured plants in Murashige and Skoog medium using semiquantitative reverse transcription-polymerase chain reaction. Ubiquitin expression was also analyzed as a control. MS, Murashige and Skoog; RT-PCR, reverse-transcription polymerase chain reaction.

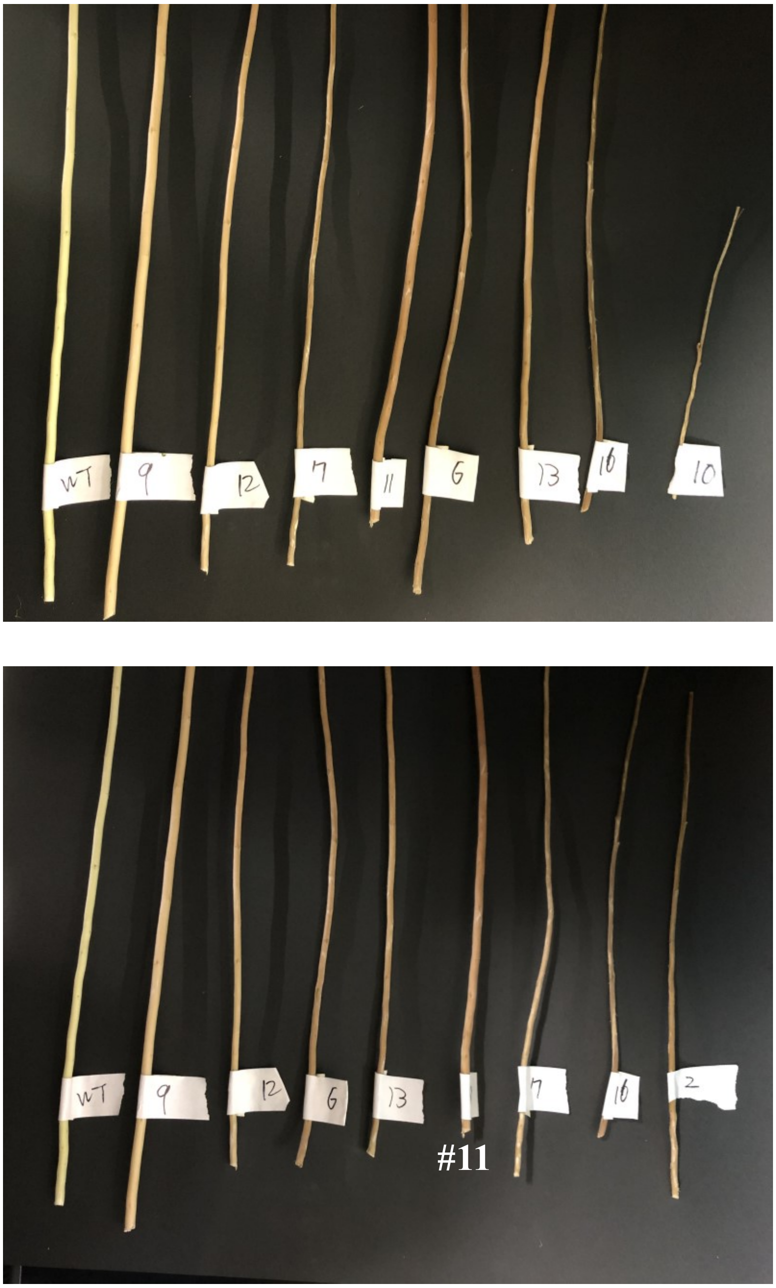

Supplementary Figure 2. Debarked of F6′H1 lines and the wild-type plants. Unlike the wild-type stem, transgenic xylem appeared in brown (lines #11, #6, #7, #16, #2, and #10) or pale brown (line #9 and #12). The stems shown in the top and bottom photographs differ only in the one on the far right (lines #2 and #10). These photographs were taken immediately after the debarking.

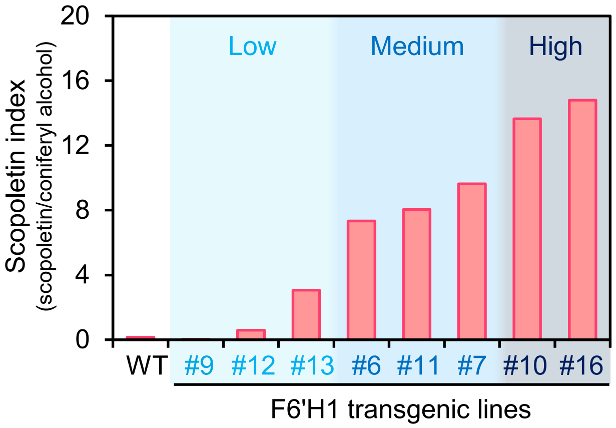

Supplementary Figure 3. Scopoletin levels in cell wall residues prepared from *in vitro*-cultured plants in MS medium. The levels are expressed as the ratio of the peak area of scopoletin to the peak area of coniferyl alcohol detected via Py-GC/MS.

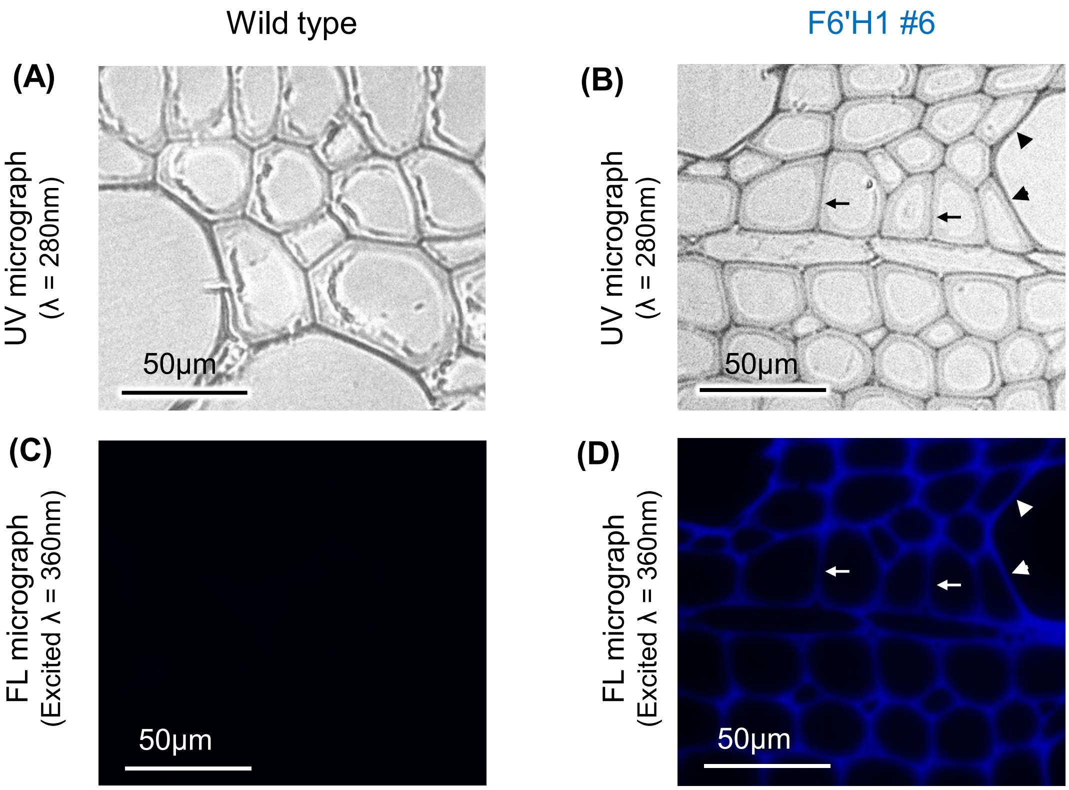

Supplementary Figure 4. Typical UV micrographs of ultra-thin transverse sections taken under the wavelength of 280 nm **(A and B)**. Fluorescence micrographs of the same fields with UV micrographs under the excitation wavelength of 360 nm **(C and D)**. Cell walls of a vessel element and fiber cells are shown with arrowheads and arrows, respectively.

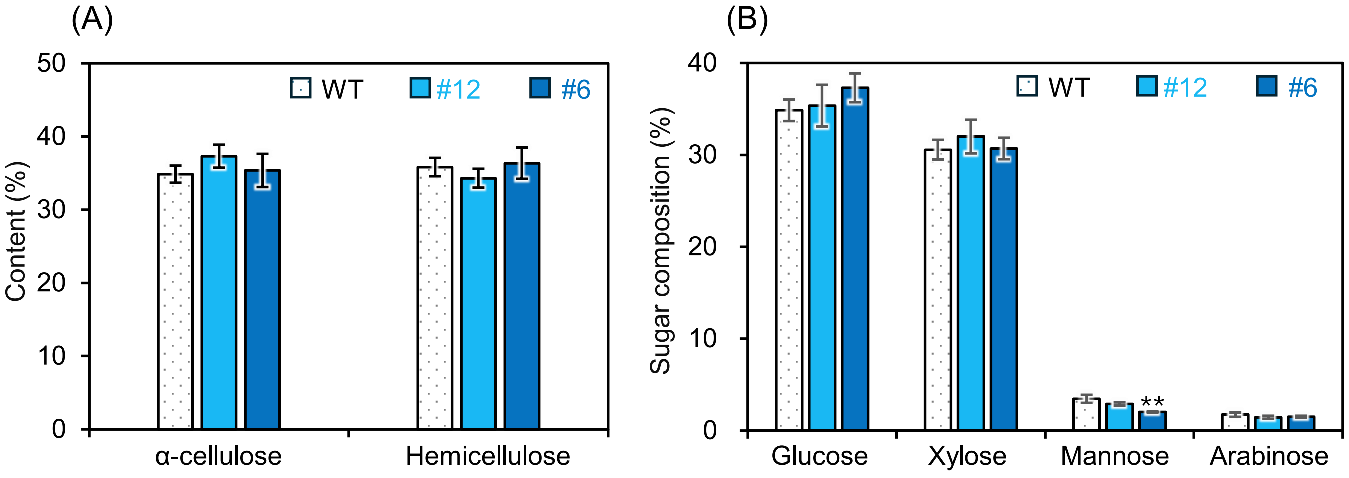

Supplementary Figure 5. Contents of α-cellulose, hemicellulose **(A)**, and monosaccharide composition **(B)** in cell wall residue prepared from plants grown in pots with soil. Values represent means with standard deviation from three independent biological replicates.

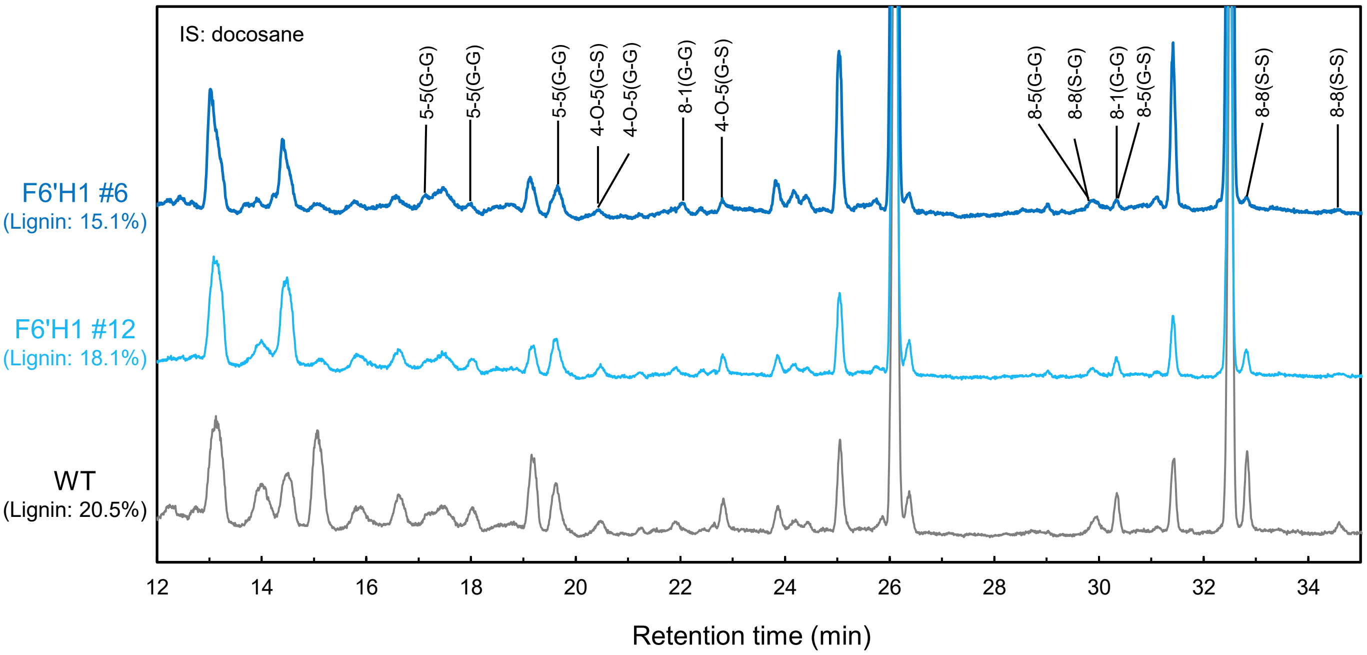

Samples	Yields of thioacidolysis dimers (μmol/g of lignin) (SS/GS/GG)					
	8-5	8-8	8-1	5-5	4-O-5	Total
WT	6.7 (0/3/97)	76.7 (100/0/0)	29.0 (3/0/97)	95.7 (0/0/100)	26.0 (0/86/14)	234 (27/16/57)
F6'H1 #12	6.0 (0/100/0)	9.3 (100/0/0)	8.1 (15/0/85)	41.7 (0/0/100)	7.0 (0/100/0)	72 (15/18/67)
F6'H1 #6	1.9 (0/100/0)	0.6 (100/0/0)	4.8 (4/0/96)	28.2 (0/0/100)	7.2 (0/100/0)	43 (2/21/77)

Supplementary Figure 6. Analysis of thioacidolytic dimers released from cell wall residue.

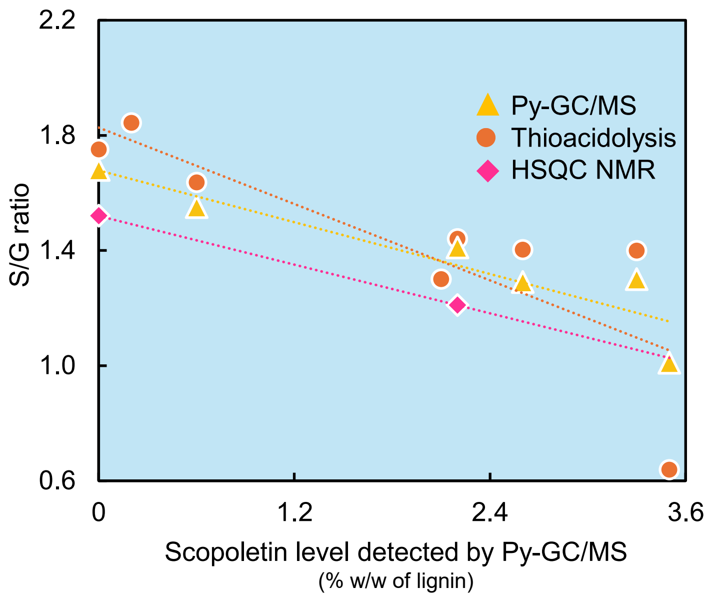

Supplementary Figure 7. Relationship between the S/G ratio and scopoletin content in different F6'H1 lines. Scopoletin levels in cell wall residues were estimated using Py-GC/MS, and S/G ratios were calculated from thioacidolysis, HSQC-NMR, and Py-GC/MS.

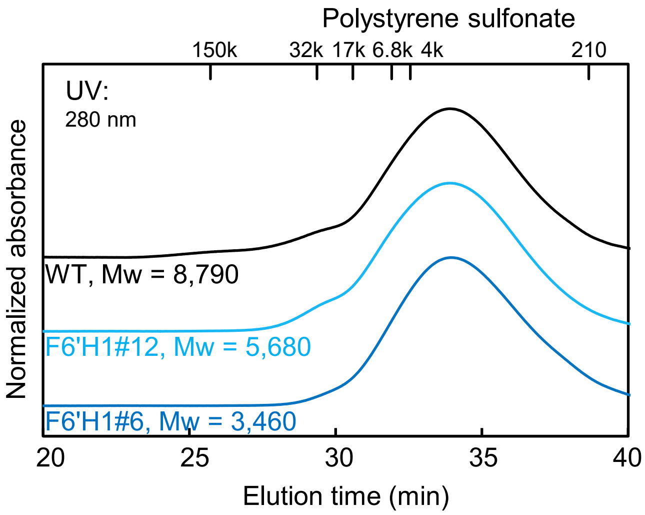

Supplementary Figure 8. Molar mass analysis of enzyme lignins using size exclusion chromatography. Polystyrene sulfonates with different molar masses were used to create a calibration curve.

Supplementary Figure 9. ^{1}H–^{13}C correlation HSQC-NMR spectra of enzyme lignins isolated from wild-type (WT, A and E) and F6′H1 (B and F) transgenic plants. Major well-separated correlation peaks are color-coded to their assigned structures. A scopoletin standard (C) was used to identify resonance peaks related to scopoletin incorporation in the F6′H1 (B) and difference (D) spectra. The difference spectrum (D) was obtained by 2D subtraction of the WT (A) from the F6′H1 (B) transgenic. Quantification was performed by volume-integration of correlation peaks; sidechain units are on the basis A + B + C = 100%; aromatics are on the basis S + G + H=100%; *p*HB (*p*-hydroxybenzoate) and Sc (scopoletin) quantification is on that S+G+H basis. HSQC-NMR, heteronuclear single-quantum coherence nuclear magnetic resonance (spectroscopy).

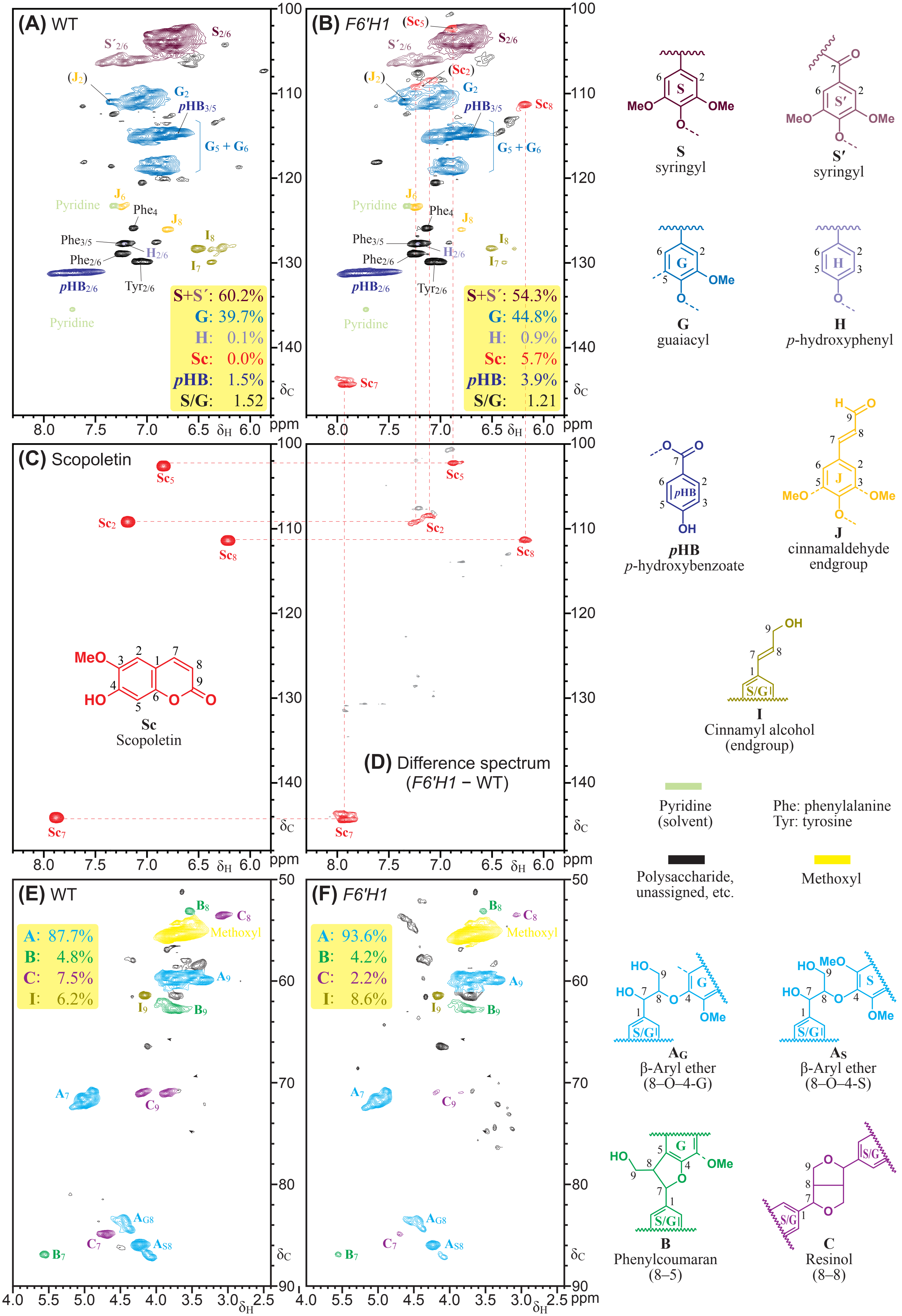
(A) WT
(B) F6'H1
(C) Scopoletin
(D) Difference spectrum (F6'H1 − WT)
(E) WT
(F) F6'H1
S+S´: 60.2%
G: 39.7%
H: 0.1%
Sc: 0.0%
pHB: 1.5%
S/G: 1.52
S+S´: 54.3%
G: 44.8%
H: 0.9%
Sc: 5.7%
pHB: 3.9%
S/G: 1.21
A: 87.7%
B: 4.8%
C: 7.5%
I: 6.2%
A: 93.6%
B: 4.2%
C: 2.2%
I: 8.6%
Pyridine
Methoxyl
Sc
Scopoletin
S
syringyl
S′
syringyl
G
guaiacyl
H
p-hydroxyphenyl
pHB
p-hydroxybenzoate
J
cinnamaldehyde endgroup
I
Cinnamyl alcohol (endgroup)
Pyridine (solvent)
Phe: phenylalanine
Tyr: tyrosine
Polysaccharide, unassigned, etc.
Methoxyl
AG
β-Aryl ether (8–O–4-G)
AS
β-Aryl ether (8–O-4-S)
B
Phenylcoumaran (8–5)
C
Resinol (8–8)

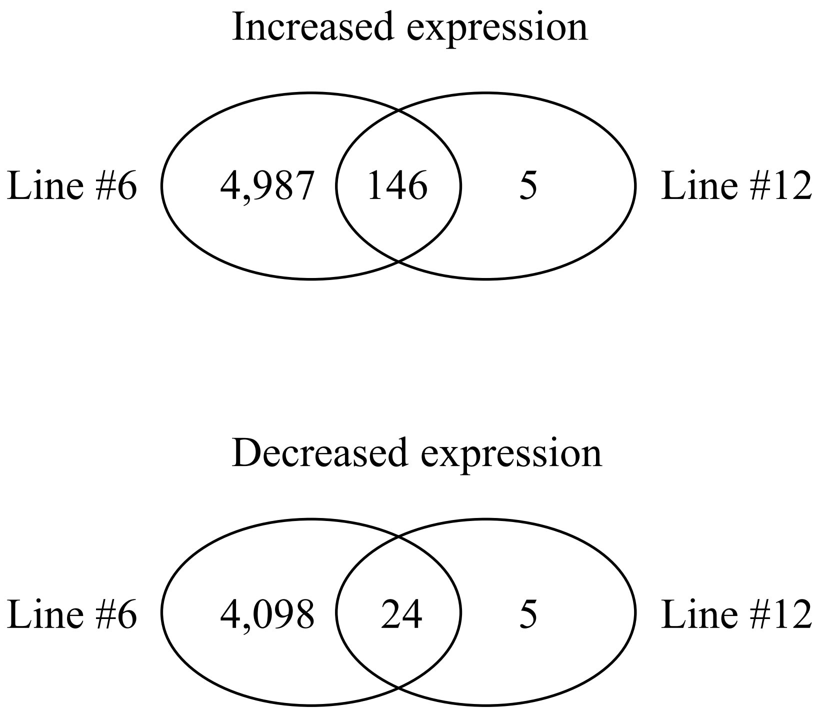

Supplementary Figure 10. Venn-diagram of the number of up- and down-regulated genes in line #6 and #12.

Supplementary Figure 11. MSMS-based structural characterization of the differential compounds in the *F6'H1* overexpression lines #6 and #12. The neutral loss of CO_2 , •CH_3 radical, and $C_6H_{10}O_5$ hints the presence of a carboxylic acid or ester, a methoxy on an aromatic system, and a hexose, respectively.

Scientific literature used to elucidate the structures of the compounds is given above the spectra:

- De Meester, B. et al. (2021) Vessel- and ray-specific monolignol biosynthesis as an approach to engineer fiber-hypolignification and enhanced saccharification in poplar. *Plant J* 108(3):752-765
- Hoengenaert, L. et al. (2022) Overexpression of the scopoletin biosynthetic pathway enhances lignocellulosic biomass processing. *Sci Adv* 8 (28), eabo5738.
- Morreel, K. et al., (2004) Profiling of oligolignols reveals monolignol coupling conditions in lignifying poplar xylem. *Plant Physiol* 136(3):3537-49
- Morreel, K. et al., (2010a) Mass spectrometry-based sequencing of lignin oligomers. *Plant Physiol* 153(4):1464-78
- Morreel, K. et al., (2010b) Mass spectrometry-based fragmentation as an identification tool in lignomics. *Anal Chem* 82(19):8095-10
- Vanholme R. et al., (2019) COSY catalyses *trans-cis* isomerization and lactonization in the biosynthesis of coumarins. *Nat Plants* 5(10):1066-1075

1 esculetin 3/4-O-hexoside, 6.12 min, $m/z_{experimental}$ 339.0719

Structural characterization as in Hoengenaert et al., 2022

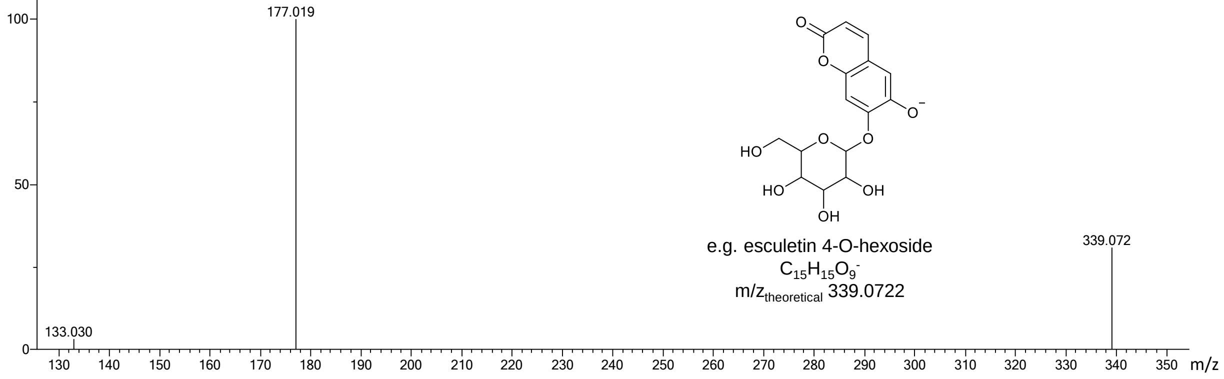

2 scopoletin, 10.77 min, $m/z_{experimental}$ 191.0343
(in the top twenty differential signals detected as the in-source fragment $m/z_{experimental}$ 176.0106)

Structural characterization as in Hoengenaert et al., 2022

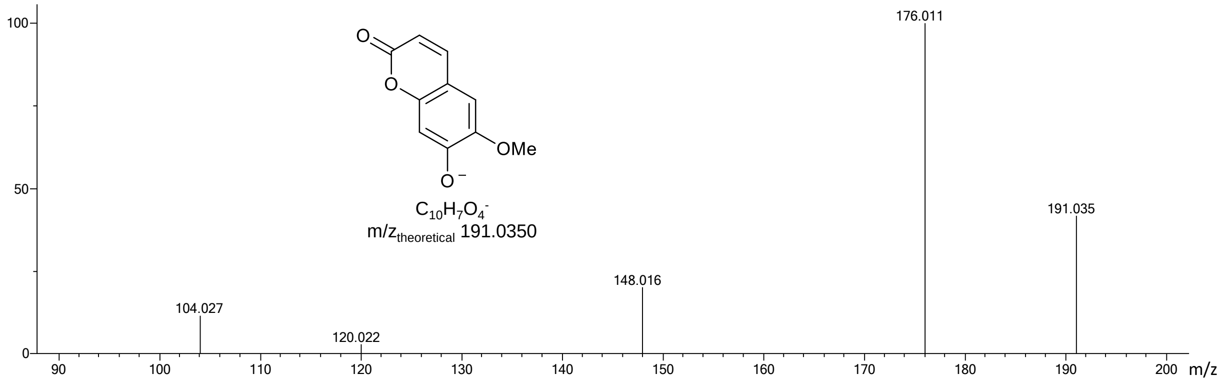

3 scopolin formic acid adduct, 7.37 min, $m/z_{experimental}$ 399.0931
(in the top twenty differential signals detected as the in-source fragment $m/z_{experimental}$ 191.0345)

Structural characterization as in Vanholme et al., 2019 and Hoengenaert et al., 2022

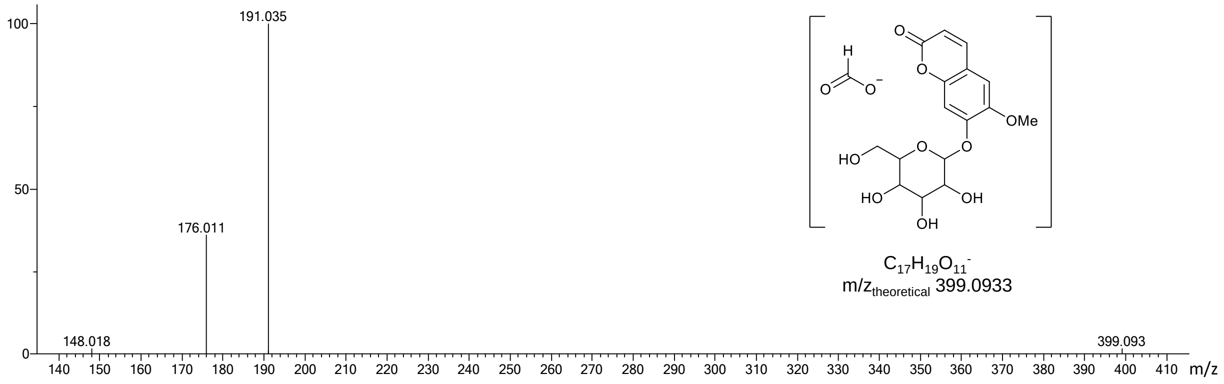

4 fraxetin 4/5-O-hexoside 1, 6.56 min, m/z$_{experimental}$ 369.0823

Structural characterization as in Hoengenaert et al., 2022

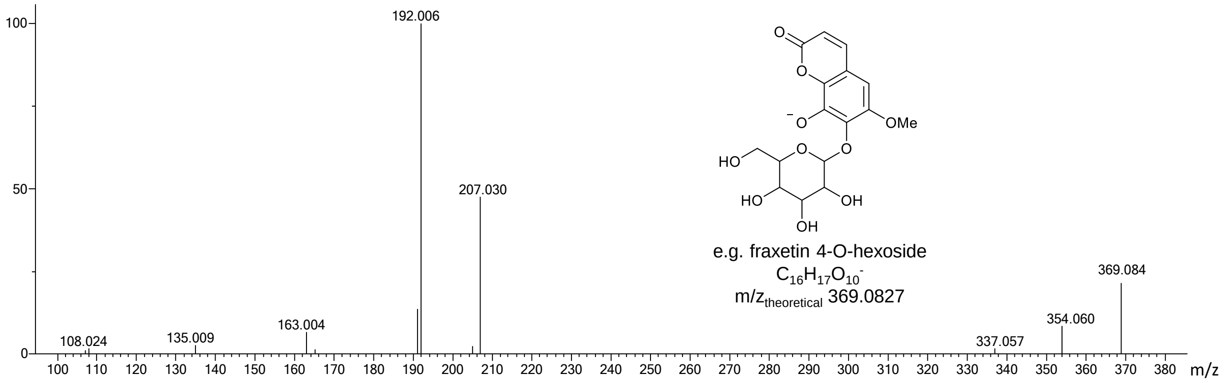

5 fraxetin 4/5-O-hexoside 2, 8.05 min, m/z$_{experimental}$ 369.0823

Structural characterization as in Hoengenaert et al., 2022

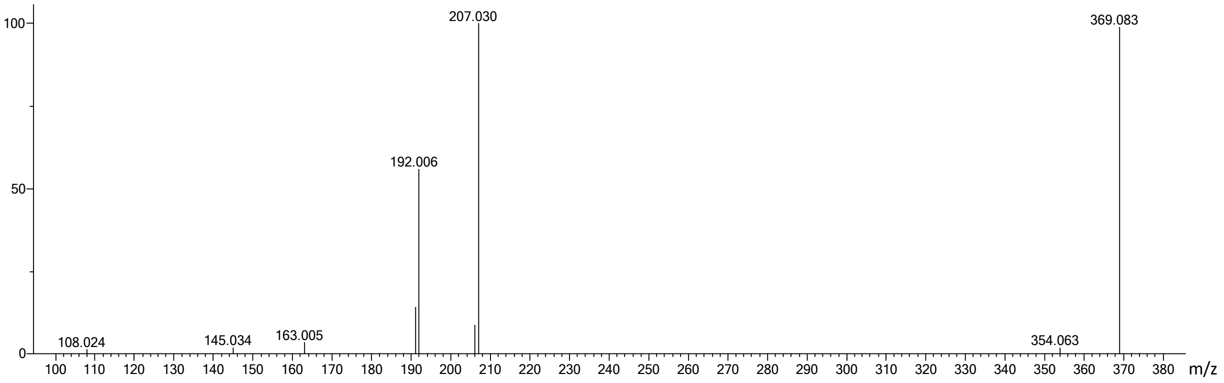

6 6-hydroxyferulic acid hexoside, 7.34 min, m/z$_{experimental}$ 371.0977

Structural characterization as in Vanholme et al., 2019

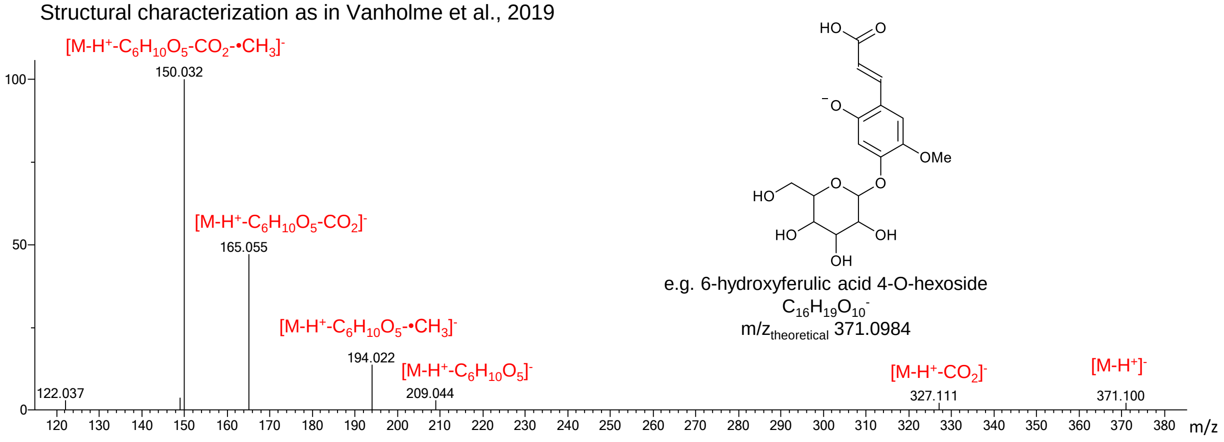

7 6-hydroxyferulic acid dihexoside formic acid adduct, 5.45 min, $m/z_{experimental}$ 579.1557
(in the top twenty differential signals detected as the homodimer $m/z_{experimental}$ 1067.3077)

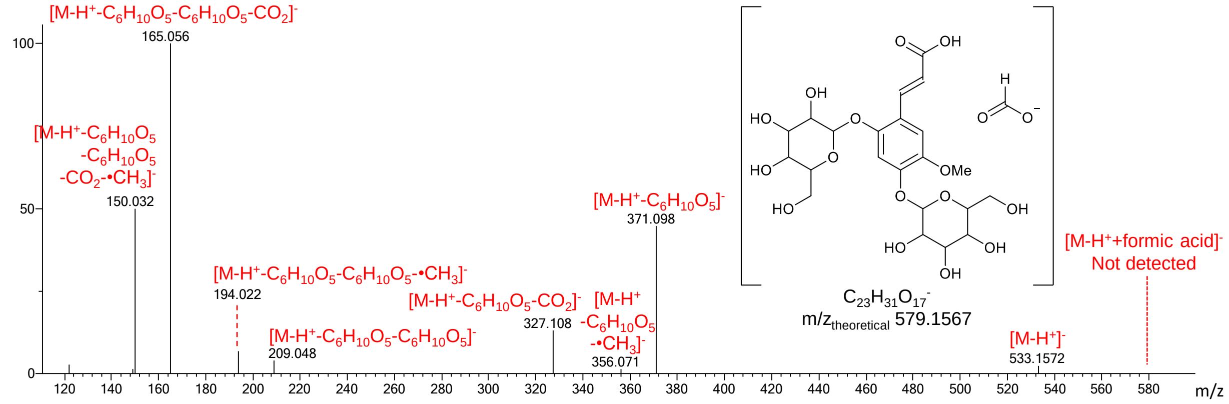

8 dihydro-6-hydroxyferulic acid + hexose 1, 6.32 min, $m/z_{experimental}$ 373.1132
Structural characterization as in Vanholme et al., 2019

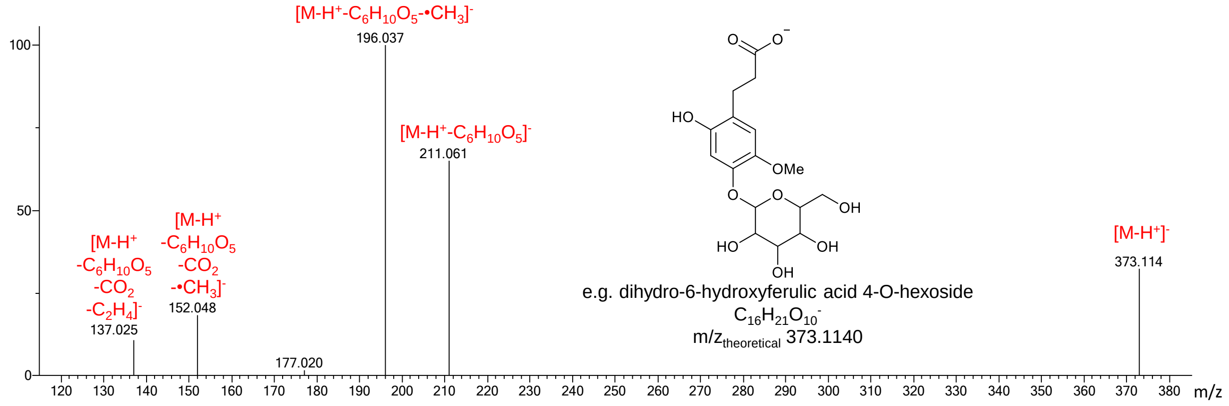

9 dihydro-6-hydroxyferulic acid + hexose 2, 6.70 min, $m/z_{experimental}$ 373.1129
Structural characterization as compound **8**. Only additional detected fragments are annotated in the spectrum below.

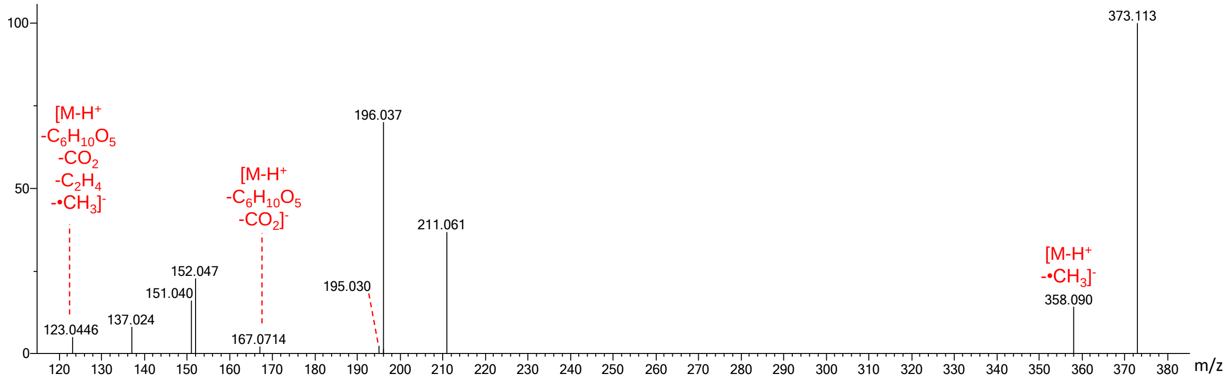

10 dihydro-6-hydroxycaffeic acid + hexose, 4.81 min, $m/z_{experimental}$ 359.0978

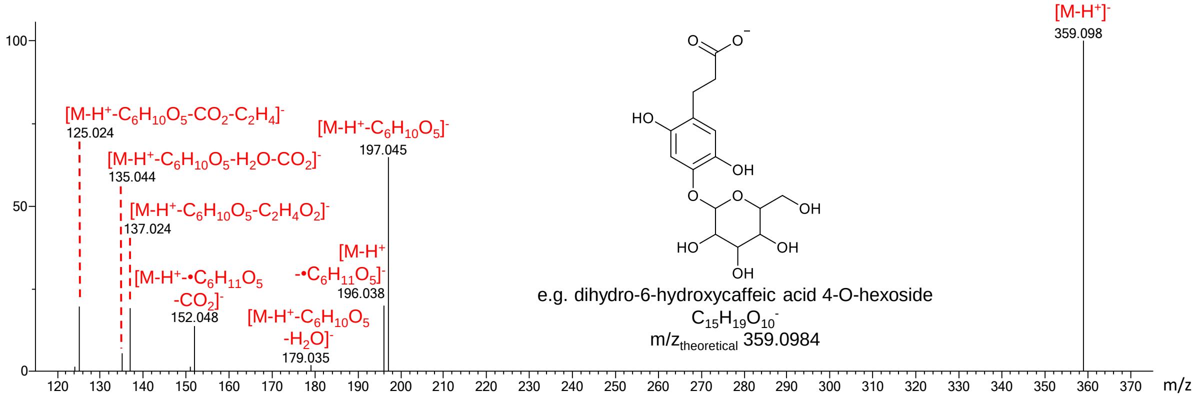

11 vanilic acid 4-O-hexoside, 4.63 min, $m/z_{experimental}$ 329.0872

(in the top twenty differential signals detected as the homodimer $m/z_{experimental}$ 659.1824)

Structural characterization as in De Meester et al., 2021

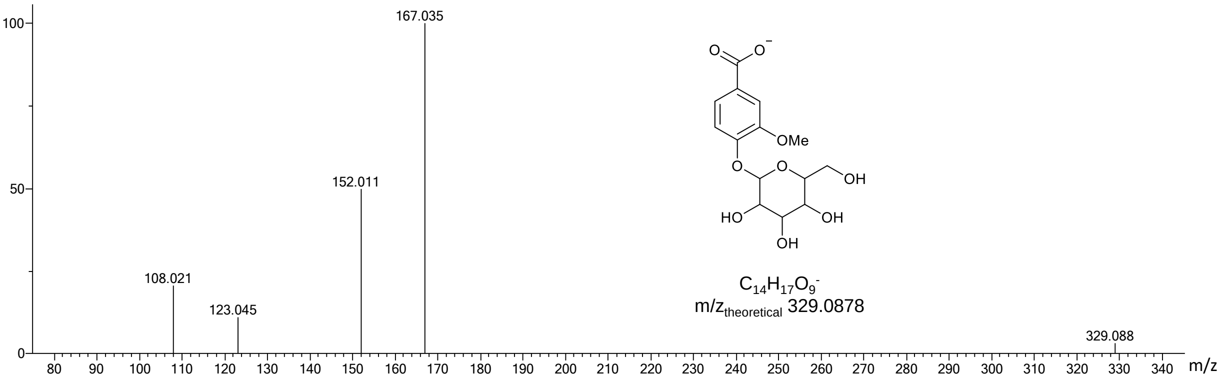

12 caffeic acid 3/4-O-hexoside, 6.88 min, $m/z_{experimental}$ 341.0878

Structural characterization as in De Meester et al., 2021

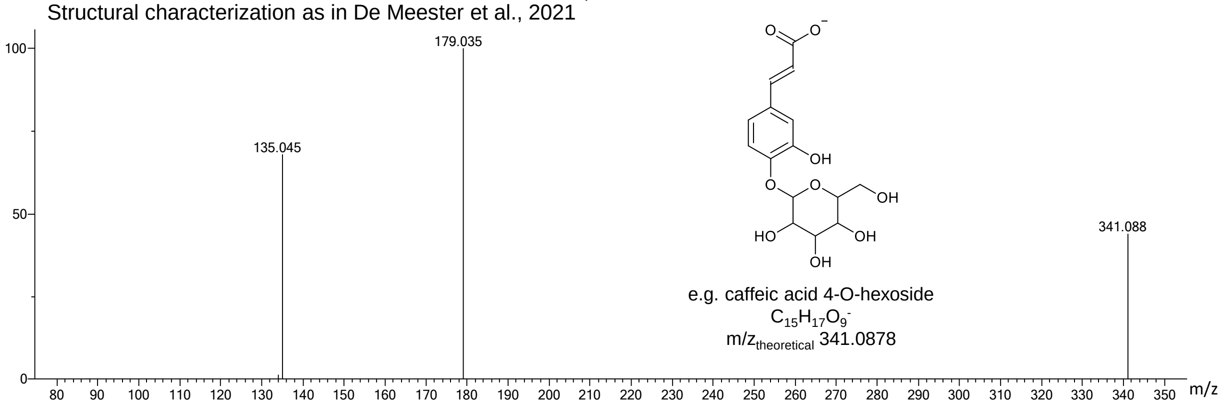

13 sinapic acid 4-O-hexoside, 7.28 min, $m/z_{experimental}$ 385.1122

Structural characterization as in De Meester et al., 2021

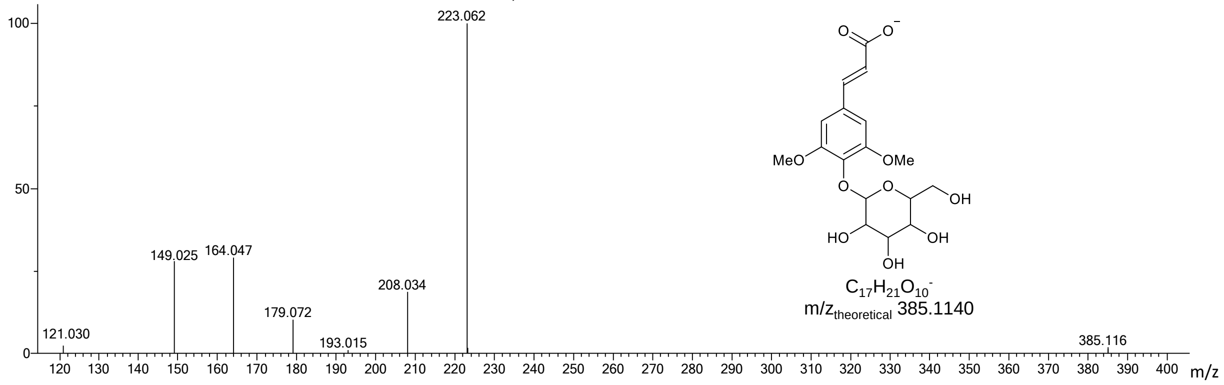

14 G(8-5)G, 15.62 min, m/z$_{experimental}$ 357.1341

Structural characterization as in Morreel et al., 2004, 2010a, 2010b

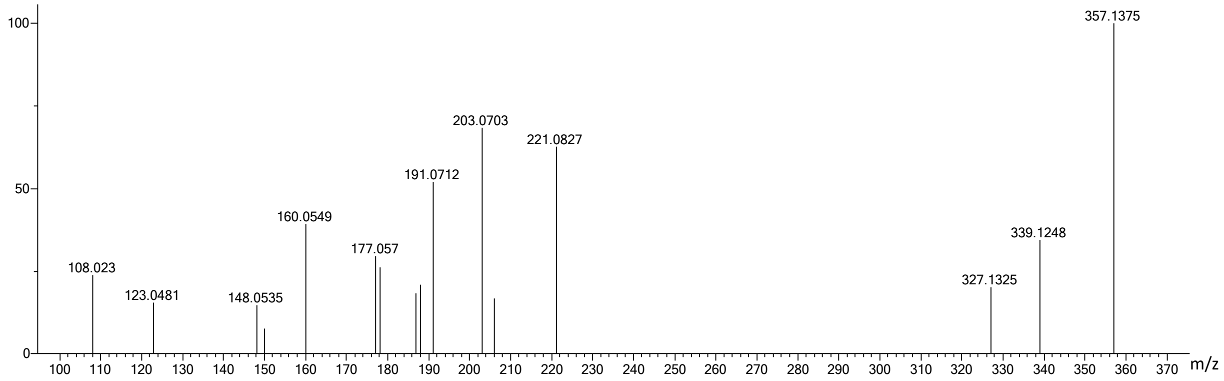

G(8-5)G-H_2O in-source fragment, 15.62 min, m/z$_{experimental}$ 339.1233

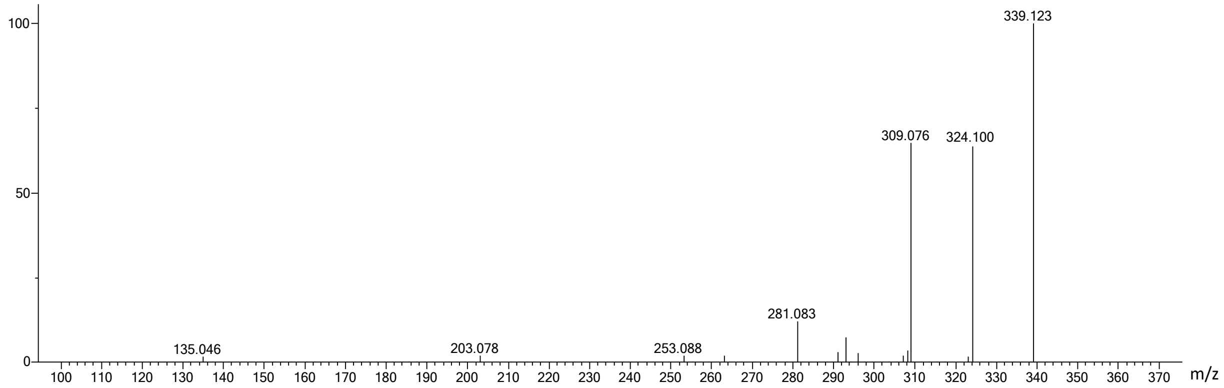

15 S(8-5)G-H_2O in-source fragment, 15.40 min, m/z$_{experimental}$ 369.1338

Structural characterization as in Morreel et al., 2004, 2010a, 2010b

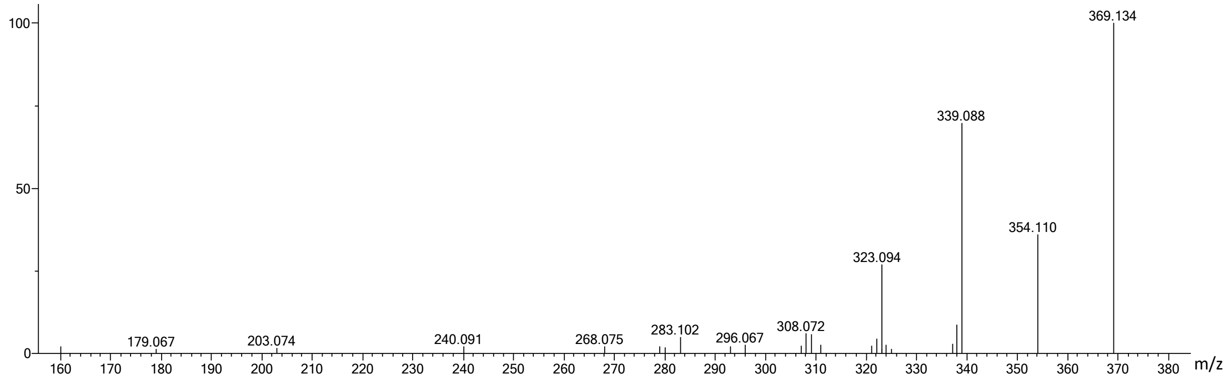

16 G(8-O-4)S^{glycerol} *p*-hydroxybenzoate, 13.63 min, m/z$_{experimental}$ 559.1816

Structural characterization as in Morreel et al., 2004, 2010a, 2010b

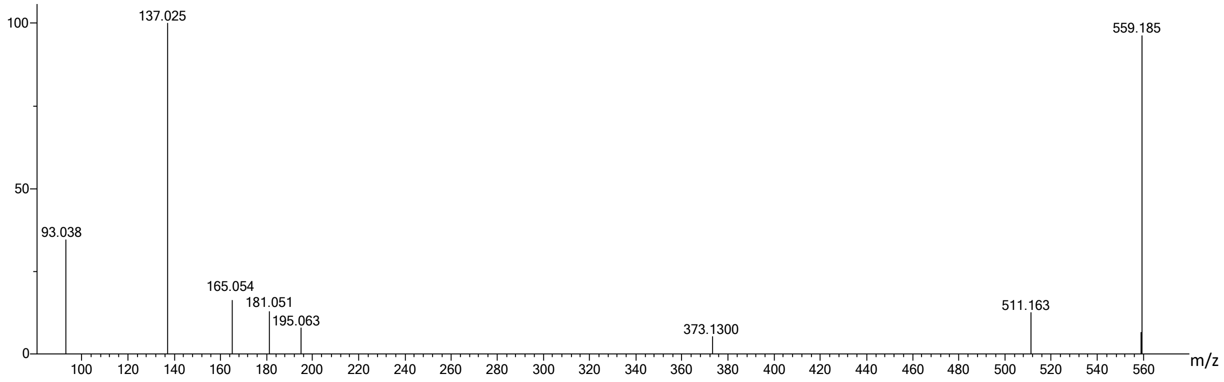

17 S(8-8)S *p*-hydroxybenzoate, 17.15 min, m/z$_{experimental}$ 555.1859

Structural characterization as in Morreel et al., 2004, 2010a, 2010b

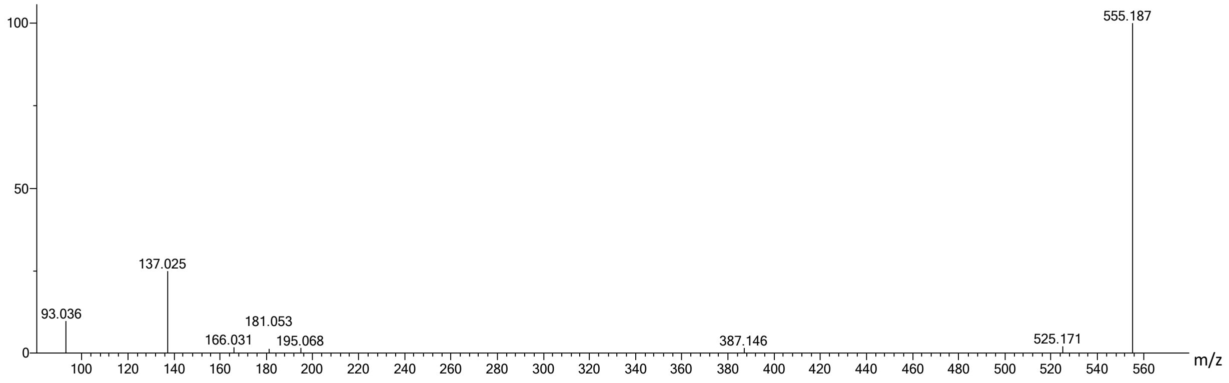

18 G(8-O-4)G(red8-5)G, 15.24 min, m/z$_{experimental}$ 555.2229

Structural characterization as in Morreel et al., 2004, 2010a, 2010b

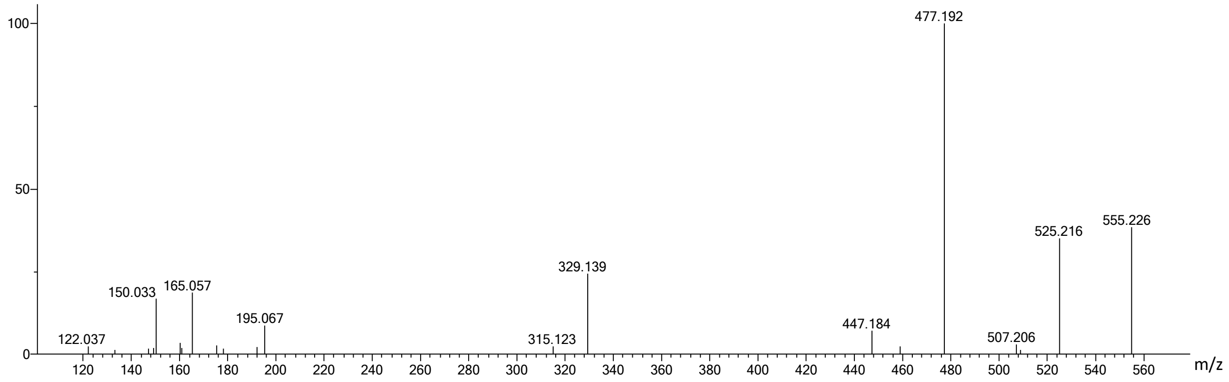

19 G(8-O-4)S(8-5)G 1, 17.05 min, m/z$_{experimental}$ 583.2174

Structural characterization as in Morreel et al., 2004, 2010a, 2010b

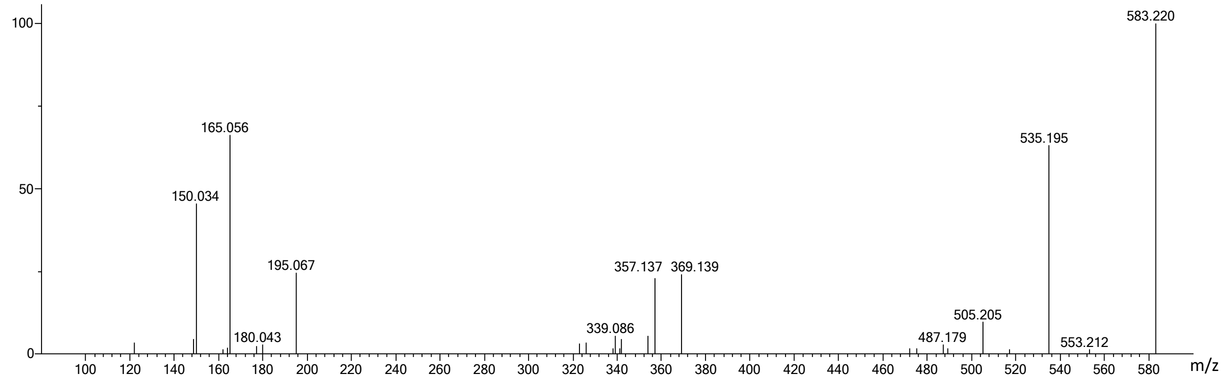

20 G(8-O-4)S(8-5)G 2, 17.81 min, m/z$_{experimental}$ 583.2180

Structural characterization as in Morreel et al., 2004, 2010a, 2010b

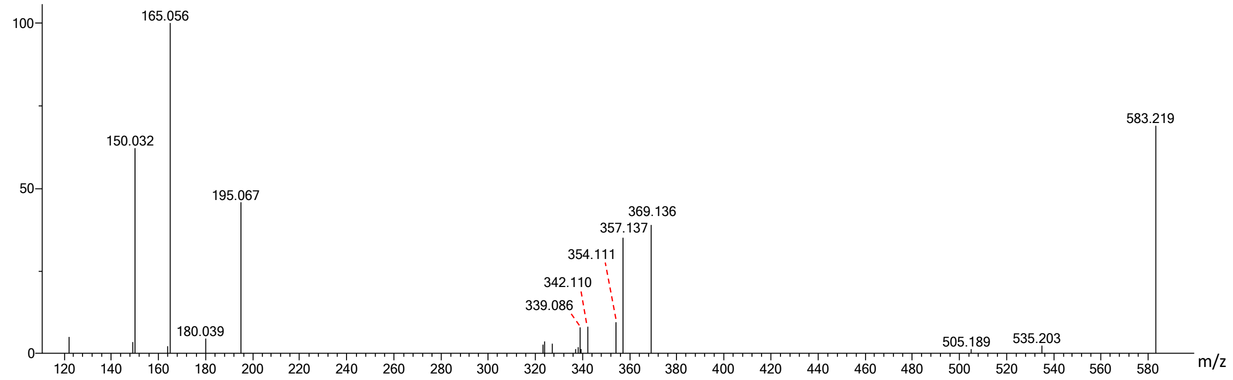

21 7-MeO-G(8-O-4)S(8-5)G, 20.24 min, m/z$_{experimental}$ 597.2334

Structural characterization as in Morreel et al., 2004, 2010a, 2010b

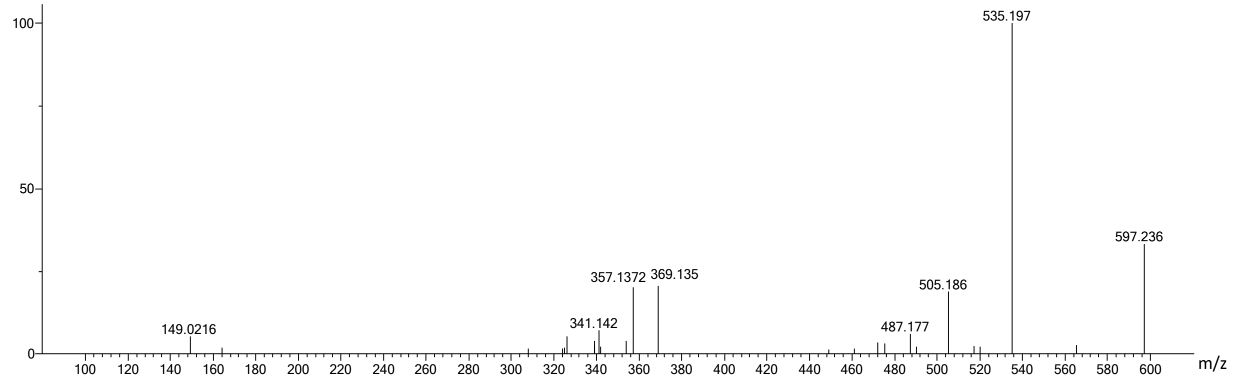

22 S(8-O-4)S(8-5)G, 16.81 min, m/z$_{experimental}$ 613.2281

Structural characterization as in Morreel et al., 2004, 2010a, 2010b

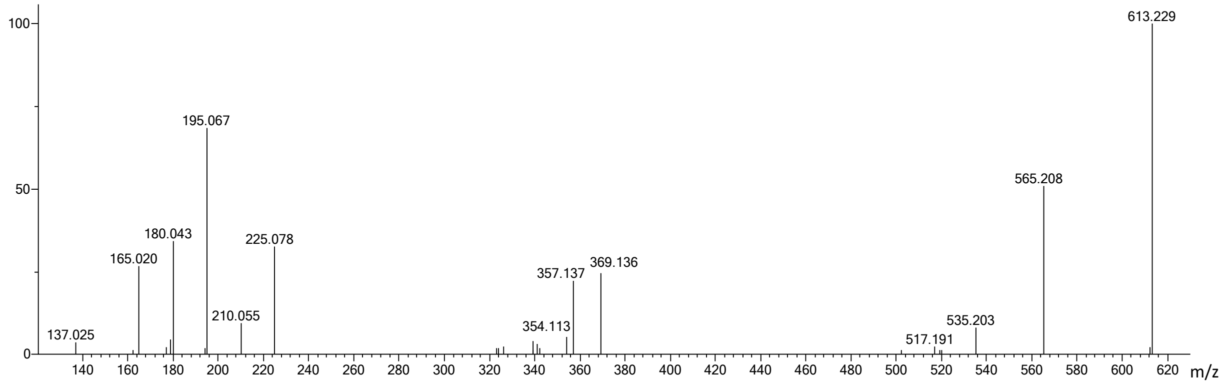

23 G(8-O-4)G(8-8)S, 19.91 min, m/z$_{experimental}$ 583.2179

Structural characterization as in Morreel et al., 2004, 2010a, 2010b

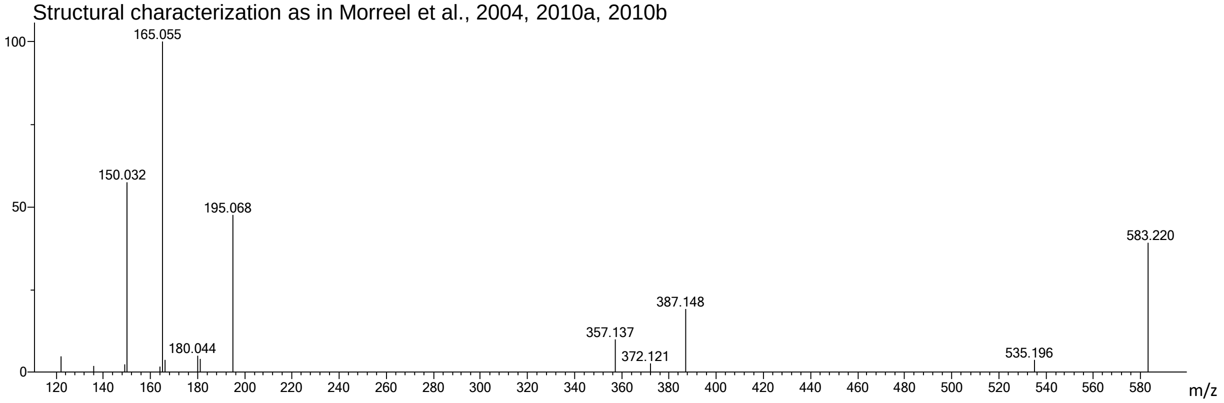

24 G(8-O-4)S(8-8)S 1, 18.82 min, m/z$_{experimental}$ 613.2283

Structural characterization as in Morreel et al., 2004, 2010a, 2010b

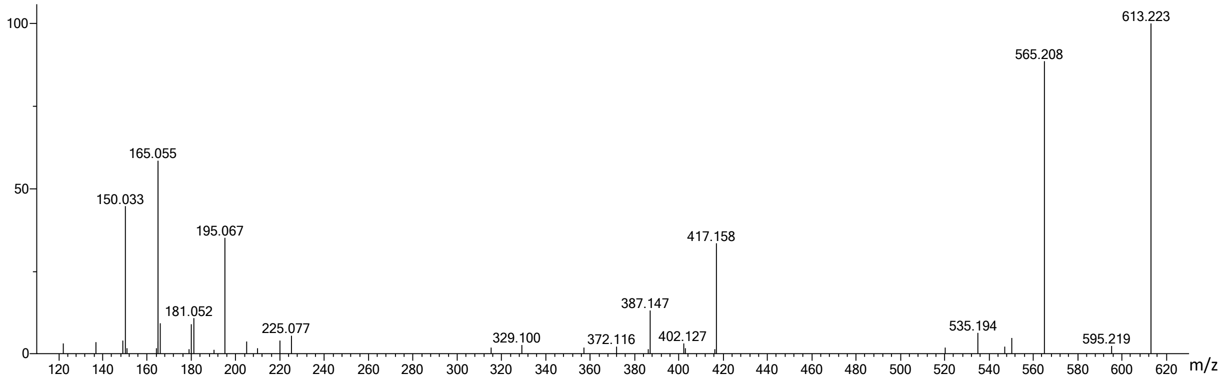

25 G(8-O-4)S(8-8)S 2, 19.60 min, m/z$_{experimental}$ 613.2286

Structural characterization as in Morreel et al., 2004, 2010a, 2010b

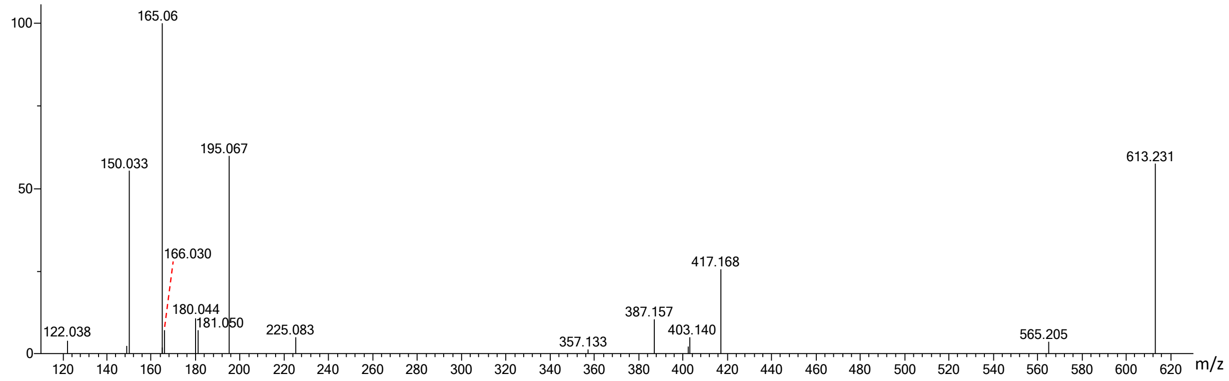

26 S(8-O-4)S(8-8)S, 18.53 min, m/z$_{experimental}$ 643.2385

Structural characterization as in Morreel et al., 2004, 2010a, 2010b

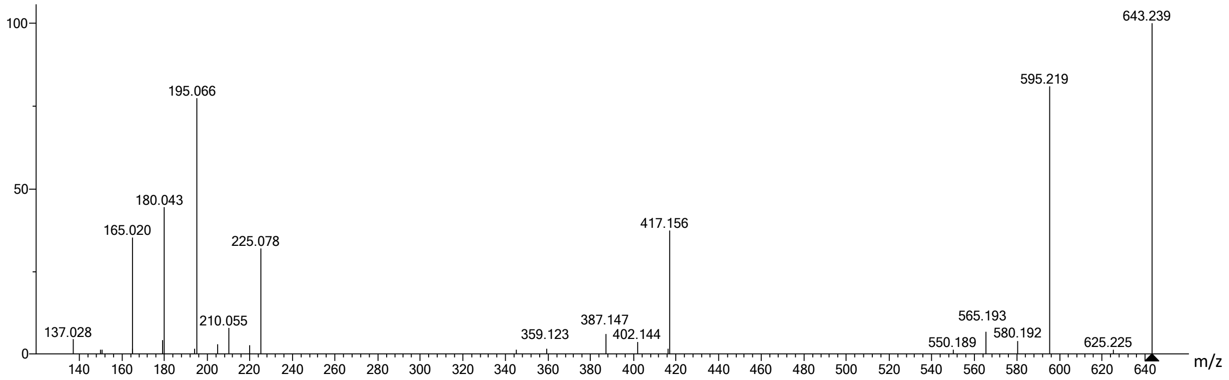

27 G(8-O-4)G(8-O-4)S(8-5)G, 17.20 min, m/z$_{experimental}$ 779.2912

Structural characterization as in Morreel et al., 2004, 2010a, 2010b

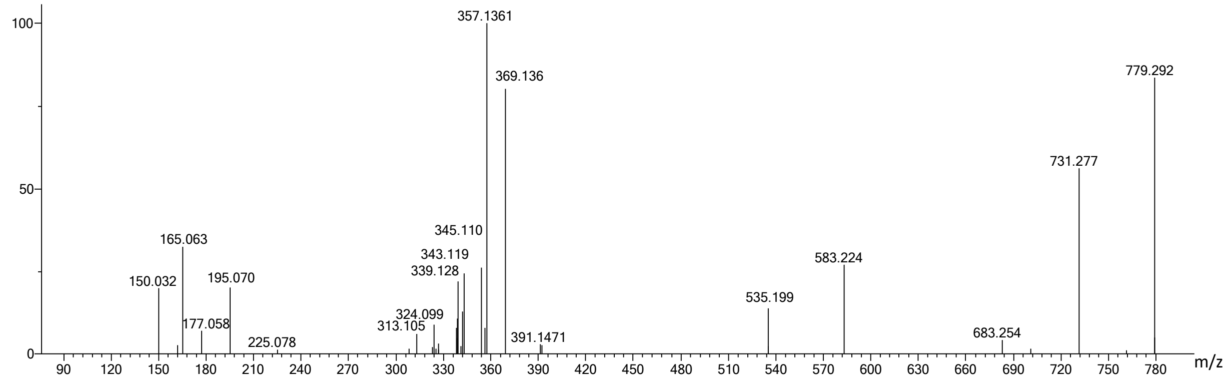

28 G(8-O-4)S(8-8)S(4-O-8)S, 19.66 min, m/$z_{experimental}$ 839.3128

Structural characterization as in Morreel et al., 2004, 2010a, 2010b

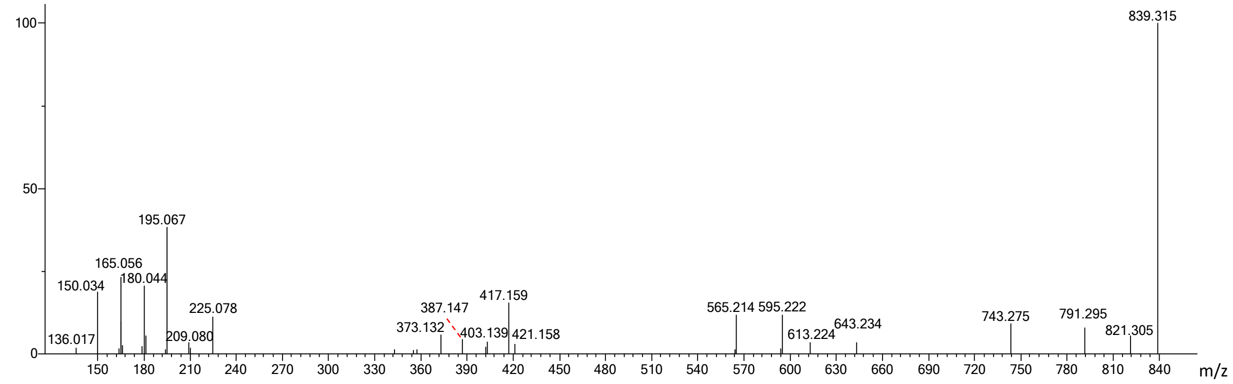

29 unknown 1, 8.40 min, m/$z_{experimental}$ 319.1393

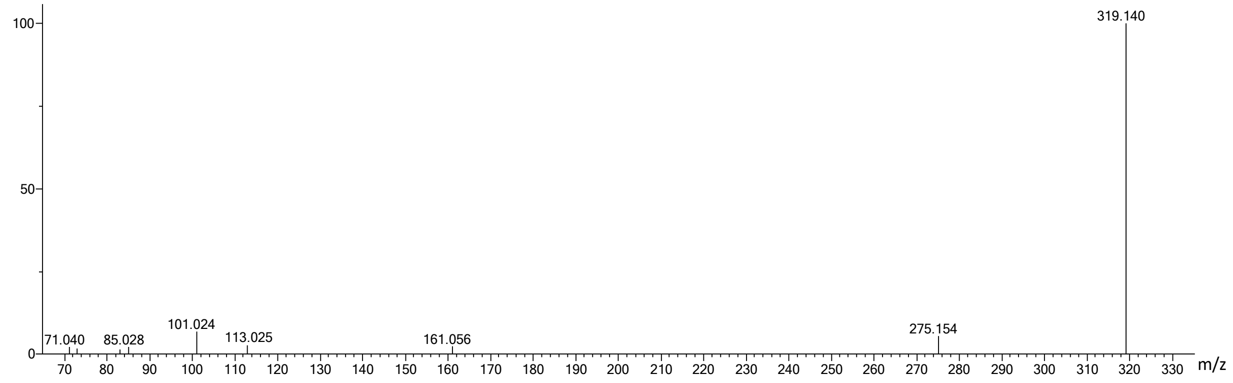

30 unknown 2, 9.16 min, m/$z_{experimental}$ 423.1660

(in the top twenty differential signals detected as in-source fragment m/$z_{experimental}$ 375.1444)

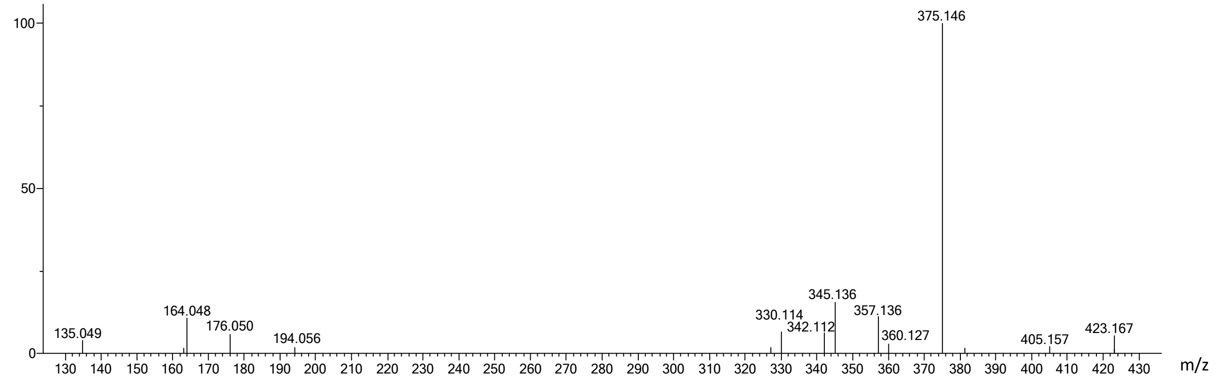

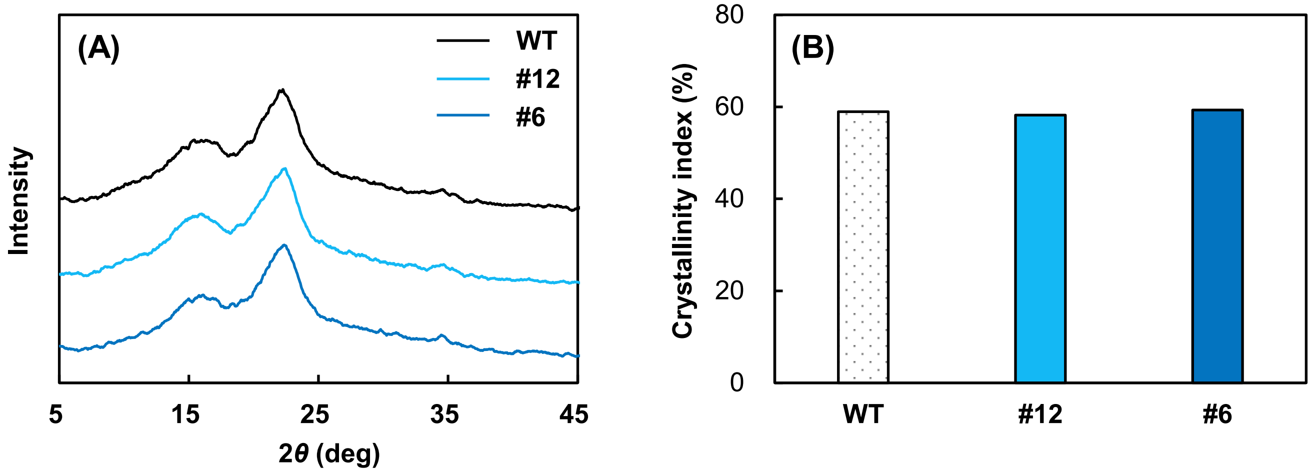

Supplementary Figure 12. (A) X-ray diffraction (XRD) patterns of extractive-free wood powder prepared from the WT and F6'H1 lines. (B) Crystallinity index was calculated using the spectral data.

Supplementary Table 1. Compounds detected in pyrolysates.

		WT	#12	#6	#11	#16
Scopoletin（%, w/w of lignin）		ND	0.6	3.3	2.2	3.5
Isofraxidin（%, w/w of lignin）		ND	0.1	0.3	0.2	0.2
G type product（%, w/w of lignin）	Guaiacol[1]	1.09	0.40	0.54	0.60	0.46
	4-Methylguaiacol[2]	0.46	0.31	0.22	0.27	0.06
	4-Vinylguaiacol[3]	1.20	0.59	1.03	0.89	0.81
	Isoeugenol[6]	0.97	0.55	0.42	0.50	0.24
	Coniferaldehyde[9]	0.23	0.35	0.23	0.29	0.07
	Coniferyl alcohol[10]	1.88	0.40	0.40	0.23	0.12
S type product（%, w/w of lignin）	Syringol[4]	2.38	1.00	1.18	1.25	0.69
	4-Methylsyringol[5]	1.15	0.47	0.40	0.48	0.22
	4-Vinylsyringol[7]	2.74	1.33	1.42	1.50	0.67
	4-trans-Propenylsyringol[8]	2.11	1.03	0.86	0.97	0.37
	Sinapaldehyde[11]	1.10	0.47	0.31	0.37	0.13
	Sinapyl alcohol[12]	2.09	0.16	0.15	0.09	0.03
S/G ratio	Py-GC/MS	1.7	1.6	1.3	1.4	1.0
	Thioacidolysis	1.8	1.6	1.4	1.4	0.6
Scopoletin index (scopoletin/coniferyl alcohol)		0	0.6	3.2	3.6	11.4
