# Supplementary material for: Effects of feruloyl-CoA 6′-hydroxylase 1 overexpression on lignin and cell wall characteristics in transgenic hybrid aspen
Source: Front Plant Sci. 2025 Mar 28;16:1543168. doi: 10.3389/fpls.2025.1543168 (PMC11985793; doi:10.3389/fpls.2025.1543168)
Supplement: Supplementary file 2 [file DataSheet2.pdf]

Supplementary table 2. RNAseq data comparing F6'H1 lines (#6 and #12) with wild type.

Potra	F6H1-6 log2FoldChange	F6H1-12 log2FoldChange	F6H1-6 padj	F6H1-12 padj	Ptrichocarpa_444_v3.1. annotation_info.txt transcriptName	Best-hit-arabi-name
Potra2c131s34673.1	1.257641718	2.302220102	0.249968772	0.72101822	Potri.013G138500.1	ATCG01280.1
Potra2c131s34674.1	1.372716579	2.454712646	0.233970415	0.72101822	Potri.013G143400.1	ATCG00860.1
Potra2c131s34675.1	1.269538987	1.960518266	0.3746635	0.856781867	Potri.013G143400.1	ATCG00860.1
Potra2c131s34676.1	3.002228097	3.124717598	0.002805243	0.389989789	Potri.013G143300.1	ATCG00830.1
Potra2c131s34677.1	2.848017165	2.951212375	0.001518655	0.391859375	Potri.011G074300.1	
Potra2c131s34678.1	3.066166413	2.950338571	0.000308524	0.406658442	Potri.013G140500.1	ATCG00790.1
Potra2c131s34679.1	2.937567206	2.897698509	0.000909122	0.443657809	Potri.013G136664.1	ATCG00780.1
Potra2c131s34680.1	2.918787179	2.902480635	0.000210605	0.382839636	Potri.013G140700.1	ATCG00770.1
Potra2c131s34681.1	3.318999714	3.224872228	1.00796E-05	0.269555635	Potri.013G136730.1	ATCG00750.1
Potra2c131s34682.1	2.551428618	3.08496942	1.18497E-08	0.221056825	Potri.011G095300.1	ATCG00580.1
Potra2c131s34683.1	1.956212596	2.554766765	0.055166375	0.558624809	Potri.016G094200.1	ATCG00540.1
Potra2c131s34684.1	2.076284388	2.648177507	0.00860352	0.58999816	Potri.016G094100.2	ATCG00530.1
Potra2c131s34685.1	2.163480376	2.931553154	0.010997147	0.34027789	Potri.005G150200.1	ATCG00500.1
Potra2c131s34686.3	2.886432524	3.344866677	0.002057827	0.310353725	Potri.013G141500.1	ATCG00380.1
Potra2c131s34687.1	2.320333087	2.456121844	0.005110055	0.551999152	Potri.016G089700.1	ATCG00350.1
Potra2c131s34688.1	1.97561791	2.33861465	1.36553E-08	0.056247855		
Potra2c131s34689.1	2.173944189	2.87150486	0.022662048	0.477503371	Potri.008G207200.1	ATCG00270.1
Potra2c131s34690.1	2.416545931	2.559373352	3.44279E-05	0.280832479	Potri.013G138300.1	ATCG00020.1
Potra2c499s35818.1	1.664088974	2.772792684	0.076102341	0.497568278		
Potra2c499s35819.1	2.80994679	3.313061213	0.000211159	0.365910084		
Potra2c499s35820.2	2.824301214	2.901074324	0.0044578	0.497568278	Potri.011G075100.1	ATCG00890.1
Potra2c499s35821.1	3.666899918	3.455041461	0.000115126	0.294409158	Potri.013G138900.1	ATCG01240.1
Potra2c499s35822.1	3.261760765	3.285316561	7.51883E-05	0.523216367	Potri.013G139100.1	
Potra2c499s35823.1	2.556612748	2.823605528	0.004371928	0.398418982	Potri.013G074700.1	ATCG01110.1
Potra2c499s35824.1	3.164432807	3.41985276	0.023144221	0.476972044	Potri.013G139100.1	
Potra2c499s35825.1	2.979913052	3.305490895	0.006605518	0.37476373	Potri.013G138900.1	ATCG01240.1
Potra2c499s35826.1	2.682837043	2.744138221	0.019328219	0.586953826	Potri.011G075100.1	ATCG00890.1

Potra2c499s35827.1	2.480398326	3.040406471	0.167661184	0.544330018		
Potra2c499s35828.1	1.788532793	2.585782955	0.192482948	0.832270846		
Potra2c499s35829.1	1.550326821	2.47712261	0.178572404	0.585428557	Potri.013G143400.1	ATCG00860.1
Potra2c499s35830.1	2.936367656	3.020221787	0.003860894	0.439251891	Potri.005G154300.1	ATCG00830.1
Potra2c868s36849.1	3.095347789	3.377430404	0.003272968	0.470156741	Potri.013G139100.1	
Potra2c868s36850.1	3.134019375	3.265377597	0.002616987	0.351047506	Potri.013G138900.1	ATCG01240.1
Potra2c868s36851.2	2.704746506	2.807263842	0.009882227	0.50924822	Potri.011G075100.1	ATCG00890.1
Potra2c868s36852.1	2.281243491	2.582170451	0.001508586	0.32064055		
Potra2c868s36853.1	2.224046079	2.693070087	0.116410621	0.863682136		
Potra2c868s36854.1	1.850986538	2.272906623	0.11228675	0.821709751	Potri.019G028400.1	ATCG00860.1
Potra2n1000s36919.1	-2.151847317	-0.397161678	0.035377861	0.895474078	Potri.005G014900.1	AT4G21390.1
Potra2n1000s36920.1	2.549191961	NA	NA	NA	Potri.005G018100.1	AT4G21390.1
Potra2n1014s36921.1	0.785935675	0.537968609	0.597354999	0.941260209	Potri.008G054000.1	AT5G02500.1
Potra2n1014s36922.1	NA	NA	NA	NA		
Potra2n1015s36923.1	0.216448625	-0.663588202	0.87992751	0.902080412	Potri.015G030100.1	AT1G48405.1
Potra2n1015s36924.1						
Potra2n1016s36925.1						
Potra2n1018s36926.1						
Potra2n1018s36927.1	NA	2.490806657	NA	NA		
Potra2n1018s36928.1						
Potra2n1019s36929.1						
Potra2n1019s36930.1					Potri.004G232250.1	AT5G49760.1
Potra2n1022s36931.4	-0.204398773	0.006315085	0.23114726	0.996154416	Potri.009G124900.1	AT2G21270.2
Potra2n1022s36932.1	1.305298912	0.031264912	4.33926E-11	0.9896961	Potri.004G163300.1	AT4G34710.2
Potra2n1024s36933.1					Potri.017G012200.3	AT4G12560.1
Potra2n1024s36934.1	NA	NA	NA	NA	Potri.017G012200.3	AT4G12560.1
Potra2n1025s36935.1	2.109441168	0.584656015	0.256009208	NA	Potri.011G016400.1	AT3G54070.1
Potra2n1025s36936.1	0.82357019	0.674963689	NA	NA		
Potra2n1028s36937.1	0.814611986	0.212122242	0.000747871	0.855611673	Potri.014G187300.1	AT3G07930.3
Potra2n1032s36938.1	5.376673166	1.746388982	1.26519E-29	0.508891091	Potri.001G043800.1	AT2G34930.1
Potra2n1034s36939.1						
Potra2n1034s36940.1						

Potra2n1037s36941.1						
Potra2n1039s36942.1	NA	NA	NA	NA		
Potra2n1041s36943.1	0.592329957	0.189705602	0.004882714	0.911339141	Potri.001G409800.1	AT5G54580.1
Potra2n1042s36944.1	1.545832463	1.363811008	NA	NA	Potri.013G133400.2	AT1G03670.1
Potra2n1048s36945.1	0.363960676	0.141718522	0.121439629	0.948577865	Potri.001G423700.1	AT3G20500.1
Potra2n1048s36946.1	-0.377971591	-0.01171602	0.242584743	0.995579852	Potri.001G423800.1	AT5G54890.1
Potra2n1049s36947.1	0.474052245	0.457176244	0.404219991	0.895129305	Potri.005G014700.1	AT4G21390.1
Potra2n1049s36948.1	-0.2878809	0.414454443	0.605927278	0.885375133		
Potra2n1049s36949.1					Potri.T038300.2	AT5G36930.2
Potra2n1049s36950.1	1.877781931	1.159346394	0.047184323	0.716548554	Potri.005G018300.1	AT4G21390.1
Potra2n1052s36951.1	5.080471277	2.86450839	0.003707535	NA	Potri.005G143800.1	AT4G37370.1
Potra2n1055s36952.1	-1.15865214	-0.565387819	5.64309E-05	0.140770927	Potri.019G073900.2	AT1G71500.1
Potra2n1055s36953.1	-0.282755109	0.405343444	0.932027938	NA	Potri.019G074000.1	AT3G14460.1
Potra2n1058s36954.1	NA	NA	NA	NA	Potri.016G072000.1	AT5G06300.1
Potra2n1059s36955.1	-0.481231027	-0.724918121	0.881357926	NA	Potri.017G137100.1	AT5G55490.1
Potra2n1059s36956.1	NA	NA	NA	NA	Potri.005G109700.1	AT1G23780.1
Potra2n1062s36957.1	1.64774763	1.363811008	NA	NA		
Potra2n1069s36958.1	0.426002995	0.576274064	0.480914254	0.906679354	Potri.005G014700.1	AT4G21390.1
Potra2n1069s36959.3	0.426002995	0.576274064	0.480914254	0.906679354	Potri.005G017800.1	
Potra2n1069s36960.1						
Potra2n106s34534.1					Potri.004G135500.1	AT1G07650.1
Potra2n106s34535.1	0.065628267	-0.00121454	0.872228996	0.999536469	Potri.001G155750.1	AT1G21280.1
Potra2n106s34536.1	0.22414696	0.08108921	0.340117101	0.962661188	Potri.001G155750.1	AT1G21280.1
Potra2n106s34537.1	4.842890073	2.33905615	1.17147E-06	NA		
Potra2n106s34538.1	1.352069243	0.25685656	1.03845E-08	0.842247476	Potri.018G136000.1	
Potra2n106s34539.2	1.427571027	0.355194774	1.88711E-10	0.716548554		
Potra2n106s34540.1	-0.827269434	-0.642505249	0.0019335	0.202843914	Potri.T045500.2	AT4G33420.1
Potra2n106s34541.1	-0.41990285	-0.071545308	0.466384244	0.98177802	Potri.007G000700.2	AT1G50410.1
Potra2n106s34542.1	-0.653436012	-0.079433953	0.440420824	0.985964146	Potri.018G135700.1	AT3G14460.1
Potra2n106s34543.1	-0.369188642	0.062115821	0.203485513	0.974367223	Potri.018G135700.1	AT3G14460.1
Potra2n106s34544.1	0.76711299	0.184660069	0.076929613	0.948577865	Potri.001G039600.1	
Potra2n106s34545.1	0.026126359	0.1492027	0.977980016	0.98409016		

Potra2n106s34546.2	-0.323993278	0.058609607	0.568672764	0.986636967	Potri.018G135800.1	AT3G14460.1
Potra2n106s34547.1	-0.085310549	0.070266529	0.790597083	0.982422288	Potri.T045400.1	AT4G15093.1
Potra2n106s34548.1	2.267300436	-1.465929376	0.404908978	NA		
Potra2n106s34549.1					Potri.004G117600.1	
Potra2n106s34550.1	-1.35927387	-0.097655521	NA	NA	Potri.004G135500.1	AT1G07650.1
Potra2n106s34551.1	NA	NA	NA	NA	Potri.T015000.1	
Potra2n106s34552.1	-0.804316301	-0.841653192	0.335900846	0.788065047	Potri.018G136100.1	AT3G14470.1
Potra2n106s34553.1	-0.310859842	0.761557049	0.903290649	NA	Potri.018G135800.1	AT3G14460.1
Potra2n106s34554.1	-0.081517123	-1.213779213	0.9478728	0.817452127		
Potra2n106s34555.1	NA	NA	NA	NA	Potri.T014800.1	AT1G07650.2
Potra2n1070s36961.1	NA	2.283425176	NA	NA	Potri.T103000.2	AT4G08850.1
Potra2n1070s36962.1					Potri.005G249100.1	AT2G17080.1
Potra2n1070s36963.1					Potri.002G012400.1	AT2G17080.1
Potra2n1070s36964.1					Potri.002G012300.1	AT5G44265.1
Potra2n1075s36965.1	1.056769652	0.151993677	NA	NA	Potri.008G223500.1	AT3G26300.1
Potra2n1075s36966.1						
Potra2n1075s36967.1	NA	1.571529889	NA	NA		
Potra2n1085s36968.1	NA	1.571529889	NA	NA	Potri.017G126000.1	AT1G66370.1
Potra2n10c20097.1	-0.146449889	0.005963377	0.126407577	0.995579852	Potri.010G255600.1	AT5G23450.1
Potra2n10c20098.1	-0.356720554	0.297895541	0.046644724	0.893333193	Potri.010G255500.1	AT5G08410.1
Potra2n10c20099.1	-0.447134324	-0.133050021	0.024980867	0.913709125	Potri.010G255400.1	AT1G61210.1
Potra2n10c20100.1	0.623086647	0.29320795	7.13755E-07	0.605147753	Potri.010G255200.1	AT5G08380.1
Potra2n10c20101.1	1.841366028	-0.658363516	0.130212364	NA		
Potra2n10c20102.1	0.026298713	0.030196828	0.86942748	0.979719961	Potri.010G255000.2	AT5G63670.1
Potra2n10c20103.1	-0.366394924	0.123519502	0.091619577	0.951326702	Potri.010G254900.1	AT5G08570.1
Potra2n10c20104.1	-0.148447895	0.115226322	0.487080943	0.953831244	Potri.010G254800.1	AT5G08540.1
Potra2n10c20105.2	0.430546327	0.133100188	0.060307037	0.930598202	Potri.010G254700.15	AT4G37930.1
Potra2n10c20106.1	1.573074789	0.535951499	3.72829E-14	0.551744322	Potri.010G254600.1	AT5G23395.1
Potra2n10c20107.4	-0.03485549	-0.027203957	0.689824732	0.967284786	Potri.010G254500.1	AT1G50370.1
Potra2n10c20108.1	0.074153947	0.046181135	0.479298981	0.957877578	Potri.010G254400.1	AT3G54540.1
Potra2n10c20109.1	1.64774763	1.571529889	NA	NA	Potri.010G254300.1	AT5G08560.2
Potra2n10c20110.3	0.03553068	0.072269514	0.843897285	0.951394085	Potri.010G254200.1	AT1G16780.1

Potra2n10c20111.1	0.017556491	0.102728769	0.939152929	0.934439727	Potri.010G254100.1	AT1G16790.1
Potra2n10c20112.1	9.617990596	6.563669251	1.62651E-17	0.011329548	Potri.010G254000.1	AT5G07610.1
Potra2n10c20113.1	9.617990596	6.526354797	1.62651E-17	0.011950008		
Potra2n10c20114.1					Potri.010G253900.1	AT3G20210.2
Potra2n10c20115.1	-2.295337707	-1.01793971	NA	NA	Potri.010G253800.1	AT2G24400.1
Potra2n10c20116.1	0.46829477	0.381089503	4.02284E-06	0.349381798	Potri.010G253700.1	AT1G16800.1
Potra2n10c20117.1	0.219822011	0.075190308	0.039847595	0.933996725	Potri.010G253500.1	AT1G78900.1
Potra2n10c20118.1	-0.277849216	-0.027049425	0.072311129	0.98177802	Potri.010G253400.1	AT1G16810.2
Potra2n10c20119.1	0.134889861	0.798982725	0.940308659	0.949966783	Potri.010G253300.1	AT1G50430.1
Potra2n10c20120.1	0.105571824	0.219856136	0.535222756	0.475986198	Potri.010G253200.1	AT1G50430.1
Potra2n10c20121.2	0.302494336	0.114244545	0.000528076	0.865591169	Potri.010G253100.1	AT2G03070.1
Potra2n10c20122.1	0.45652709	0.042821664	0.014976976	0.98177802	Potri.010G253001.1	
Potra2n10c20123.1	0.368059602	0.270703883	0.00060646	0.526356391	Potri.010G252900.1	AT1G50440.1
Potra2n10c20124.2	0.110743815	0.099249028	0.287236193	0.875861859	Potri.010G252800.10	AT1G16840.4
Potra2n10c20125.1	0.92641307	-0.767708831	NA	NA	Potri.010G252700.1	AT1G16850.1
Potra2n10c20126.1	-0.621991399	-0.301638907	0.000204658	0.773063484	Potri.010G252400.1	AT1G16860.1
Potra2n10c20127.1	-0.032915667	-0.080082876	0.877427237	0.927547456	Potri.010G252300.1	AT1G51560.1
Potra2n10c20128.1	0.154646054	0.426901284	0.791884344	0.874507876	Potri.010G252200.1	AT1G51550.1
Potra2n10c20129.1	-0.484810404	-0.118236075	0.000896219	0.903398621	Potri.010G252100.1	AT1G51540.1
Potra2n10c20130.1	0.351925576	-0.13807166	0.014592488	0.867193235	Potri.010G251900.1	AT1G71070.1
Potra2n10c20131.1	-1.162291196	-0.064867278	1.55425E-06	0.979822671	Potri.010G251800.1	AT3G21150.1
Potra2n10c20132.1	3.076036896	NA	NA	NA		
Potra2n10c20133.1	0.624440273	0.865951334	0.816532532	NA		
Potra2n10c20134.1	-0.086876605	0.024667791	0.558893765	0.985393296	Potri.010G251700.1	AT1G51580.1
Potra2n10c20135.1	0.036240116	0.11793909	0.897107376	0.936402128	Potri.010G251600.1	AT1G51600.2
Potra2n10c20136.1	NA	1.381953902	NA	NA	Potri.005G092500.1	AT4G14320.1
Potra2n10c20137.1	NA	NA	NA	NA	Potri.008G007500.1	AT1G51590.1
Potra2n10c20138.1	0.068421924	-0.024007899	0.602254792	0.977559521	Potri.010G251300.1	AT1G51610.1
Potra2n10c20139.1	-1.441537389	-1.471892015	0.122552166	0.579121694	Potri.010G251200.1	AT3G47570.1
Potra2n10c20140.1	-2.307432468	-2.456974026	NA	NA	Potri.010G251100.1	AT1G61566.1
Potra2n10c20141.1	4.273966086	2.569555825	4.97165E-38	0.001910782	Potri.010G251000.1	AT5G10695.1
Potra2n10c20142.1	0.800709334	0.215746849	1.22463E-06	0.842247476	Potri.010G250900.1	AT3G13580.3

Potra2n10c20143.1	NA	NA	NA	NA		
Potra2n10c20144.1	0.087600912	-0.435169565	0.979565066	NA		
Potra2n10c20145.1	-0.021327541	0.04371424	0.93508862	0.976302215	Potri.010G251700.1	AT1G51580.1
Potra2n10c20146.1	-0.112547437	0.213401881	0.91454696	0.937846405	Potri.010G251600.1	AT1G51600.2
Potra2n10c20147.1	2.203792851	0.371001886	3.16931E-11	0.442002381	Potri.010G250800.1	AT3G21180.1
Potra2n10c20148.1	-0.268098833	-0.330040385	0.309560718	0.772556533	Potri.010G250600.1	AT1G51630.1
Potra2n10c20149.1	1.239692581	0.882043139	0.000666568	0.368305629	Potri.010G250500.1	AT4G31540.1
Potra2n10c20150.1	-0.052342297	-0.100302512	0.736318468	0.903698951	Potri.010G250200.1	AT3G21200.1
Potra2n10c20151.1	-0.019033198	-0.152997682	0.906155656	0.824317405	Potri.010G250100.1	AT1G73090.1
Potra2n10c20152.1	-0.000121837	-0.159584977	0.999650925	0.834610342	Potri.008G008900.2	AT1G51650.1
Potra2n10c20153.1	-0.41526977	-0.181279981	0.001931378	0.862824894	Potri.010G249900.2	AT5G49830.1
Potra2n10c20154.1	-0.340892061	-0.181173493	0.00221902	0.805259903		
Potra2n10c20155.1	-0.07018168	0.122299606	0.622195226	0.880553952	Potri.010G249700.6	AT3G21215.1
Potra2n10c20156.1	-0.582207696	-0.369099403	0.010469319	0.842247476	Potri.010G249600.1	AT1G17745.1
Potra2n10c20157.1	-1.264824307	-0.425219097	0.001949101	0.814981587	Potri.008G009600.1	AT1G73050.1
Potra2n10c20158.1	-0.225399422	0.043668348	0.563512974	0.987226805	Potri.008G009700.1	AT1G55915.1
Potra2n10c20159.1	0.679682788	-0.015580952	0.002323258	0.991089517	Potri.010G249300.1	AT1G51660.1
Potra2n10c20160.1	-0.779746818	-1.02632046	NA	NA	Potri.001G414200.2	AT4G21380.1
Potra2n10c20161.2	0.651902121	0.102797128	1.10205E-05	0.93426898	Potri.010G249200.6	AT1G55730.2
Potra2n10c20162.1	0.736185737	0.040081603	0.001277645	0.985393296	Potri.008G010100.1	AT2G28690.1
Potra2n10c20163.1	0.553836602	0.169773719	3.7784E-07	0.662120811	Potri.010G248900.2	AT2G33840.1
Potra2n10c20164.1	-0.293373026	-0.089568135	0.37779281	0.976302215	Potri.010G248800.2	AT1G10865.1
Potra2n10c20165.1	-1.628999795	-0.303707607	6.12747E-11	0.372242801	Potri.010G248700.2	AT1G16880.1
Potra2n10c20166.1	-2.694005571	-1.425032364	NA	NA	Potri.010G248600.1	AT1G73120.1
Potra2n10c20167.1					Potri.T154800.1	AT1G13470.1
Potra2n10c20168.1	0.82357019	NA	NA	NA	Potri.012G075000.1	AT3G48150.1
Potra2n10c20169.1	-0.204370642	-0.580275391	0.849857113	0.910815461	Potri.010G248800.2	AT1G10865.1
Potra2n10c20170.3	-0.130318563	0.387235797	0.883262595	0.913577577	Potri.010G248600.1	AT1G73120.1
Potra2n10c20171.1	2.340207562	NA	NA	NA	Potri.T154800.1	AT1G13470.1
Potra2n10c20172.1	0.701774852	0.698484233	0.261655761	0.80889461		
Potra2n10c20173.1	-1.046355295	-0.525503533	0.20051617	0.903698951		
Potra2n10c20174.1	-0.552839342	-0.273097746	1.95106E-05	0.608586563	Potri.010G248100.1	AT3G56850.1

Potra2n10c20175.1	-0.142148118	0.020764441	0.631935929	0.991732307	Potri.001G186900.1	AT3G16260.1
Potra2n10c20176.1	-0.142938861	-0.094545504	0.172491408	0.930610374	Potri.010G247900.1	AT5G04990.1
Potra2n10c20177.1	5.48434323	2.878256876	0.007257323	NA	Potri.010G247800.1	AT5G04980.2
Potra2n10c20178.1					Potri.010G247750.1	
Potra2n10c20179.1	0.616173122	-1.49777686	0.810524805	NA	Potri.010G247700.1	AT3G10720.2
Potra2n10c20180.1	-1.521097258	-2.308489934	NA	NA	Potri.010G247600.1	AT3G10710.1
Potra2n10c20181.2	0.484162431	-0.160793578	0.143034863	0.939653927	Potri.010G247500.2	AT3G20770.1
Potra2n10c20182.1	-0.224606567	-0.159300086	0.845735739	0.985313207		
Potra2n10c20183.1	0.588994042	0.654156395	0.106020463	0.692457748	Potri.010G247400.1	AT3G10660.1
Potra2n10c20184.1	1.477758025	0.622132858	0.502551685	NA	Potri.008G011800.1	AT2G27110.2
Potra2n10c20185.1	-0.180557587	-0.077036645	0.533882988	0.966917036	Potri.010G247100.1	AT5G50350.1
Potra2n10c20186.1	0.050580094	-0.119225671	0.729468854	0.864223715	Potri.010G247000.1	AT3G10690.1
Potra2n10c20187.1	-1.349648418	-0.861430354	0.22195539	0.808716407	Potri.010G246800.1	AT5G17770.1
Potra2n10c20188.1	-1.690303153	-0.423149096	3.82976E-05	0.678727836	Potri.010G246800.1	AT5G17770.1
Potra2n10c20189.1	0.933112607	1.043185747	0.703585477	NA	Potri.005G035400.2	AT3G07040.1
Potra2n10c20190.1	0.677258199	0.237250972	0.000130048	0.900866967	Potri.010G246500.2	AT5G18440.1
Potra2n10c20191.1	0.142055145	0.111625432	0.245823097	0.888450013	Potri.010G246400.1	AT5G04920.1
Potra2n10c20192.1	NA	NA	NA	NA		
Potra2n10c20193.1	NA	NA	NA	NA	Potri.010G246900.1	AT1G32583.1
Potra2n10c20194.1	3.828742926	1.584736851	9.37752E-82	0.319620241	Potri.010G246200.1	AT5G04930.1
Potra2n10c20195.1	0.138024368	1.281703412	0.950798136	NA	Potri.010G246101.1	AT5G04930.1
Potra2n10c20196.1	0.050753811	0.090108122	0.761069366	0.933286951	Potri.010G246000.1	AT5G04895.1
Potra2n10c20197.1	-2.286316826	1.246119397	NA	NA		
Potra2n10c20198.1	-0.61910233	-0.767708831	NA	NA	Potri.010G245800.1	AT5G10380.1
Potra2n10c20199.2	NA	NA	NA	NA	Potri.010G245700.1	AT5G04890.1
Potra2n10c20200.1	0.492378831	-0.622352855	NA	NA	Potri.010G245600.1	AT5G04890.1
Potra2n10c20201.1	2.014656863	-0.767708831	NA	NA	Potri.010G245500.1	AT5G04890.1
Potra2n10c20202.1	0.857522442	0.207517672	8.88613E-06	0.903477395	Potri.008G013200.1	AT5G64140.1
Potra2n10c20203.1	0.992684894	0.150778572	0.00034264	0.953108412	Potri.010G245332.1	AT5G16010.1
Potra2n10c20204.1						
Potra2n10c20205.1	NA	NA	NA	NA		
Potra2n10c20206.1	2.315713269	1.381953902	NA	NA	Potri.010G245200.1	AT5G16010.1

Potra2n10c20207.1	0.109119549	0.1972715	0.151285254	0.475435867	Potri.010G245100.1	AT5G06600.1
Potra2n10c20208.1	-0.598125043	-0.105038747	0.00866931	0.963557834	Potri.010G244900.1	AT5G06390.1
Potra2n10c20209.1	0.346370691	0.08221672	0.001729512	0.932447954	Potri.010G244800.2	AT5G04870.1
Potra2n10c20210.1	0.072778022	-0.064466453	0.692742301	0.949485515	Potri.010G244700.10	AT3G10650.1
Potra2n10c20211.1	-0.023013838	0.033459878	0.925073436	0.981772014	Potri.010G244600.1	AT5G22040.1
Potra2n10c20212.1	-0.22957075	0.032993243	0.068655694	0.978751709	Potri.010G244500.2	AT4G32605.1
Potra2n10c20213.1	-0.757290251	-0.015246424	0.348035358	0.998482202	Potri.010G244400.1	AT2G36540.1
Potra2n10c20214.1	-2.791747448	-1.860956585	0.094146268	0.749416134	Potri.010G244400.1	AT2G36540.1
Potra2n10c20215.1	1.497318918	-0.138465075	8.74048E-12	0.935099709	Potri.010G244200.2	AT4G26190.1
Potra2n10c20216.1	-1.460235708	-0.23028682	7.12531E-08	0.813575895	Potri.010G244100.1	AT3G11760.1
Potra2n10c20217.1	-4.26223483	-1.260443962	0.042673065	NA	Potri.010G244000.1	AT5G61412.1
Potra2n10c20218.1	-0.026742065	-0.267436062	0.942659197	0.821709751	Potri.010G243900.1	AT2G38680.1
Potra2n10c20219.1	NA	2.0078969	NA	NA	Potri.010G243800.1	AT1G73970.1
Potra2n10c20220.1	-0.481398691	-0.411117774	0.340574343	0.838160646	Potri.010G243700.1	AT5G01420.1
Potra2n10c20221.1	-2.590456254	-4.115848242	0.303752086	NA	Potri.010G243600.1	AT5G20230.1
Potra2n10c20222.1	NA	NA	NA	NA	Potri.010G243500.1	AT5G06490.1
Potra2n10c20223.1	2.492428534	1.388295429	NA	NA	Potri.010G243200.1	AT5G06490.1
Potra2n10c20224.1						
Potra2n10c20225.1	0.710515743	0.417664982	0.000157787	0.635323636	Potri.010G243100.2	AT5G22060.1
Potra2n10c20226.1	0.045464055	0.157507924	0.935579527	0.951752151	Potri.004G066600.1	AT2G26790.1
Potra2n10c20227.1	-0.030952484	-0.033052677	0.885408522	0.986636967	Potri.010G242900.1	AT3G52060.2
Potra2n10c20228.2	-0.942210706	-0.550559208	3.50254E-09	0.420046656	Potri.010G242800.2	AT5G04840.1
Potra2n10c20229.1	-0.313775063	-0.22158544	0.043299163	0.777400198	Potri.010G242700.1	AT3G10640.1
Potra2n10c20230.1	0.488476482	0.028387243	0.01857354	0.985912891	Potri.010G242600.2	AT2G26230.1
Potra2n10c20231.1	0.437449129	-0.516992666	0.501216967	0.864649639	Potri.008G018200.2	AT3G10630.1
Potra2n10c20232.1	3.030808703	2.994412272	0.345122603	NA	Potri.010G242300.1	AT3G06260.1
Potra2n10c20233.1	0.078425879	-0.033727022	0.679357228	0.976302215	Potri.010G242100.1	AT3G10620.1
Potra2n10c20234.1	-0.182572968	-0.055646165	0.033531292	0.938501048	Potri.010G241800.1	AT5G03540.1
Potra2n10c20235.1	-1.518537527	-1.148173365	0.032854013	0.681756026	Potri.010G241600.1	AT3G52490.1
Potra2n10c20236.1	0.82357019	NA	NA	NA		
Potra2n10c20237.1	-1.555910594	-2.42671106	NA	NA	Potri.010G241500.1	AT5G04820.1
Potra2n10c20238.1	NA	0.674963689	NA	NA	Potri.010G241400.1	AT3G11600.1

Potra2n10c20239.1	-0.390418534	-0.215927708	0.00250514	0.784407529	Potri.010G241300.1	AT5G04810.1
Potra2n10c20240.1	-0.2818394	-0.026050968	0.472859324	0.9896961		
Potra2n10c20241.3	0.844221768	0.120345283	1.0251E-06	0.939653927	Potri.010G241100.1	AT5G04780.1
Potra2n10c20242.1	0.386564393	1.269226823	0.603188047	0.590087814	Potri.010G241000.2	AT5G04770.1
Potra2n10c20243.1	1.578268123	2.289452763	NA	NA		
Potra2n10c20244.1	1.217965664	-1.113930047	0.724205693	NA	Potri.010G240900.1	AT5G03700.1
Potra2n10c20245.1	1.703503024	0.246849298	0.000105187	0.863682136	Potri.010G240800.1	AT5G04760.1
Potra2n10c20246.1						
Potra2n10c20247.1	NA	NA	NA	NA	Potri.010G240700.1	AT1G02335.1
Potra2n10c20248.1	2.340207562	1.381953902	NA	NA	Potri.010G240600.1	AT3G62020.2
Potra2n10c20249.1	0.732368966	-0.05020672	NA	NA	Potri.010G240500.1	AT3G04200.1
Potra2n10c20250.1	-1.284453198	-0.001650488	0.441094386	NA		
Potra2n10c20251.1	-0.285422962	0.38986317	0.590003514	0.857606509	Potri.010G240300.1	AT3G52220.1
Potra2n10c20252.1	0.953815973	-0.061493505	1.86526E-05	0.973347974	Potri.010G240200.1	AT3G19330.1
Potra2n10c20253.1	1.385496292	-1.225305808	0.306521568	NA		
Potra2n10c20254.1	-0.052493647	-0.11727478	0.766141118	0.937789963	Potri.010G240000.2	AT2G38710.2
Potra2n10c20255.3	0.175193503	0.047665216	0.164460899	0.953831244	Potri.010G239900.1	AT3G54300.2
Potra2n10c20256.1	NA	NA	NA	NA	Potri.010G239800.1	AT5G22390.1
Potra2n10c20257.1	-1.190631415	-0.135758737	0.000289293	0.954400397	Potri.010G239700.1	AT3G54290.1
Potra2n10c20258.1	-1.313461801	0.100910145	0.024445032	0.983113084	Potri.010G239400.3	
Potra2n10c20259.1	0.977351803	0.113565576	7.70244E-05	0.959881296	Potri.010G239300.1	AT4G39200.1
Potra2n10c20260.1	0.483316422	-0.691219397	0.375391369	0.561968923	Potri.010G239200.3	AT4G18590.1
Potra2n10c20261.1	0.105151623	-0.195561159	0.618670808	0.805461106	Potri.010G239100.1	AT2G27510.1
Potra2n10c20262.1	0.829795609	0.121226431	2.849E-06	0.943483421	Potri.010G239000.2	AT4G24830.1
Potra2n10c20263.1	-1.274200156	0.754761043	NA	NA		
Potra2n10c20264.1	-0.91952342	0.944874477	NA	NA	Potri.010G238900.1	AT3G18650.1
Potra2n10c20265.1	-0.356218812	-0.304889708	0.136788626	0.715585895	Potri.010G238800.1	AT2G16710.1
Potra2n10c20266.1	-0.073241499	-0.031804602	0.678005206	0.981702181	Potri.010G238700.1	
Potra2n10c20267.1	-0.021400744	0.163796739	0.948891974	0.906545272		
Potra2n10c20268.9	-0.140283519	-0.000787129	0.159502706	0.999458638	Potri.010G238400.1	AT5G22450.1
Potra2n10c20269.1	-0.223030986	0.017748765	0.102855194	0.9869326	Potri.010G238500.1	AT3G11470.2
Potra2n10c20270.1	0.108908536	-0.036561798	0.426303023	0.974275337	Potri.010G238300.1	AT5G04740.1

Potra2n10c20271.1	-1.505721135	-1.026437031	0.119065839	0.807306793	Potri.010G238200.1	AT3G10080.1
Potra2n10c20272.1	NA	NA	NA	NA	Potri.010G238100.1	AT3G10080.1
Potra2n10c20273.1	0.524912879	0.045568249	0.017333883	0.97950444	Potri.010G238000.1	AT1G30240.2
Potra2n10c20274.1	1.567759439	0.122618928	1.53816E-15	0.930598202	Potri.010G237900.1	AT3G54250.1
Potra2n10c20275.1	0.71632103	-0.252260556	0.005909075	0.854980698	Potri.010G237800.2	AT2G36290.1
Potra2n10c20276.1	-0.537512694	-0.307260057	0.007648768	0.817452127	Potri.008G021400.1	AT2G36290.1
Potra2n10c20277.1	-1.324059873	-0.575651865	0.42377034	NA	Potri.010G237600.1	AT3G52770.1
Potra2n10c20278.1	-0.164455894	-0.124521783	0.130881155	0.836954885	Potri.010G237500.3	AT5G05100.1
Potra2n10c20279.1	1.824031643	1.752495167	0.064385724	0.485694701		
Potra2n10c20280.1	0.712606086	0.959457327	0.678528427	NA	Potri.008G220300.1	
Potra2n10c20281.1	-0.053297039	-0.091070306	0.723076853	0.875559093	Potri.010G237400.1	AT3G20970.1
Potra2n10c20282.1	-1.533582979	-0.772007247	0.000721004	0.378220766	Potri.010G237300.1	AT2G16980.2
Potra2n10c20283.1	-0.219610995	-0.187158425	0.120467066	0.773779643	Potri.010G237200.1	AT3G20930.1
Potra2n10c20284.4	-0.138476992	0.137827633	0.19495514	0.778425859	Potri.010G237100.3	AT2G27600.1
Potra2n10c20285.1	1.756814008	1.216686359	0.285155721	NA	Potri.010G237000.1	AT5G03610.1
Potra2n10c20286.2	NA	1.381953902	NA	NA	Potri.010G236951.1	AT5G03610.1
Potra2n10c20287.1	5.198155901	1.759189143	4.24236E-22	0.759797728	Potri.010G236800.1	AT5G03610.1
Potra2n10c20288.1	-0.623733154	0.398049906	0.042535715	0.835819291		
Potra2n10c20289.1	-0.729142297	0.3552316	0.01949949	0.874507876	Potri.010G236700.1	AT2G27690.1
Potra2n10c20290.1	0.430465502	0.338904801	0.055352509	0.650882346	Potri.010G236500.1	AT1G66240.1
Potra2n10c20291.1	NA	0.674963689	NA	NA		
Potra2n10c20292.1	-0.61910233	-0.767708831	NA	NA	Potri.010G236300.1	AT3G21510.1
Potra2n10c20293.1	-1.948931972	-0.525536909	5.62644E-09	0.651973886	Potri.010G236200.2	AT3G44610.1
Potra2n10c20294.2	-0.645101776	-0.29865405	0.000308524	0.781279942	Potri.010G236100.1	AT5G22510.1
Potra2n10c20295.1	0.02509492	0.248780045	0.932592436	0.833952007		
Potra2n10c20296.1	-0.185527496	-0.067409096	0.070846491	0.923761531	Potri.010G235900.1	AT5G04670.1
Potra2n10c20297.1	1.64774763	NA	NA	NA		
Potra2n10c20298.1					Potri.008G024400.1	AT1G66950.1
Potra2n10c20299.1	0.512081137	1.066897328	NA	NA	Potri.010G235800.1	AT2G36380.1
Potra2n10c20300.1	-0.161027561	0.184113239	0.259224891	0.788065047	Potri.010G235700.2	
Potra2n10c20301.1	-1.197593195	-0.29522076	2.24949E-05	0.885375133	Potri.010G235600.1	AT3G52900.1
Potra2n10c20302.3	0.557269752	0.240001978	9.83716E-06	0.726606341	Potri.008G025000.1	AT3G52240.1

Potra2n10c20303.1	NA	NA	NA	NA	Potri.010G235400.1	AT2G38790.1
Potra2n10c20304.1	-1.189362373	-0.379671048	2.15178E-11	0.379734246	Potri.010G235200.1	AT4G40070.1
Potra2n10c20305.1	NA	NA	NA	NA	Potri.010G235100.1	AT3G10560.1
Potra2n10c20306.1	0.685090663	0.465179206	0.000196715	0.475788609	Potri.010G235000.1	AT5G03680.1
Potra2n10c20307.1	-0.236504991	-0.071674332	0.248652616	0.953831244	Potri.010G234900.1	AT4G40045.1
Potra2n10c20308.2	0.565827721	0.30046778	0.000336724	0.696847835	Potri.010G234800.1	AT5G04600.1
Potra2n10c20309.1	1.151056633	0.447477493	0.107405304	0.937687311		
Potra2n10c20310.3	-0.721825664	-0.09786452	0.069505102	0.977559521	Potri.010G234700.1	AT5G03730.2
Potra2n10c20311.1	-0.017935814	0.216554518	0.936669792	0.816392132	Potri.010G234600.1	AT5G04610.1
Potra2n10c20312.1	0.051690021	0.314716523	0.816902013	0.739520512	Potri.010G234500.4	AT1G74580.1
Potra2n10c20313.1	2.511556279	1.961864545	0.23598526	NA		
Potra2n10c20314.2	1.095433266	0.401222598	6.23032E-13	0.839117844	Potri.010G234400.1	AT5G04560.1
Potra2n10c20315.1	0.210995232	-0.12684442	0.13031686	0.94728769	Potri.010G234300.1	AT3G57810.2
Potra2n10c20316.1	0.332091645	-0.151636624	0.11696655	0.943411075	Potri.008G026200.1	AT5G24910.1
Potra2n10c20317.1	-0.469189432	-0.232348323	0.731464017	0.977323635	Potri.010G234200.1	AT3G29034.1
Potra2n10c20318.2	-1.002629845	-0.350672422	4.87579E-06	0.885375133	Potri.010G234100.1	AT5G03760.1
Potra2n10c20319.1	0.202584698	0.017475308	0.144198524	0.992934677	Potri.010G234000.1	AT2G28600.1
Potra2n10c20320.2	-3.625754728	-0.918238773	4.48045E-16	0.771787573	Potri.010G233900.1	AT1G08230.2
Potra2n10c20321.1	-5.642457283	-0.947336625	0.000121556	0.833952007	Potri.008G026700.1	AT1G08230.2
Potra2n10c20322.1	-0.416334136	-0.415951739	0.001190339	0.401395702	Potri.010G233700.1	AT5G04550.1
Potra2n10c20323.1	-0.288112087	0.108208909	0.069747482	0.935474933	Potri.010G233600.1	AT3G10550.1
Potra2n10c20324.1	-0.295568702	0.060376963	0.080257366	0.967464191	Potri.010G233500.1	AT3G10030.1
Potra2n10c20325.2	0.048315872	-0.067581042	0.784869834	0.963878456	Potri.010G233400.1	AT3G52230.1
Potra2n10c20326.1	1.695488747	-0.075422326	5.64963E-12	0.972470034	Potri.010G233200.1	AT3G52990.1
Potra2n10c20327.1	0.232784527	0.158351048	0.2732466	0.920141992	Potri.010G233100.1	AT3G52210.3
Potra2n10c20328.1	0.056356516	-0.182776109	0.841588375	0.922011871	Potri.010G233000.1	AT5G04520.1
Potra2n10c20329.1	-0.197562198	0.158578036	0.366634113	0.935751704	Potri.010G232800.1	AT3G10540.1
Potra2n10c20330.6	6.074778622	1.979565016	2.89313E-12	NA	Potri.010G232700.3	AT5G04500.1
Potra2n10c20331.1	1.320303449	0.118950963	0.00848978	0.977559521	Potri.010G232600.2	AT5G04490.1
Potra2n10c20332.1	0.290569812	-0.047983388	0.127811939	0.976302215	Potri.010G232500.1	AT3G10530.1
Potra2n10c20333.1	1.563965933	0.674936573	2.77191E-16	0.303446787	Potri.010G232400.1	AT3G52190.1
Potra2n10c20334.1	1.64774763	NA	NA	NA	Potri.010G232300.1	AT5G22860.1

Potra2n10c20335.1	-0.087499018	0.073973705	0.615962406	0.940703488	Potri.010G232200.1	AT3G52180.1
Potra2n10c20336.1	-0.228307594	0.102339352	0.164455795	0.950878725	Potri.010G232000.2	AT5G04480.1
Potra2n10c20337.1	3.073325943	1.394613675	NA	NA	Potri.016G118500.1	AT2G29420.1
Potra2n10c20338.1	0.297483521	0.167273481	0.093680929	0.910878765	Potri.010G231900.2	AT5G58210.2
Potra2n10c20339.1	NA	1.571529889	NA	NA	Potri.010G231700.1	AT5G02420.1
Potra2n10c20340.1	-0.558225712	-0.159025354	0.071937382	0.955139324	Potri.010G231600.1	AT5G02420.1
Potra2n10c20341.1	9.938864602	2.611445458	9.37511E-16	NA	Potri.010G231500.1	AT2G36690.1
Potra2n10c20342.1	0.152462134	2.754754705	NA	NA		
Potra2n10c20343.1	0.864012265	0.11094256	0.000500771	0.940376196		
Potra2n10c20344.1	-0.075942882	0.331992233	0.633949968	0.322673812	Potri.010G231100.3	AT5G04460.1
Potra2n10c20345.1	-0.569647643	-0.129518823	0.057236597	0.956865545	Potri.010G231000.1	AT3G52790.1
Potra2n10c20346.2	-0.266232144	-0.061629342	0.143453079	0.954559968	Potri.010G230900.2	AT5G04440.1
Potra2n10c20347.1	0.975215155	-0.549010207	0.701188143	NA		
Potra2n10c20348.1	1.516550202	1.120452084	0.610089879	NA	Potri.010G230700.1	
Potra2n10c20349.1	0.163118339	0.013457285	0.305736559	0.99114586	Potri.010G230500.2	AT5G04430.1
Potra2n10c20350.1	-1.893491175	-0.037364163	7.62404E-05	0.993589787	Potri.010G230366.1	
Potra2n10c20351.1	0.119809516	0.050445875	0.260369793	0.969957134	Potri.010G230300.1	AT5G04420.1
Potra2n10c20352.1	-1.207268554	-0.66414769	0.546197289	0.88643982	Potri.008G031500.1	AT4G05160.1
Potra2n10c20353.1	2.685015931	NA	NA	NA	Potri.010G230200.1	AT1G20510.1
Potra2n10c20354.1	3.08710878	0.119583975	NA	0.9896961	Potri.010G230100.1	AT5G03610.1
Potra2n10c20355.1	0.658462499	0.260615019	0.336105428	0.960981446	Potri.010G230100.1	AT5G03610.1
Potra2n10c20356.3	0.158365972	0.081278501	0.030879521	0.890446513	Potri.010G229900.1	AT5G04410.1
Potra2n10c20357.1	-0.251577125	-0.148535941	0.022920839	0.79477084	Potri.010G229700.1	AT3G10480.1
Potra2n10c20358.2	-0.872682805	-0.007385665	0.000104194	0.99736971	Potri.010G229600.1	AT5G03530.1
Potra2n10c20359.1	NA	NA	NA	NA	Potri.008G032200.1	AT4G12420.2
Potra2n10c20360.1	-0.835312296	-0.411043136	0.23218545	0.921015963	Potri.010G229400.1	AT5G04390.1
Potra2n10c20361.1	0.275963977	-0.010939797	0.036527571	0.996032145	Potri.010G229300.1	AT5G04360.1
Potra2n10c20362.1	1.664677056	NA	NA	NA		
Potra2n10c20363.1	0.308984491	-0.036554755	0.392054253	0.987226805	Potri.010G229200.1	AT5G22930.1
Potra2n10c20364.1	0.157558117	0.124188382	0.338767145	0.880003199	Potri.010G229100.1	AT5G04320.2
Potra2n10c20365.2	-0.09923737	-0.221397868	0.68318406	0.887678043	Potri.010G229000.1	AT5G04310.1
Potra2n10c20366.1	-1.151382818	0.252961174	9.00277E-06	0.910815461		

Potra2n10c20367.1	-0.308240317	-0.079763934	0.011010397	0.903698951	Potri.010G228700.1	AT5G04280.1
Potra2n10c20368.1	-0.378464815	-0.204616104	0.067603448	0.824265661		
Potra2n10c20369.1	-0.433740205	-0.042486951	0.031443731	0.979719961	Potri.010G228600.1	AT3G10420.2
Potra2n10c20370.1	-0.642426663	-0.155932221	0.616323594	NA		
Potra2n10c20371.1	-0.727268913	-0.118140681	8.48602E-05	0.934579595	Potri.017G152200.2	AT3G47570.1
Potra2n10c20372.1	-0.705068893	-0.118389639	0.000234215	0.937789963	Potri.019G012400.1	
Potra2n10c20373.1	-0.652290725	0.126747285	0.001324227	0.936549944	Potri.010G228500.1	AT2G36840.1
Potra2n10c20374.1	5.076205433	2.878643434	0.004222853	NA	Potri.010G228400.1	AT3G54940.2
Potra2n10c20375.1					Potri.010G228100.1	AT1G11925.1
Potra2n10c20376.1	0.274794774	0.389724684	0.144948822	0.531258209	Potri.010G228000.2	AT5G03450.1
Potra2n10c20377.1	-0.615768645	0.845120723	0.809459732	NA	Potri.010G227900.1	AT1G67410.1
Potra2n10c20378.1	0.07058061	0.04857336	0.810662793	0.984292161	Potri.010G227800.1	AT3G54880.1
Potra2n10c20379.1	-0.4053388	0.055591096	0.002422977	0.972402328	Potri.010G227700.1	AT3G10410.1
Potra2n10c20380.1	1.564753971	1.696292695	0.304347987	NA	Potri.010G227600.1	AT3G10405.1
Potra2n10c20381.1	-0.43531579	-0.183622318	0.004753698	0.825511903	Potri.010G227500.1	AT3G10400.1
Potra2n10c20382.1	0.802434161	0.930596916	0.023340388	0.620763649	Potri.010G227500.1	AT3G10400.1
Potra2n10c20383.1	-0.363823127	0.265819867	0.02088472	0.816567218	Potri.010G227400.1	AT2G39170.1
Potra2n10c20384.1	-0.351936546	-3.026482152	0.932394948	NA	Potri.006G057900.1	AT2G42250.1
Potra2n10c20385.1	-3.166782164	-2.592248683	NA	NA	Potri.006G058000.1	AT2G42250.1
Potra2n10c20386.1	-0.06240049	0.040463529	0.906716188	0.988601759	Potri.010G227300.1	AT2G42250.1
Potra2n10c20387.1	-0.40175448	-0.070232818	0.078695509	0.968407862	Potri.010G227200.1	AT3G10390.1
Potra2n10c20388.1	0.037587032	0.090605289	0.736701148	0.902857376	Potri.010G227100.2	AT3G10380.1
Potra2n10c20389.1	0.20414664	1.592640616	0.748640242	0.389989789	Potri.010G227000.1	AT1G01950.1
Potra2n10c20390.1	0.415161389	0.205172695	6.4473E-05	0.729969215	Potri.010G226700.1	AT3G10370.1
Potra2n10c20391.1	-0.01775173	-0.034265251	0.90242472	0.974091403	Potri.010G226600.1	AT3G54860.1
Potra2n10c20392.1	0.164347433	-0.099987478	0.358153995	0.896306549	Potri.010G226500.1	AT3G54850.1
Potra2n10c20393.1	0.144012456	0.192901428	0.315403352	0.742084477	Potri.010G226300.2	AT3G54840.1
Potra2n10c20394.1	1.664677056	1.571529889	NA	NA		
Potra2n10c20395.1	1.459962178	NA	NA	NA	Potri.010G226200.1	
Potra2n10c20396.2	0.479503592	0.395884898	3.77097E-05	0.634528737	Potri.010G226100.10	AT5G04270.1
Potra2n10c20397.1	0.641848136	0.679645202	1.50648E-05	0.728883321	Potri.010G226000.2	AT2G39130.1
Potra2n10c20398.1	NA	0.674963689	NA	NA	Potri.010G225900.1	

Potra2n10c20399.1	-0.009699155	0.15730263	0.979901018	0.906107926	Potri.010G225600.2	AT3G10360.1
Potra2n10c20400.1	0.457222979	0.123562687	0.203164489	0.949485515	Potri.010G225400.1	AT5G04250.2
Potra2n10c20401.1	0.124994992	0.172567116	0.656901589	0.914551931	Potri.010G225300.3	AT3G09830.1
Potra2n10c20402.2	1.265371598	0.319573137	0.000190011	0.785747421	Potri.010G225200.1	AT1G53390.1
Potra2n10c20403.1	0.477382328	0.153088136	0.031030192	0.860325259	Potri.010G225100.1	AT3G10350.1
Potra2n10c20404.1	-2.337106966	-0.141149871	NA	NA	Potri.008G037200.2	AT5G41990.1
Potra2n10c20405.1	NA	NA	NA	NA	Potri.010G224900.1	AT3G57120.1
Potra2n10c20406.1	1.460196932	0.311034342	4.6053E-08	0.829716187	Potri.010G224800.1	AT2G39020.1
Potra2n10c20407.2	-0.249183518	0.045776016	0.142823273	0.967695582	Potri.010G224700.2	AT5G04240.1
Potra2n10c20408.1	2.476290371	NA	NA	NA	Potri.010G224600.2	AT3G60750.1
Potra2n10c20409.1	NA	NA	NA	NA	Potri.010G224500.1	AT2G37030.1
Potra2n10c20410.1	-0.236989841	-0.288255299	0.374365482	0.842247476	Potri.010G224300.2	AT5G03300.1
Potra2n10c20411.2	-0.133850296	-0.019525987	0.680839239	0.995579852	Potri.010G224100.1	AT2G37040.1
Potra2n10c20412.1	-0.533351736	-1.40363234	NA	NA		
Potra2n10c20413.1	-0.561837974	0.847823733	0.807443453	0.936890993		
Potra2n10c20414.1	-0.553736667	-0.188448251	0.134595858	0.924951347	Potri.007G030000.1	
Potra2n10c20415.1	-0.446997842	0.038235903	0.861801728	NA	Potri.010G160300.1	
Potra2n10c20416.1	0.136616615	0.183343815	0.726055962	0.953608209	Potri.010G223900.1	AT5G04220.2
Potra2n10c20417.1	-1.070608998	1.137964118	0.038155384	0.65293511		
Potra2n10c20418.1	-0.870306989	0.321223762	0.000163782	0.87679119	Potri.010G223766.1	AT2G38970.1
Potra2n10c20419.1	-0.083499663	-0.04592279	0.580662772	0.968979184	Potri.010G223700.1	AT4G14145.1
Potra2n10c20420.1	3.09680358	-0.378536483	5.82943E-07	0.972159498	Potri.010G223600.1	AT5G03250.1
Potra2n10c20421.2	1.120677982	0.298537047	1.45624E-15	0.660456866	Potri.008G038700.1	AT2G01720.1
Potra2n10c20422.1	-0.440422774	-0.515015239	0.66935334	0.93125845	Potri.010G223400.1	AT3G54800.1
Potra2n10c20423.1	-0.688683973	-0.527247591	2.53701E-05	0.375012853	Potri.010G223300.1	AT3G60530.1
Potra2n10c20424.1	-0.744004019	-0.211809605	1.59518E-05	0.692084498	Potri.010G223200.1	AT4G30080.1
Potra2n10c20425.1	0.158747665	-0.008947315	0.324900735	0.995203254	Potri.010G223100.1	AT5G42300.1
Potra2n10c20426.1	0.127923537	0.105759913	0.236214467	0.847981555	Potri.010G223000.1	AT5G17530.3
Potra2n10c20427.1	-0.102546808	-0.03359536	0.538211796	0.975578749	Potri.010G222900.1	AT2G39000.1
Potra2n10c20428.1	-0.095721529	0.107019361	0.497368851	0.870293488	Potri.010G222800.1	AT5G13570.1
Potra2n10c20429.1	-0.084518917	-0.368325733	0.670690912	0.628040312	Potri.010G222700.1	AT3G54820.1
Potra2n10c20430.1	-0.27771195	0.137864116	0.089021125	0.913622521	Potri.010G222500.1	AT1G13810.1

Potra2n10c20431.1	-0.31887507	0.205941058	0.172445916	0.874808359		
Potra2n10c20432.1	0.436898788	0.23634161	0.07434547	0.734273603	Potri.010G222400.1	AT5G49015.2
Potra2n10c20433.1	0.200875432	0.254352792	0.374488659	0.859892315	Potri.010G222300.1	AT5G19290.1
Potra2n10c20434.1	0.238498382	0.425429586	0.925365602	NA	Potri.010G222200.2	AT5G14990.1
Potra2n10c20435.1	NA	0.674963689	NA	NA		
Potra2n10c20436.1	-0.267387045	-0.164422438	0.02134285	0.788065047	Potri.010G222000.5	AT3G01390.2
Potra2n10c20437.1	-2.608054875	-0.927314061	0.00071508	0.672348168		
Potra2n10c20438.1	-2.447068316	-1.057148109	0.000237477	0.340356592	Potri.010G221900.10	AT5G19500.1
Potra2n10c20439.1	-1.007510724	-0.116208091	3.02557E-05	0.909675463	Potri.010G221700.1	AT2G39220.1
Potra2n10c20440.1	-0.095470521	-0.128617454	0.649523915	0.948577865	Potri.010G221500.2	AT2G28880.1
Potra2n10c20441.1	-0.091268709	-0.363614536	0.897107376	0.948577865	Potri.010G221400.1	AT1G16670.1
Potra2n10c20442.1	0.220493655	1.416805703	0.880849052	0.768791799		
Potra2n10c20443.1	0.379304446	1.50046037	0.812132795	0.773063484	Potri.010G221200.1	AT2G39180.1
Potra2n10c20444.1	-0.965217653	-0.554383136	1.15328E-06	0.054304482	Potri.010G221100.4	AT3G54890.1
Potra2n10c20445.2	-0.543643122	-0.055996078	4.26002E-08	0.948834049	Potri.010G221000.1	AT2G39190.2
Potra2n10c20446.1	-0.181654423	0.031277581	0.290714935	0.981772014	Potri.010G220900.1	AT3G07950.1
Potra2n10c20447.1	0.066508846	0.081401638	0.679127614	0.939653927	Potri.010G220700.1	AT2G37150.1
Potra2n10c20448.1	0.643817345	0.020767343	0.001872888	0.987288338	Potri.010G220600.1	AT3G20060.1
Potra2n10c20449.1	4.590163725	1.013091296	0.003365119	NA	Potri.010G220500.1	AT1G61560.1
Potra2n10c20450.1	4.007547412	0.469823058	2.45877E-07	NA	Potri.010G220500.1	AT1G61560.1
Potra2n10c20451.1	0.660156869	0.440708108	0.379700197	0.895474078	Potri.010G220300.1	AT3G12090.1
Potra2n10c20452.1	0.123805925	0.153967792	0.735847062	0.899853949	Potri.010G220100.1	AT3G07990.1
Potra2n10c20453.1	-0.085782549	0.022075356	0.410375206	0.981057849	Potri.010G220000.1	AT3G52250.1
Potra2n10c20454.1	-2.401232525	0.086720102	NA	NA		
Potra2n10c20455.2	0.252062144	-0.146340798	0.018435451	0.851898193	Potri.010G219900.1	AT4G25030.1
Potra2n10c20456.1	0.222745858	-0.177585778	0.470679915	0.917751882	Potri.010G219800.1	AT3G52270.1
Potra2n10c20457.1	-0.076449317	-0.054939545	0.614218623	0.950878725	Potri.010G219700.1	AT5G50840.2
Potra2n10c20458.1	0.050382638	0.108356221	0.722617461	0.889258723	Potri.010G219600.5	AT5G60410.2
Potra2n10c20459.1	0.558692974	0.048441105	4.9534E-06	0.974367223	Potri.010G218700.1	AT1G07930.1
Potra2n10c20460.1	0.055773629	-0.010676431	0.802084523	0.995005375	Potri.010G219200.1	AT2G34900.1
Potra2n10c20461.1	0.344107273	0.003556534	0.015186604	0.99819408	Potri.010G218700.1	AT1G07930.1
Potra2n10c20462.1	0.5212081	0.029697937	0.000167417	0.985393296	Potri.010G218700.1	AT1G07930.1

Potra2n10c20463.1	0.479081678	0.034215794	0.002359005	0.983944804	Potri.010G218600.1	AT1G07930.1
Potra2n10c20464.1	-1.820893546	-0.378880382	0.002548234	0.910477984	Potri.010G218500.1	
Potra2n10c20465.1	-4.00804407	0.150689648	0.15158716	NA	Potri.010G218400.1	
Potra2n10c20466.1	-0.530856863	-0.096871785	0.000289945	0.936549944	Potri.010G218100.1	AT5G03040.2
Potra2n10c20467.1	0.820588046	-0.277697834	0.170874408	0.935851032	Potri.010G218300.1	
Potra2n10c20468.1	NA	NA	NA	NA		
Potra2n10c20469.1	0.125598567	0.013994425	0.301889822	0.989878071	Potri.010G217900.1	AT2G35800.1
Potra2n10c20470.1	0.222672427	0.04901729	0.055478389	0.969421568	Potri.010G217800.1	AT3G52300.1
Potra2n10c20471.1	3.71249492	0.697227639	4.1237E-33	0.858481608	Potri.010G217700.1	AT1G07900.1
Potra2n10c20472.1	-0.060501687	0.000821436	0.727687564	0.999600335	Potri.010G217600.1	AT2G16880.1
Potra2n10c20473.1	0.595405371	0.183787475	0.000164603	0.755774501	Potri.010G217500.6	AT2G35830.2
Potra2n10c20474.1	0.333774984	0.075346214	0.005267602	0.923397137	Potri.010G217400.2	AT5G63220.1
Potra2n10c20475.1	-0.120344895	-0.285478887	0.785949404	0.880477186	Potri.010G217300.1	AT5G51960.1
Potra2n10c20476.1	1.071117261	0.402025644	6.97331E-09	0.643558659	Potri.006G131500.2	AT5G02960.1
Potra2n10c20477.1	1.072201302	0.401289932	5.90993E-09	0.64423174	Potri.010G217000.2	AT2G38890.1
Potra2n10c20478.2	0.206762548	0.391038492	0.173342827	0.605100478	Potri.010G216900.2	AT5G02950.1
Potra2n10c20479.3	-1.036272587	0.027291022	0.000135131	0.992934677	Potri.010G216800.1	AT3G54770.1
Potra2n10c20480.1	-0.201440608	0.185468156	0.116517885	0.855432225	Potri.010G216500.1	AT3G54970.1
Potra2n10c20481.1	-0.424592762	0.053739252	0.219806875	0.985393296		
Potra2n10c20482.1	0.033600089	-1.55593228	NA	NA	Potri.010G216400.1	AT1G49290.1
Potra2n10c20483.1	0.144901684	-0.06240091	0.287405396	0.95671968	Potri.010G216300.2	AT5G13610.1
Potra2n10c20484.1	0.142547933	0.216048283	0.583472796	0.794641467	Potri.013G123000.1	AT1G28510.1
Potra2n10c20485.1	-0.419787399	0.053839501	0.064967114	0.979713846	Potri.010G216200.1	AT2G28550.3
Potra2n10c20486.1	-0.148923945	0.064500555	0.242830714	0.940014069	Potri.010G216100.2	AT2G39260.1
Potra2n10c20487.1	3.186035459	0.318145376	NA	0.951731753	Potri.008G045466.1	
Potra2n10c20488.2	1.7935139	0.378993715	1.16028E-25	0.803444125	Potri.010G216000.2	AT3G45740.1
Potra2n10c20489.1	-0.526368001	-0.319788759	0.067481078	0.753288535	Potri.010G215900.1	AT5G13510.1
Potra2n10c20490.1	-0.050190974	-0.118227154	0.745387717	0.805842801	Potri.010G215700.1	AT5G13520.1
Potra2n10c20491.1	-0.509422323	-0.185415639	8.92173E-10	0.654454883	Potri.010G215500.1	AT3G55005.1
Potra2n10c20492.2	0.238838259	0.002061783	0.09732268	0.999325699	Potri.010G215300.1	AT2G37250.1
Potra2n10c20493.2	-0.200476687	-0.094800218	0.183241755	0.943411075	Potri.010G215200.1	AT5G60100.1
Potra2n10c20494.1						

Potra2n10c20495.1	-0.077900904	0.091692583	0.708927082	0.909675463	Potri.010G215100.1	AT5G18610.2
Potra2n10c20496.1	0.464751711	0.468313908	0.000429335	0.172347373	Potri.010G215000.1	AT3G55020.1
Potra2n10c20497.1	-0.124127708	-0.157366548	0.427340097	0.8689387	Potri.010G214900.1	AT3G55030.1
Potra2n10c20498.2	-0.127168635	0.045226952	0.275245348	0.973347974	Potri.010G214700.1	AT3G12620.2
Potra2n10c20499.1	-0.430816122	-0.082037172	0.010970898	0.953831244	Potri.010G214600.1	AT4G08790.1
Potra2n10c20500.1	-0.459825254	0.120768876	0.002180044	0.906679354	Potri.010G214500.2	
Potra2n10c20501.1	-0.444525359	-0.478436707	0.053357609	0.444948931	Potri.010G214300.1	AT3G55060.1
Potra2n10c20502.1	-1.076248672	-0.135880418	4.53266E-07	0.922778027	Potri.010G214200.1	
Potra2n10c20503.1	-0.944309281	-0.085776867	3.94861E-05	0.947527087	Potri.010G214200.1	
Potra2n10c20504.1	-0.611188028	0.181474378	0.096561068	0.901671593	Potri.010G214200.1	
Potra2n10c20505.1	-0.910626858	0.005163856	0.014077237	0.998883928	Potri.010G214200.1	
Potra2n10c20506.1	-0.5342648	0.542372797	0.418852662	0.842247476	Potri.010G214200.1	
Potra2n10c20507.1	0.254881368	0.075581676	0.477272175	0.972159498	Potri.005G115300.1	AT5G59690.1
Potra2n10c20508.1	0.720952751	0.318728078	0.016646577	0.799049555	Potri.005G115300.1	AT5G59690.1
Potra2n10c20509.1	0.402469098	0.161521403	0.00119056	0.814981587	Potri.010G213800.1	AT5G13590.1
Potra2n10c20510.2	-0.206083895	0.052688174	0.199625099	0.963557834	Potri.010G213700.2	AT2G39340.1
Potra2n10c20511.1	-0.526845071	0.332976502	0.275367341	0.903698951	Potri.010G213600.1	AT3G12345.1
Potra2n10c20512.1	-2.080886332	-0.49092202	8.18679E-06	0.79078676	Potri.008G047900.1	AT3G55090.1
Potra2n10c20513.1	0.328167581	0.197645311	0.000143655	0.783812825	Potri.010G213300.1	AT1G28060.1
Potra2n10c20514.1	-0.262477667	-0.064735442	0.463458468	0.959009636	Potri.010G213200.1	AT3G46290.1
Potra2n10c20515.3	3.687877615	0.958866081	1.72009E-41	0.631417984	Potri.010G213100.1	AT1G07550.1
Potra2n10c20516.1	-0.219892113	0.188597197	0.176823719	0.849063549	Potri.010G212900.1	AT5G13560.1
Potra2n10c20517.1	-7.05103706	-0.185596147	4.14362E-06	0.98409016	Potri.010G212800.1	AT2G39370.1
Potra2n10c20518.1	1.157749066	0.2663354	6.27178E-07	0.833198716	Potri.010G212700.1	AT3G12360.1
Potra2n10c20519.1	0.943350674	0.692133997	NA	NA	Potri.010G212600.1	AT5G04530.1
Potra2n10c20520.1	3.141483188	-0.396580754	3.05028E-15	0.953608209		
Potra2n10c20521.1	3.204293114	-0.493792492	1.48243E-15	0.94169165	Potri.010G212400.1	AT2G39380.1
Potra2n10c20522.1	0.786110813	0.110369199	2.18772E-06	0.947290893	Potri.010G212300.1	AT5G02610.1
Potra2n10c20523.1	-0.415557247	-0.023336851	9.44798E-06	0.974275337	Potri.010G212200.1	AT2G39420.1
Potra2n10c20524.1	-0.407717686	-0.103951408	1.00092E-05	0.784407529	Potri.010G212100.1	AT3G55200.1
Potra2n10c20525.1	-1.409241409	-0.754901547	0.001160297	0.409505482	Potri.010G212000.1	AT1G07730.2
Potra2n10c20526.1	NA	NA	NA	NA	Potri.010G211800.1	AT2G28670.1

Potra2n10c20527.1	0.964401099	0.203290558	6.67054E-11	0.855957346	Potri.010G211700.1	AT3G53540.1
Potra2n10c20528.1	0.36951557	0.419164839	0.259858085	0.822292402	Potri.010G211600.2	AT1G75270.1
Potra2n10c20529.2	2.051727827	1.206763692	0.049500219	0.826906752	Potri.010G211500.1	AT3G55240.1
Potra2n10c20530.1	1.100892109	-0.971463225	0.526519271	NA		
Potra2n10c20531.1	0.278506316	-0.170055132	0.073511505	0.93263929	Potri.010G211400.1	AT5G13550.1
Potra2n10c20532.1	-0.181792269	-0.004393555	0.182048879	0.996999319	Potri.010G211300.1	AT2G39450.1
Potra2n10c20533.1	-0.008412013	0.046312822	0.950127264	0.928823901	Potri.010G211100.1	AT3G07100.1
Potra2n10c20534.2	-0.146110868	0.057145016	0.28951137	0.937687311	Potri.010G211000.1	AT3G55260.1
Potra2n10c20535.1	-0.424956434	0.017707416	0.008760791	0.988154814	Potri.010G210900.1	AT3G55270.1
Potra2n10c20536.1	0.382372506	-0.563942251	0.684600048	0.932984974	Potri.010G210800.1	AT3G09110.1
Potra2n10c20537.1	NA	0.674963689	NA	NA		
Potra2n10c20538.1	0.188970892	-0.423910726	0.625049247	0.621109642	Potri.010G210800.1	AT3G09110.1
Potra2n10c20539.1	0.919046589	0.988895819	0.64699297	NA	Potri.010G210800.1	AT3G09110.1
Potra2n10c20540.1	0.233945212	-0.2599094	0.868659454	0.974275337	Potri.010G210800.1	AT3G09110.1
Potra2n10c20541.1	0.318224149	0.225899406	0.81069053	0.977227089	Potri.010G210800.1	AT3G09110.1
Potra2n10c20542.1						
Potra2n10c20543.1	-0.819137748	0.082299418	0.752190211	NA	Potri.010G210800.1	AT3G09110.1
Potra2n10c20544.1	0.023175801	0.660139725	0.980234048	0.843443324	Potri.010G210800.1	AT3G09110.1
Potra2n10c20545.1	0.516480122	0.196917409	0.001234567	0.855957346	Potri.010G210300.1	AT3G55280.2
Potra2n10c20546.1	0.470640781	0.176699537	0.0029312	0.875559093	Potri.010G210200.2	AT3G55330.1
Potra2n10c20547.1	-0.311272901	-0.071418711	0.03170249	0.937438252	Potri.010G210100.1	AT3G55320.1
Potra2n10c20548.1	-0.462552056	-0.15559607	0.001178601	0.802631112	Potri.010G210000.1	AT2G39470.1
Potra2n10c20549.1	-0.361517754	-0.47045392	0.162800317	0.276620704	Potri.010G209900.1	AT4G01470.1
Potra2n10c20550.1	NA	NA	NA	NA	Potri.010G209800.1	AT5G64620.1
Potra2n10c20551.1	NA	NA	NA	NA	Potri.010G209800.1	AT5G64620.1
Potra2n10c20552.3	0.715166793	0.264996278	1.26818E-06	0.895575326	Potri.010G209700.2	AT3G55340.1
Potra2n10c20553.1	-0.510328453	-0.629998947	0.552354842	0.80654978		
Potra2n10c20554.3	-0.151783566	-0.042052139	0.730434436	0.987226805		
Potra2n10c20555.1	-0.867288932	-0.601371376	0.000861292	0.249850181	Potri.010G209400.1	AT2G37430.1
Potra2n10c20556.3	-0.387666514	-0.128107352	0.001747883	0.88643982	Potri.010G209300.2	AT5G59830.1
Potra2n10c20557.1	-1.98950516	-0.560061834	9.70245E-06	0.811742964	Potri.010G209200.1	AT2G39510.1
Potra2n10c20558.1	NA	NA	NA	NA	Potri.008G051600.1	AT5G65750.1

Potra2n10c20559.1	0.563501671	0.066608052	3.56061E-07	0.902857376	Potri.010G209000.1	AT5G65750.1
Potra2n10c20560.1	0.097287598	0.096259176	0.248610275	0.902857376	Potri.010G208900.1	AT3G46060.2
Potra2n10c20561.1	-1.272206729	-0.208762255	0.001330728	0.937146435		
Potra2n10c20562.1	-1.358286033	-0.130255063	4.09996E-05	0.954419252	Potri.010G208800.1	
Potra2n10c20563.1	0.590685805	0.139979215	0.000331072	0.912777843	Potri.008G051900.1	AT1G07770.2
Potra2n10c20564.1	2.028715368	0.360353304	0.032836993	0.943819961	Potri.008G052000.10	AT1G27660.1
Potra2n10c20565.2	-0.242807766	-0.107027871	0.001069481	0.788065047	Potri.010G208400.1	AT5G13680.1
Potra2n10c20566.1	-0.276664341	-0.141223326	0.002107379	0.774693915	Potri.010G208500.2	AT5G59890.1
Potra2n10c20567.1	-0.326723682	0.101658972	0.133954174	0.905695561	Potri.010G208300.1	AT5G54980.1
Potra2n10c20568.1	0.4147186	0.391634343	0.017224412	0.642397767	Potri.010G208200.2	AT2G39580.1
Potra2n10c20569.1	0.500421487	-1.478536539	NA	NA	Potri.010G208100.1	AT3G29635.1
Potra2n10c20570.1	0.088073461	-0.121682748	0.662256327	0.911339141	Potri.010G208000.1	AT5G59950.5
Potra2n10c20571.1	0.353100082	0.145119118	0.30853241	0.71299041	Potri.010G207900.2	AT3G12590.1
Potra2n10c20572.1	-0.881941035	-0.608591572	0.477272175	NA	Potri.010G208000.1	AT5G59950.5
Potra2n10c20573.1	1.172928009	0.394389085	1.01805E-08	0.677013336	Potri.010G207800.1	AT1G65520.1
Potra2n10c20574.1	-0.540086436	0.035630721	0.430644296	0.992934677		
Potra2n10c20575.1	-1.190233143	-0.55865597	4.79824E-07	0.478429061	Potri.010G207700.1	AT2G39570.1
Potra2n10c20576.1	0.263257254	-0.011757816	0.074760262	0.994403137	Potri.010G207600.1	AT5G59613.1
Potra2n10c20577.1	-0.467062787	-1.425458398	0.771227122	NA	Potri.010G207500.1	AT4G12560.2
Potra2n10c20578.1	-0.02132454	0.206156934	0.957660064	0.902847514	Potri.010G207400.1	AT3G07870.1
Potra2n10c20579.1	-0.155936761	-0.085480219	0.155736999	0.921099436	Potri.010G207300.1	AT3G55400.1
Potra2n10c20580.1	-0.578024983	-0.317504584	0.004611161	0.833651746	Potri.010G207200.1	AT2G28760.2
Potra2n10c20581.1	0.636508769	0.263153758	0.19176062	0.907073354	Potri.010G207000.1	AT3G55390.1
Potra2n10c20582.1	0.126391371	0.095783326	0.440877695	0.945769042	Potri.010G206900.2	AT2G27490.1
Potra2n10c20583.5	-0.352061042	0.044007669	1.94268E-05	0.937687311	Potri.010G206766.1	AT5G59300.1
Potra2n10c20584.1	-1.368991757	-0.185405485	0.026102922	0.939221607	Potri.010G206700.1	
Potra2n10c20585.1	-0.449087176	0.241552759	0.218651256	0.919722977	Potri.010G206600.1	AT3G12580.1
Potra2n10c20586.1	0.533866484	0.046855483	0.010145646	0.9571583	Potri.010G206400.1	AT3G12570.3
Potra2n10c20587.1	NA	NA	NA	NA	Potri.010G206300.1	AT3G16720.1
Potra2n10c20588.1	-1.183515956	-0.327659043	0.032977179	0.886633917	Potri.010G206200.1	AT5G17600.1
Potra2n10c20589.1	-0.115375867	0.030073121	0.609973689	0.989304496	Potri.010G206100.2	AT5G59350.1
Potra2n10c20590.1	-1.35927387	2.83596237	NA	NA	Potri.010G206001.1	AT2G39550.1

Potra2n10c20591.1	-0.187149896	-0.169617928	0.299288251	0.896306549	Potri.010G205900.1	AT3G04020.1
Potra2n10c20592.1	0.267024746	-0.08788852	0.113011335	0.960981446	Potri.010G205800.1	AT5G02500.1
Potra2n10c20593.1	-0.229531914	0.00764681	0.883361457	0.999458638	Potri.008G054900.1	AT3G12580.1
Potra2n10c20594.1	-1.085100248	-0.901236351	0.421310676	NA	Potri.008G054900.1	AT3G12580.1
Potra2n10c20595.1	-0.370920785	0.138770026	0.316123691	0.954419252	Potri.008G054900.1	AT3G12580.1
Potra2n10c20596.1	-0.684310107	-0.189025641	0.545618023	0.97950444	Potri.010G205400.1	AT3G55370.2
Potra2n10c20597.1	4.104103269	2.176984196	4.37011E-58	0.000689703	Potri.010G204700.1	AT2G39530.1
Potra2n10c20598.1	0.131408478	-0.385633466	0.825692542	0.862335622	Potri.010G204500.1	AT1G55460.1
Potra2n10c20599.1	-0.926945606	-0.41396796	0.000343626	0.767368598	Potri.010G205000.1	AT2G16050.1
Potra2n10c20600.1	NA	2.253733581	NA	NA	Potri.010G204700.1	AT2G39530.1
Potra2n10c20601.1	-2.322544056	-1.358664385	1.44966E-07	0.192894257	Potri.010G205000.1	AT2G16050.1
Potra2n10c20602.1	-2.69878723	-0.491587248	3.38388E-05	0.473464658	Potri.010G205000.1	AT2G16050.1
Potra2n10c20603.1	-2.367142768	-0.391578496	0.001023675	0.660636854	Potri.010G205100.1	AT2G16050.1
Potra2n10c20604.1	-0.190761983	-0.097437357	0.583970488	0.963124118	Potri.010G204500.1	AT1G55460.1
Potra2n10c20605.1	0.009956275	-0.462227501	0.99007111	0.88807569	Potri.010G204400.1	AT3G55360.1
Potra2n10c20606.1	-0.440377531	-0.274103732	0.014134245	0.84487051	Potri.010G204300.1	AT3G12110.1
Potra2n10c20607.1	0.060662994	-0.047171072	0.881466484	0.987226805	Potri.010G204200.1	AT4G26055.1
Potra2n10c20608.5	0.215221883	0.005814446	0.173967966	0.996032145	Potri.010G204000.1	AT2G39630.1
Potra2n10c20609.1	-1.35927387	-1.507841271	NA	NA		
Potra2n10c20610.1	-0.422991554	-0.398757713	0.068874687	0.628886823	Potri.010G203900.1	AT3G55420.1
Potra2n10c20611.1	-0.228764683	0.740651406	0.889041734	0.88066693	Potri.010G203800.1	AT3G55430.1
Potra2n10c20612.1	0.112131355	-0.081372647	0.649556646	0.941260209	Potri.010G203700.1	AT2G39650.1
Potra2n10c20613.1	0.748779211	0.023202434	0.002506007	0.990128003	Potri.010G203600.2	AT3G12530.1
Potra2n10c20614.1	0.253318613	-0.038666034	0.046024063	0.978146566	Potri.010G203500.1	AT3G55440.1
Potra2n10c20615.1	1.641266768	0.393845243	3.96985E-14	0.657844103	Potri.010G203400.2	AT1G07570.2
Potra2n10c20616.1	0.096583377	0.003226723	0.363223788	0.996851113	Potri.010G203200.1	AT3G55460.1
Potra2n10c20617.1	NA	1.413466566	NA	NA	Potri.010G203100.1	AT3G55470.1
Potra2n10c20618.1	0.174048333	-0.26429462	0.615331729	0.874496903	Potri.010G203000.2	AT3G55470.2
Potra2n10c20619.2	-0.040105998	0.015008603	0.768856214	0.987824571	Potri.010G202800.1	AT3G55480.2
Potra2n10c20620.1	0.130769697	0.229113095	0.467808783	0.802942736	Potri.010G202700.1	AT3G48670.1
Potra2n10c20621.1	1.01484567	0.441988104	0.008612443	0.812696706	Potri.010G202600.1	
Potra2n10c20622.1	1.406678053	1.396483408	0.116941321	0.581957562		

Potra2n10c20623.1	0.96239675	-0.269143759	2.99182E-06	0.863871294	Potri.010G202500.1	AT2G39700.1
Potra2n10c20624.1	NA	NA	NA	NA		
Potra2n10c20625.2	-0.211424682	-0.234890001	0.050068011	0.72101822	Potri.010G202400.1	AT3G12560.1
Potra2n10c20626.1	0.503341844	0.083346946	0.113915189	0.95981322	Potri.008G057400.1	AT2G37650.1
Potra2n10c20627.1	-0.157941759	-0.297913715	0.647184182	0.817452127	Potri.010G202200.1	AT5G13830.1
Potra2n10c20628.2	-0.095435257	-0.089926841	0.743239981	0.948577865	Potri.010G202100.1	AT5G13840.2
Potra2n10c20629.1	0.058296998	0.072957317	0.653962853	0.926808461	Potri.010G202000.1	AT3G12380.1
Potra2n10c20630.1	0.92649098	1.039895108	0.133630317	0.428801064	Potri.010G201900.1	AT5G02220.1
Potra2n10c20631.1	0.245919779	-0.36710018	0.826277673	0.943483421		
Potra2n10c20632.1	1.613498266	-0.154765232	0.166631832	0.961021731		
Potra2n10c20633.1	-0.080624776	0.366777952	0.904495235	0.907073354	Potri.010G201700.1	AT2G39705.1
Potra2n10c20634.1	-0.164399176	-0.042159927	0.156569544	0.966077009	Potri.010G201600.1	AT3G55520.1
Potra2n10c20635.1	0.017683911	0.194751486	0.911212154	0.84801014	Potri.010G201500.3	AT3G55530.1
Potra2n10c20636.1	0.82357019	NA	NA	NA	Potri.010G201400.1	AT2G39710.1
Potra2n10c20637.1	-0.033256775	0.128961936	0.868576608	0.937276488	Potri.010G201300.1	AT2G39720.1
Potra2n10c20638.1	1.069585831	-0.381477692	0.439629427	NA	Potri.010G201200.1	AT3G11430.1
Potra2n10c20639.1	0.141650038	0.109627678	0.204521529	0.900869139	Potri.010G201100.1	AT2G29200.1
Potra2n10c20640.1	-1.864134742	-2.013568191	NA	NA	Potri.010G201000.2	AT3G21420.1
Potra2n10c20641.1	-2.597631092	-1.141395251	0.015883792	0.636889033	Potri.010G200800.1	AT5G02140.1
Potra2n10c20642.1	-0.493292875	0.005857958	0.012092119	0.996032145	Potri.T045300.3	AT4G10780.1
Potra2n10c20643.1						
Potra2n10c20644.1	-1.482718563	-0.027784605	8.43563E-08	0.989878071	Potri.010G200500.1	AT2G39730.1
Potra2n10c20645.1	NA	NA	NA	NA		
Potra2n10c20646.1	-2.307432468	-2.456974026	NA	NA	Potri.010G200400.1	AT5G06080.1
Potra2n10c20647.2	0.280532746	0.130981586	0.095296685	0.917751882	Potri.010G200200.1	AT1G74260.1
Potra2n10c20648.1	0.380222018	0.355182859	0.674212536	0.936335995		
Potra2n10c20649.2	2.255308366	-0.750399449	1.99134E-08	0.807261523	Potri.010G200100.2	AT3G55560.1
Potra2n10c20650.1	0.523056878	-0.290387661	0.057346137	0.828625141	Potri.010G200000.4	AT2G44920.1
Potra2n10c20651.1	-0.004882043	0.224825505	0.986324422	0.857189843	Potri.010G199900.1	AT3G11420.1
Potra2n10c20652.1	0.487603788	0.125968631	0.122439869	0.966407607	Potri.010G199800.1	AT5G06070.1
Potra2n10c20653.1	-0.754717619	0.077215891	1.46045E-09	0.96478728	Potri.010G199600.1	AT3G11410.1
Potra2n10c20654.1	0.663476885	0.190054935	4.30359E-07	0.82876584	Potri.010G199500.6	AT2G39740.1

Potra2n10c20655.1	0.567601691	0.123491133	0.00114422	0.922681078	Potri.010G199400.1	AT1G74270.1
Potra2n10c20656.1	-0.364055568	-0.001871953	0.009401361	0.999325699	Potri.010G199300.1	AT2G39750.1
Potra2n10c20657.1	-0.132475813	-0.079953055	0.296357364	0.914172897	Potri.010G199200.1	AT2G39760.1
Potra2n10c20658.1	NA	NA	NA	NA		
Potra2n10c20659.2	-0.60689183	-0.222228894	0.011985186	0.880065728	Potri.010G199100.1	AT3G55580.1
Potra2n10c20660.1	0.241679766	-0.13761747	0.0478975	0.810265332	Potri.010G199000.2	AT3G11400.1
Potra2n10c20661.1	-1.05178223	-0.721093758	6.84752E-09	0.459055713	Potri.010G198800.3	AT2G39770.1
Potra2n10c20662.1	-0.494943684	-0.226099333	0.006625766	0.802942736	Potri.010G198700.1	AT3G53850.1
Potra2n10c20663.1	0.578051307	-0.053263383	0.004451027	0.983113084	Potri.010G198600.1	AT2G39795.1
Potra2n10c20664.2	0.452641403	-0.100997311	0.003384195	0.937276488	Potri.010G198400.2	AT3G55610.1
Potra2n10c20665.1	0.088271172	-0.079602021	0.796137771	0.956865545	Potri.010G198500.1	AT2G39795.1
Potra2n10c20666.1	0.196665562	-0.038087796	0.167609497	0.97206049	Potri.010G198300.7	AT2G39805.1
Potra2n10c20667.1	2.121234607	0.585191654	1.76239E-16	0.422139068		
Potra2n10c20668.1	2.121138263	0.584947979	1.75884E-16	0.422139068	Potri.010G198100.1	
Potra2n10c20669.1	-0.065891718	-0.085977175	0.742394353	0.941855873	Potri.010G198000.1	AT3G11397.1
Potra2n10c20670.1	-0.191307146	-0.260632106	0.703344752	0.924309478	Potri.010G197900.1	AT4G38040.1
Potra2n10c20671.1	-0.014537111	-0.081130869	0.94367107	0.956865545	Potri.010G197800.2	AT5G05980.2
Potra2n10c20672.1	-0.636677706	-0.228942216	0.000449446	0.788065047	Potri.010G197700.3	AT5G05970.1
Potra2n10c20673.2	0.077378658	-0.009949056	0.554655234	0.992934677	Potri.010G197600.1	AT2G39840.1
Potra2n10c20674.1	-0.108516561	-0.145141296	0.741365703	0.951726178	Potri.010G197500.1	AT2G39830.1
Potra2n10c20675.1	-0.046247155	-0.073014527	0.743244422	0.919340668	Potri.010G197400.1	AT3G55640.1
Potra2n10c20676.1	-0.30698605	-0.073160722	0.273205124	0.976324121	Potri.010G197300.1	AT5G02030.1
Potra2n10c20677.1	-1.998050469	0.772942615	NA	NA	Potri.010G197200.1	AT1G07410.1
Potra2n10c20678.1						
Potra2n10c20679.1					Potri.010G196800.1	AT5G59100.1
Potra2n10c20680.1	1.688480072	0.50626044	0.392650018	NA	Potri.010G196800.1	AT5G59100.1
Potra2n10c20681.1						
Potra2n10c20682.1	1.545832463	NA	NA	NA	Potri.010G196500.1	
Potra2n10c20683.3	-1.101879801	0.503645546	2.25161E-06	0.907073354	Potri.010G196400.2	AT2G39855.2
Potra2n10c20684.1	-0.901792883	-0.06764667	3.51109E-05	0.981772014	Potri.010G196100.1	AT5G05950.1
Potra2n10c20685.1	-1.020995194	-0.796864824	0.001658555	0.349558394	Potri.010G196000.1	AT5G05940.1
Potra2n10c20686.1	NA	NA	NA	NA		

Potra2n10c20687.1	-0.059810991	0.087473546	0.891149247	0.974367223	Potri.008G062100.1	AT5G05930.1
Potra2n10c20688.1						
Potra2n10c20689.1	-1.042116251	-0.653771538	1.6689E-06	0.498594799	Potri.010G195800.1	AT2G39870.1
Potra2n10c20690.1	1.654609476	-0.208292308	6.38863E-05	0.93706162	Potri.010G195700.1	AT1G53540.1
Potra2n10c20691.1	1.063096255	0.133962496	0.00289807	0.967122333	Potri.010G195600.1	AT3G55700.1
Potra2n10c20692.1	-0.61910233	-0.767708831	NA	NA	Potri.010G195400.1	AT3G55700.1
Potra2n10c20693.1	0.916202705	0.400625299	0.000241562	0.468672041	Potri.010G195300.1	AT3G11340.1
Potra2n10c20694.1	1.664677056	NA	NA	NA	Potri.008G062500.1	AT5G05840.1
Potra2n10c20695.1	NA	NA	NA	NA	Potri.010G195500.1	AT3G11340.1
Potra2n10c20696.2	0.518394372	0.209555313	0.04057368	0.811742964	Potri.010G195500.1	AT3G11340.1
Potra2n10c20697.1	3.507550988	0.520202957	3.32437E-40	0.80340893	Potri.010G195200.1	AT5G05840.1
Potra2n10c20698.1	-0.048654694	0.04360971	0.833203179	0.985393296	Potri.010G195100.1	AT5G05830.1
Potra2n10c20699.1	-0.082152771	0.184221451	0.805460366	0.93010505		
Potra2n10c20700.1	0.449822493	0.110329768	0.005585233	0.940376196	Potri.010G195000.1	AT3G55730.1
Potra2n10c20701.1	-0.626350345	0.22984894	0.391152842	0.937687311	Potri.010G194800.1	
Potra2n10c20702.1	-0.849052151	-0.071106059	0.190487261	0.98876314		
Potra2n10c20703.1	-0.973834001	-0.503914655	1.48331E-05	0.621109642	Potri.010G194700.1	AT5G01090.1
Potra2n10c20704.1	-0.037880528	0.142614582	0.899820644	0.948577865	Potri.010G194600.1	AT2G39890.1
Potra2n10c20705.1	0.241621966	0.028414872	0.058026593	0.98036348	Potri.010G194300.1	AT1G51740.1
Potra2n10c20706.1	0.087826768	0.071787029	0.701134627	0.953831244	Potri.010G194400.1	AT2G37975.1
Potra2n10c20707.1	0.273196724	-0.106852523	0.14184239	0.936890993	Potri.010G194100.1	AT3G11320.1
Potra2n10c20708.1	2.165919294	0.674963689	NA	NA		
Potra2n10c20709.1	1.483903765	-1.465929376	NA	NA	Potri.008G063500.1	AT2G39910.1
Potra2n10c20710.1	-0.348127162	-0.311083018	0.04291545	0.578686469	Potri.010G193800.1	AT3G55770.4
Potra2n10c20711.1	-0.619113557	-0.908248044	0.759007309	NA	Potri.010G193700.1	AT3G55780.1
Potra2n10c20712.1	0.19587391	0.412481588	0.291631208	0.814981587	Potri.010G193500.1	AT2G39920.2
Potra2n10c20713.1	-0.273721917	0.104443209	0.062826191	0.949966783	Potri.010G193400.2	AT1G36280.1
Potra2n10c20714.1	-0.789065813	-0.609003554	5.70571E-05	0.592872379	Potri.010G193100.1	AT2G38080.1
Potra2n10c20715.1	-0.827468323	-0.043728936	0.000187237	0.982827151	Potri.010G193000.1	AT5G05790.1
Potra2n10c20716.1	0.059033973	-0.061180017	0.693316918	0.953831244	Potri.010G192900.1	AT2G39940.1
Potra2n10c20717.1	-0.541064309	-0.282703979	3.62842E-05	0.650882346	Potri.010G192800.2	AT2G39950.1
Potra2n10c20718.2	0.448952761	0.040155967	0.000155214	0.976302215	Potri.008G064700.1	AT2G39960.1

Potra2n10c20719.1	0.318796358	0.046894197	0.012725814	0.966917036	Potri.010G192600.1	AT2G39970.1
Potra2n10c20720.2	-0.070870874	-0.250648584	0.686233426	0.764266707	Potri.008G064900.1	AT1G07310.1
Potra2n10c20721.1	0.713202385	-0.31525673	0.668654956	0.976043496	Potri.010G192400.1	AT2G39980.1
Potra2n10c20722.1	0.203107298	-0.034098815	0.240346995	0.983113084	Potri.010G192200.1	AT3G11270.1
Potra2n10c20723.1	-0.061409278	-0.222143231	0.900177242	0.920028955	Potri.010G192300.1	AT3G55820.1
Potra2n10c20724.1	0.100386345	-0.18595649	0.719018355	0.895575326		
Potra2n10c20725.1	-0.683483802	-1.315920889	0.082324565	0.130626245	Potri.010G192100.1	AT3G11260.1
Potra2n10c20726.1	0.614428927	0.144688105	7.50647E-05	0.885375133	Potri.010G192000.2	AT2G39990.1
Potra2n10c20727.1	-1.031163111	-0.034213805	4.66518E-07	0.990654009	Potri.010G191900.3	AT4G00430.1
Potra2n10c20728.1	0.27109336	0.133391295	0.004971161	0.885459527	Potri.010G191800.2	AT3G06060.1
Potra2n10c20729.1	-0.116793266	0.163185372	0.335437706	0.903769998	Potri.010G191700.1	AT3G55830.1
Potra2n10c20730.1	1.534574546	-0.404297275	0.001191253	0.900551258	Potri.010G191600.1	AT2G29670.1
Potra2n10c20731.1	-2.238525738	-0.402906588	0.079069745	0.934579595	Potri.010G191500.1	AT3G18990.1
Potra2n10c20732.1	1.459962178	1.571529889	NA	NA	Potri.010G191400.1	AT4G13440.1
Potra2n10c20733.1	1.040131549	0.357386392	0.000602192	0.790362046	Potri.010G191300.1	AT2G40000.1
Potra2n10c20734.1	0.479627537	0.032068274	0.002916467	0.983944804	Potri.010G191200.1	AT2G40010.1
Potra2n10c20735.1	-0.365855097	-0.177287458	0.050422516	0.886059841	Potri.010G191100.1	AT5G05760.1
Potra2n10c20736.1	-1.250080411	-0.322283089	3.02714E-08	0.814981587	Potri.010G191000.1	AT2G38120.1
Potra2n10c20737.1	0.085780273	0.042270656	0.929555595	0.995261215	Potri.010G190900.2	
Potra2n10c20738.1	-0.060400922	-0.066034138	0.749185027	0.959881296	Potri.010G190800.1	AT1G04050.1
Potra2n10c20739.1	-0.91826929	-0.181118024	0.187212233	0.963124118	Potri.010G190700.1	AT5G05740.2
Potra2n10c20740.1	-0.821399313	-0.31713299	0.067435444	0.896878863	Potri.010G190600.2	AT5G05730.1
Potra2n10c20741.1	0.048851032	-0.106060502	0.751969465	0.863768668	Potri.010G190400.1	AT2G40060.1
Potra2n10c20742.1	-0.57892903	-0.293842736	0.003079381	0.651973886	Potri.010G190300.1	AT2G40070.1
Potra2n10c20743.1	-0.107500998	0.172055644	0.32368424	0.764173811	Potri.010G190100.1	AT3G11240.1
Potra2n10c20744.3	-0.094084549	0.039271302	0.558642631	0.98036348	Potri.010G190000.1	AT2G40110.1
Potra2n10c20745.1	-0.58611873	-0.292165383	5.93534E-05	0.728883321	Potri.010G189900.2	AT3G51880.2
Potra2n10c20746.1	1.280938371	0.604567955	0.000501788	0.578635193	Potri.010G189800.2	AT5G05690.1
Potra2n10c20747.1	0.094479502	0.01946046	0.427227175	0.983113084	Potri.010G189700.1	AT5G05680.1
Potra2n10c20748.2	1.913518138	0.889275756	1.17386E-19	0.077114891	Potri.010G189600.1	AT2G40095.1
Potra2n10c20749.1	0.165201032	0.031074148	0.370562188	0.982422288	Potri.010G189500.1	AT3G11220.1
Potra2n10c20750.1	-0.706963366	0.010453485	0.653478314	NA	Potri.010G189400.1	AT2G40090.1

Potra2n10c20751.1	0.686225862	0.360127338	0.002821987	0.482012772	Potri.010G189300.1	AT3G11210.1
Potra2n10c20752.3	0.626813789	-0.241796676	0.165972148	0.930203195	Potri.010G189200.1	AT3G55490.1
Potra2n10c20753.1	1.049714145	0.046988299	0.02824841	0.99114586	Potri.010G189100.1	AT2G13620.1
Potra2n10c20754.1	0.248258364	0.157884165	0.038861935	0.849258845	Potri.010G189000.1	AT3G55920.1
Potra2n10c20755.1	-1.422850487	-0.295488224	0.000909064	0.909450222	Potri.010G188900.1	AT2G40116.1
Potra2n10c20756.2	0.106454387	0.18225351	0.283776193	0.790362046	Potri.010G188700.1	AT5G05660.1
Potra2n10c20757.1	-0.430884705	-0.222890853	0.386489702	0.954559968	Potri.010G188600.1	AT3G55950.1
Potra2n10c20758.1	0.511225738	0.134535616	0.000242708	0.885375133	Potri.010G188500.1	AT3G55960.1
Potra2n10c20759.1	-0.660887605	-0.415940399	0.001178601	0.738746172	Potri.010G188400.1	AT2G40120.1
Potra2n10c20760.1	-2.136826305	-0.434303788	7.27955E-12	0.277578139	Potri.010G188200.1	AT2G40130.2
Potra2n10c20761.2	0.132630497	-0.047790915	0.386797999	0.95671968	Potri.010G188100.2	AT5G05610.1
Potra2n10c20762.1	1.994540652	-0.056169947	2.40029E-06	0.97950444		
Potra2n10c20763.1	2.091531407	-0.080128644	7.4505E-07	0.970432679	Potri.010G188000.1	AT5G05600.1
Potra2n10c20764.1	0.961259884	0.477175824	5.93166E-06	0.442809314		
Potra2n10c20765.2	0.486146626	-0.00617065	0.296845387	0.99838704	Potri.010G187800.1	AT5G05580.1
Potra2n10c20766.1	0.090309604	0.09722359	0.362251936	0.888222129	Potri.010G187700.2	AT5G05570.2
Potra2n10c20767.1	-0.66070573	-0.231531782	0.014458896	0.925184207	Potri.010G187600.2	AT3G55990.1
Potra2n10c20768.5	-0.719875661	-0.165856813	2.54705E-05	0.912044974	Potri.010G187300.1	AT3G55990.1
Potra2n10c20769.1	-2.382094996	-1.130167094	0.008255987	0.619615765	Potri.010G187100.1	AT2G40170.1
Potra2n10c20770.1	0.485018341	0.078991969	0.233793793	0.968407862	Potri.010G187000.1	AT2G40180.1
Potra2n10c20771.1	-0.081212727	0.018942797	0.594254663	0.987288338	Potri.010G186900.1	AT3G11130.1
Potra2n10c20772.1	-0.613246829	-0.192601975	0.000379292	0.687833812	Potri.010G186800.1	AT3G56010.1
Potra2n10c20773.1	1.549084226	1.291382247	0.7533214	NA		
Potra2n10c20774.1	-0.39062196	0.133765958	0.278429242	0.923413596	Potri.010G186700.1	AT2G40200.1
Potra2n10c20775.1	0.354416289	0.831448842	0.663965751	0.736203664	Potri.008G070900.1	AT4G37260.1
Potra2n10c20776.1	0.262929291	-0.203913762	0.281880224	0.8499308	Potri.010G186500.1	
Potra2n10c20777.1	NA	NA	NA	NA		
Potra2n10c20778.1	2.793788318	-0.998383439	0.104245448	NA	Potri.010G186400.1	AT2G40220.1
Potra2n10c20779.1	0.936768128	0.956062251	0.448986275	0.864019364		
Potra2n10c20780.1	0.800969752	0.713298863	0.55122192	0.921099436	Potri.010G186300.1	AT2G40230.1
Potra2n10c20781.1	-0.743155989	0.23950309	0.376519032	0.966191551	Potri.010G186200.2	AT5G05550.1
Potra2n10c20782.2	-0.517874167	0.163980712	0.290491003	0.953177702	Potri.008G071300.2	AT5G05550.1

Potra2n10c20783.1	-0.280546246	-0.633165711	0.615980577	0.662187972	Potri.010G186000.1	AT3G11090.1
Potra2n10c20784.1	-0.658250121	-0.20616255	0.480311919	0.966534713	Potri.010G185900.1	AT3G46510.1
Potra2n10c20785.1	-0.72305487	-0.174638324	2.03712E-07	0.804796567	Potri.010G185800.1	AT3G56040.1
Potra2n10c20786.1	1.184031517	0.840067044	0.617506988	NA	Potri.008G071700.1	AT2G40260.1
Potra2n10c20787.1	0.423947371	0.062113985	0.00035273	0.947317412	Potri.010G185600.1	AT5G05520.1
Potra2n10c20788.1	0.662671533	-0.107902408	0.122438237	0.980890614	Potri.010G185500.1	AT5G05510.1
Potra2n10c20789.1	0.06412574	-0.812208047	0.971096226	NA	Potri.010G185400.1	AT5G05500.1
Potra2n10c20790.1	0.468509298	0.05937631	0.013111946	0.949966783	Potri.010G185300.1	AT4G18640.1
Potra2n10c20791.1	-0.272088859	1.049152909	0.496050314	0.57046267	Potri.010G185200.1	AT2G31820.1
Potra2n10c20792.1	-0.344478281	-0.033958018	0.019399536	0.982827151	Potri.010G185100.1	AT5G05480.1
Potra2n10c20793.1	-0.410541453	0.239892941	0.045046234	0.895474078	Potri.010G185000.1	AT3G56080.1
Potra2n10c20794.1	0.139572838	-0.015175438	0.222043631	0.987288338	Potri.010G184800.1	AT1G13790.1
Potra2n10c20795.2	0.297412988	0.110192469	0.719125987	0.981515297	Potri.010G184700.2	AT4G35920.1
Potra2n10c20796.1	-0.551763743	-0.111118998	0.075820085	0.962661188	Potri.010G184600.1	AT1G09870.1
Potra2n10c20797.1	2.036865844	1.00709914	0.078545842	0.922011871	Potri.010G184400.1	AT2G30130.1
Potra2n10c20798.1	0.704840369	0.154739526	1.43055E-08	0.874808359	Potri.010G184300.2	AT5G05460.1
Potra2n10c20799.1	7.531265471	4.572102622	1.7932E-137	0.000368647	Potri.010G184200.2	AT5G05460.1
Potra2n10c20800.3	-0.290518199	-0.073942089	0.127413098	0.959881296	Potri.008G073100.1	AT5G05450.1
Potra2n10c20801.1	-0.704806464	-0.231190913	0.004781769	0.91998377	Potri.010G184000.1	AT2G40320.1
Potra2n10c20802.1	0.05536307	0.262247116	0.862637044	0.817452127	Potri.012G023300.1	AT5G24430.1
Potra2n10c20803.1	-0.344952601	-0.166768808	0.287746763	0.922993749	Potri.010G183900.1	AT2G40330.1
Potra2n10c20804.1	-0.18978285	-0.01152246	0.384524002	0.996030289	Potri.008G073500.1	AT5G20420.1
Potra2n10c20805.1	0.21366111	-0.182276377	0.348596667	0.83521351	Potri.010G183700.1	AT2G40340.1
Potra2n10c20806.1	1.298928932	0.009017595	7.48499E-19	0.995994807	Potri.010G183500.1	AT5G05390.1
Potra2n10c20807.1	0.310038326	-0.25419676	0.662632773	0.94795983	Potri.010G183400.1	AT3G51740.1
Potra2n10c20808.1	-0.148488764	-0.174756535	0.488964242	0.856402923	Potri.010G183300.1	AT2G38360.1
Potra2n10c20809.1	0.51055638	-1.695430083	0.790775648	NA	Potri.010G183100.1	AT2G40390.1
Potra2n10c20810.1	2.918219423	3.025856371	NA	NA	Potri.010G182900.1	AT3G44900.1
Potra2n10c20811.1	0.967596794	0.578115631	8.08812E-09	0.736000077	Potri.010G182800.1	AT4G14270.1
Potra2n10c20812.1	0.999845851	0.544049072	1.97244E-09	0.731968019	Potri.010G182900.1	AT3G44900.1
Potra2n10c20813.1	1.492028149	0.414530365	5.3126E-08	0.582021931	Potri.010G182575.1	AT5G49690.1
Potra2n10c20814.1	-0.155542166	0.39620435	0.90253824	0.940698837	Potri.010G182500.1	AT2G40410.2

Potra2n10c20815.1	3.045510786	1.104043903	4.33188E-16	0.318243807	Potri.010G182575.1	AT5G49690.1
Potra2n10c20816.1	1.315261764	0.579364475	0.001962705	0.769509519	Potri.010G182575.1	AT5G49690.1
Potra2n10c20817.1	-0.296987297	-0.490621857	0.674573713	0.865591169	Potri.010G182500.1	AT2G40410.2
Potra2n10c20818.1	3.185000454	1.160524121	8.16375E-17	0.267238451	Potri.010G182575.1	AT5G49690.1
Potra2n10c20819.3	0.29291994	0.261854847	0.102929424	0.744763949	Potri.010G182400.1	AT2G38450.1
Potra2n10c20820.1	0.60916813	-0.306478197	0.194082237	0.927861136	Potri.010G182500.1	AT2G40410.2
Potra2n10c20821.1	-1.317292482	-1.465929376	NA	NA	Potri.010G182300.1	AT3G56180.1
Potra2n10c20822.1	0.942844721	0.615767004	0.040994148	0.791589438	Potri.003G011850.1	
Potra2n10c20823.1	1.431031317	1.330703064	3.99658E-11	0.035075805	Potri.010G182200.1	AT3G10985.1
Potra2n10c20824.1	-0.158845698	-0.165584651	0.135401164	0.813575895	Potri.010G181900.1	AT3G56190.1
Potra2n10c20825.1	2.095615365	0.53840167	4.30786E-05	0.858507057	Potri.010G181800.2	AT3G56200.1
Potra2n10c20826.2	2.106236272	0.890119877	0.005033354	0.79549388	Potri.010G181700.1	
Potra2n10c20827.1	3.77469917	NA	0.348630516	NA	Potri.010G174400.1	AT1G16390.1
Potra2n10c20828.1	1.771514413	0.663230398	8.35517E-14	0.547799891	Potri.008G075900.1	AT3G03300.1
Potra2n10c20829.3	0.435212042	-0.109099601	0.008785917	0.904958151	Potri.010G181300.1	AT5G65330.1
Potra2n10c20830.2	1.459962178	NA	NA	NA	Potri.010G181000.1	AT5G17430.1
Potra2n10c20831.1	0.032914779	0.173685284	0.766141118	0.590087814	Potri.010G180800.2	AT3G03305.1
Potra2n10c20832.1	-0.007002764	0.013896559	0.955748565	0.982981438	Potri.010G180700.2	AT5G17410.1
Potra2n10c20833.1	0.01577302	0.050617072	0.896181046	0.930598202	Potri.010G180600.1	AT1G65430.1
Potra2n10c20834.1	-0.709598898	-0.358533915	7.87703E-06	0.630517286	Potri.010G180500.1	AT3G59360.2
Potra2n10c20835.1	-0.743782809	-0.480551118	1.063E-06	0.404632832		
Potra2n10c20836.1	0.111318943	0.336315243	0.562758528	0.585188125	Potri.010G180300.2	AT3G48480.1
Potra2n10c20837.1	0.121480301	0.131439331	0.615053042	0.821704644	Potri.010G180200.1	AT2G31600.1
Potra2n10c20838.1	-1.203432559	-0.084221897	0.007337164	0.972336961	Potri.010G180000.1	AT1G65450.1
Potra2n10c20839.1					Potri.010G179700.1	AT1G65480.1
Potra2n10c20840.1	-1.152939707	-1.566852638	0.226901226	0.688448032	Potri.008G077800.1	AT1G65470.1
Potra2n10c20841.1	NA	NA	NA	NA		
Potra2n10c20842.1	2.165919294	NA	NA	NA	Potri.010G179700.1	AT1G65480.1
Potra2n10c20843.1	0.672092345	0.387446269	0.015343432	0.812159187	Potri.008G077800.1	AT1G65470.1
Potra2n10c20844.1	0.82357019	1.394613675	NA	NA	Potri.010G179500.1	AT3G02630.1
Potra2n10c20845.1	-0.147640087	-0.146777651	0.586246237	0.922724172	Potri.010G179400.1	AT3G02630.1
Potra2n10c20846.1	-0.401106269	-0.48911053	0.405031564	0.786030651	Potri.010G179300.2	AT5G16250.1

Potra2n10c20847.1	-0.471084354	0.085537494	9.17702E-05	0.909675463	Potri.010G179000.1	AT5G16260.1
Potra2n10c20848.1	0.083854137	0.102201971	0.689312598	0.849273165	Potri.010G178800.1	AT5G36890.1
Potra2n10c20849.1	-0.205021758	0.145579143	0.068332939	0.80848715	Potri.010G178600.2	AT5G16270.1
Potra2n10c20850.3	0.225322812	0.132905053	0.265764833	0.896878863	Potri.010G178400.1	AT1G65540.1
Potra2n10c20851.1	4.498840405	1.150822013	1.38663E-14	NA	Potri.010G178200.1	AT5G16290.2
Potra2n10c20852.1	2.593263468	1.000758381	1.13394E-47	0.112761068	Potri.010G178050.1	AT5G48380.1
Potra2n10c20853.1	2.675988294	1.241608327	8.29448E-43	0.001943145	Potri.010G178050.1	AT5G48380.1
Potra2n10c20854.1	1.303081112	0.369063468	3.0721E-05	0.849790859		
Potra2n10c20855.1	2.523112262	0.969240851	2.6733E-12	0.427640327	Potri.010G177800.1	
Potra2n10c20856.1	2.027856836	0.829832867	2.21304E-22	0.019034375	Potri.010G177900.1	AT5G48380.1
Potra2n10c20857.1	1.380003897	0.69855685	0.041329067	0.802942736	Potri.010G177800.1	
Potra2n10c20858.1	0.36479718	-0.144133677	0.087721979	0.936890993	Potri.010G177700.1	AT1G65560.1
Potra2n10c20859.1	1.015547815	1.390455322	0.614008808	NA	Potri.010G177600.1	AT1G65570.1
Potra2n10c20860.5	-0.073082683	0.021916332	0.638364085	0.985393296	Potri.010G177400.1	AT1G65580.1
Potra2n10c20861.1	1.862057208	-0.068584673	2.47814E-26	0.974756511	Potri.010G177300.1	AT1G65610.1
Potra2n10c20862.1	-0.058424393	0.009259743	0.678769155	0.994762886	Potri.010G177200.1	AT5G16300.1
Potra2n10c20863.1	1.053998986	1.482223731	0.081183548	0.351105764		
Potra2n10c20864.1	-2.281788984	-0.001578092	NA	NA	Potri.010G177100.1	AT1G65620.2
Potra2n10c20865.3	-0.214376778	0.092648125	0.14685158	0.937276488	Potri.008G079900.2	AT5G36950.1
Potra2n10c20866.1	NA	NA	NA	NA	Potri.010G177000.2	AT2G40070.1
Potra2n10c20867.1	3.853935137	NA	0.31197289	NA	Potri.010G176600.2	AT1G79580.1
Potra2n10c20868.3	0.366902413	0.194299986	0.071939825	0.880645865	Potri.008G080100.2	AT3G12670.1
Potra2n10c20869.1	0.52284066	0.216479452	0.012024473	0.884214233	Potri.008G080200.1	
Potra2n10c20870.2	-0.182958666	-0.002297016	0.054143872	0.998860994	Potri.010G176400.2	AT1G54350.1
Potra2n10c20871.3	-0.258645888	0.101482976	0.073191688	0.937846405	Potri.010G176100.1	AT3G12685.1
Potra2n10c20872.2	1.266065211	0.3511888	1.30369E-11	0.694477853	Potri.010G176000.1	AT1G65930.1
Potra2n10c20873.1	NA	1.363811008	NA	NA	Potri.010G175900.2	AT1G79250.2
Potra2n10c20874.1	0.42425056	-0.384374845	0.825356745	NA	Potri.T166100.1	AT3G06130.1
Potra2n10c20875.1	2.382834059	0.513667628	1.0636E-24	0.674068297	Potri.010G175700.1	AT4G02860.1
Potra2n10c20876.1	0.930945942	0.441014196	8.44382E-09	0.420956018	Potri.010G175600.1	AT1G79260.1
Potra2n10c20877.1	-0.688652026	-0.127619696	NA	NA	Potri.T166100.1	AT3G06130.1
Potra2n10c20878.1	2.159762706	0.654964111	4.94598E-26	0.566644161	Potri.T166200.1	AT4G02860.1

Potra2n10c20879.1	-0.536214648	-0.181805554	1.285E-05	0.821266061	Potri.010G175500.10	AT1G79270.1
Potra2n10c20880.1	-1.496411539	-0.69943309	8.61763E-31	0.002392219	Potri.010G175300.2	AT5G44680.1
Potra2n10c20881.1	-1.100365436	-2.06379145	NA	NA		
Potra2n10c20882.1	1.914523853	0.030959171	0.361315231	NA	Potri.010G175100.1	AT1G30870.1
Potra2n10c20883.1	0.012068563	-0.011450738	0.939555423	0.99114586	Potri.010G175000.1	AT1G79340.1
Potra2n10c20884.1	-0.173914552	-0.005009837	0.222378159	0.996032145	Potri.010G174900.1	AT1G79350.1
Potra2n10c20885.2	0.581647746	0.233813746	5.92872E-05	0.694769714	Potri.010G174800.4	AT1G79380.1
Potra2n10c20886.1	0.582098464	0.515391999	0.056980546	0.737112468	Potri.010G174700.1	AT1G79390.1
Potra2n10c20887.1	-0.052270173	0.773842379	NA	NA	Potri.010G174600.1	AT1G65910.1
Potra2n10c20888.1	1.88024439	-1.121886881	0.207735173	0.919424623	Potri.010G174500.1	AT3G12720.1
Potra2n10c20889.1	-1.109905931	-0.533873115	5.60038E-06	0.069751212	Potri.010G174400.1	AT1G16390.1
Potra2n10c20890.1	-1.317292482	-1.465929376	NA	NA	Potri.010G174300.1	AT1G79360.1
Potra2n10c20891.1	-0.726342828	-0.32728479	0.000563086	0.81510213	Potri.010G174200.1	AT1G79420.1
Potra2n10c20892.1	1.278035475	-0.541310075	0.560209096	NA	Potri.010G174100.1	AT1G79430.2
Potra2n10c20893.1	0.164960923	0.062494144	0.068673038	0.937687311	Potri.010G174000.1	AT1G79440.1
Potra2n10c20894.1	-0.317439415	-0.184030467	0.015282157	0.847341104	Potri.010G173900.1	AT3G12740.1
Potra2n10c20895.1	-0.288100735	-0.198033416	0.022624856	0.80848715	Potri.010G173800.1	AT1G54310.2
Potra2n10c20896.1	2.196235887	NA	NA	NA	Potri.010G173700.1	AT1G79460.1
Potra2n10c20897.1	0.864211382	0.438444386	5.34008E-12	0.360126886	Potri.010G173600.1	AT1G16350.1
Potra2n10c20898.1	-0.206299447	-1.17278798	0.937839897	NA	Potri.010G173500.1	AT1G79480.1
Potra2n10c20899.1	0.130884552	0.040014401	0.430816839	0.978646026	Potri.010G173400.1	AT1G79490.1
Potra2n10c20900.1	NA	NA	NA	NA	Potri.010G173300.1	AT3G12750.1
Potra2n10c20901.1	NA	NA	NA	NA		
Potra2n10c20902.1	-0.00897521	-0.020656216	0.975028595	0.99016876	Potri.010G173200.1	AT1G16330.1
Potra2n10c20903.1	-0.206977926	-0.087829673	0.041225451	0.882998329	Potri.010G173100.4	AT2G40980.1
Potra2n10c20904.1	-0.326438657	-0.220018064	0.103552526	0.863022165	Potri.010G173000.1	AT1G79510.1
Potra2n10c20905.1	-3.109929666	-0.44489802	2.70678E-13	0.677013336		
Potra2n10c20906.1	-3.25336968	-0.555799159	1.4938E-21	0.650882346	Potri.010G172900.2	AT1G16310.1
Potra2n10c20907.1	-2.918771355	-0.060148614	2.09067E-08	0.986636967	Potri.010G172800.1	AT1G16310.1
Potra2n10c20908.1	-2.865392387	-0.016604145	1.24764E-17	0.995579852	Potri.010G172800.1	AT1G16310.1
Potra2n10c20909.1	-2.975986307	-0.010520308	2.76869E-10	0.99736971	Potri.010G172800.1	AT1G16310.1
Potra2n10c20910.2	1.904779805	-0.237812297	5.96068E-09	0.934579595	Potri.010G172600.1	AT1G16310.1

Potra2n10c20911.1	0.05068274	0.047362684	0.697136994	0.948577865	Potri.010G172500.1	AT5G13010.1
Potra2n10c20912.2	0.976920535	0.148115433	2.50886E-12	0.694477853	Potri.010G172400.1	AT1G16300.1
Potra2n10c20913.1	-0.673388181	0.022749091	0.643901359	0.99819408	Potri.010G172300.3	AT1G16290.1
Potra2n10c20914.1	-0.304600958	0.278114812	0.06248564	0.797425838	Potri.010G172100.2	AT5G44800.1
Potra2n10c20915.1	0.552026722	0.044316461	0.000923334	0.974275337	Potri.010G171500.1	AT3G12780.1
Potra2n10c20916.1	-0.544516187	-0.153446653	0.001306978	0.900637717	Potri.010G171400.1	AT1G56180.1
Potra2n10c20917.1	0.039862272	0.080790833	0.777562457	0.948577865	Potri.010G171300.1	AT1G79560.1
Potra2n10c20918.1	-3.282628901	0.037241008	0.059601951	0.996032145		
Potra2n10c20919.1	-0.032641022	0.010397266	0.81547267	0.99114586	Potri.010G171100.6	AT3G19895.1
Potra2n10c20920.1	1.196534544	0.479198302	1.07722E-17	0.295461515	Potri.010G171000.1	AT1G01550.1
Potra2n10c20921.1	2.645548637	0.936773371	1.41181E-11	0.349381798	Potri.010G170900.1	AT3G19850.1
Potra2n10c20922.1	-0.13002929	-0.05162454	0.291473512	0.956865545	Potri.010G170700.1	AT1G50300.1
Potra2n10c20923.3	-0.070792051	0.101339702	0.525987335	0.899720386	Potri.010G170400.1	AT3G19840.1
Potra2n10c20924.7	0.526512071	-0.06128165	0.001069479	0.949048812	Potri.010G170300.1	AT3G19830.1
Potra2n10c20925.1	-0.793377128	0.69339605	0.7326842	NA	Potri.010G170200.2	AT5G65940.1
Potra2n10c20926.1	-0.217378938	0.028538341	0.257825965	0.985393296	Potri.010G170100.1	AT3G19810.1
Potra2n10c20927.1	-0.894357114	-0.408453238	9.48129E-14	0.635323636	Potri.010G170000.1	AT3G19820.2
Potra2n10c20928.3	0.501485243	0.205327262	0.002430867	0.820608924	Potri.010G169900.1	AT4G33540.1
Potra2n10c20929.1	0.330264909	0.080871433	0.01928536	0.938653363	Potri.010G169800.1	AT4G27640.1
Potra2n10c20930.1	-0.364321324	-0.16184908	0.232741054	0.863022165	Potri.010G169700.2	AT3G19900.1
Potra2n10c20931.6	-0.019452127	-0.030359062	0.886098461	0.971317865	Potri.010G169500.1	AT2G02955.1
Potra2n10c20932.1	-1.609454623	0.038277776	0.000611334	0.993630002	Potri.010G169400.1	AT1G69600.1
Potra2n10c20933.1	-3.085135207	0.185738345	2.68419E-07	0.953831244		
Potra2n10c20934.1	-2.509647039	-0.264307284	0.017194494	0.971828097	Potri.010G169300.1	AT1G69588.1
Potra2n10c20935.1	4.315718994	3.010689165	0.08575539	NA		
Potra2n10c20936.2	0.242314107	-0.069426737	0.026363543	0.949048812	Potri.010G169200.1	AT2G02960.4
Potra2n10c20937.1	-0.048217476	0.119809074	0.723133495	0.904791794	Potri.010G169100.1	AT2G02970.1
Potra2n10c20938.2	-3.018030076	0.655053834	0.101773695	0.909675463		
Potra2n10c20939.1	-2.65127572	-1.280919337	9.30822E-05	0.51571418	Potri.010G169000.1	AT3G28960.1
Potra2n10c20940.2	-1.151572026	-0.481400592	0.000950199	0.700935593	Potri.010G168900.1	
Potra2n10c20941.1	-0.883732442	-0.445397242	0.026032236	0.79819686	Potri.010G168800.1	AT2G02980.1
Potra2n10c20942.1	-1.317292482	-1.465929376	NA	NA	Potri.010G168700.1	AT2G02990.1

Potra2n10c20943.1	1.256343872	0.006711296	3.55974E-10	0.997808109	Potri.010G168500.4	AT1G26810.1
Potra2n10c20944.1	-0.1319344	-2.59744079	0.975590728	NA	Potri.010G168400.1	AT1G09157.1
Potra2n10c20945.1	-0.179237279	-0.16901204	0.428117259	0.875559093	Potri.010G168300.1	AT1G26800.1
Potra2n10c20946.1	0.483895539	0.379467476	0.118337989	0.715585895	Potri.010G168200.1	AT1G14185.1
Potra2n10c20947.1	-0.585935217	-0.337828733	0.017575538	0.683816919	Potri.010G168100.1	AT1G14185.1
Potra2n10c20948.1	-0.910608695	-1.898364051	0.814843623	NA		
Potra2n10c20949.2	-0.151124132	0.454204795	0.684600048	0.824378527	Potri.010G167600.1	AT5G39660.1
Potra2n10c20950.1	1.256636482	-0.037599366	NA	NA	Potri.010G167500.2	AT1G69560.1
Potra2n10c20951.1	-0.315828002	-0.143334848	0.226380839	0.906679354	Potri.010G167400.1	AT2G03050.1
Potra2n10c20952.1	0.006972563	0.73135382	0.987803155	0.376293157	Potri.010G167300.1	AT2G03060.2
Potra2n10c20953.1	-0.596663814	-0.548709683	0.108612473	0.108031251	Potri.010G167200.3	AT1G69530.2
Potra2n10c20954.1	-0.418101233	-0.471225752	0.341364297	0.418644133		
Potra2n10c20955.1	NA	1.879759176	NA	NA		
Potra2n10c20956.1	-1.317292482	-1.465929376	NA	NA		
Potra2n10c20957.1	0.028338698	0.031864007	0.810581748	0.973347974	Potri.010G167100.11	AT5G15270.2
Potra2n10c20958.1	0.312408208	0.758902508	0.202724953	0.215210112	Potri.010G167000.3	AT1G69510.3
Potra2n10c20959.1	0.102236182	0.660619236	NA	NA	Potri.010G166900.1	AT3G53720.1
Potra2n10c20960.1	-1.789052127	-0.402460632	0.0001378	0.757518018	Potri.010G166800.1	AT1G14150.1
Potra2n10c20961.3	0.114849628	0.319686344	0.269222867	0.71411664	Potri.010G166500.2	AT1G18720.1
Potra2n10c20962.1	0.036696931	0.608852105	0.983047839	NA	Potri.008G088900.2	AT1G69500.1
Potra2n10c20963.1	2.297758818	2.439754403	0.058529591	0.433369183	Potri.010G166200.1	AT1G69490.1
Potra2n10c20964.1	0.225832547	0.219697148	0.010050213	0.59929692	Potri.010G166100.1	AT2G03150.1
Potra2n10c20965.1	-0.160644449	0.13217366	0.426347443	0.894276674	Potri.010G165900.3	AT1G14140.1
Potra2n10c20966.1	0.82357019	NA	NA	NA	Potri.010G165700.1	AT3G01140.1
Potra2n10c20967.1	0.984742704	0.78086734	0.028566851	0.702237139	Potri.010G165600.1	AT1G26550.1
Potra2n10c20968.1	0.306596497	-0.040795767	0.036830707	0.974264269	Potri.010G165500.2	AT1G26750.1
Potra2n10c20969.1	-0.081357279	0.117446441	0.636615591	0.900551258	Potri.010G164900.1	AT1G14040.1
Potra2n10c20970.1	0.244730021	0.27524672	0.440724881	0.759797728	Potri.010G165100.1	AT1G26740.1
Potra2n10c20971.4	-0.079221492	0.096059353	0.661273429	0.924951347	Potri.010G165000.2	AT1G69480.1
Potra2n10c20972.2	-0.420023156	0.006854107	0.025882599	0.996952066	Potri.010G164700.2	AT1G14020.1
Potra2n10c20973.2	0.089745046	-0.071433596	0.543268681	0.95671968	Potri.010G164600.1	AT1G26690.1
Potra2n10c20974.1	0.302745825	0.026539794	2.75255E-05	0.982221696	Potri.010G164500.1	AT1G14000.1

Potra2n10c20975.1	-0.321462783	-0.046942958	0.012260468	0.956610193	Potri.010G164400.1	AT1G13990.1
Potra2n10c20976.1	-0.179152601	-0.102936207	0.114337123	0.931774514	Potri.010G164300.1	AT1G26670.1
Potra2n10c20977.1	-0.194023416	-0.176963902	0.104549774	0.832492778	Potri.010G164200.1	AT1G29150.1
Potra2n10c20978.1	1.596269253	0.466951163	7.79755E-16	0.712599153	Potri.010G164100.1	AT1G69450.1
Potra2n10c20979.1	-0.61910233	-0.767708831	NA	NA	Potri.008G091300.1	AT5G19790.1
Potra2n10c20980.1	-1.35927387	-0.079512623	NA	NA		
Potra2n10c20981.1	-0.371657787	0.351984841	0.257077011	0.857189843	Potri.010G163800.1	AT1G69440.1
Potra2n10c20982.2	-0.560332664	-0.989321022	0.777122002	NA	Potri.010G163700.1	
Potra2n10c20983.2	0.250582539	0.984485346	0.391266432	0.166599048	Potri.010G163600.1	AT1G26660.1
Potra2n10c20984.1	-0.48287426	0.765937718	0.840517519	NA	Potri.010G163501.1	
Potra2n10c20985.1	-0.17654494	-1.822970894	0.947767069	NA		
Potra2n10c20986.1	-0.304055309	-0.305498536	0.13717317	0.808716407	Potri.010G163400.1	AT1G26650.1
Potra2n10c20987.1	-1.042132331	-1.117428469	0.148942702	0.32906562		
Potra2n10c20988.1	-0.400419883	-0.302629267	0.095247186	0.79078676	Potri.010G163200.1	AT1G69420.2
Potra2n10c20989.2	-0.902717647	0.076671237	0.032047567	0.98036348	Potri.010G163100.1	AT1G13970.1
Potra2n10c20990.1	0.255363023	0.325200444	0.167760272	0.70570456	Potri.010G163000.1	AT1G13960.1
Potra2n10c20991.1	-0.711912189	0.076134974	3.55548E-06	0.940255038	Potri.010G162900.1	AT1G26640.1
Potra2n10c20992.1	0.002063408	-0.199128499	0.992253987	0.842247476	Potri.010G162800.1	AT1G13950.1
Potra2n10c20993.1	-0.232237858	0.030795166	0.096781437	0.983113084	Potri.010G162700.2	AT1G69400.1
Potra2n10c20994.1	-1.917567131	-0.942267356	0.360383473	NA		
Potra2n10c20995.1	-0.086045528	0.060429994	0.692129544	0.963993404	Potri.010G162500.1	AT1G69380.1
Potra2n10c20996.1	-0.39654946	-0.101273965	0.006716408	0.935474933	Potri.010G162400.1	AT2G03350.1
Potra2n10c20997.1	-1.109987411	-0.631474222	5.25888E-08	0.436464795	Potri.010G162300.1	AT1G69370.1
Potra2n10c20998.1	-0.061682353	-3.894232364	0.985138956	NA	Potri.010G162200.1	AT2G03360.2
Potra2n10c20999.1	0.417737812	-0.636252219	0.775036025	NA	Potri.010G162100.1	AT2G03360.2
Potra2n10c21000.1	-0.223595447	0.155585447	0.029775381	0.794641467	Potri.010G162000.2	AT1G13940.1
Potra2n10c21001.1	-0.238877777	0.208933839	0.247719922	0.775618506		
Potra2n10c21002.1	-0.299174349	0.174497853	0.205401514	0.903698951	Potri.010G161800.1	AT2G03380.1
Potra2n10c21003.2	-1.317292482	1.998377713	NA	NA	Potri.010G161700.1	AT2G20540.1
Potra2n10c21004.1	0.001383194	0.124208489	0.994233629	0.820991574	Potri.010G161600.1	AT2G03390.1
Potra2n10c21005.1	-0.358449237	-0.003190756	0.015916348	0.999212697	Potri.010G161500.10	AT1G69340.1
Potra2n10c21006.1	-1.196640192	-0.464003146	3.94129E-09	0.349381798	Potri.010G161400.7	AT2G03420.1

Potra2n10c21007.1	0.349564037	0.102415119	0.023253462	0.902857376	Potri.010G161300.7	AT2G03430.1
Potra2n10c21008.1					Potri.010G161250.1	
Potra2n10c21009.1	0.275236788	0.253069607	0.201590287	0.854980698	Potri.010G161200.1	AT2G03440.1
Potra2n10c21010.2	-0.058591351	-0.155370961	0.802680794	0.896593025	Potri.010G160900.1	AT1G13920.1
Potra2n10c21011.1	0.689515946	0.09596433	0.002548249	0.956865545	Potri.010G161100.1	AT1G69330.1
Potra2n10c21012.1	-1.357956641	-0.595752269	6.54681E-06	0.602611218		
Potra2n10c21013.1	0.763165149	0.206042467	NA	0.82876584	Potri.010G160800.2	AT5G61250.2
Potra2n10c21014.1	0.043993587	0.056058989	0.835681093	0.972159498	Potri.010G160700.1	AT5G61240.1
Potra2n10c21015.1	0.82357019	NA	NA	NA	Potri.010G160600.1	AT1G26600.1
Potra2n10c21016.1	-0.353676414	-0.185415706	0.016827624	0.864223715	Potri.010G160500.1	AT1G13900.1
Potra2n10c21017.1	-0.352430139	-0.184302279	0.017194494	0.865538396	Potri.008G093800.1	AT1G13890.1
Potra2n10c21018.1	1.263238793	0.053612373	1.72147E-05	0.987226805	Potri.010G160200.5	AT3G29320.1
Potra2n10c21019.1	0.467509936	0.206955442	0.178097849	0.930871994	Potri.010G160100.1	AT1G69310.2
Potra2n10c21020.1	1.256438031	1.154565285	0.042892992	0.679710442	Potri.008G094100.1	AT1G26520.1
Potra2n10c21021.3	-0.662777653	-0.262885756	0.100826375	0.911509355	Potri.010G159900.1	AT1G26560.1
Potra2n10c21022.1	NA	0.674963689	NA	NA		
Potra2n10c21023.1	-0.456978964	-0.30762641	0.007254636	0.846499552	Potri.010G159800.1	AT5G15490.1
Potra2n10c21024.1	0.222815284	-0.004557444	0.07929195	0.996032145	Potri.010G159700.1	AT1G26580.1
Potra2n10c21025.1	-0.29851027	0.058517449	0.509782677	0.974756511	Potri.010G159600.1	AT1G26610.1
Potra2n10c21026.1	0.147327113	0.337438609	0.570524587	0.672348168	Potri.008G094600.1	AT1G13870.1
Potra2n10c21027.1	-0.453262458	-0.153458152	0.008879428	0.926546506	Potri.010G159400.1	AT1G13860.4
Potra2n10c21028.1	-0.61768271	0.837995283	0.162578179	0.621109642	Potri.010G159300.1	AT1G26480.1
Potra2n10c21029.1	-0.033583658	0.229879095	0.926382957	0.869186333	Potri.010G159200.1	AT1G26470.1
Potra2n10c21030.1	0.238070003	-0.023463498	0.084344363	0.985393296	Potri.010G159100.1	AT1G26460.1
Potra2n10c21031.1	-0.645832216	-0.041663689	5.30746E-08	0.964766975	Potri.010G158900.2	AT1G18650.1
Potra2n10c21032.1	-0.961492892	-0.078422192	0.000145222	0.956773505	Potri.010G158800.1	AT1G13820.1
Potra2n10c21033.1	0.4547287	0.30343055	0.009710236	0.562306981	Potri.010G158700.5	AT2G03510.1
Potra2n10c21034.1	1.46860876	0.822764631	0.021630684	0.792511865		
Potra2n10c21035.1	1.441623671	0.45174888	0.049400514	0.922687399	Potri.010G158601.1	AT1G69290.1
Potra2n10c21036.1	0.940970868	-0.226674282	0.000292209	0.946201594	Potri.010G158500.1	AT1G10380.1
Potra2n10c21037.1	1.14446344	0.090324022	3.35708E-11	0.943819961	Potri.010G158400.1	AT1G13750.1
Potra2n10c21038.1	-0.028870102	0.599284185	0.956225996	0.610748456	Potri.010G158400.1	AT1G13750.1

Potra2n10c21039.1	-0.112787122	0.013116794	0.454690347	0.989878071	Potri.010G158100.2	AT2G03620.1
Potra2n10c21040.3	-0.080474232	0.030031587	0.724162132	0.983113084	Potri.010G158000.3	AT2G03630.1
Potra2n10c21041.1	0.440555455	0.567771925	0.130934465	0.751093503	Potri.010G157900.1	AT1G13740.1
Potra2n10c21042.1	-0.286673034	-0.178945781	0.011097021	0.863768668	Potri.010G157800.1	AT1G13730.1
Potra2n10c21043.1	0.145512286	-0.014508569	0.315585004	0.992452189	Potri.010G157700.2	AT2G03667.1
Potra2n10c21044.1	-0.376023542	-0.146740442	0.002962345	0.885115918	Potri.010G157600.2	AT1G26355.1
Potra2n10c21045.1	0.34198663	-0.03365847	0.518897539	0.992934677	Potri.010G157500.1	AT2G03690.1
Potra2n10c21046.1	-0.117787789	0.190312818	0.266959086	0.798141431	Potri.010G157400.1	AT1G69220.1
Potra2n10c21047.1	-0.426354301	-0.20959509	0.018208923	0.815419191		
Potra2n10c21048.1	NA	1.394613675	NA	NA		
Potra2n10c21049.2	-1.221847016	-0.099961044	5.475E-05	0.953608209	Potri.010G157200.2	AT1G13700.1
Potra2n10c21050.1	1.790070016	0.3883904	4.43273E-15	0.601615073	Potri.010G157000.1	AT1G13690.1
Potra2n10c21051.1	NA	1.381953902	NA	NA	Potri.010G156800.2	AT5G38970.2
Potra2n10c21052.1	0.260122043	-0.064707381	0.164467322	0.977559521	Potri.010G156700.1	AT3G08900.1
Potra2n10c21053.1	3.002914674	3.927121039	0.254526645	NA	Potri.008G097700.2	AT1G26330.1
Potra2n10c21054.1	NA	NA	NA	NA	Potri.010G154350.1	AT1G69180.1
Potra2n10c21055.1	0.522248916	0.379111592	0.000439557	0.401060435	Potri.010G154300.2	AT1G69170.1
Potra2n10c21056.1	-1.800448636	-0.744007136	3.55105E-13	0.303446787	Potri.010G154200.1	AT1G69160.1
Potra2n10c21057.1	0.525747133	2.054928618	0.695817456	0.247573674	Potri.010G154100.1	AT1G69120.1
Potra2n10c21058.1	0.219080444	0.22071687	0.022611216	0.759797728	Potri.010G154000.1	AT2G47070.1
Potra2n10c21059.1	-0.317190155	-0.129082778	0.049907451	0.915011483	Potri.010G153900.1	AT3G06430.1
Potra2n10c21060.1	0.82357019	0.674963689	NA	NA	Potri.015G006100.1	AT1G64940.1
Potra2n10c21061.1	-0.539654846	-3.048372702	0.823351332	NA	Potri.015G006000.1	AT3G53480.1
Potra2n10c21062.1	-2.582081003	-0.632820339	9.16767E-17	0.495345445	Potri.010G153700.1	AT3G53480.1
Potra2n10c21063.1	-2.446899573	-0.501488528	8.23812E-15	0.619575855	Potri.010G153600.1	AT3G53480.1
Potra2n10c21064.1						
Potra2n10c21065.1	0.390708964	-0.256844674	0.024770438	0.870293488	Potri.010G153500.2	AT1G02500.2
Potra2n10c21066.1	-0.578642361	-0.58129826	0.061288527	0.783936798	Potri.010G153400.1	AT3G15580.1
Potra2n10c21067.1	0.11270608	0.109064795	0.441094386	0.896306549	Potri.010G153300.1	AT5G18525.1
Potra2n10c21068.1	-0.266444586	-0.212075051	0.344199628	0.924951347	Potri.010G153200.1	AT3G17365.1
Potra2n10c21069.1	0.951573445	0.474158143	0.010713994	0.180908519	Potri.010G153100.1	AT2G22300.2
Potra2n10c21070.2	-0.121895066	-0.198109739	0.796231475	0.953831244	Potri.010G153000.1	AT3G17360.1

Potra2n10c21071.1	-0.985562083	-0.831296807	0.009255176	0.674283226	Potri.010G152900.1	AT3G17350.1
Potra2n10c21072.1	0.258920121	0.00730021	0.032309551	0.995920419	Potri.010G152700.1	AT3G17340.1
Potra2n10c21073.1	0.221446306	-0.169971308	0.262567763	0.865591169	Potri.010G152600.1	AT1G10630.1
Potra2n10c21074.3	-0.150218834	-0.440784979	0.963157425	NA	Potri.008G100100.2	AT5G15640.1
Potra2n10c21075.2	0.063860071	-0.035422186	0.676932037	0.976172519	Potri.010G152400.4	AT5G14610.2
Potra2n10c21076.1	-0.152609718	0.019035336	0.116063504	0.98177802	Potri.010G152200.10	AT3G17310.2
Potra2n10c21077.1	-0.170098285	0.040454833	0.328877351	0.979273211	Potri.008G100400.7	AT3G17300.1
Potra2n10c21078.1	1.518947654	-0.016838194	0.000619799	0.996032145	Potri.010G152000.1	AT1G48100.1
Potra2n10c21079.1	2.65257033	0.674781307	1.81559E-16	0.677268873	Potri.008G100600.1	AT1G48090.1
Potra2n10c21080.1	-0.205358326	-0.131041875	0.543780691	0.849067086	Potri.010G151600.1	AT3G06440.1
Potra2n10c21081.1	0.023145944	0.343663427	0.913190102	0.809437659	Potri.010G151500.1	AT1G48040.1
Potra2n10c21082.1	-0.542705999	0.093883293	0.706001526	0.989304496	Potri.008G100800.1	AT3G17240.3
Potra2n10c21083.1	-0.14913278	0.068619686	0.218393526	0.943819961	Potri.010G151300.1	AT5G30490.2
Potra2n10c21084.1	1.976597668	1.363811008	NA	NA	Potri.010G151200.2	AT3G17210.1
Potra2n10c21085.1	0.207245906	0.202208327	0.715179775	0.945254452	Potri.005G019400.1	AT1G09190.1
Potra2n10c21086.1	3.012693887	0.648375545	9.7989E-26	0.64562236	Potri.010G151000.1	AT3G17210.1
Potra2n10c21087.1	1.584100755	0.472938717	3.91465E-06	0.817452127	Potri.010G150900.1	AT3G17210.1
Potra2n10c21088.1	-0.38739664	0.020709633	0.767339996	0.99859284	Potri.010G150750.1	
Potra2n10c21089.1	-0.293594619	0.166166446	0.823328453	0.985136323		
Potra2n10c21090.1	2.887035953	2.146414233	NA	NA		
Potra2n10c21091.1	1.898033602	1.036988469	0.017340297	0.792511865	Potri.010G150700.1	AT3G17210.1
Potra2n10c21092.1	2.463183016	0.501938928	4.61784E-08	0.829781359	Potri.010G150500.1	AT3G17210.1
Potra2n10c21093.1	NA	2.0078969	NA	NA	Potri.010G150400.1	AT2G47180.1
Potra2n10c21094.1	-0.005037708	0.021299637	0.980901266	0.985136323	Potri.010G150300.1	AT5G30510.1
Potra2n10c21095.1	0.277559264	0.042720677	0.00143664	0.966917036	Potri.010G150000.2	AT3G17205.1
Potra2n10c21096.1	1.663921017	0.524296181	0.005568468	0.865611082	Potri.010G149900.1	AT3G06490.1
Potra2n10c21097.1	NA	0.674963689	NA	NA	Potri.008G101500.2	AT3G06500.1
Potra2n10c21098.1	2.196235887	3.7769567	NA	NA	Potri.010G149700.3	AT1G47990.1
Potra2n10c21099.1	-0.219908718	-0.029081401	0.444174022	0.987548286	Potri.010G149600.1	
Potra2n10c21100.1	-0.230936561	-0.029420818	0.098071554	0.985964146	Potri.010G149500.1	AT3G06510.1
Potra2n10c21101.1	0.500206555	0.433317122	0.003540653	0.185331515	Potri.010G149400.2	AT3G17180.1
Potra2n10c21102.1	-0.932809944	-0.352896643	0.446507198	0.924309478	Potri.010G149301.1	AT3G06520.1

Potra2n10c21103.1	NA	NA	NA	NA	Potri.010G149100.2	AT5G49600.1
Potra2n10c21104.1	-0.336172404	-0.058651482	0.044036669	0.960713293	Potri.010G149000.2	AT5G49610.1
Potra2n10c21105.1	-0.003357326	0.110039132	0.985467958	0.903477395	Potri.010G148900.1	AT5G49580.1
Potra2n10c21106.1	NA	1.571529889	NA	NA	Potri.008G102000.1	AT1G47980.1
Potra2n10c21107.1	-0.005248937	-0.112951305	0.99519204	0.980475138		
Potra2n10c21108.1	-0.003844294	0.046622081	0.980762763	0.960656795	Potri.008G102200.2	AT5G49540.1
Potra2n10c21109.1	0.173357993	0.234405127	0.569725121	0.813575895	Potri.010G148701.1	AT3G48810.1
Potra2n10c21110.1	-0.784684499	-0.373821134	1.0622E-10	0.516019823	Potri.010G148500.3	AT3G06550.1
Potra2n10c21111.1	0.075678124	0.070117297	0.735045875	0.946464685	Potri.010G148400.1	AT1G47970.1
Potra2n10c21112.1	0.214210407	-0.369702236	0.814592756	0.937789963	Potri.010G148300.1	AT5G49525.1
Potra2n10c21113.1	2.281870856	0.350033044	1.95785E-10	0.862418769	Potri.010G148200.10	AT3G17120.1
Potra2n10c21114.1	-0.540100236	-0.217963805	0.014543871	0.875559093	Potri.010G148100.1	AT3G06580.1
Potra2n10c21115.1	0.030719443	0.03283476	0.912638404	0.981401592	Potri.010G148000.1	AT4G21800.2
Potra2n10c21116.1	-0.096621782	0.04687624	0.43972019	0.949966783	Potri.010G147900.2	AT4G16280.4
Potra2n10c21117.1	-2.295337707	-1.036082888	NA	NA	Potri.010G147800.1	AT4G16270.1
Potra2n10c21118.1	2.446094736	0.567967581	0.009815484	0.922011871	Potri.010G147700.1	AT5G49520.1
Potra2n10c21119.2	0.02450209	0.01460254	0.89207564	0.9896961	Potri.010G147600.1	AT5G49510.2
Potra2n10c21120.1	NA	0.674963689	NA	NA	Potri.008G178200.1	AT1G67580.2
Potra2n10c21121.1	-0.5097132	0.306585092	0.839014629	NA		
Potra2n10c21122.1	-0.62747243	-0.192387499	0.000304309	0.895474078	Potri.010G147400.1	AT3G17100.2
Potra2n10c21123.1	-1.409983009	-1.55593228	NA	NA		
Potra2n10c21124.1	-1.717000707	-0.663360564	0.402992605	0.956110315		
Potra2n10c21125.1	-1.35927387	-1.507841271	NA	NA	Potri.010G147200.2	
Potra2n10c21126.1	-0.930305304	-0.094770169	6.53246E-07	0.951726178	Potri.008G103800.1	AT5G49010.1
Potra2n10c21127.1	-0.896801836	-0.331589031	0.002729014	0.71411664		
Potra2n10c21128.1	-0.266438529	0.033064208	0.073320965	0.976139323	Potri.010G146900.1	AT5G49480.1
Potra2n10c21129.1	0.21276254	0.168976499	0.020227424	0.741833788	Potri.010G146800.1	AT4G16180.2
Potra2n10c21130.1	-0.226791394	0.239943703	0.430072046	0.884553748	Potri.010G146700.1	AT3G17090.1
Potra2n10c21131.1	-1.387812713	-0.183210464	0.005122209	0.900884796	Potri.010G146500.1	AT2G16920.1
Potra2n10c21132.1	0.243897978	0.681381395	0.265460489	0.641910002	Potri.010G146400.2	AT4G16210.1
Potra2n10c21133.1	-2.337106966	-2.486725844	NA	NA	Potri.003G076000.1	AT4G25200.1
Potra2n10c21134.3	NA	NA	NA	NA	Potri.010G146200.10	

Potra2n10c21135.1	0.257261369	0.067145553	0.287408591	0.957872702	Potri.010G146100.1	AT3G06610.1
Potra2n10c21136.1	-0.280811269	0.017182345	0.052818525	0.9896961	Potri.010G146000.1	AT3G06620.1
Potra2n10c21137.1	-0.429324253	0.20313739	0.010465455	0.843443324	Potri.010G145900.2	AT2G18790.1
Potra2n10c21138.1	-2.501712481	-1.117686956	6.60479E-05	0.687729258	Potri.010G145766.1	AT5G49460.1
Potra2n10c21139.1	-1.248151541	-1.51969956	0.607711361	NA	Potri.012G075700.1	
Potra2n10c21140.1	-0.545328205	-0.187283976	0.005082649	0.849446235	Potri.010G145700.1	
Potra2n10c21141.1	-1.328951947	-0.05020672	NA	NA	Potri.010G145600.1	AT3G04690.1
Potra2n10c21142.1	2.920445301	2.181896282	0.258609682	NA	Potri.008G105600.1	AT4G24972.1
Potra2n10c21143.1	3.647052408	2.32414027	0.223860161	NA	Potri.010G145400.1	AT1G18390.2
Potra2n10c21144.1	0.414546563	-0.007139203	0.113996113	0.998438878	Potri.010G145150.1	AT3G66652.1
Potra2n10c21145.1	-0.585530703	-0.371463214	7.46397E-07	0.483645683	Potri.010G145000.1	AT3G17040.1
Potra2n10c21146.1	0.419533067	-0.128127799	0.079289498	0.963557834	Potri.010G144900.1	AT1G05760.1
Potra2n10c21147.1	NA	NA	NA	NA	Potri.010G144700.1	AT4G37580.1
Potra2n10c21148.1	-1.503094001	0.412854131	0.108877899	0.93466433	Potri.010G144600.1	
Potra2n10c21149.4	-0.144287032	0.060146812	0.319601159	0.956317179	Potri.008G105800.1	AT3G66654.2
Potra2n10c21150.1	0.723828162	-0.058751572	0.360780902	0.992319504	Potri.010G144400.1	AT3G17030.1
Potra2n10c21151.1	-1.964112374	-2.044898885	0.482885988	NA	Potri.010G144200.1	AT3G50140.1
Potra2n10c21152.1	0.404515588	0.151887424	0.032419255	0.886633917	Potri.010G144100.1	AT3G17020.1
Potra2n10c21153.1	0.071216601	-0.035923991	0.642417831	0.970930149	Potri.010G143900.1	AT3G17000.1
Potra2n10c21154.1	NA	NA	NA	NA	Potri.006G265300.1	AT5G57870.2
Potra2n10c21155.1	-2.023369572	-0.44984749	0.002724933	0.912044974	Potri.010G143100.1	AT1G09240.1
Potra2n10c21156.1	-0.4740589	0.037716938	0.000189181	0.972159498	Potri.010G143000.2	AT1G79790.1
Potra2n10c21157.1	6.392183284	1.672303158	2.44827E-30	0.270576747	Potri.010G142800.1	AT4G16260.1
Potra2n10c21158.1	-0.283241145	0.055974111	0.07387302	0.963993404	Potri.010G142700.1	AT5G49430.1
Potra2n10c21159.2	0.620092267	0.063178333	0.021738171	0.98177802	Potri.008G106900.1	AT3G16980.1
Potra2n10c21160.1	0.082879563	-0.076366597	0.826238817	0.974275337	Potri.005G030800.1	AT3G47570.1
Potra2n10c21161.2	0.856086369	0.314916137	2.26985E-06	0.641037344	Potri.010G142600.1	AT2G29290.2
Potra2n10c21162.1	0.035126629	-0.058876845	0.789528961	0.923348116	Potri.010G142400.1	AT5G49400.1
Potra2n10c21163.1	3.25596299	0.05052436	0.001892211	NA	Potri.010G142300.2	AT4G16160.2
Potra2n10c21164.1	-0.001954242	0.121724676	0.989380475	0.824317405	Potri.010G142200.1	AT3G06670.1
Potra2n10c21165.2	0.058341704	-0.025094212	0.748263939	0.991036771	Potri.010G142100.1	AT3G16950.1
Potra2n10c21166.1	0.545231466	0.034497636	0.000723339	0.982422288	Potri.010G142001.1	AT3G06700.1

Potra2n10c21167.1	0.550711519	-0.06666206	0.116410621	0.948671168	Potri.010G141900.1	AT4G02080.1
Potra2n10c21168.1	1.240232281	0.61716999	0.053016551	0.896089262	Potri.010G141800.1	AT1G02620.1
Potra2n10c21169.2	0.311683937	0.042595881	0.009416826	0.9574223	Potri.010G141700.1	AT4G16150.1
Potra2n10c21170.1	-0.765856275	-0.326052476	4.45735E-05	0.803501364	Potri.010G141600.1	AT3G16920.1
Potra2n10c21171.1	-0.730939022	0.26045616	0.506955459	0.97064682	Potri.008G108000.5	AT3G16910.1
Potra2n10c21172.1	-1.35740146	-0.070360207	0.000411873	0.99005311	Potri.010G141400.1	AT5G49360.1
Potra2n10c21173.2	-0.120560793	-0.050730645	0.635523827	0.983113084	Potri.010G141300.2	AT4G16146.1
Potra2n10c21174.4	0.559679409	-0.173827775	0.380092014	0.971828097	Potri.008G108400.1	AT5G49350.1
Potra2n10c21175.1	-0.008547761	0.098923877	0.952890246	0.917510096	Potri.010G141100.1	AT4G16144.1
Potra2n10c21176.1	-0.030109773	0.026745892	0.866440767	0.984292161		
Potra2n10c21177.1	-0.19562466	0.583123023	0.81307028	0.92522551	Potri.010G141000.1	AT5G49330.1
Potra2n10c21178.1	-1.911725814	-0.327569398	1.03091E-08	0.828625141	Potri.010G140900.1	AT3G58650.1
Potra2n10c21179.1	-0.777015488	0.04999093	0.362251936	0.994698397	Potri.010G140800.1	AT2G47480.1
Potra2n10c21180.1	-1.989396276	1.199544956	0.191917765	0.817452127	Potri.010G140700.1	AT5G14010.1
Potra2n10c21181.2	1.315149098	0.681781706	1.65175E-07	0.245596462	Potri.008G108800.2	AT1G10240.1
Potra2n10c21182.1	0.23351152	-1.478536539	NA	NA	Potri.010G140400.1	AT5G49320.1
Potra2n10c21183.1	0.555296363	-0.864666357	0.81239541	NA	Potri.010G140300.1	AT5G38895.3
Potra2n10c21184.1	-0.958966202	-0.474195295	0.001053462	0.475175307	Potri.010G140200.1	AT1G69080.1
Potra2n10c21185.1	0.50120523	0.184821632	0.001253845	0.903477395	Potri.010G140100.1	AT1G69070.1
Potra2n10c21186.1	-0.003336686	0.60310681	0.998515484	0.930203195	Potri.T142900.1	AT3G51550.1
Potra2n10c21187.1	1.664677056	NA	NA	NA	Potri.010G139600.1	AT2G26710.1
Potra2n10c21188.1	1.664677056	1.571529889	NA	NA	Potri.010G139700.1	
Potra2n10c21189.1	-3.521088867	-0.354587846	0.01585996	0.951394085	Potri.010G139600.1	AT2G26710.1
Potra2n10c21190.1	0.057869369	0.068092916	0.909541599	0.980475138		
Potra2n10c21191.1	NA	NA	NA	NA	Potri.010G139200.1	AT2G26710.1
Potra2n10c21192.1	NA	NA	NA	NA	Potri.010G139200.1	AT2G26710.1
Potra2n10c21193.1	0.009898799	0.192614823	0.961589514	0.796890714	Potri.010G139100.1	AT1G69060.1
Potra2n10c21194.1	-0.180598246	-0.040102776	0.164631854	0.977559521	Potri.010G138900.1	AT1G73430.1
Potra2n10c21195.1	-1.328951947	-1.478536539	NA	NA	Potri.006G135200.2	AT5G20850.1
Potra2n10c21196.1	0.457987538	0.337148271	2.16869E-05	0.323147091	Potri.010G138800.1	AT2G34630.2
Potra2n10c21197.1	NA	NA	NA	NA	Potri.010G138700.1	AT1G69050.1
Potra2n10c21198.1	-1.07319894	-0.311391518	1.63607E-09	0.638236514	Potri.010G138600.1	AT1G69040.2

Potra2n10c21199.1	1.459962178	1.394613675	NA	NA	Potri.010G138400.1	AT3G45070.1
Potra2n10c21200.1	1.383056169	0.94451133	0.585430214	NA	Potri.010G138400.1	AT3G45070.1
Potra2n10c21201.1	0.033185187	0.115140834	0.805797728	0.849446235	Potri.010G138300.2	AT2G03780.1
Potra2n10c21202.1	0.780960044	0.382611308	0.05803142	0.489910701	Potri.010G138100.2	AT2G03810.4
Potra2n10c21203.1	NA	1.571529889	NA	NA	Potri.010G138001.1	AT2G03830.1
Potra2n10c21204.1	-0.198908276	-0.126931472	0.296578429	0.938967511	Potri.010G137900.1	AT1G26300.1
Potra2n10c21205.1	-0.186074078	-0.161389908	0.366634113	0.91233528	Potri.010G137801.1	AT1G69020.1
Potra2n10c21206.1	-0.50088896	-0.177727283	1.50708E-07	0.694611009	Potri.010G137600.1	AT1G69010.1
Potra2n10c21207.1	NA	NA	NA	NA		
Potra2n10c21208.1	NA	NA	NA	NA	Potri.010G137500.1	
Potra2n10c21209.1	-0.627392619	-0.01020726	0.000180128	0.994692557		
Potra2n10c21210.1	-0.525114274	-0.085427557	0.000471526	0.917814867	Potri.010G137400.1	AT2G03890.1
Potra2n10c21211.1	-0.788212436	-0.199322377	0.001219834	0.917965182	Potri.010G137300.1	AT1G13635.2
Potra2n10c21212.1	-0.186072879	-0.14103476	0.047362504	0.835819291	Potri.010G137200.2	AT1G13630.1
Potra2n10c21213.1	-0.153474455	-0.1997646	0.106981883	0.790362046	Potri.010G137000.1	AT5G03490.1
Potra2n10c21214.3	-3.21087744	-1.182697402	0.001108154	0.738746172	Potri.010G136900.1	AT3G30380.2
Potra2n10c21215.1	-0.451324345	-0.099956512	0.000840325	0.934579595	Potri.010G136800.1	AT2G04030.1
Potra2n10c21216.1	0.427669266	0.357525314	9.43311E-05	0.404632832	Potri.010G136700.1	AT1G68990.1
Potra2n10c21217.2	0.217755765	0.073370195	0.472372733	0.966917036	Potri.004G111400.1	AT3G30390.2
Potra2n10c21218.1	0.219792958	-0.174526814	0.83402163	0.981401592	Potri.010G136500.1	AT1G68940.1
Potra2n10c21219.1	-0.479088018	-0.143957641	0.304118263	0.953954892	Potri.010G136300.1	AT2G37240.1
Potra2n10c21220.1	-0.396057978	0.047451623	0.00294677	0.948577865	Potri.010G136200.1	AT1G68930.1
Potra2n10c21221.1	1.64774763	NA	NA	NA		
Potra2n10c21222.1	0.02970761	0.113031378	0.85235168	0.885627229	Potri.010G135900.1	AT1G68910.1
Potra2n10c21223.1	NA	NA	NA	NA	Potri.010G135633.1	
Potra2n10c21224.1	NA	0.674963689	NA	NA	Potri.010G135600.1	
Potra2n10c21225.1	-0.152225508	-0.098497394	0.249931164	0.869186333	Potri.010G135500.1	AT1G68890.1
Potra2n10c21226.1	-2.200573815	0.085554011	NA	NA	Potri.010G134300.1	AT2G04032.1
Potra2n10c21227.1	-0.140848037	1.310667938	0.944315411	0.814981587		
Potra2n10c21228.1	-1.648371621	-0.089691206	1.45623E-08	0.974275337	Potri.010G135200.1	AT3G30530.1
Potra2n10c21229.1	-1.107778913	-0.466837256	0.554305387	NA	Potri.010G135150.1	AT1G13590.1
Potra2n10c21230.1	-1.297951005	-0.06107493	2.63292E-09	0.977323635	Potri.010G135100.2	AT1G26230.1

Potra2n10c21231.1	-0.823019951	0.368226428	0.002267874	0.807983579	Potri.010G134900.1	AT3G02360.2
Potra2n10c21232.1	-0.889091884	0.298569271	0.232833666	0.940376196		
Potra2n10c21233.1	-2.256250291	0.080258407	0.002977214	0.985964146	Potri.010G134800.2	AT4G33800.1
Potra2n10c21234.1	-0.426246069	-0.231804159	0.006099077	0.83433596	Potri.010G134700.1	AT1G13580.2
Potra2n10c21235.1	0.348471626	-0.047865336	0.000911005	0.958071483	Potri.010G134600.1	AT3G25545.1
Potra2n10c21236.1	1.877706717	1.370089628	0.057565355	0.72680202	Potri.010G134500.1	AT1G68850.1
Potra2n10c21237.1	1.555925255	0.381285601	7.79138E-35	0.751093503	Potri.010G134400.1	AT1G26190.1
Potra2n10c21238.1	0.82357019	1.895229922	NA	NA	Potri.010G134300.1	AT2G04032.1
Potra2n10c21239.1	-0.392978373	-0.296711747	0.0371859	0.784407529	Potri.010G134200.2	AT1G13570.1
Potra2n10c21240.1	-0.388861224	-0.297708807	0.041568257	0.773063484	Potri.010G134100.3	AT3G25560.1
Potra2n10c21241.1	NA	NA	NA	NA		
Potra2n10c21242.1						
Potra2n10c21243.1	-0.922354892	0.030959171	NA	NA	Potri.008G110100.1	AT5G08180.2
Potra2n10c21244.1	-0.477389347	-0.253367625	9.59833E-08	0.728883321	Potri.010G133900.1	AT3G25570.1
Potra2n10c21245.1	-0.509064525	-0.103122113	5.74938E-05	0.937687311	Potri.010G133800.1	AT1G13560.1
Potra2n10c21246.1	2.165919294	1.871975723	NA	NA	Potri.010G133300.1	AT3G61460.1
Potra2n10c21247.1	0.229489863	-0.091744155	0.275012908	0.937276488	Potri.010G133600.1	AT3G25590.1
Potra2n10c21248.1	-0.770878729	-0.117397694	0.033428111	0.953831244	Potri.010G133500.1	AT1G13480.1
Potra2n10c21249.1	0.771706157	-0.657019125	0.618755927	NA	Potri.010G133400.1	AT1G26170.1
Potra2n10c21250.1	1.97743578	1.183512341	0.481349263	NA	Potri.010G133300.1	AT3G61460.1
Potra2n10c21251.2	-0.309751178	0.193986712	0.093738023	0.827781566	Potri.008G111700.1	AT1G26160.1
Potra2n10c21252.1	0.155246895	-0.285320828	0.564448063	0.855587395		
Potra2n10c21253.1	0.199642464	-0.14263235	0.244441924	0.919424623	Potri.010G132900.1	AT1G26150.1
Potra2n10c21254.1	0.113469355	-0.432131242	0.902231706	0.946464685	Potri.010G132800.1	AT3G25600.1
Potra2n10c21255.1	1.149719871	0.207739283	5.0694E-11	0.914314663	Potri.010G132700.1	AT1G68710.1
Potra2n10c21256.1	0.412946472	0.317432916	0.00057827	0.606710262	Potri.010G132600.1	AT1G68720.1
Potra2n10c21257.1	0.114320721	-0.066986676	0.605864321	0.966917036		
Potra2n10c21258.1	-0.885460068	0.006043279	0.002676051	0.999315506	Potri.010G132500.1	AT1G68725.1
Potra2n10c21259.1	-0.861875227	0.007309349	0.003187873	0.998883928	Potri.010G132400.1	AT1G68730.1
Potra2n10c21260.1	-2.412064299	-0.7714784	2.4575E-11	0.333353361	Potri.010G132300.1	AT1G68740.1
Potra2n10c21261.1	-0.654497884	-0.093314876	5.59205E-05	0.945173238	Potri.010G132200.2	AT1G26120.1
Potra2n10c21262.1	0.95827354	-0.701182802	0.079662074	0.796890714	Potri.010G132101.1	

Potra2n10c21263.1	NA	NA	NA	NA		
Potra2n10c21264.1	0.366059024	-0.665716497	0.712426811	0.859892315		
Potra2n10c21265.1	-1.373342163	-0.175358381	0.011536489	0.922011871	Potri.010G131900.1	AT3G25620.2
Potra2n10c21266.1	0.420140222	0.080005174	0.000617615	0.955198964	Potri.010G131800.1	AT1G68750.1
Potra2n10c21267.1	NA	NA	NA	NA	Potri.010G131700.1	AT1G68760.1
Potra2n10c21268.1	-0.985953699	-0.469808969	0.326566114	0.924951347	Potri.010G131600.1	AT3G25640.1
Potra2n10c21269.1	2.383959494	1.483716055	0.035807453	NA	Potri.010G131500.1	AT1G68765.1
Potra2n10c21270.1	0.015520707	0.037203893	0.912780919	0.962661188	Potri.010G131400.2	AT1G26110.1
Potra2n10c21271.1	1.012323868	2.545587121	0.65302283	0.361949049	Potri.010G131300.1	AT3G21360.1
Potra2n10c21272.1	0.841126103	0.379233599	0.00879487	0.799868271	Potri.010G131100.1	AT1G26100.1
Potra2n10c21273.3	-0.034051171	-0.116860653	0.87154634	0.819793708	Potri.010G130800.1	AT3G25690.2
Potra2n10c21274.1	-0.044380887	0.099526103	0.829720025	0.937687311	Potri.010G130700.1	AT1G26090.1
Potra2n10c21275.1					Potri.010G130600.1	AT1G49000.1
Potra2n10c21276.1	-1.209837309	-0.746922125	0.000734257	0.183625365	Potri.010G130400.1	AT1G73965.1
Potra2n10c21277.1	-0.329729487	-0.136363457	0.028654044	0.922993749	Potri.010G130300.1	AT1G25682.1
Potra2n10c21278.1						
Potra2n10c21279.1	NA	NA	NA	NA	Potri.010G130200.1	AT1G68800.1
Potra2n10c21280.1	-1.875147379	-0.51073032	1.97792E-23	0.027320927	Potri.010G130000.1	AT1G68810.1
Potra2n10c21281.2	-1.159640596	-1.368495784	0.109098601	0.192524277	Potri.010G129932.1	AT1G68820.1
Potra2n10c21282.1	0.201264779	-0.693207506	0.437976038	0.131562686	Potri.010G129800.1	AT1G75290.1
Potra2n10c21283.1	0.431208024	0.001632593	0.023923029	0.999325699	Potri.010G129700.2	AT1G25580.1
Potra2n10c21284.1	-0.926811258	-0.621994765	0.03701332	0.714411171	Potri.010G129600.1	AT1G13245.1
Potra2n10c21285.1	-0.674223851	0.08231607	0.000321451	0.961680875	Potri.008G116800.1	AT1G68830.1
Potra2n10c21286.1	0.053662606	-0.079069949	0.890232551	0.973347974	Potri.010G129400.1	AT1G13250.1
Potra2n10c21287.1	-0.214911186	0.180994879	0.137108265	0.817452127	Potri.010G129300.1	AT1G25570.1
Potra2n10c21288.1	2.080491655	0.475840579	NA	0.80723942	Potri.008G117100.1	AT1G25560.1
Potra2n10c21289.4	-0.295678883	0.281423184	0.51262906	0.868623185	Potri.008G117200.1	AT1G13270.1
Potra2n10c21290.1	-1.881679658	-1.311198622	NA	NA	Potri.010G129000.1	AT1G13290.1
Potra2n10c21291.1	NA	NA	NA	NA		
Potra2n10c21292.1	-0.61910233	-0.767708831	NA	NA		
Potra2n10c21293.1	-0.594486423	0.080406107	0.01758175	0.972159498	Potri.010G128900.1	AT1G25550.1
Potra2n10c21294.1	-0.642588026	1.33797996	0.789511594	0.844485734		

Potra2n10c21295.1	0.316235485	-0.244913283	0.157232415	0.798648673	Potri.010G128700.1	AT1G13310.1
Potra2n10c21296.1	-0.171748345	0.087060884	0.259515669	0.943332134	Potri.010G128600.1	AT1G25540.1
Potra2n10c21297.1	-1.0008129	-0.064116243	2.77787E-05	0.974275337	Potri.010G128500.1	AT1G68660.1
Potra2n10c21298.1	0.131413133	-0.008931454	0.298871827	0.991407187	Potri.008G117900.1	AT1G25520.1
Potra2n10c21299.1	-0.111980842	-0.023090381	0.651521698	0.989300076	Potri.010G128300.1	AT1G25530.1
Potra2n10c21300.1	-0.436183775	-0.773266995	0.174956639	0.389989789	Potri.010G128200.1	AT1G25510.1
Potra2n10c21301.1	0.471673159	0.163067323	0.072639569	0.926808461	Potri.010G128100.1	AT1G25500.2
Potra2n10c21302.1	2.681825316	2.770395976	0.315677425	NA	Potri.008G118300.1	AT1G68640.1
Potra2n10c21303.1	2.675102123	2.000334024	0.001351382	0.109782024	Potri.010G127900.1	
Potra2n10c21304.1	2.998806453	NA	NA	NA	Potri.010G127800.1	AT1G68630.1
Potra2n10c21305.1	NA	1.372906117	NA	NA	Potri.010G127700.1	AT1G68610.1
Potra2n10c21306.1	0.264873639	0.121897471	0.635523827	0.971752582	Potri.010G127600.1	AT5G16080.1
Potra2n10c21307.1	-0.070528915	-0.089258966	0.58937489	0.900884796	Potri.010G127500.1	AT3G25800.1
Potra2n10c21308.1	3.751032717	0.041911433	5.29375E-05	NA	Potri.010G127400.6	AT3G18440.1
Potra2n10c21309.1	-0.251482422	-0.303755377	0.313640819	0.660636854	Potri.010G127200.1	AT3G25805.1
Potra2n10c21310.1	-0.251482422	-0.303755377	0.313640819	0.660636854	Potri.010G127300.1	AT1G68590.1
Potra2n10c21311.1	0.508084867	-0.146986792	0.289284641	0.95671968	Potri.008G118800.1	AT1G13330.1
Potra2n10c21312.1	0.17687582	-0.529703819	0.561950091	0.420180236	Potri.010G127000.1	AT1G13340.1
Potra2n10c21313.3	0.381829873	0.372836115	0.011635945	0.71102669	Potri.010G126900.10	AT3G25840.1
Potra2n10c21314.1	-0.610659417	-0.114318009	0.004881947	0.936549944	Potri.010G126800.1	AT1G68585.1
Potra2n10c21315.1	0.27104345	0.044496637	0.169626179	0.963495407	Potri.010G126700.1	AT1G68580.2
Potra2n10c21316.1	0.209866671	0.00408519	0.283924307	0.998895625	Potri.010G126600.1	AT3G25860.1
Potra2n10c21317.1	NA	NA	NA	NA	Potri.010G126500.1	AT4G39670.1
Potra2n10c21318.1	1.11010989	0.06197156	NA	NA		
Potra2n10c21319.1	1.657864123	1.593624745	0.167414196	0.724604666	Potri.010G126300.1	AT1G68570.1
Potra2n10c21320.1	-0.057365119	-0.123929015	0.642215825	0.853325932	Potri.004G100700.1	AT3G02540.1
Potra2n10c21321.1	1.756558861	0.822568259	0.178480868	NA	Potri.010G126100.1	
Potra2n10c21322.1	-0.235698141	-0.18794102	0.282780919	0.88661378	Potri.010G126000.1	AT1G13360.1
Potra2n10c21323.1	1.459962178	NA	NA	NA		
Potra2n10c21324.2	8.262637803	4.936645502	1.96896E-34	0.005003462	Potri.010G125900.2	AT3G25882.1
Potra2n10c21325.1	NA	1.394613675	NA	NA		
Potra2n10c21326.1	-0.127615137	-0.293818138	0.696112573	0.886633917	Potri.010G125800.1	AT1G68560.1

Potra2n10c21327.1	0.184873014	0.050795225	0.326454879	0.974756511	Potri.010G125700.1	AT1G68552.1
Potra2n10c21328.1	-0.44276684	0.151112765	0.010810868	0.843443324	Potri.010G125400.1	AT1G68540.1
Potra2n10c21329.1	-0.39437081	0.016273198	0.793625234	0.99905083	Potri.010G125300.1	AT1G68530.1
Potra2n10c21330.1	-0.029348317	-0.235623995	0.913070473	0.900869139	Potri.010G125200.1	AT3G25900.1
Potra2n10c21331.2	-0.030839925	-0.235981565	0.907012078	0.896627841		
Potra2n10c21332.1	1.069438096	-0.430846256	0.000561876	0.896138506	Potri.010G125100.1	AT1G68520.1
Potra2n10c21333.1	7.741158121	0.02067467	2.58965E-38	NA	Potri.010G125000.1	AT1G68510.1
Potra2n10c21334.1	1.414602299	1.573738353	0.377288022	NA	Potri.010G124900.1	AT1G25425.1
Potra2n10c21335.1	0.546900542	0.186864208	0.003988101	0.817777046	Potri.010G124800.1	AT1G13380.1
Potra2n10c21336.1	-1.06334989	0.098314669	0.262513767	0.987226805	Potri.010G124700.2	AT1G68500.1
Potra2n10c21337.1	-0.557818862	-0.081198264	0.125918355	0.974275337	Potri.010G124400.1	AT1G68490.1
Potra2n10c21338.1	-0.674983822	0.024414347	0.031390922	0.995261215	Potri.008G121100.1	AT3G25920.1
Potra2n10c21339.1	1.256636482	-1.465929376	NA	NA	Potri.010G124200.1	AT1G68480.1
Potra2n10c21340.1	0.988345373	0.282416859	0.005424966	0.890978957	Potri.008G121300.1	AT1G25420.1
Potra2n10c21341.2	0.239045666	-0.018553673	0.045443074	0.990685446	Potri.010G124000.1	AT1G77420.1
Potra2n10c21342.1	-0.190055193	0.055156019	0.775585225	0.987548286	Potri.008G121400.2	AT1G13410.1
Potra2n10c21343.1	0.511813994	-1.029574229	0.388205125	0.587693276	Potri.008G121500.1	AT1G68460.1
Potra2n10c21344.1	2.063471427	NA	NA	NA	Potri.010G123700.1	AT1G68450.1
Potra2n10c21345.1	0.099945937	0.111373811	0.685181546	0.955628098	Potri.010G123600.1	AT1G68440.1
Potra2n10c21346.1	2.10509245	0.5533433	2.05302E-33	0.549719769	Potri.010G123500.2	AT1G68290.1
Potra2n10c21347.1	2.229012273	0.434206049	1.4422E-35	0.817452127	Potri.010G123400.1	AT1G68300.1
Potra2n10c21348.1	-0.735823061	0.195469821	0.129347878	0.9091242	Potri.010G123300.1	AT3G25930.1
Potra2n10c21349.3	-0.063479817	0.054533085	0.71535012	0.973347974	Potri.010G123101.1	AT1G68260.1
Potra2n10c21350.1	1.468230514	-1.081374223	0.50982588	NA	Potri.010G123000.1	AT1G68320.1
Potra2n10c21351.1	0.623400686	0.142695474	9.62536E-05	0.943483421	Potri.008G122200.1	AT1G25350.1
Potra2n10c21352.1	0.809399417	0.276998651	0.095541548	0.908467119	Potri.010G122800.1	AT1G68330.1
Potra2n10c21353.1	-1.317292482	-1.465929376	NA	NA	Potri.004G108901.1	AT3G02360.1
Potra2n10c21354.1	0.309570628	0.081194306	0.12207402	0.940735643	Potri.010G122700.1	AT1G25370.1
Potra2n10c21355.1	0.197569125	-0.801182374	0.674401568	0.50924822	Potri.010G122600.1	AT1G68350.1
Potra2n10c21356.1	0.171374674	0.152151115	0.061297699	0.719850204	Potri.010G122500.1	AT5G38830.1
Potra2n10c21357.1	-0.060554243	-0.063994484	0.923153927	0.978251387	Potri.010G122400.1	AT1G68360.1
Potra2n10c21358.1	-0.024304711	0.018227499	0.873789146	0.9869326	Potri.010G122300.1	AT1G68370.1

Potra2n10c21359.1	-1.110631584	-0.056979949	8.11355E-09	0.97137556	Potri.010G122200.1	AT3G18290.1
Potra2n10c21360.1	-0.209964979	0.050510716	0.066960871	0.956689222	Potri.010G122100.2	AT1G25375.1
Potra2n10c21361.1	2.340207562	NA	NA	NA		
Potra2n10c21362.1	-0.556015979	-0.152367839	0.004895613	0.943483421	Potri.010G121700.1	AT1G68400.1
Potra2n10c21363.1	-0.557100571	-0.151890726	0.004799072	0.943685779	Potri.010G121800.1	AT1G68390.1
Potra2n10c21364.1	0.822488487	-1.465929376	NA	NA		
Potra2n10c21365.4	-0.508870533	-0.243051069	0.040508609	0.764173811	Potri.008G123600.4	AT1G68410.1
Potra2n10c21366.2	1.519968292	0.274748886	5.83513E-12	0.64562236	Potri.010G121500.2	AT1G25380.1
Potra2n10c21367.1	-0.393466467	-0.503419068	0.451248047	0.835819291	Potri.010G121300.1	AT1G68430.1
Potra2n10c21368.1	5.950698647	3.110957745	5.26538E-10	0.154436219	Potri.010G121100.1	AT1G25390.1
Potra2n10c21369.1	6.648344665	3.882988986	8.14458E-21	0.197483716	Potri.010G121100.1	AT1G25390.1
Potra2n10c21370.1	3.676464262	0.8502037	0.047163696	NA	Potri.010G121400.1	AT1G25390.1
Potra2n10c21371.1	3.781855394	1.421696721	4.54016E-08	0.728206951	Potri.010G120800.1	AT1G18390.2
Potra2n10c21372.2	0.953920696	0.572708709	0.071400853	0.728418847	Potri.010G120800.1	AT1G18390.2
Potra2n10c21373.1					Potri.004G076300.1	
Potra2n10c21374.1	-0.07014249	0.091645508	0.756688099	0.953831244	Potri.010G120700.1	AT2G01260.1
Potra2n10c21375.1	0.240473185	0.387227145	0.867893922	0.971828097	Potri.010G120600.1	AT5G38200.1
Potra2n10c21376.1	1.093665128	0.300703687	6.7139E-07	0.833651746	Potri.010G120500.1	AT1G68220.1
Potra2n10c21377.1	0.156731982	-0.115655957	0.699368447	0.972159498	Potri.010G120300.1	AT1G68238.1
Potra2n10c21378.2	0.462433266	0.120471379	5.50275E-06	0.849273165	Potri.010G120200.1	AT2G01220.2
Potra2n10c21379.1	-0.185468413	-0.21102904	0.28572664	0.907032966	Potri.010G120100.1	AT2G01210.1
Potra2n10c21380.1	-0.438125416	0.33769905	0.115612207	0.830470159	Potri.010G120000.1	
Potra2n10c21381.1	2.027191929	0.862675244	0.460358048	NA	Potri.010G119900.1	AT2G01200.2
Potra2n10c21382.1	0.924878678	0.103253317	0.000735392	0.950478187	Potri.010G119700.1	AT1G15060.1
Potra2n10c21383.1	0.302168469	0.091797097	0.015615644	0.912666272	Potri.010G119600.1	AT1G49540.1
Potra2n10c21384.1	0.272524656	0.043449538	0.14779838	0.970992968	Potri.010G119500.1	AT2G01190.1
Potra2n10c21385.1	-0.574749786	0.07037979	0.017128098	0.946464685	Potri.010G119400.1	AT3G02600.2
Potra2n10c21386.1	-0.365009136	0.088563472	0.032815509	0.914551931	Potri.010G119300.1	AT1G15080.1
Potra2n10c21387.1	2.5907721	0.902623211	5.24181E-11	0.616163505	Potri.010G119100.1	AT2G01170.1
Potra2n10c21388.1	0.001683521	0.026253882	0.993689529	0.982422288	Potri.010G118900.1	AT1G68185.1
Potra2n10c21389.1	-1.05504014	-0.077514192	0.000125346	0.981772014	Potri.010G118700.1	AT1G68200.2
Potra2n10c21390.1	1.275854632	0.400661605	0.040616429	0.936360532	Potri.010G118500.1	

Potra2n10c21391.1	1.105422184	-0.912972762	0.524079404	NA	Potri.010G118400.1	
Potra2n10c21392.1	-0.387181136	-0.103810481	0.090547032	0.921065381	Potri.010G118300.1	AT1G66920.2
Potra2n10c21393.2	-0.61910233	0.715370254	NA	NA	Potri.010G118200.1	AT5G08370.1
Potra2n10c21394.1	-1.35927387	-1.507841271	NA	NA	Potri.010G118100.1	AT1G79620.1
Potra2n10c21395.1	NA	NA	NA	NA		
Potra2n10c21396.1	-0.514625424	-0.48272495	0.000192276	0.605100478	Potri.010G118000.1	AT1G15100.1
Potra2n10c21397.2	-0.023800357	0.279047382	0.915664997	0.673588661	Potri.008G125800.3	AT3G18210.2
Potra2n10c21398.1	0.588211476	0.067779492	4.60328E-08	0.953831244	Potri.010G117900.1	AT2G01140.1
Potra2n10c21399.1	-0.00872038	-0.014998272	0.944384812	0.9859176	Potri.010G117800.2	AT2G01130.1
Potra2n10c21400.1	-0.511708038	1.303926972	NA	NA		
Potra2n10c21401.1	-0.236897687	0.117517745	0.431198315	0.945150162	Potri.010G117500.2	AT1G15110.1
Potra2n10c21402.1	-0.355361467	0.089775137	0.215897831	0.960656795		
Potra2n10c21403.1	-1.96050712	-2.108318007	NA	NA	Potri.010G117400.1	AT1G08510.1
Potra2n10c21404.1	2.735991243	NA	NA	NA	Potri.010G117300.1	AT2G01120.2
Potra2n10c21405.1	-0.095996491	-0.122265904	0.466862311	0.824905557	Potri.010G117200.1	AT2G01110.1
Potra2n10c21406.1	0.138833288	0.672436242	0.757092983	0.475175307	Potri.010G117100.2	AT1G71150.1
Potra2n10c21407.3	2.909744113	-2.594682774	0.00034264	NA	Potri.010G116900.1	AT1G15160.1
Potra2n10c21408.1	3.421818159	0.417743116	6.06298E-25	0.926235628	Potri.010G116700.1	AT5G52450.1
Potra2n10c21409.1	0.409576817	0.083431644	0.003236681	0.932328711	Potri.010G116600.1	AT5G08680.1
Potra2n10c21410.1	0.061459005	-0.15974939	0.752084781	0.872233138	Potri.010G116500.2	AT1G15140.1
Potra2n10c21411.1	-0.27526753	-0.158565015	0.020533689	0.842247476	Potri.010G116400.1	AT1G15130.1
Potra2n10c21412.1	-2.670675713	-1.214343233	0.273366548	NA	Potri.010G116300.1	AT5G24910.1
Potra2n10c21413.1	-0.086355212	-0.022139207	0.475689861	0.984292161	Potri.010G116200.1	
Potra2n10c21414.1	1.018746736	0.319849577	0.016575157	0.372755083	Potri.010G116000.1	AT3G03280.1
Potra2n10c21415.1	NA	NA	NA	NA	Potri.010G115900.1	
Potra2n10c21416.1	-0.825691689	-0.185493607	1.77936E-07	0.885375133	Potri.010G115800.2	AT5G13240.1
Potra2n10c21417.1	-0.003746444	0.046840271	0.980963321	0.9761362	Potri.010G115700.2	AT2G01270.1
Potra2n10c21418.1	0.321405999	0.017305124	0.716512176	0.998084737		
Potra2n10c21419.1	-1.284231867	-0.323611626	3.28289E-06	0.817452127	Potri.010G115600.1	AT2G01275.1
Potra2n10c21420.1	-0.751078052	-0.297101599	1.01262E-05	0.5722922	Potri.010G115500.1	AT1G71110.1
Potra2n10c21421.1	NA	3.495980907	NA	NA		
Potra2n10c21422.1	-0.037422874	-0.38978698	0.868659454	0.525835282		

Potra2n10c21423.1	-0.056147602	-0.402740912	0.814166413	0.502241868	Potri.010G115300.1	AT2G01290.1
Potra2n10c21424.1	-0.025251481	0.023522663	0.890958567	0.985393296		
Potra2n10c21425.1	3.617438048	1.363811008	0.229419662	NA	Potri.010G115100.1	AT2G01300.1
Potra2n10c21426.1	2.328120265	1.872529565	0.400444451	NA	Potri.010G115100.1	AT2G01300.1
Potra2n10c21427.3	0.325391641	0.17130564	0.060527715	0.871720501	Potri.010G115000.1	AT2G01320.2
Potra2n10c21428.1	0.034910284	-0.069295109	0.802763301	0.949705962		
Potra2n10c21429.1	0.073526013	-0.01933696	0.570831101	0.987226805	Potri.010G114900.1	AT1G71080.1
Potra2n10c21430.1	0.108256797	-2.486725844	0.979833934	NA		
Potra2n10c21431.1	0.294052097	0.081029914	0.025335362	0.930598202	Potri.010G114800.3	AT1G71070.1
Potra2n10c21432.1	0.121826717	-0.09646651	0.574647746	0.948577865	Potri.010G114700.1	AT1G71060.1
Potra2n10c21433.1	3.567834099	-0.157732018	NA	0.978203092	Potri.010G114600.1	AT1G22990.1
Potra2n10c21434.1	2.632524198	NA	NA	NA	Potri.010G114500.1	AT5G39390.1
Potra2n10c21435.1	0.412271688	0.080122638	0.026604729	0.959045168	Potri.010G114400.1	AT2G35040.1
Potra2n10c21436.1	-1.455604087	-0.855921967	5.28691E-06	0.646171083	Potri.010G114300.1	AT1G23000.1
Potra2n10c21437.1	1.592475263	0.553469576	8.09723E-16	0.621109642	Potri.010G114200.1	AT1G71040.1
Potra2n10c21438.1	0.325745858	0.300104731	0.109513173	0.835819291	Potri.010G114100.1	
Potra2n10c21439.1	5.709591889	1.937598597	1.59395E-05	NA	Potri.010G114000.1	AT4G38620.1
Potra2n10c21440.1	-0.469931954	-0.095128161	0.021218547	0.959881296	Potri.010G113900.1	AT1G23030.1
Potra2n10c21441.1	2.409567423	NA	NA	NA	Potri.010G113800.1	AT2G01340.1
Potra2n10c21442.1	-0.018961291	0.04074511	0.955822915	0.9761362	Potri.010G113700.2	AT1G71010.1
Potra2n10c21443.2	-1.330972179	1.585408671	0.353068893	0.68282906	Potri.010G113500.2	AT2G01350.1
Potra2n10c21444.1	2.900414336	1.381953902	NA	NA		
Potra2n10c21445.2	0.483690533	-0.757252357	0.851256252	NA		
Potra2n10c21446.1	-0.330832	0.023052685	0.422171404	0.995592415	Potri.010G113400.1	AT1G71000.1
Potra2n10c21447.1	0.396103114	-0.073126511	0.019937084	0.972159498	Potri.010G113300.2	AT1G23040.1
Potra2n10c21448.1	-0.187166897	-0.278463713	0.373926908	0.624804486	Potri.010G113100.1	AT1G23050.1
Potra2n10c21449.1	-0.115537855	-0.191943416	0.580085616	0.755774501	Potri.010G113000.1	AT1G70950.1
Potra2n10c21450.1	1.017231371	3.483342167	0.395777094	NA	Potri.010G112900.1	AT5G37810.1
Potra2n10c21451.2	0.49259261	0.692131828	0.105806855	0.660238812	Potri.010G112800.1	AT1G70940.1
Potra2n10c21452.1	1.459962178	NA	NA	NA		
Potra2n10c21453.1	-0.938178003	-0.16265807	0.143812707	0.97137556	Potri.010G112600.1	AT2G01430.1
Potra2n10c21454.1	-0.377166323	-0.009920217	0.00085103	0.995579852	Potri.010G112500.1	AT2G01440.1

Potra2n10c21455.1	-0.062830981	0.010713063	0.863953054	0.996032145	Potri.010G112300.1	AT1G14990.1
Potra2n10c21456.1	NA	2.032498096	NA	NA	Potri.008G129800.1	AT1G15000.1
Potra2n10c21457.1	0.807060218	0.296342687	7.55464E-10	0.586156493	Potri.010G112200.2	AT1G73670.1
Potra2n10c21458.1	-0.204372344	-0.001604653	0.305482822	0.999325699	Potri.010G112000.1	AT1G30110.1
Potra2n10c21459.3	-0.521111135	-0.135282513	0.001060072	0.852284301	Potri.010G111900.1	AT2G01460.1
Potra2n10c21460.1	0.008152251	0.029189452	0.965525849	0.98177802	Potri.008G130400.1	AT1G23090.1
Potra2n10c21461.1						
Potra2n10c21462.1	0.648805134	-0.023836113	0.003006319	0.992986441	Potri.010G111600.1	AT1G23100.1
Potra2n10c21463.1	0.1154386	0.054494613	0.518193253	0.973347974	Potri.010G111500.1	AT2G01480.1
Potra2n10c21464.1	1.459962178	NA	NA	NA	Potri.010G111400.1	AT3G18010.1
Potra2n10c21465.1	0.434176884	0.053520836	0.287649245	0.985393296	Potri.010G111300.1	AT1G23110.5
Potra2n10c21466.1	1.64774763	NA	NA	NA	Potri.010G111200.1	AT2G01505.1
Potra2n10c21467.1	0.725169359	-0.022327097	0.028367558	0.993815835	Potri.010G111100.1	
Potra2n10c21468.1	2.110346213	-0.767708831	NA	NA	Potri.010G111000.1	AT1G70830.1
Potra2n10c21469.1	-0.034977457	-1.474967291	0.960466322	0.341783585	Potri.010G111000.1	AT1G70830.1
Potra2n10c21470.1	-2.337106966	-1.07654148	NA	NA	Potri.010G110900.1	AT1G70790.2
Potra2n10c21471.1	0.462732115	-0.111006409	0.064359397	0.937846405	Potri.010G110800.1	AT1G70780.1
Potra2n10c21472.1	-1.955977263	-1.013515382	0.543210155	NA		
Potra2n10c21473.1	NA	2.141662216	NA	NA		
Potra2n10c21474.1	-0.276702197	-0.045419275	0.004328279	0.975578749	Potri.010G110700.1	AT2G01570.1
Potra2n10c21475.1	0.82357019	1.571529889	NA	NA	Potri.010G110600.1	AT5G19120.1
Potra2n10c21476.1	0.454317208	0.108334085	0.000140098	0.881062472	Potri.010G110500.1	AT1G23170.2
Potra2n10c21477.1	-0.103132782	-0.02011849	0.772939684	0.993589787		
Potra2n10c21478.1	0.806034137	0.24893365	1.85707E-07	0.760731888	Potri.010G110400.1	AT1G70760.1
Potra2n10c21479.1	0.167571911	0.094075181	0.404196736	0.955628098	Potri.008G131900.1	AT1G23180.1
Potra2n10c21480.1	0.420420363	0.438007854	0.261747293	0.779077802		
Potra2n10c21481.1	-0.971688449	-0.187074116	0.000579788	0.917746944	Potri.010G110100.2	AT2G01590.1
Potra2n10c21482.1	0.067395733	0.063975083	0.553248411	0.953831244	Potri.010G110000.1	AT2G01600.1
Potra2n10c21483.1	1.245744892	-0.059153904	NA	NA		
Potra2n10c21484.2	-0.210091433	0.131329092	0.239034357	0.864223715	Potri.010G109900.1	AT1G70750.1
Potra2n10c21485.1	-0.049512683	0.053837251	0.824805896	0.959122697	Potri.010G109700.2	AT1G70740.1
Potra2n10c21486.1	-0.505270142	-0.731184952	0.184786561	0.50924822	Potri.010G109600.1	AT1G14900.1

Potra2n10c21487.1	-0.34958964	-0.195528973	0.055653511	0.878942767	Potri.010G109500.2	AT1G70730.3
Potra2n10c21488.1	0.896190698	0.863873905	NA	NA	Potri.010G109400.1	AT1G23200.1
Potra2n10c21489.1	-1.705331034	-0.429177209	4.39348E-06	0.305728522	Potri.010G109300.2	AT1G14890.1
Potra2n10c21490.1	-2.178414235	-0.884568375	0.000255133	0.576548403	Potri.010G109200.1	AT1G70710.1
Potra2n10c21491.1	2.87091053	0.979701152	7.06661E-15	0.335923144	Potri.010G109100.4	AT1G14870.1
Potra2n10c21492.1	2.293478653	0.877738927	1.51514E-35	0.101151554	Potri.010G108800.1	AT1G14870.1
Potra2n10c21493.3	0.506918644	0.159614589	0.00014611	0.874808359	Potri.010G108600.1	AT2G01620.1
Potra2n10c21494.1	0.248819532	0.306611056	0.204557414	0.815348505	Potri.010G108500.1	AT2G01630.1
Potra2n10c21495.5	1.718879826	1.777849161	0.373191819	NA	Potri.010G108350.1	AT1G23230.1
Potra2n10c21496.1	1.351401812	0.517851893	8.21935E-21	0.372672736	Potri.010G108200.3	AT3G17860.1
Potra2n10c21497.1	-0.324466474	0.101996797	0.032193203	0.962905796	Potri.010G108100.1	AT2G01660.1
Potra2n10c21498.1	0.031749057	-0.118235585	0.874395676	0.912990315	Potri.010G108000.1	AT5G17190.1
Potra2n10c21499.1	NA	NA	NA	NA		
Potra2n10c21500.1	-1.271207155	-0.240230556	2.63327E-05	0.846399928	Potri.008G133800.1	AT1G70670.1
Potra2n10c21501.1	-0.057283047	-0.07680984	0.704509669	0.939653927	Potri.010G107700.2	AT1G23260.1
Potra2n10c21502.1	NA	1.363811008	NA	NA	Potri.010G107600.1	AT2G12646.1
Potra2n10c21503.1	0.84889602	0.179478742	2.6781E-08	0.814981587	Potri.010G107500.1	AT1G03400.1
Potra2n10c21504.3	0.670035001	0.169237098	1.05188E-06	0.790623525	Potri.010G107400.1	AT1G70650.1
Potra2n10c21505.1	3.103340168	1.042760648	0.000538224	0.59929692	Potri.010G107300.1	AT2G01670.1
Potra2n10c21506.1	3.581556092	1.279657449	0.002405158	NA	Potri.010G107200.1	AT1G67920.1
Potra2n10c21507.1	-0.192641319	-1.064169424	0.899624647	0.817799498	Potri.010G107100.1	AT1G24620.1
Potra2n10c21508.1	1.620279082	0.885360232	1.60859E-10	0.192524277	Potri.010G106900.1	AT2G34930.1
Potra2n10c21509.1	-0.070717161	0.143329132	0.761285074	0.809914834	Potri.010G106800.1	AT2G01680.1
Potra2n10c21510.1	0.228136595	0.230257157	0.021180085	0.549979426	Potri.010G106700.1	AT2G01690.1
Potra2n10c21511.2	-0.035153263	0.531770103	0.955822915	0.835819291	Potri.010G106600.1	
Potra2n10c21512.1	-0.040264225	0.083858651	0.781598944	0.94795983	Potri.010G106400.2	AT1G24880.1
Potra2n10c21513.1	0.214636034	0.03702855	0.009790644	0.955628098	Potri.010G106300.1	AT1G68070.1
Potra2n10c21514.1	-0.042286984	-0.011223482	0.855703789	0.995565508	Potri.010G106200.1	AT2G01730.1
Potra2n10c21515.1	-0.18681799	-0.028899535	0.057766415	0.982933413	Potri.010G106100.1	AT1G68060.1
Potra2n10c21516.3	-0.460303359	-0.170702473	0.009722053	0.922011871	Potri.010G105900.1	AT1G14830.1
Potra2n10c21517.1	-0.376694192	1.675830635	NA	NA	Potri.010G105800.1	AT2G34930.1
Potra2n10c21518.1	-1.521616275	-0.43455339	7.94534E-16	0.859892315	Potri.010G105700.10	AT1G68050.1

Potra2n10c21519.1	-0.906035219	0.040215259	0.092558503	0.995005375		
Potra2n10c21520.1	-0.533805972	-0.061029782	1.54119E-07	0.94795983	Potri.010G105600.1	AT4G16110.1
Potra2n10c21521.1	1.089609259	1.894822715	0.542033434	NA	Potri.010G105400.1	AT1G14820.3
Potra2n10c21522.1	0.861583113	0.355799382	1.78408E-09	0.874808359	Potri.010G104900.1	AT1G14790.1
Potra2n10c21523.1	1.64774763	1.363811008	NA	NA	Potri.010G104800.1	AT1G68040.1
Potra2n10c21524.1	0.498008168	0.942899662	0.185396538	0.110163244	Potri.010G104600.1	AT1G14780.1
Potra2n10c21525.1	0.273413176	0.586359546	0.293746748	0.672267894	Potri.010G104500.1	AT1G68020.2
Potra2n10c21526.1	NA	2.676364087	NA	NA	Potri.010G104400.1	AT1G67980.1
Potra2n10c21527.1	1.686700389	0.481096128	7.40097E-18	0.657719957	Potri.010G104300.1	AT1G67970.1
Potra2n10c21528.1	0.776219125	0.264982735	0.015995087	0.917510096	Potri.010G104200.1	AT2G01770.1
Potra2n10c21529.1	NA	NA	NA	NA	Potri.010G104100.1	AT2G01770.1
Potra2n10c21530.1	NA	NA	NA	NA	Potri.010G103900.1	AT1G16020.2
Potra2n10c21531.1	-1.454523418	-0.407196709	0.106023177	0.943819961	Potri.010G103800.1	AT1G14750.1
Potra2n10c21532.2	-2.591310468	-0.516520068	0.04116075	0.914162682	Potri.010G103600.2	AT4G17720.1
Potra2n10c21533.1	-0.588154652	-0.118708618	0.295976825	0.981772014	Potri.010G103500.1	AT2G01818.1
Potra2n10c21534.1	0.104050345	0.069703835	0.559988148	0.93263929	Potri.010G103400.1	AT1G67960.1
Potra2n10c21535.1	3.926397274	1.632884875	1.87871E-25	0.089821513	Potri.010G103300.1	AT1G34300.1
Potra2n10c21536.1	0.112048675	-0.282555718	0.757813744	0.566924986	Potri.010G103000.1	AT1G66150.1
Potra2n10c21537.1	-0.292371923	-0.109177998	0.274729829	0.922681078		
Potra2n10c21538.1	-0.424287497	0.144710507	0.062179291	0.910878765		
Potra2n10c21539.1	-2.381304249	-1.855448163	0.353847753	NA	Potri.010G102800.1	
Potra2n10c21540.1	NA	2.02236598	NA	NA	Potri.010G102800.1	
Potra2n10c21541.1	-0.090346784	0.069193509	0.758293533	0.971537796	Potri.010G102700.1	AT1G66140.1
Potra2n10c21542.1	-0.047406605	0.068864807	0.876539082	0.971593102	Potri.001G285500.1	AT5G09590.1
Potra2n10c21543.1	0.216465565	0.296461321	0.076322289	0.759797728	Potri.010G102600.1	AT1G14740.1
Potra2n10c21544.1	NA	NA	NA	NA		
Potra2n10c21545.1	1.416019654	0.624214004	0.074923487	0.858174358	Potri.010G102400.1	AT1G14730.1
Potra2n10c21546.1	-0.164909234	0.242707713	0.439561856	0.903934429	Potri.010G102300.1	AT1G14720.1
Potra2n10c21547.1	1.64774763	1.363811008	NA	NA		
Potra2n10c21548.1	0.385917318	0.189311907	0.313258378	0.93154079	Potri.010G102200.1	AT1G25220.1
Potra2n10c21549.1	1.192536951	0.701196662	0.383537345	0.886633917		
Potra2n10c21550.2	0.680016108	0.069959377	6.79782E-09	0.960981446	Potri.010G102100.2	AT1G14710.2

Potra2n10c21551.1	0.156571986	0.098098372	0.197494037	0.87679119	Potri.010G102000.1	AT1G03280.1
Potra2n10c21552.1	0.525769639	0.144666764	0.022690069	0.905404355	Potri.010G101900.1	AT3G17790.1
Potra2n10c21553.1	-2.660724343	-2.784318059	0.267999484	NA	Potri.010G101700.1	AT2G01900.1
Potra2n10c21554.1					Potri.008G139900.1	AT1G68100.1
Potra2n10c21555.1	-1.432122289	-0.549950126	0.178078162	0.920141992	Potri.010G101500.1	AT2G35585.1
Potra2n10c21556.1	-0.244263917	-0.196500924	0.017731692	0.501154172	Potri.010G101400.1	AT2G01930.1
Potra2n10c21557.1	-2.379490373	-3.251802187	NA	NA	Potri.010G101300.1	AT5G22260.1
Potra2n10c21558.1	1.459962178	NA	NA	NA		
Potra2n10c21559.1	0.065289523	-0.154537812	0.679357228	0.813575895	Potri.010G101200.1	AT2G01940.1
Potra2n10c21560.1	-0.825881361	-0.500510831	0.023823664	0.808168288	Potri.010G101100.1	AT2G01950.1
Potra2n10c21561.1	0.081565576	0.124179593	0.496142482	0.786910796	Potri.010G100900.1	AT2G01980.1
Potra2n10c21562.1	3.219065373	1.553619495	1.30941E-53	9.49435E-07	Potri.010G100800.1	AT2G30070.1
Potra2n10c21563.1	2.898909687	2.103414927	4.76011E-11	0.00782857		
Potra2n10c21564.1	NA	NA	NA	NA		
Potra2n10c21565.1	-1.637853815	-0.301380571	4.70351E-12	0.82876584	Potri.010G100700.1	AT1G14630.1
Potra2n10c21566.4	-0.52783958	-0.255592673	3.91282E-06	0.824905557	Potri.019G047200.4	AT5G16760.1
Potra2n10c21567.1	5.937398943	2.676364087	0.00010923	NA	Potri.010G100500.1	AT2G02010.1
Potra2n10c21568.2	0.016221415	-0.035116483	0.905806632	0.960981446	Potri.010G100400.1	AT2G30050.1
Potra2n10c21569.1	-0.359325446	-0.186351027	0.010366522	0.88365924	Potri.010G100200.1	AT2G02040.1
Potra2n10c21570.1	-0.301759456	-0.107860259	0.071638241	0.936625864	Potri.008G141400.1	AT2G46290.1
Potra2n10c21571.1	0.044615798	-0.128000869	0.822743722	0.930598202		
Potra2n10c21572.1	-0.064386904	-0.169545131	0.682984614	0.822982825	Potri.008G141900.1	AT2G02050.1
Potra2n10c21573.1	-0.17594044	0.04036844	0.584188122	0.983408601		
Potra2n10c21574.1	0.095087744	0.033470057	0.733434296	0.983944804	Potri.010G099800.1	
Potra2n10c21575.1	1.961598165	-0.519378403	0.139451037	NA	Potri.010G099600.1	AT1G14600.1
Potra2n10c21576.1						
Potra2n10c21577.1	-0.018502387	0.073476935	0.916196387	0.924951347	Potri.010G099500.1	AT1G09290.1
Potra2n10c21578.3	-0.023618239	0.078508463	0.905492593	0.910878765	Potri.010G099400.1	AT1G14590.1
Potra2n10c21579.2	-0.202854205	-0.012464128	0.231050937	0.992934677	Potri.010G099300.1	AT1G75010.1
Potra2n10c21580.1	0.826191958	0.548557656	0.095905724	0.728883321	Potri.010G099200.1	AT2G02070.1
Potra2n10c21581.1	0.713736675	-0.787255911	0.355560807	0.845920228	Potri.010G099100.1	AT2G02070.1
Potra2n10c21582.2	0.243128759	0.172368783	0.017868162	0.846680547	Potri.010G099000.2	AT2G02090.1

Potra2n10c21583.1	0.542281922	0.48730612	0.148990049	0.606240119	Potri.008G142700.1	AT1G31050.1
Potra2n10c21584.2	-0.025682423	-0.010846457	0.815531558	0.988601759	Potri.010G098800.2	AT1G14570.2
Potra2n10c21585.1	-0.804445913	-0.439236125	0.005418776	0.590825672	Potri.010G098700.1	AT5G13770.1
Potra2n10c21586.1	0.277200487	0.288745867	0.370610122	0.817452127	Potri.010G098600.1	AT1G73400.1
Potra2n10c21587.1	0.624110793	-0.020477194	0.000451975	0.989878071	Potri.010G098500.1	AT3G18420.1
Potra2n10c21588.1	-0.030965759	-0.067834605	0.934514469	0.973896127	Potri.010G098350.1	
Potra2n10c21589.2	-0.18528903	-0.022830441	0.05350307	0.986636967	Potri.010G098300.1	AT5G19990.1
Potra2n10c21590.1	-0.021117095	-0.076887515	0.865480481	0.878606485	Potri.010G098200.1	AT2G02160.1
Potra2n10c21591.2	-0.377405262	-0.300221735	0.025764566	0.683816919	Potri.010G098000.1	AT2G02170.2
Potra2n10c21592.1	0.110279193	0.004424849	0.315543764	0.996307365	Potri.010G097900.1	AT2G02180.1
Potra2n10c21593.1	0.090797962	-0.0641101	0.494087616	0.947290893		
Potra2n10c21594.1	1.0469945	0.258299507	1.69566E-22	0.794521408	Potri.010G097800.1	AT2G37770.2
Potra2n10c21595.1	-0.403604852	-0.264482157	0.301002279	0.87458517	Potri.010G097700.1	AT2G02220.1
Potra2n10c21596.1	0.397478776	0.21498534	0.08773808	0.724504724	Potri.010G097600.1	AT3G11920.1
Potra2n10c21597.1	-0.714169014	0.448418237	0.768857825	NA		
Potra2n10c21598.1	0.219763908	-0.181522081	0.739003016	0.961820855	Potri.010G097400.1	AT3G02820.1
Potra2n10c21599.1	-0.695019921	-0.050560326	0.022852636	0.982827151	Potri.010G097200.1	AT1G10850.1
Potra2n10c21600.1	-0.297414291	-0.357543675	0.440724881	0.798673448	Potri.010G097100.1	
Potra2n10c21601.2	-0.270765813	0.023306161	0.037051868	0.983113084	Potri.010G097000.1	AT1G60670.2
Potra2n10c21602.1	-0.302998071	0.307042413	0.267855231	0.821709751	Potri.010G096900.1	
Potra2n10c21603.1	0.222994553	0.407338577	0.747212008	0.892391656	Potri.010G096800.1	AT4G21200.1
Potra2n10c21604.2	-0.695911271	0.013814302	0.007353608	0.995409858	Potri.010G096700.2	
Potra2n10c21605.1	0.473519268	0.279148966	0.152646314	0.850863374	Potri.010G096600.1	AT1G68660.1
Potra2n10c21606.2	0.24030541	0.082401571	0.198144242	0.953608209	Potri.008G145600.1	AT5G26830.1
Potra2n10c21607.1	-0.91952342	-0.056195971	NA	NA		
Potra2n10c21608.1	-1.740460848	-0.326555926	3.85943E-07	0.859478649	Potri.010G096400.1	AT1G60060.1
Potra2n10c21609.1	NA	NA	NA	NA		
Potra2n10c21610.1	-1.523297568	-0.012249629	1.43497E-17	0.996032145	Potri.010G096100.1	AT1G70260.1
Potra2n10c21611.1	-0.596197253	-0.578258263	0.557158499	0.910815461	Potri.010G096000.1	AT1G24020.1
Potra2n10c21612.5	-0.59147896	-0.060537159	0.000548436	0.977559521	Potri.010G095900.1	AT1G24030.2
Potra2n10c21613.1	-0.586727571	-0.063644174	0.000280737	0.975578749	Potri.010G095800.1	AT1G24040.2
Potra2n10c21614.1	1.313062177	-0.003328585	5.96319E-05	0.999325699	Potri.010G095700.1	AT1G70230.1

Potra2n10c21615.1	0.105473894	-0.204909727	0.52338025	0.834823243	Potri.010G095600.1	AT1G24050.1
Potra2n10c21616.2	-0.171304374	-0.614705519	0.813638317	0.825064676	Potri.010G095500.1	AT5G62890.2
Potra2n10c21617.1	1.327004482	0.691736295	0.19998932	0.917892599	Potri.010G095400.1	AT1G60010.1
Potra2n10c21618.1	1.589103719	-0.077018216	3.20533E-07	0.976963804	Potri.010G095200.2	AT1G70210.1
Potra2n10c21619.1	-2.938187892	-0.752581347	0.237925006	NA	Potri.001G344400.1	AT5G40510.1
Potra2n10c21620.1	0.24899851	0.151934471	0.163166213	0.857189843	Potri.010G095100.1	AT1G24090.1
Potra2n10c21621.1	0.078770057	-0.043788609	0.795907567	0.98177802	Potri.010G094900.1	AT1G10522.1
Potra2n10c21622.1	0.347016929	0.062499088	0.180283098	0.948577865	Potri.010G094800.1	AT1G10520.1
Potra2n10c21623.1	0.058161081	0.117588577	0.723143802	0.906377597	Potri.010G094700.1	AT1G60200.1
Potra2n10c21624.1	0.587029652	-0.009435968	0.020389762	0.99702095	Potri.010G094600.1	AT1G70310.1
Potra2n10c21625.1	0.255713362	0.525519619	0.114462571	0.601577719	Potri.010G094400.1	AT1G60160.1
Potra2n10c21626.1	-0.044267043	-0.029965201	0.820389214	0.974275337	Potri.010G094300.1	AT1G70300.1
Potra2n10c21627.1	-0.257882126	-0.307222016	0.406626755	0.859892315		
Potra2n10c21628.3	-0.257588642	-0.240246779	0.31246177	0.865611082	Potri.010G094200.1	AT1G70280.2
Potra2n10c21629.1	0.212105984	0.069048331	0.344541793	0.971432539	Potri.010G094100.1	AT1G26850.1
Potra2n10c21630.1	0.208358481	0.086599172	0.169498445	0.939653927	Potri.010G094000.1	AT2G02910.1
Potra2n10c21631.1	-0.39097211	-0.177880804	0.090353333	0.866705403	Potri.010G093900.1	AT1G14310.1
Potra2n10c21632.1	0.052557945	-0.000123825	0.798599493	0.999937671	Potri.010G093700.1	AT2G02800.1
Potra2n10c21633.2	-0.192060224	-0.08764107	0.271558702	0.959881296	Potri.010G093600.2	AT2G02860.1
Potra2n10c21634.1	-0.367838866	0.074536665	0.180443126	0.956889095	Potri.010G093400.1	AT1G69780.1
Potra2n10c21635.1	0.576738107	0.222165415	0.845188404	NA		
Potra2n10c21636.1	-0.910616288	-0.309673279	0.00370171	0.865611082	Potri.010G093300.1	AT1G14345.1
Potra2n10c21637.1	-0.000208591	-0.07302861	0.998675155	0.912666272	Potri.010G093200.1	AT1G26830.1
Potra2n10c21638.2	-0.430969857	-0.443850109	0.185396538	0.81916677	Potri.010G093000.1	AT2G02820.2
Potra2n10c21639.1	-0.653798315	-0.456751792	0.078407554	0.83433596	Potri.010G093100.1	
Potra2n10c21640.1	-0.255701522	0.222633743	0.022960581	0.855587395	Potri.010G092900.1	AT1G69830.1
Potra2n10c21641.1	0.00858508	-0.07574268	0.95480435	0.949960494	Potri.010G092700.1	AT1G27000.1
Potra2n10c21642.1	0.82357019	NA	NA	NA	Potri.010G092800.1	AT3G54320.1
Potra2n10c21643.1	-0.345667403	-0.117135702	0.065874919	0.910815461	Potri.010G092600.2	AT5G20220.2
Potra2n10c21644.1	0.123335863	0.177310949	0.659731258	0.899757743	Potri.010G092500.1	AT1G14410.1
Potra2n10c21645.1	-2.780964575	1.465957704	0.013313891	0.302692927	Potri.008G149200.2	AT1G54870.1
Potra2n10c21646.1	0.092667732	-0.152196373	0.61983939	0.884155312	Potri.010G092300.1	AT4G14220.1

Potra2n10c21647.1	0.06230632	-0.076739829	0.753798468	0.951726178	Potri.010G092200.1	AT5G19210.2
Potra2n10c21648.1	-0.377882993	-0.078993272	0.012613584	0.933286951	Potri.010G092100.1	AT3G05290.1
Potra2n10c21649.2	-0.957930141	-0.742154368	0.064045333	0.694769714	Potri.010G092000.3	AT3G06030.1
Potra2n10c21650.1	-0.97909608	-0.700881806	0.059159283	0.752657973		
Potra2n10c21651.1	-0.149453686	-0.094644843	0.285066055	0.910515824	Potri.010G091500.1	AT5G19280.1
Potra2n10c21652.3	-0.089979653	-0.025136066	0.67957233	0.989304496	Potri.008G149800.1	AT5G19300.1
Potra2n10c21653.3	0.021557205	0.020242045	0.861722736	0.985016122	Potri.010G091200.1	AT3G06010.1
Potra2n10c21654.1	-0.043690339	-0.148311441	0.829027321	0.902857376	Potri.010G091100.1	AT5G19320.1
Potra2n10c21655.2	-0.28922086	-0.088463685	0.000989013	0.882699919	Potri.010G090900.1	AT5G19330.1
Potra2n10c21656.1	-0.177815611	-0.073117281	0.23224281	0.973347974	Potri.010G090800.1	AT3G05990.1
Potra2n10c21657.1	-0.228834289	-0.170288312	0.197660866	0.926546506	Potri.010G090700.1	AT3G05980.1
Potra2n10c21658.1	-1.948246706	-1.124051852	0.000531851	0.449772714	Potri.010G090500.1	AT5G26230.1
Potra2n10c21659.1	0.134008748	0.023833964	0.240639722	0.9761362	Potri.010G090400.1	AT4G19600.1
Potra2n10c21660.1	1.011639793	-0.204248008	1.80758E-10	0.907032966	Potri.010G090200.1	AT3G05970.1
Potra2n10c21661.1	-0.108197364	0.040890086	0.324232407	0.966191551	Potri.010G090100.1	AT5G26240.1
Potra2n10c21662.1	-2.396052862	-1.112412337	NA	NA	Potri.003G186300.1	AT5G22250.1
Potra2n10c21663.1	-2.426667562	-3.940257936	0.483030954	NA	Potri.010G090000.1	AT5G26250.1
Potra2n10c21664.1	-1.094062421	-0.521999971	6.21153E-06	0.773063484	Potri.010G089900.1	AT5G26330.1
Potra2n10c21665.1	1.890794058	0.448897027	1.06119E-14	0.778013962	Potri.010G089800.1	AT5G26340.1
Potra2n10c21666.3	-0.328590161	-0.084321138	0.031797297	0.943483421	Potri.010G089700.1	AT3G22380.1
Potra2n10c21667.2	-0.138236211	0.08440916	0.302253907	0.939653927	Potri.010G089600.1	AT4G14950.1
Potra2n10c21668.1	NA	NA	NA	NA		
Potra2n10c21669.1	0.329115063	0.315049759	0.123109881	0.707347586	Potri.010G089500.1	AT3G22400.1
Potra2n10c21670.1	-0.744695687	0.043389028	0.016137678	0.989475841	Potri.010G089400.1	AT1G03130.1
Potra2n10c21671.1	-0.458235977	0.839267601	0.563861704	0.469515757	Potri.010G089300.2	AT2G32150.1
Potra2n10c21672.1	-2.367361922	-1.072293905	NA	NA	Potri.006G065300.1	AT2G25320.1
Potra2n10c21673.1	-0.912538756	-0.144526116	0.02483577	0.966917036	Potri.010G089200.1	AT5G19530.1
Potra2n10c21674.1	2.085266755	-0.240037865	1.5458E-19	0.869204496	Potri.010G089050.1	AT1G31690.1
Potra2n10c21675.1	-0.15648844	-1.148323497	0.871683154	0.733421509	Potri.010G088900.1	AT4G14940.1
Potra2n10c21676.1	-1.293563118	-0.24130576	0.372381332	0.98177802	Potri.010G088500.1	AT1G77780.1
Potra2n10c21677.2	-1.442933864	-0.589231436	0.000188125	0.764173811	Potri.010G088300.1	AT1G05370.1
Potra2n10c21678.1	-0.388280438	0.539935349	0.342498354	0.848205259	Potri.010G088200.1	AT5G43150.1

Potra2n10c21679.1	0.685800909	0.164234594	4.65708E-05	0.828625141	Potri.010G088000.1	AT4G14930.1
Potra2n10c21680.1	0.295181001	-0.212055225	0.275455593	0.885375133	Potri.010G087900.1	AT3G04910.1
Potra2n10c21681.1	0.285513368	-0.151196125	0.103652913	0.874808359	Potri.010G087800.1	AT5G28060.1
Potra2n10c21682.1	0.114324236	0.172672942	0.352833217	0.818161208	Potri.010G087700.1	AT4G14920.1
Potra2n10c21683.1	0.066779983	0.032588862	0.635523827	0.974275337	Potri.010G087600.2	AT4G14910.1
Potra2n10c21684.1	0.01503486	-0.411630725	0.994130857	NA		
Potra2n10c21685.1	0.03869711	0.163394779	0.739269509	0.646274123	Potri.010G087400.1	AT3G22430.1
Potra2n10c21686.1	-3.268273293	-0.606852027	0.188453031	NA	Potri.010G087300.1	AT4G14890.1
Potra2n10c21687.1	-0.800646979	-0.188223294	0.132970309	0.951002893	Potri.010G087200.2	AT1G05420.1
Potra2n10c21688.1	-0.182021206	0.265131996	0.459685124	0.699482741	Potri.010G086900.1	AT4G14850.1
Potra2n10c21689.1	0.286697066	0.165362827	0.050253671	0.89438295	Potri.010G086700.2	AT3G22520.1
Potra2n10c21690.1	-0.065456624	0.10806762	0.603951766	0.856891638	Potri.010G086600.1	AT1G05410.1
Potra2n10c21691.1	-0.115808129	0.174710447	0.849105077	0.945454119		
Potra2n10c21692.1	-0.955245968	-0.246541888	1.28244E-10	0.759797728	Potri.010G086400.1	AT3G22530.1
Potra2n10c21693.1	-0.014103188	-0.120813611	0.951546539	0.856053092	Potri.010G086300.1	AT1G05430.1
Potra2n10c21694.1	-0.17022624	0.073459412	0.571611705	0.976000742	Potri.010G086200.1	AT4G14820.1
Potra2n10c21695.1	0.202031675	-0.335375549	0.792170633	0.950368039	Potri.010G086100.1	AT1G05440.1
Potra2n10c21696.1	-1.126265759	-0.501894933	0.002754893	0.558624809	Potri.010G086000.1	AT3G22540.1
Potra2n10c21697.1	0.2691306	0.255446031	0.077111976	0.670644878	Potri.010G085900.1	AT2G32040.1
Potra2n10c21698.1	-0.484374271	0.271050889	0.02703878	0.82876584	Potri.010G085700.1	AT3G22550.1
Potra2n10c21699.2	0.044902138	0.226364547	0.919869723	0.91998377		
Potra2n10c21700.1	1.93455854	0.515456801	1.76798E-07	0.661611864	Potri.010G085600.1	AT2G32030.1
Potra2n10c21701.3	0.027086261	0.055539678	0.841597573	0.950182516	Potri.010G085400.1	AT3G22600.1
Potra2n10c21702.1	0.020082691	0.051072773	0.884012764	0.954098985	Potri.010G085500.1	AT3G22590.1
Potra2n10c21703.1	NA	NA	NA	NA	Potri.010G085300.1	AT3G22600.1
Potra2n10c21704.1	3.547110739	-0.116467334	0.008622246	NA	Potri.010G085200.1	AT3G22620.1
Potra2n10c21705.1	NA	NA	NA	NA	Potri.010G085100.1	AT2G48130.1
Potra2n10c21706.1	NA	NA	NA	NA	Potri.010G085000.1	AT4G14805.1
Potra2n10c21707.2	-1.335899768	-0.498453693	0.001227711	0.813731863	Potri.010G084300.1	AT2G32010.2
Potra2n10c21708.1	-0.15187817	0.024389689	0.222500479	0.984596335	Potri.010G084100.1	AT4G14790.1
Potra2n10c21709.1	-0.181288077	-0.002524703	0.635949207	0.999458638	Potri.010G084000.1	AT3G22670.1
Potra2n10c21710.1	0.432697593	0.604795216	0.475256587	0.79819686	Potri.010G083900.1	AT3G22670.1

Potra2n10c21711.1	0.483136878	0.257013574	0.063949399	0.902857376	Potri.010G083900.1	AT3G22670.1
Potra2n10c21712.1	0.860093969	0.415663394	0.155210399	0.87909151	Potri.010G083800.1	AT3G22670.1
Potra2n10c21713.1	2.370533105	1.954023475	8.78333E-08	0.360126886	Potri.010G083700.1	AT3G22690.2
Potra2n10c21714.1	0.002064896	0.152306247	0.992229174	0.920729156	Potri.010G083500.1	AT3G22750.1
Potra2n10c21715.1	-0.516269093	-0.367935842	0.015650393	0.784845657	Potri.010G083400.1	AT4G14770.1
Potra2n10c21716.1	0.33315068	0.408549046	0.091657454	0.409656627	Potri.010G083300.1	AT4G14760.1
Potra2n10c21717.1	1.005540025	0.194950755	4.34985E-09	0.856053092	Potri.010G083200.1	AT4G34670.1
Potra2n10c21718.1	NA	NA	NA	NA	Potri.010G083100.1	AT3G22800.1
Potra2n10c21719.1	7.746804631	NA	5.88612E-11	NA	Potri.010G083000.1	AT3G22800.1
Potra2n10c21720.1	-0.147583867	-0.037269065	0.405047846	0.976302215	Potri.010G082900.1	AT1G04190.1
Potra2n10c21721.1	-0.061798823	-0.519292822	0.919504198	0.853917305	Potri.010G082700.1	AT3G51895.1
Potra2n10c21722.1	-1.8449514	-0.446390933	1.98425E-05	0.712127394	Potri.010G082600.1	AT4G14750.1
Potra2n10c21723.1	0.037413306	0.082921765	0.890371353	0.966434509	Potri.010G082500.2	AT4G14746.1
Potra2n10c21724.1	-0.736118659	0.382713648	0.098523253	0.874507876	Potri.010G082400.4	AT3G22810.1
Potra2n10c21725.1	0.955169738	0.18054959	0.01235252	0.948577865	Potri.010G082300.1	AT5G43860.1
Potra2n10c21726.1	-0.490076044	0.996392167	NA	NA		
Potra2n10c21727.1	-0.112022192	0.149759285	0.943523434	0.985393296	Potri.010G082200.1	AT4G14723.1
Potra2n10c21728.1	-0.096832054	0.239168605	0.695790879	0.821709751	Potri.008G157500.1	AT4G14710.1
Potra2n10c21729.1	0.535077341	0.051596866	0.183795899	0.992934677	Potri.010G082000.1	AT3G22830.1
Potra2n10c21730.1	-0.053655174	-0.140486569	0.707772375	0.896089262	Potri.010G081800.1	AT1G04200.1
Potra2n10c21731.1	1.235555174	1.91748751	0.79930293	NA	Potri.008G158300.1	AT1G60680.1
Potra2n10c21732.1	-0.001737985	0.023403789	0.98838652	0.98177802	Potri.010G081700.1	AT3G22845.1
Potra2n10c21733.1	0.337936349	0.026583101	0.002577916	0.97950444	Potri.010G081600.1	AT5G43830.1
Potra2n10c21734.1	-0.2266247	-0.380639677	0.717962111	0.885459527	Potri.010G081500.1	AT3G22880.1
Potra2n10c21735.2	0.173227192	0.116451604	0.028884984	0.787483807	Potri.010G081400.1	AT1G04210.1
Potra2n10c21736.1	2.125312886	NA	NA	NA		
Potra2n10c21737.1	-0.829899303	-0.107974658	4.64459E-06	0.923397137	Potri.010G081300.10	AT5G43810.2
Potra2n10c21738.1	0.053669228	-0.313252083	0.839560788	0.520258821	Potri.010G081200.1	AT4G14680.1
Potra2n10c21739.1	1.925907109	1.256145471	0.012741721	0.64462335	Potri.010G081100.1	AT3G22910.1
Potra2n10c21740.1	-0.278645764	-1.072518354	0.552598509	0.323954554	Potri.010G081000.2	
Potra2n10c21741.1	0.206987368	-0.276603885	0.742639004	0.857189843	Potri.010G080900.1	AT4G14640.1
Potra2n10c21742.1	2.735991243	1.571529889	NA	NA	Potri.010G080800.1	AT3G63400.1

Potra2n10c21743.1	0.135932405	0.101922928	0.281174219	0.900551258	Potri.010G080700.1	AT3G22960.1
Potra2n10c21744.1	-0.379723659	-0.258678226	0.104295878	0.773707293	Potri.010G080600.1	AT3G22970.1
Potra2n10c21745.1	0.82357019	NA	NA	NA	Potri.010G080200.1	AT2G26640.1
Potra2n10c21746.1	2.445718799	-0.379199713	0.000498847	0.947415012	Potri.010G079400.1	AT1G04220.1
Potra2n10c21747.1	1.382121387	1.274394047	0.691992133	NA		
Potra2n10c21748.1	2.121735209	0.794992988	9.63178E-16	0.136172083	Potri.010G079200.3	AT1G20780.1
Potra2n10c21749.1	-0.205813085	0.024475253	0.059561302	0.976324121	Potri.010G079100.1	AT4G14615.1
Potra2n10c21750.1	-0.736205128	-0.114354448	0.005310361	0.937687311	Potri.010G079000.1	AT4G14580.1
Potra2n10c21751.1	0.253038968	-2.42671106	0.961890434	NA	Potri.017G078000.2	AT5G62740.1
Potra2n10c21752.1	-0.45082422	-0.227270415	0.012541113	0.890446513	Potri.010G078900.2	AT3G23020.1
Potra2n10c21753.1	-2.763363497	-1.185017808	2.02599E-09	0.679365931	Potri.010G078800.1	AT5G43750.1
Potra2n10c21754.1	2.38226145	0.472386236	2.12237E-10	0.802942736	Potri.010G078700.1	AT2G23770.1
Potra2n10c21755.1	1.986179645	0.212718027	7.17636E-07	0.941855873		
Potra2n10c21756.1	0.107465616	0.145084159	0.807279934	0.958719661	Potri.010G078600.2	AT5G43745.1
Potra2n10c21757.1	-0.2649789	-0.071428929	0.12302791	0.94076214	Potri.010G078500.1	AT1G04230.1
Potra2n10c21758.1	-0.435668254	-0.356226674	0.302957099	0.832630441	Potri.010G078400.3	AT5G43700.1
Potra2n10c21759.5	-1.407110223	0.571550479	0.019048581	0.875292974	Potri.010G078300.2	AT4G14550.1
Potra2n10c21760.1	-0.488496134	-0.015010953	0.001661441	0.989304496	Potri.010G078200.1	AT5G43670.1
Potra2n10c21761.2	-0.057747334	0.115452154	0.848476364	0.936549944	Potri.010G077900.2	AT5G64560.1
Potra2n10c21762.1	-0.031471477	0.258507944	0.936143804	0.874808359		
Potra2n10c21763.1	0.916802616	0.349606578	8.86308E-09	0.695163357	Potri.010G077800.1	AT2G30580.1
Potra2n10c21764.3	0.78132276	2.555018106	0.135909976	0.214363327		
Potra2n10c21765.1	-0.302559531	2.481395538	0.748132376	0.34109377	Potri.010G077600.1	
Potra2n10c21766.1	0.824622389	2.807860765	0.467081008	0.241968662	Potri.010G077500.1	
Potra2n10c21767.1	0.649150012	2.764950722	0.477657315	0.295873565		
Potra2n10c21768.1	0.423117288	1.316415022	0.012218202	0.208981586	Potri.010G077400.1	AT2G37230.1
Potra2n10c21769.1	-0.166894556	-0.018491413	0.16458358	0.98409016	Potri.010G077200.1	AT4G14660.1
Potra2n10c21770.3	-0.122059435	0.076471145	0.5186707	0.957668459	Potri.010G077100.1	AT3G23070.1
Potra2n10c21771.1	5.489273524	2.889205991	0.002221153	NA	Potri.010G077000.1	AT5G43650.1
Potra2n10c21772.1	0.368814675	0.015982682	0.114890283	0.995261215	Potri.010G076900.1	AT5G09510.1
Potra2n10c21773.1	-0.752396189	-0.410336239	0.016492125	0.648884421		
Potra2n10c21774.1	-0.627308138	-0.005760481	2.91487E-07	0.996032145	Potri.010G076700.6	AT5G43630.1

Potra2n10c21775.2	0.179257864	0.087003679	0.087889292	0.91917102	Potri.010G076600.10	AT1G17350.1
Potra2n10c21776.1	-0.389476574	-0.070289411	0.00201562	0.953831244	Potri.010G076500.1	AT4G14500.1
Potra2n10c21777.1	-2.172152075	-0.946134333	0.065724886	0.786730624	Potri.010G076400.1	
Potra2n10c21778.1	-0.626990508	0.127871919	0.021672565	0.956865545	Potri.010G076200.2	AT3G23090.1
Potra2n10c21779.1	-0.448896538	-0.004347544	0.000427869	0.996032145	Potri.010G076100.1	AT5G43600.1
Potra2n10c21780.1	0.342445176	0.130755535	0.014428637	0.871720501	Potri.010G075900.1	AT3G23100.2
Potra2n10c21781.1	5.380255993	NA	0.003931129	NA	Potri.010G075800.1	AT2G38870.1
Potra2n10c21782.1	0.102236182	-0.767708831	NA	NA		
Potra2n10c21783.1	1.037485876	-2.660845019	0.654985336	NA	Potri.010G075600.1	AT5G43580.1
Potra2n10c21784.1	3.78298983	0.110081123	0.022126371	NA	Potri.010G075300.1	AT5G43580.1
Potra2n10c21785.1	3.32669507	0.110081123	0.126562291	NA	Potri.010G075600.1	AT5G43580.1
Potra2n10c21786.1	5.345736475	2.283425176	0.001833467	NA	Potri.010G075200.1	AT5G43580.1
Potra2n10c21787.1	1.578268123	NA	NA	NA	Potri.010G075133.1	
Potra2n10c21788.1	4.329999253	1.363811008	0.024802303	NA	Potri.010G075133.1	
Potra2n10c21789.1	4.05936134	NA	0.061435465	NA	Potri.010G075100.1	AT1G06137.1
Potra2n10c21790.1	0.216672824	0.173521313	0.05407591	0.773063484	Potri.010G075000.1	AT1G04300.1
Potra2n10c21791.1	0.702740883	-0.33755818	0.194766639	0.925035363	Potri.010G074900.1	AT3G02400.1
Potra2n10c21792.1	-0.071933507	0.139140834	0.880582501	0.95671968	Potri.010G074800.7	AT4G24000.1
Potra2n10c21793.1	2.071290888	1.056844989	3.64803E-10	0.30671111	Potri.010G074700.1	AT4G24010.1
Potra2n10c21794.1	-1.317292482	-1.465929376	NA	NA	Potri.010G074500.1	AT3G23130.1
Potra2n10c21795.1					Potri.010G074400.1	AT5G43540.1
Potra2n10c21796.1	1.559094468	-0.174284056	0.009911438	0.953831244	Potri.010G074300.1	AT3G23150.1
Potra2n10c21797.1	-1.248900143	0.493108206	0.004559649	0.731112481	Potri.010G074201.1	AT4G14465.1
Potra2n10c21798.1	-0.002613396	0.071721134	0.99238112	0.954419252	Potri.010G074100.1	AT4G14455.1
Potra2n10c21799.1	0.791620245	-0.254827873	0.38865524	0.973347974	Potri.010G074000.2	AT3G23160.1
Potra2n10c21800.1	-0.060969338	-0.240807428	0.915664997	0.912044974	Potri.010G073900.1	AT4G14450.1
Potra2n10c21801.1	-0.607612345	-1.478536539	NA	NA		
Potra2n10c21802.1	0.251986173	0.08640066	0.180983484	0.946146503	Potri.003G083400.1	AT5G43530.1
Potra2n10c21803.1	0.190038748	0.058323411	0.180530631	0.953831244	Potri.010G073700.1	AT4G14440.1
Potra2n10c21804.1	0.122791515	0.101932081	0.342846164	0.896138506	Potri.010G073600.2	AT5G43500.2
Potra2n10c21805.1	-0.117253447	-0.046929929	0.571024878	0.979822671	Potri.010G073500.1	AT1G04340.1
Potra2n10c21806.1	0.582660211	0.292599262	1.45097E-05	0.344206454	Potri.010G073400.1	AT4G14420.1

Potra2n10c21807.1	0.378063083	0.608193053	0.218001832	0.688499606	Potri.010G073300.2	AT1G06620.1
Potra2n10c21808.2	0.482004414	-0.724918121	0.876713723	NA	Potri.010G073166.1	AT1G06620.1
Potra2n10c21809.1	-0.639196163	-0.082061661	1.12434E-05	0.918722297	Potri.010G073100.1	AT1G06650.2
Potra2n10c21810.1	0.422830516	0.262845801	0.034447544	0.72101822	Potri.010G073000.1	AT3G23200.1
Potra2n10c21811.1	-0.100096918	-0.014003954	0.490701159	0.988132773	Potri.010G072900.1	AT4G14410.2
Potra2n10c21812.1	0.216107851	-0.27349411	0.428127029	0.910878765	Potri.010G072700.1	AT5G43420.1
Potra2n10c21813.1	2.970198548	-1.478536539	0.341987238	NA	Potri.008G166000.1	AT3G23230.1
Potra2n10c21814.1	3.461817051	-0.068349583	0.220441126	NA	Potri.010G072400.1	AT3G23230.1
Potra2n10c21815.1	2.524458381	0.222368831	0.270064798	NA	Potri.010G072300.1	AT3G23240.1
Potra2n10c21816.1	-0.62473114	0.36871475	8.19357E-05	0.647022274	Potri.008G167800.1	AT4G14350.1
Potra2n10c21817.1	0.081630167	0.094841468	0.509802994	0.926030989	Potri.010G070500.1	AT3G23325.1
Potra2n10c21818.1	-0.090854559	0.092160099	0.534515465	0.847981555	Potri.010G070400.1	AT4G14340.1
Potra2n10c21819.1	-0.047791668	-0.513158929	0.942302763	0.835627384	Potri.008G168200.1	
Potra2n10c21820.1	-0.256686107	0.074390069	0.027201665	0.969421568	Potri.010G070200.1	AT5G43310.2
Potra2n10c21821.1	-0.071731868	-0.008425957	0.721951153	0.996154416	Potri.010G070100.1	AT3G23360.1
Potra2n10c21822.1	-0.122543185	-0.128386584	0.82558901	0.974275337	Potri.010G070001.1	
Potra2n10c21823.1	0.797939044	0.180304792	2.03592E-07	0.8689387	Potri.010G069900.1	AT4G27090.1
Potra2n10c21824.1	0.195399354	-0.250580526	0.665443077	0.910815461	Potri.010G069800.1	AT4G21410.1
Potra2n10c21825.1	-3.788112614	-0.527593386	0.207395392	NA		
Potra2n10c21826.1	3.29455747	1.737092527	2.20585E-12	0.197749543	Potri.010G069900.1	AT4G27090.1
Potra2n10c21827.2	0.335838847	-0.36487003	0.139689798	0.771626158	Potri.010G069800.1	AT4G21410.1
Potra2n10c21828.1	-3.723145049	-1.133916949	1.36553E-08	0.606364174	Potri.010G069700.1	AT3G25400.1
Potra2n10c21829.1	-0.3012621	-0.013602756	0.009105271	0.9896961	Potri.010G069600.1	AT2G33470.2
Potra2n10c21830.1	-2.159303427	-0.276588035	0.034135334	0.973347974	Potri.010G069500.1	AT2G33460.1
Potra2n10c21831.1	-0.017813032	-0.226278209	0.978525619	0.937276488	Potri.010G069400.1	AT4G14330.1
Potra2n10c21832.1	1.64774763	NA	NA	NA	Potri.010G069300.1	AT3G23400.1
Potra2n10c21833.1	0.235149246	-0.178711319	0.47591005	0.935099709	Potri.010G069200.1	AT4G14310.1
Potra2n10c21834.1	-1.644688365	-2.514137983	NA	NA	Potri.008G146100.1	AT1G70230.1
Potra2n10c21835.1	-0.377918175	-0.620082655	0.171716338	0.482012772	Potri.010G069100.1	AT3G23410.1
Potra2n10c21836.2	-1.41654322	-0.173554834	2.88131E-11	0.902847514	Potri.010G069000.2	AT3G23430.1
Potra2n10c21837.1	-1.48156093	-0.28723472	1.02061E-13	0.77384886		
Potra2n10c21838.1	2.158111082	0.517118644	2.52428E-07	0.842247476	Potri.010G068900.1	AT4G14305.1

Potra2n10c21839.1	1.375266961	0.264978484	0.021052062	0.927125651	Potri.010G068800.1	AT3G23440.1
Potra2n10c21840.1	-0.670252827	-0.131606634	3.07478E-06	0.924251575	Potri.010G068700.1	AT4G28600.1
Potra2n10c21841.3	0.020489982	-3.485182377	0.994891611	NA	Potri.010G068600.1	AT3G07300.1
Potra2n10c21842.1	0.10014905	0.152967395	0.46524769	0.874808359	Potri.010G068400.10	AT2G33435.1
Potra2n10c21843.3	0.153361351	0.16546513	0.388005876	0.875861859	Potri.010G068300.1	AT1G32580.1
Potra2n10c21844.3	2.141698517	3.042098221	0.44461568	NA	Potri.010G068100.1	AT5G46050.1
Potra2n10c21845.1	3.228234647	3.77706165	0.117962743	NA	Potri.010G068000.1	AT2G33420.1
Potra2n10c21846.2	0.745551357	0.458919168	6.46779E-14	0.044568289	Potri.010G067900.1	AT3G23530.1
Potra2n10c21847.1	-0.61910233	-0.046370319	NA	NA	Potri.010G067500.1	AT2G33410.1
Potra2n10c21848.1	1.720979443	1.869164924	0.334858908	0.842247476	Potri.010G067700.2	AT3G23530.1
Potra2n10c21849.1	-1.35927387	-1.507841271	NA	NA	Potri.001G039600.1	
Potra2n10c21850.1	4.293535295	4.118402673	0.00600508	NA	Potri.010G067700.2	AT3G23530.1
Potra2n10c21851.1	NA	1.394613675	NA	NA		
Potra2n10c21852.1	4.67191876	1.350324572	6.32122E-06	NA	Potri.010G067700.2	AT3G23530.1
Potra2n10c21853.1	-0.659813289	1.278820126	0.693671947	0.817452127	Potri.010G067700.2	AT3G23530.1
Potra2n10c21854.1	-0.269059814	0.003789383	0.034382435	0.996246891	Potri.010G067500.1	AT2G33410.1
Potra2n10c21855.1	-0.501155117	-0.896794417	0.215528019	0.370792873	Potri.010G067300.2	AT2G33400.1
Potra2n10c21856.1	-0.1197883	-0.341736273	0.507026939	0.526401612	Potri.010G067200.1	AT2G33390.1
Potra2n10c21857.1	-0.926202338	-0.107370335	1.81694E-11	0.954419252	Potri.010G067100.1	AT2G33385.2
Potra2n10c21858.1	-0.921446011	-0.106563293	2.23682E-11	0.954419252	Potri.010G067000.1	AT5G13890.2
Potra2n10c21859.1	-0.507988613	0.04533763	0.001328705	0.98177802	Potri.010G066900.1	AT2G05620.1
Potra2n10c21860.1	-0.259072546	0.015999054	0.018009764	0.990896655	Potri.010G066700.2	AT5G35620.1
Potra2n10c21861.1	0.491395469	0.084862304	0.000360041	0.943819961	Potri.010G066600.1	AT4G26740.1
Potra2n10c21862.1	-0.924170717	-0.613225085	0.273651934	0.88643982	Potri.010G066500.1	AT1G04040.1
Potra2n10c21863.5	0.167059421	0.13212468	0.177655444	0.767368598	Potri.010G066200.1	AT2G33360.1
Potra2n10c21864.1	0.165695161	1.410001728	0.95834966	NA	Potri.010G066100.2	AT1G04500.1
Potra2n10c21865.1	0.117986335	-0.034593817	0.37584636	0.972159498	Potri.010G066000.1	AT2G33340.2
Potra2n10c21866.1	-0.527830856	-0.228170839	0.006053566	0.812696706	Potri.010G065900.1	AT2G05590.2
Potra2n10c21867.2	0.266996568	0.106478514	0.332578614	0.946464685	Potri.010G065800.1	AT1G04520.1
Potra2n10c21868.2	-0.339397604	-0.078393517	0.103915626	0.943819961		
Potra2n10c21869.1	-0.008639544	-0.169897557	0.981113169	0.922011871	Potri.010G065600.2	AT1G60000.1
Potra2n10c21870.1	0.01949658	-0.026847028	0.909203175	0.986636967	Potri.010G065500.2	AT5G43960.1

Potra2n10c21871.1	0.43851745	0.211994856	0.002002356	0.643173103	Potri.010G065400.1	AT1G04530.1
Potra2n10c21872.1	0.216565131	1.054749676	NA	NA	Potri.010G065300.1	AT1G04540.1
Potra2n10c21873.1	-0.992351792	-0.227009867	5.58308E-07	0.820966257	Potri.008G172400.1	AT2G33310.3
Potra2n10c21874.1	0.749707854	0.134025345	6.28749E-07	0.920028955	Potri.010G065100.1	AT4G13430.1
Potra2n10c21875.1	0.016266972	0.037375608	0.948233428	0.985393296	Potri.010G065000.1	AT3G24320.1
Potra2n10c21876.2	0.132614067	0.087105808	0.240471973	0.931811835	Potri.010G064900.1	AT3G24315.1
Potra2n10c21877.1	-0.04793888	-0.112645056	0.823625679	0.930598202	Potri.010G064800.1	AT4G13450.1
Potra2n10c21878.1	1.440775088	0.206894361	2.39861E-08	0.900452689	Potri.010G064600.1	AT1G10580.1
Potra2n10c21879.1	0.138236032	0.288375804	0.657755991	0.863186953		
Potra2n10c21880.1	0.324184871	0.283251347	0.028381283	0.736000077	Potri.010G064500.1	AT1G04555.1
Potra2n10c21881.1	-0.109835699	-0.04453641	0.365513312	0.962661188	Potri.010G064300.1	AT4G13460.1
Potra2n10c21882.1	1.79170333	2.647494723	0.1748049	0.305728522	Potri.010G064200.1	AT1G04560.1
Potra2n10c21883.1	0.438861698	0.264947319	0.007933686	0.804641567	Potri.010G064100.1	AT5G05210.1
Potra2n10c21884.1	1.889506358	3.168871091	0.492780682	0.659942944	Potri.010G064000.1	AT4G13480.1
Potra2n10c21885.2	0.316540497	-0.053965048	0.468013221	0.989304496	Potri.010G063900.1	AT3G12770.1
Potra2n10c21886.2	-3.052147357	-0.695734981	1.36377E-05	0.740524465	Potri.010G063800.1	AT1G04570.1
Potra2n10c21887.1	-0.433237652	-0.162309649	0.000245306	0.874808359	Potri.010G063700.1	AT2G33255.1
Potra2n10c21888.1	0.470105897	0.24432829	0.000120461	0.776296899	Potri.010G063600.2	AT5G60990.1
Potra2n10c21889.1	0.580726908	-0.014265753	0.000974866	0.992405049	Potri.010G063500.1	AT4G13510.1
Potra2n10c21890.3	4.324061796	0.065975336	0.000133205	NA		
Potra2n10c21891.1	-0.781391287	-0.127460884	0.000101402	0.932447954	Potri.010G063300.2	AT3G04310.1
Potra2n10c21892.1	0.320169296	0.3853235	0.022180215	0.802631112	Potri.010G062900.1	AT3G23920.1
Potra2n10c21893.1	0.82357019	NA	NA	NA	Potri.010G062800.1	AT4G13560.1
Potra2n10c21894.2	-0.397716431	0.249517785	0.027959475	0.784407529	Potri.010G062700.4	AT4G13550.1
Potra2n10c21895.1	-2.166659195	-0.133606443	3.89321E-22	0.95671968	Potri.010G062600.2	AT4G13540.1
Potra2n10c21896.1	NA	NA	NA	NA		
Potra2n10c21897.1	-0.544394981	0.457078629	0.686840131	0.924951347	Potri.008G174500.1	AT5G43900.3
Potra2n10c21898.1	1.000916591	0.397733467	0.004485228	0.822292402	Potri.010G062400.2	AT1G04610.1
Potra2n10c21899.1	-0.564318836	0.045942106	0.000693875	0.974275337	Potri.010G062300.1	AT1G04620.1
Potra2n10c21900.4	0.187905173	0.109968045	0.217411538	0.896937427	Potri.010G062000.1	AT3G23980.1
Potra2n10c21901.1	2.708034945	0.668843218	2.64951E-45	0.773063484	Potri.010G061900.1	AT2G29420.1
Potra2n10c21902.1	6.772340781	3.362460899	6.9555E-92	0.000724087	Potri.010G061700.1	AT2G29420.1

Potra2n10c21903.1	6.697271781	2.878379175	1.00569E-30	0.176054801	Potri.010G061600.2	AT2G29420.1
Potra2n10c21904.1					Potri.010G061400.1	AT2G29420.1
Potra2n10c21905.1	6.425101232	3.569924494	2.10534E-22	0.103422934	Potri.010G061700.1	AT2G29420.1
Potra2n10c21906.1	0.245680011	0.410510612	0.128751559	0.59929692	Potri.010G060700.2	AT1G67400.1
Potra2n10c21907.1	0.256270773	0.406541482	0.117943744	0.616163505	Potri.010G060800.1	AT4G24210.1
Potra2n10c21908.1	0.059177416	1.459584467	NA	NA	Potri.010G060700.2	AT1G67400.1
Potra2n10c21909.1	0.609933617	0.233361997	3.86973E-13	0.558624809	Potri.010G060500.1	AT1G67420.1
Potra2n10c21910.1	0.948970086	0.482180701	4.63595E-06	0.652685073	Potri.010G060400.1	AT1G67430.1
Potra2n10c21911.1	1.984741028	-2.870418371	NA	0.369370786	Potri.010G059700.1	AT1G60420.1
Potra2n10c21912.1	2.046489159	0.373056739	1.25769E-31	0.719713755	Potri.010G060200.1	AT1G60420.1
Potra2n10c21913.1	-0.134316105	-0.070574426	0.295976825	0.940376196	Potri.010G059300.1	AT4G40042.1
Potra2n10c21914.1	0.005234888	0.151582658	0.992286753	0.960981446	Potri.010G059200.1	AT1G67480.2
Potra2n10c21915.1	0.104172705	-0.075366056	0.501723977	0.943438281	Potri.010G059100.1	AT1G24310.1
Potra2n10c21916.1	0.648273592	0.103424772	8.29598E-07	0.884553748	Potri.010G059000.1	AT1G67490.1
Potra2n10c21917.1	2.563896372	NA	NA	NA	Potri.010G058900.1	AT2G01950.1
Potra2n10c21918.1					Potri.010G058900.1	AT2G01950.1
Potra2n10c21919.1	-0.96292283	-0.048222295	0.000113327	0.978294428	Potri.010G058700.1	AT3G01860.1
Potra2n10c21920.1	-0.602968754	-0.108482153	0.000205029	0.896937427	Potri.008G176801.1	AT5G20165.1
Potra2n10c21921.1	-0.584792202	0.26544261	0.211088658	0.909715408	Potri.008G176700.2	
Potra2n10c21922.1	-0.618029056	-0.274498968	0.032090482	0.885375133	Potri.010G058200.1	AT1G67510.1
Potra2n10c21923.2	0.063743886	0.018730554	0.60153742	0.986636967	Potri.010G058100.3	AT3G26340.1
Potra2n10c21924.1	-0.093742978	0.221552047	0.68310118	0.645919795	Potri.010G058000.3	AT1G24330.1
Potra2n10c21925.1	4.629288698	3.311481645	0.076756835	NA	Potri.008G177200.1	AT3G50940.1
Potra2n10c21926.1	-0.847620578	-0.121915164	0.319947266	0.984026889	Potri.010G057800.1	AT1G67540.2
Potra2n10c21927.1	-0.010821534	0.093690269	0.964797847	0.935751704	Potri.008G177500.1	AT5G43230.1
Potra2n10c21928.2	-0.301441241	-0.161453361	0.188159804	0.937846405	Potri.010G057600.1	AT1G76340.1
Potra2n10c21929.1	-0.002824394	-0.060592714	0.987039542	0.956689222	Potri.010G057400.4	AT5G08530.1
Potra2n10c21930.1	-2.824446863	-1.503364867	0.065096525	0.812118278	Potri.010G057300.1	AT5G14230.1
Potra2n10c21931.1	-0.084523297	0.436547957	0.742786545	0.691680177	Potri.010G057200.1	AT1G27900.1
Potra2n10c21932.1	-0.692241075	-0.224439966	5.87082E-07	0.847978165	Potri.010G057100.1	AT1G67560.1
Potra2n10c21933.1	-0.904127301	-0.361929802	0.000148471	0.725595998		
Potra2n10c21934.1	-0.521300346	-0.228369467	3.67479E-05	0.821709751	Potri.010G057000.1	AT5G63380.1

Potra2n10c21935.1	-0.168183111	0.146372837	0.131551167	0.880553952	Potri.010G056900.4	AT1G67580.1
Potra2n10c21936.2	-0.773881388	-1.335128501	0.382588445	0.686256504	Potri.010G056800.1	AT1G67590.1
Potra2n10c21937.1	NA	NA	NA	NA	Potri.010G056700.1	AT1G24340.1
Potra2n10c21938.1	0.712093927	0.099254226	3.06175E-05	0.941855873	Potri.010G056600.1	AT1G67600.1
Potra2n10c21939.1	0.8741417	-0.429152203	0.144265854	0.943483421	Potri.010G056500.1	AT1G27880.1
Potra2n10c21940.1	3.073325943	NA	NA	NA		
Potra2n10c21941.1					Potri.019G027600.1	AT1G21280.1
Potra2n10c21942.1	2.547568189	1.235314309	5.4148E-39	0.033646795	Potri.010G056300.1	AT3G26040.1
Potra2n10c21943.1	-0.315065656	0.308793754	0.150790862	0.821709751	Potri.010G056200.1	AT1G27850.1
Potra2n10c21944.1	-0.007839386	0.067462887	0.974739413	0.957117835	Potri.010G056100.1	AT1G24360.1
Potra2n10c21945.1	0.545826603	-0.009291145	0.007853157	0.996154416	Potri.010G055900.1	AT1G67630.1
Potra2n10c21946.1	NA	NA	NA	NA	Potri.010G055800.1	AT5G40780.1
Potra2n10c21947.1	-1.238448028	-0.460917741	5.64953E-09	0.691005224	Potri.010G055700.1	AT3G01750.1
Potra2n10c21948.2	-0.040077981	0.125185271	0.797686424	0.906107926	Potri.010G055500.1	AT3G04130.1
Potra2n10c21949.1	2.348313144	0.141753741	7.07302E-16	0.961680875	Potri.010G055400.1	AT3G04120.1
Potra2n10c21950.1	3.900281801	1.16128855	8.02913E-07	0.554040943	Potri.010G055300.2	AT3G48140.1
Potra2n10c21951.5	6.012041785	2.685308539	9.43259E-14	NA	Potri.010G055200.1	AT3G13080.1
Potra2n10c21952.1	-0.294242919	-0.073100868	0.159566562	0.95671968	Potri.010G055100.1	AT3G25980.1
Potra2n10c21953.1	0.012283178	0.277173341	0.932027938	0.770975905	Potri.010G055000.1	AT1G13450.1
Potra2n10c21954.1	0.268604312	0.164479551	0.270169745	0.937846405	Potri.010G054800.1	AT1G67660.1
Potra2n10c21955.1	-0.457382435	0.016438918	7.42357E-05	0.990146174	Potri.010G054600.1	AT3G26000.1
Potra2n10c21956.1	0.275327304	-0.024472186	0.133954174	0.9896961		
Potra2n10c21957.1	0.291085482	0.002247604	0.014561972	0.9989808	Potri.010G054400.1	AT3G26020.1
Potra2n10c21958.1	-0.137337338	0.021630772	0.227441311	0.978646026	Potri.010G054300.10	AT3G26020.2
Potra2n10c21959.1	1.824432563	-0.629202521	2.01042E-17	0.60759374	Potri.010G054100.1	AT3G26040.1
Potra2n10c21960.1	0.82357019	NA	NA	NA	Potri.T112000.1	AT1G24430.1
Potra2n10c21961.1	NA	NA	NA	NA	Potri.T111900.1	AT1G24430.1
Potra2n10c21962.1	-0.688652026	-1.55593228	NA	NA	Potri.T111900.1	AT1G24430.1
Potra2n10c21963.4	-0.175159087	-0.208922927	0.311647974	0.797111689	Potri.010G053700.1	AT3G27320.2
Potra2n10c21964.1	-0.071048875	0.086788087	0.85216727	0.967789176	Potri.010G053600.3	AT1G67700.2
Potra2n10c21965.1	0.105142395	0.035532492	0.835976357	0.97950444	Potri.010G053500.1	AT2G02710.2
Potra2n10c21966.1	-0.896585459	0.080433592	0.022419819	0.976302215	Potri.010G053400.1	AT4G27670.1

Potra2n10c21967.1	0.592170256	-0.317166604	0.032514313	0.886633917	Potri.010G053200.1	AT1G70950.1
Potra2n10c21968.1	-0.549848665	0.010556225	0.029570377	0.996032145	Potri.010G053100.1	AT1G67710.1
Potra2n10c21969.1	-0.688675779	0.023879852	0.002233039	0.992934677	Potri.010G053000.2	AT1G13195.1
Potra2n10c21970.1	-0.244074756	-0.295302903	0.668908966	0.900002201	Potri.010G052800.1	AT1G67720.1
Potra2n10c21971.1	0.008727468	0.263603631	0.965020464	0.873955419	Potri.010G052700.1	AT1G24440.1
Potra2n10c21972.2	0.037532079	0.264755307	0.851983278	0.619575855	Potri.010G052600.1	AT1G24460.1
Potra2n10c21973.1	-1.52523637	0.651788196	0.0518369	0.870044019	Potri.010G052500.1	AT1G24470.1
Potra2n10c21974.1	0.29401528	-0.291282458	0.373059455	0.859813626	Potri.010G052400.1	AT1G67730.1
Potra2n10c21975.1	0.201584234	-0.007384273	0.096588806	0.996032145	Potri.010G052300.1	AT1G67730.1
Potra2n10c21976.1	0.877842683	0.66221249	1.41668E-07	0.343290193	Potri.010G052200.1	AT4G00930.1
Potra2n10c21977.1	-0.892726636	0.665535895	0.012709514	0.674283226	Potri.010G052100.1	AT1G24480.1
Potra2n10c21978.1	-0.574085055	-0.168497303	0.017676485	0.900869139	Potri.010G052000.1	AT1G67740.1
Potra2n10c21979.1	-0.127805885	0.188181074	0.307457754	0.791848264	Potri.010G051900.2	AT1G24490.1
Potra2n10c21980.1	-5.863213651	-1.809144185	6.47837E-15	0.32906562	Potri.010G051800.1	AT1G67750.1
Potra2n10c21981.4	-0.024038635	-0.117829234	0.884055982	0.912083068	Potri.010G051700.3	AT1G24510.1
Potra2n10c21982.1	-0.159010325	0.128895086	0.214837997	0.886265374	Potri.010G051600.1	AT1G13190.1
Potra2n10c21983.1	-0.165877947	-0.057848184	0.278680114	0.973347974	Potri.010G051500.1	AT1G13180.1
Potra2n10c21984.1	-0.210432816	-0.216063064	0.036145601	0.829455551	Potri.010G051400.1	AT1G13170.1
Potra2n10c21985.1	0.58444778	0.302450213	0.364444799	0.938054545	Potri.010G051332.1	AT1G13160.1
Potra2n10c21986.1	0.02782333	0.031312229	0.914271709	0.984292161	Potri.010G051200.1	AT3G26115.1
Potra2n10c21987.1	1.826718837	-0.767708831	NA	NA	Potri.010G051100.1	AT3G26120.1
Potra2n10c21988.1	-0.595952689	0.151993677	NA	NA	Potri.T124100.1	AT5G50260.1
Potra2n10c21989.1	0.82357019	NA	NA	NA		
Potra2n10c21990.1	0.806502508	-0.343476626	0.012628728	0.857189843	Potri.010G050900.1	AT1G24530.1
Potra2n10c21991.1	NA	2.283425176	NA	NA		
Potra2n10c21992.1	2.881180365	0.482007216	0.001901389	0.950878725	Potri.010G050200.1	AT3G01680.1
Potra2n10c21993.1	0.82357019	NA	NA	NA	Potri.010G050200.1	AT3G01680.1
Potra2n10c21994.1	-0.271660727	-2.526435732	0.916093503	NA	Potri.010G050200.1	AT3G01680.1
Potra2n10c21995.1					Potri.010G050100.1	AT1G24540.1
Potra2n10c21996.1	2.427182574	0.60574299	1.09492E-05	0.865040003		
Potra2n10c21997.1	-0.9215255	-0.258670169	6.9656E-05	0.843683654	Potri.008G183400.1	AT1G13130.1
Potra2n10c21998.1	2.934222348	-0.958221426	0.003450101	NA		

Potra2n10c21999.1	0.965478917	0.299187468	0.000327745	0.863186953	Potri.010G050000.2	AT3G27430.2
Potra2n10c22000.1	1.408105154	0.319164667	1.08701E-10	0.759821197	Potri.010G049600.3	AT5G14420.3
Potra2n10c22001.1	1.498762316	0.34565505	6.37994E-10	0.813731863		
Potra2n10c22002.1					Potri.010G049500.2	AT1G18350.1
Potra2n10c22003.1	0.042592939	0.029114156	0.805945756	0.981726976	Potri.010G049400.1	AT1G24560.1
Potra2n10c22004.1	-2.320402105	-0.921612486	0.118615175	0.902857376	Potri.008G184000.1	AT3G26320.1
Potra2n10c22005.1	NA	0.674963689	NA	NA		
Potra2n10c22006.1	-0.231229701	0.11562944	0.167760272	0.903698951	Potri.010G049100.1	AT4G24740.1
Potra2n10c22007.1	-0.276625134	0.047321423	0.099266696	0.977559521	Potri.010G048700.1	AT1G13030.1
Potra2n10c22008.3	-0.12400271	0.038315877	0.132467644	0.974628351	Potri.010G048400.2	AT3G26380.1
Potra2n10c22009.3	0.431051256	0.247016728	0.000142874	0.579358413	Potri.010G048600.1	AT3G26360.1
Potra2n10c22010.2	0.04974617	0.103935437	0.902529301	0.955628098	Potri.010G047900.2	AT3G26430.1
Potra2n10c22011.1	0.17550015	-0.120693712	0.668181749	0.956889095	Potri.010G047700.1	AT5G48270.1
Potra2n10c22012.1	2.632524198	3.603514974	NA	NA		
Potra2n10c22013.1	-0.105675135	0.066523198	0.444551361	0.922681078	Potri.010G047500.1	AT1G67840.1
Potra2n10c22014.1	NA	NA	NA	NA	Potri.009G067800.1	AT5G12220.1
Potra2n10c22015.1	0.149835342	0.06333183	0.223631693	0.966917036	Potri.010G047200.5	AT1G67850.1
Potra2n10c22016.1	0.102113124	-0.966432843	0.961589514	NA	Potri.010G047000.1	
Potra2n10c22017.1	NA	1.879759176	NA	NA	Potri.010G047100.1	AT1G67856.1
Potra2n10c22018.1	-0.354441342	-0.340728778	0.125507758	0.693793454	Potri.010G046900.1	AT1G12990.1
Potra2n10c22019.1	NA	NA	NA	NA		
Potra2n10c22020.1	-1.44746499	-0.29560528	2.01247E-15	0.610808563	Potri.010G046800.2	AT1G67900.3
Potra2n10c22021.1	-1.829504954	-0.335299692	7.95658E-12	0.511337291	Potri.010G046700.3	AT1G67910.1
Potra2n10c22022.1					Potri.010G046600.1	AT1G24590.1
Potra2n10c22023.1	0.82357019	NA	NA	NA		
Potra2n10c22024.1	2.274258217	1.214363886	1.18366E-18	0.021137209	Potri.010G046500.1	AT1G12970.1
Potra2n10c22025.1	0.360566444	-0.093099148	0.927397595	NA	Potri.010G046400.1	AT3G26510.4
Potra2n10c22026.1	0.065771931	0.0605814	0.664124762	0.953177702	Potri.010G046300.1	AT3G26570.2
Potra2n10c22027.1	-0.01680344	0.099673083	0.921959326	0.91998377	Potri.008G186800.1	AT1G70630.1
Potra2n10c22028.1	-0.029077337	0.129171274	0.9478728	0.962696825	Potri.010G046075.1	
Potra2n10c22029.1	-0.295110065	0.088045174	0.030336473	0.931774514	Potri.010G046000.1	AT1G70620.3
Potra2n10c22030.1	0.055069255	-0.121884255	0.768454229	0.890978957	Potri.010G045900.2	AT1G70610.1

Potra2n10c22031.1	0.618489739	0.013736533	8.52748E-05	0.993630002	Potri.010G045800.1	AT1G70600.1
Potra2n10c22032.1	0.282039197	-0.454768529	0.81168646	NA		
Potra2n10c22033.1	-0.038292313	-0.012857817	0.808274148	0.9869326	Potri.010G045600.1	AT2G42490.1
Potra2n10c22034.1	0.564251372	0.127719361	0.000181098	0.866784674	Potri.010G045500.1	AT3G26600.1
Potra2n10c22035.1	-0.057144615	-0.037359498	0.627165427	0.974275337	Potri.010G045300.1	AT1G70590.1
Potra2n10c22036.1	-2.245157539	-0.09240306	0.000883469	0.985393296	Potri.010G045200.1	AT1G10690.1
Potra2n10c22037.1	0.255343756	0.155014744	0.00085216	0.859892315	Potri.010G045100.1	AT1G70580.2
Potra2n10c22038.1	0.050775276	0.113779417	0.770327629	0.87679119	Potri.010G045000.1	AT1G10700.1
Potra2n10c22039.1	-0.028417125	0.085775754	0.820198008	0.893730488	Potri.010G044900.2	AT1G70570.1
Potra2n10c22040.1	1.976597668	2.490806657	NA	NA	Potri.009G007500.1	
Potra2n10c22041.1	-0.35851219	-0.471732304	0.784869834	0.921099436	Potri.010G044800.1	AT1G10710.1
Potra2n10c22042.1	-0.160797164	-0.056986594	0.38330418	0.965801986	Potri.010G044700.1	AT1G60780.1
Potra2n10c22043.4	0.844823569	0.263489279	1.53539E-06	0.70570456	Potri.008G187700.1	AT3G07040.1
Potra2n10c22044.1	0.618431417	0.147876612	0.011137334	0.932242797	Potri.010G044500.1	AT1G70560.1
Potra2n10c22045.2	1.401662996	0.214532626	3.67479E-05	0.934579595	Potri.010G044400.1	AT1G10740.4
Potra2n10c22046.1	-1.389772782	-0.372466402	7.13741E-09	0.887678043	Potri.010G044300.1	AT1G70550.1
Potra2n10c22047.1	1.545832463	NA	NA	NA		
Potra2n10c22048.1	-0.642407296	-0.124464642	4.5599E-07	0.948577865	Potri.010G044200.1	AT1G17100.1
Potra2n10c22049.1	-0.375835554	0.107126497	0.004388113	0.953608209	Potri.010G044100.1	AT1G10760.1
Potra2n10c22050.1	NA	NA	NA	NA	Potri.008G188300.1	AT3G26610.1
Potra2n10c22051.1	0.350768309	0.422333114	0.130667418	0.565419698	Potri.010G043900.1	AT1G70530.1
Potra2n10c22052.1	0.311336012	-0.356079886	0.27442011	0.801784697	Potri.010G043700.1	AT1G70520.1
Potra2n10c22053.1	1.36725459	1.411932363	0.086112125	0.350242504	Potri.008G188600.1	AT1G23360.1
Potra2n10c22054.1	-0.163933704	0.072956797	0.256583866	0.937687311	Potri.010G043500.1	AT1G23380.1
Potra2n10c22055.1	1.459962178	NA	NA	NA		
Potra2n10c22056.1	-0.380978707	-0.177567811	0.044046831	0.84487051	Potri.010G043300.1	AT1G60790.1
Potra2n10c22057.6	0.18806082	-0.141741446	0.325305982	0.881894261	Potri.010G043200.2	AT1G60800.1
Potra2n10c22058.1	0.260312356	0.306780117	0.304131489	0.917746944	Potri.010G042900.1	AT1G23390.1
Potra2n10c22059.1	0.357244827	0.019157586	0.016108856	0.990654009	Potri.010G042600.1	AT1G10660.3
Potra2n10c22060.1	-0.07924661	-0.179325893	0.746317271	0.900629215	Potri.010G042500.1	AT1G23400.1
Potra2n10c22061.1	NA	NA	NA	NA	Potri.010G042400.1	AT1G23420.1
Potra2n10c22062.1	2.764756783	4.569333034	0.060055203	NA	Potri.010G042300.6	AT1G10657.2

Potra2n10c22063.1	-0.010318138	0.119105998	0.955073576	0.932328711	Potri.010G042200.1	AT1G10650.1
Potra2n10c22064.1	-0.411927528	-0.408712119	0.608462821	0.953831244		
Potra2n10c22065.3	1.396602304	0.194874933	8.64639E-06	0.900514809	Potri.010G042100.1	AT1G60590.1
Potra2n10c22066.1	1.397385701	0.195891695	8.68526E-06	0.898993569		
Potra2n10c22067.1	0.060646115	-0.385393694	0.949423827	0.935099709	Potri.010G042000.1	AT1G60470.1
Potra2n10c22068.1	-0.200206641	-0.374262113	0.719694594	0.892986206	Potri.010G041900.3	AT1G60460.1
Potra2n10c22069.1	0.913781627	0.453043166	5.09075E-07	0.301814135	Potri.010G041800.1	AT4G32180.2
Potra2n10c22070.1	0.366838056	0.131070194	0.059285165	0.869175327	Potri.010G041700.1	AT1G70480.2
Potra2n10c22071.1	0.128174719	-1.081374223	0.970103579	NA	Potri.010G041600.1	AT1G23530.1
Potra2n10c22072.2	0.882003392	-0.182803715	0.55232441	NA	Potri.010G041400.2	AT1G70460.1
Potra2n10c22073.1	-0.061745095	0.259966653	0.714171632	0.848005416	Potri.010G041200.10	AT1G10600.1
Potra2n10c22074.1	0.164971864	0.176349976	0.36641296	0.786910796	Potri.010G041100.1	AT5G14740.1
Potra2n10c22075.2	0.108100437	-0.038395072	0.554655234	0.97191638	Potri.010G041000.2	AT1G23750.1
Potra2n10c22076.1	-0.28164965	-0.312091068	0.509488412	0.874879295	Potri.010G040800.1	AT1G70370.1
Potra2n10c22077.5	-0.60222722	-0.178380153	0.001335214	0.900551258	Potri.010G040700.1	AT1G60230.1
Potra2n10c22078.1	0.143791787	0.32727347	0.331687124	0.43680237	Potri.010G040600.2	AT1G60220.1
Potra2n10c22079.1	-0.345374383	-1.877452442	0.911212154	NA	Potri.010G040500.1	AT1G10380.1
Potra2n10c22080.1	-0.59300587	-0.194292446	0.000368382	0.810224017	Potri.010G040400.1	AT5G62620.1
Potra2n10c22081.1						
Potra2n10c22082.1					Potri.002G125500.1	
Potra2n10c22083.1	-0.115773945	-0.052454873	0.480774917	0.97521505	Potri.010G040100.5	AT1G10410.1
Potra2n10c22084.1	-0.840558593	-0.488473102	0.156462713	0.886633917	Potri.010G040200.1	AT4G20140.1
Potra2n10c22085.1	NA	NA	NA	NA		
Potra2n10c22086.1	-0.272278948	0.280500927	0.141023495	0.824448251	Potri.010G040000.1	AT1G59640.1
Potra2n10c22087.1	-1.39792598	-0.096217773	0.416373658	0.993577196	Potri.008G190900.1	AT1G69935.1
Potra2n10c22088.1	-0.042729552	0.152068497	0.743923142	0.849446235	Potri.010G039900.1	AT1G59820.1
Potra2n10c22089.1	-0.237021589	-0.936350916	0.94084912	NA	Potri.010G039800.1	AT1G69970.2
Potra2n10c22090.1	-0.105505256	-0.130228637	0.380000122	0.786213998	Potri.010G039700.2	AT1G10430.1
Potra2n10c22091.1	-0.00677372	0.120553173	0.969250175	0.909715408	Potri.008G191700.3	AT1G69980.1
Potra2n10c22092.1	-0.738765537	-0.369765895	0.000540359	0.558277497	Potri.008G191800.1	AT1G70000.1
Potra2n10c22093.1	0.376751543	0.244028317	0.132467644	0.817452127	Potri.010G039000.1	AT1G27200.1
Potra2n10c22094.2	-0.719372793	-0.318503326	0.001544005	0.798096728	Potri.010G038900.2	AT1G27210.1

Potra2n10c22095.1	-0.533053395	1.249001423	0.633768927	0.79819686	Potri.010G038800.1	
Potra2n10c22096.1	-0.107435295	0.060063624	0.525033291	0.953177702	Potri.010G038700.8	AT1G70060.1
Potra2n10c22097.1	0.101711719	-0.013475831	0.5779878	0.992934677	Potri.010G038500.1	AT1G70070.1
Potra2n10c22098.1	-0.046710829	-0.302219313	0.931878364	0.865591169	Potri.010G038600.1	AT1G23790.1
Potra2n10c22099.1	-0.135761552	-0.296028648	0.52749107	0.845627381	Potri.010G038400.2	AT1G59900.1
Potra2n10c22100.1	-1.003904941	-0.212777869	0.014827831	0.942409925	Potri.010G038300.1	AT1G70090.1
Potra2n10c22101.1	-0.986364718	-0.365857464	7.15668E-05	0.724604666	Potri.010G038200.2	AT1G10460.1
Potra2n10c22102.1	0.192109632	0.246513232	0.288323703	0.794641467	Potri.010G038100.1	AT1G70100.3
Potra2n10c22103.1	-0.537321018	-0.011179901	0.141051452	0.996032145	Potri.010G037900.1	AT1G70140.1
Potra2n10c22104.1	-1.632688493	-0.740230743	2.37023E-10	0.066359789	Potri.010G037800.2	AT1G59940.1
Potra2n10c22105.2	0.032964912	0.216212203	0.89250214	0.814001428	Potri.010G037600.3	AT1G70150.1
Potra2n10c22106.1	-0.123345426	-0.018401216	0.442872226	0.99226031	Potri.010G037500.2	AT3G17210.1
Potra2n10c22107.1	-0.10880325	-0.029626079	0.45865276	0.985393296	Potri.010G037400.1	
Potra2n10c22108.1	0.402228562	0.068159961	2.77245E-06	0.947290893	Potri.010G036900.1	AT1G70160.1
Potra2n10c22109.1	0.5126321	0.189924855	0.002903085	0.813731863	Potri.008G193100.1	AT1G59960.1
Potra2n10c22110.1	0.526320177	0.155012067	1.21567E-05	0.849446235	Potri.010G036700.1	AT1G51980.1
Potra2n10c22111.1	0.331579016	0.14427886	0.004070294	0.816392132	Potri.010G036500.1	AT1G70180.2
Potra2n10c22112.1	2.95088259	1.882832705	3.402E-09	0.371280386	Potri.010G036400.1	AT1G10480.1
Potra2n10c22113.1	-0.233097629	-0.083142282	0.026255457	0.950182516	Potri.010G036200.2	AT1G24120.1
Potra2n10c22114.1	0.219402855	-0.036659101	0.663371766	0.991909603	Potri.010G036100.1	AT1G24110.1
Potra2n10c22115.1	0.710706506	-1.046444899	0.820198008	NA	Potri.010G035900.1	AT1G51760.1
Potra2n10c22116.1	0.159033429	-0.19758933	0.699368447	0.924828248	Potri.010G035700.1	AT1G05670.2
Potra2n10c22117.1	0.961337619	0.02637406	0.028156293	0.996032145	Potri.010G035700.1	AT1G05670.2
Potra2n10c22118.1	2.268464071	0.777106677	1.08179E-07	0.82876584	Potri.010G032800.1	AT1G10370.1
Potra2n10c22119.1	-0.052662491	-0.03393763	0.649469817	0.966501596	Potri.010G035400.1	AT1G27150.1
Potra2n10c22120.1	0.713727346	0.294769593	0.001818551	0.627991232	Potri.010G035300.1	AT1G69910.1
Potra2n10c22121.1	NA	NA	NA	NA		
Potra2n10c22122.1	0.609132496	0.220307508	3.02643E-07	0.699390593	Potri.010G035100.1	AT1G27100.1
Potra2n10c22123.1	1.060749661	0.624051719	0.001596239	0.474295608		
Potra2n10c22124.1	-0.539039607	-0.192648593	0.000298998	0.794820393	Potri.010G035000.1	AT1G10350.1
Potra2n10c22125.1	0.063044538	-0.001315764	0.457065516	0.999325699	Potri.010G034900.1	AT1G27090.1
Potra2n10c22126.1	0.414665416	0.147211437	0.001510866	0.847341104	Potri.010G034800.1	AT2G38280.2

Potra2n10c22127.1	0.431884148	0.799297805	0.421530105	0.817452127	Potri.010G034700.1	AT1G69870.1
Potra2n10c22128.1	-0.339046396	0.045651904	0.042203989	0.971828097	Potri.010G034600.1	AT1G27070.1
Potra2n10c22129.1	-0.58598469	-0.160608448	0.000258097	0.855245045	Potri.010G034500.1	AT1G27050.1
Potra2n10c22130.1	0.864911819	0.429614985	0.035312692	0.814981587	Potri.010G034400.1	AT2G02590.1
Potra2n10c22131.1	3.33480486	2.110159696	6.33123E-13	0.007318585	Potri.010G034300.1	AT1G69850.1
Potra2n10c22132.1	2.58657067	1.462863326	0.421913856	NA	Potri.010G034200.1	AT1G14430.1
Potra2n10c22133.2	-0.056178994	-0.030723222	0.723815711	0.978646026	Potri.010G033900.2	AT3G17900.1
Potra2n10c22134.1	-0.055936586	0.00756906	0.564884406	0.995203254	Potri.010G033500.1	AT3G01280.1
Potra2n10c22135.1	-0.419499976	-0.118141371	0.023294644	0.951726178	Potri.010G033400.1	AT3G06035.1
Potra2n10c22136.1	0.384651595	1.310957488	0.840674256	0.826906752	Potri.008G195100.1	AT5G19220.1
Potra2n10c22137.1	-0.398888937	-0.159907629	0.002724527	0.798429987	Potri.010G031000.2	AT2G47900.3
Potra2n10c22138.1	-1.317292482	0.834181121	NA	NA	Potri.003G058100.1	AT2G01050.1
Potra2n10c22139.1	-0.607612345	0.139386737	NA	NA	Potri.010G013800.1	AT2G01050.1
Potra2n10c22140.1	-0.18023457	0.054810157	0.711822304	0.984292161		
Potra2n10c22141.1	-0.17761711	0.040689166	0.320920517	0.973006135	Potri.008G195300.1	
Potra2n10c22142.1	-1.22962587	-0.524514517	6.09669E-07	0.813575895	Potri.010G031500.1	AT3G05030.1
Potra2n10c22143.1	NA	1.571529889	NA	NA		
Potra2n10c22144.1	-1.303534324	-0.155293946	0.000114142	0.927861136	Potri.010G031700.4	
Potra2n10c22145.1	0.077801846	0.042393098	0.649847657	0.973631794	Potri.010G031800.3	AT5G18700.1
Potra2n10c22146.1	0.566601754	0.257650212	4.21114E-09	0.452988225	Potri.010G031900.1	AT5G19180.1
Potra2n10c22147.1	0.246150622	0.031442604	0.071642671	0.981702181	Potri.010G030200.1	AT4G14240.1
Potra2n10c22148.1	-1.50593335	-0.178878608	0.258892299	0.985016122	Potri.010G030500.1	AT5G19040.1
Potra2n10c22149.1	-0.421888407	-0.095894264	0.003223165	0.939824856	Potri.010G030600.4	AT3G06140.1
Potra2n10c22150.1	-0.474740225	0.100461293	0.005914939	0.936890993	Potri.010G030900.1	AT3G06150.1
Potra2n10c22151.4	0.017155253	-0.015610856	0.9280008	0.985393296	Potri.010G029700.1	AT5G19010.1
Potra2n10c22152.1	-0.31080583	-0.101007318	8.61249E-05	0.855432225	Potri.010G029500.1	AT3G06190.1
Potra2n10c22153.1	NA	1.363811008	NA	NA	Potri.006G163800.1	
Potra2n10c22154.1	-0.165778259	0.087232208	0.348488914	0.935474933		
Potra2n10c22155.1	-1.427449909	-0.402251579	0.002683013	0.72476666	Potri.010G029300.2	AT5G18980.1
Potra2n10c22156.1	-0.265711458	0.007455053	0.035186839	0.996032145	Potri.010G029200.1	AT4G14290.1
Potra2n10c22157.1	-1.108178442	-0.344805056	2.91085E-06	0.816206685	Potri.010G029100.1	AT5G35630.2
Potra2n10c22158.1	0.430761077	0.112028274	0.002057018	0.886633917	Potri.010G029000.1	AT3G06250.1

Potra2n10c22159.1	0.238589544	0.061000819	0.014625859	0.947969749	Potri.010G028900.1	AT4G15470.1
Potra2n10c22160.4	-0.155138353	-0.187876883	0.333206034	0.859892315	Potri.T137700.1	AT1G03350.1
Potra2n10c22161.1	0.492216577	-0.255308254	0.222116665	0.949485515	Potri.008G199000.1	AT1G03350.1
Potra2n10c22162.3	-0.243453326	-0.045614322	0.012165733	0.940771888	Potri.010G028700.1	AT5G18940.1
Potra2n10c22163.1	-0.087138175	-0.030191294	0.331178666	0.957064107	Potri.010G028600.1	AT2G20790.1
Potra2n10c22164.1	NA	NA	NA	NA		
Potra2n10c22165.1	-2.088254239	-1.180287005	5.94195E-07	0.666699452	Potri.010G028500.1	AT5G18930.1
Potra2n10c22166.1	-0.266316153	0.134923673	0.064086671	0.846740083	Potri.010G028300.2	AT3G06270.1
Potra2n10c22167.1	0.039746213	0.030830157	0.772939684	0.966917036	Potri.010G028200.1	AT3G06290.1
Potra2n10c22168.1	-0.011178004	-0.150784396	0.970161727	0.914903229	Potri.010G028000.1	AT5G18920.1
Potra2n10c22169.1	-0.02351702	0.120387635	0.865220902	0.830470159	Potri.010G027900.1	AT4G03000.1
Potra2n10c22170.4	-1.643246406	0.321736476	9.49087E-05	0.899853949	Potri.010G027800.5	AT4G15530.5
Potra2n10c22171.1	-1.380521239	-0.348159603	4.50617E-05	0.815929492	Potri.010G027600.1	AT3G21550.1
Potra2n10c22172.1	-0.302816835	0.034162513	0.051057841	0.977559521	Potri.010G027500.1	AT3G21570.1
Potra2n10c22173.1	-0.193982364	0.109896394	0.354726288	0.935851032	Potri.010G027400.2	AT4G15545.1
Potra2n10c22174.2	-1.670071102	-0.252375802	5.00275E-15	0.73224482	Potri.010G027301.1	AT5G18910.1
Potra2n10c22175.1	-0.590551807	0.030590041	2.67759E-11	0.98036348	Potri.010G027100.2	AT3G21510.1
Potra2n10c22176.1	-3.943860599	0.32845669	0.011442141	0.937687311	Potri.008G197400.1	AT5G18860.1
Potra2n10c22177.1	1.011643597	0.00922444	0.001457337	0.99819408	Potri.010G026900.1	AT5G18850.1
Potra2n10c22178.1	0.378091881	0.227501018	0.000288043	0.582507808	Potri.010G026800.1	AT4G03020.2
Potra2n10c22179.1	1.622383676	-0.14207913	NA	0.963878456	Potri.010G026500.1	AT5G18840.1
Potra2n10c22180.1	0.147233083	0.109548254	0.425776555	0.885628484	Potri.010G026200.1	AT5G18830.3
Potra2n10c22181.3	-0.01416006	0.171380221	0.917999487	0.843852055	Potri.010G026100.1	AT5G18810.1
Potra2n10c22182.1	0.446888282	0.09941815	0.001901876	0.91647306	Potri.010G026000.2	AT5G18800.1
Potra2n10c22183.1	-0.198086153	-0.165736986	0.402403288	0.903769998	Potri.010G025300.1	AT4G02500.1
Potra2n10c22184.1	3.20104637	2.791785709	NA	NA	Potri.010G025100.1	AT3G62720.2
Potra2n10c22185.1	-0.263528049	0.027405509	0.020578111	0.982422288	Potri.010G025000.1	AT4G15802.1
Potra2n10c22186.1						
Potra2n10c22187.1	0.878442764	0.108618256	2.10179E-05	0.924256651	Potri.010G024900.5	AT3G16565.2
Potra2n10c22188.1	-1.603368379	-1.133005867	1.75962E-06	0.465182594	Potri.010G024800.1	AT3G16560.1
Potra2n10c22189.1	-0.433407412	-0.500837163	0.045683465	0.453270097	Potri.010G024700.1	AT5G27690.1
Potra2n10c22190.1	2.648033966	0.964013228	0.008072882	NA	Potri.010G024500.1	AT5G24860.1

Potra2n10c22191.1	NA	1.381953902	NA	NA	Potri.008G223100.2	AT1G30480.1
Potra2n10c22192.1	0.242920225	-0.057940505	0.178962324	0.966917036	Potri.010G024400.1	AT4G15770.1
Potra2n10c22193.1	NA	NA	NA	NA		
Potra2n10c22194.1	0.332089439	-1.125666963	0.865510472	NA	Potri.010G024300.1	AT2G47730.1
Potra2n10c22195.1	0.094916985	-0.105126219	0.592772003	0.902857376	Potri.010G024200.1	AT1G09070.1
Potra2n10c22196.4	0.156076477	-0.099552371	0.467045871	0.956738778	Potri.010G024100.1	AT5G48970.1
Potra2n10c22197.1	-0.297421389	-0.151011802	0.462046513	0.949358779	Potri.008G210100.1	AT3G21400.1
Potra2n10c22198.2	-0.118568377	0.367524406	0.690172858	0.456112976	Potri.010G023900.1	AT2G47820.1
Potra2n10c22199.1	-0.227831079	0.109855205	0.005763394	0.863165286	Potri.010G023800.1	AT4G02570.2
Potra2n10c22200.1	-1.696716111	-0.519986107	0.016676831	0.948577865	Potri.010G023600.1	AT3G21420.1
Potra2n10c22201.1	-0.299874439	-0.069712152	0.553420399	0.981761466		
Potra2n10c22202.4	-0.507467564	-0.109726863	0.000225369	0.907032582	Potri.010G023500.1	AT1G09020.1
Potra2n10c22203.1	-0.135826397	0.14124246	0.36443289	0.821140081	Potri.010G023201.1	AT3G21430.2
Potra2n10c22204.2	0.110223871	-0.03366027	0.398695068	0.978410516	Potri.010G023100.1	AT1G29900.1
Potra2n10c22205.1	0.099763278	1.345098989	0.940854462	0.80654978	Potri.009G003600.1	
Potra2n10c22206.1	0.608303174	1.925002723	0.696177417	0.727843918	Potri.010G022900.1	
Potra2n10c22207.1	0.027644155	-0.045607412	0.913874321	0.976302215	Potri.010G022800.1	AT5G46160.2
Potra2n10c22208.1	-0.613064959	-0.259916617	0.000600793	0.677589151	Potri.010G022700.1	AT5G07900.1
Potra2n10c22209.1	-0.214428813	-0.158251437	0.1425833	0.821709751	Potri.008G215700.1	AT5G18550.1
Potra2n10c22210.1	-0.398418162	-0.058876998	0.017015174	0.947317412	Potri.010G022400.1	AT5G18570.1
Potra2n10c22211.1	-0.266369202	-0.145766418	0.014072325	0.849364091	Potri.010G022300.1	AT5G18580.1
Potra2n10c22212.1	0.038088111	-0.005629559	0.769758043	0.995579852	Potri.010G022100.1	AT2G39090.1
Potra2n10c22213.1	0.214833505	0.079183931	0.111839875	0.935851032	Potri.010G022000.1	AT5G18590.1
Potra2n10c22214.1						
Potra2n10c22215.1					Potri.010G021800.1	AT5G18600.1
Potra2n10c22216.1	NA	NA	NA	NA	Potri.010G021800.1	AT5G18600.1
Potra2n10c22217.1	-0.223318147	-0.065313213	0.054753718	0.930598202	Potri.010G021500.2	AT5G18610.2
Potra2n10c22218.1	0.036056799	0.187870465	0.761069366	0.643558659	Potri.010G019800.1	AT3G06400.2
Potra2n10c22219.1	-0.506489428	0.201255962	0.128676099	0.912777843		
Potra2n10c22220.1	0.114879352	0.065913355	0.396834556	0.943483421	Potri.010G019600.1	AT4G39160.2
Potra2n10c22221.1	2.803317047	1.571529889	NA	NA		
Potra2n10c22222.1	-0.214890165	0.106124396	0.140061683	0.939915707	Potri.010G019500.1	AT5G18640.1

Potra2n10c22223.1	0.220785127	0.341170157	0.148651302	0.591292872	Potri.010G019150.1	AT2G28290.2
Potra2n10c22224.1	-0.165143786	-0.154898832	0.304761116	0.912777843	Potri.010G019000.2	AT3G06350.1
Potra2n10c22225.1	0.127985129	-0.12528484	0.829239534	0.971317865	Potri.010G020500.1	AT2G36980.1
Potra2n10c22226.1	-0.61910233	-0.767708831	NA	NA		
Potra2n10c22227.1	-0.057556113	0.103471761	0.790886932	0.926808461	Potri.010G018800.1	AT5G18750.1
Potra2n10c22228.1	-1.178187834	-0.152748524	1.55428E-07	0.912777843		
Potra2n10c22229.1	1.64774763	1.394613675	NA	NA		
Potra2n10c22230.1	1.083855144	1.70131109	0.6471414	NA	Potri.010G015300.1	AT3G21490.1
Potra2n10c22231.1	-0.250095297	0.015587297	0.208755972	0.984292161	Potri.010G015200.1	AT4G15560.1
Potra2n10c22232.1	0.18571387	0.077763594	0.48855768	0.974367223	Potri.010G015100.1	
Potra2n10c22233.2	0.099582022	-0.18621785	0.58388363	0.808630999	Potri.010G014800.1	AT3G06330.1
Potra2n10c22234.1	0.03537903	-0.014912252	0.771155491	0.99114586	Potri.010G014700.1	AT3G21480.1
Potra2n10c22235.1	0.321459136	0.110667509	0.032706885	0.925184207	Potri.010G014600.1	AT5G49020.1
Potra2n10c22236.1	0.398479976	-0.037186733	0.004214921	0.982933413	Potri.010G010800.1	AT4G15960.1
Potra2n10c22237.1	0.872047442	0.21509097	1.18246E-14	0.794596445	Potri.010G011000.1	AT4G15940.1
Potra2n10c22238.1					Potri.010G011100.1	AT1G02790.1
Potra2n10c22239.1					Potri.010G011200.1	AT1G02790.1
Potra2n10c22240.1	0.347165847	0.219132035	0.001303803	0.725908641	Potri.010G011300.2	AT3G06850.1
Potra2n10c22241.1	-2.051387217	-0.175366381	2.06683E-07	0.953831244	Potri.010G011400.1	AT5G49130.1
Potra2n10c22242.1	-0.601685476	0.386274568	0.122012424	NA	Potri.010G011700.1	AT5G49120.1
Potra2n10c22243.1	0.24718395	-0.356818662	0.706144277	0.895560236	Potri.010G011800.1	AT5G49110.1
Potra2n10c22244.1	NA	1.571529889	NA	NA	Potri.T030500.1	AT5G27870.1
Potra2n10c22245.1	0.958552667	0.464383028	5.23811E-06	0.788065047	Potri.T030600.2	AT3G06810.1
Potra2n10c22246.1	0.233855104	-1.87008002	0.9309217	NA	Potri.010G010400.1	
Potra2n10c22247.1	2.876731936	-3.036858925	0.085401719	NA	Potri.T030800.1	
Potra2n10c22248.1	-0.110497855	-3.949103581	0.952890024	0.273841527	Potri.T072000.1	
Potra2n10c22249.1	-1.409983009	0.805829526	NA	NA	Potri.T031100.1	
Potra2n10c22250.2	-0.33605761	-0.598174199	0.777892409	0.876272842	Potri.T011750.1	AT5G36930.1
Potra2n10c22251.1	0.087950768	-0.370606554	0.729974804	0.442809314	Potri.010G014800.1	AT3G06330.1
Potra2n10c22252.2	-0.15810155	0.061033853	0.401721889	0.972159498	Potri.010G014700.1	AT3G21480.1
Potra2n10c22253.1	0.172914161	0.029177554	0.272357034	0.985393296	Potri.010G014600.1	AT5G49020.1
Potra2n10c22254.1	0.270046742	0.072092514	0.026529409	0.95671968	Potri.010G014500.1	AT5G49030.3

Potra2n10c22255.2	-0.182957237	0.431668349	0.577342482	0.820880672	Potri.010G014300.3	AT4G02510.1
Potra2n10c22256.1	-1.766535825	-1.015493184	1.24185E-08	0.090727184	Potri.010G036300.1	
Potra2n10c22257.2	-0.274353841	-0.112538673	0.061888473	0.918573321	Potri.008G222000.1	AT4G15820.1
Potra2n10c22258.1	-0.258022681	-0.155201001	0.133704635	0.890446513	Potri.010G014200.1	AT4G15830.1
Potra2n10c22259.1	0.062131898	-0.04235969	0.775585225	0.97521505	Potri.010G014100.2	AT3G06920.1
Potra2n10c22260.1	0.074106668	0.112231267	0.53691241	0.889258723	Potri.010G014000.1	AT4G15840.1
Potra2n10c22261.1						
Potra2n10c22262.1	-1.317292482	2.500583903	NA	NA		
Potra2n10c22263.1	NA	NA	NA	NA		
Potra2n10c22264.1	NA	NA	NA	NA	Potri.010G009600.1	AT4G27680.1
Potra2n10c22265.1	NA	NA	NA	NA	Potri.011G055100.1	AT5G24490.1
Potra2n10c22266.2	-0.357027419	-0.06356088	0.015466481	0.954559968	Potri.010G013700.2	AT4G15880.1
Potra2n10c22267.2	-0.58750334	-0.263170267	0.000722072	0.853030738	Potri.010G013500.1	AT3G16630.2
Potra2n10c22268.1	0.593137669	0.344318397	0.001915489	0.745183467	Potri.010G013400.1	AT3G16640.1
Potra2n10c22269.2	-0.012197372	0.012378844	0.96695444	0.994123336	Potri.010G013300.2	AT4G15890.1
Potra2n10c22270.1	-0.06464831	0.01796525	0.696705527	0.987226805	Potri.010G013200.1	AT5G49060.1
Potra2n10c22271.1	-0.439441382	-0.033611245	0.021392556	0.98036348	Potri.010G013100.1	AT3G06890.1
Potra2n10c22272.3	0.269225687	0.196902776	0.038482579	0.704368358	Potri.010G012900.1	AT1G26940.1
Potra2n10c22273.4	0.285241521	0.033612184	0.0088083	0.972159498	Potri.010G012800.1	AT4G15900.1
Potra2n10c22274.1	0.319842165	-0.196630955	0.867999908	0.986636967	Potri.010G012400.1	AT3G16660.1
Potra2n10c22275.2	-1.731760303	-2.59744079	NA	NA	Potri.010G012400.1	AT3G16660.1
Potra2n10c22276.1	1.459962178	2.85418539	NA	NA	Potri.010G012400.1	AT3G16660.1
Potra2n10c22277.1	NA	NA	NA	NA	Potri.010G012300.1	
Potra2n10c22278.1	0.405108788	1.206886772	0.419352267	0.759932222	Potri.010G012100.1	AT4G15910.1
Potra2n10c22279.1	0.958807588	-0.182161692	0.005682282	0.966917036	Potri.010G012000.1	AT5G49100.1
Potra2n10c22280.1	2.844666364	-1.521566558	0.003877942	NA	Potri.T003001.1	AT5G36930.2
Potra2n10c22281.1	-0.490120936	0.516558688	0.815531558	0.956865545	Potri.T072000.1	
Potra2n10c22282.1	-2.367361922	-0.919325569	NA	NA	Potri.T031100.1	
Potra2n10c22283.3	3.506004117	-3.431336884	0.00099936	NA	Potri.T001500.1	AT5G36930.2
Potra2n10c22284.1	-0.537987459	-0.510314346	0.703274665	0.921099436	Potri.T029250.1	
Potra2n10c22285.1	-0.435587299	-0.510314346	0.717005953	0.921099436	Potri.T072000.1	
Potra2n10c22286.1	0.600206023	0.434885684	0.488325659	0.935099709	Potri.T011750.1	AT5G36930.1

Potra2n10c22287.1	-1.934126624	-0.481161456	NA	NA	Potri.010G007401.1	
Potra2n10c22288.1					Potri.010G007300.1	AT4G18180.1
Potra2n10c22289.1	-0.05994582	-0.162879969	0.901577437	0.907073354	Potri.010G007200.1	AT3G06790.2
Potra2n10c22290.1	0.075990897	-0.015021654	0.649961718	0.992934677	Potri.010G007000.1	AT3G16760.1
Potra2n10c22291.1	3.598363628	0.944460005	4.84898E-09	0.84487051	Potri.010G006800.1	AT3G16770.1
Potra2n10c22292.1	3.444383376	2.283425176	NA	NA	Potri.T082850.1	
Potra2n10c22293.1						
Potra2n10c22294.1	2.492428534	NA	NA	NA		
Potra2n10c22295.1	NA	NA	NA	NA	Potri.001G153400.1	AT3G27740.1
Potra2n10c22296.1	0.251512668	0.083526426	0.311445833	0.919424623	Potri.010G006300.1	AT3G16785.1
Potra2n10c22297.1					Potri.001G386550.1	
Potra2n10c22298.5	-0.358624936	-0.070977906	0.187695676	0.948577865	Potri.010G006200.1	AT3G05640.1
Potra2n10c22299.1	-0.266381364	0.007533094	0.031878238	0.995579852	Potri.010G009000.1	AT4G16060.1
Potra2n10c22300.1					Potri.010G005900.1	AT1G78850.1
Potra2n10c22301.1	-0.696084024	-0.237429553	0.004507324	0.760731888	Potri.010G005800.2	AT4G16070.1
Potra2n10c22302.1	NA	NA	NA	NA	Potri.010G005700.2	AT3G06778.1
Potra2n10c22303.1	0.886409595	-0.151470518	1.2929E-05	0.960656795	Potri.010G005600.1	AT3G16810.1
Potra2n10c22304.1	-0.530329883	-0.300777493	0.13383195	0.845656549	Potri.010G005500.1	AT3G16850.1
Potra2n10c22305.1	0.211839291	-0.102050345	0.144658009	0.948002367	Potri.010G005400.2	AT5G11240.1
Potra2n10c22306.2	0.675128858	-0.879098022	0.766801836	NA	Potri.010G005300.1	AT1G06330.1
Potra2n10c22307.1	-0.081117867	0.022356981	0.682573584	0.986671181	Potri.010G004800.1	AT3G17630.1
Potra2n10c22308.1	-0.115723655	-2.514137983	NA	NA	Potri.010G005100.1	AT4G24975.1
Potra2n10c22309.1	-0.048253206	0.381459608	0.769216137	0.602611218	Potri.010G004700.1	AT3G06980.1
Potra2n10c22310.1	-0.256354044	0.042886103	0.342261206	0.974628351	Potri.010G004600.1	AT3G17611.1
Potra2n10c22311.1					Potri.T006500.1	AT2G01050.1
Potra2n10c22312.1	0.335953338	0.02032835	0.005600733	0.983944804	Potri.010G004500.2	AT1G16250.1
Potra2n10c22313.1	-0.876853999	-0.575992839	0.000736375	0.72101822	Potri.010G004400.2	AT5G26670.1
Potra2n10c22314.1	-0.1082943	0.019288718	0.804818382	0.992934677	Potri.010G004300.1	AT1G57560.1
Potra2n10c22315.3	-0.094689983	-0.597770424	0.85462632	0.70570456	Potri.010G004200.1	AT3G17609.2
Potra2n10c22316.1	0.065535384	0.003036607	0.714234031	0.998359779	Potri.010G004100.1	AT3G17590.1
Potra2n10c22317.1	-0.035141328	-0.303206296	0.923426254	0.865538396		
Potra2n10c22318.1	1.664677056	NA	NA	NA		

Potra2n10c22319.1					Potri.010G003900.1	AT3G05858.1
Potra2n10c22320.1	0.373666898	0.10545168	0.03631699	0.945254452		
Potra2n10c22321.1	-0.438323278	0.215831054	0.554655234	0.962661188	Potri.010G003700.3	AT1G08530.1
Potra2n10c22322.1	0.025254046	0.297146053	0.950215421	0.916192318	Potri.010G003800.2	AT1G48320.1
Potra2n10c22323.4	0.528806439	0.142140549	0.000240737	0.867910364	Potri.010G003400.1	AT1G62290.2
Potra2n10c22324.1	0.533627807	0.130251132	0.000233696	0.887571031	Potri.010G003500.1	AT5G28050.1
Potra2n10c22325.1	-0.830040421	-0.304773398	3.61597E-09	0.687847759	Potri.010G003300.1	AT1G48310.1
Potra2n10c22326.1	-0.253940175	0.026682655	0.605862084	0.994762886		
Potra2n10c22327.1	-0.958311191	-0.349591948	2.25787E-10	0.662120811	Potri.010G003200.1	AT1G48300.1
Potra2n10c22328.1	-0.673840336	-0.092691683	0.003175002	0.967942029	Potri.010G003100.1	AT1G48280.1
Potra2n10c22329.1	-0.370918209	-0.055357022	0.226380839	0.972159498	Potri.010G003000.3	AT2G47000.1
Potra2n10c22330.5	-0.762618464	-0.070342334	6.74833E-08	0.95076703	Potri.010G002900.2	AT5G48960.1
Potra2n10c22331.1	0.476797637	0.091559325	0.000468709	0.936890993	Potri.010G002800.2	AT3G06960.1
Potra2n10c22332.1	0.114648624	-1.478536539	NA	NA	Potri.010G002700.1	AT3G06950.1
Potra2n10c22333.1	-0.874066258	2.731560428	0.138858651	NA	Potri.010G002600.1	AT1G72100.1
Potra2n10c22334.1	-0.59657338	0.083675161	0.023389256	0.973006135	Potri.010G002500.1	AT3G17510.1
Potra2n10c22335.1	-0.438202803	-0.035758313	0.005151723	0.98177802	Potri.010G002400.2	AT3G17470.1
Potra2n10c22336.1	0.311382929	-0.049009637	0.125675125	0.974275337	Potri.010G002300.1	AT3G17465.1
Potra2n10c22337.1	0.28076819	0.307103788	0.465172206	0.860901249	Potri.010G002200.1	AT1G09520.1
Potra2n10c22338.1	-0.177063872	0.226693648	0.517821595	0.91974459	Potri.010G002100.1	AT5G30495.2
Potra2n10c22339.1	-0.469712129	0.012788123	0.02529252	0.994329048	Potri.010G002000.1	AT3G06483.1
Potra2n10c22340.1	-0.361679723	-0.111449598	0.009093511	0.921432293	Potri.010G001900.1	AT3G17440.1
Potra2n10c22341.1	NA	NA	NA	NA	Potri.010G001800.1	
Potra2n10c22342.1	0.385329044	0.015413838	0.004899677	0.993589787	Potri.010G001700.1	AT3G17430.1
Potra2n10c22343.1	0.298269231	-0.212201233	0.100863396	0.874496903	Potri.008G214400.1	
Potra2n10c22344.3	-0.452306558	-0.575977199	0.023510249	0.576228147	Potri.010G001600.1	AT5G18500.2
Potra2n10c22345.1	1.662541915	0.629146745	6.63736E-17	0.094434177	Potri.010G001500.10	AT1G48210.1
Potra2n10c22346.1	0.169587721	0.121687119	0.061566587	0.814513989	Potri.010G001400.1	AT3G06720.1
Potra2n10c22347.1	3.963588101	3.725495318	0.008838689	NA	Potri.013G136730.1	ATCG00750.1
Potra2n10c22348.1	3.071213876	2.833095976	0.000313353	0.425426555	Potri.013G136598.1	ATCG00800.1
Potra2n10c22349.1	3.025775931	2.92379847	0.000223983	0.389989789	Potri.013G140500.1	ATCG00790.1
Potra2n10c22350.1	2.821688055	2.818260918	0.001868575	0.466145447	Potri.013G136664.1	ATCG00780.1

Potra2n10c22351.1	2.579552933	2.504368632	0.000591002	0.497568278	Potri.013G140700.1	ATCG00770.1
Potra2n10c22352.1	0.047849879	-0.28066389	0.90253824	0.811159921	Potri.010G001300.1	AT3G06740.1
Potra2n10c22353.1	2.346574334	0.8502037	NA	NA		
Potra2n10c22354.1	0.320126581	-0.167612164	0.829724042	NA		
Potra2n10c22355.1	0.105832892	-0.28521833	0.76941808	0.844261648	Potri.010G001100.1	AT4G16120.1
Potra2n10c22356.1	-0.050204064	0.15986503	0.868659454	0.880553952	Potri.010G001000.1	AT4G16110.1
Potra2n10c22357.1	-0.61910233	-0.767708831	NA	NA		
Potra2n10c22358.3	0.836727184	0.320003604	1.01193E-06	0.592872379	Potri.010G000800.1	AT5G49230.1
Potra2n10c22359.1	0.790058487	0.286870884	2.44651E-05	0.745183467		
Potra2n10c22360.1	0.114839053	0.084139549	0.448986275	0.918311446	Potri.010G000700.1	AT5G49220.1
Potra2n10c22361.2	3.72535964	0.704334478	1.56434E-14	0.854309045	Potri.010G000500.1	AT1G24020.2
Potra2n10c22362.1	NA	1.381953902	NA	NA	Potri.010G000200.1	AT1G24020.2
Potra2n10c22363.1	-2.182455212	-0.356240697	0.450936574	NA	Potri.005G000100.1	AT5G26742.2
Potra2n10c22364.2	-0.171076679	0.025699055	0.258644951	0.98409016	Potri.005G000100.1	AT5G26742.2
Potra2n10c22365.1	1.268645186	0.791977232	0.222215243	0.874595634	Potri.001G338700.1	AT5G57280.1
Potra2n10c22366.1						
Potra2n1100s36969.1	-1.446374305	-3.129047247	0.704854965	NA	Potri.016G006400.1	AT4G16360.3
Potra2n1102s36970.1					Potri.002G209000.1	AT3G62930.1
Potra2n1106s36971.1	4.429169598	-1.55593228	0.043009087	NA	Potri.013G063200.1	AT3G05950.1
Potra2n1113s36972.1	-0.117387684	0.634313651	0.919336539	0.864223715	Potri.008G027100.1	AT3G52990.1
Potra2n1116s36973.1	0.253038968	1.104727189	0.961890434	NA		
Potra2n1116s36974.1	1.783968262	1.664378486	0.327454964	0.784036768	Potri.017G064000.2	AT1G01630.1
Potra2n1118s36975.1	2.735991243	NA	NA	NA	Potri.T029300.1	
Potra2n1118s36976.1	1.664677056	NA	NA	NA		
Potra2n1130s36977.1	NA	NA	NA	NA	Potri.002G257700.1	AT1G04040.1
Potra2n1136s36978.1	-2.500018624	-0.953714446	0.067891457	0.851551409		
Potra2n1139s36979.1						
Potra2n114s34556.1	-1.497564707	-0.47580727	6.30082E-06	0.672658526	Potri.015G127400.1	AT1G12260.1
Potra2n114s34557.1	-1.154304157	-0.637774491	0.041854072	0.661257773		
Potra2n114s34558.1	0.910129571	-0.26009883	0.001021806	0.894513758	Potri.015G127200.1	AT4G25240.1
Potra2n114s34559.1	-0.250266108	-0.628212757	0.437834172	0.811159921	Potri.015G127100.1	AT1G62660.1
Potra2n114s34560.1	NA	NA	NA	NA	Potri.015G127000.1	AT3G26040.1

Potra2n114s34561.1	0.269558119	-1.026845944	0.694970178	0.582021931	Potri.015G126900.1	AT5G51460.2
Potra2n114s34562.1	-0.139196142	0.067106145	0.6072569	0.974756511	Potri.015G126800.1	AT5G62390.1
Potra2n114s34563.1	NA	2.906556465	NA	NA	Potri.015G126700.1	
Potra2n114s34564.1	0.102236182	-0.767708831	NA	NA	Potri.015G126600.1	AT3G26040.1
Potra2n114s34565.1	-0.194090704	-0.098950579	0.091089306	0.902857376	Potri.015G126400.1	
Potra2n114s34566.1	4.497193703	0.695388691	6.66035E-55	0.842247476	Potri.015G125700.3	AT4G33050.2
Potra2n114s34567.1	-0.491096126	-0.187473818	0.02138747	0.859892315	Potri.015G125600.1	AT5G44860.1
Potra2n114s34568.1	NA	NA	NA	NA	Potri.013G117150.1	
Potra2n114s34569.1	NA	NA	NA	NA	Potri.015G125500.2	AT5G23260.1
Potra2n114s34570.1	0.08775315	-0.038356989	0.615585282	0.976302215	Potri.015G125400.1	AT5G05010.2
Potra2n114s34571.1	1.512372546	-1.408359186	0.338940203	NA	Potri.015G125300.1	
Potra2n114s34572.1	-0.160512762	-0.216045549	0.336058566	0.860901249	Potri.015G125100.1	AT3G48890.1
Potra2n114s34573.1	-1.049661642	-0.48272662	2.00117E-09	0.365818347	Potri.015G124800.1	AT2G35100.1
Potra2n114s34574.1	0.133073597	-0.020282153	0.458409658	0.991204249	Potri.015G124700.1	AT1G31160.1
Potra2n114s34575.1	-3.708908839	-3.855344786	0.337266134	NA	Potri.015G124600.1	AT1G31170.1
Potra2n114s34576.1	-0.989399638	0.162724003	0.000369443	0.924251575	Potri.012G124400.1	AT2G35110.2
Potra2n114s34577.1	-0.969841473	-0.038346196	0.000187126	0.991147673	Potri.015G124466.1	AT2G35110.1
Potra2n114s34578.1	-0.001545106	-1.507841271	NA	NA	Potri.015G123300.1	AT3G58660.1
Potra2n114s34579.1	-0.28027946	-1.101901928	0.83358255	0.822998494	Potri.015G122800.1	AT5G18820.1
Potra2n114s34580.1	NA	NA	NA	NA	Potri.015G122800.1	AT5G18820.1
Potra2n114s34581.1					Potri.015G123300.1	AT3G58660.1
Potra2n114s34582.1	0.474298835	0.420584432	0.386880762	0.866777645	Potri.015G122800.1	AT5G18820.1
Potra2n114s34583.1					Potri.T175900.1	
Potra2n114s34584.1	-0.18110927	0.118780367	0.706567337	0.967129771	Potri.015G123500.1	AT2G35110.1
Potra2n1164s36980.1	1.459962178	NA	NA	NA	Potri.016G072000.1	AT5G06300.1
Potra2n1182s36981.1	1.459962178	NA	NA	NA		
Potra2n1185s36982.1	-0.098390976	0.018889257	0.456612254	0.986636967	Potri.017G048400.1	AT1G12680.1
Potra2n1187s36983.1	NA	1.571529889	NA	NA	Potri.005G008100.1	AT4G34138.1
Potra2n1189s36984.1	-1.090234723	-0.483347034	1.88778E-06	0.745183467	Potri.004G039700.1	AT3G51000.1
Potra2n1190s36985.1	-0.092185749	-0.064489918	0.579274304	0.960981446	Potri.007G011000.2	AT5G65470.1
Potra2n1197s36986.1						
Potra2n1199s36987.1	NA	1.571529889	NA	NA		

Potra2n11c22367.1	1.779100735	0.562258877	1.00145E-15	0.5736141	Potri.011G169700.1	AT5G56260.1
Potra2n11c22368.1	-0.129387365	-0.0224858	0.538780831	0.9896961	Potri.011G169800.1	AT1G55590.1
Potra2n11c22369.1	0.207015015	0.042066724	0.100549908	0.974275337	Potri.011G169900.1	AT5G56280.1
Potra2n11c22370.2	0.066178279	0.068751161	0.663371766	0.948028999	Potri.011G170000.2	AT5G56290.1
Potra2n11c22371.1					Potri.017G155100.2	AT1G18340.1
Potra2n11c22372.1	NA	1.571529889	NA	NA		
Potra2n11c22373.1					Potri.T020900.1	AT2G19400.1
Potra2n11c22374.1	0.325775529	0.002309472	0.058496446	0.99905083	Potri.011G169600.1	AT1G55610.2
Potra2n11c22375.1	-0.576463603	0.026900781	0.005930246	0.988883632	Potri.011G169300.1	AT5G56270.1
Potra2n11c22376.1	-0.040167332	-0.074013715	0.728420707	0.910815461	Potri.011G169200.1	AT4G26450.1
Potra2n11c22377.1	0.296825409	0.260971087	0.00940286	0.779201817	Potri.011G169100.1	AT5G56240.1
Potra2n11c22378.1	-0.268600691	-0.003035323	0.005465504	0.99736971	Potri.011G169000.3	AT4G26455.1
Potra2n11c22379.1	-3.708908839	-1.489281425	0.337266134	NA	Potri.011G168900.1	AT3G60580.1
Potra2n11c22380.1	-0.333229138	-0.126779287	0.000979245	0.752794139	Potri.011G168600.1	AT3G13340.1
Potra2n11c22381.1	0.081757318	-0.000115191	0.908299948	0.999937671	Potri.011G168500.1	AT5G56170.1
Potra2n11c22382.1	NA	NA	NA	NA	Potri.011G168400.1	AT4G39230.1
Potra2n11c22383.1	-0.183708926	0.014249873	0.128392873	0.989304496	Potri.011G168300.1	AT1G55690.2
Potra2n11c22384.1	0.263589093	-0.095147276	0.07008043	0.91998377	Potri.011G168200.1	AT5G41700.1
Potra2n11c22385.1	-3.274103322	-3.423611766	NA	NA		
Potra2n11c22386.1	0.172481992	0.127017402	0.170164292	0.911339141	Potri.011G168000.1	AT5G56140.1
Potra2n11c22387.1	-4.332710338	-1.530351889	0.02989212	NA	Potri.011G167900.1	AT5G56120.1
Potra2n11c22388.1	-0.61910233	-0.767708831	NA	NA	Potri.011G167800.1	AT1G60950.1
Potra2n11c22389.1	0.007692032	0.102216674	0.958381239	0.896627841	Potri.011G167700.1	AT3G13330.1
Potra2n11c22390.1	0.378289291	0.250899194	0.145886706	0.803444125	Potri.011G167600.1	AT5G56110.1
Potra2n11c22391.1	-0.49632088	-0.117426752	0.034993291	0.921099436	Potri.011G167466.1	AT5G23810.1
Potra2n11c22392.1	0.382004542	0.891034468	0.155599531	0.657465297	Potri.011G167300.1	AT5G09220.1
Potra2n11c22393.1	-0.987658825	-0.494762706	1.09932E-07	0.624602712	Potri.011G167200.1	AT5G23810.1
Potra2n11c22394.1	0.232326021	-0.00074389	0.102545658	0.999650703	Potri.011G166900.1	AT3G13320.1
Potra2n11c22395.1	0.002525769	-0.100290828	0.996119966	0.956865545		
Potra2n11c22396.1	-0.095181467	-0.125134078	0.551730136	0.864349786	Potri.011G166800.1	AT5G56090.1
Potra2n11c22397.1	-1.996242007	-1.338203999	0.013633454	0.651550434	Potri.011G166700.5	AT1G55740.1
Potra2n11c22398.1	3.496836607	NA	0.41259682	NA	Potri.011G166600.1	AT3G13235.1

Potra2n11c22399.1	0.82357019	NA	NA	NA		
Potra2n11c22400.1	-0.657321162	-0.647598977	3.19027E-07	0.025278554	Potri.011G166400.1	AT3G13300.1
Potra2n11c22401.1	-0.673423849	-0.681514053	2.51306E-06	0.035841202		
Potra2n11c22402.1	-1.212304588	-0.138219952	9.49271E-08	0.951726178	Potri.001G469401.1	AT3G13275.1
Potra2n11c22403.1	-0.019050228	0.052581364	0.93600412	0.972159498	Potri.011G166100.1	AT3G13235.3
Potra2n11c22404.1	0.209876231	-0.071097929	0.183534304	0.945173238	Potri.011G166000.1	AT3G13230.1
Potra2n11c22405.1	0.068458264	0.267223526	0.665374947	0.664404282	Potri.011G165900.1	AT5G59810.1
Potra2n11c22406.1	0.818393192	0.516367328	0.282785903	0.895474078	Potri.011G165900.1	AT5G59810.1
Potra2n11c22407.1	4.878205005	2.923753382	0.000296424	NA	Potri.011G165800.1	AT3G51550.1
Potra2n11c22408.1	1.88940142	0.558639294	0.028627617	0.936549944	Potri.011G165900.1	AT5G59810.1
Potra2n11c22409.1	0.006691308	0.227641802	0.978607976	0.855587395	Potri.T143000.1	AT3G07040.1
Potra2n11c22410.1	-0.012166699	0.126119138	0.979169506	0.953177702	Potri.011G165500.1	AT4G26490.1
Potra2n11c22411.1	0.12588862	0.214377791	0.638293307	0.89793147	Potri.001G468300.1	AT4G26500.1
Potra2n11c22412.1	-0.7798611	0.065975336	NA	NA	Potri.011G165300.1	
Potra2n11c22413.1	-1.396563117	-0.551917457	6.64754E-11	0.584487746	Potri.011G165200.1	AT3G44730.1
Potra2n11c22414.1	NA	2.303900616	NA	NA	Potri.011G165100.1	AT4G26510.1
Potra2n11c22415.2	-2.371992074	-0.966290673	0.000808532	0.79078676	Potri.011G165000.1	AT4G26530.2
Potra2n11c22416.1	-0.110354594	0.020063997	0.454949016	0.989878071	Potri.011G164900.2	AT5G56020.1
Potra2n11c22417.1	-1.431010069	-0.07826015	0.044349932	0.985393296	Potri.011G164800.1	AT5G56040.2
Potra2n11c22418.1	NA	NA	NA	NA	Potri.011G164700.1	AT1G30570.1
Potra2n11c22419.1	0.51258639	0.296688517	8.44825E-06	0.590863214	Potri.011G164500.1	AT1G30540.1
Potra2n11c22420.1	0.398914803	0.328945178	0.000134244	0.595538573	Potri.011G164400.1	AT2G34730.1
Potra2n11c22421.1	-3.795375217	0.128139644	0.000221156	0.990896655	Potri.001G466700.1	AT5G14980.1
Potra2n11c22422.1	-0.167946698	-0.054789108	0.120113601	0.953608209	Potri.011G164100.1	AT1G30520.1
Potra2n11c22423.1	0.163355867	-0.233197343	0.569153753	0.912602562	Potri.001G286700.1	AT5G56000.1
Potra2n11c22424.1	0.456711402	0.080901968	0.000883642	0.943483421	Potri.001G371500.2	AT1G30475.1
Potra2n11c22425.1	-0.46052643	-0.233548253	0.230661593	0.902847514	Potri.011G163800.1	AT3G05950.1
Potra2n11c22426.1	-0.359759332	-0.057331643	0.256440278	0.983944804	Potri.011G163700.2	AT4G20940.1
Potra2n11c22427.1	-0.35560736	-0.236156453	0.010610682	0.724504724	Potri.011G163600.1	AT4G20910.1
Potra2n11c22428.1	-0.989855374	-0.701477364	0.049638289	0.688499606	Potri.011G163500.1	AT4G20900.1
Potra2n11c22429.1	0.716548067	0.34420323	6.04492E-07	0.498594799	Potri.011G163400.1	AT5G63890.2
Potra2n11c22430.1	0.697352199	0.330660836	3.89327E-06	0.595538573	Potri.011G163300.1	AT5G39160.1

Potra2n11c22431.1	-0.522581374	-0.129435458	0.629893915	0.985964146	Potri.011G162800.1	AT5G44440.1
Potra2n11c22432.1	-2.71697528	-2.13940518	NA	NA	Potri.011G162800.1	AT5G44440.1
Potra2n11c22433.1	-0.61910233	-0.767708831	NA	NA	Potri.011G162732.1	AT4G20820.1
Potra2n11c22434.1	NA	2.0078969	NA	NA	Potri.011G162600.1	AT3G05950.1
Potra2n11c22435.1	-0.520750127	-0.096494001	0.000875902	0.947969749	Potri.011G162500.1	AT5G23860.2
Potra2n11c22436.2	-0.462048561	-0.079801314	0.001155272	0.955473249		
Potra2n11c22437.1	0.758810898	0.332311889	0.026621434	0.849815276	Potri.001G464300.1	AT4G20880.1
Potra2n11c22438.1	4.120432206	1.363811008	0.122723175	NA	Potri.011G162100.1	AT4G13920.1
Potra2n11c22439.1	-0.672583544	-0.385858914	0.005373154	0.794817498	Potri.011G162000.1	AT2G34770.1
Potra2n11c22440.1	-2.901582317	0.311197827	NA	NA	Potri.005G019400.1	AT1G09190.1
Potra2n11c22441.1	0.970615115	0.481900351	6.86886E-15	0.663037599	Potri.011G161901.1	AT2G34780.1
Potra2n11c22442.1	3.998456404	NA	0.151780887	NA	Potri.011G161600.1	AT5G44400.1
Potra2n11c22443.1	0.178479884	3.233340905	0.925904848	0.445000674	Potri.011G161500.1	AT2G34790.1
Potra2n11c22444.1	4.493881274	NA	0.031229354	NA	Potri.011G161400.1	AT1G30760.1
Potra2n11c22445.1	NA	4.344917737	NA	NA	Potri.011G161600.1	AT5G44400.1
Potra2n11c22446.1	0.262882609	3.551730505	0.882271988	0.422517924	Potri.011G161500.1	AT2G34790.1
Potra2n11c22447.1	-0.887021791	-0.927118871	0.715227272	0.903477395	Potri.011G159600.1	AT1G30760.1
Potra2n11c22448.1	-1.072282108	-0.197981259	7.10739E-06	0.903477395	Potri.011G159000.1	AT1G23460.1
Potra2n11c22449.1	NA	NA	NA	NA		
Potra2n11c22450.1	-1.242723893	-0.428296067	5.30822E-17	0.278663901	Potri.011G158700.1	AT2G34790.1
Potra2n11c22451.1	-1.96050712	-2.108318007	NA	NA	Potri.011G158600.1	AT5G44390.1
Potra2n11c22452.1	-0.898853006	-0.404256275	0.000577055	0.774820633	Potri.011G158400.1	AT1G34575.1
Potra2n11c22453.1	-1.443336056	-0.588312075	3.61564E-05	0.439251891	Potri.011G158300.1	AT1G30700.1
Potra2n11c22454.1	4.361836853	2.064026477	1.31509E-08	0.79078676	Potri.011G158100.1	AT5G44390.1
Potra2n11c22455.1	5.853626498	NA	0.032854013	NA	Potri.011G157900.1	AT5G44440.1
Potra2n11c22456.1	NA	NA	NA	NA	Potri.011G157800.1	AT2G44510.1
Potra2n11c22457.1	-1.090038955	-0.437863602	0.48855768	0.948577865	Potri.011G157700.1	AT2G34820.1
Potra2n11c22458.1	-1.409983009	0.797607471	NA	NA	Potri.011G157600.1	AT4G20790.1
Potra2n11c22459.1	0.217016005	0.301817899	0.129522789	0.613101952	Potri.011G157500.2	AT5G44450.1
Potra2n11c22460.1	-0.868292689	-0.057749142	0.233406249	0.987226805	Potri.011G157400.1	
Potra2n11c22461.1	-0.492695424	-0.325597612	0.000717787	0.813575895	Potri.011G157300.2	AT4G20760.1
Potra2n11c22462.1	-0.646127024	0.129903833	0.321650287	0.984292161	Potri.011G157100.1	AT1G30650.1

Potra2n11c22463.1	0.009341961	0.132805262	0.968096143	0.66362266	Potri.011G157000.2	AT4G14350.1
Potra2n11c22464.1					Potri.014G116600.1	AT2G25580.1
Potra2n11c22465.1	0.135515374	-1.55593228	NA	NA	Potri.014G116600.1	AT2G25580.1
Potra2n11c22466.1	0.204392177	-0.066554331	0.035615613	0.937789963	Potri.011G156900.1	AT1G73720.1
Potra2n11c22467.1	-1.035481738	-0.278384297	0.006196872	0.842680333	Potri.011G156800.2	AT4G21300.1
Potra2n11c22468.1	-1.407120134	-0.52903595	1.40204E-12	0.498467227	Potri.011G156700.1	
Potra2n11c22469.1	-1.474681841	-0.546988561	2.49445E-10	0.518528577	Potri.011G156600.1	AT1G07090.1
Potra2n11c22470.1	-0.877481173	-0.781974282	0.06152349	0.744962075	Potri.011G156500.1	AT3G25690.2
Potra2n11c22471.2	0.173615225	0.024923376	0.177225937	0.985393296	Potri.011G156400.1	AT4G21105.1
Potra2n11c22472.1	0.564315688	0.038608286	0.00048156	0.983446961	Potri.011G156300.1	AT1G25260.1
Potra2n11c22473.1	NA	NA	NA	NA	Potri.001G460000.1	AT3G44680.1
Potra2n11c22474.1	0.674967672	0.701022236	0.106640159	0.70570456	Potri.011G156200.1	AT1G06650.2
Potra2n11c22475.1	-0.497092366	-0.405963309	0.001695756	0.809914834	Potri.011G156100.10	AT1G30620.1
Potra2n11c22476.1	-0.200333731	-0.141672189	0.269155973	0.914903229	Potri.011G155800.1	AT1G30630.1
Potra2n11c22477.1	-0.199114883	-0.274352648	0.219130106	0.728418847	Potri.011G155700.1	AT5G44500.2
Potra2n11c22478.1	-0.912426215	-0.384438444	1.42854E-06	0.679485917	Potri.011G155600.1	AT5G36110.1
Potra2n11c22479.2	-1.19007961	-0.000935324	1.56631E-06	0.999937671	Potri.011G155500.1	AT5G44520.1
Potra2n11c22480.1	-1.581123285	-0.533132166	2.95703E-16	0.514351348	Potri.011G155400.1	AT4G20430.1
Potra2n11c22481.1	NA	NA	NA	NA	Potri.011G155300.1	
Potra2n11c22482.1	0.350093613	0.083998862	0.004953622	0.95671968	Potri.011G155200.1	AT4G20410.1
Potra2n11c22483.1	0.837442692	0.153881695	3.31468E-07	0.894276674	Potri.011G155100.1	AT4G20400.1
Potra2n11c22484.1	0.947613032	-0.920485533	0.159366086	0.860810425	Potri.011G154900.1	AT5G44550.1
Potra2n11c22485.1	-0.225878783	-0.080720095	0.04232974	0.94169165	Potri.011G154800.2	AT4G20380.2
Potra2n11c22486.1	0.345076393	-0.137014148	0.24255514	0.956865545	Potri.011G154700.1	AT5G44560.1
Potra2n11c22487.1	0.491801301	-0.059182418	0.09501367	0.9869326		
Potra2n11c22488.1	-0.538186736	-0.341370111	8.63364E-06	0.518597946	Potri.011G154600.1	AT2G36840.1
Potra2n11c22489.1	-0.499293363	0.108250084	0.035387736	0.925184207	Potri.011G154500.1	AT5G11720.1
Potra2n11c22490.1	-0.603039285	0.126756148	0.025627363	0.955322605	Potri.011G154200.1	AT5G11720.1
Potra2n11c22491.2	-0.165420652	-0.026039324	0.238265576	0.983944804	Potri.011G154000.2	AT4G19490.2
Potra2n11c22492.1	0.833873316	1.832730519	0.357015289	0.637618802	Potri.011G153900.1	
Potra2n11c22493.2	-0.028451924	1.717560592	0.969920774	0.582021931	Potri.011G153800.3	
Potra2n11c22494.1	-0.582421994	0.00342981	0.004876274	0.99905083	Potri.011G153700.2	AT2G35630.1

Potra2n11c22495.1	-0.531740276	-0.168795891	0.027896932	0.859892315	Potri.011G153600.1	AT1G79160.1
Potra2n11c22496.1	-0.91952342	1.205801735	NA	NA	Potri.011G153400.1	AT4G00820.1
Potra2n11c22497.1	NA	NA	NA	NA		
Potra2n11c22498.1	-0.765809121	-0.062655221	0.000240595	0.9761362	Potri.011G153300.1	AT2G46770.1
Potra2n11c22499.1	1.365183748	-1.301680417	0.07339872	0.782659274	Potri.011G153000.1	AT1G64700.1
Potra2n11c22500.1	NA	NA	NA	NA	Potri.011G152900.1	AT1G32780.1
Potra2n11c22501.1	0.222493122	0.019516746	0.508871448	0.992934677	Potri.011G152900.1	AT1G32780.1
Potra2n11c22502.1	0.238192848	0.126478527	0.215741437	0.894276674	Potri.011G152700.4	AT1G32790.1
Potra2n11c22503.1	-0.191670337	0.170979076	0.154912002	0.808901406	Potri.011G152600.1	AT1G32810.2
Potra2n11c22504.2	-0.261126242	-0.063978819	0.007362991	0.941260209	Potri.011G152500.1	AT4G10570.1
Potra2n11c22505.1	-1.308902203	-0.333038065	0.000522243	0.863768668	Potri.011G152400.1	AT1G32860.1
Potra2n11c22506.1	-0.930723159	-0.855037425	0.587793633	0.919722977	Potri.011G152300.1	AT4G38190.1
Potra2n11c22507.1	-2.660638902	-0.054301545	2.61925E-05	0.991147673	Potri.011G152200.1	AT1G32900.1
Potra2n11c22508.1						
Potra2n11c22509.1	0.838814363	-0.024435519	0.15563495	0.996032145	Potri.011G151900.1	AT5G42110.1
Potra2n11c22510.1	NA	NA	NA	NA		
Potra2n11c22511.2	0.408789307	-0.236447137	0.809709349	0.978203092	Potri.011G152100.1	AT3G15850.1
Potra2n11c22512.3	0.353897433	0.126610356	0.383791098	0.921065381	Potri.011G151500.1	AT1G64720.1
Potra2n11c22513.1	-0.234166015	-0.279815184	0.590179383	0.808716407	Potri.011G151600.1	AT1G32928.1
Potra2n11c22514.1	-0.263792237	1.009031531	0.339457099	0.453118701	Potri.011G151400.1	AT1G32930.1
Potra2n11c22515.1	0.748123341	1.000785771	0.168222432	NA	Potri.011G151300.1	AT5G14230.1
Potra2n11c22516.1	0.549212848	0.011068533	6.31082E-06	0.995579852	Potri.011G151200.1	AT1G32960.1
Potra2n11c22517.1	0.044379952	-1.119578101	0.978525619	NA	Potri.011G151100.1	AT4G21630.1
Potra2n11c22518.2	-0.112874184	0.610434798	0.940308659	0.912777843	Potri.011G150900.1	AT1G32960.1
Potra2n11c22519.1	-0.789393235	-0.134261648	0.050558331	0.942026808	Potri.011G150800.1	AT3G61460.1
Potra2n11c22520.1	-1.26260041	-0.426402019	8.01438E-05	0.859892315	Potri.011G150700.1	AT1G32990.1
Potra2n11c22521.1	-2.231457345	-0.243341085	0.124714069	0.98177802	Potri.011G150600.1	AT3G58810.1
Potra2n11c22522.1	1.018795236	0.1177753	0.598740213	NA		
Potra2n11c22523.1	1.517773647	0.47981395	0.371897924	NA	Potri.011G150500.1	AT5G54160.1
Potra2n11c22524.1	-1.726779156	-0.324958802	6.67792E-13	0.475175307	Potri.011G164200.1	AT4G10500.1
Potra2n11c22525.1	7.805631171	2.568574979	9.65965E-07	0.885649686	Potri.011G150100.1	AT4G10490.1
Potra2n11c22526.1	2.409567423	NA	NA	NA	Potri.001G452000.1	AT2G36090.1

Potra2n11c22527.1	2.165919294	NA	NA	NA	Potri.011G149900.1	
Potra2n11c22528.1	3.788570059	NA	0.345734809	NA	Potri.001G452000.1	AT2G36090.1
Potra2n11c22529.1	0.002124506	-0.088291427	0.993727333	0.943483421		
Potra2n11c22530.1	2.241085399	-0.503342041	3.62087E-07	0.937160994	Potri.011G149700.1	AT1G01030.1
Potra2n11c22531.1	2.798132233	1.327965981	2.05367E-20	0.090340507	Potri.011G149601.1	AT1G33050.1
Potra2n11c22532.1	-0.734164508	-0.064498277	0.850276188	NA	Potri.011G149500.1	AT2G18196.1
Potra2n11c22533.1	0.261383992	0.046202872	0.141696901	0.965445938	Potri.011G149300.1	AT1G33060.2
Potra2n11c22534.1	-0.058038344	-0.137141623	0.717011098	0.855310126	Potri.011G149200.1	AT2G45530.1
Potra2n11c22535.1	0.500979176	0.524438178	0.66657096	0.920028955	Potri.011G149000.1	AT3G30340.1
Potra2n11c22536.1	0.485926843	0.56991207	0.678005206	0.918666656	Potri.011G149100.1	AT1G63310.1
Potra2n11c22537.1	NA	NA	NA	NA	Potri.001G453100.1	AT5G19790.1
Potra2n11c22538.1	0.168221334	0.106795676	0.089608286	0.817452127	Potri.011G148800.1	AT1G10390.2
Potra2n11c22539.1	0.86411468	0.144481879	2.70671E-06	0.930124632	Potri.011G148700.2	AT1G33140.1
Potra2n11c22540.1	-1.152020037	-0.02492427	4.69651E-08	0.985393296	Potri.011G148400.4	AT5G07050.1
Potra2n11c22541.1	0.754497252	0.34525021	1.37866E-15	0.170208245	Potri.011G148100.1	AT1G72680.1
Potra2n11c22542.1	NA	NA	NA	NA		
Potra2n11c22543.1	NA	0.674963689	NA	NA	Potri.011G148000.1	AT5G09810.1
Potra2n11c22544.1	2.251683411	-0.066852884	0.512700046	NA	Potri.011G147900.1	AT5G47610.1
Potra2n11c22545.1	0.74952408	0.217923714	6.04405E-06	0.815119099	Potri.011G148400.4	AT5G07050.1
Potra2n11c22546.1	0.736530932	0.177648905	1.40562E-05	0.885115918	Potri.001G454100.1	
Potra2n11c22547.1	0.604501429	-0.860458403	0.768205027	NA	Potri.011G147600.1	AT1G33170.1
Potra2n11c22548.1	2.125312886	NA	NA	NA		
Potra2n11c22549.1	-0.263994276	-0.071788678	0.002153783	0.937846405	Potri.011G147500.1	AT4G10430.3
Potra2n11c22550.1	-0.235632804	0.037683676	0.240410055	0.979719961	Potri.001G454500.1	AT1G76890.2
Potra2n11c22551.1	NA	NA	NA	NA		
Potra2n11c22552.1	1.475807849	0.072438471	1.36151E-09	0.981726976	Potri.011G147300.1	AT1G33250.1
Potra2n11c22553.1	0.135662044	-0.18387283	0.793802004	0.956760199	Potri.011G147100.1	AT4G10390.1
Potra2n11c22554.1	-0.560201376	-0.485646232	0.041604435	0.713085324	Potri.011G147000.4	AT1G33265.1
Potra2n11c22555.1	-0.683132948	-0.316525526	0.006578795	0.842247476	Potri.011G146900.1	AT4G10380.1
Potra2n11c22556.1	-0.272290824	-0.01165291	0.077758122	0.993191555	Potri.011G146800.2	AT1G78690.1
Potra2n11c22557.2	-0.857101449	-0.140669991	1.3893E-06	0.871893718	Potri.011G146700.4	AT5G65930.2
Potra2n11c22558.1	0.799060871	1.071758369	0.67957233	0.894138111	Potri.011G146600.1	AT1G14130.1

Potra2n11c22559.1	2.839656544	1.362996957	1.17746E-37	7.55527E-06	Potri.011G146500.2	AT5G06740.1
Potra2n11c22560.1	-0.229921238	-0.008525675	0.100863396	0.993562283	Potri.011G146400.1	AT3G51500.1
Potra2n11c22561.1	-0.872190801	-0.220951763	1.23068E-07	0.84487051	Potri.011G146300.1	AT4G10550.3
Potra2n11c22562.3	-0.290794923	-0.249918508	0.393089456	0.817191104	Potri.001G456000.1	AT3G43660.1
Potra2n11c22563.1	-0.927031396	0.493689884	NA	NA	Potri.011G146000.1	
Potra2n11c22564.1	0.330930553	0.154580558	0.027173488	0.843683654	Potri.011G145900.1	AT3G51520.1
Potra2n11c22565.1	0.294342954	0.070467476	0.102321133	0.966534713	Potri.011G145800.1	AT4G38180.1
Potra2n11c22566.1	1.287669193	-0.028965331	0.0050187	0.994403137	Potri.011G145700.2	AT1G76520.1
Potra2n11c22567.1	1.676494337	0.49457881	1.32837E-15	0.879147863	Potri.011G145600.1	AT1G76520.1
Potra2n11c22568.1	1.455129132	0.433131177	7.58438E-15	0.890369631		
Potra2n11c22569.4	-0.355321869	-0.174353244	0.000155134	0.884553748	Potri.011G145450.1	AT2G17410.2
Potra2n11c22570.1	0.02172232	-0.131523822	0.916683177	0.874595634	Potri.011G145300.1	AT5G63870.2
Potra2n11c22571.1	-0.688652026	-1.55593228	NA	NA		
Potra2n11c22572.1	0.895223234	-0.147328145	9.41456E-05	0.953608209	Potri.011G145200.1	
Potra2n11c22573.1	1.606582855	-0.247756609	0.049052519	NA	Potri.011G145100.1	AT2G17420.1
Potra2n11c22574.1	2.014674417	-0.585117169	0.001896897	0.921099436	Potri.011G145000.1	AT1G76490.1
Potra2n11c22575.3	0.80438864	0.708601583	0.441945601	0.910970884	Potri.011G144900.1	AT1G13680.1
Potra2n11c22576.1	0.129181689	-0.062858877	0.415963158	0.945150162	Potri.011G144800.2	AT5G41010.1
Potra2n11c22577.1	-0.085633258	0.110109636	0.655338832	0.89372314	Potri.011G144700.1	AT5G65950.1
Potra2n11c22578.1	0.0516771	0.075315565	0.791079968	0.939607998	Potri.011G144500.3	AT1G19100.1
Potra2n11c22579.1	0.210682629	0.206512423	0.169293435	0.676711874	Potri.011G144400.1	AT1G79070.1
Potra2n11c22580.1	0.283902706	-0.193511311	0.13033194	0.842247476		
Potra2n11c22581.1	-0.781379802	-0.033843471	0.000338204	0.987226805	Potri.011G144300.2	AT1G79080.1
Potra2n11c22582.2	-3.14679169	-2.369605713	0.022219911	0.459449783	Potri.011G144100.1	AT3G28150.1
Potra2n11c22583.1	NA	NA	NA	NA	Potri.011G144000.1	AT1G22240.1
Potra2n11c22584.1	0.017324022	0.066247737	0.920224147	0.941855873	Potri.011G143900.1	AT1G79090.1
Potra2n11c22585.1	-1.908795789	-2.058800192	NA	NA		
Potra2n11c22586.1	-2.223296759	0.178541658	0.03381323	0.974367223	Potri.001G457900.1	AT1G56050.1
Potra2n11c22587.1	NA	0.674963689	NA	NA	Potri.011G143500.1	
Potra2n11c22588.1	NA	1.394613675	NA	NA		
Potra2n11c22589.1	-1.683977492	-0.236848756	7.2839E-09	0.924951347	Potri.011G143400.2	AT3G12955.1
Potra2n11c22590.1	0.285008968	-0.014446916	0.376553927	0.996032145	Potri.011G143300.1	AT5G16930.1

Potra2n11c22591.1	-0.996844606	-0.220614172	4.38554E-06	0.824448251	Potri.011G143200.1	AT5G45030.2
Potra2n11c22592.1	0.012589029	-0.121057809	0.947072939	0.922011871	Potri.011G143100.1	AT1G16610.3
Potra2n11c22593.1	0.141616657	-0.010797133	0.302933587	0.99226031	Potri.011G143000.2	AT3G12940.1
Potra2n11c22594.1	-0.148589042	-0.047081336	0.331720732	0.965249957	Potri.011G142900.1	AT3G12930.1
Potra2n11c22595.1	-2.031467177	-0.210753488	0.001183663	0.961820855	Potri.011G142800.1	AT5G23960.2
Potra2n11c22596.1						
Potra2n11c22597.1	NA	NA	NA	NA		
Potra2n11c22598.1	-0.737746792	-0.321613415	3.09246E-05	0.78811993	Potri.011G142700.1	AT3G12920.1
Potra2n11c22599.1	0.468918163	-0.496716432	0.253004707	0.867246972	Potri.011G142600.1	
Potra2n11c22600.1	-0.454701527	-0.225678095	0.00015067	0.895460013	Potri.011G142300.1	AT1G79040.1
Potra2n11c22601.2	-0.106181234	0.112795474	0.556207835	0.930448915	Potri.011G142100.1	AT1G16670.1
Potra2n11c22602.1	-0.706237682	0.011019359	3.68816E-06	0.994762886	Potri.011G142000.1	AT1G79030.1
Potra2n11c22603.6	-0.308274344	0.211556741	0.324036094	0.902676973	Potri.011G141700.2	AT1G55790.1
Potra2n11c22604.1	0.199657447	0.205563591	0.140927412	0.792864576	Potri.011G141400.1	AT3G15120.1
Potra2n11c22605.1	-0.401347366	-0.317295212	0.061673443	0.715585895	Potri.011G141300.1	AT3G15140.1
Potra2n11c22606.1	-0.237575721	-0.182789241	0.311647974	0.885375133	Potri.011G141200.4	AT3G15150.1
Potra2n11c22607.2	-0.294595	-0.250650951	0.231254837	0.816206685		
Potra2n11c22608.1					Potri.011G141100.1	AT2G33060.1
Potra2n11c22609.1	4.522387011	3.690341978	0.031622523	NA	Potri.011G140900.2	AT3G47570.1
Potra2n11c22610.1						
Potra2n11c22611.1					Potri.010G163800.1	AT1G69440.1
Potra2n11c22612.1	5.217772683	1.857934455	0.000229255	NA	Potri.011G140900.2	AT3G47570.1
Potra2n11c22613.1	-0.251078045	0.142514082	0.177947868	0.900176416	Potri.011G138700.1	AT5G54930.1
Potra2n11c22614.1	-0.199593028	0.123250871	0.251619844	0.897285619	Potri.011G138600.1	AT5G54920.1
Potra2n11c22615.1	1.436220289	1.263824576	0.062063828	0.486388477	Potri.011G138500.1	AT3G28210.1
Potra2n11c22616.2	0.145956836	0.222452906	0.207772465	0.788065047	Potri.011G138400.2	AT5G54900.1
Potra2n11c22617.1	-1.51820558	-0.250089111	0.01234245	0.962820058	Potri.011G138300.1	
Potra2n11c22618.1	-0.05680388	-0.040879746	0.639609732	0.966917036	Potri.011G138200.1	AT3G20500.1
Potra2n11c22619.2	0.273179267	0.110705817	0.103047378	0.912295985	Potri.011G138100.1	AT1G50740.1
Potra2n11c22620.1	NA	NA	NA	NA	Potri.011G137800.1	AT5G36110.1
Potra2n11c22621.1	-0.15058276	0.102531543	0.473181238	0.925184207	Potri.011G137700.1	AT4G27020.1
Potra2n11c22622.1	-1.073558501	-0.689950939	0.644741818	NA		

Potra2n11c22623.1	-1.35927387	-1.507841271	NA	NA		
Potra2n11c22624.2	-0.61910233	-0.767708831	NA	NA	Potri.001G424600.1	AT4G27040.1
Potra2n11c22625.1	-1.409983009	-1.55593228	NA	NA	Potri.011G137350.1	AT4G27040.2
Potra2n11c22626.1	-0.085581252	0.092989981	0.654886259	0.930598202	Potri.011G137400.1	AT5G54860.1
Potra2n11c22627.1	0.594171816	0.209976928	0.00257715	0.895575326	Potri.011G137200.1	AT4G12560.2
Potra2n11c22628.1	0.062019693	0.024235526	0.721804213	0.982981438	Potri.011G137100.1	AT5G54855.1
Potra2n11c22629.1	1.212999622	-1.465929376	NA	NA	Potri.011G137000.1	AT3G20530.1
Potra2n11c22630.1	-0.592908592	-0.173094231	0.00233975	0.890369631	Potri.011G136900.3	AT1G50840.1
Potra2n11c22631.1	-0.664107633	-0.194557726	0.001990242	0.894138111	Potri.011G136700.1	AT4G27060.1
Potra2n11c22632.1						
Potra2n11c22633.1	NA	NA	NA	NA	Potri.011G136600.1	AT5G54850.1
Potra2n11c22634.1	NA	NA	NA	NA	Potri.011G136600.1	AT5G54850.1
Potra2n11c22635.1	NA	NA	NA	NA	Potri.011G136600.1	AT5G54850.1
Potra2n11c22636.1	-0.021265382	0.006887066	0.929283727	0.996292992	Potri.011G136400.1	AT1G50910.1
Potra2n11c22637.2	0.639780715	-0.084992738	0.000111061	0.936402128	Potri.011G136300.1	AT5G54840.1
Potra2n11c22638.1	-0.398148504	-3.043858706	0.90253824	NA	Potri.011G136200.1	AT1G56400.1
Potra2n11c22639.2	-0.367795303	0.182007699	0.201939272	0.903034826	Potri.011G136101.1	AT3G20550.1
Potra2n11c22640.1	-0.425759083	0.074023688	0.471088153	0.983113084		
Potra2n11c22641.1	0.113731098	-0.011508385	0.519815671	0.992934677	Potri.011G136000.1	AT4G27070.1
Potra2n11c22642.1	-2.799188917	-3.669882102	0.210799483	NA		
Potra2n11c22643.1	-0.299126283	-0.14710197	0.132324835	0.915488947	Potri.011G135900.1	AT5G54800.1
Potra2n11c22644.1	-0.197479405	0.062959979	0.091892879	0.953177702	Potri.011G135800.1	AT3G15160.1
Potra2n11c22645.1	NA	NA	NA	NA		
Potra2n11c22646.1	-0.39444694	0.359691886	0.231265618	0.863768668	Potri.011G135700.1	AT3G21710.2
Potra2n11c22647.2	0.130284867	-0.009204496	0.487473151	0.996032145	Potri.011G135600.1	AT1G50940.1
Potra2n11c22648.2	-0.212850944	-0.088824621	0.139834487	0.946464685	Potri.011G135500.1	AT4G27080.1
Potra2n11c22649.1	-0.023273419	-0.372306168	0.951305402	0.742764478	Potri.011G135400.1	AT3G20570.1
Potra2n11c22650.1	-0.402473467	0.020268414	0.03301717	0.993589787	Potri.011G135300.2	AT4G27100.2
Potra2n11c22651.1	3.703184167	1.611552969	0.001023675	NA	Potri.011G135200.1	AT3G20580.1
Potra2n11c22652.1	3.521139008	NA	0.247651528	NA		
Potra2n11c22653.1	0.859334057	-0.567392624	0.03560988	0.842247476	Potri.011G135000.1	AT1G11580.1
Potra2n11c22654.1	NA	NA	NA	NA		

Potra2n11c22655.1	-1.065458636	-1.108768083	1.03181E-06	0.001147057	Potri.011G134900.1	AT5G54770.1
Potra2n11c22656.1	-0.829387292	-1.048777273	0.011675426	0.063354245		
Potra2n11c22657.1	-1.300009494	-1.366663596	0.00090265	0.04665809		
Potra2n11c22658.1	0.141871135	0.60463364	0.729081784	0.720488481	Potri.011G134700.1	AT4G27130.1
Potra2n11c22659.1	1.459962178	NA	NA	NA	Potri.011G134600.1	AT5G54740.1
Potra2n11c22660.1	NA	NA	NA	NA	Potri.011G134400.1	AT5G54740.1
Potra2n11c22661.1	NA	1.571529889	NA	NA	Potri.011G134300.1	AT5G03240.1
Potra2n11c22662.1	0.250274165	0.121488296	0.02667304	0.842247476		
Potra2n11c22663.1	0.247915621	0.129109015	0.031015473	0.835819291	Potri.011G134200.1	AT4G05320.4
Potra2n11c22664.1	1.265283853	-0.43624383	0.60293989	NA	Potri.011G134000.1	AT4G21200.1
Potra2n11c22665.1	1.893977425	0.787253256	2.82922E-06	0.71411664	Potri.011G133900.1	AT3G20600.1
Potra2n11c22666.1	5.990568413	4.778002244	0.000181709	NA	Potri.011G133800.1	AT3G28390.1
Potra2n11c22667.1	5.373104075	2.490806657	0.001071204	NA		
Potra2n11c22668.1	3.851270347	1.669090078	1.78167E-11	0.587597195	Potri.011G133300.1	AT1G03670.1
Potra2n11c22669.1	-0.330746994	0.099937788	0.031196616	0.896937427	Potri.011G133100.1	AT4G21880.1
Potra2n11c22670.1	-0.516233541	0.028225335	0.001168877	0.984292161	Potri.011G132900.1	AT1G51100.1
Potra2n11c22671.1	-0.103204947	0.073121851	0.521689671	0.960656795	Potri.011G132800.1	AT3G20720.2
Potra2n11c22672.1	-0.346245808	0.054796477	0.007660991	0.973948905	Potri.011G132700.1	AT5G54730.1
Potra2n11c22673.1	-0.53478594	-0.197382776	0.037780927	0.920141992	Potri.011G132600.1	AT5G54690.1
Potra2n11c22674.3	-0.237148977	-0.013599253	0.062351054	0.988210253	Potri.011G132500.1	AT3G20680.1
Potra2n11c22675.1	-2.674776464	-2.114280153	0.425153588	NA		
Potra2n11c22676.1	-1.409983009	-1.55593228	NA	NA	Potri.011G132100.1	AT1G76820.1
Potra2n11c22677.1	-0.835701465	-0.366221331	2.32684E-05	0.409505482	Potri.011G131900.1	AT5G54670.1
Potra2n11c22678.1	-2.918478698	-0.833957797	0.252441961	NA	Potri.001G416400.1	AT3G01440.1
Potra2n11c22679.1	1.64774763	1.571529889	NA	NA	Potri.001G416400.1	AT3G01440.1
Potra2n11c22680.1	-3.429438695	0.071200245	1.08841E-12	0.9761362	Potri.011G131800.1	AT5G54660.1
Potra2n11c22681.1						
Potra2n11c22682.1	0.482006885	0.023756406	0.050267329	0.987226805	Potri.011G131700.1	AT5G54650.1
Potra2n11c22683.1	-0.216704103	-0.082031272	0.06125163	0.909617075	Potri.011G131500.2	AT4G38070.1
Potra2n11c22684.1	-0.223385802	-0.083098701	0.05153497	0.908764369	Potri.011G131400.1	AT1G51060.1
Potra2n11c22685.1	-0.723723705	-1.827672895	0.604750662	0.692084498	Potri.011G131300.1	AT5G54630.1
Potra2n11c22686.1	-0.988633715	0.190386177	0.000357143	0.930159505	Potri.011G131200.1	AT5G54590.2

Potra2n11c22687.3	1.295954752	0.508683988	1.11048E-15	0.41884898	Potri.011G131100.1	AT1G50600.1
Potra2n11c22688.1	1.724994882	-0.757196931	0.558562855	NA	Potri.011G130800.1	AT3G26300.1
Potra2n11c22689.1	-1.35927387	-0.079512623	NA	NA	Potri.005G095500.1	AT5G23960.1
Potra2n11c22690.1	0.48715348	-0.378327917	0.143155965	0.885375133	Potri.011G130500.2	AT3G20660.1
Potra2n11c22691.1	-1.656169206	0.115491119	2.47624E-09	0.973347974	Potri.011G130400.2	AT5G54585.1
Potra2n11c22692.1	0.338794202	-0.022317425	0.050758195	0.987960806	Potri.011G130300.1	AT5G54580.1
Potra2n11c22693.1	0.427925619	-0.025172121	0.135604949	0.989304496	Potri.011G130200.1	AT5G54570.1
Potra2n11c22694.1					Potri.011G130200.1	AT5G54570.1
Potra2n11c22695.1					Potri.011G130200.1	AT5G54570.1
Potra2n11c22696.1	0.316081768	0.068442973	0.008968046	0.912665563	Potri.011G130100.7	AT3G20650.1
Potra2n11c22697.1	0.550569623	0.089294625	5.30219E-07	0.94076214	Potri.011G130000.1	AT5G54540.1
Potra2n11c22698.1	-0.887434296	-0.284466785	0.000318209	0.88643982	Potri.011G129900.1	AT5G54530.1
Potra2n11c22699.1	-0.985918572	-0.009803221	0.05017895	0.99812851	Potri.006G014400.1	AT3G50950.2
Potra2n11c22700.1	-2.46356382	-0.668133936	3.02714E-08	0.781339094	Potri.011G129700.1	AT5G54510.1
Potra2n11c22701.1	0.872867328	1.178349447	0.156592399	0.328000211	Potri.011G129600.1	
Potra2n11c22702.1	-0.045551284	0.082139683	0.927762795	0.963557834	Potri.011G129500.2	AT3G20640.1
Potra2n11c22703.1	-0.662187337	-0.555099333	0.003368473	0.157412365	Potri.011G129400.1	AT4G27270.1
Potra2n11c22704.1	1.145041455	0.832193492	0.274537902	0.910878765	Potri.011G129300.1	AT4G27290.1
Potra2n11c22705.1	0.237812562	0.534454922	0.495924074	0.813731863	Potri.011G129300.1	AT4G27290.1
Potra2n11c22706.1	1.811896348	0.616328653	2.34003E-11	0.687729258	Potri.011G129100.2	AT4G27280.1
Potra2n11c22707.1	0.697906054	0.414643939	5.36091E-07	0.328000211	Potri.011G128900.1	AT4G21380.1
Potra2n11c22708.1	NA	NA	NA	NA		
Potra2n11c22709.1						
Potra2n11c22710.1						
Potra2n11c22711.1	1.416807913	0.38250433	0.002533867	0.885375133		
Potra2n11c22712.1	1.545832463	1.871975723	NA	NA		
Potra2n11c22713.1	0.012847974	-0.217472013	0.961400394	0.841517891	Potri.T022600.1	AT4G27290.1
Potra2n11c22714.1	-0.013879916	-0.225617905	0.957698574	0.828847087	Potri.T022500.1	AT2G31670.1
Potra2n11c22715.1	5.36101177	0.663364782	6.13725E-05	0.953608209	Potri.T022800.1	AT4G27290.1
Potra2n11c22716.1	5.927999944	2.490806657	2.60131E-05	NA	Potri.T023000.1	AT4G27290.1
Potra2n11c22717.1	1.179497397	2.129138414	NA	NA	Potri.T023100.1	AT4G27290.1
Potra2n11c22718.1	6.621131237	2.349349412	2.61317E-19	0.745183467	Potri.T023200.1	AT4G27290.1

Potra2n11c22719.1	2.645136168	1.911450358	NA	NA	Potri.T023100.1	AT4G27290.1
Potra2n11c22720.1	1.410460047	-1.465929376	NA	NA	Potri.T023100.1	AT4G27290.1
Potra2n11c22721.1						
Potra2n11c22722.1	2.283942031	1.637533156	0.019668556	0.612122112	Potri.T023500.1	AT4G27290.1
Potra2n11c22723.1	6.20666731	2.283425176	4.37415E-06	NA	Potri.011G125000.1	AT4G21380.1
Potra2n11c22724.1	1.296335475	-0.068349583	NA	NA	Potri.011G125100.1	AT4G27290.1
Potra2n11c22725.1	0.914556021	0.565515843	2.96849E-08	0.122109873	Potri.011G125200.1	AT4G27290.1
Potra2n11c22726.1	7.77706145	4.094024515	8.51558E-11	0.483549741	Potri.T024000.1	AT4G27280.1
Potra2n11c22727.1	0.121936046	0.145593229	0.423918958	0.885628484	Potri.T024100.2	AT4G27310.1
Potra2n11c22728.1	0.106937003	-0.216869834	0.436801792	0.751428922	Potri.T024200.1	AT1G11360.2
Potra2n11c22729.1	NA	NA	NA	NA	Potri.011G125600.1	
Potra2n11c22730.1	0.007427984	0.203707838	0.966642966	0.773157146	Potri.011G125700.3	AT4G27340.1
Potra2n11c22731.1	0.13084356	0.021069358	0.223691807	0.981702181	Potri.011G125800.2	AT3G20630.1
Potra2n11c22732.1	-0.001545106	-0.097655521	NA	NA	Potri.011G125900.1	AT4G21440.1
Potra2n11c22733.1	0.359668589	-1.737065466	0.700791502	0.563971296	Potri.011G126100.1	
Potra2n11c22734.1	-0.225587874	0.117124429	0.053556707	0.920141992	Potri.011G126200.2	AT5G54110.1
Potra2n11c22735.1	-0.814102549	-0.356265476	7.69474E-05	0.819353048	Potri.011G126300.1	AT5G54240.1
Potra2n11c22736.1	-1.317292482	2.436555253	NA	NA	Potri.011G126400.1	AT4G27360.1
Potra2n11c22737.1	-0.000938176	0.411585417	0.997714093	0.565689191	Potri.011G126600.1	
Potra2n11c22738.1	-1.317292482	-0.05574223	NA	NA		
Potra2n11c22739.1	-0.514389264	-0.029604605	0.002342189	0.9869326	Potri.001G407100.1	AT5G54270.1
Potra2n11c22740.1	-0.388556451	0.023487745	0.002634755	0.986636967	Potri.011G126800.10	AT5G54280.2
Potra2n11c22741.1	NA	NA	NA	NA		
Potra2n11c22742.1	NA	NA	NA	NA		
Potra2n11c22743.1	0.287427819	-0.355345001	0.826845319	0.965445938	Potri.011G126900.3	AT1G61260.1
Potra2n11c22744.1	0.446343888	0.333727637	0.000311271	0.586851629	Potri.011G127000.1	AT5G54310.1
Potra2n11c22745.1	NA	NA	NA	NA		
Potra2n11c22746.1	0.971135998	0.281571846	0.165143305	0.940703488		
Potra2n11c22747.1	0.693095041	-0.372752989	0.697408194	NA		
Potra2n11c22748.1	-0.025180998	0.191710922	0.918831664	0.865591169	Potri.001G406200.3	AT3G13677.2
Potra2n11c22749.1	-0.284875007	0.07953663	0.013832869	0.908764369		
Potra2n11c22750.1	-0.162824457	-0.004225428	0.565194614	0.998438878	Potri.011G127400.1	

Potra2n11c22751.1	-0.090012522	0.069500384	0.586236544	0.943483421	Potri.011G127600.1	AT3G13682.1
Potra2n11c22752.1	2.95591572	NA	NA	NA		
Potra2n11c22753.1	0.82357019	2.32414027	NA	NA	Potri.011G127900.1	AT5G54370.1
Potra2n11c22754.1	0.280202718	1.193486536	0.587818776	NA	Potri.011G127800.1	AT1G24020.2
Potra2n11c22755.1	0.790188851	1.457860494	0.617768545	0.74860342	Potri.011G127900.1	AT5G54370.1
Potra2n11c22756.1	-0.313892789	-0.047014181	0.077709593	0.971828097	Potri.011G128000.1	AT5G54380.1
Potra2n11c22757.1	0.261398036	0.022166256	0.042096442	0.981726976	Potri.011G124700.1	AT5G54390.1
Potra2n11c22758.1	0.964878307	0.099134666	3.68025E-06	0.931212951	Potri.011G124600.1	AT5G54400.1
Potra2n11c22759.1	0.974830481	-0.250962683	0.000769594	0.863682136		
Potra2n11c22760.1	0.312488054	0.284122955	0.631294798	0.937036471	Potri.011G124300.1	AT3G26040.1
Potra2n11c22761.3	-0.017856443	-0.05981209	0.917751896	0.954419252	Potri.011G123900.1	AT5G54440.1
Potra2n11c22762.1	NA	1.571529889	NA	NA	Potri.011G123800.1	AT3G15520.1
Potra2n11c22763.1	-0.181917595	0.143592444	0.484481912	0.936549944	Potri.011G123700.2	AT1G52870.2
Potra2n11c22764.1	0.662612763	-0.03942236	0.591475752	0.996032145	Potri.011G123600.1	AT3G15518.1
Potra2n11c22765.1	-1.463235674	-0.763097667	3.56242E-09	0.188620592	Potri.011G123500.1	AT3G15510.1
Potra2n11c22766.1	-0.989219295	-1.158019373	0.755773162	NA		
Potra2n11c22767.1	-1.372859353	0.436263644	0.003534899	NA	Potri.011G123300.1	AT4G27410.2
Potra2n11c22768.1	-1.328951947	-0.005869733	NA	NA	Potri.011G123200.1	AT4G27420.1
Potra2n11c22769.1	NA	NA	NA	NA		
Potra2n11c22770.1	NA	NA	NA	NA	Potri.011G123100.1	AT1G52900.1
Potra2n11c22771.1	NA	0.674963689	NA	NA	Potri.011G123000.2	AT2G20875.1
Potra2n11c22772.1	-0.972852797	-0.380380553	2.74013E-06	0.689693427	Potri.011G122900.1	AT4G27430.1
Potra2n11c22773.1	0.796826484	-0.046370319	NA	NA	Potri.011G122800.1	AT2G14830.1
Potra2n11c22774.1	-0.801534413	-0.285167774	1.17431E-05	0.821709751	Potri.011G122700.2	AT4G27435.1
Potra2n11c22775.1	-0.001545106	-1.507841271	NA	NA	Potri.011G122600.1	AT1G61065.1
Potra2n11c22776.1	NA	NA	NA	NA		
Potra2n11c22777.1	-0.240271686	-0.070897159	0.0967713	0.948577865	Potri.011G122500.1	AT3G15470.1
Potra2n11c22778.1	NA	2.490806657	NA	NA	Potri.011G122400.1	AT5G54190.1
Potra2n11c22779.1	1.275607896	-0.13992182	3.41311E-08	0.935851032	Potri.011G122200.1	AT3G15460.1
Potra2n11c22780.1	1.521876908	-0.104103675	1.18334E-09	0.969421568	Potri.011G122100.1	AT4G27450.1
Potra2n11c22781.1	1.721859941	0.489884501	2.10934E-05	0.838742197	Potri.011G122000.1	AT5G53750.1
Potra2n11c22782.1	0.495141474	-0.09033909	0.011721466	0.953831244	Potri.011G121900.1	AT3G15430.1

Potra2n11c22783.1	0.287946839	0.097500642	0.119325737	0.939653927	Potri.011G121800.1	AT3G15420.1
Potra2n11c22784.1	-0.238469826	0.071160729	0.013301874	0.936890993	Potri.011G121700.1	AT3G15410.1
Potra2n11c22785.1	-0.747648863	0.779461954	0.050782131	0.229802222	Potri.011G121600.1	AT5G53760.2
Potra2n11c22786.1	NA	NA	NA	NA	Potri.011G121500.2	AT4G14103.2
Potra2n11c22787.1	-2.233812304	0.926973878	0.227019294	0.809914834	Potri.011G121400.1	AT1G16930.1
Potra2n11c22788.1	-1.060086885	0.559172547	0.014042188	0.677589151		
Potra2n11c22789.1	-3.3854576	1.07214336	0.056106026	0.900884796	Potri.011G121300.1	AT5G17260.1
Potra2n11c22790.1	3.577563304	1.356921795	3.26474E-33	0.166599048	Potri.011G121200.1	AT4G12560.2
Potra2n11c22791.1	0.039578419	-0.094050799	0.845685506	0.912083068	Potri.011G121100.1	AT3G15380.1
Potra2n11c22792.1	0.023884273	-0.045182226	0.887594488	0.956865545	Potri.011G121000.1	AT5G53800.1
Potra2n11c22793.1	-0.580571642	-0.354942327	0.062793963	0.794641467	Potri.011G120900.1	AT4G21705.1
Potra2n11c22794.1	0.383052668	-0.162026754	0.288005728	0.943586317	Potri.011G120800.1	AT4G27470.1
Potra2n11c22795.1	-0.016825614	0.156439514	0.920914494	0.755774501	Potri.011G120500.1	AT3G15360.1
Potra2n11c22796.1	3.073325943	2.501508747	NA	NA	Potri.011G120400.1	AT1G23220.1
Potra2n11c22797.1	-0.47593382	-0.116765475	0.165143305	0.966228173	Potri.011G120300.1	AT5G60020.1
Potra2n11c22798.1	-0.172326879	-0.067695769	0.300053715	0.965595755	Potri.011G120100.1	AT1G53000.1
Potra2n11c22799.1	-0.291139982	0.127802721	0.041040652	0.911339141	Potri.011G120000.1	AT2G02040.1
Potra2n11c22800.1	NA	NA	NA	NA	Potri.011G119900.1	AT1G04360.1
Potra2n11c22801.1	-1.216199648	-0.257829872	3.55988E-15	0.642397767	Potri.011G119800.2	AT1G53120.1
Potra2n11c22802.1	-1.164857696	-0.21273201	7.35899E-11	0.815348505		
Potra2n11c22803.1	0.565491153	0.33075414	1.3118E-07	0.371280386	Potri.011G119700.1	AT4G27500.1
Potra2n11c22804.1	1.401996969	0.104099249	8.90169E-07	0.955473249	Potri.011G119600.1	AT3G15350.2
Potra2n11c22805.1	-0.012149937	0.191222438	0.961148207	0.875861859	Potri.011G119500.1	AT3G15354.1
Potra2n11c22806.1	-0.485875701	-0.19498354	4.95379E-05	0.80340893	Potri.011G119400.1	AT1G53050.1
Potra2n11c22807.1	-0.481464317	-0.016638904	0.000114588	0.990128003	Potri.011G119300.1	AT1G53040.2
Potra2n11c22808.1	-2.872622301	-0.650724173	NA	NA	Potri.011G119200.1	AT1G53035.1
Potra2n11c22809.3	-1.793960541	-0.142518799	1.35334E-31	0.912777843		
Potra2n11c22810.1	-1.875374956	-1.254562026	0.137005559	0.826265918		
Potra2n11c22811.1	-1.808678096	-0.143010511	6.27018E-32	0.912777843	Potri.011G118900.2	AT3G15353.1
Potra2n11c22812.1	NA	NA	NA	NA		
Potra2n11c22813.2	0.992671099	0.406587908	5.20106E-09	0.428801064	Potri.011G118800.1	AT1G53025.1
Potra2n11c22814.1	-0.902404687	0.945504454	NA	NA	Potri.011G118600.1	AT1G53130.1

Potra2n11c22815.1	0.909107629	1.420317805	0.030833472	0.284918677	Potri.011G118500.1	AT1G53140.1
Potra2n11c22816.1	-0.141010628	1.502868803	0.956243052	0.900869139		
Potra2n11c22817.1	-0.285115827	1.766095894	0.932027938	NA	Potri.011G118300.1	
Potra2n11c22818.1	-0.113603864	0.681040691	0.705135334	0.659635889		
Potra2n11c22819.1	-0.08451841	0.204824232	0.697549587	0.79314783	Potri.001G399000.1	AT5G53850.2
Potra2n11c22820.1	0.330735043	0.375474976	0.010516266	0.534852254	Potri.011G118000.1	AT5G53860.2
Potra2n11c22821.1	1.262047987	-0.54048692	0.360383473	NA	Potri.011G117800.1	AT5G53870.1
Potra2n11c22822.1	0.334143933	-0.233049957	0.103622309	0.859892315	Potri.011G117600.1	AT3G14620.1
Potra2n11c22823.1	0.350268272	-0.115994596	0.051496692	0.909715408	Potri.011G117600.1	AT3G14620.1
Potra2n11c22824.1	0.673577339	-0.050678397	0.000155434	0.973948905	Potri.011G117600.1	AT3G14620.1
Potra2n11c22825.1	-0.261577277	-0.364952567	0.322982075	0.799049555	Potri.011G117500.1	AT1G64980.1
Potra2n11c22826.1	-1.328951947	-1.478536539	NA	NA	Potri.011G117400.2	AT3G23560.1
Potra2n11c22827.1	1.341619736	0.752747093	0.135790449	0.8954569	Potri.011G117300.1	AT3G23560.1
Potra2n11c22828.1	1.403695518	0.265691916	5.47647E-14	0.873955419	Potri.011G117001.1	AT5G53880.1
Potra2n11c22829.1	0.700529074	0.027060985	3.76363E-06	0.987226805	Potri.011G116900.1	AT5G53890.1
Potra2n11c22830.1	NA	2.283425176	NA	NA	Potri.014G116300.1	
Potra2n11c22831.1	0.819737347	-0.305778905	0.000938875	0.907073354	Potri.011G116800.1	AT3G15270.1
Potra2n11c22832.1	1.663267765	0.373606853	1.53883E-14	0.858462498	Potri.011G116700.1	AT3G15260.1
Potra2n11c22833.1	-2.316346109	-0.097625916	NA	NA	Potri.011G116600.1	AT1G53163.1
Potra2n11c22834.1	1.066426227	0.515386825	0.000424944	0.724604666	Potri.011G116400.1	AT5G53900.2
Potra2n11c22835.3	-0.26947576	0.233257489	0.101075428	0.868145269	Potri.011G116300.10	AT3G15220.1
Potra2n11c22836.1	1.165858921	0.150979459	0.493676007	NA		
Potra2n11c22837.2	0.14187634	0.14070972	0.686022324	0.941260209	Potri.011G116200.1	AT4G27540.1
Potra2n11c22838.1	0.366542052	-0.078558923	0.28736795	0.97950444	Potri.011G116100.1	AT5G53920.1
Potra2n11c22839.1	-0.220698254	-0.003747953	0.04289952	0.99702095	Potri.011G115900.1	AT5G53930.1
Potra2n11c22840.1	-0.079106171	0.171889607	0.786371199	0.924251575	Potri.011G115700.1	AT5G53940.1
Potra2n11c22841.1	-0.024696012	0.200745051	0.944696114	0.920664994	Potri.019G028100.1	ATCG00350.1
Potra2n11c22842.1	0.033600089	0.627277983	NA	NA		
Potra2n11c22843.1	0.673254754	0.489716912	0.014005594	0.788065047	Potri.011G115600.1	AT3G15210.1
Potra2n11c22844.1	-0.042756664	0.041439494	0.896718971	0.989329144	Potri.011G115500.1	AT3G15200.1
Potra2n11c22845.1	1.545832463	1.571529889	NA	NA	Potri.011G115400.1	AT5G53950.1
Potra2n11c22846.1					Potri.011G115200.1	AT3G30390.1

Potra2n11c22847.1					Potri.011G115200.1	AT3G30390.1
Potra2n11c22848.1	NA	NA	NA	NA	Potri.016G105000.2	AT3G54000.1
Potra2n11c22849.1	-0.16714619	0.115694922	0.648082639	0.960981446	Potri.011G115100.1	AT5G53970.1
Potra2n11c22850.1	0.010090556	0.010953882	0.967316784	0.995920419	Potri.011G115000.1	AT5G57850.1
Potra2n11c22851.3	-0.417269636	-0.166380565	0.015533486	0.851826887	Potri.011G115000.1	AT5G57850.1
Potra2n11c22852.1	2.409567423	NA	NA	NA	Potri.011G114900.1	AT1G78720.1
Potra2n11c22853.1	0.569383116	0.06294947	2.39432E-05	0.953831244	Potri.011G114800.1	AT2G33845.1
Potra2n11c22854.1	4.003725508	1.444801915	1.92502E-14	0.259011748	Potri.011G114600.1	AT2G20562.1
Potra2n11c22855.1	0.147982256	-2.284754124	0.922576401	0.71411664	Potri.011G114500.2	AT5G53980.1
Potra2n11c22856.1	0.229140543	0.16185959	0.205850638	0.915339763	Potri.011G114300.2	AT1G53670.1
Potra2n11c22857.1					Potri.011G114900.1	AT1G78720.1
Potra2n11c22858.1	-0.187006585	0.123356539	0.304275656	0.880553952	Potri.011G114100.1	AT1G17160.1
Potra2n11c22859.1	1.086289099	-0.105915544	1.70459E-07	0.935851032	Potri.013G137200.2	AT1G17180.1
Potra2n11c22860.1	0.054810819	-0.268914019	0.795703739	0.544498018	Potri.002G099100.1	AT4G08980.3
Potra2n11c22861.1	4.77293529	0.831588573	0.00209975	NA	Potri.011G114000.1	AT1G78380.1
Potra2n11c22862.1	NA	1.571529889	NA	NA	Potri.010G044400.1	AT1G10740.4
Potra2n11c22863.1	3.358738996	-1.507841271	0.050253671	NA	Potri.011G114000.1	AT1G78380.1
Potra2n11c22864.1	NA	NA	NA	NA	Potri.011G112900.1	AT1G17180.1
Potra2n11c22865.1	5.2004754	-0.167267867	2.22472E-08	NA	Potri.011G113000.1	AT1G78380.1
Potra2n11c22866.1						
Potra2n11c22867.1	4.811226006	1.372906117	0.010935329	NA	Potri.011G113400.2	AT1G17170.1
Potra2n11c22868.1						
Potra2n11c22869.1	2.556135291	NA	NA	NA	Potri.011G114000.1	AT1G78380.1
Potra2n11c22870.1	-0.262485663	-1.612047656	0.861801728	NA	Potri.011G113300.1	AT1G17180.1
Potra2n11c22871.1	NA	NA	NA	NA		
Potra2n11c22872.1	0.177893727	0.095590537	0.092746754	0.889391488	Potri.011G112800.1	AT3G14400.1
Potra2n11c22873.1	0.524047441	0.53515829	0.064390627	0.734057255	Potri.011G112700.1	AT3G14420.1
Potra2n11c22874.1	0.188370944	-0.040333416	0.239562301	0.976983641	Potri.011G112500.1	AT1G31660.1
Potra2n11c22875.1	1.64774763	NA	NA	NA	Potri.011G131000.1	
Potra2n11c22876.1	-0.626945754	-0.274349274	0.079216349	0.885375133	Potri.011G112400.1	AT3G14440.1
Potra2n11c22877.1	-0.439662544	-0.178809013	0.091026195	0.927879595	Potri.011G112300.2	AT1G53250.1
Potra2n11c22878.1	0.009621676	-0.246049469	0.96695444	0.839942912	Potri.011G112200.1	AT3G15000.1

Potra2n11c22879.1	2.231876712	0.54061484	0.139738908	NA	Potri.011G112100.1	AT1G53270.1
Potra2n11c22880.1	1.531819558	0.244244381	6.44093E-08	0.922993749	Potri.011G112000.1	AT1G16670.1
Potra2n11c22881.1	3.743577741	NA	0.341195965	NA		
Potra2n11c22882.1	1.144396238	0.529228838	5.87856E-13	0.163343066	Potri.011G111900.1	AT1G53280.1
Potra2n11c22883.1	-0.057532243	0.010802779	0.955595032	0.999053662	Potri.011G111700.1	AT1G53290.1
Potra2n11c22884.1	-2.277249482	0.926230009	NA	NA		
Potra2n11c22885.1	-0.503623647	-0.666071277	0.022365184	0.593648258	Potri.011G111500.1	AT1G53300.1
Potra2n11c22886.2	-0.028704843	-0.020840926	0.821863149	0.985393296	Potri.011G111400.1	AT1G78870.2
Potra2n11c22887.1					Potri.011G111300.1	AT1G29140.1
Potra2n11c22888.1	0.102184087	0.097956991	0.483030954	0.911094206	Potri.011G110900.1	AT2G42590.2
Potra2n11c22889.1	0.173318963	0.035651896	0.345384154	0.978080344	Potri.011G110800.1	AT1G16900.1
Potra2n11c22890.1	-0.380563564	0.064645432	0.00490638	0.945254452	Potri.011G110700.2	AT3G14940.1
Potra2n11c22891.1	1.129060808	2.07592521	0.443048855	NA	Potri.011G110500.1	AT1G78850.1
Potra2n11c22892.2	7.043321564	1.571529889	1.82478E-05	NA	Potri.011G110300.1	AT1G78860.1
Potra2n11c22893.1	4.225497907	-0.381005366	8.50132E-12	0.919424623	Potri.011G110200.1	AT1G78860.1
Potra2n11c22894.1	-1.091436862	-0.461937002	0.507142063	0.945254452	Potri.011G110100.1	AT5G43580.1
Potra2n11c22895.1	-0.440804238	0.244632008	0.00575302	0.775618506	Potri.011G109900.1	AT2G42610.2
Potra2n11c22896.1	-0.725859219	-0.145891948	0.035809289	0.945254452	Potri.001G390800.1	AT3G14930.1
Potra2n11c22897.1	-0.410848139	-2.039847804	0.923023837	NA		
Potra2n11c22898.1	-0.24985291	-0.192187945	0.137217992	0.841527401	Potri.011G109700.1	AT3G14920.1
Potra2n11c22899.1	-0.134180561	0.051715983	0.257816827	0.959881296	Potri.011G109600.2	AT1G78810.1
Potra2n11c22900.1	-0.042403211	-0.107112044	0.75806895	0.862335622	Potri.011G109500.1	AT3G58680.1
Potra2n11c22901.1	NA	NA	NA	NA		
Potra2n11c22902.1	-0.019761157	0.081465607	0.911424294	0.91998377	Potri.011G109300.1	AT1G53320.1
Potra2n11c22903.1	0.360819901	0.131124561	0.014472211	0.857374454	Potri.011G109000.1	AT1G78800.1
Potra2n11c22904.2	0.254899745	0.162567117	0.094764522	0.84253168		
Potra2n11c22905.1	-1.280458166	-0.486222092	7.15734E-40	0.250129374	Potri.011G108800.1	AT1G78770.1
Potra2n11c22906.1	-2.159802904	-0.636448629	3.83566E-51	0.168252739	Potri.011G108900.2	AT1G78780.2
Potra2n11c22907.2	NA	NA	NA	NA	Potri.011G108700.1	
Potra2n11c22908.1	-0.528367927	-0.121871145	0.042981195	0.943483421	Potri.011G108500.1	AT1G53330.1
Potra2n11c22909.1	1.824649808	1.214896031	0.618928621	NA		
Potra2n11c22910.1	1.911189818	-0.767708831	NA	NA	Potri.011G108300.1	

Potra2n11c22911.1					Potri.005G240400.1	
Potra2n11c22912.1						
Potra2n11c22913.1					Potri.010G012200.1	
Potra2n11c22914.1	1.117140574	0.067719152	0.000980227	0.975578749	Potri.011G108000.1	AT1G53380.1
Potra2n11c22915.1	0.382187305	0.098208198	0.001220503	0.892320444	Potri.011G107900.1	AT2G34250.2
Potra2n11c22916.1	-0.003955655	-0.082704734	0.988925849	0.955379045	Potri.011G107800.1	AT3G14860.2
Potra2n11c22917.1	-0.219805495	-0.362035551	0.259171905	0.616163505	Potri.011G107700.1	AT1G53400.1
Potra2n11c22918.1						
Potra2n11c22919.1	-0.091073274	0.012233211	0.673522099	0.994788236	Potri.011G107600.1	AT1G78680.1
Potra2n11c22920.1	0.79450471	0.120999536	0.382887121	0.97950444	Potri.011G107500.1	
Potra2n11c22921.1	0.235930007	0.182829422	0.007386776	0.541062638	Potri.011G107400.2	AT1G16970.1
Potra2n11c22922.1	0.632700487	-0.199442933	0.55739612	0.974264269	Potri.011G104500.1	AT1G53490.1
Potra2n11c22923.1	7.800367473	5.102586928	9.44682E-11	0.054066632	Potri.011G107301.1	
Potra2n11c22924.1	0.140100938	0.015458438	0.476270023	0.989304496	Potri.011G106800.1	AT1G78700.1
Potra2n11c22925.2	-0.065203639	-0.015714072	0.699368447	0.985393296	Potri.011G106700.1	AT1G29800.2
Potra2n11c22926.1	-0.188330724	-0.019532918	0.17776251	0.983113084	Potri.011G106600.1	AT3G14830.1
Potra2n11c22927.1	NA	0.674963689	NA	NA	Potri.011G106500.1	AT2G34380.1
Potra2n11c22928.1	2.586399915	0.474202462	3.22433E-06	0.895474078	Potri.011G106400.1	AT1G53440.1
Potra2n11c22929.1	-0.223698865	0.097615055	0.348155042	0.948577865	Potri.011G106300.1	AT3G14850.2
Potra2n11c22930.1	-1.816024556	-0.797658522	2.91415E-10	0.510352711	Potri.011G106200.1	AT1G29050.1
Potra2n11c22931.1	0.199713461	0.298899932	0.158617437	0.739520512	Potri.011G106100.1	AT1G78630.1
Potra2n11c22932.1	0.566446073	0.075513802	0.000180619	0.948577865	Potri.011G105700.1	AT1G78620.1
Potra2n11c22933.1	1.464278026	0.67829777	0.245515394	NA	Potri.001G384200.1	AT3G49180.1
Potra2n11c22934.1	-0.647349863	-0.317050734	0.002105724	0.87909151	Potri.011G105500.1	AT1G78610.1
Potra2n11c22935.1	-0.596056042	-0.329357701	8.46222E-10	0.736477029	Potri.011G105400.1	AT1G78600.1
Potra2n11c22936.1	1.283539525	-0.422641423	0.560351789	NA	Potri.011G104500.1	AT1G53490.1
Potra2n11c22937.1	1.573571787	0.090644079	0.265251049	NA	Potri.011G104500.1	AT1G53490.1
Potra2n11c22938.1	0.752080141	0.250231397	0.005910528	0.900869139	Potri.T085300.1	AT1G78590.1
Potra2n11c22939.4	0.0645008	0.077192145	0.641298858	0.927794025	Potri.011G104200.1	AT3G26922.1
Potra2n11c22940.1	-0.034578868	0.060094823	0.853764072	0.963557834	Potri.012G059200.1	AT1G18560.1
Potra2n11c22941.1	-1.548449532	-2.318405279	0.467254172	NA		
Potra2n11c22942.1	-1.620652703	-0.468305793	2.17486E-16	0.829716187	Potri.011G103900.2	AT1G78580.1

Potra2n11c22943.1	0.042779643	-0.094692305	0.80658794	0.951731753	Potri.011G103700.1	AT1G78570.1
Potra2n11c22944.1	0.169940157	-0.072941731	0.424446745	0.956865545	Potri.011G103600.1	AT3G14770.1
Potra2n11c22945.6	-0.064060624	0.278614225	0.883361457	0.794222545	Potri.011G103500.1	AT3G14760.1
Potra2n11c22946.1	-0.068888097	0.025899972	0.887301876	0.993589787	Potri.011G103200.1	AT1G78560.1
Potra2n11c22947.1	-0.907930168	-1.690258449	0.468969202	0.597141087	Potri.011G103100.1	AT3G14740.2
Potra2n11c22948.1	-0.368647664	-0.06170776	0.261683265	0.9574223	Potri.011G102500.1	AT3G14720.1
Potra2n11c22949.1	-0.696502185	-0.454339026	2.28874E-07	0.665081972	Potri.011G098800.4	AT3G14690.1
Potra2n11c22950.1	0.680375604	-0.7090195	0.744323131	NA		
Potra2n11c22951.2	-0.32932592	-0.09613866	0.144484879	0.956865545	Potri.011G101800.1	AT1G53520.1
Potra2n11c22952.1	0.73862675	-0.046370319	NA	NA		
Potra2n11c22953.2	-0.409451295	-0.23460402	0.230331538	0.90226843	Potri.011G101900.1	AT1G78530.1
Potra2n11c22954.1	-0.138397668	-0.157754585	0.490776169	0.896937427	Potri.011G102100.1	AT1G78540.1
Potra2n11c22955.1	1.317394203	0.424101103	0.003900778	0.902676973	Potri.001G381000.1	AT4G16580.1
Potra2n11c22956.1	-0.488349558	-0.51925447	0.804157521	0.947317412	Potri.011G102400.1	
Potra2n11c22957.3	NA	1.571529889	NA	NA	Potri.004G232250.1	AT5G49760.1
Potra2n11c22958.1	-0.076949676	-0.129173962	0.644644963	0.901671593	Potri.011G085100.1	AT1G30070.1
Potra2n11c22959.2	3.161264147	NA	NA	NA	Potri.011G084900.2	AT4G18340.1
Potra2n11c22960.1	NA	NA	NA	NA	Potri.011G087000.1	
Potra2n11c22961.1						
Potra2n11c22962.1	0.068921016	0.076223832	0.782352029	0.954419252	Potri.011G087100.1	AT3G21610.1
Potra2n11c22963.1	-0.695707431	-0.081294106	0.000355553	0.961021731	Potri.012G013800.1	AT5G53440.1
Potra2n11c22964.1	-0.337174024	0.288362556	0.301728653	0.850863374	Potri.011G087200.1	AT4G26850.1
Potra2n11c22965.1					Potri.001G355700.1	AT1G50650.1
Potra2n11c22966.1						
Potra2n11c22967.2	-0.655674631	-0.340586392	0.000923492	0.711204954	Potri.011G087500.1	AT1G29980.1
Potra2n11c22968.1	0.796620512	0.638595775	1.211E-07	0.408808106	Potri.011G087900.2	AT4G26640.2
Potra2n11c22969.1					Potri.011G088000.1	AT2G26730.1
Potra2n11c22970.1					Potri.011G088000.1	AT2G26730.1
Potra2n11c22971.1	-0.255490907	-0.138444047	0.122974359	0.903477395	Potri.011G088100.1	AT3G07810.2
Potra2n11c22972.1	0.207486809	0.103429334	0.045683805	0.886059841	Potri.011G088400.1	AT5G55530.1
Potra2n11c22973.1	0.82357019	3.157019191	NA	NA	Potri.011G020000.1	
Potra2n11c22974.2	-0.100349501	0.22815	0.681776892	0.812394185		

Potra2n11c22975.1	-0.34932489	0.2071333	0.099986829	0.86716397	Potri.011G091200.12	AT2G34660.1
Potra2n11c22976.1	NA	NA	NA	NA	Potri.013G100900.1	AT2G01050.1
Potra2n11c22977.1	-1.803522348	-2.15347766	0.483315617	0.793963403	Potri.011G085501.1	AT5G55570.1
Potra2n11c22978.1	0.188065142	0.0657273	0.423244917	0.962661188	Potri.011G088500.1	AT5G55510.1
Potra2n11c22979.1	-0.004082044	-0.205583402	0.987039542	0.821709751		
Potra2n11c22980.1	1.545832463	NA	NA	NA	Potri.001G262100.1	AT5G59240.1
Potra2n11c22981.3	0.123098734	0.499250514	0.650662626	0.680396395	Potri.001G356400.3	AT4G22320.1
Potra2n11c22982.1	0.192037232	-0.08411104	0.248892879	0.953831244	Potri.011G089000.2	AT5G55200.1
Potra2n11c22983.1	-0.13959469	-0.375822073	0.959084559	NA	Potri.011G089100.1	AT5G42570.1
Potra2n11c22984.1	0.303346881	-0.016571746	0.008058444	0.987186186	Potri.011G089200.1	AT4G26910.1
Potra2n11c22985.1	0.133667503	0.162943074	0.256073482	0.760731888	Potri.011G089300.1	AT5G55060.1
Potra2n11c22986.1	-0.091978822	0.076544223	0.702080806	0.953831244	Potri.011G089500.3	AT3G20475.1
Potra2n11c22987.1	1.787381571	1.076135738	0.00072209	0.476937791	Potri.011G089700.4	AT5G55050.1
Potra2n11c22988.1	1.787381571	1.076135738	0.00072209	0.476937791		
Potra2n11c22989.2	-0.02454354	0.035307203	0.877097351	0.972159498	Potri.011G089901.1	AT1G30460.1
Potra2n11c22990.1	0.224805658	0.175281367	0.107174798	0.817452127	Potri.011G090100.1	AT1G50660.1
Potra2n11c22991.1	-0.120152238	0.17839727	0.456590688	0.71411664	Potri.011G090200.1	AT2G34680.1
Potra2n11c22992.1	-0.238648976	0.993785176	0.849336555	0.821709751		
Potra2n11c22993.1	0.205789273	0.00120125	0.169038097	0.999458638	Potri.011G090900.1	AT1G30450.2
Potra2n11c22994.1	-0.801978343	-0.544658913	9.41715E-07	0.657795666	Potri.011G091100.1	AT1G30440.1
Potra2n11c22995.2	0.729943	0.589924778	3.97538E-12	3.06759E-05	Potri.011G091200.12	AT2G34660.1
Potra2n11c22996.1	1.637470394	1.6485862	0.000949434	0.001481258		
Potra2n11c22997.1	-0.358908696	-0.208275387	0.0287702	0.849446235	Potri.001G357900.1	AT4G05420.1
Potra2n11c22998.1	6.209276118	3.600724457	4.43784E-06	NA	Potri.011G091700.1	AT4G03620.1
Potra2n11c22999.1	-1.317292482	-1.465929376	NA	NA	Potri.001G358100.1	AT4G21070.1
Potra2n11c23000.1	NA	NA	NA	NA	Potri.011G091800.1	AT2G34640.1
Potra2n11c23001.1	-0.141308533	0.003169766	0.244414617	0.999223888	Potri.011G091900.4	AT1G30330.1
Potra2n11c23002.2	-0.148375082	0.265500461	0.932694741	NA	Potri.001G358600.1	AT1G30320.1
Potra2n11c23003.1	-0.278450669	0.112530815	0.006225791	0.935099709	Potri.011G092200.1	AT5G55260.1
Potra2n11c23004.2	0.210443148	0.158676653	0.150744687	0.885900567	Potri.011G092500.1	AT5G55230.1
Potra2n11c23005.1					Potri.011G084800.1	AT3G56710.1
Potra2n11c23006.1	-0.244430368	-0.107254129	0.069777773	0.912777843		

Potra2n11c23007.1	0.244586118	0.219637348	0.000843719	0.512736781	Potri.011G092700.3	AT5G55310.1
Potra2n11c23008.1	-0.438866468	0.299684448	0.227130179	0.857189843	Potri.011G092800.1	AT3G13120.2
Potra2n11c23009.1	-0.258976914	-0.110034929	0.37698876	0.948834049	Potri.011G092900.2	AT1G55915.1
Potra2n11c23010.1	-0.274890538	-0.207166866	0.139052282	0.889809456	Potri.011G093000.1	AT1G55900.1
Potra2n11c23011.2	-0.050156618	0.254916269	0.68310118	0.558624809	Potri.001G367400.2	AT4G26630.1
Potra2n11c23012.1	-0.510441249	0.270913823	0.039566376	0.825615044		
Potra2n11c23013.1	-0.513498267	0.280668155	0.044865685	0.829862776	Potri.011G093400.2	AT5G63180.1
Potra2n11c23014.1	0.637057337	0.132392517	0.698749969	NA	Potri.011G093500.1	AT5G55730.1
Potra2n11c23015.1	0.049294799	-0.592963998	0.922369699	0.638236514	Potri.011G093700.1	AT5G55830.1
Potra2n11c23016.2	0.390644315	0.279176818	0.000723168	0.677520889	Potri.011G094100.1	AT1G55860.2
Potra2n11c23017.1	0.363667245	0.25638507	1.2787E-05	0.784407529	Potri.011G094200.2	AT5G55850.1
Potra2n11c23018.1	-0.336745236	-0.12741843	0.118293065	0.953608209	Potri.011G094400.1	AT5G55180.1
Potra2n11c23019.1	NA	1.571529889	NA	NA		
Potra2n11c23020.1	-0.343278321	1.318418146	0.872372978	NA		
Potra2n11c23021.1	0.530435704	0.062885045	9.48362E-05	0.953831244	Potri.011G094500.1	AT5G39850.1
Potra2n11c23022.2	-0.61910233	0.666960761	NA	NA	Potri.001G370600.1	AT3G13220.1
Potra2n11c23023.1	-0.21572162	0.269825943	0.526618067	0.912295985	Potri.011G094800.1	AT5G55970.2
Potra2n11c23024.1	-0.477235561	-0.190926302	0.000406012	0.874507876	Potri.011G094900.1	AT4G26570.1
Potra2n11c23025.1	-0.649289686	-0.05476264	0.206808829	0.985393296	Potri.011G095000.1	AT4G21740.1
Potra2n11c23026.1	-1.968543118	0.322081026	0.432489762	0.969524231		
Potra2n11c23027.1	-0.415116095	0.41314458	0.112634145	0.72680202	Potri.011G095100.2	AT3G14570.1
Potra2n11c23028.1	0.231335331	0.116905917	0.100588135	0.893333193	Potri.011G095200.1	AT1G17110.1
Potra2n11c23029.1					Potri.011G095300.1	ATCG00580.1
Potra2n11c23030.1					Potri.T058600.2	ATCG00540.1
Potra2n11c23031.1						
Potra2n11c23032.1	1.293859738	-0.062866504	0.347669476	NA	Potri.011G095500.1	
Potra2n11c23033.1	1.663022003	0.403431636	0.028196493	0.942558727	Potri.011G095600.1	AT1G78440.1
Potra2n11c23034.2	0.198486431	-0.012019551	0.241300472	0.992345356	Potri.011G096100.1	AT1G17130.1
Potra2n11c23035.1	0.233550954	0.112339458	0.051033529	0.875423265	Potri.011G096000.1	AT1G78420.1
Potra2n11c23036.1	-1.317292482	0.863873905	NA	NA	Potri.011G095900.1	AT1G78410.1
Potra2n11c23037.1	0.142696629	0.21042269	0.473789752	0.609209505	Potri.001G377500.1	AT3G14450.1
Potra2n11c23038.1					Potri.T046500.1	

Potra2n11c23039.1	NA	NA	NA	NA		
Potra2n11c23040.3	0.428509094	0.230016881	0.007293671	0.842247476	Potri.011G096200.1	
Potra2n11c23041.1	0.364274569	0.101845441	0.0004095	0.912510104	Potri.011G096300.1	AT1G53240.1
Potra2n11c23042.1	-0.150324953	0.151623496	0.441864918	0.89393732	Potri.011G096400.1	AT1G06230.1
Potra2n11c23043.1	-1.040850361	-0.259964957	1.90228E-06	0.869175327	Potri.011G096500.1	AT3G15050.1
Potra2n11c23044.1	0.072405892	0.443264113	0.752408441	0.442002381		
Potra2n11c23045.1	0.015908923	0.553781802	0.978525619	0.771626158		
Potra2n11c23046.1	-0.060582355	0.409599417	0.941970394	0.902857376		
Potra2n11c23047.1	-0.289006828	-3.783029238	0.925672737	NA	Potri.011G097000.1	AT1G73160.1
Potra2n11c23048.1	-0.026913048	-0.030486197	0.901577437	0.983113084	Potri.011G097100.1	AT1G53190.1
Potra2n11c23049.5	0.737911424	0.174273729	1.25277E-07	0.791589438		
Potra2n11c23050.1	-0.144899855	-0.008216128	0.369881631	0.995409858	Potri.011G097500.1	AT3G18380.1
Potra2n11c23051.1	NA	0.674963689	NA	NA		
Potra2n11c23052.1	-0.301279391	0.094233848	0.404968745	0.974091403	Potri.011G097700.1	AT3G15095.1
Potra2n11c23053.1	-1.017713376	-0.528829079	0.002552003	0.538978673	Potri.011G097800.1	AT3G15110.1
Potra2n11c23054.1	NA	NA	NA	NA	Potri.011G097900.1	AT5G54010.1
Potra2n11c23055.1	NA	NA	NA	NA		
Potra2n11c23056.1	-0.871233916	0.340218185	0.395837052	0.943685779	Potri.001G155750.1	AT1G21280.1
Potra2n11c23057.1	NA	NA	NA	NA	Potri.011G097900.1	AT5G54010.1
Potra2n11c23058.1	NA	NA	NA	NA	Potri.011G097900.1	AT5G54010.1
Potra2n11c23059.1	1.664677056	NA	NA	NA	Potri.011G097900.1	AT5G54010.1
Potra2n11c23060.1	0.677025989	0.110799973	0.00030542	0.951394085		
Potra2n11c23061.1	0.670014985	0.110742598	0.000423629	0.938967511	Potri.011G098000.1	AT4G05400.2
Potra2n11c23062.1	0.333783446	-0.013396701	0.00816233	0.987226805	Potri.011G098100.1	AT1G30480.1
Potra2n11c23063.1	0.025192687	0.185599541	0.919504198	0.828625141	Potri.011G098200.4	AT3G29130.2
Potra2n11c23064.1	-0.349741698	-0.001780404	0.004817248	0.999325699	Potri.011G098300.1	AT2G34710.1
Potra2n11c23065.1	-0.434628555	0.03609237	0.015368953	0.978080344	Potri.011G098400.1	AT1G30500.2
Potra2n11c23066.1	0.033065438	0.397798474	0.88999699	0.805461106	Potri.011G098500.1	AT4G21160.4
Potra2n11c23067.2	0.492562153	0.094820944	6.7887E-05	0.924309478	Potri.011G100800.1	AT3G12290.1
Potra2n11c23068.1					Potri.011G098700.1	AT1G80960.2
Potra2n11c23069.1	0.116049627	-0.22094192	0.476557361	0.869999596	Potri.011G098800.4	AT3G14690.1
Potra2n11c23070.1	-0.483412276	-0.396496086	0.000368569	0.731968019	Potri.011G098800.4	AT3G14690.1

Potra2n11c23071.1					Potri.T037000.2	AT2G01050.1
Potra2n11c23072.2	0.407904883	0.224994354	0.168774008	0.92223649		
Potra2n11c23073.1	-0.098367591	0.162660137	0.472820481	0.886633917	Potri.011G082900.1	AT5G46170.1
Potra2n11c23074.1	-0.10803004	0.370331191	0.517295558	0.777047113	Potri.011G082800.2	AT5G46180.1
Potra2n11c23075.1	NA	NA	NA	NA	Potri.011G082700.1	
Potra2n11c23076.3	-0.026006664	0.133843248	0.885684811	0.897331585	Potri.011G082500.1	AT5G46190.1
Potra2n11c23077.1	-0.163153624	-0.25648147	0.664316293	0.874507876	Potri.011G082300.2	AT1G30290.1
Potra2n11c23078.2	0.071469698	0.155865659	0.587644829	0.826906752	Potri.011G082100.1	AT5G46150.2
Potra2n11c23079.1	-0.153655702	0.259549098	0.452432163	0.776094736	Potri.011G091200.12	AT2G34660.1
Potra2n11c23080.1	-0.27383874	0.097258008	0.044436248	0.914551931	Potri.011G091200.12	AT2G34660.1
Potra2n11c23081.1	-1.435890552	-0.511949817	1.44693E-11	0.509370219	Potri.011G081500.2	AT1G30280.1
Potra2n11c23082.1	-1.360671363	-0.695560895	3.36712E-07	0.026667631	Potri.011G081400.1	AT2G34620.1
Potra2n11c23083.1	0.091702614	0.109771678	0.333035481	0.899757743	Potri.011G081200.4	AT1G17210.1
Potra2n11c23084.1	NA	NA	NA	NA	Potri.011G081001.1	AT1G30260.1
Potra2n11c23085.1	0.35183973	-3.089949151	0.892888016	NA	Potri.011G019000.1	AT3G54070.1
Potra2n11c23086.1	0.058107596	0.837905211	NA	NA	Potri.011G018550.1	AT3G54070.1
Potra2n11c23087.2	1.751688385	0.409637006	0.124044266	0.943483421	Potri.011G018550.1	AT3G54070.1
Potra2n11c23088.1	-0.289295167	-0.170790094	0.343269494	0.93263929	Potri.011G080900.2	
Potra2n11c23089.1	-0.267726845	-0.09778811	0.453828433	0.966434509		
Potra2n11c23090.1	0.035309522	0.173692586	0.818277011	0.799690151	Potri.011G080700.1	AT5G46250.1
Potra2n11c23091.1	-0.332375869	-0.008348588	0.053520497	0.99492996	Potri.011G080600.1	AT4G18270.1
Potra2n11c23092.1	0.223355347	0.014235175	0.133229766	0.990916799	Potri.009G019400.2	AT3G52580.1
Potra2n11c23093.2	-1.150421071	-0.770487177	0.001046443	0.360181553	Potri.011G080400.2	AT4G18260.1
Potra2n11c23094.2	-0.170092411	-0.075415431	0.35882756	0.935851032	Potri.011G080300.1	AT4G18260.1
Potra2n11c23095.1	-0.495690879	-0.128348249	3.24295E-06	0.79078676	Potri.011G080000.1	AT1G29950.1
Potra2n11c23096.1	0.122568944	0.084252684	0.405670289	0.939653927	Potri.001G370900.1	AT3G07660.1
Potra2n11c23097.1	-0.050512062	-0.109755406	0.771227122	0.885459527	Potri.011G079700.1	AT5G46290.1
Potra2n11c23098.1	-0.850148189	-0.351103508	3.67479E-05	0.674283226		
Potra2n11c23099.6	-0.709846413	-0.251994372	0.005710055	0.911339141	Potri.011G079400.2	AT2G34410.2
Potra2n11c23100.1	9.240337244	5.077897786	2.26712E-17	0.679485917	Potri.011G079300.1	AT4G18170.1
Potra2n11c23101.1	8.012850732	4.081935946	4.07595E-12	NA		
Potra2n11c23102.1	-0.076577041	0.050314937	0.746040599	0.96478728	Potri.011G079100.1	AT4G18160.1

Potra2n11c23103.1	0.333169315	0.067753824	0.129563311	0.975578749	Potri.011G079000.1	AT1G29790.1
Potra2n11c23104.1	0.778401848	0.294979582	0.000311574	0.874808359	Potri.004G193200.1	AT1G06340.1
Potra2n11c23105.1	0.168688479	0.170420745	0.369167904	0.870321018	Potri.011G130000.1	AT5G54540.1
Potra2n11c23106.2	0.227371318	-0.060665256	0.053639243	0.930203195	Potri.011G078800.2	AT1G29350.1
Potra2n11c23107.1	-0.282175226	0.075404681	0.017209723	0.924309478	Potri.011G078700.1	AT5G46390.2
Potra2n11c23108.1	0.720608647	0.4375085	0.377250328	0.935851032	Potri.011G078500.1	AT1G29380.1
Potra2n11c23109.1	0.211715462	0.252892149	0.020627411	0.536575584	Potri.011G078300.10	AT5G46410.2
Potra2n11c23110.1	-0.207369051	-0.046976975	0.426597077	0.978203092	Potri.011G078200.1	AT4G18100.1
Potra2n11c23111.2	1.544909243	1.178206983	0.004158853	0.554040943	Potri.011G078050.1	AT5G63700.1
Potra2n11c23112.1	1.222284026	1.111597876	0.196327616	0.773707293	Potri.011G078050.1	AT5G63700.1
Potra2n11c23113.1	0.242693414	-0.261601096	0.837006058	0.966191551	Potri.002G262000.1	AT5G42740.1
Potra2n11c23114.1	0.244650286	0.592821109	0.81510772	0.895460013	Potri.001G082900.1	AT1G56140.1
Potra2n11c23115.1	-0.050253835	0.37912553	0.856999834	0.704224102	Potri.011G077900.1	AT3G47810.1
Potra2n11c23116.1	0.069602925	-0.061567839	0.899820644	0.987226805	Potri.011G077800.1	AT5G46530.1
Potra2n11c23117.1	-0.315966436	0.071173872	0.008148119	0.965249957	Potri.011G077700.1	AT1G29530.1
Potra2n11c23118.1	-0.057183264	0.245720164	0.755486583	0.849446235	Potri.011G077500.1	AT4G18060.1
Potra2n11c23119.1	0.129091386	-0.212117456	0.762926139	0.910815461	Potri.011G077450.1	
Potra2n11c23120.1	2.685015931	NA	NA	NA	Potri.T112400.1	AT4G14130.1
Potra2n11c23121.1	-0.595952689	-0.744589582	NA	NA	Potri.T112300.1	AT3G23730.1
Potra2n11c23122.1	-0.134764435	-0.126561892	0.299221864	0.874648591	Potri.011G077200.1	AT4G18040.1
Potra2n11c23123.1	0.011319372	0.169065584	0.939522301	0.826906752	Potri.011G077100.1	AT5G14270.2
Potra2n11c23124.1	-0.404869404	-0.482134526	0.388591656	0.754009738	Potri.011G077000.1	AT1G29630.2
Potra2n11c23125.1	-2.736154587	-1.095334218	6.53547E-07	0.327435845	Potri.011G076900.1	AT1G29640.1
Potra2n11c23126.2	-0.267277673	0.019000694	0.05311533	0.985393296	Potri.011G076800.1	AT4G18975.4
Potra2n11c23127.1	1.157275325	1.186863962	6.36665E-05	0.046701305	Potri.011G076700.1	AT5G45650.1
Potra2n11c23128.1	0.82357019	2.86450839	NA	NA	Potri.018G089600.1	AT2G01050.1
Potra2n11c23129.1	-0.283996582	0.125765258	0.159566562	0.920763566	Potri.011G076600.1	AT5G45660.1
Potra2n11c23130.1	3.645563783	1.285705001	0.004471793	NA	Potri.011G076500.1	AT1G29670.1
Potra2n11c23131.1	NA	NA	NA	NA		
Potra2n11c23132.4	3.323379971	5.279658394	0.081875469	0.149897924	Potri.011G076400.1	AT5G45670.1
Potra2n11c23133.1	NA	3.00300511	NA	NA	Potri.011G075900.1	AT1G29660.1
Potra2n11c23134.1	0.81139189	2.71235385	NA	NA		

Potra2n11c23135.1	2.151323863	NA	NA	NA	Potri.011G075800.1	AT4G18960.1
Potra2n11c23136.1	NA	NA	NA	NA	Potri.004G150000.2	AT5G66600.1
Potra2n11c23137.1	-0.345999456	0.231610011	0.179826835	0.831597503	Potri.004G064400.1	AT4G18950.1
Potra2n11c23138.1	0.033060332	0.12950051	0.855521724	0.882609368	Potri.011G075600.1	AT4G18930.1
Potra2n11c23139.2	0.62761394	-0.021915435	0.002637091	0.9896961	Potri.011G075500.1	AT1G29690.1
Potra2n11c23140.1	-1.35927387	-1.507841271	NA	NA		
Potra2n11c23141.1	1.202614859	0.502912231	3.20063E-05	0.794596445	Potri.T032684.1	AT1G29740.1
Potra2n11c23142.1	NA	NA	NA	NA		
Potra2n11c23143.1						
Potra2n11c23144.1	1.299130241	0.660132119	9.19494E-09	0.560452893	Potri.T032684.1	AT1G29740.1
Potra2n11c23145.1	0.562461685	0.33767549	0.069574335	0.827673117	Potri.T032400.1	AT1G29750.2
Potra2n11c23146.1	-0.06135881	0.291482557	0.871550708	0.82876584	Potri.T032400.1	AT1G29750.2
Potra2n11c23147.2	1.820449668	0.53561686	2.27984E-06	0.841487301	Potri.011G075400.10	AT1G29740.1
Potra2n11c23148.1	1.459962178	NA	NA	NA		
Potra2n11c23149.1	1.145188426	1.072876929	0.000114346	0.194302431	Potri.011G075400.10	AT1G29740.1
Potra2n11c23150.1	1.810931787	3.395806065	0.302258578	0.344206454	Potri.T032684.1	AT1G29740.1
Potra2n11c23151.1	0.177521885	0.319194666	NA	NA	Potri.011G072732.1	AT1G29730.1
Potra2n11c23152.1						
Potra2n11c23153.1	-0.42534661	-1.029246207	0.870706708	NA	Potri.T032966.1	AT1G29730.1
Potra2n11c23154.1	-0.26494064	-0.274979155	0.626365982	0.885649686	Potri.T032400.1	AT1G29750.2
Potra2n11c23155.1	-0.052270173	-1.55593228	NA	NA	Potri.016G083700.1	
Potra2n11c23156.1	1.39147675	0.986938149	0.000263767	0.301110935	Potri.011G075400.10	AT1G29740.1
Potra2n11c23157.1	NA	1.571529889	NA	NA	Potri.016G111200.1	AT1G19260.1
Potra2n11c23158.3	NA	1.571529889	NA	NA	Potri.T032966.1	AT1G29730.1
Potra2n11c23159.1	0.295320687	0.622909391	0.372381332	0.121610607	Potri.T032400.1	AT1G29750.2
Potra2n11c23160.1	0.232921275	0.602487168	0.517071995	0.180635806		
Potra2n11c23161.1	NA	NA	NA	NA	Potri.T032684.1	AT1G29740.1
Potra2n11c23162.1	NA	1.571529889	NA	NA		
Potra2n11c23163.1	0.194477491	0.400827689	0.569486117	0.455529955	Potri.011G072566.1	AT1G29750.2
Potra2n11c23164.1	3.052532068	1.467275004	0.012973073	NA	Potri.011G073700.4	AT1G29740.1
Potra2n11c23165.2	5.23234356	2.02782542	2.51901E-30	0.301110935	Potri.011G075400.10	AT1G29740.1
Potra2n11c23166.1	1.566421348	0.767538338	0.000491222	0.733263164	Potri.T032684.1	AT1G29740.1

Potra2n11c23167.1	1.106988568	1.326079401	0.281658034	0.833952007	Potri.011G073700.4	AT1G29740.1
Potra2n11c23168.1	3.238949128	1.413252665	0.000171749	NA	Potri.T009700.1	AT1G29740.1
Potra2n11c23169.1	1.018900976	0.905159461	0.013728615	0.420046656	Potri.011G075400.10	AT1G29740.1
Potra2n11c23170.1	0.854292211	0.88069076	0.148468467	0.663037599		
Potra2n11c23171.1	NA	NA	NA	NA		
Potra2n11c23172.1	-0.196435178	-0.304896115	0.424526128	0.679710442	Potri.T032400.1	AT1G29750.2
Potra2n11c23173.1	0.34088352	0.114698964	0.01004831	0.915432211	Potri.011G072400.1	AT2G34480.1
Potra2n11c23174.5	0.490129178	0.180823631	0.016792704	0.915020432	Potri.011G072300.1	AT1G29750.2
Potra2n11c23175.1	NA	NA	NA	NA		
Potra2n11c23176.1	-0.61910233	0.660619236	NA	NA	Potri.002G217200.1	AT4G12770.1
Potra2n11c23177.1	-0.065405567	-0.129516236	0.635214512	0.885375133	Potri.011G072200.1	AT1G29760.1
Potra2n11c23178.1	1.644580903	0.790550539	1.47975E-09	0.20654467	Potri.011G072100.1	AT4G18910.1
Potra2n11c23179.1	-0.70585142	-0.659514153	0.159880317	0.775830532	Potri.011G072000.1	AT5G45700.1
Potra2n11c23180.1	0.027608626	-0.026490222	0.896605074	0.987226805	Potri.011G071900.1	AT1G29800.1
Potra2n11c23181.1	-0.757443628	-0.147440714	5.82256E-05	0.88661378	Potri.011G071800.1	AT1G78700.1
Potra2n11c23182.1	-0.554776986	0.081043067	5.74029E-06	0.949949701	Potri.011G071700.1	AT4G18880.1
Potra2n11c23183.1	-0.789496473	-0.31034072	0.750710659	NA		
Potra2n11c23184.1	0.089327109	0.104659827	0.464954489	0.93010505	Potri.011G071400.1	AT1G29340.1
Potra2n11c23185.1	-0.197458091	0.306633209	0.442872226	0.796890714	Potri.011G071300.1	AT5G45720.1
Potra2n11c23186.1	0.006119672	-0.04148044	0.970843122	0.96245401	Potri.011G071200.1	AT1G29330.1
Potra2n11c23187.1	NA	NA	NA	NA	Potri.011G071100.1	AT5G09360.1
Potra2n11c23188.1	-0.153033018	0.242949776	0.708064025	0.857189843	Potri.011G071000.1	AT1G29300.1
Potra2n11c23189.1	NA	NA	NA	NA		
Potra2n11c23190.1	0.645648108	0.370216574	0.010128601	0.788065047	Potri.011G070900.1	AT1G06410.1
Potra2n11c23191.1	5.168422643	2.295459777	0.007308395	NA	Potri.011G070500.1	AT1G29290.1
Potra2n11c23192.1	-2.975854928	-0.442232685	4.55089E-09	0.813575895	Potri.011G070400.1	
Potra2n11c23193.1	-0.37265411	-0.184375052	0.000106076	0.663037599	Potri.011G070300.1	AT5G45750.1
Potra2n11c23194.1	0.296320933	-0.282677552	0.771155436	0.966776325	Potri.011G070100.1	AT1G29280.1
Potra2n11c23195.1	-0.538386227	-0.541356988	0.400098031	0.857320245	Potri.011G070000.1	AT1G29270.1
Potra2n11c23196.1	NA	NA	NA	NA		
Potra2n11c23197.1	0.563243419	0.310634545	0.046369533	0.834560473	Potri.011G069900.1	AT2G42760.1
Potra2n11c23198.1	2.957608432	1.033328573	0.075750337	NA	Potri.011G069800.1	

Potra2n11c23199.1	-0.627832261	-0.194127759	0.007506366	0.924869625	Potri.011G069600.1	AT4G18780.1
Potra2n11c23200.1	-0.202174831	-0.169347465	0.429061365	0.894138111	Potri.011G069300.2	AT4G18740.1
Potra2n11c23201.1	0.771520818	0.152829609	4.82287E-07	0.907815163	Potri.006G181500.1	AT5G45775.2
Potra2n11c23202.1	0.670727628	-0.042182006	7.1017E-06	0.976665671	Potri.006G181500.1	AT5G45775.2
Potra2n11c23203.1	-0.945587213	-0.220916152	2.15982E-07	0.842247476	Potri.011G068800.1	AT2G34200.1
Potra2n11c23204.1	-0.239934264	-0.311132869	0.588952256	0.845379753	Potri.011G068700.3	AT5G45780.1
Potra2n11c23205.3	-0.15806789	0.104838447	0.211045835	0.880316339	Potri.011G068600.6	AT4G18710.1
Potra2n11c23206.2	-0.602588081	-0.180663169	0.015401958	0.938967511	Potri.011G068500.1	AT3G47570.1
Potra2n11c23207.1	-0.773991806	0.031022546	0.008339702	0.992934677		
Potra2n11c23208.1	-0.52208197	0.591502989	0.532891745	0.869327705		
Potra2n11c23209.1	-1.104159775	-0.363282985	5.92885E-08	0.68015285	Potri.011G068300.1	AT1G29200.2
Potra2n11c23210.1	-1.302629418	-0.852878935	0.051496692	0.676147701	Potri.011G068200.1	AT2G34190.1
Potra2n11c23211.1	0.003054081	-0.006017746	0.985511494	0.995579852	Potri.011G068100.1	AT1G29220.1
Potra2n11c23212.1	1.963228805	1.4914951	0.000497214	0.23556479	Potri.011G067900.1	AT5G45800.1
Potra2n11c23213.1	0.102729136	0.259247208	0.57327046	0.84487051	Potri.011G067600.1	AT4G18700.1
Potra2n11c23214.1	-1.59396316	-0.854773901	0.322887385	0.886265374	Potri.011G067500.1	AT5G45820.1
Potra2n11c23215.1	-1.336737103	-0.763669453	NA	NA		
Potra2n11c23216.1	-0.727024211	-0.093150945	0.000831752	0.960981446	Potri.011G067400.10	AT4G18640.1
Potra2n11c23217.1	-0.236669119	-0.183044579	0.153890873	0.844261648	Potri.011G067300.1	AT2G42670.1
Potra2n11c23218.2	-1.096478987	-0.347396856	3.48779E-05	0.821709751	Potri.011G067000.4	AT1G29240.1
Potra2n11c23219.1	0.134756865	0.230982636	0.608685112	0.853768491	Potri.011G066900.5	AT1G09850.1
Potra2n11c23220.1	4.473683132	1.143688155	7.53043E-22	0.629615756	Potri.011G066800.1	AT5G43060.1
Potra2n11c23221.1	-0.862770853	0.685425285	0.095027957	0.798429987	Potri.011G066700.1	AT2G42620.1
Potra2n11c23222.1	-0.107689097	0.125161329	0.699368447	0.935851032	Potri.004G057600.1	AT1G29260.1
Potra2n11c23223.1	-0.933112077	0.353470487	0.00015867	0.865591169	Potri.011G066500.1	AT1G29195.1
Potra2n11c23224.1	-0.481241447	0.08250139	0.000143764	0.938246513	Potri.011G066400.2	AT2G42610.2
Potra2n11c23225.1						
Potra2n11c23226.1	NA	NA	NA	NA		
Potra2n11c23227.1	NA	NA	NA	NA	Potri.011G066200.1	AT1G78830.1
Potra2n11c23228.1	-0.514998782	-0.133954237	0.006212818	0.875292974	Potri.011G066100.1	AT2G34150.2
Potra2n11c23229.1	0.600833633	0.002657557	0.000177849	0.999325699	Potri.011G066000.1	AT3G53740.4
Potra2n11c23230.1	-0.544635929	-0.43843409	0.244374459	0.848131816	Potri.011G065900.1	AT1G29160.1

Potra2n11c23231.1	-0.538440705	-0.146330416	0.000768732	0.902857376	Potri.011G065700.1	AT1G29120.1
Potra2n11c23232.1	-1.293883543	-1.03053859	0.023510249	0.5048539	Potri.011G065600.1	AT1G06330.1
Potra2n11c23233.1	1.64774763	NA	NA	NA	Potri.011G065500.1	AT2G40200.1
Potra2n11c23234.2	0.145795089	0.211896149	0.587793633	0.857189843	Potri.004G055600.1	AT5G45900.1
Potra2n11c23235.1	-0.492569595	-0.287961529	0.090398533	0.71411664	Potri.011G065300.1	AT2G27430.1
Potra2n11c23236.1	-0.021604891	0.01051037	0.839038986	0.9896961	Potri.011G065200.1	AT3G15010.1
Potra2n11c23237.1	0.199547975	0.13411811	0.249955027	0.88643982	Potri.011G065100.1	AT2G31130.1
Potra2n11c23238.3	-0.412337787	-0.006218555	0.007104258	0.996032145	Potri.011G065000.1	AT2G34090.1
Potra2n11c23239.1	0.020557555	-1.735764922	NA	NA	Potri.011G064900.1	AT5G45890.1
Potra2n11c23240.1	-0.281711224	-0.397524657	0.200018816	0.372755083	Potri.011G064800.1	AT1G29070.1
Potra2n11c23241.1						
Potra2n11c23242.1	0.076236626	0.064060737	0.961457519	0.995565508		
Potra2n11c23243.1	1.531451951	1.05494268	0.521199323	NA		
Potra2n11c23244.1	0.873346159	1.366143925	0.022945429	0.536628281	Potri.011G064700.1	AT1G29050.1
Potra2n11c23245.1	0.643569743	1.007031723	0.028705644	0.412966174	Potri.011G064600.1	AT1G29040.1
Potra2n11c23246.1	-1.206272106	-0.429631343	6.89413E-06	0.674175338	Potri.011G064500.1	AT4G18570.1
Potra2n11c23247.1						
Potra2n11c23248.1	-1.28851197	-0.087479564	1.26543E-05	0.976172519	Potri.011G064400.1	AT4G18550.1
Potra2n11c23249.1	6.799807877	1.363811008	4.91558E-07	NA	Potri.011G064300.1	AT4G18540.1
Potra2n11c23250.1	0.004143438	-0.326063552	0.997777998	0.961028365	Potri.004G054200.1	AT1G53240.1
Potra2n11c23251.1	0.20014253	-0.132652338	0.195149701	0.848421003	Potri.011G064000.1	AT5G01320.1
Potra2n11c23252.1	1.629834986	0.616116079	0.003476981	0.926234768	Potri.011G063900.1	AT2G31090.1
Potra2n11c23253.1	1.560931906	0.547112795	0.059976967	0.953108412		
Potra2n11c23254.1	-1.579922675	-0.320320764	1.02962E-21	0.607211644	Potri.011G063800.1	AT5G45920.1
Potra2n11c23255.1	1.459962178	NA	NA	NA	Potri.011G063750.1	AT2G31083.1
Potra2n11c23256.1					Potri.011G063750.1	AT2G31083.1
Potra2n11c23257.1	NA	NA	NA	NA		
Potra2n11c23258.1	1.459962178	2.60798204	NA	NA	Potri.011G063700.1	AT1G73165.1
Potra2n11c23259.1	1.547594587	1.248874	0.136858373	0.724604666	Potri.011G063500.1	AT4G18490.1
Potra2n11c23260.1	0.307413413	0.375041179	0.238499656	0.767933406	Potri.011G063400.1	AT5G45930.1
Potra2n11c23261.2	0.060674233	0.064695482	0.661884739	0.93154079	Potri.011G063300.1	AT1G28960.3
Potra2n11c23262.1	-0.90715977	-0.23219768	0.003971566	0.903137886	Potri.011G063200.1	AT2G33990.1

Potra2n11c23263.1	4.952867396	NA	0.008625942	NA	Potri.011G062900.2	AT2G29120.1
Potra2n11c23264.1	3.110343611	2.490806657	NA	NA	Potri.011G062900.2	AT2G29120.1
Potra2n11c23265.1	0.114538882	0.022857802	0.321740662	0.98177802	Potri.004G052300.1	AT2G34040.1
Potra2n11c23266.1	1.150144593	0.111949909	0.003079805	0.971437706	Potri.011G062300.1	AT2G34060.1
Potra2n11c23267.1	0.713439128	0.234702456	0.000139054	0.83404888	Potri.011G062200.1	AT4G18460.1
Potra2n11c23268.1	0.826538802	0.241874508	0.045471864	0.912777843	Potri.011G062100.1	AT4G18470.1
Potra2n11c23269.3	0.692545548	0.916421988	0.72512369	NA	Potri.011G062000.1	AT5G45960.1
Potra2n11c23270.1	6.67865818	3.654010821	8.38914E-07	0.433369183	Potri.011G061700.2	AT3G23240.1
Potra2n11c23271.1	-0.834911863	0.405947029	0.003145863	0.853030738	Potri.011G061500.1	AT5G45970.1
Potra2n11c23272.1	NA	NA	NA	NA	Potri.011G061400.1	AT2G33880.1
Potra2n11c23273.1	-1.08724433	0.884071863	0.629177801	0.900015282	Potri.011G060900.1	AT5G60860.1
Potra2n11c23274.2	-0.037078606	-0.064487078	0.853159942	0.959431187	Potri.011G060800.3	AT5G46020.1
Potra2n11c23275.1	-0.098777372	-0.029501109	0.914832288	0.996032145	Potri.011G060300.1	AT5G54010.1
Potra2n11c23276.1	0.076561594	0.023667034	0.526245115	0.98036348	Potri.011G060000.1	AT1G28520.2
Potra2n11c23277.1	0.402783307	0.22339973	0.015956707	0.860325259	Potri.011G059800.1	AT1G28510.1
Potra2n11c23278.1	-0.02520697	0.089730847	0.872636358	0.923766642	Potri.011G059700.3	AT1G28490.1
Potra2n11c23279.1	6.88918839	1.571529889	1.1453E-07	NA	Potri.011G059600.1	AT4G35160.1
Potra2n11c23280.1	-0.973316817	-0.231809319	0.001712151	0.895474078	Potri.011G059500.1	AT4G35160.1
Potra2n11c23281.4	-0.153298011	0.094412756	0.572909427	0.974275337	Potri.011G059300.1	AT2G33860.1
Potra2n11c23282.1	0.73862675	-0.046370319	NA	NA	Potri.011G058900.1	AT2G39200.1
Potra2n11c23283.2	-0.522971832	-0.506521797	0.299618366	0.881321405		
Potra2n11c23284.1	2.317410979	0.89030339	9.39025E-40	0.32068134	Potri.011G058800.1	AT1G28480.1
Potra2n11c23285.1	NA	NA	NA	NA	Potri.011G058600.1	AT3G15510.1
Potra2n11c23286.1	0.212507034	0.192535321	0.058878806	0.79314783	Potri.011G058500.2	AT5G46070.1
Potra2n11c23287.1	-0.520725297	-0.755038357	0.014716881	0.032723882	Potri.011G058400.1	AT4G28500.1
Potra2n11c23288.1	-0.347619968	-1.275029701	0.729315659	0.600720177	Potri.011G058300.1	AT5G46080.1
Potra2n11c23289.1	-1.32443457	-0.548949076	1.92769E-09	0.274284752	Potri.011G058100.1	AT1G28440.1
Potra2n11c23290.1	-1.599062884	-0.572260132	9.56089E-16	0.48231244	Potri.011G058000.1	AT4G18425.1
Potra2n11c23291.1	-0.169380383	0.000354327	0.714234031	0.999937671	Potri.011G057900.1	AT5G46100.1
Potra2n11c23292.1	-0.69755986	0.083629355	0.001153776	0.973347974	Potri.011G057800.2	AT5G46110.1
Potra2n11c23293.1	0.45207707	0.283048531	1.92101E-09	0.583548322	Potri.011G057700.1	AT1G28420.1
Potra2n11c23294.1	0.151129961	0.057824011	0.215386287	0.935474933	Potri.017G016100.1	AT2G20560.1

Potra2n11c23295.1					Potri.018G137200.1	
Potra2n11c23296.1	-1.497577112	-0.826493435	NA	NA		
Potra2n11c23297.1	-0.684266926	-0.348104658	0.008817673	0.830672839	Potri.011G057500.1	AT1G28400.1
Potra2n11c23298.2	-0.505257054	-0.295615903	0.000198791	0.643173103	Potri.011G057400.1	
Potra2n11c23299.1	-0.176421132	-0.212111945	0.243348907	0.743612955	Potri.011G057200.2	AT3G05700.1
Potra2n11c23300.1	4.853716334	2.125107129	7.6137E-11	0.124471148	Potri.011G057100.1	AT1G28380.1
Potra2n11c23301.1	2.093938335	0.061259527	1.37833E-06	0.981423914	Potri.011G057000.1	AT5G44210.1
Potra2n11c23302.1	3.373516173	-0.26331599	6.53042E-11	0.9761362	Potri.011G056900.1	AT1G28360.1
Potra2n11c23303.1	1.670091015	0.344115287	9.50446E-10	0.897285619	Potri.011G056800.1	AT1G28340.1
Potra2n11c23304.3	0.685307434	0.458776976	6.25252E-07	0.455529955	Potri.011G056600.1	AT2G33835.1
Potra2n11c23305.1	0.331667433	0.244572042	0.905795166	NA	Potri.011G056500.1	
Potra2n11c23306.1	-0.272957542	-0.013095132	0.213595453	0.995203254	Potri.011G056300.1	AT5G44280.1
Potra2n11c23307.1	0.46068657	0.207047704	1.51513E-05	0.718667387	Potri.011G056100.1	AT1G28320.1
Potra2n11c23308.1	-0.165247229	-0.202312604	0.454436556	0.834773282	Potri.011G056000.1	AT2G33820.1
Potra2n11c23309.1	-0.902042832	-0.083599732	0.460936799	0.99114586	Potri.011G055900.1	AT2G33810.1
Potra2n11c23310.1					Potri.011G052000.1	AT3G57880.1
Potra2n11c23311.1	-0.486838954	0.093064184	0.013468657	0.914321114	Potri.011G055600.1	AT1G28310.2
Potra2n11c23312.1	0.213660771	0.316701223	0.607867859	0.71411664	Potri.011G055500.2	AT1G69690.1
Potra2n11c23313.1	1.459962178	NA	NA	NA		
Potra2n11c23314.1	2.221805117	0.898119879	0.000312594	0.803948068		
Potra2n11c23315.1	2.109087265	0.795211008	0.008552861	0.885143969	Potri.011G054300.1	AT5G60850.1
Potra2n11c23316.1	-1.82041082	-0.331471029	0.204340699	0.951394085	Potri.011G054200.1	AT4G21020.1
Potra2n11c23317.1	0.062929135	0.094185343	0.895997202	0.974275337	Potri.011G054000.1	AT1G28300.1
Potra2n11c23318.1	-1.396095729	0.057372495	0.593213242	NA	Potri.004G045700.1	AT2G33800.1
Potra2n11c23319.2	-0.174835436	0.231392059	0.280541483	0.784116758	Potri.011G053800.1	AT1G28260.1
Potra2n11c23320.1	-0.612706772	-0.874942449	0.525198388	0.825276013		
Potra2n11c23321.1	0.008458189	-0.090157658	0.976262397	0.948577865	Potri.011G053700.1	AT1G28280.2
Potra2n11c23322.1	-1.218155194	-0.606156022	1.64497E-05	0.526938837	Potri.011G053600.1	AT2G33790.1
Potra2n11c23323.1	-0.174742927	-0.097304186	0.276386407	0.907073354	Potri.011G053500.1	AT4G20980.2
Potra2n11c23324.1						
Potra2n11c23325.2	2.322051582	0.672398686	4.53051E-07	0.630775933	Potri.001G063000.4	AT4G20970.1
Potra2n11c23326.1	0.827968017	2.396435764	NA	NA	Potri.011G053300.1	AT4G32480.1

Potra2n11c23327.1	-0.69204917	-0.343374255	9.33437E-05	0.830056077	Potri.011G053100.1	AT4G04960.1
Potra2n11c23328.1	-0.159353972	0.48680597	0.444965958	0.677460178	Potri.011G053000.1	AT1G28240.1
Potra2n11c23329.1	0.294305183	0.13209981	0.07572777	0.853030738	Potri.011G052800.2	AT4G04910.1
Potra2n11c23330.1	0.946203603	0.603902345	1.40048E-05	0.540010302	Potri.011G052700.1	AT3G58610.3
Potra2n11c23331.1	-0.346462878	-0.076560176	0.026652729	0.960670343	Potri.011G052600.1	AT2G33770.1
Potra2n11c23332.1	-0.138215845	0.000855427	0.515433	0.999771727		
Potra2n11c23333.1	-0.107679095	0.099555454	0.617652043	0.875559093	Potri.011G052400.1	AT4G04970.1
Potra2n11c23334.1	0.344810659	-0.267570599	0.14044801	0.843683654	Potri.011G052300.1	AT2G33760.1
Potra2n11c23335.1	1.459962178	NA	NA	NA		
Potra2n11c23336.1	0.559829816	0.207183631	0.06349761	0.917751882	Potri.011G052200.1	AT5G22620.2
Potra2n11c23337.1	1.341002201	-2.799286845	0.553983731	NA	Potri.011G052100.1	AT1G03700.1
Potra2n11c23338.1	0.097167313	-0.647754327	0.888911601	0.830470159	Potri.011G052000.1	AT3G57880.1
Potra2n11c23339.2	-0.525213773	-0.608675439	0.361273874	0.716548554	Potri.004G042800.1	AT2G33793.1
Potra2n11c23340.1	0.044071416	-0.02895689	0.668785127	0.976888173	Potri.011G051800.1	AT4G03510.1
Potra2n11c23341.1	5.669535575	3.320235483	0.000212016	NA	Potri.011G051600.1	AT5G16820.2
Potra2n11c23342.1	0.202343504	-0.165165738	0.22213484	0.859892315	Potri.011G051500.1	AT4G21705.1
Potra2n11c23343.1	0.190216601	-0.153086966	0.275962228	0.844261648	Potri.011G051400.1	AT4G04950.1
Potra2n11c23344.1	0.696332756	0.294071328	4.10129E-08	0.595667201	Potri.011G051300.1	AT3G15390.1
Potra2n11c23345.1					Potri.011G051000.1	
Potra2n11c23346.1	0.300600515	-0.046615922	0.662632773	0.992314551	Potri.011G050900.2	AT2G20740.1
Potra2n11c23347.1	-0.994829577	-0.257527423	0.000307891	0.874808359	Potri.011G050800.1	AT4G04930.1
Potra2n11c23348.1	-0.116501377	0.111465817	0.725544247	0.917746944	Potri.011G050700.1	AT3G47160.1
Potra2n11c23349.1	0.118318308	0.127881938	0.488288044	0.865611082	Potri.011G050600.1	AT5G41950.1
Potra2n11c23350.1	-0.156693396	-0.216051926	0.545611576	0.901195443	Potri.011G050500.1	AT5G53750.1
Potra2n11c23351.3	-0.021668248	-0.027829019	0.898284564	0.981702181	Potri.011G050400.1	AT1G60900.1
Potra2n11c23352.1	3.237405663	0.355495143	0.051893545	NA	Potri.011G050000.1	AT2G04160.1
Potra2n11c23353.1	5.052545818	0.674963689	0.026446243	NA	Potri.011G050000.1	AT2G04160.1
Potra2n11c23354.1	3.718761751	0.469124698	0.036236769	NA	Potri.011G050000.1	AT2G04160.1
Potra2n11c23355.1	0.264015699	1.869036058	0.785333403	0.711204954	Potri.011G049900.1	AT4G27450.1
Potra2n11c23356.1	-0.517156798	-0.554801586	0.050993938	0.417133103	Potri.004G040500.1	AT2G47960.1
Potra2n11c23357.1	0.230761909	-0.021355573	0.149084404	0.983944804	Potri.011G049700.2	AT4G04920.2
Potra2n11c23358.1	-0.143833237	-0.066941948	0.238106301	0.948577865	Potri.011G049600.1	AT3G09010.1

Potra2n11c23359.1	-0.109651483	-0.072631174	0.443048855	0.953608209	Potri.011G049500.1	AT2G20760.1
Potra2n11c23360.1	-0.183696413	0.124768035	0.111503276	0.87679119	Potri.011G049400.1	AT4G21670.1
Potra2n11c23361.1	0.004882685	0.120779125	0.973860713	0.884463725	Potri.011G049300.1	AT1G11060.1
Potra2n11c23362.1	-3.343043275	-1.825442313	0.001737455	0.5048539	Potri.011G048200.1	AT1G18590.1
Potra2n11c23363.1	NA	NA	NA	NA	Potri.003G076000.1	AT4G25200.1
Potra2n11c23364.1	-0.099766055	-1.822689043	0.948176003	0.70570456	Potri.011G048200.1	AT1G18590.1
Potra2n11c23365.3	0.347703646	0.189461452	0.004996374	0.788065047	Potri.011G048000.1	AT3G51000.1
Potra2n11c23366.1	-0.047706969	0.074243806	0.677819273	0.93125845	Potri.011G047900.1	AT1G61040.1
Potra2n11c23367.1	-2.428879984	-1.468068323	0.002025259	0.477594648	Potri.011G047800.1	AT1G79760.1
Potra2n11c23368.1	-0.455598335	-0.31599567	0.147291921	0.799868271	Potri.011G047700.1	AT1G61050.1
Potra2n11c23369.1	-0.122700266	-0.209535225	0.813360459	0.939915707		
Potra2n11c23370.1	-0.240254691	0.005109245	0.014169804	0.995579852	Potri.011G047600.1	AT4G21660.1
Potra2n11c23371.1	1.069353139	-0.681987738	0.096483882	0.834823243	Potri.011G047500.1	AT5G60850.1
Potra2n11c23372.1	-0.003943818	0.252520469	0.992479698	0.857357345	Potri.011G047400.1	AT1G61065.1
Potra2n11c23373.1	10.71574855	6.29842149	1.28121E-17	NA	Potri.011G047300.1	
Potra2n11c23374.1	0.378991064	-0.026337312	0.381577267	0.995581218	Potri.011G047200.1	
Potra2n11c23375.1	2.080346469	0.481991785	1.03689E-08	0.833952007	Potri.011G047100.2	AT1G11070.1
Potra2n11c23376.1	NA	NA	NA	NA		
Potra2n11c23377.1	0.700045457	0.252704426	0.394943368	0.902847514	Potri.011G046900.1	AT1G61100.2
Potra2n11c23378.1	1.545832463	NA	NA	NA	Potri.011G046800.1	AT1G61105.1
Potra2n11c23379.1	-0.927031396	1.922273136	NA	NA	Potri.011G046700.1	AT1G61110.1
Potra2n11c23380.1	-1.753693737	0.290998127	9.62978E-09	0.882699919	Potri.011G046600.1	AT1G11080.2
Potra2n11c23381.1	0.730571618	0.263517935	4.36526E-05	0.233903684	Potri.011G046400.1	AT1G61140.1
Potra2n11c23382.1	-0.177290541	0.166101365	0.396207175	0.767932972	Potri.011G046500.1	AT1G11090.1
Potra2n11c23383.1	-1.196065379	0.121933897	0.267632241	0.985393296	Potri.011G044000.1	AT1G61260.1
Potra2n11c23384.1	NA	NA	NA	NA	Potri.011G043700.1	AT1G61290.1
Potra2n11c23385.1	-0.772797375	-0.402695985	9.35336E-08	0.662187972	Potri.011G043600.1	AT4G32010.1
Potra2n11c23386.1	-0.018136395	0.09250045	0.883380107	0.921099436	Potri.011G043500.1	AT4G21540.1
Potra2n11c23387.1	-0.126463701	-0.083925004	0.437317785	0.923519834	Potri.011G043400.1	AT1G11240.1
Potra2n11c23388.1	0.477531453	0.199396948	2.42639E-06	0.788348835	Potri.011G043300.1	AT5G57580.1
Potra2n11c23389.1	1.448711311	0.264653171	1.41365E-15	0.879379661	Potri.011G043100.1	AT1G30420.1
Potra2n11c23390.1	0.334842669	-0.069221566	0.20049424	0.977559521	Potri.011G043000.1	AT4G21520.1

Potra2n11c23391.1	-0.14888572	0.951741349	0.85488664	0.688448032	Potri.011G042900.1	AT1G61330.1
Potra2n11c23392.1	0.001238584	-0.417395631	0.997734801	0.670268314	Potri.011G042800.1	AT1G61340.1
Potra2n11c23393.1	-1.347406701	-2.009944876	0.006098417	9.12973E-05	Potri.011G042600.1	AT4G21500.1
Potra2n11c23394.1	0.152064302	0.258554899	0.689312598	0.902857376	Potri.011G042500.1	AT4G05020.1
Potra2n11c23395.1	-0.843987639	-0.150699639	2.19785E-07	0.756757704	Potri.011G042400.1	AT3G62980.1
Potra2n11c23396.1	-0.862920153	-0.288449413	7.75767E-05	0.579724936		
Potra2n11c23397.1	0.573853361	2.125375534	0.527053354	0.207211174	Potri.011G042300.1	AT1G61350.1
Potra2n11c23398.1	3.197659465	3.146104274	0.099822285	0.4602924	Potri.011G042100.1	AT1G11260.1
Potra2n11c23399.1	-2.577269065	-0.351520279	0.41696435	NA	Potri.011G042000.1	
Potra2n11c23400.1	0.489244407	0.056123418	0.002286789	0.93154079	Potri.011G041900.1	AT4G21450.1
Potra2n11c23401.1	NA	NA	NA	NA	Potri.011G041800.1	AT4G21445.1
Potra2n11c23402.1	-0.869643417	0.882016546	0.349480573	0.879437792	Potri.011G041700.2	AT4G05070.1
Potra2n11c23403.1	0.92641307	1.955029706	NA	NA	Potri.011G041600.1	AT4G21440.1
Potra2n11c23404.1	-0.044122808	-0.017007588	0.736698154	0.985016122	Potri.011G041500.1	AT1G11390.1
Potra2n11c23405.1	-0.299700789	0.468655985	0.082274191	0.759797728	Potri.011G041400.1	AT4G21430.1
Potra2n11c23406.1	-0.429935741	0.192173735	0.421116508	0.956889095		
Potra2n11c23407.1	-0.013790304	0.370863702	0.986469562	0.912295985	Potri.004G032300.1	AT4G05120.1
Potra2n11c23408.1	-1.852469434	-0.350412317	4.97958E-25	0.498403695	Potri.011G041100.1	AT4G05150.1
Potra2n11c23409.1	-0.828337496	-0.770366698	0.210938237	0.813731863	Potri.011G040900.1	AT4G03270.1
Potra2n11c23410.1	4.505500088	2.708619563	4.95045E-32	0.00435868	Potri.011G040800.1	AT5G45490.2
Potra2n11c23411.1	1.186188552	0.577756827	4.36724E-11	0.206141768		
Potra2n11c23412.1	0.92641307	-0.046370319	NA	NA	Potri.011G040500.1	AT4G03270.1
Potra2n11c23413.1	1.545832463	NA	NA	NA	Potri.011G040400.1	AT2G31180.1
Potra2n11c23414.1	NA	NA	NA	NA	Potri.011G040300.1	AT4G38620.1
Potra2n11c23415.1	1.459962178	NA	NA	NA	Potri.T144800.1	AT4G38620.1
Potra2n11c23416.1	-1.478142584	-0.184998987	7.67445E-08	0.9045874	Potri.011G040100.1	AT1G11380.1
Potra2n11c23417.1	-0.118161679	-0.060723183	0.338940203	0.936170918	Potri.011G040000.1	AT1G61620.1
Potra2n11c23418.1	-0.324438063	0.181750915	0.005361355	0.883788696	Potri.011G039800.2	AT1G11360.1
Potra2n11c23419.1	1.259049913	-0.528921099	0.008231078	0.833104983		
Potra2n11c23420.1	0.844000664	-0.227052716	0.001874509	0.895129305	Potri.011G039700.1	AT4G27310.1
Potra2n11c23421.1	2.024424109	0.110081123	0.565437769	NA	Potri.011G039500.1	AT4G27280.1
Potra2n11c23422.2	2.556188936	1.142800052	9.60072E-05	0.443657809	Potri.011G039300.1	AT1G11330.1

Potra2n11c23423.1	2.125312886	NA	NA	NA	Potri.011G039000.1	AT1G11330.1
Potra2n11c23424.1	0.190602987	-0.222567051	0.377248845	0.829862776	Potri.011G037100.1	AT4G21380.1
Potra2n11c23425.1	-4.46466661	-3.054249205	0.178446973	NA	Potri.004G028100.1	AT2G32235.1
Potra2n11c23426.1	0.380708737	0.145540301	0.040050364	0.913459614	Potri.011G035200.1	AT1G11340.1
Potra2n11c23427.1	0.07098819	-0.340894189	0.804734077	0.792511865		
Potra2n11c23428.1	1.521789543	0.520708167	3.18444E-05	0.71411664	Potri.011G036600.1	AT1G11340.1
Potra2n11c23429.2	0.787477951	0.366815815	2.41506E-13	0.32906562	Potri.011G035200.1	AT1G11340.1
Potra2n11c23430.1	0.245214068	0.301875542	0.452710435	0.883216393		
Potra2n11c23431.1	-0.881469294	-0.146013576	0.004381709	0.919740601	Potri.011G036900.1	AT4G27290.1
Potra2n11c23432.1	-0.046500011	0.35411292	0.829720025	0.660319089	Potri.011G034766.1	AT1G11340.1
Potra2n11c23433.1	0.113035048	0.367246434	0.682033938	0.814405987	Potri.011G034400.1	AT1G11340.1
Potra2n11c23434.1	-0.562693146	0.209966709	0.001431791	0.935474933	Potri.011G034900.1	AT1G11340.1
Potra2n11c23435.1	0.256692689	0.37884339	0.237195486	0.792864576	Potri.011G034300.1	AT1G11340.1
Potra2n11c23436.1	-1.104789901	-1.28354091	0.178033461	0.586883316	Potri.001G421000.1	AT5G54850.1
Potra2n11c23437.1	1.535924563	-0.713382849	0.468873066	NA	Potri.001G421100.1	AT4G21780.1
Potra2n11c23438.1	-0.80727642	-0.064528955	4.28447E-06	0.9761362	Potri.011G034400.1	AT1G11340.1
Potra2n11c23439.1	-0.536908455	0.029532898	0.0103526	0.989329144	Potri.011G034900.1	AT1G11340.1
Potra2n11c23440.1	0.537058979	0.376290846	0.023600282	0.724778905	Potri.011G036100.1	AT1G11340.1
Potra2n11c23441.1	-0.801906679	-0.051444624	0.029081122	0.987226805	Potri.011G033400.1	AT4G21380.1
Potra2n11c23442.1	-0.203940608	0.343122873	0.38352348	0.839016145	Potri.011G033300.1	AT4G21350.1
Potra2n11c23443.1	0.775948382	-0.242921956	1.76416E-05	0.821704644	Potri.011G033100.1	AT4G27270.1
Potra2n11c23444.1	-0.36228945	-0.075343397	0.335491464	0.957525726	Potri.011G033000.1	AT1G61660.1
Potra2n11c23445.1	-0.409102654	-0.182902606	0.000218544	0.562306981	Potri.011G032900.1	AT1G11430.1
Potra2n11c23446.1	NA	0.674963689	NA	NA	Potri.011G032800.1	AT2G22590.1
Potra2n11c23447.1	-0.576899203	-0.207993669	0.013295331	0.857042516		
Potra2n11c23448.1	-0.492012332	-0.183480734	0.028293169	0.859892315		
Potra2n11c23449.1	-0.460451716	-0.15192834	0.009438313	0.885649686		
Potra2n11c23450.1	-1.378779378	-0.35443689	2.54707E-09	0.821709751	Potri.011G032600.1	AT1G61667.1
Potra2n11c23451.3	-0.066793128	-0.201811906	0.788982504	0.843443324	Potri.011G032500.1	AT3G13160.1
Potra2n11c23452.2	0.224718037	-0.217033518	0.481198814	0.883381753	Potri.011G032400.1	AT3G13160.1
Potra2n11c23453.1	-0.21918769	-0.413410222	0.46863479	0.779493734	Potri.T178500.1	AT3G13160.1
Potra2n11c23454.1	3.091622206	0.906933686	0.00577756	NA	Potri.011G032300.1	AT1G61680.1

Potra2n11c23455.1	0.52019134	0.302615189	1.00434E-07	0.482012772	Potri.011G032200.1	AT1G61690.1
Potra2n11c23456.1	-1.07735429	-2.596635878	0.713427838	NA	Potri.011G032100.1	AT3G22104.1
Potra2n11c23457.1	1.459962178	NA	NA	NA	Potri.011G032000.1	
Potra2n11c23458.1	0.090308954	0.095797568	0.539649116	0.922778027	Potri.011G031900.1	AT1G11480.1
Potra2n11c23459.2	2.632524198	4.081935946	NA	NA	Potri.011G031800.2	AT3G25820.1
Potra2n11c23460.1	0.149035162	0.460867101	0.598109618	0.684244573	Potri.011G031600.1	AT4G21320.1
Potra2n11c23461.1	0.148660671	-0.016847705	0.619242116	0.996032145	Potri.011G031500.1	AT4G21310.1
Potra2n11c23462.1	-0.301729662	-0.284925061	0.194151421	0.794641467	Potri.011G031300.1	AT4G05180.1
Potra2n11c23463.1	0.671233471	-0.189900159	0.030992888	0.916006053	Potri.011G031200.1	AT4G21270.1
Potra2n11c23464.2	-0.844727838	-0.665413562	0.634155593	0.924951347	Potri.004G031700.1	AT1G11530.1
Potra2n11c23465.1	-0.187631051	-0.081004324	0.365513312	0.960981446	Potri.011G031000.1	AT5G54680.1
Potra2n11c23466.1	0.738345907	0.401912345	0.00031853	0.775519829	Potri.011G030900.3	AT1G61740.1
Potra2n11c23467.1	2.632524198	NA	NA	NA	Potri.011G030500.1	AT5G48540.1
Potra2n11c23468.1					Potri.011G030500.1	AT5G48540.1
Potra2n11c23469.1	NA	NA	NA	NA	Potri.011G029100.1	AT4G23220.1
Potra2n11c23470.3	7.167811495	1.571529889	1.21904E-08	NA	Potri.011G029000.1	AT4G21410.1
Potra2n11c23471.1						
Potra2n11c23472.1					Potri.011G028700.1	AT4G38830.1
Potra2n11c23473.1	NA	NA	NA	NA		
Potra2n11c23474.1	3.935637317	NA	0.183309281	NA	Potri.011G030000.2	AT4G21410.1
Potra2n11c23475.1	0.82357019	NA	NA	NA	Potri.011G028800.1	AT4G21410.1
Potra2n11c23476.1					Potri.011G028700.1	AT4G38830.1
Potra2n11c23477.1					Potri.011G028700.1	AT4G38830.1
Potra2n11c23478.1					Potri.011G028700.1	AT4G38830.1
Potra2n11c23479.1	7.780890148	4.362055296	7.14542E-12	0.50924822	Potri.011G028600.1	AT4G21410.1
Potra2n11c23480.1	7.324414735	3.65203283	8.0391E-15	0.517743314	Potri.011G028600.1	AT4G21410.1
Potra2n11c23481.1	4.101927257	-1.507841271	0.015309664	NA	Potri.011G030000.2	AT4G21410.1
Potra2n11c23482.1	2.375226855	NA	NA	NA	Potri.011G028800.1	AT4G21410.1
Potra2n11c23483.1						
Potra2n11c23484.1	4.452322155	NA	0.029409426	NA		
Potra2n11c23485.1	1.545832463	NA	NA	NA	Potri.011G028700.1	AT4G38830.1
Potra2n11c23486.1	2.196235887	NA	NA	NA	Potri.011G028700.1	AT4G38830.1

Potra2n11c23487.2	8.456886606	4.705656739	1.02291E-31	0.595538573	Potri.011G028600.1	AT4G21410.1
Potra2n11c23488.1	9.675764111	6.093589276	1.37046E-48	0.072307355	Potri.011G028500.1	AT4G05200.1
Potra2n11c23489.1	NA	NA	NA	NA		
Potra2n11c23490.1	NA	NA	NA	NA	Potri.004G026100.1	AT4G23180.1
Potra2n11c23491.1						
Potra2n11c23492.1					Potri.004G012600.1	AT4G23180.1
Potra2n11c23493.3	1.272454275	0.75635119	5.59156E-06	0.210620025	Potri.011G028300.1	AT4G05200.1
Potra2n11c23494.1					Potri.T128400.1	AT4G05200.1
Potra2n11c23495.1	0.023943348	-0.088831462	0.939555423	0.972402328	Potri.T128400.1	AT4G05200.1
Potra2n11c23496.1	2.492428534	NA	NA	NA	Potri.T128600.2	AT4G05200.1
Potra2n11c23497.1	3.302003742	0.57320632	0.017003333	NA	Potri.011G029100.1	AT4G23220.1
Potra2n11c23498.1	-2.0942565	-2.330905847	0.536934919	NA		
Potra2n11c23499.1					Potri.T038300.2	AT5G36930.2
Potra2n11c23500.1	2.632524198	NA	NA	NA	Potri.011G024900.3	AT5G28237.1
Potra2n11c23501.4	-0.263257584	-0.057083781	0.020717477	0.937146435	Potri.011G024800.3	AT4G04885.1
Potra2n11c23502.1	1.120436621	0.474088562	1.70421E-07	0.694611009	Potri.011G024700.1	AT4G04640.1
Potra2n11c23503.1	0.891149838	0.310820574	0.009032797	0.897285619	Potri.011G024600.1	AT3G51530.1
Potra2n11c23504.1	0.396121197	-0.035089392	0.021945479	0.979719961	Potri.011G024500.2	AT1G68680.1
Potra2n11c23505.1	-0.326192917	-0.700979802	0.868640339	NA	Potri.011G024300.1	AT3G49030.1
Potra2n11c23506.1	-0.413987229	0.125802391	0.236417823	0.957525726	Potri.011G024200.10	AT4G26340.1
Potra2n11c23507.1	-0.143968556	-0.011231179	0.316924886	0.993936896	Potri.011G024100.1	AT4G16330.2
Potra2n11c23508.1	-0.020209619	-0.050694012	0.900466547	0.954559968	Potri.011G024000.1	AT4G16340.1
Potra2n11c23509.1	0.892131386	0.33060081	4.01639E-07	0.742764478	Potri.011G023900.2	AT1G03475.1
Potra2n11c23510.1	0.842005589	0.296005767	2.08352E-07	0.805461106		
Potra2n11c23511.2	0.00462565	0.019078317	0.980577352	0.99114586	Potri.011G023801.1	AT1G24290.1
Potra2n11c23512.1	-1.564596165	-0.309711203	1.87066E-11	0.553966993	Potri.004G000600.1	AT5G60870.1
Potra2n11c23513.1	-1.576406375	-0.588985895	4.1666E-09	0.13137548	Potri.011G023600.1	AT1G79430.2
Potra2n11c23514.1	0.654848213	0.7921527	0.627052725	0.931774514	Potri.011G023500.1	AT3G17640.1
Potra2n11c23515.1	0.110581149	-1.100310053	0.974419268	NA		
Potra2n11c23516.1	0.326732655	0.153638277	0.055807972	0.862432993	Potri.T157700.1	AT4G22330.1
Potra2n11c23517.1	NA	NA	NA	NA	Potri.T157800.1	AT1G12030.1
Potra2n11c23518.1	0.171730261	-0.009218995	0.121528166	0.992934677	Potri.011G023200.1	AT4G22320.2

Potra2n11c23519.2	0.192865564	0.022088946	0.065091522	0.98036348	Potri.011G023100.2	AT4G22310.1
Potra2n11c23520.1	0.518013749	0.058449968	0.00117527	0.972159498	Potri.011G023000.1	AT4G22300.1
Potra2n11c23521.1	-0.962498768	-0.06147618	1.58771E-05	0.978646026	Potri.011G022800.1	AT1G62400.1
Potra2n11c23522.1	-0.61910233	-0.767708831	NA	NA		
Potra2n11c23523.1	-0.37329534	0.110256999	0.01823748	0.906107926	Potri.011G022600.1	AT4G22290.1
Potra2n11c23524.1	0.204205643	0.007790429	0.152316719	0.995579852	Potri.011G022500.1	AT4G05530.1
Potra2n11c23525.4	-0.066727369	-0.256735786	0.782578631	0.563998615	Potri.011G022300.2	AT4G05520.2
Potra2n11c23526.1	1.459962178	NA	NA	NA	Potri.011G022200.1	AT3G51590.1
Potra2n11c23527.2	-0.499584179	-0.187027948	0.651523522	0.977227089	Potri.011G022000.3	AT3G25070.1
Potra2n11c23528.1	NA	NA	NA	NA	Potri.019G059700.1	AT2G40770.1
Potra2n11c23529.1	-1.008952537	-1.057407554	0.7903321	NA	Potri.011G021900.2	AT3G51590.1
Potra2n11c23530.1	0.596013411	0.121346056	0.000542274	0.930598202	Potri.011G021800.1	AT4G22260.1
Potra2n11c23531.1	-1.747519038	0.304779052	0.000670123	0.921063801	Potri.004G002700.1	AT2G32950.1
Potra2n11c23532.1	NA	NA	NA	NA	Potri.011G021500.2	AT5G25910.1
Potra2n11c23533.1	0.00970777	-0.484773079	0.996689854	NA		
Potra2n11c23534.1	-1.389284009	-0.611345925	0.015449025	0.736149483	Potri.011G021400.2	AT3G11080.1
Potra2n11c23535.1	-0.048725452	0.225447137	0.771847901	0.880316339	Potri.011G021100.1	AT4G03960.1
Potra2n11c23536.1	-0.024199102	-0.075899111	0.885208399	0.927547456	Potri.011G021000.1	AT1G62390.1
Potra2n11c23537.1	NA	NA	NA	NA	Potri.011G019200.2	AT3G18670.1
Potra2n11c23538.1	0.102236182	0.8502037	NA	NA	Potri.011G019200.2	AT3G18670.1
Potra2n11c23539.1	-2.754058864	-1.305173887	NA	NA	Potri.011G019200.2	AT3G18670.1
Potra2n11c23540.1	NA	NA	NA	NA	Potri.011G020250.1	AT3G18670.1
Potra2n11c23541.1	1.206870348	0.259625759	0.466868649	0.977559521	Potri.011G019200.2	AT3G18670.1
Potra2n11c23542.1	1.459962178	NA	NA	NA		
Potra2n11c23543.1	NA	NA	NA	NA	Potri.011G019200.2	AT3G18670.1
Potra2n11c23544.1	0.53596802	-2.951075327	0.675363779	NA	Potri.011G019750.1	AT3G54070.1
Potra2n11c23545.1					Potri.011G020500.1	AT3G54070.1
Potra2n11c23546.1	2.030909952	1.571529889	NA	NA	Potri.011G020250.1	AT3G18670.1
Potra2n11c23547.1	4.357772509	3.010689165	0.086477293	NA	Potri.011G019200.2	AT3G18670.1
Potra2n11c23548.1	NA	NA	NA	NA	Potri.011G020250.1	AT3G18670.1
Potra2n11c23549.1	NA	NA	NA	NA		
Potra2n11c23550.1	-0.683649508	-1.084635497	0.857576477	NA	Potri.011G016400.1	AT3G54070.1

Potra2n11c23551.1	1.545832463	1.571529889	NA	NA	Potri.001G155750.1	AT1G21280.1
Potra2n11c23552.1	1.459962178	NA	NA	NA		
Potra2n11c23553.1	1.459962178	1.571529889	NA	NA	Potri.008G022500.1	
Potra2n11c23554.1	NA	NA	NA	NA	Potri.008G022500.1	
Potra2n11c23555.1	2.340207562	NA	NA	NA		
Potra2n11c23556.1	-0.65902361	-0.461935791	0.01672219	0.737406921	Potri.011G016200.1	AT3G18670.1
Potra2n11c23557.1	2.735991243	NA	NA	NA	Potri.011G019000.1	AT3G54070.1
Potra2n11c23558.1	2.282243673	-0.412460505	0.094924819	NA	Potri.011G017550.1	AT3G54070.1
Potra2n11c23559.1					Potri.011G018550.1	AT3G54070.1
Potra2n11c23560.1	NA	NA	NA	NA		
Potra2n11c23561.1	0.82357019	NA	NA	NA	Potri.011G016400.1	AT3G54070.1
Potra2n11c23562.1	-0.845064928	-0.426609075	0.000787157	0.767368598	Potri.011G016200.1	AT3G18670.1
Potra2n11c23563.1	-0.468430807	-0.08584559	0.059900397	0.974275337	Potri.011G015900.1	AT1G12000.1
Potra2n11c23564.1						
Potra2n11c23565.1	1.369350324	-2.032537384	0.574647746	NA	Potri.006G270000.1	AT5G36930.2
Potra2n11c23566.1	2.853057733	NA	NA	NA	Potri.T069100.1	AT5G36930.2
Potra2n11c23567.1	-0.297660459	0.891783545	0.800874614	0.775830532		
Potra2n11c23568.1	1.233470932	0.492015202	2.87717E-05	0.759797728	Potri.011G012000.1	AT5G36930.2
Potra2n11c23569.2	1.090433842	0.57156391	1.74305E-10	0.199636221	Potri.011G011600.2	AT1G27170.1
Potra2n11c23570.1	-0.263898242	-0.169148877	0.061907928	0.843443324	Potri.006G270200.1	AT4G22160.2
Potra2n11c23571.1	-0.411662157	-0.167291329	2.74452E-05	0.694477853	Potri.011G011300.1	AT4G04210.1
Potra2n11c23572.1	-1.943759112	-0.444898162	9.00765E-14	0.478429061	Potri.011G011200.1	
Potra2n11c23573.1	-1.126452962	-0.21568851	1.09425E-11	0.673131445	Potri.011G011100.1	AT1G62360.1
Potra2n11c23574.1	1.048678312	0.291626049	2.35196E-07	0.596355475	Potri.011G012000.1	AT5G36930.2
Potra2n11c23575.2	0.077208357	0.00825881	0.764237465	0.996032145	Potri.011G011000.1	AT2G20060.1
Potra2n11c23576.1	-0.193380675	0.014778675	0.064530333	0.988601759	Potri.011G010800.1	AT4G22140.2
Potra2n11c23577.1	-0.275692402	-0.01979875	0.296546266	0.991147673	Potri.011G010700.3	AT1G62340.1
Potra2n11c23578.1	-0.115111406	0.034464432	0.61983939	0.982422288	Potri.011G010600.1	AT1G62330.1
Potra2n11c23579.1	0.4223585	-0.16778837	0.145848753	0.944323367	Potri.011G010500.1	AT4G22130.1
Potra2n11c23580.3	-0.903358132	-0.307459416	0.030713197	0.881090429	Potri.011G010400.1	AT4G04320.1
Potra2n11c23581.1	-1.112750014	-0.355390409	0.003112258	0.824731243	Potri.011G010300.1	AT4G04330.1
Potra2n11c23582.1	0.786193138	0.423320125	0.01146041	0.869186333	Potri.011G010200.1	AT5G63520.1

Potra2n11c23583.1	1.459190298	0.759491699	0.124799977	0.850863374	Potri.011G010200.1	AT5G63520.1
Potra2n11c23584.2	-0.216961689	-0.128173456	0.82813194	0.921099436	Potri.011G009900.1	AT4G22120.4
Potra2n11c23585.3	0.398287081	0.203800115	2.47631E-05	0.709110307	Potri.011G009700.3	AT1G62310.1
Potra2n11c23586.1	-0.202937801	-0.021288791	0.341987238	0.990672454	Potri.011G009600.2	AT4G04350.1
Potra2n11c23587.1	-0.21638904	-0.082376126	0.049694272	0.91022164	Potri.011G009500.1	AT1G11940.1
Potra2n11c23588.1	-0.328477245	0.153424694	0.315340673	0.907073354	Potri.T037800.2	AT5G36930.2
Potra2n11c23589.1	-0.842503228	-0.62119353	0.743244422	NA		
Potra2n11c23590.3	3.968107254	2.530675949	0.047458698	NA	Potri.T037601.1	AT1G27170.1
Potra2n11c23591.1	-0.436566018	0.024266332	0.312994992	0.993589787	Potri.T039300.1	AT1G27170.1
Potra2n11c23592.1	1.664677056	0.674963689	NA	NA	Potri.011G009200.1	AT1G05070.1
Potra2n11c23593.1	0.071312288	-0.267624314	0.786215277	0.777168467	Potri.T039450.1	AT1G27180.1
Potra2n11c23594.1	4.601121408	2.676364087	0.015689732	NA	Potri.011G012401.1	AT5G40100.1
Potra2n11c23595.2	0.096756375	0.341623568	0.694116184	0.727745332	Potri.T039300.1	AT1G27170.1
Potra2n11c23596.1	1.64774763	NA	NA	NA	Potri.T166500.1	
Potra2n11c23597.1	2.44324887	NA	NA	NA		
Potra2n11c23598.2	0.337647603	0.12725124	0.242053388	0.937846405	Potri.T039450.1	AT1G27180.1
Potra2n11c23599.1					Potri.018G074600.2	AT1G03670.1
Potra2n11c23600.1	NA	1.571529889	NA	NA	Potri.001G155750.1	AT1G21280.1
Potra2n11c23601.1	0.419390405	-0.118709963	0.49848524	0.976302215	Potri.T038600.1	AT5G36930.2
Potra2n11c23602.1	1.000995104	-0.160122029	0.186318355	0.979775311	Potri.T038650.1	
Potra2n11c23603.1	0.984849108	-0.160122029	0.196332802	0.979775311	Potri.T068300.1	
Potra2n11c23604.1	0.586138031	-0.869188953	0.756337299	NA	Potri.011G014300.1	AT4G04670.1
Potra2n11c23605.1	0.597924558	0.037249505	0.495793048	0.996032145		
Potra2n11c23606.1	0.282492534	-0.068552079	0.380371228	0.976324121	Potri.011G008800.1	AT1G27170.1
Potra2n11c23607.1	0.962783847	1.225906779	0.018114299	0.028754802	Potri.011G060600.1	AT1G27170.1
Potra2n11c23608.1	0.232195587	0.343691563	0.767826975	0.910815461	Potri.T074866.1	AT1G27180.1
Potra2n11c23609.1	-3.619321934	-0.381032148	0.377947018	NA	Potri.T037601.1	AT1G27170.1
Potra2n11c23610.1	1.64774763	3.01657805	NA	NA	Potri.T038700.1	
Potra2n11c23611.1	2.032494738	0.151993677	0.459686411	NA	Potri.011G008100.1	AT4G22080.1
Potra2n11c23612.1	1.518874079	0.205491962	0.576753485	NA	Potri.011G008000.1	AT4G22080.1
Potra2n11c23613.1	-1.743594378	-0.451163631	5.55685E-08	0.68704882	Potri.011G007900.1	AT1G11915.1
Potra2n11c23614.1	5.437005629	2.485810044	4.45533E-35	0.016457585	Potri.011G007800.1	AT1G62300.1

Potra2n11c23615.2	-1.507046709	-0.192947801	1.16671E-05	0.903934429	Potri.011G007600.5	AT1G11910.1
Potra2n11c23616.1	-0.887859161	-0.448934764	0.109072276	0.637618802	Potri.011G007400.1	AT5G55090.1
Potra2n11c23617.1	-0.365650001	-0.163213563	0.110239355	0.922011871	Potri.011G007200.1	AT5G42570.1
Potra2n11c23618.1	0.063542947	-0.226243112	0.740973109	0.846680547	Potri.011G007100.1	AT4G04470.1
Potra2n11c23619.1	NA	NA	NA	NA		
Potra2n11c23620.1	NA	NA	NA	NA	Potri.011G006900.1	AT4G22030.1
Potra2n11c23621.1	-2.878822174	-2.308489934	NA	NA	Potri.011G007000.1	AT4G22030.1
Potra2n11c23622.1	0.329789087	0.197215758	0.013644157	0.841487301	Potri.011G006800.1	AT1G11860.3
Potra2n11c23623.2	0.183912933	0.009041645	0.427941301	0.995579852	Potri.011G006700.2	AT1G62250.1
Potra2n11c23624.1	-4.306083341	-1.291888046	0.082356572	NA	Potri.011G006350.1	AT2G02060.1
Potra2n11c23625.1	-0.61910233	0.64247635	NA	NA	Potri.011G006200.1	AT1G09910.1
Potra2n11c23626.1	0.958204654	0.189192429	5.53283E-08	0.907073354	Potri.011G006100.1	AT1G11820.2
Potra2n11c23627.1	0.82357019	NA	NA	NA	Potri.T117200.1	
Potra2n11c23628.1					Potri.011G005800.1	
Potra2n11c23629.1	3.362250609	1.571529889	NA	NA	Potri.011G005601.1	
Potra2n11c23630.1	2.610910166	1.571529889	NA	NA		
Potra2n11c23631.1	1.025785189	0.88991892	0.060247003	0.606364174	Potri.T117100.1	AT3G44480.1
Potra2n11c23632.1	0.82357019	NA	NA	NA	Potri.T117200.1	
Potra2n11c23633.1	1.545832463	1.571529889	NA	NA	Potri.011G005800.1	
Potra2n11c23634.1	0.310754335	0.281031129	0.21099274	0.773063484	Potri.011G005900.1	AT3G14460.1
Potra2n11c23635.1	0.661975877	0.599145401	0.060511229	0.513156191	Potri.011G005900.1	AT3G14460.1
Potra2n11c23636.1	1.072266479	0.950776214	0.024666998	0.576228147	Potri.011G005900.1	AT3G14460.1
Potra2n11c23637.1	1.459962178	NA	NA	NA	Potri.011G005400.1	
Potra2n11c23638.1	1.03474785	1.403043657	NA	NA	Potri.011G005200.1	AT4G01500.1
Potra2n11c23639.1	0.92641307	0.64247635	NA	NA	Potri.011G005200.1	AT4G01500.1
Potra2n11c23640.1	1.366921589	0.505648407	5.74537E-11	0.605100478	Potri.004G013200.2	AT1G11840.6
Potra2n11c23641.1	2.340207562	1.363811008	NA	NA		
Potra2n11c23642.1	NA	NA	NA	NA	Potri.011G004900.1	
Potra2n11c23643.1						
Potra2n11c23644.1	NA	NA	NA	NA	Potri.011G006000.1	
Potra2n11c23645.1	-0.12506157	0.127985062	0.468255822	0.910074792	Potri.011G004800.2	
Potra2n11c23646.1	0.349649296	-0.117502009	0.274609381	0.959881296	Potri.011G004700.1	AT1G11790.1

Potra2n11c23647.1	4.533998444	1.066897328	0.004938145	NA		
Potra2n11c23648.1	-1.507585912	-0.45927949	5.90941E-05	0.72101822	Potri.011G004500.1	AT1G62050.1
Potra2n11c23649.1	-1.356781018	-0.293919205	6.30821E-05	0.840888112	Potri.011G004400.1	AT1G11740.1
Potra2n11c23650.1	-0.650107837	-0.120031293	3.20543E-05	0.914903229	Potri.011G004300.1	AT1G62040.2
Potra2n11c23651.1	-0.676699504	0.182803932	0.59967634	0.973347974		
Potra2n11c23652.1	-1.197291669	-0.438198331	0.097463869	0.89438295	Potri.011G004200.2	AT1G05170.1
Potra2n11c23653.1	-2.816671128	-0.632425679	1.96035E-11	0.322221366	Potri.004G014000.1	AT4G04630.1
Potra2n11c23654.1	-2.334665665	-0.729557071	4.92774E-53	0.000146941	Potri.001G226000.1	AT5G24550.1
Potra2n11c23655.1	-2.153654726	-0.616608236	1.93536E-25	0.611072002	Potri.001G226000.1	AT5G24550.1
Potra2n11c23656.1						
Potra2n11c23657.1	0.797244629	-0.160120756	0.058737274	0.951726178	Potri.011G004000.1	AT5G38280.1
Potra2n11c23658.1	-0.357672517	-0.064208528	0.210938237	0.973347974	Potri.011G003700.1	AT1G11710.1
Potra2n11c23659.1	-0.153001193	-0.005893879	0.323319144	0.996032145	Potri.011G003500.2	AT1G64570.1
Potra2n11c23660.1	0.682916589	0.165763691	1.36022E-06	0.823519367	Potri.011G003400.1	AT4G04720.1
Potra2n11c23661.1	-0.392540389	-0.158887245	0.163947314	0.813731863	Potri.011G003300.1	AT1G11700.1
Potra2n11c23662.1	-2.295337707	-2.446267299	NA	NA		
Potra2n11c23663.1	0.649513567	0.591819844	0.719916516	0.948577865	Potri.011G003200.1	AT1G50660.1
Potra2n11c23664.1	-1.317292482	-1.465929376	NA	NA	Potri.011G003100.1	AT3G20340.1
Potra2n11c23665.1	-0.052319079	-0.087321773	0.74998416	0.946464685	Potri.011G003000.1	AT1G61900.1
Potra2n11c23666.1	0.736801623	0.112299929	0.000116409	0.939149035	Potri.011G002900.1	AT3G21690.1
Potra2n11c23667.1	0.642090859	0.044362872	0.003750339	0.986636967	Potri.011G002800.1	AT3G21690.1
Potra2n11c23668.1	0.250762043	-0.258893631	0.702289402	0.946146503	Potri.011G002700.1	AT3G21690.1
Potra2n11c23669.1	0.487750392	-0.72871782	0.675135872	0.895474078		
Potra2n11c23670.1	-0.230585084	-2.456974026	NA	NA	Potri.011G002600.2	AT3G21690.1
Potra2n11c23671.1	2.734003598	0.342533799	0.000180001	0.948577865	Potri.011G002500.1	AT3G21690.1
Potra2n11c23672.1	3.024560918	-0.559063014	1.48407E-06	0.950706289	Potri.011G002400.1	AT3G21690.1
Potra2n11c23673.1	0.943350674	1.955029706	NA	NA	Potri.011G002200.1	AT1G11670.1
Potra2n11c23674.1	NA	NA	NA	NA	Potri.011G002100.1	AT1G11655.1
Potra2n11c23675.1	NA	1.363811008	NA	NA	Potri.004G017000.1	AT5G52890.2
Potra2n11c23676.1	0.333315465	0.064386371	0.007467262	0.941677175	Potri.011G001800.1	AT4G21890.1
Potra2n11c23677.3	-0.296425026	-0.108609871	0.032950243	0.910477984	Potri.011G001100.1	AT1G61870.1
Potra2n11c23678.1	-0.296425026	-0.108609871	0.032950243	0.910477984	Potri.011G001500.2	AT5G36110.1

Potra2n11c23679.1	-0.023307432	0.19008158	0.897496184	0.813731863	Potri.011G001000.1	AT4G21790.1
Potra2n11c23680.1	0.025550323	0.136545726	0.837164216	0.843683654	Potri.011G000900.1	AT4G04860.1
Potra2n11c23681.1					Potri.011G000801.1	
Potra2n11c23682.1	1.545832463	NA	NA	NA	Potri.011G000700.1	AT5G07610.1
Potra2n11c23683.1	0.884531052	1.212408117	NA	NA	Potri.011G000600.1	AT1G61860.1
Potra2n11c23684.1	1.594379011	2.406846372	NA	NA	Potri.011G000600.1	AT1G61860.1
Potra2n11c23685.1	0.085212716	1.196302976	0.924876493	0.504797581	Potri.011G000500.1	AT3G20530.1
Potra2n11c23686.1	0.277098221	-0.050013222	0.081141165	0.974367223	Potri.011G000200.1	AT1G61850.2
Potra2n1202s36988.1					Potri.017G005200.1	AT5G48380.1
Potra2n1227s36989.1	1.545832463	NA	NA	NA	Potri.004G052500.1	AT2G29120.1
Potra2n1228s36990.1						
Potra2n1235s36991.1	-1.317292482	-0.05574223	NA	NA	Potri.004G074200.1	AT5G09500.1
Potra2n1236s36992.1	0.393991154	-0.069091718	0.319031643	0.970432679	Potri.002G086600.1	AT4G08230.1
Potra2n1240s36993.1	NA	1.571529889	NA	NA		
Potra2n1242s36994.1					Potri.T053500.1	AT3G60470.1
Potra2n1242s36995.1					Potri.012G012400.1	
Potra2n1249s36996.1	-1.635716171	-1.461968611	0.002077849	0.386015987	Potri.009G058800.1	AT3G08030.1
Potra2n124s34585.1	1.135883844	0.485491683	1.5874E-06	0.719104446	Potri.011G025000.3	AT4G04890.1
Potra2n124s34586.1					Potri.011G025100.1	AT2G46150.1
Potra2n124s34587.3	0.083417446	-1.102424974	0.947527277	0.824448251	Potri.011G025200.1	AT5G54770.1
Potra2n124s34588.1	2.955064574	0.85209299	2.42169E-16	0.815929492	Potri.011G025400.1	AT1G11580.1
Potra2n124s34589.1	2.856357149	4.081519175	0.126242219	0.276027277	Potri.011G025500.1	AT1G11570.2
Potra2n124s34590.1	0.050586149	-0.100963696	0.708397159	0.910878765	Potri.011G025700.1	AT1G61790.1
Potra2n124s34591.1	-0.410996922	1.524656008	0.7720146	0.897389232	Potri.011G025800.2	AT1G11545.1
Potra2n124s34592.1	1.545832463	NA	NA	NA		
Potra2n124s34593.1	0.82357019	NA	NA	NA	Potri.004G117600.1	
Potra2n124s34594.2	5.401750266	2.623753514	8.04316E-13	0.22650515	Potri.011G026200.1	AT1G24020.2
Potra2n124s34595.1						
Potra2n124s34596.1	0.421589618	0.129929366	0.481134904	0.974264269		
Potra2n124s34597.1	-1.450042104	0.284814604	0.001178279	0.931774514	Potri.011G026300.1	AT1G01380.1
Potra2n124s34598.1	-0.090600227	-0.245832128	0.633069025	0.767368598	Potri.001G418500.1	AT4G05320.2
Potra2n124s34599.1	1.651706702	NA	NA	NA	Potri.011G026700.4	AT4G21200.1

Potra2n124s34600.1					Potri.011G026800.1	AT4G21210.1
Potra2n124s34601.1	-0.58311662	-0.280163731	0.001062405	0.761354735	Potri.011G026900.2	AT4G21215.2
Potra2n124s34602.1	-2.9348217	-3.083418285	NA	NA	Potri.004G101100.1	AT3G02280.1
Potra2n124s34603.1						
Potra2n124s34604.2	0.82357019	4.224456144	NA	NA	Potri.011G027300.1	AT3G01190.1
Potra2n124s34605.1	1.664677056	NA	NA	NA	Potri.011G027400.2	AT4G05220.1
Potra2n124s34606.1	1.664677056	0.674963689	NA	NA	Potri.011G029800.1	AT4G23220.1
Potra2n124s34607.1	5.257362342	1.921063811	1.09968E-08	NA	Potri.T128700.1	AT4G23180.1
Potra2n124s34608.1	4.178591508	2.501508747	0.041613325	NA	Potri.011G030200.1	AT4G23220.1
Potra2n124s34609.1	0.82357019	NA	NA	NA		
Potra2n124s34610.1	NA	NA	NA	NA	Potri.001G234900.1	AT5G59750.1
Potra2n1251s36997.1	NA	1.394613675	NA	NA	Potri.008G001900.1	AT2G01050.1
Potra2n1254s36998.1	1.493438217	-0.540796453	0.165247767	0.894747711	Potri.010G176000.1	AT1G65930.1
Potra2n1259s36999.1					Potri.017G063900.2	AT3G27060.1
Potra2n125s34611.1	0.050942684	0.209381255	0.838383051	0.865611082		
Potra2n125s34612.1	3.329564962	1.394613675	NA	NA	Potri.007G078900.1	AT5G64790.1
Potra2n125s34613.1					Potri.005G085800.1	AT2G21860.1
Potra2n125s34614.1	0.001474921	0.055244123	0.995973022	0.976302215	Potri.007G079200.1	AT4G39470.1
Potra2n125s34615.1	0.586929814	0.155417522	7.90731E-05	0.900866967	Potri.007G079300.1	AT1G10490.1
Potra2n125s34616.1	0.257155988	0.231334938	0.795031639	0.953831244		
Potra2n125s34617.1	0.268192391	0.199547725	0.402690119	0.909675463	Potri.007G079400.1	AT1G02420.1
Potra2n125s34618.1	-0.04255355	-0.022957269	0.797093024	0.985393296	Potri.007G079500.1	AT2G21870.1
Potra2n125s34619.1	0.288997267	-0.054866475	0.080876538	0.973347974	Potri.007G079600.2	AT1G10500.1
Potra2n125s34620.1					Potri.007G079701.1	AT4G09720.1
Potra2n125s34621.1					Potri.001G155750.1	AT1G21280.1
Potra2n125s34622.1	0.102236182	1.1901161	NA	NA		
Potra2n125s34623.1	5.966772585	-1.478536539	4.33304E-06	NA		
Potra2n125s34624.1	8.181930739	5.449477317	6.79652E-33	0.01476039	Potri.007G079800.1	AT5G64810.1
Potra2n125s34625.4	-0.408010795	0.185727195	0.004232208	0.87909151	Potri.007G080100.2	AT5G64813.1
Potra2n125s34626.1	-1.25246262	-0.264358476	0.00624349	0.924951347	Potri.007G080200.1	
Potra2n125s34627.1	-1.610838496	-0.571174882	1.00976E-05	0.351105764	Potri.007G080300.1	AT4G39490.1
Potra2n125s34628.1	-0.692637809	-1.513020689	0.411017292	0.298167012	Potri.015G132800.1	AT4G39490.1

Potra2n125s34629.1	0.288225033	0.013309037	0.028477076	0.989304496	Potri.005G084900.1	AT5G09920.1
Potra2n125s34630.1	0.05559939	0.082540905	0.681434235	0.921065381	Potri.007G080500.1	AT5G64830.1
Potra2n125s34631.1	-0.161786964	0.563547095	0.222926907	0.736203664	Potri.007G080600.1	AT5G64840.1
Potra2n125s34632.1	1.545832463	0.674963689	NA	NA	Potri.011G119300.1	AT1G53040.2
Potra2n125s34633.1	-1.26760488	-0.540083043	0.029189129	0.557595248	Potri.007G080800.2	AT5G09960.1
Potra2n125s34634.1	0.248113148	0.007453991	0.054885385	0.995579852	Potri.007G080900.1	AT4G39520.1
Potra2n125s34635.1	-0.217366486	0.002231091	0.262216284	0.999458638	Potri.007G081000.1	AT2G21940.4
Potra2n125s34636.1	-0.429644154	0.02975538	0.08898055	0.992891045	Potri.007G081100.1	AT2G21950.1
Potra2n125s34637.1	NA	NA	NA	NA	Potri.007G081200.1	AT5G09970.1
Potra2n1265s37000.1					Potri.001G226000.1	AT5G24550.1
Potra2n1269s37001.1	0.299866513	0.43215534	0.492362931	0.90158221		
Potra2n126s34638.1	3.653660862	3.179179018	0.21794331	NA	Potri.013G133800.1	AT1G03670.1
Potra2n126s34639.1	0.549492303	0.728557867	0.103192805	0.679125998	Potri.013G134000.1	AT1G03650.1
Potra2n126s34640.1	-0.089545357	-0.23631986	0.91245003	0.946461635	Potri.013G133900.2	AT1G03670.1
Potra2n126s34641.1					Potri.013G133900.2	AT1G03670.1
Potra2n126s34642.1	NA	NA	NA	NA	Potri.013G133900.2	AT1G03670.1
Potra2n126s34643.1	NA	NA	NA	NA	Potri.013G134500.1	AT1G03670.1
Potra2n126s34644.1	-0.172944692	0.47090937	0.921117663	NA	Potri.013G133800.1	AT1G03670.1
Potra2n126s34645.1	-0.190764053	0.163328578	0.251163279	0.869175327		
Potra2n126s34646.1	-1.262214856	0.455585708	0.505602537	NA	Potri.013G133700.1	AT1G03670.1
Potra2n1272s37002.1	2.219472599	2.718088762	0.520831434	NA	Potri.016G094600.1	AT3G09830.2
Potra2n1273s37003.1	0.612236273	0.205350226	0.011472277	0.813731863	Potri.019G070460.1	AT5G17680.1
Potra2n1277s37004.1	-0.005904265	-0.015124702	0.965525849	0.989304496	Potri.018G056600.1	AT1G53750.1
Potra2n127s34647.1	4.569174139	NA	0.079217918	NA	Potri.001G012700.1	AT3G12120.2
Potra2n127s34648.1	0.214505153	-0.192400815	0.234808074	0.821709751	Potri.001G012800.1	AT2G18220.1
Potra2n127s34649.1	0.058439304	0.064198601	0.803370936	0.966501596	Potri.001G013200.1	AT3G12130.1
Potra2n127s34650.1	0.40533565	0.023692648	0.048962697	0.987226805	Potri.001G013300.1	AT1G73240.1
Potra2n127s34651.1	1.068709457	0.824221071	0.000941814	0.663950227	Potri.001G013400.1	AT5G19640.1
Potra2n127s34652.2	0.208650172	0.103480704	0.185294424	0.895430113	Potri.001G013500.1	AT2G05990.1
Potra2n127s34653.1	0.604497456	0.231012763	0.000122986	0.84487051	Potri.001G013600.1	AT2G44430.1
Potra2n127s34654.1	NA	NA	NA	NA		
Potra2n127s34655.1	-0.296105679	-0.068727978	0.109177367	0.953608209	Potri.001G013700.1	AT5G19630.1

Potra2n127s34656.1	-0.38120949	-0.06339462	2.24518E-05	0.935099709	Potri.001G013800.1	AT5G13020.1
Potra2n127s34657.1	-0.072756736	-0.13907383	0.804752836	0.910878765	Potri.001G014000.1	AT1G04640.2
Potra2n127s34658.1	4.706754365	1.571529889	0.048934793	NA	Potri.003G212000.1	AT5G60900.1
Potra2n127s34659.1	6.056298899	2.409230874	2.35853E-22	0.725595998	Potri.001G014600.1	AT5G60900.1
Potra2n127s34660.1	0.82357019	1.381953902	NA	NA	Potri.001G014500.1	AT5G60900.1
Potra2n127s34661.2	0.142692936	-0.837764393	0.857576477	0.574239453	Potri.001G014700.1	AT5G60900.1
Potra2n127s34662.1	NA	NA	NA	NA	Potri.T084800.1	AT5G60900.1
Potra2n127s34663.1	-0.274850884	0.045669882	0.31469284	0.981772014	Potri.001G014900.2	AT5G59600.1
Potra2n127s34664.1	-0.042236864	-0.085033221	0.814166413	0.920141992	Potri.001G015000.1	AT3G57930.2
Potra2n127s34665.1	-0.034770297	-0.071512576	0.851411404	0.938967511		
Potra2n127s34666.1	2.735991243	NA	NA	NA	Potri.003G211100.1	AT5G44640.1
Potra2n1281s37005.1	NA	NA	NA	NA		
Potra2n128s34667.1	3.13301321	1.758240378	0.004150734	NA	Potri.011G104900.1	AT1G71400.1
Potra2n128s34668.1	0.82357019	1.363811008	NA	NA	Potri.011G104900.1	AT1G71400.1
Potra2n128s34669.1	-0.116325323	-0.382091806	0.821063023	0.88066693	Potri.011G104400.1	AT1G78590.1
Potra2n128s34670.1	3.063484644	0.261513902	6.41433E-05	0.978203092	Potri.T086001.1	AT1G53490.1
Potra2n128s34671.1	NA	0.674963689	NA	NA	Potri.011G104400.1	AT1G78590.1
Potra2n128s34672.1	5.854240934	3.339438951	8.81155E-11	0.466211853	Potri.011G104900.1	AT1G71400.1
Potra2n12c23687.1	0.169490684	0.347078802	0.691375459	0.859939646	Potri.012G024700.3	AT5G53550.1
Potra2n12c23688.1	-0.144926123	0.215730422	0.271394941	0.792896411	Potri.011G055300.1	
Potra2n12c23689.1	-0.036650984	0.072213349	0.926330568	0.981772014	Potri.012G024700.3	AT5G53550.1
Potra2n12c23690.1	0.075422647	-0.101344242	0.600110575	0.914321114	Potri.012G024600.1	AT5G53560.1
Potra2n12c23691.1	0.105143361	-0.020999979	0.351742085	0.983446961	Potri.015G007400.1	AT3G49350.1
Potra2n12c23692.1	0.369961596	-0.190068672	0.025886843	0.753052725	Potri.012G024400.1	AT5G24400.1
Potra2n12c23693.1	0.797023185	0.186008441	2.48379E-05	0.917751882	Potri.004G195200.2	AT3G14470.1
Potra2n12c23694.1	4.455509501	NA	0.022502868	NA	Potri.012G024200.3	AT5G17260.1
Potra2n12c23695.1	0.82357019	NA	NA	NA	Potri.012G024100.1	AT5G17260.1
Potra2n12c23696.1	1.664677056	NA	NA	NA		
Potra2n12c23697.1	1.21027164	-0.767708831	NA	NA	Potri.012G023900.1	AT5G17260.1
Potra2n12c23698.1	-0.814849721	0.007223314	0.083883865	0.99905083	Potri.012G023800.1	AT5G53580.1
Potra2n12c23699.1	-2.640944097	0.049154326	0.001013839	NA	Potri.012G023700.1	AT2G46680.1
Potra2n12c23700.1	1.459962178	NA	NA	NA	Potri.012G023500.1	AT1G57680.2

Potra2n12c23701.1	-0.72858711	-3.129047247	0.851256252	NA		
Potra2n12c23702.1	-0.714947328	-0.143591083	2.17365E-05	0.953327073	Potri.012G023400.1	AT2G46690.1
Potra2n12c23703.1	2.01148533	0.515341796	1.37254E-13	0.50924822	Potri.012G023300.1	AT5G24430.1
Potra2n12c23704.1	0.201266299	0.0673816	0.056982245	0.940376196	Potri.012G023200.1	AT5G53620.3
Potra2n12c23705.1	0.324300517	0.101474176	0.00066605	0.914551931		
Potra2n12c23706.1	-0.449854289	-0.195602199	0.151767059	0.865591169	Potri.012G023100.1	AT4G00850.1
Potra2n12c23707.1	-0.256485357	-0.056415234	0.039011564	0.961670176	Potri.012G022900.1	AT5G53650.1
Potra2n12c23708.1	-0.506331865	-0.403861341	0.819361047	NA	Potri.012G023000.1	
Potra2n12c23709.1	-0.533862516	-0.303857825	0.023315505	0.755109612	Potri.012G022800.1	AT1G73080.1
Potra2n12c23710.1	-0.288757042	0.038554062	0.606583759	0.99005311		
Potra2n12c23711.1	-0.217176329	-0.635343104	0.654743058	0.739520512		
Potra2n12c23712.1	0.301583067	0.107381416	0.03289613	0.900869139	Potri.012G022700.2	AT5G51660.1
Potra2n12c23713.1	-1.043845123	-0.595921979	0.040626614	0.773063484	Potri.012G022600.2	AT5G53660.1
Potra2n12c23714.1	1.652877029	1.849701723	NA	NA	Potri.012G022500.1	AT1G01110.2
Potra2n12c23715.1	0.14882527	0.213840522	0.80896923	0.953608209	Potri.012G022400.1	AT4G27670.1
Potra2n12c23716.1	-0.227286103	-0.128987269	0.501501622	0.922618347	Potri.012G022300.1	AT5G03890.1
Potra2n12c23717.2	-0.029542699	-0.019552938	0.880849052	0.984910847	Potri.012G022200.4	AT5G64180.1
Potra2n12c23718.1	1.026873066	0.766504588	0.019148749	0.497543905	Potri.012G022100.1	AT2G46780.1
Potra2n12c23719.1	0.191378751	0.01854898	0.14003975	0.981424427	Potri.012G022000.1	AT1G32790.1
Potra2n12c23720.1	0.503240657	0.320923838	0.025823035	0.755774501	Potri.012G021900.1	AT3G49400.1
Potra2n12c23721.1	-0.03536678	0.155905697	0.768092065	0.885649686	Potri.012G021800.1	AT3G49430.2
Potra2n12c23722.1	0.902905251	0.026279078	1.6575E-05	0.991110595	Potri.012G021700.1	AT5G24510.1
Potra2n12c23723.1	1.834858865	0.358993653	0.014602567	0.953177702	Potri.012G021601.1	
Potra2n12c23724.1	3.822055991	0.661678451	0.000427869	NA	Potri.015G004800.1	AT5G54165.1
Potra2n12c23725.1	-0.149972304	0.0382593	0.278279481	0.981259756	Potri.012G021500.1	
Potra2n12c23726.1	-0.22780189	0.000359663	0.080121069	0.999937671	Potri.012G021400.1	AT1G64720.1
Potra2n12c23727.1	-0.106369468	-0.071091128	0.725030404	0.966148753	Potri.012G021300.3	AT5G24500.1
Potra2n12c23728.1	-1.35927387	-1.507841271	NA	NA	Potri.012G021100.1	AT3G04580.1
Potra2n12c23729.1	-1.453732868	-0.108218062	0.014642373	0.97950444	Potri.015G004400.2	AT2G02880.1
Potra2n12c23730.1					Potri.T119400.1	
Potra2n12c23731.1						
Potra2n12c23732.1	1.235274523	-2.173467376	0.097042834	0.516164384	Potri.012G020800.1	AT2G46620.1

Potra2n12c23733.1	1.547754582	0.088161099	0.011680134	0.98669034	Potri.012G020700.2	AT4G27700.1
Potra2n12c23734.1	0.92641307	-0.767708831	NA	NA		
Potra2n12c23735.1	3.994409482	1.695875574	6.62599E-17	0.547799891		
Potra2n12c23736.1	-0.637934825	-0.786502229	NA	NA		
Potra2n12c23737.1	1.175261528	-1.422635021	0.109401405	0.672658526	Potri.012G020700.2	AT4G27700.1
Potra2n12c23738.1	-0.046205257	0.10436146	0.839060544	0.94608223	Potri.011G055300.1	
Potra2n12c23739.2	0.190456043	0.254594561	0.588004213	0.922618347	Potri.012G024700.3	AT5G53550.1
Potra2n12c23740.1	-0.318433731	-0.255939542	0.565706099	0.917746944	Potri.012G027300.1	AT2G37460.1
Potra2n12c23741.1	1.788123314	-0.295134577	0.006484525	0.954419252	Potri.015G007700.1	AT4G27680.1
Potra2n12c23742.1	0.172289198	0.255365947	0.783097851	0.948116504	Potri.012G027800.1	AT5G53550.1
Potra2n12c23743.1	-0.053655295	0.060188067	0.825715913	0.967103157	Potri.015G007700.1	AT4G27680.1
Potra2n12c23744.1	-0.032868952	0.114883829	0.932047582	0.966323513	Potri.012G024700.3	AT5G53550.1
Potra2n12c23745.1	-1.407512651	0.578658715	0.41344179	0.949966783	Potri.010G009600.1	AT4G27680.1
Potra2n12c23746.1	1.40280235	-0.088192017	NA	NA	Potri.012G027901.1	AT5G53540.1
Potra2n12c23747.1	-0.075350126	0.000717588	0.850530623	0.999937671	Potri.012G008800.1	AT4G27680.1
Potra2n12c23748.1	-0.293110553	0.046391698	0.100252927	0.983446961	Potri.015G007700.1	AT4G27680.1
Potra2n12c23749.2	2.658032118	NA	NA	NA		
Potra2n12c23750.1	-0.156326597	0.060974391	0.66098412	0.982753986	Potri.012G024700.3	AT5G53550.1
Potra2n12c23751.1	NA	NA	NA	NA	Potri.012G027300.1	AT2G37460.1
Potra2n12c23752.1	0.003272406	0.211271522	0.99016258	0.866777645	Potri.010G009600.1	AT4G27680.1
Potra2n12c23753.1	5.287372845	3.021780406	0.073874412	NA		
Potra2n12c23754.1						
Potra2n12c23755.1					Potri.012G026300.1	
Potra2n12c23756.1	2.658032118	2.315796285	NA	NA	Potri.012G007700.1	AT2G37460.1
Potra2n12c23757.1	-0.009744479	-0.394950011	0.98336506	0.893770808	Potri.012G007600.1	AT5G54130.2
Potra2n12c23758.1	1.131616542	0.514396155	4.31913E-06	0.586594557	Potri.012G007500.1	AT3G49530.1
Potra2n12c23759.1	0.139054098	0.058409276	0.902171959	0.991089517	Potri.012G007300.1	AT5G24580.1
Potra2n12c23760.1	2.521362334	NA	NA	NA	Potri.012G007200.1	AT4G27590.1
Potra2n12c23761.3	0.17609808	0.120621645	0.234401988	0.913532867	Potri.012G007000.1	AT3G49490.1
Potra2n12c23762.1	-0.595952689	-1.465929376	NA	NA	Potri.012G006900.1	AT5G17820.1
Potra2n12c23763.1					Potri.012G006800.2	AT1G05260.1
Potra2n12c23764.1	6.266225524	2.749662303	2.06104E-10	NA	Potri.012G006800.2	AT1G05260.1

Potra2n12c23765.1	0.186274657	-0.113872464	0.276997615	0.935099709	Potri.012G006501.1	AT4G27660.1
Potra2n12c23766.1	-0.396288591	-0.33042561	0.033305497	0.72680202	Potri.012G006400.1	AT5G54160.1
Potra2n12c23767.1	-1.216620031	0.177671899	0.43391927	0.983944804	Potri.012G006400.1	AT5G54160.1
Potra2n12c23768.1	7.906144002	4.570318609	3.943E-128	0.039206622	Potri.012G006300.1	AT5G24530.1
Potra2n12c23769.1	0.501363876	0.123812474	4.45258E-09	0.857418365	Potri.012G006100.1	AT5G24520.2
Potra2n12c23770.1						
Potra2n12c23771.1	-0.690921874	-0.522978882	0.002523251	0.50924822		
Potra2n12c23772.1	2.161910035	0.256413732	0.001073998	0.966228173	Potri.012G004000.1	AT4G27870.1
Potra2n12c23773.1	0.893682768	0.11673175	1.92225E-05	0.943483421	Potri.012G004300.1	AT5G24680.1
Potra2n12c23774.1	1.47768453	-1.465929376	NA	NA	Potri.012G004200.1	AT1G27940.1
Potra2n12c23775.1	-0.148939145	0.052638179	0.558867257	0.974367223	Potri.012G004400.1	AT3G49590.1
Potra2n12c23776.1	-0.330866767	0.172861893	0.271374519	0.896937427	Potri.012G004500.1	AT5G24660.1
Potra2n12c23777.1	0.36634243	0.443218061	0.050242821	0.413717625	Potri.012G004600.1	AT5G24650.1
Potra2n12c23778.1	-0.194698498	0.098466276	0.046880511	0.846499552	Potri.012G004700.2	AT5G24630.5
Potra2n12c23779.1	-0.287900307	-0.174965144	0.806908209	0.983446961	Potri.012G004800.2	AT5G24620.1
Potra2n12c23780.2	0.166824262	-0.022651184	0.529859516	0.987226805	Potri.012G004900.1	AT3G49550.1
Potra2n12c23781.6	-0.250329546	-0.121191182	0.060746256	0.92651938	Potri.012G005000.1	AT5G54090.1
Potra2n12c23782.1	-0.565705661	-0.231116113	0.00025639	0.70570456	Potri.012G005100.1	AT1G18010.1
Potra2n12c23783.1	-1.190783454	-1.963526752	0.465587113	0.597931989	Potri.012G005300.1	
Potra2n12c23784.1	1.349692889	0.632393252	3.42161E-22	0.022879829	Potri.012G010800.1	AT4G27585.1
Potra2n12c23785.1	0.669398309	-1.709906057	0.601051052	NA	Potri.015G001800.1	AT4G01575.1
Potra2n12c23786.1	NA	NA	NA	NA		
Potra2n12c23787.1	0.550129422	0.564703691	0.001156885	0.282148815	Potri.012G010800.1	AT4G27585.1
Potra2n12c23788.1	NA	NA	NA	NA		
Potra2n12c23789.1	-0.070232456	-0.027485109	0.845021538	0.992934677	Potri.012G005800.2	AT5G24490.1
Potra2n12c23790.3	0.709656899	0.560008674	0.002676025	0.183934002	Potri.012G010800.1	AT4G27585.1
Potra2n12c23791.1						
Potra2n12c23792.1	5.795450825	2.720941519	2.4413E-24	0.055523637		
Potra2n12c23793.1	0.110397808	-0.14074829	0.603589744	0.936890993	Potri.012G020500.1	AT4G27720.1
Potra2n12c23794.1	-0.483676236	-0.237987855	0.000942187	0.749416134	Potri.012G020400.2	
Potra2n12c23795.1	NA	0.674963689	NA	NA	Potri.012G020200.1	AT1G55580.1
Potra2n12c23796.1	0.161245075	-0.094534247	0.948081695	NA	Potri.012G024400.1	AT5G24400.1

Potra2n12c23797.1	-0.075513317	0.150606112	0.499635012	0.80723942	Potri.012G020000.4	AT5G24350.1
Potra2n12c23798.1	3.796221266	1.533970022	1.00422E-51	0.122109873	Potri.012G019900.1	AT1G76390.1
Potra2n12c23799.1	-2.575488693	-0.332473414	1.00785E-14	0.932984974	Potri.012G019700.1	AT4G27730.1
Potra2n12c23800.1						
Potra2n12c23801.1					Potri.012G019600.1	AT5G52260.1
Potra2n12c23802.1	NA	NA	NA	NA	Potri.012G019600.1	AT5G52260.1
Potra2n12c23803.1	-1.253128035	2.055124102	0.682573584	NA	Potri.012G019500.1	AT4G27730.1
Potra2n12c23804.1	1.92091852	0.986542505	0.037503473	0.720242695	Potri.012G019400.1	AT3G24770.1
Potra2n12c23805.1	0.152531188	-0.484132873	0.950176674	NA		
Potra2n12c23806.2	-0.220751095	-0.276225647	0.17776251	0.643173103	Potri.012G019300.2	AT1G21320.2
Potra2n12c23807.1	5.865324354	1.73534116	8.71985E-41	0.417067084	Potri.012G019200.1	AT4G27740.1
Potra2n12c23808.2	0.372367003	0.109218144	0.035698663	0.903477395	Potri.012G019100.1	AT4G27745.1
Potra2n12c23809.1	2.476290371	1.381953902	NA	NA	Potri.012G018900.1	
Potra2n12c23810.1	0.663324007	0.46316149	0.068090715	0.701494362	Potri.012G018800.1	AT2G13600.1
Potra2n12c23811.1	-0.230309521	-0.638305939	0.417120439	0.010494842	Potri.012G018700.1	AT3G61850.4
Potra2n12c23812.3	-0.32488771	-0.023218687	0.017042986	0.983944804	Potri.015G009400.2	AT3G26100.2
Potra2n12c23813.1	2.340207562	NA	NA	NA	Potri.012G018500.2	AT5G45470.1
Potra2n12c23814.1	1.5452556	1.051226576	NA	NA		
Potra2n12c23815.1	-0.746088649	-1.003185017	0.647863569	NA	Potri.012G018400.1	AT5G45470.1
Potra2n12c23816.1	-1.657471635	-0.387223647	0.014667826	0.930610374	Potri.012G018300.1	AT5G24330.1
Potra2n12c23817.1	-2.8269424	-0.32474141	0.000996597	0.937687311		
Potra2n12c23818.1	-1.065631692	-0.513666513	0.000958105	0.775618506	Potri.012G018200.10	AT5G24320.1
Potra2n12c23819.1	-1.028058375	-0.587326252	0.002106911	0.726091974		
Potra2n12c23820.1	-0.375676413	0.133413845	0.42967252	0.973347974	Potri.012G018100.1	AT5G53490.2
Potra2n12c23821.1	-0.678563655	-0.150369356	0.095749987	0.946314915	Potri.012G017900.1	
Potra2n12c23822.1	0.252201838	-0.65865126	0.88011322	0.920028955	Potri.012G017800.2	AT5G24318.1
Potra2n12c23823.1	-0.000196467	0.00039567	0.999234635	0.999937671	Potri.015G010200.1	AT4G27780.1
Potra2n12c23824.1	0.560767651	0.110405366	3.8783E-05	0.910815461	Potri.012G017600.1	AT5G42020.1
Potra2n12c23825.1	-0.033928422	0.019293913	0.831107468	0.981772014	Potri.012G017500.1	AT5G53480.1
Potra2n12c23826.3	-1.423137312	-0.133874847	0.000435874	0.974275337		
Potra2n12c23827.1	0.257443497	-0.14388884	0.410871083	0.926065782	Potri.T089300.1	AT5G53070.1
Potra2n12c23828.1	0.227832859	0.051867678	0.098096966	0.959431187	Potri.012G017200.1	AT5G53060.1

Potra2n12c23829.1	0.139628311	-0.124182339	0.547539431	0.924869625	Potri.012G017100.1	AT5G53050.2
Potra2n12c23830.1	-0.184917181	-0.236050536	0.458483099	0.813731863	Potri.015G011500.1	AT5G53045.1
Potra2n12c23831.1	-0.363396503	-0.357025038	0.015351822	0.719366603	Potri.012G016900.2	AT3G49290.2
Potra2n12c23832.1					Potri.012G016800.1	AT5G53040.1
Potra2n12c23833.1	NA	NA	NA	NA	Potri.012G016700.1	AT5G53030.2
Potra2n12c23834.1	-0.002928598	-0.041638042	0.993776121	0.981752423	Potri.012G016600.3	AT5G53020.1
Potra2n12c23835.1	-0.13065469	-0.036998126	0.318645196	0.974367223	Potri.012G016500.1	AT5G53000.1
Potra2n12c23836.1	-0.550633583	0.289826821	0.01372449	0.880553952	Potri.012G016400.1	AT5G52990.1
Potra2n12c23837.2	-0.846737763	-0.377795455	4.02007E-06	0.746877722	Potri.012G016200.1	AT3G49260.3
Potra2n12c23838.1	-0.534540372	0.293301368	0.025795219	0.88643982	Potri.012G016100.3	AT5G24280.1
Potra2n12c23839.2	-0.611864968	-0.189833536	0.055653511	0.943057979	Potri.012G016000.1	AT3G49250.1
Potra2n12c23840.2	-0.717175666	0.003915005	2.17835E-05	0.998895625		
Potra2n12c23841.1	0.102236182	0.64247635	NA	NA	Potri.012G015700.1	
Potra2n12c23842.1	-0.828489122	-0.066009443	0.393267139	0.992934677	Potri.012G015700.1	
Potra2n12c23843.1	-0.628603798	-0.12773228	5.32153E-06	0.88661378	Potri.012G015600.2	AT5G53350.1
Potra2n12c23844.2	-0.610531776	-0.218369253	1.71138E-06	0.830095229	Potri.012G015500.1	AT4G27880.1
Potra2n12c23845.1	-0.551340105	-0.268796256	5.43041E-07	0.667038977	Potri.012G015400.5	AT3G49240.1
Potra2n12c23846.1	-0.706146536	-0.620834059	0.011609263	0.401934535	Potri.012G015100.1	AT5G24270.1
Potra2n12c23847.1	0.218014748	1.733492604	0.899946031	0.698345631	Potri.012G015000.4	AT5G03170.1
Potra2n12c23848.1	-0.109327237	0.087544591	0.5186707	0.941677175	Potri.012G014900.2	AT5G24260.1
Potra2n12c23849.1	2.096139174	1.630707616	0.310733914	NA	Potri.012G014800.1	
Potra2n12c23850.1	1.628648623	0.484431011	0.013124488	0.895474078	Potri.012G014700.1	AT1G12170.1
Potra2n12c23851.1	-0.060627821	-0.013761542	0.697773064	0.986636967	Potri.012G014600.1	AT5G24240.1
Potra2n12c23852.3	-0.379205029	-0.199881365	0.224515845	0.896627841		
Potra2n12c23853.1	1.157781063	0.170210813	1.17222E-19	0.566750433	Potri.012G014500.1	AT3G49220.1
Potra2n12c23854.2	0.317749113	0.088214386	0.040846904	0.948577865	Potri.012G014300.1	AT3G06240.1
Potra2n12c23855.1	0.65624299	0.228617893	0.00308957	0.915339763	Potri.012G014200.1	AT5G53390.1
Potra2n12c23856.1	0.431926962	0.017929504	0.006069208	0.986636967	Potri.012G014100.2	AT5G53400.1
Potra2n12c23857.1	0.92641307	-0.046370319	NA	NA		
Potra2n12c23858.1	-0.432722776	-0.228609504	0.159064323	0.893240619	Potri.012G014000.1	AT5G53420.1
Potra2n12c23859.1	-0.267498676	-0.005562945	0.04440861	0.996032145	Potri.012G013900.1	AT4G27910.1
Potra2n12c23860.1	0.100835457	0.113269012	0.374365482	0.903934429	Potri.012G013800.1	AT5G53440.1

Potra2n12c23861.1	1.264151475	0.214838425	0.045106179	0.959881296		
Potra2n12c23862.1	-0.185379488	0.074977125	0.636924428	0.983446961		
Potra2n12c23863.1	4.116351634	1.943088886	0.012623516	NA	Potri.012G013700.1	AT4G01500.1
Potra2n12c23864.1					Potri.009G038100.1	AT1G78400.1
Potra2n12c23865.1	1.963471857	-3.089949151	0.147133535	NA	Potri.012G013501.1	AT4G01500.1
Potra2n12c23866.1						
Potra2n12c23867.1	1.578268123	NA	NA	NA	Potri.012G013000.1	AT4G01500.1
Potra2n12c23868.1	1.64774763	NA	NA	NA	Potri.T054900.1	AT1G43950.1
Potra2n12c23869.1					Potri.012G013000.1	AT4G01500.1
Potra2n12c23870.1	NA	1.363811008	NA	NA	Potri.012G013000.1	AT4G01500.1
Potra2n12c23871.1						
Potra2n12c23872.1					Potri.T054900.1	AT1G43950.1
Potra2n12c23873.1					Potri.012G013000.1	AT4G01500.1
Potra2n12c23874.1					Potri.012G012950.1	AT4G01500.1
Potra2n12c23875.1	NA	NA	NA	NA	Potri.T000633.1	AT3G09510.1
Potra2n12c23876.1					Potri.T054900.1	AT1G43950.1
Potra2n12c23877.3	1.664677056	1.363811008	NA	NA	Potri.T054700.1	AT1G43950.1
Potra2n12c23878.1	-2.295337707	-2.446267299	NA	NA		
Potra2n12c23879.1	0.373644983	0.264592665	0.001429093	0.433369183	Potri.012G012800.1	AT5G06160.1
Potra2n12c23880.1	0.351534285	0.550088598	0.456962186	0.695756198	Potri.012G012700.1	AT5G24170.1
Potra2n12c23881.3	6.50975237	0.256309899	4.06672E-63	0.976302215	Potri.012G012100.1	
Potra2n12c23882.1	1.64774763	NA	NA	NA		
Potra2n12c23883.1					Potri.012G012100.1	
Potra2n12c23884.1	0.53273108	0.323955214	0.81510772	NA	Potri.T053800.1	AT3G60470.1
Potra2n12c23885.1	-1.377465904	-0.505073751	0.691954081	NA		
Potra2n12c23886.1	2.870105119	-0.506273741	0.116169968	NA	Potri.012G011800.1	AT3G60470.1
Potra2n12c23887.1	-0.837113133	-2.128989755	0.665925773	NA	Potri.012G012100.1	
Potra2n12c23888.1	-0.336184386	0.327702447	NA	NA	Potri.T053800.1	AT3G60470.1
Potra2n12c23889.1	6.297502737	0.674963689	2.35836E-05	NA	Potri.012G011800.1	AT3G60470.1
Potra2n12c23890.1					Potri.T053600.1	AT3G60470.1
Potra2n12c23891.1						
Potra2n12c23892.1						

Potra2n12c23893.1	NA	NA	NA	NA		
Potra2n12c23894.1	0.82357019	NA	NA	NA	Potri.T053500.1	AT3G60470.1
Potra2n12c23895.1	1.74862186	2.569874007	0.354699865	0.418644133		
Potra2n12c23896.1	-0.22137631	0.157942095	0.938098085	NA	Potri.012G012100.1	
Potra2n12c23897.1	-1.35927387	0.110081123	NA	NA	Potri.T053800.1	AT3G60470.1
Potra2n12c23898.1	3.061176985	1.571529889	NA	NA	Potri.T053300.1	AT3G60470.1
Potra2n12c23899.1					Potri.012G012400.1	
Potra2n12c23900.1	2.632524198	NA	NA	NA		
Potra2n12c23901.1	2.315713269	2.876942638	NA	NA	Potri.012G012100.1	
Potra2n12c23902.1	0.172764567	0.026712991	0.928123604	NA	Potri.T053800.1	AT3G60470.1
Potra2n12c23903.1	3.80981895	3.215761253	0.084004775	NA	Potri.T053300.1	AT3G60470.1
Potra2n12c23904.1						
Potra2n12c23905.1	0.028121076	0.353844322	0.976449721	0.948237589	Potri.012G012700.1	AT5G24170.1
Potra2n12c23906.1	0.82357019	1.363811008	NA	NA	Potri.012G012100.1	
Potra2n12c23907.1	NA	NA	NA	NA	Potri.012G012400.1	
Potra2n12c23908.1	0.134694647	-0.130329886	0.967487218	NA		
Potra2n12c23909.1	NA	NA	NA	NA	Potri.T053500.1	AT3G60470.1
Potra2n12c23910.1	NA	NA	NA	NA		
Potra2n12c23911.1	0.82357019	NA	NA	NA		
Potra2n12c23912.3	0.345352777	0.267711984	0.017430083	0.76726123	Potri.012G011700.2	AT5G53460.3
Potra2n12c23913.2	0.144097995	0.137980187	0.374365482	0.895474078	Potri.012G011600.2	AT5G24165.1
Potra2n12c23914.1	2.338376306	0.802599013	4.67298E-32	0.176166373	Potri.T136300.1	AT5G53110.1
Potra2n12c23915.1	4.386061586	2.676364087	0.077293027	NA	Potri.002G020400.1	AT4G38660.1
Potra2n12c23916.1	-0.174300063	-0.016101248	0.027688285	0.98177802	Potri.012G003700.2	AT5G10270.1
Potra2n12c23917.1	1.396875645	0.260811189	7.72627E-19	0.607885566	Potri.012G003800.1	
Potra2n12c23918.1	5.689839016	3.013684852	7.54526E-22	0.077114891	Potri.012G003300.1	AT1G18390.2
Potra2n12c23919.1	NA	NA	NA	NA	Potri.012G002900.2	AT3G46400.1
Potra2n12c23920.2	2.09778301	0.643572835	5.72895E-08	0.810774554	Potri.012G002700.2	AT2G29380.1
Potra2n12c23921.1	0.005034707	-0.208807541	0.986348622	0.879275726	Potri.012G002600.7	AT5G53120.4
Potra2n12c23922.1	0.709834181	0.398150765	7.07027E-05	0.669679648	Potri.012G002500.1	AT3G49160.1
Potra2n12c23923.1	0.903840443	-0.227063605	0.012851235	0.914903229		
Potra2n12c23924.2	0.590458367	0.283864745	8.6516E-05	0.663950227	Potri.012G002200.1	AT5G53130.1

Potra2n12c23925.3	-0.453782229	0.011363267	4.11552E-06	0.993485167	Potri.012G002100.12	AT5G53140.1
Potra2n12c23926.1	NA	NA	NA	NA		
Potra2n12c23927.1	-0.071862046	-0.014933153	0.727526633	0.99114586	Potri.012G001900.2	AT3G61700.2
Potra2n12c23928.1	-1.014154227	0.070989106	9.89999E-10	0.953954892	Potri.012G001700.2	AT2G46420.1
Potra2n12c23929.2	0.654874685	0.538274861	0.191443624	0.903496619	Potri.012G001500.2	AT3G22370.1
Potra2n12c23930.1	1.093975743	0.397829681	0.018194891	0.809195602	Potri.012G001400.1	AT5G61430.1
Potra2n12c23931.1	0.067540377	-0.284554609	0.957315974	0.956865545		
Potra2n12c23932.3	-0.369511766	-0.0920493	0.004128564	0.925184207	Potri.012G001100.1	AT5G53150.1
Potra2n12c23933.1	-0.192662091	-0.011102126	0.038949859	0.990128003	Potri.012G001000.3	AT1G35910.1
Potra2n12c23934.2	-0.176827868	0.013909183	0.054915351	0.985964146	Potri.012G000900.1	AT3G61690.1
Potra2n12c23935.1	-1.808298152	-0.003378094	0.521399076	NA		
Potra2n12c23936.1	-0.42238102	-0.100516795	0.045106179	0.937846405	Potri.012G000800.1	AT5G53160.2
Potra2n12c23937.1	-0.516223367	-0.145295032	0.000288043	0.843683654	Potri.012G000700.3	AT5G53170.1
Potra2n12c23938.1	0.044885132	0.021762854	0.689349312	0.97601212	Potri.012G029300.1	AT5G53180.1
Potra2n12c23939.1	0.119216406	-0.943850448	0.935760818	0.78923506	Potri.012G029900.1	
Potra2n12c23940.1	-0.182703356	-0.361894436	0.804298484	0.909675463	Potri.012G000400.1	AT1G51730.1
Potra2n12c23941.1	1.111251874	1.26426115	0.020296466	0.250156624	Potri.012G030301.1	
Potra2n12c23942.1	0.087124794	-0.757196931	NA	NA	Potri.012G030150.1	
Potra2n12c23943.1	2.409567423	1.906059102	NA	NA		
Potra2n12c23944.1	1.459962178	NA	NA	NA	Potri.012G030600.1	
Potra2n12c23945.1	0.178316783	0.41189486	0.667149704	0.812696706	Potri.012G029500.1	
Potra2n12c23946.1	-0.111926505	0.48773048	0.938532223	0.957525726		
Potra2n12c23947.1	0.314154334	2.583469412	0.872571062	0.357747948	Potri.012G000400.1	AT1G51730.1
Potra2n12c23948.1	0.164979063	0.10061593	0.94873602	NA	Potri.012G029400.1	AT1G51730.1
Potra2n12c23949.1					Potri.012G029600.1	
Potra2n12c23950.1					Potri.012G029900.1	
Potra2n12c23951.1	-0.02707021	0.281431205	0.985467958	0.965249957	Potri.012G030800.1	AT1G51730.1
Potra2n12c23952.1	NA	1.571529889	NA	NA	Potri.012G029700.1	
Potra2n12c23953.1	-2.610763384	-1.037609126	0.325849939	NA	Potri.012G029600.1	
Potra2n12c23954.1	0.934999004	0.390079635	0.051095562	0.897285619	Potri.012G030301.1	
Potra2n12c23955.1					Potri.012G030600.1	
Potra2n12c23956.1	-0.06326252	-0.028064231	0.900340077	0.993577196	Potri.012G029500.1	

Potra2n12c23957.1	0.232896355	0.198992129	0.577072229	0.918757695	Potri.012G000400.1	AT1G51730.1
Potra2n12c23958.1	-1.486169567	0.03331384	0.263631896	0.996032145	Potri.012G030600.1	
Potra2n12c23959.1	0.021700159	0.255008021	0.972081219	0.912666272	Potri.012G000400.1	AT1G51730.1
Potra2n12c23960.1	2.149166949	1.251310454	0.002393612	0.636765909		
Potra2n12c23961.1	NA	NA	NA	NA		
Potra2n12c23962.1					Potri.012G000400.1	AT1G51730.1
Potra2n12c23963.1	-0.61910233	2.581340402	NA	NA	Potri.012G000500.1	
Potra2n12c23964.1	NA	NA	NA	NA	Potri.012G031000.1	AT5G24130.1
Potra2n12c23965.1	-0.735076042	0.073618609	0.00038983	0.961670176	Potri.012G031100.1	AT5G24120.1
Potra2n12c23966.1	1.084566084	1.493883946	0.198771159	0.648132513	Potri.012G031200.2	AT5G53200.1
Potra2n12c23967.1	-0.824619939	-1.130864772	0.718917385	NA	Potri.015G021500.2	AT5G53190.1
Potra2n12c23968.2	0.067538598	-0.01278103	0.740596912	0.992934677	Potri.012G031600.1	AT5G16420.1
Potra2n12c23969.1	4.640771426	1.919059254	3.55702E-16	0.500705998	Potri.012G031700.1	AT5G24110.1
Potra2n12c23970.1	NA	NA	NA	NA	Potri.012G031800.1	AT5G53210.1
Potra2n12c23971.1	-0.20639212	-0.503068022	0.743112579	0.842247476	Potri.012G031900.1	AT5G41761.1
Potra2n12c23972.1	-0.774388233	-1.220885712	0.637275271	NA	Potri.012G032000.1	AT3G61640.1
Potra2n12c23973.3	-0.297899665	0.064447698	0.218372196	0.972159498	Potri.012G032100.1	AT4G27940.1
Potra2n12c23974.1	-0.304620502	-0.198308584	0.223631693	0.868051677	Potri.012G032200.1	AT5G53280.1
Potra2n12c23975.1	NA	2.283425176	NA	NA	Potri.012G032300.1	AT5G15290.1
Potra2n12c23976.1	-0.377361315	0.114210272	0.424076184	0.966323513		
Potra2n12c23977.1	1.671720939	0.835677739	2.47278E-11	0.394938299	Potri.001G167300.1	AT3G14067.1
Potra2n12c23978.1	3.805061805	1.485304556	3.26006E-43	0.233903684	Potri.012G032400.1	AT3G14470.1
Potra2n12c23979.1	-1.281657171	2.767651847	0.478836282	0.236975465	Potri.012G032500.1	AT3G57450.1
Potra2n12c23980.2	-1.033042416	0.608324308	0.53482605	0.917516356	Potri.010G213500.1	
Potra2n12c23981.1	0.015227501	-0.397146331	0.973296382	0.83433596	Potri.012G032700.1	AT1G74100.1
Potra2n12c23982.1	-0.210666656	-0.165098171	0.840790911	0.978646026		
Potra2n12c23983.1	0.916550384	-0.314826826	0.017620141	0.911749053	Potri.012G032900.1	AT4G27950.1
Potra2n12c23984.1	0.048783454	-0.068176415	0.669612068	0.940503323	Potri.012G033000.1	AT5G53300.4
Potra2n12c23985.1						
Potra2n12c23986.1	-0.205020576	-0.063610002	0.255799783	0.968979184	Potri.012G033100.1	AT5G53310.1
Potra2n12c23987.1	1.348575115	0.445032235	2.65052E-21	0.492230064	Potri.012G033200.1	AT4G23740.1
Potra2n12c23988.1	0.179542891	0.090608626	0.300561514	0.932932656		

Potra2n12c23989.1	0.060861685	-0.087352252	0.614933521	0.885375133	Potri.012G033300.1	AT5G53330.1
Potra2n12c23990.1	5.664156372	2.321230577	3.92952E-11	0.202843914	Potri.012G033500.1	AT3G59140.1
Potra2n12c23991.1	-0.314802402	-0.180045141	0.271194523	0.937687311	Potri.012G033700.1	AT5G52960.1
Potra2n12c23992.1	0.518989728	-0.056419758	0.06594536	0.978203092	Potri.012G033800.1	AT5G52950.1
Potra2n12c23993.1	7.770408542	2.807515318	4.2202E-11	NA	Potri.012G033866.1	AT5G24090.1
Potra2n12c23994.1	-0.001288805	0.002504216	0.995976473	0.999315506	Potri.012G034000.1	AT5G52920.1
Potra2n12c23995.1	0.075043792	-0.184787231	0.902208478	0.94608223	Potri.012G033932.1	AT5G24090.1
Potra2n12c23996.1	-0.236853069	-0.1295467	0.69334392	0.971828097		
Potra2n12c23997.1	-0.248300764	-0.343352177	0.402823147	0.824905557	Potri.012G034100.1	AT2G22590.1
Potra2n12c23998.2	0.417902928	0.222166256	0.00053694	0.776094736	Potri.012G034200.1	AT3G26560.1
Potra2n12c23999.1	0.644773877	0.404397387	1.0068E-09	0.522127498	Potri.012G034300.1	AT5G65770.1
Potra2n12c24000.1	0.757320854	0.471368175	1.71207E-06	0.164277499	Potri.015G024600.1	AT2G34930.1
Potra2n12c24001.1	0.095663512	0.226765855	0.527468932	0.684244573	Potri.012G034800.1	AT2G41620.1
Potra2n12c24002.1	-1.186998678	-0.720779169	0.114914205	0.8043603	Potri.015G026300.1	AT5G24080.1
Potra2n12c24003.1						
Potra2n12c24004.1						
Potra2n12c24005.2	1.879061959	-0.110700569	1.70905E-16	0.972159498	Potri.012G035200.2	AT5G52900.1
Potra2n12c24006.1	NA	NA	NA	NA		
Potra2n12c24007.1	-0.449496258	-0.16501991	0.102999854	0.943826727	Potri.012G035400.2	AT4G28000.1
Potra2n12c24008.1	-0.13466807	-0.110113367	0.424211593	0.903698951	Potri.012G035500.1	AT5G52880.1
Potra2n12c24009.1	2.549191961	3.302674971	NA	NA	Potri.012G035600.1	AT4G23630.1
Potra2n12c24010.1	0.419704888	-0.07482662	0.185792377	0.980843634	Potri.012G035700.1	AT5G52870.1
Potra2n12c24011.3	-0.428747274	-0.054113896	0.000121587	0.95671968	Potri.012G035800.1	AT3G16520.3
Potra2n12c24012.1	-0.332470886	-0.018028475	0.009407481	0.985393296	Potri.012G035900.3	AT3G19180.1
Potra2n12c24013.1	-0.61910233	-0.767708831	NA	NA	Potri.010G022900.1	
Potra2n12c24014.1	-0.020413714	-0.104195346	0.920094019	0.930306937	Potri.012G036200.1	AT5G23250.1
Potra2n12c24015.1	0.301880329	0.441061367	0.033856676	0.497568278	Potri.012G036300.1	AT1G34130.1
Potra2n12c24016.3	-0.484753762	-0.002581744	0.076196149	0.999325699	Potri.012G036400.1	
Potra2n12c24017.3	-0.524556354	-0.124350165	0.002009119	0.934579595	Potri.012G036500.1	AT5G20600.1
Potra2n12c24018.1	-0.105794661	0.002145184	0.476092488	0.999325699	Potri.012G036600.1	AT5G23130.1
Potra2n12c24019.1	0.033945786	-0.066866645	0.835817481	0.953608209	Potri.012G036700.1	AT3G19950.1
Potra2n12c24020.1	0.265880239	-0.017516695	0.214730525	0.992508835	Potri.012G036800.1	AT1G55805.1

Potra2n12c24021.1	3.490733385	3.633011854	0.395449675	NA	Potri.012G036900.1	AT3G17660.1
Potra2n12c24022.1	-0.083054247	-0.568911314	0.933277265	0.900869139	Potri.015G028700.1	AT1G61260.1
Potra2n12c24023.1	-0.075253732	0.135091241	0.565437769	0.82104194	Potri.012G037100.1	AT1G48410.1
Potra2n12c24024.1	0.798611938	-0.523619619	0.397296572	0.920028955	Potri.012G037200.1	AT1G73370.1
Potra2n12c24025.2	-2.300709737	-0.103658748	8.84744E-05	0.974275337	Potri.012G037300.1	AT1G14590.1
Potra2n12c24026.1	-3.089185541	-0.647997865	0.000774284	0.885375133	Potri.012G037400.1	AT1G73220.1
Potra2n12c24027.1	0.851447214	0.111851494	1.70679E-06	0.943819961	Potri.004G078000.1	AT3G16780.1
Potra2n12c24028.2	0.676390205	-0.009492735	0.026981234	0.997932292	Potri.012G037700.1	AT3G07930.3
Potra2n12c24029.1					Potri.012G037800.1	AT4G03140.1
Potra2n12c24030.1	0.061201567	-0.033807538	0.740749106	0.977559521	Potri.012G037900.1	AT1G17880.1
Potra2n12c24031.1	-0.031452342	-1.295530424	0.973759782	0.700935593	Potri.015G030100.1	AT1G48405.1
Potra2n12c24032.1	1.482774152	0.726419539	0.590676919	NA	Potri.012G038100.3	AT3G17730.1
Potra2n12c24033.1	0.067946363	-0.163505688	0.761069366	0.89438295	Potri.012G038200.1	AT1G73530.1
Potra2n12c24034.1	0.109594558	0.544578786	0.625997133	0.71180938	Potri.012G038300.2	AT1G18330.1
Potra2n12c24035.1	4.194674967	1.421854748	1.39147E-09	0.506597964	Potri.012G038400.1	AT2G27660.1
Potra2n12c24036.1	-1.877010706	-0.478516139	7.25012E-05	0.842247476	Potri.012G038500.1	AT1G73360.1
Potra2n12c24037.1	-0.29868259	-0.156293091	0.049139951	0.817452127	Potri.012G038600.1	AT4G17010.1
Potra2n12c24038.4	0.39569742	0.121627713	0.579262926	0.973347974	Potri.012G038700.1	AT3G17700.1
Potra2n12c24039.1	0.771059752	0.154989824	0.391152842	0.985393296	Potri.012G038800.1	AT2G35795.1
Potra2n12c24040.1	1.414132828	0.017979585	0.205199604	NA	Potri.012G038800.1	AT2G35795.1
Potra2n12c24041.1	0.350925977	0.23369933	0.00199267	0.635323636	Potri.012G038900.1	AT1G48420.1
Potra2n12c24042.1	-1.251835992	-0.363282982	1.48851E-05	0.412949064	Potri.012G039000.2	AT3G17760.2
Potra2n12c24043.1	0.477493547	-0.094282824	0.004156129	0.951012317	Potri.012G039100.1	AT3G46560.1
Potra2n12c24044.1	0.724888042	0.34966947	2.07401E-29	0.327435845	Potri.012G039200.1	AT1G76810.1
Potra2n12c24045.1	1.060529719	-0.452319082	0.033552526	0.840807554	Potri.012G039300.1	AT5G22110.1
Potra2n12c24046.2	-0.406229051	-0.18696499	0.116187901	0.924951347	Potri.012G039400.1	AT1G17950.1
Potra2n12c24047.1	1.459962178	NA	NA	NA	Potri.007G124150.1	AT1G54380.1
Potra2n12c24048.1					Potri.004G205800.1	AT2G02160.1
Potra2n12c24049.1	-0.368263778	0.02672366	0.007933686	0.983944804	Potri.012G039700.1	AT1G73450.1
Potra2n12c24050.1	0.104529594	0.483510381	0.877382559	0.771626158	Potri.012G042300.1	AT2G01970.1
Potra2n12c24051.1	-0.010079628	0.627420066	0.981204603	0.314466151	Potri.012G039901.1	AT1G59960.1
Potra2n12c24052.1	-0.272291538	-0.218186558	0.3746635	0.912044974	Potri.012G040200.1	AT2G01940.1

Potra2n12c24053.1	-0.202324954	-0.020095581	0.156158085	0.98177802	Potri.012G040600.1	AT2G01930.1
Potra2n12c24054.1	-1.35927387	-1.507841271	NA	NA	Potri.004G035100.2	AT5G57580.1
Potra2n12c24055.1	0.5389099	0.187119996	8.19061E-05	0.767368598	Potri.012G040400.1	AT2G01930.1
Potra2n12c24056.1	-0.749823207	-2.446267299	NA	NA	Potri.004G117600.1	
Potra2n12c24057.1	0.23444036	-0.112806081	0.138543966	0.917751882	Potri.012G041000.1	AT5G37780.1
Potra2n12c24058.1	-3.247937753	-1.057340482	0.004538898	0.811931525	Potri.012G040900.1	AT1G75240.1
Potra2n12c24059.1	-0.079911649	-0.15577257	0.66175142	0.865591169	Potri.012G041100.1	AT3G17770.1
Potra2n12c24060.1	0.341781819	0.061541619	0.422995745	0.985393296	Potri.012G041200.1	AT5G16860.1
Potra2n12c24061.1	-1.725005586	1.051121491	0.328340572	0.826489083	Potri.002G120900.1	AT5G56510.1
Potra2n12c24062.1	0.05183228	0.040953751	0.810641548	0.976302215		
Potra2n12c24063.1	-0.110924923	0.08093073	0.560821269	0.943411075	Potri.012G041400.3	AT1G18335.1
Potra2n12c24064.1	NA	NA	NA	NA	Potri.012G041500.1	AT2G25540.1
Potra2n12c24065.1					Potri.012G041500.1	AT2G25540.1
Potra2n12c24066.1					Potri.012G041600.1	AT2G01900.1
Potra2n12c24067.1	0.569686837	0.480861028	0.001004185	0.582419816	Potri.012G041700.1	AT3G58130.2
Potra2n12c24068.1	0.730773659	0.535904694	0.00012507	0.574820121	Potri.012G041800.1	AT1G73480.1
Potra2n12c24069.2	0.177672611	-0.022674373	0.495008007	0.988601759	Potri.012G041900.2	AT1G68090.1
Potra2n12c24070.1	1.237448707	-0.608103366	0.195273847	0.920028955		
Potra2n12c24071.1	1.869562378	0.812597276	0.002668837	0.745183467	Potri.012G042100.1	AT5G16560.1
Potra2n12c24072.1	-0.398758391	0.063040712	0.310062176	0.982221696	Potri.012G042200.1	AT3G17790.1
Potra2n12c24073.1	1.950055668	1.041758891	1.3954E-06	0.19180022	Potri.012G042300.1	AT2G01970.1
Potra2n12c24074.1	0.148484204	-0.100033914	0.344947242	0.910878765	Potri.012G042400.1	AT1G48450.1
Potra2n12c24075.1	-0.008387065	0.146977963	0.940160916	0.660636854	Potri.012G042500.1	AT1G31930.3
Potra2n12c24076.1	6.182457669	3.010689165	7.84122E-05	NA	Potri.012G042600.1	AT5G37490.1
Potra2n12c24077.1	0.162458843	0.129060795	0.383895751	0.910815461	Potri.012G042700.2	AT1G48440.1
Potra2n12c24078.1	NA	NA	NA	NA	Potri.012G042800.1	AT1G05260.1
Potra2n12c24079.3	-0.457827893	-0.10244728	0.015077377	0.935851032	Potri.012G043000.1	AT3G02850.1
Potra2n12c24080.1	1.692581666	0.656123518	0.000325992	0.775519829	Potri.012G043200.1	AT1G73500.1
Potra2n12c24081.1	-1.360567531	-3.043858706	0.723815711	NA	Potri.015G030600.1	AT3G17810.1
Potra2n12c24082.2	1.805702146	0.122898849	0.49717051	NA	Potri.012G043400.1	AT1G14760.2
Potra2n12c24083.1	0.896003577	0.554452192	0.035552487	0.859892315	Potri.012G043501.1	AT2G40540.2
Potra2n12c24084.1	0.780505952	0.170471464	0.012545742	0.947211418	Potri.012G043501.1	AT2G40540.2

Potra2n12c24085.1	0.190938113	-0.016327916	0.027040289	0.985393296	Potri.012G043700.1	AT1G10170.1
Potra2n12c24086.1	0.498972954	0.190274689	2.77945E-06	0.621599038	Potri.012G043800.1	AT4G36480.2
Potra2n12c24087.1	-0.057835454	-0.13595414	0.553819364	0.759797728	Potri.012G043900.1	AT5G37600.1
Potra2n12c24088.1	1.098567672	-0.540491563	0.200790937	0.948834049		
Potra2n12c24089.1	0.478132121	0.074149302	0.01985697	0.985016122	Potri.012G044300.2	AT1G18300.1
Potra2n12c24090.4	1.435285833	-0.520869668	9.17702E-05	0.771173235	Potri.012G044400.1	AT4G37110.1
Potra2n12c24091.1	-0.212951889	-0.273425558	0.685892515	0.911339141	Potri.012G044400.1	AT4G37110.1
Potra2n12c24092.1	-0.863123609	-0.204229377	1.32262E-05	0.850863374	Potri.012G044600.1	AT1G48480.1
Potra2n12c24093.1	-0.096618069	0.144895178	0.448249256	0.835819291	Potri.015G035600.1	AT3G17850.1
Potra2n12c24094.1	-0.890543827	-0.34737321	2.35227E-07	0.247665016	Potri.012G044900.1	AT3G17860.1
Potra2n12c24095.1	-0.398287673	-0.027967522	0.073935573	0.9896961	Potri.012G045000.2	AT3G17880.1
Potra2n12c24096.1	-1.336793218	-1.48500824	NA	NA	Potri.012G045100.1	AT2G37360.1
Potra2n12c24097.1	0.385284221	0.196582923	0.113761339	0.926808461	Potri.012G045200.1	AT3G17910.1
Potra2n12c24098.1	-0.275558917	-0.074340498	0.053487502	0.948577865	Potri.012G045300.1	AT3G17920.1
Potra2n12c24099.1	NA	NA	NA	NA	Potri.012G045400.1	
Potra2n12c24100.1	-0.193367265	-0.046000145	0.097637341	0.956865545	Potri.012G045500.1	AT1G48160.1
Potra2n12c24101.1	NA	NA	NA	NA	Potri.012G045600.1	AT1G18290.1
Potra2n12c24102.1	-0.56411764	-0.446770195	0.00031097	0.083053039		
Potra2n12c24103.1	0.086493071	0.012869673	0.341671974	0.992934677	Potri.012G045800.1	AT2G33730.1
Potra2n12c24104.1	-0.102476014	-0.127576639	0.596915674	0.928470728	Potri.006G037300.1	AT3G17940.1
Potra2n12c24105.1	-1.317292482	-1.465929376	NA	NA	Potri.006G260300.1	
Potra2n12c24106.1	0.186694433	-0.086778079	0.586688493	0.953177702	Potri.012G046000.1	AT3G18240.2
Potra2n12c24107.1	NA	0.674963689	NA	NA	Potri.012G046100.1	AT1G78640.1
Potra2n12c24108.2	0.019954394	0.065858411	0.889475637	0.930598202	Potri.012G046300.1	AT1G18260.1
Potra2n12c24109.1	0.288259483	0.130234301	0.064316844	0.862830524	Potri.012G046400.1	AT5G06310.1
Potra2n12c24110.2	0.83313334	0.019819277	0.00246263	0.994403137	Potri.012G046500.1	AT1G48570.1
Potra2n12c24111.1	-0.153302177	-0.134236203	0.355844275	0.921099436	Potri.012G046600.1	AT5G42150.1
Potra2n12c24112.1	0.293554903	0.342201583	0.329982566	0.779077802	Potri.012G046700.1	AT5G07900.1
Potra2n12c24113.1	0.907538437	0.668117634	2.41339E-06	0.008032632	Potri.002G131900.1	
Potra2n12c24114.1	1.26931246	0.885863424	1.67582E-07	0.005453789	Potri.002G131750.1	
Potra2n12c24115.1	0.267680632	0.111023099	0.186626607	0.939653927	Potri.012G046800.1	AT5G07900.1
Potra2n12c24116.1	-0.145391773	0.048062608	0.259868462	0.957163493	Potri.012G046900.1	AT3G17970.1

Potra2n12c24117.2	0.37626642	0.229413073	0.487402665	0.939824856	Potri.012G047000.2	AT1G48580.1
Potra2n12c24118.1	-0.115713722	-0.210761014	0.761069366	0.907073354	Potri.012G047100.1	AT3G17980.1
Potra2n12c24119.1	-0.554767934	-0.150830087	0.123710822	0.954559968	Potri.012G047200.2	AT1G73590.1
Potra2n12c24120.1	-0.002186439	-0.288684616	0.995345479	0.87062349	Potri.012G047400.1	AT1G48600.2
Potra2n12c24121.1	NA	NA	NA	NA	Potri.012G047600.1	AT5G12250.1
Potra2n12c24122.1	NA	NA	NA	NA	Potri.012G047700.1	AT3G18010.1
Potra2n12c24123.1	NA	1.363811008	NA	NA		
Potra2n12c24124.1	-2.467120255	0.467613484	0.125245916	0.936549944		
Potra2n12c24125.1	1.131872593	1.635840724	0.522527673	0.788065047	Potri.012G048000.1	AT3G14470.1
Potra2n12c24126.1	0.773981384	0.132183752	0.057227547	0.946472435	Potri.012G048200.1	AT1G18210.1
Potra2n12c24127.1	-0.560383295	0.099709081	0.360543197	0.979896883	Potri.012G048300.1	AT1G18170.1
Potra2n12c24128.4	0.055914375	0.196103994	0.720816161	0.714084948	Potri.012G048400.1	AT1G73660.1
Potra2n12c24129.1	-0.302840462	-0.118211649	0.015285087	0.858174358	Potri.012G048500.1	AT1G48620.1
Potra2n12c24130.1	-0.349275538	0.019555939	0.036827632	0.989304496	Potri.012G048600.2	AT3G18040.1
Potra2n12c24131.1	-0.289828603	-0.081427642	0.452988772	0.982422288	Potri.012G048800.1	AT4G02750.1
Potra2n12c24132.1	1.64774763	NA	NA	NA	Potri.012G048900.1	AT1G18140.1
Potra2n12c24133.1	-0.759078027	-0.116958158	0.003800813	0.953608209	Potri.012G049000.1	AT3G18050.1
Potra2n12c24134.1	-0.096940821	0.102583205	0.3760641	0.893394174	Potri.012G049200.1	AT3G18060.1
Potra2n12c24135.2	-0.489295461	-0.157451652	0.141530796	0.940856252	Potri.015G041300.2	AT3G18080.1
Potra2n12c24136.1	1.147075632	0.837905211	NA	NA	Potri.012G049500.1	AT1G73680.1
Potra2n12c24137.1	0.756744925	0.14191667	1.45166E-07	0.901195443	Potri.012G049600.1	AT1G18080.1
Potra2n12c24138.1	-0.258937781	-0.047665554	0.067255808	0.966501596	Potri.012G049700.1	AT1G48635.1
Potra2n12c24139.1	-1.328951947	-1.478536539	NA	NA	Potri.012G049900.1	AT1G15125.1
Potra2n12c24140.1	-0.980988339	-0.470455046	0.000170876	0.303693795	Potri.012G050000.1	AT1G18060.1
Potra2n12c24141.1	6.395849324	NA	3.1334E-06	NA	Potri.012G052200.1	AT2G29420.1
Potra2n12c24142.2	0.027383127	0.017095451	0.89676902	0.989329144	Potri.012G050300.1	AT1G18040.1
Potra2n12c24143.1	0.485086181	0.163951987	0.002674919	0.842247476	Potri.012G050400.1	AT4G35410.2
Potra2n12c24144.2	6.403889563	2.953573663	2.27742E-59	0.066662721	Potri.012G051900.1	AT5G52450.1
Potra2n12c24145.1	0.135515374	-0.114960142	NA	NA	Potri.012G051700.1	AT3G18180.1
Potra2n12c24146.1	-1.314575584	-0.318435374	4.51465E-07	0.859892315	Potri.012G051500.1	AT3G18170.1
Potra2n12c24147.1	0.205104685	0.004103563	0.106445255	0.996032145	Potri.012G051300.1	AT3G18190.1
Potra2n12c24148.1	-0.169807907	0.060168887	0.601438697	0.974264269	Potri.012G051200.1	AT3G18210.1

Potra2n12c24149.1	-1.624315147	0.479487209	0.247170423	0.937846405		
Potra2n12c24150.1	1.459962178	NA	NA	NA	Potri.012G052950.1	
Potra2n12c24151.1	-0.153007059	-0.034669383	0.398846301	0.973347974	Potri.012G053200.1	AT1G27170.2
Potra2n12c24152.1	3.835856331	NA	0.078976319	NA		
Potra2n12c24153.1	-0.961588533	-1.207593603	0.46810353	0.816392132	Potri.012G053400.1	
Potra2n12c24154.1	NA	1.571529889	NA	NA	Potri.012G053500.1	AT2G01190.1
Potra2n12c24155.2	0.058262561	-0.182135057	0.816904713	0.917510096	Potri.012G053600.1	AT1G73740.1
Potra2n12c24156.1	-0.349661779	0.157408323	0.036700422	0.900866967	Potri.012G053700.1	AT1G15060.1
Potra2n12c24157.1	0.082897754	-0.092960158	0.594603004	0.927423678	Potri.012G053800.1	AT1G73760.1
Potra2n12c24158.1	0.625370609	0.77425684	0.415199011	0.724604666	Potri.008G152701.1	AT3G22425.2
Potra2n12c24159.1	1.366641944	1.242415692	0.109812176	0.558277497	Potri.004G205200.1	AT4G38280.1
Potra2n12c24160.1	-2.024277535	-0.648359268	5.93389E-07	0.650097433	Potri.012G054000.1	AT1G68238.1
Potra2n12c24161.1	1.664677056	1.394613675	NA	NA	Potri.012G054100.1	AT3G18260.1
Potra2n12c24162.1	-0.243803172	-0.077098566	0.043637291	0.936549944	Potri.015G044401.1	AT3G18270.1
Potra2n12c24163.2	-0.617627344	-0.212908379	0.189371462	0.954419252	Potri.012G054400.3	AT1G18370.1
Potra2n12c24164.1	-0.757581356	-0.222548097	0.16280017	0.955198964	Potri.012G054300.1	AT3G18280.1
Potra2n12c24165.1	4.294812104	1.633540402	2.11859E-18	0.586594557	Potri.012G054550.1	AT5G38260.1
Potra2n12c24166.1	3.84575401	1.46893301	1.41421E-08	0.700697262	Potri.012G054700.1	AT1G18390.1
Potra2n12c24167.1	3.33570075	0.405778224	5.42987E-11	0.933037048	Potri.012G054700.1	AT1G18390.1
Potra2n12c24168.1	4.716260716	2.201595876	5.25999E-11	0.498403695	Potri.012G054550.1	AT5G38260.1
Potra2n12c24169.1	7.572578695	4.010187478	2.39658E-18	0.170793744	Potri.012G054900.1	AT1G73805.1
Potra2n12c24170.1	-0.242314234	0.09879747	0.076725212	0.910074792	Potri.015G045600.1	AT1G48760.2
Potra2n12c24171.1	-1.501002626	-0.008815757	5.72912E-07	0.99736971	Potri.012G055100.1	AT3G18290.1
Potra2n12c24172.1	0.267789721	0.241989292	0.375841505	0.849790859	Potri.012G055200.1	AT1G25370.1
Potra2n12c24173.1	-0.569137664	-0.643133899	0.08337186	0.579121694	Potri.012G055300.1	AT1G48780.1
Potra2n12c24174.1					Potri.012G055400.1	AT1G48790.1
Potra2n12c24175.4	0.631807525	0.2499252	2.25443E-06	0.736000077	Potri.012G055500.1	AT4G23180.1
Potra2n12c24176.1	3.318721072	1.683194933	0.000473142	NA	Potri.012G055600.1	AT1G68320.1
Potra2n12c24177.1	-0.717849406	0.051148565	0.540268379	0.995579852	Potri.012G055700.1	AT1G73830.1
Potra2n12c24178.1	-0.398065517	0.178365809	0.004685636	0.926808461	Potri.012G055800.3	AT1G48840.1
Potra2n12c24179.1	3.739225906	NA	0.333587171	NA	Potri.012G055900.1	AT3G18360.1
Potra2n12c24180.3	0.875305633	0.384752704	2.48855E-09	0.233903684	Potri.012G056000.1	AT3G18370.1

Potra2n12c24181.1	-0.475342316	0.92556264	0.809709349	0.866777645	Potri.003G000900.2	AT5G49880.1
Potra2n12c24182.3	-0.243459438	0.09174187	0.029945909	0.936625864	Potri.012G056100.1	AT3G18390.1
Potra2n12c24183.1	0.097183811	0.05368887	0.462346407	0.960981446	Potri.012G056200.1	AT1G73840.1
Potra2n12c24184.1	3.628027076	-0.786502229	0.050869008	NA	Potri.012G056300.2	AT3G18400.1
Potra2n12c24185.2	0.728773836	-0.082353788	0.581899648	NA	Potri.012G056400.1	AT1G73850.1
Potra2n12c24186.1	-0.121683913	0.551133353	0.801539088	0.824317405	Potri.012G056500.1	AT1G68490.1
Potra2n12c24187.1	-2.43316869	0.047262842	0.390584346	NA	Potri.012G056700.2	AT1G63640.1
Potra2n12c24188.1	2.029303878	-0.444857694	3.47334E-07	0.874648591	Potri.012G056800.1	AT3G02550.1
Potra2n12c24189.1	-1.611007907	-0.921505318	0.29615337	0.842247476		
Potra2n12c24190.1	-0.055252397	-0.121423462	0.75580414	0.832211114		
Potra2n12c24191.1	-0.614046831	-0.189987031	0.44065742	0.96670549	Potri.012G058400.2	AT1G18550.1
Potra2n12c24192.3	-0.000690807	0.179056843	0.997296738	0.845379753	Potri.012G057100.1	AT1G73875.1
Potra2n12c24193.1	1.871013399	1.736020639	0.000834424	0.466145447	Potri.017G083500.1	AT5G15240.1
Potra2n12c24194.1	0.100304226	0.089246272	0.458313135	0.943411075	Potri.012G057300.1	AT5G08160.1
Potra2n12c24195.2	0.122657578	0.143772354	0.452694809	0.880316339	Potri.012G057400.1	AT1G18490.1
Potra2n12c24196.1	0.816359115	0.058588393	1.52228E-05	0.974275337	Potri.012G057500.2	AT1G74030.1
Potra2n12c24197.2	0.471277417	0.332360283	0.00223658	0.621832412	Potri.012G057700.1	AT5G23040.1
Potra2n12c24198.1	NA	NA	NA	NA	Potri.001G207400.1	
Potra2n12c24199.1	0.456125498	0.120281213	0.006222979	0.899585103	Potri.012G058000.2	AT1G68710.1
Potra2n12c24200.1	-0.156494265	0.134882628	0.197276554	0.882657052	Potri.012G058100.1	AT5G23080.1
Potra2n12c24201.3	-0.269661836	-0.09910716	0.004031962	0.895474078	Potri.012G058300.1	AT5G23100.1
Potra2n12c24202.1	0.039729477	-0.578871202	0.956225996	0.856119486	Potri.012G058400.2	AT1G18550.1
Potra2n12c24203.2	-0.255139841	0.153808555	0.325215163	0.933286951	Potri.015G052400.3	AT1G48950.1
Potra2n12c24204.1	-0.697999351	0.110248314	0.003463988	0.953831244	Potri.012G058700.3	AT1G74070.1
Potra2n12c24205.2	0.032052846	-0.266029681	0.884699231	0.788065047	Potri.012G058800.1	AT3G18580.1
Potra2n12c24206.1	-0.397846023	-0.172366054	0.005677621	0.88643982	Potri.012G058900.2	AT1G70330.1
Potra2n12c24207.2	-0.17114306	0.021646392	0.170859076	0.986636967	Potri.012G059000.4	AT2G42780.1
Potra2n12c24208.1	-1.196503206	0.564505445	0.012725814	0.817489719	Potri.012G059100.2	AT1G48960.1
Potra2n12c24209.1	2.030909952	NA	NA	NA	Potri.004G232250.1	AT5G49760.1
Potra2n12c24210.1	0.231045295	-0.010349013	0.029286809	0.9896961	Potri.012G059300.5	AT5G38640.1
Potra2n12c24211.3	0.061955262	-0.034706112	0.701567707	0.974275337	Potri.012G059200.1	AT1G18560.1
Potra2n12c24212.1	-0.939445553	0.976424739	0.288831192	0.802855012	Potri.012G059400.1	AT3G18570.1

Potra2n12c24213.1	-1.684020477	-0.292255268	6.79782E-09	0.888595254	Potri.012G059500.1	AT1G49000.1
Potra2n12c24214.1	-1.813228972	-0.51394572	4.57236E-05	0.592695906		
Potra2n12c24215.1	NA	2.283425176	NA	NA	Potri.012G059800.1	
Potra2n12c24216.1	-1.614271363	-0.081771426	0.01910382	0.987226805	Potri.012G059900.1	AT3G18550.2
Potra2n12c24217.1	3.521139008	NA	0.247651528	NA	Potri.002G080700.1	AT1G77410.1
Potra2n12c24218.1	-0.004234357	-0.034714131	0.986017666	0.982422288	Potri.012G060000.1	AT3G18524.1
Potra2n12c24219.1	1.664677056	NA	NA	NA		
Potra2n12c24220.1	-0.251947689	0.064728038	0.040882643	0.948002367	Potri.012G060100.1	AT1G18470.1
Potra2n12c24221.1	-0.769830868	0.270725009	0.782588293	0.983944804		
Potra2n12c24222.1	0.113844181	0.221617154	0.559124097	0.741833788	Potri.012G060200.1	AT1G73930.1
Potra2n12c24223.2	-0.126766033	0.033586384	0.233708824	0.962661188	Potri.012G060400.1	AT3G18520.1
Potra2n12c24224.1	-0.31385535	-0.1294883	0.025044034	0.857189843	Potri.012G060300.1	AT1G49010.1
Potra2n12c24225.1	-0.219059715	0.041769827	0.1950591	0.983944804	Potri.012G060600.1	AT1G73920.1
Potra2n12c24226.1	3.836841769	3.515276588	0.021078485	NA		
Potra2n12c24227.1	-1.328951947	-1.478536539	NA	NA		
Potra2n12c24228.1	0.154653516	0.453367494	0.652259042	0.351105764	Potri.014G039500.1	
Potra2n12c24229.1	-0.022007368	0.228577794	0.935969507	0.677013336	Potri.014G039400.1	
Potra2n12c24230.1	-0.895427184	-0.598695356	0.001660089	0.415978723	Potri.012G060700.1	AT1G29660.1
Potra2n12c24231.1	-0.089894736	-0.008163785	0.523052273	0.995779349	Potri.015G049500.1	AT1G49040.1
Potra2n12c24232.2	5.939301199	3.359745708	3.43318E-58	0.003092968	Potri.012G060951.1	AT1G49030.1
Potra2n12c24233.1	0.146071346	0.152914739	0.271706565	0.814981587	Potri.012G061000.2	AT3G18480.1
Potra2n12c24234.1	-0.021242145	-0.010375691	0.923938028	0.995261215	Potri.012G061100.1	AT1G18440.1
Potra2n12c24235.1	0.273716393	0.088529635	0.053080011	0.941855873	Potri.012G061300.1	AT1G18450.1
Potra2n12c24236.1	0.048147375	0.088739569	0.749836518	0.915580711	Potri.012G061400.4	AT5G07980.1
Potra2n12c24237.1					Potri.012G061500.1	AT3G02910.1
Potra2n12c24238.1					Potri.012G061600.1	AT5G61030.1
Potra2n12c24239.1	-0.193902725	-0.12377344	0.185396538	0.871072057	Potri.012G061900.4	AT1G74160.1
Potra2n12c24240.2	-0.128115098	0.038639167	0.179826321	0.954874132	Potri.012G062100.1	AT1G18610.1
Potra2n12c24241.1	-3.04142869	-3.185647093	NA	NA	Potri.012G062200.2	AT5G15630.1
Potra2n12c24242.1					Potri.014G189300.1	AT2G01050.1
Potra2n12c24243.2	-0.386206271	0.018128134	0.000248543	0.981772014	Potri.012G062300.1	AT5G60910.1
Potra2n12c24244.1					Potri.012G062400.1	

Potra2n12c24245.1	NA	NA	NA	NA	Potri.012G062400.1	
Potra2n12c24246.1	-0.023219796	0.252651937	0.880439254	0.741833788	Potri.012G062500.1	AT5G17400.1
Potra2n12c24247.1	-0.019868145	0.143795021	0.89776877	0.773063484	Potri.012G062700.1	AT3G25800.1
Potra2n12c24248.1	-0.343078118	0.287949254	0.229171055	0.798429987	Potri.012G063500.1	AT5G08110.1
Potra2n12c24249.1	-0.076498713	-0.023143337	0.491995844	0.983008617	Potri.012G064500.1	AT1G18580.1
Potra2n12c24250.1	-0.100351183	0.427370394	0.970837375	NA	Potri.017G063600.1	AT4G08480.1
Potra2n12c24251.1	-0.225413284	-0.150763271	0.913874321	NA	Potri.005G148100.1	AT5G09880.1
Potra2n12c24252.1						
Potra2n12c24253.1	-0.134540232	-0.124238779	0.452965046	0.898711301	Potri.012G065000.1	AT5G08130.2
Potra2n12c24254.1	-1.053924992	-0.911347916	0.008424542	0.564418199	Potri.012G065100.1	AT1G73880.1
Potra2n12c24255.1	0.38140196	0.071741288	0.075839958	0.943819961	Potri.012G065200.1	AT3G42660.1
Potra2n12c24256.1	-0.057708058	0.07656076	0.717473485	0.940841616	Potri.012G065300.1	AT5G08139.1
Potra2n12c24257.1	NA	1.381953902	NA	NA		
Potra2n12c24258.1	0.379962755	-0.188557958	0.101622801	0.874808359	Potri.012G065400.1	AT5G60840.1
Potra2n12c24259.1	-0.370095626	0.032659299	0.006919218	0.974367223	Potri.012G065500.1	AT5G07960.1
Potra2n12c24260.1	-0.073856671	0.218539791	0.85554311	0.913459614		
Potra2n12c24261.1	-0.05807596	0.047423196	0.532208489	0.935474933	Potri.012G065600.1	AT5G61150.2
Potra2n12c24262.1	-1.286922091	-0.531273944	1.9262E-06	0.554947759	Potri.012G065750.1	AT1G18650.1
Potra2n12c24263.1	-1.521478925	-0.616634064	9.57726E-12	0.318243807	Potri.012G065900.1	
Potra2n12c24264.1	1.64774763	1.571529889	NA	NA	Potri.010G013800.1	AT2G01050.1
Potra2n12c24265.1	0.118986337	-0.057199922	0.442259016	0.972159498	Potri.012G066000.1	AT1G74240.1
Potra2n12c24266.1	0.082949107	-0.045245398	0.61525839	0.966917036	Potri.012G066100.1	AT2G33040.1
Potra2n12c24267.1	-0.045526111	-0.087806372	0.816683064	0.941291229	Potri.012G066200.1	AT1G18640.2
Potra2n12c24268.1	-0.042645422	0.116273185	0.775099398	0.844277327	Potri.012G066300.1	AT5G07920.1
Potra2n12c24269.1	-0.526627811	0.501810818	0.892558039	NA	Potri.012G066250.1	
Potra2n12c24270.1	-0.076279077	0.03783218	0.666647061	0.959881296	Potri.012G066600.2	AT5G07980.1
Potra2n12c24271.1	5.156381053	1.422961162	1.08964E-20	0.80723942	Potri.012G066700.1	AT5G61210.1
Potra2n12c24272.2	0.219543723	0.310377895	0.105003587	0.601577719	Potri.012G066900.1	
Potra2n12c24273.1	0.652801236	0.150234184	3.53408E-06	0.875559093	Potri.012G067000.1	AT5G07810.1
Potra2n12c24274.1	-0.771369836	1.07714729	NA	NA	Potri.012G067100.2	AT5G15430.1
Potra2n12c24275.1	-0.387061807	-0.211586821	0.074561855	0.874808359	Potri.012G067200.1	AT3G29270.2
Potra2n12c24276.1	-1.35927387	-1.507841271	NA	NA	Potri.012G067400.1	AT5G07800.1

Potra2n12c24277.1	-1.098473623	-0.277335389	1.47942E-05	0.902847514	Potri.012G067500.1	AT5G07800.1
Potra2n12c24278.1	4.769749101	1.42215079	2.78397E-18	0.728922445	Potri.015G061600.1	AT1G74360.1
Potra2n12c24279.1						
Potra2n12c24280.1					Potri.003G125600.1	AT1G63440.1
Potra2n12c24281.1					Potri.009G003600.1	
Potra2n12c24282.2	NA	NA	NA	NA	Potri.T170600.1	AT1G37113.1
Potra2n12c24283.1	0.119634312	-0.096140648	0.529371611	0.91404112	Potri.012G067700.1	AT1G18680.1
Potra2n12c24284.1	-0.731223023	-0.306095341	6.13751E-05	0.567971082	Potri.012G067800.1	AT1G18670.1
Potra2n12c24285.1	-0.203963208	-0.046229404	0.133841867	0.959110452	Potri.012G067900.1	AT5G07740.1
Potra2n12c24286.1	-0.005042243	-0.02867721	0.982819285	0.98177802	Potri.012G068100.1	AT1G18660.1
Potra2n12c24287.1	-1.895286376	-2.045714426	NA	NA		
Potra2n12c24288.1	-0.289855062	-0.167640713	0.033305497	0.786910796	Potri.012G068300.1	AT5G07720.1
Potra2n12c24289.1	-1.049527274	-0.136085007	0.007408298	0.955139324	Potri.012G068400.1	AT5G61370.1
Potra2n12c24290.1	-2.229956613	-1.814800659	0.002841997	0.167360381	Potri.012G068500.1	AT1G18730.3
Potra2n12c24291.1	-1.857679243	-1.131824854	0.001073998	0.241676826	Potri.012G068500.1	AT1G18730.3
Potra2n12c24292.9	-0.060921061	0.082991921	0.732432303	0.910477984	Potri.015G062400.1	AT1G66680.1
Potra2n12c24293.1	0.548994276	0.178317479	0.273384631	0.95671968	Potri.012G068700.2	AT1G74460.1
Potra2n12c24294.1	-0.461153572	-0.461758252	0.017073973	0.335782206	Potri.T161200.1	AT1G74470.1
Potra2n12c24295.1	-0.011660636	0.118349882	0.950018016	0.912777843	Potri.012G068900.3	AT3G47610.1
Potra2n12c24296.1	0.021183496	0.169950012	0.913874321	0.850863374	Potri.012G069000.2	AT1G26850.2
Potra2n12c24297.1	-0.222110495	-0.034615343	0.116288272	0.974137889	Potri.012G069100.1	AT1G18800.1
Potra2n12c24298.1	0.856271432	-0.158568015	0.478507515	NA	Potri.012G069200.1	AT3G19000.1
Potra2n12c24299.1	-0.085484029	-0.096125924	0.596894746	0.903477395	Potri.012G069300.1	AT1G74520.1
Potra2n12c24300.1	0.376693258	-0.560962534	0.708927082	0.912777843		
Potra2n12c24301.1	-0.05674037	1.557920821	0.917757681	0.453004317	Potri.012G069400.1	AT5G62500.1
Potra2n12c24302.1	NA	5.840303983	NA	NA	Potri.012G069500.1	AT3G47710.1
Potra2n12c24303.1	NA	1.381953902	NA	NA	Potri.012G069600.1	AT2G33500.1
Potra2n12c24304.1	4.68346033	2.595829503	2.12843E-41	2.48854E-07	Potri.012G069700.2	AT3G47780.1
Potra2n12c24305.1	2.476290371	1.571529889	NA	NA		
Potra2n12c24306.1	4.316671172	2.295304126	5.5478E-47	0.000109397	Potri.012G069800.1	AT3G47730.1
Potra2n12c24307.1	-0.510211065	-0.050087085	0.001823545	0.964333742	Potri.012G069900.2	AT1G74690.1
Potra2n12c24308.1	-0.739248664	-0.196991031	4.70778E-05	0.874507876	Potri.012G070000.1	AT1G74710.1

Potra2n12c24309.1	NA	NA	NA	NA	Potri.012G070100.1	AT2G27140.1
Potra2n12c24310.1	1.077219787	0.672024188	0.000372119	0.596719268	Potri.012G070300.1	AT3G01280.1
Potra2n12c24311.1	-0.029759833	0.002817918	0.840255966	0.998245864	Potri.012G070500.2	AT5G62740.1
Potra2n12c24312.1	-1.207728927	-0.340724381	0.006002641	0.859892315	Potri.012G070600.1	AT1G74730.1
Potra2n12c24313.2	-2.41166272	0.166315497	0.03665251	0.97950444	Potri.012G070700.1	AT1G69850.1
Potra2n12c24314.1	NA	4.776902725	NA	NA		
Potra2n12c24315.1	-0.101959746	0.481868895	0.630364184	0.504797581	Potri.012G070900.1	AT3G01470.1
Potra2n12c24316.1	-1.063456791	-0.250707869	0.000708044	0.859892315	Potri.012G071100.1	AT5G62710.1
Potra2n12c24317.1	NA	NA	NA	NA	Potri.012G071200.1	AT1G05160.1
Potra2n12c24318.1	1.814777689	0.258312669	0.108196756	NA	Potri.012G071400.1	AT5G62680.1
Potra2n12c24319.1	-0.031017018	-0.146449874	0.906831398	0.912975882	Potri.012G071600.3	AT5G62670.1
Potra2n12c24320.1	2.212218677	0.993186107	9.84552E-05	0.520533674	Potri.012G071500.1	AT5G62680.1
Potra2n12c24321.1	0.145273304	-0.130854972	0.443431559	0.932447954	Potri.012G071700.1	AT1G74740.1
Potra2n12c24322.1	0.040437313	-1.465929376	NA	NA		
Potra2n12c24323.1	0.533140672	0.67889972	0.67447056	0.896306549		
Potra2n12c24324.1	-0.153019988	0.331360084	0.928407856	0.9761362	Potri.001G344400.1	AT5G40510.1
Potra2n12c24325.2	-0.097166068	0.082034792	0.529708129	0.928266269	Potri.012G071900.1	AT3G47890.1
Potra2n12c24326.1	-2.754058864	-2.899343921	NA	NA	Potri.012G072000.1	AT3G47870.1
Potra2n12c24327.1	1.106491125	3.04767016	0.643098878	0.693544424	Potri.012G072101.1	AT1G74780.1
Potra2n12c24328.1	0.139195315	0.12381267	0.129493941	0.715585895	Potri.012G072200.1	AT1G18950.1
Potra2n12c24329.1	-0.101172937	0.448172752	0.875798978	0.865591169	Potri.012G072300.1	AT5G40010.1
Potra2n12c24330.1	-0.531602619	-0.116845987	8.28457E-06	0.91232186	Potri.012G072400.1	AT5G62620.1
Potra2n12c24331.1					Potri.012G072500.1	AT3G28470.1
Potra2n12c24332.2	-0.143606777	0.009711781	0.335378117	0.992934677	Potri.012G072600.1	AT3G47850.1
Potra2n12c24333.1	-0.049133363	0.256520616	0.815607	0.602611218		
Potra2n12c24334.1	0.108477134	0.082677978	0.540432087	0.927879595	Potri.012G072700.1	AT5G62610.1
Potra2n12c24335.1	0.060511506	-0.113984074	0.637644431	0.91540496	Potri.012G072800.1	AT5G13050.1
Potra2n12c24336.1	0.232785375	-0.001006881	0.056582557	0.999458638	Potri.012G073300.1	AT1G10430.1
Potra2n12c24337.1	0.21861795	0.006958627	0.946825869	NA	Potri.012G073400.1	AT1G74830.1
Potra2n12c24338.3	0.051601786	-0.031783082	0.783513804	0.981825681	Potri.012G073500.1	AT5G11010.2
Potra2n12c24339.4	0.082200512	0.064042766	0.451071808	0.919424623	Potri.012G073600.2	AT5G62600.1
Potra2n12c24340.1	0.103623801	-0.290793303	0.757444295	0.823519367		

Potra2n12c24341.2	-0.323833315	0.080880149	0.013015715	0.947369645	Potri.012G073700.1	AT3G08720.1
Potra2n12c24342.1	0.061940669	0.19578689	0.83271736	0.891695245	Potri.012G073900.1	AT1G19000.2
Potra2n12c24343.1	-0.722485803	-0.280746495	0.001230813	0.759797728	Potri.012G074400.1	AT5G62580.1
Potra2n12c24344.1	0.234022296	0.178320558	0.091978428	0.797130371	Potri.011G095700.4	AT5G06350.1
Potra2n12c24345.2	0.157528004	-0.161758286	0.392650018	0.788065047	Potri.012G074500.1	AT5G62910.1
Potra2n12c24346.1	-0.688652026	2.370463579	NA	NA		
Potra2n12c24347.1	2.563896372	3.225316855	NA	NA	Potri.012G074700.1	AT3G48080.1
Potra2n12c24348.1	1.754762222	0.581311035	1.23503E-29	0.131506737	Potri.012G074700.1	AT3G48080.1
Potra2n12c24349.1	-0.021005801	0.014142641	0.868071581	0.988609622	Potri.012G074800.1	AT1G62020.1
Potra2n12c24350.1	0.578645644	0.064192277	0.486989186	0.992314551		
Potra2n12c24351.1	-0.088482952	-0.050363769	0.341183492	0.956865545	Potri.012G074900.2	AT3G48140.1
Potra2n12c24352.1	0.102494349	0.120860021	0.60733085	0.903140487	Potri.012G075000.1	AT3G48150.1
Potra2n12c24353.1	-0.147076505	0.042877298	0.181936568	0.9571583	Potri.012G075100.1	AT1G60490.1
Potra2n12c24354.1	-0.483851043	-1.027557249	0.483179607	0.449772714	Potri.012G075300.1	AT3G48160.2
Potra2n12c24355.2	-0.18657118	0.205299611	0.266608039	0.824448251	Potri.012G075500.1	AT1G74910.2
Potra2n12c24356.1	0.253755139	0.105455634	0.014590963	0.889835753	Potri.012G075600.1	AT1G74920.1
Potra2n12c24357.1	NA	NA	NA	NA		
Potra2n12c24358.1	1.323709383	0.775029777	0.00370661	0.365622999	Potri.012G075800.1	AT1G19020.1
Potra2n12c24359.1	-0.139340379	-0.124551445	0.565437769	0.943483421	Potri.012G075932.1	AT5G13690.1
Potra2n12c24360.1	-2.994643429	-1.057998238	0.060289774	0.865871651	Potri.012G076000.1	AT5G50120.1
Potra2n12c24361.1	-0.335381043	-0.246308495	0.16444668	0.896089262	Potri.012G076100.1	AT5G50110.1
Potra2n12c24362.1	-0.132328784	-0.062709214	0.330198763	0.948002367		
Potra2n12c24363.1	0.289380626	0.024625888	0.323464374	0.985393296	Potri.012G076400.1	AT5G65200.1
Potra2n12c24364.1					Potri.012G076500.1	AT5G47000.1
Potra2n12c24365.1	NA	1.363811008	NA	NA	Potri.T076201.1	AT5G45400.1
Potra2n12c24366.1	0.192133184	0.298565998	0.467256544	0.684244573	Potri.012G076600.1	AT1G27520.1
Potra2n12c24367.1	-2.839712827	0.149737316	2.64228E-18	0.977559521	Potri.012G076700.1	AT5G14920.1
Potra2n12c24368.1	0.398892489	0.107872555	0.009985867	0.939824856	Potri.015G071700.1	AT5G35530.1
Potra2n12c24369.1	NA	NA	NA	NA	Potri.012G076900.1	
Potra2n12c24370.1	-0.277308585	0.39861728	0.479840853	0.792896411	Potri.012G077000.1	AT5G62570.2
Potra2n12c24371.1	0.38852193	-0.008917404	0.003930019	0.996032145	Potri.012G077101.1	AT5G62575.1
Potra2n12c24372.1	2.086885818	0.969472933	3.53981E-14	0.176256892	Potri.012G077200.1	AT1G10200.1

Potra2n12c24373.1	-0.657979774	-0.336931779	0.005201185	0.728206951	Potri.012G077300.1	AT5G50090.1
Potra2n12c24374.1	-0.495457368	-0.365232337	0.043076343	0.814981587	Potri.012G077400.10	AT5G62890.3
Potra2n12c24375.1	-0.11369324	0.063710996	0.426845156	0.936890993	Potri.012G077500.1	AT3G48050.1
Potra2n12c24376.1	-1.423704892	-0.485543287	2.48497E-06	0.153430901	Potri.012G077800.1	AT3G48040.1
Potra2n12c24377.1	0.077771269	-0.113179643	0.615098937	0.863705586	Potri.012G077900.1	AT5G40200.1
Potra2n12c24378.1	-0.263464512	-0.161639708	0.356766896	0.923397137	Potri.012G078000.1	AT3G48030.1
Potra2n12c24379.1	-0.985341194	-0.424537267	6.87509E-08	0.517110447	Potri.012G078200.1	AT5G40250.1
Potra2n12c24380.1	-0.871747953	-0.825969328	0.003411495	0.293583922	Potri.012G078300.1	AT5G62865.1
Potra2n12c24381.1	0.074683406	-0.215714052	0.709707902	0.817452127	Potri.012G078400.1	AT1G24450.1
Potra2n12c24382.1	-0.516299357	-0.515818615	0.150576136	0.694769714	Potri.012G078500.1	AT1G23870.1
Potra2n12c24383.1	-0.091246675	0.132334188	0.531964423	0.817799498	Potri.012G078600.1	
Potra2n12c24384.1	-1.568956654	-0.165754203	2.69578E-07	0.909675463	Potri.012G078700.1	AT3G48000.1
Potra2n12c24385.1	-0.462955991	0.684302562	0.135950623	0.595538573	Potri.012G078900.1	AT1G60190.1
Potra2n12c24386.1	0.285593447	0.132989143	0.004586831	0.802942736	Potri.012G079000.1	AT5G50000.1
Potra2n12c24387.1	3.173895879	-0.39068863	0.003064708	NA	Potri.012G079100.1	AT4G20970.1
Potra2n12c24388.1	-2.108565621	-0.82075765	3.63201E-06	0.449772714	Potri.012G079200.1	AT1G70370.2
Potra2n12c24389.1	0.769815874	-0.222227821	0.141567795	0.914162682	Potri.012G079500.1	AT5G13700.1
Potra2n12c24390.1	0.414120378	0.114783091	8.29751E-06	0.823250234	Potri.012G079600.2	AT3G47990.1
Potra2n12c24391.1	-0.308919714	0.105904247	0.016115745	0.930871994	Potri.012G079700.1	AT1G27510.1
Potra2n12c24392.1	0.111706377	-0.081926295	0.352725301	0.935474933	Potri.012G079800.1	AT2G36330.1
Potra2n12c24393.2	-0.896634974	-0.42698942	1.43757E-05	0.526356391	Potri.012G080000.1	AT1G62400.1
Potra2n12c24394.1	-0.382036981	-0.232925577	0.01143168	0.821709751	Potri.012G080100.1	AT1G27500.1
Potra2n12c24395.1	-0.116353631	0.019344409	0.25446624	0.985393296	Potri.012G080300.2	AT4G32175.1
Potra2n12c24396.1	-0.001006573	0.007492264	0.996224005	0.996032145	Potri.012G080200.1	AT3G57430.1
Potra2n12c24397.1	-5.092137822	0.178864614	0.001057172	0.976043496	Potri.012G080400.1	AT5G14750.1
Potra2n12c24398.1	-0.888251696	0.459795015	0.112898013	0.889258723		
Potra2n12c24399.1	-0.734362319	0.744948413	0.012932604	0.749144604	Potri.012G080700.1	
Potra2n12c24400.1	0.089023277	0.124441932	0.39587622	0.848696379	Potri.012G080800.2	AT5G62810.1
Potra2n12c24401.1	0.308042275	0.246356394	0.112152587	0.804007296	Potri.012G080900.3	AT5G62790.1
Potra2n12c24402.1	-0.561707489	-0.483453649	9.3223E-05	0.378409561	Potri.012G081000.1	AT1G70420.1
Potra2n12c24403.1	-0.345137053	-0.513740754	0.3780965	0.79477084	Potri.012G081100.1	AT5G62520.1
Potra2n12c24404.1	2.359423114	0.727599369	0.001081064	0.849446235	Potri.012G081300.1	AT5G62940.1

Potra2n12c24405.1	-0.38761681	0.07984711	0.04909415	0.962696825		
Potra2n12c24406.1	0.177132564	0.113666868	0.08482743	0.821709751	Potri.012G081600.1	AT4G32180.1
Potra2n12c24407.1	-0.238698358	0.009779057	0.04257852	0.994403137	Potri.012G081700.3	AT4G22220.1
Potra2n12c24408.1	-1.593865365	-0.414770625	6.83831E-24	0.605100478	Potri.012G081800.1	AT3G62270.1
Potra2n12c24409.1	-0.151723172	-0.091367996	0.267038863	0.908342215	Potri.012G081900.1	AT5G62930.1
Potra2n12c24410.1	NA	NA	NA	NA	Potri.012G082000.1	AT4G18770.1
Potra2n12c24411.1	0.582609009	-0.026688416	0.000119549	0.989878071	Potri.012G082100.1	AT5G33320.1
Potra2n12c24412.1	-0.011909885	0.02094776	0.937031898	0.987226805	Potri.012G082200.2	AT1G10650.1
Potra2n12c24413.1	0.359560394	0.130655732	0.138344371	0.922993749	Potri.012G082300.1	AT1G27510.1
Potra2n12c24414.1	-1.323742038	-0.064935976	0.000354028	0.981259756	Potri.012G082400.1	AT5G62960.1
Potra2n12c24415.1	-0.331998505	-0.240579906	0.472944964	0.940856252	Potri.012G084400.1	AT2G42490.1
Potra2n12c24416.1	-0.053958722	-0.133282642	0.755634439	0.862335622	Potri.012G084600.1	AT1G55150.1
Potra2n12c24417.2	-1.89109336	-0.479618733	2.60748E-20	0.451575262	Potri.012G084700.1	AT5G47740.1
Potra2n12c24418.1	-0.003687328	0.253945495	0.993811559	0.825454445	Potri.015G083200.1	AT5G50160.1
Potra2n12c24419.1	0.057637507	0.28892286	0.912597015	0.81583604		
Potra2n12c24420.1	-0.216571597	0.352624947	0.624568741	0.779077802		
Potra2n12c24421.1	-0.345771587	-0.28265743	0.109164846	0.803684688	Potri.012G084900.1	AT1G15530.1
Potra2n12c24422.1	-0.147870437	-0.003362007	0.739426411	0.999325699	Potri.012G085000.2	AT5G63130.1
Potra2n12c24423.1	0.143432488	-0.050514512	0.554959529	0.974275337	Potri.012G085100.1	AT5G63135.1
Potra2n12c24424.1	-1.35927387	-1.507841271	NA	NA	Potri.012G085200.1	AT4G34210.1
Potra2n12c24425.1	-0.226537032	-0.252081301	0.104090998	0.445000674	Potri.012G085300.1	AT1G23280.1
Potra2n12c24426.1	-0.061606498	-0.009081505	0.539844549	0.992479764	Potri.012G085600.1	AT3G11330.1
Potra2n12c24427.1	-0.047506918	-0.000441003	0.76665155	0.999937671	Potri.012G085700.2	AT4G24690.1
Potra2n12c24428.1						
Potra2n12c24429.1	NA	NA	NA	NA		
Potra2n12c24430.1	-1.976262351	0.107821657	0.002716608	0.97950444	Potri.012G085800.2	
Potra2n12c24431.1	-3.546652277	-0.045447911	0.233569003	NA		
Potra2n12c24432.1	-0.599815778	-1.11511037	0.548764003	0.594207424	Potri.012G085800.2	
Potra2n12c24433.1	-3.35221096	-1.118431544	NA	NA	Potri.012G086300.1	AT4G24700.1
Potra2n12c24434.1	0.70729092	0.035027782	0.056131956	0.990390312	Potri.012G086400.1	AT4G24710.1
Potra2n12c24435.1	0.698124582	0.1941816	0.290758922	0.970011758		
Potra2n12c24436.1	0.794243922	-0.040206244	0.001282668	0.985393296	Potri.012G086500.1	AT1G56280.1

Potra2n12c24437.1	0.530313368	0.029685509	0.000258022	0.97950444	Potri.012G086600.2	AT4G29390.1
Potra2n12c24438.1	-0.363618354	0.02713775	0.018366972	0.984292161		
Potra2n12c24439.1	0.325381661	-0.231724202	0.102183245	0.880095503	Potri.012G086800.1	AT5G11750.1
Potra2n12c24440.1	0.598876276	0.019375292	0.473988737	0.99702095	Potri.012G086900.1	AT3G21850.1
Potra2n12c24441.1	NA	2.32414027	NA	NA	Potri.012G087000.2	AT5G40380.1
Potra2n12c24442.1	-0.554396444	-0.096516728	0.00010373	0.903140487	Potri.012G087100.1	AT1G23380.1
Potra2n12c24443.1	-0.61910233	-0.767708831	NA	NA	Potri.012G087300.1	AT5G63010.1
Potra2n12c24444.1					Potri.005G203700.1	AT1G48120.1
Potra2n12c24445.3	-1.378181233	-0.20828287	6.61098E-05	0.956631493	Potri.012G087400.1	AT3G48200.1
Potra2n12c24446.1	1.889091226	0.9381024	1.47271E-14	0.311727657	Potri.015G081500.1	AT1G12940.1
Potra2n12c24447.1	0.754744083	1.100150921	0.000685496	0.09016192	Potri.012G087800.1	AT5G50130.1
Potra2n12c24448.1	0.315410536	0.270133198	0.071099163	0.72101822	Potri.012G088000.1	AT3G48210.1
Potra2n12c24449.1	-0.485184755	0.111796586	0.276115103	0.966228173	Potri.012G088100.1	AT5G56040.2
Potra2n12c24450.1	0.646169611	0.210990993	3.08438E-05	0.821709751	Potri.012G088200.1	AT5G03340.1
Potra2n12c24451.2	-1.455451994	-0.670515231	5.14776E-12	0.2070232	Potri.012G088300.3	AT5G50150.1
Potra2n12c24452.1	NA	NA	NA	NA	Potri.015G080100.1	AT5G63060.1
Potra2n12c24453.1					Potri.012G088400.1	AT1G17100.1
Potra2n12c24454.3	-0.142304011	0.036070742	0.370446	0.973863072	Potri.012G088500.1	AT4G24680.1
Potra2n12c24455.1	-0.006231472	0.017765221	0.969662601	0.986636967	Potri.012G088700.3	AT5G63040.2
Potra2n12c24456.2	-0.398020156	-0.359853415	0.215324242	0.623978146	Potri.012G088900.1	AT4G20140.1
Potra2n12c24457.1	-0.471103178	0.054522564	0.000149307	0.954419252	Potri.012G089000.2	AT1G19100.1
Potra2n12c24458.1	-1.412562268	-0.547130924	0.022919956	0.674175338	Potri.012G089100.1	AT4G12690.1
Potra2n12c24459.1	-1.211526847	0.070718671	0.306244895	0.989304496		
Potra2n12c24460.1	-0.458993357	-0.195629364	0.00725197	0.728418847	Potri.012G089300.1	AT5G50200.1
Potra2n12c24461.1	-0.484240365	-0.170261817	0.008032571	0.802245642	Potri.012G089400.1	AT4G24730.2
Potra2n12c24462.1	1.459962178	NA	NA	NA		
Potra2n12c24463.1	1.871462132	-0.136977651	2.98488E-05	0.973347974		
Potra2n12c24464.1	1.812859375	-0.23891028	8.62679E-07	0.936360532	Potri.012G089500.10	AT5G50210.1
Potra2n12c24465.1	-0.869514527	-0.915641828	0.022207001	0.135806304	Potri.012G089600.1	AT3G48310.1
Potra2n12c24466.1	0.121559031	0.101941593	0.313856234	0.889106941	Potri.012G089700.1	AT4G24740.1
Potra2n12c24467.1	0.82357019	NA	NA	NA		
Potra2n12c24468.1	1.875396351	0.259920396	0.060507961	NA	Potri.012G089800.1	

Potra2n12c24469.1	0.106598256	-0.162201541	0.656965432	0.888824902	Potri.015G086100.3	AT4G24750.1
Potra2n12c24470.1	0.157755972	-0.187136374	0.541405912	0.884793835	Potri.012G090000.1	AT3G26330.1
Potra2n12c24471.1	1.029165011	0.096898831	0.026349104	0.977559521	Potri.012G090000.1	AT3G26330.1
Potra2n12c24472.1	1.840146052	0.384702796	6.90834E-11	0.859892315		
Potra2n12c24473.1	4.141725112	NA	0.056585433	NA		
Potra2n12c24474.1	1.932405334	0.044148844	1.32879E-05	0.992934677	Potri.012G090000.1	AT3G26330.1
Potra2n12c24475.1	0.400571448	0.158266607	0.000234191	0.764173811	Potri.012G090100.2	AT5G14420.1
Potra2n12c24476.3	-0.293131525	-0.07184935	0.017548065	0.925933968	Potri.012G090200.1	AT4G24770.1
Potra2n12c24477.1	0.372373021	-0.211321215	0.655830438	0.958226175		
Potra2n12c24478.1	-0.591949287	-0.08122207	0.000571048	0.936890993	Potri.012G090300.1	AT3G48330.2
Potra2n12c24479.1	1.237744599	0.409399533	0.031620749	0.860813587	Potri.012G090400.2	AT3G01680.1
Potra2n12c24480.1	6.967058247	4.407753756	5.3943E-102	0.006142017	Potri.012G090500.1	AT2G31880.1
Potra2n12c24481.1	-0.435429859	-0.302325574	0.033791293	0.679660122	Potri.012G090600.3	AT1G78790.1
Potra2n12c24482.1	0.017976383	-0.07831777	0.961457519	0.978646026	Potri.012G090700.1	AT5G63150.1
Potra2n12c24483.1	1.545832463	2.501508747	NA	NA		
Potra2n12c24484.1	-0.434860908	-0.239256578	6.91717E-06	0.772117957	Potri.012G090800.1	AT4G24760.1
Potra2n12c24485.1	NA	0.674963689	NA	NA		
Potra2n12c24486.1	-3.268676484	-0.361337894	0.005067448	0.954098985	Potri.012G090900.1	AT5G50260.1
Potra2n12c24487.1	1.467789002	0.456507232	1.02822E-15	0.563998615	Potri.012G091100.1	AT5G61640.1
Potra2n12c24488.1	1.545568872	0.515108515	4.76302E-15	0.52733716	Potri.012G091000.1	AT5G50260.1
Potra2n12c24489.1	-0.559093357	-0.290768358	0.01599958	0.855587395	Potri.015G087700.1	AT5G63160.1
Potra2n12c24490.1	0.449549219	-0.331507171	0.194466827	0.851911116	Potri.012G091500.1	AT4G24780.2
Potra2n12c24491.1	-1.255759747	-0.513059751	0.189566149	0.91998377	Potri.015G087900.10	AT4G38040.1
Potra2n12c24492.1	-1.304717597	-0.438084277	0.159760001	0.932932656	Potri.001G155750.1	AT1G21280.1
Potra2n12c24493.1	0.093989994	0.687779408	0.940117187	0.921015963	Potri.012G091700.1	AT4G24790.2
Potra2n12c24494.1	0.062402749	0.034114136	0.813835612	0.984683464	Potri.012G091800.1	AT5G50280.1
Potra2n12c24495.1	0.029634815	0.217529389	0.863611714	0.821709751	Potri.012G091900.1	AT5G63190.2
Potra2n12c24496.1	-0.195698853	-0.207069967	0.070046492	0.773641026	Potri.012G092000.1	AT5G63200.1
Potra2n12c24497.1	0.815950438	0.581280023	0.360850151	0.876272842	Potri.015G088500.2	AT3G48420.1
Potra2n12c24498.1					Potri.012G092200.2	AT1G14870.1
Potra2n12c24499.2	0.02762882	0.274763852	0.902635586	0.828847087	Potri.012G092400.1	AT3G48430.1
Potra2n12c24500.1	0.144732946	0.205673455	0.215778635	0.69821721	Potri.012G092500.2	AT3G48440.1

Potra2n12c24501.1	0.524000724	0.150932873	0.144856909	0.954419252	Potri.012G095100.1	AT1G29320.1
Potra2n12c24502.1	0.215737845	0.152778749	0.048750149	0.847978165	Potri.015G092500.1	AT5G20090.2
Potra2n12c24503.2	-0.022964995	-0.041991136	0.904928464	0.977530072	Potri.012G095200.1	AT5G60160.1
Potra2n12c24504.1	0.018762913	-0.081538609	0.921112581	0.953837261	Potri.012G095300.1	AT5G63400.1
Potra2n12c24505.1	0.652636627	0.033209614	0.001319374	0.987226805	Potri.012G095400.1	AT1G56110.1
Potra2n12c24506.1	0.069008443	-0.025684211	0.640575221	0.981772014	Potri.012G095500.1	AT2G40190.1
Potra2n12c24507.1	-0.403564313	0.129068466	0.248846219	0.953768643	Potri.012G095600.1	AT5G14210.1
Potra2n12c24508.1	-0.425497662	0.386007704	0.48292377	0.895474078		
Potra2n12c24509.1	1.459962178	1.381953902	NA	NA	Potri.012G095700.1	AT3G01820.1
Potra2n12c24510.1	NA	0.674963689	NA	NA	Potri.015G093300.2	AT3G49180.1
Potra2n12c24511.1	-0.583295257	-0.129972801	0.000201879	0.917751882	Potri.012G095800.1	AT5G63420.1
Potra2n12c24512.1	0.084325835	1.026695244	NA	NA		
Potra2n12c24513.1	-0.031031863	-0.007804843	0.853065689	0.996032145	Potri.012G096100.1	AT4G24840.1
Potra2n12c24514.2	0.269696648	0.182527579	0.007843718	0.674165743	Potri.012G096300.1	AT1G02890.1
Potra2n12c24515.1	NA	NA	NA	NA		
Potra2n12c24516.3	0.540527774	0.113425249	0.001223821	0.920028955	Potri.012G096400.1	AT2G18110.1
Potra2n12c24517.1	0.522321285	0.130015141	0.000484165	0.909715408	Potri.012G096500.1	AT5G63440.2
Potra2n12c24518.1	0.662394082	0.150886869	8.31785E-05	0.910878765	Potri.012G096600.1	AT1G27400.1
Potra2n12c24519.1	-0.022320334	-0.045562568	0.919504198	0.971537796	Potri.012G096700.1	AT5G14170.1
Potra2n12c24520.1	NA	NA	NA	NA	Potri.012G096800.1	AT3G48520.1
Potra2n12c24521.1	0.228581952	0.110403379	0.170859076	0.924309478	Potri.012G096900.2	AT5G63460.1
Potra2n12c24522.2	1.099458971	0.855809886	0.000510789	0.547799891	Potri.012G097000.1	AT3G48530.1
Potra2n12c24523.1	0.036667584	-0.045074145	0.869202939	0.974275337	Potri.012G097100.1	AT5G50380.1
Potra2n12c24524.2	-0.144782539	-0.013566753	0.430073885	0.991036771	Potri.012G097200.1	AT2G27110.1
Potra2n12c24525.1	0.430234748	-0.193259106	0.021542843	0.885375133	Potri.012G097300.1	AT1G20960.1
Potra2n12c24526.1	0.707395809	-0.119945225	0.008411728	0.909675463	Potri.012G097400.1	AT5G50400.1
Potra2n12c24527.1	-0.375951927	-0.094479636	0.00813744	0.919912605	Potri.012G097500.1	AT5G50410.1
Potra2n12c24528.1	1.19704968	2.468104789	0.208707517	0.272381508	Potri.012G097600.1	AT1G67340.1
Potra2n12c24529.2	-0.028153194	0.270651034	0.907236255	0.736324018	Potri.012G097700.2	AT1G05350.1
Potra2n12c24530.3	-0.036918186	0.247119323	0.862637044	0.776584496	Potri.012G097800.1	AT3G48540.1
Potra2n12c24531.1	-2.18077474	-0.452301214	2.68394E-06	0.802942736	Potri.012G097901.1	AT3G48550.1
Potra2n12c24532.1	-0.016858839	0.165367913	0.939555423	0.862830524	Potri.012G098001.1	AT4G24900.1

Potra2n12c24533.1	0.117134598	0.322592666	0.788413371	0.896089262		
Potra2n12c24534.1	0.404021164	0.1946948	0.419386555	0.95671968	Potri.012G098100.1	AT4G24910.1
Potra2n12c24535.1	0.388983065	0.348379636	0.16197323	0.723580731	Potri.012G098200.1	AT4G37100.1
Potra2n12c24536.1	0.414063148	0.358504328	0.144484879	0.712700474		
Potra2n12c24537.1	1.338419073	0.102478357	7.44144E-13	0.953831244	Potri.012G098300.1	AT3G48560.1
Potra2n12c24538.1	0.537084115	0.054320645	0.007016705	0.974756511	Potri.012G098400.1	AT4G24920.1
Potra2n12c24539.1	-0.088952011	-0.042238219	0.67221739	0.978646026	Potri.015G097400.1	AT5G63470.2
Potra2n12c24540.2	0.682477936	0.327862438	0.011058285	0.853030738	Potri.012G098800.1	AT5G53150.1
Potra2n12c24541.1	0.677483224	0.335355912	0.020434057	0.855432225	Potri.012G098700.1	
Potra2n12c24542.1	-0.474842459	-0.092553959	0.018656131	0.948577865	Potri.012G098900.3	AT5G14120.1
Potra2n12c24543.2	-0.009795715	0.184222004	0.981809185	0.907073354	Potri.015G097700.1	AT4G24930.1
Potra2n12c24544.1	0.501948288	-0.168047116	0.074308421	0.913466025	Potri.012G099200.1	AT5G63480.1
Potra2n12c24545.1	-0.075547575	0.065355944	0.712864398	0.959881296	Potri.012G099300.1	AT3G48610.1
Potra2n12c24546.1	0.238346578	0.259681397	0.471556652	0.863022165	Potri.012G099400.1	AT3G03360.1
Potra2n12c24547.1	-1.165352361	-0.190196582	0.25416118	0.977227089	Potri.012G099500.2	
Potra2n12c24548.1	-0.61910233	-0.767708831	NA	NA		
Potra2n12c24549.1	0.110750937	0.303179521	0.317979841	0.696993664	Potri.012G099600.2	AT5G50530.1
Potra2n12c24550.1	NA	NA	NA	NA		
Potra2n12c24551.1	NA	NA	NA	NA		
Potra2n12c24552.1	NA	1.363811008	NA	NA	Potri.012G099800.1	AT3G16210.1
Potra2n12c24553.1	-0.384232783	0.388754559	0.29002575	0.846926485	Potri.012G100100.1	AT3G48660.1
Potra2n12c24554.1	-0.295360552	0.954777082	0.41437304	0.001987702	Potri.012G100200.1	AT1G26310.1
Potra2n12c24555.1	-0.394198541	3.5261678	0.897681575	NA	Potri.012G100300.1	
Potra2n12c24556.1	0.82357019	1.394613675	NA	NA		
Potra2n12c24557.1	0.123491217	-0.087724813	0.482470957	0.936549944	Potri.012G100400.1	AT3G48680.1
Potra2n12c24558.1	0.274597937	0.088111191	0.04741428	0.914903229	Potri.012G100500.2	AT5G63520.1
Potra2n12c24559.1	2.286159688	-1.465929376	0.400314057	NA	Potri.012G100600.1	AT5G63520.1
Potra2n12c24560.1	-0.662286438	-0.481644043	3.00505E-05	0.680030952	Potri.012G100700.2	AT5G50670.1
Potra2n12c24561.1	-0.349587435	-0.156457505	0.22354415	0.917516356	Potri.008G124700.1	AT2G01190.1
Potra2n12c24562.1	0.165076107	0.001867512	0.341987238	0.999325699	Potri.012G100800.3	AT4G24940.1
Potra2n12c24563.1	-2.453099188	0.577683555	NA	NA	Potri.012G100900.1	
Potra2n12c24564.1	5.651630646	3.785244484	1.75203E-27	0.099597414	Potri.012G101000.1	AT5G13080.1

Potra2n12c24565.1	1.563504383	0.889533452	1.54412E-11	0.172308534	Potri.012G101100.1	AT3G02230.1
Potra2n12c24566.1	-3.426239883	-1.098084595	0.005185381	0.365910084	Potri.012G101200.1	AT5G13090.1
Potra2n12c24567.1	-0.685979101	0.000399341	2.68713E-05	0.999937671	Potri.012G101400.1	AT5G63530.1
Potra2n12c24568.1	1.013548333	-0.111645807	0.130906323	0.98036348	Potri.012G101500.1	AT1G08510.1
Potra2n12c24569.1	-0.774047543	-0.285116164	4.81854E-07	0.198711864	Potri.012G101600.4	AT5G50720.1
Potra2n12c24570.1	-0.499560178	-1.816753601	0.796661843	NA	Potri.012G101800.1	AT5G50710.1
Potra2n12c24571.1	1.64774763	NA	NA	NA	Potri.012G101900.1	AT5G50590.1
Potra2n12c24572.1	NA	NA	NA	NA	Potri.012G102000.1	AT3G47350.1
Potra2n12c24573.1	-3.66284176	-0.472787554	0.102183245	NA	Potri.012G102100.1	
Potra2n12c24574.1	0.77216901	-0.520763412	0.761261477	NA	Potri.012G102200.1	AT3G01990.1
Potra2n12c24575.1					Potri.007G118000.1	AT1G18760.1
Potra2n12c24576.1	-0.655605869	-0.834216245	0.325033134	0.714411171	Potri.012G102300.1	AT3G06600.1
Potra2n12c24577.1	-0.873886266	-0.122107169	8.7204E-05	0.937687311	Potri.012G102400.1	
Potra2n12c24578.3	0.09750801	0.102540514	0.437774964	0.910815461	Potri.012G102500.2	AT3G48710.1
Potra2n12c24579.1	0.044220239	0.193183401	0.86880271	0.828625141	Potri.012G122900.1	AT3G14470.1
Potra2n12c24580.1	0.82357019	1.363811008	NA	NA		
Potra2n12c24581.1	1.048795836	1.16439016	0.488078644	NA	Potri.012G102600.1	
Potra2n12c24582.1	2.803351129	1.481284494	0.008384878	NA	Potri.012G102700.1	AT5G50760.1
Potra2n12c24583.1	-0.099635131	0.059771376	0.433831553	0.97103025	Potri.012G102800.2	AT4G24970.1
Potra2n12c24584.1	-0.108423508	-0.021711329	0.8508325	0.995388971	Potri.015G101100.1	AT3G48730.1
Potra2n12c24585.4	-0.6147942	-0.094822645	1.6765E-05	0.95671968	Potri.012G103100.1	AT4G24990.1
Potra2n12c24586.1					Potri.012G103200.1	AT5G50790.1
Potra2n12c24587.1	-0.056953813	0.042882887	0.735937875	0.967076029	Potri.012G103300.1	AT3G26935.1
Potra2n12c24588.1	0.238924098	-0.209354396	0.404077376	0.914554682	Potri.012G103400.1	AT5G50810.1
Potra2n12c24589.1	4.287266897	2.900818469	0.23135939	NA	Potri.012G103500.1	AT5G13180.1
Potra2n12c24590.1	NA	NA	NA	NA	Potri.012G103600.2	AT5G50830.2
Potra2n12c24591.1	3.272197802	1.38222938	9.39003E-49	0.371998415	Potri.012G103700.1	AT3G48770.1
Potra2n12c24592.1	NA	0.674963689	NA	NA	Potri.015G103000.1	AT3G48770.1
Potra2n12c24593.1	0.619037534	-0.007739497	0.005219798	0.996032145	Potri.012G103900.1	AT5G50860.1
Potra2n12c24594.1	0.542549303	0.257097994	0.000228175	0.684639773	Potri.012G104100.1	AT5G50870.1
Potra2n12c24595.1	0.020280222	0.0514827	0.935758071	0.974275337	Potri.012G104200.1	AT5G41110.1
Potra2n12c24596.1	-1.854659706	-0.252520845	0.008404629	0.953731124	Potri.012G104300.1	AT4G25040.1

Potra2n12c24597.1	0.957998671	0.437571052	4.46353E-05	0.663037599	Potri.012G104400.1	AT5G50900.1
Potra2n12c24598.1	-0.066595366	-0.039002186	0.657494854	0.976302215	Potri.012G104500.1	AT5G23670.1
Potra2n12c24599.1	3.076608979	-3.770825714	0.001633569	NA	Potri.012G104600.1	AT1G24140.1
Potra2n12c24600.6	-0.236981664	0.090246531	0.116705339	0.954559968	Potri.012G104700.1	AT5G23680.1
Potra2n12c24601.1	2.199892549	3.119470721	0.525229281	NA	Potri.012G104900.1	AT5G50915.1
Potra2n12c24602.2	-0.229246543	0.012604993	0.138344371	0.992934677	Potri.012G105000.1	AT5G23690.1
Potra2n12c24603.1	0.449334507	0.001002008	0.594033679	0.999937671	Potri.012G105100.1	AT5G14040.1
Potra2n12c24604.1	5.060636482	NA	0.004867813	NA	Potri.017G153100.2	
Potra2n12c24605.1	-1.95939979	-0.664947593	0.000251013	0.645919795	Potri.012G105300.1	AT4G25050.1
Potra2n12c24606.3	-0.467141915	-0.175822079	0.000192041	0.874808359	Potri.012G105400.3	AT3G48860.2
Potra2n12c24607.1	-2.228939415	-0.471367456	1.06732E-12	0.624602712	Potri.012G105500.1	AT1G28110.2
Potra2n12c24608.1	0.670582992	0.042306354	0.000188339	0.985393296	Potri.012G105700.1	AT2G47140.1
Potra2n12c24609.1	0.108163423	0.052027224	0.372351239	0.945454119	Potri.012G105800.1	AT5G23720.1
Potra2n12c24610.1	0.154822249	0.132071073	0.122486886	0.767368598	Potri.012G105900.1	AT5G50920.1
Potra2n12c24611.1	-0.110934208	0.014033098	0.543266595	0.992934677	Potri.012G106000.1	AT3G26744.2
Potra2n12c24612.1	-0.228297051	0.107840038	0.154789695	0.925184207	Potri.012G106100.1	AT5G62000.2
Potra2n12c24613.3	-0.142346702	-0.088977052	0.30159595	0.900676614	Potri.015G105600.2	AT5G61970.1
Potra2n12c24614.1	-0.331186678	-0.035806184	0.063424016	0.97950444	Potri.012G106300.1	AT1G53200.1
Potra2n12c24615.1	0.403422818	0.572877077	0.023017779	0.661611864	Potri.012G106500.1	AT5G13980.1
Potra2n12c24616.1	-0.119036766	-0.115898086	0.450890165	0.919424623	Potri.012G106700.1	AT3G48880.1
Potra2n12c24617.1	-1.177949848	0.606760875	NA	NA		
Potra2n12c24618.1	0.610031788	0.522072415	0.000205029	0.72101822		
Potra2n12c24619.1	0.575473926	0.625021878	0.083450804	0.805227557	Potri.012G106800.1	AT3G18720.1
Potra2n12c24620.1	0.221841943	0.569508894	0.604675633	0.741407257	Potri.T173600.2	AT5G39600.1
Potra2n12c24621.1	-0.447581333	0.393681298	0.378662333	0.775830532		
Potra2n12c24622.2	-0.625003804	-0.242762144	0.000416748	0.507997681	Potri.012G107100.1	AT5G61960.1
Potra2n12c24623.1	-1.615768078	-1.058398303	NA	NA	Potri.012G107800.1	AT4G23490.1
Potra2n12c24624.1	0.160291374	-0.631191363	0.925977289	NA	Potri.012G107600.1	AT1G07080.1
Potra2n12c24625.1	0.420345571	0.199804389	0.013917956	0.897285619	Potri.012G107500.1	AT1G31220.1
Potra2n12c24626.1	0.485061048	-0.042573143	0.007105915	0.981702181	Potri.012G107400.1	AT1G65030.1
Potra2n12c24627.1	-0.443122679	0.101615985	0.135590786	0.959431187		
Potra2n12c24628.1	-0.091876584	-0.019145054	0.729468854	0.990896655	Potri.012G108000.1	AT2G34400.1

Potra2n12c24629.1	0.515852748	0.312670369	4.57508E-06	0.418644133	Potri.012G108100.1	AT5G50970.1
Potra2n12c24630.1	0.578586185	0.206015537	0.008123793	0.874808359		
Potra2n12c24631.1	0.530747999	0.084323648	0.008615675	0.943483421	Potri.012G108200.1	AT5G07300.1
Potra2n12c24632.1	0.652782359	0.19534453	3.08288E-05	0.873955419	Potri.017G082200.1	AT1G26880.1
Potra2n12c24633.1	3.244879335	-0.050631851	0.006589778	0.995581218	Potri.012G108500.1	AT5G61890.1
Potra2n12c24634.2	0.360418339	0.227479219	0.106494815	0.871738422	Potri.012G108600.1	AT3G59280.1
Potra2n12c24635.1	0.200767637	1.092583562	0.676379927	0.792511865	Potri.012G108701.1	
Potra2n12c24636.1	0.476919665	-0.908924563	0.630042446	0.885375133	Potri.012G108900.3	AT5G61865.1
Potra2n12c24637.1	-0.61910233	-0.767708831	NA	NA	Potri.002G263900.1	AT3G24060.1
Potra2n12c24638.1	-0.463808294	0.092023043	0.029262194	0.968101851	Potri.012G109300.1	AT5G50990.1
Potra2n12c24639.1	0.361727094	0.185755963	0.022753167	0.84231643	Potri.006G228500.11	AT5G60340.1
Potra2n12c24640.1	-0.210685399	0.129887575	0.338326093	0.939824856	Potri.012G109600.1	AT5G61840.1
Potra2n12c24641.1	1.555049812	-0.046370319	NA	NA	Potri.012G109700.1	AT5G51010.1
Potra2n12c24642.4	0.062304726	0.055097095	0.644540878	0.948577865	Potri.012G109800.1	AT5G51020.1
Potra2n12c24643.1	-1.328951947	-1.478536539	NA	NA	Potri.012G109900.1	AT5G55690.1
Potra2n12c24644.1	1.64774763	NA	NA	NA	Potri.012G110100.1	AT5G60440.1
Potra2n12c24645.1	0.130857791	0.027590737	0.47542421	0.98317667	Potri.012G110200.1	AT5G51030.1
Potra2n12c24646.1	-3.045117845	-0.53296195	0.124733266	0.943819961		
Potra2n12c24647.1	1.610545025	-0.319280038	0.003596984	0.918630346	Potri.012G110300.1	AT5G61820.1
Potra2n12c24648.1	-0.553038473	-0.137545018	0.06941405	0.948577865	Potri.012G110600.1	AT5G61820.1
Potra2n12c24649.1	0.132654215	0.0964994	0.395531149	0.909675463	Potri.012G110700.1	AT5G51050.1
Potra2n12c24650.1	-0.471845152	0.774802632	0.204380453	0.619252328	Potri.012G110800.1	AT5G61800.1
Potra2n12c24651.1	0.231255847	0.059255046	0.135867549	0.95359265	Potri.012G111100.1	AT5G61790.1
Potra2n12c24652.1	-0.261519446	-0.108325566	0.032276668	0.907032966	Potri.012G111200.1	AT5G07350.2
Potra2n12c24653.3	0.004168824	-0.108979588	0.990895323	0.922687399	Potri.012G111600.1	AT5G51060.1
Potra2n12c24654.1	-0.860042432	-0.205632265	1.48407E-06	0.843683654	Potri.012G111400.1	AT5G61760.1
Potra2n12c24655.1	0.042444211	-0.016348484	0.884678662	0.990927357	Potri.012G111800.1	AT5G07400.1
Potra2n12c24656.1	0.659566788	-0.335343243	0.221544305	0.936549944	Potri.012G111900.1	AT4G33990.1
Potra2n12c24657.1	0.21860581	0.067344423	0.116837383	0.949312638	Potri.012G112000.1	AT5G51070.1
Potra2n12c24658.1	-0.454799829	-0.229898263	0.014587638	0.798272324	Potri.012G112200.1	AT1G02170.1
Potra2n12c24659.1	1.354395441	0.148764843	0.005066478	0.975578749	Potri.012G113100.1	AT1G02170.1
Potra2n12c24660.1					Potri.015G110700.1	AT1G69940.1

Potra2n12c24661.1	-1.328951947	-1.478536539	NA	NA	Potri.012G112800.1	AT1G69940.1
Potra2n12c24662.1	-0.142334289	-0.029616384	0.141333056	0.974367223	Potri.012G115100.1	AT5G65260.1
Potra2n12c24663.4	-0.146562292	0.096935491	0.60419865	0.954247482	Potri.012G113500.1	AT5G07440.2
Potra2n12c24664.1	0.158026372	-0.169181617	0.269210863	0.739555983	Potri.012G113700.1	AT5G51150.1
Potra2n12c24665.1	-0.326035492	-2.40821597	0.624568741	0.01335459	Potri.012G113800.1	AT5G51160.1
Potra2n12c24666.1	NA	NA	NA	NA	Potri.012G113800.1	AT5G51160.1
Potra2n12c24667.1	0.115757085	-0.786502229	NA	NA	Potri.012G113900.1	AT4G10720.1
Potra2n12c24668.1	NA	NA	NA	NA		
Potra2n12c24669.1	0.683957371	0.085132382	9.44713E-06	0.953108412	Potri.001G025300.1	AT2G47110.1
Potra2n12c24670.1	0.443105138	0.328090233	0.003472165	0.590087814	Potri.012G114100.1	AT4G25120.1
Potra2n12c24671.2	0.341674146	0.378945851	0.028500655	0.595538573	Potri.012G114200.1	AT5G61670.2
Potra2n12c24672.1	-0.61910233	1.915304303	NA	NA		
Potra2n12c24673.1	-0.748110412	0.508417037	0.357590981	0.859892315	Potri.012G114300.1	
Potra2n12c24674.2	0.29865374	0.224735574	0.080642542	0.859892315	Potri.012G114400.1	AT2G35160.1
Potra2n12c24675.1	-0.497295429	-0.433698582	0.124313536	0.650882346	Potri.012G114600.1	AT2G44740.1
Potra2n12c24676.1	-0.095414984	-0.093018853	0.473169178	0.930598202	Potri.012G115800.1	AT4G25130.1
Potra2n12c24677.1	1.691538803	0.365233598	3.75254E-20	0.714673422	Potri.012G115900.1	AT2G37500.1
Potra2n12c24678.1	1.368882238	0.373459272	1.91621E-15	0.749691449	Potri.T123300.2	AT5G45160.1
Potra2n12c24679.1	1.159618818	0.248021163	1.47054E-17	0.903398621	Potri.T123300.2	AT5G45160.1
Potra2n12c24680.1	4.140420525	-2.466612692	0.000553531	NA	Potri.012G117100.1	AT5G45160.1
Potra2n12c24681.1	0.82357019	NA	NA	NA	Potri.015G112700.1	AT5G45160.1
Potra2n12c24682.3	1.64774763	NA	NA	NA		
Potra2n12c24683.1					Potri.012G117001.1	AT5G45160.1
Potra2n12c24684.7	1.469265777	0.544374787	1.44693E-11	0.767368598	Potri.012G116700.7	AT5G45160.1
Potra2n12c24685.1					Potri.T128500.1	
Potra2n12c24686.1	NA	NA	NA	NA	Potri.012G117100.1	AT5G45160.1
Potra2n12c24687.1	0.940688237	0.343348074	0.009512474	0.874595634	Potri.012G116700.7	AT5G45160.1
Potra2n12c24688.1	2.53913187	1.414217384	3.30448E-28	0.329167823	Potri.012G117400.1	AT5G19990.1
Potra2n12c24689.1	NA	NA	NA	NA		
Potra2n12c24690.1	1.545832463	NA	NA	NA	Potri.012G117100.1	AT5G45160.1
Potra2n12c24691.2	2.712223522	1.709022816	1.10111E-32	0.183479474	Potri.012G116151.1	AT5G45160.1
Potra2n12c24692.1	NA	NA	NA	NA	Potri.015G112700.1	AT5G45160.1

Potra2n12c24693.1	1.459962178	NA	NA	NA	Potri.012G117500.1	AT2G35640.1
Potra2n12c24694.1	0.551849033	0.029891207	0.001388697	0.987577016	Potri.012G117600.1	AT3G04770.2
Potra2n12c24695.1	-0.060149976	-0.054291396	0.678923333	0.958874237	Potri.012G117800.3	AT5G45130.1
Potra2n12c24696.1	NA	0.674963689	NA	NA	Potri.012G118000.1	AT5G45120.1
Potra2n12c24697.1	-0.586498886	-0.238535175	8.78071E-05	0.775830532	Potri.012G118100.1	AT1G31300.1
Potra2n12c24698.1	0.060875241	0.160484797	0.874951209	0.920141992	Potri.012G118200.1	AT4G19650.1
Potra2n12c24699.1	1.153869488	0.499080915	2.75495E-34	0.005453789	Potri.012G118500.1	AT5G45110.1
Potra2n12c24700.1	-0.018804736	0.431926867	0.965315424	0.744962075	Potri.012G118600.1	AT3G06880.2
Potra2n12c24701.3	2.022476101	0.424228072	2.24532E-26	0.788065047	Potri.012G118700.2	AT1G31280.1
Potra2n12c24702.1	-0.565273712	-0.141159641	0.003226545	0.937146435		
Potra2n12c24703.2	0.259129558	0.03281042	0.049477977	0.982422288	Potri.012G119000.1	AT4G19670.2
Potra2n12c24704.1					Potri.012G119100.1	AT1G31260.1
Potra2n12c24705.1	0.079077215	-0.173878646	0.795107202	0.912814353	Potri.012G119200.1	AT5G45100.1
Potra2n12c24706.1	1.241393551	0.127312086	0.022863098	0.977559521	Potri.012G119500.1	AT1G31240.1
Potra2n12c24707.1	-0.42740978	-0.273034151	0.000529436	0.759797728	Potri.012G119600.1	AT1G04760.1
Potra2n12c24708.1	-1.637525425	-0.002683703	0.456516069	NA	Potri.012G119700.1	AT5G17200.1
Potra2n12c24709.1	-0.987294968	1.287939001	0.138664014	0.743612955	Potri.012G119800.2	AT4G19830.1
Potra2n12c24710.2	-0.053397745	0.018023263	0.59672298	0.98394486	Potri.012G120000.1	AT1G10180.1
Potra2n12c24711.1	0.629763315	-0.382777183	0.147822744	0.880553952	Potri.T088300.1	AT1G10155.1
Potra2n12c24712.1	-0.627264373	-1.107904393	0.477358447	0.693906898	Potri.T088300.1	AT1G10155.1
Potra2n12c24713.1	0.945465536	-0.256980877	0.44850341	0.967229803	Potri.012G120300.1	AT1G10155.1
Potra2n12c24714.1	-0.470205481	-0.235924692	0.482549635	0.943483421	Potri.012G120400.1	AT4G19840.1
Potra2n12c24715.1	0.19258267	0.021791776	0.149524328	0.9896961	Potri.T088700.2	AT1G31190.1
Potra2n12c24716.1	8.035922923	4.819367539	2.52335E-13	0.283433036		
Potra2n12c24717.1	0.108317977	0.043012167	0.52713045	0.977559521	Potri.T089300.1	AT5G53070.1
Potra2n12c24718.1	-0.23955985	0.421566575	0.820837414	0.938246513		
Potra2n12c24719.1	0.066486214	-0.006137856	0.599681734	0.996032145	Potri.T089500.1	AT4G19860.1
Potra2n12c24720.1	-2.941099988	0.25767357	0.077758122	0.972159498		
Potra2n12c24721.2	-0.529658385	-0.154033132	0.005864616	0.918687733	Potri.012G121700.10	AT5G45030.1
Potra2n12c24722.1	-0.541143595	-0.131454005	0.019900629	0.912083068	Potri.012G123600.1	AT2G35130.1
Potra2n12c24723.1	NA	NA	NA	NA	Potri.T117900.1	AT5G12260.1
Potra2n12c24724.1	-0.426919309	0.171402101	0.40677691	0.940841616	Potri.012G122900.1	AT3G14470.1

Potra2n12c24725.1	NA	1.394613675	NA	NA		
Potra2n12c24726.1	-0.261229112	0.114682547	0.409235833	0.94169165	Potri.012G122301.1	AT3G14470.1
Potra2n12c24727.1	-0.279016214	-0.368960513	0.190210266	0.724504724	Potri.012G123700.1	AT2G35120.1
Potra2n12c24728.1	-0.628268752	1.152952889	0.094870101	0.441161882	Potri.012G123900.1	AT3G54950.1
Potra2n12c24729.1	-0.052548438	-0.139211364	0.905795166	0.947969749	Potri.015G122800.1	AT5G18820.1
Potra2n12c24730.1	0.827968017	1.166773781	NA	NA		
Potra2n12c24731.1	0.556435462	0.373278002	0.685892515	0.959881296	Potri.012G124300.1	AT4G08850.1
Potra2n12c24732.1	-0.393345396	0.135559547	0.001863856	0.91974459	Potri.012G124400.1	AT2G35110.2
Potra2n12c24733.1	0.00846865	0.103665191	0.973296382	0.925184207	Potri.012G124500.1	AT1G31170.2
Potra2n12c24734.1	0.029629498	0.103427156	0.853595313	0.885375133	Potri.012G124600.1	AT2G35100.1
Potra2n12c24735.1	5.300649532	2.969403606	0.000586245	NA	Potri.012G125400.1	
Potra2n12c24736.1	1.83332447	1.10567912	0.031794957	0.699507809	Potri.012G124766.1	
Potra2n12c24737.1	1.578268123	0.674963689	NA	NA	Potri.012G125400.1	
Potra2n12c24738.1	5.009906354	0.64247635	0.000774913	NA	Potri.012G125400.1	
Potra2n12c24739.1	1.367073612	0.118863724	0.003877942	0.974275337	Potri.012G124766.1	
Potra2n12c24740.1	0.377878115	0.539299721	0.643381169	0.886059841	Potri.012G125400.1	
Potra2n12c24741.1	-0.201564843	-0.128921671	0.204099202	0.922778027	Potri.012G125500.4	AT5G05010.2
Potra2n12c24742.1	NA	NA	NA	NA	Potri.015G125500.2	AT5G23260.1
Potra2n12c24743.1	-0.619018343	-0.157013121	0.003947333	0.930598202	Potri.012G125800.1	AT5G44860.1
Potra2n12c24744.1	0.552561902	0.04219179	1.21342E-06	0.972201869	Potri.012G125900.1	AT3G12800.1
Potra2n12c24745.2	0.295264603	-0.051652592	0.146228138	0.972649703	Potri.012G126000.2	AT5G62390.1
Potra2n12c24746.1	3.36332354	0.594360668	2.26175E-13	0.727440762	Potri.012G126100.2	AT5G51460.2
Potra2n12c24747.1	NA	NA	NA	NA	Potri.015G127000.1	AT3G26040.1
Potra2n12c24748.1	0.08357059	0.228428612	0.948199824	0.974275337	Potri.012G126300.1	
Potra2n12c24749.1	0.521731642	0.028735737	0.002831824	0.987577016	Potri.012G126400.1	AT4G25240.1
Potra2n12c24750.1	-1.36418401	-0.483852833	9.06012E-14	0.50924822	Potri.012G126500.2	AT1G12260.1
Potra2n12c24751.1	1.64774763	NA	NA	NA	Potri.012G126700.1	AT1G68180.1
Potra2n12c24752.1	4.114008516	1.363811008	0.252529645	NA	Potri.012G126800.1	AT2G45220.1
Potra2n12c24753.1	-1.09611987	-0.52758269	0.553429572	NA	Potri.012G126900.1	AT3G47400.1
Potra2n12c24754.1	0.239069212	0.000786611	0.241865932	0.999536469	Potri.012G127000.1	
Potra2n12c24755.1	0.103070273	-0.034906826	0.553192663	0.976888173	Potri.012G127100.1	AT5G51510.1
Potra2n12c24756.1	-0.362878577	-0.155991855	0.012169156	0.880553952	Potri.012G127300.1	AT4G37880.1

Potra2n12c24757.1	1.148791118	0.307896742	0.532262836	NA	Potri.012G127400.1	AT4G25250.1
Potra2n12c24758.1	0.021914325	-0.139859989	0.924326514	0.905743118	Potri.012G127500.1	AT5G62350.1
Potra2n12c24759.1	NA	1.381953902	NA	NA	Potri.012G108400.1	AT1G26880.1
Potra2n12c24760.1	-1.047584301	-0.639071049	5.88655E-08	0.333044935	Potri.012G127700.1	AT4G12350.1
Potra2n12c24761.1	-0.889985946	-0.141617051	0.001093542	0.919333834	Potri.012G127800.1	AT5G62310.1
Potra2n12c24762.1	-1.566153353	-0.08501018	0.000222211	0.98177802	Potri.012G127900.1	AT5G03170.1
Potra2n12c24763.1	NA	NA	NA	NA	Potri.015G129600.1	AT5G51545.1
Potra2n12c24764.1	-0.968532441	-0.520537104	6.53694E-05	0.788065047	Potri.012G128100.1	AT5G51550.1
Potra2n12c24765.1	-4.689372158	-0.846775581	0.020286395	NA		
Potra2n12c24766.1	-0.423641174	-0.130559319	0.271243925	0.947969749	Potri.012G128200.1	AT1G02000.1
Potra2n12c24767.1	0.704799117	0.055446319	4.46318E-05	0.975578749	Potri.012G128300.1	AT5G62300.2
Potra2n12c24768.2	0.562243299	0.001044165	0.001692653	0.999600335	Potri.012G128500.1	AT5G62290.2
Potra2n12c24769.1	-1.959068943	-0.581213123	7.57015E-06	0.833952007	Potri.012G128700.1	AT5G51560.1
Potra2n12c24770.1	1.279640887	-1.082527277	0.40655701	NA	Potri.012G128800.1	AT3G47800.1
Potra2n12c24771.1	0.525686754	-1.19106518	0.558065739	0.652535461	Potri.012G128900.1	AT5G62280.1
Potra2n12c24772.1	0.483202018	-0.011536855	0.000242621	0.994403137	Potri.012G129000.1	AT5G51570.1
Potra2n12c24773.1	-0.070598982	-0.082850674	0.728709598	0.978203092		
Potra2n12c24774.3	0.468827043	0.077174145	0.03393801	0.972159498	Potri.012G129200.1	AT4G25340.1
Potra2n12c24775.1	1.512458438	0.325009382	0.009075819	0.956865545	Potri.012G129300.1	AT2G36530.1
Potra2n12c24776.1	0.89773274	-0.035708422	0.035834558	0.993819913	Potri.012G129400.1	
Potra2n12c24777.1	-0.143353091	-0.271330898	0.415769423	0.7259398	Potri.012G129500.1	AT5G62260.1
Potra2n12c24778.1	-0.135553418	-0.296405936	0.553983731	0.683816919	Potri.012G129600.1	AT5G51600.1
Potra2n12c24779.3	-0.252903295	-0.080976611	0.102046725	0.948577865	Potri.012G129700.1	AT5G51640.1
Potra2n12c24780.1	0.138923971	0.075946424	0.59383307	0.95671968	Potri.012G129800.3	AT4G25370.1
Potra2n12c24781.1	-0.877898941	-0.305747809	1.13612E-11	0.509370219	Potri.012G129900.1	AT5G51670.1
Potra2n12c24782.2	-0.200948387	0.672591018	0.760513285	0.827798439	Potri.012G130100.1	AT4G22820.2
Potra2n12c24783.1	3.08839762	0.928100445	0.024069564	NA		
Potra2n12c24784.1	-0.090432752	-0.369005028	0.926528249	0.930448915	Potri.012G130300.1	AT4G22860.1
Potra2n12c24785.1	0.053364491	0.150423643	0.979547874	0.98669034	Potri.012G130400.2	AT5G62230.1
Potra2n12c24786.1	0.022843762	0.671940851	0.930591613	0.389056998	Potri.012G130500.1	AT5G51710.2
Potra2n12c24787.1	0.528088276	0.111898237	0.095027957	0.962120332	Potri.012G130600.1	AT5G51720.1
Potra2n12c24788.1	0.713062456	0.123162416	0.011825661	0.951012317	Potri.012G130700.2	AT5G62220.1

Potra2n12c24789.1	0.250319742	-0.245407305	0.6851063	0.940703488		
Potra2n12c24790.1	3.657139282	1.00685728	2.07756E-52	0.657795666	Potri.012G130900.1	AT5G62200.1
Potra2n12c24791.1	0.698664001	0.482818569	0.001377057	0.610104055	Potri.012G131000.1	AT5G62190.1
Potra2n12c24792.1	-0.028583196	0.027865475	0.880260053	0.982221696	Potri.012G131100.1	AT5G51740.1
Potra2n12c24793.1	1.162675682	0.73082292	9.62854E-07	0.262895038	Potri.012G131300.1	AT4G21120.1
Potra2n12c24794.1	0.696563621	0.070965443	0.004559649	0.96243167	Potri.012G131400.1	AT2G28100.1
Potra2n12c24795.1	0.579087578	-0.134649496	0.087978974	0.954098985		
Potra2n12c24796.1	0.771106962	-0.159107097	0.001729095	0.939387548	Potri.012G131500.1	AT5G51750.1
Potra2n12c24797.1	-5.016397585	-0.490096599	0.006758318	0.941677175	Potri.012G131600.1	AT4G32300.1
Potra2n12c24798.2	-0.052432509	0.078338897	0.919447697	0.975578749	Potri.012G131800.1	AT5G51760.1
Potra2n12c24799.1	0.214218164	0.21294841	0.415340677	0.885628484	Potri.012G131900.2	AT4G25390.1
Potra2n12c24800.1	NA	NA	NA	NA	Potri.012G132000.1	AT4G25410.1
Potra2n12c24801.1	3.20104637	0.674963689	NA	NA	Potri.012G132100.2	AT4G25410.1
Potra2n12c24802.1	NA	1.571529889	NA	NA	Potri.012G132300.1	AT5G51800.1
Potra2n12c24803.1	NA	NA	NA	NA		
Potra2n12c24804.1	-0.718938695	-0.857945906	0.080073845	0.606364174	Potri.012G132400.1	AT5G51810.1
Potra2n12c24805.2	0.008727797	0.042149591	0.964610993	0.960713293	Potri.012G132500.1	AT5G51820.1
Potra2n12c24806.2	-0.25723445	-0.038002204	0.693340361	0.994861477	Potri.012G132600.1	AT2G45650.1
Potra2n12c24807.1	-0.194655485	1.737617067	0.945122827	NA		
Potra2n12c24808.2	0.041143539	0.129030926	0.814547757	0.886633917	Potri.012G132700.1	AT5G51830.1
Potra2n12c24809.1	-0.777981119	-0.268391379	0.002588258	0.773063484	Potri.015G135000.1	AT5G51840.1
Potra2n12c24810.1	-1.883692145	-2.033177723	NA	NA	Potri.012G132900.1	AT5G62170.1
Potra2n12c24811.1	-1.593540752	-0.719283457	0.353062104	0.91632176	Potri.012G133000.1	AT5G62165.1
Potra2n12c24812.1	-0.266708912	0.189238575	0.193932468	0.892756772	Potri.012G133101.1	AT5G51880.1
Potra2n12c24813.2	-0.162451993	0.141786189	0.514991582	0.926635994	Potri.012G133200.1	AT5G59090.2
Potra2n12c24814.1	NA	0.674963689	NA	NA	Potri.012G133300.1	AT5G52100.1
Potra2n12c24815.1	0.232591079	-0.585877614	0.609348461	0.439251891	Potri.012G133400.3	AT5G52060.1
Potra2n12c24816.1	0.827740894	-1.190536548	0.299912508	0.772556533	Potri.015G135600.1	AT4G23030.1
Potra2n12c24817.1	NA	3.145099921	NA	NA		
Potra2n12c24818.1	-0.034640724	0.044555868	0.785028212	0.966228173	Potri.012G133600.2	AT4G25500.4
Potra2n12c24819.1					Potri.012G133700.1	AT3G26660.1
Potra2n12c24820.1	-1.35927387	-1.507841271	NA	NA	Potri.012G133800.1	AT5G07210.1

Potra2n12c24821.1	-1.532201102	-0.298152652	0.000704813	0.885900567	Potri.012G133900.1	AT5G52030.2
Potra2n12c24822.1	3.471710278	0.58461751	NA	0.813731863	Potri.012G134000.1	AT5G52020.1
Potra2n12c24823.1	1.928859185	0.295783681	NA	0.885900567	Potri.012G134100.1	AT5G51990.1
Potra2n12c24824.1	-0.345514006	-0.155397355	0.194175831	0.925184207	Potri.012G134200.1	AT1G06010.1
Potra2n12c24825.1	0.499141414	0.290739317	2.05791E-07	0.168761454	Potri.012G134300.2	AT1G16560.3
Potra2n12c24826.1	NA	NA	NA	NA		
Potra2n12c24827.1	-1.357377208	-0.517089876	0.000342523	0.775830532	Potri.012G134500.1	AT5G62140.1
Potra2n12c24828.1	-0.61910233	2.554755255	NA	NA	Potri.012G134400.1	AT4G25450.1
Potra2n12c24829.1	-0.301429329	0.196221407	0.024552556	0.824378527	Potri.012G134600.1	AT1G12580.1
Potra2n12c24830.1	-0.382296097	-0.143230557	0.07177173	0.894138111	Potri.012G134700.1	AT5G43050.1
Potra2n12c24831.1	0.146717688	0.092741164	0.232655412	0.89521294	Potri.012G134800.1	AT4G25440.1
Potra2n12c24832.1	0.685372136	0.65989676	0.000250394	0.683816919	Potri.012G134900.1	AT5G51970.1
Potra2n12c24833.1	0.0861132	0.33478396	0.663033735	0.821709751	Potri.012G135000.1	AT5G51970.2
Potra2n12c24834.1	-0.683099657	-0.244503026	0.000276869	0.844120859	Potri.012G135100.2	AT4G25433.1
Potra2n12c24835.1	-1.921650332	-0.545481417	0.090272844	0.920174237	Potri.012G135200.1	AT5G51950.2
Potra2n12c24836.1	-1.959694598	-0.681046842	0.074593908	0.912777843		
Potra2n12c24837.1	-3.27514021	-1.462347546	0.022342036	0.585704919	Potri.012G135500.1	
Potra2n12c24838.1	-0.140242463	-0.116655446	0.549956357	0.93263929	Potri.012G135600.1	AT4G38010.1
Potra2n12c24839.1	-0.542693195	-0.182951678	0.367384385	0.949966783	Potri.012G135800.1	AT3G45400.1
Potra2n12c24840.1	-0.352733856	0.102580095	0.518354085	0.977530072	Potri.012G135900.1	AT5G51910.2
Potra2n12c24841.1					Potri.015G138300.1	AT5G51890.1
Potra2n12c24842.1	-0.130668216	-0.006482046	0.268469127	0.995579852	Potri.015G138500.1	AT5G62090.2
Potra2n12c24843.1	0.699729494	0.156866384	0.000932995	0.945254452	Potri.012G136200.1	AT5G52120.1
Potra2n12c24844.1	1.459962178	1.394613675	NA	NA	Potri.012G136500.1	
Potra2n12c24845.1	NA	NA	NA	NA	Potri.004G119700.1	AT3G02100.1
Potra2n12c24846.1	2.632524198	1.571529889	NA	NA	Potri.012G136800.1	AT2G45560.1
Potra2n12c24847.1	0.312307643	0.01101546	0.035748176	0.993145334	Potri.012G136900.1	AT5G51940.1
Potra2n12c24848.1	NA	NA	NA	NA	Potri.015G138900.1	AT1G33720.1
Potra2n12c24849.1	NA	NA	NA	NA		
Potra2n12c24850.1	NA	NA	NA	NA	Potri.015G138900.1	AT1G33720.1
Potra2n12c24851.1	2.556135291	NA	NA	NA	Potri.012G137300.1	AT4G00750.1
Potra2n12c24852.1	0.361254711	0.323661239	0.04542507	0.578635193	Potri.012G137500.5	AT4G38180.1

Potra2n12c24853.1	0.135915436	0.075187899	0.662942405	0.970247254	Potri.012G137500.5	AT4G38180.1
Potra2n12c24854.1	5.047045723	1.394613675	0.005764568	NA	Potri.012G137600.1	AT5G45670.1
Potra2n12c24855.1	0.92641307	-0.767708831	NA	NA	Potri.017G125800.1	AT1G66370.1
Potra2n12c24856.1	NA	1.571529889	NA	NA	Potri.017G125800.1	AT1G66370.1
Potra2n12c24857.1	0.102236182	-0.767708831	NA	NA	Potri.017G125800.1	AT1G66370.1
Potra2n12c24858.1	2.131384661	1.037154465	0.434961255	NA	Potri.017G126000.1	AT1G66370.1
Potra2n12c24859.1	3.6691394	1.810643286	0.025159129	NA	Potri.012G137600.1	AT5G45670.1
Potra2n12c24860.1	0.626404585	0.285021498	0.001314209	0.808901406	Potri.012G137700.1	AT3G48880.1
Potra2n12c24861.1	0.449209903	0.067220674	0.000902096	0.936549944	Potri.012G137800.1	AT3G48890.1
Potra2n12c24862.1	-4.593897505	-0.776527933	0.164587139	NA	Potri.012G137900.1	AT2G44250.1
Potra2n12c24863.1	0.366760049	-0.408142388	0.701189843	0.948002367	Potri.012G138100.1	AT1G63245.1
Potra2n12c24864.1	0.028084358	0.186727683	0.865393721	0.815767573	Potri.012G138300.1	AT5G52230.2
Potra2n12c24865.1	NA	1.394613675	NA	NA	Potri.012G138400.1	AT5G52220.2
Potra2n12c24866.1	-0.123957459	-0.049515683	0.355477524	0.960981446	Potri.012G138500.2	AT5G52210.1
Potra2n12c24867.1	0.153828783	-0.024872191	0.315771659	0.981014593	Potri.012G138600.2	AT5G52200.1
Potra2n12c24868.1	NA	NA	NA	NA	Potri.015G140600.1	AT5G52190.1
Potra2n12c24869.1	NA	2.872807905	NA	NA	Potri.012G138800.1	AT5G13930.1
Potra2n12c24870.2	0.443187968	-0.284921967	0.781227731	NA	Potri.015G140800.1	
Potra2n12c24871.1	0.126050103	0.125271713	0.348177208	0.890446513	Potri.012G138900.1	AT5G62020.1
Potra2n12c24872.2	0.264343961	0.224161391	0.220003778	0.831785211	Potri.012G139000.1	AT5G07280.1
Potra2n12c24873.1	1.64774763	1.363811008	NA	NA		
Potra2n12c24874.1	0.181390194	0.656396631	0.950988001	NA	Potri.015G141300.1	AT5G62040.1
Potra2n12c24875.1	-0.540683931	-0.135998705	0.000567371	0.924251575	Potri.012G139100.1	AT5G07270.1
Potra2n12c24876.1	0.592349844	0.039793216	0.00456052	0.984292161	Potri.015G141700.2	AT4G33495.1
Potra2n12c24877.2	0.629705543	0.010187602	0.000192276	0.996032145	Potri.012G139400.2	AT2G36620.1
Potra2n12c24878.1	-0.667852493	-0.210989443	0.02108069	0.914554682	Potri.012G139600.1	AT5G07250.1
Potra2n12c24879.1	-0.837911805	-0.294902358	0.015779549	0.809323756	Potri.012G139800.1	AT5G07240.1
Potra2n12c24880.1	0.056979591	0.09913562	0.688425779	0.914321114	Potri.012G139900.1	AT3G22950.2
Potra2n12c24881.1	0.464277257	0.046046617	0.644076398	0.995005375	Potri.012G140001.1	AT1G73040.1
Potra2n12c24882.1	-2.714588646	0.376910179	NA	NA	Potri.015G139600.1	AT3G13760.1
Potra2n12c24883.1	1.079976385	1.913308015	0.723815711	NA	Potri.007G040500.1	
Potra2n12c24884.1	-1.221377232	-0.364489612	1.46611E-05	0.870614204	Potri.012G140200.1	AT5G23730.1

Potra2n12c24885.1	-0.475591921	0.08582471	3.65977E-05	0.931774514	Potri.015G143300.1	AT4G25550.1
Potra2n12c24886.1	-0.356815364	0.011867086	0.01091888	0.995005375	Potri.012G140400.2	AT3G29230.1
Potra2n12c24887.1	-1.624887715	-0.731835152	0.000167357	0.578635193	Potri.012G140500.1	AT5G52260.1
Potra2n12c24888.1	-2.036536428	-0.840576809	2.6161E-06	0.346327696	Potri.012G140600.1	AT5G52270.1
Potra2n12c24889.1	-0.126957244	0.626944354	0.855643598	0.811017626	Potri.012G140700.1	AT3G61250.1
Potra2n12c24890.1	-1.543531075	-0.56388277	1.21724E-11	0.420046656	Potri.012G140800.1	AT5G23750.2
Potra2n12c24891.1	-2.506352931	-1.411873109	0.473988737	NA	Potri.012G140900.1	AT3G48940.1
Potra2n12c24892.1	-1.365722332	-1.11241438	1.01429E-06	0.000513928	Potri.012G141100.1	AT5G52290.1
Potra2n12c24893.1	0.495365992	0.113850076	0.233158141	0.967122333	Potri.012G141200.2	AT1G63300.1
Potra2n12c24894.1	3.255719224	6.139741377	NA	NA	Potri.012G141300.1	AT4G25580.1
Potra2n12c24895.1	0.555537269	0.141523039	0.237480641	0.956773505	Potri.015G144100.1	AT1G55630.1
Potra2n12c24896.1	-2.783930497	-0.751336968	1.85775E-27	0.347954127	Potri.012G141500.1	AT5G23760.1
Potra2n12c24897.1	-0.954531544	-0.028969095	0.030718982	0.992934677	Potri.012G141700.1	AT5G52380.1
Potra2n12c24898.1	5.873481931	3.818480817	1.07057E-20	0.270576747	Potri.012G141800.1	AT5G52390.1
Potra2n12c24899.1	-1.616881853	0.716576233	0.143117943	0.920141992	Potri.012G141900.1	
Potra2n12c24900.1	2.474718685	-0.108273178	0.272251936	NA	Potri.012G142000.1	AT5G52400.1
Potra2n12c24901.1						
Potra2n12c24902.1	0.27730133	0.104798275	0.00595181	0.836066102	Potri.012G142200.1	AT4G20850.1
Potra2n12c24903.1	0.100767282	0.643334903	0.751630112	0.724604666	Potri.012G142300.1	AT5G23870.3
Potra2n12c24904.1	-0.049394984	-0.053896617	0.726809929	0.965445938	Potri.012G142400.1	AT4G33410.1
Potra2n12c24905.1	0.34625882	-0.07050696	0.030081795	0.953177702	Potri.015G145700.2	AT5G23890.1
Potra2n12c24906.1	0.526653948	-0.015420402	0.00533389	0.993485167	Potri.012G142600.1	AT3G53740.3
Potra2n12c24907.1	-0.050288223	0.05768725	0.760937809	0.939653927	Potri.012G142700.1	AT3G49050.1
Potra2n12c24908.1	0.057475726	0.033337385	0.705131906	0.978251387	Potri.012G142800.1	AT4G25600.1
Potra2n12c24909.1	-0.123734292	0.020868918	0.201394435	0.984292161	Potri.012G143100.1	AT3G49060.1
Potra2n12c24910.1	-0.164513845	-0.014853702	0.943861652	NA	Potri.015G146100.1	AT3G49055.1
Potra2n12c24911.1	-0.218225422	0.033994541	0.232467142	0.978646026	Potri.015G146400.1	AT5G52420.1
Potra2n12c24912.1	-0.248454149	-0.032918936	0.039204229	0.971537796	Potri.012G143500.5	AT3G61415.2
Potra2n12c24913.1	NA	NA	NA	NA	Potri.012G143600.1	AT4G23430.2
Potra2n12c24914.1	-0.111110955	0.100443629	0.607886373	0.940255038	Potri.012G143800.1	AT4G23420.2
Potra2n12c24915.1	-0.215346533	0.11741936	0.288897786	0.923746681	Potri.012G143900.1	AT1G63700.1
Potra2n12c24916.1	NA	NA	NA	NA		

Potra2n12c24917.2	-0.277314949	-0.248064579	0.699593691	0.956865545	Potri.012G144000.1	AT1G02260.1
Potra2n12c24918.1	-0.51282542	-0.075540651	0.003311954	0.956622854	Potri.012G144200.1	AT4G25620.1
Potra2n12c24919.1	-0.274789008	-0.006324895	0.216079951	0.996281171	Potri.012G144300.1	AT5G52440.1
Potra2n12c24920.1	0.370263661	0.214424234	1.90172E-05	0.770451625	Potri.012G144400.1	AT4G09980.1
Potra2n12c24921.1	1.105905054	-1.152261675	0.526173834	NA	Potri.012G144500.1	AT5G23940.1
Potra2n12c24922.2	1.722246737	0.680747533	8.14472E-13	0.148448694	Potri.012G144600.1	AT5G23950.1
Potra2n12c24923.1	0.515460163	0.023709168	0.000929761	0.987577016	Potri.012G144700.1	AT3G49080.1
Potra2n12c24924.1	4.717054785	1.425879898	1.45555E-21	0.318243807	Potri.012G144900.1	AT4G25640.1
Potra2n12c24925.1	0.499209307	0.050842669	0.002681908	0.976888173	Potri.012G145100.2	AT2G24350.1
Potra2n12c24926.2	0.555739207	0.022173515	0.004266554	0.99114586	Potri.012G145000.1	AT3G58660.1
Potra2n12c24927.1	0.031226873	-0.423592592	0.968488669	0.910477984	Potri.012G145200.1	AT5G37010.1
Potra2n1314s37006.2	0.594148665	-0.440649855	0.700675308	NA		
Potra2n1338s37007.1	3.75141623	2.900818469	0.196535134	NA	Potri.009G044800.1	AT5G01260.2
Potra2n133s34691.1	-0.115324112	-0.058105635	0.809382597	0.98394486	Potri.017G019850.1	AT2G43465.1
Potra2n133s34692.1	3.308624605	1.678546078	0.094827226	NA		
Potra2n134s34693.1	NA	NA	NA	NA		
Potra2n134s34694.1	0.005747204	0.025378747	0.977010069	0.981726976	Potri.017G068100.2	AT3G26980.1
Potra2n134s34695.1	1.545832463	1.381953902	NA	NA		
Potra2n134s34696.1	1.082508852	-0.208405684	0.108677245	0.957436721	Potri.017G068400.1	AT5G41050.1
Potra2n134s34697.1	NA	NA	NA	NA	Potri.001G326300.1	AT5G41040.1
Potra2n134s34698.1					Potri.011G085300.1	
Potra2n134s34699.1					Potri.010G034000.1	
Potra2n134s34700.1					Potri.005G151900.1	
Potra2n1358s37008.1	0.642711283	-0.733346312	0.67599643	NA	Potri.017G131700.1	AT3G19940.1
Potra2n1372s37009.1	NA	NA	NA	NA	Potri.005G008200.1	AT5G15020.1
Potra2n1379s37010.1	6.585480033	4.390394609	5.95771E-06	NA	Potri.T102900.2	AT4G08850.1
Potra2n1379s37011.1	6.895886503	4.471573468	3.34038E-07	NA	Potri.T102900.2	AT4G08850.1
Potra2n1379s37012.1						
Potra2n1379s37013.1	3.712344818	0.674963689	0.224023427	NA	Potri.T103200.2	AT4G08850.1
Potra2n1379s37014.1	3.647951417	2.86450839	0.236175444	NA	Potri.T103000.2	AT4G08850.1
Potra2n137s34701.1	1.277130695	0.660048219	0.0003272	0.712599153	Potri.017G033932.1	AT4G10780.1
Potra2n137s34702.1	0.982831945	0.575035307	0.017642245	0.743272231		

Potra2n137s34703.1	2.356819226	0.470297541	0.053141948	NA	Potri.017G034300.1	
Potra2n137s34704.1	0.945740494	0.470297541	0.623546977	NA		
Potra2n137s34705.1	1.458972998	0.570010622	2.1255E-13	0.545369933	Potri.T107066.1	AT5G43740.1
Potra2n137s34706.1	NA	NA	NA	NA	Potri.001G155750.1	AT1G21280.1
Potra2n137s34707.1	NA	2.253733581	NA	NA	Potri.017G034300.1	
Potra2n137s34708.1	-2.307432468	1.357211568	NA	NA	Potri.017G035300.1	AT5G43740.2
Potra2n137s34709.1	1.570452948	0.694588384	0.000410113	0.713368567	Potri.T107066.1	AT5G43740.1
Potra2n137s34710.1	2.160878217	1.760656476	0.35493099	NA	Potri.017G034000.2	
Potra2n137s34711.1					Potri.017G034300.1	
Potra2n137s34712.1	1.30217693	0.463967307	0.160930635	0.945173238	Potri.017G033866.1	AT1G79740.1
Potra2n137s34713.2	6.447517207	3.690341978	2.86077E-05	NA	Potri.017G031000.1	AT4G27410.2
Potra2n137s34714.1	NA	NA	NA	NA	Potri.017G030900.2	AT1G05910.1
Potra2n137s34715.1	1.664677056	NA	NA	NA		
Potra2n137s34716.1	NA	0.674963689	NA	NA	Potri.001G155750.1	AT1G21280.1
Potra2n137s34717.1	6.173401232	2.86450839	0.000456458	NA	Potri.017G031600.1	AT3G10480.3
Potra2n137s34718.1	2.125312886	NA	NA	NA	Potri.017G030900.2	AT1G05910.1
Potra2n137s34719.1	0.372379363	-1.065963596	0.875960973	NA	Potri.017G031500.2	AT1G05910.1
Potra2n1382s37015.1	-0.279316126	-0.293023005	0.01949949	0.32064055	Potri.017G138500.1	AT3G54230.1
Potra2n1382s37016.1	-1.089044269	-0.417756782	0.022509724	0.886059841	Potri.017G136900.1	AT3G14460.1
Potra2n1383s37017.2	-0.012122873	-0.079628637	0.99519204	NA	Potri.018G147800.1	AT4G30130.1
Potra2n1383s37018.1	0.123204879	-0.293538749	0.950127264	NA	Potri.018G147900.1	AT2G34400.1
Potra2n1383s37019.1	1.612329778	0.74214396	0.092213266	0.907073354	Potri.018G144300.4	AT2G25470.1
Potra2n1387s37020.1	NA	2.283425176	NA	NA		
Potra2n1388s37021.1	-0.312763816	-0.004829998	0.070643826	0.997567541	Potri.010G002000.1	AT3G06483.1
Potra2n1388s37022.1	0.02589462	0.287672065	0.940126215	0.824731243	Potri.010G002100.1	AT5G30495.2
Potra2n1389s37023.1	1.664677056	NA	NA	NA	Potri.018G147400.2	AT5G35400.1
Potra2n1389s37024.1					Potri.001G063200.1	AT4G37070.1
Potra2n1389s37025.1	7.633525247	4.247704464	5.05735E-10	0.773779643	Potri.001G063300.5	AT1G74170.1
Potra2n1389s37026.1	-0.890620466	1.55613583	NA	NA		
Potra2n1389s37027.1	3.255719224	1.571529889	NA	NA		
Potra2n1390s37028.1					Potri.017G145901.1	AT3G48950.1
Potra2n1390s37029.1					Potri.013G138500.1	ATCG01280.1

Potra2n1390s37030.1	4.307039283	4.689678277	0.086437551	NA	Potri.013G143400.1	ATCG00860.1
Potra2n1390s37031.1	-1.861177961	0.438356919	0.009812608	0.862830524	Potri.004G073900.1	AT3G48950.1
Potra2n1390s37032.1	NA	NA	NA	NA		
Potra2n1391s37033.1	3.186696357	1.244202865	0.162830977	NA	Potri.011G104600.1	AT2G15080.2
Potra2n1391s37034.1					Potri.001G383900.10	AT1G53490.1
Potra2n1391s37035.1					Potri.001G207400.1	
Potra2n1392s37036.1	0.032673984	0.223356546	0.886063449	0.845014308	Potri.004G101600.1	
Potra2n1392s37037.1	-0.451078692	0.119616679	0.049018155	0.95671968	Potri.011G039800.2	AT1G11360.1
Potra2n1392s37038.1	0.493707106	-1.217755705	0.422609013	0.52733716	Potri.001G155750.1	AT1G21280.1
Potra2n1393s37039.2	3.828734571	4.2754886	0.322975474	NA	Potri.004G052400.1	AT2G29120.1
Potra2n1395s37040.1					Potri.006G078800.1	AT2G26590.2
Potra2n1396s37041.1	9.135360344	5.115610103	4.74509E-14	NA	Potri.003G013700.1	AT1G58190.2
Potra2n1396s37042.1					Potri.001G063400.1	
Potra2n1396s37043.1	1.509857342	-0.835002147	0.353924843	NA	Potri.003G013600.1	
Potra2n1398s37044.1	0.314458006	-0.015496369	0.900583952	NA	Potri.011G017550.1	AT3G54070.1
Potra2n1398s37045.1						
Potra2n13c24928.1	0.085744014	0.206015232	0.628255433	0.84745523	Potri.013G161700.1	AT4G10180.1
Potra2n13c24929.1	0.099928075	0.435365365	0.668118215	0.813731863	Potri.013G161600.6	AT4G20350.2
Potra2n13c24930.1	0.152109023	0.40693331	0.495512896	0.826153221	Potri.013G161500.2	AT5G10860.1
Potra2n13c24931.3	-0.088235574	-0.096443076	0.595313907	0.918791843	Potri.013G161200.1	AT1G31360.1
Potra2n13c24932.1	0.14217072	0.022086757	0.520320997	0.989304496	Potri.013G161300.1	AT1G09575.1
Potra2n13c24933.1	5.940309202	2.261559358	1.325E-25	0.190971261	Potri.013G161100.3	AT5G58240.1
Potra2n13c24934.1	-0.372000142	-0.287742743	0.066774344	0.650005812	Potri.013G161000.1	AT5G58250.1
Potra2n13c24935.1	-1.080103752	-0.383643799	0.031717407	0.895474078	Potri.013G160900.2	AT4G23630.1
Potra2n13c24936.2	7.771920459	3.985036489	5.29338E-08	0.704368358	Potri.013G160800.1	AT5G24910.1
Potra2n13c24937.1	-3.650653274	-1.02677788	7.55824E-11	0.436464795	Potri.013G160600.1	AT5G58260.1
Potra2n13c24938.1	0.828004246	0.093488606	4.59735E-11	0.936549944	Potri.013G160500.1	AT5G58270.1
Potra2n13c24939.1	1.213539903	0.138133171	0.003150189	0.974275337	Potri.013G160300.1	
Potra2n13c24940.3	0.387041547	-0.443846218	0.058461682	0.749682671	Potri.013G159200.5	AT1G06840.1
Potra2n13c24941.1						
Potra2n13c24942.1	0.740371016	0.060381523	1.80747E-07	0.973365801	Potri.013G159600.2	AT1G26910.1
Potra2n13c24943.1	0.116705242	-0.060293319	0.496725742	0.95999889	Potri.013G159700.1	AT3G10860.1

Potra2n13c24944.1	0.182740483	-0.104627861	0.495793048	0.943483421	Potri.013G159800.2	AT2G38610.1
Potra2n13c24945.1	1.722601943	1.150472198	0.430847938	0.886281281		
Potra2n13c24946.1	0.03616795	-0.316518552	0.934461586	0.819353048		
Potra2n13c24947.3	0.031310372	-0.329599575	0.944544393	0.817452127	Potri.008G220300.1	
Potra2n13c24948.1	-0.408366453	-0.716273012	0.822843166	NA	Potri.T140200.1	AT1G30760.1
Potra2n13c24949.1	-0.324215007	0.427938949	0.136675663	0.874507876	Potri.013G159000.1	AT2G30520.1
Potra2n13c24950.1	0.396009747	-0.094108159	0.886098461	NA		
Potra2n13c24951.1	-0.52976541	-0.297626291	0.00483792	0.887678043	Potri.013G158800.1	AT5G58300.2
Potra2n13c24952.1	2.17394525	0.12962447	6.69372E-06	0.971424617	Potri.013G158500.1	AT4G27950.1
Potra2n13c24953.1	0.084506843	-0.075992964	0.449576765	0.882922789	Potri.013G158400.1	AT5G20920.3
Potra2n13c24954.1	-2.396052862	-0.165608181	NA	NA	Potri.013G158300.1	AT5G49350.1
Potra2n13c24955.2	-1.343582011	0.52546057	0.04949543	0.879168574	Potri.013G158200.1	AT3G10870.1
Potra2n13c24956.1	-0.449499287	0.20384093	0.003438589	0.80340893	Potri.013G158100.1	AT5G58320.2
Potra2n13c24957.1	-0.336271717	0.152113346	0.002674695	0.806022635	Potri.013G158000.1	AT1G55150.1
Potra2n13c24958.1	-0.08104558	-0.203880665	0.871704302	0.937687311	Potri.009G061100.1	AT3G08040.1
Potra2n13c24959.1	-0.338219444	-0.138608368	0.036743811	0.935099709	Potri.013G157900.1	AT2G30490.1
Potra2n13c24960.1	0.275712617	0.261360516	0.077073198	0.669204552	Potri.013G157800.1	AT2G30480.3
Potra2n13c24961.1	-0.282117976	-0.396713935	0.740968222	0.912083068	Potri.019G133600.3	AT3G56380.1
Potra2n13c24962.1	0.067909902	0.228644514	0.91070032	0.941565139	Potri.013G157700.1	AT2G40630.1
Potra2n13c24963.2	0.44879909	-1.006670486	0.434717125	0.421053611	Potri.013G157500.2	AT2G30470.1
Potra2n13c24964.1	2.235338028	NA	NA	NA	Potri.013G157200.1	AT4G39950.1
Potra2n13c24965.1	4.322904203	NA	0.206085923	NA	Potri.013G157200.1	AT4G39950.1
Potra2n13c24966.1	4.419375996	NA	0.188270688	NA	Potri.013G157200.1	AT4G39950.1
Potra2n13c24967.1						
Potra2n13c24968.1	NA	NA	NA	NA	Potri.001G155750.1	AT1G21280.1
Potra2n13c24969.1	0.82357019	1.363811008	NA	NA	Potri.013G157100.1	AT1G31930.3
Potra2n13c24970.1	-1.514288653	-0.402473007	3.33634E-10	0.677626898	Potri.013G157000.1	AT3G10910.1
Potra2n13c24971.1	-0.307741329	-0.291539338	0.087839194	0.755774501	Potri.013G156900.1	AT2G40620.1
Potra2n13c24972.1	-0.429243093	-0.173408244	0.004117895	0.829862776	Potri.013G156700.1	AT2G30330.1
Potra2n13c24973.1	3.927379673	0.836541019	3.11324E-06	0.857374454	Potri.013G156500.2	AT5G05340.1
Potra2n13c24974.1	1.64774763	0.674963689	NA	NA	Potri.013G156400.2	AT5G05340.1
Potra2n13c24975.1	-0.495138137	-0.172376926	0.026594055	0.917516356	Potri.013G156300.1	

Potra2n13c24976.1	-0.662679266	-0.462627089	0.002008529	0.653804505	Potri.013G156200.1	AT2G40470.1
Potra2n13c24977.1	-0.808963147	-0.387254464	0.006674763	0.788065047	Potri.013G156100.1	AT1G06930.1
Potra2n13c24978.2	-0.818762978	-0.003156122	3.20995E-06	0.999325699	Potri.013G156000.1	AT5G58380.1
Potra2n13c24979.1	0.269946043	0.154661095	0.547320409	0.948002367	Potri.013G155900.1	AT2G30350.2
Potra2n13c24980.1	0.070459137	-0.012852272	0.750554952	0.993815835	Potri.013G155800.1	AT5G58375.1
Potra2n13c24981.1	-3.303937549	-0.822102035	0.062357522	0.903698951	Potri.019G128000.2	AT5G58370.2
Potra2n13c24982.1	-0.922354892	0.794819032	NA	NA		
Potra2n13c24983.1	-0.204953516	-0.065985952	0.242684411	0.974367223	Potri.013G155600.1	AT2G30360.1
Potra2n13c24984.1	0.720811644	-0.70126896	0.248810832	0.856660279	Potri.013G155500.1	AT2G30370.1
Potra2n13c24985.1	0.203188763	1.574146241	NA	NA	Potri.013G155400.1	AT2G45260.1
Potra2n13c24986.1	-1.123778913	-0.360815088	3.98108E-08	0.564764759		
Potra2n13c24987.1	-1.288107066	-0.375633711	1.48726E-05	0.712127394	Potri.013G155300.1	AT2G30395.1
Potra2n13c24988.1	-3.497025542	-1.724449672	0.000862635	0.349324124	Potri.013G155200.1	AT1G06920.1
Potra2n13c24989.1	0.45883838	0.02686873	0.000523674	0.987226805	Potri.013G155100.5	AT5G58350.1
Potra2n13c24990.1	-0.139220856	0.151426609	0.507009208	0.930871994	Potri.013G154900.1	AT3G51620.2
Potra2n13c24991.1	-0.323173876	-0.240759409	0.009566646	0.830470159	Potri.013G154800.1	AT1G06890.1
Potra2n13c24992.1	0.922088396	0.547660118	0.051496692	0.872570395	Potri.013G154700.1	AT2G40610.1
Potra2n13c24993.1	0.338895769	0.117892202	0.00082129	0.885375133	Potri.013G154600.1	AT1G06900.1
Potra2n13c24994.1						
Potra2n13c24995.1	-0.155726398	0.009608054	0.258619586	0.993589888	Potri.013G154500.1	AT5G58340.1
Potra2n13c24996.1	3.38618284	NA	NA	NA	Potri.013G154400.1	AT5G05340.1
Potra2n13c24997.1	-0.205206059	0.174050453	0.1011314	0.869327705	Potri.013G154300.2	AT1G06950.1
Potra2n13c24998.1	0.323333859	0.157954856	0.137313092	0.930059926	Potri.013G154200.1	AT2G30320.1
Potra2n13c24999.1	1.214571816	1.035793545	0.000110125	0.130979124	Potri.013G154100.1	AT2G40435.1
Potra2n13c25000.1	-0.937155612	1.32774173	0.071528672	NA	Potri.013G154000.1	AT2G30300.1
Potra2n13c25001.1	-0.01574342	-0.281012295	0.944755644	0.716548554	Potri.013G153900.1	AT1G16870.1
Potra2n13c25002.1	NA	NA	NA	NA	Potri.013G153800.2	AT3G10985.1
Potra2n13c25003.2	-0.215143811	-0.050947839	0.161404275	0.959881296	Potri.013G153700.1	AT2G30260.1
Potra2n13c25004.1	-1.981502873	0.236007949	1.66749E-12	0.873823526	Potri.019G123400.1	AT5G01720.1
Potra2n13c25005.1	1.658591096	0.532433509	5.32117E-09	0.441009549	Potri.013G153400.1	AT2G38470.1
Potra2n13c25006.1	4.15973402	2.141662216	0.119335818	NA	Potri.013G153200.1	AT2G13620.1
Potra2n13c25007.1	-0.430275818	-0.310009502	0.572391034	0.906545272	Potri.013G153100.1	AT2G30230.1

Potra2n13c25008.1	1.213777106	-0.172445457	0.195345533	0.98036348	Potri.013G153000.1	AT2G30220.1
Potra2n13c25009.1	0.373535733	-0.011827129	0.091362556	0.995005375	Potri.013G152900.1	AT5G58430.1
Potra2n13c25010.1	0.311182274	0.157234923	7.03003E-05	0.643884997	Potri.013G152800.1	AT5G58450.1
Potra2n13c25011.1	2.028027975	-0.004544353	1.08653E-22	0.99905083	Potri.013G152700.1	AT2G30210.1
Potra2n13c25012.1	0.340940249	-0.155840924	0.004731542	0.814981587	Potri.013G152600.1	AT5G37055.1
Potra2n13c25013.1	NA	3.116166474	NA	NA		
Potra2n13c25014.1	1.392974253	-0.429985658	6.37884E-08	0.759797728	Potri.013G152400.1	AT4G03210.1
Potra2n13c25015.2	2.094522508	0.332987035	NA	0.624684308	Potri.013G152200.1	AT5G06920.1
Potra2n13c25016.1	0.374185455	0.167040372	0.131443114	0.923397137	Potri.013G152100.1	AT2G16030.1
Potra2n13c25017.1	0.023268983	-0.031470872	0.939738031	0.987226805	Potri.013G151900.2	
Potra2n13c25018.1	0.226375422	0.158594938	0.097100898	0.824905557	Potri.013G151700.1	AT4G14096.1
Potra2n13c25019.1	0.454552124	2.228161041	0.630012226	0.311528258	Potri.013G151600.1	AT3G01610.1
Potra2n13c25020.1	0.618854541	3.240139632	0.67873787	0.259010764		
Potra2n13c25021.1	0.29042082	2.267068257	0.870183035	0.585704919	Potri.013G151300.1	AT5G60490.1
Potra2n13c25022.1	-2.337106966	-2.486725844	NA	NA	Potri.013G151200.1	AT4G03220.1
Potra2n13c25023.1	-2.00991008	-0.461047409	0.167496671	0.962971989	Potri.013G151100.1	AT5G54510.1
Potra2n13c25024.1	2.157167715	0.604044356	0.000559059	0.724604666	Potri.013G151000.1	AT5G54490.1
Potra2n13c25025.1	0.09008872	-0.400321016	0.892011075	0.813731863	Potri.019G120300.1	AT4G21380.1
Potra2n13c25026.1	1.471255377	1.258712002	0.024993593	0.53785485	Potri.013G150800.1	AT4G03230.1
Potra2n13c25027.2	0.457674965	-2.094579377	0.849362987	NA	Potri.019G119600.3	AT4G03230.1
Potra2n13c25028.1	-0.391944417	0.089244695	0.045404669	0.974264269	Potri.013G150500.1	AT5G54470.1
Potra2n13c25029.1	2.963977803	NA	NA	NA		
Potra2n13c25030.1	-0.160381892	-0.04647967	0.453868244	0.9761362	Potri.013G150400.2	AT4G03240.1
Potra2n13c25031.1	-0.141973096	0.041486877	0.468579368	0.977227089	Potri.013G150300.2	AT4G03260.2
Potra2n13c25032.2	0.060261133	-0.0071598	0.818786663	0.996748906	Potri.013G150200.1	AT1G11360.2
Potra2n13c25033.2	-0.234446483	0.126867241	0.029173465	0.910815461	Potri.013G150100.1	AT1G62930.1
Potra2n13c25034.1	0.531726429	0.409427957	0.213302867	0.864019566	Potri.013G149800.1	AT1G12700.1
Potra2n13c25035.1					Potri.013G122801.1	AT1G27410.1
Potra2n13c25036.1	-0.052270173	1.286595799	NA	NA	Potri.013G121700.2	AT1G27410.1
Potra2n13c25037.1					Potri.013G121600.1	AT1G12700.1
Potra2n13c25038.1					Potri.013G121600.1	AT1G12700.1
Potra2n13c25039.1	3.090810333	1.338035069	1.20502E-18	0.231944696	Potri.013G149500.1	AT2G19130.1

Potra2n13c25040.1	-0.095097642	-0.676973753	0.865510472	0.650936786	Potri.013G149400.1	AT1G15030.1
Potra2n13c25041.1					Potri.013G149300.1	AT5G23160.1
Potra2n13c25042.1	2.173277918	-3.470212978	0.120147311	NA	Potri.013G149200.1	AT2G31180.1
Potra2n13c25043.1	4.912946778	2.173157871	8.64101E-07	NA	Potri.013G149100.1	AT3G23250.1
Potra2n13c25044.1	-0.379616425	-0.339089016	0.721146495	0.935851032	Potri.013G149000.1	AT4G03270.1
Potra2n13c25045.1	-2.233316291	-0.405223079	1.27745E-14	0.526367527	Potri.013G148900.1	AT4G03280.1
Potra2n13c25046.1	1.64774763	NA	NA	NA	Potri.019G118400.1	AT4G05120.1
Potra2n13c25047.1	-0.367884958	-0.289325356	0.15674787	0.794641467	Potri.013G148700.1	AT4G28100.1
Potra2n13c25048.1	-2.649754791	-2.799286845	NA	NA	Potri.013G148600.1	AT4G21440.1
Potra2n13c25049.1	-0.623044286	-0.370921414	0.000216815	0.595352688	Potri.013G148500.1	AT2G20900.2
Potra2n13c25050.3	-0.715493773	-0.352184411	2.90647E-07	0.587011639	Potri.013G148400.2	AT2G20890.1
Potra2n13c25051.1	-0.890620466	0.310682761	NA	NA		
Potra2n13c25052.1	NA	NA	NA	NA	Potri.013G147900.1	AT4G10270.1
Potra2n13c25053.1	-0.898428161	-0.487264802	3.20959E-06	0.442002381	Potri.013G147800.2	AT4G21450.1
Potra2n13c25054.1	-1.463997576	-0.15629376	0.235789489	0.979896883		
Potra2n13c25055.1	-0.644094367	-0.267545227	0.002292624	0.814001428	Potri.013G147700.1	AT4G28240.1
Potra2n13c25056.1	-0.162095634	0.132917077	0.220960511	0.866777645	Potri.013G147600.1	AT1G12470.1
Potra2n13c25057.1	-0.15227839	0.148962495	0.342104956	0.874595634		
Potra2n13c25058.1	0.256534367	-0.634116007	0.653183563	0.76936007	Potri.013G147500.1	AT4G28230.1
Potra2n13c25059.1	0.678793431	-0.720109479	0.72239025	NA	Potri.013G147400.1	AT2G20800.1
Potra2n13c25060.1	-0.170081267	0.153166357	0.406196499	0.90634987	Potri.013G147300.1	AT2G20800.1
Potra2n13c25061.1	-0.624424802	-0.132556653	0.607661738	0.98177802	Potri.013G147100.1	
Potra2n13c25062.1	0.255377933	0.18111912	0.095703541	0.871720501	Potri.013G146900.1	AT4G28210.1
Potra2n13c25063.1					Potri.013G146800.1	AT1G69630.1
Potra2n13c25064.1	0.49162386	0.391386438	0.08549487	0.679485917	Potri.013G146700.3	AT4G31980.1
Potra2n13c25065.1	3.571706601	-0.037599366	0.039096336	NA	Potri.013G146300.1	AT4G31980.1
Potra2n13c25066.1	0.057414035	0.083248299	0.835116827	0.953831244	Potri.013G146200.1	AT2G20810.1
Potra2n13c25067.1	2.81664765	1.866265004	0.266995808	NA	Potri.013G146100.3	AT2G20815.1
Potra2n13c25068.1	0.289923858	-0.023914288	0.056079648	0.985393296	Potri.013G146000.2	AT2G20820.1
Potra2n13c25069.1	0.88191549	0.652873851	0.358784676	0.896306549	Potri.013G145900.1	
Potra2n13c25070.1	-0.260279457	0.015858989	0.165247767	0.992934677	Potri.013G145700.1	AT2G20830.1
Potra2n13c25071.1	-0.618958998	0.712373328	0.191917514	0.826265918	Potri.013G145600.1	AT2G20830.1

Potra2n13c25072.1	-0.538025905	-0.139935669	0.006390337	0.89438295	Potri.013G145500.1	AT1G03520.1
Potra2n13c25073.2	-0.664211385	-0.34293654	3.31962E-07	0.657795666	Potri.013G145400.1	AT4G28190.1
Potra2n13c25074.1	0.225377552	-0.0021966	0.153353626	0.999458638	Potri.019G104400.1	AT1G03530.1
Potra2n13c25075.1	-0.052270173	-1.55593228	NA	NA		
Potra2n13c25076.1	-0.390328722	-0.251624296	0.520099865	0.909715408	Potri.013G145000.1	AT1G03540.1
Potra2n13c25077.1	-0.29541335	0.014656952	0.124044787	0.993589787	Potri.013G144800.2	AT1G73710.1
Potra2n13c25078.1	-0.289814429	-0.073969876	0.075625138	0.956865545	Potri.013G144700.1	AT2G20840.1
Potra2n13c25079.1	0.252842083	0.052643468	0.321703789	0.974367223	Potri.013G144600.1	AT4G03390.1
Potra2n13c25080.1	1.274052861	0.123244526	0.136675604	0.9869326	Potri.019G103800.1	AT1G11120.2
Potra2n13c25081.1					Potri.013G144400.1	
Potra2n13c25082.1	1.459962178	0.674963689	NA	NA	Potri.013G144300.1	AT4G03400.1
Potra2n13c25083.1	-1.35927387	-1.507841271	NA	NA		
Potra2n13c25084.1	-0.270583397	-0.115802406	0.055575155	0.920528558	Potri.013G144250.1	
Potra2n13c25085.1	-0.9751313	0.082864717	0.074272	0.979273211	Potri.013G144200.1	AT2G20870.1
Potra2n13c25086.1	0.736631443	0.986718585	1.96356E-06	0.220736763	Potri.013G144100.1	AT4G03415.2
Potra2n13c25087.3	0.054836362	0.773981784	0.864942132	0.607742267	Potri.013G144000.1	AT4G03420.1
Potra2n13c25088.1	0.277536295	0.303521108	0.027406097	0.836633315	Potri.013G143900.1	AT5G17050.1
Potra2n13c25089.1	3.049300294	0.861581023	0.006748725	NA		
Potra2n13c25090.1	1.664677056	1.363811008	NA	NA	Potri.013G136400.1	AT4G35160.1
Potra2n13c25091.3	0.585897627	-0.279388494	0.003382635	0.788065047	Potri.013G136400.1	AT4G35160.1
Potra2n13c25092.1	NA	2.0078969	NA	NA		
Potra2n13c25093.1	-1.121306029	-0.449037087	0.002236639	0.778425859		
Potra2n13c25094.1	-1.00602167	-0.374494122	0.011876208	0.842247476	Potri.013G136200.1	AT1G03620.1
Potra2n13c25095.1	NA	NA	NA	NA	Potri.013G136100.1	AT2G20875.1
Potra2n13c25096.1	-0.261633183	-0.135766168	0.107258854	0.910477984	Potri.013G136000.1	AT5G08415.1
Potra2n13c25097.1	2.758733088	-2.759845607	0.037984001	NA	Potri.013G135900.1	
Potra2n13c25098.1	7.0711281	2.490806657	1.14279E-07	NA	Potri.013G135800.1	AT1G65690.1
Potra2n13c25099.1	-0.672826433	0.043273015	0.097009309	0.989878071	Potri.013G135700.1	AT2G14830.1
Potra2n13c25100.1	-1.317292482	1.066897328	NA	NA		
Potra2n13c25101.1	-0.216024165	-0.331598632	0.662542203	0.677589151	Potri.013G135600.1	AT2G20880.1
Potra2n13c25102.1	-0.567165043	-0.391974674	0.014344345	0.367555634	Potri.013G135500.1	AT1G03630.1
Potra2n13c25103.1	-0.169509361	-0.082122024	0.116063504	0.910477984	Potri.013G135300.2	AT4G03430.1

Potra2n13c25104.1	-0.593939516	-0.304014288	0.000377867	0.76350478	Potri.013G135200.1	AT2G20780.1
Potra2n13c25105.1	-0.071101738	-0.032342501	0.773377768	0.982221696	Potri.013G135100.1	AT4G02340.1
Potra2n13c25106.1	0.613311481	0.153402531	1.56229E-06	0.859892315	Potri.013G134700.3	AT2G20770.1
Potra2n13c25107.1	0.343232958	0.189062859	0.250149335	0.869186333	Potri.013G134800.1	AT4G02340.1
Potra2n13c25108.1	1.692473834	1.167753621	5.9698E-06	0.550830296	Potri.013G133400.2	AT1G03670.1
Potra2n13c25109.1	1.459962178	NA	NA	NA	Potri.013G133400.2	AT1G03670.1
Potra2n13c25110.1	1.815822694	1.253807004	2.13427E-10	0.53266226	Potri.013G133800.1	AT1G03670.1
Potra2n13c25111.1						
Potra2n13c25112.1	NA	NA	NA	NA	Potri.005G047800.1	AT2G38050.1
Potra2n13c25113.1	NA	1.885226318	NA	NA	Potri.019G102000.1	AT2G20760.1
Potra2n13c25114.1	1.637972453	1.934843381	0.000724727	0.00111573	Potri.013G133800.1	AT1G03670.1
Potra2n13c25115.1	NA	NA	NA	NA	Potri.019G102000.1	AT2G20760.1
Potra2n13c25116.1	0.82357019	NA	NA	NA	Potri.013G134400.1	AT2G02990.1
Potra2n13c25117.1	NA	NA	NA	NA	Potri.013G134400.1	AT2G02990.1
Potra2n13c25118.1	1.150967413	2.048277104	0.307418726	0.489910701	Potri.013G134500.1	AT1G03670.1
Potra2n13c25119.1	5.482619366	2.582059139	3.85217E-05	NA	Potri.013G133400.2	AT1G03670.1
Potra2n13c25120.1	-1.64473862	-0.155875588	NA	NA	Potri.013G134400.1	AT2G02990.1
Potra2n13c25121.1	-2.277249482	-2.42671106	NA	NA	Potri.013G134300.1	AT4G28250.1
Potra2n13c25122.2	-0.271951997	0.015596051	0.228434813	0.993577196	Potri.013G134200.1	AT2G20740.1
Potra2n13c25123.1	0.77892458	0.43211216	0.076771973	0.902676973	Potri.013G133400.2	AT1G03670.1
Potra2n13c25124.1	NA	1.363811008	NA	NA	Potri.013G133400.2	AT1G03670.1
Potra2n13c25125.1	0.844200644	-0.617774907	0.732641754	NA	Potri.013G133900.2	AT1G03670.1
Potra2n13c25126.1	2.084574005	0.465984278	0.157031765	NA	Potri.013G133800.1	AT1G03670.1
Potra2n13c25127.4	-0.302871662	-0.210059821	0.646201507	0.947969749		
Potra2n13c25128.2	-0.599831099	-0.671905907	0.332472639	0.821709751	Potri.013G133800.1	AT1G03670.1
Potra2n13c25129.1	0.848315298	0.784329453	0.059697667	0.741833788	Potri.013G133400.2	AT1G03670.1
Potra2n13c25130.1	NA	1.363811008	NA	NA	Potri.013G133400.2	AT1G03670.1
Potra2n13c25131.1	0.82357019	0.674963689	NA	NA	Potri.013G133900.2	AT1G03670.1
Potra2n13c25132.1	NA	NA	NA	NA		
Potra2n13c25133.1	2.417930419	2.032498096	NA	NA	Potri.013G133400.2	AT1G03670.1
Potra2n13c25134.1	-0.220360814	-0.197672773	0.850530623	0.974275337	Potri.013G133700.1	AT1G03670.1
Potra2n13c25135.1	0.849301826	0.826774492	0.195103175	0.844152485	Potri.013G133400.2	AT1G03670.1

Potra2n13c25136.1	0.402608274	0.829035108	0.22599665	0.736149483	Potri.013G134000.1	AT1G03650.1
Potra2n13c25137.1	-0.231083924	-2.296022074	0.929923226	NA	Potri.013G133800.1	AT1G03670.1
Potra2n13c25138.1	0.398236305	0.465067366	0.149197455	0.565419698		
Potra2n13c25139.1	-0.17875522	0.207255911	0.926983455	NA	Potri.013G133900.2	AT1G03670.1
Potra2n13c25140.1	-1.068970793	-0.7396777	0.266709036	0.88661378	Potri.013G133800.1	AT1G03670.1
Potra2n13c25141.1	-0.852750936	-0.005733182	0.266636192	0.999325699	Potri.013G133700.1	AT1G03670.1
Potra2n13c25142.1	1.099467585	-0.235868928	0.002130575	0.939824856	Potri.013G133400.2	AT1G03670.1
Potra2n13c25143.1	-1.068139791	-0.52939357	4.39376E-09	0.414931285		
Potra2n13c25144.1	-0.558867449	-0.089604401	0.000221769	0.935099709	Potri.013G133201.1	AT4G28260.1
Potra2n13c25145.1	-0.009452376	0.169666164	0.975866516	0.900551258	Potri.013G133000.2	AT2G20710.1
Potra2n13c25146.1	-0.048691177	0.089910701	0.893747461	0.966917036	Potri.013G132700.1	AT2G20710.1
Potra2n13c25147.1	0.013209662	0.079060055	0.956717998	0.961820855	Potri.013G132500.10	AT2G20710.1
Potra2n13c25148.1	0.46062302	0.517334196	0.268015824	0.779077802	Potri.013G132300.1	AT4G03510.2
Potra2n13c25149.1	-2.531499847	-0.34511478	0.182244293	NA		
Potra2n13c25150.1	-2.341229479	-0.294170631	0.027362501	0.954476684	Potri.013G132200.1	AT4G03520.1
Potra2n13c25151.5	0.161286104	0.289683251	0.364362464	0.488245855	Potri.013G132100.1	AT1G03687.1
Potra2n13c25152.1	-1.036773363	-0.367864666	0.000854683	0.779135176	Potri.013G131900.1	AT4G28290.1
Potra2n13c25153.1	-0.160554149	-0.258233717	0.372351239	0.71411664	Potri.013G131800.1	AT2G20690.1
Potra2n13c25154.1	-0.393801525	-0.725807918	0.649435682	0.821709751	Potri.013G131700.1	AT1G03700.1
Potra2n13c25155.1	0.36250187	-0.100861857	0.011609425	0.879379661	Potri.013G131600.2	AT4G28300.1
Potra2n13c25156.1	5.199673997	3.408150094	0.001822272	NA	Potri.013G131500.1	AT5G13900.1
Potra2n13c25157.2	0.110103305	-0.063630453	0.497457908	0.95671968	Potri.011G052400.1	AT4G04970.1
Potra2n13c25158.1	1.887337639	-0.035298164	1.1763E-29	0.982422288	Potri.013G131200.2	AT3G09390.1
Potra2n13c25159.1	-0.456918171	-0.001586514	0.095150035	0.999458638	Potri.013G131100.2	AT4G03560.1
Potra2n13c25160.1	-0.362837891	0.228129298	0.738597859	0.974367223	Potri.013G131000.1	AT1G23120.1
Potra2n13c25161.1	-0.020296696	-0.082137535	0.899385929	0.929938231	Potri.013G130800.1	AT1G53025.1
Potra2n13c25162.1	-0.043562463	-0.117142129	0.807443453	0.877655612	Potri.013G130900.1	AT4G26930.1
Potra2n13c25163.1	-0.990478718	-2.593986469	0.671828528	NA		
Potra2n13c25164.3	-0.189427351	-0.09526714	0.640575221	0.964927721	Potri.013G130600.3	AT1G12775.1
Potra2n13c25165.1	-1.815782399	-0.013242699	0.366634113	NA	Potri.013G130500.1	AT1G10880.1
Potra2n13c25166.1	-0.639317488	-0.450693417	0.004253921	0.788065047	Potri.013G130400.1	AT2G20680.1
Potra2n13c25167.1	1.315395558	-0.145699301	0.578982771	NA		

Potra2n13c25168.1	-0.434871021	-0.19082524	0.020934636	0.775343511	Potri.013G130300.1	AT4G03600.1
Potra2n13c25169.1	-2.522632374	-1.70156226	0.34478556	NA	Potri.013G130200.1	
Potra2n13c25170.1	-0.682467168	-0.044697298	0.729854608	0.996032145	Potri.013G130100.1	AT1G53035.1
Potra2n13c25171.1	0.357239691	0.086701841	0.178744397	0.910815461	Potri.013G130000.1	AT1G53050.1
Potra2n13c25172.1	-0.858221915	-0.127330721	0.011865636	0.965230114	Potri.013G129900.1	AT4G32480.1
Potra2n13c25173.1	3.951466579	1.600093172	1.34524E-06	0.390804044	Potri.013G129800.2	AT4G20970.1
Potra2n13c25174.1	-2.307432468	-0.10092819	NA	NA	Potri.013G129100.2	AT5G09950.1
Potra2n13c25175.1	NA	NA	NA	NA	Potri.013G129500.2	AT5G09950.1
Potra2n13c25176.2	-0.182677559	-0.024105238	0.309626838	0.981980715	Potri.013G129600.1	AT5G47320.1
Potra2n13c25177.1	-0.070442091	0.217354177	0.747677927	0.736944354	Potri.013G129500.2	AT5G09950.1
Potra2n13c25178.1	-0.440062873	0.119645871	0.001174576	0.875559093	Potri.013G128900.3	AT1G17680.2
Potra2n13c25179.1	0.675073579	0.268066262	1.32878E-06	0.72101822	Potri.013G128600.4	AT5G39740.2
Potra2n13c25180.1	NA	2.676364087	NA	NA	Potri.013G128800.1	AT4G12480.1
Potra2n13c25181.1	0.139355187	0.055432397	0.23407596	0.939915707	Potri.013G128500.1	AT3G59770.1
Potra2n13c25182.1	-0.495755084	-0.045045543	0.000344771	0.959889842	Potri.013G128300.5	AT3G59780.1
Potra2n13c25183.1	-0.24483094	-0.13672649	0.201001427	0.926808461	Potri.013G128200.1	AT4G34620.1
Potra2n13c25184.2	0.285910934	0.065559705	0.003277642	0.924256651	Potri.013G128100.1	AT5G49960.1
Potra2n13c25185.1	-0.68854982	-0.563697302	0.011777045	0.588358083	Potri.013G127900.1	AT5G16250.1
Potra2n13c25186.2	0.398500041	0.309067133	0.00034608	0.220736763	Potri.013G127800.1	AT2G43750.1
Potra2n13c25187.1	NA	2.676364087	NA	NA	Potri.013G127700.1	AT3G59710.1
Potra2n13c25188.1	0.180198966	-0.132844279	0.405670289	0.896089262	Potri.001G337200.1	AT5G22660.2
Potra2n13c25189.1	0.063612612	-0.124556406	0.733802386	0.876739056	Potri.013G127500.1	AT1G18480.1
Potra2n13c25190.1	-0.124617249	-0.25641192	0.655175107	0.842247476	Potri.013G127400.1	AT2G31725.1
Potra2n13c25191.2	0.488266779	-0.067595357	0.304029015	0.986088609		
Potra2n13c25192.1	-0.361290304	-0.129493636	0.009740194	0.900551258	Potri.013G127300.4	AT2G43710.1
Potra2n13c25193.1	-0.746026072	-0.301331585	3.48913E-05	0.819475509	Potri.013G127200.1	AT3G59690.1
Potra2n13c25194.1	1.083156625	0.233885839	1.61699E-05	0.922681078	Potri.013G127100.2	AT3G59670.1
Potra2n13c25195.1	0.334068686	0.14726918	0.005806683	0.82876584	Potri.013G127000.1	AT3G59660.1
Potra2n13c25196.1	0.348541821	0.079308507	0.055762119	0.948577865	Potri.013G126900.1	AT1G05720.1
Potra2n13c25197.1	0.806450726	0.240202228	0.006415627	0.816567218	Potri.013G126800.2	AT1G05710.4
Potra2n13c25198.1	0.676970569	0.164085803	0.031976353	0.909675463		
Potra2n13c25199.1	0.383780574	-0.30019983	0.37307673	0.903698951	Potri.013G126701.1	AT5G60030.1

Potra2n13c25200.2	-1.405019096	-0.573624309	7.41703E-06	0.666052699	Potri.013G126600.2	AT2G02960.3
Potra2n13c25201.1	-2.016814622	-0.680350819	0.025193111	0.838684028	Potri.013G126500.1	
Potra2n13c25202.1	-0.870724437	-1.156027553	0.588575358	NA	Potri.013G126400.1	AT2G26710.1
Potra2n13c25203.1	0.140826065	0.170682798	0.186840469	0.814981587	Potri.013G126300.1	AT1G73180.1
Potra2n13c25204.1	-0.075780018	-0.079894207	0.647571044	0.961820855	Potri.013G126200.1	AT3G59650.1
Potra2n13c25205.1	0.82357019	NA	NA	NA	Potri.013G126100.1	AT5G45890.1
Potra2n13c25206.1	-0.157491957	-0.049869798	0.276969295	0.95981322	Potri.013G126000.1	
Potra2n13c25207.1	-0.104284827	-0.049262761	0.439368164	0.953608209	Potri.013G125800.1	AT2G43650.1
Potra2n13c25208.1	-0.677236746	-0.229140881	5.6901E-05	0.813575895	Potri.013G125700.1	AT3G52050.3
Potra2n13c25209.1	-1.45286665	-0.354706934	0.065688434	0.925582271		
Potra2n13c25210.1	-0.154225672	-0.073160387	0.517343188	0.953831244	Potri.013G125600.1	AT2G30695.1
Potra2n13c25211.2	-0.314939418	-0.080053235	0.110581732	0.953608209	Potri.013G125500.1	AT2G30695.1
Potra2n13c25212.1	7.164744522	4.884181748	5.5265E-99	3.06238E-11	Potri.013G125300.1	AT3G25180.1
Potra2n13c25213.1	0.120455712	-0.103944872	0.471556652	0.895560236	Potri.013G125200.1	AT2G43640.1
Potra2n13c25214.1	NA	NA	NA	NA	Potri.013G125100.1	AT3G54420.1
Potra2n13c25215.1	0.812207797	0.059961112	0.008934468	0.973347974	Potri.013G125000.1	AT3G54420.1
Potra2n13c25216.1	0.081038812	-0.517238527	0.918354372	0.853030738	Potri.013G124900.2	AT5G09640.1
Potra2n13c25217.1	0.82357019	NA	NA	NA	Potri.013G124600.1	AT1G60500.1
Potra2n13c25218.1	NA	NA	NA	NA	Potri.013G124500.1	AT5G11180.1
Potra2n13c25219.1	-2.120173875	-0.525658721	0.036937464	0.918311446	Potri.013G124400.1	AT3G58120.1
Potra2n13c25220.1	1.164772457	0.280436703	0.561096259	NA		
Potra2n13c25221.1	0.104157615	-0.081861482	0.882419352	0.986636967	Potri.013G124200.1	AT1G06260.1
Potra2n13c25222.1	-0.310123648	-0.118420827	0.30177599	0.943483421	Potri.013G124100.1	AT1G06270.1
Potra2n13c25223.2	0.087993535	0.0538837	0.569763252	0.96059667	Potri.013G124050.1	AT1G14300.1
Potra2n13c25224.1	0.852757295	0.101514598	0.006196872	0.956865545	Potri.013G124000.1	AT2G42390.1
Potra2n13c25225.1	NA	1.571529889	NA	NA	Potri.013G123900.1	AT1G06280.1
Potra2n13c25226.1	-0.757410585	-0.297639394	3.04857E-06	0.605140419	Potri.013G123800.1	AT4G35020.2
Potra2n13c25227.3	0.156991774	0.054458582	0.431236392	0.970011758	Potri.013G123700.1	AT2G31060.2
Potra2n13c25228.1	-0.549700235	-0.071135446	0.004498915	0.970930327	Potri.013G123600.2	AT5G60860.1
Potra2n13c25229.1	0.308505188	0.00209026	0.12060724	0.999325699	Potri.013G123500.1	AT1G06290.1
Potra2n13c25230.1	-0.22472568	-0.087326806	0.26418413	0.940703488	Potri.013G123100.1	AT1G28520.2
Potra2n13c25231.1	-0.080277623	-1.244840521	0.970664812	NA	Potri.013G121900.1	AT4G35160.1

Potra2n13c25232.1	1.232859617	0.694570896	0.032080781	0.805461106	Potri.013G149600.1	AT1G27410.1
Potra2n13c25233.1	1.585443773	1.26088106	0.131454419	0.6472326	Potri.013G149600.1	AT1G27410.1
Potra2n13c25234.1	-0.322367589	0.2821813	0.581448911	0.903769998	Potri.013G123000.1	AT1G28510.1
Potra2n13c25235.1	-1.343512567	-1.492571599	NA	NA	Potri.013G120800.1	AT4G35160.1
Potra2n13c25236.1	1.01601933	0.415382641	5.38136E-06	0.672348168	Potri.013G149600.1	AT1G27410.1
Potra2n13c25237.1	1.517671607	0.674963689	NA	NA		
Potra2n13c25238.3	0.181574796	0.312678554	0.669963758	0.88643982	Potri.013G123000.1	AT1G28510.1
Potra2n13c25239.1	1.594379011	0.64247635	NA	NA	Potri.013G122400.1	AT4G35160.1
Potra2n13c25240.1	NA	NA	NA	NA	Potri.013G122900.1	AT4G35150.1
Potra2n13c25241.1	1.147075632	0.862675244	NA	NA	Potri.013G122400.1	AT4G35160.1
Potra2n13c25242.1	1.184768565	0.522660005	0.062144696	0.835819291	Potri.013G121400.1	AT4G35160.1
Potra2n13c25243.1	-1.666818231	0.214341678	0.206697642	0.982827151	Potri.013G121300.1	AT4G35160.1
Potra2n13c25244.1	1.28973754	-0.273012958	0.009770124	0.939644353	Potri.013G121400.1	AT4G35160.1
Potra2n13c25245.1	2.58005889	1.189493042	4.21114E-09	0.362823288	Potri.013G121000.2	AT2G19130.1
Potra2n13c25246.1	NA	NA	NA	NA	Potri.013G120800.1	AT4G35160.1
Potra2n13c25247.1	-0.346867227	-0.154114903	0.020636714	0.897285619		
Potra2n13c25248.1	-0.96258944	-1.267485234	0.202089802	0.534094881	Potri.013G120600.1	AT2G20520.1
Potra2n13c25249.1	-0.01459766	-0.080110898	0.930203504	0.923746681	Potri.013G120500.1	AT3G59600.1
Potra2n13c25250.1	NA	NA	NA	NA		
Potra2n13c25251.1	1.459962178	1.363811008	NA	NA	Potri.013G120400.1	AT3G59600.1
Potra2n13c25252.1	0.200858605	-0.18590248	0.453087718	0.910815461	Potri.013G120300.13	AT3G53690.1
Potra2n13c25253.1	0.82545086	0.112917896	4.92263E-06	0.956292878	Potri.013G124900.2	AT5G09640.1
Potra2n13c25254.1	-1.147968017	0.370996155	0.589033706	0.953831244	Potri.013G119900.1	AT1G60500.1
Potra2n13c25255.1	0.82357019	NA	NA	NA	Potri.013G119800.1	AT5G04530.1
Potra2n13c25256.1	-0.241792369	-0.676485116	0.87014555	0.900551258		
Potra2n13c25257.1	-0.954668409	-0.600429697	0.549791409	0.945454119	Potri.003G140900.1	AT4G10960.1
Potra2n13c25258.1	0.491203558	-0.715155849	0.76469946	NA		
Potra2n13c25259.1	0.172119034	0.096058586	0.164211706	0.906545272	Potri.013G119700.1	AT3G58110.2
Potra2n13c25260.1	0.042865153	0.069336434	0.878008087	0.963878456		
Potra2n13c25261.1	1.400354191	-1.101661743	0.178250013	0.88066693	Potri.013G119600.1	AT5G04530.1
Potra2n13c25262.1	-0.591525666	-0.347070186	0.210309309	0.870628567	Potri.013G119500.2	AT3G58100.1
Potra2n13c25263.1	-1.240225192	-0.379946336	2.0897E-06	0.857418365		

Potra2n13c25264.1	1.001156385	-0.10708464	0.733558469	NA	Potri.013G119300.1	
Potra2n13c25265.1	NA	1.37928045	NA	NA		
Potra2n13c25266.1	NA	NA	NA	NA		
Potra2n13c25267.1					Potri.019G026600.1	
Potra2n13c25268.1	-2.674726205	0.599753635	NA	NA	Potri.013G119100.1	AT2G31081.1
Potra2n13c25269.1	NA	NA	NA	NA	Potri.013G119000.1	
Potra2n13c25270.1	0.92641307	0.660619236	NA	NA	Potri.013G119000.1	
Potra2n13c25271.1	-0.906975177	0.116248715	0.150266341	0.985393296	Potri.013G117200.1	AT1G71830.1
Potra2n13c25272.1	-0.429918915	-0.015491708	6.99796E-05	0.988354153	Potri.013G118900.1	AT1G06230.2
Potra2n13c25273.1	-2.316346109	-0.873002103	NA	NA		
Potra2n13c25274.1	-0.191024435	-0.012413594	0.546275926	0.996032145		
Potra2n13c25275.1	-0.111277396	-0.060187389	0.767311744	0.979431938		
Potra2n13c25276.1	-0.3108599	0.022892038	0.316846265	0.989329144		
Potra2n13c25277.1	-1.87597783	-0.022424032	0.121998898	0.997821888	Potri.013G118700.1	AT5G17050.1
Potra2n13c25278.1	1.030141409	-0.02595295	0.001138367	0.995579852	Potri.013G118500.1	AT2G42570.1
Potra2n13c25279.2	1.64774763	NA	NA	NA	Potri.013G118200.1	AT5G45890.1
Potra2n13c25280.1	-0.419982387	-2.254868371	0.786017637	NA	Potri.019G090000.1	AT3G12060.1
Potra2n13c25281.4	-0.597311189	-0.211604037	2.26999E-06	0.775618506	Potri.013G118000.2	AT2G31130.1
Potra2n13c25282.1	0.82357019	NA	NA	NA	Potri.019G089700.1	AT3G18000.1
Potra2n13c25283.1						
Potra2n13c25284.1	-0.61910233	-0.767708831	NA	NA	Potri.013G117700.1	AT1G53270.1
Potra2n13c25285.1	-2.959848622	0.040194412	0.0860578	0.996032145	Potri.013G117600.1	AT3G56770.1
Potra2n13c25286.1	NA	NA	NA	NA	Potri.011G052700.1	AT3G58610.3
Potra2n13c25287.1	0.130581943	-0.025839326	0.399465859	0.985393296	Potri.013G117400.1	AT1G71820.1
Potra2n13c25288.1	1.459962178	NA	NA	NA	Potri.013G117300.1	
Potra2n13c25289.1	-0.579866369	0.118786344	0.006625619	0.940703488	Potri.013G117200.1	AT1G71830.1
Potra2n13c25290.1	-0.208817473	0.053156201	0.07548175	0.930598202	Potri.013G117100.4	AT1G34220.2
Potra2n13c25291.2	0.586378067	0.172817912	0.000597789	0.820074794	Potri.013G116900.1	AT1G71840.1
Potra2n13c25292.1	-4.400354995	-2.0029097	0.003300921	0.442733499	Potri.013G117000.1	AT4G09650.1
Potra2n13c25293.1	0.470049004	-0.050813112	0.000168862	0.976139323	Potri.013G116800.1	AT4G12600.1
Potra2n13c25294.1	-0.167438843	-0.086430896	0.42086527	0.95671968	Potri.013G116700.1	AT2G44020.1
Potra2n13c25295.1	3.636265227	NA	0.41570728	NA	Potri.013G116600.1	AT1G34245.1

Potra2n13c25296.2	-0.363449427	-0.09860476	6.13791E-05	0.890718125	Potri.013G116400.1	AT1G34260.1
Potra2n13c25297.1	0.097377571	-0.078405664	0.655265695	0.948577865	Potri.013G116200.1	AT1G34270.1
Potra2n13c25298.1	0.665767418	0.12134239	0.371546234	0.979755286		
Potra2n13c25299.1	0.352241624	0.135195041	0.199355256	0.938967511		
Potra2n13c25300.1					Potri.013G116000.1	AT4G32600.1
Potra2n13c25301.1						
Potra2n13c25302.1					Potri.013G116000.1	AT4G32600.1
Potra2n13c25303.1						
Potra2n13c25304.1					Potri.013G116000.1	AT4G32600.1
Potra2n13c25305.1					Potri.013G116000.1	AT4G32600.1
Potra2n13c25306.1					Potri.013G116000.1	AT4G32600.1
Potra2n13c25307.1					Potri.013G116000.1	AT4G32600.1
Potra2n13c25308.1					Potri.013G116000.1	AT4G32600.1
Potra2n13c25309.1	3.525541794	1.986336619	0.040008692	NA	Potri.013G115900.1	AT1G29670.1
Potra2n13c25310.1	2.823935421	-0.141508297	2.63327E-05	0.987172498	Potri.013G115800.1	AT1G34300.1
Potra2n13c25311.3	1.524291899	0.37986166	0.000915471	0.912083068	Potri.013G115800.1	AT1G34300.1
Potra2n13c25312.1	0.661759159	0.152051023	0.000109154	0.88643982		
Potra2n13c25313.1	-2.712503671	-0.504826348	7.17487E-06	0.843529893	Potri.013G115600.1	AT1G71870.1
Potra2n13c25314.1	1.41619508	0.760788916	2.5405E-06	0.606364174	Potri.013G115500.1	AT1G71850.1
Potra2n13c25315.1	0.469887449	0.079396467	7.69899E-05	0.954559968	Potri.013G115400.1	AT1G71860.1
Potra2n13c25316.1	0.287228393	0.142365543	0.003120981	0.849258845	Potri.013G115300.2	AT1G34320.1
Potra2n13c25317.2	0.927798033	-1.005280469	0.847488983	NA	Potri.013G115200.1	AT1G22710.1
Potra2n13c25318.1	1.005427503	0.235827916	5.28472E-08	0.88661378	Potri.013G115000.1	AT1G34340.1
Potra2n13c25319.1	0.97605246	-9.33167E-05	0.00046985	0.999937671	Potri.013G114900.1	AT1G50420.1
Potra2n13c25320.1	0.131345361	0.044915607	0.542565112	0.95946978	Potri.013G114800.1	AT1G34350.1
Potra2n13c25321.2	0.042442616	0.03334336	0.800770212	0.98177802	Potri.013G114700.1	AT1G34355.1
Potra2n13c25322.1	0.335494099	0.138729513	0.03517963	0.912295985	Potri.013G114600.1	AT1G34370.2
Potra2n13c25323.2	-0.265951248	0.161384539	0.227479633	0.779077802	Potri.013G114500.1	AT1G22700.1
Potra2n13c25324.1	0.055342002	0.212075846	0.77521002	0.736000077	Potri.013G114400.6	AT1G34380.2
Potra2n13c25325.1	-0.03527111	-0.06471148	0.853482638	0.943438281	Potri.013G114300.1	AT1G34430.1
Potra2n13c25326.1	3.156859767	1.480119829	5.40578E-44	0.011950008	Potri.013G114200.1	AT1G34420.1
Potra2n13c25327.1	-0.15522646	-0.14463261	0.397296572	0.890446513	Potri.013G114100.1	AT1G71900.1

Potra2n13c25328.1	-0.549566104	-0.399181331	0.055344027	0.776094736	Potri.013G114000.1	
Potra2n13c25329.1	0.24038955	-0.069801438	0.139127538	0.941855873	Potri.013G113900.1	AT5G10330.2
Potra2n13c25330.1	-0.370049356	-2.058800192	NA	NA	Potri.013G113800.1	AT1G77855.1
Potra2n13c25331.1	-0.474093118	0.016858035	0.000350948	0.989300076	Potri.013G113700.1	AT1G34550.1
Potra2n13c25332.1	NA	NA	NA	NA	Potri.016G130300.1	AT5G52605.1
Potra2n13c25333.1	-0.153256465	-0.102062445	0.357885813	0.930598202	Potri.013G113500.1	AT4G09620.1
Potra2n13c25334.1	2.735991243	NA	NA	NA	Potri.013G113400.2	AT2G18420.1
Potra2n13c25335.1						
Potra2n13c25336.3	-0.098320774	0.231680467	0.679459292	0.813076433	Potri.013G113200.1	AT1G65280.1
Potra2n13c25337.1	0.249130826	-1.049232944	0.699761557	0.660636854	Potri.013G113100.1	AT1G71930.1
Potra2n13c25338.1	-0.015633606	-0.024462835	0.928561475	0.986636967	Potri.013G113000.1	AT1G71940.1
Potra2n13c25339.1	-0.6210892	0.021059556	0.534989736	0.998245864	Potri.013G112900.1	AT1G35680.1
Potra2n13c25340.1	-0.002021481	0.06721239	0.991169783	0.937276488	Potri.013G112800.2	AT1G71950.1
Potra2n13c25341.1	0.876643002	0.817667172	0.166256399	0.869327705	Potri.008G211200.1	AT4G16070.1
Potra2n13c25342.1	0.188895825	0.197057657	0.363719904	0.724604666	Potri.013G112500.4	AT1G35670.1
Potra2n13c25343.1	-1.359810209	-0.540491698	3.48129E-14	0.12687792	Potri.013G112300.1	AT1G68300.1
Potra2n13c25344.1	NA	NA	NA	NA	Potri.013G112200.1	AT5G47430.3
Potra2n13c25345.1	NA	NA	NA	NA		
Potra2n13c25346.1	-0.28559232	-2.542948491	NA	NA	Potri.013G112100.1	AT5G17540.1
Potra2n13c25347.1	2.632524198	2.86450839	NA	NA	Potri.013G112100.1	AT5G17540.1
Potra2n13c25348.1	-0.70537887	-0.138996166	0.000607474	0.849815276	Potri.013G111900.1	AT1G71960.1
Potra2n13c25349.1	0.087914358	-0.475175521	0.830489344	0.306363984	Potri.013G111700.2	AT1G22680.1
Potra2n13c25350.1	-1.371268264	-1.519215768	NA	NA		
Potra2n13c25351.2	-0.767534008	-1.311309528	0.1232018	0.360181553		
Potra2n13c25352.1	-0.136778455	0.041302186	0.549001495	0.981772014	Potri.013G111500.2	AT1G71980.1
Potra2n13c25353.1	0.013035193	-0.014179947	0.949015624	0.989878071	Potri.013G111400.1	AT1G35620.1
Potra2n13c25354.1	0.152506651	0.018692295	0.239884036	0.987226805	Potri.013G111300.1	AT1G22660.1
Potra2n13c25355.1	-0.008998513	0.099547357	0.984308674	0.953108412	Potri.013G111200.1	AT1G71990.1
Potra2n13c25356.1	-2.375978331	-1.093480325	NA	NA	Potri.008G202600.3	AT5G19070.1
Potra2n13c25357.1	1.664677056	NA	NA	NA	Potri.013G111000.1	AT4G09530.1
Potra2n13c25358.1	0.82357019	NA	NA	NA		
Potra2n13c25359.4	0.227879038	0.158324798	0.234028987	0.925184207	Potri.013G110800.1	AT4G09510.1

Potra2n13c25360.1	0.224239238	0.367654923	0.308301878	0.720488481	Potri.013G110700.1	AT1G35560.1
Potra2n13c25361.1	0.182415476	1.251042414	0.860246372	0.552944332		
Potra2n13c25362.2	0.55418319	0.215468708	0.00045056	0.794641467	Potri.013G110600.2	AT1G72020.1
Potra2n13c25363.1	1.459962178	NA	NA	NA	Potri.007G128300.1	AT3G59410.1
Potra2n13c25364.1	NA	NA	NA	NA	Potri.007G128300.1	AT3G59410.1
Potra2n13c25365.1	0.16345918	0.169991735	0.490768397	0.870889425	Potri.013G109600.1	AT1G35530.2
Potra2n13c25366.1	0.219622944	-0.085480312	0.145761036	0.956865545	Potri.013G109500.1	AT5G56760.1
Potra2n13c25367.1	0.628916414	-0.113360254	0.071261626	0.974091403	Potri.013G109400.1	AT1G72040.1
Potra2n13c25368.1	0.265978051	0.660653671	0.447626891	NA	Potri.013G109300.1	AT1G22640.1
Potra2n13c25369.1	1.468269227	0.541929627	2.15482E-06	0.788925071	Potri.013G109100.1	AT1G14060.1
Potra2n13c25370.1	-0.631984997	-0.019587929	0.218480733	0.995203254	Potri.013G109000.1	AT4G09350.1
Potra2n13c25371.1	1.099179502	0.90685089	0.007494304	0.576541514	Potri.013G108900.1	AT1G75240.1
Potra2n13c25372.1	-1.587646083	0.362756179	NA	NA	Potri.004G187400.2	AT1G07400.1
Potra2n13c25373.1	-0.104674881	0.005916321	0.603483475	0.996154416	Potri.013G108700.1	AT3G20230.1
Potra2n13c25374.1	1.521448873	0.356748729	0.112666069	NA		
Potra2n13c25375.2	-0.142124165	-0.120902627	0.464086275	0.910815461	Potri.013G108600.1	AT1G72050.2
Potra2n13c25376.1	NA	1.363811008	NA	NA	Potri.013G108500.1	AT2G16920.1
Potra2n13c25377.1	-0.650649728	-0.214499253	0.00413826	0.905274942	Potri.013G108400.2	AT1G35470.2
Potra2n13c25378.1	-1.570989645	-0.135173953	NA	NA	Potri.013G108300.1	AT4G23710.1
Potra2n13c25379.1	-1.686862489	-0.434736769	0.000228088	0.829862776	Potri.013G108200.1	AT5G14870.1
Potra2n13c25380.4	-1.870420754	-0.330021187	9.50026E-06	0.871720501	Potri.013G108100.1	AT1G22630.1
Potra2n13c25381.1	0.26068268	0.158975153	0.02900641	0.755774501	Potri.019G080300.2	AT1G22620.1
Potra2n13c25382.1	-0.291608354	-0.163450543	0.029307811	0.799049555	Potri.013G107800.1	AT1G72090.1
Potra2n13c25383.1	-0.328829196	-0.172626765	0.02211358	0.799294261	Potri.013G107700.1	AT1G22610.1
Potra2n13c25384.1	-2.197417251	-0.178034527	0.098386163	0.974275337	Potri.013G107600.1	AT1G22590.2
Potra2n13c25385.1	-0.710575432	0.245867592	0.00143664	0.824905557	Potri.013G107500.1	AT1G35460.1
Potra2n13c25386.1	-0.275350998	-0.163973284	0.12687197	0.814981587	Potri.013G107300.2	AT1G35430.1
Potra2n13c25387.1	-0.034666044	0.015942489	0.85554311	0.990927357	Potri.013G107100.1	AT3G16510.1
Potra2n13c25388.1	-0.012591078	-0.091272613	0.90175358	0.909675463	Potri.013G106600.1	AT1G22540.1
Potra2n13c25389.1	3.155373554	-0.108918438	7.80298E-12	0.987226805	Potri.013G106200.1	AT5G36110.1
Potra2n13c25390.1	-0.113835501	0.065385642	0.901640877	0.988233879	Potri.013G106100.1	AT5G36110.1
Potra2n13c25391.1	-0.19184952	-0.265767188	0.520603691	0.669027738	Potri.013G106000.1	AT4G09160.1

Potra2n13c25392.1	-0.012821399	-0.123502595	0.938321852	0.84801014	Potri.013G105900.1	AT1G72160.1
Potra2n13c25393.1	0.243349311	0.077742573	0.044402136	0.953608209	Potri.013G105766.1	AT1G22520.1
Potra2n13c25394.1	-0.80124203	-0.330310444	0.537008393	0.962120332	Potri.019G079000.1	AT1G35420.2
Potra2n13c25395.1	-0.566969546	-0.270632118	0.000513121	0.682137097	Potri.013G105500.1	AT1G35340.1
Potra2n13c25396.1	-0.188917001	0.490703602	0.898274352	0.936549944		
Potra2n13c25397.1	-0.418703089	-0.141976989	0.000872251	0.832270846	Potri.013G105400.1	AT1G72175.1
Potra2n13c25398.1	-0.22128188	-0.075323334	0.13675763	0.936549944	Potri.013G105300.1	AT4G09150.2
Potra2n13c25399.1	0.376788276	0.173901296	0.009182238	0.855754617	Potri.013G105200.2	AT3G58140.1
Potra2n13c25400.2	0.026790378	-0.171949808	0.936339755	0.87679119	Potri.013G105100.1	AT1G77410.1
Potra2n13c25401.1	0.027871613	-0.168619094	0.933427894	0.879788771	Potri.013G105000.1	AT1G51965.1
Potra2n13c25402.1	2.249249553	3.821936724	NA	NA	Potri.013G104900.1	AT4G09960.1
Potra2n13c25403.2	-0.047164744	-0.000257729	0.686916743	0.999937671	Potri.013G104800.1	AT1G33970.2
Potra2n13c25404.2	-0.428845776	-0.318247605	0.000477454	0.361949049	Potri.013G104700.1	AT4G09900.1
Potra2n13c25405.1	-1.409983009	0.797829514	NA	NA	Potri.013G104500.1	AT1G33980.2
Potra2n13c25406.1	-0.648082884	-0.514947806	0.004282363	0.357747948		
Potra2n13c25407.1	-1.137247535	-0.419357953	1.42478E-06	0.378057299	Potri.013G104400.1	AT1G58350.2
Potra2n13c25408.1	-0.975361626	-0.438118984	0.032302548	0.849446235		
Potra2n13c25409.1	4.084108279	4.055602075	0.049702749	NA	Potri.009G001800.1	AT2G01050.1
Potra2n13c25410.1	-0.250475723	-0.174038636	0.020181611	0.740929862	Potri.013G104300.2	AT1G67480.1
Potra2n13c25411.1	0.757117369	0.171716767	1.48898E-05	0.917516356	Potri.013G104200.6	AT1G72190.1
Potra2n13c25412.2	1.563284578	0.621357178	7.04394E-26	0.226847819	Potri.013G104100.2	AT5G10340.1
Potra2n13c25413.1	3.36811362	3.497054526	NA	NA	Potri.013G103800.1	AT1G75280.1
Potra2n13c25414.1	2.492428534	NA	NA	NA	Potri.013G103700.2	AT4G39230.1
Potra2n13c25415.1	-0.139723839	0.299019817	0.740396594	0.893135869	Potri.013G103600.1	AT1G08070.1
Potra2n13c25416.1	2.476290371	0.674963689	NA	NA	Potri.013G103551.1	AT1G10010.1
Potra2n13c25417.1	0.128806346	0.216357651	0.387102318	0.829862776	Potri.013G103400.2	AT5G47840.1
Potra2n13c25418.1	7.80686874	3.860295133	1.02577E-08	NA	Potri.013G103300.1	AT5G47850.1
Potra2n13c25419.1					Potri.019G078400.1	AT1G72180.1
Potra2n13c25420.1	1.046281831	0.374282724	0.088146315	0.910815461	Potri.013G103000.1	AT1G76690.1
Potra2n13c25421.1	0.713617329	0.685260734	0.041911664	0.643065116	Potri.013G103000.1	AT1G76690.1
Potra2n13c25422.4	1.400698164	0.711711543	7.92392E-08	0.689158687	Potri.013G102700.1	AT1G76690.1
Potra2n13c25423.1	1.864921895	2.636205833	0.524621981	NA	Potri.013G102600.1	AT1G71692.1

Potra2n13c25424.1	0.549632363	-1.828058479	0.811933215	NA	Potri.013G102500.1	AT1G71691.2
Potra2n13c25425.1	1.459962178	NA	NA	NA	Potri.013G102400.1	AT1G33811.1
Potra2n13c25426.1	1.464719402	0.49520542	6.72506E-06	0.606364174	Potri.013G102300.2	AT4G09970.1
Potra2n13c25427.1	-0.915533486	-0.530926324	1.21063E-05	0.597141087	Potri.013G102200.1	AT1G33800.1
Potra2n13c25428.1	-0.88488974	-0.516128249	3.92723E-05	0.635323636	Potri.003G003800.1	
Potra2n13c25429.1	-0.874967127	-0.507068801	6.68673E-05	0.667562773	Potri.013G102200.1	AT1G33800.1
Potra2n13c25430.1	-0.213223652	-0.049157052	0.208191382	0.97137556	Potri.013G102000.1	AT4G10000.1
Potra2n13c25431.1	0.500673257	0.143041862	0.000269841	0.898853929	Potri.013G101700.2	AT1G22800.1
Potra2n13c25432.1	0.403811134	-0.007911903	0.023563016	0.996154416	Potri.013G101601.1	AT4G10040.1
Potra2n13c25433.1	0.82357019	NA	NA	NA		
Potra2n13c25434.1	2.20732395	1.15717706	0.418635394	NA	Potri.013G101300.2	AT1G24160.1
Potra2n13c25435.1	1.918538301	0.436954798	0.314552256	NA	Potri.013G101200.2	AT1G33760.1
Potra2n13c25436.1	2.757247056	1.281157124	1.69312E-05	0.273841527	Potri.013G101100.1	AT1G71450.1
Potra2n13c25437.1	2.011034025	2.125377829	0.329222341	0.791002527	Potri.013G101000.1	AT4G24340.1
Potra2n13c25438.1	-3.062337229	2.408260317	NA	0.788065047	Potri.013G100800.2	AT4G24340.1
Potra2n13c25439.1	-3.099944324	2.255800291	NA	0.802274905	Potri.013G100800.2	AT4G24340.1
Potra2n13c25440.1	-3.45320591	2.214041207	NA	0.814981587	Potri.013G100800.2	AT4G24340.1
Potra2n13c25441.1						
Potra2n13c25442.1	2.0450667	-0.339525034	0.041884763	NA	Potri.013G100500.1	AT4G32480.1
Potra2n13c25443.1					Potri.013G100400.1	AT1G59970.1
Potra2n13c25444.1	NA	NA	NA	NA		
Potra2n13c25445.1	0.829052573	-0.358494744	NA	0.936549944	Potri.013G100300.1	AT1G22810.1
Potra2n13c25446.1	-2.318158601	-1.398338193	0.020641093	0.511337291	Potri.013G100200.1	AT4G10020.1
Potra2n13c25447.1	-2.86015326	-3.00964539	NA	NA	Potri.019G073100.1	AT1G71530.1
Potra2n13c25448.1	0.012503148	0.132261507	0.953008093	0.887678043	Potri.013G100000.1	AT1G33780.1
Potra2n13c25449.1	-0.728805054	-0.039385434	3.87006E-05	0.9761362	Potri.013G099900.1	AT4G10050.1
Potra2n13c25450.1	NA	NA	NA	NA	Potri.006G097400.1	AT1G23710.1
Potra2n13c25451.3	-0.105585926	0.025845095	0.391775661	0.978251387	Potri.013G099800.1	AT4G10070.1
Potra2n13c25452.1	-0.07621673	0.134291948	0.453523675	0.842247476	Potri.013G099700.1	AT1G31480.1
Potra2n13c25453.1	-1.317292482	-1.465929376	NA	NA	Potri.007G102100.1	AT2G04160.1
Potra2n13c25454.1	-0.621506395	-0.154376244	4.20461E-05	0.802942736	Potri.013G099600.6	AT1G22850.1
Potra2n13c25455.1	-1.024520297	-0.704114848	0.002488171	0.367135531	Potri.013G099500.1	AT4G19710.2

Potra2n13c25456.1	NA	2.141662216	NA	NA		
Potra2n13c25457.1	0.002339599	0.586983589	0.995149271	0.753084543	Potri.013G103000.1	AT1G76690.1
Potra2n13c25458.1	7.159033651	1.363811008	3.80133E-08	NA	Potri.013G099400.1	AT1G07160.1
Potra2n13c25459.1	NA	NA	NA	NA		
Potra2n13c25460.1	NA	NA	NA	NA		
Potra2n13c25461.1						
Potra2n13c25462.1	-0.335970872	-0.079072098	0.026352871	0.941260209	Potri.013G099200.1	AT4G13530.1
Potra2n13c25463.1	-0.220800656	-0.611964704	0.499924925	0.585240923	Potri.013G099100.1	AT1G14060.1
Potra2n13c25464.1	2.177244015	0.449671401	1.88562E-08	0.805052871	Potri.013G098900.1	AT1G33590.1
Potra2n13c25465.1	2.18442874	0.48533355	5.11237E-08	0.792511865		
Potra2n13c25466.1	0.446061228	0.139466406	0.032854013	0.930598202	Potri.013G098800.1	AT1G71420.1
Potra2n13c25467.1	0.1311778	0.0084386	0.144417738	0.994119828	Potri.013G098700.1	AT1G22870.1
Potra2n13c25468.1					Potri.013G098400.1	AT3G10890.1
Potra2n13c25469.2	0.689748365	0.337074586	0.00193163	0.492144381	Potri.013G098000.1	AT5G17680.1
Potra2n13c25470.1	0.836549266	0.661006763	0.003500721	0.234967741	Potri.013G098550.1	AT5G17680.1
Potra2n13c25471.1	2.125312886	NA	NA	NA	Potri.004G082400.1	AT5G16560.1
Potra2n13c25472.1	-0.253636349	0.808207125	0.899946031	0.897331585	Potri.010G101200.1	AT2G01940.1
Potra2n13c25473.1	1.576009199	0.401841261	2.23136E-16	0.762561685	Potri.013G098550.1	AT5G17680.1
Potra2n13c25474.1	1.059916116	0.51324731	0.000698906	0.805461106		
Potra2n13c25475.1	0.068088595	0.14215954	0.745475708	0.874648591	Potri.019G068700.1	AT1G59610.1
Potra2n13c25476.1					Potri.014G188700.1	AT2G01050.1
Potra2n13c25477.1	NA	1.571529889	NA	NA	Potri.018G069000.1	AT2G01050.1
Potra2n13c25478.1	0.268563676	0.204547323	0.110639709	0.769631196	Potri.013G096600.1	AT1G71350.1
Potra2n13c25479.1	0.767247868	0.704497839	0.796395517	NA		
Potra2n13c25480.1	NA	NA	NA	NA		
Potra2n13c25481.1	0.945298599	-0.633723227	0.566463496	NA	Potri.013G096400.1	AT2G19130.1
Potra2n13c25482.1					Potri.013G093900.1	
Potra2n13c25483.1	0.708593929	-0.595346793	0.645923882	0.954559968	Potri.013G095800.1	AT2G19130.1
Potra2n13c25484.1	-2.74607172	-0.018532991	0.303912422	NA	Potri.013G096200.1	AT5G16340.1
Potra2n13c25485.1	0.444708916	-1.618019137	0.689312598	0.720242695	Potri.013G096400.1	AT2G19130.1
Potra2n13c25486.1	-1.317292482	1.288985234	NA	NA	Potri.013G096200.1	AT5G16340.1
Potra2n13c25487.5	-0.389005709	0.240089409	0.066590533	0.856660279	Potri.013G095500.1	AT4G10120.1

Potra2n13c25488.1	-0.227224733	0.418413212	0.400615231	0.772556533	Potri.013G095400.1	AT1G33500.1
Potra2n13c25489.1	-0.289138881	0.3975377	0.422720414	0.684244573	Potri.013G095400.1	AT1G33500.1
Potra2n13c25490.1	-0.036080313	0.006485711	0.853891539	0.996032145	Potri.013G095300.1	AT1G71340.1
Potra2n13c25491.1	-0.269409537	-0.033585844	0.174647843	0.985393296	Potri.013G095200.1	AT1G33490.1
Potra2n13c25492.1	-0.383410313	-0.106568904	0.040779761	0.930598202	Potri.013G095100.2	AT4G10150.1
Potra2n13c25493.1	-0.049861063	0.107535185	0.781731978	0.89321847	Potri.013G094900.1	AT4G10170.2
Potra2n13c25494.1	0.029316122	0.143104736	0.874138042	0.845014308	Potri.013G094800.1	AT5G24670.1
Potra2n13c25495.1	-0.06280661	-0.364195715	0.937839897	0.943483421	Potri.002G117000.1	AT2G45330.2
Potra2n13c25496.1	0.866155081	0.79580573	0.714025221	NA	Potri.013G094400.1	AT1G33470.2
Potra2n13c25497.1	0.786646035	0.038638605	2.32184E-06	0.981772014	Potri.013G093700.1	AT2G40510.1
Potra2n13c25498.2	0.359161652	0.109235282	0.386796804	0.971828097	Potri.013G093500.3	AT3G56330.1
Potra2n13c25499.1	5.019374363	NA	0.002839328	NA	Potri.T001500.1	AT5G36930.2
Potra2n13c25500.1	0.138885776	0.10817513	0.176596711	0.82876584	Potri.013G093400.1	AT1G18070.1
Potra2n13c25501.2	-2.312077755	-0.206364484	5.96182E-09	0.947969749	Potri.013G093200.1	
Potra2n13c25502.1	-0.763640641	-3.444126063	0.874237578	NA	Potri.013G093100.1	AT1G50870.1
Potra2n13c25503.1	-0.214190002	0.036833923	0.145947218	0.969821472	Potri.013G092900.1	AT3G10840.1
Potra2n13c25504.1	0.727056146	0.13722486	4.3042E-05	0.938967511	Potri.013G092700.2	AT2G40600.1
Potra2n13c25505.2	0.311036422	0.09906401	0.038968501	0.936890993	Potri.013G092600.1	AT3G10920.1
Potra2n13c25506.1	-1.998050469	-1.421887915	NA	NA	Potri.013G092400.1	AT4G10350.1
Potra2n13c25507.1	-0.284944792	0.029752427	0.012738624	0.974782554	Potri.019G066200.2	AT3G19770.1
Potra2n13c25508.1	-0.32326159	-0.035762751	0.012304316	0.966917036	Potri.013G092000.1	AT1G33420.1
Potra2n13c25509.1	-0.240306089	-0.090568763	0.365787336	0.972159498	Potri.013G091900.1	AT3G14172.1
Potra2n13c25510.1	-0.081858254	0.15939177	0.635523827	0.78974049	Potri.013G091700.1	AT3G54190.1
Potra2n13c25511.1	-2.017068792	-0.243561653	0.009981153	0.954098985	Potri.013G091300.1	AT3G10910.1
Potra2n13c25512.1	0.245170784	-0.024939653	NA	NA		
Potra2n13c25513.1	NA	NA	NA	NA	Potri.016G026000.4	AT2G01570.1
Potra2n13c25514.1	2.125481248	0.761677608	2.50139E-21	0.04665809	Potri.016G026000.4	AT2G01570.1
Potra2n13c25515.1	-1.760137916	-2.000107017	0.627880972	NA		
Potra2n13c25516.1	-1.348763187	-3.030544583	NA	NA	Potri.001G422600.1	AT3G56360.1
Potra2n13c25517.5	-0.325487461	-0.2755738	0.119340942	0.704663803	Potri.019G058200.1	AT5G05240.1
Potra2n13c25518.1	-1.338239179	-0.317982374	0.004133175	0.788065047	Potri.013G091000.1	AT3G56370.1
Potra2n13c25519.2	0.404501069	0.145780152	0.028072239	0.943819961	Potri.013G090900.1	AT2G40690.1

Potra2n13c25520.1	-0.685223985	-0.0002789	0.101440531	0.999937671	Potri.013G090800.2	AT3G01090.2
Potra2n13c25521.1	-0.020899532	0.156757614	0.965525849	0.943819961	Potri.013G090600.3	AT2G40720.1
Potra2n13c25522.1	-0.226759631	-0.065544045	0.067603448	0.953108412	Potri.013G090500.1	AT2G40730.1
Potra2n13c25523.1	5.947537409	2.86450839	0.002654774	NA	Potri.013G090400.1	AT2G40740.1
Potra2n13c25524.1	7.031649155	4.356966391	7.14542E-12	NA	Potri.013G090300.1	AT3G56400.1
Potra2n13c25525.1	6.758602142	1.871975723	1.75143E-07	NA	Potri.013G090200.1	AT5G05220.1
Potra2n13c25526.1	-0.332806921	0.065559756	0.038688513	0.935474933	Potri.013G090000.3	AT3G26580.1
Potra2n13c25527.1	0.220101458	-0.087723864	0.143895642	0.930203195	Potri.013G089600.1	AT4G10300.1
Potra2n13c25528.1	-0.079005625	0.148854348	0.560351789	0.859882283	Potri.013G089500.5	AT1G33360.1
Potra2n13c25529.1	-0.455771763	-0.258433983	0.093822112	0.859813626	Potri.013G089400.1	AT1G33350.1
Potra2n13c25530.1	-1.515952518	-1.608802864	0.001857304	0.009694555	Potri.013G089200.2	AT4G10250.1
Potra2n13c25531.1	0.20132492	0.340532473	0.321241033	0.522715829	Potri.013G089000.1	AT3G14050.1
Potra2n13c25532.1	-0.127199111	-0.011548892	0.155285597	0.9921442	Potri.019G052200.1	AT5G17060.1
Potra2n13c25533.1	-0.230668553	0.072914089	0.107325266	0.943483421	Potri.013G088600.1	AT5G17020.1
Potra2n13c25534.1	0.170859888	0.117051263	0.076697222	0.933286951	Potri.013G088100.3	AT1G31800.1
Potra2n13c25535.1	0.387605233	0.45855762	0.044295547	0.562306981	Potri.013G087950.1	AT3G44370.1
Potra2n13c25536.1	0.046994188	0.123396303	0.699487746	0.865611082	Potri.013G087400.1	AT5G16520.1
Potra2n13c25537.2	0.030310833	0.136204337	0.789195218	0.854980698	Potri.013G087300.1	
Potra2n13c25538.1	-0.145185967	-0.011720542	0.419846232	0.99114586	Potri.013G087200.1	AT1G65380.1
Potra2n13c25539.1	-0.287542663	-0.048562166	0.060922733	0.964160823	Potri.013G087100.1	AT1G65320.1
Potra2n13c25540.2	-0.278965009	-0.044242739	0.104218449	0.964160823		
Potra2n13c25541.1	-0.828134719	2.968066177	NA	0.444948931	Potri.013G087000.1	AT5G16530.1
Potra2n13c25542.1	-0.318073816	0.075404971	0.021328666	0.940856252	Potri.013G086800.1	AT3G02830.1
Potra2n13c25543.1	-0.10697381	0.001095656	0.320678619	0.999458638	Potri.013G086700.1	AT5G36290.2
Potra2n13c25544.1	-0.468690075	-0.317345177	0.082385696	0.788065047	Potri.013G086600.2	AT5G16490.1
Potra2n13c25545.3	0.156383832	-0.188641407	0.569514642	0.880477186	Potri.013G086500.1	AT3G02800.1
Potra2n13c25546.2	-0.154487707	0.218981905	0.38352348	0.888366602	Potri.013G086400.1	AT3G02790.1
Potra2n13c25547.1	1.719777493	-0.487390589	0.078194452	0.95671968	Potri.013G086100.1	AT2G19130.1
Potra2n13c25548.1	-0.596961031	-0.252291296	0.011639066	0.821354851	Potri.013G086000.1	AT5G56270.1
Potra2n13c25549.1	NA	NA	NA	NA	Potri.013G085800.2	AT5G16460.1
Potra2n13c25550.1	-0.579743122	-0.300114239	0.009588789	0.71411664	Potri.013G085700.1	AT5G16450.2
Potra2n13c25551.1	0.018947492	0.059710141	0.891757192	0.930203195	Potri.013G085600.1	AT5G36230.1

Potra2n13c25552.4	-0.020194806	-0.052263618	0.864568228	0.94795983	Potri.013G085600.1	AT5G36230.1
Potra2n13c25553.1	2.340207562	NA	NA	NA		
Potra2n13c25554.1	1.459962178	NA	NA	NA		
Potra2n13c25555.1	-0.308651375	-0.020432579	2.36723E-05	0.98317667	Potri.013G085500.1	AT3G02750.1
Potra2n13c25556.2	-1.026991234	0.16799031	5.59496E-06	0.948002367	Potri.013G085300.1	AT1G65230.1
Potra2n13c25557.1	NA	NA	NA	NA	Potri.014G188600.1	AT2G01050.1
Potra2n13c25558.1	0.460824432	0.304796056	2.37066E-09	0.621894291	Potri.013G085000.1	AT1G24706.1
Potra2n13c25559.1	-0.375786721	-0.201065688	0.039304577	0.843683654	Potri.013G084900.1	AT2G47840.1
Potra2n13c25560.1	-0.062537435	-0.088316277	0.604868251	0.880553952	Potri.019G055000.1	AT3G03340.1
Potra2n13c25561.1	2.016811923	-0.532556097	0.18149084	NA	Potri.013G084600.1	
Potra2n13c25562.1	0.411857399	0.094842584	0.023486616	0.940556596	Potri.013G084500.1	AT1G65290.1
Potra2n13c25563.1	1.597614873	-1.478536539	NA	NA	Potri.013G084400.1	AT3G26120.1
Potra2n13c25564.1	-0.275927201	-0.052473156	0.038166414	0.951726178	Potri.013G084300.1	AT2G43945.1
Potra2n13c25565.1	-0.086575552	0.066505405	0.522142987	0.945454119	Potri.013G084100.2	AT3G03330.1
Potra2n13c25566.1	-0.61910233	-0.767708831	NA	NA	Potri.013G084000.1	AT2G30300.1
Potra2n13c25567.1	-1.003351732	-0.360095224	6.75429E-06	0.847341104	Potri.013G083800.2	AT3G56230.1
Potra2n13c25568.1	-0.574635645	0.158685372	0.012836591	0.937846405	Potri.013G083700.2	AT3G56250.1
Potra2n13c25569.1	7.107019445	2.510360669	1.81577E-12	0.686344651	Potri.013G083600.1	AT5G05340.1
Potra2n13c25570.1	4.50503792	2.522677944	8.30457E-44	0.014382816	Potri.013G083500.1	AT5G05300.1
Potra2n13c25571.1	-0.326376036	-0.157835356	0.02888923	0.901251375	Potri.013G083400.1	AT2G40540.2
Potra2n13c25572.1	-1.306751055	-0.452530639	2.57175E-13	0.79078676	Potri.013G083300.1	AT3G10960.1
Potra2n13c25573.1	1.545832463	NA	NA	NA	Potri.013G080866.1	AT2G40490.1
Potra2n13c25574.1					Potri.013G083101.1	AT2G40475.1
Potra2n13c25575.1	-0.926658527	0.017842895	3.26069E-05	0.99114586	Potri.013G081100.1	AT4G13070.1
Potra2n13c25576.1	-0.61910233	1.532399325	NA	NA	Potri.013G081200.1	AT2G40470.1
Potra2n13c25577.1	0.057855872	-0.011080575	0.568711092	0.991032223	Potri.013G081300.7	AT1G15750.2
Potra2n13c25578.1	0.133020556	0.09381374	0.2404754	0.886059841	Potri.013G081400.1	AT3G03140.1
Potra2n13c25579.1	1.34631885	-0.245347715	0.000449414	0.907073354	Potri.013G081600.2	AT3G03130.1
Potra2n13c25580.1	-0.088434096	0.217835094	0.751378326	0.874595634	Potri.013G081700.1	AT5G17165.1
Potra2n13c25581.1	0.124548498	0.174259932	0.341531809	0.787760802	Potri.013G081900.1	
Potra2n13c25582.1	0.213997869	0.052144561	0.320052193	0.974367223	Potri.013G082000.1	
Potra2n13c25583.1	0.116126226	-0.020331784	0.431538365	0.9869326	Potri.013G082100.1	AT3G03070.1

Potra2n13c25584.3	0.433303607	-0.082215907	0.029834309	0.953608209	Potri.013G082200.1	AT3G03050.1
Potra2n13c25585.1						
Potra2n13c25586.1	1.870906322	0.555644897	NA	0.513411699		
Potra2n13c25587.2	0.007717563	-0.123468463	0.96626883	0.923475785	Potri.013G082500.1	AT5G16880.2
Potra2n13c25588.1	0.07501211	0.114756379	0.615496311	0.924251575	Potri.019G050100.2	AT5G16870.1
Potra2n13c25589.1	3.887747155	0.64247635	0.164011643	NA	Potri.013G080500.2	AT4G24340.1
Potra2n13c25590.1	0.180013251	0.092902498	0.160717648	0.930871994	Potri.013G080200.1	AT5G16790.1
Potra2n13c25591.3	0.404104898	0.118979193	0.003377877	0.866676143	Potri.013G080100.1	AT3G02970.1
Potra2n13c25592.1	0.961114947	0.260635564	2.4518E-25	0.676711874	Potri.013G079800.1	AT5G16820.2
Potra2n13c25593.1	1.223303509	0.268099351	0.000329355	0.941115972	Potri.013G079700.1	AT3G10480.3
Potra2n13c25594.1	-0.00022213	-0.162162879	0.999038588	0.834088274	Potri.013G079600.2	AT5G16750.1
Potra2n13c25595.1	0.090834982	0.032854507	0.896155321	0.989329144	Potri.013G079500.1	AT2G23910.1
Potra2n13c25596.2	-0.32050813	-0.00434556	0.088953044	0.996999319	Potri.T153100.1	AT1G65020.1
Potra2n13c25597.3	0.038450595	0.129929514	0.789295787	0.858481608	Potri.T153000.1	AT1G65020.1
Potra2n13c25598.1	-0.473493691	0.016623372	0.007812811	0.991407187	Potri.013G079000.2	AT5G16730.1
Potra2n13c25599.1	0.186556458	0.171331138	0.324232407	0.84487051		
Potra2n13c25600.1	0.018474644	-0.106220447	0.906866499	0.917892599	Potri.019G047766.1	AT3G42050.1
Potra2n13c25601.1	-0.24966909	-0.091791625	0.106748962	0.936890993	Potri.013G078600.1	AT4G27600.1
Potra2n13c25602.1	0.138185022	0.233807128	0.449162985	0.817452127	Potri.013G078500.1	AT4G17900.1
Potra2n13c25603.1					Potri.013G078400.1	AT2G31720.1
Potra2n13c25604.1	-0.171516346	0.079251112	0.400630934	0.967229803	Potri.013G078300.1	AT5G62030.1
Potra2n13c25605.1	-0.238234261	0.117837588	0.278620754	0.91392603	Potri.013G078200.1	AT5G16650.1
Potra2n13c25606.1	0.230765045	0.003649499	0.102140599	0.996999319	Potri.013G078100.1	AT5G16660.1
Potra2n13c25607.1	0.157951745	0.23500592	0.143182066	0.591292872	Potri.013G077900.1	AT5G16680.1
Potra2n13c25608.1	-0.162049018	-0.114516882	0.237703545	0.912665563	Potri.013G077800.1	AT5G60570.1
Potra2n13c25609.1	-0.329053845	-0.02769294	0.013099474	0.983113084	Potri.013G077700.1	AT4G27630.2
Potra2n13c25610.1	-0.506296196	0.317182926	0.345171535	0.907032966	Potri.013G077500.1	AT3G13960.1
Potra2n13c25611.1	NA	NA	NA	NA		
Potra2n13c25612.1	1.11200252	0.160739849	0.00402268	0.953831244	Potri.013G077200.1	AT5G16690.1
Potra2n13c25613.1	0.399477057	0.213028049	0.046144301	0.803943952	Potri.013G077000.1	AT1G64970.1
Potra2n13c25614.1	-0.113249077	-0.138019137	0.370598291	0.850863374	Potri.013G076900.1	AT4G27610.2
Potra2n13c25615.1	0.110794189	-0.009130484	0.726816858	0.993589787	Potri.013G076800.1	AT5G17510.1

Potra2n13c25616.1	1.750669184	0.196403057	1.07057E-20	0.907073354	Potri.013G076600.1	AT1G64940.1
Potra2n13c25617.1	-0.597042199	-0.362641528	0.00322298	0.704224102	Potri.013G076500.1	AT1G64940.1
Potra2n13c25618.1	-0.269548401	-0.258667479	0.068179048	0.71411664	Potri.013G076400.1	AT1G09815.1
Potra2n13c25619.1	0.169995578	-0.015175931	0.570788021	0.995005375	Potri.013G076300.1	AT1G64890.1
Potra2n13c25620.1	0.149782873	-0.040293763	0.313258378	0.97521505	Potri.013G076200.1	AT1G64880.1
Potra2n13c25621.1	0.238328432	0.223205925	0.266995808	0.881624318	Potri.013G076100.1	AT5G17520.1
Potra2n13c25622.1	0.363980143	0.256535213	0.000530944	0.807983579	Potri.013G076000.1	AT1G17070.1
Potra2n13c25623.1						
Potra2n13c25624.1	-2.334378375	-1.641062365	0.484863927	NA	Potri.013G075600.1	AT2G27690.1
Potra2n13c25625.2	0.280739547	0.112201072	0.089034742	0.886281281	Potri.013G075500.1	AT2G44820.2
Potra2n13c25626.1	-0.903416675	-0.190746962	0.043211575	0.896353214	Potri.013G075400.1	AT1G64860.1
Potra2n13c25627.2	-0.042275154	0.024189101	0.77628232	0.985016122	Potri.013G075300.2	AT5G35980.1
Potra2n13c25628.1	3.006675986	1.409511062	0.095396553	NA	Potri.013G074400.1	AT5G17540.1
Potra2n13c25629.1	0.82357019	NA	NA	NA	Potri.013G074400.1	AT5G17540.1
Potra2n13c25630.1	0.390903336	-0.665725954	0.919447697	NA	Potri.013G074400.1	AT5G17540.1
Potra2n13c25631.1	NA	1.571529889	NA	NA	Potri.001G155750.1	AT1G21280.1
Potra2n13c25632.1	0.929193425	-1.101769153	0.760902895	NA	Potri.013G074400.1	AT5G17540.1
Potra2n13c25633.1	0.161831668	0.010701799	0.360383473	0.994591388	Potri.013G074100.2	AT5G17560.1
Potra2n13c25634.1	-2.439056823	-2.633566658	0.210806125	NA	Potri.013G074000.1	AT1G68765.1
Potra2n13c25635.1	-0.604014346	0.027479793	0.00031052	0.989406722	Potri.013G073900.2	AT5G35970.1
Potra2n13c25636.1	-0.40208493	0.044377001	0.001440975	0.95981322	Potri.013G073700.1	AT5G17580.1
Potra2n13c25637.1	-0.732458918	-0.254513065	0.013163153	0.924309478	Potri.013G073600.1	AT3G03530.1
Potra2n13c25638.1	-0.926364392	-0.482467979	1.55487E-05	0.646274123	Potri.013G073500.1	AT3G03550.1
Potra2n13c25639.2	0.691388904	0.182808475	5.14833E-05	0.859813626	Potri.013G073400.1	AT3G03560.1
Potra2n13c25640.1	2.878559998	0.140467306	2.73529E-28	0.959881296	Potri.013G073300.1	AT5G07990.1
Potra2n13c25641.1	2.805383572	0.08747579	6.01507E-26	0.978251387		
Potra2n13c25642.1	-0.067524289	0.020389833	0.508142039	0.98177802	Potri.013G073200.3	AT3G03570.1
Potra2n13c25643.2	0.24426674	0.137684369	0.128922637	0.890446513	Potri.013G073100.1	AT5G17610.1
Potra2n13c25644.1	-0.366491526	-0.674654022	0.324232407	0.520533674	Potri.013G072800.1	AT5G12080.2
Potra2n13c25645.1	-1.029107245	-0.357689362	2.95923E-08	0.32906562	Potri.013G072800.1	AT5G12080.2
Potra2n13c25646.1	-0.342528628	-0.028347549	0.011821508	0.973733734	Potri.013G072600.2	AT1G64850.1
Potra2n13c25647.1	-0.342528628	-0.028347549	0.011821508	0.973733734	Potri.013G072700.1	AT1G49245.1

Potra2n13c25648.1	-0.078837577	-0.10533579	0.707972292	0.898381609	Potri.013G072300.1	AT5G17620.1
Potra2n13c25649.1	-0.191758867	0.075818824	0.068004858	0.948577865		
Potra2n13c25650.1	0.241965047	0.019459217	0.081557425	0.987226805	Potri.013G072000.1	AT3G59600.1
Potra2n13c25651.1	-0.367182235	-0.180157316	0.036176306	0.866777645	Potri.013G071900.1	AT5G17630.1
Potra2n13c25652.1	0.118768416	0.064868473	0.230661593	0.907073354	Potri.013G071800.5	
Potra2n13c25653.1	0.053451492	0.014375961	0.792062622	0.990672454	Potri.013G071100.1	AT2G30410.1
Potra2n13c25654.3	0.142686413	0.321135617	0.363655541	0.562306981	Potri.013G071000.1	AT5G17640.1
Potra2n13c25655.1	-0.372350839	-0.327097361	0.031229354	0.48299238	Potri.013G070900.1	
Potra2n13c25656.1	0.256906841	0.296298582	0.169475119	0.817452127	Potri.013G070800.1	AT5G17660.1
Potra2n13c25657.1	-0.327419402	-0.183906226	0.009196898	0.812159187	Potri.013G070700.3	AT5G17670.1
Potra2n13c25658.1	-0.306762548	-0.191035627	0.015082912	0.788065047	Potri.013G070600.3	AT3G03590.1
Potra2n13c25659.1	-0.496609734	-0.409578459	0.209948159	0.72680202	Potri.013G070500.1	AT1G08540.1
Potra2n13c25660.1	-0.367256329	-0.091822387	0.558366575	0.975578749		
Potra2n13c25661.1	-0.428679892	0.027317925	0.00124433	0.98036348	Potri.013G070400.2	AT5G17690.1
Potra2n13c25662.1	1.643626983	-0.791452839	0.203570569	NA	Potri.013G070300.1	AT1G03220.1
Potra2n13c25663.1	0.834859816	0.166526271	4.51344E-09	0.897331585	Potri.013G070200.1	AT5G42540.1
Potra2n13c25664.1	-0.319988447	-0.328916744	0.08683132	0.495345445	Potri.013G070100.1	AT2G43030.1
Potra2n13c25665.1	0.230059073	0.07661288	0.044684619	0.91540496	Potri.013G070001.1	AT3G03250.1
Potra2n13c25666.2	-0.466613436	-0.086547284	1.95257E-05	0.935751704	Potri.013G069900.2	AT4G02600.1
Potra2n13c25667.1	1.284600235	0.105832706	1.18573E-07	0.95671968	Potri.013G069600.1	AT3G03620.1
Potra2n13c25668.1	1.498138134	0.302241218	5.94195E-07	0.904709467	Potri.013G069400.2	AT3G03620.1
Potra2n13c25669.1	-0.190303147	0.146998231	0.105245825	0.92298795	Potri.013G069000.4	AT3G03610.1
Potra2n13c25670.2	0.038815861	0.93155701	0.986740308	NA		
Potra2n13c25671.1	-0.145809724	0.048072535	0.322914041	0.959431187	Potri.013G068900.1	AT2G27110.2
Potra2n13c25672.1	0.102236182	-0.046370319	NA	NA		
Potra2n13c25673.1	-0.100921636	0.181021768	0.579759095	0.796890714	Potri.013G068800.2	AT5G17910.2
Potra2n13c25674.2	-0.203357437	-0.034557387	0.109449587	0.980430814	Potri.013G068700.1	AT1G22180.2
Potra2n13c25675.1	1.057584912	0.054040091	2.39861E-08	0.972159498	Potri.001G192000.1	AT3G15810.1
Potra2n13c25676.1	-3.264013574	-1.078779761	0.019058721	0.829862776	Potri.001G191900.1	AT1G80100.1
Potra2n13c25677.1	-0.737523818	-0.05557189	0.743452345	0.995581218	Potri.001G191800.1	AT3G21330.1
Potra2n13c25678.1	NA	NA	NA	NA	Potri.001G191700.1	AT2G19210.1
Potra2n13c25679.1					Potri.006G144400.1	AT3G16370.1

Potra2n13c25680.1	-0.369613737	-0.159025693	0.792354286	0.976302215	Potri.001G191500.1	AT3G16380.1
Potra2n13c25681.1	0.114648624	1.432350639	NA	NA	Potri.001G191400.1	AT3G16370.1
Potra2n13c25682.2	0.549582308	0.152912941	0.005484344	0.839956004	Potri.013G068300.1	AT1G08680.1
Potra2n13c25683.1	0.344783049	0.02303029	0.006962391	0.985263255	Potri.013G068200.1	AT5G56740.1
Potra2n13c25684.2	0.271274105	-0.001008813	0.007669687	0.999450033	Potri.018G070200.1	AT1G72550.1
Potra2n13c25685.3	0.073820226	0.067761302	0.746072266	0.970930327	Potri.013G067900.1	AT5G17710.2
Potra2n13c25686.1	-0.004508322	0.017431779	0.991063565	0.995005375	Potri.013G068000.1	
Potra2n13c25687.1	0.10285727	0.047417789	0.527975944	0.972159498	Potri.013G067700.1	AT1G08700.1
Potra2n13c25688.1	-0.11380626	-0.135480158	0.583472796	0.903477395	Potri.013G067600.1	AT1G08710.1
Potra2n13c25689.1	1.046227786	-0.828335558	0.778285071	NA	Potri.013G067500.1	AT1G08810.1
Potra2n13c25690.1	-0.119466951	0.85734424	0.769438506	0.574820121	Potri.013G067400.1	AT3G45400.1
Potra2n13c25691.1	0.128832405	-0.089651668	0.535108743	0.951754672	Potri.013G067300.2	AT5G17770.1
Potra2n13c25692.1	-0.144514815	-0.000324844	0.182567186	0.999937671	Potri.013G067200.1	AT5G17790.1
Potra2n13c25693.1	5.241580206	2.476400846	4.3024E-13	0.155975578	Potri.013G067000.1	AT5G17800.1
Potra2n13c25694.1	1.545832463	0.674963689	NA	NA	Potri.013G066900.1	AT3G03660.1
Potra2n13c25695.1	3.061176985	NA	NA	NA	Potri.013G066800.1	AT4G26010.1
Potra2n13c25696.1	-0.013028774	0.061071973	0.966225255	0.963557834	Potri.013G066700.1	AT3G47500.1
Potra2n13c25697.1	-1.342769532	-0.192413587	4.21027E-21	0.770943539	Potri.013G066600.1	
Potra2n13c25698.2	-0.105839035	0.064424703	0.731748234	0.957877578	Potri.001G246100.1	AT3G09360.1
Potra2n13c25699.1	2.578615813	-0.40859142	5.66604E-06	0.935474933	Potri.013G066400.1	AT3G03680.1
Potra2n13c25700.1	NA	NA	NA	NA		
Potra2n13c25701.1	1.64774763	NA	NA	NA	Potri.016G096100.1	
Potra2n13c25702.1	NA	1.363811008	NA	NA		
Potra2n13c25703.1	2.527157467	-0.775577439	6.91579E-05	0.878118098	Potri.013G066400.1	AT3G03680.1
Potra2n13c25704.1	-0.883480967	0.044416853	0.000546925	0.987226805	Potri.013G066200.1	AT3G03690.1
Potra2n13c25705.1	1.603826503	0.149095225	9.42233E-09	0.912777843	Potri.013G066100.1	AT3G03700.1
Potra2n13c25706.1	1.4510132	0.129091773	7.12519E-08	0.919722977	Potri.013G066000.1	AT5G17840.1
Potra2n13c25707.1	-1.409983009	0.06197156	NA	NA		
Potra2n13c25708.1	0.602928951	0.738456764	0.020819214	0.47454828		
Potra2n13c25709.1	0.63229074	0.756038083	0.01469156	0.46696299	Potri.013G065900.2	AT5G17850.1
Potra2n13c25710.1	1.01867856	0.233263031	0.000545967	0.902857376	Potri.013G065800.1	AT5G17860.1
Potra2n13c25711.1	-0.136545474	0.028149297	0.265347276	0.98036348	Potri.013G065700.2	AT3G03710.1

Potra2n13c25712.1	-1.282374531	0.706096468	0.531964423	NA		
Potra2n13c25713.1	0.867874688	0.408342378	1.96537E-06	0.322673812	Potri.013G065500.1	AT5G07610.1
Potra2n13c25714.1	NA	NA	NA	NA	Potri.013G078400.1	AT2G31720.1
Potra2n13c25715.3	0.94976918	0.305676512	9.57109E-09	0.441009549	Potri.013G065200.2	AT5G37480.1
Potra2n13c25716.1	-0.250048964	-0.051409268	0.215333686	0.979719961	Potri.013G065000.1	AT1G58030.1
Potra2n13c25717.1	-0.262691541	-0.167393254	0.353418589	0.842680333	Potri.013G064800.2	AT1G28760.1
Potra2n13c25718.1	-0.093257523	0.041117399	0.643964938	0.973347974	Potri.013G064700.1	AT5G35220.1
Potra2n13c25719.1	-0.322491526	-0.169031451	0.207678974	0.885375133	Potri.013G064600.1	AT3G03750.2
Potra2n13c25720.1	2.756638378	0.846107584	0.248050491	NA	Potri.013G064501.1	AT3G03760.1
Potra2n13c25721.1	NA	NA	NA	NA		
Potra2n13c25722.1	-0.412589244	-0.130807807	4.91094E-08	0.755741462	Potri.013G064400.1	AT2G02760.1
Potra2n13c25723.1	-0.311401327	-0.069029547	0.0879661	0.963557834	Potri.013G064300.3	AT3G03770.2
Potra2n13c25724.1	-0.800978159	-0.26944776	0.038895154	0.92978159		
Potra2n13c25725.1	0.793044786	-0.006809565	0.021536104	0.99859284	Potri.013G064200.1	AT5G15120.1
Potra2n13c25726.1	4.126932396	NA	0.06743462	NA	Potri.013G063200.1	AT3G05950.1
Potra2n13c25727.1	4.837594001	NA	0.00702301	NA	Potri.013G063200.1	AT3G05950.1
Potra2n13c25728.1	2.165919294	NA	NA	NA	Potri.013G063200.1	AT3G05950.1
Potra2n13c25729.1	5.29084885	-0.767708831	0.001509254	NA	Potri.013G063200.1	AT3G05950.1
Potra2n13c25730.1	5.933274919	2.134902029	0.002127419	NA	Potri.013G063200.1	AT3G05950.1
Potra2n13c25731.1	5.712299531	NA	0.002499988	NA	Potri.013G063200.1	AT3G05950.1
Potra2n13c25732.1	4.390180378	NA	0.223691807	NA	Potri.013G063200.1	AT3G05950.1
Potra2n13c25733.1	4.805105667	NA	0.01017475	NA	Potri.013G063200.1	AT3G05950.1
Potra2n13c25734.1	5.133789133	NA	0.007724898	NA	Potri.013G063200.1	AT3G05950.1
Potra2n13c25735.1	4.958281685	-0.767708831	0.002499717	NA	Potri.013G063200.1	AT3G05950.1
Potra2n13c25736.1	1.43326642	-1.049507415	0.174398668	NA	Potri.013G063200.1	AT3G05950.1
Potra2n13c25737.1	0.808110406	-2.466612692	0.795340993	NA	Potri.013G063050.1	AT3G05950.1
Potra2n13c25738.1	-0.124338881	-0.00802246	0.301318985	0.995581218	Potri.013G062900.1	AT2G36900.1
Potra2n13c25739.1	-0.015178156	0.017161209	0.914832288	0.986266735	Potri.013G062800.1	AT5G35910.1
Potra2n13c25740.1	0.587381732	0.189182631	0.683389524	0.984292161	Potri.013G062700.1	AT2G21720.1
Potra2n13c25741.1	-0.060112928	-0.184634422	0.846211775	0.890978957	Potri.013G062600.2	AT3G03773.1
Potra2n13c25742.1	0.646288856	0.145558281	9.08859E-05	0.922778027	Potri.013G062500.12	AT5G55000.2
Potra2n13c25743.1	NA	1.394613675	NA	NA		

Potra2n13c25744.7	-0.304878342	0.263572192	0.314552256	0.937846405	Potri.013G062300.11	
Potra2n13c25745.1	-0.695180573	-0.320499103	0.030028258	0.900015282	Potri.013G062000.1	AT5G13530.2
Potra2n13c25746.3	-0.079622598	-0.319118378	0.840798673	0.903496619	Potri.013G061800.2	AT5G17920.2
Potra2n13c25747.1	-0.034900834	0.444045362	0.843166114	0.688499606	Potri.013G061500.1	AT3G62800.2
Potra2n13c25748.3	0.671520689	0.333088961	1.80668E-06	0.656082742	Potri.013G061400.1	AT5G17930.1
Potra2n13c25749.1	0.147460216	0.759136184	0.678015032	0.814981587	Potri.013G061200.1	AT1G08570.1
Potra2n13c25750.1	7.30033255	3.970045918	3.43901E-19	0.098336438		
Potra2n13c25751.1	0.173413154	0.056970706	0.194151421	0.9571583	Potri.013G060900.1	AT5G18260.1
Potra2n13c25752.1	0.634303179	0.179422065	0.001148312	0.835819291	Potri.013G060800.1	AT2G03090.1
Potra2n13c25753.1	0.546226141	0.170526872	0.000553418	0.849446235		
Potra2n13c25754.1	-0.61196956	-0.415158057	0.440724881	0.912777843		
Potra2n13c25755.1	-0.325257975	-0.057812563	0.127528872	0.978251387		
Potra2n13c25756.1	-0.131793575	-0.075987726	0.536312245	0.957525726	Potri.013G060500.1	AT5G56340.1
Potra2n13c25757.1	-0.409189164	-0.345242713	0.813762465	NA	Potri.013G060400.1	AT5G56350.1
Potra2n13c25758.1	0.02462806	0.15370441	0.851145589	0.829862776	Potri.013G060300.1	AT5G56360.1
Potra2n13c25759.1	2.856599909	3.229757782	NA	NA		
Potra2n13c25760.1	0.274563388	0.075159354	0.516115091	0.971752582	Potri.013G060200.4	AT3G04030.3
Potra2n13c25761.1	3.934419061	1.307200853	6.6295E-21	0.426555455	Potri.013G060000.1	AT5G60900.1
Potra2n13c25762.1	0.211766064	0.04581318	0.069934815	0.966917036	Potri.013G059800.2	AT5G18230.1
Potra2n13c25763.1	-0.286973632	-0.262426998	0.015551216	0.5048539	Potri.013G059700.1	AT1G64760.2
Potra2n13c25764.1	3.606627378	-0.553471547	0.000528821	NA	Potri.013G059600.1	AT5G25830.1
Potra2n13c25765.1	0.089203785	-0.016310122	0.7794143	0.996032145	Potri.013G059500.1	AT2G23840.1
Potra2n13c25766.2	-0.052233394	0.076161847	0.638371295	0.935099709		
Potra2n13c25767.1	0.698561141	0.219060416	1.91859E-08	0.794596445	Potri.013G059100.2	AT3G03980.1
Potra2n13c25768.1	0.271544588	-0.175794516	0.404283755	0.909675463	Potri.013G058900.1	AT4G13650.1
Potra2n13c25769.1	0.027641917	-0.032365845	0.889028062	0.97653548	Potri.013G058800.1	AT1G54390.4
Potra2n13c25770.1	0.301636943	0.06260793	0.293657832	0.974275337		
Potra2n13c25771.1	0.289079751	-0.194587955	0.673536999	0.96820892	Potri.013G058700.1	AT1G54385.2
Potra2n13c25772.1	-0.005524238	-0.051219564	0.970972505	0.955322605	Potri.013G058500.1	AT3G03960.1
Potra2n13c25773.1	0.847727993	0.018002403	4.59143E-07	0.993589787	Potri.013G058400.1	AT3G03920.1
Potra2n13c25774.1	-5.00763E-05	-0.331592485	0.999895833	0.821709751	Potri.013G058300.11	AT5G18170.1
Potra2n13c25775.1	-0.200683555	0.18145084	0.319135475	0.914551931	Potri.013G058200.1	AT5G18150.1

Potra2n13c25776.1	-0.200683555	0.18145084	0.319135475	0.914551931	Potri.013G058100.1	AT5G18140.1
Potra2n13c25777.1	0.337081886	-2.37217E-05	0.249143352	0.999950004	Potri.013G057900.2	AT3G03900.1
Potra2n13c25778.1	-0.125275358	-0.33565248	0.719735338	0.784063198		
Potra2n13c25779.1	-0.107267139	-0.327413616	0.765028481	0.799868271	Potri.001G003500.1	AT5G56520.1
Potra2n13c25780.1	-0.31833051	0.21666062	0.582511993	0.948577865	Potri.001G003500.1	AT5G56520.1
Potra2n13c25781.1	0.398463514	0.327890474	0.004934062	0.577136028	Potri.013G057700.2	AT3G03890.1
Potra2n13c25782.1	-0.353546044	-0.148927101	0.0518369	0.882488511	Potri.013G057600.1	AT1G55340.1
Potra2n13c25783.1	-2.04757808	-0.539044225	2.47278E-11	0.396758188	Potri.019G035500.1	AT5G56540.1
Potra2n13c25784.1	-0.859473557	-0.253046496	4.00048E-05	0.786746168	Potri.013G057200.3	AT5G18130.1
Potra2n13c25785.1	0.119558636	-0.045628759	0.29547187	0.953831244	Potri.013G057100.1	AT3G03860.1
Potra2n13c25786.1	0.043276514	0.0327976	0.722066667	0.95981322	Potri.013G057000.2	AT5G18110.1
Potra2n13c25787.1	0.079103687	0.16742233	0.742639004	0.893135869	Potri.013G056900.2	AT5G18100.1
Potra2n13c25788.2	-0.554476568	-0.138041884	0.000265881	0.817799498	Potri.013G056800.1	AT5G19780.1
Potra2n13c25789.1	-0.037334162	0.338631684	0.95662095	0.937146435	Potri.013G056700.1	AT5G19790.1
Potra2n13c25790.1	0.214652414	0.065381316	0.04085258	0.935851032	Potri.013G056600.2	AT5G19820.1
Potra2n13c25791.1	3.240873759	2.780317053	0.042875982	NA	Potri.013G056500.1	AT3G13540.1
Potra2n13c25792.1	3.632665346	2.274780034	0.00046131	NA		
Potra2n13c25793.1	2.703550807	-0.134975629	0.12579456	NA	Potri.013G056400.1	AT3G13540.1
Potra2n13c25794.1	-0.61910233	-0.767708831	NA	NA		
Potra2n13c25795.1	-0.263147779	0.059446241	0.030150187	0.954476684	Potri.013G056200.1	AT3G03810.1
Potra2n13c25796.1	1.576740549	1.705529685	0.274683036	NA		
Potra2n13c25797.1	0.894947559	0.127068574	0.000302834	0.947969749	Potri.013G056000.2	AT3G50950.1
Potra2n13c25798.1	0.552058431	-0.417225422	0.292287373	0.919740601		
Potra2n13c25799.1	0.106828089	0.002611504	0.773127715	0.999458638		
Potra2n13c25800.1	-0.273406303	-0.048891855	0.589172411	0.9869326	Potri.013G055800.1	
Potra2n13c25801.1	-0.032865659	0.058006092	0.933277265	0.976043496		
Potra2n13c25802.1	NA	NA	NA	NA	Potri.013G055700.2	AT5G20950.1
Potra2n13c25803.1	1.986113985	1.442131939	0.002077849	0.306363984	Potri.013G055500.1	AT5G17980.1
Potra2n13c25804.1	3.406415142	3.551062057	0.069517364	NA	Potri.013G055500.1	AT5G17980.1
Potra2n13c25805.1	-1.09536862	-2.59744079	NA	NA		
Potra2n13c25806.1	-0.350525005	-0.134647611	0.060928247	0.923397137	Potri.013G055300.1	AT3G03790.2
Potra2n13c25807.3	-1.056411318	-0.529716626	1.27502E-07	0.395174837	Potri.013G055100.2	AT5G32450.1

Potra2n13c25808.1	-2.403631343	-0.331037094	1.40425E-09	0.718629174	Potri.013G055000.1	AT5G12380.1
Potra2n13c25809.1	-2.965098888	-1.226701545	9.92199E-10	0.351105764	Potri.013G054900.1	AT1G54400.1
Potra2n13c25810.1	0.073015395	-2.523469902	0.983047839	NA	Potri.013G054800.1	AT1G54400.1
Potra2n13c25811.1	-3.413595555	-3.559471648	0.415060834	NA	Potri.013G054600.1	AT4G31320.1
Potra2n13c25812.1	-0.209991544	-0.156318857	0.073336013	0.850863374	Potri.013G054400.1	AT1G09220.1
Potra2n13c25813.1	-0.276497814	-0.086039761	0.250001421	0.953108412		
Potra2n13c25814.1	-1.064493006	-0.565046899	0.002244025	0.821348554	Potri.013G054200.1	AT5G18270.1
Potra2n13c25815.1	2.047043968	-0.095913674	0.067899128	NA	Potri.013G054100.1	AT2G39050.1
Potra2n13c25816.1	-0.643771344	-0.923232116	0.828744105	NA	Potri.013G054000.1	AT3G04070.1
Potra2n13c25817.1	NA	NA	NA	NA		
Potra2n13c25818.1	-0.507738782	0.244416558	0.118337989	0.890446513	Potri.013G053800.1	
Potra2n13c25819.1	0.313155779	0.042081281	0.237876745	0.983446961	Potri.013G053600.1	AT5G15010.1
Potra2n13c25820.1	-0.231543515	-0.187471533	0.209151073	0.910815461	Potri.013G053500.1	AT5G18280.1
Potra2n13c25821.1	-0.765507251	-0.191139437	2.69287E-08	0.821140081	Potri.013G053400.2	AT3G04090.1
Potra2n13c25822.2	-0.488060872	-0.694710051	0.055115643	0.269659806	Potri.013G053200.1	AT1G08560.1
Potra2n13c25823.2	-0.521972319	-0.659016302	0.022162777	0.295777432	Potri.013G053100.2	AT1G08550.2
Potra2n13c25824.3	-1.328951947	-1.478536539	NA	NA	Potri.019G030600.1	AT5G48740.1
Potra2n13c25825.1	-0.153084792	-0.961730314	0.90047059	NA	Potri.013G052800.1	AT2G20340.1
Potra2n13c25826.1	0.374039225	-0.17592049	0.041861246	0.751093503		
Potra2n13c25827.1	0.362354317	-0.106673233	0.023928038	0.865591169	Potri.013G052700.2	AT1G08530.1
Potra2n13c25828.1	NA	0.674963689	NA	NA		
Potra2n13c25829.1	9.285947272	5.363644491	6.77101E-17	NA	Potri.013G052600.1	AT5G18310.2
Potra2n13c25830.1	-0.570505959	-0.65728209	0.204817415	0.753998054	Potri.013G052500.1	AT3G07360.1
Potra2n13c25831.3	1.433255306	0.916495385	0.633416297	NA	Potri.013G052100.1	AT5G39110.1
Potra2n13c25832.1	6.987864183	NA	1.38941E-07	NA	Potri.013G051700.1	AT3G05950.1
Potra2n13c25833.1	-0.043119493	-0.097887424	0.684106116	0.874507876	Potri.013G051500.1	AT4G11150.1
Potra2n13c25834.1	-0.06100969	0.01813496	0.63612409	0.985016122	Potri.013G051400.2	AT3G04240.1
Potra2n13c25835.1	-0.473591254	0.320387601	0.160326821	0.822982825	Potri.013G051300.1	AT5G63930.1
Potra2n13c25836.2	-0.522737254	-0.058237735	0.000475967	0.941677175	Potri.013G051200.2	AT5G18410.2
Potra2n13c25837.1	-1.386406621	-0.270716867	7.00245E-05	0.936402128	Potri.013G051100.1	AT5G33370.1
Potra2n13c25838.1	-0.941761399	-0.027416588	1.55591E-08	0.988601759	Potri.013G050900.1	AT5G18460.1
Potra2n13c25839.2	-0.106714706	-0.061390295	0.528050037	0.953768643	Potri.013G049900.1	AT1G69680.1

Potra2n13c25840.1	5.850351677	2.690879854	7.32526E-05	NA	Potri.013G050500.1	AT5G18470.1
Potra2n13c25841.1	0.190360104	-0.017774215	0.401370717	0.992934677	Potri.013G049800.1	AT5G38890.1
Potra2n13c25842.1	-0.227913347	0.170418321	0.506822737	0.903934429	Potri.013G050300.1	AT5G37360.1
Potra2n13c25843.1					Potri.001G215300.1	
Potra2n13c25844.1	NA	1.571529889	NA	NA	Potri.013G049600.1	AT1G64780.1
Potra2n13c25845.1	-0.479651788	0.210977871	0.900340077	NA	Potri.013G049500.1	AT5G18520.1
Potra2n13c25846.1	0.034446478	-0.073652093	0.853312076	0.939653927	Potri.013G049300.2	AT5G32470.1
Potra2n13c25847.1	-0.525914296	-0.065698192	3.07214E-05	0.948577865	Potri.013G049100.4	AT5G18480.1
Potra2n13c25848.1	-0.490419164	-0.415734536	0.498067375	0.913281585		
Potra2n13c25849.1	0.088648543	0.175500067	0.880312043	0.966917036	Potri.014G002800.2	AT1G16760.1
Potra2n13c25850.1	0.551154448	0.478520752	0.011857441	0.108619662	Potri.014G009400.1	AT3G14470.1
Potra2n13c25851.1	1.052503158	0.605280112	0.000780338	0.237361979		
Potra2n13c25852.1	-0.382550779	-0.565084101	0.518511753	0.791589438	Potri.013G048900.1	AT4G33270.1
Potra2n13c25853.1	0.348878637	0.277805926	0.000110535	0.692084498	Potri.013G048700.1	AT3G04340.1
Potra2n13c25854.1	0.274682034	-0.144915906	0.403054166	0.937276488	Potri.013G048800.1	AT1G08590.1
Potra2n13c25855.1	-0.159661302	0.156235502	0.222245784	0.842247476	Potri.019G021500.1	AT1G08600.4
Potra2n13c25856.1	3.85960322	1.363811008	0.07720464	NA	Potri.013G048400.1	AT2G33320.1
Potra2n13c25857.1	-0.7373305	-0.322834076	0.000176177	0.635323636	Potri.013G048300.1	AT3G04350.1
Potra2n13c25858.1	3.31653803	1.394613675	NA	NA	Potri.010G065800.1	AT1G04520.1
Potra2n13c25859.1	-0.385049087	0.002545122	0.018031905	0.999282348	Potri.013G048100.2	AT3G04380.2
Potra2n13c25860.1	-0.813708678	0.230770367	0.562896253	0.975244691		
Potra2n13c25861.1	0.695053496	0.368752579	9.21129E-05	0.773157146	Potri.013G048000.1	AT5G29000.2
Potra2n13c25862.1	2.328820319	0.576017248	6.09763E-40	0.195423497		
Potra2n13c25863.1	3.381987842	0.810376539	4.18525E-53	0.077114891	Potri.013G047900.2	AT5G40010.1
Potra2n13c25864.1	0.98577132	0.407297816	1.18666E-08	0.755774501	Potri.013G047900.2	AT5G40010.1
Potra2n13c25865.2	-0.226455369	-0.011820812	0.082183136	0.992319504	Potri.019G020000.1	AT3G04460.1
Potra2n13c25866.1	-1.495684284	-1.218911784	0.011625487	0.262207477	Potri.013G047700.1	AT1G04030.1
Potra2n13c25867.1	-1.199367852	-0.503778893	1.30368E-08	0.401060435	Potri.013G047600.4	AT5G28910.2
Potra2n13c25868.1	-0.195373521	-0.065624252	0.030131249	0.919424623	Potri.013G047500.1	AT3G04470.1
Potra2n13c25869.1	0.82357019	NA	NA	NA	Potri.006G184400.1	AT3G09840.1
Potra2n13c25870.1	0.393660972	0.102741784	3.76706E-05	0.906107926	Potri.013G047400.1	AT3G04480.1
Potra2n13c25871.1	-0.06797979	-0.025321767	0.644060565	0.985393296	Potri.013G047300.2	AT5G28850.2

Potra2n13c25872.1	-0.146993813	-0.069126727	0.278005025	0.946464685	Potri.013G047200.1	AT4G14340.1
Potra2n13c25873.1	0.032364596	0.060309416	0.847505177	0.969898621	Potri.013G047100.1	AT1G10095.1
Potra2n13c25874.1	-0.908686112	-0.494383217	0.004839973	0.720488481	Potri.019G019300.1	AT3G04760.1
Potra2n13c25875.1	-0.150878176	0.090635557	0.27004717	0.935751704	Potri.013G046900.1	AT3G04500.1
Potra2n13c25876.1	-1.040965727	-0.214924005	0.33267354	0.978251387		
Potra2n13c25877.1	-1.35927387	-0.066852884	NA	NA	Potri.013G117150.1	
Potra2n13c25878.1	0.172211875	0.051886962	0.166685938	0.95981322	Potri.013G046700.1	AT1G08620.1
Potra2n13c25879.1	0.01595368	0.013880763	0.964797847	0.993589888		
Potra2n13c25880.1	-0.379533464	-0.070196883	0.005267602	0.966191551	Potri.013G046500.1	AT1G07090.1
Potra2n13c25881.1	0.92641307	2.203303301	NA	NA		
Potra2n13c25882.1	4.69175405	0.885879807	2.49509E-31	0.704368358	Potri.013G046400.1	AT1G08630.4
Potra2n13c25883.2	0.088521567	0.088613112	0.362100147	0.923240242	Potri.013G046300.1	AT1G09770.1
Potra2n13c25884.1	0.753824471	0.285275298	0.0315442	0.874808359	Potri.013G046100.1	AT3G04530.1
Potra2n13c25885.1						
Potra2n13c25886.1	0.412302933	0.142620047	7.35927E-05	0.784830006	Potri.013G045900.1	AT2G39840.1
Potra2n13c25887.1	0.195408981	-0.168086515	0.643809959	0.924309478	Potri.013G045800.2	AT2G20410.1
Potra2n13c25888.1	3.662802866	NA	0.36293887	NA	Potri.013G045700.1	
Potra2n13c25889.1	1.459962178	NA	NA	NA	Potri.013G045500.1	
Potra2n13c25890.1	8.268318887	5.212784584	8.49443E-12	NA	Potri.013G045333.1	
Potra2n13c25891.1	6.961017862	1.797133164	8.61991E-16	NA	Potri.T077910.1	
Potra2n13c25892.1	5.772268566	0.674963689	9.654E-05	NA	Potri.013G045333.1	
Potra2n13c25893.1	5.198698192	1.871975723	0.001207788	NA	Potri.T051940.1	
Potra2n13c25894.1	7.292858894	5.276046566	2.19332E-08	NA	Potri.013G045300.1	
Potra2n13c25895.1	7.688839518	3.584701124	8.4125E-32	0.40904397	Potri.T038955.1	
Potra2n13c25896.1	10.7581182	6.374973556	2.49873E-21	0.23623457	Potri.013G045300.1	
Potra2n13c25897.1	2.86853061	NA	NA	NA	Potri.T038955.1	
Potra2n13c25898.1	4.110370104	NA	0.078543663	NA	Potri.T038955.1	
Potra2n13c25899.1					Potri.T090900.1	
Potra2n13c25900.1	5.476309858	2.162677504	1.29E-05	NA	Potri.013G045700.1	
Potra2n13c25901.1	0.962940164	0.830241072	0.098409417	0.734057255	Potri.013G045200.1	AT3G23240.1
Potra2n13c25902.1					Potri.013G045101.1	AT1G06620.1
Potra2n13c25903.1	NA	NA	NA	NA		

Potra2n13c25904.1	2.972559951	1.195403485	4.47493E-11	0.532409631	Potri.013G045000.1	AT1G06620.1
Potra2n13c25905.1	-0.38844084	-0.077116827	0.043148606	0.943685779	Potri.013G044700.1	AT1G08070.1
Potra2n13c25906.1	-0.073361256	0.08016262	0.625792075	0.930598202	Potri.013G044600.1	AT3G04560.1
Potra2n13c25907.1	1.64774763	NA	NA	NA	Potri.013G044500.1	AT4G14465.1
Potra2n13c25908.1	0.365635187	0.082376175	0.006795094	0.935508417	Potri.013G044300.1	AT1G54650.2
Potra2n13c25909.1	-0.359819169	0.032502817	0.022611216	0.979094708	Potri.013G044100.1	AT3G04580.1
Potra2n13c25910.1	-0.211309437	-1.313912451	0.868921042	0.804007296	Potri.013G043900.1	AT1G54560.1
Potra2n13c25911.1	8.223082424	4.595469611	1.08132E-09	NA	Potri.013G043800.2	AT3G56710.1
Potra2n13c25912.1	-1.295728824	-0.09443736	1.34105E-26	0.966917036	Potri.013G043700.1	AT5G28640.1
Potra2n13c25913.1	-0.111666764	0.221141482	0.703308363	0.884463725	Potri.013G043500.1	AT2G37690.1
Potra2n13c25914.1	-2.743206852	-1.01653665	0.057715998	0.836066102	Potri.013G043400.1	AT1G08800.2
Potra2n13c25915.1	-0.193755843	-0.087105116	0.282660236	0.936549944		
Potra2n13c25916.1	1.265737184	-0.120867519	0.095207419	0.971317865	Potri.013G043100.2	AT1G01225.1
Potra2n13c25917.4	-0.136778639	-0.005575869	0.340485545	0.996032145	Potri.013G043000.1	AT3G04610.1
Potra2n13c25918.1	1.308046817	1.63687976	0.452432163	NA	Potri.013G042900.1	AT3G04620.1
Potra2n13c25919.1	-0.558286541	0.060779618	0.002037257	0.973347974	Potri.013G042800.1	AT1G54460.1
Potra2n13c25920.1	-1.35927387	0.439464228	NA	NA		
Potra2n13c25921.1	-1.051802361	0.148614421	9.02285E-06	0.961069142	Potri.013G042700.1	AT2G30620.1
Potra2n13c25922.1	0.040437313	-1.465929376	NA	NA	Potri.002G043200.1	AT5G09500.1
Potra2n13c25923.1	-0.025822995	0.200372435	0.935252261	0.870293488	Potri.013G042600.1	AT3G04670.1
Potra2n13c25924.1	2.735991243	NA	NA	NA	Potri.013G042500.1	AT3G04690.1
Potra2n13c25925.1	NA	NA	NA	NA	Potri.013G042400.1	AT1G08790.1
Potra2n13c25926.1	0.166311104	0.034749621	0.245806584	0.98317667	Potri.013G042300.1	AT3G04710.3
Potra2n13c25927.1	NA	NA	NA	NA	Potri.005G054700.1	AT1G08770.1
Potra2n13c25928.1	-0.503559907	-0.190911854	0.000606096	0.775343511	Potri.013G042100.1	AT1G08780.1
Potra2n13c25929.3	-0.085482937	0.079599317	0.806908209	0.973948905	Potri.013G042000.1	AT2G30580.1
Potra2n13c25930.1	4.923795455	3.620884376	0.000517827	NA	Potri.013G041900.1	AT3G04720.1
Potra2n13c25931.1	7.212219831	3.120714617	2.86187E-05	0.693680634	Potri.013G041700.1	AT3G04720.1
Potra2n13c25932.1	4.95547841	2.704213029	0.000527276	0.667076083	Potri.013G041600.1	AT3G04720.1
Potra2n13c25933.1	3.052905509	2.533912481	0.000824045	0.443657809		
Potra2n13c25934.1	-0.879143358	-0.064105974	2.55804E-06	0.956865545	Potri.013G041400.1	AT3G04730.1
Potra2n13c25935.1	-1.874330958	-0.211047274	7.92513E-30	0.849067086	Potri.013G041300.1	AT5G43700.1

Potra2n13c25936.1	-0.433618254	-0.074893652	0.000400008	0.951726178	Potri.005G053700.1	AT3G04740.1
Potra2n13c25937.1	-2.308227488	-2.412394178	0.48246076	NA	Potri.005G053600.1	AT3G04750.1
Potra2n13c25938.1	-0.252725393	-0.046103501	0.072282142	0.960981446	Potri.013G041000.1	AT4G02590.1
Potra2n13c25939.1	-0.747607673	-0.177147637	4.29326E-07	0.759797728	Potri.013G040900.1	AT5G28750.1
Potra2n13c25940.1	-0.726535651	-0.3012764	0.00040141	0.71248439	Potri.013G040800.3	AT4G12540.1
Potra2n13c25941.1	-0.98324083	-0.138246828	0.000248868	0.956865545	Potri.013G040700.1	AT5G28770.2
Potra2n13c25942.1	0.718986422	0.179234613	3.78357E-06	0.804796567	Potri.013G040600.1	AT5G28840.2
Potra2n13c25943.1	1.029489298	0.112064845	2.29075E-10	0.921099436	Potri.013G040500.1	AT5G28830.1
Potra2n13c25944.1	-0.172806563	-0.810803212	0.965822291	NA		
Potra2n13c25945.1	-1.409983009	-1.55593228	NA	NA	Potri.T103600.2	AT2G38290.1
Potra2n13c25946.1	7.970608502	3.78959661	1.57057E-12	NA	Potri.013G040300.1	AT3G22930.1
Potra2n13c25947.1	5.731151716	1.571529889	0.001565798	NA	Potri.013G040200.1	AT3G63380.1
Potra2n13c25948.1	5.271543815	NA	0.020036047	NA		
Potra2n13c25949.1	-4.408548648	0.041597284	0.022052441	0.996851113		
Potra2n13c25950.1	1.694858984	1.343177551	6.47258E-10	7.55527E-06	Potri.013G040000.1	AT1G60710.1
Potra2n13c25951.1	0.316817802	0.281360942	0.804106544	0.971593102	Potri.013G039900.1	AT4G29250.1
Potra2n13c25952.2	0.003391458	0.055218805	0.983579883	0.947547562	Potri.013G039600.1	AT5G49570.1
Potra2n13c25953.1	-0.256020468	-0.178943913	0.114566774	0.861385911	Potri.013G039500.2	AT3G04780.1
Potra2n13c25954.1	0.011870517	-0.333156387	0.961810082	0.682454638		
Potra2n13c25955.1	0.052029908	-0.23987764	0.833244739	0.813731863	Potri.013G039400.1	AT3G04790.1
Potra2n13c25956.1	3.430204174	-1.271893716	2.49636E-05	NA	Potri.013G039300.1	AT4G14746.1
Potra2n13c25957.1	-2.337106966	-2.486725844	NA	NA		
Potra2n13c25958.1	0.246609741	-0.034538708	0.106108902	0.984292161	Potri.013G039200.1	AT3G04800.1
Potra2n13c25959.1	4.026561636	2.391628587	4.12322E-06	0.466660959	Potri.013G039100.2	AT5G28300.1
Potra2n13c25960.1	-0.366561337	-0.264579431	0.372351239	0.890611161	Potri.013G039000.2	AT3G04810.2
Potra2n13c25961.1	0.228224791	-1.465929376	NA	NA	Potri.013G038901.1	AT4G14750.1
Potra2n13c25962.1	0.062807912	-0.050748964	0.785146268	0.9761362	Potri.013G038700.1	AT3G04830.1
Potra2n13c25963.1	NA	0.674963689	NA	NA	Potri.013G038400.1	AT3G22910.1
Potra2n13c25964.1	0.440620796	-0.130242773	0.169168928	0.954559968	Potri.005G051100.1	AT3G22780.1
Potra2n13c25965.1	-1.35927387	-1.507841271	NA	NA		
Potra2n13c25966.1	-0.260639826	-0.080157551	0.662542203	0.966917036	Potri.013G038100.2	AT5G56750.1
Potra2n13c25967.1	0.61091313	0.388719803	0.044814996	0.855587395	Potri.013G038000.1	AT5G28150.1

Potra2n13c25968.1	-0.52889814	0.439227696	0.158538959	0.831597503	Potri.013G037900.1	AT1G79590.1
Potra2n13c25969.1	4.093385408	2.283425176	0.12725361	NA	Potri.013G037800.1	AT5G43150.1
Potra2n13c25970.1	0.938700725	0.243833289	1.36412E-18	0.671682202	Potri.013G037700.1	AT3G04870.1
Potra2n13c25971.1	-0.6063148	-0.358160965	0.498934307	0.936890993	Potri.005G050600.1	AT3G04880.1
Potra2n13c25972.1	NA	NA	NA	NA	Potri.013G036500.1	AT3G04890.1
Potra2n13c25973.1	-0.754542447	-0.486908809	0.58865225	0.937846405	Potri.005G050600.1	AT3G04880.1
Potra2n13c25974.1	2.686710473	NA	NA	NA	Potri.013G037300.1	AT5G36930.2
Potra2n13c25975.1						
Potra2n13c25976.1	2.831353739	-0.584451815	1.09962E-05	NA	Potri.013G036500.1	AT3G04890.1
Potra2n13c25977.3	-0.572132082	0.171881219	0.046769477	0.921099436	Potri.013G036500.1	AT3G04890.1
Potra2n13c25978.1	NA	0.674963689	NA	NA		
Potra2n13c25979.1	2.110518669	0.674963689	NA	NA	Potri.013G037200.1	
Potra2n13c25980.1	NA	NA	NA	NA	Potri.013G037400.1	
Potra2n13c25981.1	-0.255548061	0.054980393	0.339457099	0.972159498	Potri.013G036500.1	AT3G04890.1
Potra2n13c25982.1	-1.791822974	-0.723635624	1.11825E-10	0.513600996	Potri.013G036400.1	AT3G04860.1
Potra2n13c25983.1	-1.118584206	-0.347573839	1.45568E-11	0.326024562	Potri.013G036300.1	AT3G04910.1
Potra2n13c25984.1	-0.537521574	-0.113540046	0.642445231	0.986547807	Potri.013G036200.1	AT1G54520.1
Potra2n13c25985.1	0.451867957	-0.036779139	0.004996374	0.982812163	Potri.013G036100.1	AT5G28060.1
Potra2n13c25986.1	4.122994579	1.394613675	0.050412181	NA	Potri.013G036000.1	AT5G28050.2
Potra2n13c25987.1	-0.950143242	-0.058082558	0.098271395	0.9896961	Potri.013G035900.1	AT3G23750.1
Potra2n13c25988.1	0.033908847	0.009068103	0.843396409	0.993577196	Potri.013G035800.7	AT4G32640.2
Potra2n13c25989.1	1.076036249	1.635457753	0.65593764	0.64462335	Potri.005G049000.1	AT1G54500.1
Potra2n13c25990.7	-0.141415562	0.005349102	0.325189905	0.996032145	Potri.013G035600.1	AT1G54490.1
Potra2n13c25991.1	-0.112317346	-0.123646601	0.34673508	0.863957913	Potri.013G035200.2	AT3G04940.1
Potra2n13c25992.1	4.670183242	2.501508747	0.016353478	NA		
Potra2n13c25993.1	-0.028755303	0.060507916	0.890275617	0.947969749	Potri.013G035100.1	AT5G27970.1
Potra2n13c25994.1	0.365639772	0.026373788	0.092427148	0.982422288	Potri.013G034800.1	AT1G08720.1
Potra2n13c25995.1	-0.34839918	0.438488581	0.228998981	0.623736221	Potri.013G034600.1	
Potra2n13c25996.1	-0.519770096	0.102393101	0.386748848	0.981726976	Potri.002G026800.1	AT4G40030.2
Potra2n13c25997.1	0.285777167	0.486635205	0.256870613	0.689540835	Potri.013G034500.1	
Potra2n13c25998.2	0.16307766	0.366936645	0.662700995	0.828625141	Potri.013G032600.2	AT1G12700.1
Potra2n13c25999.1	0.074081023	-0.01593039	0.761620457	0.991407187	Potri.013G034100.1	AT3G04950.1

Potra2n13c26000.1	-0.093682798	0.008903325	0.668151219	0.995579852	Potri.013G034000.1	AT3G05100.1
Potra2n13c26001.1	-0.357755442	-0.073733453	0.077758122	0.948577865	Potri.013G033900.1	AT5G27950.1
Potra2n13c26002.1	-0.155586174	0.004132979	0.063852431	0.996154416	Potri.013G033800.3	AT3G05090.1
Potra2n13c26003.1	-1.139437934	-0.481053001	0.004236321	0.724604666	Potri.013G033300.1	AT1G54570.1
Potra2n13c26004.1	9.460121068	6.437199687	5.56961E-25	0.069594409	Potri.013G033200.1	AT1G24140.1
Potra2n13c26005.1	8.640511518	5.53108091	1.09168E-63	0.044004179	Potri.013G033200.1	AT1G24140.1
Potra2n13c26006.1	0.82357019	NA	NA	NA		
Potra2n13c26007.2	-0.449578321	-0.500654784	0.303993363	0.742369403	Potri.013G033000.1	AT1G54570.1
Potra2n13c26008.1	1.341587218	0.099334298	1.32956E-06	0.974756511	Potri.013G032900.1	
Potra2n13c26009.1	2.068454309	NA	NA	NA	Potri.013G032700.1	AT3G04945.1
Potra2n13c26010.1	0.103707939	0.042141979	0.809977246	0.986636967	Potri.013G032600.2	AT1G12700.1
Potra2n13c26011.2	0.249748321	0.178899173	0.022059139	0.788066587	Potri.013G032400.1	AT5G41070.1
Potra2n13c26012.7	0.511451419	-0.077771087	0.000260956	0.955628098	Potri.013G032300.1	AT3G05060.1
Potra2n13c26013.5	0.172545398	0.037156249	0.169472078	0.974782554	Potri.013G032200.1	AT1G54610.1
Potra2n13c26014.1	0.167972725	0.05923254	0.118615175	0.930598202	Potri.013G032100.1	AT3G05040.1
Potra2n13c26015.1	2.882853291	0.046281864	6.72623E-11	0.994403137		
Potra2n13c26016.1	-0.037746284	0.079620594	0.856034304	0.940449094	Potri.013G031700.1	AT3G05030.1
Potra2n13c26017.1	-1.409983009	-1.55593228	NA	NA	Potri.013G031600.2	AT2G27410.1
Potra2n13c26018.1	-0.744834904	-0.208004239	0.655249024	NA		
Potra2n13c26019.1	-0.472101389	-0.147795551	0.00287153	0.885375133	Potri.013G031400.1	AT1G08820.2
Potra2n13c26020.1	0.170602059	0.015126219	0.221639683	0.987226805	Potri.013G031300.1	AT3G05020.1
Potra2n13c26021.3	-0.060740966	-0.044537823	0.716665968	0.956622854	Potri.013G031200.1	AT3G05010.1
Potra2n13c26022.1	2.632524198	NA	NA	NA	Potri.005G044600.2	AT4G08630.1
Potra2n13c26023.1	0.405095531	0.069402725	0.020909588	0.951726178	Potri.013G031100.3	AT1G08830.1
Potra2n13c26024.1	-0.020705723	-0.08109194	0.914495957	0.943483421	Potri.013G031000.1	AT3G05000.1
Potra2n13c26025.1	1.664677056	NA	NA	NA	Potri.013G030900.1	AT4G21120.1
Potra2n13c26026.1	NA	NA	NA	NA	Potri.013G030800.1	AT1G05700.1
Potra2n13c26027.3	-0.61910233	-0.767708831	NA	NA	Potri.013G030100.1	AT1G05700.1
Potra2n13c26028.1					Potri.013G030000.1	AT2G32300.1
Potra2n13c26029.1	NA	1.571529889	NA	NA	Potri.013G030000.1	AT2G32300.1
Potra2n13c26030.1	0.622793752	0.261348693	2.75104E-08	0.630774341	Potri.013G029800.1	AT3G06350.1
Potra2n13c26031.1	0.518562473	0.171357658	0.019254058	0.907073354		

Potra2n13c26032.1	-0.031585778	0.187877779	0.861722736	0.79989661	Potri.013G029500.1	AT3G04980.1
Potra2n13c26033.1	0.374708325	0.28673286	0.253244261	0.874808359	Potri.013G029300.1	AT1G08845.2
Potra2n13c26034.1	0.091328198	0.087950815	0.546185719	0.922681078	Potri.013G029200.1	AT2G32910.1
Potra2n13c26035.1	1.450873726	0.462551766	0.029863942	0.920174237	Potri.013G029100.1	AT1G08860.1
Potra2n13c26036.1	-0.481012335	-0.221267916	0.011288189	0.810774554	Potri.013G029000.1	AT5G27290.1
Potra2n13c26037.1	-0.065428427	-0.265149807	0.825762545	0.794641467	Potri.013G028800.2	AT1G08880.1
Potra2n13c26038.1	-0.076939066	0.201560916	0.696074928	0.917751882	Potri.013G028700.1	AT5G27320.1
Potra2n13c26039.1	0.275877844	-0.085098351	0.127948674	0.951731753	Potri.013G028600.1	AT5G27330.1
Potra2n13c26040.2	-0.097493515	1.668943269	0.965962678	0.814001428	Potri.013G028500.1	AT5G18860.1
Potra2n13c26041.1	-2.639000626	0.043945383	0.175152767	NA	Potri.013G028400.1	
Potra2n13c26042.1	0.69911361	0.673206615	0.03773397	0.636664382	Potri.013G028300.1	AT3G21510.1
Potra2n13c26043.1	-0.352835688	-0.10188361	0.018270745	0.93263929	Potri.013G028200.4	AT1G54710.1
Potra2n13c26044.1	-0.907561528	-0.367101233	0.016891516	0.860810425	Potri.013G028100.1	AT3G05140.1
Potra2n13c26045.1	-1.317292482	-1.465929376	NA	NA		
Potra2n13c26046.1					Potri.013G028000.1	AT2G48010.1
Potra2n13c26047.1	0.60528301	-0.016423385	2.4529E-05	0.99226031	Potri.013G027900.1	AT4G25740.1
Potra2n13c26048.1	-0.787512322	-0.265512242	6.90605E-09	0.674175338	Potri.013G027800.1	AT5G27360.1
Potra2n13c26049.1	-1.026610521	-0.286564458	0.00763169	0.903698951		
Potra2n13c26050.1	0.598119144	0.462703755	0.000528076	0.599212057	Potri.013G027600.1	AT3G49010.3
Potra2n13c26051.1	1.346313459	0.504399586	1.37293E-12	0.600720177	Potri.013G027500.1	AT1G54730.2
Potra2n13c26052.1	0.938885879	0.634943124	3.24536E-17	0.247348782	Potri.013G027300.1	AT4G27190.1
Potra2n13c26053.1	1.381584813	0.032030026	0.023092999	0.996185763	Potri.005G113900.1	AT3G51240.1
Potra2n13c26054.1	0.85359906	-0.484169709	0.630845302	0.954559968	Potri.013G027200.2	AT5G27370.1
Potra2n13c26055.1	2.207366683	0.04011104	2.28064E-06	0.995139072		
Potra2n13c26056.1	2.234284014	0.029004139	6.827E-07	0.996032145	Potri.013G027100.1	AT1G54740.1
Potra2n13c26057.1	0.943350674	-0.767708831	NA	NA	Potri.013G027000.1	AT5G06900.1
Potra2n13c26058.1	-0.259339009	0.267777781	0.619817344	0.935851032	Potri.013G026900.1	AT2G32580.1
Potra2n13c26059.1	0.461392327	0.10386055	0.000809251	0.895575326	Potri.013G026800.1	AT5G27380.1
Potra2n13c26060.1	-0.833652022	-2.42671106	NA	NA	Potri.005G038200.1	
Potra2n13c26061.1	0.650212861	0.086289512	0.00511455	0.974275337	Potri.013G026700.1	AT5G27395.2
Potra2n13c26062.1	0.107368721	0.031873062	0.743239981	0.989329144	Potri.013G026600.1	AT3G05030.1
Potra2n13c26063.2	-0.797648679	-0.185720992	9.65115E-05	0.87062349	Potri.013G026300.1	AT1G54790.3

Potra2n13c26064.1	-0.859905785	-0.216471898	2.4529E-05	0.842247476	Potri.013G026400.1	AT1G54780.1
Potra2n13c26065.1	-1.634637034	-0.404493616	4.84103E-05	0.833667976	Potri.002G216000.1	AT1G54790.3
Potra2n13c26066.1	2.165919294	NA	NA	NA	Potri.005G038900.1	AT3G63480.2
Potra2n13c26067.1	-0.093586272	0.160356177	0.601727217	0.84487051	Potri.013G026100.2	AT2G29150.1
Potra2n13c26068.1	-1.0331284	-0.531600615	0.000183987	0.499664969	Potri.013G025900.1	AT1G72210.1
Potra2n13c26069.1	0.873557667	-0.033452706	2.43153E-07	0.983446961	Potri.013G025800.1	AT3G05200.1
Potra2n13c26070.1	-2.922814453	-3.070419929	NA	NA		
Potra2n13c26071.1	0.481989083	0.017908544	0.153530069	0.995579852	Potri.013G025700.2	AT1G54820.1
Potra2n13c26072.1	-0.250921016	0.001442449	0.05874455	0.999325699	Potri.013G025600.1	AT4G16710.1
Potra2n13c26073.1	-0.245538592	-0.288581084	0.160006665	0.842247476	Potri.013G025400.2	AT5G19080.1
Potra2n13c26074.1	0.901078828	0.254494663	0.813360459	NA		
Potra2n13c26075.1	-0.373150295	-0.18074953	0.134650977	0.86902274	Potri.013G025300.1	AT3G06130.1
Potra2n13c26076.1	-0.364397536	-0.170478243	0.13948642	0.885459527		
Potra2n13c26077.1	-0.341189018	0.20797943	0.004692875	0.850863374	Potri.013G025200.2	AT2G24960.2
Potra2n13c26078.1	-0.007659065	-0.179016845	0.964797847	0.885375133	Potri.013G025100.1	AT1G08960.1
Potra2n13c26079.1	0.173707601	0.018162835	0.032390516	0.979822671	Potri.013G025000.1	AT1G08970.1
Potra2n13c26080.1	0.130659138	0.362053912	0.884189012	0.927547456		
Potra2n13c26081.1	-1.433322345	-0.252959492	9.19829E-07	0.72680202	Potri.013G024900.1	AT1G54840.1
Potra2n13c26082.1	-2.295337707	-1.01793971	NA	NA	Potri.013G024900.1	AT1G54840.1
Potra2n13c26083.1	1.022731501	0.286539366	1.65518E-05	0.744763949	Potri.013G024600.2	AT1G54850.1
Potra2n13c26084.1	-0.893482552	-0.342779288	0.378244164	0.95981322	Potri.013G024500.2	AT5G27440.1
Potra2n13c26085.1	-1.002220165	-0.479236664	0.228362731	0.943483421		
Potra2n13c26086.1	0.22046188	0.269284636	0.446350845	0.912044974	Potri.013G024400.1	AT5G64330.1
Potra2n13c26087.1	0.242968064	0.518979694	0.359023099	0.663037599	Potri.013G024100.3	AT1G08980.1
Potra2n13c26088.1	0.321639225	0.672848813	0.243568174	0.543740237		
Potra2n13c26089.1	0.427284652	0.092252873	0.002278221	0.921065381	Potri.013G024000.1	AT5G27450.2
Potra2n13c26090.1	0.376416563	0.031839092	0.037771677	0.985393296		
Potra2n13c26091.1	-0.104720345	0.11550403	0.351521536	0.880706351	Potri.013G023600.1	AT1G54920.2
Potra2n13c26092.1	4.148168055	2.82956253	0.000185385	0.274923694	Potri.013G023500.1	
Potra2n13c26093.1	-0.030132211	-0.053991152	0.867285642	0.972159498	Potri.013G023400.1	AT5G27490.1
Potra2n13c26094.1	0.844734973	0.143369324	5.75446E-05	0.899157534	Potri.013G023200.1	AT3G05290.1
Potra2n13c26095.1	0.308523705	0.320536479	0.002458461	0.585240923	Potri.013G023100.2	AT5G27540.1

Potra2n13c26096.1	0.401137386	0.461303968	0.005427156	0.628040312	Potri.013G023000.1	AT3G05327.1
Potra2n13c26097.1	NA	2.676364087	NA	NA	Potri.013G022900.2	AT1G08990.1
Potra2n13c26098.1	NA	2.315796285	NA	NA	Potri.013G022800.1	AT3G02100.1
Potra2n13c26099.1	-0.044055089	0.374065473	0.854775094	0.736000077	Potri.013G022700.2	AT1G09000.1
Potra2n13c26100.1	0.188795783	-0.357231317	0.761069366	0.900551258	Potri.013G022600.1	AT3G05330.1
Potra2n13c26101.1	0.863462487	0.333467871	2.69442E-05	0.779493734	Potri.013G022500.1	AT5G62440.1
Potra2n13c26102.1	0.904918448	0.316150546	1.35009E-05	0.817407708		
Potra2n13c26103.1	NA	NA	NA	NA	Potri.013G022350.1	
Potra2n13c26104.1	NA	1.381953902	NA	NA	Potri.013G022350.1	
Potra2n13c26105.1	NA	NA	NA	NA	Potri.015G002900.1	AT1G71930.1
Potra2n13c26106.1	-0.130623558	-0.175952539	0.480107063	0.899720386	Potri.013G022300.1	AT1G55040.1
Potra2n13c26107.1	0.005206938	-0.051396833	0.978607976	0.959009636	Potri.013G022200.1	AT1G67500.1
Potra2n13c26108.2	0.854954974	0.009101469	0.000824045	0.996032145	Potri.013G022000.1	AT1G55020.1
Potra2n13c26109.1	-0.591026009	-0.11182587	0.001377265	0.89372314	Potri.013G021900.1	AT5G55810.1
Potra2n13c26110.1	-0.664738877	-0.10693276	0.02107939	0.910815461		
Potra2n13c26111.1	-0.751616844	-0.272103144	0.007671508	0.841127912	Potri.013G021700.1	AT4G14950.1
Potra2n13c26112.1	0.243947479	-0.1038017	0.020469363	0.862830524	Potri.013G021600.1	AT1G55000.2
Potra2n13c26113.1	-0.151579751	-0.266531885	0.46691421	0.849446235	Potri.013G021500.1	AT1G54990.1
Potra2n13c26114.2	-0.465962789	-0.249734868	0.002078478	0.370539059	Potri.013G021400.1	AT3G05350.1
Potra2n13c26115.1	-0.299387989	0.163430702	0.038949859	0.935851032	Potri.013G021300.2	AT5G27600.1
Potra2n13c26116.2	1.043801313	-0.043881668	1.89055E-05	0.985393296	Potri.001G016900.1	AT5G10400.1
Potra2n13c26117.1	1.290882478	0.285464052	0.000965619	0.922011871	Potri.003G207900.2	AT4G31980.1
Potra2n13c26118.1	1.356060069	0.340019104	3.26413E-20	0.501154172	Potri.013G021200.2	AT1G09010.1
Potra2n13c26119.2	-1.046754602	-0.11809535	4.12882E-09	0.907073354	Potri.013G020900.1	AT3G47570.1
Potra2n13c26120.1	-0.561586806	-0.196073427	0.009373024	0.829862776	Potri.013G020800.1	AT5G27560.1
Potra2n13c26121.1	0.450930206	-0.405648574	0.230760373	0.849815276	Potri.013G020700.1	AT5G27550.1
Potra2n13c26122.1	NA	NA	NA	NA	Potri.013G020600.1	
Potra2n13c26123.4	0.062933206	0.11743762	0.572616882	0.909675463	Potri.013G020500.2	AT3G05380.2
Potra2n13c26124.1	-0.364122203	-0.202124384	0.027201665	0.817452127	Potri.013G020400.2	AT1G09020.1
Potra2n13c26125.1	-0.463357615	-0.217051243	0.000460317	0.829862776	Potri.013G020300.2	AT1G29150.1
Potra2n13c26126.1	2.409567423	2.295459777	NA	NA		
Potra2n13c26127.1	-0.283371488	0.013793663	0.453028057	0.995203254	Potri.013G020200.2	AT4G15730.1

Potra2n13c26128.2	0.92641307	0.64247635	NA	NA	Potri.013G020000.2	AT4G02570.1
Potra2n13c26129.4	-0.45517527	-0.194952087	1.89337E-05	0.658679593	Potri.013G019900.1	AT1G62620.1
Potra2n13c26130.1	0.14071212	0.17668854	0.538496916	0.910815461	Potri.005G027600.1	AT5G64740.1
Potra2n13c26131.1	-0.237096403	0.89255494	0.670983733	0.53266226	Potri.013G019700.1	
Potra2n13c26132.1	1.459962178	1.911450358	NA	NA	Potri.002G079900.1	AT1G21680.1
Potra2n13c26133.1	-0.61910233	-0.767708831	NA	NA	Potri.013G019600.1	AT2G47810.1
Potra2n13c26134.1	1.664677056	2.86450839	NA	NA	Potri.013G019500.1	AT1G09030.1
Potra2n13c26135.1	NA	1.394613675	NA	NA	Potri.013G019400.1	AT3G05390.1
Potra2n13c26136.1	0.317881205	0.065546289	0.083923801	0.969253125	Potri.013G019300.1	AT1G09040.1
Potra2n13c26137.1	0.908192292	0.066230903	1.01527E-05	0.975578749	Potri.013G019200.1	AT3G50700.1
Potra2n13c26138.3	0.841868436	0.246851617	8.19194E-20	0.618582467	Potri.013G019000.1	AT1G09060.3
Potra2n13c26139.1	0.033525427	-0.122475756	0.990387902	NA	Potri.005G027100.2	AT3G05410.2
Potra2n13c26140.1	-0.356080544	-0.149121439	0.0120567	0.916403589	Potri.013G018800.2	AT3G05420.2
Potra2n13c26141.1	-0.092003564	0.097472981	0.480672993	0.929691255	Potri.013G018700.1	AT5G27650.1
Potra2n13c26142.1	0.82279647	0.099340653	4.3986E-07	0.953831244	Potri.013G018600.1	AT1G09070.1
Potra2n13c26143.1	0.200868558	-0.01666133	0.237431798	0.992934677	Potri.013G018400.1	AT3G62770.1
Potra2n13c26144.2	0.223807271	-0.0059121	0.089853751	0.996032145	Potri.013G018300.1	AT5G27660.1
Potra2n13c26145.1	0.259596199	-0.788501251	0.804752836	0.857189843	Potri.013G018100.1	AT5G48670.1
Potra2n13c26146.1	NA	NA	NA	NA		
Potra2n13c26147.1	0.276384944	0.754463278	0.9309217	NA	Potri.013G018000.1	AT5G28540.1
Potra2n13c26148.1	-0.919193531	-0.061044971	0.073924381	0.98409016	Potri.013G017900.1	AT5G67470.1
Potra2n13c26149.1	1.701581667	0.775917782	2.18772E-06	0.421571437	Potri.013G017700.1	AT5G27690.1
Potra2n13c26150.1	0.835369159	0.1461828	1.58443E-07	0.896353214	Potri.005G026000.1	AT5G27700.1
Potra2n13c26151.1	0.814617719	0.123626244	1.58502E-06	0.924951347		
Potra2n13c26152.1	-0.090323616	0.088083701	0.682531888	0.956889095	Potri.013G017500.2	AT3G05480.1
Potra2n13c26153.1	-0.528682536	-0.769859267	0.008952214	0.02130794	Potri.013G017400.1	AT1G02900.1
Potra2n13c26154.1	-1.446278711	0.297946469	0.000334673	0.858174358	Potri.013G017300.1	AT3G05500.1
Potra2n13c26155.1	-3.304361185	-0.782130747	NA	NA	Potri.T171300.1	AT5G15500.2
Potra2n13c26156.1	0.108735726	0.105470461	0.249805352	0.894138111	Potri.013G017166.1	AT1G09130.3
Potra2n13c26157.1	-0.38975795	-0.243375446	0.008293129	0.713370907	Potri.013G017000.1	AT3G05520.2
Potra2n13c26158.1	0.210985048	0.597067737	0.307032608	0.46223393	Potri.013G016900.2	AT5G27730.1
Potra2n13c26159.1	0.150671596	0.154263731	0.175976558	0.794521408	Potri.013G016800.1	AT3G05530.1

Potra2n13c26160.1	0.18840196	0.156269292	0.136712395	0.773063484		
Potra2n13c26161.1	0.435202247	0.186020973	0.005944513	0.902857376	Potri.013G016600.2	AT4G27190.1
Potra2n13c26162.1	2.302046585	1.794168371	0.221797313	NA	Potri.004G078300.2	AT1G17880.1
Potra2n13c26163.2	-0.205646885	-0.095773126	0.08532689	0.842247476	Potri.013G015600.1	AT3G05545.1
Potra2n13c26164.1	0.021224901	0.399182219	0.885208399	0.586851629	Potri.013G015500.2	AT1G09140.1
Potra2n13c26165.1	0.525699683	0.038438625	5.17548E-05	0.974789803	Potri.013G015400.2	AT3G05550.1
Potra2n13c26166.1	0.597999007	0.049526078	0.000146962	0.965608095	Potri.013G015300.1	AT3G05560.1
Potra2n13c26167.1	NA	NA	NA	NA	Potri.013G015200.1	AT1G28590.1
Potra2n13c26168.1	-0.25802634	-0.186577181	0.047216423	0.817452127	Potri.013G015000.1	AT3G25940.1
Potra2n13c26169.1	-0.39485256	-0.608819194	0.461543759	0.540010302	Potri.013G015100.2	AT1G71160.1
Potra2n13c26170.1	-0.478238311	0.098576805	0.042675414	0.956865545		
Potra2n13c26171.1	0.238064872	0.080667009	0.071858586	0.920174237	Potri.013G014800.1	AT5G27830.1
Potra2n13c26172.1	-0.330288521	-0.098229069	0.03914881	0.935474933	Potri.013G014700.1	AT3G05580.1
Potra2n13c26173.1	-0.459725858	-0.138592524	0.013281876	0.916425544	Potri.013G014600.1	AT1G02816.1
Potra2n13c26174.1	NA	NA	NA	NA	Potri.013G014500.1	AT4G15920.1
Potra2n13c26175.1	-0.23052471	-0.207085642	0.364440388	0.93263929	Potri.013G014400.1	AT4G15920.1
Potra2n13c26176.1	-1.926895505	-0.882143343	0.039039842	0.872398633		
Potra2n13c26177.1	0.220445124	0.654442025	0.077091693	0.407558142		
Potra2n13c26178.1	7.729515862	-0.165107148	3.96983E-20	0.985393296	Potri.013G013900.1	AT4G15920.1
Potra2n13c26179.1	5.287377147	2.542125408	3.19014E-65	NA	Potri.013G013800.1	AT4G15920.1
Potra2n13c26180.1	0.527117065	0.042672138	0.000921719	0.974367223	Potri.013G013700.1	AT1G09150.1
Potra2n13c26181.1	-0.830450468	-0.421819594	0.202724953	0.896042644	Potri.013G013500.1	AT3G05600.1
Potra2n13c26182.1	0.342566415	-0.608696511	0.87014555	NA	Potri.013G013400.1	AT3G05610.1
Potra2n13c26183.2	-0.891180632	-0.362984474	8.18846E-06	0.669901617	Potri.005G022600.1	AT5G27920.1
Potra2n13c26184.1	-0.851519801	-0.267263258	8.76918E-14	0.72672395	Potri.013G013000.1	AT1G56230.1
Potra2n13c26185.1	0.167271241	-0.366186183	0.922210786	NA	Potri.005G022250.1	
Potra2n13c26186.2	0.82357019	NA	NA	NA	Potri.013G012766.1	AT4G24640.1
Potra2n13c26187.1	0.142778378	-0.147455883	0.81069053	0.966917036	Potri.013G012600.1	AT1G09155.1
Potra2n13c26188.1	0.10759011	-0.109604678	0.429243192	0.885375133	Potri.013G012500.1	AT5G12210.1
Potra2n13c26189.1	-1.137210927	-0.269362555	1.38077E-06	0.869186333	Potri.013G012300.1	AT3G05630.1
Potra2n13c26190.1	-0.397737762	-0.593879533	0.182410944	0.586851629	Potri.013G012200.2	AT5G27930.2
Potra2n13c26191.1	0.693327834	-0.089001144	0.786532542	NA		

Potra2n13c26192.1	-0.080598057	0.219240553	0.555636297	0.759797728	Potri.013G012100.1	AT5G27030.1
Potra2n13c26193.1	-0.574290414	-0.307071555	1.73997E-05	0.322083088	Potri.013G012000.1	AT2G40650.1
Potra2n13c26194.1	-1.289380312	-0.486506994	1.64635E-06	0.137620511	Potri.005G021500.1	AT5G27020.1
Potra2n13c26195.1	0.220726503	0.023211952	0.002460946	0.975578749	Potri.013G011900.2	AT4G11420.1
Potra2n13c26196.1	-0.049540454	-0.213442099	0.834041617	0.893394174	Potri.013G011800.1	AT4G11060.1
Potra2n13c26197.1	-0.6970908	-0.250408762	0.022415946	0.833667976	Potri.013G011600.2	AT1G09160.2
Potra2n13c26198.1	-0.546526591	0.883725658	0.205665413	0.573728088	Potri.013G011500.1	AT5G27000.1
Potra2n13c26199.1	0.248704252	0.150898866	0.035837939	0.829862776	Potri.013G011400.1	AT3G05670.1
Potra2n13c26200.2	0.116128561	0.344828522	0.733147069	0.842247476	Potri.013G011200.1	AT3G05700.1
Potra2n13c26201.1	2.943863668	1.14138789	0.005171144	NA	Potri.013G011300.1	AT2G20710.1
Potra2n13c26202.1	-0.271208104	-0.124156451	0.071360457	0.920028955	Potri.013G011100.1	AT3G05710.2
Potra2n13c26203.1	0.940074289	0.118601812	8.73827E-08	0.922993749	Potri.013G011000.1	AT5G07090.1
Potra2n13c26204.2	0.204210317	-0.001706596	0.031205869	0.999282348	Potri.013G010900.1	AT3G06720.1
Potra2n13c26205.1	-0.71865271	-0.294512447	2.41039E-11	0.862830524	Potri.013G010800.2	AT1G56300.1
Potra2n13c26206.1	1.569823039	0.353463093	2.60987E-24	0.663950227	Potri.013G010700.2	AT4G25800.2
Potra2n13c26207.1	0.647799923	-0.058470081	0.122868674	0.982827151	Potri.013G010600.1	AT3G05725.1
Potra2n13c26208.1	-0.976276243	-0.262761022	7.93207E-06	0.743612955	Potri.013G010500.1	AT5G26910.1
Potra2n13c26209.1	-1.139171219	0.310049948	0.036827632	0.920141992	Potri.013G010400.1	AT3G62630.1
Potra2n13c26210.1	-0.267104671	-0.079429327	0.015368953	0.93154079	Potri.013G010300.1	AT3G05760.1
Potra2n13c26211.1	0.82357019	NA	NA	NA	Potri.013G010200.1	
Potra2n13c26212.1	-0.160350035	0.005210546	0.086654965	0.996032145	Potri.013G009900.1	AT4G02080.1
Potra2n13c26213.1	1.092830501	0.057454196	9.25173E-07	0.976302215	Potri.013G009800.1	AT3G62550.1
Potra2n13c26214.1	0.805402595	2.207342265	0.30177599	0.261662674	Potri.013G009700.1	AT2G47430.1
Potra2n13c26215.1	0.404795194	0.15278742	0.004341542	0.888366602	Potri.013G009500.2	AT1G09210.1
Potra2n13c26216.1	1.481248937	0.267063686	0.001204516	0.929435576	Potri.013G009400.1	AT1G15690.1
Potra2n13c26217.1	0.095297124	-0.084601916	0.580767827	0.941115972	Potri.013G009200.1	AT1G56350.1
Potra2n13c26218.1	-0.143643776	-0.427822352	0.694534583	0.71102669	Potri.013G009300.1	AT1G56345.1
Potra2n13c26219.1	0.391193305	-0.014595368	0.416902801	0.996292992		
Potra2n13c26220.1	0.30328251	0.10915064	0.047815245	0.894138111	Potri.013G009100.1	AT1G56420.1
Potra2n13c26221.1	0.840012491	1.421908496	0.366266264	0.679485917	Potri.013G009000.1	AT2G24100.1
Potra2n13c26222.1	0.310754665	0.022126261	0.059658722	0.990390312	Potri.013G008900.1	AT5G26860.1
Potra2n13c26223.1	-2.921912828	-3.071566347	NA	NA	Potri.013G008800.1	AT1G67623.1

Potra2n13c26224.1	-0.134790238	-0.072790142	0.498935475	0.954419252	Potri.013G008700.1	AT1G56423.1
Potra2n13c26225.1	0.656277327	0.196393181	7.99816E-08	0.794596445	Potri.013G008600.1	AT1G23380.1
Potra2n13c26226.1	-1.050919156	-0.304921868	3.41482E-08	0.489910701	Potri.013G008500.1	AT5G26850.2
Potra2n13c26227.1	-0.232745801	-0.129023765	0.119179043	0.849815276	Potri.013G008400.2	AT1G56440.1
Potra2n13c26228.1	-0.752921692	-0.130793402	1.71419E-08	0.867960518	Potri.013G008300.2	AT1G56460.1
Potra2n13c26229.2	-1.27237456	-0.502288556	0.00028615	0.531832321	Potri.013G008200.1	AT1G74150.1
Potra2n13c26230.1	NA	0.674963689	NA	NA	Potri.013G008100.1	AT5G18990.1
Potra2n13c26231.1	-3.21389583	0.469131384	NA	NA	Potri.013G008000.1	AT5G48910.1
Potra2n13c26232.1	-1.537265238	0.115792943	1.78755E-05	0.971828097	Potri.013G007900.1	
Potra2n13c26233.1	-0.277406865	-0.088616061	0.072259431	0.926808461	Potri.013G007800.5	AT1G56500.1
Potra2n13c26234.1	0.144313296	0.324551239	0.566329321	0.892986206	Potri.013G007700.1	AT1G09250.1
Potra2n13c26235.1	-0.026747708	-0.256979718	0.991034693	NA		
Potra2n13c26236.1	0.172450762	0.268746624	0.426247923	0.718626343	Potri.013G007600.1	AT5G26800.1
Potra2n13c26237.1	-0.870849692	0.028266394	0.004292051	0.989304496	Potri.013G007500.1	AT5G49525.1
Potra2n13c26238.1	0.551326896	0.393835243	0.739719898	NA	Potri.013G007401.1	AT5G63020.1
Potra2n13c26239.1	-0.017341076	-0.046682202	0.906996136	0.958152146	Potri.013G007300.1	AT1G09270.1
Potra2n13c26240.1	-0.228869147	0.109404636	0.305736559	0.926234768	Potri.005G011900.1	AT1G09280.1
Potra2n13c26241.1	0.105272507	0.091747641	0.594833634	0.936890993	Potri.013G007100.1	AT1G09300.1
Potra2n13c26242.1	-1.132425712	-0.588599805	0.031299821	0.725673516	Potri.013G007000.1	AT1G56580.1
Potra2n13c26243.1	-0.131292324	-0.08383614	0.695325916	0.960656795	Potri.013G006900.2	AT1G09320.1
Potra2n13c26244.1	0.109831989	-0.015937078	0.764050412	0.995579852	Potri.013G006700.3	AT1G56570.1
Potra2n13c26245.1	1.457795346	0.244907311	3.67133E-08	0.84487051	Potri.013G006600.1	AT1G56560.1
Potra2n13c26246.1	-0.312943286	0.191941757	0.176091056	0.880316339	Potri.013G006500.1	AT4G15510.1
Potra2n13c26247.1	-0.353733898	-0.091048571	0.027838868	0.923510751	Potri.013G006400.1	AT1G56590.1
Potra2n13c26248.1	-0.157046729	0.047463846	0.274753451	0.976302215	Potri.013G006250.1	AT1G09330.1
Potra2n13c26249.1	-2.702692642	-0.523409233	4.60135E-15	0.692084498	Potri.013G006100.1	AT1G09340.1
Potra2n13c26250.1	NA	NA	NA	NA		
Potra2n13c26251.1	-0.040592448	0.260192529	0.827129585	0.677013336	Potri.013G006000.1	AT1G08060.1
Potra2n13c26252.1	1.469724287	1.200607322	0.061826396	0.521534461	Potri.013G005800.1	AT1G56600.1
Potra2n13c26253.1	NA	0.674963689	NA	NA		
Potra2n13c26254.1	0.669298453	-0.145717815	0.826238817	NA	Potri.005G007100.1	AT1G09360.1
Potra2n13c26255.1	0.465811511	0.388927586	0.467511396	0.701494362	Potri.013G005700.1	AT4G25810.1

Potra2n13c26256.2	-0.159132454	-0.086166198	0.138881303	0.930598202	Potri.005G007300.1	AT1G10630.1
Potra2n13c26257.1	-0.460841198	-0.118764029	0.041127406	0.938246513	Potri.013G005400.1	AT1G56690.1
Potra2n13c26258.1	-0.48720675	-0.133777839	0.056090618	0.933532804	Potri.013G005300.3	
Potra2n13c26259.1	-0.38942428	-0.089372673	0.014613781	0.953768643	Potri.013G005200.1	AT1G09420.1
Potra2n13c26260.1	-0.213703543	-0.047079275	0.162460783	0.95671968	Potri.013G005100.1	AT1G56700.1
Potra2n13c26261.1	-3.144789355	-0.769489589	NA	NA	Potri.013G005000.1	AT1G02460.1
Potra2n13c26262.1	-0.301667578	-2.013568191	NA	NA	Potri.013G004901.1	AT2G29940.1
Potra2n13c26263.1	0.252657818	-0.172234004	0.359056594	0.900551258	Potri.013G004801.1	AT5G26749.1
Potra2n13c26264.1	-0.185027861	-0.00391644	0.059294237	0.995579852	Potri.013G004600.1	AT4G10710.1
Potra2n13c26265.1	-0.294812906	-0.039423107	0.002223476	0.949847686	Potri.013G004500.1	AT4G10710.1
Potra2n13c26266.1	-0.155294725	-0.050403783	0.366714932	0.977559521	Potri.013G004400.1	AT1G09430.1
Potra2n13c26267.1	-0.17492865	0.048980677	0.225987546	0.956889095	Potri.013G004200.1	AT3G62240.1
Potra2n13c26268.1	0.005031709	-0.287535568	0.987935873	0.902857376	Potri.013G004100.1	AT3G17390.1
Potra2n13c26269.1	1.931605898	NA	NA	NA	Potri.013G004000.1	
Potra2n13c26270.1	1.373435974	0.87143929	NA	NA	Potri.013G003850.1	
Potra2n13c26271.1	NA	NA	NA	NA	Potri.013G003900.1	
Potra2n13c26272.1	3.013298587	NA	NA	NA	Potri.013G003850.1	
Potra2n13c26273.1	-0.347826908	0.235595179	0.151383044	0.912777843	Potri.013G003700.2	AT1G56720.3
Potra2n13c26274.1	-0.010810793	-0.29066815	0.974164289	0.901376543	Potri.013G004100.1	AT3G17390.1
Potra2n13c26275.1	1.262947684	-0.786502229	NA	NA		
Potra2n13c26276.1	NA	NA	NA	NA	Potri.013G003900.1	
Potra2n13c26277.6	-0.385291083	0.232148788	0.198170716	0.919986989	Potri.013G003700.2	AT1G56720.3
Potra2n13c26278.1	-0.628027531	-0.635542548	0.304761116	0.790362046	Potri.005G003400.2	AT1G09450.1
Potra2n13c26279.1	-0.433650584	-0.702322175	0.766141118	0.91753191		
Potra2n13c26280.3	-1.306352696	-0.421777786	1.2543E-05	0.663037599	Potri.013G003500.2	AT4G26830.1
Potra2n13c26281.1	-0.461466818	0.258791179	0.036820973	0.80340893	Potri.013G003400.1	AT5G26770.2
Potra2n13c26282.1	-0.372213513	-0.056476743	0.290741925	0.983446961	Potri.013G003200.1	AT3G62200.1
Potra2n13c26283.1	0.025976975	0.011992243	0.903891012	0.995005375	Potri.013G003200.1	AT3G62200.1
Potra2n13c26284.1	0.373482102	0.071589559	0.126546372	0.960981446	Potri.013G003000.1	AT5G61190.1
Potra2n13c26285.1	0.409444454	0.149516942	0.000555883	0.82876584	Potri.013G003000.1	AT5G61190.1
Potra2n13c26286.1	1.64774763	NA	NA	NA		
Potra2n13c26287.1	-0.053714538	0.293367011	0.799668664	0.751233909	Potri.013G002900.1	

Potra2n13c26288.1	-0.353296308	0.045530134	0.005939449	0.956865545	Potri.013G002700.1	AT5G60190.1
Potra2n13c26289.2	-0.386571524	-0.024050445	0.000135502	0.981401592		
Potra2n13c26290.1	-0.43107413	0.018688124	4.82413E-05	0.987960246	Potri.013G002400.1	AT5G26600.1
Potra2n13c26291.1	NA	NA	NA	NA	Potri.005G002900.3	AT1G57540.2
Potra2n13c26292.1	5.344656066	1.205764701	4.33604E-50	0.643173103	Potri.013G002200.1	AT1G11910.1
Potra2n13c26293.1	0.048540897	0.162960682	0.736327815	0.792544431	Potri.013G002100.1	AT5G26610.3
Potra2n13c26294.1	NA	NA	NA	NA	Potri.013G001800.1	AT3G05858.1
Potra2n13c26295.1	3.325944336	3.036481957	NA	NA	Potri.013G001800.1	AT3G05858.1
Potra2n13c26296.1	NA	0.674963689	NA	NA	Potri.013G001800.1	AT3G05858.1
Potra2n13c26297.1	NA	NA	NA	NA	Potri.013G001700.1	AT5G48670.1
Potra2n13c26298.1	-0.3184355	0.154354675	0.312396414	0.87062349	Potri.013G001600.1	AT3G05880.1
Potra2n13c26299.1	NA	NA	NA	NA		
Potra2n13c26300.1	NA	NA	NA	NA		
Potra2n13c26301.1	-0.147566051	0.1732078	0.543628138	0.900551258	Potri.013G001300.2	AT1G09530.1
Potra2n13c26302.1	-0.656333743	0.301582785	5.23713E-05	0.704368358	Potri.013G001150.1	AT3G05900.1
Potra2n13c26303.2	-2.422059795	-0.795892565	1.416E-13	0.340356592	Potri.013G001000.1	AT1G09540.1
Potra2n13c26304.1	-1.501938539	-0.31586731	1.50613E-06	0.885143969	Potri.013G000900.1	AT3G05910.1
Potra2n13c26305.1	0.093652908	0.047232951	0.674075675	0.972159498	Potri.013G000700.1	AT5G26680.2
Potra2n13c26306.1	-0.116387876	0.087918808	0.454209448	0.923746681	Potri.013G000600.1	AT1G57600.1
Potra2n13c26307.1	3.023575298	0.317915326	3.32817E-25	0.925184207	Potri.013G000500.1	AT1G09560.1
Potra2n13c26308.1	0.043021743	0.137053682	0.78282403	0.817452127	Potri.013G000300.2	AT1G09570.1
Potra2n13c26309.1	0.364864177	0.170320664	0.339930742	0.912642491	Potri.013G000200.2	AT5G26720.1
Potra2n13c26310.1	-0.313604642	-0.416586685	0.899483173	NA	Potri.013G000100.1	AT3G05936.1
Potra2n13c26311.1	-0.396439884	0.041746881	0.000402748	0.975313758	Potri.005G000600.1	AT3G05940.1
Potra2n1404s37046.1						
Potra2n1405s37047.1	1.760605721	3.451544688	0.083823665	0.125577936	Potri.001G473200.1	AT1G55580.1
Potra2n1406s37048.1	0.08761141	0.084293533	0.96219785	0.992934677	Potri.006G271720.1	
Potra2n1406s37049.1	-2.00128719	-0.520166048	0.003936606	0.914321114	Potri.006G271800.2	AT3G14470.1
Potra2n1407s37050.1	-0.234827422	-0.096759051	0.363631332	0.937789963	Potri.003G008400.1	AT1G09810.1
Potra2n1407s37051.1	NA	NA	NA	NA		
Potra2n1408s37052.1	5.968558393	3.25175354	3.06628E-50	0.054304482	Potri.005G007900.1	AT1G07390.3
Potra2n1412s37053.1						

Potra2n1412s37054.1					Potri.004G117600.1	
Potra2n1412s37055.1						
Potra2n1413s37056.1	-0.055610439	0.164279929	0.884628951	0.901251375	Potri.011G130000.1	AT5G54540.1
Potra2n1414s37057.1	-0.928118206	0.060609181	0.056982266	0.989304496		
Potra2n1414s37058.1	0.971004094	-0.128407294	0.510139706	NA	Potri.015G053700.1	AT1G73890.1
Potra2n1415s37059.1	-2.375978331	-2.523469902	NA	NA	Potri.004G008000.1	AT4G22030.1
Potra2n1416s37060.1	1.545832463	NA	NA	NA	Potri.005G009300.1	AT1G07390.3
Potra2n1416s37061.1	2.429009245	-0.137073329	0.047891557	NA	Potri.001G063400.1	
Potra2n1417s37062.1	-0.405233101	0.180597761	0.194446774	0.936402128	Potri.015G007700.1	AT4G27680.1
Potra2n1418s37063.1	-0.368232725	-0.086193357	0.068198903	0.951718113	Potri.001G043700.1	AT1G80870.1
Potra2n1422s37064.1	NA	NA	NA	NA	Potri.009G132000.1	AT4G36020.1
Potra2n1424s37065.1					Potri.006G107800.1	AT2G38510.1
Potra2n1425s37066.1	-1.539408836	-1.610946392	NA	NA	Potri.006G050900.3	AT3G57440.1
Potra2n1428s37067.1	2.473142592	2.005962485	2.61197E-09	0.000146941	Potri.013G133800.1	AT1G03670.1
Potra2n1429s37068.1					Potri.004G232250.1	AT5G49760.1
Potra2n1432s37069.1					Potri.007G039300.1	AT5G66900.1
Potra2n1433s37070.1	6.839538272	1.381953902	8.70487E-07	NA	Potri.T080900.1	AT1G16260.2
Potra2n1434s37071.1	NA	NA	NA	NA		
Potra2n1435s37072.1	0.607830769	0.136259982	4.31002E-05	0.856379924	Potri.004G125900.1	AT1G69360.1
Potra2n1435s37073.1	0.49318585	0.13736422	0.405572062	0.974264269	Potri.004G126000.1	AT3G16520.3
Potra2n1436s37074.1	0.051317103	0.297355315	0.963428414	0.948577865	Potri.014G003600.3	AT3G14460.1
Potra2n1440s37075.1	0.594929425	0.513538446	0.05890345	0.607924647		
Potra2n1443s37076.1					Potri.004G135300.1	AT4G15093.1
Potra2n1445s37077.1	NA	NA	NA	NA		
Potra2n1453s37078.1	0.77226113	-1.416318346	0.691424548	NA	Potri.018G135800.1	AT3G14460.1
Potra2n1453s37079.4	-0.903335501	-0.454277095	0.203663558	0.88661378	Potri.018G135800.1	AT3G14460.1
Potra2n1458s37080.1					Potri.008G001900.1	AT2G01050.1
Potra2n1458s37081.1					Potri.019G047900.1	AT2G01050.1
Potra2n1459s37082.1						
Potra2n1460s37083.1	5.918030443	2.86450839	2.39522E-05	NA	Potri.T023200.1	AT4G27290.1
Potra2n1463s37084.1					Potri.001G183900.1	
Potra2n1463s37085.1	3.443068432	1.571529889	NA	NA	Potri.007G089300.1	AT2G01050.1

Potra2n1465s37086.1	NA	NA	NA	NA		
Potra2n1467s37087.1						
Potra2n1468s37088.1	1.201339976	-1.478536539	NA	NA	Potri.T098100.3	AT4G21390.1
Potra2n1468s37089.1					Potri.001G441700.1	AT5G18470.1
Potra2n1469s37090.1	8.965987043	7.960308354	9.78315E-12	0.334090819	Potri.001G462000.1	AT5G44440.1
Potra2n1470s37091.1	2.905035641	1.13729403	0.003150189	NA	Potri.011G104900.1	AT1G71400.1
Potra2n1474s37092.1	-0.947943101	-0.112885789	0.001842441	0.953831244	Potri.004G162800.3	AT2G21300.2
Potra2n1475s37093.1	NA	NA	NA	NA		
Potra2n1476s37094.1	0.851175553	0.160886512	5.7952E-07	0.830470159	Potri.003G185600.1	AT1G79690.1
Potra2n1481s37095.1					Potri.019G004232.1	
Potra2n1481s37096.3	3.062889694	-1.465929376	0.173995315	NA	Potri.014G034000.1	AT2G26560.1
Potra2n1483s37097.1	-0.756996334	0.273081949	0.025192705	0.817452127	Potri.006G271800.2	AT3G14470.1
Potra2n1484s37098.1					Potri.001G362700.1	AT5G25120.1
Potra2n1487s37099.1						
Potra2n1488s37100.1					Potri.017G133950.1	AT2G26560.1
Potra2n148s34720.1	NA	NA	NA	NA		
Potra2n148s34721.1	-1.747702926	-0.396154016	1.628E-07	0.824378527	Potri.011G015500.1	AT4G22190.1
Potra2n148s34722.1	-0.402802376	0.004729722	0.249805297	0.999121696		
Potra2n148s34723.3	-0.114237953	0.199051324	0.539010782	0.824905557	Potri.011G013900.2	AT1G27170.1
Potra2n148s34724.1	1.545832463	3.145099921	NA	NA	Potri.004G004000.1	AT4G04180.1
Potra2n148s34725.1	-1.102228906	-0.271119212	0.043813721	0.922681078		
Potra2n148s34726.1	-0.782560864	0.20689197	0.46775709	0.953831244		
Potra2n148s34727.1	1.036899875	-0.062934707	0.000164616	0.983446961	Potri.006G269900.2	AT5G36930.2
Potra2n148s34728.1	-0.892000483	-0.069198394	0.030144267	0.98036348	Potri.T038700.1	
Potra2n148s34729.1	-0.774502088	0.34075835	0.092454205	0.897389232	Potri.011G013300.1	AT4G04670.1
Potra2n148s34730.2	0.559944354	0.208593105	0.001395311	0.759797728	Potri.011G015400.3	AT1G27170.1
Potra2n148s34731.1	0.203188763	0.110081123	NA	NA	Potri.006G270100.1	AT4G22190.1
Potra2n148s34732.1	-0.634862229	0.182811084	0.043636362	0.917892599		
Potra2n148s34733.1	-0.19589933	-0.298142273	0.668595828	0.900884796	Potri.T068200.1	
Potra2n148s34734.1	2.966442973	3.77685348	NA	NA	Potri.011G012300.1	AT5G13950.3
Potra2n148s34735.1	-3.491755887	-0.110389825	0.163400177	NA	Potri.011G012201.1	
Potra2n148s34736.1	-3.503044764	-2.195846806	0.161638262	NA	Potri.011G014800.1	AT1G56520.2

Potra2n148s34737.1	-0.439871846	0.005066259	0.380613546	0.999325699	Potri.011G013800.1	AT5G41540.1
Potra2n1490s37101.1	0.499541158	0.341290965	0.136680528	0.817452127	Potri.001G013300.1	AT1G73240.1
Potra2n1491s37102.1	-0.25787777	-0.134065307	0.267417138	0.924256651	Potri.015G000100.1	AT4G27870.1
Potra2n1491s37103.1	NA	NA	NA	NA	Potri.008G001900.1	AT2G01050.1
Potra2n1491s37104.1						
Potra2n1493s37105.1	-0.106532183	0.002969772	0.712843138	0.999325699	Potri.010G014800.1	AT3G06330.1
Potra2n1494s37106.1	0.566177747	0.707362006	0.645722222	0.880870972	Potri.001G425700.1	
Potra2n1495s37107.1	0.897820983	0.608276733	0.278303011	0.892570555	Potri.003G026900.1	AT3G14460.1
Potra2n1495s37108.1	NA	NA	NA	NA	Potri.003G013400.1	
Potra2n1498s37109.1	-0.443362028	0.033084452	0.032390516	0.993562283	Potri.001G374100.2	AT5G54080.1
Potra2n1499s37110.1	9.400430635	5.735111313	3.45132E-17	0.351105764	Potri.004G026100.1	AT4G23180.1
Potra2n14c26312.1	-0.001734939	0.220704146	0.993567031	0.679365931	Potri.014G000500.1	AT1G16710.1
Potra2n14c26313.1	0.005830566	0.044113802	0.967126058	0.966704706	Potri.014G000600.1	AT1G79020.1
Potra2n14c26314.1	-0.996512015	-0.087526194	1.43124E-06	0.914162682	Potri.014G000700.1	AT3G13040.1
Potra2n14c26315.1	-0.344445643	-0.132628938	0.097354398	0.950878725	Potri.014G000800.1	AT3G13050.1
Potra2n14c26316.1	-3.297168727	-2.727091928	NA	NA	Potri.015G079000.1	AT3G48200.1
Potra2n14c26317.2	0.292614437	0.195401448	0.229824918	0.880095503	Potri.014G000900.1	AT5G63010.1
Potra2n14c26318.1	0.103110571	0.081236812	0.239395257	0.923992681	Potri.014G001000.1	AT3G13060.2
Potra2n14c26319.2	-0.360576573	0.839109167	0.11696655	0.621832412	Potri.014G001200.1	AT1G78980.1
Potra2n14c26320.3	1.689350051	0.757514584	1.23365E-11	0.098336438	Potri.014G002400.1	AT1G78950.1
Potra2n14c26321.1	0.634279584	-0.309564319	0.661493119	0.974275337		
Potra2n14c26322.1	-0.246207276	-0.012082273	0.727804654	0.998027171	Potri.014G001500.1	AT1G78955.1
Potra2n14c26323.1	-0.022440119	-0.038039975	0.944211339	0.987226805	Potri.014G001500.1	AT1G78955.1
Potra2n14c26324.1	-0.112206474	0.018003048	0.782604797	0.995579852	Potri.014G001500.1	AT1G78955.1
Potra2n14c26325.1	NA	1.394613675	NA	NA	Potri.014G002700.1	AT3G20190.1
Potra2n14c26326.1	0.089321189	0.395151364	0.822811449	0.821266061	Potri.014G011200.1	
Potra2n14c26327.1	0.07549602	0.383789521	0.853184672	0.823422145	Potri.014G001700.2	AT1G16760.1
Potra2n14c26328.1	-0.133088465	0.025093145	0.513295865	0.984292161	Potri.014G003600.3	AT3G14460.1
Potra2n14c26329.1	0.495163196	0.643274046	0.779991719	NA	Potri.007G005500.1	AT5G67610.1
Potra2n14c26330.1	0.42526619	-3.555211762	0.776301813	NA	Potri.014G003100.1	
Potra2n14c26331.1	0.302903983	-1.81149025	0.804863346	NA		
Potra2n14c26332.1	0.187073384	0.099601257	0.316924886	0.947969749	Potri.004G169800.1	AT3G14460.1

Potra2n14c26333.1	0.493973306	0.332155985	0.036636029	0.651550434	Potri.014G003200.1	AT3G14460.1
Potra2n14c26334.1	-1.266300433	-0.299022615	0.341030069	0.962661188	Potri.014G003100.1	
Potra2n14c26335.1	-1.222659825	-0.41291426	NA	NA	Potri.014G009300.2	AT3G14470.1
Potra2n14c26336.1	0.102236182	-0.046370319	NA	NA		
Potra2n14c26337.1	-0.61910233	0.673278781	NA	NA	Potri.007G000900.1	AT5G23420.1
Potra2n14c26338.1	NA	1.381953902	NA	NA		
Potra2n14c26339.1	0.620817025	-1.342115412	0.551196196	0.826906752	Potri.004G170600.1	
Potra2n14c26340.1	NA	NA	NA	NA		
Potra2n14c26341.1	-0.077376223	0.089502406	0.88057189	0.974275337	Potri.004G169800.1	AT3G14460.1
Potra2n14c26342.1	-0.96834082	0.078056748	0.01336134	0.978507994	Potri.014G011800.1	
Potra2n14c26343.1	NA	1.394613675	NA	NA	Potri.003G072800.1	AT3G14310.1
Potra2n14c26344.1	-0.32752381	-0.073809437	0.055910195	0.937276488	Potri.007G000300.1	AT3G20020.1
Potra2n14c26345.3	0.264110212	-0.082831179	0.045592144	0.952773082	Potri.014G004100.1	AT3G20000.1
Potra2n14c26346.1	1.143269557	3.016290051	0.472492884	0.342638993	Potri.014G004500.1	AT2G22620.1
Potra2n14c26347.1	0.260375444	-0.037980126	0.064573525	0.98036348	Potri.014G010100.1	AT5G65620.1
Potra2n14c26348.1	-0.766971455	-0.059612309	0.002994871	0.985393296	Potri.014G004700.1	AT2G22640.1
Potra2n14c26349.1	1.721135608	0.597495575	1.01312E-09	0.614953826	Potri.014G004800.1	AT5G67570.1
Potra2n14c26350.2	-0.077634888	0.040330535	0.52713045	0.970992968	Potri.014G004900.1	AT5G67580.1
Potra2n14c26351.4	0.24578416	0.060059641	0.062403375	0.94795983	Potri.014G005000.1	AT4G37670.2
Potra2n14c26352.1	1.545832463	1.381953902	NA	NA	Potri.007G005500.1	AT5G67610.1
Potra2n14c26353.1	-0.065662381	-0.110435767	0.718917385	0.942105753	Potri.014G005100.1	ATCG00540.1
Potra2n14c26354.1	-0.217474878	0.332631515	0.58076009	0.875641095	Potri.014G007600.2	AT3G14460.1
Potra2n14c26355.1	0.363288261	0.290009498	0.735147089	0.956865545	Potri.004G169800.1	AT3G14460.1
Potra2n14c26356.2	-0.44390328	0.441481262	0.016600234	0.524773399	Potri.007G000700.2	AT1G50410.1
Potra2n14c26357.1	0.029831357	0.959784316	0.985256748	0.860001323		
Potra2n14c26358.1	2.262610718	-0.442542371	0.0009762	0.949966783	Potri.014G005900.1	AT1G70210.1
Potra2n14c26359.1	-0.282443562	-0.108234957	0.031941697	0.914554682	Potri.014G006000.1	AT4G37680.2
Potra2n14c26360.1	2.121464847	0.536070109	3.00029E-15	0.805451998	Potri.014G006100.1	AT2G22900.1
Potra2n14c26361.1	2.276581012	0.545329991	2.88732E-12	0.835819291		
Potra2n14c26362.1	3.49831717	1.147179208	0.000948876	NA	Potri.014G006300.1	AT1G58420.1
Potra2n14c26363.1	-1.776682053	-3.075434499	0.671773205	NA	Potri.014G006400.2	AT2G22880.1
Potra2n14c26364.1	0.569769105	-0.04095103	0.156228786	0.991407187		

Potra2n14c26365.1	-0.033184585	-0.027136498	0.843552324	0.985393296	Potri.014G006700.3	AT5G65890.1
Potra2n14c26366.1	-0.218682858	-0.030956734	0.143680972	0.983446961	Potri.014G008400.1	AT3G49800.1
Potra2n14c26367.1	7.488356579	NA	1.0978E-09	NA	Potri.014G006700.3	AT5G65890.1
Potra2n14c26368.1	4.028754859	0.737682254	0.00182055	NA	Potri.014G006800.1	AT1G10150.1
Potra2n14c26369.1	3.725692629	0.909497646	2.67341E-31	0.349381798	Potri.014G006900.1	AT3G49780.1
Potra2n14c26370.1	1.64774763	NA	NA	NA	Potri.014G007000.1	AT4G10810.1
Potra2n14c26371.1	0.130065554	0.010485335	0.701277748	0.996281171	Potri.014G007100.2	AT2G22850.1
Potra2n14c26372.1	-0.671222908	0.07213831	0.072680692	0.978203092	Potri.014G007200.1	AT2G22840.1
Potra2n14c26373.1	-2.192080174	0.576555815	0.046185033	0.907865169	Potri.014G007701.1	
Potra2n14c26374.1	0.294180277	0.297121386	0.675168448	0.935099709	Potri.010G171300.1	AT1G79560.1
Potra2n14c26375.2	-0.124904789	0.032853974	0.840802438	0.993589787	Potri.004G169800.1	AT3G14460.1
Potra2n14c26376.1	1.130900272	0.470204208	NA	NA	Potri.014G002900.1	AT3G14460.1
Potra2n14c26377.2	0.111953872	0.133994222	0.681917379	0.940841616	Potri.014G012100.10	AT5G65840.1
Potra2n14c26378.1					Potri.007G005600.1	AT5G67620.1
Potra2n14c26379.2	0.056312397	0.933672058	0.979033535	NA	Potri.014G107500.2	AT4G35580.3
Potra2n14c26380.1	NA	NA	NA	NA	Potri.014G011800.1	
Potra2n14c26381.1	0.840274147	0.611207583	0.007292094	0.466127673	Potri.014G007900.1	AT3G14470.1
Potra2n14c26382.1	-0.541394313	0.121907951	0.264753466	0.960981446		
Potra2n14c26383.1	2.409567423	NA	NA	NA	Potri.014G009600.1	AT3G14470.1
Potra2n14c26384.1	0.44247923	-0.418769948	0.856110252	NA	Potri.004G169800.1	AT3G14460.1
Potra2n14c26385.1	-2.231073944	-1.660245878	NA	NA	Potri.014G001700.2	AT1G16760.1
Potra2n14c26386.1	0.228224791	-0.040308748	NA	NA	Potri.014G011800.1	
Potra2n14c26387.1					Potri.T055866.1	
Potra2n14c26388.1	-0.60201888	-0.061938916	NA	NA	Potri.014G007500.1	
Potra2n14c26389.1	0.36089035	0.623487645	0.293763874	0.36195005	Potri.014G005600.3	AT3G14470.1
Potra2n14c26390.3	0.273510406	0.189472777	0.22316083	0.912777843	Potri.014G012100.10	AT5G65840.1
Potra2n14c26391.1	-1.253174088	-0.577538025	0.00145411	0.724604666	Potri.014G012200.1	AT4G37750.1
Potra2n14c26392.1	-0.976997379	-0.291875826	0.001149348	0.806022635	Potri.014G012300.1	AT3G49750.1
Potra2n14c26393.1	2.398539511	3.436355007	0.464547509	NA	Potri.014G012400.1	AT2G14520.1
Potra2n14c26394.1	-0.212263813	0.211105044	0.540432087	0.921099436	Potri.014G012500.1	AT3G49710.1
Potra2n14c26395.1	-0.438557406	-1.474228928	0.625037305	0.530383957	Potri.014G012600.1	AT4G37770.1
Potra2n14c26396.1	-0.63339211	-0.316845501	0.006251899	0.525556195	Potri.014G012700.1	AT1G58470.1

Potra2n14c26397.1	3.710647568	2.878643434	0.339753101	NA	Potri.014G012800.1	AT2G22840.1
Potra2n14c26398.1	4.137462896	NA	0.137768486	NA	Potri.014G012900.1	
Potra2n14c26399.1	5.864334756	NA	0.000221626	NA	Potri.014G013000.1	
Potra2n14c26400.1	NA	NA	NA	NA		
Potra2n14c26401.1	0.003376178	0.096964384	0.975922676	0.918674657	Potri.014G013100.2	AT1G44910.1
Potra2n14c26402.1	0.102734087	0.145506337	0.435433378	0.920141992	Potri.014G013200.1	
Potra2n14c26403.1	1.12906545	0.088457249	0.028696452	0.98177802	Potri.014G013300.1	AT1G59540.1
Potra2n14c26404.1	2.340207562	NA	NA	NA	Potri.014G013400.1	AT1G59530.1
Potra2n14c26405.1	0.616523849	0.080409234	1.64495E-06	0.936549944	Potri.014G013500.1	AT1G59520.1
Potra2n14c26406.1	-0.989332955	-0.512307701	2.22939E-10	0.260170161	Potri.014G013600.1	AT4G10810.1
Potra2n14c26407.1						
Potra2n14c26408.1					Potri.015G077300.3	AT4G32180.1
Potra2n14c26409.1	9.168948191	5.827096675	2.87379E-45	0.000564937	Potri.014G048300.3	
Potra2n14c26410.1	5.763454987	1.987699895	2.03518E-22	0.494304334	Potri.014G014000.1	AT3G49780.1
Potra2n14c26411.1	-0.397842533	-0.078391766	0.62823097	0.987226805		
Potra2n14c26412.1	-0.574608503	-0.09885322	0.040576029	0.946404907	Potri.014G014100.1	AT1G10150.1
Potra2n14c26413.1	-0.517393346	-0.033373047	0.000296185	0.987548286	Potri.014G014200.1	AT1G10720.1
Potra2n14c26414.1	0.808776149	0.00476626	0.000355281	0.99819408	Potri.014G014300.1	AT3G49810.1
Potra2n14c26415.1	0.253274488	0.08538327	0.56887219	0.97950444	Potri.014G014400.2	AT1G27480.1
Potra2n14c26416.1	2.640478978	0.284898362	3.70855E-24	0.936549944	Potri.014G014500.1	AT1G58420.1
Potra2n14c26417.1	2.606069542	0.058296761	9.1829E-06	0.995579852	Potri.014G014600.1	AT1G58420.1
Potra2n14c26418.1	0.021863475	0.024783746	0.882673699	0.978251387	Potri.014G014700.2	AT1G10130.1
Potra2n14c26419.1	0.639663679	-0.0372909	0.00051034	0.975578749	Potri.014G014900.1	AT4G07410.1
Potra2n14c26420.1	2.327043947	0.488763964	NA	0.894138111	Potri.014G015000.1	AT4G33800.1
Potra2n14c26421.1	0.943350674	1.562090794	NA	NA	Potri.014G015100.2	AT1G65550.1
Potra2n14c26422.1	-0.513561705	-0.105977448	0.004664434	0.937687311	Potri.014G015166.1	
Potra2n14c26423.1	-0.709757828	-0.353952217	0.110201614	0.891529107	Potri.014G015300.1	AT1G27461.1
Potra2n14c26424.1	3.550497389	NA	0.257931469	NA		
Potra2n14c26425.1	-2.295337707	-2.446267299	NA	NA	Potri.014G015500.1	AT4G31540.1
Potra2n14c26426.1	-0.588841247	-0.247231093	3.26854E-07	0.72665641	Potri.014G015600.1	AT1G10200.1
Potra2n14c26427.1	-0.154469092	-0.032729326	0.14302203	0.974264269	Potri.014G015700.1	AT5G22370.1
Potra2n14c26428.1	0.823593783	-0.457135334	0.230160693	0.922993749	Potri.014G015800.1	AT1G59590.1

Potra2n14c26429.1	0.056674843	-0.093575081	0.983764755	NA	Potri.002G118300.1	AT1G59600.1
Potra2n14c26430.2	-0.155617755	0.107013138	0.261792677	0.949966783	Potri.014G015932.1	
Potra2n14c26431.1	-0.292067193	0.022795083	0.007417451	0.989304496	Potri.014G016000.1	AT1G27460.1
Potra2n14c26432.2	-0.189268056	0.035890491	0.102855194	0.974367223	Potri.014G016100.1	AT1G27840.1
Potra2n14c26433.2	-0.499141006	0.435204839	0.100863396	0.823455455	Potri.014G016200.1	AT5G43190.1
Potra2n14c26434.1	1.108528418	0.404143935	1.30938E-08	0.774693915	Potri.014G016300.1	AT5G67500.1
Potra2n14c26435.1	0.674911769	0.119374312	9.45695E-08	0.948577865	Potri.014G016400.2	AT3G01400.1
Potra2n14c26436.1	2.159605525	1.774614041	0.000157501	0.61887385	Potri.014G016500.1	AT3G03990.1
Potra2n14c26437.2	0.146846972	-0.00266438	0.426182269	0.999053662	Potri.014G016600.4	AT1G27770.1
Potra2n14c26438.1	-1.066145097	0.017351392	0.189207808	0.997279462	Potri.014G016700.3	AT4G37630.2
Potra2n14c26439.1	NA	NA	NA	NA	Potri.001G155750.1	AT1G21280.1
Potra2n14c26440.1	-0.116319634	-0.063943719	0.29615337	0.948577865	Potri.014G016800.2	AT1G27760.3
Potra2n14c26441.1	0.530818487	0.517457024	0.026931701	0.724604666	Potri.014G016900.1	AT1G27752.1
Potra2n14c26442.1	0.094046089	0.161417684	0.333206034	0.807509123	Potri.014G016900.1	AT1G27752.1
Potra2n14c26443.1	2.632524198	NA	NA	NA	Potri.014G017100.1	AT2G14760.1
Potra2n14c26444.1	-0.04522616	-0.263285273	0.740255794	0.829716187	Potri.014G017200.2	AT1G27450.1
Potra2n14c26445.1	2.145670735	0.768183896	0.020254142	0.653059746	Potri.014G017300.1	AT1G27730.1
Potra2n14c26446.1	-1.291325866	-0.805366181	9.1954E-06	0.248783868		
Potra2n14c26447.1	-1.326694696	-0.860026502	2.94928E-06	0.172308534	Potri.014G017400.1	AT3G49940.1
Potra2n14c26448.1	-2.605356563	-2.035619085	NA	NA		
Potra2n14c26449.1	-1.301744846	-0.361864773	1.07695E-06	0.72680202	Potri.014G017500.1	AT1G49890.1
Potra2n14c26450.1	-1.257397838	-1.146887887	0.075990958	0.49278277	Potri.014G017600.1	AT5G01075.1
Potra2n14c26451.1	0.562231012	0.080904784	0.000139525	0.941855873	Potri.002G119600.1	AT4G33865.1
Potra2n14c26452.1	1.311912872	0.265289405	0.301002279	NA	Potri.014G017800.1	AT5G43140.1
Potra2n14c26453.1	-0.165859799	-0.092733281	0.155448948	0.876608977	Potri.014G017900.1	AT2G26680.1
Potra2n14c26454.1	-0.079703448	-0.004643731	0.525140466	0.996032145	Potri.014G018000.1	AT5G43130.1
Potra2n14c26455.1	0.240926956	-0.033208387	0.072423962	0.9761362	Potri.002G120100.1	AT2G23090.1
Potra2n14c26456.2	-0.814385734	-0.015753748	0.000120937	0.993589787	Potri.014G018700.3	AT4G24230.6
Potra2n14c26457.1	-1.373337465	-1.31043843	0.427227175	NA	Potri.014G018801.1	
Potra2n14c26458.1	0.180732128	0.101031404	0.520696696	0.945173238	Potri.014G019000.1	AT5G65860.1
Potra2n14c26459.1	0.150838685	0.097800084	0.621929671	0.949966783	Potri.014G018900.1	AT5G67360.1
Potra2n14c26460.1	-0.107063002	-0.005803569	0.478048019	0.996154416	Potri.014G019100.2	AT1G27700.1

Potra2n14c26461.1	3.497472566	2.304288993	3.23791E-52	0.000178362	Potri.014G019300.1	AT4G24220.1
Potra2n14c26462.1	1.168002448	0.533529827	1.3146E-08	0.759797728	Potri.014G019500.1	AT4G24220.2
Potra2n14c26463.1	1.161475876	0.857735924	6.18002E-15	0.258140976	Potri.014G019600.1	AT4G24220.1
Potra2n14c26464.1	3.77323644	0.495681655	8.35011E-11	0.88080281	Potri.014G019700.1	AT4G24220.1
Potra2n14c26465.1	-0.001936496	0.060193967	0.993535447	0.943586317	Potri.014G019900.1	AT3G19540.1
Potra2n14c26466.1	-0.009461714	-0.853056172	0.991169783	0.767368598	Potri.014G020100.1	AT5G59845.1
Potra2n14c26467.1	0.569240127	0.255337698	8.01654E-07	0.651973886	Potri.014G020200.1	AT5G43100.1
Potra2n14c26468.1	2.778336227	2.88689365	0.374412606	NA	Potri.014G020300.1	AT5G56510.1
Potra2n14c26469.1	0.056258387	0.581542875	0.865579252	0.564195735	Potri.014G020600.1	AT4G37370.1
Potra2n14c26470.1					Potri.014G020800.1	AT4G37370.1
Potra2n14c26471.1	1.773969762	0.310262406	6.09669E-07	0.934439727	Potri.014G020900.1	AT2G38185.1
Potra2n14c26472.1	-0.206100809	-0.174950674	0.705147681	0.922993749	Potri.014G021100.1	AT1G47230.1
Potra2n14c26473.1	-1.718501513	-0.286727515	0.15345909	0.953831244	Potri.014G021300.1	
Potra2n14c26474.1						
Potra2n14c26475.1	-1.154362666	-2.74743915	NA	NA		
Potra2n14c26476.1	2.355302221	0.506568304	4.19485E-12	0.821709751	Potri.014G021400.1	AT5G10610.1
Potra2n14c26477.1	1.075423306	0.06850473	2.77748E-10	0.95981322	Potri.014G021800.1	AT4G37340.1
Potra2n14c26478.1	-0.342118511	-0.269481259	0.007950096	0.48299238	Potri.014G021900.1	AT1G47200.1
Potra2n14c26479.1	2.492428534	1.363811008	NA	NA	Potri.014G022000.2	
Potra2n14c26480.1	-0.142312867	0.39837763	0.552379354	0.69121391	Potri.014G022100.1	AT4G24210.1
Potra2n14c26481.1					Potri.014G022200.1	AT2G23270.1
Potra2n14c26482.1	NA	NA	NA	NA	Potri.014G022300.1	
Potra2n14c26483.1	0.604335114	0.049747517	0.000617883	0.973365801	Potri.014G022400.2	AT4G37280.1
Potra2n14c26484.1	1.813026521	0.534157064	1.08131E-05	0.474295608	Potri.014G022500.1	AT4G37260.1
Potra2n14c26485.1	0.583404408	3.253784057	0.237359388	0.138040185	Potri.014G022600.1	AT3G53010.1
Potra2n14c26486.1	1.07958994	0.423138976	0.000680369	0.857189843	Potri.014G022800.1	AT4G34200.1
Potra2n14c26487.1	-1.180675774	0.089828831	0.007254636	0.981772014	Potri.014G022900.1	AT3G16150.1
Potra2n14c26488.1	0.457248066	1.093905355	0.020866731	0.000134272	Potri.014G023000.1	AT4G34160.1
Potra2n14c26489.5	-0.124593777	0.048081171	0.458139225	0.974756511	Potri.014G023200.2	AT3G46630.1
Potra2n14c26490.3	-0.063467525	0.047238506	0.661185747	0.960981446	Potri.014G023500.1	AT1G47056.1
Potra2n14c26491.2	0.123736097	0.034738775	0.374412606	0.972159498	Potri.014G023800.1	AT4G07390.1
Potra2n14c26492.1	-0.059350729	-0.05427639	0.830792924	0.979896883		

Potra2n14c26493.1	0.833236666	1.50500868	0.125082538	0.478936926	Potri.014G024000.2	AT1G60060.1
Potra2n14c26494.1	-0.335067319	-0.281359014	0.05078612	0.812936459	Potri.014G024100.1	AT5G43060.1
Potra2n14c26495.2	-0.550913343	0.205132788	0.000928485	0.817452127	Potri.014G024200.2	AT4G24240.1
Potra2n14c26496.1	0.102236182	1.166926133	NA	NA		
Potra2n14c26497.1	0.351792582	0.343516083	0.075886289	0.677013336	Potri.014G024300.1	AT4G06744.1
Potra2n14c26498.2	-0.265282212	-0.389838818	0.29449137	0.72101822	Potri.014G024400.1	AT5G43020.1
Potra2n14c26499.1	-0.063158359	-0.071244037	0.763252826	0.965249957	Potri.014G024500.1	AT5G47830.1
Potra2n14c26500.3	-0.436080438	0.004923313	0.022853762	0.996292992	Potri.014G024700.2	AT5G47820.2
Potra2n14c26501.6	0.547744035	0.126511	1.99383E-07	0.833651746	Potri.014G024801.1	AT3G57870.1
Potra2n14c26502.1	1.41711724	0.285905947	4.48134E-07	0.864349786	Potri.014G025000.1	AT4G06536.1
Potra2n14c26503.1	-0.569219479	-0.201910349	0.005882272	0.810265332	Potri.014G025100.1	AT5G52010.1
Potra2n14c26504.1	-0.854815278	0.029530072	0.000718583	0.99114586	Potri.014G025200.3	AT1G46768.1
Potra2n14c26505.1						
Potra2n14c26506.1	-0.749662363	-0.307379078	7.6072E-06	0.763747409	Potri.014G025300.1	AT1G46480.1
Potra2n14c26507.1	-0.241503241	0.003344706	0.000595882	0.996292992	Potri.014G025400.1	AT1G49760.1
Potra2n14c26508.1	1.97876049	0.36739031	6.9347E-12	0.862335622	Potri.014G025500.1	AT5G42830.1
Potra2n14c26509.1	-0.61910233	-0.767708831	NA	NA	Potri.010G010300.1	
Potra2n14c26510.1	4.842393182	1.954083538	1.1667E-42	0.289065497		
Potra2n14c26511.1	0.011300174	0.030293768	0.973215259	0.987288338		
Potra2n14c26512.1	-0.193745324	-0.021429985	0.486184629	0.989878071	Potri.014G025600.1	AT5G42830.1
Potra2n14c26513.1	2.865144131	0.753486387	1.07257E-12	0.869186333	Potri.014G025500.1	AT5G42830.1
Potra2n14c26514.1	-0.106375583	-0.322726677	0.763754089	0.795928465	Potri.014G025600.1	AT5G42830.1
Potra2n14c26515.1	0.494698856	0.176644362	1.25875E-06	0.708329624	Potri.014G025700.1	AT4G27410.2
Potra2n14c26516.1	-0.426845322	-0.519451361	0.135081914	0.755774501	Potri.014G025800.1	AT4G36930.1
Potra2n14c26517.1	-2.121629222	-0.810664308	0.27694891	NA	Potri.014G025900.1	AT1G49770.1
Potra2n14c26518.1	-0.368138835	-0.279170599	0.029394543	0.802925711	Potri.014G026100.1	AT4G02630.1
Potra2n14c26519.1	0.835386472	0.353958282	0.257538227	0.936890993	Potri.006G010000.1	AT1G07250.1
Potra2n14c26520.1	1.523815397	0.389331558	0.140794032	0.95981322	Potri.014G026500.1	AT5G67090.1
Potra2n14c26521.1	-0.863736213	-0.069216575	0.005910528	0.97950444	Potri.014G026600.1	AT5G67360.1
Potra2n14c26522.1	-0.655157209	-0.029264621	0.009659726	0.992736072	Potri.014G026700.1	AT5G67090.1
Potra2n14c26523.1	1.136495461	0.232351047	6.37961E-06	0.88661378	Potri.014G026800.1	AT1G45688.1
Potra2n14c26524.1					Potri.014G026900.1	AT5G67070.1

Potra2n14c26525.1	0.03737285	0.222540422	0.826845319	0.840807554	Potri.014G027000.1	AT1G45976.1
Potra2n14c26526.2	-1.442699982	-0.50210854	2.35728E-06	0.679365931	Potri.014G027100.1	AT1G46264.1
Potra2n14c26527.1	0.2526465	-0.072747831	0.248221635	0.97137556	Potri.014G027200.1	AT3G49180.1
Potra2n14c26528.1	1.64774763	NA	NA	NA	Potri.014G027300.1	AT5G67060.1
Potra2n14c26529.1	1.14966934	0.373353268	0.18817689	0.903496619	Potri.014G027700.1	AT5G42930.1
Potra2n14c26530.2	1.347323972	0.402212801	0.103399367	0.892986206	Potri.014G027700.1	AT5G42930.1
Potra2n14c26531.1	1.503280372	0.359575843	0.042096442	0.909450222	Potri.014G027700.1	AT5G42930.1
Potra2n14c26532.2	0.82357019	NA	NA	NA		
Potra2n14c26533.2	-0.881005224	-0.396698223	0.000334259	0.706051231	Potri.014G027900.1	AT1G45207.2
Potra2n14c26534.1	-0.053645622	-0.093802306	0.766287393	0.925176712		
Potra2n14c26535.1	0.389405099	0.207530708	7.52222E-06	0.6619505	Potri.014G028100.1	AT5G42920.2
Potra2n14c26536.2	-0.122166013	0.091492771	0.447626891	0.95671968	Potri.014G028200.2	AT1G45249.1
Potra2n14c26537.1	-0.061778198	0.086957115	0.748424337	0.951046602	Potri.014G028300.1	AT1G45230.1
Potra2n14c26538.3	-1.328951947	0.851266564	NA	NA	Potri.014G028500.1	AT5G11210.1
Potra2n14c26539.1	-0.61910233	0.651571454	NA	NA	Potri.014G028800.1	AT2G29120.1
Potra2n14c26540.2	0.389436705	-0.084363291	0.296542812	0.942558727	Potri.014G028900.5	AT5G09890.1
Potra2n14c26541.1	-0.806671483	-0.36594363	6.14365E-07	0.816567218	Potri.014G029000.1	AT5G42900.3
Potra2n14c26542.1	-0.241427045	0.230364592	0.67957233	0.943819961	Potri.014G029100.1	AT3G19270.1
Potra2n14c26543.1	-0.315418347	-0.231900521	0.106447034	0.712599153	Potri.014G029200.2	AT1G07700.1
Potra2n14c26544.1	-2.111107164	-0.815018386	1.88249E-06	0.760284045	Potri.014G029300.1	AT5G42890.1
Potra2n14c26545.1	0.139730517	-0.131907014	0.575309524	0.900551258	Potri.014G029600.1	AT4G33400.1
Potra2n14c26546.1	0.944780655	0.436128224	1.03905E-29	0.048463789	Potri.014G029800.1	AT2G26570.1
Potra2n14c26547.1	-0.870069324	-0.301292293	8.38638E-07	0.762142808	Potri.014G029900.1	AT4G33330.2
Potra2n14c26548.2	0.112589553	0.296242972	0.53691241	0.724604666	Potri.014G030000.1	AT4G33350.1
Potra2n14c26549.1	NA	NA	NA	NA	Potri.014G030100.1	AT1G18210.1
Potra2n14c26550.1	-1.387295071	0.193786741	0.187224345	0.980355037	Potri.014G030200.1	AT1G32250.1
Potra2n14c26551.1	0.301497475	-0.010801393	0.908299948	NA	Potri.014G030500.1	AT5G42380.1
Potra2n14c26552.1	-1.328951947	-0.757196931	NA	NA	Potri.014G030600.1	
Potra2n14c26553.1	-0.595194773	-1.305887732	0.806034284	NA	Potri.014G030700.1	AT1G18890.1
Potra2n14c26554.1	1.060206708	-2.354181095	0.697335348	NA	Potri.014G030800.1	
Potra2n14c26555.3	-0.007029633	0.133459221	0.961400394	0.821709751	Potri.014G031000.2	AT5G42870.1
Potra2n14c26556.1	0.883512348	-0.98988064	0.630492842	NA	Potri.014G030900.1	AT5G42380.1

Potra2n14c26557.1	0.82357019	NA	NA	NA		
Potra2n14c26558.1	0.355948661	0.028774183	0.018582622	0.981726976	Potri.014G031200.1	AT1G47380.1
Potra2n14c26559.2	0.058116429	0.217345525	0.775036025	0.817452127	Potri.014G031800.1	AT5G44250.1
Potra2n14c26560.1	0.122620939	0.026185634	0.463620492	0.983113084	Potri.014G031900.1	AT4G33380.1
Potra2n14c26561.2	0.162998318	0.114293305	0.514755176	0.938121133	Potri.014G032000.1	AT4G10130.1
Potra2n14c26562.1	-0.562163598	-0.085430365	0.002467888	0.947317412	Potri.014G032100.1	
Potra2n14c26563.2	-0.400556722	-0.033344978	0.006428767	0.974756511	Potri.014G032400.1	AT1G47420.1
Potra2n14c26564.1	-0.501769738	-0.02685735	0.000992291	0.98177802	Potri.014G032300.1	AT1G47410.2
Potra2n14c26565.1	0.032536243	-0.297727943	0.914708845	0.791848264		
Potra2n14c26566.1	0.693454618	-0.358947666	0.009450014	0.830056077	Potri.014G032600.1	AT1G47480.1
Potra2n14c26567.1	-0.827227635	-0.329470049	0.006803548	0.728883321	Potri.014G032600.1	AT1G47480.1
Potra2n14c26568.1	1.008542522	-0.211347241	0.015039984	0.93154079	Potri.014G032800.1	AT1G47480.1
Potra2n14c26569.1	-0.168953312	-0.104012201	0.210501285	0.912777843	Potri.014G032900.2	AT5G23540.1
Potra2n14c26570.1	0.951468423	0.449512523	0.494583186	0.940556596	Potri.014G033400.1	AT2G26560.1
Potra2n14c26571.2	3.54442244	1.615304226	0.12669745	NA		
Potra2n14c26572.1	-1.886175408	-3.478805014	0.610303942	NA		
Potra2n14c26573.1	-2.191001697	-3.061406609	NA	NA		
Potra2n14c26574.4	5.062418653	2.104468949	1.32024E-08	0.775391304	Potri.014G033400.1	AT2G26560.1
Potra2n14c26575.1	4.957124055	2.319042347	7.84749E-06	0.794521408	Potri.014G033300.1	AT2G26560.1
Potra2n14c26576.1	0.057358282	-0.080496818	0.680476687	0.91913357	Potri.014G034100.2	AT4G34310.1
Potra2n14c26577.1	-0.723326769	-0.341493358	7.10803E-06	0.784830006	Potri.014G034200.1	AT2G26550.2
Potra2n14c26578.1	0.075871717	0.0040429	0.703552858	0.99812851	Potri.014G034300.1	AT2G15730.1
Potra2n14c26579.1	0.124930336	0.082020543	0.282785903	0.937846405	Potri.014G034400.4	AT4G11800.1
Potra2n14c26580.1	-9.624234257	-0.489748302	3.49429E-10	0.9761362	Potri.014G034500.1	AT3G50610.1
Potra2n14c26581.1	0.770937352	-0.101656532	8.13154E-08	0.949966783	Potri.014G034600.1	AT1G47490.1
Potra2n14c26582.1	0.4658447	0.280907857	0.004646948	0.59929692	Potri.014G034700.1	AT2G26540.1
Potra2n14c26583.1	-0.357910676	0.459298144	0.396941874	0.812696706	Potri.014G034800.1	AT5G42690.1
Potra2n14c26584.1	4.633055016	3.15459614	0.001670062	NA	Potri.014G034900.1	AT2G26530.1
Potra2n14c26585.1	-0.728232202	0.647737099	0.303085519	0.821709751	Potri.014G035000.1	AT5G42680.2
Potra2n14c26586.1	4.47257836	2.016183737	6.34611E-06	0.293583922	Potri.014G035100.1	AT4G37260.1
Potra2n14c26587.1	-0.034657088	0.087153032	0.85307658	0.923348116	Potri.014G035300.1	AT5G06430.1
Potra2n14c26588.1	-2.464899732	-1.300503599	0.001628934	0.49278277	Potri.014G035400.1	AT2G26520.1

Potra2n14c26589.1	1.303578555	0.371707506	9.49845E-07	0.735542465	Potri.014G035500.2	AT5G66850.1
Potra2n14c26590.1	0.424072808	0.018298917	0.019610778	0.989328794	Potri.014G035600.1	AT1G47720.1
Potra2n14c26591.1	0.644339057	0.480816752	2.0071E-13	0.008032632	Potri.014G035700.1	AT4G33300.2
Potra2n14c26592.1	-0.226866901	-0.048581368	0.081174945	0.966005919	Potri.014G035800.3	AT2G26510.1
Potra2n14c26593.2	1.168606629	0.584943419	5.13301E-08	0.344206454	Potri.014G036000.1	AT1G47710.1
Potra2n14c26594.1	0.640652861	1.724493342	0.750682579	NA	Potri.014G035900.1	
Potra2n14c26595.1					Potri.014G036100.1	AT1G47710.1
Potra2n14c26596.1	-0.583865593	0.027073695	0.821198325	NA	Potri.014G036200.1	AT1G27040.1
Potra2n14c26597.1	-0.625424658	-0.251688247	0.001708752	0.820966257	Potri.014G036300.2	AT1G47310.1
Potra2n14c26598.1	1.157054078	0.563271029	1.49947E-27	0.055371208	Potri.014G036400.1	AT1G06870.1
Potra2n14c26599.2	-1.303489113	-0.344679446	0.000104788	0.865591169	Potri.014G036500.1	AT1G47670.1
Potra2n14c26600.1	0.283306864	-0.101929797	0.179086784	0.951394085	Potri.014G036600.1	AT1G47655.1
Potra2n14c26601.1	-1.734679053	-0.510234651	0.190421494	0.925933968	Potri.014G036700.1	AT2G15880.1
Potra2n14c26602.1	-0.181221941	0.045439747	0.150764219	0.94608223	Potri.014G036800.2	AT4G33280.1
Potra2n14c26603.3	2.256108821	1.085472069	9.4455E-05	0.727843918	Potri.014G036900.1	AT4G33280.1
Potra2n14c26604.1	0.198456798	0.195147337	0.245523467	0.721982409	Potri.002G130100.1	AT1G47640.1
Potra2n14c26605.1	-0.590218784	-0.328508562	0.081101064	0.87679119	Potri.014G037000.1	AT2G26500.1
Potra2n14c26606.2	0.914195407	0.226528146	0.000451237	0.836066102	Potri.014G037200.1	AT5G42630.1
Potra2n14c26607.1	-1.271401051	-0.392935648	0.078129291	0.924951347	Potri.014G037500.1	AT4G31940.1
Potra2n14c26608.1	2.492428534	1.871975723	NA	NA	Potri.T156400.1	AT4G31940.1
Potra2n14c26609.1	4.570506576	0.548302453	1.67651E-06	NA	Potri.014G037900.1	AT4G31940.1
Potra2n14c26610.1	1.657833832	0.034521437	0.000284248	0.996032145	Potri.014G038000.1	AT4G31940.1
Potra2n14c26611.1	-1.733985759	-0.668697906	8.93387E-36	0.138040185	Potri.014G038300.2	AT3G13690.1
Potra2n14c26612.1	-0.101983685	-0.266764193	0.915754458	0.948577865	Potri.014G038500.1	
Potra2n14c26613.1	4.016360855	1.40291113	2.02588E-15	0.574820121	Potri.014G038600.1	AT2G23450.2
Potra2n14c26614.1	1.210809951	0.204713008	0.037985867	0.95981322	Potri.014G038700.1	AT5G42650.1
Potra2n14c26615.1	0.290628894	-0.154333639	0.326321507	0.956865545	Potri.014G038800.1	AT1G48460.1
Potra2n14c26616.1	-0.250837919	0.103194325	0.635474981	0.98177802	Potri.014G038900.1	AT3G23330.1
Potra2n14c26617.2	-0.386253697	-0.081816002	0.874448171	NA	Potri.014G039000.1	AT4G19390.1
Potra2n14c26618.1	0.116723443	-0.010885573	0.275700227	0.99114586	Potri.002G131400.1	AT1G47550.1
Potra2n14c26619.3	-0.449322166	-0.181083909	0.001648601	0.784063198	Potri.014G039200.1	AT5G53220.1
Potra2n14c26620.4	0.693805106	0.562129075	0.011095566	0.221840363	Potri.014G039400.1	

Potra2n14c26621.1	-0.170100113	-0.117772885	0.319135475	0.895474078	Potri.014G039600.1	AT1G74970.1
Potra2n14c26622.1	0.628831807	0.210320186	1.27266E-05	0.820212646	Potri.014G039700.1	AT1G13570.1
Potra2n14c26623.1	-0.591936966	-0.34537894	0.141978752	0.907073354	Potri.014G039800.1	AT1G47510.1
Potra2n14c26624.1	0.822270247	0.473548933	0.002933663	0.306363984	Potri.014G039900.1	AT1G19270.1
Potra2n14c26625.1	0.123943621	-0.873002103	0.97100389	NA	Potri.014G040100.2	AT3G02890.1
Potra2n14c26626.4	0.129907092	0.316866395	0.637637346	0.749682671		
Potra2n14c26627.1	-0.175807432	-0.178067621	0.752084781	0.950044491		
Potra2n14c26628.1	-0.569109688	-0.537548471	0.050723461	0.750123244	Potri.014G040300.1	AT1G19300.1
Potra2n14c26629.1	0.08522757	-0.077809074	0.572604578	0.917014822	Potri.014G040400.1	AT1G19310.1
Potra2n14c26630.1	3.620827449	3.698854129	0.228924926	NA	Potri.014G040500.1	AT4G36830.1
Potra2n14c26631.1	0.192080292	0.028448967	0.326863444	0.983446961	Potri.014G040600.1	AT1G75020.2
Potra2n14c26632.1	-0.400830728	-0.108663456	0.053483128	0.935995049	Potri.014G040800.1	AT5G42620.2
Potra2n14c26633.1	0.244629366	-1.055809802	0.942430144	NA	Potri.014G040900.1	
Potra2n14c26634.1	-2.592344392	-0.363742961	3.46524E-13	0.836066102	Potri.014G041000.1	AT5G42610.1
Potra2n14c26635.1	-0.640199977	-0.148791644	0.000125512	0.817452127	Potri.014G041200.1	AT1G75060.1
Potra2n14c26636.1	0.280369475	0.19762998	0.047238711	0.814981587	Potri.014G041300.1	AT1G65910.1
Potra2n14c26637.2	0.172269152	-0.01538848	0.456115102	0.991407187	Potri.014G041400.1	AT1G19340.1
Potra2n14c26638.1	2.044530442	0.997542923	0.047232787	0.885375133	Potri.009G113000.5	AT4G36800.1
Potra2n14c26639.1	0.328958383	0.382182187	0.223860161	0.802631112	Potri.014G041600.1	AT1G75080.2
Potra2n14c26640.1	-0.773158302	-0.351017945	0.003960287	0.792544431	Potri.014G041700.1	AT1G75090.1
Potra2n14c26641.1	7.18969233	3.337765006	1.3285E-125	0.001652897	Potri.014G041800.1	AT4G01070.1
Potra2n14c26642.1	0.325735429	-0.147197834	0.034638783	0.927879595	Potri.014G041900.1	AT4G01070.1
Potra2n14c26643.1	0.124419953	0.046413691	0.509641055	0.981401592	Potri.014G042000.1	AT1G47750.1
Potra2n14c26644.2	-0.458163897	-0.126925759	7.36591E-05	0.923397137	Potri.014G042200.1	AT1G80950.1
Potra2n14c26645.1	-0.572562432	-0.357902417	0.000199978	0.709241338	Potri.014G042300.1	AT1G47740.1
Potra2n14c26646.3	1.032837816	0.267520092	0.271194523	0.963557834	Potri.014G042600.1	AT1G75100.1
Potra2n14c26647.1	0.610941003	0.329850219	1.42291E-05	0.666052699	Potri.014G042700.3	AT1G19360.1
Potra2n14c26648.1	0.032194327	0.001474124	0.760884746	0.999276367	Potri.014G042800.1	AT4G16580.1
Potra2n14c26649.1	-0.371956086	0.153208482	0.12860877	0.858174358	Potri.014G043100.1	AT1G75130.1
Potra2n14c26650.1	0.026774619	-0.207788102	0.916504687	0.88853753	Potri.014G043300.2	AT5G26667.1
Potra2n14c26651.1	2.476290371	1.571529889	NA	NA	Potri.014G043401.1	AT2G39445.1
Potra2n14c26652.1	1.545832463	1.571529889	NA	NA		

Potra2n14c26653.1	-0.14047099	-0.047178951	0.305901908	0.951394085	Potri.014G043500.1	AT2G38740.1
Potra2n14c26654.1	0.09842488	-0.049420664	0.348794414	0.953108412	Potri.014G043600.1	AT3G60190.1
Potra2n14c26655.1	-1.082154994	-0.839041636	2.5702E-08	0.023928609	Potri.014G043800.2	AT2G44600.1
Potra2n14c26656.1	0.06359361	0.097410436	0.779866847	0.945173238	Potri.014G043900.1	AT1G44750.1
Potra2n14c26657.1	-0.056772697	-0.113455531	0.743598448	0.885375133		
Potra2n14c26658.1	-0.856811008	-0.057073625	0.002870597	0.984292161	Potri.014G044100.1	AT5G46940.1
Potra2n14c26659.1	0.026742841	0.10472156	0.905547017	0.938246513	Potri.014G044200.1	AT2G44640.1
Potra2n14c26660.1	0.063280699	0.161675842	0.743474902	0.889258723	Potri.014G044300.1	AT3G60210.1
Potra2n14c26661.1					Potri.014G044400.1	AT4G11050.1
Potra2n14c26662.1					Potri.014G044400.1	AT4G11050.1
Potra2n14c26663.1	-0.147913994	-0.565659456	0.749575067	0.848131816	Potri.014G044500.1	AT1G02180.1
Potra2n14c26664.1	2.182277928	1.182829296	0.416106739	NA	Potri.014G044700.1	AT3G60410.1
Potra2n14c26665.1	-0.033122944	0.025190126	0.869659777	0.980069287	Potri.014G044800.1	AT3G60410.2
Potra2n14c26666.2	0.005865687	0.189426481	0.979287879	0.815348505	Potri.014G044900.1	AT3G60400.1
Potra2n14c26667.1	0.036309955	0.021797029	0.853445815	0.987226805	Potri.014G045000.3	AT3G04610.1
Potra2n14c26668.1	-0.605631431	-0.093823201	0.085474308	0.974367223	Potri.014G045100.1	AT3G60390.1
Potra2n14c26669.1	-0.229758064	-0.148955682	0.177225937	0.886827605	Potri.014G045200.1	AT4G01710.1
Potra2n14c26670.1	-0.713449329	-0.389947399	0.003098027	0.724604666	Potri.014G045300.1	AT3G60380.1
Potra2n14c26671.2	-0.41793424	-0.269493668	0.000401817	0.642417298	Potri.014G045500.1	AT2G35680.1
Potra2n14c26672.1	-0.541730267	-0.097745983	0.000165668	0.949966783	Potri.014G045600.2	AT3G60370.1
Potra2n14c26673.1	-0.523597495	-0.104990713	0.016236627	0.957436721	Potri.014G045900.1	AT1G05940.1
Potra2n14c26674.1	-0.126277913	0.61368446	0.90746733	0.874808359		
Potra2n14c26675.2	-0.102088185	-0.106546843	0.417225216	0.855587395	Potri.014G046000.1	AT2G44900.1
Potra2n14c26676.1	2.400676626	2.749102281	2.06814E-05	0.252090755	Potri.013G074800.1	ATCG01100.1
Potra2n14c26677.1	-0.206151158	-0.235510053	0.572363404	0.867910364	Potri.014G046200.1	AT2G44880.1
Potra2n14c26678.1	0.997513525	1.512070502	NA	NA	Potri.014G046300.1	AT3G60330.2
Potra2n14c26679.1	NA	NA	NA	NA	Potri.014G046400.1	AT2G44850.2
Potra2n14c26680.1	NA	NA	NA	NA	Potri.014G046500.1	AT4G33355.1
Potra2n14c26681.1					Potri.014G046600.1	AT2G44840.1
Potra2n14c26682.1	2.151323863	NA	NA	NA	Potri.014G046700.1	AT2G44840.1
Potra2n14c26683.1	1.624417064	0.730781543	0.062105482	0.867692916	Potri.014G046900.1	AT2G44840.1
Potra2n14c26684.1	8.973975961	5.445352286	6.34459E-11	NA	Potri.014G047000.1	AT2G44840.1

Potra2n14c26685.1	0.711996298	0.634641592	0.720804422	NA	Potri.014G047100.1	AT1G51660.1
Potra2n14c26686.1	0.142967829	-0.0462029	0.183796187	0.967284786	Potri.014G047200.1	AT1G02090.1
Potra2n14c26687.1	0.528678128	0.184569609	0.000257621	0.585240923	Potri.014G047300.2	AT1G02100.1
Potra2n14c26688.1	0.339356896	0.127721139	0.047815245	0.893135869	Potri.014G047400.1	AT3G60320.1
Potra2n14c26689.1	-1.001135482	-0.235684365	1.45008E-19	0.663950227	Potri.014G047500.1	AT2G44830.1
Potra2n14c26690.1	0.041885632	0.430093497	0.853317343	0.416170423	Potri.014G047600.2	AT1G02120.1
Potra2n14c26691.1	0.139227473	0.587534838	0.691454661	0.46151188		
Potra2n14c26692.1	-2.86015326	-1.581318067	NA	NA	Potri.014G047700.2	AT2G44810.1
Potra2n14c26693.1	0.033791847	-1.235659004	0.963012027	0.378220766		
Potra2n14c26694.1	3.20104637	NA	NA	NA	Potri.014G047800.1	
Potra2n14c26695.1	1.434322715	0.64247635	NA	NA		
Potra2n14c26696.1	0.82357019	1.363811008	NA	NA	Potri.014G047900.1	AT2G44810.1
Potra2n14c26697.1	-0.162731711	0.092209792	0.641593815	0.971432539	Potri.014G048000.1	AT3G60300.1
Potra2n14c26698.2	2.14708848	0.637235163	7.91057E-24	0.386802792	Potri.014G048700.1	
Potra2n14c26699.1	0.001060249	0.249190883	0.997305141	0.896627841	Potri.014G048000.1	AT3G60300.1
Potra2n14c26700.1	2.696919189	0.960515683	7.93246E-31	0.23464336	Potri.014G048100.10	
Potra2n14c26701.1	4.347213469	1.615266042	9.34409E-31	0.566010792	Potri.014G048900.2	
Potra2n14c26702.1	3.410608638	0.477835902	7.6061E-07	0.951394085	Potri.014G048300.3	
Potra2n14c26703.1	-0.203481855	-0.042470749	0.027209855	0.953608209	Potri.014G049200.1	AT3G48110.1
Potra2n14c26704.1	0.420864988	0.084920524	0.000338782	0.903769998	Potri.002G138300.1	AT4G17520.1
Potra2n14c26705.1	-0.113847585	-0.060068832	0.61007059	0.97521505	Potri.014G049400.1	AT1G02130.1
Potra2n14c26706.1	2.340207562	NA	NA	NA	Potri.014G049500.1	AT5G14620.1
Potra2n14c26707.1	2.834199377	0.389945594	9.67998E-05	0.939644353	Potri.014G049600.1	AT3G60280.1
Potra2n14c26708.1	0.305191372	0.024965982	0.04863997	0.983836797	Potri.014G049700.1	AT1G02140.1
Potra2n14c26709.3	-0.594875214	-0.224418851	7.23955E-09	0.723658895	Potri.014G049800.6	AT2G44770.1
Potra2n14c26710.1	0.763353592	0.109325928	6.91462E-07	0.953177702	Potri.014G049900.1	AT4G09320.1
Potra2n14c26711.2	-0.739619938	0.101327731	1.87583E-05	0.935851032	Potri.014G050000.1	AT2G44745.1
Potra2n14c26712.1	0.546993072	-0.277645947	0.059826469	0.859892315	Potri.014G050200.3	AT1G05490.1
Potra2n14c26713.1	-0.587384212	-0.455970797	3.12601E-05	0.443055334	Potri.014G050300.1	AT1G02150.1
Potra2n14c26714.1	0.391830372	-0.011057701	0.237236455	0.996032145	Potri.014G050400.1	AT2G44740.1
Potra2n14c26715.1	6.650464092	3.1735372	4.60478E-54	0.453118701	Potri.014G050532.1	AT1G34050.1
Potra2n14c26716.1	2.809259857	1.363811008	NA	NA	Potri.014G050532.1	AT1G34050.1

Potra2n14c26717.1	1.664677056	NA	NA	NA	Potri.014G050800.2	AT5G51160.1
Potra2n14c26718.1	-1.151581535	1.359561594	NA	NA	Potri.014G050400.1	AT2G44740.1
Potra2n14c26719.1	9.000284736	4.181387537	1.3496E-16	0.485359736	Potri.014G050532.1	AT1G34050.1
Potra2n14c26720.1	8.272544934	3.508380568	5.78068E-30	0.438817025	Potri.014G050532.1	AT1G34050.1
Potra2n14c26721.1	7.755320332	3.95975615	2.16915E-54	0.135796543	Potri.014G050532.1	AT1G34050.1
Potra2n14c26722.1	NA	NA	NA	NA	Potri.014G050800.2	AT5G51160.1
Potra2n14c26723.1	0.199627519	2.928568354	0.927567758	NA	Potri.014G051100.1	AT5G51160.1
Potra2n14c26724.1	-0.307851761	-0.112397243	0.006932933	0.814001428	Potri.002G139500.1	AT2G44730.1
Potra2n14c26725.1	-0.276306721	-0.099223385	0.028144345	0.911339141	Potri.002G139500.1	AT2G44730.1
Potra2n14c26726.2	-0.436365757	0.018705579	0.024262563	0.989304496	Potri.014G051300.1	AT4G17070.1
Potra2n14c26727.1	-0.081538138	0.084715805	0.527585613	0.882124231	Potri.014G051500.1	AT2G44710.1
Potra2n14c26728.1	-0.61910233	-0.767708831	NA	NA	Potri.014G051600.2	AT1G28710.3
Potra2n14c26729.3	-1.064297859	-0.735670399	3.70825E-06	0.135796543	Potri.014G051700.1	
Potra2n14c26730.1	0.055581056	-0.03688099	0.780417341	0.976302215		
Potra2n14c26731.1	0.055581056	-0.03688099	0.780417341	0.976302215	Potri.014G052000.2	AT5G24910.1
Potra2n14c26732.2	0.059335693	0.037038377	0.595327607	0.960981446	Potri.002G139900.6	AT5G47080.1
Potra2n14c26733.1	0.490552174	0.087049724	0.000403089	0.935851032	Potri.014G052300.1	AT4G17080.1
Potra2n14c26734.1	0.559119976	0.107805609	0.000573443	0.924256651	Potri.001G149300.1	AT3G10950.1
Potra2n14c26735.2	0.302296116	0.04177907	0.248686519	0.956865545	Potri.014G052500.1	AT1G02170.1
Potra2n14c26736.2	0.016535236	-0.021900581	0.921117663	0.985016122	Potri.014G052600.1	AT3G60240.2
Potra2n14c26737.1	0.757302056	0.332912834	6.03055E-05	0.629434457	Potri.014G052700.1	AT5G47070.1
Potra2n14c26738.1	-0.142814755	-0.036972289	0.517821595	0.979755286	Potri.014G052801.1	AT1G12775.1
Potra2n14c26739.1	0.094117153	-0.28561675	0.707841163	0.788065047	Potri.014G052900.1	AT2G44670.1
Potra2n14c26740.1	0.114957864	-0.178042091	0.551198008	0.896627841	Potri.014G053000.1	AT5G47030.1
Potra2n14c26741.2	0.348595875	0.101477133	0.053575722	0.937846405	Potri.014G053200.1	AT2G44660.1
Potra2n14c26742.1	0.730223257	0.059940463	4.64374E-06	0.966005919	Potri.014G053300.1	AT5G23740.1
Potra2n14c26743.1	0.931488865	0.209581202	2.31384E-14	0.784830006	Potri.014G053500.1	AT5G45290.1
Potra2n14c26744.1	2.514947433	0.909577281	2.10188E-22	0.180635806	Potri.014G053600.1	AT3G60220.1
Potra2n14c26745.1	-0.087326755	-0.088163667	0.860948705	0.976302215		
Potra2n14c26746.1	2.139243677	-3.618593119	0.135365324	NA	Potri.014G054100.2	AT3G60470.1
Potra2n14c26747.1	0.384274293	0.119126426	0.025902321	0.919424623	Potri.014G054300.1	AT3G60480.1
Potra2n14c26748.1	-0.094491335	0.030068922	0.712864398	0.985393296		

Potra2n14c26749.1	-0.07404137	0.041762684	0.780907958	0.97950444	Potri.014G054400.1	AT4G00026.1
Potra2n14c26750.1	0.055969695	0.014952022	0.657141098	0.987226805	Potri.014G054600.1	AT4G13970.1
Potra2n14c26751.1	-2.295337707	0.708152954	NA	NA	Potri.014G054700.1	AT3G60460.1
Potra2n14c26752.1	7.45522825	4.240825344	1.14519E-68	0.00111573	Potri.014G054800.3	AT3G60450.1
Potra2n14c26753.1					Potri.014G054900.1	AT1G64870.1
Potra2n14c26754.2	6.675939774	3.454936038	6.1139E-96	0.066859849	Potri.014G055400.3	AT3G17380.1
Potra2n14c26755.1	7.591531358	4.015822992	1.0209E-102	0.061800879	Potri.002G141100.2	AT5G22060.1
Potra2n14c26756.1	1.627574241	0.891916711	7.44477E-12	0.276865642	Potri.002G141100.2	AT5G22060.1
Potra2n14c26757.1	3.356708675	NA	NA	NA		
Potra2n14c26758.1	4.82198341	2.053508169	7.94879E-13	0.650882346	Potri.014G055700.1	AT2G44940.1
Potra2n14c26759.1	-0.036378624	0.169263594	0.775055725	0.788065047	Potri.014G055800.2	AT2G44950.1
Potra2n14c26760.1	-0.140276366	0.009420801	0.096819892	0.993589787	Potri.014G055900.1	AT2G44970.1
Potra2n14c26761.1					Potri.014G056000.1	AT5G46030.1
Potra2n14c26762.1					Potri.014G056000.1	AT5G46030.1
Potra2n14c26763.1	NA	NA	NA	NA	Potri.014G056300.2	AT1G02050.1
Potra2n14c26764.2	0.502119498	0.318435572	0.029566422	0.791002527	Potri.014G056400.1	AT1G02060.1
Potra2n14c26765.1	-0.333022021	-0.0092525	0.195506873	0.996032145		
Potra2n14c26766.1	-0.29602335	-0.060831698	0.074164294	0.974275337	Potri.014G056700.1	AT2G44980.2
Potra2n14c26767.1	-3.77752818	-1.196691838	7.17636E-07	0.831597503	Potri.014G056800.1	AT2G44990.1
Potra2n14c26768.3	0.031222953	-0.093222943	0.839759039	0.926454067	Potri.014G057000.1	AT2G45000.1
Potra2n14c26769.1	-0.014898539	-0.1765925	0.956650616	0.886818995		
Potra2n14c26770.1	0.949445351	0.104029689	0.807343183	NA	Potri.014G057100.1	AT3G18990.1
Potra2n14c26771.1	-0.482416642	-1.783128658	0.358318796	0.008780881	Potri.004G230100.1	AT3G18990.1
Potra2n14c26772.1	-1.384904012	-0.348278232	3.97311E-08	0.641037344	Potri.014G057200.1	AT4G25170.1
Potra2n14c26773.1	0.763872737	0.151176944	1.42072E-05	0.909715408	Potri.002G141500.1	AT4G16720.1
Potra2n14c26774.1	-0.088724862	-0.048672176	0.553388799	0.949966783	Potri.014G057400.1	AT3G60510.1
Potra2n14c26775.1	NA	NA	NA	NA	Potri.014G057500.1	AT5G43330.1
Potra2n14c26776.1	0.942750388	0.054275841	0.128574225	0.991407187		
Potra2n14c26777.2	4.780627148	2.260485611	1.49259E-09	0.734829722	Potri.014G057700.1	AT1G02065.1
Potra2n14c26778.1	-0.332828153	-0.444630562	0.694018767	0.896353214	Potri.014G057800.1	AT1G27370.3
Potra2n14c26779.1	-0.416594337	-2.324454457	0.884628951	NA		
Potra2n14c26780.1	-2.034504944	-0.949021963	2.09433E-05	0.170845723	Potri.014G057950.1	AT3G54070.1

Potra2n14c26781.1	-1.177745186	-0.292844875	9.15134E-06	0.847341104	Potri.014G058200.1	AT2G45040.1
Potra2n14c26782.1	-0.458655808	-0.410576391	0.402813421	0.885375133	Potri.014G058300.1	AT3G60520.1
Potra2n14c26783.2	-0.681389545	-0.196781684	0.167104306	0.930159505	Potri.014G058400.1	AT4G00030.1
Potra2n14c26784.1	0.477006157	0.087500461	0.00076284	0.912777843	Potri.014G058500.1	AT3G20050.1
Potra2n14c26785.1	2.632524198	2.490806657	NA	NA	Potri.014G058600.1	AT2G45050.1
Potra2n14c26786.1	0.531991628	0.164129671	0.001344847	0.882699919	Potri.014G058700.1	AT2G45060.1
Potra2n14c26787.1	0.041097839	0.080284605	0.758074585	0.896937427	Potri.014G058800.1	AT1G02080.2
Potra2n14c26788.1	-0.035604154	-0.011790296	0.847444877	0.992934677	Potri.014G058900.1	AT3G57540.1
Potra2n14c26789.1	0.355422025	0.047250152	0.015977536	0.972159498	Potri.014G059000.1	AT3G60540.1
Potra2n14c26790.1	-0.131183483	0.071909809	0.682573584	0.966228173	Potri.014G059200.1	
Potra2n14c26791.1	0.919379596	1.543431871	NA	NA	Potri.014G059300.1	AT3G60670.1
Potra2n14c26792.1	0.279601263	0.116618042	0.227311685	0.93154079	Potri.002G144800.1	AT4G00170.1
Potra2n14c26793.1	NA	0.674963689	NA	NA	Potri.014G059800.1	AT4G00165.1
Potra2n14c26794.1	1.420328098	0.671607327	0.223703369	0.916403589	Potri.014G059600.1	AT4G25225.1
Potra2n14c26795.1	0.803473017	-0.114960142	NA	NA	Potri.014G059800.1	AT4G00165.1
Potra2n14c26796.1	0.578504307	-0.41765001	0.292394439	0.885375133	Potri.014G060000.1	AT3G60660.1
Potra2n14c26797.1	-0.319315367	0.006528757	0.020614123	0.996032145	Potri.014G060100.1	AT2G45150.1
Potra2n14c26798.1	0.580802917	0.144976455	0.011857441	0.846926485	Potri.014G060200.1	AT4G00150.1
Potra2n14c26799.1	-0.070042958	-0.179221155	0.715927379	0.808901406	Potri.014G060300.3	AT4G16520.2
Potra2n14c26800.1	3.20104637	NA	NA	NA	Potri.014G060400.1	AT3G60650.1
Potra2n14c26801.1	0.247963291	0.063814035	0.203601189	0.955139324	Potri.014G060500.1	AT4G00150.1
Potra2n14c26802.4	-0.010461393	-0.005969749	0.939522301	0.996032145	Potri.014G060800.1	AT1G05030.1
Potra2n14c26803.1	-0.093791685	0.002614427	0.355927106	0.997983035	Potri.014G060900.1	AT2G45140.1
Potra2n14c26804.1	-0.208160747	0.135444324	0.344712506	0.914238347	Potri.014G061000.2	AT3G60590.3
Potra2n14c26805.1	NA	NA	NA	NA	Potri.014G061300.1	
Potra2n14c26806.1	-0.641374725	0.530353538	0.660402125	0.920028955	Potri.014G061400.1	AT2G45130.1
Potra2n14c26807.1	NA	NA	NA	NA	Potri.014G061300.1	
Potra2n14c26808.1	NA	1.372906117	NA	NA	Potri.014G061400.1	AT2G45130.1
Potra2n14c26809.1	NA	NA	NA	NA	Potri.014G061500.1	
Potra2n14c26810.1	1.300415245	-0.386126446	3.9406E-05	0.843774546	Potri.014G061850.1	
Potra2n14c26811.1	1.810682408	1.822378711	0.380833066	NA	Potri.014G065200.1	
Potra2n14c26812.1	2.030909952	3.959542273	NA	NA	Potri.014G065100.1	

Potra2n14c26813.1	3.399004252	1.296438229	0.047043784	NA	Potri.014G065200.1	
Potra2n14c26814.1	2.532107831	0.776002699	0.129953265	NA	Potri.014G065300.1	
Potra2n14c26815.1	1.072327463	0.52724603	0.017877212	0.831597503	Potri.014G065700.1	
Potra2n14c26816.1	NA	NA	NA	NA	Potri.014G065900.1	
Potra2n14c26817.2	-0.434381628	0.005806501	0.001162637	0.996032145	Potri.014G066000.1	AT4G00060.1
Potra2n14c26818.1	-0.621854498	-0.218174958	0.007648768	0.831463341	Potri.014G066100.1	AT2G45120.1
Potra2n14c26819.1					Potri.004G130500.1	AT3G20270.3
Potra2n14c26820.1	-0.611861434	-0.341302075	0.070571518	0.835819291	Potri.014G066200.1	AT1G02040.1
Potra2n14c26821.1	8.583087716	5.283043485	2.31515E-11	0.624602712	Potri.014G066300.1	AT1G65680.1
Potra2n14c26822.1	-0.984082821	-0.65511012	0.000143291	0.378307581	Potri.014G066400.1	AT3G60550.1
Potra2n14c26823.1	-0.618160882	-0.881933273	0.449525789	0.623736221	Potri.014G066500.1	AT4G00050.1
Potra2n14c26824.1	NA	NA	NA	NA		
Potra2n14c26825.1	-1.072995197	-0.34289289	8.08929E-07	0.661611864	Potri.014G066600.1	AT3G60680.1
Potra2n14c26826.2	-0.858644013	-0.976096013	0.602826679	0.907073354	Potri.014G066700.1	AT2G45190.1
Potra2n14c26827.1	-0.153193985	-0.072657235	0.4585723	0.957163493	Potri.014G066800.1	AT2G45200.1
Potra2n14c26828.1	0.97878608	0.129426032	0.000993916	0.948309446	Potri.014G066900.1	AT3G60690.1
Potra2n14c26829.1	1.072963759	0.486262765	0.651325791	NA	Potri.014G067000.1	AT3G60720.1
Potra2n14c26830.1	6.995150227	NA	1.29871E-07	NA	Potri.014G067100.1	AT2G45220.1
Potra2n14c26831.1	0.565763432	0.107543028	1.17321E-05	0.859813626	Potri.014G067300.1	AT2G45240.1
Potra2n14c26832.1	0.180808075	0.065752668	0.212371363	0.960670343	Potri.014G067400.3	AT3G60740.1
Potra2n14c26833.1	-1.281280421	0.192625169	0.283924307	0.977559521	Potri.014G067500.1	AT4G00080.1
Potra2n14c26834.1	NA	1.381953902	NA	NA	Potri.002G145900.1	AT2G45260.1
Potra2n14c26835.1	0.82357019	0.674963689	NA	NA	Potri.002G145900.1	AT2G45260.1
Potra2n14c26836.1	-0.013244209	0.092874539	0.928387777	0.870321018	Potri.014G067700.1	AT4G08900.1
Potra2n14c26837.1	0.249888492	0.041915968	0.10856544	0.976302215	Potri.014G067800.1	AT2G45270.1
Potra2n14c26838.1	1.64774763	NA	NA	NA	Potri.014G067900.1	AT4G21550.1
Potra2n14c26839.1	0.82357019	NA	NA	NA	Potri.014G068000.1	AT2G36080.2
Potra2n14c26840.1	0.012174852	0.152286664	0.969250175	0.903934429	Potri.014G068100.1	AT2G45280.2
Potra2n14c26841.1	-0.072553102	-0.240171552	0.723830952	0.854214303	Potri.014G068200.2	AT2G45290.1
Potra2n14c26842.1	-0.356960705	0.111823158	0.027729007	0.924951347	Potri.002G146400.1	AT2G45300.1
Potra2n14c26843.1	-0.693369501	-0.315087762	0.003495753	0.814001428	Potri.014G068400.1	AT1G02000.1
Potra2n14c26844.1	-0.046909856	0.07759696	0.816264586	0.948577865	Potri.014G068500.1	AT2G45320.1

Potra2n14c26845.1	0.902761029	0.19026943	1.96653E-05	0.909715408	Potri.014G068600.1	AT3G60770.1
Potra2n14c26846.1	0.207788295	-0.106768626	0.416987499	0.922993749	Potri.014G068700.1	AT2G45340.1
Potra2n14c26847.1	-0.466548745	-0.268914513	0.009228477	0.696743275	Potri.014G068800.1	AT2G45350.1
Potra2n14c26848.1	1.245744892	0.139386737	NA	NA	Potri.014G068900.1	AT3G60780.1
Potra2n14c26849.1	0.335014508	-1.396865918	0.715009738	0.612901012	Potri.014G069000.1	AT5G62280.1
Potra2n14c26850.1	0.02724596	0.027888165	0.901192084	0.981772014	Potri.014G069100.1	AT2G45380.1
Potra2n14c26851.1	-0.354350372	-0.191138665	0.0050187	0.871025364	Potri.014G069200.1	AT3G60800.1
Potra2n14c26852.1	-2.21297805	0.277656309	NA	NA	Potri.014G069300.1	AT1G02010.1
Potra2n14c26853.2	-0.00669316	0.108160522	0.975748686	0.903698951	Potri.014G069400.1	AT4G00090.1
Potra2n14c26854.1	-0.61910233	-0.767708831	NA	NA	Potri.014G069500.1	AT4G12110.1
Potra2n14c26855.1	0.091936318	0.036247481	0.578136802	0.96820892	Potri.014G069700.2	AT1G48050.1
Potra2n14c26856.1	0.234781859	0.073978305	0.082063253	0.950095151	Potri.014G069800.1	AT3G60820.3
Potra2n14c26857.1	6.104712695	2.676364087	0.000277139	NA	Potri.014G069900.1	
Potra2n14c26858.1	-0.222751101	-0.196903067	0.171931455	0.663037599	Potri.014G070000.1	AT4G12080.1
Potra2n14c26859.1	0.023252635	-0.152879723	0.93655587	0.905231727		
Potra2n14c26860.1	1.235599143	-0.029651556	0.001401138	0.989611637	Potri.014G070100.1	AT5G51600.1
Potra2n14c26861.1	-0.085872395	0.019743877	0.546289833	0.985393296	Potri.014G070201.1	AT3G60850.1
Potra2n14c26862.2	-0.038219849	0.020976227	0.848651961	0.987288338		
Potra2n14c26863.1	-1.850127539	-0.571898359	2.37904E-05	0.371918078	Potri.014G070300.1	AT4G00210.1
Potra2n14c26864.1	-1.302366632	-0.47087562	1.8457E-08	0.585704919	Potri.014G070400.1	AT2G45420.1
Potra2n14c26865.1	-0.247453896	-0.038524348	0.22098358	0.98036348	Potri.014G070500.1	AT1G01970.1
Potra2n14c26866.1	0.148240073	0.044694279	0.231753555	0.954419252	Potri.014G070600.1	AT3G60860.1
Potra2n14c26867.1	-1.21573455	-1.277320162	0.195125779	0.636923863	Potri.014G070800.1	AT2G45430.1
Potra2n14c26868.1	-0.334572974	-0.07044825	0.063867293	0.961286138	Potri.014G070900.1	AT1G01950.1
Potra2n14c26869.1	0.899424024	0.128827422	3.14285E-06	0.924951347	Potri.014G071100.1	AT2G45440.1
Potra2n14c26870.1	5.961349742	2.142521041	1.88376E-28	0.189955186	Potri.014G071200.1	AT3G60890.2
Potra2n14c26871.1	-0.099057762	0.220414181	0.501547303	0.824378527	Potri.014G071400.1	AT2G45460.2
Potra2n14c26872.1	0.121187721	0.159207878	0.431341077	0.896314971	Potri.014G071600.1	AT1G01940.1
Potra2n14c26873.1	-1.328597784	-0.48805989	1.00581E-25	0.679485917	Potri.014G071700.1	AT3G60900.1
Potra2n14c26874.1	-0.556982587	-0.208448049	0.41247182	0.956865545	Potri.014G071800.1	AT2G45480.1
Potra2n14c26875.2	-0.004038767	0.0421043	0.986324422	0.971828097	Potri.014G071800.1	AT2G45480.1
Potra2n14c26876.1	0.01925983	0.112118772	0.929728394	0.899964141	Potri.014G071900.1	AT2G45500.1

Potra2n14c26877.1	0.364273637	0.713687788	0.165090257	0.068616987	Potri.014G072000.1	AT2G45510.1
Potra2n14c26878.1	0.11279017	-0.003316713	0.33975225	0.996851113	Potri.014G072400.3	AT2G45520.1
Potra2n14c26879.2	0.153595202	0.041281065	0.251847531	0.963433057	Potri.014G072600.2	AT2G45540.1
Potra2n14c26880.1					Potri.002G150600.1	AT1G08500.1
Potra2n14c26881.4	0.043907378	-0.112678213	0.982938372	0.992934677	Potri.014G072800.1	AT3G61050.2
Potra2n14c26882.1	-0.477552961	-0.18007411	0.11136287	0.92963647	Potri.014G072900.1	AT2G45590.1
Potra2n14c26883.1	-0.184385469	-0.273226293	0.315983103	0.564643938	Potri.014G073000.1	AT2G45600.1
Potra2n14c26884.1	-1.409983009	1.817051317	NA	NA		
Potra2n14c26885.1	-0.405070557	-0.136505712	0.014871637	0.874808359	Potri.014G073300.1	AT2G45620.1
Potra2n14c26886.1	1.545832463	4.033282328	NA	NA		
Potra2n14c26887.1	0.00405239	0.096475012	0.989294206	0.95258232	Potri.014G073400.5	AT2G45630.2
Potra2n14c26888.1	-0.685011256	0.004359756	2.33678E-05	0.996154416	Potri.014G073500.1	AT2G45630.2
Potra2n14c26889.1	1.586833948	-0.748146007	0.020374057	0.896937427	Potri.014G073700.1	AT4G25420.1
Potra2n14c26890.3	0.25343064	0.111321797	0.32276423	0.932932656	Potri.014G073800.3	AT3G61130.1
Potra2n14c26891.1	0.522725489	0.095684138	0.899548449	NA	Potri.014G073900.1	
Potra2n14c26892.1	-1.343177486	-0.388432516	0.330674843	0.959497566	Potri.014G074000.1	AT2G45640.1
Potra2n14c26893.1	1.223743313	-0.834601298	NA	NA	Potri.014G074100.1	AT2G45650.1
Potra2n14c26894.1	NA	NA	NA	NA		
Potra2n14c26895.1	-0.007483606	-0.194958388	0.989255328	0.974751051	Potri.014G074200.2	AT2G45660.1
Potra2n14c26896.1	0.087688916	0.02145664	0.582511993	0.981772014	Potri.014G074300.1	AT2G45670.1
Potra2n14c26897.1	-0.636465218	-0.264352624	2.10863E-06	0.355901487	Potri.014G074400.1	AT4G31010.1
Potra2n14c26898.1	-1.253637771	0.149472313	0.003706393	0.957525726	Potri.014G074500.1	AT4G00230.1
Potra2n14c26899.1	-0.295474519	-0.156382958	0.454793536	0.948577865	Potri.014G074600.1	AT1G01900.1
Potra2n14c26900.1	-0.351433158	-0.217414601	0.34313333	0.922993749	Potri.014G074600.1	AT1G01900.1
Potra2n14c26901.1	-0.476841735	0.00461116	0.004209481	0.99736971	Potri.014G074700.1	AT2G42010.1
Potra2n14c26902.1	-0.183098654	0.10881373	0.061499469	0.88643982	Potri.014G074900.1	AT4G00231.1
Potra2n14c26903.1	1.27660744	-0.401406097	0.41259181	NA		
Potra2n14c26904.2	-0.046335576	-0.147241482	0.902529301	0.95671968	Potri.014G075000.4	AT3G61180.1
Potra2n14c26905.1	-0.225005843	0.212333265	0.191443624	0.910878765	Potri.014G075100.1	AT4G00720.1
Potra2n14c26906.1						
Potra2n14c26907.1	-0.946603026	-0.530726784	0.448009899	0.900884796	Potri.014G075200.1	AT4G00730.1
Potra2n14c26908.1						

Potra2n14c26909.2	0.099061659	-0.050747429	0.467660537	0.970442612	Potri.014G075300.1	AT4G00740.1
Potra2n14c26910.1	-0.273209198	-0.110551105	0.031115724	0.917014822	Potri.014G075500.1	AT3G61140.1
Potra2n14c26911.1	-0.531229612	-0.246138354	0.093194329	0.903477395	Potri.014G075600.1	AT4G00752.1
Potra2n14c26912.1	-0.573696889	-0.167032215	0.119303554	0.949847686	Potri.014G075700.2	AT4G00750.1
Potra2n14c26913.1	-0.652022159	0.083199056	0.04671907	0.978325961	Potri.014G075700.2	AT4G00750.1
Potra2n14c26914.1	-0.737300668	0.118452559	0.013496615	0.965515007	Potri.014G075700.2	AT4G00750.1
Potra2n14c26915.1	-0.683483643	0.156707085	0.000732505	0.923397137	Potri.014G075800.1	AT3G61060.1
Potra2n14c26916.1					Potri.014G075900.1	AT1G33060.2
Potra2n14c26917.1	6.00456798	4.100073088	2.41077E-05	NA	Potri.014G075900.1	AT1G33060.2
Potra2n14c26918.1	-0.111702623	0.250749285	0.964117917	NA	Potri.014G076000.1	AT4G35580.2
Potra2n14c26919.1	3.020182714	1.432370139	5.12325E-41	0.22901885	Potri.014G076100.1	AT1G33060.2
Potra2n14c26920.1	2.107844127	0.971581033	1.5942E-09	0.672803026	Potri.014G076200.1	AT4G00755.1
Potra2n14c26921.1	-0.66282081	-0.251259453	2.09362E-05	0.733303543	Potri.014G076300.2	AT4G00755.2
Potra2n14c26922.1	0.123871547	0.05901912	0.257911722	0.948834049	Potri.014G076400.10	AT2G45740.3
Potra2n14c26923.1	-0.934540034	-0.387940679	1.47956E-05	0.683816919	Potri.014G076500.1	AT2G45720.2
Potra2n14c26924.1	0.036412041	0.031680815	0.813464476	0.976139323	Potri.014G076600.1	AT4G25500.1
Potra2n14c26925.1	-1.567516215	1.056183866	NA	NA	Potri.002G153500.1	AT5G19790.1
Potra2n14c26926.1	-0.963098528	-0.623465587	2.66458E-06	0.247587303	Potri.014G076800.2	AT2G20030.1
Potra2n14c26927.1	-0.745541374	-0.324988136	0.001684766	0.679485917	Potri.014G077000.1	AT1G01840.1
Potra2n14c26928.1	-0.069855625	0.005769416	0.809612816	0.99819408	Potri.014G077100.2	AT1G01860.1
Potra2n14c26929.1	-1.409983009	-0.834601298	NA	NA		
Potra2n14c26930.2	-0.528624442	-0.145781508	4.84345E-05	0.693793454	Potri.014G077300.1	AT1G01880.1
Potra2n14c26931.1	2.684966943	0.207175582	0.238152481	NA	Potri.014G077400.2	AT4G00770.1
Potra2n14c26932.1	0.23351152	0.889597961	NA	NA	Potri.014G077500.1	AT1G12600.1
Potra2n14c26933.1	0.044557666	0.036720955	0.940181937	0.9896961	Potri.014G077600.1	AT2G01770.1
Potra2n14c26934.1	-0.056800289	-0.164864678	0.813416735	0.902847514	Potri.014G077700.2	AT1G01920.2
Potra2n14c26935.1	0.207779272	-0.026780314	0.087353106	0.983113084	Potri.014G077800.1	AT5G51980.1
Potra2n14c26936.1	-0.06562519	-0.012774895	0.851256252	0.996032145	Potri.014G077900.1	AT4G25433.1
Potra2n14c26937.1	-2.387624497	0.2126289	6.78287E-07	0.943483421	Potri.014G078000.1	AT4G22990.2
Potra2n14c26938.1	-0.091987615	-0.018192556	0.393785723	0.985263255	Potri.014G078100.1	AT1G01910.1
Potra2n14c26939.1	0.257318026	0.159903888	0.137988977	0.857189843	Potri.014G078200.1	AT3G61113.1
Potra2n14c26940.1	4.881447016	4.149003379	0.006700676	NA	Potri.014G078300.1	AT2G45690.1

Potra2n14c26941.1	0.229513533	0.289779904	0.083468955	0.377730381	Potri.002G152300.2	AT2G45690.1
Potra2n14c26942.1	-0.3116764	0.132429462	0.337772977	0.936402128	Potri.014G078500.1	AT2G45680.1
Potra2n14c26943.1	-0.513273941	-0.101052296	0.096819892	0.966917036		
Potra2n14c26944.1	NA	NA	NA	NA		
Potra2n14c26945.1	1.077891938	-0.302268681	0.00032984	0.900551258	Potri.014G078600.1	AT2G45760.1
Potra2n14c26946.1	2.125008159	1.562606126	7.97155E-07	0.101036442	Potri.014G078700.1	AT2G45760.1
Potra2n14c26947.2	-0.135957293	0.107709558	0.490198706	0.923348116	Potri.014G078800.2	AT2G45770.1
Potra2n14c26948.1	0.216302878	0.302420711	0.111073523	0.679660122	Potri.014G078900.2	AT3G61200.1
Potra2n14c26949.1	-0.288681411	-0.156582141	0.082675908	0.903477395	Potri.014G079000.1	AT4G35410.2
Potra2n14c26950.1	0.025443179	0.215396908	0.913708313	0.824378527	Potri.014G079100.1	AT1G73690.1
Potra2n14c26951.1	-0.408655339	0.481941362	0.790797002	0.922993749	Potri.014G079300.1	AT4G32730.2
Potra2n14c26952.1	-0.319876969	-0.089991361	0.044263695	0.900551258	Potri.014G079400.1	AT4G00550.1
Potra2n14c26953.1	0.841754632	0.926667025	0.040444897	0.485997361	Potri.014G079500.1	
Potra2n14c26954.2	0.054056933	0.081874326	0.57079892	0.91392603	Potri.014G079700.1	AT4G00560.1
Potra2n14c26955.2	0.577778124	0.121413279	0.000153567	0.922011871		
Potra2n14c26956.1	0.677658328	0.122341038	3.88413E-05	0.936549944	Potri.014G079900.1	AT4G00570.1
Potra2n14c26957.1	1.132780423	1.717262252	0.31095434	0.643173103	Potri.014G080400.1	
Potra2n14c26958.1	0.329821448	1.244363512	0.451240868	0.666699452	Potri.014G080300.1	AT2G45790.1
Potra2n14c26959.1	-0.169300716	0.121541201	0.302398912	0.91392603	Potri.014G080600.1	AT4G00590.1
Potra2n14c26960.1	NA	NA	NA	NA	Potri.002G157100.1	AT4G00620.1
Potra2n14c26961.1	0.073059671	0.137923947	0.546978115	0.854292371	Potri.014G080800.1	AT4G00630.1
Potra2n14c26962.1	-1.01121671	-0.102002582	0.000290805	0.96957478	Potri.014G080900.1	AT1G01780.1
Potra2n14c26963.1	-0.214953232	0.874169236	0.660250224	0.817799498	Potri.014G081000.1	AT2G38040.1
Potra2n14c26964.1	0.207735862	0.073963488	0.00966611	0.843683654	Potri.014G081100.2	AT4G00660.1
Potra2n14c26965.1	-1.828127997	-2.069599561	0.517117305	NA	Potri.014G081200.1	AT3G61250.1
Potra2n14c26966.1	-0.338701608	0.27709495	0.327012736	0.855754617	Potri.014G081300.1	AT3G61260.1
Potra2n14c26967.1	-0.245220187	0.137434868	0.23104383	0.880316339	Potri.014G081400.1	AT1G01760.1
Potra2n14c26968.1	-0.062646551	0.132210533	0.952461133	0.984292161		
Potra2n14c26969.1	2.755763217	2.460920877	4.89077E-15	0.156853598	Potri.014G081600.1	AT2G45830.1
Potra2n14c26970.2	0.444822996	0.096432536	3.1206E-06	0.833952007	Potri.014G081700.1	AT4G11610.1
Potra2n14c26971.1	NA	NA	NA	NA	Potri.014G081900.1	
Potra2n14c26972.1	-0.668092582	-0.377220565	6.38729E-07	0.628040312	Potri.014G082100.1	AT2G45850.2

Potra2n14c26973.1	-0.523158319	-0.248542239	0.000445949	0.812984049	Potri.014G082400.1	AT4G00710.1
Potra2n14c26974.1	-0.702222225	-0.186014617	5.30975E-05	0.82104194	Potri.014G082300.1	AT2G45860.1
Potra2n14c26975.1	-0.416545092	-0.237998171	0.000738685	0.38054227	Potri.014G082600.1	AT1G01730.1
Potra2n14c26976.1	-0.478033012	-0.116437207	0.074445362	0.936581945		
Potra2n14c26977.1	-0.136954689	0.044110029	0.518179661	0.970930149	Potri.014G082800.1	AT4G00530.1
Potra2n14c26978.1	-2.32006017	-2.46431287	NA	NA	Potri.014G082700.1	
Potra2n14c26979.1	-0.084493915	-0.230054494	0.70938128	0.805451998	Potri.014G082900.1	AT3G61320.1
Potra2n14c26980.1	-0.582430858	1.189117379	0.733766885	0.744103214	Potri.002G158800.1	AT4G20960.1
Potra2n14c26981.1	-0.191417716	-0.075993133	0.311766192	0.949485515	Potri.014G083100.1	AT4G00525.1
Potra2n14c26982.1	-0.191417716	-0.075993133	0.311766192	0.949485515	Potri.014G083200.1	AT3G61350.1
Potra2n14c26983.1	0.224930294	0.139594258	0.003286291	0.741833788	Potri.014G083300.1	AT1G01710.1
Potra2n14c26984.1	-0.0606157	-0.058761845	0.899999775	0.981772014		
Potra2n14c26985.1	-0.147883239	0.043245547	0.476556769	0.972159498	Potri.014G083600.1	AT3G49050.1
Potra2n14c26986.1	0.044160086	0.116629403	0.782632249	0.895560236	Potri.014G083700.3	AT2G45880.1
Potra2n14c26987.1	1.401216622	0.798642469	0.000698929	0.742308935	Potri.014G083900.2	AT5G41315.1
Potra2n14c26988.1	-2.928516434	-1.530157672	NA	NA	Potri.014G084000.1	AT4G23340.2
Potra2n14c26989.1	1.752791404	-0.808779522	0.500703524	NA	Potri.014G084100.2	AT4G00467.1
Potra2n14c26990.1	-0.258039568	-0.165602657	0.545230558	0.92963647	Potri.014G084200.3	AT4G00460.2
Potra2n14c26991.1	0.23351152	-0.068349583	NA	NA	Potri.014G084300.1	
Potra2n14c26992.1	-0.04402512	0.112783701	0.801525362	0.880870972	Potri.014G084500.3	AT4G00450.1
Potra2n14c26993.1	-0.016193189	0.230394885	0.906996136	0.728883321	Potri.014G084600.1	AT4G00440.2
Potra2n14c26994.1	-3.145081584	0.599125	NA	NA		
Potra2n14c26995.1	-3.025465965	-0.743311564	4.60644E-18	0.734313982	Potri.014G084800.1	AT1G01690.1
Potra2n14c26996.1	0.877516784	0.506220677	0.000208365	0.36759872	Potri.014G084900.1	AT2G45910.1
Potra2n14c26997.1	0.071677468	0.119145205	0.654207994	0.882699919	Potri.014G085300.1	AT1G01650.1
Potra2n14c26998.1	-0.251854602	-0.090551441	0.165837009	0.957668459	Potri.014G085400.2	AT3G61415.1
Potra2n14c26999.1	-0.337940972	-0.805042791	0.375947918	0.697112686	Potri.014G085500.1	AT4G00400.1
Potra2n14c27000.2	-0.44410988	-0.07489277	0.009055481	0.953831244	Potri.014G085700.1	AT4G00370.1
Potra2n14c27001.1	2.089049435	-0.787964375	0.147260518	NA	Potri.014G085800.1	AT2G45970.1
Potra2n14c27002.1	1.202437587	-0.0787192	0.00214603	0.985393296	Potri.014G085900.1	AT4G29910.1
Potra2n14c27003.1	0.12064914	0.214998576	0.252698297	0.84745523	Potri.014G086000.1	AT4G00355.2
Potra2n14c27004.1	0.059345449	0.117384025	0.717145448	0.872366576	Potri.014G086100.1	AT2G45990.2

Potra2n14c27005.1	-0.148414421	-0.00065055	0.495793048	0.999937671	Potri.014G086200.1	AT2G46000.1
Potra2n14c27006.1	1.848637944	0.756206836	1.71223E-09	0.305728522	Potri.014G086300.10	AT3G61440.1
Potra2n14c27007.1	NA	NA	NA	NA	Potri.014G086400.1	AT4G25630.1
Potra2n14c27008.3	-0.106636601	0.151464742	0.526015477	0.802942736	Potri.014G086500.1	AT2G46020.2
Potra2n14c27009.1	-0.390364409	-0.21765302	0.018662869	0.849446235	Potri.014G086600.1	AT1G63800.1
Potra2n14c27010.1	0.330738523	-0.014127446	0.038439567	0.992934677	Potri.002G161100.1	AT2G46040.1
Potra2n14c27011.1	-0.635525328	-0.004763979	0.044058976	0.998802143		
Potra2n14c27012.1	-0.193190803	-0.210547377	0.392650018	0.845014308	Potri.014G086900.1	AT4G00340.1
Potra2n14c27013.1	-0.176303355	0.302094066	0.213603738	0.782659274	Potri.014G087000.1	AT4G00335.1
Potra2n14c27014.1	-0.048445035	-0.16354054	0.921959326	0.949485515	Potri.014G087100.1	AT2G46050.1
Potra2n14c27015.1	-0.372469865	-0.176709347	0.003101511	0.855587395	Potri.014G087200.1	AT4G00330.1
Potra2n14c27016.1	0.944389858	0.440967739	7.45549E-14	0.372242801	Potri.014G087300.2	AT2G46060.1
Potra2n14c27017.1	-0.341343984	-0.191176851	0.544200555	0.948002367	Potri.014G087400.1	AT3G09740.1
Potra2n14c27018.1	-0.175567684	-0.170917943	0.743474902	0.93888209	Potri.014G087500.1	AT4G00310.2
Potra2n14c27019.1	0.243442828	0.111305594	0.200778784	0.936549944	Potri.014G027200.1	AT3G49180.1
Potra2n14c27020.1	1.03861684	-0.162492642	0.000121265	0.921596614	Potri.014G087600.1	AT4G23470.1
Potra2n14c27021.1	-0.102481681	0.199616894	0.857969058	0.939915707	Potri.014G087700.1	AT3G61460.1
Potra2n14c27022.1	NA	NA	NA	NA	Potri.014G087800.1	
Potra2n14c27023.1	-0.230431063	-0.185257096	0.123067005	0.866606065	Potri.014G087900.1	AT5G28350.1
Potra2n14c27024.1	-0.522428509	-0.484504243	0.028861855	0.647631655	Potri.014G088000.1	AT1G01580.1
Potra2n14c27025.1	0.153174731	-0.065204835	0.491385078	0.94795983	Potri.014G088100.1	AT4G00300.2
Potra2n14c27026.1	-0.380773764	-0.118157799	3.02558E-06	0.902676973	Potri.014G088200.1	AT4G00290.1
Potra2n14c27027.1	NA	NA	NA	NA		
Potra2n14c27028.1	3.125519396	0.285055009	1.60903E-05	0.959042326	Potri.014G088400.1	AT2G22590.1
Potra2n14c27029.1	3.303110362	0.413834836	3.12341E-18	0.902857376		
Potra2n14c27030.1	3.071524033	0.654662163	1.38026E-16	0.786910796	Potri.014G088400.1	AT2G22590.1
Potra2n14c27031.1	3.799677693	-0.111377485	1.43094E-05	0.991036771	Potri.014G088400.1	AT2G22590.1
Potra2n14c27032.1	2.799046237	0.704609524	1.68574E-25	0.741833788	Potri.014G088500.1	AT4G01370.1
Potra2n14c27033.1	0.022739731	-0.090859249	0.911631193	0.903477395	Potri.014G088600.1	AT2G46080.1
Potra2n14c27034.1	-0.152760918	-0.27833626	0.455072804	0.823250234	Potri.014G088800.1	AT1G01540.2
Potra2n14c27035.1	0.233835028	-0.001669373	0.018268796	0.999325699	Potri.014G088900.1	AT4G01320.1
Potra2n14c27036.1	0.327004022	-0.188010355	0.036487831	0.905231727	Potri.014G089100.1	AT4G01310.1

Potra2n14c27037.1	-0.007803325	0.05048182	0.947527277	0.937146435	Potri.014G089200.1	AT4G01290.2
Potra2n14c27038.1	-0.162182985	-0.099349173	0.094674019	0.845014308	Potri.014G089300.1	AT4G01280.2
Potra2n14c27039.1	-0.328576987	-0.023610756	0.050907753	0.984292161	Potri.014G089400.1	AT1G01510.1
Potra2n14c27040.1	0.183249428	0.023599692	0.388310385	0.985313207	Potri.014G089600.1	AT1G01500.1
Potra2n14c27041.1	-1.328951947	-1.478536539	NA	NA		
Potra2n14c27042.1	-1.328676965	0.036936107	7.12248E-19	0.985393296	Potri.014G089700.1	AT1G01490.1
Potra2n14c27043.1	-0.041624195	-0.150701067	0.893476022	0.953608209	Potri.014G089800.1	AT4G01270.1
Potra2n14c27044.1	NA	NA	NA	NA	Potri.014G089900.1	
Potra2n14c27045.1	-0.835600296	0.162453143	0.313796836	0.974275337	Potri.014G090000.1	
Potra2n14c27046.1	-0.019568364	0.012540445	0.909844509	0.993577196	Potri.014G090100.2	AT5G12370.2
Potra2n14c27047.1	-0.582046996	0.375374864	0.132160866	0.786548546	Potri.014G090300.1	AT4G01250.1
Potra2n14c27048.3	-0.301409324	0.222172286	0.317878677	0.850175528	Potri.014G090400.1	AT5G28460.1
Potra2n14c27049.1	1.824969047	0.667732305	2.37104E-18	0.357834052	Potri.014G090500.1	AT2G46110.1
Potra2n14c27050.1	0.602204219	0.131399753	0.011431898	0.913007989	Potri.002G164700.1	AT3G61540.1
Potra2n14c27051.1	3.871516585	2.793061808	0.017175983	NA	Potri.014G090700.1	AT2G46130.1
Potra2n14c27052.1	1.64774763	NA	NA	NA	Potri.014G090800.1	AT2G46140.1
Potra2n14c27053.1					Potri.002G165100.1	AT2G46150.1
Potra2n14c27054.1	-0.777849535	-0.337330368	0.174147451	0.87679119	Potri.014G091100.1	AT4G01240.1
Potra2n14c27055.1	-2.661952289	0.053111627	0.318140386	NA		
Potra2n14c27056.4	-0.440233642	0.104572568	1.23676E-05	0.90634987	Potri.014G091300.3	AT2G46180.1
Potra2n14c27057.1	-0.483428331	0.102354593	1.19721E-06	0.921015963	Potri.014G091200.1	AT2G46170.1
Potra2n14c27058.1	1.710002847	1.361711925	0.026309269	0.817452127		
Potra2n14c27059.1	1.902628172	2.06986652	0.053367081	0.586594557		
Potra2n14c27060.1	0.915623583	1.793023828	0.60293989	0.825454445		
Potra2n14c27061.1	2.277205756	2.898639769	0.31567289	0.784407529		
Potra2n14c27062.1	0.078214661	0.109013186	0.660096027	0.922011871	Potri.014G091400.4	AT2G46200.2
Potra2n14c27063.1	-1.804229302	-0.951584025	0.617679881	NA	Potri.014G091500.2	AT5G52900.1
Potra2n14c27064.1	NA	NA	NA	NA	Potri.014G091600.1	AT5G24090.1
Potra2n14c27065.1	NA	1.571529889	NA	NA	Potri.014G091700.1	AT5G24090.1
Potra2n14c27066.1	-0.738424024	-0.337161076	1.88441E-06	0.720242695	Potri.014G091800.1	AT2G46210.1
Potra2n14c27067.1	-0.156390765	-0.045514989	0.510054641	0.98177802	Potri.014G092200.1	AT2G46220.1
Potra2n14c27068.1	-0.687338968	-0.185843085	6.46819E-05	0.900551258	Potri.014G092300.1	AT2G46225.1

Potra2n14c27069.1	-0.359035983	-0.140728736	0.02876717	0.876740025	Potri.014G092400.1	AT4G01220.1
Potra2n14c27070.1	-0.289116518	0.08320749	0.164455795	0.958414802	Potri.014G092500.1	AT4G01210.1
Potra2n14c27071.1	0.281491181	-0.236741887	0.152223338	0.750330162	Potri.014G092600.1	AT2G46230.1
Potra2n14c27072.1	5.416423559	2.294320117	3.60845E-60	0.080583445	Potri.014G092800.1	AT5G24090.1
Potra2n14c27073.1	3.211507158	1.052901345	5.21638E-19	0.777674368	Potri.014G092800.1	AT5G24090.1
Potra2n14c27074.1	0.110526	-0.01646409	0.557013511	0.991846003	Potri.014G093100.2	AT2G30920.1
Potra2n14c27075.1	-0.444377235	-0.337902178	0.01846414	0.275352864	Potri.014G093200.1	AT3G61590.1
Potra2n14c27076.1	-1.144312118	-1.794587181	0.352160684	0.66405778	Potri.014G093300.2	AT1G01460.1
Potra2n14c27077.1	0.125165865	-0.100830688	0.637637346	0.96820892	Potri.014G093400.1	AT5G41620.1
Potra2n14c27078.2	-0.09455141	-0.060656399	0.551903805	0.936890993	Potri.014G093700.1	AT3G61600.2
Potra2n14c27079.1	-0.285255108	-0.289594319	0.166741194	0.683816919	Potri.014G093900.3	AT4G01150.1
Potra2n14c27080.1	-0.068973902	0.092730685	0.907236255	0.97601212	Potri.014G094100.1	AT4G01130.1
Potra2n14c27081.1	0.533830043	0.35217789	0.437763638	0.902857376	Potri.014G094000.1	AT4G01140.1
Potra2n14c27082.1	0.02336041	-0.127274298	0.967921668	0.881191525	Potri.014G094200.2	AT2G46270.1
Potra2n14c27083.4	0.060402097	0.130988422	0.64785915	0.860435458	Potri.014G094300.1	AT3G61620.1
Potra2n14c27084.1	-1.033222384	-0.644115001	2.96054E-05	0.656082742	Potri.014G094400.1	AT2G46300.1
Potra2n14c27085.1	-1.021338241	-1.617801231	0.177388662	0.156405333	Potri.014G094500.1	AT2G46310.1
Potra2n14c27086.1	-1.738183821	-0.468235411	0.026296511	0.9045874	Potri.014G094600.2	AT2G16070.2
Potra2n14c27087.1	-1.453098892	-0.66157433	1.28051E-06	0.516836114		
Potra2n14c27088.1	-1.450938	-0.665020981	1.38141E-06	0.517463209	Potri.014G094800.1	AT3G61640.1
Potra2n14c27089.1	-0.220205988	-0.019474514	0.04039561	0.983944804	Potri.014G094900.2	AT3G61650.1
Potra2n14c27090.1	-2.759857202	-1.166956831	0.017438573	0.856645555	Potri.014G095000.1	AT5G41761.1
Potra2n14c27091.1	-0.797791405	-0.611218473	0.012982556	0.605100478	Potri.014G095100.1	AT5G41761.1
Potra2n14c27092.1	0.63033793	0.640806573	0.605495156	0.935474933	Potri.014G095200.2	AT1G64300.1
Potra2n14c27093.1	-0.413200081	-0.118937672	1.75121E-06	0.755109612	Potri.014G095300.4	AT2G46340.1
Potra2n14c27094.1	0.448527375	0.054052042	0.002358032	0.96551968	Potri.014G095400.1	AT4G01100.1
Potra2n14c27095.1	1.012725807	0.284205811	0.009032132	0.844546105	Potri.014G095500.1	AT2G46370.4
Potra2n14c27096.1	1.459962178	NA	NA	NA	Potri.014G095600.1	AT3G61660.1
Potra2n14c27097.1	-0.003583216	0.003524455	0.978525619	0.996292992	Potri.014G095700.1	AT3G61670.1
Potra2n14c27098.1	-0.837963715	-0.529208217	1.61499E-07	0.568340663	Potri.014G095800.1	AT1G01430.1
Potra2n14c27099.1	-0.193455607	-0.578704516	0.550803163	0.557090855	Potri.014G096000.1	AT4G01070.1
Potra2n14c27100.1	-1.114266646	-0.535779767	6.28321E-09	5.24515E-05	Potri.014G096100.1	AT4G01070.1

Potra2n14c27101.1	2.522822085	1.913035715	8.76894E-29	0.002915668	Potri.014G096200.1	AT4G23810.1
Potra2n14c27102.1	1.116518847	0.96601054	0.21551196	0.804007296	Potri.014G096300.1	AT2G46410.1
Potra2n14c27103.1	-0.199732772	-0.100800047	0.373499656	0.935851032	Potri.014G096500.2	AT4G01050.1
Potra2n14c27104.1	0.314291643	0.155052646	0.002481203	0.840666702	Potri.014G096700.1	AT4G01040.1
Potra2n14c27105.1	-0.483189173	-0.249769932	0.014301558	0.824378527	Potri.002G169300.2	AT4G01037.1
Potra2n14c27106.1	-0.269444295	-0.17888227	0.640607542	0.954419252	Potri.001G016900.1	AT5G10400.1
Potra2n14c27107.1	0.131704661	0.437922364	0.622301693	0.646306582	Potri.014G097100.1	AT1G01360.1
Potra2n14c27108.1	-0.025307729	-0.480848484	0.949535358	0.727961068	Potri.014G097000.1	AT4G01030.1
Potra2n14c27109.1	-0.249409686	-0.138417203	0.253244261	0.915580711	Potri.014G097200.2	AT3G61680.1
Potra2n14c27110.1	-0.120946654	-0.032361385	0.404013366	0.97754057	Potri.014G097300.1	AT1G01350.1
Potra2n14c27111.3	-0.092285999	0.167296015	0.546581074	0.775618506	Potri.014G097600.1	AT3G61690.1
Potra2n14c27112.1	-0.304968624	-0.403296068	0.187237972	0.807509123	Potri.014G097700.1	AT5G08610.1
Potra2n14c27113.1	0.110289016	-0.182107941	0.552962266	0.8818744	Potri.014G097800.4	AT2G46420.1
Potra2n14c27114.3	8.904281659	5.714366377	2.98583E-13	0.209237269	Potri.014G097900.2	AT5G53130.1
Potra2n14c27115.1	2.476290371	NA	NA	NA	Potri.014G098000.1	AT5G59310.1
Potra2n14c27116.1	0.082425894	-0.01897897	0.577767951	0.983113084	Potri.014G098100.1	AT4G01000.1
Potra2n14c27117.1	2.740987495	4.566350014	0.001425404	0.211345761	Potri.014G098200.1	AT2G46495.1
Potra2n14c27118.1	-0.291461271	0.054526007	0.164942895	0.975578749	Potri.014G098400.1	AT3G61740.2
Potra2n14c27119.2	-0.927201674	-0.193703824	0.000242708	0.912777843	Potri.014G098600.2	AT1G01320.2
Potra2n14c27120.1	-0.929539671	-0.193286631	0.000241788	0.912975882	Potri.014G098700.1	AT3G61750.1
Potra2n14c27121.1	-1.024763084	0.161080388	0.028705239	0.948834049	Potri.014G098800.1	AT2G45360.1
Potra2n14c27122.1	-0.235654969	-0.190224803	0.18349367	0.895560236	Potri.014G099000.1	AT2G46500.1
Potra2n14c27123.1	0.20536737	0.340752716	0.156729215	0.631834086	Potri.014G099100.1	AT3G61780.1
Potra2n14c27124.1	-0.541808522	0.031380608	0.000582118	0.983446961	Potri.014G099200.1	AT3G61790.1
Potra2n14c27125.1	0.034822267	0.112494821	0.85249903	0.913842367	Potri.014G099300.1	AT3G61800.1
Potra2n14c27126.1	-0.797290363	-0.514626338	0.075515365	0.834823243	Potri.014G099400.1	AT1G01300.1
Potra2n14c27127.1	-0.323540868	0.00330899	0.376014366	0.999223888	Potri.014G099500.1	AT1G01290.2
Potra2n14c27128.1					Potri.002G171800.1	AT1G01280.1
Potra2n14c27129.1	0.776065458	0.159139672	0.002307679	0.816392132	Potri.014G099700.1	AT1G01260.2
Potra2n14c27130.1	2.347562855	-1.465929376	0.48855768	NA		
Potra2n14c27131.1	1.459962178	0.674963689	NA	NA	Potri.014G099800.1	AT4G00310.2
Potra2n14c27132.1	-0.081857642	-1.724928411	NA	NA	Potri.014G099900.1	AT1G01250.1

Potra2n14c27133.1	0.283972422	0.033665665	0.020994765	0.972336961	Potri.014G100000.1	AT2G46520.1
Potra2n14c27134.2	-1.386691644	-0.023395002	3.47191E-05	0.992934677	Potri.014G100100.5	AT2G46530.1
Potra2n14c27135.1	1.944135754	0.42486454	1.08784E-11	0.71411664	Potri.014G100200.1	AT4G27810.1
Potra2n14c27136.1	-0.15661986	-0.152582403	0.680182456	0.943819961	Potri.002G173101.1	AT2G46540.1
Potra2n14c27137.1	-0.243478934	0.025285018	0.067718765	0.985393296	Potri.014G100300.3	AT2G46550.1
Potra2n14c27138.1	-0.720269912	-0.378619113	1.37166E-06	0.592872379	Potri.014G100400.2	AT4G10840.1
Potra2n14c27139.1	-0.58057844	-0.219791694	0.003871446	0.909675463	Potri.014G100500.1	AT4G34860.1
Potra2n14c27140.1	-0.198970424	0.389563197	0.571905131	0.817407708	Potri.014G100600.1	AT2G46570.1
Potra2n14c27141.1	-0.266462203	0.413202193	0.553819364	0.843683654		
Potra2n14c27142.1	-0.05100329	0.222096366	0.81209402	0.854309045	Potri.014G100700.3	AT2G46580.1
Potra2n14c27143.1	5.673665746	-0.599587173	0.000630981	0.965344432	Potri.014G100800.1	AT2G16720.1
Potra2n14c27144.1	1.692945767	-0.073025885	6.79773E-09	0.971537796	Potri.014G100900.1	AT3G61850.1
Potra2n14c27145.2	-0.485428678	-0.03629634	0.000228406	0.983446961	Potri.014G101000.1	AT1G01230.1
Potra2n14c27146.1	3.636265227	1.363811008	0.41570728	NA	Potri.014G101100.1	AT2G35930.1
Potra2n14c27147.1	NA	NA	NA	NA	Potri.014G101200.1	AT4G00910.1
Potra2n14c27148.1	0.153135307	-0.502051199	0.650960692	0.712599153	Potri.014G101300.1	AT1G01225.1
Potra2n14c27149.1	0.63129364	0.08358704	4.29266E-06	0.954098985	Potri.014G101400.3	AT1G01220.1
Potra2n14c27150.1	-0.49784329	-0.567837169	0.563688429	0.910815461	Potri.014G101500.1	AT3G18750.1
Potra2n14c27151.1	-2.072475262	-0.216340424	0.001354755	0.954559968	Potri.014G101600.1	AT4G27745.1
Potra2n14c27152.1	2.030494426	0.73287685	0.005863438	0.845379753	Potri.014G101700.1	AT2G46600.1
Potra2n14c27153.1	-0.237545281	-0.05812926	0.234706074	0.966333508	Potri.002G175000.2	AT3G61860.1
Potra2n14c27154.2	0.38045816	0.035455234	0.042624232	0.98177802	Potri.014G101900.1	AT4G00900.1
Potra2n14c27155.1	1.195370608	0.283008556	6.54347E-12	0.792864576	Potri.014G102000.1	AT1G76390.1
Potra2n14c27156.1	-0.138631564	-0.09990115	0.568783484	0.924256651	Potri.014G102100.1	AT4G09650.1
Potra2n14c27157.1	-0.412552196	-0.339163307	0.093097654	0.809914834	Potri.014G102200.1	AT1G01200.1
Potra2n14c27158.1	0.395344122	0.505512177	0.044753363	0.549719769	Potri.014G102300.1	AT2G46620.1
Potra2n14c27159.1	-0.052532004	-0.050463173	0.92931414	0.987288338		
Potra2n14c27160.1	-0.0397381	-0.22617433	0.863953054	0.816567218	Potri.014G102400.1	AT3G61870.1
Potra2n14c27161.1	0.797291129	-0.762779109	0.24483259	0.833952007	Potri.002G175400.1	AT2G46630.1
Potra2n14c27162.2	0.32085462	0.385211728	0.698136303	0.909754446	Potri.014G103100.1	
Potra2n14c27163.1	0.5429655	-0.156521144	0.127731193	0.948577865	Potri.014G103300.1	AT2G46690.1
Potra2n14c27164.1	4.369177244	NA	0.212779404	NA	Potri.014G103400.1	

Potra2n14c27165.1	-0.20672255	0.030854713	0.057202028	0.981702181	Potri.014G103500.6	AT2G46700.1
Potra2n14c27166.1	-0.782920625	-0.297801703	2.55603E-05	0.824448251	Potri.002G176800.1	AT2G46710.1
Potra2n14c27167.1	1.933891244	0.675595962	4.72913E-09	0.633830624	Potri.014G103700.1	AT1G32640.1
Potra2n14c27168.2	-0.148998038	-0.067996906	0.276468457	0.941260209	Potri.002G177500.1	AT1G01180.1
Potra2n14c27169.2	0.083447742	0.119134488	0.481974102	0.845379753	Potri.014G104000.3	AT4G00840.1
Potra2n14c27170.1	0.613702601	2.510275855	0.813360459	0.585240923	Potri.014G104100.1	
Potra2n14c27171.1	0.901285982	1.003616434	0.00619196	0.364341782	Potri.014G104200.5	AT1G01140.1
Potra2n14c27172.1	1.545832463	NA	NA	NA	Potri.014G104300.1	AT1G01120.1
Potra2n14c27173.1	0.016668616	-0.032857276	0.885367595	0.954247482	Potri.014G104500.10	AT4G00830.2
Potra2n14c27174.1	-1.22090656	-0.83700239	0.080565157	0.621832412	Potri.002G178500.1	AT4G00820.1
Potra2n14c27175.1	0.376684857	-0.32159535	0.268403088	0.930059926	Potri.014G104700.1	AT5G26667.2
Potra2n14c27176.1	-2.203869162	-0.70400926	NA	NA		
Potra2n14c27177.1	-0.47373932	-0.265647718	0.165093203	0.869327705	Potri.014G050300.1	AT1G02150.1
Potra2n14c27178.1	0.55552751	-0.111708558	0.013788627	0.966077009	Potri.014G104800.1	AT2G46770.1
Potra2n14c27179.1	1.338437451	1.172988829	0.288713918	0.833667976	Potri.014G104900.1	AT3G61920.1
Potra2n14c27180.1	-1.729718954	-1.430225213	0.502611202	NA	Potri.014G105000.1	AT3G61930.1
Potra2n14c27181.1	-0.668026313	-0.210467803	2.25544E-08	0.720242695	Potri.014G105101.1	AT2G46780.1
Potra2n14c27182.1	NA	NA	NA	NA	Potri.002G179300.1	AT1G05600.2
Potra2n14c27183.1	0.474214594	-0.041262005	0.007258586	0.981772014	Potri.014G105400.1	AT5G24510.1
Potra2n14c27184.1	0.052680704	0.09244006	0.748134767	0.876231686	Potri.014G105600.1	AT4G00800.1
Potra2n14c27185.1	0.148630062	0.118032801	0.508665705	0.940255038	Potri.014G105800.1	AT1G01080.1
Potra2n14c27186.1	0.626330247	0.081313652	0.000103974	0.956865545	Potri.014G105900.1	AT4G01400.3
Potra2n14c27187.1	-0.187607305	0.141160611	0.09957117	0.925184207	Potri.014G106000.1	AT5G24470.1
Potra2n14c27188.1	0.075088464	-2.899343921	0.985527201	NA	Potri.014G106100.1	AT4G01410.1
Potra2n14c27189.1	-0.209224825	0.08804958	0.34752054	0.947969749	Potri.014G106200.1	AT2G46800.2
Potra2n14c27190.1	NA	0.674963689	NA	NA		
Potra2n14c27191.1	2.492428534	NA	NA	NA	Potri.014G106300.1	AT2G46810.1
Potra2n14c27192.1	-0.364721621	-0.550618614	0.084383721	0.279083157	Potri.014G106400.3	AT2G46820.2
Potra2n14c27193.1	0.286966096	-0.526832022	0.235789489	0.477503371	Potri.014G106500.1	AT5G54160.1
Potra2n14c27194.1	7.631409459	1.208691619	1.14539E-35	0.854980698	Potri.014G106600.1	AT5G54160.1
Potra2n14c27195.1	NA	NA	NA	NA	Potri.014G106700.1	AT4G10490.1
Potra2n14c27196.1	-0.11539379	0.676970166	0.582045415	0.619575855	Potri.014G106800.12	AT1G01060.4

Potra2n14c27197.1	-0.310212275	-0.25344639	0.283108893	0.799758281	Potri.014G107000.1	AT3G61960.1
Potra2n14c27198.2	0.274260974	-0.226488415	0.081795947	0.863165286	Potri.014G107100.1	AT1G01050.1
Potra2n14c27199.4	3.620827449	2.878643434	0.228924926	NA	Potri.002G181600.1	AT2G46870.1
Potra2n14c27200.1	0.318306149	0.202090598	0.176127517	0.86212354	Potri.014G107300.10	AT4G01510.1
Potra2n14c27201.1	-1.864134742	-2.013568191	NA	NA	Potri.014G107400.1	
Potra2n14c27202.1	1.51491104	0.531887728	2.29223E-10	0.70570456	Potri.014G107500.2	AT4G35580.3
Potra2n14c27203.1	1.475920347	1.117191964	NA	NA	Potri.014G108000.1	AT4G35580.2
Potra2n14c27204.3	1.178785759	0.611239294	9.69995E-07	0.531649858	Potri.014G108100.1	AT5G24590.2
Potra2n14c27205.1	-0.528060627	-0.16858011	0.002242185	0.935474933	Potri.014G108200.1	AT5G54130.2
Potra2n14c27206.1	-0.680461251	-0.222438185	4.3565E-05	0.865164113	Potri.014G108400.1	AT4G01560.1
Potra2n14c27207.1	-0.845092559	-0.246004474	1.24527E-05	0.86716397	Potri.014G108300.1	AT2G41610.1
Potra2n14c27208.1	NA	NA	NA	NA	Potri.014G108500.1	AT3G30340.1
Potra2n14c27209.1	-0.634647916	-0.371864216	0.001182445	0.605147753	Potri.014G108600.1	AT4G01575.1
Potra2n14c27210.1	0.407541923	-0.423255316	0.840255966	NA	Potri.014G108700.1	AT3G61980.1
Potra2n14c27211.1	4.943105415	NA	0.026842211	NA		
Potra2n14c27212.1	0.82357019	NA	NA	NA	Potri.014G108900.1	AT3G61980.1
Potra2n14c27213.1	2.115384252	NA	NA	NA	Potri.014G109000.1	AT3G61980.1
Potra2n14c27214.1	-0.220368702	0.059138654	0.561140587	0.977559521	Potri.014G109100.1	AT5G63140.1
Potra2n14c27215.1	-1.311568144	-0.607118459	2.47631E-15	0.663967121	Potri.014G109200.1	AT2G46890.1
Potra2n14c27216.1	-0.217388658	-0.063145345	0.124775459	0.930871994	Potri.014G109400.1	AT1G02280.2
Potra2n14c27217.1	-0.290664545	-0.243677684	0.087969543	0.674283226		
Potra2n14c27218.1	-0.054021647	0.052871696	0.683895738	0.957525726	Potri.014G109500.1	AT2G46900.1
Potra2n14c27219.3	-0.115637625	0.030429382	0.451186678	0.983446961	Potri.014G109600.1	AT1G02290.1
Potra2n14c27220.1	-0.704309123	0.029075613	0.000312102	0.985393296	Potri.014G109700.1	AT1G28200.1
Potra2n14c27221.1	-0.026829151	0.099190188	0.861101651	0.950878725	Potri.002G184300.3	AT1G02305.1
Potra2n14c27222.1	-0.072160241	0.088418419	0.605113948	0.956865545		
Potra2n14c27223.3	0.475553742	0.394949597	0.088809244	0.734057255	Potri.014G109900.1	AT3G62010.1
Potra2n14c27224.1	-2.922504018	-0.555493111	0.209013551	NA	Potri.014G110000.1	AT1G27680.1
Potra2n14c27225.1	0.086717289	-0.039636692	0.469547474	0.963878456	Potri.014G110200.1	AT1G02330.1
Potra2n14c27226.2	-2.046914277	-0.18062369	0.565627623	NA	Potri.014G110300.1	AT5G03050.1
Potra2n14c27227.1	-0.24949599	-0.301256928	0.197736404	0.727440762	Potri.014G110400.1	AT3G62020.1
Potra2n14c27228.1	-0.399138137	-0.111976738	0.005706808	0.88643982	Potri.014G110500.1	AT2G46920.1

Potra2n14c27229.1	-0.367843605	-0.127521581	0.019972953	0.897186976	Potri.014G110600.1	AT4G01650.1
Potra2n14c27230.1	-0.657947797	-0.321508913	0.085562922	0.848005416	Potri.014G110700.1	
Potra2n14c27231.1	-0.651704521	-0.437413379	0.158223016	0.811159921	Potri.014G110800.3	AT3G62040.1
Potra2n14c27232.1	-1.573998819	-2.446267299	NA	NA		
Potra2n14c27233.2	0.323910093	0.263588707	0.102421525	0.737914109	Potri.014G110900.1	AT3G62060.1
Potra2n14c27234.1	-0.117286045	0.128153369	0.687267741	0.833401866	Potri.014G111000.1	AT2G46940.1
Potra2n14c27235.1	0.767881406	0.161416844	9.98407E-06	0.89321847	Potri.014G111100.1	AT1G07830.1
Potra2n14c27236.1	-0.160923828	-0.02305846	0.5585646	0.992934677	Potri.014G111200.1	AT4G01680.1
Potra2n14c27237.2	-1.128937472	1.430840659	0.347941625	0.754074409	Potri.014G111400.1	AT1G09530.2
Potra2n14c27238.1	0.842987321	-0.255200849	0.190210266	0.936890993	Potri.014G111500.1	AT2G46980.2
Potra2n14c27239.1	-0.545474944	0.049998325	0.025144753	0.9761362	Potri.014G111600.3	AT4G01690.1
Potra2n14c27240.1	0.161794316	-0.142820638	0.732240473	0.949847686	Potri.014G111700.1	AT3G62100.1
Potra2n14c27241.1	1.469711991	1.287837313	0.013752024	0.4057897	Potri.014G111800.1	AT4G01700.1
Potra2n14c27242.1	2.062319254	-0.062656001	3.77544E-15	0.981761466	Potri.014G111900.1	AT4G01720.1
Potra2n14c27243.1	NA	NA	NA	NA		
Potra2n14c27244.1	-0.608665358	-0.31511277	0.059031875	0.824905557	Potri.014G112000.1	AT4G01730.1
Potra2n14c27245.1	-0.638311646	-0.167024669	0.000966052	0.900869139	Potri.014G112100.1	AT3G62110.1
Potra2n14c27246.1	-0.259064588	-0.079691264	0.059760999	0.935474933	Potri.014G112266.1	AT4G01800.1
Potra2n14c27247.1	-0.206636319	-0.077019924	0.13442141	0.937846405	Potri.014G112200.1	AT4G01790.1
Potra2n14c27248.1	0.385036061	0.085451861	0.000852292	0.896353602	Potri.014G112400.1	AT3G62120.1
Potra2n14c27249.8	0.151367618	0.118612237	0.398953238	0.828625141	Potri.014G112600.1	AT4G01810.1
Potra2n14c27250.1	-0.030990954	0.034247965	0.837164216	0.974782554	Potri.014G112700.1	AT3G62130.1
Potra2n14c27251.1	-0.511822617	-0.097713106	0.099169479	0.970817579	Potri.014G113000.1	AT2G47000.1
Potra2n14c27252.5	0.85677188	1.214617845	0.000263222	0.050626449	Potri.014G113100.1	AT1G02520.1
Potra2n14c27253.1	-0.655741707	-0.339024711	0.016309779	0.856891638	Potri.014G113600.1	AT3G62160.1
Potra2n14c27254.1	-0.144875673	-0.141493811	0.490952944	0.922029226	Potri.014G113700.1	AT4G01840.1
Potra2n14c27255.2	-0.157208586	-0.060531566	0.442181965	0.9652407	Potri.003G207100.1	AT1G76320.1
Potra2n14c27256.1	0.095985264	0.222181872	0.590726756	0.93154079	Potri.014G113900.1	AT1G75250.1
Potra2n14c27257.2	-0.071619376	0.035794023	0.664316293	0.978203092	Potri.014G114000.1	AT2G47020.1
Potra2n14c27258.2	0.49232719	0.331738923	1.29837E-05	0.61887385	Potri.019G052900.1	AT3G17450.1
Potra2n14c27259.2	0.209769579	-0.046837363	0.114943811	0.970432679	Potri.014G114200.1	AT3G62220.1
Potra2n14c27260.1	-0.26845922	-0.11353764	0.015100635	0.835819291	Potri.014G114300.1	AT3G60030.1

Potra2n14c27261.1	-0.61910233	0.64247635	NA	NA	Potri.014G114400.1	AT5G33340.1
Potra2n14c27262.1	NA	NA	NA	NA	Potri.014G114500.1	AT4G34480.1
Potra2n14c27263.1						
Potra2n14c27264.2	-0.658856851	-0.296172275	0.000658897	0.821709751	Potri.002G189200.1	AT4G01850.1
Potra2n14c27265.1	NA	NA	NA	NA	Potri.002G189450.1	AT3G15580.1
Potra2n14c27266.2	0.325656672	0.075078322	0.000318163	0.927547456	Potri.014G114900.1	AT3G62240.1
Potra2n14c27267.1	0.392680402	0.072319008	0.006957293	0.954559968	Potri.014G115000.1	AT1G10550.1
Potra2n14c27268.1	0.57589208	0.140051304	0.000161945	0.915488947	Potri.014G115100.1	AT2G47110.1
Potra2n14c27269.1	2.257303534	0.686323087	2.11146E-16	0.820074794	Potri.014G115200.1	AT2G47115.1
Potra2n14c27270.1	-2.531819517	-0.146545311	0.005796648	0.979775311	Potri.014G115300.1	AT2G47140.1
Potra2n14c27271.2	0.648614648	0.135273614	4.65464E-07	0.936360532	Potri.014G115400.1	AT1G02475.1
Potra2n14c27272.1	0.8875368	0.191795373	5.16663E-05	0.829781359	Potri.014G115500.1	AT3G62260.2
Potra2n14c27273.1	0.40958193	0.072853892	0.006973609	0.937276488	Potri.014G115600.1	AT5G26751.1
Potra2n14c27274.1	-1.864134742	-2.013568191	NA	NA	Potri.014G115700.1	AT1G02460.1
Potra2n14c27275.1	9.126341576	5.63745305	3.73305E-17	0.030956956	Potri.014G115800.1	
Potra2n14c27276.1	NA	NA	NA	NA	Potri.014G115900.1	
Potra2n14c27277.1	-0.028950116	-0.227687455	0.947072939	0.79078676	Potri.014G116000.1	AT3G62270.1
Potra2n14c27278.1	NA	NA	NA	NA	Potri.014G116100.1	AT3G62280.1
Potra2n14c27279.1						
Potra2n14c27280.3	-0.078204049	-0.068049893	0.638516404	0.953177702	Potri.002G191400.2	AT1G10630.1
Potra2n14c27281.1	0.046092905	0.1337537	0.698606408	0.820063065		
Potra2n14c27282.1	-1.125155912	-0.616046331	0.757677194	NA	Potri.014G116800.1	AT2G47180.1
Potra2n14c27283.1	5.523359445	2.484574128	2.1613E-14	0.07535679	Potri.014G117000.1	AT2G47190.1
Potra2n14c27284.4	-1.447645392	-0.967424081	0.230783543	0.837874974	Potri.014G117100.1	AT5G19730.1
Potra2n14c27285.1	-0.384600365	-1.147771674	0.900978295	NA	Potri.014G117200.1	
Potra2n14c27286.1	-0.473996868	-0.426423014	0.398641834	0.925184207	Potri.014G117300.1	AT1G02400.1
Potra2n14c27287.1	0.075411002	-0.054704597	0.54182728	0.939824856	Potri.014G117400.1	AT2G47210.1
Potra2n14c27288.1	-0.003380206	0.082816357	0.989650924	0.956865545	Potri.014G117500.1	AT4G01935.1
Potra2n14c27289.1	0.451155308	0.345654766	0.026402141	0.592872379	Potri.014G117600.1	AT1G12700.1
Potra2n14c27290.1	-0.408544729	-0.040793346	0.001290874	0.9761362	Potri.014G117700.5	AT4G01940.1
Potra2n14c27291.1	-0.06261134	0.021072198	0.529467077	0.986636967	Potri.014G117800.1	AT2G47230.1
Potra2n14c27292.1	0.040437313	0.586757891	NA	NA		

Potra2n14c27293.1	-0.074654224	0.038696117	0.470964858	0.953831244	Potri.014G117900.1	AT3G62310.1
Potra2n14c27294.1	-0.323441581	-0.010884066	0.065721023	0.992934677	Potri.014G118000.2	AT1G02380.1
Potra2n14c27295.2	0.088753434	0.279212903	0.631494964	0.773063484	Potri.014G118100.1	AT3G62330.1
Potra2n14c27296.1	-0.193965026	0.090269605	0.608012824	0.960981446		
Potra2n14c27297.2	0.991616452	0.566484596	0.004948208	0.715585895	Potri.014G118200.1	AT2G47260.1
Potra2n14c27298.1	0.126308302	-1.465929376	NA	NA	Potri.014G118300.1	AT2G47270.1
Potra2n14c27299.1	-2.472898378	-0.038832101	5.20276E-06	0.991732307	Potri.014G118400.2	AT4G01970.1
Potra2n14c27300.1	0.016507416	0.048874628	0.889896222	0.930813462	Potri.014G118500.1	AT2G31400.1
Potra2n14c27301.2	0.38484601	0.26234125	0.016692192	0.704368358	Potri.014G118600.1	AT3G62370.1
Potra2n14c27302.1	-0.363704347	-0.23633949	0.199637369	0.91540496	Potri.014G119000.1	AT1G02370.1
Potra2n14c27303.1	-0.450515769	-0.174332913	9.80684E-07	0.816392132	Potri.014G119100.1	AT4G02010.1
Potra2n14c27304.1	-1.824976639	-2.032694279	0.113996113	0.276620704		
Potra2n14c27305.1	-0.460643507	-2.05258594	NA	NA		
Potra2n14c27306.1						
Potra2n14c27307.2	-0.177936694	0.057189755	0.161424133	0.943483421	Potri.014G119600.1	AT2G47350.1
Potra2n14c27308.1	0.103883148	-0.005909487	0.55232441	0.996032145	Potri.014G119700.1	AT1G02560.1
Potra2n14c27309.1	0.618215064	0.667504722	0.015414037	0.701287572	Potri.014G119800.1	AT2G34830.1
Potra2n14c27310.1	-1.040749799	-0.545795098	1.41095E-05	0.674175338	Potri.014G119900.1	AT2G47360.1
Potra2n14c27311.1	-1.170618407	-1.435550125	0.752190722	NA	Potri.014G120000.2	AT3G03450.1
Potra2n14c27312.1	-0.710414975	0.120381807	3.60519E-07	0.912777843	Potri.014G120100.2	AT4G02020.1
Potra2n14c27313.1	-0.809507072	0.12644827	0.000788766	0.941677175	Potri.014G120200.1	AT1G78710.1
Potra2n14c27314.1						
Potra2n14c27315.1	0.448099481	0.187672158	0.015495131	0.834112275	Potri.014G120300.2	AT2G16760.1
Potra2n14c27316.1	0.401972804	0.224362819	0.073001509	0.819950844	Potri.014G120300.2	AT2G16760.1
Potra2n14c27317.1	0.210349658	-0.097849263	0.138297539	0.923746681	Potri.014G120500.1	AT5G61310.2
Potra2n14c27318.1	-0.254690429	-0.071932328	0.061916249	0.950095151	Potri.002G196000.1	AT2G47390.1
Potra2n14c27319.1	-3.757171646	-1.489122283	3.46964E-09	0.247573674	Potri.014G120700.1	AT3G62410.1
Potra2n14c27320.1	0.380130528	0.100070329	0.068959496	0.954325106	Potri.014G120800.1	AT3G62420.1
Potra2n14c27321.1	-0.129126928	0.032567308	0.464717814	0.978485478	Potri.014G120900.1	AT4G02030.1
Potra2n14c27322.1	0.778849037	-0.491930846	0.10092752	0.811159921	Potri.014G121000.1	AT4G02060.1
Potra2n14c27323.1	NA	NA	NA	NA	Potri.014G196100.1	
Potra2n14c27324.1					Potri.014G196100.1	

Potra2n14c27325.1	-0.132653599	0.075849318	0.279001598	0.935995049	Potri.014G121100.1	AT2G47410.1
Potra2n14c27326.3	0.362320249	0.044593104	0.017930531	0.963225999	Potri.014G121500.1	AT2G47430.1
Potra2n14c27327.1	0.413691669	-0.082717633	0.034785918	0.930598202	Potri.014G121200.1	AT2G47420.1
Potra2n14c27328.1	0.671717415	0.143985329	0.005090442	0.94076214	Potri.014G121300.1	AT1G47820.2
Potra2n14c27329.1	-0.049743725	0.200711194	0.859789236	0.878370858	Potri.T171400.1	AT4G02070.1
Potra2n14c27330.1	0.448012589	-0.541867609	0.566780083	0.910815461	Potri.014G121200.1	AT2G47420.1
Potra2n14c27331.1	0.933834211	1.048236367	0.531562089	0.857418365	Potri.014G121400.2	AT3G62470.1
Potra2n14c27332.1	0.7959733	1.150686305	0.529175037	0.754292559	Potri.014G121300.1	AT1G47820.2
Potra2n14c27333.1	-0.145882149	0.119865319	0.245251711	0.937687311	Potri.T171400.1	AT4G02070.1
Potra2n14c27334.1	-0.829346803	0.249188029	0.001356914	0.930871994	Potri.014G121900.1	AT1G02610.1
Potra2n14c27335.1	-1.852007946	-0.803777935	0.262372941	0.936402128	Potri.014G122000.1	AT3G62550.1
Potra2n14c27336.1	0.157374504	-0.312712335	0.813464476	0.924251575	Potri.014G122100.1	AT4G02090.1
Potra2n14c27337.2	-2.06614492	-0.44269363	1.05529E-08	0.784845657	Potri.014G122200.1	AT1G02640.1
Potra2n14c27338.1	NA	NA	NA	NA		
Potra2n14c27339.1	-0.730560869	-0.291445262	0.007716424	0.792544431	Potri.014G122300.1	AT4G02100.1
Potra2n14c27340.2	1.058908488	-0.331345116	4.80971E-05	0.770451625	Potri.014G122400.1	AT4G02110.1
Potra2n14c27341.1	0.12124991	0.26711644	0.488288044	0.821709751	Potri.014G122500.1	AT1G02680.1
Potra2n14c27342.1	0.750252861	0.164402992	4.96901E-06	0.902857376	Potri.014G122600.1	AT3G62600.1
Potra2n14c27343.1	-2.332245436	-0.230763965	0.369260697	NA	Potri.014G122700.1	AT5G49330.1
Potra2n14c27344.1	-1.446466166	0.326238711	0.704501169	NA		
Potra2n14c27345.1	0.434398137	-0.006410153	0.000646281	0.996032145	Potri.014G122800.1	AT2G47470.1
Potra2n14c27346.1	-0.012142278	0.039448281	0.96650991	0.982422288	Potri.014G122900.1	AT2G25920.1
Potra2n14c27347.1	0.120789533	0.090230203	0.764165417	0.972159498	Potri.014G123000.1	AT3G55010.1
Potra2n14c27348.1	0.198814269	0.046931735	0.654886259	0.985393296	Potri.014G123100.1	AT3G26400.1
Potra2n14c27349.1	0.093237552	0.061055393	0.545618023	0.936549944	Potri.014G123200.1	AT4G02120.1
Potra2n14c27350.1	2.059840642	0.433792351	0.006176095	0.894138111	Potri.014G123400.1	AT5G38700.1
Potra2n14c27351.1	1.758341361	-0.079512623	0.714234031	NA	Potri.014G123500.1	AT5G61710.1
Potra2n14c27352.1	-1.043078936	-2.424354098	0.381577267	0.389989789	Potri.014G123600.1	AT2G47480.1
Potra2n14c27353.1	-2.174001837	-0.956643037	0.090509788	0.82876584	Potri.014G123700.1	AT1G67030.1
Potra2n14c27354.10	0.020393914	0.143843364	0.874562823	0.799601397	Potri.014G123800.1	AT5G47900.1
Potra2n14c27355.1	-0.478881675	-0.138333601	0.030127257	0.94787297	Potri.014G124200.1	AT3G06720.1
Potra2n14c27356.1	-0.151484298	-0.377597508	0.911452368	0.965230114	Potri.014G124300.1	AT4G13030.1

Potra2n14c27357.1	1.104371899	0.298139954	2.37066E-09	0.830056077	Potri.014G124400.1	AT5G49300.1
Potra2n14c27358.1	-0.817093583	0.485842992	0.250639102	0.87062349	Potri.014G124500.1	AT1G02700.1
Potra2n14c27359.1	0.22388555	-2.42671106	0.957841838	NA		
Potra2n14c27360.1	3.029147424	1.265752828	0.000296335	0.729969215	Potri.014G124550.1	
Potra2n14c27361.1	2.685015931	NA	NA	NA		
Potra2n14c27362.1	0.157992191	0.115884657	0.731140447	0.960981446	Potri.014G124900.1	AT3G62650.1
Potra2n14c27363.1	NA	NA	NA	NA		
Potra2n14c27364.1	-0.350194359	-0.169667348	0.099161034	0.885649686	Potri.014G125000.1	AT3G62660.1
Potra2n14c27365.1	0.70823665	-0.514722412	0.089365112	0.834560473	Potri.014G125100.1	AT1G02730.1
Potra2n14c27366.1	-1.297830546	-0.586027411	0.001492048	0.138040185	Potri.014G125300.1	AT4G16120.1
Potra2n14c27367.1	-0.134691595	0.12886783	0.85488664	0.97950444	Potri.014G125400.1	AT1G67710.1
Potra2n14c27368.2	0.472216973	1.130860628	0.595628016	0.810185146		
Potra2n14c27369.1	0.978533667	0.318153043	4.59039E-07	0.655802209	Potri.014G125500.3	AT3G05700.1
Potra2n14c27370.1	1.459962178	NA	NA	NA		
Potra2n14c27371.1	-0.054758142	-0.207943943	0.708927082	0.68282906	Potri.014G125600.1	AT2G47490.1
Potra2n14c27372.1	-0.9010305	-0.28760237	0.00046551	0.80654978	Potri.014G125700.1	AT2G47500.1
Potra2n14c27373.1	1.790181641	1.623358995	0.105160952	0.443657809	Potri.005G020300.1	AT1G56300.1
Potra2n14c27374.1	1.865976368	1.522532963	0.138043635	0.527469948	Potri.014G126000.2	AT5G64260.1
Potra2n14c27375.5	0.062472747	0.346282772	0.934302315	0.895929932	Potri.002G201400.1	AT1G33390.1
Potra2n14c27376.1	2.89436961	0.660662525	2.58748E-21	0.792864576	Potri.014G126100.1	AT2G47520.1
Potra2n14c27377.1	NA	NA	NA	NA	Potri.014G126200.1	AT2G47540.1
Potra2n14c27378.1	NA	NA	NA	NA	Potri.014G126200.1	AT2G47540.1
Potra2n14c27379.1	NA	NA	NA	NA	Potri.014G126500.2	AT2G47540.1
Potra2n14c27380.1	NA	NA	NA	NA		
Potra2n14c27381.1	-0.61910233	-0.767708831	NA	NA	Potri.014G126200.1	AT2G47540.1
Potra2n14c27382.1	0.084723017	-0.244334511	0.802617534	0.811104553	Potri.014G126600.1	
Potra2n14c27383.1	-0.019786303	-0.11755751	0.947527277	0.904328952	Potri.014G126700.1	AT4G02260.2
Potra2n14c27384.1	1.64774763	NA	NA	NA	Potri.014G126800.1	AT4G02280.1
Potra2n14c27385.2	-0.380914876	-0.620756015	0.793252803	0.940522228	Potri.014G126900.1	AT4G02290.1
Potra2n14c27386.1	-1.107498672	0.166561979	0.043009087	0.971126663	Potri.014G127000.1	AT1G02810.1
Potra2n14c27387.1	-1.261915808	0.121961441	0.006062122	0.981772014	Potri.014G127000.1	AT1G02810.1
Potra2n14c27388.1	-0.958801653	-0.161562908	1.85014E-06	0.910878765	Potri.014G127200.1	AT4G02340.1

Potra2n14c27389.1	0.45642159	0.012992172	0.004867519	0.993815835	Potri.014G127300.2	AT3G05590.1
Potra2n14c27390.1	-0.174348674	-0.073582618	0.210136446	0.922618347	Potri.014G127400.1	AT4G02350.1
Potra2n14c27391.1	0.758056671	-0.121531099	0.00404239	0.943483421	Potri.014G127600.2	AT1G02816.1
Potra2n14c27392.1	-0.024619753	1.298093716	0.990124907	NA		
Potra2n14c27393.1	-0.867179375	-0.327036126	0.199337801	0.922011871	Potri.014G127700.1	AT4G02380.1
Potra2n14c27394.1	-0.040016113	0.025523807	0.813464476	0.98177802	Potri.014G127800.1	AT2G47580.1
Potra2n14c27395.1	0.211462415	0.199370247	0.138096135	0.788065047	Potri.014G127900.1	AT3G52860.1
Potra2n14c27396.1	-0.550861101	-1.109863537	0.796981798	NA	Potri.014G128000.1	AT4G02390.1
Potra2n14c27397.1						
Potra2n14c27398.1	-0.222299121	0.01707318	0.136891696	0.9921442	Potri.014G128200.1	AT4G02390.1
Potra2n14c27399.1	0.102236182	-0.767708831	NA	NA		
Potra2n14c27400.1	1.196956486	0.340078353	1.20089E-14	0.730733927	Potri.014G128300.1	AT4G02400.1
Potra2n14c27401.1	-0.136716464	0.004374936	0.367377299	0.996154416	Potri.014G128400.1	AT3G53760.1
Potra2n14c27402.1	0.28515983	-0.145519258	0.142482607	0.931774514	Potri.002G204000.3	AT2G47590.1
Potra2n14c27403.1	1.508694885	0.321949904	1.52794E-10	0.763747409	Potri.014G128600.1	AT2G47600.1
Potra2n14c27404.1	0.659343054	0.105010525	0.000234271	0.937846405	Potri.014G128900.4	AT3G62880.1
Potra2n14c27405.1	0.682135245	0.103112129	0.000193214	0.940556596	Potri.014G128800.1	AT3G05560.2
Potra2n14c27406.1	1.64774763	NA	NA	NA		
Potra2n14c27407.1	-2.353307976	1.001524113	0.015220621	0.831597503	Potri.014G129000.1	AT3G62860.1
Potra2n14c27408.1	-0.00348339	-0.050963167	0.982926159	0.963557834	Potri.002G204300.1	AT2G47640.3
Potra2n14c27409.1	1.099628937	0.142487446	8.96796E-07	0.930159505	Potri.014G129200.1	AT3G62830.2
Potra2n14c27410.2	0.272304657	-0.017614021	0.055555734	0.985393296	Potri.014G129500.1	AT4G02425.1
Potra2n14c27411.1	0.199830802	0.123874281	0.11441802	0.88661378	Potri.014G129600.11	AT2G47690.1
Potra2n14c27412.1	0.134830336	-0.130742777	0.607457108	0.899964228	Potri.014G129700.1	AT5G61770.3
Potra2n14c27413.1	0.296640413	0.089504908	0.13948642	0.956865545		
Potra2n14c27414.2	0.161892215	0.119866681	0.119759425	0.855432225	Potri.014G129900.1	AT1G02840.3
Potra2n14c27415.1	-0.010983468	0.017151309	0.936772151	0.988240685	Potri.014G130100.4	AT2G47710.1
Potra2n14c27416.1	-0.705524541	0.207524178	0.001211161	0.772458566	Potri.014G130300.1	AT1G02860.1
Potra2n14c27417.2	1.031033533	0.44589648	8.4758E-10	0.736000077	Potri.014G130400.1	AT1G02870.1
Potra2n14c27418.1	-0.865885081	-0.283406643	0.816683064	NA	Potri.014G130500.1	AT2G47800.1
Potra2n14c27419.1	0.001821477	-0.136507643	0.994166747	0.920141992	Potri.014G130600.1	AT2G47790.1
Potra2n14c27420.1	-0.145123703	0.021937144	0.204664222	0.98408134	Potri.002G205700.1	AT5G27720.1

Potra2n14c27421.1	0.076826878	-0.032919072	0.777562457	0.985912891	Potri.014G130800.1	AT4G02460.1
Potra2n14c27422.1	-0.183524451	-0.065994052	0.162870973	0.956865545	Potri.014G130900.3	AT1G02880.3
Potra2n14c27423.1	-0.109935948	0.11956101	0.4310373	0.874009898	Potri.014G131000.2	AT1G02890.1
Potra2n14c27424.1	0.095433767	0.217285808	0.657548884	0.857189843	Potri.014G131200.1	AT4G02485.1
Potra2n14c27425.2	0.095433767	0.217285808	0.657548884	0.857189843	Potri.014G131300.1	AT2G21045.1
Potra2n14c27426.2	0.220705783	0.038895859	0.095125199	0.966501596	Potri.014G131600.1	AT2G47760.1
Potra2n14c27427.1	0.101649619	-0.588692813	0.867285642	0.779077802	Potri.014G131701.1	AT4G15802.1
Potra2n14c27428.1	0.846891143	0.514666126	0.605332844	NA	Potri.014G131800.1	AT1G02900.1
Potra2n14c27429.1	0.028777894	2.296542998	NA	NA	Potri.002G206300.1	AT1G02910.1
Potra2n14c27430.1	-2.963722285	-1.160459429	0.012027104	0.801458793	Potri.014G132000.1	AT5G27690.1
Potra2n14c27431.1	2.32613152	1.950801572	0.36574277	NA		
Potra2n14c27432.1	-0.596033587	-0.565396741	0.176905568	0.733263164	Potri.014G132100.1	AT5G66520.1
Potra2n14c27433.1	2.632524198	NA	NA	NA	Potri.014G132200.1	AT3G62760.1
Potra2n14c27434.1	-0.288846757	-0.109394344	0.117775359	0.933312406	Potri.014G132300.1	AT3G62770.1
Potra2n14c27435.1	-1.104630046	-0.274142865	0.001310303	0.631417984	Potri.014G132400.1	AT1G02970.1
Potra2n14c27436.1	6.041352812	3.693867737	5.42215E-13	0.351090479	Potri.014G132500.1	AT4G13420.1
Potra2n14c27437.1	-5.674972304	0.441128842	9.13916E-05	0.953608209	Potri.014G132600.1	AT2G47810.1
Potra2n14c27438.1					Potri.T034600.2	AT3G50700.1
Potra2n14c27439.2	-0.13910512	-0.022561508	0.288005728	0.978646026		
Potra2n14c27440.1	-0.153977227	-0.008022398	0.132410107	0.992934677	Potri.014G132800.1	AT4G02560.1
Potra2n14c27441.1	0.268125783	0.374630419	0.054476723	0.421699593	Potri.014G132900.1	AT3G62900.1
Potra2n14c27442.1	0.712828937	0.519143789	2.99792E-11	0.221840363	Potri.014G133000.1	AT1G03000.1
Potra2n14c27443.1	-0.069137027	0.19587582	0.883380107	0.909450222	Potri.014G133200.1	AT5G07900.1
Potra2n14c27444.1	-0.655105278	0.25018399	0.000413637	0.88643982	Potri.014G133400.1	AT3G62910.1
Potra2n14c27445.1	-0.498804915	-0.718355589	0.238548643	0.351810165	Potri.014G133500.2	AT1G03010.1
Potra2n14c27446.1	-0.080601831	-0.060184974	0.699368447	0.957163493	Potri.014G133600.2	AT3G62920.1
Potra2n14c27447.1	NA	NA	NA	NA	Potri.014G133700.1	AT3G62930.1
Potra2n14c27448.1	NA	NA	NA	NA	Potri.014G133900.1	AT3G62930.1
Potra2n14c27449.1					Potri.014G134000.1	AT3G62930.1
Potra2n14c27450.1					Potri.014G133700.1	AT3G62930.1
Potra2n14c27451.1	-1.328951947	-1.478536539	NA	NA	Potri.014G133800.1	AT1G03020.1
Potra2n14c27452.1					Potri.014G133900.1	AT3G62930.1

Potra2n14c27453.1	NA	NA	NA	NA	Potri.014G134000.1	AT3G62930.1
Potra2n14c27454.1	0.585915229	0.015372523	0.000147123	0.987891912	Potri.014G134100.1	AT3G62940.2
Potra2n14c27455.1	-1.315387013	-3.362093328	0.70524434	NA	Potri.014G134200.1	AT3G62950.1
Potra2n14c27456.1	NA	0.674963689	NA	NA		
Potra2n14c27457.1	-2.476964973	0.044446126	0.445979555	NA	Potri.014G134300.1	AT2G47880.1
Potra2n14c27458.7	0.025698854	0.102309831	0.896442264	0.939915707	Potri.014G134400.2	AT3G62970.1
Potra2n14c27459.1	0.250717188	-0.126837496	0.818199931	0.986190118	Potri.014G134601.1	AT2G47890.1
Potra2n14c27460.1	2.098203165	0.592745828	8.06317E-31	0.522878464	Potri.014G134700.1	AT4G03180.1
Potra2n14c27461.1	0.701409726	-0.239542381	0.000197195	0.854880262	Potri.014G134900.2	AT3G05030.1
Potra2n14c27462.1	-0.649519348	-0.547316465	0.180006983	0.886281281	Potri.014G135000.1	AT2G47920.1
Potra2n14c27463.1	-2.696502606	-0.380584455	0.000204957	0.934579595	Potri.014G135100.1	AT2G47930.1
Potra2n14c27464.1	0.365180164	0.099195672	0.025687608	0.941855873	Potri.014G135200.3	AT2G47940.1
Potra2n14c27465.1	1.021570057	0.230624314	0.659431659	NA	Potri.014G135300.1	AT5G62000.2
Potra2n14c27466.1	-0.136139211	-0.333487147	0.774106313	0.869327705	Potri.014G135400.1	AT4G03150.1
Potra2n14c27467.1	0.704726222	0.193011526	1.22003E-10	0.831597503	Potri.014G135500.3	AT3G06350.1
Potra2n14c27468.1	0.696966535	0.204250094	2.98738E-09	0.820880672	Potri.T106900.1	AT4G03140.1
Potra2n14c27469.3	-0.430592053	0.180339329	0.005286099	0.82104194	Potri.004G011300.1	
Potra2n14c27470.1	-0.046099098	0.12555958	0.867358782	0.917516356	Potri.004G011300.1	
Potra2n14c27471.1	2.50218194	2.260749926	0.086503903	NA	Potri.T106800.1	AT4G03130.1
Potra2n14c27472.1	-0.133327469	0.096978153	0.417225216	0.940687904	Potri.T106800.1	AT4G03130.1
Potra2n14c27473.1	0.333753718	0.210261353	0.054656813	0.7696553	Potri.T106600.1	AT4G03110.1
Potra2n14c27474.1	-0.905356903	-1.207413157	0.000124637	0.00782857	Potri.T106400.1	AT3G62990.1
Potra2n14c27475.1	-0.922470321	-0.241321742	0.002825187	0.896118765	Potri.T106300.2	AT4G03100.1
Potra2n14c27476.1	-1.917039903	-0.401426335	2.77245E-06	0.869186333	Potri.012G042800.1	AT1G05260.1
Potra2n14c27477.1	0.09599784	-0.026794517	0.464954489	0.983446961	Potri.T106100.1	AT3G63000.1
Potra2n14c27478.1	0.261202754	-0.350850701	0.264519914	0.824448251	Potri.014G135600.1	AT2G47990.1
Potra2n14c27479.1	-0.286531043	-0.043195574	0.011055728	0.959042326	Potri.014G135700.2	AT4G03090.1
Potra2n14c27480.1	0.134180599	0.177823371	0.401647263	0.684244573	Potri.014G135800.3	AT4G03080.1
Potra2n14c27481.1	3.568941069	2.067868982	2.37643E-22	0.027320927	Potri.014G135900.3	AT3G63010.1
Potra2n14c27482.1	-0.602185121	0.008654031	0.000117634	0.996032145	Potri.014G136000.1	AT4G03020.1
Potra2n14c27483.1	-1.201034152	-0.308699678	0.01152221	0.917977725	Potri.014G136100.1	AT1G03440.1
Potra2n14c27484.1	0.332312502	0.589885346	0.22448613	0.74792604	Potri.014G136200.1	AT1G03430.1

Potra2n14c27485.1	-0.748085339	0.072171965	0.027472666	0.981726976	Potri.014G136300.1	AT5G35960.1
Potra2n14c27486.1	0.696238187	0.194694971	9.08737E-05	0.831597503	Potri.014G136400.1	AT2G48010.1
Potra2n14c27487.1	1.942501097	-2.071795494	0.57980349	NA		
Potra2n14c27488.1	0.897106703	0.225274056	1.10798E-12	0.80008188	Potri.014G136400.1	AT2G48010.1
Potra2n14c27489.4	-0.232982549	-0.23722161	0.64307008	0.941260209	Potri.014G136600.1	AT2G48020.1
Potra2n14c27490.1	1.351752501	0.075338796	3.03373E-14	0.95172055	Potri.014G136800.1	AT1G23160.1
Potra2n14c27491.1	0.417483279	-0.408964432	0.523988186	0.846680547	Potri.014G136900.1	AT2G48030.1
Potra2n14c27492.3	0.193319443	0.006767679	0.179163476	0.996032145	Potri.014G137000.1	AT1G65410.1
Potra2n14c27493.1	-0.040356916	-0.08826489	0.81009489	0.900869139	Potri.014G137100.1	AT3G63030.1
Potra2n14c27494.4	0.176656788	0.228999061	0.282658403	0.752523551	Potri.014G137200.1	AT2G48060.1
Potra2n14c27495.1	-0.689467601	-0.235859084	2.62377E-06	0.765390763	Potri.014G137300.1	AT2G48070.1
Potra2n14c27496.1	-0.389927478	-0.20548256	0.038155384	0.775618506	Potri.014G137400.1	AT4G02990.1
Potra2n14c27497.2	0.167236506	0.120575071	0.393267139	0.922778027	Potri.014G137700.2	AT4G02940.1
Potra2n14c27498.1	1.857817522	0.534924055	0.581433038	NA	Potri.001G287500.1	
Potra2n14c27499.1	-0.165297907	-0.038557031	0.118279849	0.948577865	Potri.014G138000.1	AT1G63900.1
Potra2n14c27500.1	0.643731896	0.009045942	0.001724809	0.997811695	Potri.014G138100.1	AT4G02930.1
Potra2n14c27501.1	0.163327548	-0.011649706	0.241780135	0.989304496	Potri.014G138200.1	AT4G02920.1
Potra2n14c27502.1	0.983571693	0.284948078	1.27457E-10	0.683816919	Potri.014G138400.1	AT3G23590.1
Potra2n14c27503.1	0.046512746	0.161383037	0.807159346	0.850175528	Potri.014G138500.1	AT2G48120.1
Potra2n14c27504.1	-2.893310937	-1.458939475	NA	NA	Potri.002G215400.1	AT1G03310.1
Potra2n14c27505.1	-2.893310937	-1.458939475	NA	NA		
Potra2n14c27506.1	0.07714068	0.098317097	0.542232405	0.875861859	Potri.014G138700.1	AT2G48160.1
Potra2n14c27507.1	-0.423113456	-0.381929685	0.397222135	0.874648591	Potri.002G215400.1	AT1G03310.1
Potra2n14c27508.1	-0.062425173	-0.065551407	0.700675308	0.961820855	Potri.014G138800.1	AT2G48150.1
Potra2n14c27509.1	0.230502498	-0.032270992	0.051835577	0.971432539	Potri.014G139000.2	AT1G03290.2
Potra2n14c27510.1					Potri.014G139100.1	AT3G63095.1
Potra2n14c27511.1	-2.03072916	0.735564371	0.564426137	NA	Potri.014G139200.1	AT3G63095.1
Potra2n14c27512.1	NA	0.674963689	NA	NA	Potri.014G139300.1	AT3G63110.1
Potra2n14c27513.1	-1.036641468	-0.834786896	0.141198846	0.735605179	Potri.014G139400.1	AT3G58720.2
Potra2n14c27514.2	-0.422515394	-0.16065334	0.013264237	0.867960518	Potri.014G139500.1	AT2G30950.1
Potra2n14c27515.1	0.384273679	-0.068073474	0.00488579	0.96670549	Potri.014G139600.1	AT3G26680.2
Potra2n14c27516.1	-0.074657497	-0.058656333	0.649968243	0.9761362	Potri.014G139700.1	AT3G26670.1

Potra2n14c27517.1	-0.907929205	-0.288731373	0.000490058	0.849446235	Potri.014G139900.1	AT1G52140.1
Potra2n14c27518.1	-1.161334855	-1.385210147	0.600904374	NA		
Potra2n14c27519.1	-1.241316465	-0.567004085	3.11041E-12	0.657719957	Potri.014G140000.1	AT5G47920.1
Potra2n14c27520.2	-0.281807481	-0.064613229	0.021702505	0.940361195	Potri.014G140100.1	AT3G02065.2
Potra2n14c27521.1	-2.494785529	-0.552003708	0.000575349	0.842247476	Potri.014G140300.1	AT5G13870.1
Potra2n14c27522.1	-0.076401495	0.000267958	0.791366938	0.999937671	Potri.014G140400.1	AT3G02660.1
Potra2n14c27523.2	-1.18788985	-0.494261743	0.000330778	0.605100478	Potri.014G140500.2	AT3G26650.1
Potra2n14c27524.1	0.503474779	-0.0670216	6.56113E-05	0.964971544	Potri.014G140600.1	AT5G19090.1
Potra2n14c27525.1	0.860099293	0.163433314	4.43996E-13	0.715585895	Potri.014G140900.6	AT5G36880.2
Potra2n14c27526.1	0.963854728	0.093936454	9.4729E-15	0.915488947		
Potra2n14c27527.1	0.189696618	0.261080521	0.110302698	0.66774826	Potri.014G141100.1	AT1G12930.1
Potra2n14c27528.1	0.351094839	0.239104644	0.000131292	0.71411664	Potri.014G141200.1	AT3G54900.1
Potra2n14c27529.1	2.954110844	1.274182508	3.61767E-15	0.107143374	Potri.014G141400.1	AT5G45710.1
Potra2n14c27530.1	-1.409983009	2.352766567	NA	NA	Potri.014G141500.1	AT1G06460.1
Potra2n14c27531.2	-0.229314256	-0.040240502	0.047489086	0.973347974	Potri.014G141700.1	AT2G30910.3
Potra2n14c27532.1	-0.294908939	-0.030984592	0.068031414	0.983113084		
Potra2n14c27533.1	-0.007289926	0.058744492	0.954555699	0.923348116	Potri.014G141800.6	AT2G42810.2
Potra2n14c27534.1	1.059533788	0.18186975	6.80068E-16	0.815119099	Potri.014G141900.1	AT3G58750.1
Potra2n14c27535.1	0.118041654	0.19208818	0.618670808	0.643956625	Potri.014G142000.1	AT3G58790.1
Potra2n14c27536.1	0.147695063	0.119385108	0.554655234	0.910878765		
Potra2n14c27537.1	0.114683277	0.128776557	0.390078	0.87062349	Potri.014G142200.1	AT2G31040.1
Potra2n14c27538.1	0.506148393	0.640127447	0.22688172	0.777553704	Potri.002G214100.1	AT1G53310.2
Potra2n14c27539.1	-0.427583241	-0.055809494	0.006832168	0.976302215	Potri.014G142400.1	AT2G42610.2
Potra2n14c27540.2	0.330784574	0.165244544	0.050004453	0.910477984	Potri.014G142600.1	AT2G42620.1
Potra2n14c27541.4	-0.030443833	-0.011533944	0.810790884	0.987810003	Potri.014G142700.1	AT3G58640.1
Potra2n14c27542.1	-0.22380211	-0.266662392	0.312132659	0.755741462		
Potra2n14c27543.1	-0.138871219	0.041810531	0.455072804	0.974275337		
Potra2n14c27544.1	-0.916276099	-0.396606565	9.23244E-11	0.616163505	Potri.014G142800.1	AT2G30990.2
Potra2n14c27545.1	0.156938373	-0.007662217	0.173725455	0.994692557	Potri.014G142900.1	AT2G42700.1
Potra2n14c27546.3	0.213583816	0.067105195	0.269198328	0.976302215	Potri.014G143000.2	AT2G42710.1
Potra2n14c27547.3	-0.537124597	-0.220561136	0.120273645	0.930159505	Potri.014G143100.1	AT3G58690.1
Potra2n14c27548.1	0.82357019	1.394613675	NA	NA	Potri.014G143200.2	AT5G05340.1

Potra2n14c27549.2	0.211029987	-0.092466005	0.298110794	0.927423678	Potri.014G143300.1	AT2G30970.2
Potra2n14c27550.1	-1.074418103	-0.24232187	3.32955E-07	0.722616537	Potri.014G143400.1	AT2G42750.1
Potra2n14c27551.1	-0.945349331	-0.412565133	7.44858E-05	0.216891326	Potri.014G143500.1	AT1G30540.1
Potra2n14c27552.1	0.74423228	0.395784088	2.46723E-13	0.375012853	Potri.014G143600.1	AT5G13980.2
Potra2n14c27553.1	0.328928954	0.101958096	0.042117232	0.880553952	Potri.014G143800.1	AT5G13970.1
Potra2n14c27554.2	0.283485438	-0.097171323	0.57757509	0.977559521	Potri.014G143900.1	AT5G13960.1
Potra2n14c27555.1	NA	NA	NA	NA	Potri.014G144000.1	
Potra2n14c27556.1	-0.106263668	0.118672719	0.418928484	0.897789832	Potri.014G144100.1	AT5G13950.2
Potra2n14c27557.3	-0.345244387	-0.017668796	0.021258634	0.99114586	Potri.014G144300.4	AT3G19340.1
Potra2n14c27558.1	0.517950999	-0.120717894	0.017119572	0.934165971	Potri.014G144500.1	AT3G19340.1
Potra2n14c27559.1	0.577610654	-0.141375155	0.006590493	0.917751882	Potri.014G144600.1	AT3G02130.1
Potra2n14c27560.1	-0.742525973	-1.710027945	0.326454879	0.49456927	Potri.009G125100.1	AT2G21250.1
Potra2n14c27561.1	-0.426355764	-0.277401939	0.005551903	0.568587629	Potri.014G144800.1	AT3G26700.1
Potra2n14c27562.1	-0.051279256	-0.02146488	0.762294616	0.983113084	Potri.014G144900.1	AT3G02050.1
Potra2n14c27563.1	-0.937023193	-0.481792479	9.01961E-05	0.563998615	Potri.014G145000.1	AT3G02060.1
Potra2n14c27564.1	5.339915649	1.23228457	1.39718E-10	0.71424814	Potri.014G145100.2	AT5G13930.1
Potra2n14c27565.1	0.120472088	0.135806374	0.333336805	0.835819291	Potri.014G145200.1	AT5G46860.1
Potra2n14c27566.1	0.183540797	0.059643025	0.007591694	0.904798925	Potri.014G145300.4	AT3G26730.1
Potra2n14c27567.1	-0.190413279	-0.045669962	0.076192515	0.954419252	Potri.014G145400.1	AT1G08660.1
Potra2n14c27568.1	0.29060461	0.04010773	0.28635534	0.981772014	Potri.014G145600.1	AT2G31410.1
Potra2n14c27569.1	1.227945156	-0.007408616	1.02873E-05	0.998245864	Potri.014G145700.1	AT5G14060.1
Potra2n14c27570.1	-2.943260674	-1.171167745	1.34624E-10	0.382821403	Potri.014G145800.2	AT3G26760.1
Potra2n14c27571.1	0.248919525	-0.059991678	0.008371089	0.943438281	Potri.014G145900.1	AT3G26780.1
Potra2n14c27572.1	-0.902399384	-0.525356811	0.105806855	0.859892315	Potri.014G146100.1	AT3G23730.1
Potra2n14c27573.1	-0.018790633	0.092847008	0.926546404	0.934439727	Potri.014G146200.1	AT4G14200.1
Potra2n14c27574.1	3.025037209	NA	NA	NA	Potri.014G149500.2	AT4G14190.1
Potra2n14c27575.1	0.82357019	NA	NA	NA		
Potra2n14c27576.2	0.002975042	0.303959699	0.984443375	0.655979264	Potri.014G146300.1	AT1G05830.1
Potra2n14c27577.1	0.038502738	0.15212674	0.794952803	0.836066102	Potri.014G146400.1	AT1G05840.1
Potra2n14c27578.1	NA	1.394613675	NA	NA	Potri.014G146500.1	AT1G55200.1
Potra2n14c27579.1	0.239531651	0.168207245	0.031005553	0.806746126	Potri.014G146600.1	AT1G05850.1
Potra2n14c27580.1	3.743577741	NA	0.341195965	NA	Potri.014G146700.1	AT5G16740.1

Potra2n14c27581.1	2.067109072	0.134512884	0.001487769	0.985393296	Potri.014G146800.1	AT5G57090.1
Potra2n14c27582.1	-0.444034094	-0.097462881	0.00252681	0.903698951	Potri.014G146900.1	AT1G10830.1
Potra2n14c27583.1	-0.928274188	0.11191122	0.001096808	0.966228173	Potri.014G147000.1	AT5G16490.1
Potra2n14c27584.1	0.174667175	0.073338456	0.235789489	0.943826727	Potri.014G147100.1	AT1G10840.1
Potra2n14c27585.1	0.508783596	-0.004072612	0.003002164	0.99812851	Potri.012G086600.2	AT4G29390.1
Potra2n14c27586.1	1.840890986	0.290196925	4.15796E-10	0.900869139	Potri.014G147300.1	AT3G28040.1
Potra2n14c27587.4	0.204060217	0.470020531	0.121493497	0.694678604	Potri.014G147400.13	AT3G13340.2
Potra2n14c27588.1	-1.090238685	-0.373115082	6.33524E-07	0.728418847	Potri.014G147600.1	AT1G10800.1
Potra2n14c27589.1	0.891725198	0.415103766	1.86074E-08	0.312622509	Potri.014G147700.1	AT1G60710.1
Potra2n14c27590.1	-0.691379553	-0.877696107	0.455793869	0.843765296	Potri.014G147800.1	AT4G13990.1
Potra2n14c27591.1	-0.009960042	0.094143829	0.946593727	0.96245401	Potri.014G147900.1	AT5G26570.1
Potra2n14c27592.1					Potri.002G234900.1	AT1G05510.1
Potra2n14c27593.1	-2.745923194	-0.962032061	0.022330109	0.858174358	Potri.014G148200.1	AT3G25400.1
Potra2n14c27594.1	-0.123378743	-0.030971782	0.699368447	0.9896961	Potri.014G148300.1	AT4G29400.1
Potra2n14c27595.1	-1.344226216	-0.205049346	4.0348E-14	0.821709751	Potri.014G148500.1	AT3G23700.1
Potra2n14c27596.1	1.459962178	NA	NA	NA	Potri.014G148600.1	AT2G42820.1
Potra2n14c27597.1	-0.025662718	0.006975783	0.755766216	0.991846003	Potri.014G148800.1	AT2G32000.1
Potra2n14c27598.1	-0.22068971	0.001119567	0.270955371	0.999458638	Potri.014G148900.2	AT3G23690.1
Potra2n14c27599.2	-0.41344812	-0.207297347	0.000594962	0.76443278	Potri.014G149000.1	AT3G23670.1
Potra2n14c27600.1	-0.166538875	-0.018071102	0.132909225	0.985393296	Potri.002G235700.2	AT5G42390.1
Potra2n14c27601.1	NA	NA	NA	NA	Potri.014G149300.1	AT2G31980.1
Potra2n14c27602.1	-0.009943978	0.063815162	0.96940627	0.957668459	Potri.014G149400.1	AT5G48570.1
Potra2n14c27603.1	-0.172190397	0.145803081	0.317133386	0.924251575	Potri.014G149500.2	AT4G14190.1
Potra2n14c27604.1	0.414760075	0.035886059	0.0001548	0.96756018	Potri.014G149600.2	AT2G31660.1
Potra2n14c27605.1	-2.396052862	-1.12977434	NA	NA	Potri.014G149700.1	AT3G59010.1
Potra2n14c27606.1	-0.314143677	0.353297773	0.072078118	0.874648591	Potri.014G149800.1	AT2G43060.1
Potra2n14c27607.1	0.171695564	0.267773722	0.34043296	0.865611082	Potri.014G149900.1	AT5G48490.1
Potra2n14c27608.1	-0.164097764	0.508185851	0.463078337	0.741833788	Potri.014G150000.1	AT2G43070.1
Potra2n14c27609.1	1.172248904	0.203864008	3.48269E-08	0.903741471	Potri.014G150100.1	AT2G43110.1
Potra2n14c27610.2	-0.715338521	0.399363736	0.005531335	0.817452127	Potri.014G150200.1	AT2G43120.1
Potra2n14c27611.2	0.056692341	0.124490653	0.760513285	0.922011871	Potri.014G150300.1	AT2G31680.1
Potra2n14c27612.1	0.096015001	0.512299249	0.565706099	0.518816993	Potri.014G150600.1	AT1G05805.1

Potra2n14c27613.1	1.545832463	NA	NA	NA	Potri.014G150700.1	AT2G31690.1
Potra2n14c27614.1	0.172260896	0.151179456	0.351359944	0.880316339	Potri.014G150800.1	AT1G05790.1
Potra2n14c27615.1	1.664677056	NA	NA	NA	Potri.014G150900.1	AT4G08910.1
Potra2n14c27616.1	0.193628978	-0.098835553	0.122317531	0.905231727	Potri.014G151000.1	AT1G05785.1
Potra2n14c27617.1	1.931605898	NA	NA	NA	Potri.009G001800.1	AT2G01050.1
Potra2n14c27618.1	-0.070276824	-0.149199496	0.731709001	0.920893228	Potri.014G151200.1	AT3G59280.1
Potra2n14c27619.1	-0.163270966	-0.065570882	0.260567161	0.954098985	Potri.014G151300.2	AT2G43160.2
Potra2n14c27620.1	0.024485064	0.146964992	0.909675833	0.9045874	Potri.002G230800.1	AT2G47980.1
Potra2n14c27621.1	2.402541569	-0.048194598	0.466218034	NA	Potri.014G151700.4	AT3G59300.1
Potra2n14c27622.1	-0.3719019	-0.507214679	0.28736795	0.590087814	Potri.014G151800.1	AT3G59310.1
Potra2n14c27623.1	1.64774763	NA	NA	NA		
Potra2n14c27624.1	2.195892536	1.439893019	0.001244692	0.70570456	Potri.014G151900.1	AT3G21710.2
Potra2n14c27625.2	0.033600089	0.06197156	NA	NA	Potri.014G152000.2	AT1G05230.1
Potra2n14c27626.1	NA	NA	NA	NA	Potri.014G152100.1	AT3G21700.3
Potra2n14c27627.2	-0.068053434	0.11683569	0.790321042	0.886059841	Potri.014G152200.1	AT1G05200.2
Potra2n14c27628.2	-0.385412687	-0.493114352	0.843345852	0.94494655	Potri.014G152600.1	AT1G02205.2
Potra2n14c27629.1	-0.226435709	0.08623352	0.370074899	0.962661188	Potri.014G153000.1	AT1G05190.1
Potra2n14c27630.1	NA	1.571529889	NA	NA	Potri.014G153100.1	AT3G21690.1
Potra2n14c27631.1	-0.901624049	-0.499398687	0.476985029	0.935474933	Potri.014G153200.1	AT3G21680.1
Potra2n14c27632.1	-0.61910233	-0.767708831	NA	NA		
Potra2n14c27633.1	-0.190681182	-0.007122381	0.266709036	0.996032145	Potri.014G153400.3	AT2G05710.1
Potra2n14c27634.1	NA	NA	NA	NA	Potri.014G153500.1	AT1G05180.1
Potra2n14c27635.1	NA	NA	NA	NA	Potri.014G153600.1	AT5G41060.1
Potra2n14c27636.1	-0.309222048	-0.071073311	0.00673696	0.95671968	Potri.014G153700.1	AT1G05170.2
Potra2n14c27637.1	-0.324958388	-0.151358762	0.005185007	0.903496619	Potri.014G153900.1	AT1G19020.1
Potra2n14c27638.1	-0.511559403	-0.331761961	0.008716573	0.603936691	Potri.014G154000.1	AT2G32480.1
Potra2n14c27639.1	2.242197065	0.725365883	1.32192E-18	0.61887385	Potri.014G154200.2	AT4G21990.1
Potra2n14c27640.1	0.870577288	0.499606551	1.63816E-06	0.365622999	Potri.014G154400.1	AT5G18290.1
Potra2n14c27641.1	3.965401043	0.750928034	1.64219E-06	0.906545272	Potri.014G154500.1	AT4G22010.1
Potra2n14c27642.2	0.577021022	0.228091775	7.41421E-12	0.242879358	Potri.014G154600.3	AT1G05120.1
Potra2n14c27643.1	0.588824629	0.102343014	4.1541E-06	0.903698951	Potri.014G154700.1	AT4G26910.1
Potra2n14c27644.1	0.299048836	0.031043252	0.021891416	0.975578749	Potri.014G154800.1	AT5G42570.1

Potra2n14c27645.2	-0.40396623	0.241949046	0.125476553	0.880870972	Potri.014G154900.1	AT2G32500.1
Potra2n14c27646.1	-0.357642888	-0.058197657	0.286741647	0.977559521	Potri.014G155000.1	AT1G05100.1
Potra2n14c27647.1	5.895752185	1.424306606	1.48148E-09	0.809209908	Potri.014G155100.1	AT1G62300.1
Potra2n14c27648.1	-0.212206775	0.085992599	0.047496617	0.921099436	Potri.014G155200.1	AT2G32520.1
Potra2n14c27649.1	2.199113796	2.32414027	NA	NA	Potri.014G155300.1	AT2G32540.1
Potra2n14c27650.1	0.39441113	-1.347133835	0.839759039	NA	Potri.014G155500.1	AT5G12980.1
Potra2n14c27651.2	0.124748975	0.156080831	0.257340755	0.804641567	Potri.014G155600.1	AT2G32580.1
Potra2n14c27652.1	0.163147355	0.145173216	0.689824732	0.953658357		
Potra2n14c27653.1	0.044837878	-0.36603735	0.845322375	0.787393135	Potri.014G155800.1	AT4G15440.1
Potra2n14c27654.1	0.841769035	-0.283875298	0.026108132	0.919424623	Potri.014G155900.1	AT2G32590.1
Potra2n14c27655.1	0.313317881	0.161983497	0.076455226	0.900869139	Potri.014G156000.2	AT3G21610.1
Potra2n14c27656.1	0.709388924	0.344425933	0.065541957	0.857374454	Potri.014G156100.1	AT3G21620.1
Potra2n14c27657.1	-0.35549666	-0.19946686	0.007809748	0.852534479	Potri.014G156300.1	AT4G15420.1
Potra2n14c27658.1	0.674513259	-0.030525156	0.011622471	0.987548286	Potri.014G156400.1	AT3G21630.1
Potra2n14c27659.1	-0.327699629	-0.024493187	0.047108138	0.984292161	Potri.014G156700.1	AT1G05060.1
Potra2n14c27660.1	-0.40245419	-0.019593078	0.005782697	0.987203719	Potri.014G156800.1	AT2G32640.1
Potra2n14c27661.1	-0.005063264	-0.221911053	0.989443483	0.895560236	Potri.014G156900.1	AT2G32180.1
Potra2n14c27662.1	-0.048531735	0.045428167	0.75759142	0.953831244	Potri.014G157000.1	AT3G21650.1
Potra2n14c27663.1	-0.11402839	-0.017633205	0.33600792	0.985393296	Potri.014G157100.1	AT4G22140.2
Potra2n14c27664.1	-0.456148688	-0.278480905	0.013930842	0.743252386	Potri.014G157200.1	AT4G15410.1
Potra2n14c27665.1	0.199742894	-0.086140876	0.643437407	0.974367223	Potri.014G157300.1	AT3G08000.1
Potra2n14c27666.1	0.934643627	0.13115635	7.64859E-05	0.937846405	Potri.014G157400.1	AT5G55140.1
Potra2n14c27667.1	1.194598093	0.286756375	1.06375E-06	0.835819291	Potri.014G157500.1	AT3G21670.1
Potra2n14c27668.1	2.071626522	0.723699544	0.001735657	0.875559093		
Potra2n14c27669.1	0.611712157	-0.058699974	0.003930472	0.982827151	Potri.014G157600.1	AT2G32990.1
Potra2n14c27670.1	-0.015008535	0.010202163	0.936680329	0.993579699	Potri.014G157700.1	AT2G05755.1
Potra2n14c27671.1	0.775074197	0.253613848	0.005228214	0.89521294	Potri.014G157800.1	AT2G05760.1
Potra2n14c27672.1	-0.468834661	-0.275572609	0.002155557	0.759797728	Potri.014G158000.1	AT2G32980.1
Potra2n14c27673.1	-0.098774768	-0.005368713	0.290328206	0.995203254	Potri.014G158200.7	AT2G32970.2
Potra2n14c27674.1	0.314000539	0.028902737	0.044744752	0.97950444	Potri.014G158300.1	AT5G55160.1
Potra2n14c27675.1	-0.638694446	-0.61723617	0.009470218	0.586594557	Potri.014G158400.2	AT2G05790.1
Potra2n14c27676.1	-1.29106958	-0.524881133	0.005263448	0.725595998		

Potra2n14c27677.1	NA	NA	NA	NA	Potri.014G158600.2	AT1G05020.1
Potra2n14c27678.3	1.462516515	0.487813046	1.18463E-17	0.693384896	Potri.014G158700.1	AT2G05810.1
Potra2n14c27679.1	-2.720325861	-0.456138444	5.77242E-07	0.854980698	Potri.002G224300.2	AT2G31340.1
Potra2n14c27680.1	-2.129819812	-0.748752661	1.43392E-06	0.4583793	Potri.014G158900.2	AT4G22250.1
Potra2n14c27681.1	NA	NA	NA	NA	Potri.014G176800.1	
Potra2n14c27682.1	4.565003338	2.731560625	7.39049E-53	1.94301E-05	Potri.014G159000.1	AT1G05010.1
Potra2n14c27683.2	-0.745400055	-0.007418696	0.045735853	0.996999319	Potri.014G159100.1	AT1G05000.1
Potra2n14c27684.1	-0.263984317	0.126007765	0.020740249	0.904709467	Potri.014G159300.2	AT2G32950.1
Potra2n14c27685.1	-0.26615801	-0.050858613	0.318527441	0.977323635	Potri.014G159400.1	AT2G32940.1
Potra2n14c27686.1	NA	NA	NA	NA		
Potra2n14c27687.1	0.447261321	0.20552178	7.06948E-06	0.82747159	Potri.014G159500.2	AT1G04990.2
Potra2n14c27688.1	0.471886039	0.139569618	0.000382664	0.898208704	Potri.014G159600.1	AT1G04985.1
Potra2n14c27689.1	-1.056982839	-0.633833086	0.652834099	NA	Potri.014G159800.1	AT5G14180.1
Potra2n14c27690.1	0.494852263	0.049425856	0.000102591	0.95981322	Potri.014G160000.1	AT1G04980.1
Potra2n14c27691.1	3.550120751	3.01657805	0.381577267	NA	Potri.014G160100.1	AT2G04570.1
Potra2n14c27692.1	-1.903049284	-2.05258594	NA	NA	Potri.014G160200.1	AT2G42990.1
Potra2n14c27693.1	0.197653924	-0.12085911	0.58954762	0.950878725	Potri.014G160300.1	AT2G04560.1
Potra2n14c27694.1	-0.034791629	-0.003959199	0.813948328	0.998245864	Potri.014G160400.1	AT5G35690.1
Potra2n14c27695.1	-0.0136852	-0.051587235	0.925068918	0.971828097		
Potra2n14c27696.1	0.081067684	0.066541045	0.484481912	0.954559968	Potri.014G160500.1	AT2G04550.1
Potra2n14c27697.1	0.115128669	-0.095922486	0.602358553	0.935099709	Potri.014G160600.1	AT2G04530.1
Potra2n14c27698.1	-0.633224721	-0.29726902	0.002992795	0.815767573	Potri.014G160700.1	AT1G04970.1
Potra2n14c27699.1	-0.386057197	-0.1344141	0.005133895	0.935995049	Potri.014G160800.2	AT1G04960.1
Potra2n14c27700.3	0.243821237	0.149852421	0.036623493	0.858632929	Potri.014G161000.2	AT5G35670.1
Potra2n14c27701.1	0.251346028	0.142963935	0.029342577	0.874496903	Potri.014G160900.1	AT2G04520.1
Potra2n14c27702.1	-0.140139453	0.140846388	0.266810648	0.77486864	Potri.014G161100.1	AT1G04950.1
Potra2n14c27703.1	5.299544995	-1.187251647	4.43527E-05	NA	Potri.014G161200.1	AT2G44310.1
Potra2n14c27704.1	1.871792595	0.645653659	2.39973E-18	0.343929	Potri.014G161300.1	AT5G14780.1
Potra2n14c27705.1	NA	NA	NA	NA	Potri.017G098750.1	AT3G43505.1
Potra2n14c27706.1	0.135838171	0.111710386	0.736461401	0.957533867	Potri.001G222600.14	AT2G26980.4
Potra2n14c27707.2	0.18427033	0.040920552	0.412359866	0.975578749	Potri.014G161600.2	AT3G07810.1
Potra2n14c27708.1	-0.818530197	-0.454870245	0.000675014	0.706078246	Potri.014G161700.1	AT2G04850.1

Potra2n14c27709.1	-0.211383866	0.005473137	0.022228394	0.996999319	Potri.014G161800.3	AT2G04845.1
Potra2n14c27710.1	-0.201608913	0.054560967	0.616080579	0.984292161	Potri.014G161900.1	AT4G17280.1
Potra2n14c27711.1	0.868442339	-0.000571825	0.000240918	0.999937671	Potri.014G162000.1	AT5G47530.1
Potra2n14c27712.1	0.397273719	-0.24911887	0.415930857	0.953768643	Potri.014G162100.2	AT5G47540.1
Potra2n14c27713.1	-0.097693779	-0.063789829	0.560821269	0.948834049	Potri.014G162200.1	AT2G04842.1
Potra2n14c27714.1	-0.749823207	-1.036082888	NA	NA	Potri.014G162300.2	AT3G07850.1
Potra2n14c27715.1	NA	NA	NA	NA		
Potra2n14c27716.1	-0.230101668	0.179557901	0.451525106	0.917516356	Potri.014G162400.1	AT5G35732.1
Potra2n14c27717.1	1.080730322	0.387579585	1.53976E-11	0.362892394	Potri.014G162600.1	AT3G07870.1
Potra2n14c27718.1	0.755270743	0.318649524	8.42245E-06	0.540010302	Potri.014G162700.1	AT2G04790.2
Potra2n14c27719.1	-1.486259858	-0.089035513	2.06008E-17	0.963557834	Potri.014G162800.1	
Potra2n14c27720.1	-0.387519089	-0.650595511	0.165420266	0.534852254		
Potra2n14c27721.1	0.890277661	1.017843843	0.283738963	0.765464081	Potri.014G163000.1	AT3G07880.1
Potra2n14c27722.1	0.119155853	0.049881856	0.353700362	0.953831244	Potri.014G163100.1	AT5G35700.1
Potra2n14c27723.3	0.098662196	0.045924906	0.479981113	0.959009636	Potri.014G163200.2	AT2G04740.1
Potra2n14c27724.2	-0.311789133	-0.002127252	0.063335294	0.999315506	Potri.014G163400.1	AT3G07890.1
Potra2n14c27725.1	NA	NA	NA	NA	Potri.014G163600.1	AT5G62380.1
Potra2n14c27726.1						
Potra2n14c27727.1	-0.21765713	-0.031898817	0.183288355	0.982221696		
Potra2n14c27728.1	4.882536045	4.09300154	0.033564189	NA	Potri.014G164000.1	AT5G48130.1
Potra2n14c27729.1	-1.464651138	-2.823786847	0.041121443	0.065625109	Potri.014G164100.1	AT5G55560.1
Potra2n14c27730.1	-0.03649987	-0.154936122	0.838420741	0.882699919	Potri.014G164200.1	AT3G07790.1
Potra2n14c27731.1	-0.139145198	-0.002623232	0.24716809	0.998245864	Potri.014G164300.1	AT2G04880.2
Potra2n14c27732.2	-0.986373483	-0.379961116	1.20523E-10	0.625226738	Potri.014G164400.1	AT5G48150.2
Potra2n14c27733.1	-0.081026088	0.053843556	0.618660032	0.951394085	Potri.014G164500.1	AT2G04900.1
Potra2n14c27734.3	-0.307260169	-0.086321689	0.506822737	0.956865545	Potri.014G164700.2	AT5G35750.1
Potra2n14c27735.1	-0.372582131	0.301936548	0.477685424	0.912777843		
Potra2n14c27736.1	-0.004568341	-0.008556526	0.970620128	0.993332869	Potri.014G164800.6	AT5G48160.2
Potra2n14c27737.2	0.654405094	0.015346553	0.000161982	0.995998037	Potri.014G164900.1	AT3G07770.1
Potra2n14c27738.1	-1.456789032	-0.042787025	1.66731E-05	0.98131997	Potri.014G165000.1	AT4G16447.1
Potra2n14c27739.1	0.008526961	-0.674402903	0.984043808	0.181966669	Potri.014G165100.1	AT2G05100.1
Potra2n14c27740.1	-0.534185455	-0.192762547	1.51342E-06	0.762567828	Potri.014G165300.1	AT3G07760.2

Potra2n14c27741.1	-0.257966966	0.058323059	0.03712394	0.943819961	Potri.014G165400.1	AT2G05170.1
Potra2n14c27742.1	-0.768101429	0.121144903	0.064004694	0.961820855	Potri.014G165500.1	AT2G05160.1
Potra2n14c27743.1	-0.147697831	-0.044240132	0.101661913	0.955771272	Potri.014G165600.1	AT2G05250.1
Potra2n14c27744.1	0.33510337	0.261070782	0.587371621	0.953831244	Potri.014G165700.1	AT5G48170.1
Potra2n14c27745.1	-2.513471462	-2.258392155	0.044883818	0.289193458	Potri.001G363000.1	AT3G26230.1
Potra2n14c27746.3	-0.173400301	0.061954205	0.52713045	0.979719961	Potri.014G166001.1	AT4G26680.2
Potra2n14c27747.1	-0.467243851	0.178620253	0.645711428	0.972159498	Potri.014G166100.1	AT2G45560.1
Potra2n14c27748.1	0.306319239	0.085889705	0.019415606	0.936549944	Potri.014G166200.2	AT3G07750.2
Potra2n14c27749.1	0.124027334	0.041416897	0.489030349	0.965249957	Potri.014G166300.1	AT3G07740.1
Potra2n14c27750.1	-0.135501038	-0.067325159	0.935969507	NA	Potri.014G166400.2	AT5G35770.1
Potra2n14c27751.1					Potri.014G166600.1	AT5G41040.1
Potra2n14c27752.1	-0.513014141	-0.292711757	0.006532448	0.688296575	Potri.014G166600.1	AT5G41040.1
Potra2n14c27753.1	-0.441140784	-0.275932067	0.03623269	0.718991569	Potri.014G166800.10	AT5G35790.1
Potra2n14c27754.1						
Potra2n14c27755.1	3.12074376	0.923975712	1.09096E-16	0.802942736	Potri.014G166900.2	AT2G04515.1
Potra2n14c27756.1	3.165887816	1.004889481	3.6918E-24	0.70570456	Potri.014G166900.2	AT2G04515.1
Potra2n14c27757.1	-0.20392539	0.003042345	0.140289851	0.99905083	Potri.014G167000.1	AT5G28530.1
Potra2n14c27758.1	5.920087914	1.037154465	4.26434E-06	NA	Potri.014G167100.1	AT2G28500.1
Potra2n14c27759.1	1.821349543	-0.074712211	0.682531888	NA		
Potra2n14c27760.1	0.541492138	-0.031914477	0.000672399	0.983763506	Potri.014G167200.1	AT2G05320.1
Potra2n14c27761.1	-0.419677589	-0.235911348	0.003239857	0.305728522	Potri.014G167300.1	AT2G05310.1
Potra2n14c27762.1	-0.490620041	-0.213422367	0.000234797	0.670058837	Potri.014G167400.1	AT4G16360.3
Potra2n14c27763.1	-0.381951224	0.192453924	0.272611419	0.907032966	Potri.014G167700.1	AT2G04480.1
Potra2n14c27764.1	0.094119564	0.031257149	0.701189843	0.985393296	Potri.014G167800.1	AT4G14540.1
Potra2n14c27765.1	0.203188763	0.837905211	NA	NA		
Potra2n14c27766.1	-0.338255814	-0.474564685	0.5186707	0.865591169	Potri.014G168100.1	AT4G12730.1
Potra2n14c27767.1	-0.828057725	-0.157655728	NA	NA	Potri.014G168166.1	AT5G66430.1
Potra2n14c27768.1	-0.314577809	-0.026015646	0.008582422	0.976302215	Potri.014G168300.1	AT3G07720.1
Potra2n14c27769.3	1.379617691	0.531146977	6.40145E-05	0.420956018	Potri.014G168400.1	AT5G47650.1
Potra2n14c27770.1	0.678493501	0.172496414	7.26296E-05	0.869175327	Potri.014G168600.1	AT3G07700.1
Potra2n14c27771.2	1.170609262	0.244759177	1.14906E-15	0.790362046	Potri.014G168700.2	AT5G48230.2
Potra2n14c27772.1	0.033008869	0.125455336	0.856999834	0.87062349	Potri.014G168900.2	AT5G55850.3

Potra2n14c27773.1	0.273123515	0.033234246	0.005682636	0.972159498	Potri.014G169100.2	AT5G48220.1
Potra2n14c27774.1	-0.4603766	0.023619138	0.00986413	0.989304496	Potri.014G169200.2	AT2G04360.1
Potra2n14c27775.1	-0.442679321	0.054363198	0.032407913	0.974275337	Potri.014G169300.2	AT3G07670.1
Potra2n14c27776.1	-0.206157224	0.066670825	0.09731427	0.941855873	Potri.014G169500.1	AT2G04340.1
Potra2n14c27777.1	-0.246395109	0.039677452	0.103548758	0.973006135	Potri.014G169400.7	AT2G04350.1
Potra2n14c27778.1	0.190080255	0.138334747	0.108123371	0.712891471	Potri.014G169900.1	AT3G07660.1
Potra2n14c27779.1	-0.170280989	-0.058401688	0.250416635	0.963557834	Potri.014G170100.1	AT4G12710.1
Potra2n14c27780.1	-0.099713252	-0.097452845	0.686303212	0.95671968	Potri.014G170200.1	AT2G04280.1
Potra2n14c27781.2	-0.439543271	-0.088620879	0.001775533	0.921115332	Potri.014G170300.1	AT2G04270.5
Potra2n14c27782.2	-0.386880105	-0.082270894	0.126562291	0.946464685		
Potra2n14c27783.1					Potri.014G176800.1	
Potra2n14c27784.4	0.358778807	0.039346284	0.082311586	0.983113084	Potri.014G170500.2	AT5G48240.1
Potra2n14c27785.1	-0.520498774	-0.163770194	4.67643E-05	0.802631112	Potri.014G170600.1	AT3G07650.2
Potra2n14c27786.1	-0.178290596	-0.405173691	0.542997477	0.602611218	Potri.014G170700.1	AT4G26550.1
Potra2n14c27787.1	-0.399391499	-0.117081963	0.016327718	0.95671968	Potri.014G170700.1	AT4G26550.1
Potra2n14c27788.1	-0.353134682	-0.137114097	0.014036772	0.913007989	Potri.014G170800.1	AT4G12680.1
Potra2n14c27789.1	-0.346146263	-0.18555405	0.017584352	0.851911116	Potri.014G170700.1	AT4G26550.1
Potra2n14c27790.1					Potri.014G170900.2	AT3G07620.1
Potra2n14c27791.1	1.459962178	NA	NA	NA	Potri.014G171100.1	AT5G03795.1
Potra2n14c27792.1	1.757091712	1.606865533	2.68005E-05	0.531258209	Potri.014G171300.2	AT3G07600.1
Potra2n14c27793.1	1.758182413	0.128529723	3.34399E-05	0.98177802	Potri.014G171600.1	AT2G04160.1
Potra2n14c27794.1	1.608209984	0.268096232	0.000331072	0.95671968	Potri.014G171700.2	AT5G14400.1
Potra2n14c27795.1	0.194472358	0.155494071	0.242815621	0.867960518	Potri.014G171800.1	AT5G48300.1
Potra2n14c27796.1	0.117358819	-0.004561922	0.367037356	0.996032145	Potri.014G171900.1	AT4G12640.1
Potra2n14c27797.1	0.648961678	-0.30870549	0.057279642	0.846680547	Potri.014G172000.4	AT5G48310.2
Potra2n14c27798.1	-0.179415378	-0.099939018	0.269947869	0.939607998	Potri.014G172050.1	AT2G06020.1
Potra2n14c27799.1	-0.182516793	-0.100500594	0.268403088	0.939653927	Potri.014G172200.1	AT4G12650.1
Potra2n14c27800.1	0.24655676	0.181355421	0.116642454	0.746877722	Potri.014G172300.1	AT4G11120.1
Potra2n14c27801.1	-1.047326426	-0.357700701	3.19681E-06	0.744391933	Potri.014G172400.1	AT1G61520.1
Potra2n14c27802.1	0.224287536	-0.073851249	0.15553341	0.969421568	Potri.014G198300.1	AT5G48330.2
Potra2n14c27803.1						
Potra2n14c27804.1	0.044242593	0.08810378	0.683141947	0.943411075	Potri.014G172600.2	AT3G07580.1

Potra2n14c27805.1	-0.142266138	-0.146128508	0.678201011	0.941260209	Potri.014G172800.1	AT2G04039.1
Potra2n14c27806.1	0.399471953	0.100914829	0.015828071	0.925115611	Potri.014G173000.2	AT4G12740.1
Potra2n14c27807.1	-0.072152318	-0.036813767	0.455679781	0.963225999	Potri.014G173100.1	AT3G25530.1
Potra2n14c27808.1	-0.13362051	-0.064952994	0.122521725	0.923773723	Potri.014G197800.1	AT4G12750.1
Potra2n14c27809.1	-0.191602163	0.03202492	0.296952246	0.980165413	Potri.014G173400.1	AT4G12760.1
Potra2n14c27810.1	-0.016147638	0.024256515	0.917433775	0.980475138	Potri.014G197600.1	AT4G12780.1
Potra2n14c27811.1	-0.094023328	-0.125868178	0.635523827	0.898993569	Potri.014G173600.1	AT3G07570.1
Potra2n14c27812.3	0.753089229	0.205688235	0.002833788	0.881090429	Potri.014G173700.2	AT3G07568.1
Potra2n14c27813.1	0.971531119	0.494119071	5.19197E-08	0.295461515	Potri.014G173800.1	AT3G07565.1
Potra2n14c27814.2	0.42664864	0.139414343	1.0306E-06	0.775343511	Potri.014G173900.1	AT3G07560.1
Potra2n14c27815.1	0.645353623	-0.023943651	0.000227815	0.99114586	Potri.014G174000.1	AT5G39740.2
Potra2n14c27816.1	0.904842129	0.472723375	0.00416348	0.674175338	Potri.014G174101.1	AT4G08330.1
Potra2n14c27817.1	0.451573676	0.072515108	0.002141781	0.936890993	Potri.014G174200.1	AT4G12790.5
Potra2n14c27818.1	1.804003651	-2.095573221	0.300107033	NA		
Potra2n14c27819.1	-0.243789315	-0.011491217	0.016252601	0.994236852	Potri.014G174400.1	AT2G39780.1
Potra2n14c27820.1	-0.12928686	-0.130710231	0.627880972	0.903477395	Potri.014G174300.1	AT5G48340.1
Potra2n14c27821.1	-0.521065308	-0.017401939	0.057132793	0.992934677		
Potra2n14c27822.1						
Potra2n14c27823.1						
Potra2n14c27824.1	0.767699179	-0.081228047	0.805584451	NA	Potri.014G174600.1	
Potra2n14c27825.1	0.643661746	-0.028581042	1.07573E-05	0.9896961	Potri.014G174700.1	AT3G25500.1
Potra2n14c27826.1	0.869391916	0.591210018	0.052877614	0.541196221	Potri.014G174800.1	AT5G48370.1
Potra2n14c27827.1	1.459962178	NA	NA	NA	Potri.010G011600.1	AT2G35040.1
Potra2n14c27828.1	1.464747721	0.786979054	1.3516E-09	0.323066615	Potri.T074200.2	AT5G17680.1
Potra2n14c27829.1	1.545832463	NA	NA	NA		
Potra2n14c27830.1	-0.087901756	0.176466348	0.539560175	0.896593025	Potri.014G175200.4	AT5G48385.1
Potra2n14c27831.1	0.519258545	0.230841097	0.403728148	0.93263929	Potri.014G175350.1	AT5G48390.1
Potra2n14c27832.1	0.354544311	0.229926103	0.036522343	0.733263164	Potri.014G175500.2	AT3G25470.1
Potra2n14c27833.1	0.395165683	0.199940909	0.031938172	0.821709751	Potri.014G175350.1	AT5G48390.1
Potra2n14c27834.1	-0.479584168	-0.121595991	0.023495781	0.946464685	Potri.014G175600.1	AT4G12800.1
Potra2n14c27835.1	-0.466070006	-0.406234188	0.469432978	0.895474078	Potri.014G175700.2	AT4G12830.1
Potra2n14c27836.1	-1.328951947	-1.478536539	NA	NA	Potri.014G175800.2	AT4G12840.2

Potra2n14c27837.1	1.147075632	-0.079512623	NA	NA	Potri.014G175900.1	AT3G59500.1
Potra2n14c27838.1	0.79283351	0.08690965	4.76986E-07	0.948914372	Potri.014G176000.1	AT3G07510.1
Potra2n14c27839.1	1.664677056	NA	NA	NA	Potri.001G076200.1	AT3G47630.2
Potra2n14c27840.1	0.356505499	-0.998383439	0.945572916	NA	Potri.014G176300.1	AT3G07510.2
Potra2n14c27841.1	-0.40341562	-0.061218175	0.02364957	0.960981446	Potri.014G176500.1	AT3G07500.1
Potra2n14c27842.1	-0.918368551	-0.246790751	0.003759309	0.896306549	Potri.014G176400.1	AT3G07510.1
Potra2n14c27843.1						
Potra2n14c27844.1	0.385802509	0.471928637	0.036743811	0.614346379	Potri.014G176600.1	AT4G12850.1
Potra2n14c27845.1					Potri.014G176800.1	
Potra2n14c27846.1	0.039568622	0.126946688	0.891757192	0.906107926	Potri.014G176900.1	AT3G25440.1
Potra2n14c27847.1	0.088664812	-0.083984789	0.663648221	0.915414833	Potri.014G177100.1	AT3G07480.1
Potra2n14c27848.2	0.434137554	0.071553757	0.001180689	0.949048812	Potri.014G018300.2	AT5G48440.1
Potra2n14c27849.1	-1.168333886	-0.367294366	6.4202E-12	0.438335662	Potri.014G177200.1	AT3G22400.1
Potra2n14c27850.1	-0.016022924	-0.163231875	0.951258553	0.903140487	Potri.014G177400.1	AT3G25430.1
Potra2n14c27851.1	-0.328080616	-0.040076145	0.033597213	0.981772014	Potri.014G177500.1	AT4G12910.1
Potra2n14c27852.1	-1.032320695	-0.210031442	0.000348201	0.930598202	Potri.014G177700.1	AT5G48450.1
Potra2n14c27853.1	-0.454843934	-0.15373444	0.00946207	0.902752176	Potri.014G177800.1	AT3G07470.1
Potra2n14c27854.2	-0.876945963	-0.343435624	0.000456652	0.547799891	Potri.014G178000.2	AT3G25410.1
Potra2n14c27855.1						
Potra2n14c27856.2	-0.127388916	-0.377837255	0.728955553	0.306363984	Potri.014G178100.1	AT4G13710.1
Potra2n14c27857.2	NA	1.571529889	NA	NA	Potri.009G002500.1	
Potra2n14c27858.1	-0.08466837	-0.03283604	0.354419087	0.965445938	Potri.014G178300.1	AT3G07020.2
Potra2n14c27859.1	0.072116791	0.163518752	0.569084928	0.727843918	Potri.014G178700.2	AT5G27740.1
Potra2n14c27860.1	0.088666075	0.109557775	0.902149403	0.976139323	Potri.014G178800.1	AT1G29000.1
Potra2n14c27861.1	-1.328951947	-1.478536539	NA	NA	Potri.014G178900.1	
Potra2n14c27862.1	0.398913526	0.36463841	0.000398199	0.48299238	Potri.014G179000.1	AT5G65940.1
Potra2n14c27863.2	-0.185534622	0.267955955	0.320839664	0.756757704	Potri.014G179100.2	AT1G05160.1
Potra2n14c27864.1	-2.774861406	-3.716329347	0.309585925	NA	Potri.014G179000.1	AT5G65940.1
Potra2n14c27865.1					Potri.T002066.1	AT5G36930.2
Potra2n14c27866.1	-0.563492656	1.128015027	0.004120438	0.294386607	Potri.014G179100.2	AT1G05160.1
Potra2n14c27867.2	-0.42814043	0.961935086	0.029672466	0.367683703	Potri.014G179100.2	AT1G05160.1
Potra2n14c27868.1	1.091249762	0.521979907	2.90343E-07	0.349324124	Potri.014G179300.1	AT5G46570.1

Potra2n14c27869.1	5.389532339	1.058467066	1.07111E-07	0.675169873	Potri.014G179400.1	AT1G32450.1
Potra2n14c27870.1	0.247812899	0.178968199	0.08258713	0.824905557	Potri.014G179500.1	AT2G44525.1
Potra2n14c27871.1	0.179073006	0.084806282	0.073636037	0.917516356	Potri.014G179600.1	AT2G44530.1
Potra2n14c27872.1	0.255327763	-0.030957344	0.293932163	0.987226805	Potri.014G179800.1	AT3G17668.1
Potra2n14c27873.1	0.426372163	0.023305928	0.034536159	0.987577016	Potri.014G179900.1	AT4G19020.1
Potra2n14c27874.1	0.411035308	0.060527663	0.028609687	0.961328014	Potri.014G180100.1	AT3G60160.1
Potra2n14c27875.1	1.124824408	0.179971216	2.40273E-08	0.93125845	Potri.014G180200.1	AT1G08840.2
Potra2n14c27876.1					Potri.005G035200.1	AT1G75130.1
Potra2n14c27877.1	0.82357019	2.295459777	NA	NA	Potri.014G180501.1	AT1G75130.1
Potra2n14c27878.1					Potri.014G180300.5	AT1G02205.2
Potra2n14c27879.1						
Potra2n14c27880.1	1.545832463	NA	NA	NA	Potri.014G180300.5	AT1G02205.2
Potra2n14c27881.1	NA	2.293692629	NA	NA	Potri.005G035200.1	AT1G75130.1
Potra2n14c27882.1						
Potra2n14c27883.1	1.077145761	-0.407706537	0.258641849	0.963557834	Potri.014G180600.1	AT5G66730.1
Potra2n14c27884.1	-0.041334433	0.061186244	0.770497347	0.937846405	Potri.014G180700.1	AT1G45150.1
Potra2n14c27885.1	0.229145332	0.088945178	0.157099458	0.939653927	Potri.014G180800.1	AT5G42970.1
Potra2n14c27886.1	0.82357019	NA	NA	NA		
Potra2n14c27887.1						
Potra2n14c27888.1	-0.069609706	-0.161832049	0.667620413	0.82876584	Potri.014G181000.1	AT1G45170.1
Potra2n14c27889.1	0.806893606	0.068893389	0.194295534	0.979967506	Potri.014G181200.1	AT3G56870.1
Potra2n14c27890.1	0.80459295	0.137384613	0.382451497	0.974756511		
Potra2n14c27891.3	-0.278747362	0.584930804	0.256111844	0.576548403	Potri.014G181500.1	AT1G75160.1
Potra2n14c27892.1	0.037035322	0.064246013	0.919504198	0.976324121		
Potra2n14c27893.1	0.309665564	-0.137249908	0.190143063	0.896089262	Potri.014G181600.1	AT4G36660.1
Potra2n14c27894.1	0.82357019	NA	NA	NA	Potri.002G261900.1	AT3G46940.1
Potra2n14c27895.1	2.579373492	3.415098005	0.299922152	NA	Potri.014G181700.1	AT5G42950.1
Potra2n14c27896.2	1.644810934	0.553030966	5.83807E-11	0.623736221	Potri.014G195100.3	AT1G35710.1
Potra2n14c27897.1	1.572027155	0.580842708	1.3796E-09	0.576228147	Potri.002G246600.1	AT3G25150.2
Potra2n14c27898.1	2.785339844	1.002090198	0.00029116	0.843683654	Potri.014G195200.1	AT4G08850.1
Potra2n14c27899.1	-0.213153059	-0.832825989	0.818925596	0.817191104	Potri.014G195400.1	AT1G10180.1
Potra2n14c27900.1						

Potra2n14c27901.1	0.796692023	0.297390022	4.60645E-08	0.639176817	Potri.014G195500.2	AT1G04010.1
Potra2n14c27902.1						
Potra2n14c27903.1	0.129668925	-0.007582366	0.518193253	0.996032145	Potri.014G195600.1	AT4G28590.1
Potra2n14c27904.1	0.174199786	0.024890286	0.168663475	0.981702181	Potri.014G195700.1	AT2G20420.1
Potra2n14c27905.1						
Potra2n14c27906.1	0.267561691	0.241520669	0.005765098	0.544461626	Potri.014G195800.1	AT5G44070.1
Potra2n14c27907.1	0.599124902	0.109583231	0.000164122	0.933996725	Potri.014G195900.1	AT5G44070.1
Potra2n14c27908.1	0.82357019	1.571529889	NA	NA		
Potra2n14c27909.1						
Potra2n14c27910.1	-0.048903623	-0.09621283	0.796661843	0.935474933	Potri.014G196200.1	AT1G19440.1
Potra2n14c27911.1	0.213470312	0.091250052	0.153750505	0.912814353	Potri.014G196400.1	AT5G42770.2
Potra2n14c27912.1	-0.963830478	-0.056543194	0.487555393	0.993589787	Potri.014G196600.1	AT2G16250.1
Potra2n14c27913.1	-0.065228453	0.147719024	0.743437497	0.874507876	Potri.014G196700.1	AT1G75210.1
Potra2n14c27914.1	-0.932780606	-0.262469938	0.003455055	0.842247476	Potri.014G196800.1	AT5G42765.1
Potra2n14c27915.1	0.162763484	0.319605106	0.201592724	0.590087814	Potri.014G196900.1	AT1G47330.1
Potra2n14c27916.1	0.82357019	2.490806657	NA	NA		
Potra2n14c27917.1	0.407033841	0.158809149	0.000919657	0.701494362	Potri.014G194900.2	AT5G48630.1
Potra2n14c27918.1	1.603639121	0.488538078	4.67724E-07	0.790362046	Potri.014G195000.4	AT3G07270.1
Potra2n14c27919.1					Potri.016G106400.1	
Potra2n14c27920.1					Potri.016G106400.1	
Potra2n14c27921.1						
Potra2n14c27922.1	-0.29626822	-0.052729538	0.190354307	0.971317865	Potri.019G046400.1	AT1G03250.1
Potra2n14c27923.1	0.142159723	0.121383528	0.227430129	0.900015282	Potri.014G191900.1	AT5G61540.1
Potra2n14c27924.1	NA	NA	NA	NA	Potri.002G257700.1	AT1G04040.1
Potra2n14c27925.1	0.206319969	0.334654977	0.209949032	0.379776942	Potri.014G192200.1	AT5G44010.1
Potra2n14c27926.1	-0.328519065	-0.207844054	0.000548436	0.41743216	Potri.014G190300.1	AT5G55160.1
Potra2n14c27927.1	0.661738069	0.307889025	3.6463E-08	0.269417208	Potri.014G190600.1	AT3G07140.1
Potra2n14c27928.1	0.001182354	0.207387521	0.993527652	0.492226875	Potri.014G190800.1	AT3G07170.1
Potra2n14c27929.1	-0.027441671	0.363048062	0.908686821	0.494451924	Potri.002G248400.2	AT3G07330.1
Potra2n14c27930.1	0.9457344	0.403085006	4.15341E-06	0.696743275	Potri.001G353900.1	AT4G18100.1
Potra2n14c27931.2	0.117170018	0.147290959	0.123660533	0.794596445	Potri.014G191100.2	AT5G48520.1
Potra2n14c27932.1	-0.26398717	0.273393258	0.387676937	0.883788696	Potri.014G191200.1	AT5G48910.1

Potra2n14c27933.1	0.415160968	0.290431915	0.014613781	0.439101966	Potri.014G191400.1	AT5G48655.2
Potra2n14c27934.1					Potri.014G191500.1	AT5G48660.1
Potra2n14c27935.1	2.940737952	1.390735714	1.02698E-05	0.859377816		
Potra2n14c27936.1	2.895730044	2.181488689	9.42916E-06	0.580376662		
Potra2n14c27937.1	2.001656147	1.697156617	1.09005E-12	0.240647938	Potri.012G145600.1	AT1G07780.1
Potra2n14c27938.1	1.234372646	0.961565762	0.336085249	0.862335622	Potri.012G145600.1	AT1G07780.1
Potra2n14c27939.1	0.588425086	0.097770657	0.010520211	0.943411075	Potri.014G189400.1	AT3G07930.3
Potra2n14c27940.1	0.628464917	0.07271186	0.004203046	0.961077436	Potri.014G187300.1	AT3G07930.3
Potra2n14c27941.1	-0.24720026	-0.041642279	0.075264154	0.973948905	Potri.014G189500.1	AT3G07080.1
Potra2n14c27942.1	0.783665733	0.044913118	0.057439915	0.986636967	Potri.014G189600.1	AT5G01630.1
Potra2n14c27943.1	NA	NA	NA	NA	Potri.014G188600.1	AT2G01050.1
Potra2n14c27944.1						
Potra2n14c27945.1	-0.086972486	0.0568531	0.674573713	0.965535524	Potri.014G189900.1	AT3G24870.1
Potra2n14c27946.1	NA	1.571529889	NA	NA	Potri.013G129500.2	AT5G09950.1
Potra2n14c27947.1	-0.325700887	2.985467097	0.899483173	0.379734246	Potri.014G187800.1	AT2G01050.1
Potra2n14c27948.2	-0.219001099	-0.024116724	0.211045835	0.989304496	Potri.014G194300.1	AT2G20300.1
Potra2n14c27949.1	0.36788117	0.322601968	0.058826314	0.462241775	Potri.014G194500.1	AT4G28740.1
Potra2n14c27950.1	-0.518127115	0.064365419	0.001978056	0.958679295	Potri.014G194600.10	AT4G28760.2
Potra2n14c27951.2	-0.121119437	0.136106452	0.290457459	0.895460013	Potri.014G194800.3	AT2G20210.1
Potra2n14c27952.1	0.119085266	0.013714879	0.339994014	0.987226805	Potri.014G194700.1	AT4G29040.1
Potra2n14c27953.1	-0.701976515	-0.030098946	0.566384981	0.996032145	Potri.T037000.2	AT2G01050.1
Potra2n14c27954.1						
Potra2n1500s37111.1	1.664677056	NA	NA	NA	Potri.004G005000.1	AT1G62340.1
Potra2n1506s37112.1	-0.590101329	-0.583185841	0.1388044	0.495389495	Potri.011G136400.1	AT1G50910.1
Potra2n1509s37113.1	2.422808771	1.541470449	0.000424931	0.677013336	Potri.001G235800.1	AT2G37430.1
Potra2n1509s37114.1	2.380895921	1.36006021	0.001810177	0.677013336	Potri.001G235800.1	AT2G37430.1
Potra2n1510s37115.1	1.151368808	0.146824206	0.385661598	NA	Potri.001G441000.1	AT2G19130.1
Potra2n1511s37116.1					Potri.008G111700.1	AT1G26160.1
Potra2n1512s37117.2					Potri.014G132500.1	AT4G13420.1
Potra2n1513s37118.1	NA	NA	NA	NA	Potri.012G009300.1	AT5G53540.1
Potra2n1517s37119.1	NA	NA	NA	NA		
Potra2n1522s37120.1					Potri.004G170300.2	AT3G14470.1

Potra2n1524s37121.1					Potri.004G192500.1	AT1G16260.2
Potra2n1527s37122.1	NA	NA	NA	NA	Potri.001G357900.1	AT4G05420.1
Potra2n1529s37123.2	4.595142855	1.68758834	1.58341E-06	NA	Potri.001G018700.1	AT3G20860.1
Potra2n1533s37124.1	-0.001545106	-1.507841271	NA	NA		
Potra2n1534s37125.1	1.64774763	NA	NA	NA	Potri.014G037400.1	AT4G31940.1
Potra2n1537s37126.2	NA	NA	NA	NA	Potri.016G020400.1	AT1G22340.1
Potra2n1537s37127.1					Potri.016G020700.1	AT1G22380.1
Potra2n1538s37128.1					Potri.001G462200.1	AT1G30700.1
Potra2n1542s37129.1	0.82357019	1.571529889	NA	NA	Potri.012G109200.1	AT5G61840.1
Potra2n1545s37130.1	-0.949755873	0.829702751	NA	NA	Potri.006G060100.2	AT2G47910.1
Potra2n1547s37131.1					Potri.018G033100.1	
Potra2n1548s37132.1					Potri.018G135800.1	AT3G14460.1
Potra2n1549s37133.1	0.999340838	0.709691105	0.350975729	0.928921164	Potri.007G082900.1	AT3G26300.1
Potra2n154s34738.1	NA	NA	NA	NA		
Potra2n154s34739.1						
Potra2n154s34740.1					Potri.T087000.1	AT4G31980.1
Potra2n154s34741.1	NA	NA	NA	NA	Potri.T087300.1	AT4G31980.1
Potra2n154s34742.1	-1.024567177	-0.268039334	0.005720324	0.842227981	Potri.003G206000.1	AT5G12950.1
Potra2n154s34743.1	-0.666814057	-0.289034431	0.051005364	0.809914834		
Potra2n1550s37134.1	3.942031811	2.095966774	2.25351E-06	0.531258209	Potri.001G415100.3	AT5G23960.2
Potra2n1551s37135.1	0.82357019	NA	NA	NA		
Potra2n1552s37136.1						
Potra2n155s34744.1	-1.074089494	-0.47084187	3.76316E-07	0.309829472	Potri.006G106900.1	AT2G40435.1
Potra2n155s34745.1	3.20104637	3.722944443	NA	NA		
Potra2n1561s37137.1	NA	NA	NA	NA	Potri.T038300.2	AT5G36930.2
Potra2n1562s37138.1	1.629933523	-0.261743785	0.027143231	0.966917036	Potri.016G102700.1	AT2G19130.1
Potra2n156s34746.1	6.011020975	5.773048174	NA	NA	Potri.010G007900.1	AT1G17860.1
Potra2n156s34747.1	1.161961513	-2.013568191	0.75881589	NA	Potri.010G007401.1	
Potra2n156s34748.1	1.729713438	-0.767708831	NA	NA	Potri.010G007401.1	
Potra2n156s34749.1	1.729713438	0.8502037	NA	NA	Potri.008G218700.1	
Potra2n156s34750.1					Potri.010G007300.1	AT4G18180.1
Potra2n156s34751.1	-0.09782259	-0.094786542	0.574511175	0.942348271	Potri.010G007200.1	AT3G06790.2

Potra2n1570s37139.1	5.057608272	NA	0.001769337	NA	Potri.T011750.1	AT5G36930.1
Potra2n1572s37140.1	1.64774763	NA	NA	NA		
Potra2n157s34752.1	2.492428534	NA	NA	NA	Potri.003G186100.1	AT1G16260.2
Potra2n157s34753.1					Potri.003G186100.1	AT1G16260.2
Potra2n157s34754.1	4.955019556	2.435719143	0.000550081	NA	Potri.003G186200.1	AT1G16260.2
Potra2n157s34755.1	2.837750539	NA	NA	NA	Potri.T080900.1	AT1G16260.2
Potra2n157s34756.1	NA	NA	NA	NA	Potri.003G186300.1	AT5G22250.1
Potra2n157s34757.1						
Potra2n157s34758.1	4.402655224	1.777204181	0.037580879	NA	Potri.T080900.1	AT1G16260.2
Potra2n157s34759.1	8.168223379	3.558750257	6.4743E-30	0.365910084	Potri.003G185800.1	AT1G16260.2
Potra2n157s34760.1	0.978788686	0.340878469	1.50674E-07	0.34027789	Potri.003G185600.1	AT1G79690.1
Potra2n157s34761.1	7.614720993	2.36439873	6.94667E-08	NA	Potri.T080900.1	AT1G16260.2
Potra2n157s34762.1	0.764381185	0.33100976	5.20265E-08	0.415978723	Potri.003G185600.1	AT1G79690.1
Potra2n1585s37141.1	-1.875849517	-0.586350402	NA	NA	Potri.003G029132.1	AT1G15780.1
Potra2n158s34763.1	1.855136033	-0.158766283	0.006468356	0.985393296	Potri.019G050900.2	AT5G16770.1
Potra2n158s34764.1	0.213043357	0.068267936	0.201315132	0.956865545	Potri.019G050600.10	AT1G23860.1
Potra2n158s34765.1	-0.462811415	-0.028755629	0.003480544	0.985263255	Potri.019G050500.1	AT5G16810.1
Potra2n158s34766.1	1.242240208	0.498185797	1.86594E-25	0.350883772	Potri.013G128300.5	AT3G59780.1
Potra2n158s34767.1	3.554274858	1.778926038	2.57381E-16	0.144632206	Potri.019G113701.1	AT5G17680.1
Potra2n158s34768.1	2.22704651	1.028141614	0.205900258	NA	Potri.019G098300.1	AT5G49960.1
Potra2n158s34769.1	2.476290371	NA	NA	NA		
Potra2n158s34770.1	1.277743719	0.136749065	NA	NA		
Potra2n158s34771.1	0.165816801	-1.218203907	0.941219977	NA	Potri.008G054801.1	AT5G02500.1
Potra2n158s34772.1	0.202497203	0.365192885	0.790752262	0.920141992	Potri.013G128300.5	AT3G59780.1
Potra2n158s34773.1	0.376247659	0.272152719	0.673580998	0.969421568		
Potra2n158s34774.1	1.920912974	-0.736471817	0.230760373	NA	Potri.019G112600.1	AT4G12010.1
Potra2n158s34775.1	4.379605168	2.60306301	0.10241372	NA	Potri.019G113701.1	AT5G17680.1
Potra2n158s34776.1	0.028777894	0.885772804	NA	NA	Potri.019G050200.1	AT4G24340.1
Potra2n1591s37142.1	NA	NA	NA	NA	Potri.015G004400.2	AT2G02880.1
Potra2n1591s37143.1	NA	NA	NA	NA		
Potra2n1593s37144.1	5.955987719	1.571529889	0.000220523	NA	Potri.011G028300.1	AT4G05200.1
Potra2n1595s37145.1	-0.298240696	-0.215264788	0.824050596	0.961670176	Potri.004G178700.1	AT4G35140.1

Potra2n159s34777.2	-0.071873378	0.033385666	0.883720657	0.992934677	Potri.T087750.1	AT1G61180.1
Potra2n159s34778.1	NA	NA	NA	NA	Potri.001G446800.1	AT1G79160.1
Potra2n15c27955.1	-0.235551424	0.179841274	0.125897001	0.884463725	Potri.015G148300.1	AT3G02680.1
Potra2n15c27956.1	4.584671077	1.216728991	3.914E-13	0.779493734	Potri.015G148200.1	AT1G65690.1
Potra2n15c27957.1	0.946703684	0.056484588	1.85709E-08	0.976302215	Potri.015G147900.1	AT5G37010.1
Potra2n15c27958.1	0.722232365	1.935709526	0.763707554	0.679485917	Potri.015G147800.1	AT1G58110.1
Potra2n15c27959.1	1.12261643	0.004671178	4.99391E-08	0.999121696	Potri.015G147600.2	AT4G25640.1
Potra2n15c27960.1	-0.957813858	-0.385107462	1.51091E-05	0.543273485	Potri.015G147400.2	AT3G49070.1
Potra2n15c27961.1	-0.853401956	-0.693464622	1.08636E-05	0.663309616	Potri.015G147300.1	AT5G23940.1
Potra2n15c27962.2	0.560809102	0.360146276	2.90343E-07	0.524846781	Potri.015G147200.1	AT4G09980.1
Potra2n15c27963.1	0.114333406	0.035527839	0.507100261	0.976302215	Potri.015G147100.1	AT5G52440.1
Potra2n15c27964.2	-0.010557719	-0.188304268	0.943195536	0.570201093	Potri.015G147000.1	AT5G56760.1
Potra2n15c27965.3	-0.541158421	-0.296874665	0.035452426	0.854608397	Potri.015G146900.1	AT1G02260.1
Potra2n15c27966.4	-0.334771559	-0.063489846	0.00046668	0.936402128	Potri.015G146700.1	AT1G63700.1
Potra2n15c27967.2	0.23289305	0.053552261	0.335378117	0.978251387	Potri.015G146600.1	AT4G23420.2
Potra2n15c27968.4	-0.104385294	0.012210489	0.506945193	0.993630002	Potri.015G146500.1	AT3G61415.1
Potra2n15c27969.1	0.242006949	0.098810785	0.431736664	0.967076029	Potri.015G146400.1	AT5G52420.1
Potra2n15c27970.1	0.120067544	0.168087701	0.255296753	0.798673448	Potri.015G146300.1	AT4G25610.1
Potra2n15c27971.1	0.236001567	-0.08777688	0.186153174	0.935995049	Potri.015G146100.1	AT3G49055.1
Potra2n15c27972.1	0.362828239	-0.056227709	0.00383857	0.950878725	Potri.015G146000.1	AT5G23910.1
Potra2n15c27973.1	0.23719676	-0.001265408	0.100199985	0.999458638	Potri.015G145800.1	AT3G53740.2
Potra2n15c27974.1	-0.136208607	-0.033884866	0.239705153	0.954559968	Potri.015G145700.2	AT5G23890.1
Potra2n15c27975.1	0.604413658	0.206700155	0.000208916	0.807968523	Potri.015G145600.2	AT4G33410.1
Potra2n15c27976.1	1.545820342	0.598261177	1.06423E-07	0.652378517	Potri.015G145400.1	AT5G23870.3
Potra2n15c27977.1	1.0127999	0.284925938	0.002229608	0.907073354	Potri.015G145400.1	AT5G23870.3
Potra2n15c27978.3	-0.625012561	0.059265546	0.000158939	0.978203092	Potri.015G145300.2	AT4G20850.1
Potra2n15c27979.1	NA	NA	NA	NA	Potri.015G145200.1	AT4G20850.1
Potra2n15c27980.1	NA	NA	NA	NA	Potri.015G145100.1	AT5G52400.1
Potra2n15c27981.1	0.061893993	-0.159745535	0.929046558	0.971508752	Potri.015G145000.1	AT1G01730.1
Potra2n15c27982.2	-0.279878173	-0.14482938	0.012102987	0.838650065	Potri.015G144900.5	AT3G49000.1
Potra2n15c27983.1	7.748369124	5.421167566	2.76113E-33	0.026868285	Potri.015G144800.1	AT5G52390.1
Potra2n15c27984.1	-0.164631562	0.487804733	0.656514348	0.764774301	Potri.015G144700.1	AT3G48990.1

Potra2n15c27985.1	0.122382178	-0.100710733	0.624363301	0.948577865	Potri.015G144600.1	AT5G52380.1
Potra2n15c27986.3	0.76151171	0.138460928	6.3674E-06	0.918482984	Potri.015G144400.1	AT5G23850.1
Potra2n15c27987.1	-1.998051083	-1.066237839	3.62545E-14	0.524846781	Potri.015G144300.1	AT3G48970.1
Potra2n15c27988.1	-0.797510891	-0.251521438	3.89674E-05	0.880316339	Potri.015G144200.1	AT5G23760.1
Potra2n15c27989.3	-0.238335363	-0.148607757	0.18384396	0.851087483	Potri.015G144100.1	AT1G55630.1
Potra2n15c27990.1	-0.718066709	-1.552032129	0.422609013	0.217664621	Potri.012G141300.1	AT4G25580.1
Potra2n15c27991.1	-0.279499168	-0.066548424	0.102999854	0.971828097	Potri.015G143800.1	AT5G52280.1
Potra2n15c27992.1	-0.194807185	0.032840164	0.502806306	0.987226805		
Potra2n15c27993.1	1.501924108	0.892228835	9.73577E-23	1.06561E-06	Potri.015G143700.1	AT4G25570.1
Potra2n15c27994.1	-1.240965888	-0.155682846	3.75741E-09	0.935851032	Potri.015G143600.1	AT5G23750.2
Potra2n15c27995.1	-0.356564142	-0.316714843	0.668181749	0.920028955	Potri.015G143500.1	AT3G61250.1
Potra2n15c27996.1	0.156433189	-0.085654957	0.247719922	0.935099709	Potri.015G143200.1	AT5G23730.1
Potra2n15c27997.4	0.070778474	0.142217146	0.724405874	0.872233138	Potri.015G142900.1	AT4G25540.1
Potra2n15c27998.1	0.631602988	0.426120856	0.03962477	0.762567828	Potri.015G143100.1	AT1G79600.1
Potra2n15c27999.1	0.068847599	0.108635001	0.785044651	0.922778027	Potri.015G142800.4	AT3G48900.2
Potra2n15c28000.1	0.081386143	-0.059279891	0.837727792	0.971828097	Potri.015G142700.1	AT3G47640.3
Potra2n15c28001.1	-0.212808283	-0.193106604	0.131380999	0.816567218	Potri.015G142600.1	AT3G22950.2
Potra2n15c28002.1	0.078300408	-0.098280148	0.637341124	0.914172897	Potri.015G142500.1	AT3G22942.1
Potra2n15c28003.1	-0.119615676	0.138387062	0.438160718	0.855611673	Potri.015G142200.1	AT5G62070.1
Potra2n15c28004.1	-1.041038655	-0.468921701	3.45179E-11	0.526367527	Potri.015G142000.1	AT5G07250.1
Potra2n15c28005.1	0.396010883	-0.069283209	0.020401765	0.952831766	Potri.015G141900.2	AT2G36620.1
Potra2n15c28006.1	0.278306699	-0.461239682	0.847078107	NA	Potri.015G141800.1	AT4G00730.1
Potra2n15c28007.1	-0.366905864	-0.119025212	0.009926407	0.874808359	Potri.015G141700.2	AT4G33495.1
Potra2n15c28008.1	-1.192640724	-0.602959358	1.57421E-09	0.050626449	Potri.015G141400.1	AT5G07270.1
Potra2n15c28009.1	1.64774763	NA	NA	NA	Potri.015G141300.1	AT5G62040.1
Potra2n15c28010.1	-0.979932101	-0.502630901	0.237933773	0.890369631	Potri.015G141200.1	AT5G07280.1
Potra2n15c28011.1	-0.247880481	-0.416847377	0.382718199	0.444573414	Potri.015G141100.1	AT5G62020.1
Potra2n15c28012.1	0.790087566	-1.458939475	0.740596912	NA	Potri.015G141000.1	
Potra2n15c28013.1	0.713179005	-2.079390114	0.820627186	NA		
Potra2n15c28014.1	-0.437516337	-0.362268258	0.107207306	0.626455563	Potri.015G140900.1	AT5G52180.1
Potra2n15c28015.1	-1.57268507	-0.551997577	7.07096E-05	0.810799242	Potri.015G140800.1	
Potra2n15c28016.1	0.059262708	0.03825927	0.577342482	0.951726178	Potri.015G140500.2	AT5G52200.2

Potra2n15c28017.1	-1.133360102	0.721628554	0.770450143	NA	Potri.012G138800.1	AT5G13930.1
Potra2n15c28018.1	-0.174232782	-0.099954235	0.231811853	0.88643982	Potri.015G140400.1	AT5G52210.1
Potra2n15c28019.3	0.446876318	-0.432553645	0.653183563	0.92978159	Potri.015G140200.1	AT5G52220.2
Potra2n15c28020.1	0.194517838	0.243020593	0.204999842	0.792864576	Potri.015G140100.1	AT5G52230.1
Potra2n15c28021.1	-0.490813737	0.316755195	0.680819199	0.954419252	Potri.015G139966.1	
Potra2n15c28022.1	1.459962178	0.674963689	NA	NA	Potri.015G139900.1	
Potra2n15c28023.1	-0.022873842	-0.028331497	0.906996136	0.981772014	Potri.015G139800.1	AT3G48890.1
Potra2n15c28024.1	-0.222953536	-0.028942316	0.321740662	0.983641743	Potri.015G139700.1	AT3G48880.2
Potra2n15c28025.2	-1.889064842	-1.94054323	0.500600896	NA	Potri.015G139600.1	AT3G13760.1
Potra2n15c28026.1	0.82357019	2.146414233	NA	NA		
Potra2n15c28027.2	-0.67204558	-0.334541187	0.034860809	0.884463725	Potri.015G139400.1	AT3G03620.1
Potra2n15c28028.1	0.954666532	-0.04496425	0.005288532	0.987288338	Potri.015G139300.1	AT4G38180.1
Potra2n15c28029.1	1.830287453	0.797763456	0.032319422	0.917516356	Potri.015G139100.1	AT5G52160.1
Potra2n15c28030.1	NA	NA	NA	NA	Potri.015G139000.1	AT2G45750.1
Potra2n15c28031.1	-0.100213025	-0.317486987	0.743474902	0.804796567	Potri.015G138900.1	AT1G33720.1
Potra2n15c28032.3	0.210613723	0.10189422	0.21121589	0.915580711	Potri.015G138800.1	AT5G51940.1
Potra2n15c28033.1	0.68668294	0.507267615	0.038375816	0.842247476	Potri.015G138600.1	AT5G52120.1
Potra2n15c28034.2	-0.062890743	-0.09781514	0.613600734	0.906877588	Potri.015G138500.1	AT5G62090.2
Potra2n15c28035.1	-0.272378527	-0.108755332	0.190986253	0.943819961	Potri.015G138400.1	AT5G52110.1
Potra2n15c28036.1	0.895755918	0.236148435	0.001900835	0.910815461	Potri.015G138200.2	AT5G51910.2
Potra2n15c28037.1	0.133139464	0.076748502	0.509829485	0.959881296	Potri.015G138100.1	AT4G18372.1
Potra2n15c28038.1	-0.047427743	-0.181610982	0.882790403	0.905231727	Potri.012G135600.1	AT4G38010.1
Potra2n15c28039.1	-0.013408321	-0.14494814	0.965959132	0.929424842	Potri.015G137900.2	AT5G51920.1
Potra2n15c28040.1	0.021690109	-0.10695778	0.945572916	0.95671968		
Potra2n15c28041.1	0.487573957	0.140772921	0.047149315	0.914162682	Potri.015G137600.1	AT4G26810.1
Potra2n15c28042.1	1.322851998	0.446995267	0.008781757	0.854880262	Potri.015G137500.2	AT4G25433.1
Potra2n15c28043.1	0.439865948	0.682262923	0.586902714	0.788065047	Potri.015G137400.1	
Potra2n15c28044.1	0.029295292	-0.033001763	0.802437691	0.974367223	Potri.015G137200.1	AT4G25440.1
Potra2n15c28045.1	1.910110323	0.643144385	0.00062554	0.856337524	Potri.015G137100.1	AT5G62180.1
Potra2n15c28046.1	0.089079005	-0.217447433	0.478484811	0.776094736	Potri.015G137000.1	AT1G53645.1
Potra2n15c28047.1	-0.334992406	0.177434399	0.034103982	0.897285619	Potri.015G136900.1	AT1G12580.1
Potra2n15c28048.1	0.132235622	0.281095168	0.534099215	0.759797728	Potri.015G136700.2	AT4G25450.1

Potra2n15c28049.1	0.447503101	0.204797056	0.014301903	0.613016988	Potri.015G136600.1	AT1G16560.3
Potra2n15c28050.1	0.451848559	-0.263444904	0.50752196	0.924256651	Potri.015G136500.1	AT1G06010.1
Potra2n15c28051.1	2.200482673	-1.161634323	NA	0.82876584	Potri.015G136300.1	AT5G52020.1
Potra2n15c28052.1	0.245170784	-0.024939653	NA	NA	Potri.012G133900.1	AT5G52030.2
Potra2n15c28053.1	NA	NA	NA	NA	Potri.015G136000.1	AT5G07210.1
Potra2n15c28054.1					Potri.012G133700.1	AT3G26660.1
Potra2n15c28055.1	-0.219566873	-0.036610267	0.253335298	0.976302215	Potri.015G135800.1	AT4G25500.4
Potra2n15c28056.1					Potri.015G136000.1	AT5G07210.1
Potra2n15c28057.1	-0.61910233	2.143173882	NA	NA	Potri.012G133700.1	AT3G26660.1
Potra2n15c28058.4	-0.199961607	-0.158426123	0.021306223	0.811742964	Potri.015G135800.1	AT4G25500.4
Potra2n15c28059.1	-0.057142944	0.756827186	0.945122827	0.485359736	Potri.015G135600.1	AT4G23030.1
Potra2n15c28060.1	-2.277249482	-0.808779522	NA	NA	Potri.003G008100.1	AT1G30480.1
Potra2n15c28061.2	-0.556085055	-0.477979023	0.013452949	0.743252386	Potri.015G135500.1	AT5G52060.1
Potra2n15c28062.1	-2.411785055	-0.58210084	3.56333E-07	0.817452127	Potri.015G135400.2	AT5G52100.1
Potra2n15c28063.1	0.194744586	-0.080532223	0.221498531	0.953108412	Potri.015G135300.1	AT5G51880.1
Potra2n15c28064.1	-0.942598987	0.21054386	0.374738907	0.971828097	Potri.015G135200.1	AT5G62170.1
Potra2n15c28065.1	-0.155807877	-0.153028567	0.44393635	0.890446513	Potri.015G135000.1	AT5G51840.1
Potra2n15c28066.1	-0.61910233	-0.767708831	NA	NA	Potri.003G152300.3	
Potra2n15c28067.1	0.908090776	0.149455856	6.07291E-07	0.749682671	Potri.015G134900.2	AT5G51830.1
Potra2n15c28068.1	0.73862675	-0.767708831	NA	NA	Potri.015G134800.1	AT5G15800.1
Potra2n15c28069.1	-0.803676091	-0.315480376	9.53389E-08	0.578615075	Potri.015G134700.1	AT5G51820.1
Potra2n15c28070.1	-2.569998343	-0.653895999	5.82641E-36	0.408488494	Potri.015G134600.1	AT5G51810.1
Potra2n15c28071.1	NA	NA	NA	NA	Potri.015G134500.1	AT1G52540.1
Potra2n15c28072.1	5.568094239	NA	0.001392023	NA	Potri.015G134400.1	AT4G25410.1
Potra2n15c28073.1	0.53297932	-0.178792859	0.590676919	0.983474808	Potri.015G134300.1	AT4G25410.1
Potra2n15c28074.1	0.019803589	0.364543321	0.938790694	0.322673812	Potri.015G134100.1	AT4G25390.1
Potra2n15c28075.1	-1.400615984	1.831646675	0.601438697	0.833198716	Potri.015G133900.4	AT5G51760.1
Potra2n15c28076.1	1.758341361	1.044491047	0.714234031	NA		
Potra2n15c28077.1	3.234730334	0.302277194	0.027965031	NA	Potri.015G133800.1	AT5G51750.1
Potra2n15c28078.1	0.943350674	2.713958262	NA	NA	Potri.015G133700.1	AT1G68530.1
Potra2n15c28079.2	0.767505358	0.130392992	1.6798E-06	0.872201371	Potri.015G133400.1	AT5G62190.1
Potra2n15c28080.1	-1.014723541	0.105233786	0.006647772	0.972159498	Potri.015G133100.1	AT4G21120.1

Potra2n15c28081.1	-1.723462217	0.306236446	0.033185908	0.92978159	Potri.015G133001.1	
Potra2n15c28082.1	0.82357019	3.119987687	NA	NA		
Potra2n15c28083.1						
Potra2n15c28084.1	2.970327426	1.115061349	1.25078E-19	0.439101966	Potri.015G132900.1	AT5G62200.1
Potra2n15c28085.1	NA	NA	NA	NA	Potri.015G132800.1	AT4G39490.1
Potra2n15c28086.1	-0.322857705	-0.328212897	0.014360265	0.684244573	Potri.015G132700.1	AT5G62200.1
Potra2n15c28087.1	1.067944059	-0.0375794	0.005199544	0.987226805	Potri.015G132600.1	AT5G62220.1
Potra2n15c28088.1	0.166944908	0.181525465	0.549590391	0.88661378	Potri.015G132500.1	AT5G51720.1
Potra2n15c28089.1	-0.169466581	0.047505489	0.346092608	0.976963804	Potri.015G132400.1	AT5G51710.2
Potra2n15c28090.1	0.781365074	0.261093575	1.13507E-07	0.805461106	Potri.015G132300.1	AT5G51700.1
Potra2n15c28091.2	2.45422369	1.944362655	0.385014278	NA	Potri.015G132200.1	AT5G62230.1
Potra2n15c28092.1	-0.151567972	-0.164071238	0.206211297	0.805026352	Potri.015G132100.3	AT5G51690.1
Potra2n15c28093.1	-1.045563602	-0.130788531	7.99418E-18	0.833952007	Potri.015G131800.1	AT5G51670.1
Potra2n15c28094.1	0.019603453	0.057212211	0.934573625	0.95671968	Potri.015G131700.2	AT4G25370.1
Potra2n15c28095.1	0.272814155	-0.034232381	0.132296517	0.974367223	Potri.015G131600.1	AT5G51640.1
Potra2n15c28096.3	-0.067277558	-0.724807735	0.896155321	0.483142108	Potri.015G131400.1	AT5G51600.1
Potra2n15c28097.1	0.763863259	-0.013198755	6.46994E-10	0.992934677	Potri.015G131200.1	AT3G22660.1
Potra2n15c28098.1	1.010760185	0.214517225	7.47314E-08	0.849815276	Potri.015G131100.1	AT2G36530.1
Potra2n15c28099.1	0.311317913	0.207317676	0.048467997	0.885375133	Potri.015G130900.2	AT4G25340.1
Potra2n15c28100.3	-0.660289898	-0.176816882	0.010923699	0.901070867		
Potra2n15c28101.1	0.203110321	0.093175147	0.269839529	0.936549944	Potri.015G130700.2	AT2G44710.1
Potra2n15c28102.1	0.538575763	-0.027283179	3.96133E-06	0.976302215	Potri.015G130600.1	AT5G51570.1
Potra2n15c28103.1	1.545832463	1.381953902	NA	NA	Potri.015G130500.1	AT5G62280.1
Potra2n15c28104.1	-0.343765122	0.135733641	0.934296165	NA	Potri.015G130400.1	AT3G47800.1
Potra2n15c28105.1	NA	NA	NA	NA	Potri.015G130300.1	AT3G55254.1
Potra2n15c28106.1	-0.645249035	-0.105730489	0.47591005	0.985393296		
Potra2n15c28107.1	-0.595952689	1.897378757	NA	NA	Potri.015G130200.1	AT4G35830.1
Potra2n15c28108.1	-1.591589099	-0.489762549	7.16388E-06	0.839137085	Potri.012G128700.1	AT5G51560.1
Potra2n15c28109.1	0.567118692	0.052890404	0.000275347	0.975578749	Potri.015G130000.1	AT4G00100.1
Potra2n15c28110.1	-0.079563244	-0.01122194	0.713121702	0.996032145	Potri.015G129900.1	AT5G62290.2
Potra2n15c28111.1	0.735614428	0.173133394	6.00262E-06	0.86849915	Potri.015G129800.1	AT5G62300.2
Potra2n15c28112.1	-0.081513966	0.034670732	0.75580414	0.9869326	Potri.015G129700.1	AT5G51550.1

Potra2n15c28113.1	-0.40930302	0.005664456	0.009195594	0.996154416	Potri.015G129600.1	AT5G51545.1
Potra2n15c28114.1	-1.053847255	-0.40658262	3.13126E-05	0.754074409	Potri.015G129500.1	AT4G25290.1
Potra2n15c28115.1	-1.060519834	-0.412078089	3.04928E-05	0.749682671	Potri.015G129400.1	AT5G03170.1
Potra2n15c28116.1	-0.413187281	-0.249518214	0.010277377	0.772690179	Potri.015G129300.1	AT5G51540.1
Potra2n15c28117.1	-0.750443699	0.286943801	0.028452686	0.899205262	Potri.015G129200.2	AT5G62310.1
Potra2n15c28118.1	0.537101982	-0.258371569	0.220805697	0.911339141	Potri.015G129100.1	AT4G22680.1
Potra2n15c28119.3	-0.222457061	-0.162940521	0.112897094	0.909040554	Potri.015G129000.1	AT4G25280.1
Potra2n15c28120.1	0.102236182	-0.767708831	NA	NA	Potri.015G128900.1	AT4G25250.1
Potra2n15c28121.1	-0.076677294	-0.287277701	0.823328453	0.845014308	Potri.015G128800.1	AT4G25270.1
Potra2n15c28122.1	-0.684113082	-0.319535342	0.061582364	0.788065047	Potri.015G128700.1	AT5G62350.1
Potra2n15c28123.1	-0.904263015	-0.339946856	9.21874E-06	0.667038977	Potri.015G128600.1	AT4G37880.1
Potra2n15c28124.1	1.664677056	NA	NA	NA	Potri.015G128400.1	AT5G62360.1
Potra2n15c28125.1	0.39203266	0.970625952	0.755611214	0.828625141	Potri.015G128300.1	AT5G62360.1
Potra2n15c28126.2	-2.277249482	-0.808779522	NA	NA	Potri.015G128000.1	AT1G70710.1
Potra2n15c28127.1	-1.297522392	0.189569047	0.099773782	0.920763566	Potri.015G127900.1	AT1G23210.1
Potra2n15c28128.1	-0.633627601	-0.125054243	0.110715672	0.967122333	Potri.015G127800.1	AT3G47400.1
Potra2n15c28129.1	NA	NA	NA	NA	Potri.015G127700.1	AT2G45220.1
Potra2n15c28130.1	2.19270719	4.346425026	0.42066211	0.522715829	Potri.015G127600.1	AT1G68180.1
Potra2n15c28131.1	1.664677056	1.394613675	NA	NA	Potri.015G127500.1	AT2G45220.1
Potra2n15c28132.1	-0.353862994	0.066176174	0.04232974	0.950182516	Potri.015G122800.1	AT5G18820.1
Potra2n15c28133.1	-0.57295971	0.421165583	0.477545759	0.937146435	Potri.012G124400.1	AT2G35110.2
Potra2n15c28134.1	-1.035987267	-0.171772418	0.004772333	0.941260209	Potri.012G124400.1	AT2G35110.2
Potra2n15c28135.1	0.050166708	0.001701725	0.939555423	0.999536469	Potri.015G122800.1	AT5G18820.1
Potra2n15c28136.1					Potri.002G213700.1	
Potra2n15c28137.1	NA	NA	NA	NA		
Potra2n15c28138.1	-0.335554452	-0.223866575	0.116247604	0.857189843	Potri.015G122800.1	AT5G18820.1
Potra2n15c28139.1	-1.766642691	-0.538556953	2.45807E-07	0.674283226	Potri.015G122700.1	AT3G54950.1
Potra2n15c28140.2	-0.301720197	-0.226951494	0.125918355	0.882699919	Potri.015G122500.1	AT2G35120.1
Potra2n15c28141.2	0.016457804	-0.051735385	0.9309217	0.970817579	Potri.012G123550.1	AT1G16470.2
Potra2n15c28142.1	-1.210842827	-0.15330458	1.55976E-12	0.922011871	Potri.015G122300.1	AT2G35140.1
Potra2n15c28143.1	-0.217001802	0.045889919	0.252406584	0.9761362	Potri.015G122200.1	AT4G19900.1
Potra2n15c28144.1	-0.354817064	-0.39264053	0.39618134	0.814001428	Potri.015G122100.1	AT2G35150.1

Potra2n15c28145.2	7.151195955	4.556354257	3.93487E-46	0.000423591	Potri.017G009100.1	AT1G67000.1
Potra2n15c28146.1	4.170757396	0.731857741	8.25596E-36	0.803948068	Potri.015G121800.1	AT3G14470.1
Potra2n15c28147.1	0.50329558	0.141600472	3.79699E-05	0.877796011	Potri.015G121600.1	AT4G19880.1
Potra2n15c28148.1	-0.389194239	-0.252851477	0.554157883	0.930203195	Potri.015G121500.1	AT1G31175.1
Potra2n15c28149.3	0.24223631	0.074214668	0.112566142	0.941855873	Potri.015G121400.1	AT5G45030.2
Potra2n15c28150.1	0.243630083	0.084419822	0.233014	0.93888209		
Potra2n15c28151.1	-1.721478285	-0.276072147	1.49465E-08	0.900514809	Potri.015G121300.1	
Potra2n15c28152.1	-2.286316826	-0.370791165	NA	NA	Potri.015G121201.1	AT2G22830.1
Potra2n15c28153.1	-0.378062214	-0.115813825	0.002671297	0.836066102	Potri.T135400.1	AT4G25130.1
Potra2n15c28154.1	2.404005651	-0.767708831	NA	NA	Potri.015G112200.1	AT2G44740.1
Potra2n15c28155.1	-0.59160984	-0.169739278	0.006614393	0.889258723	Potri.T135400.1	AT4G25130.1
Potra2n15c28156.1	1.965040177	0.110081123	0.483861335	NA	Potri.015G112200.1	AT2G44740.1
Potra2n15c28157.1	-1.337274518	0.64910011	0.459213485	NA	Potri.015G114800.2	AT5G45540.1
Potra2n15c28158.1	-0.145807471	-0.253331734	0.735232366	0.928738655	Potri.015G111800.1	AT5G61660.1
Potra2n15c28159.1	-0.331376355	-0.077062943	0.037422944	0.926546506	Potri.T136200.1	AT5G51170.1
Potra2n15c28160.1	0.525388144	0.161929188	0.000263767	0.818007496	Potri.001G025300.1	AT2G47110.1
Potra2n15c28161.1	-0.400973756	0.092967798	0.879868832	NA	Potri.015G111300.1	AT5G51160.1
Potra2n15c28162.1	-0.61910233	-0.767708831	NA	NA	Potri.015G111300.1	AT5G51160.1
Potra2n15c28163.1	-0.104373303	-0.089585983	0.48872961	0.932447954	Potri.015G111200.1	AT5G51150.1
Potra2n15c28164.1	0.341969309	0.362487614	0.726073122	0.925035363	Potri.015G111100.1	AT4G17600.1
Potra2n15c28165.1	0.747479479	-0.029613012	0.007741785	0.990389888	Potri.015G111000.1	AT5G07440.1
Potra2n15c28166.1	-0.238780708	-0.233908842	0.074773563	0.642417298	Potri.015G110900.2	AT5G19910.1
Potra2n15c28167.2	0.031760373	-0.07310532	0.836490279	0.936549944	Potri.015G110800.1	AT5G51120.1
Potra2n15c28168.1	2.409567423	NA	NA	NA	Potri.015G110700.1	AT1G69940.1
Potra2n15c28169.1	NA	1.381953902	NA	NA	Potri.015G110600.2	AT1G28710.3
Potra2n15c28170.1	0.048361662	0.444046692	0.909675833	0.80340893	Potri.015G110400.2	AT5G51100.1
Potra2n15c28171.1	-0.067490537	0.381228787	0.885684811	0.849446235	Potri.015G110300.1	AT4G33270.1
Potra2n15c28172.1	0.102236182	-0.046370319	NA	NA	Potri.015G110200.1	AT5G22410.1
Potra2n15c28173.1	0.127238266	0.012561479	0.322626671	0.989304496	Potri.015G110100.1	AT4G00650.1
Potra2n15c28174.1	0.255816691	0.132697174	0.161558452	0.909715408	Potri.015G110000.1	AT5G51070.1
Potra2n15c28175.1	1.537373362	0.582756977	1.53744E-09	0.66992341	Potri.015G109800.2	AT5G51060.1
Potra2n15c28176.2	1.356304792	0.436546831	4.34082E-07	0.798684605	Potri.015G109700.3	AT5G07380.1

Potra2n15c28177.1	-0.181625347	0.165424209	0.250388764	0.845014308	Potri.015G109500.1	AT5G61760.1
Potra2n15c28178.1	2.249600457	1.074459314	3.45556E-10	0.362601699	Potri.015G109400.1	AT5G07360.1
Potra2n15c28179.1	0.710000234	0.03758166	4.61428E-07	0.9761362	Potri.015G109300.1	AT5G07350.1
Potra2n15c28180.1	0.409236619	0.114092839	0.007201899	0.917516356	Potri.015G109200.1	AT5G61790.1
Potra2n15c28181.1	-2.337106966	1.463577599	NA	NA	Potri.015G109100.1	AT5G07330.1
Potra2n15c28182.1					Potri.015G109000.1	AT5G61800.1
Potra2n15c28183.1	0.23252236	0.011498571	0.144193618	0.992934677	Potri.015G108900.2	AT5G51050.1
Potra2n15c28184.1	-1.29947678	0.016165177	0.000201309	0.996032145		
Potra2n15c28185.1	6.16134858	3.192687783	3.51475E-53	0.009063291	Potri.015G108800.1	AT5G61820.1
Potra2n15c28186.1	-0.065641558	-0.175618779	0.785146268	0.907073354	Potri.015G108600.2	AT5G61820.1
Potra2n15c28187.1	6.751300361	3.524904999	3.5271E-111	1.21086E-09	Potri.015G108500.1	AT5G61820.1
Potra2n15c28188.1	0.118548949	0.119643446	0.680513408	0.943483421	Potri.015G108400.1	AT5G51030.1
Potra2n15c28189.1	0.207717139	0.163315506	0.300413429	0.849045107	Potri.015G108300.1	AT5G51040.1
Potra2n15c28190.1	-1.368589964	-0.524587211	0.020707969	0.874348516	Potri.015G108200.1	AT3G23760.1
Potra2n15c28191.3	0.170870985	-0.221187141	0.609012783	0.915432211	Potri.015G108100.1	AT5G51010.1
Potra2n15c28192.1	-0.595952689	0.151993677	NA	NA	Potri.015G116300.1	
Potra2n15c28193.1	NA	2.501508747	NA	NA	Potri.015G113950.1	AT5G45470.1
Potra2n15c28194.1	NA	NA	NA	NA		
Potra2n15c28195.1	-0.61910233	0.673278781	NA	NA		
Potra2n15c28196.1	NA	NA	NA	NA		
Potra2n15c28197.1					Potri.015G115400.1	AT5G45110.1
Potra2n15c28198.1					Potri.015G115400.1	AT5G45110.1
Potra2n15c28199.1	0.312161645	-0.016394376	0.612449713	0.996999319	Potri.012G109600.1	AT5G61840.1
Potra2n15c28200.1	1.536800874	0.130515614	0.561629019	NA	Potri.017G064000.2	AT1G01630.1
Potra2n15c28201.1					Potri.012G109300.1	AT5G50990.1
Potra2n15c28202.1	-0.598454678	0.418125114	0.455611455	0.907073354		
Potra2n15c28203.1	0.425435295	0.215929592	0.2640126	0.909715408	Potri.015G107000.6	AT1G27060.1
Potra2n15c28204.1	-0.11907689	-1.733525037	NA	NA	Potri.004G071600.3	AT2G01570.1
Potra2n15c28205.1	0.757595055	-0.128069172	0.438813602	0.984806304	Potri.012G109600.1	AT5G61840.1
Potra2n15c28206.1	0.855772607	1.911951029	NA	NA	Potri.T042700.1	AT1G01630.1
Potra2n15c28207.1	NA	NA	NA	NA	Potri.012G109300.1	AT5G50990.1
Potra2n15c28208.2	0.565160982	0.370680813	0.025294371	0.590087814	Potri.015G107000.6	AT1G27060.1

Potra2n15c28209.1	NA	NA	NA	NA	Potri.015G106900.1	AT5G61850.1
Potra2n15c28210.1						
Potra2n15c28211.1					Potri.015G106800.1	AT1G29880.1
Potra2n15c28212.1	0.855297899	0.911441888	0.002214927	0.586398858	Potri.015G106700.1	
Potra2n15c28213.2	-0.035095658	0.276933616	0.884645783	0.808174893	Potri.015G106600.2	AT3G59280.1
Potra2n15c28214.1	0.903115266	0.291885822	3.36179E-10	0.654663553	Potri.015G106500.1	AT1G26880.1
Potra2n15c28215.2	0.140702223	0.064550953	0.504634291	0.967103157	Potri.015G106400.1	AT5G61910.4
Potra2n15c28216.1	0.04970371	-0.652997323	0.913874321	0.612648422	Potri.015G106300.1	AT5G50970.1
Potra2n15c28217.1	-0.602732541	-0.177586507	0.010556874	0.885375133	Potri.015G106200.3	AT5G61930.2
Potra2n15c28218.1					Potri.012G107800.1	AT4G23490.1
Potra2n15c28219.1	-3.694134688	-0.282105945	0.018404258	0.955508429	Potri.015G106100.1	AT4G23490.1
Potra2n15c28220.1	-0.352110523	-0.197606048	0.038465322	0.857189843	Potri.015G106000.1	AT5G61960.2
Potra2n15c28221.1	-0.02201711	-0.034350346	0.950609962	0.986190118	Potri.015G105900.1	AT5G50930.1
Potra2n15c28222.1	0.426322298	0.373908735	0.095890308	0.679485917	Potri.012G106600.2	AT4G25080.5
Potra2n15c28223.2	-1.806781808	0.48808696	0.098523253	0.910074792	Potri.015G105800.2	AT2G33550.1
Potra2n15c28224.1	0.158246727	-0.008690587	0.155109923	0.995005375	Potri.015G105600.2	AT5G61970.1
Potra2n15c28225.5	-0.19547476	0.116416436	0.08636761	0.897285619	Potri.015G105500.1	AT5G61980.1
Potra2n15c28226.1	-0.18366722	0.130284243	0.113504925	0.870044019	Potri.015G105400.2	AT5G61990.1
Potra2n15c28227.1	-0.158294719	0.223193523	0.37270991	0.773157146	Potri.015G105300.3	AT5G62000.2
Potra2n15c28228.1	-0.219624904	0.066742868	0.156945391	0.949966783	Potri.015G105200.1	AT3G26744.2
Potra2n15c28229.1	-0.279316927	-0.01910064	0.007733035	0.983113084	Potri.015G105100.3	AT5G50920.1
Potra2n15c28230.1	-0.391969498	-0.159142489	2.61688E-07	0.619575855	Potri.015G105000.1	AT5G23720.1
Potra2n15c28231.1	-1.177613867	-0.484868093	1.12708E-09	0.112106631	Potri.015G104800.1	AT2G47140.1
Potra2n15c28232.1	-1.95770711	-0.482679024	6.54646E-13	0.724604666	Potri.015G104700.1	AT1G28110.1
Potra2n15c28233.1	2.215123189	NA	NA	NA		
Potra2n15c28234.2	0.033453767	-0.014352655	0.803370936	0.987288338	Potri.015G104600.1	AT3G48860.2
Potra2n15c28235.1	-2.9348217	-1.65391544	NA	NA	Potri.015G104500.1	AT4G25050.1
Potra2n15c28236.1	1.217521073	0.388099846	1.261E-05	0.773063484	Potri.015G104400.1	AT5G14040.1
Potra2n15c28237.1	0.133225841	0.013490585	0.530052432	0.994403137	Potri.015G104300.1	AT5G23690.1
Potra2n15c28238.2	3.9835021	1.807390524	0.001066539	NA	Potri.015G104200.1	AT5G50915.1
Potra2n15c28239.1						
Potra2n15c28240.1	NA	NA	NA	NA	Potri.001G155750.1	AT1G21280.1

Potra2n15c28241.1	-0.101195072	0.079546275	0.559692548	0.930598202	Potri.015G104100.1	AT3G48820.1
Potra2n15c28242.1	0.055299993	0.041074517	0.846289785	0.979896883	Potri.015G104000.1	AT3G48800.1
Potra2n15c28243.1	1.477296237	0.674887393	1.92981E-08	0.476972044		
Potra2n15c28244.1	1.428353031	-0.131300484	0.016231909	0.979896883	Potri.015G103800.1	AT5G23670.2
Potra2n15c28245.1	-0.520500007	-0.34990841	0.00156399	0.733263164	Potri.015G103700.1	AT5G50900.1
Potra2n15c28246.1	-2.64692139	-0.439715641	NA	NA		
Potra2n15c28247.1	-3.338967345	-0.889521874	0.002141656	0.902857376	Potri.015G103600.1	AT4G25040.1
Potra2n15c28248.2	-0.060481487	-0.059690926	0.796102175	0.973347974	Potri.015G103500.1	AT5G41110.1
Potra2n15c28249.1	-0.18984215	-0.140727151	0.123067005	0.880553952	Potri.015G103400.1	AT5G50870.1
Potra2n15c28250.7	0.692784117	-0.039360251	1.2693E-10	0.978646026	Potri.015G103300.1	AT5G50860.1
Potra2n15c28251.1	0.367275299	0.13825168	0.890708998	NA	Potri.015G103200.1	
Potra2n15c28252.1	1.813874327	-0.222316429	4.21521E-06	0.972159498	Potri.015G102200.1	AT3G48770.1
Potra2n15c28253.1	-1.139429001	-3.135330602	0.766151016	NA	Potri.015G102400.1	AT5G62000.3
Potra2n15c28254.1	4.515065311	3.110123834	0.070961473	NA	Potri.015G102200.1	AT3G48770.1
Potra2n15c28255.1	0.272573406	0.772115034	0.383895751	0.59185656	Potri.015G102700.1	AT3G48770.1
Potra2n15c28256.1	1.931605898	NA	NA	NA	Potri.015G102400.1	AT5G62000.3
Potra2n15c28257.1	1.76096571	1.529544475	0.450044145	NA	Potri.015G103000.1	AT3G48770.1
Potra2n15c28258.1	1.687109441	-0.405233946	5.63322E-05	0.912083068	Potri.015G102200.1	AT3G48770.1
Potra2n15c28259.1	0.53834727	0.004335663	0.716829396	NA		
Potra2n15c28260.1	NA	NA	NA	NA	Potri.015G102600.1	AT5G54770.1
Potra2n15c28261.1						
Potra2n15c28262.1	0.993997391	-0.336738021	0.108143305	0.937687311	Potri.015G102100.1	AT5G13180.1
Potra2n15c28263.1	0.15910395	-0.158561206	0.395455408	0.930598202	Potri.015G102000.1	AT5G50810.1
Potra2n15c28264.1	0.241161335	-0.132313257	0.068606735	0.886988579	Potri.015G101900.1	AT3G26935.1
Potra2n15c28265.1	NA	1.381953902	NA	NA	Potri.015G101700.1	AT5G23660.1
Potra2n15c28266.1	0.82357019	NA	NA	NA	Potri.015G101600.1	AT5G50790.1
Potra2n15c28267.1	-1.770545368	0.549337907	0.136993051	0.916409329	Potri.015G101500.2	AT5G50790.1
Potra2n15c28268.1	1.64774763	1.363811008	NA	NA	Potri.015G101400.1	AT5G50790.1
Potra2n15c28269.1	-0.43873339	-0.177679585	0.000336951	0.855432225	Potri.015G101300.1	AT5G51140.2
Potra2n15c28270.1	-0.555981703	-0.236578826	0.000104711	0.830470159	Potri.015G101200.1	AT4G24990.1
Potra2n15c28271.1	0.60858896	0.185351004	0.000113599	0.85823491	Potri.015G101100.1	AT3G48730.1
Potra2n15c28272.1	1.580287846	0.499741317	0.343207407	NA	Potri.015G101000.1	AT1G32583.1

Potra2n15c28273.1	1.578268123	NA	NA	NA		
Potra2n15c28274.2	0.134371762	0.006773267	0.436400756	0.996154416	Potri.015G100900.2	AT4G24970.1
Potra2n15c28275.1	0.410129197	0.255594708	0.000577391	0.493990341	Potri.015G100700.1	AT3G48710.1
Potra2n15c28276.1	NA	NA	NA	NA	Potri.015G100600.1	
Potra2n15c28277.1	-0.172374365	0.023943142	0.162099942	0.979299627	Potri.015G100400.1	AT2G47620.1
Potra2n15c28278.1	-0.947002707	-0.128850206	0.004344157	0.949341946	Potri.015G100300.1	AT1G69040.2
Potra2n15c28279.1	-1.644257419	-0.133969738	0.007192463	0.971828097		
Potra2n15c28280.2	0.260881102	0.258311601	0.450619171	0.865591169	Potri.015G100200.2	AT5G63540.1
Potra2n15c28281.1	-0.80847157	0.409011545	0.375082582	0.910815461	Potri.015G099900.1	AT5G50600.1
Potra2n15c28282.1	NA	NA	NA	NA	Potri.015G099900.1	AT5G50600.1
Potra2n15c28283.1	-0.003021928	1.211201538	0.996829767	0.282148815	Potri.015G099800.1	AT5G50610.1
Potra2n15c28284.1	-1.11807772	-0.80917741	0.000233696	0.278072658	Potri.015G099700.2	AT5G50720.1
Potra2n15c28285.1	-1.758786125	-0.29626364	5.7192E-08	0.909617075	Potri.015G099600.1	AT1G08510.1
Potra2n15c28286.1	-1.054591934	-0.318824936	2.6725E-09	0.640068027	Potri.015G099500.1	AT5G50740.1
Potra2n15c28287.1	-0.960361966	-0.290893648	6.52555E-06	0.64562236		
Potra2n15c28288.1	-2.459844035	0.267655777	0.227441311	NA	Potri.015G099400.2	AT5G13090.1
Potra2n15c28289.1	-0.04722373	-1.961262154	0.97620317	NA	Potri.012G101100.1	AT3G02230.1
Potra2n15c28290.1	1.73424227	0.314202717	4.29612E-06	0.890369631	Potri.015G099200.1	AT5G13080.1
Potra2n15c28291.1	-0.913193402	-0.548126521	0.021258634	0.747036979	Potri.015G099100.1	
Potra2n15c28292.1	-0.148719471	0.050244512	0.164448044	0.93835374	Potri.015G099000.2	AT4G24940.1
Potra2n15c28293.1	0.527826531	0.140522247	0.170508627	0.934439727	Potri.015G098900.1	AT5G50670.1
Potra2n15c28294.1	-0.026595574	-0.254775782	0.98838652	NA	Potri.015G098800.1	AT5G63520.1
Potra2n15c28295.1	-0.041383984	0.023584522	0.795703739	0.985393296	Potri.015G098700.1	AT5G63520.1
Potra2n15c28296.1	0.032960378	0.020589851	0.836415445	0.985393296	Potri.015G098600.1	AT3G48680.1
Potra2n15c28297.1	NA	NA	NA	NA	Potri.015G098500.1	
Potra2n15c28298.1	-0.064629688	0.133669726	0.71837676	0.937789963	Potri.015G098400.1	AT4G22950.1
Potra2n15c28299.1	-0.156129119	0.149688288	0.210458757	0.936395694		
Potra2n15c28300.1	-0.796523029	-0.207150579	0.715481319	0.985393296	Potri.015G098200.1	AT3G27030.1
Potra2n15c28301.1	0.216664027	0.100205768	0.94367107	NA		
Potra2n15c28302.1	NA	NA	NA	NA		
Potra2n15c28303.4	-0.459347851	-0.180044807	0.010694633	0.874808359	Potri.015G098100.1	AT5G50640.1
Potra2n15c28304.1	-0.355646008	-0.148790249	NA	NA	Potri.015G098000.1	

Potra2n15c28305.1	-0.186013967	0.126480654	0.250451871	0.935474933	Potri.015G097900.1	AT3G48610.1
Potra2n15c28306.1	-0.239409714	-0.471188144	0.417766672	0.595538573	Potri.015G097800.1	AT5G63480.1
Potra2n15c28307.1	-0.196547084	-0.139755587	0.389224559	0.907073354	Potri.015G097700.1	AT4G24930.1
Potra2n15c28308.1	0.657617849	0.356391243	0.000518638	0.779077802	Potri.015G097500.1	AT5G53150.1
Potra2n15c28309.1	-0.363190437	-0.23851661	0.001729651	0.570643303	Potri.015G097400.1	AT5G63470.2
Potra2n15c28310.1	0.117619497	-0.049933468	0.462522382	0.960656795		
Potra2n15c28311.1	0.587931769	0.278172152	0.001173621	0.817452127	Potri.015G097200.1	AT3G48560.1
Potra2n15c28312.1						
Potra2n15c28313.1	-0.61910233	0.660619236	NA	NA	Potri.015G097100.1	AT3G48560.1
Potra2n15c28314.1	-1.011017111	-0.049813092	5.13442E-06	0.98177802	Potri.015G097000.1	AT4G37100.1
Potra2n15c28315.1	-0.754913208	0.108844883	0.084834062	0.972159498		
Potra2n15c28316.1	-0.904548718	0.117249672	0.001486441	0.966005919	Potri.015G096900.1	AT4G24910.1
Potra2n15c28317.1	-0.90272326	0.135939581	0.00158869	0.958152146		
Potra2n15c28318.1	-0.290882307	-0.140124796	0.242588605	0.911339141	Potri.015G096700.2	AT4G24900.1
Potra2n15c28319.1	2.685015931	NA	NA	NA	Potri.015G096600.1	
Potra2n15c28320.1	-1.219402765	0.229531982	0.025403909	NA	Potri.015G096500.1	AT3G48550.1
Potra2n15c28321.1	0.381697701	0.116458053	0.048374043	0.898853929	Potri.015G096400.1	AT3G48540.1
Potra2n15c28322.1	0.397316997	0.208840184	0.002147575	0.718991569	Potri.015G096300.1	AT1G05350.1
Potra2n15c28323.1	1.141757863	0.768769893	0.001264682	0.723580731	Potri.015G096200.1	AT1G67340.1
Potra2n15c28324.1	0.184242123	-0.067656758	0.154844142	0.947969749	Potri.015G096100.1	AT5G37350.1
Potra2n15c28325.1	-0.210756413	-0.025009969	0.139286155	0.98177802	Potri.015G096000.4	AT5G50410.1
Potra2n15c28326.1	4.951472558	1.402739887	1.04409E-41	0.472327847	Potri.015G095900.1	AT5G50400.1
Potra2n15c28327.1	2.685015931	NA	NA	NA	Potri.015G096000.4	AT5G50410.1
Potra2n15c28328.1	0.200356329	-0.060778989	0.229486631	0.978203092	Potri.015G095700.3	AT4G30930.1
Potra2n15c28329.1	-0.610950617	-0.401167536	0.797470267	NA		
Potra2n15c28330.1	NA	NA	NA	NA		
Potra2n15c28331.1	NA	NA	NA	NA	Potri.005G079600.1	AT1G64940.1
Potra2n15c28332.1	0.288538453	0.22745309	0.016231588	0.788782589	Potri.015G095500.1	AT1G20960.2
Potra2n15c28333.1	0.345830102	0.012490783	0.016186074	0.991036771	Potri.015G095400.2	AT4G24880.1
Potra2n15c28334.1	0.017886765	0.06336799	0.951372889	0.964027241	Potri.015G095100.1	AT5G50390.1
Potra2n15c28335.1	0.109429541	0.067032412	0.422651624	0.953831244	Potri.015G095000.1	AT5G41020.1
Potra2n15c28336.1	-0.167308473	0.02183877	0.654970978	0.993589787		

Potra2n15c28337.1	-0.572726142	0.289257467	NA	NA	Potri.015G094900.1	AT5G25120.1
Potra2n15c28338.2	-0.315132967	-0.029853877	0.002335231	0.98177802	Potri.015G094800.1	AT5G50380.1
Potra2n15c28339.1	0.103877265	0.742266543	0.716577343	0.674175338	Potri.015G094700.1	AT3G48530.1
Potra2n15c28340.1	-0.106919165	-0.064794699	0.444406238	0.943411075	Potri.015G094600.2	AT5G63460.1
Potra2n15c28341.1	0.247761871	0.054919429	0.115570886	0.95946978	Potri.015G094500.1	AT5G14170.1
Potra2n15c28342.1	0.911605635	0.143024986	1.01005E-06	0.935474933	Potri.015G094400.2	AT1G27400.1
Potra2n15c28343.1	0.405146828	-0.015785578	0.007579488	0.991732307	Potri.015G094200.1	AT2G18110.1
Potra2n15c28344.6	0.285588257	0.165358746	0.000162532	0.802942736	Potri.015G094100.2	AT4G02480.1
Potra2n15c28345.1	0.11945094	0.073466097	0.313640819	0.940273987	Potri.015G094000.1	AT4G24840.1
Potra2n15c28346.1	5.267161101	1.571529889	0.001602097	NA	Potri.015G093900.1	
Potra2n15c28347.2	-0.598628374	-0.105745089	0.001310417	0.95671968	Potri.015G093600.1	AT5G63420.1
Potra2n15c28348.1	0.634061596	0.233797724	4.37974E-05	0.697520011	Potri.015G093500.1	AT5G50375.1
Potra2n15c28349.1	0.591183806	-0.122351918	0.705807591	NA	Potri.015G093400.1	AT2G26700.1
Potra2n15c28350.1	-0.630347629	-0.128665249	7.30789E-06	0.903477395	Potri.015G093300.2	AT3G49180.1
Potra2n15c28351.3	-0.747782411	-0.201485713	1.1865E-07	0.817452127	Potri.015G093100.10	AT5G14210.1
Potra2n15c28352.1	-1.317292482	-1.465929376	NA	NA		
Potra2n15c28353.1	0.481234228	0.004496579	0.00384249	0.99812851	Potri.015G092900.1	AT1G56110.1
Potra2n15c28354.1	-0.121860633	-0.428185541	0.733929866	0.802631112	Potri.015G092800.1	AT5G63400.1
Potra2n15c28355.1	0.436614067	0.136736542	5.4642E-06	0.817452127	Potri.015G092700.1	AT5G60160.1
Potra2n15c28356.1	3.058853255	NA	NA	NA	Potri.015G092600.1	AT5G63390.1
Potra2n15c28357.1	0.273862712	0.007459353	0.136544993	0.996032145	Potri.015G092400.2	AT1G29320.1
Potra2n15c28358.1	0.25942403	-0.173430784	0.757092983	0.971977744	Potri.015G092300.1	AT5G63380.1
Potra2n15c28359.1	0.005168367	0.149429758	0.980408129	0.903496619	Potri.015G092200.1	AT1G67570.1
Potra2n15c28360.2	-0.230656268	0.236962215	0.230292164	0.843443324	Potri.015G092100.1	AT1G67580.2
Potra2n15c28361.1	-0.602595215	2.327873159	0.57980349	0.550401019	Potri.015G092000.1	AT5G03860.1
Potra2n15c28362.1	-5.58422158	0.036166684	0.00075239	0.996154416	Potri.015G091900.1	AT5G50360.1
Potra2n15c28363.1	0.11557133	0.218706801	0.465168812	0.775519829	Potri.015G091800.1	AT5G63320.1
Potra2n15c28364.1	0.676544094	0.387837964	0.007908527	0.596772738	Potri.015G091750.1	
Potra2n15c28365.2	-0.476603919	0.012899093	0.000872302	0.994403137	Potri.015G091700.1	AT3G48500.1
Potra2n15c28366.1	NA	NA	NA	NA	Potri.015G091600.1	AT5G40780.1
Potra2n15c28367.1	0.82357019	NA	NA	NA	Potri.015G091600.1	AT5G40780.1
Potra2n15c28368.1	0.102236182	0.8502037	NA	NA	Potri.015G091600.1	AT5G40780.1

Potra2n15c28369.1	1.052699739	0.369115007	0.000611135	0.813575895	Potri.015G091500.1	AT5G50350.1
Potra2n15c28370.3	0.084904023	-0.050948741	0.553829415	0.954419252	Potri.015G091400.1	AT1G13440.1
Potra2n15c28371.1	-0.354784646	-0.588430234	0.933277265	NA		
Potra2n15c28372.1	0.376581275	0.236827207	0.775046561	0.976000742	Potri.015G091100.1	AT3G48490.1
Potra2n15c28373.1	-1.12866173	-0.396308439	6.02113E-05	0.172053694	Potri.015G091000.2	AT5G50330.1
Potra2n15c28374.2	-0.11795502	-0.044067636	0.245617163	0.966005919	Potri.015G090900.1	AT4G24820.2
Potra2n15c28375.1	0.094592306	-0.05107819	0.792616166	0.985393296	Potri.015G090800.1	AT3G48480.1
Potra2n15c28376.1	-0.093270583	-0.015511242	0.585731474	0.987226805	Potri.015G090700.1	AT3G48470.1
Potra2n15c28377.1	0.299235695	-0.337830643	0.460936799	0.797111689	Potri.015G090600.1	AT3G25100.1
Potra2n15c28378.1	0.263190895	0.170076752	0.050322774	0.784407529	Potri.015G090500.1	AT5G50320.1
Potra2n15c28379.1	0.675217375	1.002087278	0.776412162	NA	Potri.015G090400.1	AT5G63290.1
Potra2n15c28380.1					Potri.012G092900.1	AT5G63290.1
Potra2n15c28381.1	-0.228370375	-0.127152944	0.065999259	0.862335622	Potri.015G090300.1	AT5G63280.1
Potra2n15c28382.1	NA	1.381953902	NA	NA	Potri.014G026800.1	AT1G45688.1
Potra2n15c28383.1	NA	1.381953902	NA	NA		
Potra2n15c28384.1					Potri.008G220300.1	
Potra2n15c28385.2	0.018281532	-0.10346596	0.909353922	0.889258723	Potri.015G090200.1	AT5G62640.1
Potra2n15c28386.1	0.54997075	0.168703976	0.000217026	0.788065047	Potri.015G090100.1	AT5G50310.1
Potra2n15c28387.1	3.076036896	0.674963689	NA	NA	Potri.015G090000.1	AT5G50300.1
Potra2n15c28388.1	8.667260966	3.28382	7.95822E-14	NA	Potri.015G089800.1	AT1G24440.1
Potra2n15c28389.1	-1.328951947	-0.068349583	NA	NA	Potri.015G089700.1	AT3G48460.1
Potra2n15c28390.1	0.057568959	-0.01449782	0.741838507	0.989878071	Potri.015G089600.1	AT3G18030.1
Potra2n15c28391.4	0.065773039	0.053181725	0.497262657	0.94728769	Potri.015G089300.1	AT2G36850.1
Potra2n15c28392.1	-1.675062782	-0.605856485	1.1444E-10	0.759797728	Potri.015G089201.1	AT2G04410.1
Potra2n15c28393.1	-0.268845909	-0.006498516	0.007079345	0.996032145	Potri.015G089100.1	AT3G48440.1
Potra2n15c28394.1	-0.873131911	0.19798584	0.489403596	0.974275337		
Potra2n15c28395.1	0.00951453	0.14290372	0.961533984	0.924951347	Potri.015G089000.1	AT3G48430.1
Potra2n15c28396.1	-0.089158058	0.047417551	0.581295067	0.978203092	Potri.015G088900.1	AT3G48425.1
Potra2n15c28397.1	-0.179694649	0.103700499	0.522299735	0.953177702		
Potra2n15c28398.3	-0.058023759	0.018149687	0.774770581	0.992934677	Potri.012G092200.2	AT1G14870.1
Potra2n15c28399.1	0.498796393	0.008622425	0.005289804	0.996185763	Potri.015G088700.1	AT3G23620.1
Potra2n15c28400.1	-0.478626245	-0.014032326	0.072007959	0.994692557	Potri.015G088500.2	AT3G48420.1

Potra2n15c28401.1	-0.328830443	0.14149092	0.703016519	0.981962116	Potri.015G088400.1	AT1G24480.1
Potra2n15c28402.1	0.3804846	0.218843795	0.000106864	0.821709751	Potri.015G088300.1	AT5G63190.2
Potra2n15c28403.1	0.013313718	0.04064737	0.946903025	0.974275337	Potri.015G088200.1	AT3G48380.1
Potra2n15c28404.1	-1.115819298	-0.526967024	0.045774237	0.864223715	Potri.015G088100.3	AT4G24790.2
Potra2n15c28405.1	1.459962178	NA	NA	NA		
Potra2n15c28406.1	-0.616654445	-0.998140339	0.445660395	0.701494362	Potri.015G087900.10	AT4G38040.1
Potra2n15c28407.1	-0.178151574	-0.434663389	0.484496066	0.598566355	Potri.015G087800.1	AT5G63180.1
Potra2n15c28408.1	1.429255566	-0.453316363	0.3205194	0.947969749	Potri.015G087700.1	AT5G63160.1
Potra2n15c28409.1	1.412767451	-0.36557821	0.34297392	0.962696825	Potri.015G087700.1	AT5G63160.1
Potra2n15c28410.1	-0.394036415	-0.164737732	0.01623387	0.941855873	Potri.015G087600.1	AT5G61640.1
Potra2n15c28411.1	-1.386774904	-0.391171923	4.48134E-07	0.82233198	Potri.015G087400.1	AT5G50260.1
Potra2n15c28412.1	-0.039690269	-0.017033274	0.857576477	0.992934677	Potri.015G087301.1	AT1G67785.1
Potra2n15c28413.1	-0.665488536	-0.225499953	3.96719E-08	0.814383461	Potri.015G087200.3	AT5G50230.1
Potra2n15c28414.1	-0.841071288	0.183100452	0.198921284	0.9632996		
Potra2n15c28415.1	-0.280007123	-1.466098182	0.944755644	NA		
Potra2n15c28416.1	-0.859671674	-2.131990325	NA	NA	Potri.015G087000.1	AT2G23180.1
Potra2n15c28417.1	-1.305288983	-1.348311782	0.713395956	NA	Potri.015G087000.1	AT2G23180.1
Potra2n15c28418.1	1.64774763	NA	NA	NA	Potri.015G086900.1	AT2G23180.1
Potra2n15c28419.1	5.283512674	2.715321116	7.8657E-114	0.000155155	Potri.015G086800.1	AT2G31880.1
Potra2n15c28420.1	-0.200572459	-0.069281433	0.044697069	0.922029226	Potri.015G086600.2	AT3G48330.1
Potra2n15c28421.1	-0.352006007	-0.129899017	0.111671151	0.914162682	Potri.015G086500.1	AT4G24770.1
Potra2n15c28422.1	0.871195371	0.037786206	0.001082095	0.985393296	Potri.015G086400.1	AT3G57520.1
Potra2n15c28423.1	0.525021695	0.065153794	0.001620971	0.958226175	Potri.015G086200.1	AT5G14420.1
Potra2n15c28424.1	0.470525344	0.196274754	0.657148416	0.966407607		
Potra2n15c28425.1	-0.407806868	0.056920976	0.122221521	0.977227089	Potri.015G086100.3	AT4G24750.1
Potra2n15c28426.1	0.581728248	0.347942561	0.004062227	0.699507809	Potri.015G086000.1	AT1G13080.1
Potra2n15c28427.1						
Potra2n15c28428.1					Potri.T103000.2	AT4G08850.1
Potra2n15c28429.1						
Potra2n15c28430.1	2.771995342	2.390724774	0.260458229	NA	Potri.002G008500.1	AT3G25560.3
Potra2n15c28431.1	1.636209054	0.704958365	1.09859E-06	0.683816919	Potri.015G085800.1	AT3G48270.1
Potra2n15c28432.1	-0.02903408	0.228677238	0.847505177	0.874808359	Potri.015G085700.1	AT4G24740.1

Potra2n15c28433.3	1.458678931	0.703472571	0.362100147	NA	Potri.015G085500.1	AT5G23960.1
Potra2n15c28434.1	0.23351152	-1.478536539	NA	NA	Potri.015G085600.2	AT3G48280.1
Potra2n15c28435.1					Potri.015G085400.2	AT2G33580.1
Potra2n15c28436.1					Potri.015G085350.1	AT2G23770.1
Potra2n15c28437.1	3.907334348	1.346458582	3.27011E-25	0.521534461	Potri.015G085300.1	AT5G50210.1
Potra2n15c28438.1	-0.068985917	0.377295036	0.944860129	0.917516356		
Potra2n15c28439.1	-0.192144419	-0.174217235	0.849110885	0.972159498	Potri.012G089400.1	AT4G24730.2
Potra2n15c28440.1	0.125771761	-0.014625602	0.79675235	0.99812851	Potri.015G085100.1	AT5G50200.1
Potra2n15c28441.1	0.138932005	0.075855819	0.763151964	0.988601759	Potri.015G085100.1	AT5G50200.1
Potra2n15c28442.1	0.506769071	-0.117000354	0.003795275	0.922029226	Potri.015G084900.1	AT4G24710.1
Potra2n15c28443.1	0.357474287	-0.120854085	0.163035793	0.941260209	Potri.015G084800.1	AT3G48250.1
Potra2n15c28444.1	0.232949184	-0.147535292	0.137223921	0.893394174	Potri.012G086600.2	AT4G29390.1
Potra2n15c28445.1						
Potra2n15c28446.1	-0.318280536	-0.211058358	0.028705644	0.71299041	Potri.015G084600.1	AT3G48260.1
Potra2n15c28447.1	-1.677497556	0.405365407	0.367002618	0.962432149		
Potra2n15c28448.1	-2.555428083	-0.408532216	0.000194347	0.859813626	Potri.015G084300.1	
Potra2n15c28449.1	-2.344928421	-0.380212504	0.007044764	0.899990252	Potri.015G084500.1	AT5G48270.1
Potra2n15c28450.1	-2.329697952	-0.466283094	0.002768663	0.896593025	Potri.015G084400.1	
Potra2n15c28451.1	-2.011109822	-1.352451312	0.119917033	0.704224102	Potri.015G084200.1	AT4G24700.1
Potra2n15c28452.1	0.252693117	0.97034693	0.775402078	0.637188449	Potri.015G084100.1	
Potra2n15c28453.1	-0.071676228	0.057971146	0.676991319	0.962696825	Potri.015G084000.1	AT4G24690.1
Potra2n15c28454.1	-1.875734646	-0.442612803	4.26057E-06	0.79477084	Potri.015G083900.1	AT5G50175.1
Potra2n15c28455.1	-0.115288332	0.282027323	0.41567357	0.770451625	Potri.015G083800.1	AT3G11330.1
Potra2n15c28456.2	0.25297606	0.143370827	0.128722686	0.882699919	Potri.015G083700.1	AT1G23280.1
Potra2n15c28457.1	-1.193302133	0.066936733	0.00026942	0.98036348	Potri.015G083500.2	AT5G50170.1
Potra2n15c28458.1	-0.247283914	0.16758386	0.365119268	0.874648591	Potri.015G083400.1	AT3G48240.1
Potra2n15c28459.1	-0.392060851	-0.035114785	0.004544463	0.976302215	Potri.015G083300.1	AT1G15530.1
Potra2n15c28460.1	-1.376873785	0.250816288	0.000816281	0.917751882	Potri.015G083200.1	AT5G50160.1
Potra2n15c28461.1	1.660654431	0.852072895	6.27232E-07	0.540010302	Potri.015G083100.1	AT5G47740.1
Potra2n15c28462.1	-0.210325904	-0.117066275	0.023895188	0.912777843	Potri.015G083000.1	AT5G63120.2
Potra2n15c28463.1	1.079562543	-0.267438304	5.90523E-05	0.920763566	Potri.015G082900.1	AT2G42490.1
Potra2n15c28464.1	1.136201929	-0.22646283	0.000769816	0.935851032	Potri.015G082900.1	AT2G42490.1

Potra2n15c28465.1	0.271435192	-0.122080545	0.088953044	0.903477395	Potri.015G082800.1	AT3G26600.1
Potra2n15c28466.1	-0.336264355	-0.620228492	0.316181932	0.57046267	Potri.015G082700.1	AT1G57560.1
Potra2n15c28467.1	0.72322425	0.025140455	6.76346E-08	0.985393296	Potri.015G082500.1	AT5G63110.1
Potra2n15c28468.1	-1.052874054	-0.746156151	4.91346E-05	0.149735105	Potri.015G082432.1	AT5G63100.1
Potra2n15c28469.1	-0.139409082	0.143797447	0.822175513	0.956889095	Potri.015G082366.1	AT1G64310.1
Potra2n15c28470.1	0.105575202	0.402121882	0.807554276	0.784407529	Potri.015G082250.1	
Potra2n15c28471.1	0.118791076	-0.486001562	0.883876299	0.852185853	Potri.015G082300.1	AT3G07040.1
Potra2n15c28472.1	-2.177315227	0.149535492	6.15883E-08	0.987288338	Potri.015G082200.1	AT5G63090.1
Potra2n15c28473.1					Potri.015G082100.1	AT3G01570.1
Potra2n15c28474.2	-0.470836572	0.018958993	0.391825549	0.996032145	Potri.015G082000.1	AT1G51940.1
Potra2n15c28475.1	-0.67416835	0.00582278	0.001833632	0.997956347	Potri.015G081900.1	AT4G24670.2
Potra2n15c28476.1	-0.518776841	-0.148222718	1.15431E-07	0.812696706	Potri.015G081800.1	AT5G14540.1
Potra2n15c28477.1	-2.307432468	0.473446601	NA	NA	Potri.015G081700.2	AT5G40390.1
Potra2n15c28478.3	0.128960516	0.078144121	0.582978346	0.936890993	Potri.015G081600.1	
Potra2n15c28479.1	-0.084900449	0.089772655	0.719018355	0.942409925	Potri.015G081300.1	AT1G12940.1
Potra2n15c28480.1	0.591869547	0.050912016	0.046112074	0.981761466	Potri.015G081300.1	AT1G12940.1
Potra2n15c28481.1	-0.001545106	2.241500475	NA	NA	Potri.015G081200.1	
Potra2n15c28482.1	-1.872365494	-0.483172565	0.053080011	0.894019146		
Potra2n15c28483.1	-1.356828526	-0.176066753	7.26147E-06	0.927541745	Potri.015G081000.1	AT5G50130.1
Potra2n15c28484.1	0.800303932	-0.211701572	0.229835832	0.940556596	Potri.015G080900.1	AT3G48210.1
Potra2n15c28485.1	-0.07275594	1.050481497	0.899962902	0.512736781	Potri.015G080800.1	AT5G56040.2
Potra2n15c28486.1	0.155451161	0.772579668	0.830792924	0.831597503		
Potra2n15c28487.1	0.43593412	0.067196963	0.000979839	0.949358779	Potri.015G080600.1	AT5G03340.1
Potra2n15c28488.1	0.047344153	0.150023485	0.80867727	0.831738681	Potri.015G080400.2	AT5G63080.1
Potra2n15c28489.1	0.473155391	-0.319930465	0.23688157	0.901195443	Potri.015G080300.1	AT5G50150.1
Potra2n15c28490.1	0.345132259	-0.547761705	0.421335856	0.838959551		
Potra2n15c28491.1	0.514449611	-0.747641198	0.424446745	0.827781566		
Potra2n15c28492.1	1.168165125	0.442145989	0.019757799	0.900884796		
Potra2n15c28493.1	-0.144473773	-0.668159806	0.840808095	0.778013962	Potri.015G080200.1	AT4G10790.1
Potra2n15c28494.1	0.02915735	-0.090902375	0.878021117	0.921065381	Potri.015G080100.1	AT5G63060.1
Potra2n15c28495.1	0.520154073	0.22945438	0.003025386	0.790362046	Potri.015G080000.1	AT1G17100.1
Potra2n15c28496.1	0.480683231	0.208765593	0.003335191	0.790623525	Potri.015G079900.1	AT4G19180.1

Potra2n15c28497.1	NA	1.394613675	NA	NA		
Potra2n15c28498.1	0.1908768	0.234869795	0.084424521	0.48472253	Potri.015G079800.1	AT4G24680.1
Potra2n15c28499.1	-0.00812667	0.037358527	0.967730952	0.973347974	Potri.015G079700.1	AT5G63050.1
Potra2n15c28500.1	NA	1.363811008	NA	NA		
Potra2n15c28501.1	0.544173964	0.357945764	0.013424661	0.705502567	Potri.015G079600.1	AT5G07090.1
Potra2n15c28502.1	-0.023649397	0.105137703	0.844199566	0.874808359	Potri.015G079500.1	AT2G04940.1
Potra2n15c28503.1	-0.033826464	0.219251603	0.900279381	0.777674368		
Potra2n15c28504.3	-0.121773949	0.075547591	0.465968069	0.922011871	Potri.015G079400.1	AT1G19100.1
Potra2n15c28505.1	-0.24377145	0.157011164	0.170070378	0.849067086	Potri.015G079300.2	AT1G17720.1
Potra2n15c28506.1	4.905706192	NA	0.040050364	NA	Potri.015G079200.1	AT1G19090.1
Potra2n15c28507.1	0.23620215	0.156450984	0.113996113	0.84640933	Potri.015G079100.1	AT1G23380.1
Potra2n15c28508.1	1.077464376	1.655071684	0.456776786	0.72476666		
Potra2n15c28509.1	0.115250668	-0.741439893	0.938859078	NA		
Potra2n15c28510.1	-1.22875193	-0.135562745	0.001078933	0.934548357	Potri.015G079000.1	AT3G48200.1
Potra2n15c28511.1	0.098699369	0.297583645	0.682861697	0.836066102	Potri.015G078900.1	AT5G63030.1
Potra2n15c28512.1	0.164865076	0.312735745	0.489707015	0.833651746	Potri.012G082700.2	AT5G17990.1
Potra2n15c28513.1	-0.921674986	-0.658834983	0.183736615	0.831597503	Potri.015G078700.1	AT5G50290.1
Potra2n15c28514.1	-0.006000797	-0.021622045	0.976628771	0.98177802	Potri.015G078600.1	AT5G63000.1
Potra2n15c28515.1	0.464728928	0.031277569	0.000162357	0.985393296	Potri.015G078500.1	AT5G66540.1
Potra2n15c28516.1	-0.24208252	0.040629756	0.734972634	0.989329144	Potri.015G078400.1	
Potra2n15c28517.1	-0.219644494	-0.025793405	0.010626993	0.978646026	Potri.012G082500.1	AT3G48195.1
Potra2n15c28518.1	-1.64149752	-0.18571233	9.67998E-05	0.935474933	Potri.015G078200.1	AT5G62960.1
Potra2n15c28519.1	0.82357019	NA	NA	NA		
Potra2n15c28520.1	-0.054187092	-0.09537716	0.867369341	0.949847686	Potri.015G078100.2	AT1G27510.1
Potra2n15c28521.1	0.054861978	-0.082447477	0.748551939	0.92812284	Potri.015G078000.1	AT5G09590.1
Potra2n15c28522.1	-0.432596873	-0.290372258	0.061887378	0.859892315	Potri.015G077900.1	AT5G33320.1
Potra2n15c28523.1	-2.085583805	-1.306223242	0.180841579	NA	Potri.015G077800.1	AT4G02235.1
Potra2n15c28524.1	NA	NA	NA	NA	Potri.015G077700.1	AT4G18770.1
Potra2n15c28525.1	-0.753797224	-0.314561885	0.018200878	0.637188449	Potri.015G077600.1	AT3G62270.1
Potra2n15c28526.1	-0.152218585	-0.064826363	0.197391431	0.935851032	Potri.015G077500.1	AT4G22220.1
Potra2n15c28527.1	-0.023375422	0.142119744	0.883876299	0.892756772	Potri.015G077300.3	AT4G32180.1
Potra2n15c28528.1	0.325854867	0.229124938	0.078079358	0.819475509	Potri.015G077200.1	AT3G06040.3

Potra2n15c28529.1	-1.817182327	-0.030794597	0.001330518	0.991407187	Potri.015G077100.1	AT5G62940.1
Potra2n15c28530.1	-1.205323358	-0.093501982	5.56883E-14	0.947969749	Potri.015G076650.1	AT3G48190.1
Potra2n15c28531.1	-1.048555912	-0.358869377	0.006143526	0.456112976	Potri.015G076500.1	AT5G62520.1
Potra2n15c28532.1	-0.861235662	-0.596215011	0.000116681	0.091708693	Potri.015G076400.1	AT1G23710.1
Potra2n15c28533.1	0.462025144	1.029125916	0.751846757	0.870293488	Potri.015G076300.1	
Potra2n15c28534.1	-0.604045883	0.346801458	0.006783007	0.733006577	Potri.015G076200.1	AT5G62790.1
Potra2n15c28535.1	0.117085448	0.106202052	0.247210637	0.922993749	Potri.015G076100.1	AT5G62810.1
Potra2n15c28536.1	3.024908169	-4.071423703	0.069099834	NA	Potri.015G076000.1	AT5G14740.2
Potra2n15c28537.2	-0.979381066	-2.486725844	NA	NA	Potri.015G075800.3	AT5G14750.1
Potra2n15c28538.1	-0.61910233	0.660619236	NA	NA	Potri.015G075600.2	AT5G14750.1
Potra2n15c28539.1	-1.123527882	0.09472744	0.095358435	0.983113084	Potri.015G075400.1	AT2G25355.1
Potra2n15c28540.1	-1.01415189	-0.492987485	0.797785175	NA		
Potra2n15c28541.1	-0.791209204	-0.396029365	6.65345E-07	0.679485917	Potri.015G075300.1	AT1G27500.1
Potra2n15c28542.2	-0.386185128	-0.018467309	0.122868674	0.99193658	Potri.015G075200.1	AT1G62400.1
Potra2n15c28543.1	-0.149673672	0.02880626	0.21099274	0.985393296	Potri.015G075100.1	AT5G62820.1
Potra2n15c28544.1	-0.506274697	0.026844454	1.63973E-05	0.985393296	Potri.015G075000.1	AT1G27510.1
Potra2n15c28545.1	1.664677056	NA	NA	NA		
Potra2n15c28546.1	-0.597953907	0.177089129	0.001738349	0.930871994	Potri.015G074900.1	AT3G47990.1
Potra2n15c28547.1	-0.40196	-0.014684091	0.122197151	0.996032145	Potri.015G074700.1	AT5G62840.1
Potra2n15c28548.1	2.924742346	0.609408818	2.91457E-19	0.523342013	Potri.015G074600.3	AT5G13700.1
Potra2n15c28549.4	2.699330442	0.009320959	0.212190776	0.999458638	Potri.015G074500.1	AT4G20970.1
Potra2n15c28550.1	-0.17317492	-0.084300895	0.394233427	0.950491075	Potri.015G074400.1	AT5G50000.1
Potra2n15c28551.1	2.658032118	2.676364087	NA	NA	Potri.015G074300.1	AT5G62850.1
Potra2n15c28552.1	-0.42482573	0.190995205	0.376494348	0.950095151	Potri.015G074200.1	AT1G60190.1
Potra2n15c28553.1						
Potra2n15c28554.2	-1.278934283	0.614970522	5.06699E-06	0.751093503	Potri.015G074100.1	AT3G48000.1
Potra2n15c28555.1	0.328381405	0.259673976	0.347636135	0.876608977	Potri.015G074000.1	AT1G23870.1
Potra2n15c28556.1	0.154188495	-0.104463918	0.441774845	0.930598202	Potri.012G078400.1	AT1G24450.1
Potra2n15c28557.1	-0.84166117	-0.159767262	0.000125286	0.921063801	Potri.015G073800.2	AT5G62865.1
Potra2n15c28558.1	-0.155413543	0.062122763	0.416066193	0.950478187	Potri.015G073700.1	AT4G11130.1
Potra2n15c28559.1	-0.638032443	-0.302486823	0.015443479	0.843482077	Potri.015G073600.1	AT5G40250.1
Potra2n15c28560.1	1.037795312	0.348061209	6.11475E-08	0.453270097	Potri.015G073400.1	AT1G63800.1

Potra2n15c28561.1	0.29034898	-0.162890888	0.035570738	0.848005416	Potri.015G073300.5	AT3G48030.1
Potra2n15c28562.1	0.125418309	0.055487893	0.154099163	0.968407862	Potri.015G073200.1	AT5G40240.1
Potra2n15c28563.1	-0.18271918	0.089594817	0.095821054	0.921099436	Potri.015G073100.1	AT5G40200.1
Potra2n15c28564.6	-0.469898231	-0.20477144	7.31817E-06	0.707347586	Potri.015G073000.1	AT3G48040.1
Potra2n15c28565.1	-0.048595743	0.042692232	0.678923333	0.955030919	Potri.015G072900.1	AT3G48050.2
Potra2n15c28566.1	-0.327013959	0.042794881	0.001361766	0.957163493	Potri.015G072800.1	AT3G48050.2
Potra2n15c28567.1	-0.06281353	1.000659205	0.978384236	NA		
Potra2n15c28568.1	-0.71318054	-0.164535863	0.004859855	0.885375133	Potri.015G072600.1	AT5G62890.1
Potra2n15c28569.1	-2.436728931	-0.999243766	2.35115E-08	0.466184064	Potri.012G077300.1	AT5G50090.1
Potra2n15c28570.1	-0.035647779	-0.253803577	0.896303838	0.786910796	Potri.015G072400.1	AT1G10200.1
Potra2n15c28571.4	0.033365341	0.002279655	0.804157521	0.99905083	Potri.015G072300.1	
Potra2n15c28572.1	0.036542835	0.000740292	0.786326071	0.999536469	Potri.015G072100.1	AT5G62575.2
Potra2n15c28573.1	1.009614593	0.22397409	6.73904E-06	0.759941508	Potri.015G072000.2	AT5G50100.1
Potra2n15c28574.1	1.542870419	0.380546186	2.28143E-10	0.590905253	Potri.015G071900.1	AT1G24100.1
Potra2n15c28575.1	0.430419567	0.255219541	0.03953234	0.812920176	Potri.015G071800.1	AT5G62570.2
Potra2n15c28576.1	0.569911355	-0.063447181	0.007223494	0.961820855	Potri.015G071700.1	AT5G35530.1
Potra2n15c28577.1	-1.004134982	-0.816329971	0.069445936	0.736000077	Potri.015G071500.1	AT5G14920.1
Potra2n15c28578.4	-0.075887859	-0.004807787	0.65320222	0.996032145	Potri.015G071400.1	AT1G27520.1
Potra2n15c28579.1	-0.681904549	-0.148469566	0.024578512	0.965249957	Potri.015G071300.1	AT3G47830.1
Potra2n15c28580.1	-0.265868	-0.221608697	0.061520856	0.772458566	Potri.015G071200.1	AT5G65200.1
Potra2n15c28581.1	0.416939787	0.021823505	0.003175002	0.985393296		
Potra2n15c28582.1	1.64774763	NA	NA	NA	Potri.012G076100.1	AT5G50110.1
Potra2n15c28583.1	-1.317292482	-1.465929376	NA	NA	Potri.012G076100.1	AT5G50110.1
Potra2n15c28584.1	0.226984943	0.054712199	0.56234177	0.985393296	Potri.015G070900.1	AT5G50120.1
Potra2n15c28585.1					Potri.001G236600.1	AT5G05800.2
Potra2n15c28586.1	NA	1.363811008	NA	NA	Potri.002G213700.1	
Potra2n15c28587.1	0.998896507	0.418716909	0.239114415	0.935474933	Potri.015G070700.1	AT1G19020.1
Potra2n15c28588.1	-0.203351596	-0.004715092	0.024413908	0.995579852	Potri.015G070600.2	AT1G74920.1
Potra2n15c28589.1	0.121062178	0.339143037	0.429636039	0.57046267	Potri.015G070500.1	AT1G74910.1
Potra2n15c28590.1	0.634342447	-0.275045237	0.135399019	0.93877195	Potri.015G070300.2	AT3G48160.2
Potra2n15c28591.1	-0.150293841	0.151005507	0.730669787	0.948577865		
Potra2n15c28592.1	0.267676047	0.083200046	0.453620188	0.965595755	Potri.015G070200.1	AT1G74900.1

Potra2n15c28593.1	-0.032078463	0.098670247	0.819010841	0.935474933	Potri.015G070100.1	AT3G48120.1
Potra2n15c28594.1	-0.012252207	0.484176907	0.980326987	0.814981587	Potri.015G070000.1	AT3G48100.1
Potra2n15c28595.1	-0.158891922	0.174760057	0.846655219	0.953608209	Potri.015G069800.2	AT1G70140.1
Potra2n15c28596.1	-0.158901669	0.075624537	0.853184672	0.983113084	Potri.015G069900.1	
Potra2n15c28597.1	-0.04159146	-0.112715718	0.805788717	0.923397137	Potri.015G069700.1	AT1G62020.1
Potra2n15c28598.1	6.475991263	2.848607213	8.48311E-91	0.017390344	Potri.015G069600.1	AT3G48090.1
Potra2n15c28599.1	4.117353689	NA	0.138448551	NA		
Potra2n15c28600.1	0.029865195	-0.111172957	0.895670753	0.903934429	Potri.015G069400.1	AT5G62910.1
Potra2n15c28601.1	1.545832463	1.381953902	NA	NA		
Potra2n15c28602.1	NA	0.674963689	NA	NA	Potri.T006500.1	AT2G01050.1
Potra2n15c28603.1	-0.61910233	-0.046370319	NA	NA	Potri.005G146200.1	AT2G01050.1
Potra2n15c28604.3	-0.556819361	-0.283003487	0.000285894	0.791848264	Potri.015G069200.2	AT5G62580.1
Potra2n15c28605.2	-0.235485004	0.0091905	0.18519867	0.993589787	Potri.015G069100.1	AT1G74850.1
Potra2n15c28606.1	1.073733986	1.793854103	NA	NA		
Potra2n15c28607.1	-0.161353084	0.127177747	0.397004601	0.912777843	Potri.015G069000.1	AT1G19000.1
Potra2n15c28608.1	2.383351842	0.83221427	0.271703321	NA	Potri.015G068800.1	
Potra2n15c28609.1	-0.61910233	0.64247635	NA	NA	Potri.015G068700.1	AT3G08720.2
Potra2n15c28610.1	-0.030421494	0.044095475	0.796981798	0.951394085	Potri.015G068600.1	AT5G62600.1
Potra2n15c28611.1	-1.354083966	0.44685105	0.554520689	NA		
Potra2n15c28612.1	NA	NA	NA	NA	Potri.015G068400.1	AT1G18990.1
Potra2n15c28613.1	0.120260993	0.0087788	0.229272717	0.992934677	Potri.015G068300.1	AT1G10430.1
Potra2n15c28614.1	1.459962178	NA	NA	NA		
Potra2n15c28615.2	0.920129192	-0.072965238	0.019499684	0.979719961	Potri.015G068200.1	AT1G18980.1
Potra2n15c28616.1	0.03864388	0.108065854	0.872777315	0.918871056	Potri.015G068100.1	AT5G62610.1
Potra2n15c28617.1	0.144434731	-0.038234726	0.741644292	0.99114586		
Potra2n15c28618.1	-0.019892335	-0.13890286	0.942587173	0.919424623	Potri.015G067800.1	AT5G20110.1
Potra2n15c28619.1	-3.336863947	-3.486535826	NA	NA	Potri.015G067700.1	AT3G28470.1
Potra2n15c28620.1	-1.021447256	0.378164535	0.011407162	0.884185938		
Potra2n15c28621.2	-0.304269277	-0.012735529	0.024556164	0.992934677	Potri.015G067600.1	AT5G62620.1
Potra2n15c28622.1						
Potra2n15c28623.1	-3.91159001	0.181992151	0.010943053	0.978251387		
Potra2n15c28624.1	-2.27347486	-0.17408939	4.26603E-05	0.951726178	Potri.015G067500.1	AT3G10330.1

Potra2n15c28625.1	0.111284785	0.08834133	0.353599204	0.888096054	Potri.015G067300.2	AT1G18950.1
Potra2n15c28626.1	0.183784577	0.082619608	0.635262769	0.972159498	Potri.015G067400.1	AT5G40010.1
Potra2n15c28627.1	0.5168101	-0.341693344	0.222376488	0.866976165	Potri.015G067200.1	AT1G74790.1
Potra2n15c28628.1	2.409567423	NA	NA	NA		
Potra2n15c28629.1					Potri.018G062700.2	AT1G19170.1
Potra2n15c28630.1	-0.808155162	-0.264869834	1.28576E-06	0.759807319	Potri.015G067000.6	AT1G74780.1
Potra2n15c28631.1	-0.381510987	0.065389822	0.005171797	0.948002367	Potri.015G066600.1	AT1G74770.1
Potra2n15c28632.1	-0.055728664	1.208658643	0.93983846	0.276027277	Potri.015G066700.1	AT3G47870.1
Potra2n15c28633.1	0.195602118	0.187131722	0.156707318	0.816392132	Potri.015G066500.1	AT3G47890.1
Potra2n15c28634.1	0.173238396	0.010392732	0.200642129	0.994486196	Potri.015G066400.4	AT1G18900.2
Potra2n15c28635.2	2.685015931	NA	NA	NA	Potri.006G172300.1	
Potra2n15c28636.1	0.202935945	-0.159884716	0.199174918	0.894138111	Potri.015G066200.2	AT1G74740.1
Potra2n15c28637.1	0.066399724	0.113930114	0.721035467	0.886511888	Potri.015G066100.1	AT3G47940.1
Potra2n15c28638.1	-0.320434858	0.156734332	0.028735029	0.817452127	Potri.015G066000.2	AT5G62670.1
Potra2n15c28639.1	-0.16172286	0.072021784	0.349733636	0.953831244	Potri.015G065900.1	AT5G55220.1
Potra2n15c28640.2	0.103674043	0.057819721	0.631194504	0.966917036	Potri.015G065800.1	AT4G26780.1
Potra2n15c28641.1	NA	1.381953902	NA	NA	Potri.015G065700.1	AT2G47140.1
Potra2n15c28642.1	-2.367361922	-1.072293905	NA	NA	Potri.015G065600.1	AT2G47140.1
Potra2n15c28643.1	-1.613158729	0.171730105	6.22869E-10	0.948577865	Potri.015G065500.1	AT5G62720.1
Potra2n15c28644.1	-0.550127983	-0.040953103	0.001916815	0.975887373	Potri.012G070900.1	AT3G01470.1
Potra2n15c28645.1	-0.708665232	-0.084608036	0.009519988	0.97064682		
Potra2n15c28646.1						
Potra2n15c28647.1	NA	NA	NA	NA	Potri.015G064800.1	AT2G27140.1
Potra2n15c28648.1	0.050923025	0.18529049	0.743838896	0.848005416	Potri.015G064600.2	AT5G62760.3
Potra2n15c28649.1	-0.055849876	-0.00714115	0.776940292	0.996032145	Potri.015G064400.1	AT1G74700.1
Potra2n15c28650.1	0.569504303	0.058819983	0.00034264	0.978646026	Potri.015G064300.1	AT1G18850.1
Potra2n15c28651.1	-0.119071536	-0.000426315	0.424137674	0.99984869	Potri.015G064200.2	AT5G62530.1
Potra2n15c28652.1	-1.409983009	-1.55593228	NA	NA	Potri.001G184450.1	
Potra2n15c28653.1	1.738031013	-0.451643077	0.535443101	NA	Potri.015G064100.1	AT5G15130.1
Potra2n15c28654.1	-0.007185187	0.131635959	0.952869364	0.885628484	Potri.015G064000.2	AT2G02760.1
Potra2n15c28655.1						
Potra2n15c28656.1	-0.065934732	-0.747367733	0.90253824	0.514425946	Potri.015G063700.1	AT5G62550.1

Potra2n15c28657.1	-0.581971429	-0.145272682	6.41793E-06	0.869186333	Potri.015G063600.1	AT1G74690.1
Potra2n15c28658.1	-0.875097196	-0.192123826	5.23185E-06	0.885375133	Potri.015G063500.1	AT3G47730.1
Potra2n15c28659.1	-0.63225362	0.07456581	0.4914008	0.989304496	Potri.012G069700.2	AT3G47780.1
Potra2n15c28660.1					Potri.015G063300.1	AT3G47710.1
Potra2n15c28661.2	-0.497585369	-0.085856659	0.017999279	0.953108412	Potri.015G063200.10	AT1G74510.1
Potra2n15c28662.1	-0.090674391	0.055360183	0.36730591	0.953831244	Potri.015G063100.1	AT3G47700.1
Potra2n15c28663.1	-0.923535696	-0.236909325	2.21391E-06	0.750123244	Potri.015G062900.1	AT5G62500.1
Potra2n15c28664.1	-0.536987099	-0.398960561	0.00265176	0.602611218	Potri.015G062800.2	AT1G74520.1
Potra2n15c28665.1	0.066359957	-0.040985759	0.719888837	0.976302215	Potri.015G062700.2	AT1G18800.1
Potra2n15c28666.1	1.618206915	2.617732019	0.543283426	NA	Potri.015G062600.1	AT2G02950.1
Potra2n15c28667.1	-0.002592545	0.069419201	0.996119966	0.979755286	Potri.012G068800.1	AT1G74470.1
Potra2n15c28668.1	0.363199714	0.102133765	0.007492558	0.885375133	Potri.015G062400.1	AT1G66680.1
Potra2n15c28669.1	-0.516684141	-0.234524277	0.019773494	0.820880672	Potri.015G062300.1	AT3G47510.1
Potra2n15c28670.1	-0.639195264	-0.459558344	0.002082816	0.148037164	Potri.015G062200.1	AT3G47470.1
Potra2n15c28671.1	-0.345121571	-0.173055934	0.000423531	0.734313982	Potri.015G062100.3	AT5G61410.1
Potra2n15c28672.1	0.280806619	0.158564569	0.02020297	0.723580731	Potri.015G062000.1	AT5G61390.1
Potra2n15c28673.1	-0.197241651	0.017978434	0.122242962	0.986671181	Potri.015G061900.5	AT5G61380.1
Potra2n15c28674.1	0.340985855	-0.001223611	0.066097498	0.999458638	Potri.015G061800.1	AT5G07720.1
Potra2n15c28675.1	NA	0.674963689	NA	NA	Potri.015G061700.1	AT5G61350.1
Potra2n15c28676.1	2.309661873	-0.037236492	2.40744E-22	0.99114586	Potri.015G061600.1	AT1G74360.1
Potra2n15c28677.1	1.459962178	NA	NA	NA	Potri.015G061500.1	AT2G26570.1
Potra2n15c28678.3	0.411451494	0.215342004	0.005625356	0.827673117	Potri.015G061400.1	AT1G18670.1
Potra2n15c28679.1	-0.61910233	-0.767708831	NA	NA	Potri.015G061300.1	ATMG00090.1
Potra2n15c28680.1	-0.117181003	0.074329776	0.43867402	0.915115814	Potri.012G067900.1	AT5G07740.1
Potra2n15c28681.2	-0.091685276	0.253219352	0.753631575	0.818161931		
Potra2n15c28682.1	-0.061938539	0.144842533	0.80258482	0.91233528		
Potra2n15c28683.1	-0.863907097	-0.174400799	0.001366694	0.936890993	Potri.015G060900.1	AT5G61340.1
Potra2n15c28684.1	0.849853773	0.005069244	2.70681E-06	0.996851113	Potri.015G060800.1	AT1G18580.1
Potra2n15c28685.1	6.838590784	5.619441723	5.45915E-08	NA	Potri.015G060700.3	AT5G17390.1
Potra2n15c28686.1	-0.189833731	1.125477277	0.940261127	0.835627384	Potri.015G060600.1	AT2G36640.1
Potra2n15c28687.1	1.860278558	1.203694519	1.20398E-08	0.218315345	Potri.015G060400.1	AT1G69170.1
Potra2n15c28688.2	-0.024689466	1.054468392	0.972502471	0.700697262	Potri.015G060300.1	AT3G08900.1

Potra2n15c28689.1	-0.606826437	-0.425808961	0.00015067	0.759932222	Potri.015G060200.1	AT5G15630.1
Potra2n15c28690.1	-0.445707997	-0.405962739	0.001887715	0.726820815	Potri.015G060000.1	AT5G60920.1
Potra2n15c28691.1	5.090527967	1.055326005	2.54409E-41	0.667215387	Potri.015G059600.1	AT5G39040.1
Potra2n15c28692.1	-0.666761459	-0.087033004	6.48527E-05	0.949312638	Potri.015G059400.1	AT1G18610.1
Potra2n15c28693.1	-0.333908055	-0.209602116	0.008754809	0.605100478	Potri.015G059100.1	AT1G26355.1
Potra2n15c28694.1	0.111206308	-0.00351528	0.42493086	0.997821888	Potri.015G059000.2	AT4G26190.1
Potra2n15c28695.1	-0.214073072	0.086538484	0.423741654	0.931774514	Potri.015G058900.1	AT1G74160.1
Potra2n15c28696.1	NA	0.674963689	NA	NA	Potri.015G058800.1	AT5G60970.1
Potra2n15c28697.1	-4.52107582	-0.726203652	0.060495396	NA	Potri.006G106200.1	
Potra2n15c28698.1	0.010581209	-0.009641626	0.942734846	0.991204249	Potri.015G058700.1	AT5G60980.1
Potra2n15c28699.1	-0.285226927	-0.075235473	0.018516678	0.935099709	Potri.015G058600.2	AT5G08050.1
Potra2n15c28700.1	0.074753021	-0.214104954	0.719512445	0.907032966	Potri.015G058400.1	AT1G74210.1
Potra2n15c28701.2	-0.21825935	-0.119406487	0.210501285	0.909675463	Potri.015G058301.1	AT2G29900.1
Potra2n15c28702.1	0.120823232	-0.039175004	0.470014863	0.974275337	Potri.015G058200.1	AT5G61010.2
Potra2n15c28703.3	-0.590898478	-0.489164752	0.031517881	0.672348168	Potri.015G057800.1	AT1G18650.1
Potra2n15c28704.1	0.123433911	-0.06482141	0.420082997	0.953831244	Potri.015G057700.2	AT2G33040.1
Potra2n15c28705.2	-0.166911931	0.03633582	0.259792869	0.962661188	Potri.015G057600.1	AT5G61040.1
Potra2n15c28706.2	0.225673537	0.002553058	0.1678962	0.999121696	Potri.015G057400.1	AT5G61030.1
Potra2n15c28707.1	0.135027894	-0.629473793	0.834920403	0.696743275	Potri.015G057300.1	AT5G08020.1
Potra2n15c28708.1	1.664677056	NA	NA	NA	Potri.015G057200.1	AT1G74220.1
Potra2n15c28709.3	-0.042914078	0.009969624	0.742639004	0.99114586	Potri.015G057100.4	AT5G20170.1
Potra2n15c28710.2	-0.586372166	0.402056293	0.037286995	0.783936798	Potri.015G056900.1	AT1G74310.1
Potra2n15c28711.1	0.036951694	-0.13293855	NA	NA	Potri.015G056800.1	AT5G05810.1
Potra2n15c28712.1	0.507236801	0.257059243	5.41346E-09	0.517743314	Potri.015G056501.1	AT5G61140.2
Potra2n15c28713.1	0.145781515	0.186800721	0.23148781	0.727843918	Potri.015G056400.1	AT5G07940.1
Potra2n15c28714.1	-0.185284741	-0.040575491	0.254260849	0.968637287	Potri.015G056300.1	AT5G07960.1
Potra2n15c28715.1	-0.102954512	0.129143875	0.348548257	0.834560473	Potri.015G056200.1	AT5G07980.1
Potra2n15c28716.1	0.345347112	-0.031414877	0.109905189	0.986190118	Potri.015G056100.1	AT5G61170.1
Potra2n15c28717.1	-0.340101211	0.106070981	0.053616838	0.921099436	Potri.015G056000.1	AT5G23050.1
Potra2n15c28718.1	-0.139938738	1.028954837	0.950127264	NA	Potri.015G055700.2	AT1G18520.1
Potra2n15c28719.1	-0.018449218	0.045293277	0.940160916	0.971828097	Potri.015G055600.1	AT1G18490.1
Potra2n15c28720.1	-0.243547557	0.006043889	0.014926882	0.995565508	Potri.015G055500.5	AT5G35520.1

Potra2n15c28721.2	0.17760788	0.063828594	0.302380489	0.905145891	Potri.015G055400.3	AT1G73980.1
Potra2n15c28722.1	0.17507594	0.141483596	0.230144193	0.875292974	Potri.015G055300.1	AT5G08170.1
Potra2n15c28723.1	0.074323751	-0.000800019	0.687399284	0.999536469	Potri.015G055200.1	AT5G08160.1
Potra2n15c28724.1	1.40406693	0.541179883	8.35517E-14	0.43680237	Potri.015G055000.2	AT1G13580.3
Potra2n15c28725.1	0.82357019	NA	NA	NA	Potri.015G054900.1	AT5G38790.1
Potra2n15c28726.1	-0.116970681	-0.118007581	0.488482865	0.900869139	Potri.015G054700.1	AT1G49140.1
Potra2n15c28727.1	-0.191135983	0.767423154	0.927898715	NA	Potri.015G054600.1	AT1G25440.1
Potra2n15c28728.1	0.594766141	-0.083571602	0.015495131	0.963557834	Potri.015G054500.1	AT1G68550.2
Potra2n15c28729.1	-0.367217334	-0.248576796	0.071018004	0.754074409	Potri.T172014.1	
Potra2n15c28730.1	1.664677056	1.571529889	NA	NA	Potri.018G060400.1	
Potra2n15c28731.1	-2.886082568	-0.514182216	NA	NA	Potri.018G060400.1	
Potra2n15c28732.1	NA	NA	NA	NA	Potri.014G188600.1	AT2G01050.1
Potra2n15c28733.1	-1.140377071	-0.764643206	6.89745E-11	0.022481584	Potri.015G054300.1	AT1G13360.1
Potra2n15c28734.1	1.545832463	1.571529889	NA	NA		
Potra2n15c28735.1	-0.598066588	-0.161118066	0.000223567	0.919321122	Potri.015G054100.1	AT1G73885.1
Potra2n15c28736.1	0.78805245	-0.063695834	0.478338035	0.995581218	Potri.015G053700.1	AT1G73890.1
Potra2n15c28737.1	-0.71139634	-0.148658982	2.23334E-05	0.872421255	Potri.015G054100.1	AT1G73885.1
Potra2n15c28738.1	2.040671178	1.746113549	0.037624552	0.716682524	Potri.015G053700.1	AT1G73890.1
Potra2n15c28739.1	-0.61113141	-0.211259109	0.008278726	0.753052725	Potri.015G054100.1	AT1G73885.1
Potra2n15c28740.1	0.946790661	0.127767046	0.134178099	0.987226805	Potri.015G053700.1	AT1G73890.1
Potra2n15c28741.3	-0.153696136	-0.051565116	0.321636285	0.972159498	Potri.015G053400.2	AT3G18440.1
Potra2n15c28742.1	0.709507685	0.132914037	1.03954E-08	0.865591169	Potri.015G053300.2	AT1G49050.1
Potra2n15c28743.1	-0.03531679	0.140788207	0.794969841	0.792864576	Potri.015G053100.1	AT1G18450.1
Potra2n15c28744.1	-0.093620782	-0.069266731	0.523087078	0.917751882	Potri.015G052800.1	AT5G23090.2
Potra2n15c28745.1	2.137245877	-1.55593228	0.435213943	NA	Potri.T055866.1	
Potra2n15c28746.1	0.133477687	0.746251294	0.846551662	0.799049555		
Potra2n15c28747.1	0.08583482	0.80558275	0.909353922	0.786730624		
Potra2n15c28748.1	0.380840683	0.170201446	0.001664966	0.723580731	Potri.015G052400.3	AT1G48950.1
Potra2n15c28749.1	0.40247136	0.088718672	0.035227504	0.935851032	Potri.015G052500.1	AT1G74070.1
Potra2n15c28750.1	0.328733738	0.233829066	0.017557179	0.679485917	Potri.015G051000.1	AT1G03280.1
Potra2n15c28751.1	0.193283823	0.159029182	0.050099453	0.795613687	Potri.015G050800.1	AT1G73960.1
Potra2n15c28752.1	-2.177901178	-0.165996816	0.02835077	0.983008617	Potri.015G050500.1	AT3G18550.2

Potra2n15c28753.1						
Potra2n15c28754.1	-0.214569911	0.119541675	0.177843362	0.921099436	Potri.015G050300.1	AT1G18470.1
Potra2n15c28755.1	-0.002632462	0.096692808	0.991867476	0.948002367	Potri.015G050200.1	AT1G75290.1
Potra2n15c28756.1	-0.1954917	0.031508661	0.165766357	0.971537796	Potri.015G049900.1	AT1G73930.2
Potra2n15c28757.1	0.570060195	0.054795406	0.588387368	0.994329048	Potri.004G102950.1	AT1G67265.1
Potra2n15c28758.3	-0.12494649	-0.032369596	0.667620413	0.987226805	Potri.015G049800.1	AT1G61415.1
Potra2n15c28759.1	-1.448427991	-1.295194177	0.401987369	NA	Potri.015G049700.1	AT5G61280.1
Potra2n15c28760.1	1.64774763	1.394613675	NA	NA	Potri.015G049600.1	AT5G15430.1
Potra2n15c28761.1	0.037700008	0.005560745	0.819064245	0.996032145	Potri.015G049500.1	AT1G49040.1
Potra2n15c28762.1	-0.210245841	-0.040975513	0.114932226	0.977559521	Potri.015G049100.1	AT5G07830.1
Potra2n15c28763.1	0.623158722	0.087660139	0.000166004	0.94169165	Potri.015G049000.1	AT5G61210.1
Potra2n15c28764.1	-0.248227787	-0.085399976	0.109566942	0.935474933	Potri.015G048900.4	AT5G07890.1
Potra2n15c28765.1	0.205393818	0.217714153	0.148221087	0.702919534	Potri.015G048800.3	AT5G07910.1
Potra2n15c28766.1	1.783513238	0.558054417	4.72133E-15	0.554040943	Potri.015G048700.2	AT2G03800.1
Potra2n15c28767.1	-3.318226699	0.16492095	0.007072776	0.982221696	Potri.015G048600.1	AT5G60880.1
Potra2n15c28768.1	-2.622617359	-0.6095706	NA	NA	Potri.015G048500.1	AT5G51280.1
Potra2n15c28769.1	0.131645407	1.938891026	0.969250175	NA	Potri.015G048400.2	AT2G03810.2
Potra2n15c28770.1					Potri.014G188700.1	AT2G01050.1
Potra2n15c28771.1					Potri.014G188700.1	AT2G01050.1
Potra2n15c28772.1	-1.41089906	0.040886142	2.44848E-08	0.983446961	Potri.015G048300.1	AT5G60850.1
Potra2n15c28773.1	-0.037326836	0.13475967	0.767089435	0.902847514	Potri.015G048200.4	AT1G65660.1
Potra2n15c28774.1	-0.315720467	-0.220239049	0.029108893	0.865611082	Potri.015G048100.6	AT5G15740.1
Potra2n15c28775.5	-0.321200954	-0.223485519	0.125173585	0.79819686	Potri.015G048000.1	AT5G08130.4
Potra2n15c28776.1	-0.172303303	-0.104648221	0.189474949	0.863705586	Potri.012G065300.1	AT5G08139.1
Potra2n15c28777.3	0.639788542	0.006547157	0.865800747	NA	Potri.012G065200.1	AT3G42660.1
Potra2n15c28778.1					Potri.015G047700.1	AT5G60830.1
Potra2n15c28779.1	0.273883831	-0.270378569	0.338514978	0.862924935	Potri.015G047600.1	AT1G18485.1
Potra2n15c28780.1	0.286903387	0.296562008	0.002792124	0.639292153	Potri.015G047500.1	AT1G73990.1
Potra2n15c28781.1	1.826713774	0.660619236	NA	NA	Potri.015G047400.2	AT1G63640.1
Potra2n15c28782.1	0.203702391	0.876491787	0.590011788	0.774693915	Potri.015G047100.1	AT1G68490.1
Potra2n15c28783.1	1.274482656	1.421712548	NA	NA	Potri.015G046900.3	AT1G73850.1
Potra2n15c28784.1	2.891168198	-0.538369001	0.010161059	NA	Potri.015G046800.1	AT3G18400.1

Potra2n15c28785.1	0.659475168	0.492152792	0.000114588	0.287528006	Potri.015G046700.1	AT3G18370.1
Potra2n15c28786.1	NA	0.674963689	NA	NA	Potri.015G046600.1	AT3G18360.1
Potra2n15c28787.1	-0.037826792	0.061149188	0.756263726	0.930059926	Potri.015G046500.10	AT1G48840.1
Potra2n15c28788.1	-2.619478154	-0.590811439	0.003391915	0.930267575	Potri.015G046300.1	AT1G73830.1
Potra2n15c28789.1	8.848465242	4.231951561	2.58471E-26	0.033833871	Potri.015G046200.1	AT1G25340.1
Potra2n15c28790.1	1.097947092	0.279413819	0.015609807	0.935474933	Potri.015G046100.1	AT1G48830.1
Potra2n15c28791.2	1.042407509	0.338314382	2.71643E-11	0.663950227	Potri.015G045900.2	AT1G67060.1
Potra2n15c28792.3	0.188994397	0.129152841	0.164285163	0.896353214	Potri.015G045800.1	AT1G48790.1
Potra2n15c28793.1	-0.686790073	-0.313598744	0.006844715	0.763987753	Potri.015G045700.1	AT1G48780.1
Potra2n15c28794.1	-0.126110294	-0.087237542	0.504840122	0.929826942	Potri.015G045600.1	AT1G48760.2
Potra2n15c28795.1	6.298505732	2.294548607	9.09643E-18	NA	Potri.015G045500.2	AT5G16170.1
Potra2n15c28796.1	2.966442973	1.363811008	NA	NA	Potri.015G045400.1	AT1G68390.1
Potra2n15c28797.1	8.042507661	5.157066805	1.4456E-115	0.001987702	Potri.015G045300.1	AT1G73805.1
Potra2n15c28798.1	3.889110355	1.529473382	0.000333152	NA	Potri.015G045101.1	AT1G18390.2
Potra2n15c28799.1	0.997494848	0.252679449	3.28194E-06	0.791589438	Potri.015G045101.1	AT1G18390.2
Potra2n15c28800.1	1.459962178	1.363811008	NA	NA	Potri.015G044750.1	AT1G18390.2
Potra2n15c28801.8	-0.460009905	-0.340579231	0.058655845	0.846926485	Potri.T113300.1	AT1G18370.1
Potra2n15c28802.1	-0.478312433	-0.207874791	0.134955651	0.93154079	Potri.015G044500.1	AT3G18280.1
Potra2n15c28803.1	-0.01793743	-0.009944711	0.978751757	0.99819408	Potri.015G044401.1	AT3G18270.1
Potra2n15c28804.1	0.115049899	-0.928787504	0.957532384	NA	Potri.015G044300.1	AT3G18260.1
Potra2n15c28805.1	-0.278184647	-0.165133714	0.807443453	0.978251387	Potri.015G044201.1	AT1G48745.1
Potra2n15c28806.1	0.084081923	-0.067883131	0.685998731	0.961152007	Potri.015G044100.1	AT3G18240.2
Potra2n15c28807.1	-0.660159175	-0.459578168	0.010406714	0.669027738	Potri.015G043900.1	AT1G73760.1
Potra2n15c28808.1	-1.409983009	-1.55593228	NA	NA		
Potra2n15c28809.1	-0.237861454	0.570829091	0.900466547	0.923397137	Potri.012G053700.1	AT1G15060.1
Potra2n15c28810.1	0.121842454	0.220115601	0.306955276	0.409505482	Potri.015G043600.2	AT1G27170.2
Potra2n15c28811.1	6.78172194	0.958559235	3.70244E-10	NA	Potri.015G043400.1	AT4G38460.1
Potra2n15c28812.1	-1.124484593	-0.352649547	0.265633955	0.944323367	Potri.018G096100.1	AT5G57670.2
Potra2n15c28813.1						
Potra2n15c28814.1	NA	1.363811008	NA	NA	Potri.001G404600.1	AT1G52870.2
Potra2n15c28815.1	-0.054550975	0.020210772	0.684709413	0.98036348	Potri.015G043200.2	AT1G17980.1
Potra2n15c28816.1	-0.230729914	0.100298169	0.088747018	0.895474078	Potri.015G043100.2	AT3G18210.2

Potra2n15c28817.2	-0.166554527	0.049701877	0.310969779	0.966148753	Potri.015G043000.1	AT1G18030.1
Potra2n15c28818.1	-2.563772154	0.08439449	0.003009897	0.984292161	Potri.015G042900.1	AT3G18200.1
Potra2n15c28819.2	0.001606784	-0.017228817	0.993518164	0.991489035	Potri.015G042800.4	AT2G22660.2
Potra2n15c28820.1	0.020799299	0.052900142	0.920297636	0.974124362		
Potra2n15c28821.1	0.005955268	-0.040900294	0.974164289	0.976302215	Potri.015G042600.1	AT3G18190.1
Potra2n15c28822.1	-0.066227117	0.038477116	0.602395025	0.974367223	Potri.015G042500.1	AT1G48650.1
Potra2n15c28823.1	0.010599468	-0.03052284	0.951196892	0.978646026	Potri.015G042600.1	AT3G18190.1
Potra2n15c28824.1	0.281316741	-0.574097489	0.674573713	0.788065047	Potri.015G042400.1	
Potra2n15c28825.1	1.570112158	0.226545159	0.123192933	0.979273211	Potri.015G042300.1	AT3G18170.1
Potra2n15c28826.1	0.265288564	0.126185707	0.397004601	0.953831244	Potri.015G042100.1	AT1G04420.1
Potra2n15c28827.1	5.703127806	2.536805381	1.33216E-25	0.372755083	Potri.015G042000.1	AT2G29460.1
Potra2n15c28828.1	2.549191961	NA	NA	NA		
Potra2n15c28829.1	2.549642006	0.031044305	0.000850065	0.996032145	Potri.015G041900.1	AT1G15125.1
Potra2n15c28830.1	0.069060892	-0.089830665	0.60733085	0.912665563	Potri.015G041800.1	AT3G18165.1
Potra2n15c28831.1	0.130741393	0.000737396	0.240580765	0.999536469	Potri.015G041700.1	AT1G48635.1
Potra2n15c28832.1	0.843840334	0.247771364	1.00239E-07	0.817452127	Potri.015G041600.1	AT1G18080.1
Potra2n15c28833.1	1.664677056	0.674963689	NA	NA		
Potra2n15c28834.6	0.044215986	0.12864329	0.79537746	0.862097315	Potri.015G041500.1	AT3G18100.1
Potra2n15c28835.1	0.803038342	0.10921348	6.60795E-08	0.897285619	Potri.015G041400.1	AT1G18090.1
Potra2n15c28836.1	0.949068437	0.269998167	5.99473E-20	0.205878227	Potri.015G041300.2	AT3G18080.1
Potra2n15c28837.1	-0.360265308	-0.107058648	0.077033679	0.924951347	Potri.015G041200.1	AT3G18060.1
Potra2n15c28838.1	6.432447742	3.390498064	2.19937E-06	NA	Potri.015G041100.1	AT1G22640.1
Potra2n15c28839.1	-1.409983009	-1.55593228	NA	NA	Potri.015G041000.1	AT1G18100.1
Potra2n15c28840.1	-1.067054491	-0.463918711	0.001606241	0.676711874	Potri.015G040900.1	AT3G18050.1
Potra2n15c28841.1	-0.888661587	-0.295528268	0.051163084	0.902857376		
Potra2n15c28842.1	4.703085593	2.283425176	0.05344112	NA	Potri.015G040400.1	AT1G18140.1
Potra2n15c28843.1	0.088613392	-0.055199905	0.488233417	0.96313916	Potri.015G040300.2	AT1G73670.1
Potra2n15c28844.1	-0.313556092	-0.132921787	0.017445457	0.821709751	Potri.015G040200.1	AT1G48620.1
Potra2n15c28845.1	1.390545487	0.933689949	0.034909887	0.724604666		
Potra2n15c28846.1	0.076895191	0.081119042	0.927937907	0.987226805	Potri.015G040150.1	AT2G01050.1
Potra2n15c28847.1						
Potra2n15c28848.4	-0.211502669	0.012721928	0.078680095	0.994403137	Potri.015G040100.2	AT1G73660.1

Potra2n15c28849.1	-0.091542643	0.03535031	0.570217536	0.974367223	Potri.015G039900.1	AT1G18180.1
Potra2n15c28850.1	-0.094687161	1.086120379	0.957238887	0.876272842		
Potra2n15c28851.1	-0.233312146	-0.1852838	0.296649127	0.865611082		
Potra2n15c28852.1	-0.041012088	0.08435344	0.83402163	0.940255038	Potri.015G039800.1	AT1G18190.1
Potra2n15c28853.1	-0.676615876	-0.272126348	3.858E-08	0.775391304	Potri.015G039700.1	AT1G73640.1
Potra2n15c28854.1	-0.156280881	0.051534412	0.293131169	0.974756511	Potri.015G039500.1	AT1G18210.1
Potra2n15c28855.1	0.413577628	0.068466821	0.154721059	0.976139323	Potri.015G039300.1	AT3G18020.1
Potra2n15c28856.1	1.664677056	NA	NA	NA	Potri.013G148950.1	
Potra2n15c28857.1	-0.881688304	-0.147652974	0.801037589	NA	Potri.T011700.1	
Potra2n15c28858.1	0.82357019	NA	NA	NA	Potri.015G039100.1	AT3G18010.1
Potra2n15c28859.4	0.45038027	0.510133732	0.270914713	0.660443906	Potri.015G039000.2	AT1G48600.2
Potra2n15c28860.1	-2.060774514	-1.530680149	1.45639E-07	0.124471148	Potri.015G038800.1	AT5G12250.1
Potra2n15c28861.1	-0.624946857	-0.139956803	0.075550375	0.956865545	Potri.015G038700.1	AT1G73590.1
Potra2n15c28862.1	0.105993697	0.005411827	0.313630341	0.995581218	Potri.015G038600.1	AT3G17970.1
Potra2n15c28863.1	-0.093207373	0.058181035	0.748391792	0.966148753		
Potra2n15c28864.1	-0.038106704	-0.141047044	0.830396663	0.911339141	Potri.015G038300.1	AT5G42150.1
Potra2n15c28865.1	-0.123938859	-0.152226134	0.741825081	0.953831244	Potri.015G038200.1	AT1G48570.1
Potra2n15c28866.1	0.1502553	0.244376203	0.634328123	0.871920317	Potri.012G046400.1	AT5G06310.1
Potra2n15c28867.2	-0.25806092	0.009740869	0.081174945	0.995005375	Potri.015G038100.1	AT1G18260.1
Potra2n15c28868.1	0.230616434	0.13458262	0.020934636	0.849067086	Potri.015G037800.1	AT3G54660.1
Potra2n15c28869.1	NA	NA	NA	NA	Potri.015G037700.1	AT1G74000.1
Potra2n15c28870.1	0.239937697	0.1123289	0.034544223	0.829455551	Potri.015G037600.1	AT1G48550.1
Potra2n15c28871.1	-0.058413089	0.362235122	0.816495819	0.683816919	Potri.015G037000.5	AT1G18270.1
Potra2n15c28872.1	2.007137562	-0.559621953	0.169038097	NA	Potri.015G037100.1	AT3G16780.1
Potra2n15c28873.1	0.904424076	0.240011331	4.17671E-10	0.737914109	Potri.015G037000.5	AT1G18270.1
Potra2n15c28874.3	0.042362846	-0.159722792	0.822045087	0.872201371	Potri.002G216700.1	AT5G05800.1
Potra2n15c28875.1	-0.562414038	-0.130173595	0.08686818	0.948577865	Potri.015G036900.1	AT3G17950.1
Potra2n15c28876.1	-1.203603336	-0.358856836	3.70682E-05	0.814981587	Potri.015G036800.1	AT1G18290.1
Potra2n15c28877.1	-0.308153525	-6.7436E-05	0.002109071	0.999937671	Potri.015G036700.1	AT1G48160.1
Potra2n15c28878.1	-0.055856734	-0.058370008	0.723376392	0.95671968	Potri.015G036600.1	AT1G73820.1
Potra2n15c28879.1	-0.81451702	-0.114080191	0.000800146	0.941469266	Potri.015G036500.1	AT3G17930.1
Potra2n15c28880.1	-0.871278581	-0.078751467	7.28841E-07	0.948577865	Potri.015G036500.1	AT3G17930.1

Potra2n15c28881.1	0.208459751	0.257727943	0.267369687	0.798429987		
Potra2n15c28882.1	-0.186185099	0.019035326	0.220516844	0.985016122	Potri.015G036300.1	AT3G17920.1
Potra2n15c28883.1	NA	NA	NA	NA	Potri.015G036201.1	AT1G18280.1
Potra2n15c28884.1	-0.142698852	0.121893888	0.271562506	0.816567218	Potri.015G036000.2	AT3G17880.1
Potra2n15c28885.1	-0.134746737	-0.017549651	0.801996579	0.996032145	Potri.015G036100.2	AT2G37360.1
Potra2n15c28886.1	0.098071145	0.156398476	0.597354999	0.859892315	Potri.015G035800.1	AT3G17860.1
Potra2n15c28887.1	0.721314448	0.453393439	0.038397099	0.829781359	Potri.002G120600.1	AT4G24220.1
Potra2n15c28888.1	0.573272581	0.108668185	0.000108621	0.917746944	Potri.015G035700.1	AT1G71430.1
Potra2n15c28889.1	-0.005768924	0.088999434	0.968418406	0.910878765	Potri.015G035600.1	AT3G17850.1
Potra2n15c28890.1	-1.112279822	-0.045554495	0.000348029	0.986636967	Potri.015G035500.1	AT1G48480.1
Potra2n15c28891.1	-0.009555346	0.473171239	0.965525849	0.487031413	Potri.015G035300.1	AT3G17830.1
Potra2n15c28892.1	0.82357019	0.674963689	NA	NA	Potri.015G035200.1	AT3G47570.1
Potra2n15c28893.1						
Potra2n15c28894.1	-0.54527828	0.012527931	0.044359178	0.996032145	Potri.015G035000.1	AT1G18300.1
Potra2n15c28895.2	NA	NA	NA	NA	Potri.002G067200.1	AT2G01050.1
Potra2n15c28896.1	-0.384897381	0.165253515	0.384104607	0.953831244	Potri.015G034900.2	AT4G21065.1
Potra2n15c28897.1	-0.675182176	-0.488111477	0.010068107	0.282619136	Potri.015G034800.1	AT1G67920.1
Potra2n15c28898.1	0.82357019	NA	NA	NA		
Potra2n15c28899.1	-0.089312412	-0.111603644	0.644540878	0.937276488	Potri.015G034700.1	AT5G37600.1
Potra2n15c28900.1	-0.025151897	0.057824025	0.845188404	0.956865545	Potri.015G034600.1	AT4G36480.2
Potra2n15c28901.1	0.122342145	0.064788831	0.267485134	0.954559968	Potri.015G034500.3	AT1G10170.1
Potra2n15c28902.3	0.187842514	-0.016473441	0.301002279	0.992934677	Potri.015G034400.1	AT3G11520.1
Potra2n15c28903.1	-0.143693291	-0.109839266	0.535443101	0.935130474	Potri.015G034200.1	AT2G40540.2
Potra2n15c28904.1	3.913649453	1.363811008	0.176653947	NA		
Potra2n15c28905.1	2.492428534	NA	NA	NA	Potri.015G034125.1	
Potra2n15c28906.1						
Potra2n15c28907.1	-0.324039411	-0.421671756	0.265279988	0.371384156	Potri.015G034100.1	AT1G73360.1
Potra2n15c28908.1	NA	NA	NA	NA		
Potra2n15c28909.1						
Potra2n15c28910.2	3.205303717	1.429857357	3.25256E-70	0.035009642	Potri.015G034000.1	AT3G17700.1
Potra2n15c28911.1	0.488093205	-0.127054743	0.73265141	0.989407988	Potri.001G214100.1	AT4G19130.1
Potra2n15c28912.1	0.0480535	0.278734278	0.839759039	0.833651746		

Potra2n15c28913.1	2.763864888	1.088798026	1.69996E-27	0.372242801	Potri.015G033900.1	AT3G17700.1
Potra2n15c28914.1	0.458382742	0.071234652	0.001057332	0.953831244	Potri.015G033700.1	AT5G07090.1
Potra2n15c28915.1	-1.049211332	-0.619918934	3.90637E-06	0.350883772	Potri.015G033600.1	AT1G17950.1
Potra2n15c28916.1	-0.329661848	0.011654626	0.006514942	0.995579852	Potri.015G033400.1	AT1G73460.1
Potra2n15c28917.1	0.003313501	0.214563974	0.976560275	0.653059746	Potri.015G033300.2	AT3G17740.1
Potra2n15c28918.2	-0.191148282	0.184809807	0.335691212	0.786910796	Potri.015G033200.1	AT1G73470.1
Potra2n15c28919.1	-0.322969586	-0.329855267	0.916864759	NA		
Potra2n15c28920.1	-0.607349282	-0.231445845	0.000322619	0.901671593	Potri.015G033100.1	AT2G01940.1
Potra2n15c28921.1	-0.072248272	0.030880337	0.82813194	0.987891912	Potri.015G032900.1	AT1G14685.2
Potra2n15c28922.1	-0.26724775	0.076373944	0.042429099	0.930598202	Potri.015G032900.1	AT1G14685.2
Potra2n15c28923.1	-1.597707428	0.908261239	NA	NA	Potri.015G032700.1	AT3G28920.1
Potra2n15c28924.1	0.468920103	0.059486869	0.001025736	0.956889095	Potri.015G032600.1	AT5G37780.1
Potra2n15c28925.1	0.304522741	0.101230394	0.001179931	0.888571801	Potri.015G032500.1	AT3G17770.1
Potra2n15c28926.1					Potri.015G032400.1	
Potra2n15c28927.1	1.664677056	NA	NA	NA	Potri.015G032200.1	AT2G01900.1
Potra2n15c28928.1	0.135097324	0.028695031	0.911593119	0.997546582		
Potra2n15c28929.1	NA	NA	NA	NA	Potri.015G031900.4	AT1G73480.1
Potra2n15c28930.1	-2.784322941	-4.293793199	0.496402814	NA	Potri.015G085500.1	AT5G23960.1
Potra2n15c28931.2	1.70933721	0.236009838	0.510504221	NA	Potri.015G031900.4	AT1G73480.1
Potra2n15c28932.3	-0.403587674	0.304066354	0.100391852	0.857189843	Potri.015G031800.1	AT1G68090.1
Potra2n15c28933.1	-0.420170351	0.026642983	0.767102601	0.996032145	Potri.015G031700.1	AT4G14819.1
Potra2n15c28934.2	-1.503629204	-0.481474604	0.10097576	0.936549944	Potri.015G031600.1	AT5G16560.1
Potra2n15c28935.1	-1.42520112	-0.163377176	6.69228E-07	0.948577865	Potri.015G031400.1	AT3G17790.1
Potra2n15c28936.1	0.090631264	0.308939121	0.708577346	0.794389023	Potri.015G031200.2	AT1G48450.1
Potra2n15c28937.1	0.022880557	0.197110714	0.896155321	0.635323636	Potri.015G031100.1	AT1G31930.3
Potra2n15c28938.1	5.993947666	3.555033132	5.86037E-05	NA	Potri.015G031000.1	AT5G37490.1
Potra2n15c28939.1	0.508691543	0.105163468	0.009373056	0.939607998	Potri.015G030801.1	AT1G48440.1
Potra2n15c28940.1	3.544360404	1.432843529	8.01849E-06	NA	Potri.015G030700.1	AT1G73500.1
Potra2n15c28941.1	0.246355993	0.096365115	0.009942723	0.919722977	Potri.015G030600.1	AT3G17810.1
Potra2n15c28942.1	-0.142967131	0.306120239	0.327580366	0.82876584	Potri.015G030400.1	AT1G18330.2
Potra2n15c28943.2	0.711554606	0.047069536	0.001150321	0.983944804	Potri.015G030300.2	AT1G53345.1
Potra2n15c28944.1	0.465152353	-0.468874946	0.525942303	0.909675463	Potri.015G030200.1	AT3G17730.1

Potra2n15c28945.1	-2.468441083	-0.826291152	0.338933515	NA	Potri.015G030100.1	AT1G48405.1
Potra2n15c28946.2	0.446570392	-0.088930474	0.00477408	0.943438281	Potri.015G029800.2	AT1G17880.1
Potra2n15c28947.1	-0.887740152	-0.144875876	8.29267E-08	0.906877588	Potri.015G029700.2	AT3G17670.2
Potra2n15c28948.1	-0.596089909	-0.047216125	0.00114013	0.978251387	Potri.015G029600.2	AT1G17850.2
Potra2n15c28949.1	0.661048439	0.159292757	1.41123E-05	0.824905557	Potri.012G037500.1	AT3G16780.1
Potra2n15c28950.1	-1.211869808	-0.269893567	0.061389013	0.936549944	Potri.015G029400.1	AT1G73220.1
Potra2n15c28951.1	-0.063596902	0.030932363	0.708927082	0.98409016	Potri.015G029300.1	AT4G27750.1
Potra2n15c28952.1	-0.147063173	0.022887111	0.578579233	0.99114586	Potri.015G029200.3	AT1G14590.1
Potra2n15c28953.1	-0.396380543	-0.205691556	0.106814541	0.869186333	Potri.015G029200.3	AT1G14590.1
Potra2n15c28954.1	0.174806011	-0.093299969	0.926108913	0.993589787	Potri.015G029100.1	AT1G73370.1
Potra2n15c28955.1	-0.492613851	-0.033999206	0.00169138	0.977559521	Potri.015G029000.1	AT1G48410.1
Potra2n15c28956.1	-0.574655858	-0.29062333	2.30273E-06	0.616163505	Potri.015G028900.1	AT4G27760.1
Potra2n15c28957.1	NA	NA	NA	NA	Potri.015G028800.1	AT1G13570.1
Potra2n15c28958.1	-1.371665827	-0.372404817	0.000327463	0.744478336	Potri.015G028700.1	AT1G61260.1
Potra2n15c28959.1	3.982133132	0.467401765	0.023759116	NA	Potri.015G028600.1	AT2G34930.1
Potra2n15c28960.1	-1.317292482	0.151993677	NA	NA	Potri.015G028500.2	AT5G49610.1
Potra2n15c28961.1	0.184382869	0.029392233	0.125703151	0.976302215	Potri.015G028400.1	AT3G19950.1
Potra2n15c28962.3	0.371965538	0.094556618	0.014057687	0.926234768	Potri.015G028300.1	AT1G34130.1
Potra2n15c28963.1	0.175911375	-0.007655571	0.180283098	0.995579852	Potri.015G028200.1	AT5G23250.1
Potra2n15c28964.1	-0.244242709	-0.016852353	0.044834346	0.98177802	Potri.012G035900.3	AT3G19180.1
Potra2n15c28965.1	NA	NA	NA	NA	Potri.015G028000.1	AT3G26300.1
Potra2n15c28966.1					Potri.017G155100.2	AT1G18340.1
Potra2n15c28967.1					Potri.T152200.1	
Potra2n15c28968.1	-0.517804411	-0.092527712	0.517524503	0.983944804	Potri.015G027700.1	AT3G16520.3
Potra2n15c28969.1	0.049875033	-0.299480235	0.906155656	0.885627229	Potri.015G027700.1	AT3G16520.3
Potra2n15c28970.2	0.131424657	-0.326056804	0.761285074	0.750123244	Potri.015G027600.1	AT5G52860.1
Potra2n15c28971.1	-1.06633746	-0.212270208	0.005319684	0.90127796	Potri.015G027100.1	AT5G52870.1
Potra2n15c28972.1	0.365192206	0.103589271	0.008182195	0.862335622	Potri.015G027300.1	AT4G23630.1
Potra2n15c28973.1	0.203475468	0.21995225	0.043992934	0.727843918	Potri.015G026900.1	AT5G52882.1
Potra2n15c28974.1	-0.885983944	-0.332312809	0.012295401	0.831597503	Potri.015G026700.1	AT5G24030.1
Potra2n15c28975.1	2.476290371	NA	NA	NA	Potri.008G220300.1	
Potra2n15c28976.1	3.326442903	1.363811008	NA	NA	Potri.T076201.1	AT5G45400.1

Potra2n15c28977.3	-0.85773174	0.093720321	0.075557379	0.9761362	Potri.015G026700.1	AT5G24030.1
Potra2n15c28978.1	-1.857170344	-0.860996468	0.003732119	0.779077802		
Potra2n15c28979.1	1.664677056	1.363811008	NA	NA	Potri.015G026500.1	AT4G18550.1
Potra2n15c28980.1					Potri.015G026400.1	AT4G18550.1
Potra2n15c28981.2	4.267443226	NA	0.234981317	NA	Potri.015G026300.1	AT5G24080.1
Potra2n15c28982.1	NA	NA	NA	NA		
Potra2n15c28983.3	-0.088260513	-0.206885076	0.839038986	0.897285619	Potri.015G026200.1	AT2G41620.1
Potra2n15c28984.1	-0.162924522	0.077050525	0.106814541	0.943819961	Potri.015G026100.1	AT3G49660.1
Potra2n15c28985.1	0.606462994	0.160429126	2.67538E-06	0.814001428	Potri.015G025800.1	AT2G34930.1
Potra2n15c28986.1	-0.312427266	-0.030130687	0.035157354	0.978146566		
Potra2n15c28987.1	2.13791799	0.07730063	2.25647E-15	0.97601212	Potri.015G024500.2	AT2G34930.1
Potra2n15c28988.1	1.459962178	NA	NA	NA	Potri.006G094700.1	AT3G52970.1
Potra2n15c28989.2	1.643280198	0.923167051	9.29443E-09	0.20731001	Potri.015G025100.2	AT2G34930.1
Potra2n15c28990.1	0.365989469	0.272222225	0.000344559	0.684835668	Potri.015G024400.1	AT3G26560.1
Potra2n15c28991.1	0.991552366	0.135880652	0.003877942	0.970930149	Potri.015G024300.2	AT5G52910.1
Potra2n15c28992.1	2.696443636	1.625775782	8.78333E-08	0.200085324	Potri.015G024200.1	AT5G24090.1
Potra2n15c28993.1	9.211989343	4.748304727	9.86745E-36	0.12009273	Potri.015G024000.1	AT5G24090.1
Potra2n15c28994.1	0.413257991	0.002112271	0.156253258	0.999325699	Potri.012G033700.1	AT5G52960.1
Potra2n15c28995.1	2.606680607	1.648242222	0.007895297	0.79078676	Potri.015G023800.1	AT3G59140.1
Potra2n15c28996.3	2.508268894	0.744135524	0.000159789	0.926125585	Potri.015G023800.1	AT3G59140.1
Potra2n15c28997.1	-0.083831848	-0.064412001	0.515103138	0.937061187	Potri.015G023700.1	AT5G53340.1
Potra2n15c28998.1	-0.097214225	-0.090009478	0.394418427	0.910815461	Potri.015G023600.2	AT5G53330.1
Potra2n15c28999.1	0.514135247	-0.138462119	0.089917505	0.943826727	Potri.015G023500.1	AT4G23740.1
Potra2n15c29000.1	0.075454925	0.034126678	0.631935929	0.973347974	Potri.015G023400.1	AT5G53310.1
Potra2n15c29001.1	-0.05872182	-0.062454076	0.581133413	0.943438281	Potri.012G033000.1	AT5G53300.4
Potra2n15c29002.1	1.480599708	-0.037955387	0.002860662	0.9896961	Potri.015G023200.1	AT4G27950.1
Potra2n15c29003.1					Potri.015G023100.1	
Potra2n15c29004.1	-0.373337037	-0.018824194	0.007510011	0.985393296	Potri.015G022900.1	AT5G53280.1
Potra2n15c29005.1					Potri.015G022800.1	AT5G43360.1
Potra2n15c29006.2	0.502781107	0.232854071	0.028899942	0.804796567	Potri.015G022700.1	AT4G27940.1
Potra2n15c29007.1	1.47147346	1.591888353	NA	NA	Potri.015G022600.1	AT3G61640.1
Potra2n15c29008.1	-0.112166141	0.090049371	0.471741861	0.935851032	Potri.015G022500.4	AT5G53220.2

Potra2n15c29009.1	0.378502742	0.147046264	0.116855854	0.921099436	Potri.015G022400.1	AT5G41480.1
Potra2n15c29010.1	2.735991243	1.571529889	NA	NA	Potri.015G022300.2	AT5G53210.1
Potra2n15c29011.1	-0.777983414	-0.184656446	0.06807332	0.910815461	Potri.015G022100.1	AT5G24120.1
Potra2n15c29012.1	2.1464278	0.787941749	5.94401E-14	0.389989789	Potri.015G022000.1	AT5G53200.1
Potra2n15c29013.1	0.216565131	1.432350639	NA	NA	Potri.015G021500.2	AT5G53190.1
Potra2n15c29014.1					Potri.015G021800.1	AT3G02330.1
Potra2n15c29015.1	-0.181206846	-2.459965807	0.961890434	NA	Potri.015G085500.1	AT5G23960.1
Potra2n15c29016.1					Potri.015G021000.1	
Potra2n15c29017.1	3.534442511	0.674963689	0.404219991	NA	Potri.015G021100.1	AT5G24130.1
Potra2n15c29018.1	0.82357019	NA	NA	NA	Potri.015G021000.1	
Potra2n15c29019.1					Potri.015G021400.1	AT5G24130.1
Potra2n15c29020.1					Potri.015G021600.1	
Potra2n15c29021.1					Potri.015G021400.1	AT5G24130.1
Potra2n15c29022.1					Potri.015G021600.1	
Potra2n15c29023.1					Potri.015G020900.1	
Potra2n15c29024.1					Potri.015G021450.1	
Potra2n15c29025.1					Potri.015G021100.1	AT5G24130.1
Potra2n15c29026.1	-2.878822174	-1.619644824	NA	NA	Potri.005G186300.1	AT1G77060.1
Potra2n15c29027.1	-0.61910233	-0.767708831	NA	NA	Potri.015G021100.1	AT5G24130.1
Potra2n15c29028.1					Potri.015G021600.1	
Potra2n15c29029.1					Potri.015G021400.1	AT5G24130.1
Potra2n15c29030.1	NA	NA	NA	NA	Potri.009G019700.1	AT3G29590.1
Potra2n15c29031.1					Potri.015G020900.1	
Potra2n15c29032.1	-2.878822174	-3.029828947	NA	NA		
Potra2n15c29033.1						
Potra2n15c29034.1					Potri.015G021000.1	
Potra2n15c29035.1	3.420547196	0.674963689	NA	NA	Potri.015G020900.1	
Potra2n15c29036.3	-0.105043532	-0.015384378	0.331398979	0.985313207	Potri.015G020800.1	AT5G53180.1
Potra2n15c29037.1	-0.420971831	-0.064102217	0.000963123	0.940152186	Potri.015G020700.1	AT5G53170.1
Potra2n15c29038.1	-0.352084128	-0.124680799	0.007126774	0.842680333	Potri.005G109700.1	AT1G23780.1
Potra2n15c29039.1	1.616549096	0.235137386	8.09236E-12	0.889391488	Potri.015G020500.1	AT5G53160.2
Potra2n15c29040.1	0.322658167	0.074440091	0.016608589	0.94492924	Potri.015G020400.1	AT3G61690.1

Potra2n15c29041.1	7.266695713	1.363811008	1.19649E-09	NA		Potri.015G020300.2	AT1G78090.1
Potra2n15c29042.1	3.946709692	NA	0.071567484	NA			
Potra2n15c29043.1	-0.226387601	-0.129789304	0.096865675	0.90226843		Potri.015G020200.2	AT5G53150.1
Potra2n15c29044.1	0.626954713	0.323647001	0.252022182	0.824905557		Potri.015G020000.1	AT5G61430.1
Potra2n15c29045.1	3.888459545	NA	0.075038013	NA		Potri.015G019900.1	
Potra2n15c29046.1	1.213862453	-0.104689107	0.000703186	0.95981322		Potri.015G019800.3	AT3G22370.1
Potra2n15c29047.1	1.055117348	0.634607783	2.31231E-12	0.125577936		Potri.015G019500.1	AT3G61700.2
Potra2n15c29048.3	-0.389380794	-0.71303689	0.125844273	0.203824239		Potri.015G019200.1	AT5G53140.1
Potra2n15c29049.2	0.243317567	-0.147802887	0.097603553	0.890978957		Potri.015G019100.1	AT5G53130.1
Potra2n15c29050.1	3.073325943	4.889829123	NA	NA		Potri.015G019000.2	AT3G50160.1
Potra2n15c29051.1	0.82357019	1.895229922	NA	NA		Potri.T137200.2	AT3G50160.1
Potra2n15c29052.1	2.313840756	0.707296578	3.4826E-07	0.762477739		Potri.015G018800.1	AT2G29380.1
Potra2n15c29053.1	-0.218106604	-0.507837434	0.46052349	0.663656471		Potri.015G018700.1	AT3G49170.1
Potra2n15c29054.1	1.190859047	-0.142108668	5.15284E-07	0.917516356		Potri.T136800.1	AT1G66920.2
Potra2n15c29055.1	1.18245063	-0.997099362	0.625995182	NA			
Potra2n15c29056.1	0.82357019	NA	NA	NA		Potri.T137200.2	AT3G50160.1
Potra2n15c29057.1	1.829060366	0.902394348	5.10118E-07	0.185836749			
Potra2n15c29058.1	1.872191029	0.924823158	2.4979E-07	0.147107193		Potri.015G018600.1	AT5G38280.1
Potra2n15c29059.1	1.459962178	NA	NA	NA		Potri.T136300.1	AT5G53110.1
Potra2n15c29060.1	0.011482568	-0.216839339	0.968567953	0.844261648			
Potra2n15c29061.1	0.573743638	-0.002093548	0.001055243	0.999325699		Potri.015G017500.1	AT5G53460.2
Potra2n15c29062.1	-0.02371354	0.04912776	0.925677721	0.969237046		Potri.015G017300.1	AT5G24170.1
Potra2n15c29063.1	0.071612081	0.098459186	0.636767952	0.887678043		Potri.015G017200.4	AT5G06160.1
Potra2n15c29064.1	-0.520336778	-0.187299615	0.001351004	0.862824894		Potri.015G017100.3	AT5G53450.1
Potra2n15c29065.1	0.824497211	0.64247635	NA	NA		Potri.015G016450.1	AT4G01500.1
Potra2n15c29066.1	0.115737197	-0.320623404	0.911589423	0.937276488		Potri.015G017100.3	AT5G53450.1
Potra2n15c29067.1						Potri.016G096100.1	
Potra2n15c29068.1	0.409570783	0.353761836	0.574999377	0.903698951		Potri.T170400.2	AT5G53450.1
Potra2n15c29069.1	0.824497211	-0.046370319	NA	NA		Potri.015G017000.1	AT4G01500.1
Potra2n15c29070.1	0.455208081	0.850163664	0.383003484	0.52733716		Potri.015G017100.3	AT5G53450.1
Potra2n15c29071.1	NA	NA	NA	NA			
Potra2n15c29072.1							

Potra2n15c29073.1	0.102236182	-0.767708831	NA	NA	Potri.015G016200.1	AT4G01500.1
Potra2n15c29074.1	0.643883075	0.114414894	0.004031367	0.940703488	Potri.009G104900.1	AT1G47480.1
Potra2n15c29075.1	0.08566334	0.168533121	0.796661843	0.922993749	Potri.015G017100.3	AT5G53450.1
Potra2n15c29076.1					Potri.015G016450.1	AT4G01500.1
Potra2n15c29077.1	1.076051767	2.57281853	0.456516069	0.62576348	Potri.015G016100.1	AT4G01500.1
Potra2n15c29078.1	5.20958235	2.32414027	0.097241721	NA	Potri.015G016000.1	AT4G01500.1
Potra2n15c29079.1	NA	NA	NA	NA	Potri.015G015900.1	AT4G01500.1
Potra2n15c29080.1	5.866468874	NA	0.004957317	NA	Potri.015G015800.1	AT4G01500.1
Potra2n15c29081.1	NA	NA	NA	NA	Potri.015G014850.1	AT4G01500.1
Potra2n15c29082.1	3.705935136	4.103503532	0.00103195	0.198711864	Potri.T152800.1	
Potra2n15c29083.1	0.926163055	-0.767708831	NA	NA	Potri.T152900.1	AT1G13260.1
Potra2n15c29084.1	1.71697109	2.236452028	0.401901416	NA	Potri.015G015400.1	
Potra2n15c29085.1	-3.100772296	-3.251802187	NA	NA	Potri.015G015501.1	AT4G01500.1
Potra2n15c29086.1	0.355904969	0.330347984	0.108070886	0.735542465	Potri.015G014850.1	AT4G01500.1
Potra2n15c29087.1	0.48950504	0.303120087	3.49642E-10	0.449772714	Potri.015G014800.1	AT5G53440.1
Potra2n15c29088.1					Potri.015G014700.2	AT3G24850.1
Potra2n15c29089.1	1.64774763	NA	NA	NA	Potri.001G155750.1	AT1G21280.1
Potra2n15c29090.2					Potri.015G014200.1	AT3G24850.1
Potra2n15c29091.1	NA	3.498261754	NA	NA	Potri.015G014550.1	AT3G61700.2
Potra2n15c29092.2	0.094473501	0.161130251	0.506955459	0.813575895	Potri.015G014300.1	AT4G27910.1
Potra2n15c29093.1	1.459962178	NA	NA	NA	Potri.015G014700.2	AT3G24850.1
Potra2n15c29094.1	NA	NA	NA	NA	Potri.015G014700.2	AT3G24850.1
Potra2n15c29095.1	-0.193224365	-0.019975292	0.641672075	0.994834714	Potri.015G014000.1	AT5G53420.1
Potra2n15c29096.1	0.689045199	0.334585024	6.99563E-12	0.067172866	Potri.015G013900.1	AT5G53400.1
Potra2n15c29097.1	-0.128188529	-0.081771276	0.379963713	0.923746681	Potri.015G013800.1	AT4G12560.1
Potra2n15c29098.1	0.122317405	-0.895247981	0.923153927	NA		
Potra2n15c29099.1	0.759726793	0.065542574	0.002685119	0.971828097	Potri.015G013700.1	AT3G49220.1
Potra2n15c29100.1	-0.163523917	-0.043722257	0.11815836	0.959009636	Potri.015G013600.1	AT5G24240.1
Potra2n15c29101.1	0.92641307	-0.767708831	NA	NA	Potri.015G013500.1	AT3G08750.1
Potra2n15c29102.1	0.82357019	NA	NA	NA		
Potra2n15c29103.1	0.0700013	0.102434347	0.625622644	0.917516356	Potri.015G013400.1	AT5G24260.1
Potra2n15c29104.1	-0.177336461	2.671263529	0.948081695	0.637289434		

Potra2n15c29105.1	-0.086050114	1.895708492	0.96360392	0.645919795	Potri.015G013300.1	AT5G03170.1
Potra2n15c29106.1	0.394970745	1.586193548	0.704607101	0.716548554	Potri.015G013200.1	AT5G24270.1
Potra2n15c29107.2	-1.65056176	-1.914055331	0.095541548	0.294409158	Potri.012G015100.1	AT5G24270.1
Potra2n15c29108.1	0.167295443	0.125349989	0.089097085	0.924256651	Potri.015G013000.1	AT4G27880.1
Potra2n15c29109.1	-0.07764885	-0.0243193	0.563239919	0.978646026	Potri.015G012900.1	AT5G53350.1
Potra2n15c29110.1	0.285548337	0.449139574	0.048332559	0.665912531	Potri.015G012800.7	AT4G00990.1
Potra2n15c29111.1	0.218479713	-0.041861388	0.332214293	0.974782554	Potri.015G012700.1	AT4G22000.1
Potra2n15c29112.1	-0.166287181	0.051527005	0.469955749	0.970011758	Potri.015G012600.2	AT3G49250.1
Potra2n15c29113.2	-1.105707432	-0.4412168	3.35574E-09	0.722867976	Potri.015G012500.1	AT3G49260.2
Potra2n15c29114.1	-0.836978829	-0.279330712	8.94987E-12	0.357747948	Potri.015G012400.3	AT5G24300.1
Potra2n15c29115.1	-0.202915069	-0.109281477	0.440724881	0.949358779	Potri.015G012300.1	AT5G52990.1
Potra2n15c29116.1	0.023129605	0.031692208	0.849498767	0.971828097	Potri.015G012200.2	AT5G53000.1
Potra2n15c29117.1	0.923753655	0.749761473	2.28683E-05	0.259011748	Potri.015G012000.1	AT5G53020.1
Potra2n15c29118.1	1.629633803	0.264161947	5.05217E-10	0.833651746	Potri.015G011900.2	AT5G53030.1
Potra2n15c29119.1	1.545832463	NA	NA	NA	Potri.015G011800.1	AT5G53040.1
Potra2n15c29120.1	0.461038266	0.088316417	0.02518795	0.953831244	Potri.015G011500.1	AT5G53045.1
Potra2n15c29121.1	-0.382850112	0.044255857	0.010406714	0.972159498		
Potra2n15c29122.1	-0.318541724	0.053938539	0.01884655	0.954559968	Potri.015G011300.1	AT5G53050.2
Potra2n15c29123.1	-0.16850085	0.078432391	0.501212199	0.945173238	Potri.015G011200.1	AT4G03220.1
Potra2n15c29124.1	0.133661038	-0.054433425	0.296807047	0.959881296	Potri.015G011100.1	AT5G53060.1
Potra2n15c29125.1	0.180271465	0.159302254	0.234918952	0.779077802	Potri.015G010900.5	AT5G26760.2
Potra2n15c29126.1	-0.33025501	-0.324060657	0.011069196	0.383645039	Potri.015G010800.1	AT5G53080.1
Potra2n15c29127.1	-0.196366313	0.133649225	0.404309533	0.869327705	Potri.015G010600.2	AT4G27800.1
Potra2n15c29128.1	-1.271158349	-0.585776042	0.21861498	0.909675463	Potri.015G010500.1	
Potra2n15c29129.2	0.077366402	-0.004987681	0.56291108	0.996032145	Potri.015G010400.1	AT5G53480.1
Potra2n15c29130.1	NA	NA	NA	NA	Potri.015G010300.1	AT4G27790.1
Potra2n15c29131.1	NA	NA	NA	NA	Potri.012G017600.1	AT5G42020.1
Potra2n15c29132.1	-0.15094556	-0.100530166	0.200778784	0.872698255	Potri.015G010200.1	AT4G27780.1
Potra2n15c29133.1	-2.002788464	0.16991537	0.575782531	NA	Potri.015G010100.2	AT5G24318.1
Potra2n15c29134.1	0.82357019	NA	NA	NA	Potri.015G009900.1	AT5G53490.3
Potra2n15c29135.1	-0.594690085	-0.341624584	1.45604E-05	0.763747409	Potri.015G009700.1	AT5G24320.1
Potra2n15c29136.1	0.062868544	0.113620541	0.952409141	0.98177802	Potri.012G018300.1	AT5G24330.1

Potra2n15c29137.3	0.009704747	0.09852027	0.96414431	0.903698951	Potri.015G009500.1	AT5G24340.1
Potra2n15c29138.1	0.82357019	NA	NA	NA		
Potra2n15c29139.2	-0.461059443	-0.165221215	0.004520489	0.87062349	Potri.015G009400.2	AT3G26100.2
Potra2n15c29140.1	-1.00392958	-0.764800035	2.18572E-05	0.010031251	Potri.015G009300.1	AT3G61850.2
Potra2n15c29141.1	-1.38235912	-0.445511944	8.90169E-07	0.677013336	Potri.015G009200.1	AT4G27745.1
Potra2n15c29142.1	-0.196159778	-0.020601026	0.165939115	0.987288338	Potri.012G019300.2	AT1G21320.2
Potra2n15c29143.1	-0.283463273	0.360399909	0.722544727	0.916403589		
Potra2n15c29144.1	-0.149296938	0.528640821	0.77138413	0.743612955	Potri.015G008900.1	
Potra2n15c29145.1	-0.385250499	0.493655674	0.415236314	0.560708883	Potri.015G028600.1	AT2G34930.1
Potra2n15c29146.1	-0.637742013	0.329879056	0.08017502	0.775519829	Potri.015G024500.2	AT2G34930.1
Potra2n15c29147.1	0.01232213	0.263189395	0.951546539	0.679660122	Potri.015G008700.2	AT1G32490.1
Potra2n15c29148.1	NA	NA	NA	NA	Potri.015G008600.1	AT1G76390.2
Potra2n15c29149.1	-0.16301988	-0.016165977	0.230760373	0.9869326	Potri.015G008500.1	AT5G24350.1
Potra2n15c29150.1					Potri.015G008300.1	AT1G55580.1
Potra2n15c29151.2	-0.052642367	0.047624755	0.816073242	0.972159498	Potri.015G008200.2	
Potra2n15c29152.1	-0.362874068	-0.252250453	0.012002673	0.813731863	Potri.015G008100.1	AT4G27720.1
Potra2n15c29153.1	-0.822407499	-0.288131444	0.000296818	0.776370366	Potri.015G007900.1	AT5G41970.1
Potra2n15c29154.1	-0.963480977	-0.3486776	6.74341E-05	0.683341072	Potri.015G008000.1	AT4G27700.1
Potra2n15c29155.1	-0.94804615	-0.23781033	0.00012678	0.857374454	Potri.015G007800.1	AT2G46620.1
Potra2n15c29156.1	-0.33373697	-0.13264014	0.001985487	0.855754617	Potri.015G007700.1	AT4G27680.1
Potra2n15c29157.2	1.108850642	0.149976244	2.36108E-11	0.858372686	Potri.015G007600.1	AT5G53560.1
Potra2n15c29158.1	0.420391554	-0.004514744	1.64982E-05	0.996154416	Potri.015G007400.1	AT3G49350.1
Potra2n15c29159.1	0.734208513	0.187619711	2.7599E-06	0.79477084	Potri.015G007200.2	AT5G24400.1
Potra2n15c29160.2	0.414154624	0.024710594	0.004483774	0.9896961	Potri.012G024300.1	AT2G36170.1
Potra2n15c29161.1	NA	NA	NA	NA	Potri.015G007000.2	AT2G33480.1
Potra2n15c29162.1	-0.501358439	0.079405744	0.002488171	0.956865545	Potri.015G006900.2	AT5G53580.1
Potra2n15c29163.1	-0.313885511	0.347946717	0.576160466	0.884463725		
Potra2n15c29164.1	-0.277899582	0.367253417	0.646621045	0.881552217	Potri.015G006800.1	AT4G00880.1
Potra2n15c29165.2	0.169621879	-0.037902729	0.076768397	0.967695582	Potri.015G006700.1	AT5G53620.1
Potra2n15c29166.1	0.091764711	-0.145081658	0.642437689	0.857189843	Potri.015G006500.1	AT5G53650.1
Potra2n15c29167.1	-1.135905341	-0.655253104	0.001381314	0.627094798	Potri.015G006200.2	AT5G53660.1
Potra2n15c29168.1	0.329003989	0.431825303	0.877431343	0.971537796	Potri.015G006100.1	AT1G64940.1

Potra2n15c29169.1	NA	NA	NA	NA		
Potra2n15c29170.1	0.08167524	0.114038548	0.934197833	0.985393296	Potri.015G006000.1	AT3G53480.1
Potra2n15c29171.1	0.08004465	-0.396839949	0.932380832	0.927717809	Potri.015G005900.1	AT4G00820.1
Potra2n15c29172.1	NA	1.381953902	NA	NA	Potri.012G022400.1	AT4G27670.1
Potra2n15c29173.1	-0.722747496	-0.251132454	0.13669873	0.935099709	Potri.015G005700.1	AT5G03890.1
Potra2n15c29174.1	-0.118868318	-0.155252031	0.557464673	0.902847514	Potri.015G005600.4	AT5G64180.1
Potra2n15c29175.2	-0.542285599	0.193068217	0.002314888	0.84487051	Potri.015G005500.1	AT2G46780.1
Potra2n15c29176.1	-0.052407609	-0.058225544	0.776428228	0.95981322	Potri.015G005400.1	AT3G49390.2
Potra2n15c29177.1	-0.137057901	-0.090102299	0.317179478	0.903477395	Potri.015G005200.1	AT5G54180.1
Potra2n15c29178.1	-0.171508681	0.013295402	0.232655412	0.994762886	Potri.015G005000.1	AT1G64720.1
Potra2n15c29179.2	0.121568756	0.161597381	0.679436375	0.943411075	Potri.015G004800.1	AT5G54165.1
Potra2n15c29180.2	-0.128342906	0.079958738	0.291317794	0.930203195	Potri.015G004600.2	AT3G49430.2
Potra2n15c29181.1	-0.24935492	0.221383363	0.188453031	0.81510213	Potri.015G004500.1	AT5G55830.1
Potra2n15c29182.1					Potri.015G004300.1	AT4G18830.1
Potra2n15c29183.1	-0.116863896	-0.700941268	0.868320594	0.833952007	Potri.015G004200.1	AT5G54130.2
Potra2n15c29184.1	0.090964271	-0.003273264	0.510759738	0.997932292	Potri.015G004100.1	AT3G49530.1
Potra2n15c29185.1	0.434128692	-0.065206434	0.038519335	0.966148753	Potri.015G004000.1	AT5G54140.1
Potra2n15c29186.1	0.984978112	0.552420809	0.022611216	0.80723942	Potri.015G003900.1	AT5G24580.1
Potra2n15c29187.1	2.001261811	-1.55593228	0.56624232	NA	Potri.012G007200.1	AT4G27590.1
Potra2n15c29188.1	-2.427870958	-2.121082203	0.338026977	NA		
Potra2n15c29189.1	-0.931179539	-0.31321692	0.000119745	0.808716407		
Potra2n15c29190.1	-0.062957116	0.071240527	0.636301192	0.929248075	Potri.015G003700.1	AT3G49490.1
Potra2n15c29191.1	1.545832463	NA	NA	NA	Potri.015G003600.1	AT1G05260.1
Potra2n15c29192.1	0.873054917	0.481901696	3.53428E-08	0.55202558	Potri.015G003500.1	AT1G05260.1
Potra2n15c29193.1	0.823796729	0.47193461	3.78687E-07	0.563956825	Potri.015G003300.1	AT3G49470.1
Potra2n15c29194.1	NA	NA	NA	NA	Potri.012G006501.1	AT4G27660.1
Potra2n15c29195.1	-0.376562001	-0.367462843	0.137529222	0.858371617	Potri.015G003100.4	AT5G54160.1
Potra2n15c29196.3	0.021461912	0.038334978	0.917671993	0.983474808	Potri.015G003000.3	AT5G45140.1
Potra2n15c29197.1	NA	1.571529889	NA	NA	Potri.016G030800.1	AT2G40860.1
Potra2n15c29198.1	NA	1.571529889	NA	NA	Potri.015G002900.1	AT1G71930.1
Potra2n15c29199.1	5.876279112	1.757989781	8.7245E-51	0.351105764	Potri.015G002800.1	AT5G24530.1
Potra2n15c29200.1	0.267384443	0.030968033	0.190487987	0.98177802	Potri.012G006100.1	AT5G24520.2

Potra2n15c29201.1	-0.706877099	-0.679247333	0.011710748	0.388007972		
Potra2n15c29202.1	-0.447673371	-0.361941992	0.014985811	0.552458765	Potri.015G002500.1	AT5G24460.1
Potra2n15c29203.1	-1.097289796	-0.562856121	0.476434194	NA	Potri.015G002400.1	AT5G53730.1
Potra2n15c29204.1	-1.233896791	-0.472200185	1.96698E-08	0.809323756	Potri.015G002300.2	AT5G24470.1
Potra2n15c29205.1	-0.11858583	0.379293119	0.792398919	0.876272842	Potri.015G002200.6	AT5G24490.1
Potra2n15c29206.1	0.104246096	0.187294475	0.685181546	0.903140487	Potri.015G002100.1	AT4G03220.1
Potra2n15c29207.1	0.271130988	-0.026773561	0.044349932	0.983113084	Potri.015G001900.1	AT4G27585.1
Potra2n15c29208.1	-0.750482475	0.599630174	0.011098113	NA	Potri.015G001800.1	AT4G01575.1
Potra2n15c29209.1	-1.77877284	0.682029087	0.000133096	NA	Potri.015G001700.1	
Potra2n15c29210.1	NA	1.394613675	NA	NA		
Potra2n15c29211.1						
Potra2n15c29212.1	-0.522531878	-0.256976132	0.000126593	0.480580998	Potri.015G001300.1	AT5G24600.1
Potra2n15c29213.1	0.437549562	-0.297497853	0.031032453	0.847978165	Potri.015G001500.1	AT5G24600.1
Potra2n15c29214.1	0.361895003	0.125358077	0.065748317	0.919424623	Potri.015G001200.1	AT1G04635.1
Potra2n15c29215.1	0.605125685	0.210662181	5.54521E-06	0.736000077	Potri.015G000800.1	AT5G24620.1
Potra2n15c29216.1	0.72122406	0.403072895	0.289137314	0.888096054	Potri.012G004700.2	AT5G24630.5
Potra2n15c29217.1	NA	NA	NA	NA	Potri.001G155750.1	AT1G21280.1
Potra2n15c29218.1	0.800982813	0.66824537	3.49049E-05	0.389989789	Potri.015G000600.1	AT5G24650.1
Potra2n15c29219.1	1.14569093	0.548771571	5.26953E-05	0.791610698	Potri.015G000500.1	AT5G24660.1
Potra2n15c29220.1	-0.116829307	0.016131104	0.404149721	0.989300076	Potri.015G000400.1	AT3G49590.1
Potra2n15c29221.1	2.735991243	1.394613675	NA	NA	Potri.012G004300.1	AT5G24680.1
Potra2n15c29222.1	NA	0.674963689	NA	NA		
Potra2n15c29223.1	0.276585454	0.996253152	0.804734077	0.813016528	Potri.015G000200.2	AT3G28860.1
Potra2n15c29224.1	0.276585454	0.892026126	0.804734077	0.84487051		
Potra2n15c29225.1	-0.036216616	0.064880166	0.841217817	0.956865545	Potri.015G000100.1	AT4G27870.1
Potra2n15c29226.1	1.828198622	1.79446585	0.179794726	0.813731863	Potri.019G047900.1	AT2G01050.1
Potra2n15c29227.1						
Potra2n15c29228.1						
Potra2n15c29229.1	NA	NA	NA	NA		
Potra2n1600s37146.1	1.664677056	0.674963689	NA	NA	Potri.013G149600.1	AT1G27410.1
Potra2n1605s37147.1	0.694378976	0.09798066	0.000726734	0.940735643	Potri.014G189400.1	AT3G07930.3
Potra2n1608s37148.1	-0.18728096	-0.393529149	0.822743722	0.921015963	Potri.009G153600.1	AT3G43600.1

Potra2n160s34779.2					Potri.016G092700.4	AT1G53430.1
Potra2n1610s37149.1						
Potra2n1611s37150.1	-2.337106966	-2.486725844	NA	NA	Potri.003G076000.1	AT4G25200.1
Potra2n1612s37151.1	-1.318157081	-2.773492394	0.156616697	0.104142768	Potri.003G189066.1	AT5G07010.1
Potra2n1612s37152.1	5.651629658	2.319336275	6.88042E-10	NA	Potri.003G189100.1	AT5G07010.1
Potra2n161s34780.1	-0.337399433	-0.030424029	0.138802277	0.986636967	Potri.010G009600.1	AT4G27680.1
Potra2n1620s37153.1					Potri.019G078600.1	AT5G36110.1
Potra2n1621s37154.1	2.018008269	1.103590403	0.001170267	0.583548322	Potri.016G026000.4	AT2G01570.1
Potra2n1621s37155.1	NA	1.89838425	NA	NA		
Potra2n1622s37156.1	NA	NA	NA	NA		
Potra2n1625s37157.1	2.104359249	0.469015389	0.00045056	0.912777843	Potri.010G001500.10	AT1G48210.1
Potra2n162s34781.1	-0.761310653	0.124662881	0.260622935	0.973347974	Potri.010G007200.1	AT3G06790.2
Potra2n1630s37158.1	-0.387046793	-0.163080497	0.002499102	0.885628484	Potri.010G227200.1	AT3G10390.1
Potra2n1637s37159.1	NA	NA	NA	NA	Potri.003G010800.1	
Potra2n1641s37160.5	-1.037571526	-1.098780153	0.579861291	NA	Potri.004G085400.1	AT5G37600.1
Potra2n164s34782.1						
Potra2n164s34783.1						
Potra2n1656s37161.1					Potri.012G029900.1	
Potra2n1658s37162.1						
Potra2n1661s37163.1						
Potra2n1661s37164.1					Potri.018G082700.2	
Potra2n1663s37165.1	-0.508833975	-0.273177411	0.015956911	0.816451105	Potri.017G007700.1	AT2G44065.1
Potra2n1666s37166.1	NA	NA	NA	NA		
Potra2n1667s37167.1	-1.35927387	-1.507841271	NA	NA	Potri.014G131300.1	AT2G21045.1
Potra2n1670s37168.2	NA	NA	NA	NA	Potri.013G120800.1	AT4G35160.1
Potra2n167s34784.1	2.292789621	2.905971613	0.018556206	0.48231244	Potri.008G207200.1	ATCG00270.1
Potra2n1683s37169.1					Potri.019G066700.1	AT3G07820.1
Potra2n1687s37170.1	NA	NA	NA	NA	Potri.014G187400.1	
Potra2n169s34785.1						
Potra2n169s34786.1	0.60075836	1.14778412	0.704791879	0.805461106	Potri.014G064300.2	AT5G17680.1
Potra2n169s34787.1	1.461072131	1.935220324	0.417751927	NA	Potri.T129400.1	AT4G12010.1
Potra2n16c29230.2	0.428710219	0.127291293	4.12909E-07	0.762477739	Potri.016G000200.1	AT1G79610.1

Potra2n16c29231.1	-0.222932512	-0.166123127	0.287004876	0.684244573	Potri.016G000300.1	AT2G44190.1
Potra2n16c29232.1	-0.219656221	-0.148027447	0.32604847	0.674175338	Potri.016G000400.1	AT1G07410.1
Potra2n16c29233.2	-0.215966201	0.047996378	0.4937443	0.973347974	Potri.016G000500.1	AT2G44200.1
Potra2n16c29234.1	1.307385612	0.449154747	7.71366E-05	0.541196221	Potri.016G000600.1	AT5G10770.1
Potra2n16c29235.1	0.45436729	1.554877904	0.394335245	0.161675303	Potri.016G000700.1	
Potra2n16c29236.1	0.311282338	1.604202409	0.513295865	0.132580843	Potri.016G000800.1	AT1G55680.1
Potra2n16c29237.1	0.221376971	0.819717886	0.436596846	0.31367558	Potri.016G000900.1	AT3G52155.1
Potra2n16c29238.1	-0.310866056	-0.094837927	0.004339777	0.880870972	Potri.016G001000.1	AT4G17150.1
Potra2n16c29239.4	-0.186404731	-0.024586179	0.171646724	0.981761466	Potri.006G000800.10	AT2G01060.1
Potra2n16c29240.1	-0.088901419	0.022507849	0.377046485	0.983113084	Potri.016G001200.1	AT3G24120.1
Potra2n16c29241.1	-1.017448758	-0.433111459	1.86119E-06	0.650882346	Potri.016G001300.1	AT3G54780.1
Potra2n16c29242.2	-0.007581872	-0.142285885	0.980739176	0.849749156	Potri.016G001400.10	AT1G64810.2
Potra2n16c29243.1	1.852481479	-0.128948702	6.35427E-07	0.949847686	Potri.016G001600.1	AT5G64620.1
Potra2n16c29244.2	-0.0305041	-0.093704961	0.919575108	0.937846405	Potri.016G001700.2	AT5G47780.1
Potra2n16c29245.1	-1.401269417	-0.424025754	9.64072E-11	0.575977792	Potri.016G001800.1	
Potra2n16c29246.1	-1.362057359	0.019966917	0.033516068	0.995579852	Potri.016G001900.1	
Potra2n16c29247.1	-3.912263176	-1.944585402	0.070390996	NA	Potri.006G001200.1	AT3G24000.1
Potra2n16c29248.1	-2.880997526	-0.105129094	NA	NA	Potri.016G002000.1	AT3G13310.1
Potra2n16c29249.1	-0.113603379	0.096077442	0.448593921	0.902626948	Potri.016G002100.1	AT3G13300.1
Potra2n16c29250.1	-0.02996166	0.138055063	0.873145024	0.930059926	Potri.016G002300.1	AT3G57520.1
Potra2n16c29251.1	-0.069028428	-0.119324951	0.719195312	0.875559093	Potri.016G002400.1	AT5G64670.1
Potra2n16c29252.1	-0.11250867	-0.042959342	0.237195486	0.949966783	Potri.016G002700.1	AT5G47810.1
Potra2n16c29253.3	-0.7632952	-0.313429802	1.6804E-06	0.724604666	Potri.016G002800.1	AT4G17180.1
Potra2n16c29254.1	-0.755215547	-0.285781152	1.55489E-06	0.749684992		
Potra2n16c29255.1	-0.61910233	3.006803074	NA	NA	Potri.016G003000.2	AT5G14420.1
Potra2n16c29256.1	NA	NA	NA	NA	Potri.016G003033.1	
Potra2n16c29257.1	-1.317292482	0.475678052	NA	NA	Potri.016G003100.1	AT1G55800.1
Potra2n16c29258.1	-0.088304498	-0.029123769	0.672872258	0.9869326	Potri.016G003200.2	AT4G15950.1
Potra2n16c29259.1	0.92556685	0.431284531	1.5932E-05	0.734313982	Potri.016G003400.1	AT5G56000.1
Potra2n16c29260.1	-0.345797957	0.040106603	0.108772844	0.985016122	Potri.016G003700.1	AT5G47800.1
Potra2n16c29261.1	0.837442667	0.366266141	3.2453E-10	0.370539059	Potri.016G003900.2	AT1G03370.1
Potra2n16c29262.1	NA	1.571529889	NA	NA	Potri.016G004000.1	

Potra2n16c29263.2	1.319266568	0.175361689	0.072674657	0.979775311	Potri.016G004100.2	AT5G47770.1
Potra2n16c29264.1	0.131926892	0.231119426	0.456562033	0.704368358	Potri.016G004200.1	AT5G47760.1
Potra2n16c29265.1	-0.220542861	0.2018225	0.575161771	0.920141992	Potri.016G004300.1	AT1G60870.1
Potra2n16c29266.1	0.09396736	1.119138171	0.883720657	0.476972044		
Potra2n16c29267.1	1.007888379	0.879147934	0.04639625	0.392592301		
Potra2n16c29268.1	1.041220248	0.895741292	0.047067376	0.351090479	Potri.016G004500.2	
Potra2n16c29269.1	-0.796193478	0.104777354	0.001123098	0.967129771	Potri.016G004800.2	AT4G26590.1
Potra2n16c29270.1	-0.132690542	0.063860894	0.086720796	0.907032966	Potri.016G004900.1	AT5G47750.1
Potra2n16c29271.1	NA	1.363811008	NA	NA	Potri.016G005001.1	
Potra2n16c29272.1	0.192035567	-0.164787402	0.281566488	0.860810425	Potri.016G005100.1	AT5G47730.1
Potra2n16c29273.1	0.248067324	-0.1937535	0.200367495	0.831463341	Potri.016G005200.1	AT1G12150.1
Potra2n16c29274.1	5.883428364	3.414715675	2.90402E-08	NA	Potri.016G005300.1	AT5G47710.2
Potra2n16c29275.1	-0.098282892	0.184110726	0.480847323	0.773574963	Potri.016G005400.1	AT5G47690.1
Potra2n16c29276.1	-0.300065179	1.180712205	0.646868106	0.405760438	Potri.001G112300.1	AT4G11860.1
Potra2n16c29277.1	0.130090172	0.235696869	0.604472589	0.812696706	Potri.016G005500.1	AT5G47680.1
Potra2n16c29278.1	0.82357019	NA	NA	NA	Potri.016G005600.1	AT5G47670.1
Potra2n16c29279.1	NA	1.381953902	NA	NA	Potri.006G005100.1	AT4G30825.1
Potra2n16c29280.1	0.229019299	-0.134819485	0.430847938	0.946565363	Potri.016G005900.1	AT1G76880.1
Potra2n16c29281.1	3.82767169	1.363811008	0.183535485	NA	Potri.016G006000.1	AT5G47650.1
Potra2n16c29282.1	-0.034955474	0.157489182	0.881231899	0.863165286	Potri.016G006100.1	AT4G14540.1
Potra2n16c29283.1	-0.800711373	-0.331925144	1.05809E-05	0.753052725	Potri.016G006200.1	AT5G47635.1
Potra2n16c29284.1	-0.764140382	-0.319939787	5.20625E-06	0.758367608	Potri.016G006300.1	AT5G47630.2
Potra2n16c29285.1	-0.623247586	-0.097140843	0.000761996	0.940841616	Potri.016G006400.1	AT4G16360.3
Potra2n16c29286.1	-2.105531715	-0.99113148	1.34747E-12	0.314225571	Potri.006G005900.1	AT3G42640.1
Potra2n16c29287.1	-2.068978399	-0.769239878	9.03445E-15	0.531648038	Potri.016G006500.1	AT4G16370.1
Potra2n16c29288.1	-1.268089763	-0.32719427	6.59237E-07	0.718626343	Potri.016G006600.1	AT5G50740.3
Potra2n16c29289.1	-0.912041044	-0.554664692	2.15178E-11	0.248778911	Potri.016G006700.1	AT4G16380.1
Potra2n16c29290.2	-1.48939751	-0.454839765	1.39979E-11	0.69862311	Potri.016G006900.1	AT4G17220.1
Potra2n16c29291.1	-0.953503018	-0.251102127	1.69646E-05	0.847341104	Potri.016G006800.1	AT4G16390.1
Potra2n16c29292.1	-0.984653616	-0.322241813	4.95359E-08	0.650882346	Potri.016G007101.1	AT4G16400.1
Potra2n16c29293.1	-3.04142869	-1.757319919	NA	NA	Potri.016G007200.1	AT5G55630.1
Potra2n16c29294.1	-0.644553233	-0.170959281	6.93366E-07	0.788065047	Potri.016G007300.2	AT5G28530.1

Potra2n16c29295.1	-0.614659091	0.21431102	0.259648215	0.953608209	Potri.016G007400.1	AT3G13130.1
Potra2n16c29296.1	-1.480017138	-0.351251685	0.762294616	NA	Potri.016G007200.1	AT5G55630.1
Potra2n16c29297.1	-0.501017616	0.485108104	0.410481315	0.857189843	Potri.016G007400.1	AT3G13130.1
Potra2n16c29298.1	-0.047034791	0.106568458	0.792246662	0.915358268	Potri.016G007500.1	AT5G55600.3
Potra2n16c29299.1	-0.135423072	0.100953874	0.372079268	0.914162682	Potri.016G007600.1	AT3G07740.1
Potra2n16c29300.1	NA	1.394613675	NA	NA	Potri.016G007700.1	AT4G13290.1
Potra2n16c29301.1	0.710286199	-0.128886219	0.069374922	0.968926993	Potri.016G008400.1	AT4G17240.1
Potra2n16c29302.1	0.142660267	0.157615229	0.38071658	0.784063198	Potri.016G008700.1	AT4G16440.1
Potra2n16c29303.1	-0.228631487	0.07260451	0.377634154	0.968880255	Potri.016G008800.1	AT2G35760.1
Potra2n16c29304.1	NA	NA	NA	NA	Potri.016G009100.1	AT3G22910.1
Potra2n16c29305.1	NA	1.363811008	NA	NA	Potri.016G009200.1	AT4G16447.1
Potra2n16c29306.1	2.495931429	0.889581406	7.13021E-09	0.23169116		
Potra2n16c29307.1	2.257629681	0.862975157	7.36733E-14	0.34027789	Potri.016G009500.1	AT3G63380.1
Potra2n16c29308.1	3.090476637	0.966535192	0.000272384	0.343728983	Potri.016G009400.2	AT5G57110.1
Potra2n16c29309.1	2.201959875	0.836402549	1.93968E-08	0.522866183	Potri.016G009500.1	AT3G63380.1
Potra2n16c29310.1	0.58914126	0.303394046	0.013735844	0.718991569	Potri.006G016300.4	AT4G16450.2
Potra2n16c29311.1	0.65865661	-0.077351322	0.107885515	0.924951347	Potri.016G009700.1	AT4G17230.1
Potra2n16c29312.1	0.106762447	0.022521849	0.676991319	0.987959275	Potri.016G009800.1	AT4G16460.3
Potra2n16c29313.2	-0.14371705	0.142752466	0.31128277	0.900823365	Potri.016G009951.1	AT3G07800.1
Potra2n16c29314.4	-0.454582243	0.176543186	0.05914339	0.93017274	Potri.016G010200.1	AT3G07810.1
Potra2n16c29315.1	0.06268989	-0.0107449	0.773530152	0.996032145	Potri.016G010300.1	AT5G47520.1
Potra2n16c29316.1						
Potra2n16c29317.1	-0.828057725	-1.792120373	NA	NA	Potri.016G010600.3	AT4G16480.1
Potra2n16c29318.1	-0.188536427	-0.350122856	0.58897934	0.397878377	Potri.016G010800.1	AT4G16490.1
Potra2n16c29319.1	-1.047028529	-0.552505502	0.000849491	0.736000077	Potri.016G010900.1	AT5G47530.1
Potra2n16c29320.6	0.028866914	0.205407338	0.76417977	0.749144604	Potri.016G011100.1	AT5G47540.1
Potra2n16c29321.2	1.309481394	-0.652021239	0.076455226	0.903477395	Potri.016G012150.1	AT1G53440.1
Potra2n16c29322.1	0.690601775	0.381306976	0.006240185	0.842247476	Potri.010G156300.3	AT3G02870.1
Potra2n16c29323.1	-0.013634115	0.224322461	0.939055955	0.845656549		
Potra2n16c29324.3	1.184315056	-0.718991201	0.02287245	0.74700417	Potri.010G155600.1	AT1G53440.1
Potra2n16c29325.1	0.646893895	0.288722226	0.051071876	0.898208704	Potri.010G156300.3	AT3G02870.1
Potra2n16c29326.2	2.917173923	-0.093495211	8.47265E-13	0.986190118	Potri.016G011400.1	AT1G53440.1

Potra2n16c29327.1						
Potra2n16c29328.1	-0.582814947	-1.532929637	0.593213242	0.611487896		
Potra2n16c29329.4	1.856439527	-0.343455673	0.003820266	0.940449094	Potri.016G011200.2	AT1G53430.1
Potra2n16c29330.1	NA	NA	NA	NA	Potri.016G012400.1	AT5G45480.1
Potra2n16c29331.5	-0.123042983	-0.611801765	0.636075823	0.250980732	Potri.016G011200.2	AT1G53430.1
Potra2n16c29332.1	NA	0.674963689	NA	NA		
Potra2n16c29333.1	2.798378656	-0.419697372	0.00224662	0.961703915	Potri.016G092700.4	AT1G53430.1
Potra2n16c29334.1	-2.764008623	-1.995457408	0.051163084	0.485359736	Potri.016G012400.1	AT5G45480.1
Potra2n16c29335.1	NA	0.674963689	NA	NA	Potri.006G012200.2	AT5G45480.1
Potra2n16c29336.2	-0.095651044	-0.324853936	0.908973708	0.937687311	Potri.010G154800.1	
Potra2n16c29337.1	-0.589502397	-0.082058538	0.002410955	0.966077009	Potri.010G154700.1	AT4G12560.2
Potra2n16c29338.1	1.373561921	0.918462502	4.17038E-08	0.094167424	Potri.016G012600.1	AT1G47390.1
Potra2n16c29339.2	1.135081914	0.426310431	2.35488E-05	0.623736221	Potri.010G154500.1	AT4G12560.2
Potra2n16c29340.1	2.610910166	1.571529889	NA	NA	Potri.010G154400.1	AT4G19110.2
Potra2n16c29341.1	0.205192136	0.03772527	0.106603972	0.972159498	Potri.016G059800.2	AT4G12570.1
Potra2n16c29342.1	-1.313297684	-0.049645751	7.46924E-13	0.982453292		
Potra2n16c29343.1	-0.131238869	-0.092738175	0.423908607	0.923397137	Potri.016G013100.1	AT4G12590.1
Potra2n16c29344.1	NA	NA	NA	NA		
Potra2n16c29345.1	-1.323355779	-0.663072377	0.03732085	0.796023085	Potri.016G013200.1	AT4G22370.1
Potra2n16c29346.1	-0.158005231	0.082707459	0.39120263	0.956773505	Potri.016G013300.1	AT4G21060.2
Potra2n16c29347.1	-0.561104231	-0.233756509	5.68782E-05	0.743612955	Potri.016G013400.1	AT4G22360.1
Potra2n16c29348.1	-0.027312358	0.022546122	0.884012764	0.984910847	Potri.016G013500.1	AT5G20130.1
Potra2n16c29349.1	-0.595952689	0.586757891	NA	NA		
Potra2n16c29350.1	-1.128650971	-0.357375608	5.3558E-11	0.696847835	Potri.016G013700.2	AT3G07880.1
Potra2n16c29351.1	-3.854296585	-1.002472248	0.057544543	0.910815461		
Potra2n16c29352.1	0.189139283	0.024227321	0.431021019	0.985393296	Potri.016G014000.1	AT4G22350.2
Potra2n16c29353.1	0.917383428	0.078201403	0.06224336	0.974367223	Potri.016G014100.1	AT3G21760.1
Potra2n16c29354.1	-0.61910233	-0.767708831	NA	NA		
Potra2n16c29355.1	3.82714942	0.264797305	0.000174651	0.961372512	Potri.016G014300.1	AT1G07250.1
Potra2n16c29356.1	3.721573327	0.28245136	6.50021E-06	0.936170918	Potri.016G014100.1	AT3G21760.1
Potra2n16c29357.1	2.172175621	-0.046673635	3.19102E-10	0.987226805	Potri.016G014500.1	AT1G07250.1
Potra2n16c29358.1	-0.073897499	-0.241507148	0.783026683	0.857189843	Potri.016G014600.1	AT3G51970.1

Potra2n16c29359.1	-0.731914091	-0.08114057	0.595313907	0.9896961	Potri.016G014700.1	AT3G51970.1
Potra2n16c29360.1	0.818354426	0.698340425	0.091483056	0.663950227	Potri.006G009400.1	AT4G28320.1
Potra2n16c29361.1	-0.469416058	-0.092703368	0.115484736	0.95671968	Potri.016G014900.1	AT1G12060.1
Potra2n16c29362.1	NA	NA	NA	NA		
Potra2n16c29363.1	-0.036877172	0.052149669	0.779704654	0.943332134	Potri.016G015100.2	AT4G12610.2
Potra2n16c29364.2	1.839583761	0.687547657	6.75907E-05	0.754074409	Potri.016G015200.2	AT3G27200.1
Potra2n16c29365.1	4.065004306	3.010689165	0.261883304	NA	Potri.016G015300.1	
Potra2n16c29366.3	-0.113739949	-0.099234056	0.616080579	0.923348116	Potri.016G015400.1	AT3G22110.1
Potra2n16c29367.1	2.671261614	0.420430172	2.13556E-32	0.830056077		
Potra2n16c29368.1	2.55245309	0.230135307	8.61763E-31	0.917510096	Potri.016G015500.1	AT3G22142.1
Potra2n16c29369.1	2.464817023	0.125742563	2.758E-27	0.956622854		
Potra2n16c29370.1	2.752493113	0.842107139	0.027915686	NA		
Potra2n16c29371.1	7.60779323	5.291634458	4.75122E-07	NA	Potri.016G015600.1	AT3G54540.1
Potra2n16c29372.1	-0.010318772	0.054000499	0.977481423	0.975578749	Potri.016G015700.1	AT3G21760.1
Potra2n16c29373.1	NA	NA	NA	NA	Potri.006G007800.1	AT3G22150.1
Potra2n16c29374.1	-1.317292482	-0.05574223	NA	NA		
Potra2n16c29375.1	-2.089331962	0.131140786	0.000946135	0.977786506	Potri.016G016000.1	AT3G21760.1
Potra2n16c29376.1	5.000576175	2.343566208	4.9379E-50	0.019176641		
Potra2n16c29377.1	5.873198326	2.717747507	1.03963E-62	0.004949178	Potri.016G016100.1	AT3G21790.1
Potra2n16c29378.1	1.043838817	0.425805625	5.03129E-08	0.620427585	Potri.016G016700.1	AT3G21750.1
Potra2n16c29379.1	2.321776178	0.290605075	3.46653E-16	0.862335622	Potri.016G016800.1	AT3G21760.1
Potra2n16c29380.1	1.429662053	0.29235048	1.94259E-09	0.895474078	Potri.016G016400.1	AT3G21780.1
Potra2n16c29381.1	2.090539653	0.509106726	1.46113E-21	0.5722922	Potri.016G017100.1	AT3G21750.1
Potra2n16c29382.1	3.52874896	1.929973272	3.28742E-11	0.102775569	Potri.016G017100.1	AT3G21750.1
Potra2n16c29383.1	2.139409216	0.909799983	4.72215E-06	0.387987556	Potri.016G017232.1	AT3G21760.1
Potra2n16c29384.1	3.39160171	1.710065316	0.000127951	0.723658895	Potri.016G017232.1	AT3G21760.1
Potra2n16c29385.1	0.642090492	0.676841134	0.043277097	0.269417208	Potri.016G017232.1	AT3G21760.1
Potra2n16c29386.1	1.69323149	1.294899374	0.000565045	0.218867133	Potri.016G017232.1	AT3G21760.1
Potra2n16c29387.1	0.768358513	0.78232186	0.005492306	0.007318585	Potri.016G016500.1	AT3G21790.1
Potra2n16c29388.1	0.751496293	0.813272577	0.029573152	0.095938059	Potri.016G017232.1	AT3G21760.1
Potra2n16c29389.1	3.392323373	0.727387503	1.05064E-12	0.84640933	Potri.016G017400.1	AT3G21790.1
Potra2n16c29390.1	0.074669879	0.539833603	0.758888544	0.645919795	Potri.016G017500.1	AT2G32260.1

Potra2n16c29391.1	0.375707388	0.27202148	0.924126579	NA	Potri.016G017600.2	AT3G02960.1
Potra2n16c29392.1	-0.755337532	-0.230588177	0.008694218	0.935099709	Potri.016G017700.1	AT1G05310.1
Potra2n16c29393.1	-0.814220732	-0.103420431	0.000250125	0.944081499	Potri.016G017800.1	AT5G46680.1
Potra2n16c29394.1	1.499190241	0.593669702	4.42929E-06	0.601577719	Potri.016G017900.1	AT4G39720.1
Potra2n16c29395.1	1.465327202	0.493157565	3.10344E-05	0.733405498	Potri.016G017900.1	AT4G39720.1
Potra2n16c29396.1	-1.863938812	1.556458572	0.599986891	NA	Potri.016G018001.1	AT1G22340.1
Potra2n16c29397.1	-0.20042185	-0.011718439	0.1795847	0.992934677	Potri.016G018100.10	AT2G32250.2
Potra2n16c29398.1	-0.266555658	0.200104204	0.604954322	0.935851032		
Potra2n16c29399.1	1.171016105	-1.507841271	NA	NA		
Potra2n16c29400.1	-0.2099997	0.014290782	0.09708951	0.991147673	Potri.016G018300.1	AT3G22170.1
Potra2n16c29401.1	0.20089385	0.049042943	0.244058769	0.955628098	Potri.016G018400.1	AT4G15080.1
Potra2n16c29402.1	0.459114238	0.286600854	9.66541E-07	0.51104236	Potri.016G018500.1	AT3G22200.1
Potra2n16c29403.1	0.928749944	-0.438628323	NA	0.835819291	Potri.016G018600.1	AT2G36450.1
Potra2n16c29404.1	-0.063543093	0.211983245	0.69535991	0.694611009	Potri.016G018700.1	AT2G32240.1
Potra2n16c29405.1	-0.175810903	0.093003603	0.144299557	0.874496903		
Potra2n16c29406.1	0.247232264	0.280226484	0.604350334	0.885375133		
Potra2n16c29407.1	-0.230112784	-0.230893658	0.31567289	0.775391304	Potri.016G018900.1	AT5G41920.1
Potra2n16c29408.1	0.639220152	0.090005851	0.000126859	0.947317412	Potri.016G019000.1	AT4G15000.1
Potra2n16c29409.1	-0.081938561	0.048741676	0.530194858	0.956865545	Potri.016G019201.1	AT2G32210.1
Potra2n16c29410.1	1.014501944	-0.677724168	0.487080943	NA		
Potra2n16c29411.1	2.49578104	0.8056396	0.068013386	NA	Potri.016G019400.1	AT3G22250.1
Potra2n16c29412.2	-1.640534096	-0.849053731	0.017145385	0.696743275	Potri.016G019500.1	AT3G48880.2
Potra2n16c29413.1	-0.499039262	0.014571151	0.054850025	0.995592415	Potri.016G019700.2	AT3G22260.2
Potra2n16c29414.1	0.116466687	0.082238074	0.435811396	0.923475785	Potri.016G019800.1	AT4G16660.1
Potra2n16c29415.1	-0.588952197	-0.229675186	0.097642141	0.930598202	Potri.016G019900.1	AT4G02750.1
Potra2n16c29416.1	-0.594878729	-0.202803844	0.058196675	0.920763566	Potri.006G022000.1	AT1G30620.1
Potra2n16c29417.1	-0.124276011	-0.085721249	0.404003094	0.917751882	Potri.016G020100.1	AT3G22270.1
Potra2n16c29418.1	-0.017999587	-0.108124986	0.942265874	0.935099709		
Potra2n16c29419.1	2.14343195	-0.767708831	NA	NA	Potri.016G020700.1	AT1G22380.1
Potra2n16c29420.1	-0.901482562	-0.088073824	0.194694791	0.989304496	Potri.016G020900.1	AT1G22380.1
Potra2n16c29421.1	-0.184686183	-0.097812678	0.823098106	0.981726976	Potri.016G020700.1	AT1G22380.1
Potra2n16c29422.1	-2.453099188	-1.169108471	NA	NA	Potri.016G020400.1	AT1G22340.1

Potra2n16c29423.1	1.468415092	0.404012757	1.57533E-39	0.259011748	Potri.016G021300.1	AT1G22400.1
Potra2n16c29424.1	-0.266418675	-0.119670072	0.267294326	0.930598202	Potri.016G020100.1	AT3G22270.1
Potra2n16c29425.1	0.591476538	0.095905115	0.000168178	0.922029226	Potri.016G021900.2	AT2G32170.1
Potra2n16c29426.2	0.827793022	-0.117247171	0.023294644	0.966407607		
Potra2n16c29427.1	-0.30225854	-1.31672144	0.900583952	NA	Potri.016G022400.1	AT1G22380.1
Potra2n16c29428.1	-1.761655447	-2.035884287	0.413303901	NA	Potri.016G022400.1	AT1G22380.1
Potra2n16c29429.1	0.727016336	-0.005333873	0.013554978	0.99905083	Potri.016G022500.1	AT1G22340.1
Potra2n16c29430.1	0.82357019	NA	NA	NA		
Potra2n16c29431.1	-1.022018902	-4.383073491	0.587854032	NA	Potri.016G022400.1	AT1G22380.1
Potra2n16c29432.1	-0.236524672	-0.085083751	0.22662612	0.946146503	Potri.016G022500.1	AT1G22340.1
Potra2n16c29433.1	0.066135371	0.240639619	0.716382347	0.679485917	Potri.016G022700.2	AT4G14965.1
Potra2n16c29434.1	-0.049212217	0.098741865	0.692007748	0.910878765	Potri.016G022800.1	AT3G22290.1
Potra2n16c29435.1	-0.132696746	-0.03193312	0.42742718	0.9761362	Potri.016G022900.1	AT5G60580.3
Potra2n16c29436.1	-0.22152101	-0.066537974	0.100899752	0.949485515	Potri.016G023000.1	AT1G50500.1
Potra2n16c29437.1	0.043711069	-0.070484175	0.764165417	0.956865545	Potri.016G023100.2	AT3G22330.1
Potra2n16c29438.1	3.362080453	2.133662373	4.11473E-90	4.1531E-48	Potri.016G023200.1	AT2G29420.1
Potra2n16c29439.1	4.40002254	3.115476861	1.57311E-28	1.27605E-14	Potri.016G023300.1	AT1G72680.1
Potra2n16c29440.1	0.673006634	0.560356499	0.747454179	NA		
Potra2n16c29441.1	0.967702342	0.195271659	0.145346193	0.964230988	Potri.016G023400.1	AT2G41050.1
Potra2n16c29442.1	-0.067074543	0.10890534	0.748214164	0.895560236	Potri.016G023500.1	AT1G67440.1
Potra2n16c29443.1	0.11634536	-0.132938101	0.437763638	0.881526761	Potri.016G023700.1	AT3G56490.1
Potra2n16c29444.1	0.266436305	0.044377975	0.001644906	0.937146435	Potri.016G023800.1	AT2G41060.2
Potra2n16c29445.1	-1.360864691	-0.091764578	0.003196475	0.98405126	Potri.016G024000.1	AT3G56850.1
Potra2n16c29446.1	0.440443961	0.326212854	0.001156885	0.498467227	Potri.016G024200.1	AT2G27040.2
Potra2n16c29447.1	0.238428246	0.083056547	0.079381217	0.895560236	Potri.016G024400.2	AT5G13750.1
Potra2n16c29448.1	-1.125525962	-0.54151478	4.34052E-06	0.323147091	Potri.016G024500.2	AT5G04200.1
Potra2n16c29449.1	-1.006181061	-0.750189896	0.009298449	0.59185656	Potri.016G024600.1	AT3G56810.1
Potra2n16c29450.1	-0.116742231	-0.166284742	0.397618609	0.791610698	Potri.016G024800.1	AT5G05080.1
Potra2n16c29451.1	-0.031469526	-0.15464298	0.873377583	0.865611082	Potri.006G026700.1	AT2G27030.3
Potra2n16c29452.1	0.908353279	0.2828899	8.62636E-05	0.802631112	Potri.016G024900.1	AT3G56950.2
Potra2n16c29453.1	-0.404199625	-0.196882123	0.089969367	0.875641095	Potri.006G027300.2	AT3G56940.1
Potra2n16c29454.1	0.050799813	0.140908512	0.860846665	0.909715408	Potri.016G025100.1	AT3G56930.1

Potra2n16c29455.1	0.454605669	0.254787058	0.057117955	0.786548546	Potri.016G025200.2	AT5G05070.1
Potra2n16c29456.3	-1.108397626	-0.118593006	6.81478E-06	0.953831244	Potri.016G025500.1	AT3G10740.1
Potra2n16c29457.1	2.013614016	0.658491994	2.92174E-22	0.372426078	Potri.016G026000.4	AT2G01570.1
Potra2n16c29458.1	-0.303514257	0.174609555	0.08317602	0.919215914	Potri.016G027600.1	AT3G56910.1
Potra2n16c29459.1	0.900292434	0.217515213	6.24063E-06	0.82876584	Potri.016G026000.4	AT2G01570.1
Potra2n16c29460.1	2.735991243	NA	NA	NA	Potri.016G027900.1	AT3G17730.1
Potra2n16c29461.1	0.043773886	-0.026501816	0.736211394	0.98036348	Potri.016G028000.1	AT5G58290.1
Potra2n16c29462.1	NA	NA	NA	NA	Potri.016G028100.1	AT5G10970.1
Potra2n16c29463.1	1.186033706	-0.024939653	NA	NA	Potri.016G028200.1	AT1G15780.1
Potra2n16c29464.1					Potri.016G028300.1	AT3G21850.1
Potra2n16c29465.4	0.796596946	-0.130813788	0.002983138	0.961703915	Potri.016G028400.2	AT5G48350.1
Potra2n16c29466.1	3.664496887	NA	0.236431843	NA	Potri.016G028500.1	
Potra2n16c29467.1	0.551570229	0.219478752	0.388591656	0.917751882	Potri.016G028800.1	
Potra2n16c29468.3	-0.34139481	0.019065282	0.009013452	0.996032145	Potri.016G028900.2	AT2G40840.1
Potra2n16c29469.1	-0.408181437	-0.168159964	0.004948208	0.84487051	Potri.016G029100.1	AT3G56590.1
Potra2n16c29470.2	-0.034897601	0.12925641	0.772617772	0.862335622	Potri.016G029200.1	AT1G65440.2
Potra2n16c29471.1	NA	NA	NA	NA	Potri.019G074700.1	AT1G71490.1
Potra2n16c29472.1					Potri.019G074700.1	AT1G71490.1
Potra2n16c29473.1	0.632311226	0.195021431	2.41015E-11	0.66992341	Potri.016G029500.1	AT3G56550.1
Potra2n16c29474.1	1.094932002	0.669206404	1.2449E-07	0.039821323	Potri.016G029600.1	AT3G56880.1
Potra2n16c29475.1	-0.225119452	-0.110978525	0.270311127	0.943483421	Potri.016G029700.3	AT5G19750.1
Potra2n16c29476.1	NA	NA	NA	NA	Potri.006G032700.1	AT5G20480.1
Potra2n16c29477.1	0.175242492	0.216200588	0.127664956	0.718440134	Potri.016G030200.1	AT1G79590.1
Potra2n16c29478.1	-0.679129079	-0.104535485	0.272000616	0.977559521	Potri.016G030300.1	AT5G45790.2
Potra2n16c29479.1	-0.07974649	-0.025175566	0.53069989	0.979073072	Potri.016G030400.2	AT3G56900.1
Potra2n16c29480.1					Potri.016G030500.1	AT5G05130.1
Potra2n16c29481.1	1.664677056	NA	NA	NA	Potri.016G030600.1	AT5G05130.1
Potra2n16c29482.1	-0.142478994	-0.118767393	0.226490256	0.819727139	Potri.016G030800.1	AT2G40860.1
Potra2n16c29483.4	-0.270114488	-0.274510155	0.032180784	0.444948931	Potri.016G030700.1	
Potra2n16c29484.1	-0.581263629	-0.204616116	0.001016735	0.824905557	Potri.016G030900.1	AT5G05110.1
Potra2n16c29485.1	0.420101647	0.048314929	0.223451178	0.983113084	Potri.016G031100.8	AT2G40890.1
Potra2n16c29486.1	0.82357019	0.674963689	NA	NA	Potri.016G031200.1	AT3G25230.1

Potra2n16c29487.1	-1.339463268	-0.226147995	0.000440428	0.977559521	Potri.016G031400.1	AT5G07050.1
Potra2n16c29488.1	1.187564631	0.470435437	0.178097849	0.928154618	Potri.016G031500.1	AT1G68620.1
Potra2n16c29489.1	-1.479249936	0.193949983	0.000815808	0.940361195	Potri.016G031600.1	AT3G11980.1
Potra2n16c29490.1	-1.533228142	-0.675621391	0.037226327	0.815348505	Potri.016G031700.1	AT3G56630.1
Potra2n16c29491.1	-1.209420299	-0.515405289	8.04166E-05	0.348667924	Potri.016G031700.1	AT3G56630.1
Potra2n16c29492.1	-0.268049041	-0.188030004	0.220732399	0.881090429		
Potra2n16c29493.1	-0.427966831	-0.162240199	2.5473E-06	0.750123244	Potri.016G031900.1	AT2G40930.1
Potra2n16c29494.1	-1.112530947	-0.363826109	1.40122E-07	0.70570456	Potri.016G032000.1	AT1G68570.1
Potra2n16c29495.1	-0.342867895	-0.256284801	0.021019117	0.693793454	Potri.016G032100.1	AT3G56640.1
Potra2n16c29496.1	1.232676548	0.686391015	0.220627591	0.91998377	Potri.T039450.1	AT1G27180.1
Potra2n16c29497.1	1.05781406	0.396398161	1.26388E-17	0.498594799	Potri.016G032400.1	AT2G40950.1
Potra2n16c29498.3	-0.158896822	0.247449057	0.515462086	0.873823526	Potri.016G032500.2	AT3G56680.1
Potra2n16c29499.1	-0.776461798	-0.35891941	1.14987E-07	0.360126886	Potri.016G032600.1	AT3G10760.1
Potra2n16c29500.2	2.632524198	1.571529889	NA	NA	Potri.006G035000.1	AT3G56690.1
Potra2n16c29501.1	NA	NA	NA	NA	Potri.016G032300.1	AT2G38560.1
Potra2n16c29502.1	4.688513963	1.613176996	5.52781E-08	0.824378527	Potri.016G032900.1	AT1G14040.1
Potra2n16c29503.1	-0.35162938	0.033382987	0.06455844	0.983446961	Potri.016G033000.1	AT5G06150.1
Potra2n16c29504.1	-0.818643662	-0.224135499	1.5316E-07	0.821709751	Potri.016G033100.2	AT2G40820.1
Potra2n16c29505.1	-0.176477603	-0.345925487	0.424343221	0.759614928	Potri.016G033200.1	AT5G12250.1
Potra2n16c29506.1	0.867286527	0.210144196	1.26317E-05	0.754009738	Potri.016G033300.1	AT5G05140.1
Potra2n16c29507.1					Potri.016G033400.1	
Potra2n16c29508.1	1.502861306	1.571529889	NA	NA	Potri.016G033600.1	AT5G05130.1
Potra2n16c29509.1	3.008685987	2.0518966	0.000133096	0.534184071	Potri.016G034000.1	AT5G05130.1
Potra2n16c29510.1	0.627146018	0.00347539	0.104867207	0.999325699	Potri.016G034100.1	AT3G52870.1
Potra2n16c29511.2	0.204408364	0.065761412	0.210906238	0.948834049	Potri.016G034200.1	AT4G09550.1
Potra2n16c29512.1					Potri.016G034300.1	AT1G28110.2
Potra2n16c29513.1	-1.266538946	-0.942208482	0.195875067	0.784063198	Potri.016G034600.1	AT2G33530.1
Potra2n16c29514.1	-0.046573484	-2.346476039	0.985522878	NA	Potri.016G034800.1	AT1G66120.1
Potra2n16c29515.1	-0.595952689	-1.465929376	NA	NA		
Potra2n16c29516.1	0.069335595	0.210968406	0.787742143	0.819475509		
Potra2n16c29517.1	-0.22635278	-0.577177468	0.444191507	0.381829224	Potri.016G034950.1	
Potra2n16c29518.1	-0.797823827	-0.422133517	0.664682657	0.950878725		

Potra2n16c29519.1	-3.163868819	-0.593433392	0.166841215	NA	Potri.016G035100.1	AT2G26800.2
Potra2n16c29520.1	0.58564469	0.049531916	0.000198378	0.954476684	Potri.016G035200.1	AT3G55290.1
Potra2n16c29521.1	-1.176651283	-0.560844523	8.57579E-06	0.673884433	Potri.016G035300.1	AT1G73590.1
Potra2n16c29522.1	0.060647088	0.056096964	0.911504758	0.985393296	Potri.016G035400.1	AT2G41130.1
Potra2n16c29523.1	2.549191961	3.158770786	NA	NA		
Potra2n16c29524.1	0.284065488	0.279422082	0.010935329	0.691680177	Potri.016G035500.1	AT2G41140.1
Potra2n16c29525.1	0.218668795	0.167681774	0.160764617	0.910878765		
Potra2n16c29526.1	-0.712696191	-0.427431293	0.000394739	0.563956825	Potri.016G035600.1	AT3G56750.1
Potra2n16c29527.1	-0.236981248	-0.190429127	0.183239465	0.90891625	Potri.016G035700.1	AT4G18593.1
Potra2n16c29528.1	-0.009231046	0.078531022	0.961890434	0.917516356	Potri.016G035800.4	AT2G41160.1
Potra2n16c29529.1	-0.334891721	-0.078460672	0.437365053	0.974275337	Potri.016G035900.1	AT2G32560.1
Potra2n16c29530.1	-0.492979854	0.023400811	0.044516082	0.99114586	Potri.016G036000.2	AT3G56720.2
Potra2n16c29531.1	0.426907705	-0.073798647	0.021062108	0.972159498	Potri.016G036100.1	AT1G80270.2
Potra2n16c29532.1	0.119854278	-0.151338894	0.512700046	0.89257034	Potri.006G039400.1	AT5G04130.1
Potra2n16c29533.1	0.608430668	0.23950144	0.014586448	0.907032966	Potri.016G036300.1	AT1G68060.1
Potra2n16c29534.2	-0.393115973	-0.026904089	0.266608039	0.992934677	Potri.016G036400.3	AT5G09225.1
Potra2n16c29535.1	0.612211402	0.213547794	2.94256E-05	0.511337291	Potri.016G036500.1	AT3G12250.2
Potra2n16c29536.1	1.058156923	0.129518057	0.000570878	0.966501596	Potri.016G036600.1	AT3G56710.1
Potra2n16c29537.1	-1.407635536	-0.980740881	0.009965063	0.517463209	Potri.016G036700.2	AT3G12600.1
Potra2n16c29538.1	NA	NA	NA	NA	Potri.016G036800.1	AT3G56960.1
Potra2n16c29539.1	0.028931107	0.216747277	0.875146962	0.862143562	Potri.016G036900.11	AT5G04140.1
Potra2n16c29540.1	-0.011925392	0.117238371	0.961257187	0.948914372		
Potra2n16c29541.1	6.437942128	1.054749676	1.35333E-05	NA		
Potra2n16c29542.1	6.367615937	-0.6707161	6.51255E-09	NA		
Potra2n16c29543.1	5.002373302	0.568788329	1.42395E-08	NA		
Potra2n16c29544.1	0.237024145	0.185283113	0.664316293	0.959916003	Potri.016G037300.1	AT3G56970.1
Potra2n16c29545.1	0.420648766	0.170353105	0.016765397	0.889979251	Potri.016G037400.1	AT3G56990.1
Potra2n16c29546.1	0.003766783	-0.137600887	0.986142589	0.921099436	Potri.016G037500.1	AT3G57000.1
Potra2n16c29547.1	0.231145037	0.048534808	0.292598867	0.982422288		
Potra2n16c29548.1	-0.710322598	0.684623072	0.001337091	NA	Potri.016G037600.1	AT2G41250.1
Potra2n16c29549.1	-1.35927387	-0.066852884	NA	NA	Potri.016G037700.2	AT2G41290.1
Potra2n16c29550.1	0.143327337	0.0091492	0.333197356	0.994692557	Potri.016G037900.1	AT3G57030.1

Potra2n16c29551.1	4.273672025	2.167174679	2.7187E-05	NA		
Potra2n16c29552.1	0.150259527	0.334024869	0.744933253	0.902676973	Potri.016G038000.1	AT3G57040.1
Potra2n16c29553.1	-0.684883054	-0.097865349	3.45378E-08	0.901671593	Potri.016G038100.11	AT3G10250.1
Potra2n16c29554.1	0.1298765	-0.003935563	0.33112524	0.99819408	Potri.016G038200.1	AT3G57050.1
Potra2n16c29555.1	-0.399073879	-0.088966573	0.122012424	0.953768643	Potri.016G038300.1	AT3G57062.1
Potra2n16c29556.1	-0.340696172	-0.083872668	0.189871667	0.948834049		
Potra2n16c29557.3	-0.363902788	-0.892486764	0.39365269	0.497568278	Potri.016G038400.2	AT2G35030.1
Potra2n16c29558.1	-0.191686242	0.040074182	0.27570504	0.969421568	Potri.016G038500.1	AT3G57080.1
Potra2n16c29559.1	NA	4.349748739	NA	NA		
Potra2n16c29560.1	-0.775355026	-0.061912968	0.252476902	0.984910847	Potri.016G038600.1	AT3G57070.1
Potra2n16c29561.1	-0.140315908	0.172594752	0.94938677	NA		
Potra2n16c29562.1	0.416611009	0.215126515	0.004467974	0.720242695	Potri.016G038900.1	AT3G57090.1
Potra2n16c29563.1	1.517671607	1.89838425	NA	NA	Potri.016G039000.1	AT1G16390.1
Potra2n16c29564.1	0.505702036	0.366301995	0.76631382	NA	Potri.T164000.1	AT3G50150.1
Potra2n16c29565.1	NA	NA	NA	NA	Potri.T163900.1	AT2G41380.1
Potra2n16c29566.1	0.82357019	2.141662216	NA	NA	Potri.016G039100.1	AT3G50120.1
Potra2n16c29567.1	0.501625279	2.157645448	NA	NA	Potri.T163900.1	AT2G41380.1
Potra2n16c29568.1	1.555091481	2.442519496	NA	NA	Potri.T164000.1	AT3G50150.1
Potra2n16c29569.1	0.945760063	0.429894569	0.566384981	0.954419252	Potri.T163900.1	AT2G41380.1
Potra2n16c29570.1	1.305531382	0.963620196	0.455460106	NA	Potri.016G039000.1	AT1G16390.1
Potra2n16c29571.1	NA	3.100071756	NA	NA	Potri.T164000.1	AT3G50150.1
Potra2n16c29572.1	NA	NA	NA	NA	Potri.T164000.1	AT3G50150.1
Potra2n16c29573.1	0.166936889	1.574487856	NA	NA	Potri.T163900.1	AT2G41380.1
Potra2n16c29574.1	3.618923717	1.470125574	1.02724E-07	0.817452127	Potri.T164000.1	AT3G50150.1
Potra2n16c29575.1	NA	NA	NA	NA		
Potra2n16c29576.1	2.38822379	1.947915864	0.270436674	NA	Potri.T164000.1	AT3G50150.1
Potra2n16c29577.1	-0.668767129	0.501728049	0.716360116	0.932447954	Potri.T163900.1	AT2G41380.1
Potra2n16c29578.1	1.632160916	0.392186247	0.187753078	NA	Potri.016G039000.1	AT1G16390.1
Potra2n16c29579.1	0.167384983	-0.402934315	0.921679731	NA	Potri.T164000.1	AT3G50150.1
Potra2n16c29580.1	NA	NA	NA	NA	Potri.T163900.1	AT2G41380.1
Potra2n16c29581.1	NA	NA	NA	NA	Potri.016G039050.1	AT2G41380.1
Potra2n16c29582.1	NA	NA	NA	NA	Potri.T163900.1	AT2G41380.1

Potra2n16c29583.1	0.614550672	0.708696851	0.023133085	0.27066389	Potri.T164000.1	AT3G50150.1
Potra2n16c29584.1	-1.09536862	0.802861775	NA	NA	Potri.016G039500.1	AT3G50150.1
Potra2n16c29585.1	2.632524198	NA	NA	NA	Potri.016G039500.1	AT3G50150.1
Potra2n16c29586.1	0.10695453	0.665987675	0.953877878	NA	Potri.016G039700.2	AT3G50120.1
Potra2n16c29587.1					Potri.016G040100.1	AT3G50150.1
Potra2n16c29588.1	0.265263692	0.568129789	0.452077862	0.268264683	Potri.016G039700.2	AT3G50120.1
Potra2n16c29589.1	-1.03340488	-0.055405426	0.211890434	0.992934677	Potri.016G040100.1	AT3G50150.1
Potra2n16c29590.1	-0.335156534	0.223249606	0.040804484	0.833651746		
Potra2n16c29591.2	-0.189681102	-0.106648797	0.305523798	0.922029226	Potri.016G040300.1	AT3G47930.1
Potra2n16c29592.1	-0.525207029	0.05606153	0.039844135	0.967129771	Potri.016G040500.1	AT2G41370.1
Potra2n16c29593.4	-1.721272218	-0.249703587	0.050262818	0.968979184	Potri.006G043600.1	AT3G10200.1
Potra2n16c29594.1	1.664677056	NA	NA	NA	Potri.T164000.1	AT3G50150.1
Potra2n16c29595.1	0.186242356	1.479626436	NA	NA		
Potra2n16c29596.1	1.461576302	0.46318034	1.53491E-15	0.728206951		
Potra2n16c29597.1	0.190501413	0.111715486	0.396708985	0.902857376	Potri.016G041000.3	AT5G04040.1
Potra2n16c29598.1	1.431011624	0.861614073	0.00056718	0.55420251		
Potra2n16c29599.1	1.368038316	0.679018587	8.44063E-18	0.112106631	Potri.016G041100.1	AT5G04020.1
Potra2n16c29600.1	0.490190372	0.015350865	0.000600281	0.991489035	Potri.016G041200.1	AT3G57150.1
Potra2n16c29601.1	-0.732988282	-0.025219392	0.565437769	0.99812851	Potri.016G041301.1	
Potra2n16c29602.1	-0.325204291	-0.107174957	0.007421267	0.941712288	Potri.016G041600.1	AT2G41430.1
Potra2n16c29603.1	-0.499692743	-0.179928532	0.000622248	0.857189843	Potri.006G045400.1	AT3G10160.1
Potra2n16c29604.1	-0.314751781	0.092503682	0.023938985	0.949966783	Potri.016G041900.1	AT3G57200.1
Potra2n16c29605.1	0.423303583	0.093473105	0.000527455	0.916403589	Potri.016G042000.1	AT2G41460.1
Potra2n16c29606.1	2.040396587	0.215570264	2.03282E-12	0.939915707	Potri.006G045800.1	AT2G41500.1
Potra2n16c29607.1	-0.251338393	0.076693454	0.267215417	0.956865545	Potri.016G042400.1	AT5G03990.1
Potra2n16c29608.1	-1.074792204	0.096505139	2.06704E-05	0.959881296	Potri.016G042500.1	AT3G10150.1
Potra2n16c29609.1	-1.210350089	0.108450644	6.17109E-07	0.957877578	Potri.016G042500.1	AT3G10150.1
Potra2n16c29610.2	1.545832463	NA	NA	NA	Potri.016G042700.2	AT5G03960.1
Potra2n16c29611.1					Potri.016G042800.1	AT3G57240.1
Potra2n16c29612.1	-0.198334535	-0.688504146	0.857601345	0.828625141	Potri.016G042900.1	AT4G35270.1
Potra2n16c29613.1	-1.343512567	-0.771232352	NA	NA	Potri.006G046100.1	AT3G57270.1
Potra2n16c29614.1	-0.631024652	0.317326041	0.015961494	0.829597923	Potri.016G043100.1	AT2G41560.1

Potra2n16c29615.1	0.142711563	0.381760768	0.790495907	0.859892315	Potri.016G043200.1	AT5G04160.1
Potra2n16c29616.1	-0.49038476	-0.490528307	0.553983731	0.857189843	Potri.016G043300.10	AT2G41550.1
Potra2n16c29617.1	-0.359214303	0.157183959	0.624268093	0.974275337	Potri.016G043400.1	AT2G41540.1
Potra2n16c29618.1	-0.056530665	0.144412463	0.728010138	0.857189843	Potri.016G043500.1	AT3G57300.1
Potra2n16c29619.1						
Potra2n16c29620.1	1.394692619	0.349840622	1.30757E-11	0.733263164	Potri.016G043600.1	AT3G10300.3
Potra2n16c29621.1	-0.421966659	-0.379858215	0.018608632	0.677589151	Potri.016G043700.1	AT4G20990.1
Potra2n16c29622.1	0.376600592	0.04976394	0.001955077	0.959881296	Potri.016G043800.1	AT3G57290.1
Potra2n16c29623.2	-0.266893152	-0.061696099	0.068429321	0.956865545	Potri.016G043900.1	AT2G41520.1
Potra2n16c29624.1	-1.52163005	-0.367509663	0.000308518	0.824274341	Potri.016G044000.1	AT5G04000.2
Potra2n16c29625.1	-0.878152276	0.378791184	0.063949399	0.924104906	Potri.016G044100.1	AT2G41510.1
Potra2n16c29626.1	2.079222692	1.063577884	0.003126226	0.753288535	Potri.016G047200.1	AT5G22355.1
Potra2n16c29627.1	1.531525789	0.516639089	7.8828E-10	0.763747409	Potri.016G044600.2	AT5G22355.1
Potra2n16c29628.1					Potri.019G027600.1	AT1G21280.1
Potra2n16c29629.1	0.8609465	-0.270099566	0.599673248	NA	Potri.T110600.1	AT5G22355.1
Potra2n16c29630.1	0.85958147	0.071557387	0.02553694	0.983446961	Potri.016G044600.2	AT5G22355.1
Potra2n16c29631.1	1.497854307	0.248558522	0.000356235	0.937846405	Potri.T110400.1	AT5G22355.1
Potra2n16c29632.1	1.095289373	0.38576951	1.23824E-07	0.755355354	Potri.016G044732.1	AT5G22355.1
Potra2n16c29633.1	1.452947016	0.45212026	0.000842364	0.854980698	Potri.016G044600.2	AT5G22355.1
Potra2n16c29634.1	0.329053641	-0.34405623	0.339994014	0.849815276	Potri.016G044732.1	AT5G22355.1
Potra2n16c29635.1	0.788417693	0.066775937	0.012872579	0.978507994	Potri.016G044800.1	AT5G22355.1
Potra2n16c29636.1	0.469907797	0.117729022	0.201811813	0.966501596	Potri.016G044732.1	AT5G22355.1
Potra2n16c29637.1	0.599278261	0.075064989	0.000218418	0.943819961	Potri.016G045200.1	AT3G12100.1
Potra2n16c29638.1	-0.632489753	-0.298065935	0.000328203	0.716582974	Potri.016G045300.1	AT2G42070.1
Potra2n16c29639.1	0.278956638	0.027180836	0.157524928	0.986636967	Potri.016G045400.1	AT2G42080.1
Potra2n16c29640.1	-1.8800902	-0.455149603	1.06386E-12	0.718932208	Potri.006G192700.1	AT3G12110.1
Potra2n16c29641.1	0.1019738	-0.010961483	0.481041793	0.994861477	Potri.016G045600.1	AT5G06750.3
Potra2n16c29642.1	0.397549101	-0.131116074	0.566329565	0.962696825	Potri.016G045800.10	AT2G42120.2
Potra2n16c29643.1	-0.166653412	0.003870689	0.460639764	0.99702095	Potri.016G045900.1	AT2G42130.4
Potra2n16c29644.1	0.849818931	-0.393565044	0.704791879	NA	Potri.016G046000.2	AT3G58000.1
Potra2n16c29645.1	0.048407531	-0.301649566	0.896181046	0.787760802	Potri.016G046200.1	AT3G12120.1
Potra2n16c29646.1	-0.314819584	-0.032847959	0.570998327	0.995579852	Potri.T111300.1	AT5G06760.1

Potra2n16c29647.2	NA	1.571529889	NA	NA	Potri.T111200.1	AT1G24020.1
Potra2n16c29648.2	-0.100996738	0.131155564	0.601521168	0.913622521	Potri.016G046600.1	AT5G06770.1
Potra2n16c29649.1	0.15430235	2.195509882	0.653690127	0.284918677	Potri.T111000.1	AT2G05990.2
Potra2n16c29650.1	0.167790771	0.0516393	0.178681386	0.953831244	Potri.T110900.1	AT1G51130.1
Potra2n16c29651.1	-0.112858329	-0.053558426	0.643119668	0.975396961	Potri.T110800.1	AT3G57990.1
Potra2n16c29652.1	1.800764917	0.730487734	0.000233366	0.815348505	Potri.T110600.1	AT5G22355.1
Potra2n16c29653.1	1.189227275	0.514620279	0.025959756	0.869186333	Potri.T110400.1	AT5G22355.1
Potra2n16c29654.1	-0.166449684	-0.24985458	0.645052276	0.876272842	Potri.T110800.1	AT3G57990.1
Potra2n16c29655.1	1.873586457	0.806350802	4.22048E-08	0.530383957	Potri.016G044732.1	AT5G22355.1
Potra2n16c29656.1	-0.069326933	-1.738398046	0.972502471	NA	Potri.016G047200.1	AT5G22355.1
Potra2n16c29657.1	1.602037142	0.458542452	1.43916E-05	0.875292974	Potri.T110600.1	AT5G22355.1
Potra2n16c29658.1	1.321537177	0.134181226	0.000121982	0.967129771	Potri.016G044600.2	AT5G22355.1
Potra2n16c29659.1	1.145854561	0.279136528	6.91717E-06	0.886290594	Potri.016G044600.2	AT5G22355.1
Potra2n16c29660.1	1.03582046	0.096703038	3.55315E-06	0.953608209	Potri.016G044732.1	AT5G22355.1
Potra2n16c29661.1	1.275552161	0.104554028	0.026753618	0.98409016	Potri.T110600.1	AT5G22355.1
Potra2n16c29662.1	-0.365798607	-0.0784858	0.322938266	0.976888173	Potri.016G046900.1	AT3G57990.1
Potra2n16c29663.1	1.104458323	0.30139748	0.000179769	0.862335622	Potri.016G044600.2	AT5G22355.1
Potra2n16c29664.1	-0.369272798	-0.483349321	0.20274193	0.284360673	Potri.006G191500.5	AT1G20970.1
Potra2n16c29665.1	-1.19112816	0.309791158	0.474192086	0.953768643		
Potra2n16c29666.1	-0.040694467	0.019949978	0.737976079	0.985393296	Potri.016G047600.1	AT5G13020.1
Potra2n16c29667.1	NA	NA	NA	NA	Potri.016G047700.1	
Potra2n16c29668.1	-1.456668402	0.210106016	0.251986408	0.98177802	Potri.016G047800.1	AT5G06790.1
Potra2n16c29669.1						
Potra2n16c29670.1	-2.503131731	-0.544984766	8.36364E-09	0.49278277	Potri.016G047900.1	AT5G06800.1
Potra2n16c29671.1	0.158743123	0.605519418	0.735492993	0.550725487	Potri.016G048000.1	AT5G06800.1
Potra2n16c29672.1	0.067064071	-0.053547602	0.686938854	0.966501596	Potri.016G048100.1	AT3G15010.1
Potra2n16c29673.1	0.048329119	0.223186602	0.822754147	0.890446513	Potri.016G048200.2	AT5G06810.1
Potra2n16c29674.1	-0.086268658	0.215319304	0.754076017	0.792896411	Potri.016G048300.1	AT5G06810.1
Potra2n16c29675.1	-1.092807371	-0.189489796	0.000131537	0.833667976	Potri.016G048500.1	AT2G42200.1
Potra2n16c29676.1	-0.593608393	-0.041582626	7.12245E-08	0.974275337	Potri.016G048600.1	AT3G57910.1
Potra2n16c29677.2	-0.848973108	-0.065653906	6.26526E-05	0.966917036	Potri.016G048800.1	AT5G50590.1
Potra2n16c29678.2	0.169105784	-0.006528082	0.027670099	0.993589787	Potri.016G049000.1	AT3G57890.1

Potra2n16c29679.1	-1.350762842	-0.479672965	5.01999E-08	0.786548546	Potri.016G049100.1	AT3G57880.1
Potra2n16c29680.1	0.920612179	0.122379436	0.332727573	0.983446961	Potri.016G049200.2	AT5G06839.3
Potra2n16c29681.1	0.058282486	-0.343135194	0.923670701	0.901195443		
Potra2n16c29682.1	1.628167388	-0.369681937	0.158769238	NA	Potri.006G058700.1	AT5G06850.1
Potra2n16c29683.1					Potri.016G049400.1	AT5G06860.1
Potra2n16c29684.1	1.664677056	NA	NA	NA	Potri.016G049500.1	AT4G14480.1
Potra2n16c29685.1	0.990496985	-0.275506965	0.022944296	0.922011871	Potri.016G049600.1	AT5G06860.1
Potra2n16c29686.1	-0.518397556	-0.288246892	0.010461808	0.720242695	Potri.016G049700.1	AT3G12150.1
Potra2n16c29687.3	-1.257107485	-0.976004111	0.146426098	0.775830532	Potri.016G049800.1	AT5G06900.1
Potra2n16c29688.1	NA	NA	NA	NA	Potri.016G050200.1	AT5G06905.1
Potra2n16c29689.1	0.480205523	-0.215793552	0.529222728	0.959881296	Potri.016G050300.1	AT2G42260.1
Potra2n16c29690.1	-1.466960632	0.607206691	0.260617086	0.900869139	Potri.016G050400.1	AT3G12160.1
Potra2n16c29691.1	-0.797231519	-0.170583786	0.000182722	0.899990252	Potri.016G050500.1	AT2G42280.1
Potra2n16c29692.1	-1.853319862	-0.467221724	5.74591E-05	0.540010302	Potri.016G050800.1	AT3G57830.1
Potra2n16c29693.1	-0.137064289	0.016568583	0.177600169	0.986636967	Potri.016G050900.1	AT3G57810.3
Potra2n16c29694.2	0.054673817	0.147074149	0.835817481	0.903203316	Potri.016G051000.1	AT3G12180.1
Potra2n16c29695.1	-0.264976095	-0.074417413	0.332756586	0.972159498	Potri.016G051200.1	AT3G57790.1
Potra2n16c29696.1	-1.134193141	-0.754335735	0.000125564	0.097521997		
Potra2n16c29697.1	-1.131672728	-0.751813025	0.000131199	0.099730279	Potri.016G051300.1	AT3G13770.1
Potra2n16c29698.1	-0.133807069	-0.277981998	0.522502618	0.69318689	Potri.016G051400.1	AT2G42310.1
Potra2n16c29699.4	-1.259879894	-0.652102473	4.29704E-07	0.451062617	Potri.016G051500.1	AT2G42320.1
Potra2n16c29700.1	-0.889231088	-0.41949361	0.000253397	0.836066102	Potri.016G051600.1	AT5G06940.1
Potra2n16c29701.1	-0.320032326	-0.219316304	0.017994656	0.753615154	Potri.016G051700.1	AT1G51200.4
Potra2n16c29702.1	-0.220787555	0.004218321	0.13785489	0.998136564	Potri.016G051800.1	AT3G12170.1
Potra2n16c29703.1	-0.630685362	-0.142675125	1.26373E-05	0.903698951	Potri.016G051900.1	AT3G44200.1
Potra2n16c29704.1	-0.430297229	0.005938359	0.152156967	0.99819408	Potri.016G052000.1	AT2G41905.1
Potra2n16c29705.1	-1.137986349	-0.460886116	4.48345E-09	0.389989789	Potri.016G052100.1	
Potra2n16c29706.1	-0.570932065	-1.318856066	0.46743338	0.409505482		
Potra2n16c29707.1	1.244395674	-0.8484639	7.73791E-05	0.816392132	Potri.016G052500.1	AT4G15550.1
Potra2n16c29708.1	-0.61910233	0.666960761	NA	NA	Potri.016G052600.1	AT2G42250.1
Potra2n16c29709.1	-0.426479088	0.126803148	0.362653988	0.966917036	Potri.016G052700.1	AT3G57670.1
Potra2n16c29710.1	-0.182295372	-0.052604548	0.10915093	0.953831244	Potri.016G052800.1	AT3G18140.1

Potra2n16c29711.1	-0.185640151	-0.047805968	0.906996136	0.996032145	Potri.016G052901.1	
Potra2n16c29712.1	-0.514298133	0.02522793	3.79176E-05	0.97950444	Potri.016G053000.2	AT3G57650.1
Potra2n16c29713.1	NA	NA	NA	NA	Potri.016G053100.1	AT2G03270.1
Potra2n16c29714.1	NA	NA	NA	NA	Potri.016G053200.1	AT3G57600.1
Potra2n16c29715.1	-0.33661984	0.027676624	0.00344232	0.983113084	Potri.016G053300.1	AT1G68030.1
Potra2n16c29716.1	-0.212673644	-0.250355915	0.42719378	0.8693465	Potri.016G053400.1	AT3G52105.1
Potra2n16c29717.1	-0.01083528	0.066405705	0.969528785	0.973347974	Potri.016G053500.1	AT5G52630.1
Potra2n16c29718.1	3.409482019	1.548190756	5.47181E-22	0.019762277	Potri.016G053600.1	AT1G51340.2
Potra2n16c29719.1	0.315721174	0.350634444	0.122062901	0.729131591	Potri.016G053700.1	AT2G41900.1
Potra2n16c29720.1	0.109997866	-0.109415349	0.746099378	0.95671968	Potri.016G053800.1	AT2G41890.1
Potra2n16c29721.1	-1.205973266	-0.393295277	0.001827452	0.808819946	Potri.016G053900.2	AT3G20898.1
Potra2n16c29722.1	-1.032332304	-1.288360086	0.613896952	NA		
Potra2n16c29723.1	-0.80947053	-0.323683952	0.000470078	0.812696706	Potri.016G054200.1	AT3G57550.2
Potra2n16c29724.1	1.545832463	1.394613675	NA	NA		
Potra2n16c29725.1	1.499941259	1.39745657	7.49904E-11	0.241968662	Potri.016G054400.1	AT3G57540.1
Potra2n16c29726.1	NA	NA	NA	NA	Potri.016G054500.1	AT5G13650.2
Potra2n16c29727.1	-0.183962446	0.114833022	0.216322929	0.903477395	Potri.016G054600.3	AT3G57530.1
Potra2n16c29728.1	-0.001007224	0.051176855	0.996526651	0.980339415	Potri.016G054700.10	AT3G57520.1
Potra2n16c29729.1	-1.578609879	0.129890138	0.033400666	0.954419252	Potri.016G054800.1	AT2G41850.1
Potra2n16c29730.1	0.22643618	-0.129779495	0.189848833	0.933286951	Potri.016G054900.4	AT5G05170.1
Potra2n16c29731.1	0.758927049	0.124020522	1.21985E-06	0.917751882	Potri.016G055000.1	AT2G41840.1
Potra2n16c29732.2	-0.389925889	-0.105755543	0.020589942	0.923478213	Potri.016G055100.4	AT2G41830.1
Potra2n16c29733.1	0.110896228	0.074001101	0.53069989	0.946871883	Potri.016G055200.1	AT1G01170.2
Potra2n16c29734.1	0.434393493	0.213254381	0.014397066	0.759932222	Potri.016G055300.1	AT2G34690.1
Potra2n16c29735.2	NA	NA	NA	NA	Potri.017G144200.1	AT3G14470.1
Potra2n16c29736.1	-0.765420311	-0.223529374	0.022021181	0.912159857	Potri.016G055400.1	AT2G41820.1
Potra2n16c29737.1	-3.916117665	-2.624272642	0.061389013	NA	Potri.016G055500.1	AT1G61110.1
Potra2n16c29738.1	-0.498938424	-0.265128763	0.012603807	0.864854639	Potri.006G051100.1	AT5G13630.1
Potra2n16c29739.1	-0.439900639	-0.244232985	0.070181205	0.902857376	Potri.016G055700.1	AT2G41770.1
Potra2n16c29740.1	-0.484245472	-0.192229369	0.07205724	0.937846405	Potri.016G055700.1	AT2G41770.1
Potra2n16c29741.1	-1.27723205	-0.262213345	0.537982287	NA	Potri.016G055800.1	
Potra2n16c29742.1	1.221397895	-0.047175948	0.246010281	0.996032145	Potri.016G055901.1	AT3G57450.1

Potra2n16c29743.5	0.173297418	-1.709396419	0.878624261	NA	Potri.009G058600.1	AT5G13710.2
Potra2n16c29744.1	4.672902319	2.644355809	1.16781E-25	0.372363623	Potri.016G056100.1	AT2G41810.1
Potra2n16c29745.1	-0.227233908	0.091676347	0.002441982	0.910477984	Potri.006G050200.4	AT2G41740.1
Potra2n16c29746.1	2.441362694	0.271599715	4.62307E-09	0.895474078	Potri.006G050100.1	AT3G57400.1
Potra2n16c29747.1	1.545832463	NA	NA	NA	Potri.006G049900.1	AT2G41720.1
Potra2n16c29748.3	-0.295547418	0.209463198	0.116200867	0.797111689	Potri.016G056400.2	AT2G41710.1
Potra2n16c29749.1	0.748592914	0.030928191	0.000198378	0.989304496	Potri.016G056500.1	AT2G41690.1
Potra2n16c29750.1	0.82357019	NA	NA	NA	Potri.016G056600.1	AT3G21090.1
Potra2n16c29751.1	NA	1.571529889	NA	NA	Potri.016G056700.4	AT3G21090.1
Potra2n16c29752.2	NA	NA	NA	NA	Potri.016G056700.4	AT3G21090.1
Potra2n16c29753.1	-2.850900539	-0.689272993	1.57886E-08	0.630775933	Potri.016G056800.1	AT2G41660.1
Potra2n16c29754.1	1.545832463	1.381953902	NA	NA		
Potra2n16c29755.1					Potri.016G056900.1	
Potra2n16c29756.1	1.567398001	-0.651506661	0.439017961	NA	Potri.016G057000.1	AT2G41640.1
Potra2n16c29757.1	0.184589205	0.059957557	0.16584141	0.941260209	Potri.016G057100.1	AT3G10330.1
Potra2n16c29758.1	4.465622987	0.737682254	0.000634723	NA	Potri.016G057300.1	AT3G11340.1
Potra2n16c29759.1	5.93716781	2.699416703	4.20558E-23	0.226817729	Potri.016G057400.1	AT3G57270.1
Potra2n16c29760.1	6.910536324	3.032922238	1.20209E-10	0.151351582	Potri.016G057600.1	AT3G57270.1
Potra2n16c29761.1	-0.254298844	-0.034245189	0.077782325	0.978646026	Potri.016G057700.1	AT3G10140.1
Potra2n16c29762.1	1.099942764	-0.209074776	9.1954E-06	0.921099436	Potri.016G057800.1	AT5G45730.1
Potra2n16c29763.1	NA	NA	NA	NA	Potri.016G057900.1	AT2G27660.1
Potra2n16c29764.1	-0.049996566	-0.201100275	0.865687926	0.864223715	Potri.016G058000.1	AT2G42040.1
Potra2n16c29765.1	2.063471427	0.674963689	NA	NA	Potri.016G058100.1	AT2G42040.1
Potra2n16c29766.1						
Potra2n16c29767.1	4.321840838	1.075573963	4.85555E-20	0.842469223	Potri.016G058200.1	AT5G06720.1
Potra2n16c29768.1	-0.001934619	0.003075112	0.991063565	0.997932292	Potri.016G058300.1	AT3G58030.3
Potra2n16c29769.1	-1.247425028	-0.367431379	8.75228E-06	0.784845657	Potri.016G059000.1	AT5G06710.1
Potra2n16c29770.1	2.429302502	-1.126135538	1.2202E-06	0.799601397	Potri.016G058700.1	AT4G38180.1
Potra2n16c29771.1	1.8163407	0.809537783	2.24093E-12	0.3020127	Potri.016G058700.1	AT4G38180.1
Potra2n16c29772.1	0.447393015	0.093023881	0.010991097	0.915339763	Potri.016G059300.3	AT5G06700.1
Potra2n16c29773.1	0.479394644	-0.48524976	0.63733663	0.924951347	Potri.016G059400.2	AT2G42040.1
Potra2n16c29774.1	-1.375004874	-0.137986932	4.89762E-08	0.921015963	Potri.016G059500.3	AT5G06690.2

Potra2n16c29775.1	-1.314446099	-0.254752091	0.000150102	0.834610342	Potri.016G059600.1	AT2G41970.1
Potra2n16c29776.1	0.377391978	0.546513386	0.123875923	0.770228985	Potri.016G059700.1	AT3G58040.1
Potra2n16c29777.1	-0.914266596	-0.166199056	0.219622632	0.966501596	Potri.008G096800.1	AT1G26370.1
Potra2n16c29778.1	0.044216651	0.066232431	0.755611214	0.930059926	Potri.016G059900.1	AT5G06680.1
Potra2n16c29779.1	0.120217315	0.057891345	0.294893144	0.935099709	Potri.016G060000.1	AT3G12040.1
Potra2n16c29780.1	0.09186258	0.048144718	0.640837297	0.960981446	Potri.016G060100.1	AT3G12050.1
Potra2n16c29781.3	0.140280827	0.21002267	0.222245784	0.771626158	Potri.016G060200.2	AT3G58050.1
Potra2n16c29782.1	-0.13144784	-0.131601076	0.424446745	0.863022165	Potri.016G060300.1	AT5G06660.1
Potra2n16c29783.1	-0.024084259	-0.049863514	0.900978295	0.954419252	Potri.016G060400.1	AT3G12020.2
Potra2n16c29784.4	0.166142559	-0.02587309	0.146534617	0.98177802	Potri.016G060500.1	AT3G12010.1
Potra2n16c29785.1						
Potra2n16c29786.1	-0.61910233	0.64247635	NA	NA	Potri.016G060600.1	AT2G41940.1
Potra2n16c29787.1	-1.460517595	-0.954659589	0.018666522	0.776094736	Potri.016G060900.1	AT1G58170.1
Potra2n16c29788.1	-0.268454754	-0.185341021	0.567410898	0.969237046	Potri.016G061000.1	AT1G58170.1
Potra2n16c29789.1	1.096113666	0.185873901	1.12975E-09	0.915354501	Potri.016G061100.1	AT1G57860.1
Potra2n16c29790.1	NA	NA	NA	NA	Potri.016G061300.1	AT3G61172.1
Potra2n16c29791.1	0.398332837	-0.151712916	0.299933887	0.954559968	Potri.016G061500.1	AT1G53440.1
Potra2n16c29792.1	NA	NA	NA	NA	Potri.016G061600.1	AT3G61172.1
Potra2n16c29793.1	-0.057266663	-0.100567051	0.641920384	0.896937427	Potri.016G061700.4	AT3G58560.1
Potra2n16c29794.1	-0.027530002	-0.078397341	0.826845319	0.938237874	Potri.016G061900.1	AT2G42520.1
Potra2n16c29795.3	0.010218396	0.119542531	0.924389816	0.853030738	Potri.016G062000.1	AT3G58500.1
Potra2n16c29796.2	0.273358973	-0.020303396	0.00333232	0.979307683	Potri.016G062100.1	AT3G58490.1
Potra2n16c29797.1	-0.811276216	0.821709414	0.414073087	0.798016386	Potri.016G062200.1	AT3G11980.1
Potra2n16c29798.1	1.64774763	NA	NA	NA		
Potra2n16c29799.1	0.044955724	0.105364645	0.850530623	0.953608209	Potri.016G062400.1	AT5G06610.1
Potra2n16c29800.1	-0.143217765	0.015287561	0.45576558	0.984292161	Potri.016G062500.1	AT1G55310.1
Potra2n16c29801.2	0.244144073	0.192810617	0.111009037	0.907073354	Potri.016G062600.1	AT3G11964.1
Potra2n16c29802.1					Potri.016G062700.1	AT3G24420.1
Potra2n16c29803.1	-0.054935099	-0.013547988	0.7813982	0.992486563	Potri.016G062800.1	AT5G03500.1
Potra2n16c29804.1	-0.066435245	-0.075316006	0.763219499	0.954419252		
Potra2n16c29805.1	-0.114508309	-0.10555306	0.621139682	0.939143297	Potri.016G062900.2	AT3G11950.1
Potra2n16c29806.1	1.86088013	1.07427188	2.916E-05	0.451575262	Potri.016G063000.1	AT3G58480.1

Potra2n16c29807.1	1.85032832	1.132294597	5.64466E-12	0.26074573	Potri.015G107800.1	AT5G45540.1
Potra2n16c29808.1	0.461923719	-0.089216091	0.007854387	0.938967511	Potri.016G063100.1	AT5G64140.1
Potra2n16c29809.1	0.804060365	0.114858284	6.15152E-07	0.935851032	Potri.016G063200.1	AT2G37270.1
Potra2n16c29810.1	0.00966967	-0.156117889	0.975858976	0.914554682	Potri.016G063300.1	AT2G20710.1
Potra2n16c29811.1	0.27678667	0.047999016	0.035391262	0.960981446	Potri.016G063600.6	AT3G58470.1
Potra2n16c29812.1	2.522862329	0.805829526	0.215784836	NA	Potri.016G063700.1	AT1G10385.1
Potra2n16c29813.1	0.035929144	0.495246338	0.905576424	0.568587629	Potri.016G063800.6	AT3G43590.1
Potra2n16c29814.1	NA	NA	NA	NA		
Potra2n16c29815.1	NA	NA	NA	NA		
Potra2n16c29816.1	1.64774763	NA	NA	NA	Potri.016G063900.1	AT2G38110.1
Potra2n16c29817.3	-0.108326785	0.080786237	0.647332144	0.942644347	Potri.016G064000.1	AT3G11930.2
Potra2n16c29818.3	0.157485629	0.132594297	0.196212535	0.849446235	Potri.016G064100.1	AT5G06600.2
Potra2n16c29819.1	0.608061807	-0.62367283	0.243665339	0.809914834	Potri.016G064200.1	AT5G06590.1
Potra2n16c29820.1	-0.649365864	-0.371956277	0.015615644	0.652831518	Potri.016G064300.2	AT3G54720.1
Potra2n16c29821.1	-0.461470288	0.384308053	0.705938527	0.953608209		
Potra2n16c29822.1	-0.464182117	-0.173642775	0.004764666	0.810265332	Potri.016G064500.1	AT3G11900.1
Potra2n16c29823.1	-0.839102444	0.085272765	5.21836E-08	0.956773505	Potri.016G064600.1	AT2G38920.1
Potra2n16c29824.1	0.356244276	0.361093893	0.001412647	0.72101822	Potri.016G064800.2	AT5G06580.1
Potra2n16c29825.1	-0.499850203	0.014326895	0.002672274	0.995005375	Potri.016G065000.1	AT5G06570.2
Potra2n16c29826.1	1.21602603	0.250558268	0.521466238	NA		
Potra2n16c29827.1	0.988644571	-1.020765708	0.716665968	NA	Potri.016G065100.1	
Potra2n16c29828.1	-0.360698439	-0.128017783	0.000111895	0.895560236	Potri.016G065200.1	AT5G06560.1
Potra2n16c29829.1	0.440907971	2.161596096	0.775487343	0.386834221	Potri.006G199100.1	AT4G37980.1
Potra2n16c29830.1	0.24266643	0.144889703	0.242294219	0.871720501	Potri.016G065400.1	AT5G06550.1
Potra2n16c29831.1	0.179645643	0.356643777	0.739245652	0.876963181	Potri.016G065500.1	AT5G06540.1
Potra2n16c29832.1	-0.388837434	-0.124540085	0.722066667	0.974275337	Potri.016G065500.1	AT5G06540.1
Potra2n16c29833.2	-1.43139474	-0.284694195	1.10097E-11	0.767368598	Potri.016G065600.1	AT3G54750.3
Potra2n16c29834.1	-1.18557438	-0.195150203	0.000340271	0.918871056	Potri.016G065700.1	AT2G38910.1
Potra2n16c29835.1	NA	NA	NA	NA	Potri.016G065800.1	
Potra2n16c29836.1	NA	NA	NA	NA		
Potra2n16c29837.3	0.386075268	0.144520232	0.051715596	0.886633917	Potri.016G065900.1	AT5G06530.1
Potra2n16c29838.1	0.369220065	0.238630065	0.030757335	0.763747409	Potri.016G066000.4	AT1G06820.1

Potra2n16c29839.4	-0.070456343	0.047757605	0.645495145	0.975578749	Potri.016G066100.2	AT2G35840.2
Potra2n16c29840.1	1.419146127	-0.595043107	0.000746414	0.826474075	Potri.016G066200.1	AT3G52360.1
Potra2n16c29841.1	-0.782440729	-0.660376856	0.007162248	0.16040174	Potri.016G066300.1	AT3G11690.1
Potra2n16c29842.1	0.895596516	0.234137226	2.07266E-12	0.637421612	Potri.016G066400.1	AT5G22000.1
Potra2n16c29843.1	-0.004955681	-0.244541293	0.98445893	0.842247476	Potri.016G066500.1	AT5G06390.1
Potra2n16c29844.1	-0.902340015	0.696875817	0.012555105	0.70570456	Potri.016G066600.1	AT2G35880.1
Potra2n16c29845.1	1.341214096	0.846772423	0.000450766	0.108619662	Potri.016G066700.1	AT3G10660.1
Potra2n16c29846.1	-0.164093855	-0.090329066	0.173042801	0.885919394	Potri.016G066800.1	AT2G35900.1
Potra2n16c29847.1					Potri.016G066900.2	AT4G29035.1
Potra2n16c29848.8	0.241235616	0.222149252	0.049777281	0.643065116	Potri.016G067000.3	AT5G06440.2
Potra2n16c29849.1	-0.129963035	-0.039953845	0.297678583	0.977559521	Potri.016G067100.1	AT4G28360.1
Potra2n16c29850.1	-0.345684408	-0.114347734	0.006393331	0.942558727	Potri.016G067200.2	AT3G11750.1
Potra2n16c29851.1	-2.295337707	-0.828335558	NA	NA	Potri.016G067300.2	AT1G08465.1
Potra2n16c29852.1	NA	NA	NA	NA	Potri.016G067400.1	AT5G22030.1
Potra2n16c29853.1					Potri.016G067500.1	
Potra2n16c29854.1	-0.755461037	-0.186135737	2.78083E-06	0.834560473	Potri.016G067600.1	AT3G11760.1
Potra2n16c29855.1	-0.526563657	-0.798077109	0.634822256	0.860325259	Potri.016G067700.1	AT5G06470.1
Potra2n16c29856.1	NA	NA	NA	NA		
Potra2n16c29857.1	-0.17975499	-0.106321513	0.252186263	0.932932656	Potri.016G067800.2	AT3G11780.1
Potra2n16c29858.1	-0.19835321	-0.238767825	0.518852645	0.854980698	Potri.016G067900.1	AT2G35910.1
Potra2n16c29859.1	-0.337786196	-0.111443782	0.015320809	0.925184207	Potri.016G068000.1	AT3G44150.1
Potra2n16c29860.1	NA	NA	NA	NA		
Potra2n16c29861.1	NA	NA	NA	NA	Potri.016G068100.1	AT5G48670.1
Potra2n16c29862.4	-1.103039819	-0.249904404	2.69344E-08	0.890369631	Potri.016G068300.1	AT3G52380.1
Potra2n16c29863.1	-0.032350156	0.065847742	0.892306672	0.956889095	Potri.016G068400.1	AT3G52390.2
Potra2n16c29864.1	1.588902314	0.541607128	0.08472405	0.936964994	Potri.016G068500.1	AT2G31210.1
Potra2n16c29865.1	4.263295164	NA	0.220628279	NA		
Potra2n16c29866.1	4.548537616	0.74974738	4.1745E-20	0.871720501	Potri.016G068600.1	AT3G11820.1
Potra2n16c29867.1	0.121855631	0.092606794	0.247613623	0.909450222	Potri.016G068800.1	AT5G16280.1
Potra2n16c29868.1	0.666113326	0.395766836	1.83525E-11	0.259090305	Potri.016G068900.3	AT2G35920.1
Potra2n16c29869.1	0.519090747	-0.030488605	0.001741934	0.987548286	Potri.016G069000.1	AT1G70600.1
Potra2n16c29870.1	NA	NA	NA	NA		

Potra2n16c29871.1	-0.024924378	0.143882078	0.866883965	0.684244573	Potri.016G069100.1	AT3G05680.1
Potra2n16c29872.2	0.270544043	0.095853852	0.149506863	0.935474933	Potri.016G069200.1	AT3G61370.1
Potra2n16c29873.1	NA	2.0078969	NA	NA	Potri.016G069300.2	AT3G52440.1
Potra2n16c29874.1	7.371815029	2.408177284	2.83519E-07	NA	Potri.016G069400.1	AT3G52450.1
Potra2n16c29875.1	8.273413313	2.81373964	4.02761E-11	NA	Potri.016G069500.1	AT3G11840.1
Potra2n16c29876.1	NA	1.571529889	NA	NA	Potri.016G069600.1	AT3G05675.2
Potra2n16c29877.1					Potri.006G202900.2	AT3G05675.2
Potra2n16c29878.3	0.035015432	0.172957433	0.874562823	0.818007496	Potri.016G069700.2	AT2G35940.2
Potra2n16c29879.1	2.215123189	0.674963689	NA	NA	Potri.016G070000.1	AT3G11680.1
Potra2n16c29880.1	NA	2.676364087	NA	NA	Potri.016G070100.1	AT3G11680.1
Potra2n16c29881.1	-0.821030906	-0.02032687	1.18548E-05	0.989300076	Potri.016G070200.1	AT3G11670.1
Potra2n16c29882.1	-0.86185446	-0.109890398	0.003553221	0.943819961		
Potra2n16c29883.1	-0.634722392	0.185664009	0.0534658	0.907073354		
Potra2n16c29884.1	-0.595952689	-1.465929376	NA	NA		
Potra2n16c29885.1	0.420417498	0.018925722	0.009890187	0.989304496	Potri.006G203400.1	AT5G06360.1
Potra2n16c29886.1	-1.376818524	-0.470893131	6.33229E-07	0.797111689	Potri.016G070500.1	AT2G01820.1
Potra2n16c29887.1	0.824497211	-0.767708831	NA	NA	Potri.016G070600.1	
Potra2n16c29888.1	-0.152601827	-0.285345141	0.208313978	0.357747948	Potri.016G070700.1	AT3G09180.1
Potra2n16c29889.1	-1.347188655	-1.495886149	NA	NA		
Potra2n16c29890.1	0.695684066	-0.320302286	0.03880636	0.865591169	Potri.016G070800.1	AT4G31400.1
Potra2n16c29891.1	-0.197107406	-0.268234115	0.147201836	0.586594557	Potri.016G070900.1	AT5G06340.1
Potra2n16c29892.2	-0.795620531	-0.538170728	0.000317799	0.563971296	Potri.016G071000.1	AT5G52340.1
Potra2n16c29893.1	-0.61910233	-0.767708831	NA	NA	Potri.016G071100.2	AT3G52460.1
Potra2n16c29894.1	NA	NA	NA	NA	Potri.016G071200.1	AT3G52460.1
Potra2n16c29895.1	-0.640727853	0.399216	0.339229564	0.88643982	Potri.016G071400.1	
Potra2n16c29896.2	0.377403072	-0.103091422	0.075910036	0.948577865	Potri.016G071500.1	AT3G11660.1
Potra2n16c29897.1	4.355318022	1.201420126	3.94441E-27	0.180635806	Potri.016G071600.2	AT2G35980.1
Potra2n16c29898.1	0.397198195	0.038777517	0.025880961	0.988459807	Potri.016G071700.1	AT3G52480.1
Potra2n16c29899.1	4.27532905	1.571529889	0.216192998	NA	Potri.016G071800.1	AT3G52490.1
Potra2n16c29900.1	4.499360177	1.975074671	2.18614E-69	0.040508424	Potri.016G071900.1	AT3G52500.1
Potra2n16c29901.1	1.459962178	4.014082846	NA	NA	Potri.016G072000.1	AT5G06300.1
Potra2n16c29902.1	-0.034366935	-0.16697315	0.803370936	0.821709751	Potri.016G072100.2	AT5G06290.1

Potra2n16c29903.1	-0.739243398	-0.353842892	0.000150072	0.663656471	Potri.016G072200.1	AT2G36000.1
Potra2n16c29904.3	0.654275855	-0.06497721	0.002003308	0.9632996	Potri.016G072300.1	AT2G36010.1
Potra2n16c29905.1	-1.241756628	-0.260502142	4.3284E-07	0.846926485	Potri.006G205300.1	AT2G36020.1
Potra2n16c29906.1	-0.744761692	-0.093595126	0.000598986	0.95999889	Potri.016G072500.1	AT5G06280.3
Potra2n16c29907.1	0.439538549	0.177343298	0.010956008	0.867910364	Potri.016G072700.1	AT3G11620.1
Potra2n16c29908.1	1.414975419	-0.120223889	0.090428123	0.985393296	Potri.016G072800.2	AT2G36026.1
Potra2n16c29909.1	1.459962178	NA	NA	NA		
Potra2n16c29910.1	4.162315097	3.145099921	0.049052519	NA	Potri.016G072900.1	AT5G04820.1
Potra2n16c29911.1	0.126415004	-0.200694013	0.744549599	0.903477395	Potri.016G073000.1	AT5G22250.1
Potra2n16c29912.1	0.213241061	0.032473567	0.06329323	0.974756511	Potri.016G073100.1	AT3G52560.1
Potra2n16c29913.1	-0.637934825	-1.507841271	NA	NA	Potri.016G073200.1	AT2G36620.1
Potra2n16c29914.1						
Potra2n16c29915.1	1.545832463	NA	NA	NA		
Potra2n16c29916.1					Potri.006G205800.1	AT2G37340.1
Potra2n16c29917.1					Potri.016G073300.1	
Potra2n16c29918.1	-1.540586995	0.834006497	0.000861473	0.389989789	Potri.016G073400.1	AT5G06270.1
Potra2n16c29919.1	-0.098776598	0.004898276	0.719970628	0.997821888	Potri.016G073500.1	AT5G22280.1
Potra2n16c29920.1	2.480772152	0.317787783	7.56043E-10	0.915339763	Potri.016G073700.1	AT1G52340.1
Potra2n16c29921.1					Potri.016G073800.1	AT1G52340.1
Potra2n16c29922.1					Potri.016G073800.1	AT1G52340.1
Potra2n16c29923.1	1.664677056	NA	NA	NA	Potri.016G073900.1	AT3G51680.1
Potra2n16c29924.3	0.572145124	0.359992716	0.001296741	0.129223662	Potri.016G074100.1	AT3G02875.1
Potra2n16c29925.2	0.576389196	0.455123808	0.035638197	0.636664382		
Potra2n16c29926.2	-0.242418329	0.301601916	0.526072047	0.845014308	Potri.016G074200.1	AT5G22300.1
Potra2n16c29927.1	-0.776041424	-0.373210912	8.81792E-07	0.165433725	Potri.016G074300.1	AT3G11590.1
Potra2n16c29928.1	0.82357019	0.674963689	NA	NA	Potri.016G074400.1	AT5G65760.1
Potra2n16c29929.1	-1.121450058	-0.372545888	0.11136287	0.893394174	Potri.016G074500.2	AT5G06250.2
Potra2n16c29930.1	-1.69485378	-0.730001095	0.037192075	0.854980698		
Potra2n16c29931.4	-0.266012827	0.1943728	0.392199828	0.923475785	Potri.016G074700.1	AT5G06240.1
Potra2n16c29932.1	0.102236182	1.16723475	NA	NA		
Potra2n16c29933.1	0.357229377	-2.05552118	0.857576477	NA		
Potra2n16c29934.1	-0.716080714	-0.356367172	1.01135E-05	0.365818347	Potri.016G074800.1	AT3G11570.1

Potra2n16c29935.1	-0.649023949	-0.152585454	7.84448E-06	0.802631112	Potri.016G074900.10	AT3G11560.4
Potra2n16c29936.1	-1.785419727	-0.757142737	0.617615279	NA		
Potra2n16c29937.1	-0.558659238	-0.187370609	0.009639414	0.759797728	Potri.016G075000.1	AT2G41080.1
Potra2n16c29938.1	-0.155563641	-0.069261205	0.218845755	0.910815461	Potri.016G075200.2	AT1G08370.1
Potra2n16c29939.1	-1.206225431	0.389259737	0.741069522	NA	Potri.016G075300.1	AT3G11550.1
Potra2n16c29940.1					Potri.016G075400.1	AT2G36100.1
Potra2n16c29941.1	-0.263364413	-0.061449438	0.21272057	0.953608209	Potri.016G075500.1	AT5G22350.1
Potra2n16c29942.1	0.326458106	-0.000798593	0.015704921	0.999536469	Potri.016G075600.1	AT2G36130.1
Potra2n16c29943.1	0.281351446	0.285776858	0.074445362	0.530106124	Potri.016G075700.1	AT3G11540.1
Potra2n16c29944.1	1.64774763	1.381953902	NA	NA		
Potra2n16c29945.1	2.842991783	2.134902029	NA	NA		
Potra2n16c29946.1	2.841738279	0.349514383	2.15982E-07	0.963993404	Potri.016G075800.1	AT1G12910.1
Potra2n16c29947.1	1.056658952	0.898751367	0.137032202	0.786005488		
Potra2n16c29948.1	6.656735839	2.283425176	1.20025E-05	NA	Potri.016G076000.1	AT5G22380.1
Potra2n16c29949.1	8.789029392	6.171426837	4.96005E-26	0.002392219	Potri.016G076100.1	AT5G22380.1
Potra2n16c29950.1	1.256934522	0.389154029	6.67121E-06	0.895474078		
Potra2n16c29951.1	1.584753574	0.117411261	0.18108957	NA		
Potra2n16c29952.1	-0.228177022	-0.148497601	0.240858562	0.894138111	Potri.016G076300.1	AT3G15040.1
Potra2n16c29953.1	-0.160206156	-0.01627397	0.201365352	0.989304496	Potri.016G076400.1	AT5G06140.1
Potra2n16c29954.1	0.559868211	0.100548758	0.001034628	0.95086402	Potri.016G076500.1	AT5G39850.1
Potra2n16c29955.1	0.009238717	-0.022667124	0.961890434	0.985393296	Potri.016G077100.1	AT3G52570.1
Potra2n16c29956.1	0.695686244	-0.018959293	0.000108317	0.991407187	Potri.012G024300.1	AT2G36170.1
Potra2n16c29957.1	-1.35927387	-0.786502229	NA	NA	Potri.016G077400.2	AT3G52600.1
Potra2n16c29958.1	3.165371712	2.547923856	0.114735095	NA	Potri.016G077500.1	AT3G52600.1
Potra2n16c29959.1					Potri.013G000300.2	AT1G09570.1
Potra2n16c29960.1	0.815308259	-0.039526068	0.001108168	0.985393296	Potri.016G077800.1	AT2G36200.1
Potra2n16c29961.1	1.545832463	1.871975723	NA	NA	Potri.016G078000.1	AT3G52610.1
Potra2n16c29962.1	0.352713361	-0.036106035	0.080110721	0.982422288	Potri.016G078100.1	AT3G11500.1
Potra2n16c29963.1	1.64774763	NA	NA	NA		
Potra2n16c29964.1	-0.684702368	-0.322558572	0.000173062	0.453270097	Potri.016G078200.1	AT5G22400.1
Potra2n16c29965.1						
Potra2n16c29966.1	0.18100949	-0.067041038	0.306147814	0.954721208	Potri.016G078300.1	AT4G37970.1

Potra2n16c29967.1	-0.224722443	0.116456798	0.632040467	0.972159498	Potri.016G078400.10	AT2G02770.1
Potra2n16c29968.1	-0.17216592	0.164526704	0.475489564	0.922029226	Potri.016G078700.1	AT3G52620.1
Potra2n16c29969.1	-1.328951947	-1.478536539	NA	NA	Potri.016G078800.1	AT2G38870.1
Potra2n16c29970.1	-1.494183014	-2.515023789	NA	NA	Potri.016G079100.1	AT2G38870.1
Potra2n16c29971.1	0.005504323	0.086319901	0.968567953	0.920763566	Potri.016G079200.3	AT3G52660.1
Potra2n16c29972.1	-0.092875493	0.02923633	0.538537894	0.980475138	Potri.016G079300.1	AT1G57790.1
Potra2n16c29973.1	2.056307011	-0.18163576	0.00247156	0.979719961	Potri.016G079400.1	AT5G03870.1
Potra2n16c29974.1	0.654245737	-0.059422073	0.000507693	0.974367223	Potri.016G079500.1	AT3G52710.1
Potra2n16c29975.1	-2.903285383	0.346526509	0.089941644	0.966228173	Potri.016G079600.1	AT3G52720.1
Potra2n16c29976.1	-0.976899669	0.101983701	0.457549743	0.988210253	Potri.016G079700.1	AT3G10120.1
Potra2n16c29977.1	0.340119897	-0.079059301	0.138857445	0.95671968	Potri.016G079800.2	AT3G55280.2
Potra2n16c29978.1	0.351727307	-0.091995236	0.164942895	0.954419252		
Potra2n16c29979.1	0.408190873	-0.090294437	0.076660188	0.953177702		
Potra2n16c29980.1	-0.214736664	-0.059305393	0.04291545	0.930813462	Potri.016G079900.1	AT3G53570.2
Potra2n16c29981.1	-0.046768609	-0.16411646	0.804863346	0.859892315	Potri.016G080000.1	AT2G37400.1
Potra2n16c29982.1	-1.007209537	-0.152157817	1.15401E-05	0.878884929	Potri.016G080200.1	AT3G53540.1
Potra2n16c29983.1	-3.04142869	-3.185647093	NA	NA	Potri.016G080250.1	
Potra2n16c29984.1					Potri.016G080300.1	AT1G49330.1
Potra2n16c29985.1	0.839365451	0.517914786	0.190487987	0.842247476	Potri.016G080400.1	AT2G37390.1
Potra2n16c29986.1	-0.343451599	-0.098744549	0.039308254	0.953108412	Potri.016G080500.1	AT3G53520.1
Potra2n16c29987.1	0.000432683	-0.017052763	0.999895833	0.998883928		
Potra2n16c29988.1	0.093449761	0.094767838	0.564531952	0.918971596	Potri.016G080600.1	AT3G55140.1
Potra2n16c29989.1	0.505865515	0.034354595	0.017575538	0.977227089	Potri.016G080700.1	AT1G07710.1
Potra2n16c29990.2	0.35144105	-0.97212365	0.760513285	0.676711874	Potri.016G080800.1	AT2G39370.1
Potra2n16c29991.1	2.311133668	0.234936987	1.96305E-22	0.906679354	Potri.016G080900.2	AT2G37370.1
Potra2n16c29992.1	-0.05412089	0.102192291	0.73150709	0.910878765	Potri.016G081000.2	AT3G09560.1
Potra2n16c29993.1						
Potra2n16c29994.1	-0.016636093	-0.148513068	0.922674867	0.895460013	Potri.016G081300.1	AT5G18520.1
Potra2n16c29995.1	-0.05910037	-0.197772535	0.726545548	0.816567218	Potri.016G081400.1	
Potra2n16c29996.1					Potri.015G080600.1	AT5G03340.1
Potra2n16c29997.1	-2.96400362	-0.564624308	0.014448141	0.924251575		
Potra2n16c29998.1	-1.335292263	-0.465146347	1.01262E-05	0.543618527	Potri.016G081600.1	AT5G02640.1

Potra2n16c29999.1	-0.058285097	-0.075929228	0.696269169	0.935474933	Potri.016G081700.1	AT3G53500.2
Potra2n16c30000.1	1.068662091	0.086628536	0.005187953	0.976302215	Potri.016G081800.1	
Potra2n16c30001.1	NA	NA	NA	NA	Potri.006G215400.1	
Potra2n16c30002.1	-1.35927387	-1.507841271	NA	NA		
Potra2n16c30003.1	-0.665799139	-0.21711809	0.000260475	0.80723942	Potri.016G081900.2	AT3G53490.1
Potra2n16c30004.1	-1.658522013	-0.332241427	4.15931E-08	0.750123244	Potri.016G082000.1	AT3G09590.1
Potra2n16c30005.1	-1.655130897	-0.323983951	1.01597E-07	0.79819686	Potri.016G082100.1	AT2G37330.1
Potra2n16c30006.1					Potri.015G071600.1	
Potra2n16c30007.1	0.102236182	1.592533919	NA	NA	Potri.006G215800.1	AT5G02740.1
Potra2n16c30008.1	0.286028385	0.003520546	0.475795151	0.999325699	Potri.016G082200.1	AT2G37320.1
Potra2n16c30009.1	0.638322426	-0.110812259	0.025954892	0.969421568	Potri.016G082300.1	AT3G49010.1
Potra2n16c30010.2	-1.262305566	-0.314775644	2.61096E-08	0.855754617	Potri.016G082400.3	AT3G45850.2
Potra2n16c30011.1	-1.106568964	-0.369452053	0.246867686	0.923959006	Potri.016G082500.1	AT5G02750.1
Potra2n16c30012.1	-1.637070303	-0.456529122	1.16139E-06	0.862830524	Potri.016G082700.3	AT2G37300.2
Potra2n16c30013.1	-1.314656131	-0.353258684	2.80965E-07	0.794817498	Potri.016G082800.1	AT3G12620.1
Potra2n16c30014.1	-0.034735588	0.154954942	0.812469509	0.817799498	Potri.016G082900.1	AT5G02770.1
Potra2n16c30015.1					Potri.006G133800.1	AT5G60335.1
Potra2n16c30016.1	0.137219219	0.270906587	0.246948025	0.578651675	Potri.016G083100.6	AT1G07910.2
Potra2n16c30017.1					Potri.001G206200.1	AT5G09360.1
Potra2n16c30018.1	1.774039208	0.626880808	1.15846E-17	0.03368267	Potri.016G083500.1	AT5G02790.1
Potra2n16c30019.2	2.361382826	0.496860265	3.93994E-13	0.831597503	Potri.016G083600.1	AT2G37260.1
Potra2n16c30020.1	NA	NA	NA	NA		
Potra2n16c30021.1	-0.401212587	1.40420526	0.365990622	NA	Potri.016G083900.1	AT3G09600.1
Potra2n16c30022.1	-0.966136705	0.564554978	0.003226545	0.82104194	Potri.004G232250.1	AT5G49760.1
Potra2n16c30023.1	0.057970933	0.035988109	0.785930598	0.978725627	Potri.016G084200.1	AT5G02850.1
Potra2n16c30024.1	-0.031535566	-0.087207226	0.91454696	0.951394085	Potri.016G084300.1	
Potra2n16c30025.1	0.754279767	0.10687232	1.72043E-06	0.935851032	Potri.016G084400.1	AT3G09630.1
Potra2n16c30026.1	3.025037209	0.674963689	NA	NA	Potri.016G106400.1	
Potra2n16c30027.1	NA	NA	NA	NA		
Potra2n16c30028.1	-0.059321301	-0.06421663	0.8383069	0.960981446	Potri.016G084500.1	AT2G28550.3
Potra2n16c30029.2	-0.160337973	0.016472246	0.059874813	0.982933413	Potri.016G084700.1	AT3G45630.1
Potra2n16c30030.1	1.830220177	1.508923631	0.436004362	NA		

Potra2n16c30031.1	0.714709946	0.241305018	3.20344E-06	0.79078676	Potri.016G084800.1	AT3G09640.2
Potra2n16c30032.1	2.409567423	NA	NA	NA		
Potra2n16c30033.1	0.038015563	0.248276664	0.887101814	0.809323756	Potri.016G084900.1	AT2G37050.1
Potra2n16c30034.1	3.326442903	2.283425176	NA	NA		
Potra2n16c30035.2	-0.0720835	0.103657519	0.700234237	0.935751704	Potri.016G085000.1	AT2G37060.3
Potra2n16c30036.1	1.64774763	1.363811008	NA	NA	Potri.016G085200.3	AT5G02880.1
Potra2n16c30037.1	0.446458625	-1.292229428	NA	NA	Potri.016G085200.3	AT5G02880.1
Potra2n16c30038.1	-0.109340604	-0.135713219	0.53863879	0.832750076	Potri.016G085300.1	AT3G09650.1
Potra2n16c30039.1	0.352489036	0.099134681	0.006141509	0.912295985	Potri.016G085500.1	AT3G09670.2
Potra2n16c30040.1	-0.625584793	-1.495886149	NA	NA	Potri.016G085400.1	AT3G09660.1
Potra2n16c30041.4	-0.224236466	-0.12410783	0.483030954	0.956865545	Potri.016G085600.1	AT3G53320.1
Potra2n16c30042.1	0.64542129	0.07720899	3.56764E-05	0.951726178	Potri.006G131500.2	AT5G02960.1
Potra2n16c30043.2	-0.570010159	-0.344617977	0.019614143	0.646171083	Potri.016G085900.1	AT2G37080.1
Potra2n16c30044.1	-0.593027411	-1.332320361	NA	NA		
Potra2n16c30045.1	-0.428551383	0.159577565	0.158222939	0.92267921	Potri.016G086100.1	AT1G26500.1
Potra2n16c30046.1	0.179921168	-0.092299329	0.639898252	0.964766975	Potri.016G086200.1	AT5G02970.1
Potra2n16c30047.2	-0.639360555	-0.191243891	0.00624349	0.888366602	Potri.016G086300.4	AT5G03040.1
Potra2n16c30048.1	2.249249553	3.974736291	NA	NA	Potri.016G096100.1	
Potra2n16c30049.1	-0.557815395	0.345282714	0.813983697	0.974264269	Potri.004G232250.1	AT5G49760.1
Potra2n16c30050.1	-1.35927387	-1.507841271	NA	NA		
Potra2n16c30051.1	-0.74247354	-0.26371086	0.000353467	0.864026207	Potri.016G086400.1	AT2G37090.1
Potra2n16c30052.1	-0.550229946	0.050711967	0.353089435	0.989304496	Potri.006G130900.1	AT5G60390.1
Potra2n16c30053.1	-0.216066141	-0.083962113	0.077205007	0.885375133	Potri.016G086600.1	AT5G03070.1
Potra2n16c30054.1	-0.080286898	1.727532202	0.976449721	0.459805191		
Potra2n16c30055.2	-0.051899994	-0.133852758	0.821861277	0.885375133	Potri.016G086700.1	AT5G03080.1
Potra2n16c30056.1	NA	NA	NA	NA	Potri.006G270200.1	AT4G22160.2
Potra2n16c30057.1	-0.685274656	-0.212551723	0.001009019	0.79549388	Potri.016G086900.1	AT2G37100.1
Potra2n16c30058.1	-0.61910233	-0.767708831	NA	NA		
Potra2n16c30059.1	-0.81575177	-0.036454641	0.002898117	0.985964146	Potri.016G087000.1	AT5G03120.1
Potra2n16c30060.1	NA	NA	NA	NA	Potri.016G087100.1	AT2G16660.1
Potra2n16c30061.1	0.145342089	0.048009841	0.217609649	0.954419252	Potri.016G087200.1	AT2G37110.1
Potra2n16c30062.1	0.184007103	0.06645055	0.355222668	0.966228173	Potri.013G018000.1	AT5G28540.1

Potra2n16c30063.1	-0.08679897	-0.291867458	0.619923652	0.522372765	Potri.016G087400.1	AT2G37120.1
Potra2n16c30064.1	-0.462218229	-0.156483308	0.251121472	0.945150162	Potri.016G087800.1	AT3G53380.1
Potra2n16c30065.1	-0.49039435	-0.63555473	0.342432966	0.80848715	Potri.016G087800.1	AT3G53380.1
Potra2n16c30066.2	0.37111625	-0.140116139	0.021212594	0.923478213	Potri.016G087900.1	AT3G57610.1
Potra2n16c30067.1	7.406493178	3.625595709	4.45202E-21	0.322221366	Potri.016G088000.2	AT1G61560.1
Potra2n16c30068.1	-0.379040363	-0.295650731	0.155857464	0.828625141	Potri.017G135600.2	AT4G05320.2
Potra2n16c30069.1	-0.384901914	-0.284759157	0.001330518	0.692084498	Potri.016G088200.1	AT3G09740.1
Potra2n16c30070.1	NA	2.490806657	NA	NA	Potri.016G088300.2	AT1G69490.1
Potra2n16c30071.1	-0.52303275	-0.071434737	0.011948097	0.958392068	Potri.016G088500.1	AT5G03150.1
Potra2n16c30072.1	0.424982971	0.294872443	0.002407693	0.718626343	Potri.016G088600.1	AT5G03160.1
Potra2n16c30073.1	-1.149914632	-0.290874416	3.95299E-06	0.8693465	Potri.006G129200.1	AT5G03170.1
Potra2n16c30074.1	-0.738453061	0.003435205	0.192611456	0.999458638		
Potra2n16c30075.1	-0.162552555	0.015355611	0.223672906	0.988154814	Potri.016G088800.2	AT3G53390.1
Potra2n16c30076.1	0.237923508	0.358401888	0.517128212	0.810712263	Potri.016G088900.1	AT3G53400.1
Potra2n16c30077.1	-0.220295071	-0.215845683	0.196535134	0.70986976	Potri.016G089000.1	AT3G09770.1
Potra2n16c30078.3	-0.90197256	0.030302496	4.07906E-05	0.984683464	Potri.016G089200.1	AT3G09780.1
Potra2n16c30079.1	0.083843963	0.021458344	0.439017961	0.978203092	Potri.016G089300.1	AT3G58750.1
Potra2n16c30080.1	-0.624363764	-0.006295314	0.006548842	0.998136564	Potri.016G089500.1	AT2G37170.1
Potra2n16c30081.1	-1.456104737	-0.392664427	0.306320447	0.961021731	Potri.016G089800.1	
Potra2n16c30082.1	0.894976119	0.157528865	1.07613E-05	0.954419252	Potri.016G089900.1	AT5G03230.1
Potra2n16c30083.1	-0.097989773	-0.062756579	0.587818776	0.954559968	Potri.016G090000.1	AT2G37195.1
Potra2n16c30084.2	0.571914854	0.220179774	0.000459758	0.736000077	Potri.016G090100.1	AT2G28370.1
Potra2n16c30085.1	0.314666828	0.060860839	0.080722585	0.953831244	Potri.016G090300.1	AT4G30080.1
Potra2n16c30086.1	1.459962178	NA	NA	NA	Potri.005G219600.1	AT1G50640.1
Potra2n16c30087.1	0.291812319	0.666012702	0.576161104	0.67987831	Potri.016G090400.1	AT5G03250.1
Potra2n16c30088.1	-2.112169729	0.589737158	0.172887786	0.915580711	Potri.016G090500.1	AT2G37210.1
Potra2n16c30089.1	0.10756847	-0.112592257	0.594226165	0.936402128	Potri.016G090700.2	AT2G37220.1
Potra2n16c30090.2	-0.065756162	0.04262925	0.637325407	0.956865545	Potri.016G090800.4	AT5G03280.1
Potra2n16c30091.1	-0.062977274	-0.348850705	0.837727792	0.794596445	Potri.016G091100.1	AT2G37040.1
Potra2n16c30092.1	0.46249439	0.122348045	0.000207826	0.8645281	Potri.016G091200.2	AT5G03290.1
Potra2n16c30093.1	-0.414233858	0.067821906	0.127650841	0.956773505	Potri.006G126500.1	AT2G37030.1
Potra2n16c30094.1	-1.176485566	-0.924029868	0.713810952	NA	Potri.006G126300.3	AT2G37025.2

Potra2n16c30095.1	0.055129732	0.021146508	0.675595198	0.987226805	Potri.016G091600.1	AT5G03340.1
Potra2n16c30096.1	0.078901307	0.041989903	0.546483848	0.959623437	Potri.016G091700.1	AT2G36960.3
Potra2n16c30097.1	-0.362244782	-0.042008719	0.018091919	0.974367223	Potri.016G091800.1	AT3G12780.1
Potra2n16c30098.1	-0.29046084	-0.080146577	0.045214096	0.948577865	Potri.016G091900.1	AT5G03345.1
Potra2n16c30099.1	-0.003306378	-0.049909235	0.985791167	0.970218964	Potri.016G092000.2	AT4G34340.1
Potra2n16c30100.1	-0.022861183	-0.063421952	0.897496184	0.951734262	Potri.016G092100.3	AT3G09860.1
Potra2n16c30101.1	-0.570966079	0.286326235	0.380371228	0.933996725	Potri.016G092200.1	AT1G70280.2
Potra2n16c30102.1	-0.389091729	-0.055228859	0.167414196	0.9761362	Potri.016G092300.2	AT5G52970.1
Potra2n16c30103.1	-0.099244918	-0.214414608	0.909541599	0.960656795	Potri.016G092400.1	AT3G09870.1
Potra2n16c30104.1	1.664677056	1.871975723	NA	NA	Potri.T055866.1	
Potra2n16c30105.1	0.107926935	-0.417907447	0.775055725	0.661005194	Potri.016G092600.1	AT3G50950.1
Potra2n16c30106.1	1.591592518	0.133227142	0.255987172	NA	Potri.016G092700.4	AT1G53430.1
Potra2n16c30107.1	-0.297910731	-0.022343405	0.033184807	0.985282929	Potri.016G091800.1	AT3G12780.1
Potra2n16c30108.2	-0.097373215	-0.003995815	0.299475373	0.997164503	Potri.016G091900.1	AT5G03345.1
Potra2n16c30109.1	0.021191716	-0.032444244	0.910607951	0.98036348	Potri.016G092000.2	AT4G34340.1
Potra2n16c30110.1	-0.037784866	-0.171596616	0.819748654	0.842247476	Potri.016G092100.3	AT3G09860.1
Potra2n16c30111.1	0.13444481	0.272215697	0.834953914	0.936890993	Potri.016G092200.1	AT1G70280.2
Potra2n16c30112.3	0.307435225	-0.175650224	0.320995252	0.937146435	Potri.016G092300.2	AT5G52970.1
Potra2n16c30113.1	0.111531213	-0.366462586	0.873789146	0.927547456	Potri.016G092400.1	AT3G09870.1
Potra2n16c30114.1	-0.041999235	-0.441099092	0.89637178	0.53266226	Potri.016G092600.1	AT3G50950.1
Potra2n16c30115.2	1.827663695	0.54230429	0.045462303	0.896089262	Potri.016G092700.4	AT1G53430.1
Potra2n16c30116.1	0.082879298	-0.352243581	0.772916432	0.759797728	Potri.016G092600.1	AT3G50950.1
Potra2n16c30117.1	2.116055856	0.303107857	0.000480935	0.953831244	Potri.016G092700.4	AT1G53430.1
Potra2n16c30118.1	0.208228139	-0.000118772	0.33626337	0.999937671	Potri.016G093000.1	AT5G03370.1
Potra2n16c30119.1	-1.080486953	-0.040471227	3.56473E-08	0.974275337	Potri.016G093100.1	AT5G03380.1
Potra2n16c30120.1	0.046240335	0.1748712	0.78156958	0.737112468	Potri.016G093200.2	AT5G03415.1
Potra2n16c30121.1	NA	NA	NA	NA	Potri.016G093300.1	
Potra2n16c30122.1	0.436760669	-0.079823906	0.000996597	0.888096054	Potri.016G093500.1	AT2G36930.1
Potra2n16c30123.1	NA	1.381953902	NA	NA		
Potra2n16c30124.1						
Potra2n16c30125.1	-0.399349402	-0.320922302	0.024345217	0.547550309	Potri.016G093600.1	AT2G36910.1
Potra2n16c30126.1						

Potra2n16c30127.1	-1.328951947	0.139386737	NA	NA		
Potra2n16c30128.1					Potri.017G112800.1	AT4G05160.1
Potra2n16c30129.1	0.37176786	0.206053994	0.003212133	0.824905557	Potri.T058900.1	AT5G59950.5
Potra2n16c30130.1	0.336594669	0.133276269	0.010189379	0.875559093	Potri.016G094300.1	AT1G07570.2
Potra2n16c30131.1	0.204119144	0.134352002	0.07315628	0.881090429	Potri.016G094700.1	AT5G03330.1
Potra2n16c30132.1	0.171356937	0.342650169	0.526072047	0.835819291	Potri.016G094800.1	AT3G47620.1
Potra2n16c30133.1	0.036664426	0.185213568	0.809353297	0.578635193	Potri.012G063500.1	AT5G08110.1
Potra2n16c30134.1	-3.142086698	-0.786572208	0.32381187	NA	Potri.009G155200.3	AT2G27170.1
Potra2n16c30135.1	NA	2.253733581	NA	NA	Potri.004G232250.1	AT5G49760.1
Potra2n16c30136.1	-0.393117466	0.058301113	0.011551729	0.948577865	Potri.016G095500.1	AT5G02390.1
Potra2n16c30137.1	-1.425318927	-1.070482242	0.524079404	NA	Potri.004G193000.2	AT5G20930.1
Potra2n16c30138.2	-0.128756256	0.05862692	0.619249111	0.962696825	Potri.019G024200.1	AT1G64790.1
Potra2n16c30139.1	-0.170638138	0.61082004	0.876950308	0.859543817	Potri.004G193000.2	AT5G20930.1
Potra2n16c30140.1	2.796801753	2.871591326	NA	NA	Potri.009G155200.3	AT2G27170.1
Potra2n16c30141.1	0.82357019	2.88555167	NA	NA		
Potra2n16c30142.1	0.571310423	0.543741599	0.004911553	0.498403695	Potri.016G095750.1	AT5G03730.1
Potra2n16c30143.1	0.025807124	0.124496833	0.848972997	0.842247476	Potri.016G095900.1	AT5G03770.1
Potra2n16c30144.1	1.049963746	-0.06652061	2.57562E-05	0.974367223	Potri.016G096300.1	AT3G10040.1
Potra2n16c30145.1	0.952642524	0.265278292	0.00552074	0.918311446		
Potra2n16c30146.1	0.277656309	0.092313546	0.203990513	0.947899631	Potri.016G096400.1	AT5G03800.1
Potra2n16c30147.1	-0.24817079	0.056712376	0.026080598	0.935130474	Potri.016G096500.7	AT3G53090.2
Potra2n16c30148.2	-0.660342303	0.413317408	0.034153358	0.869327705	Potri.016G096600.1	AT3G10060.1
Potra2n16c30149.1	-0.012718394	0.225931238	0.953008093	0.843683654	Potri.016G096700.1	AT2G36670.1
Potra2n16c30150.1	0.120498501	-0.097139349	0.506983545	0.94169165	Potri.016G096800.1	AT5G67630.1
Potra2n16c30151.1	0.153047268	0.074438858	0.54182728	0.959881296	Potri.016G096900.1	AT5G03560.1
Potra2n16c30152.1	-0.066983701	-0.107170329	0.673014199	0.925184207	Potri.016G097000.1	AT2G36680.1
Potra2n16c30153.1	-0.613300992	-0.752020671	0.613178589	0.790362046	Potri.016G097100.1	AT1G32700.1
Potra2n16c30154.2	-0.112713785	0.292737559	0.609348461	0.72101822	Potri.016G097300.4	AT2G36720.1
Potra2n16c30155.1	4.93089096	1.090377446	3.57151E-16	0.885375133	Potri.016G097400.1	AT3G53150.1
Potra2n16c30156.1	0.82357019	NA	NA	NA	Potri.016G097500.1	AT2G39700.1
Potra2n16c30157.3	-0.365673654	-0.137377705	0.321209465	0.943483421	Potri.006G121300.1	AT2G32600.1
Potra2n16c30158.1	-0.286434324	-0.065395848	0.049495519	0.956865545	Potri.016G097800.1	AT5G03530.1

Potra2n16c30159.1	-0.274427762	-0.102409024	0.146864521	0.94076214		
Potra2n16c30160.3	0.355136235	-0.196577291	0.082826696	0.881943425	Potri.016G097900.1	AT3G09890.1
Potra2n16c30161.1	-2.17275421	-0.454222036	4.9096E-07	0.647178693	Potri.016G098100.1	AT2G28200.1
Potra2n16c30162.1	-1.066587018	0.550625073	4.48104E-05	NA	Potri.016G098200.1	AT2G36830.1
Potra2n16c30163.1	NA	NA	NA	NA		
Potra2n16c30164.1	-1.660812811	-0.631034327	3.05205E-05	0.210620025	Potri.016G098300.1	
Potra2n16c30165.1	-0.053209492	0.005121128	0.72515377	0.996032145	Potri.006G122300.1	AT3G09880.1
Potra2n16c30166.2	-0.801133023	-0.105197421	0.000138834	0.950182516	Potri.016G098500.2	AT3G10130.1
Potra2n16c30167.1	2.93539049	0.5921931	3.01556E-06	0.922993749	Potri.016G098600.1	AT2G36870.1
Potra2n16c30168.1					Potri.001G155750.1	AT1G21280.1
Potra2n16c30169.1	2.918219423	NA	NA	NA	Potri.016G098700.1	AT2G37640.1
Potra2n16c30170.1	NA	NA	NA	NA	Potri.007G096100.2	AT2G22400.1
Potra2n16c30171.1	0.217425492	0.014036416	0.420369767	0.995920419	Potri.016G098900.3	AT2G36885.1
Potra2n16c30172.1					Potri.016G099000.1	AT5G03420.1
Potra2n16c30173.1	2.822166348	0.468297673	9.56893E-24	0.691005224	Potri.016G099200.1	AT5G57620.1
Potra2n16c30174.1	-0.233894701	-0.10565104	0.36293887	0.953768643	Potri.016G099300.1	AT2G36895.1
Potra2n16c30175.1	-0.116654463	0.101743293	0.313829083	0.919722977	Potri.016G099400.1	AT1G65260.1
Potra2n16c30176.1	-0.049602387	0.278121236	0.732383981	0.749682671	Potri.016G099800.1	AT1G07510.1
Potra2n16c30177.1					Potri.016G099900.1	AT5G43290.1
Potra2n16c30178.1	-0.262051787	0.232140583	0.320678619	0.846680547	Potri.016G100100.1	
Potra2n16c30179.2	0.251671247	0.252908341	0.023115525	0.81043113	Potri.016G100200.1	AT3G09320.1
Potra2n16c30180.1	NA	NA	NA	NA	Potri.016G100300.1	AT5G02180.1
Potra2n16c30181.1	0.985285796	0.229376911	4.13235E-08	0.865538396	Potri.016G100400.1	AT3G02560.1
Potra2n16c30182.1	0.252873898	0.271483193	0.046973867	0.694477853	Potri.016G100500.2	AT3G09350.1
Potra2n16c30183.1	0.075049944	0.043226264	0.572391034	0.960981446	Potri.016G100600.1	AT2G37680.1
Potra2n16c30184.1	3.533323647	-1.55593228	0.055945207	NA	Potri.016G101100.2	AT2G39730.2
Potra2n16c30185.1	-1.134661881	-0.280799425	8.69777E-07	0.828371626	Potri.016G101200.1	AT2G37730.1
Potra2n16c30186.1	NA	NA	NA	NA	Potri.016G101300.1	AT3G53820.1
Potra2n16c30187.1	NA	NA	NA	NA	Potri.016G101400.1	AT2G37740.1
Potra2n16c30188.2	-0.447295739	-0.018254236	0.024292304	0.991732307	Potri.016G101700.1	AT3G53830.1
Potra2n16c30189.1	-0.415372966	0.020346973	0.133730731	0.992934677		
Potra2n16c30190.1	-0.629595297	-0.096674911	0.2181202	0.978251387	Potri.006G090000.1	AT1G06150.2

Potra2n16c30191.1	-0.321773049	0.311977643	0.252016191	0.902080412	Potri.016G101900.1	AT2G37750.1
Potra2n16c30192.1	0.264958416	0.291003232	0.727282355	0.950878725	Potri.016G102000.1	AT1G79440.1
Potra2n16c30193.1	3.400848982	1.047694689	2.79294E-29	0.035742858	Potri.016G102100.2	AT2G37770.2
Potra2n16c30194.2	0.260141809	-0.221991801	0.230429814	0.896353602	Potri.016G102300.1	AT2G37770.2
Potra2n16c30195.1	1.664677056	NA	NA	NA		
Potra2n16c30196.1	2.732458522	1.089856667	0.001387477	0.817452127	Potri.016G102500.1	AT2G19130.1
Potra2n16c30197.1	-0.615561017	1.1378613	NA	NA	Potri.016G102400.1	AT5G05987.1
Potra2n16c30198.1	0.291632406	0.15104623	0.871359449	NA		
Potra2n16c30199.1	2.103456023	0.05869522	0.018269763	0.995579852	Potri.016G102600.1	AT2G19130.1
Potra2n16c30200.1	-1.336793218	-0.008707991	NA	NA	Potri.016G103000.2	AT1G04590.1
Potra2n16c30201.1	2.857360497	1.193130634	0.000961382	NA	Potri.016G102600.1	AT2G19130.1
Potra2n16c30202.1	3.135758721	0.800517804	8.47734E-10	0.71411664	Potri.016G102700.1	AT2G19130.1
Potra2n16c30203.1	0.068393532	0.02522325	0.571997396	0.977323635	Potri.016G103000.2	AT1G04590.1
Potra2n16c30204.4	0.715940699	-0.10095554	0.000538469	0.95981322	Potri.016G103100.1	AT2G37860.3
Potra2n16c30205.1	NA	NA	NA	NA	Potri.016G103200.1	AT5G28470.1
Potra2n16c30206.1	NA	NA	NA	NA	Potri.006G091800.1	AT1G52240.2
Potra2n16c30207.1	NA	NA	NA	NA		
Potra2n16c30208.1	0.327639815	0.067971812	0.4455625	0.981726976		
Potra2n16c30209.1	0.633934125	0.040657531	0.010355079	0.985393296	Potri.016G103300.1	AT3G53940.1
Potra2n16c30210.1	0.250712855	-0.002197712	0.521399076	0.999325699	Potri.016G103400.1	AT3G53950.1
Potra2n16c30211.1	0.761084815	0.892284394	NA	0.817452127	Potri.016G103500.1	AT3G53960.1
Potra2n16c30212.1	-0.549854625	-0.192762139	0.003858705	0.833198716	Potri.016G103600.1	AT2G37920.1
Potra2n16c30213.1					Potri.016G103700.1	AT3G09280.1
Potra2n16c30214.3	-0.02055096	0.225778039	0.896155321	0.517743314	Potri.016G103800.2	AT3G53970.1
Potra2n16c30215.1	0.027624255	-0.376457492	0.984054426	0.949960494		
Potra2n16c30216.1						
Potra2n16c30217.1	8.369535206	5.102187082	2.08696E-12	0.323954554	Potri.016G103900.1	
Potra2n16c30218.1	-1.615286612	-0.314204431	6.1791E-08	0.855587395	Potri.016G104300.1	AT3G53980.1
Potra2n16c30219.1	-1.855064301	-0.506592277	1.3204E-12	0.443657809	Potri.016G104300.1	AT3G53980.1
Potra2n16c30220.1	NA	NA	NA	NA	Potri.005G203700.1	AT1G48120.1
Potra2n16c30221.1	-1.772469153	-0.465002763	2.58987E-13	0.518597946	Potri.016G104300.1	AT3G53980.1
Potra2n16c30222.1	-0.767137839	-0.205091046	0.000454225	0.915580711	Potri.016G104400.1	AT5G02010.1

Potra2n16c30223.1	2.235338028	1.885226318	NA	NA		
Potra2n16c30224.1	1.706976536	-0.978296576	0.004752356	0.864349786		
Potra2n16c30225.1	1.835920425	-0.470310238	2.91856E-05	0.92978159	Potri.016G104500.1	AT3G09270.1
Potra2n16c30226.1	1.964097693	-0.219571239	5.38128E-05	0.960656795	Potri.016G104500.1	AT3G09270.1
Potra2n16c30227.1	0.392130252	0.042775779	0.011906624	0.972159498	Potri.016G104600.2	AT3G53990.1
Potra2n16c30228.1	-0.107852595	-0.020707561	0.470167577	0.985393296	Potri.016G104700.1	AT1G60070.1
Potra2n16c30229.1	0.232834873	0.070418003	0.313956249	0.971752582	Potri.016G104900.1	AT2G37840.1
Potra2n16c30230.1	0.815206564	0.184659235	0.038015816	0.773641026	Potri.016G105000.2	AT3G54000.1
Potra2n16c30231.2	0.381354462	0.324292707	1.39572E-06	0.221840363	Potri.016G105100.3	AT3G54010.1
Potra2n16c30232.1	0.272755601	0.121024441	0.287181057	0.941260209	Potri.016G105300.1	AT1G22380.1
Potra2n16c30233.1	0.251256936	0.146400712	0.220166098	0.879538011	Potri.016G105300.1	AT1G22380.1
Potra2n16c30234.1	-1.164767244	-0.183882661	0.014938061	0.932242797	Potri.016G105500.1	AT5G01030.1
Potra2n16c30235.1	-0.238091602	0.091454211	0.402784059	0.953831244	Potri.016G105600.1	AT3G09250.1
Potra2n16c30236.1	0.26607802	-0.058442149	0.157521111	0.954419252	Potri.016G105700.1	AT3G54020.1
Potra2n16c30237.1	1.000511228	-0.252466824	3.63741E-05	0.895474078	Potri.016G105800.1	AT3G54030.1
Potra2n16c30238.1	2.68086091	0.550765581	3.41847E-17	0.803684688	Potri.016G105900.2	AT5G05830.1
Potra2n16c30239.1	1.664677056	NA	NA	NA	Potri.016G107500.1	AT3G09220.1
Potra2n16c30240.1						
Potra2n16c30241.1	2.610910166	1.381953902	NA	NA	Potri.016G107500.1	AT3G09220.1
Potra2n16c30242.1	-1.3100906	-0.418100004	0.095358435	0.919986989	Potri.016G106500.1	AT3G48750.1
Potra2n16c30243.1	-0.148964642	0.105208387	0.569084928	0.940361195	Potri.016G108200.1	AT5G56420.1
Potra2n16c30244.1	-0.289707196	0.185692628	0.269198328	0.881090429	Potri.014G068400.1	AT1G02000.1
Potra2n16c30245.1	-0.507925838	0.429217841	0.07134326	0.645919795	Potri.016G106500.1	AT3G48750.1
Potra2n16c30246.1	0.175885663	0.832282446	0.937197052	NA		
Potra2n16c30247.1	-0.549895762	0.343457618	0.085032013	0.675169873	Potri.015G059700.1	AT5G39040.1
Potra2n16c30248.1	5.220589425	1.871134536	1.92172E-35	0.428801064	Potri.016G106800.1	AT3G54040.1
Potra2n16c30249.1	2.735991243	NA	NA	NA	Potri.002G221800.1	
Potra2n16c30250.1	-0.133957326	0.158650731	0.613414301	0.934439727	Potri.016G106900.1	AT3G54050.1
Potra2n16c30251.1	-0.048241412	0.190461619	0.850654071	0.910878765	Potri.006G094600.1	AT2G37960.2
Potra2n16c30252.1	-0.832352547	-0.13166544	8.68647E-08	0.936976982	Potri.016G109200.1	AT5G01090.1
Potra2n16c30253.1	NA	NA	NA	NA	Potri.016G107300.1	AT5G23860.2
Potra2n16c30254.1	0.528844651	0.237191857	0.002634755	0.728883321	Potri.016G109500.1	AT2G37975.1

Potra2n16c30255.1	0.546829386	0.175382221	0.005487585	0.872570395	Potri.016G109400.1	AT2G37970.1
Potra2n16c30256.1	-0.575631992	-0.062555965	0.005970785	0.97137556	Potri.016G109600.1	AT3G54090.1
Potra2n16c30257.2	0.401475362	-0.014931496	0.237438929	0.993589787	Potri.016G109700.1	AT3G54100.1
Potra2n16c30258.1	1.664677056	NA	NA	NA	Potri.016G109800.1	AT2G38000.1
Potra2n16c30259.1	0.231095322	-0.074429526	0.391825549	0.902676973	Potri.016G110000.2	AT1G07380.1
Potra2n16c30260.1	0.254580601	0.101371383	0.058142042	0.917516356	Potri.016G110100.1	AT3G54110.1
Potra2n16c30261.1	-0.39410373	-0.289792718	0.018700702	0.512736781	Potri.016G110200.1	AT3G54120.1
Potra2n16c30262.1	0.247776974	-0.00516125	0.059334859	0.996032145	Potri.016G110400.2	AT2G38025.1
Potra2n16c30263.1	0.203693581	-0.021693897	0.130002735	0.9869326	Potri.016G110500.1	AT2G38040.2
Potra2n16c30264.1	-0.555198556	-0.319550153	0.018705558	0.702720367	Potri.016G110600.1	AT2G38050.1
Potra2n16c30265.1	-0.118947004	0.047303006	0.069376732	0.912814353	Potri.016G110700.1	AT3G09100.2
Potra2n16c30266.1	0.316231331	0.118884372	0.001023346	0.780939753	Potri.016G110800.1	AT3G09090.1
Potra2n16c30267.1	-0.974978877	-0.584190807	0.000183674	0.706078246	Potri.016G110900.1	AT3G09085.1
Potra2n16c30268.2	0.104015689	0.023302873	0.749575067	0.995261215	Potri.016G111000.1	AT2G38060.1
Potra2n16c30269.1	NA	1.363811008	NA	NA	Potri.016G111300.1	
Potra2n16c30270.2	0.167263919	0.11405282	0.429867269	0.901070867	Potri.016G111400.1	AT3G09070.1
Potra2n16c30271.1	-0.162629967	-0.064844434	0.450734996	0.955628098	Potri.016G111600.1	AT3G09060.1
Potra2n16c30272.1	0.180731812	0.131351978	0.655249024	0.949847686	Potri.016G111900.1	AT3G54140.1
Potra2n16c30273.1	-0.49204685	-0.003429853	0.033569945	0.999406573	Potri.016G112000.1	AT2G38080.1
Potra2n16c30274.1	-0.559749932	0.059653185	0.062123319	0.9869326	Potri.016G112100.1	AT2G38080.1
Potra2n16c30275.1	-0.61910233	0.64247635	NA	NA	Potri.016G112200.1	AT5G60010.1
Potra2n16c30276.1	-1.41398974	-0.402474232	2.99249E-07	0.72101822	Potri.016G112300.1	AT2G38090.1
Potra2n16c30277.1	3.572495178	0.893445009	0.025094694	NA	Potri.016G112400.2	AT5G01210.1
Potra2n16c30278.1					Potri.016G112450.1	
Potra2n16c30279.1	-1.328084259	-0.222733562	3.04036E-07	0.913577577	Potri.016G112600.1	AT5G01220.1
Potra2n16c30280.1	-2.957511221	-0.106224648	4.6164E-06	0.976302215	Potri.016G112700.1	AT5G01225.1
Potra2n16c30281.1	-0.233873599	-0.079089099	0.031130855	0.936890993	Potri.016G112800.1	AT1G48900.1
Potra2n16c30282.1	0.621919628	0.262399722	0.000429105	0.742848888	Potri.016G112900.1	AT3G09030.1
Potra2n16c30283.1	1.026083512	0.378946954	0.19911655	0.945454119	Potri.016G113100.1	AT2G38110.1
Potra2n16c30284.1	-0.106275117	-0.123321103	0.443396922	0.890978957	Potri.016G113200.2	AT4G35335.1
Potra2n16c30285.1	-1.003138257	-0.384093557	2.62475E-08	0.642397767	Potri.016G113300.1	AT4G00430.1
Potra2n16c30286.1	0.164782439	0.115193983	0.412552416	0.907552101	Potri.016G113500.1	AT1G03430.1

Potra2n16c30287.1	1.701226334	1.94749474	0.564206811	NA		
Potra2n16c30288.1	NA	1.571529889	NA	NA	Potri.014G176800.1	
Potra2n16c30289.1	1.64774763	1.571529889	NA	NA		
Potra2n16c30290.1	-0.41961966	0.034543018	0.021200202	0.982221696	Potri.016G113600.1	AT2G38120.1
Potra2n16c30291.1	-0.425117376	-0.062177971	0.111009278	0.974275337	Potri.016G113700.1	AT2G38140.1
Potra2n16c30292.1	-0.233378494	-2.376614165	0.913486876	NA	Potri.011G150700.1	AT1G32990.1
Potra2n16c30293.1	0.448862056	-0.04119545	0.235791663	0.989304496	Potri.016G113800.2	AT1G74720.1
Potra2n16c30294.1	-0.035786978	0.131354551	0.927181073	0.920141992	Potri.016G114100.1	
Potra2n16c30295.1	-0.656488349	0.350743089	0.552824267	0.926808461	Potri.016G114000.1	
Potra2n16c30296.1	5.465962096	0.378467958	2.11685E-05	NA	Potri.016G114200.1	AT3G09020.1
Potra2n16c30297.1	NA	NA	NA	NA	Potri.013G052800.1	AT2G20340.1
Potra2n16c30298.1					Potri.005G025000.1	AT5G03980.1
Potra2n16c30299.1	-0.688652026	-1.55593228	NA	NA		
Potra2n16c30300.1	NA	NA	NA	NA		
Potra2n16c30301.2	-1.183920418	0.029958059	8.20639E-10	0.991089517	Potri.016G114500.2	AT5G01260.2
Potra2n16c30302.1	-0.7303383	0.002439115	2.576E-11	0.999325699	Potri.016G114600.1	AT5G01270.1
Potra2n16c30303.1	-0.707935506	-0.102691571	3.50929E-06	0.900823365	Potri.016G114700.1	AT2G40060.1
Potra2n16c30304.1	-0.377043435	-0.353800181	0.777618386	0.954419252		
Potra2n16c30305.1	0.161439177	0.310123134	0.360313588	0.171550914	Potri.016G114800.1	AT3G09010.1
Potra2n16c30306.1	1.059652036	0.150165902	2.19788E-09	0.897285619	Potri.016G114900.1	AT3G09000.1
Potra2n16c30307.6	-0.153377293	-0.010499276	0.522872709	0.996032145	Potri.016G115100.1	AT3G08980.1
Potra2n16c30308.1	0.130368126	0.052334004	0.527468932	0.983944804	Potri.016G115200.1	AT3G08940.2
Potra2n16c30309.1	-0.026063441	-0.265923112	0.912143431	0.804796567	Potri.016G115300.1	AT5G01520.1
Potra2n16c30310.1	-0.146204879	0.073147852	0.435491465	0.936625864	Potri.016G115400.1	AT5G01500.1
Potra2n16c30311.1	-0.569328898	-0.588983718	0.407222578	0.91753191	Potri.016G115500.1	AT3G51860.1
Potra2n16c30312.2	1.533237582	-0.42726822	0.168320107	0.948002367	Potri.016G115600.1	
Potra2n16c30313.1	-0.547573144	0.17418094	0.248791402	0.964766975	Potri.016G115800.1	AT3G11210.1
Potra2n16c30314.1	1.243198561	0.770964896	0.007090333	0.745178903	Potri.016G116100.1	AT3G11210.1
Potra2n16c30315.1	-0.103600971	-0.026889663	0.462129748	0.98177802	Potri.016G116200.1	AT5G01460.1
Potra2n16c30316.1	0.321490042	0.378696888	0.00376061	0.47454828	Potri.016G116300.1	AT5G01450.1
Potra2n16c30317.1	-0.038939554	-0.054716095	0.820981164	0.948577865	Potri.016G116400.1	AT5G01410.1
Potra2n16c30318.1	0.10034335	0.114703134	0.404309533	0.869186333	Potri.016G116600.1	AT5G01400.1

Potra2n16c30319.1	-0.838451767	-0.080934862	0.020122907	0.983848383	Potri.016G116700.1	AT3G55960.1
Potra2n16c30320.1					Potri.016G116800.1	
Potra2n16c30321.1	0.008940612	0.014338989	0.941162057	0.990672454	Potri.016G117000.1	AT2G01060.1
Potra2n16c30322.1	1.359551673	-0.15666525	0.001498074	0.959881296	Potri.016G117100.1	AT5G05600.1
Potra2n16c30323.1					Potri.010G010300.1	
Potra2n16c30324.1	-0.570491719	-0.275493342	0.00131249	0.744977879		
Potra2n16c30325.1	-0.493379691	-0.203490153	0.003772238	0.842247476	Potri.016G117200.1	AT3G51850.1
Potra2n16c30326.1	NA	NA	NA	NA		
Potra2n16c30327.1	-0.202892044	0.714321846	0.726966257	0.362546677	Potri.016G117300.1	AT5G01380.1
Potra2n16c30328.1	2.853544131	1.191305378	8.34104E-13	0.522715829	Potri.016G117400.1	AT5G01370.1
Potra2n16c30329.1	2.849139702	1.191387996	1.31989E-12	0.556625172		
Potra2n16c30330.4	0.627481578	0.020664322	0.04489235	0.995579852	Potri.016G117500.5	AT5G05580.1
Potra2n16c30331.1	0.445714537	0.059208102	0.005746711	0.972159498	Potri.016G117600.1	AT3G47300.1
Potra2n16c30332.1	0.795801161	0.308639259	4.50482E-11	0.563998615	Potri.016G117800.1	AT5G05570.2
Potra2n16c30333.1	0.242048653	0.142287901	0.182764643	0.932242797	Potri.016G118000.1	AT3G51840.1
Potra2n16c30334.1	0.566692574	0.89564114	0.88592903	NA	Potri.016G118200.1	
Potra2n16c30335.1	-0.539405688	-0.277014792	0.000314093	0.733303543	Potri.016G118300.1	AT3G51830.1
Potra2n16c30336.1	-0.579739172	-0.652744327	0.195164243	0.726986309	Potri.001G363220.1	AT1G63880.1
Potra2n16c30337.1	-0.342430251	-0.999028766	0.344703351	0.012728814	Potri.016G118400.1	AT4G33270.1
Potra2n16c30338.1	0.379633306	0.047914457	0.023919363	0.959881296	Potri.016G118700.1	AT3G08950.1
Potra2n16c30339.1	-0.274012896	-0.185633396	0.088146315	0.812920176	Potri.016G118800.1	AT5G01350.1
Potra2n16c30340.1	-1.317292482	-1.465929376	NA	NA		
Potra2n16c30341.1	NA	NA	NA	NA	Potri.016G118900.1	AT5G60440.1
Potra2n16c30342.1	-0.895621808	-0.112398478	0.004169882	0.970817579	Potri.016G119100.1	AT5G01360.1
Potra2n16c30343.1	-0.137620333	-0.160038475	0.496725742	0.856660279	Potri.016G119200.1	AT2G38270.1
Potra2n16c30344.1	0.200041736	-0.057879773	0.179770106	0.953177702	Potri.016G119300.10	AT2G38280.1
Potra2n16c30345.2	-0.539033899	-0.139077504	0.003610453	0.903477395	Potri.016G119400.1	AT3G51820.1
Potra2n16c30346.1	0.703823496	0.322353766	0.000175173	0.794596445	Potri.016G119500.1	AT5G01340.1
Potra2n16c30347.2	-0.2888708	-0.116736035	0.007169808	0.839117844	Potri.016G119600.1	AT2G30780.1
Potra2n16c30348.2	0.613814359	0.031195178	1.47271E-06	0.9761362	Potri.006G102200.1	AT3G51800.3
Potra2n16c30349.1	-0.76241031	-0.204702479	2.39052E-12	0.704368358	Potri.016G119800.1	AT5G58590.1
Potra2n16c30350.1	-0.039493697	-0.000425454	0.782575971	0.999773567	Potri.016G119900.5	AT3G08960.1

Potra2n16c30351.1	0.394213701	0.304026988	0.055411424	0.706051231	Potri.016G120000.1	AT3G08970.1
Potra2n16c30352.1	1.340407035	-0.006891745	7.5681E-07	0.998245864	Potri.016G120100.2	AT4G33070.1
Potra2n16c30353.1	0.111160387	0.082623155	0.377762647	0.942409925	Potri.016G120200.1	AT5G01310.1
Potra2n16c30354.1	0.114949775	-0.176023235	0.876714218	0.956889095		
Potra2n16c30355.1	0.753363469	-0.31661989	0.119911656	0.930448915	Potri.016G120600.1	AT1G45616.1
Potra2n16c30356.1	2.943350736	0.918671753	1.37781E-07	0.792511865	Potri.016G120600.1	AT1G45616.1
Potra2n16c30357.1	0.09475492	-0.086857362	0.810662793	0.974367223	Potri.004G117600.1	
Potra2n16c30358.1	1.214943587	0.56642737	0.000749233	0.48299238	Potri.016G120600.1	AT1G45616.1
Potra2n16c30359.1	1.664452371	0.886065218	7.30856E-07	0.466558048	Potri.016G120600.1	AT1G45616.1
Potra2n16c30360.1	1.142819756	0.728264491	3.70239E-05	0.384289538	Potri.016G120600.1	AT1G45616.1
Potra2n16c30361.1	4.464580055	2.085764375	7.34024E-37	0.06736252	Potri.016G120700.1	AT5G61520.1
Potra2n16c30362.1	3.110343611	NA	NA	NA	Potri.016G120800.1	AT5G01310.1
Potra2n16c30363.1	0.530855539	-0.074719032	3.30011E-05	0.948577865	Potri.016G120900.1	AT3G53580.1
Potra2n16c30364.1	0.436960773	0.33898621	1.32645E-05	0.456112976	Potri.016G121100.1	AT1G80930.1
Potra2n16c30365.1	NA	2.253733581	NA	NA	Potri.004G135300.1	AT4G15093.1
Potra2n16c30366.1	-0.197896369	-0.094887238	0.553248411	0.97137556	Potri.T045400.1	AT4G15093.1
Potra2n16c30367.1	NA	NA	NA	NA		
Potra2n16c30368.1	-0.61910233	-0.767708831	NA	NA		
Potra2n16c30369.1	-1.824472673	-0.746519461	0.36730591	NA	Potri.018G135800.1	AT3G14460.1
Potra2n16c30370.1	-0.173374689	-1.897047472	0.758293533	0.059805491		
Potra2n16c30371.1	-0.217249015	0.03568043	0.051711569	0.971539972	Potri.016G121200.1	AT3G51780.1
Potra2n16c30372.1	-0.311524458	0.575571953	0.833433871	0.886633917		
Potra2n16c30373.1	1.411920723	-0.950013605	0.004164793	0.679485917	Potri.016G121400.1	AT2G38290.1
Potra2n16c30374.1	0.23351152	-0.068349583	NA	NA	Potri.016G121500.1	AT1G28960.1
Potra2n16c30375.1	-0.978623135	-0.255897111	0.090479429	0.930620147	Potri.016G121600.2	
Potra2n16c30376.1					Potri.016G121700.1	AT3G58120.1
Potra2n16c30377.1					Potri.016G121800.1	AT2G38300.1
Potra2n16c30378.1	-0.739745365	-0.346579298	0.102855194	0.858481608	Potri.016G121900.1	AT5G01580.1
Potra2n16c30379.1	1.533225613	-0.90048345	0.000375452	0.760012034	Potri.001G155750.1	AT1G21280.1
Potra2n16c30380.1	-1.391735608	-3.815571604	0.612519294	NA	Potri.016G122000.1	AT1G07080.1
Potra2n16c30381.1	0.302218784	-0.014752011	0.093822112	0.991407187	Potri.016G122350.1	AT3G08880.1
Potra2n16c30382.1	0.236173233	-0.023047425	0.351468802	0.988824384	Potri.016G122200.1	AT1G07080.1

Potra2n16c30383.1	NA	NA	NA	NA	Potri.016G122500.1	AT3G03450.1
Potra2n16c30384.1	0.245170784	1.363439077	NA	NA	Potri.T133000.1	AT5G01550.1
Potra2n16c30385.1	-4.528469683	-0.56441724	0.016042209	0.931467389	Potri.016G123300.1	AT5G01550.1
Potra2n16c30386.1	0.27853172	0.321084247	0.088158184	0.704224102	Potri.016G123600.1	AT3G08947.1
Potra2n16c30387.1	-0.288167605	-0.042932502	0.046264766	0.966917036	Potri.016G123800.5	AT3G51770.2
Potra2n16c30388.1	-0.02164751	-0.05515566	0.918572448	0.969370704	Potri.016G123900.2	AT5G01590.1
Potra2n16c30389.3	-0.512469731	0.059885058	0.839187841	NA	Potri.016G124000.1	AT3G51750.1
Potra2n16c30390.2	0.645441104	0.091721734	4.12871E-05	0.948002367	Potri.016G124100.1	AT5G58420.1
Potra2n16c30391.1	-1.110208597	-0.151612528	0.006330943	0.974756511	Potri.016G124400.1	AT1G22380.1
Potra2n16c30392.1	-1.654956887	0.050563664	0.58076009	NA	Potri.016G124400.1	AT1G22380.1
Potra2n16c30393.1	-0.595952689	-1.465929376	NA	NA	Potri.016G124400.1	AT1G22380.1
Potra2n16c30394.1	0.018102558	0.250727306	0.992773786	NA	Potri.016G124500.1	AT1G22380.1
Potra2n16c30395.2	-0.155300971	0.969377999	0.903211987	0.849790859	Potri.016G124700.1	AT5G01600.1
Potra2n16c30396.1	5.448882507	1.297775726	0.004039623	NA	Potri.016G125000.1	AT2G41480.1
Potra2n16c30397.1	3.241544177	1.906059102	NA	NA	Potri.016G125200.1	AT1G07030.1
Potra2n16c30398.1	0.197169128	0.15957323	0.195103175	0.917516356	Potri.016G125300.1	AT3G08890.1
Potra2n16c30399.1	-0.627400856	-0.722058361	0.055423287	0.402947275	Potri.016G125400.1	AT2G38310.1
Potra2n16c30400.1	-0.518586475	0.427622087	0.036869914	0.856141735	Potri.016G125500.1	AT2G38320.1
Potra2n16c30401.3	0.531381409	0.21238412	0.001584907	0.833952007	Potri.016G125600.1	AT5G01620.1
Potra2n16c30402.1	0.389066663	0.170070975	0.015792652	0.881090429	Potri.014G189600.1	AT5G01630.1
Potra2n16c30403.1	-0.789880757	-0.175849794	8.06415E-05	0.848131816	Potri.016G126000.4	AT2G38330.1
Potra2n16c30404.1	-0.559700221	-0.09079867	0.302750137	0.977559521	Potri.016G126100.1	AT2G38340.1
Potra2n16c30405.1	-0.138989736	0.046882179	0.319135475	0.976172519	Potri.016G126200.1	AT3G08780.1
Potra2n16c30406.1	-0.299420068	-0.40403163	0.003932713	0.576548403	Potri.016G126300.1	AT3G51740.1
Potra2n16c30407.1	-0.451372509	0.116373843	0.299728624	0.973545552	Potri.016G126500.3	AT5G01650.1
Potra2n16c30408.1	-0.163843284	-0.00214064	0.376199828	0.999325699	Potri.016G126600.2	AT5G01650.1
Potra2n16c30409.1	-0.700229199	-0.055305345	8.89507E-06	0.974756511	Potri.016G126700.1	AT5G01650.1
Potra2n16c30410.1	-0.21542552	-0.149869134	0.25334222	0.8693465	Potri.016G126800.1	AT5G01650.1
Potra2n16c30411.1	2.393086748	2.094751132	0.364274449	NA	Potri.T092300.1	AT1G47890.1
Potra2n16c30412.1					Potri.016G096100.1	
Potra2n16c30413.1	-0.232208123	-0.005792602	0.803218893	0.999325699	Potri.T092000.1	AT3G05660.1
Potra2n16c30414.1	-2.290272861	-2.501563462	0.110201614	0.318978101	Potri.016G127100.2	AT4G13810.1

Potra2n16c30415.1	-0.114802856	0.009887249	0.561140587	0.99458796	Potri.016G127200.1	AT4G38495.1
Potra2n16c30416.1	0.24700064	-0.0017565	0.132817647	0.999325699	Potri.016G127300.1	AT3G51660.1
Potra2n16c30417.1	-0.106367948	0.053943097	0.396175238	0.965515007	Potri.016G127500.1	AT3G08800.1
Potra2n16c30418.1	0.386139567	0.261071628	0.027207312	0.744977879	Potri.016G127600.1	AT5G01660.1
Potra2n16c30419.1	-0.419718329	-0.344419727	0.035174244	0.670481601	Potri.016G127700.1	AT3G51670.1
Potra2n16c30420.1					Potri.016G127800.1	AT5G01680.1
Potra2n16c30421.1	1.634242068	0.209435603	0.587877951	NA	Potri.016G127900.1	AT5G01700.2
Potra2n16c30422.1	NA	NA	NA	NA	Potri.001G025300.1	AT2G47110.1
Potra2n16c30423.1	-1.169651325	-0.453049595	9.91003E-09	0.610808563	Potri.006G105100.1	AT5G01710.1
Potra2n16c30424.1	-0.28652299	-0.20101764	0.110909457	0.882731785	Potri.016G128200.1	AT2G38480.1
Potra2n16c30425.1	4.320209733	2.147867692	1.52407E-11	0.350242504	Potri.016G128300.1	AT2G38470.1
Potra2n16c30426.1	2.451507307	1.579807254	0.244058769	NA	Potri.T055866.1	
Potra2n16c30427.1	0.625080916	0.573694636	0.052036062	0.5736141	Potri.016G128400.1	AT5G01720.1
Potra2n16c30428.1	-0.247224256	-0.052251674	0.100595129	0.976640014	Potri.T141700.1	AT5G03570.1
Potra2n16c30429.1	0.986462291	0.388523517	1.71455E-12	0.306363984	Potri.016G128700.1	AT2G38450.1
Potra2n16c30430.2	-0.020914536	0.045142615	0.928089398	0.977559521	Potri.016G128900.1	AT3G08820.1
Potra2n16c30431.1						
Potra2n16c30432.1	-0.014329997	0.037363407	0.925960076	0.974264269	Potri.016G129000.1	AT2G38440.1
Potra2n16c30433.1	3.481261199	3.010689165	0.255388217	NA	Potri.016G129100.1	AT3G51680.1
Potra2n16c30434.1	1.883497737	-0.298941909	0.030220899	0.974264269	Potri.016G129400.1	AT5G21280.1
Potra2n16c30435.1					Potri.016G129500.1	AT5G01740.1
Potra2n16c30436.1	-0.637934825	-1.507841271	NA	NA	Potri.016G129600.1	AT5G01740.1
Potra2n16c30437.1	0.5190312	-0.864767952	0.208191382	0.572222785	Potri.016G129700.1	AT5G05350.1
Potra2n16c30438.1	-0.843175331	-0.022322981	0.015725492	0.995579852	Potri.016G129900.1	AT3G56190.1
Potra2n16c30439.6	-0.241404724	0.094601224	0.260608188	0.956865545	Potri.016G130000.2	AT3G45100.1
Potra2n16c30440.1					Potri.016G129500.1	AT5G01740.1
Potra2n16c30441.1	0.82357019	NA	NA	NA	Potri.016G129600.1	AT5G01740.1
Potra2n16c30442.1	0.728632378	-0.758067491	0.087766792	0.76350478	Potri.016G129700.1	AT5G05350.1
Potra2n16c30443.1	-0.56706059	-0.068877822	0.009813885	0.975578749	Potri.016G129900.1	AT3G56190.1
Potra2n16c30444.1	-0.2245822	0.060097126	0.053524066	0.940376196	Potri.016G130000.2	AT3G45100.1
Potra2n16c30445.1	-1.503364133	0.142920482	0.000161131	0.960732602		
Potra2n16c30446.1	-1.266551538	-0.150163702	2.7663E-05	0.905231727	Potri.006G106400.1	AT2G38420.1

Potra2n16c30447.1	NA	1.363811008	NA	NA	Potri.016G130400.1	AT5G01750.2
Potra2n16c30448.1	-0.102720275	-0.186407085	0.665443077	0.894276674	Potri.016G130500.1	AT5G01750.2
Potra2n16c30449.1	-1.15388496	0.723103299	0.291336125	0.901251375	Potri.016G130700.1	AT5G01750.2
Potra2n16c30450.1	-0.121122235	-0.045915614	0.59967634	0.9761362	Potri.016G130500.1	AT5G01750.2
Potra2n16c30451.2	-0.643983393	0.202717727	0.343851515	0.966005919	Potri.016G130700.1	AT5G01750.2
Potra2n16c30452.1	NA	NA	NA	NA		
Potra2n16c30453.1	4.95926069	0.456221177	2.35035E-26	0.938458121	Potri.016G131300.1	AT5G01750.2
Potra2n16c30454.1	0.287507028	-0.405225639	0.832233799	0.937276488	Potri.016G130700.1	AT5G01750.2
Potra2n16c30455.1	-0.554499805	0.641468935	0.324036094	0.884553748	Potri.016G131800.1	AT5G01750.2
Potra2n16c30456.2	0.128277574	0.177600123	0.306170729	0.874808359	Potri.016G131900.1	AT5G01760.1
Potra2n16c30457.1	0.126649055	-0.118701208	0.4914008	0.879168574	Potri.016G132000.1	AT3G08850.1
Potra2n16c30458.1	0.12526731	-0.109248043	0.56551519	0.920555796		
Potra2n16c30459.1	-1.524213697	-0.432479697	0.001513353	0.753052725	Potri.016G132100.1	
Potra2n16c30460.1	-1.536326933	-0.058364858	2.75392E-10	0.955238467	Potri.016G132200.1	AT2G38400.1
Potra2n16c30461.1	-0.458647343	-0.029909169	0.033564189	0.985393296	Potri.016G132300.1	AT5G01780.2
Potra2n16c30462.1	-1.073900894	-0.51880492	7.10504E-12	0.276620704		
Potra2n16c30463.1	-1.03948604	-0.522478459	9.87529E-12	0.284918677	Potri.016G132500.1	AT3G51710.1
Potra2n16c30464.1	-1.670426173	-0.72160732	6.07005E-07	0.232093934	Potri.016G132600.1	AT2G40435.1
Potra2n16c30465.1	-1.351259401	-4.025193955	0.702717904	NA		
Potra2n16c30466.1	0.179804189	0.281887441	0.117652639	0.696755852	Potri.008G163300.2	AT1G04300.1
Potra2n16c30467.1	0.784054852	1.416251546	0.008148119	3.23926E-05	Potri.016G132666.1	AT5G05340.1
Potra2n16c30468.1	5.714366138	2.60306301	0.000229609	NA	Potri.016G132666.1	AT5G05340.1
Potra2n16c30469.1	1.664677056	NA	NA	NA	Potri.016G132800.1	AT5G05340.1
Potra2n16c30470.1	NA	NA	NA	NA	Potri.016G132800.1	AT5G05340.1
Potra2n16c30471.1	8.247486864	2.676364087	1.15113E-11	NA	Potri.016G132900.1	AT5G05340.1
Potra2n16c30472.3	1.1002663	0.445112333	0.001881488	0.782659274	Potri.016G133001.1	AT2G30970.1
Potra2n16c30473.1	-0.605556178	-0.062542747	0.191708273	0.985393296	Potri.016G133200.1	AT2G40475.1
Potra2n16c30474.1	-0.435837626	-0.02224502	0.074392279	0.990916799	Potri.016G133300.1	AT2G38370.1
Potra2n16c30475.1	-0.321664173	-0.242665651	0.043927298	0.846680547	Potri.016G133400.1	AT3G51730.1
Potra2n16c30476.1	1.086391519	0.444119038	0.000221117	0.282828785	Potri.016G133500.1	AT5G58380.1
Potra2n16c30477.1	4.971050794	1.394613675	0.041568257	NA	Potri.016G133600.1	
Potra2n16c30478.1	0.294936198	0.182713093	0.001685034	0.559737084	Potri.016G133700.1	AT3G51650.1

Potra2n16c30479.1	-0.674627964	-0.239547682	0.001443337	0.865611082	Potri.016G133800.1	AT3G10960.1
Potra2n16c30480.1	0.059785745	0.174305203	0.89732847	0.910878765	Potri.016G133900.1	AT5G01820.1
Potra2n16c30481.1	-0.647844368	-0.267623737	0.042960777	0.875559093	Potri.016G134000.1	AT5G01830.1
Potra2n16c30482.1	-0.432798524	-0.430902696	0.045433847	0.68015285	Potri.016G134200.2	AT2G30400.1
Potra2n16c30483.1	-1.121828948	-0.504758005	2.02444E-07	0.678524017	Potri.016G134500.1	AT2G38500.1
Potra2n16c30484.1	-1.283288834	0.235318564	6.45393E-07	0.918971596	Potri.016G134566.1	AT5G58350.1
Potra2n16c30485.3	0.185550341	0.16893605	0.106638309	0.821709751	Potri.016G134700.2	AT3G51610.1
Potra2n16c30486.1	NA	NA	NA	NA	Potri.016G134800.2	AT2G38510.1
Potra2n16c30487.1	-0.419109372	-0.696508581	NA	NA	Potri.016G134800.2	AT2G38510.1
Potra2n16c30488.1	0.577272182	0.106899729	0.001703474	0.920141992	Potri.016G134900.1	AT5G01850.1
Potra2n16c30489.1	0.80792306	0.026467582	0.150452591	0.995579852		
Potra2n16c30490.1	0.262522618	0.094501094	0.059732883	0.915488947	Potri.016G135100.1	AT1G29690.1
Potra2n16c30491.1	NA	0.674963689	NA	NA	Potri.016G135200.1	AT2G40610.1
Potra2n16c30492.1	NA	NA	NA	NA		
Potra2n16c30493.1	1.005879229	0.783296033	0.291742014	0.705803634	Potri.016G135400.1	AT2G38540.1
Potra2n16c30494.1	NA	NA	NA	NA	Potri.016G135500.2	AT2G38540.1
Potra2n16c30495.1	0.712086506	0.424157191	0.854179263	NA	Potri.016G136000.2	AT5G01870.1
Potra2n16c30496.1	-1.409983009	-1.55593228	NA	NA	Potri.016G135900.1	AT2G05790.1
Potra2n16c30497.1	-1.386551159	-0.0696502	0.001028792	0.985393296	Potri.016G136100.1	AT1G23740.1
Potra2n16c30498.1	-1.142778569	-0.329471634	8.19195E-08	0.38572517	Potri.016G136200.1	AT3G10910.1
Potra2n16c30499.1	-0.116909156	-0.570888945	0.944136356	0.949966783	Potri.016G136300.1	
Potra2n16c30500.1	-0.274509222	-0.334975893	0.11474678	0.679485917	Potri.016G136400.1	AT3G08760.1
Potra2n16c30501.2	-0.482381397	-0.476531446	0.002419142	0.347954127	Potri.016G136500.1	AT4G32010.1
Potra2n16c30502.1	0.063220569	-0.097602742	0.778564424	0.972159498	Potri.016G136600.1	AT3G08740.1
Potra2n16c30503.1	0.040155154	0.091799646	0.932394948	0.981772014	Potri.016G136700.1	AT1G62680.1
Potra2n16c30504.1	0.997513525	-1.55593228	NA	NA	Potri.016G136800.2	
Potra2n16c30505.1	-0.445620614	-0.252853255	1.03492E-05	0.602611218	Potri.016G136900.1	AT3G51580.1
Potra2n16c30506.1	-1.116998682	-0.365030768	0.000192904	0.804641567	Potri.016G137000.1	AT5G01890.1
Potra2n16c30507.1	-0.15496439	-0.086169391	0.260655018	0.923475785	Potri.016G137200.1	AT2G38550.1
Potra2n16c30508.3	0.073966103	0.096337092	0.534542057	0.911339141	Potri.016G137300.3	AT3G52850.1
Potra2n16c30509.1	6.186454421	5.086246383	3.84055E-11	0.058798971	Potri.016G137600.1	AT3G48280.1
Potra2n16c30510.1	2.891853652	3.205191788	0.004208477	0.301110935	Potri.016G137400.2	AT3G26320.1

Potra2n16c30511.1	0.329323898	0.128991858	0.008029528	0.857189843	Potri.016G137700.1	AT1G61670.1
Potra2n16c30512.1	4.523042968	2.523861585	3.14077E-15	0.077114891	Potri.016G137600.1	AT3G48280.1
Potra2n16c30513.1	-1.422249225	-0.143075153	0.009227973	0.974782554	Potri.016G137800.1	AT5G43180.1
Potra2n16c30514.1	5.343643163	2.308568784	3.72642E-20	0.389989789	Potri.016G137900.1	AT3G56400.1
Potra2n16c30515.3	-0.145295314	-0.005344225	0.404154476	0.996999319	Potri.016G138000.4	AT5G04930.1
Potra2n16c30516.2	-0.040024524	-0.044174031	0.79826062	0.960656795	Potri.016G138100.1	AT2G38560.1
Potra2n16c30517.1	0.109533319	-0.477415825	0.862936728	0.84487051	Potri.016G138300.1	AT5G01910.1
Potra2n16c30518.1	-0.594850473	-0.183599543	7.00805E-08	0.820074794	Potri.016G138400.1	AT3G08720.1
Potra2n16c30519.1	-1.559033303	-0.376632331	1.12088E-09	0.493244318		
Potra2n16c30520.2	-1.264588222	-0.277291994	8.28766E-07	0.842247476	Potri.016G138600.1	AT5G01930.1
Potra2n16c30521.1	0.364059991	0.089856922	0.150377937	0.953608209	Potri.016G138700.1	AT3G51550.1
Potra2n16c30522.1	-0.182118278	-0.172054764	0.750026669	0.951726178	Potri.006G109900.1	AT5G01930.1
Potra2n16c30523.1	-0.117315265	-0.017503984	0.467290992	0.991818914	Potri.016G138700.1	AT3G51550.1
Potra2n16c30524.1	-1.409983009	1.481675454	NA	NA		
Potra2n16c30525.1	-0.185424339	-0.172096707	0.153452444	0.696743275	Potri.016G138800.1	AT3G08710.1
Potra2n16c30526.1	0.169615069	0.291221388	0.493030045	0.834560473	Potri.016G138900.1	AT5G41700.2
Potra2n16c30527.1	-0.162708235	0.068002684	0.164961419	0.917746944	Potri.016G139000.11	AT2G38580.1
Potra2n16c30528.1	0.284793231	0.096298608	0.047766899	0.930871994	Potri.016G139100.2	AT1G14360.1
Potra2n16c30529.2	-0.92376453	-1.03247934	0.04949543	0.459449783	Potri.016G139200.1	AT5G58300.2
Potra2n16c30530.1	NA	NA	NA	NA	Potri.016G139350.1	
Potra2n16c30531.1	NA	NA	NA	NA	Potri.008G033800.1	
Potra2n16c30532.1	-0.242025381	0.429842783	0.940854462	NA		
Potra2n16c30533.1	-0.287128361	-0.188200644	0.026446243	0.881624318	Potri.016G139500.1	AT3G08670.1
Potra2n16c30534.1	0.160095945	-0.013087081	0.379457278	0.993589787	Potri.006G110800.1	AT2G05840.1
Potra2n16c30535.1	-0.632293127	0.86783561	0.638635714	0.79078676		
Potra2n16c30536.3	-1.560388717	-0.791738015	4.48589E-06	0.379734246	Potri.016G139700.1	AT2G38600.1
Potra2n16c30537.1	-0.263887518	0.117024884	0.195189744	0.924951347	Potri.016G139900.1	AT3G08570.1
Potra2n16c30538.1	-0.316995204	-0.027625263	0.04042634	0.984292161	Potri.016G140000.1	AT1G32070.3
Potra2n16c30539.1	2.011200578	-2.446267299	0.302493093	NA	Potri.T146400.1	AT1G06840.1
Potra2n16c30540.1	2.334317937	0.472509391	1.73178E-20	0.71703421	Potri.016G140300.1	AT5G01950.1
Potra2n16c30541.2	-0.078403151	-0.046214564	0.496764316	0.953831244	Potri.016G140500.1	AT3G08650.2
Potra2n16c30542.1	-0.24136308	-0.142518525	0.098421431	0.875093648	Potri.016G140700.1	AT3G08640.1

Potra2n16c30543.3	-0.087474826	-0.055797828	0.613980192	0.965215635	Potri.016G140800.2	AT2G38610.1
Potra2n16c30544.1	0.173462924	0.05999965	0.239278775	0.957525726		
Potra2n16c30545.1	-0.393679182	-0.14216432	0.080096157	0.893394174	Potri.016G141100.1	AT3G08600.1
Potra2n16c30546.1	-0.413131041	-0.108062058	0.079625087	0.927686227		
Potra2n16c30547.1	NA	NA	NA	NA		
Potra2n16c30548.1	0.008437863	0.153206012	0.955822915	0.879168574	Potri.016G141200.1	AT5G01960.1
Potra2n16c30549.1	0.221962518	0.298168968	0.808331314	0.959881296		
Potra2n16c30550.1	-0.799417385	-0.199241299	6.25159E-05	0.886059841	Potri.016G141300.1	AT5G01970.1
Potra2n16c30551.10	-0.680412023	-0.329908278	0.003875979	0.660636854	Potri.016G141400.1	AT1G20670.1
Potra2n16c30552.2	-0.581720194	-0.064468391	5.72787E-05	0.972402328	Potri.006G112100.1	AT5G01980.1
Potra2n16c30553.1	-0.327839767	0.006517587	0.029150941	0.996999319	Potri.016G141700.1	AT5G01990.1
Potra2n16c30554.1	0.185612723	-0.355760192	0.51173711	0.820074794	Potri.016G141900.1	
Potra2n16c30555.1	5.655603036	2.643485599	2.85002E-30	0.229179989	Potri.016G142000.1	AT4G20780.1
Potra2n16c30556.1	-0.006241319	-0.031542803	0.978525619	0.983944804	Potri.016G142100.1	AT2G28330.1
Potra2n16c30557.1	-2.142333246	-0.404005087	3.29799E-06	0.744452143	Potri.016G142200.1	AT3G05975.1
Potra2n16c30558.1	-1.35927387	-0.097655521	NA	NA		
Potra2n16c30559.1	1.593799849	0.966433657	2.56794E-06	0.23623457	Potri.016G142300.1	AT3G54200.1
Potra2n16c30560.1	0.380694171	0.325950756	0.009398909	0.491784462	Potri.016G142500.1	AT3G23590.1
Potra2n16c30561.1	-0.302524937	0.152707376	0.03024858	0.930598202	Potri.006G113000.1	AT3G54190.1
Potra2n16c30562.1	-0.799950669	0.090237462	1.19363E-05	0.97950444	Potri.016G142700.1	AT2G39840.1
Potra2n16c30563.1	-0.090470215	0.066125862	0.775402078	0.983113084	Potri.016G142800.1	AT2G38620.2
Potra2n16c30564.1	0.73086529	0.038348458	0.000477059	0.976888173	Potri.016G142900.1	AT1G09780.1
Potra2n16c30565.1	0.587832539	0.949382259	0.038895154	NA	Potri.016G143000.1	AT2G38640.1
Potra2n16c30566.1	0.33768075	0.095931444	0.216157059	0.954559968	Potri.016G143100.1	AT3G54210.1
Potra2n16c30567.1	-0.608259496	-0.292251924	1.633E-07	0.458841511	Potri.016G143200.1	AT5G01020.1
Potra2n16c30568.1	0.135988315	0.068023193	0.816786691	0.969000298	Potri.016G143400.1	AT5G01015.1
Potra2n16c30569.1	-1.05679647	-0.0584652	0.643577172	NA	Potri.016G143500.1	AT5G41590.1
Potra2n16c30570.1	-0.223652873	-0.006341066	0.378989026	0.998027171	Potri.016G143600.1	AT2G38670.1
Potra2n16c30571.1	-0.140795998	-0.040222258	0.3746635	0.978203092	Potri.016G143700.1	AT5G01010.2
Potra2n16c30572.1	-0.375738087	-0.257572431	0.640575221	0.948577865		
Potra2n16c30573.1	NA	0.674963689	NA	NA		
Potra2n16c30574.1	3.444993508	1.405941089	0.132663111	NA	Potri.T138100.1	AT3G54220.1

Potra2n16c30575.1	-0.276402724	-0.079106509	0.087560825	0.95671968	Potri.016G144000.2	AT2G38650.1
Potra2n16c30576.1	-1.017153018	-0.208754705	1.3045E-05	0.87909151	Potri.016G144100.1	AT1G79620.1
Potra2n1700s37171.1	NA	NA	NA	NA	Potri.016G039500.1	AT3G50150.1
Potra2n1709s37172.1					Potri.010G007300.1	AT4G18180.1
Potra2n170s34788.1	-0.567503312	0.170561403	0.001028751	0.921099436		
Potra2n170s34789.2	0.119842725	0.200200546	0.686289482	0.903477395	Potri.013G068501.1	AT1G20060.1
Potra2n1715s37173.1	NA	NA	NA	NA	Potri.011G078050.1	AT5G63700.1
Potra2n1717s37174.1	NA	NA	NA	NA		
Potra2n1717s37175.1	NA	NA	NA	NA	Potri.011G078050.1	AT5G63700.1
Potra2n171s34790.1	1.000515909	-0.117805725	0.074118606	0.98177802	Potri.001G335800.1	AT3G04120.1
Potra2n171s34791.1	-0.401727423	-2.899327173	0.70698021	0.286078667	Potri.001G335900.1	AT1G19250.1
Potra2n1721s37176.1	NA	1.571529889	NA	NA	Potri.019G047300.2	AT5G16750.1
Potra2n1728s37177.1					Potri.014G138700.1	AT2G48160.1
Potra2n1736s37178.1	1.545832463	2.676364087	NA	NA	Potri.012G109200.1	AT5G61840.1
Potra2n1741s37179.1	-1.310869354	-0.343273039	0.005893262	0.828847087	Potri.011G150800.1	AT3G61460.1
Potra2n1743s37180.1						
Potra2n1751s37181.1	1.852666123	1.990518358	0.00051629	0.295777432	Potri.009G038300.2	AT5G59080.1
Potra2n1752s37182.1	-1.646742478	-1.079632722	0.512939662	NA	Potri.006G004400.1	AT5G47720.2
Potra2n1764s37183.1	NA	NA	NA	NA		
Potra2n1768s37184.1	-1.816797375	0.215171798	0.091174101	0.959881296	Potri.018G094100.1	AT5G57510.1
Potra2n177s34792.1	-0.218701429	-0.083302999	NA	NA		
Potra2n177s34793.1	0.60406314	0.366072775	0.010888201	0.849790859	Potri.T129332.1	AT5G17680.1
Potra2n177s34794.1	1.092109233	0.696802531	0.132043427	0.855245045	Potri.T073500.1	AT5G15700.1
Potra2n177s34795.1	-0.147643223	0.124241523	0.879564331	0.9761362	Potri.014G064300.2	AT5G17680.1
Potra2n177s34796.1	0.431619302	0.857602635	0.811166206	0.901251375	Potri.T129400.1	AT4G12010.1
Potra2n177s34797.1	3.488755237	3.020443752	0.399930986	NA	Potri.017G105300.1	AT5G17680.1
Potra2n178s34798.1	-0.650901866	0.247948511	0.071228159	0.933286951	Potri.001G409600.1	AT5G54585.1
Potra2n178s34799.1	1.664677056	0.674963689	NA	NA	Potri.001G176100.1	AT1G52800.1
Potra2n178s34800.1	5.27403982	3.210436129	6.07005E-07	0.322673812	Potri.001G415100.3	AT5G23960.2
Potra2n178s34801.1						
Potra2n178s34802.1	NA	NA	NA	NA		
Potra2n179s34803.1						

Potra2n179s34804.1	0.902462303	1.188962642	0.754290721	NA		
Potra2n179s34805.1	5.333939385	1.071788533	4.55663E-05	0.937687311	Potri.T080900.1	AT1G16260.2
Potra2n17c30577.2	-0.709585796	-0.159393865	0.00455277	0.926808461	Potri.017G155300.1	AT1G17840.1
Potra2n17c30578.1	3.336254291	2.41339897	5.96438E-15	0.13137548	Potri.017G155200.1	AT1G17830.1
Potra2n17c30579.3	0.44918	0.278158184	0.000325215	0.281656787	Potri.017G155000.1	AT1G17820.1
Potra2n17c30580.1	0.147552085	0.309073484	0.590349871	0.862335622	Potri.017G154900.1	
Potra2n17c30581.1	0.720608652	0.260398178	0.330323017	0.956865545	Potri.017G154800.1	AT1G17810.1
Potra2n17c30582.1	1.64774763	1.571529889	NA	NA	Potri.017G154700.1	AT1G48480.1
Potra2n17c30583.1	1.545832463	NA	NA	NA	Potri.017G154600.3	AT1G73340.1
Potra2n17c30584.1	NA	NA	NA	NA	Potri.004G066900.1	AT1G28150.1
Potra2n17c30585.3	0.118479618	-0.10247631	0.313760194	0.903934429	Potri.017G154400.2	AT3G21350.1
Potra2n17c30586.1	NA	0.674963689	NA	NA		
Potra2n17c30587.1	-0.619029418	-0.111731378	0.000327044	0.855587395	Potri.017G154200.3	AT1G73390.2
Potra2n17c30588.1						
Potra2n17c30589.1						
Potra2n17c30590.1	-0.057990257	0.00141557	0.7504119	0.999325699	Potri.017G153900.2	AT1G73320.1
Potra2n17c30591.1					Potri.017G153300.1	AT1G17860.1
Potra2n17c30592.1	-0.925491616	-0.234899009	0.370598291	0.953608209	Potri.017G153700.1	AT2G29760.1
Potra2n17c30593.1	-0.748060972	-0.204894897	0.492779378	0.958152146	Potri.017G153900.2	AT1G73320.1
Potra2n17c30594.1	NA	NA	NA	NA	Potri.017G153300.1	AT1G17860.1
Potra2n17c30595.1	NA	NA	NA	NA	Potri.017G153300.1	AT1G17860.1
Potra2n17c30596.1					Potri.017G153300.1	AT1G17860.1
Potra2n17c30597.1	0.304144315	0.233168003	0.503526114	0.939653927	Potri.017G153200.1	AT1G17860.1
Potra2n17c30598.1	2.307661509	0.412006597	1.92101E-09	0.914551931	Potri.017G152900.1	AT5G23360.1
Potra2n17c30599.1	0.286935451	0.228174198	0.246948025	0.874808359		
Potra2n17c30600.1	-1.096550186	-0.080609651	7.36695E-11	0.946871883	Potri.017G152800.1	AT5G14940.1
Potra2n17c30601.2	-0.33141888	0.046602631	0.012519552	0.96670549	Potri.004G068700.1	AT1G73210.1
Potra2n17c30602.1	1.64774763	NA	NA	NA		
Potra2n17c30603.1	6.783389301	3.013952057	2.74361E-08	NA	Potri.017G150560.1	
Potra2n17c30604.1	NA	NA	NA	NA	Potri.017G151300.2	
Potra2n17c30605.1	0.112290023	0.030138779	0.716728846	0.985964146	Potri.017G151200.1	AT1G65730.1
Potra2n17c30606.1	0.379912014	0.672477611	0.27442011	0.478429061	Potri.017G150300.1	

Potra2n17c30607.1						
Potra2n17c30608.2	0.330538181	0.136576836	0.077037628	0.901195443	Potri.017G151101.1	AT1G65730.1
Potra2n17c30609.1	0.061374575	0.446181784	0.928021614	0.88643982	Potri.017G150200.1	AT1G48380.1
Potra2n17c30610.1	3.500594658	0.660619236	0.129838367	NA		
Potra2n17c30611.1	0.669397143	0.209514823	8.01384E-06	0.912777843	Potri.017G150100.1	AT3G16520.3
Potra2n17c30612.1	1.525485923	0.444793892	4.94747E-14	0.525145639	Potri.017G150000.1	AT3G16520.3
Potra2n17c30613.1	-0.004268069	0.042529388	0.974831169	0.963878456	Potri.017G149900.1	AT1G51160.1
Potra2n17c30614.1	0.186644202	0.091193205	0.069333371	0.89521294	Potri.017G149800.1	AT4G11260.1
Potra2n17c30615.1	0.276624733	0.089735022	0.036352374	0.924251575	Potri.017G020600.1	AT2G43500.2
Potra2n17c30616.1	-1.055232709	-0.840421739	0.478072645	NA		
Potra2n17c30617.1	-0.595952689	-1.465929376	NA	NA	Potri.017G149600.1	
Potra2n17c30618.1	NA	2.690879854	NA	NA		
Potra2n17c30619.1	NA	NA	NA	NA	Potri.017G149200.1	
Potra2n17c30620.1	-0.178717413	-0.055287152	0.180973298	0.953831244	Potri.017G149100.1	AT5G52840.1
Potra2n17c30621.3	-2.032553271	-0.515691396	2.77202E-12	0.376293157	Potri.017G149000.1	AT5G52830.1
Potra2n17c30622.1	-0.132058445	-0.123740077	0.335126284	0.903477395	Potri.017G148900.1	AT5G62650.1
Potra2n17c30623.1	0.083757418	-0.058771849	0.447626891	0.945454119	Potri.017G148800.1	AT1G79940.2
Potra2n17c30624.1	-1.135782065	-0.372942873	1.77634E-12	0.054508175	Potri.017G148700.1	AT4G21190.1
Potra2n17c30625.1	-1.268212825	-0.447284489	1.66328E-08	0.111190854		
Potra2n17c30626.1	-1.091028281	-0.276109562	3.0875E-08	0.843683654	Potri.017G148600.1	AT5G24060.2
Potra2n17c30627.1	0.696668146	-1.507841271	NA	NA	Potri.017G087100.1	
Potra2n17c30628.1	-0.637934825	-1.507841271	NA	NA	Potri.017G148400.1	
Potra2n17c30629.1	0.245170784	0.717104871	NA	NA	Potri.017G148400.1	
Potra2n17c30630.1	5.228288391	1.587716192	0.00010126	NA	Potri.T147000.1	
Potra2n17c30631.1	2.153810496	0.89274633	8.08327E-23	0.03366305	Potri.004G071600.3	AT2G01570.1
Potra2n17c30632.2	-0.19251825	-0.0715746	0.130780242	0.947547562	Potri.017G148100.1	AT5G37930.1
Potra2n17c30633.1	-0.241683695	0.813612582	0.942058875	NA		
Potra2n17c30634.1	0.185721494	-0.230892275	0.721315153	0.936890993	Potri.017G148000.1	AT5G52800.3
Potra2n17c30635.2	0.909026372	-0.757196931	NA	NA	Potri.017G147900.2	AT5G52790.1
Potra2n17c30636.1	-0.203618325	1.145232167	0.879754941	0.79477084	Potri.017G147700.1	
Potra2n17c30637.1						
Potra2n17c30638.1	9.651907103	5.69215125	3.81878E-18	0.205128996	Potri.017G147500.1	AT1G01490.1

Potra2n17c30639.1	3.933813785	2.57305404	3.90243E-10	0.216093729	Potri.017G147400.1	AT1G01490.1
Potra2n17c30640.1	3.255719224	NA	NA	NA	Potri.017G147300.1	AT1G01490.2
Potra2n17c30641.1	3.114341532	NA	NA	NA	Potri.017G147400.1	AT1G01490.1
Potra2n17c30642.1	5.824398351	3.427308461	1.38068E-45	0.118105879	Potri.017G147200.1	AT1G01490.2
Potra2n17c30643.1	0.204482757	0.281567611	NA	NA	Potri.004G195700.1	
Potra2n17c30644.1	5.744660182	3.408857394	1.61707E-46	0.06951573	Potri.017G147200.1	AT1G01490.2
Potra2n17c30645.1	6.564772621	3.874741572	6.53468E-19	0.039351217	Potri.017G147200.1	AT1G01490.2
Potra2n17c30646.1	-0.760349595	-0.402038833	3.23907E-09	0.401169244	Potri.017G146800.1	AT5G52660.1
Potra2n17c30647.1	0.506262201	0.168027021	0.000289802	0.871720501	Potri.017G146700.4	AT4G25740.1
Potra2n17c30648.1	-0.288595342	0.206218456	0.142469049	0.893135869	Potri.017G146600.1	AT5G52640.1
Potra2n17c30649.1	-1.409983009	1.166773781	NA	NA	Potri.017G146500.1	AT5G02920.1
Potra2n17c30650.1	1.071014981	0.264154542	6.11268E-06	0.849815276	Potri.017G146400.2	AT5G02700.1
Potra2n17c30651.1	-0.287164274	-0.412714781	NA	NA	Potri.017G146300.1	AT5G02700.1
Potra2n17c30652.1	-0.270177246	-0.289880228	0.168768471	0.679660122	Potri.017G146200.1	AT4G25720.1
Potra2n17c30653.1	1.147572315	-0.500477915	0.486989186	NA	Potri.017G146000.1	AT1G01540.2
Potra2n17c30654.1	2.653565217	3.110649902	0.336050156	NA	Potri.004G073900.1	AT3G48950.1
Potra2n17c30655.1						
Potra2n17c30656.1	0.105900424	0.128149746	0.711235897	0.924951347	Potri.017G145700.6	AT4G25700.1
Potra2n17c30657.1	0.359458111	0.212328645	0.225740272	0.895560236	Potri.017G145500.1	AT2G37900.1
Potra2n17c30658.1	0.566794132	0.720489646	0.250675244	0.562306981	Potri.T108100.1	AT2G37900.1
Potra2n17c30659.1	-0.926866123	-0.32430667	0.028627617	0.84487051		
Potra2n17c30660.1	-0.081315066	-0.126404532	0.653281989	0.896353214	Potri.017G145100.1	AT4G31980.1
Potra2n17c30661.1	-0.151763728	0.242080912	0.871209374	0.957929035	Potri.017G145000.2	AT3G14470.1
Potra2n17c30662.1	0.008319615	-0.087310343	0.967072967	0.924951347	Potri.017G144900.1	AT2G32230.1
Potra2n17c30663.1	-0.332873854	0.436440727	0.112566142	0.82965307	Potri.017G144800.1	AT5G17300.1
Potra2n17c30664.1	0.395654544	1.280305162	0.008020328	0.148037164	Potri.017G144700.1	AT5G17310.2
Potra2n17c30665.1	NA	NA	NA	NA	Potri.004G074800.1	AT1G73360.1
Potra2n17c30666.1	NA	NA	NA	NA		
Potra2n17c30667.1	0.440530832	0.088560636	6.08325E-06	0.937044511	Potri.T059000.2	AT1G65930.1
Potra2n17c30668.1	0.144988814	-0.043370953	0.604750662	0.985393296	Potri.T059200.1	AT5G17330.1
Potra2n17c30669.1	1.664677056	NA	NA	NA	Potri.004G075300.1	AT5G17330.1
Potra2n17c30670.1					Potri.T059400.2	AT4G24975.1

Potra2n17c30671.1	NA	NA	NA	NA	Potri.T059500.1	AT3G03270.2
Potra2n17c30672.1	-0.19766253	-0.082924825	0.282522539	0.957877578	Potri.T059700.5	AT2G01970.1
Potra2n17c30673.1	-0.237775321	0.103095363	0.074417635	0.900015282	Potri.T059800.2	AT5G37370.3
Potra2n17c30674.1	-1.025216349	-0.266825988	3.80256E-09	0.809323756	Potri.T059900.1	AT5G37478.1
Potra2n17c30675.4	-0.997054863	-0.339074033	0.001328705	0.859892315	Potri.T060000.1	AT1G65840.1
Potra2n17c30676.1	-0.312123143	0.166131232	0.03097087	0.846680547	Potri.T060100.1	AT3G02720.1
Potra2n17c30677.1	-0.716782043	0.127210722	0.237153909	0.972159498	Potri.018G017900.1	AT3G14470.1
Potra2n17c30678.1	-0.010850826	0.261467983	0.979134095	0.86716397	Potri.017G143800.1	AT3G14470.1
Potra2n17c30679.1	-0.144139708	0.756126358	0.85806383	0.523216367	Potri.017G143500.1	AT3G14470.1
Potra2n17c30680.1	0.637924908	0.546587283	0.064132029	0.596772738	Potri.017G143400.1	AT3G14470.1
Potra2n17c30681.1	0.43475665	0.560503485	0.306161477	0.719104446	Potri.017G143800.1	AT3G14470.1
Potra2n17c30682.6	-0.94447691	-0.396033566	0.002952938	0.745183467	Potri.T060000.1	AT1G65840.1
Potra2n17c30683.1	-0.289989709	0.231783772	0.247673034	0.833952007	Potri.T060200.2	AT3G02720.1
Potra2n17c30684.1	0.400918076	0.517168681	0.556293318	0.859892315	Potri.017G143500.1	AT3G14470.1
Potra2n17c30685.1	0.643607429	0.601735579	0.219332697	0.456112976	Potri.017G143800.1	AT3G14470.1
Potra2n17c30686.1	-0.158387084	-0.189392916	0.940335996	NA	Potri.017G143800.1	AT3G14470.1
Potra2n17c30687.1	-0.655981112	-0.04840812	2.24746E-05	0.982422288	Potri.017G143200.1	AT4G25670.1
Potra2n17c30688.1	NA	NA	NA	NA		
Potra2n17c30689.1	-0.606533456	-0.229639141	0.000224112	0.809914834	Potri.017G143100.1	AT4G25680.1
Potra2n17c30690.1	-0.043337682	0.077146992	0.928387777	0.9761362	Potri.T061100.1	AT5G24010.1
Potra2n17c30691.1	-1.409983009	-1.55593228	NA	NA	Potri.T061175.1	AT5G24000.1
Potra2n17c30692.1	-0.672930725	0.474024299	0.250217846	0.826906752	Potri.T061200.1	AT5G24000.1
Potra2n17c30693.1	-2.453099188	-2.59744079	NA	NA	Potri.T061175.1	AT5G24000.1
Potra2n17c30694.1	-0.753128817	0.26486571	0.139219394	0.910815461	Potri.017G142900.1	AT5G24000.1
Potra2n17c30695.1	-1.409983009	-1.55593228	NA	NA	Potri.T061175.1	AT5G24000.1
Potra2n17c30696.1	-0.815948533	0.035442717	0.060795301	0.989300076	Potri.017G142900.1	AT5G24000.1
Potra2n17c30697.2	1.459962178	NA	NA	NA	Potri.017G142800.1	AT5G23980.1
Potra2n17c30698.1	0.157851618	-0.208719004	0.641210583	0.896937427	Potri.017G142700.1	AT1G01580.1
Potra2n17c30699.1	0.087397976	0.104011421	0.412445525	0.879168574	Potri.017G142600.2	AT5G52530.1
Potra2n17c30700.1	-0.225775659	-0.121019327	0.149836457	0.939915707	Potri.017G142500.1	AT5G52520.1
Potra2n17c30701.1	-0.133150418	0.314879575	0.651720542	0.865538396	Potri.004G078800.1	AT5G52510.1
Potra2n17c30702.1	-0.275402146	-0.121620127	0.159960952	0.917510096	Potri.017G142300.1	AT3G02700.1

Potra2n17c30703.1	0.556055551	-0.12993377	0.000784475	0.930598202	Potri.017G142200.2	AT3G57360.1
Potra2n17c30704.1	-0.059613974	-0.064821735	0.669706009	0.950095151	Potri.017G142100.1	AT1G65720.1
Potra2n17c30705.1	1.734149248	-0.068349583	0.640974799	NA	Potri.017G141900.1	AT1G02260.1
Potra2n17c30706.1	2.027878785	0.505072126	2.54893E-23	0.759797728	Potri.017G141600.10	AT1G02260.1
Potra2n17c30707.1	-1.316343469	-1.619644824	0.792769718	NA	Potri.017G141800.2	AT1G02260.1
Potra2n17c30708.1	0.82357019	1.363811008	NA	NA	Potri.017G141900.1	AT1G02260.1
Potra2n17c30709.1	6.880854562	4.239419957	1.69659E-07	NA	Potri.017G141600.10	AT1G02260.1
Potra2n17c30710.1	2.285327717	0.599684857	1.49266E-10	0.647178693	Potri.017G141400.3	AT5G40380.1
Potra2n17c30711.1	0.067658159	0.08981821	0.864599082	0.963557834	Potri.017G141200.1	AT5G66520.1
Potra2n17c30712.1	0.354458224	0.105164366	0.001409379	0.903698951	Potri.017G141100.2	AT1G17880.1
Potra2n17c30713.2	0.42693131	0.266236374	0.037494085	0.79549388	Potri.017G141000.1	AT5G37020.1
Potra2n17c30714.1	6.048420962	2.228402577	7.86652E-76	0.204739729	Potri.017G140900.1	AT1G65820.1
Potra2n17c30715.1	0.609876101	0.025728655	0.000759318	0.981702181	Potri.017G140800.1	AT3G16780.1
Potra2n17c30716.1	7.379715672	3.571653041	8.53369E-32	0.328908681	Potri.017G140500.1	AT1G65810.1
Potra2n17c30717.1	-0.056503238	0.408489112	0.902084442	0.836066102	Potri.017G143800.1	AT3G14470.1
Potra2n17c30718.1	-0.259526286	-0.190171341	0.812469509	0.978251387	Potri.017G143400.1	AT3G14470.1
Potra2n17c30719.1	-0.304243707	0.341027046	0.720682476	0.91233528	Potri.017G140100.2	AT1G58602.2
Potra2n17c30720.1	0.559990855	1.340639093	0.862637044	NA	Potri.T060300.1	AT3G14470.1
Potra2n17c30721.1	-2.453099188	-0.23974247	NA	NA	Potri.004G077500.1	AT1G65810.1
Potra2n17c30722.1	-1.036113968	-0.342307553	1.93192E-08	0.410682206	Potri.017G140000.1	AT3G03220.1
Potra2n17c30723.2	-0.750407854	-0.412028846	0.000527882	0.764266707	Potri.017G139800.1	AT3G03210.1
Potra2n17c30724.2	-0.566283778	-0.242643729	0.001167144	0.874808359	Potri.017G139700.1	AT5G17290.1
Potra2n17c30725.1	-0.207383977	-0.21916049	0.423908607	0.921099436	Potri.017G139600.1	AT1G65920.1
Potra2n17c30726.1	2.624109636	1.004773188	0.290714935	NA	Potri.017G139500.1	AT1G65910.1
Potra2n17c30727.1	-0.162749973	-0.036026198	0.096659631	0.974634964	Potri.017G139400.1	AT1G65900.1
Potra2n17c30728.1	-0.288649259	-0.081499569	0.015685309	0.948577865	Potri.017G139300.1	AT1G54320.1
Potra2n17c30729.1	-0.261266764	-0.004274445	0.153473895	0.99859284	Potri.017G139200.1	AT1G06590.1
Potra2n17c30730.2	2.238131073	0.031357384	0.126623681	NA	Potri.017G139100.1	AT5G37180.1
Potra2n17c30731.1	1.64774763	NA	NA	NA	Potri.017G137900.2	AT3G14460.1
Potra2n17c30732.1	1.664677056	NA	NA	NA		
Potra2n17c30733.1	-0.512078484	-0.0996614	0.148863503	0.967940508	Potri.009G078500.1	AT1G74520.1
Potra2n17c30734.7	-1.095893231	-0.347540471	0.000191571	0.779077802	Potri.017G138900.4	AT5G17230.2

Potra2n17c30735.1	NA	NA	NA	NA	Potri.017G138800.1	AT5G17220.1
Potra2n17c30736.1	0.167394359	-0.043626174	0.165461911	0.965801986	Potri.017G138600.1	AT1G27190.1
Potra2n17c30737.4	-0.098922141	-0.09299754	0.455981617	0.868145269	Potri.017G138500.1	AT3G54230.1
Potra2n17c30738.1	3.555239665	0.129979301	0.011253292	NA	Potri.017G138400.1	AT1G65880.1
Potra2n17c30739.1						
Potra2n17c30740.1	0.494069249	0.301383239	0.532021979	0.948577865	Potri.T116400.1	AT5G37475.1
Potra2n17c30741.1	-1.945042539	-1.126419943	0.053722495	0.823422145	Potri.017G137851.1	AT5G44510.1
Potra2n17c30742.1	-0.031370898	0.164792872	0.923907254	0.832750076		
Potra2n17c30743.7	-0.002421909	0.2363734	0.994166747	0.662187972	Potri.T105500.1	AT3G14470.1
Potra2n17c30744.1	1.459962178	1.571529889	NA	NA	Potri.017G137600.1	AT5G16560.1
Potra2n17c30745.1	-0.731734949	1.977340268	NA	NA	Potri.T105200.1	AT5G19820.1
Potra2n17c30746.1	0.437125659	0.253681114	0.00034264	0.903698951	Potri.017G137300.1	AT1G66130.1
Potra2n17c30747.1	0.00369557	-0.118541265	0.993745022	0.972159498	Potri.017G137200.1	AT3G14330.1
Potra2n17c30748.1	-1.461571035	-1.133472114	0.211884377	0.875838685	Potri.017G137100.1	AT5G55490.1
Potra2n17c30749.1	NA	NA	NA	NA		
Potra2n17c30750.1	0.673034099	0.217530701	0.049999334	0.910074792	Potri.017G136900.1	AT3G14460.1
Potra2n17c30751.1	1.346054597	-4.897434431	0.078893791	NA	Potri.T096050.1	AT3G48690.1
Potra2n17c30752.1	NA	NA	NA	NA		
Potra2n17c30753.1	-1.317292482	-1.465929376	NA	NA	Potri.017G133600.1	AT3G14460.1
Potra2n17c30754.1	-0.944873925	-0.508633485	9.22504E-06	0.349324124	Potri.T116200.1	AT3G14460.1
Potra2n17c30755.1	-0.347699305	-0.142373296	0.519815671	0.972159498	Potri.018G080632.1	
Potra2n17c30756.1	0.216565131	1.281890655	NA	NA	Potri.004G085400.1	AT5G37600.1
Potra2n17c30757.1	-2.798197216	-2.202845644	0.395831471	NA	Potri.017G137100.1	AT5G55490.1
Potra2n17c30758.1	NA	NA	NA	NA		
Potra2n17c30759.1	-0.128102407	0.063932139	0.59907433	0.954213133	Potri.018G080500.1	AT3G14460.1
Potra2n17c30760.1	0.150997256	0.215250748	0.186587791	0.767368598	Potri.018G080400.1	AT5G37510.1
Potra2n17c30761.1					Potri.002G040700.1	AT1G06650.2
Potra2n17c30762.1	0.036337671	0.621223823	0.890682821	0.70570456	Potri.017G135800.2	AT1G06620.1
Potra2n17c30763.1	-0.231071191	0.570173394	0.934114049	NA	Potri.017G136000.1	AT1G06620.1
Potra2n17c30764.1	-0.169963243	0.087651126	0.332342059	0.921099436	Potri.017G135600.2	AT4G05320.2
Potra2n17c30765.1	-0.268280253	-0.074835788	0.164285163	0.943483421	Potri.017G135500.1	AT3G02860.2
Potra2n17c30766.1	3.478042013	0.717104871	0.06606595	NA	Potri.017G135400.1	AT3G02850.1

Potra2n17c30767.1	6.4367925	2.31492623	2.4749E-42	0.679365931	Potri.017G135300.1	
Potra2n17c30768.2	-0.790607855	-1.031051285	0.069991417	0.594980124	Potri.017G135200.1	AT1G66170.1
Potra2n17c30769.1	1.209972233	-0.017956977	0.026266497	0.996032145	Potri.017G135000.1	AT5G37490.1
Potra2n17c30770.1	-0.56400824	1.055293736	0.352685104	0.500705998	Potri.017G134900.1	AT1G66150.1
Potra2n17c30771.1	2.911270357	2.906556465	NA	NA		
Potra2n17c30772.1	0.171684468	0.149140456	0.620554519	0.943483421	Potri.017G134800.1	AT1G24625.1
Potra2n17c30773.1	2.20156813	0.671574109	0.061610548	0.948577865	Potri.T095200.1	
Potra2n17c30774.1	2.918219423	NA	NA	NA	Potri.T095200.1	
Potra2n17c30775.1	3.219950245	3.010689165	NA	NA	Potri.017G134200.1	AT2G26560.1
Potra2n17c30776.1	NA	NA	NA	NA	Potri.T095500.1	AT2G26560.1
Potra2n17c30777.1	0.160330343	-0.002026693	0.67670411	0.999458638	Potri.017G131400.2	AT1G09680.1
Potra2n17c30778.1	-2.454419314	-1.528262191	0.000122906	0.051466421	Potri.017G131300.1	AT5G37550.1
Potra2n17c30779.1	1.384701335	0.736349858	1.95608E-05	0.695304875	Potri.T105600.1	AT3G14460.1
Potra2n17c30780.1	0.964498495	0.553895537	5.08185E-07	0.256687897	Potri.017G128000.1	AT3G14460.1
Potra2n17c30781.1	0.560359112	0.562046316	0.077091693	0.32405241	Potri.T116200.1	AT3G14460.1
Potra2n17c30782.1	0.665709041	0.521373873	0.008163181	0.180107654	Potri.T116200.1	AT3G14460.1
Potra2n17c30783.1	0.97578239	0.659067543	0.002411781	0.329167823	Potri.017G127200.1	AT3G14460.1
Potra2n17c30784.1					Potri.012G139400.2	AT2G36620.1
Potra2n17c30785.1	-2.03156588	-0.484895391	0.01911638	0.936890993	Potri.017G131100.1	AT5G37600.1
Potra2n17c30786.1	1.201766839	0.874626792	0.055487155	0.650936786	Potri.017G131000.1	AT2G01670.1
Potra2n17c30787.2	-0.69535066	-0.183713363	0.045251862	0.91540496	Potri.017G130900.1	AT3G14470.1
Potra2n17c30788.1	NA	NA	NA	NA	Potri.019G071000.1	AT5G53390.1
Potra2n17c30789.1	-1.598198019	0.221881258	0.06349761	0.959009636	Potri.017G130800.1	AT5G37660.2
Potra2n17c30790.1	-0.870743592	-0.069328044	7.79859E-05	0.97137556	Potri.017G130700.2	AT5G37670.1
Potra2n17c30791.1	-0.740691086	-0.306801689	5.05462E-06	0.830470159	Potri.017G130600.1	AT1G48480.1
Potra2n17c30792.1	-0.163516332	-0.030393947	0.407318012	0.974875259	Potri.017G130500.1	AT5G37680.1
Potra2n17c30793.2	-0.123067833	-0.105988442	0.431589107	0.869327705	Potri.017G130400.1	AT5G16610.2
Potra2n17c30794.1	-0.168304692	0.086296563	0.553192663	0.959881296	Potri.017G130300.1	AT5G16600.1
Potra2n17c30795.1	-0.383805633	0.319407428	0.032439013	0.725595998	Potri.017G130200.1	AT1G66250.1
Potra2n17c30796.1	-0.404990853	0.076439931	0.80009035	0.992934677	Potri.017G130100.1	AT5G37690.1
Potra2n17c30797.1	-0.967426466	-0.311496426	1.75972E-06	0.570154529	Potri.017G130000.1	AT5G37710.1
Potra2n17c30798.2	0.170497857	0.056435807	0.287649245	0.935474933	Potri.017G129900.2	AT1G66260.1

Potra2n17c30799.3	-0.210344054	0.015705922	0.106213658	0.9896961	Potri.017G129800.1	AT5G16620.1
Potra2n17c30800.1	-0.456273935	-0.295959546	0.4310373	0.949966783	Potri.017G129700.1	AT2G01918.1
Potra2n17c30801.1	0.337461048	-0.034089676	0.112566142	0.984292161	Potri.017G129500.1	AT1G23890.2
Potra2n17c30802.1	-0.684447857	0.085204463	0.007291934	0.976302215	Potri.017G129400.1	AT1G14900.1
Potra2n17c30803.2	0.254889925	0.316500942	0.108772844	0.817452127	Potri.017G129300.1	AT3G17940.1
Potra2n17c30804.1	-0.103852978	0.644045717	0.825578035	0.706051231	Potri.017G129200.1	AT1G70750.1
Potra2n17c30805.1	-0.17404778	0.787460208	0.353089435	NA	Potri.017G129100.10	AT1G66330.2
Potra2n17c30806.5	0.955389187	0.198613799	1.41985E-08	0.78270394	Potri.017G129000.5	AT5G37850.2
Potra2n17c30807.1	NA	NA	NA	NA		
Potra2n17c30808.1	0.099347103	0.027203303	0.824198873	0.990128003	Potri.017G128900.1	AT4G09460.1
Potra2n17c30809.1	0.602329047	0.437865162	1.12444E-07	0.517743314	Potri.017G128800.1	AT1G66480.1
Potra2n17c30810.1	0.847257335	0.981965276	0.012276249	0.521534461	Potri.017G128700.1	AT3G18560.1
Potra2n17c30811.1	0.457731962	0.349000071	0.854903321	NA		
Potra2n17c30812.1	-0.217089634	0.225034545	0.580141517	0.943819961		
Potra2n17c30813.1	-4.303618682	-0.431546514	1.42482E-05	0.853030738	Potri.017G128500.1	
Potra2n17c30814.1	-3.304361185	-0.801118886	NA	NA	Potri.017G128400.1	
Potra2n17c30815.1	-0.095049295	0.006462474	0.46952864	0.995579852	Potri.017G128300.2	AT3G03380.1
Potra2n17c30816.1	NA	0.674963689	NA	NA	Potri.017G128200.1	AT5G37820.1
Potra2n17c30817.1	-0.935266132	-0.525954329	2.17636E-09	0.09951899	Potri.017G128100.1	AT3G03440.1
Potra2n17c30818.1	0.403376973	0.192298171	0.017911795	0.812394185	Potri.017G131100.1	AT5G37600.1
Potra2n17c30819.1	NA	NA	NA	NA	Potri.017G126800.1	AT5G37800.1
Potra2n17c30820.1	-0.624857256	-0.14747436	0.003999154	0.88643982	Potri.017G126700.1	AT1G09700.1
Potra2n17c30821.1	0.771125856	0.137591584	1.94071E-05	0.907073354	Potri.017G126600.2	AT3G62470.1
Potra2n17c30822.2	-0.4332991	0.146519601	0.307441134	0.943098757	Potri.017G126500.1	AT5G37790.1
Potra2n17c30823.1	-1.738309345	0.559364179	0.305734843	0.943098757	Potri.017G126400.1	AT3G03430.1
Potra2n17c30824.1	0.230707851	0.091236517	0.286439193	0.937687311	Potri.017G126300.1	AT1G66430.1
Potra2n17c30825.1	-0.987110996	-0.755098712	0.006551336	0.270862551	Potri.017G126200.1	AT5G37770.1
Potra2n17c30826.1	5.701647003	-0.05574223	1.05385E-05	NA	Potri.017G126100.1	AT4G26850.1
Potra2n17c30827.1	0.809543575	0.646187825	0.440033264	0.874808359	Potri.017G126000.1	AT1G66370.1
Potra2n17c30828.1	-0.058272915	0.341164092	0.983018123	NA	Potri.017G125600.1	AT1G66370.1
Potra2n17c30829.1	5.509508956	0.778486915	4.26361E-19	NA	Potri.017G125500.1	AT3G17980.1
Potra2n17c30830.2	-0.063676094	-0.03152411	0.78284649	0.984292161	Potri.017G125400.1	AT5G39790.1

Potra2n17c30831.1	-2.46199765	-0.323881274	5.08561E-13	0.724185549	Potri.017G125300.1	AT1G70780.1
Potra2n17c30832.1	-1.068853634	-0.070079028	1.55758E-10	0.947479563	Potri.017G125200.1	AT2G01570.1
Potra2n17c30833.1	-0.179797908	-0.073511619	0.267417138	0.953831244	Potri.017G125100.1	AT5G16710.1
Potra2n17c30834.2	-0.374696081	0.158809746	0.048057844	0.896248859	Potri.017G125000.1	AT1G27700.1
Potra2n17c30835.1	0.47801444	0.402248333	0.012741721	0.534852254	Potri.017G124800.1	AT2G36230.1
Potra2n17c30836.1	0.322713524	0.237941909	0.51233713	0.912665563	Potri.017G124700.1	AT4G36810.1
Potra2n17c30837.2	-4.931870538	-5.080283503	0.030580561	NA	Potri.017G124600.2	AT4G36810.1
Potra2n17c30838.1	-0.512500659	-0.140021419	0.000394401	0.895366828	Potri.017G124500.1	AT5G16715.1
Potra2n17c30839.1	0.816538284	0.677608406	1.27349E-06	0.509990466	Potri.017G124300.1	AT1G23170.2
Potra2n17c30840.1	4.132194923	1.778384563	1.13136E-22	0.72476666	Potri.017G124200.1	AT1G74670.1
Potra2n17c30841.1	1.456715326	-1.507841271	NA	NA	Potri.017G124100.1	AT3G02645.1
Potra2n17c30842.1	NA	NA	NA	NA	Potri.017G124100.1	AT3G02645.1
Potra2n17c30843.1	NA	NA	NA	NA	Potri.017G124100.1	AT3G02645.1
Potra2n17c30844.1	-0.141067724	0.00527776	0.455141573	0.996748906	Potri.017G123900.1	AT1G66510.1
Potra2n17c30845.1	-0.682249748	-0.077224729	0.003411495	0.956889095	Potri.004G091200.1	AT3G45980.1
Potra2n17c30846.1	0.440196118	0.092556298	0.414683664	0.979775311	Potri.004G091300.1	AT5G40010.1
Potra2n17c30847.1	-2.689793697	-0.877812329	0.431989832	NA	Potri.017G123600.1	AT3G14470.1
Potra2n17c30848.1	-2.210083486	1.177469153	0.302342745	NA	Potri.017G123500.2	AT3G14470.1
Potra2n17c30849.1	-1.317292482	1.444957976	NA	NA	Potri.017G123400.1	AT5G17450.1
Potra2n17c30850.1	-0.591351454	0.0839702	0.032317349	0.967122333	Potri.017G123300.4	AT1G66520.1
Potra2n17c30851.1					Potri.017G123200.1	
Potra2n17c30852.1	0.127383787	0.51453454	0.688335822	0.791589438	Potri.017G122900.4	AT1G15125.1
Potra2n17c30853.1	0.927439601	0.203930301	3.2643E-07	0.839718641	Potri.017G122800.2	AT5G37930.1
Potra2n17c30854.1	-0.033185358	0.059779905	0.770604464	0.937846405	Potri.017G122600.1	AT5G18420.2
Potra2n17c30855.2	-0.019741287	0.00354776	0.934573625	0.99819408	Potri.017G122400.1	AT1G71090.1
Potra2n17c30856.1	-0.164501154	-0.14950535	0.103454963	0.816012202	Potri.017G122300.3	AT2G01275.2
Potra2n17c30857.1	-0.086393306	-0.141965268	0.404013366	0.816856065	Potri.017G122200.1	AT1G36730.1
Potra2n17c30858.1	-1.317292482	-1.465929376	NA	NA	Potri.017G122100.1	
Potra2n17c30859.1	-0.361205457	-0.454885284	0.773377768	0.953831244	Potri.017G122000.1	AT5G37970.1
Potra2n17c30860.1	1.709084505	0.693634098	8.26995E-08	0.422139068	Potri.017G121900.1	AT3G14460.1
Potra2n17c30861.1	1.471955948	0.624147351	8.19647E-05	0.602816211		
Potra2n17c30862.1	1.936031092	0.875154152	1.21287E-06	0.453270097	Potri.018G080632.1	

Potra2n17c30863.1	2.163873597	0.84989966	2.84139E-07	0.692084498		
Potra2n17c30864.1	0.494337337	0.610503506	0.573716891	0.900551258	Potri.017G121200.1	AT3G03280.1
Potra2n17c30865.2	0.037299419	-0.068542899	0.86894259	0.950376522	Potri.017G121100.1	AT1G66740.1
Potra2n17c30866.1	0.486441496	0.014109452	0.004828549	0.991110595	Potri.017G121000.1	AT1G60640.2
Potra2n17c30867.1	0.254469543	0.15216395	0.18693886	0.873823526	Potri.017G120900.1	AT2G24960.1
Potra2n17c30868.1	0.040541968	-0.411666217	0.914832288	0.680030952	Potri.017G120800.1	AT3G03420.1
Potra2n17c30869.1	0.469342063	0.262703769	0.003235773	0.788065047	Potri.017G120600.2	AT1G66760.2
Potra2n17c30870.1	1.545832463	NA	NA	NA		
Potra2n17c30871.1	-1.365924493	0.426679699	0.013349985	0.881090429	Potri.006G103700.1	AT5G07090.1
Potra2n17c30872.1	-0.780735151	-0.178108506	0.30723371	0.971828097		
Potra2n17c30873.3	0.211196019	0.004692636	0.301196563	0.998895625	Potri.017G120100.1	AT2G34260.1
Potra2n17c30874.1	-0.152298314	-0.033380572	0.822135556	0.995565508		
Potra2n17c30875.1	NA	3.010689165	NA	NA	Potri.017G120000.2	AT1G56170.1
Potra2n17c30876.1	-0.906444923	-0.348589358	6.51024E-06	0.794817498	Potri.004G095100.1	AT1G66810.1
Potra2n17c30877.1	-1.547431406	-0.131878338	0.398589408	0.983113084	Potri.017G119800.1	AT2G47485.1
Potra2n17c30878.1	-0.232931988	-0.142546156	0.194151421	0.923995589	Potri.017G119600.1	AT1G72690.1
Potra2n17c30879.1	-0.075645427	-0.347270925	0.882450765	0.895474078	Potri.017G119700.1	
Potra2n17c30880.1	0.151088067	0.004773616	0.42493086	0.997932292	Potri.017G119500.2	AT3G02600.1
Potra2n17c30881.1	0.484039345	0.05469476	0.007448631	0.974756511	Potri.017G119300.1	AT1G16740.1
Potra2n17c30882.1	-0.632524573	-0.233379355	0.181787063	0.922681078	Potri.017G119200.2	AT3G58900.2
Potra2n17c30883.1	0.047348651	-0.012509166	0.775402078	0.993332869	Potri.004G095600.1	AT5G16220.1
Potra2n17c30884.1	0.137706353	0.047428405	0.308420105	0.956610193		
Potra2n17c30885.2	0.453280045	-1.128046748	0.728022949	NA	Potri.017G119000.1	AT2G01210.1
Potra2n17c30886.1	-0.793530522	-0.201644795	0.362751545	0.963225999	Potri.017G118900.1	AT1G66840.1
Potra2n17c30887.1	2.903432979	2.770987278	0.271457618	NA		
Potra2n17c30888.1	-0.800232709	-2.137378195	0.71837676	NA	Potri.017G118300.1	AT3G45850.2
Potra2n17c30889.1	3.297689914	1.590500027	0.055189416	NA	Potri.T064901.1	AT1G67000.1
Potra2n17c30890.1	7.013565138	3.20837612	1.12346E-06	NA	Potri.T064000.1	AT5G38210.1
Potra2n17c30891.1	4.65246382	2.537805496	1.89782E-06	NA	Potri.T064000.1	AT5G38210.1
Potra2n17c30892.1	2.610910166	NA	NA	NA	Potri.017G118300.1	AT3G45850.2
Potra2n17c30893.1	4.043603218	1.614151498	9.66477E-07	0.791002527	Potri.017G118100.1	AT5G38210.1
Potra2n17c30894.1	1.805397332	0.345508228	5.92682E-28	0.514425946	Potri.017G117300.1	AT5G38260.1

Potra2n17c30895.1	1.860693272	0.281805453	2.49543E-35	0.663037599	Potri.017G118100.1	AT5G38210.1
Potra2n17c30896.1	-0.121223319	-0.092125861	0.366115644	0.895474078	Potri.T064300.1	AT5G38220.3
Potra2n17c30897.1	0.82357019	1.895229922	NA	NA	Potri.017G117400.1	AT5G38260.1
Potra2n17c30898.1	0.577963262	-2.446966278	0.7403211	NA	Potri.T064100.2	AT5G38210.1
Potra2n17c30899.1	6.748950659	3.250276327	1.17508E-08	NA		
Potra2n17c30900.1	3.463644182	0.77893979	2.50326E-11	0.881526761	Potri.T064700.1	AT1G66940.1
Potra2n17c30901.1	NA	NA	NA	NA		
Potra2n17c30902.1	-0.865875583	0.090856251	0.008854722	0.96245401	Potri.017G116900.4	AT1G67000.1
Potra2n17c30903.2	-0.459821233	0.180685142	0.011475187	0.849067086	Potri.017G116700.1	AT5G16180.1
Potra2n17c30904.1	-0.453761447	0.279726714	0.249347829	0.865538396		
Potra2n17c30905.1	0.874636313	0.048365454	2.89889E-07	0.978646026	Potri.017G116600.1	AT3G02580.1
Potra2n17c30906.1	-0.642894447	-0.238625758	9.05416E-05	0.722787208	Potri.017G116500.1	AT5G16160.1
Potra2n17c30907.1	0.067691358	0.405953027	0.834088661	0.652831518	Potri.004G097800.1	AT5G16150.1
Potra2n17c30908.1	1.481779358	0.651238802	0.007154694	0.812394185	Potri.017G116300.1	AT1G67030.1
Potra2n17c30909.1	-0.124170607	0.164389074	0.741825081	0.936549944	Potri.017G116200.1	AT2G27770.1
Potra2n17c30910.1	-1.017743667	-0.338103471	1.74002E-06	0.842247476	Potri.017G116100.1	AT5G38300.1
Potra2n17c30911.4	-1.33729885	-0.197613686	2.58123E-06	0.903698951	Potri.017G116000.1	AT1G67040.1
Potra2n17c30912.1	3.073325943	NA	NA	NA	Potri.017G115800.1	AT1G67050.1
Potra2n17c30913.1	-0.158078966	-0.754761882	0.753123186	0.63682724	Potri.017G115700.1	AT1G67050.1
Potra2n17c30914.1	-0.169906234	-0.034092094	0.439478224	0.979896883	Potri.T140500.2	AT2G18410.1
Potra2n17c30915.1	0.24396475	0.063206739	0.463944365	0.976324121	Potri.017G115500.1	AT3G02570.1
Potra2n17c30916.1	0.69251473	0.063933057	0.00018174	0.968407862	Potri.017G115400.1	AT3G02560.1
Potra2n17c30917.1	-0.715169708	1.142466849	0.494276511	0.775391304	Potri.017G115300.1	AT1G25330.1
Potra2n17c30918.1	-0.155644956	0.020643761	0.247054621	0.9869326	Potri.017G115200.1	AT5G38380.1
Potra2n17c30919.1	-0.054943588	-0.12835061	0.878043686	0.939653927	Potri.017G115100.1	AT1G74640.1
Potra2n17c30920.1	1.323112261	0.479465679	4.44205E-20	0.274923694	Potri.017G114800.1	AT5G16120.1
Potra2n17c30921.2	-0.187904833	-0.059878835	0.220971429	0.926546506	Potri.017G114700.1	AT5G16110.1
Potra2n17c30922.1	-0.357267369	-0.357623592	0.182062491	0.786910796	Potri.017G114600.1	AT1G67090.1
Potra2n17c30923.1	1.650939087	-0.264837918	0.000244782	0.911831575	Potri.017G114500.1	AT3G02550.1
Potra2n17c30924.1	0.03272534	0.091930676	0.81705237	0.900884796	Potri.017G114400.1	AT5G15270.2
Potra2n17c30925.1	1.190698052	-1.002356051	0.001840712	0.475435867	Potri.017G114200.1	AT5G38450.1
Potra2n17c30926.1	0.071226792	0.031079946	0.776412162	0.982827151	Potri.017G114100.1	AT5G38460.2

Potra2n17c30927.1	0.189993684	0.309355872	0.646029377	0.857189843		
Potra2n17c30928.1	0.060417494	0.186010406	0.44460963	0.823926359	Potri.017G114000.1	AT3G02540.1
Potra2n17c30929.1	-1.409983009	-0.093067331	NA	NA	Potri.017G113900.1	AT1G13340.1
Potra2n17c30930.1	-1.849671621	0.592657011	0.69334392	NA	Potri.017G113800.1	
Potra2n17c30931.1	2.476290371	NA	NA	NA	Potri.012G020900.1	
Potra2n17c30932.1	2.907537965	NA	NA	NA		
Potra2n17c30933.1	0.308950628	0.086171219	0.039540264	0.926546506	Potri.017G113600.1	AT3G02530.1
Potra2n17c30934.1	-0.261702967	-0.315557773	0.216076855	0.715585895	Potri.T147700.1	
Potra2n17c30935.1	-0.175901827	-0.209680684	0.380371228	0.788065047	Potri.006G148600.10	AT2G19480.3
Potra2n17c30936.1	-0.148882355	-0.194464366	0.379746122	0.718991569	Potri.017G113400.1	AT5G16060.1
Potra2n17c30937.1	-0.081861412	0.020559145	0.312035229	0.983944804	Potri.017G113300.1	AT3G02520.1
Potra2n17c30938.1	-0.834542113	0.014431938	2.41692E-08	0.994283386	Potri.017G113200.1	AT5G16040.1
Potra2n17c30939.3	-0.945613365	-0.244322765	1.33778E-05	0.842962514	Potri.017G112900.1	AT5G38510.2
Potra2n17c30940.1	NA	0.674963689	NA	NA	Potri.017G112800.1	AT4G05160.1
Potra2n17c30941.1	-1.081307306	-0.654664408	0.292768545	0.865591169	Potri.017G112700.3	AT5G38660.1
Potra2n17c30942.2	0.660008591	0.362137135	0.166649002	0.749682671	Potri.017G112600.1	AT5G16030.1
Potra2n17c30943.1	0.92641307	-0.767708831	NA	NA		
Potra2n17c30944.1	-0.212850319	0.039999894	0.097325908	0.962971989	Potri.017G112500.1	
Potra2n17c30945.1	-1.317292482	1.883127743	NA	NA	Potri.004G102500.2	AT3G25780.1
Potra2n17c30946.1	0.209008689	1.876627057	NA	NA	Potri.004G102500.2	AT3G25780.1
Potra2n17c30947.1	-2.70371292	0.245968197	4.61818E-07	0.907073354	Potri.017G112300.1	AT2G37630.1
Potra2n17c30948.2	0.387657949	0.441002605	0.000227659	0.003653135	Potri.017G112200.1	AT1G18700.2
Potra2n17c30949.1	-0.570363449	-0.250023319	0.400666818	0.919424623		
Potra2n17c30950.1	0.230991342	-0.480803296	0.62646703	0.841963133		
Potra2n17c30951.1	NA	NA	NA	NA	Potri.007G058300.1	
Potra2n17c30952.1	-3.012427091	-1.571261375	0.022613767	0.586851629	Potri.017G112000.1	AT1G67260.1
Potra2n17c30953.1	-0.057788829	-0.086327621	0.687900486	0.947969749	Potri.017G111900.1	AT5G38660.2
Potra2n17c30954.1						
Potra2n17c30955.2	-0.141294476	0.013349217	0.252470103	0.988354153	Potri.017G111800.1	AT5G38640.1
Potra2n17c30956.1	-0.351191864	-0.555867061	0.554190913	0.819035854	Potri.017G111700.1	AT5G38630.1
Potra2n17c30957.1	NA	NA	NA	NA	Potri.017G111600.1	AT5G40940.1
Potra2n17c30958.1	NA	NA	NA	NA	Potri.017G111600.1	AT5G40940.1

Potra2n17c30959.1	0.052933107	0.171331542	0.69801053	0.86915032	Potri.004G104000.2	AT1G26110.2
Potra2n17c30960.1	0.346492348	0.253386117	1.03892E-05	0.656318393	Potri.017G111400.1	AT1G67230.1
Potra2n17c30961.1	0.051148308	0.092542564	0.808376552	0.88661378	Potri.017G111300.1	AT5G38600.1
Potra2n17c30962.1	4.009196056	2.173001727	5.54545E-23	0.000314928	Potri.017G111100.1	
Potra2n17c30963.1	-0.06053162	0.216358761	0.810445958	0.828625141	Potri.006G176600.1	AT5G57740.1
Potra2n17c30964.1	4.5773023	2.581943376	1.04008E-34	9.49435E-07	Potri.T100300.1	
Potra2n17c30965.1	6.204642731	3.89812252	2.98138E-75	4.03577E-05	Potri.T100200.1	
Potra2n17c30966.1	8.843124957	6.110550812	3.01375E-13	0.030919863	Potri.T100300.1	
Potra2n17c30967.1	4.758838127	1.728028487	5.70393E-11	0.817799498	Potri.T100300.1	
Potra2n17c30968.1	NA	NA	NA	NA	Potri.T100200.1	
Potra2n17c30969.1	-0.057963792	-0.051094912	0.740609164	0.975578749	Potri.017G108100.1	AT3G02490.1
Potra2n17c30970.1	NA	NA	NA	NA	Potri.017G108200.1	AT5G03340.1
Potra2n17c30971.1	-0.406964219	-0.013161556	NA	0.999244673	Potri.017G108500.1	AT5G38760.1
Potra2n17c30972.1	0.457227286	-0.455704209	0.012749737	0.636664382	Potri.017G108500.1	AT5G38760.1
Potra2n17c30973.1	0.847974589	-0.29743803	0.003341055	0.855587395	Potri.017G108500.1	AT5G38760.1
Potra2n17c30974.1	-0.457433217	0.328015953	0.246301942	0.740524465	Potri.017G108600.1	AT5G38730.1
Potra2n17c30975.1	-0.023832383	-0.008205364	0.896442264	0.995565508	Potri.017G108700.1	AT5G38720.1
Potra2n17c30976.1	1.161648889	0.554669676	0.000178737	0.723580731	Potri.017G108800.1	AT3G02470.3
Potra2n17c30977.2	-0.531263374	0.122782475	0.084929237	0.946464685	Potri.017G109000.1	AT5G15930.1
Potra2n17c30978.1	-0.61191131	0.591957328	0.461603702	0.824448251	Potri.017G109100.1	
Potra2n17c30979.1	1.545832463	2.85418539	NA	NA	Potri.006G146000.1	
Potra2n17c30980.2	-1.185495643	-0.122586574	7.235E-07	0.903477395	Potri.017G109300.1	AT5G38710.1
Potra2n17c30981.1	-0.01631111	-0.068610195	0.929485442	0.941070913	Potri.017G109500.1	AT3G30841.1
Potra2n17c30982.1	6.66323309	2.859438903	1.42011E-39	0.216891326	Potri.017G109600.1	AT5G38530.1
Potra2n17c30983.1	-0.027400224	-0.052427896	0.858771724	0.960981446	Potri.017G109700.1	AT1G67170.1
Potra2n17c30984.1	2.05713329	2.00142716	0.0125883	0.304495297	Potri.017G109800.1	AT3G19660.1
Potra2n17c30985.1	-0.463511466	0.241542509	0.024997284	0.912295985	Potri.017G109900.1	AT3G19660.1
Potra2n17c30986.1	0.693927469	0.026704557	6.92417E-05	0.989304496	Potri.017G110000.1	AT5G59240.1
Potra2n17c30987.1	-1.291594471	-0.480433351	0.000273164	0.663037599	Potri.017G110200.1	AT5G15900.1
Potra2n17c30988.2	-0.339523198	-0.112586422	0.017756612	0.881321405	Potri.017G110300.1	AT5G15880.1
Potra2n17c30989.1	-0.9271601	-0.168462538	0.000588467	0.91233528		
Potra2n17c30990.1	-0.092340215	0.205138316	0.59383307	0.871720501	Potri.017G107600.1	AT1G13570.1

Potra2n17c30991.1	-0.865402417	0.980871386	0.022089671	0.809323756	Potri.017G107500.3	AT3G02380.1
Potra2n17c30992.1	-0.966434443	-0.345062281	0.257037095	0.940856252	Potri.017G107400.1	AT4G31730.1
Potra2n17c30993.1	0.220048529	-0.938415134	0.921112581	NA		
Potra2n17c30994.1	-0.583320559	-0.564483097	0.108623239	0.73777233	Potri.017G107200.1	AT5G57685.1
Potra2n17c30995.1	NA	3.28382	NA	NA	Potri.017G107100.1	AT4G31730.1
Potra2n17c30996.1	NA	NA	NA	NA	Potri.017G107000.1	AT5G38790.1
Potra2n17c30997.1	0.275663359	-0.188555567	0.151808401	0.886633917	Potri.017G106900.1	AT3G02360.1
Potra2n17c30998.1	0.478635811	-0.109858311	0.073364332	0.953831244	Potri.017G106800.1	AT3G02350.1
Potra2n17c30999.1	-0.183646742	0.123583135	0.739280913	0.953831244	Potri.004G111100.1	AT3G30530.1
Potra2n17c31000.1	NA	NA	NA	NA		
Potra2n17c31001.1	1.545832463	1.571529889	NA	NA	Potri.017G106600.1	AT5G51430.1
Potra2n17c31002.1	-0.235149066	-0.122588823	0.292651553	0.885375133	Potri.017G106500.1	AT3G07040.1
Potra2n17c31003.1	0.177563317	0.25951699	0.194984813	0.679485917	Potri.017G106400.6	AT5G11390.1
Potra2n17c31004.1	-0.425402585	-0.10579661	0.013932519	0.916192318	Potri.017G106300.1	AT3G30390.1
Potra2n17c31005.1	-0.005087367	0.180800096	0.971641561	0.71703421	Potri.017G106100.2	AT5G15680.1
Potra2n17c31006.1	0.001975677	0.133369412	0.98838652	0.842247476	Potri.017G106200.2	AT5G49550.1
Potra2n17c31007.1	-0.61910233	0.660619236	NA	NA	Potri.017G105900.1	AT5G38840.1
Potra2n17c31008.1	0.48353868	-0.083036485	0.02139201	0.954559968		
Potra2n17c31009.1	0.331844239	-0.212176293	0.18994866	0.867822135	Potri.T073500.1	AT5G15700.1
Potra2n17c31010.1	0.556791112	0.062916821	0.030765923	0.971752582		
Potra2n17c31011.2	2.429996445	0.823598981	0.000476184	0.825454445	Potri.T129000.2	AT5G17680.1
Potra2n17c31012.2	1.572484297	0.513783265	0.020165829	0.927879595	Potri.017G105000.1	AT5G17680.1
Potra2n17c31013.1	NA	NA	NA	NA	Potri.017G105900.1	AT5G38840.1
Potra2n17c31014.1	0.040033384	-0.363359952	0.965525849	0.94728769		
Potra2n17c31015.1	0.077643933	-0.652668245	0.894124674	0.734313982	Potri.T073500.1	AT5G15700.1
Potra2n17c31016.1	0.162913994	-0.672214814	0.769438506	0.736000077		
Potra2n17c31017.1	3.470884176	1.630226664	8.90743E-05	NA	Potri.014G064100.2	AT5G17680.1
Potra2n17c31018.2	-0.137069226	0.977457746	0.955026322	0.823799522	Potri.014G064300.2	AT5G17680.1
Potra2n17c31019.1	0.535191347	0.45281821	0.066883521	0.723658895	Potri.T129000.2	AT5G17680.1
Potra2n17c31020.1	0.589417672	0.522222797	0.133762446	0.728883321		
Potra2n17c31021.1	2.610910166	1.571529889	NA	NA	Potri.004G232250.1	AT5G49760.1
Potra2n17c31022.1	-0.731734949	0.626178064	NA	NA		

Potra2n17c31023.1	0.745248998	0.807435946	0.152330963	0.694508229	Potri.017G103300.2	AT5G17680.1
Potra2n17c31024.1	0.198454368	-0.173986812	0.579321743	0.940531698	Potri.T073500.1	AT5G15700.1
Potra2n17c31025.1	1.952660907	-2.09793144	0.024993593	NA	Potri.014G064100.2	AT5G17680.1
Potra2n17c31026.1	-0.390124849	-0.122148704	0.138045904	0.931774514		
Potra2n17c31027.2	0.102236182	-0.767708831	NA	NA	Potri.017G102200.2	
Potra2n17c31028.1	0.978423755	0.290734433	7.57523E-05	0.87679119	Potri.004G112700.1	AT5G15720.1
Potra2n17c31029.1	0.6326831	0.052496057	0.000838502	0.978251387	Potri.017G102000.1	AT3G47520.1
Potra2n17c31030.2	1.039729686	0.206306572	8.04316E-13	0.835798849	Potri.017G101800.1	AT4G14090.1
Potra2n17c31031.3	1.166698037	0.25950671	2.2859E-13	0.72476666	Potri.017G101700.4	AT1G69010.1
Potra2n17c31032.1	0.035246968	-0.183974437	0.844396096	0.843683654	Potri.017G101600.1	AT3G30300.1
Potra2n17c31033.1	-0.217747526	-0.128063538	0.068747917	0.850863374	Potri.017G101500.1	AT2G03890.1
Potra2n17c31034.1	0.562776813	0.079695328	0.000536968	0.935751704	Potri.017G101400.1	AT5G15730.2
Potra2n17c31035.1	-0.179848867	1.070929229	0.731212706	0.694477853	Potri.004G113500.1	AT5G15740.1
Potra2n17c31036.1	0.266814024	0.110945839	0.006748725	0.907073354	Potri.017G101200.1	AT5G15750.1
Potra2n17c31037.1	0.158952086	0.26910591	0.275245348	0.487428919	Potri.017G101100.2	AT3G02260.1
Potra2n17c31038.1	0.582327939	0.020117643	0.000455432	0.99005311	Potri.017G101000.1	AT3G62870.1
Potra2n17c31039.1	4.289332033	1.038884283	6.19494E-13	0.738746172	Potri.017G100600.1	AT5G15780.1
Potra2n17c31040.1	-0.077733493	-0.061722556	0.789295787	0.95671968	Potri.017G100400.1	AT3G45070.1
Potra2n17c31041.1	-0.336676947	0.021705341	0.00118636	0.98177802	Potri.017G100300.3	AT5G38880.1
Potra2n17c31042.1	-0.933037748	-0.658752425	0.187663018	0.817799498		
Potra2n17c31043.1	2.322016118	1.209546697	0.025624788	0.80654978	Potri.017G100200.2	AT2G19800.1
Potra2n17c31044.1	-0.592549286	-0.155548239	6.13815E-05	0.935851032	Potri.017G100100.1	AT5G15790.2
Potra2n17c31045.1	0.613281182	0.063819087	1.25733E-06	0.949966783	Potri.017G100000.1	AT5G38900.1
Potra2n17c31046.1	0.644747623	0.272632299	1.49724E-08	0.720581396	Potri.017G099900.1	AT3G02300.1
Potra2n17c31047.1	0.943350674	3.094862151	NA	NA	Potri.017G099800.1	AT5G60910.1
Potra2n17c31048.1	-0.182656038	-0.911801744	0.952890246	NA	Potri.017G099700.1	AT3G02310.1
Potra2n17c31049.1	-0.761728022	-0.294243756	0.0075798	0.712127394	Potri.017G099600.1	AT5G15802.1
Potra2n17c31050.1	0.82357019	NA	NA	NA	Potri.004G118000.1	AT3G30210.1
Potra2n17c31051.1	3.753211788	-0.127619696	0.019312404	NA	Potri.017G099400.1	
Potra2n17c31052.1	0.337190681	0.043788139	0.005684055	0.967129771	Potri.017G099200.3	AT3G02320.1
Potra2n17c31053.2	0.511478541	-0.019009689	0.00520618	0.992934677	Potri.017G099300.1	
Potra2n17c31054.1	0.771654616	-0.080659991	0.004478117	0.979896883	Potri.017G099100.1	AT5G15650.1

Potra2n17c31055.1						Potri.012G134300.2	AT1G16560.3
Potra2n17c31056.1	3.80215699	3.904067763	5.31153E-09	NA		Potri.017G099000.1	AT3G30180.1
Potra2n17c31057.1	NA	1.571529889	NA	NA		Potri.011G144900.1	AT1G13680.1
Potra2n17c31058.1	6.972220204	NA	5.58078E-06	NA		Potri.017G098800.1	AT3G29970.1
Potra2n17c31059.1	-0.218431085	-0.507852011	0.933698062	NA		Potri.017G098600.1	AT5G60920.1
Potra2n17c31060.2	-0.082982702	0.128893349	0.724019793	0.927547456		Potri.017G098500.2	AT5G15640.1
Potra2n17c31061.1	0.834368747	0.805453552	0.000277816	0.00111573		Potri.017G098200.1	AT5G39000.1
Potra2n17c31062.1	0.486439372	1.588372316	NA	NA		Potri.017G098100.1	AT3G29780.1
Potra2n17c31063.1	0.827374588	0.862882677	4.26202E-11	6.12552E-08		Potri.017G096000.1	AT3G51550.1
Potra2n17c31064.1	0.724585539	0.479965036	0.168344615	0.864019566		Potri.017G098200.1	AT5G39000.1
Potra2n17c31065.1	1.422345555	0.004967205	0.696140383	NA			
Potra2n17c31066.1	1.12188182	0.972792205	5.68462E-07	0.001166139		Potri.017G096000.1	AT3G51550.1
Potra2n17c31067.1	1.028182914	0.788917435	3.1074E-10	3.38959E-06		Potri.017G096000.1	AT3G51550.1
Potra2n17c31068.1	1.459399143	0.845234373	3.73388E-07	0.075580473		Potri.017G098000.1	
Potra2n17c31069.1	2.659522793	2.809355139	0.001800719	0.042151965			
Potra2n17c31070.1	-0.043237388	0.420404304	0.888988297	0.65311042		Potri.017G098000.1	
Potra2n17c31071.1	0.73862675	-0.767708831	NA	NA		Potri.017G097400.1	
Potra2n17c31072.1						Potri.011G020000.1	
Potra2n17c31073.1	1.399228774	0.158339205	1.02482E-07	0.961028365		Potri.017G097500.1	AT3G51550.1
Potra2n17c31074.1	-0.133569567	-0.062060581	0.618784555	0.97950444		Potri.017G097500.1	AT3G51550.1
Potra2n17c31075.1	0.458101369	-0.125086064	0.1559802	0.971828097		Potri.017G097800.1	AT3G51550.1
Potra2n17c31076.1	2.468024407	-0.415468649	0.052230709	NA		Potri.017G097500.1	AT3G51550.1
Potra2n17c31077.1							
Potra2n17c31078.1	NA	NA	NA	NA		Potri.017G098000.1	
Potra2n17c31079.1	-0.61910233	-0.046370319	NA	NA		Potri.017G098000.1	
Potra2n17c31080.1							
Potra2n17c31081.1	3.48964704	1.607913416	9.85678E-40	0.166599048		Potri.017G097500.1	AT3G51550.1
Potra2n17c31082.1	NA	0.674963689	NA	NA		Potri.017G098000.1	
Potra2n17c31083.1						Potri.017G098000.1	
Potra2n17c31084.2	0.034570473	0.142887025	0.831219266	0.848131816		Potri.017G097200.1	AT5G39040.1
Potra2n17c31085.1	0.261482006	0.087055869	0.064133358	0.922544948		Potri.017G095300.2	AT5G15610.2
Potra2n17c31086.2	-1.537509685	-0.353332657	6.34029E-07	0.810265332		Potri.017G095200.3	AT1G26355.1

Potra2n17c31087.1	0.513587394	-0.283741768	0.009407481	0.765390763	Potri.004G116500.1	AT3G29770.1
Potra2n17c31088.1						
Potra2n17c31089.1						
Potra2n17c31090.1	0.236684062	-0.461466408	0.70962911	0.816567218	Potri.017G095000.2	AT1G74160.1
Potra2n17c31091.1	0.632926398	-0.229906615	0.427130678	0.956865545		
Potra2n17c31092.1	-0.43237439	-0.196810492	0.0315442	0.742084477	Potri.017G094900.1	AT3G02160.1
Potra2n17c31093.3	NA	3.269258361	NA	NA	Potri.017G094800.1	AT3G02150.2
Potra2n17c31094.1	-3.220366515	-1.141678849	0.043908942	0.825511903		
Potra2n17c31095.1	0.249992894	0.019541981	0.591220006	0.996030289	Potri.017G094700.1	AT5G39080.1
Potra2n17c31096.1	-0.226773977	-0.111679566	0.119672413	0.895575326	Potri.017G094600.1	AT5G60980.2
Potra2n17c31097.1	-0.456220047	-0.106901183	0.108257716	0.949485515	Potri.017G094500.1	AT3G29575.4
Potra2n17c31098.1	-0.254657954	-0.096212639	0.073120391	0.912777843	Potri.017G094400.1	AT3G02130.1
Potra2n17c31099.1	1.316466251	-1.465929376	NA	NA	Potri.017G094300.2	AT3G02125.1
Potra2n17c31100.1	-0.280546146	-0.817718042	0.550503808	0.406753035	Potri.017G094200.1	AT3G02120.1
Potra2n17c31101.1	-0.228761222	0.464851601	0.547073514	0.732363733	Potri.017G094100.3	AT3G02110.1
Potra2n17c31102.1	0.128776287	0.159303556	0.65830646	0.863022165	Potri.T169700.1	AT3G29400.1
Potra2n17c31103.1	0.366108469	0.178845591	0.086503903	0.65293511	Potri.T169700.1	AT3G29400.1
Potra2n17c31104.2	-0.123938021	0.152836877	0.926962003	0.985393296		
Potra2n17c31105.1	1.237418616	0.396894175	5.73366E-12	0.28800807	Potri.017G093901.1	AT3G29400.1
Potra2n17c31106.1	0.33249557	-0.380442197	0.415154299	0.831597503		
Potra2n17c31107.1	2.211931816	0.833964333	1.84855E-15	0.290141457	Potri.017G093901.1	AT3G29400.1
Potra2n17c31108.1	4.460049104	2.375773463	4.19844E-47	0.009063291	Potri.T169800.2	AT3G29400.1
Potra2n17c31109.1	7.588121051	4.874594745	1.05137E-50	0.000178362	Potri.017G093700.1	AT3G29400.1
Potra2n17c31110.1	0.025186155	0.071034725	0.883720657	0.951394085	Potri.017G093600.1	AT3G29390.1
Potra2n17c31111.1	-0.538102981	0.012620072	0.032307272	0.995581218	Potri.017G093500.1	AT5G39220.1
Potra2n17c31112.1	0.328983106	0.834085215	0.851880133	0.89257034	Potri.017G093400.1	
Potra2n17c31113.1	0.512703319	0.015777633	0.003626376	0.993577196	Potri.017G093300.1	AT5G15550.1
Potra2n17c31114.2	0.095816084	0.074905742	0.665271947	0.969237046	Potri.017G093200.1	AT1G69830.1
Potra2n17c31115.1	-1.248534731	-0.648086625	0.139409412	0.893734506	Potri.017G093000.1	AT5G39240.1
Potra2n17c31116.1	-0.082678215	-0.088795202	0.680881629	0.936509845	Potri.017G092800.1	AT5G39250.1
Potra2n17c31117.1	-0.595952689	-1.465929376	NA	NA	Potri.017G092700.1	AT5G39280.1
Potra2n17c31118.1	-0.14757507	0.048343449	0.199823154	0.935474933	Potri.017G092600.1	AT5G15540.1

Potra2n17c31119.1	-0.260622463	-0.191855993	0.391885931	0.84801014	Potri.017G092500.1	AT5G15530.1
Potra2n17c31120.1	0.282991339	-0.067928739	0.113076836	0.956773505	Potri.017G092400.1	AT3G02090.1
Potra2n17c31121.1	1.664677056	1.394613675	NA	NA	Potri.017G092300.1	
Potra2n17c31122.1	0.531728843	0.127977844	0.001024942	0.936890993	Potri.017G092200.1	AT5G61170.1
Potra2n17c31123.1	1.010629212	-0.806120666	0.125855271	0.724604666	Potri.017G092100.1	AT5G15510.1
Potra2n17c31124.1	0.20523242	-0.289149168	0.405572062	0.856053092	Potri.017G092000.3	AT3G29360.2
Potra2n17c31125.1	0.144758814	0.053010865	0.314597834	0.948577865	Potri.017G091900.1	AT1G26580.1
Potra2n17c31126.1	-0.817590012	-0.119351219	0.000102548	0.971537796	Potri.017G091800.1	AT1G26610.1
Potra2n17c31127.1	-0.218227596	0.158555969	0.54627543	0.934627699	Potri.017G091700.1	AT5G61190.1
Potra2n17c31128.1	0.076344408	-0.145523523	0.795340993	0.953608209	Potri.017G091600.1	AT4G37170.1
Potra2n17c31129.1	-0.14920472	0.084239651	0.44886171	0.949847686	Potri.017G091300.2	AT3G49645.1
Potra2n17c31130.1	1.039691427	0.738092207	0.539939787	NA	Potri.017G016800.2	AT5G46070.1
Potra2n17c31131.2	-0.42857469	-0.043638688	0.009145504	0.974264269	Potri.017G091100.1	AT3G29310.1
Potra2n17c31132.1	-0.557942718	0.053625498	0.440987744	0.989300076	Potri.017G091000.1	AT1G76320.2
Potra2n17c31133.1	-1.149004309	0.327170194	0.347941625	0.92978159	Potri.017G091000.1	AT1G76320.2
Potra2n17c31134.1	0.109532235	-0.04545525	0.716382347	0.983008617	Potri.017G090800.1	AT5G15470.1
Potra2n17c31135.1	1.031865281	0.292626308	5.24125E-06	0.817799498	Potri.017G090700.1	AT5G15460.2
Potra2n17c31136.1	-0.547476493	-0.062371187	0.000529428	0.966005919	Potri.017G090600.1	AT5G15450.1
Potra2n17c31137.1	-0.446685187	0.014386852	0.009033208	0.990654009	Potri.017G090500.1	AT3G29290.1
Potra2n17c31138.1	-0.064838654	-0.024248041	0.715988168	0.988835947	Potri.017G090400.2	AT3G29280.1
Potra2n17c31139.1	-0.475528623	0.049713615	0.01704926	0.98177802	Potri.017G090300.1	AT5G39360.1
Potra2n17c31140.1	-0.534622274	-0.143414747	0.007271936	0.921099436	Potri.017G090200.1	AT5G39380.1
Potra2n17c31141.1	-0.726711969	-0.052120621	3.0596E-05	0.9761362	Potri.017G090100.1	AT3G01060.1
Potra2n17c31142.1	-0.561259755	-0.027592399	0.000567411	0.97950444	Potri.017G090000.1	AT3G29270.1
Potra2n17c31143.1	1.604679771	-0.786307282	0.104090998	0.910074792	Potri.017G089900.1	AT5G15410.2
Potra2n17c31144.1	1.266168509	0.369712667	4.40664E-18	0.247573674	Potri.017G089800.1	AT5G15410.1
Potra2n17c31145.1	NA	NA	NA	NA	Potri.017G089700.1	AT5G39400.1
Potra2n17c31146.1	0.194951519	0.058149074	0.029877597	0.941855873	Potri.017G089600.2	AT5G15400.1
Potra2n17c31147.1	0.148268754	-0.159481132	0.687399284	0.925835799	Potri.017G089250.1	AT3G47910.2
Potra2n17c31148.1	1.353477566	-1.622791772	0.53976036	NA	Potri.017G089000.1	AT3G16520.3
Potra2n17c31149.1	0.36381595	0.27440508	0.007342741	0.37518236	Potri.017G088900.1	AT1G69360.1
Potra2n17c31150.1	0.066329027	0.015537949	0.707972292	0.992934677	Potri.017G088700.2	AT3G29200.1

Potra2n17c31151.1	-1.283762536	-0.598158467	1.77878E-05	0.592872379	Potri.017G088600.1	AT5G15350.1
Potra2n17c31152.1	-0.037645121	0.301138564	0.914792762	0.819475509	Potri.017G088500.1	AT2G27035.1
Potra2n17c31153.1	1.459962178	NA	NA	NA		
Potra2n17c31154.1	-0.331619899	-0.073726916	0.05196789	0.943819961	Potri.017G088300.2	AT2G03340.1
Potra2n17c31155.1	-0.349863539	0.116372087	0.074621026	0.945955513	Potri.017G088200.1	AT5G39420.1
Potra2n17c31156.1	-2.912764686	-0.919610063	NA	NA	Potri.017G088100.1	AT1G03700.1
Potra2n17c31157.1	-0.549716024	-0.175557694	0.000306341	0.895129305	Potri.017G088000.2	AT3G29180.1
Potra2n17c31158.1	-0.277300895	0.048340828	0.099266696	0.974275337	Potri.017G087900.1	AT3G01090.2
Potra2n17c31159.1	-2.159158375	-0.731465203	0.000111895	0.673884433	Potri.017G087800.1	AT5G19790.1
Potra2n17c31160.1	-0.214544569	-0.012859403	0.107731632	0.992934677	Potri.017G087700.1	AT5G39510.1
Potra2n17c31161.3	0.113853826	0.246171355	0.584286019	0.743612955	Potri.004G128900.1	AT3G29090.1
Potra2n17c31162.6	0.114506551	0.24582653	0.582511993	0.74706342	Potri.017G087600.1	AT5G15340.1
Potra2n17c31163.1	0.006295925	-0.05260836	0.972757998	0.95671968	Potri.017G087200.1	AT5G39570.1
Potra2n17c31164.1	1.261314403	1.27117775	0.634960964	NA	Potri.004G071700.1	
Potra2n17c31165.1	2.735991243	1.363811008	NA	NA	Potri.T147000.1	
Potra2n17c31166.1	NA	3.390498064	NA	NA	Potri.T147000.1	
Potra2n17c31167.1	0.185204164	-0.162914061	0.775402078	0.966077009	Potri.017G087000.1	AT5G66520.1
Potra2n17c31168.1	-0.875226745	-0.24518661	0.029619245	0.945769042	Potri.017G086900.1	AT1G26690.1
Potra2n17c31169.1	-0.686561698	-0.289042037	0.000150072	0.679660122	Potri.017G086800.2	AT3G29060.1
Potra2n17c31170.1	-0.900948179	-0.295363765	7.90089E-08	0.667038977	Potri.017G086600.1	AT5G15330.1
Potra2n17c31171.1	-0.107821481	-0.072279969	0.424166324	0.930073992	Potri.017G086500.1	AT3G01120.1
Potra2n17c31172.1	1.441522689	-2.05258594	0.629835017	NA	Potri.017G086300.1	AT5G15310.2
Potra2n17c31173.1	-1.784367979	-1.802076492	0.000890023	0.282619136	Potri.017G086200.1	AT5G61430.1
Potra2n17c31174.1	0.017674567	0.102594942	0.966080842	0.966917036	Potri.017G086100.1	AT5G15300.1
Potra2n17c31175.1	-0.250727811	0.004876875	0.476792862	0.998883928	Potri.017G086000.1	AT5G15280.1
Potra2n17c31176.1	1.171943183	2.195911186	0.698893547	NA	Potri.017G085900.1	
Potra2n17c31177.1	0.072736471	0.029636454	0.284819659	0.956865545	Potri.017G085500.1	AT5G15270.1
Potra2n17c31178.3	0.067760966	-0.17467323	0.726123606	0.818007496	Potri.017G085400.2	AT3G01150.1
Potra2n17c31179.1	0.102236182	0.64247635	NA	NA	Potri.017G085300.3	AT3G29030.1
Potra2n17c31180.1	-0.965842672	-1.150085873	0.492228502	0.831738681	Potri.017G085200.1	AT1G69560.1
Potra2n17c31181.1	-0.23219032	0.228317948	0.353700362	0.716548554	Potri.017G085000.1	AT3G28345.1
Potra2n17c31182.1	-0.460081794	0.188403107	0.140325581	0.929435576	Potri.017G084900.1	AT5G39680.1

Potra2n17c31183.1	2.268045794	1.382995232	3.71623E-10	0.170208245	Potri.017G084800.1	AT5G39670.1
Potra2n17c31184.1	0.487262523	-0.03663352	0.00993737	0.983113084	Potri.017G084700.1	
Potra2n17c31185.1	NA	1.571529889	NA	NA		
Potra2n17c31186.1	0.989343978	0.77414854	7.9643E-09	0.560708883	Potri.017G084600.1	AT5G39660.1
Potra2n17c31187.1	-0.426360467	-0.250841964	0.017854651	0.824905557	Potri.004G121900.1	AT3G01170.1
Potra2n17c31188.1	-0.351024971	0.017363054	0.422516347	0.996032145	Potri.004G133700.2	AT5G23490.1
Potra2n17c31189.1	-0.926216932	-0.070961023	0.470218194	0.991732307	Potri.001G422600.1	AT3G56360.1
Potra2n17c31190.1	0.107826676	-0.026878166	0.649757829	0.985393296	Potri.017G084300.2	AT3G28970.1
Potra2n17c31191.1	-1.357844393	-0.10391585	3.79057E-05	0.97950444	Potri.017G084200.1	AT3G01175.1
Potra2n17c31192.1	-0.088110331	0.005629689	0.42797526	0.996281171	Potri.017G084100.1	AT3G01180.1
Potra2n17c31193.1	0.135515374	-1.55593228	NA	NA	Potri.017G084000.1	AT5G15250.1
Potra2n17c31194.1	0.04767145	-0.036713756	0.882320044	0.987288338	Potri.017G083900.1	AT2G36730.1
Potra2n17c31195.1	1.459962178	NA	NA	NA		
Potra2n17c31196.1	5.498592035	2.080096253	0.000103915	0.733263164	Potri.017G083700.1	AT5G15240.1
Potra2n17c31197.3	-2.983195238	-0.841569523	NA	NA	Potri.017G083300.1	AT5G37820.1
Potra2n17c31198.1	0.664886988	-0.10609168	0.003211623	0.935851032	Potri.017G083200.1	AT5G48600.2
Potra2n17c31199.2	-1.006328974	-0.185345996	2.96154E-08	0.814383461	Potri.017G083000.1	AT5G15230.1
Potra2n17c31200.1	-0.962909557	-0.389355786	5.83197E-06	0.619575855	Potri.017G082900.2	AT3G28920.1
Potra2n17c31201.1	2.054381504	2.4990541	0.45404153	NA		
Potra2n17c31202.1	3.83705128	4.243138323	0.172636053	NA		
Potra2n17c31203.1	2.632524198	1.413466566	NA	NA	Potri.009G001800.1	AT2G01050.1
Potra2n17c31204.1	0.001372443	0.359993912	0.998969845	0.954325106	Potri.017G082800.1	
Potra2n17c31205.1	-0.701473186	-0.134206181	6.13751E-05	0.921432293		
Potra2n17c31206.1	-0.575023342	0.036226218	8.93597E-06	0.973347974	Potri.004G126600.1	AT3G28917.1
Potra2n17c31207.1	NA	1.571529889	NA	NA		
Potra2n17c31208.1	-4.109150736	-4.25885838	0.111135276	NA	Potri.017G082500.1	AT3G47600.1
Potra2n17c31209.2	-0.46143879	-0.070685139	0.001766682	0.960229584	Potri.017G082300.3	AT5G39785.1
Potra2n17c31210.2	0.615894433	-0.00016181	0.001576721	0.999937671	Potri.012G108400.1	AT1G26880.1
Potra2n17c31211.1	0.82357019	1.571529889	NA	NA	Potri.017G082000.1	AT5G39820.1
Potra2n17c31212.2	-0.135449601	-0.050261498	0.420524032	0.973347974	Potri.017G081900.1	AT5G39840.1
Potra2n17c31213.1	-1.689051501	-0.541175899	0.021797868	0.917069149	Potri.017G081700.1	AT5G15150.1
Potra2n17c31214.1	1.664677056	NA	NA	NA		

Potra2n17c31215.1	-0.140137136	-0.401997222	0.538187456	0.733006577	Potri.017G081600.1	AT5G39865.1
Potra2n17c31216.3	-0.26770596	0.00024576	0.011639237	0.999937671	Potri.017G081400.6	AT1G14340.1
Potra2n17c31217.1	1.33445541	0.77128246	0.154391659	0.910815461	Potri.017G081300.1	AT3G28857.1
Potra2n17c31218.1						
Potra2n17c31219.1	-1.647522479	-0.407141234	2.78077E-06	0.874507876	Potri.017G081100.1	AT3G28860.1
Potra2n17c31220.1	-0.075427395	-0.761100014	0.889397338	0.588432444	Potri.017G081000.1	AT4G14960.2
Potra2n17c31221.1	NA	2.0078969	NA	NA	Potri.017G080900.2	
Potra2n17c31222.1	0.285319924	-0.058863956	0.151985922	0.978646026	Potri.017G080700.1	AT1G18800.1
Potra2n17c31223.1	-0.306681727	-1.341490867	0.913190102	NA		
Potra2n17c31224.1	-0.054552492	0.043816257	0.851250384	0.985393296	Potri.017G080300.1	AT3G28760.2
Potra2n17c31225.1	8.097696441	3.945692306	5.04508E-10	NA	Potri.017G080100.1	AT3G47800.1
Potra2n17c31226.1	7.391966808	2.549298057	9.47582E-09	NA	Potri.017G080200.1	AT3G47800.1
Potra2n17c31227.1	0.105731464	0.120412474	0.788219165	0.939915707	Potri.017G080000.1	AT5G15140.1
Potra2n17c31228.1	0.353662119	0.022505996	0.042647777	0.987226805	Potri.017G079800.1	AT5G39900.1
Potra2n17c31229.1	-0.697215337	-0.259294851	0.002435893	0.859892315	Potri.017G079600.2	AT1G74690.1
Potra2n17c31230.1	4.700560885	NA	0.006701776	NA	Potri.017G079500.1	AT5G15130.1
Potra2n17c31231.1	NA	NA	NA	NA	Potri.001G155750.1	AT1G21280.1
Potra2n17c31232.1	0.567121278	-0.214218852	0.029863942	0.903477395	Potri.017G079400.1	AT5G39890.1
Potra2n17c31233.1					Potri.017G079300.1	
Potra2n17c31234.1	NA	NA	NA	NA		
Potra2n17c31235.2	-0.204768078	-0.021014467	0.082723194	0.987440949	Potri.017G079200.1	AT3G28715.1
Potra2n17c31236.1	0.584183009	0.882209642	0.270198118	0.543618527		
Potra2n17c31237.1	-0.096161446	0.018701558	0.549215036	0.986636967	Potri.017G079100.1	AT5G25150.1
Potra2n17c31238.1	0.061171965	0.000205023	0.74624056	0.999937671		
Potra2n17c31239.1	2.315713269	NA	NA	NA	Potri.014G138700.1	AT2G48160.1
Potra2n17c31240.1	-0.080609533	0.035505606	0.58938766	0.971537796	Potri.017G078900.1	AT1G13980.1
Potra2n17c31241.1	0.071040524	0.101925615	0.610010531	0.890369631	Potri.017G078800.1	AT1G13980.1
Potra2n17c31242.1					Potri.004G124000.2	AT3G28730.1
Potra2n17c31243.1	NA	0.674963689	NA	NA	Potri.017G078400.1	AT5G15110.1
Potra2n17c31244.1	-0.001545106	-1.507841271	NA	NA	Potri.017G078300.1	AT2G01420.2
Potra2n17c31245.1	0.715262875	0.239811736	0.000103792	0.885375133	Potri.017G078200.1	AT3G01280.1
Potra2n17c31246.1	0.219194969	0.14361501	0.264119965	0.908294078	Potri.017G078100.1	AT5G62740.1

Potra2n17c31247.2	-0.082539478	0.233098416	0.671535921	0.767632362	Potri.017G077300.1	AT5G15080.1
Potra2n17c31248.1	0.840532081	0.000892024	0.171923046	0.999937671	Potri.017G077800.1	AT5G12890.1
Potra2n17c31249.1	-0.103148526	0.069742085	0.505036299	0.931774514	Potri.017G077300.1	AT5G15080.1
Potra2n17c31250.2	2.215162676	1.285690598	0.06349761	NA	Potri.017G077500.1	AT5G12890.1
Potra2n17c31251.1	1.188280275	0.799876799	0.348454182	0.910878765	Potri.017G077800.1	AT5G12890.1
Potra2n17c31252.1	0.286411593	0.145059764	0.007032215	0.751093503	Potri.017G077200.3	AT3G04680.2
Potra2n17c31253.1	3.463997123	2.292347414	2.3735E-23	0.016522993	Potri.017G077100.1	
Potra2n17c31254.1	0.072725256	0.21634139	0.746211804	0.890446513	Potri.017G077000.1	AT3G28700.1
Potra2n17c31255.1	1.277406787	1.47092227	NA	NA	Potri.017G076800.1	AT5G62680.1
Potra2n17c31256.1	3.069241723	2.274383411	2.53039E-19	0.006039093	Potri.017G076800.1	AT5G62680.1
Potra2n17c31257.1	0.789942294	0.137697324	1.84835E-08	0.923880095	Potri.017G076700.1	AT5G39950.1
Potra2n17c31258.1	-0.199930049	-0.179506664	0.157762497	0.814981587	Potri.017G076500.1	AT2G34590.1
Potra2n17c31259.1	-1.002642769	-0.38954701	0.00062532	0.564019611	Potri.017G076400.4	AT5G15070.1
Potra2n17c31260.1	0.071278901	0.058890696	0.677212903	0.958786733	Potri.017G076000.1	AT1G61010.3
Potra2n17c31261.1	4.178103968	2.307412514	0.117197164	NA	Potri.017G075900.1	AT1G27100.1
Potra2n17c31262.1	-2.731937643	-1.121482221	0.053320796	0.821709751	Potri.017G075800.1	AT5G39970.1
Potra2n17c31263.1	-0.658507285	-0.304310893	0.008500253	0.810712263	Potri.017G075700.1	AT5G39980.1
Potra2n17c31264.1	0.028877522	-0.046993636	0.893144385	0.978646026	Potri.017G075600.1	AT5G15050.1
Potra2n17c31265.1	10.51946417	6.742118658	1.85327E-21	0.49278277		
Potra2n17c31266.2	3.754271711	2.394078473	0.003419315	NA		
Potra2n17c31267.1	-0.941067604	-0.476687384	0.003154883	0.724604666	Potri.017G075500.1	AT5G40020.1
Potra2n17c31268.2	-1.48241563	-1.176878058	0.003480044	0.563956825	Potri.017G075400.1	AT5G40030.1
Potra2n17c31269.1	1.26453577	1.348344405	0.469412995	NA	Potri.017G075300.1	AT3G28480.1
Potra2n17c31270.5	0.178460268	-0.023397299	0.132828899	0.979719961	Potri.017G075200.1	AT1G65650.1
Potra2n17c31271.1	0.894546734	0.077845888	8.254E-07	0.941855873	Potri.017G075100.1	AT3G28480.1
Potra2n17c31272.1	-0.765599697	-0.312707067	0.171362648	0.948577865	Potri.017G075000.1	AT3G28470.1
Potra2n17c31273.3	0.230048758	0.049223692	0.046949366	0.953608209	Potri.017G074800.1	AT1G59650.1
Potra2n17c31274.1	NA	1.363811008	NA	NA	Potri.017G074900.1	
Potra2n17c31275.1	-0.61910233	0.64247635	NA	NA		
Potra2n17c31276.1	-0.15524955	0.080292983	0.685553108	0.968706951	Potri.017G074700.1	AT3G28460.1
Potra2n17c31277.1	-0.237255006	-0.02500547	0.489392159	0.991110595	Potri.017G074600.1	AT3G28455.1
Potra2n17c31278.1	-1.026340578	-0.359833609	2.57074E-10	0.753615154	Potri.017G074400.1	AT3G28450.1

Potra2n17c31279.2	-0.039223373	0.216254277	0.838452019	0.813731863	Potri.017G074300.1	AT1G74860.1
Potra2n17c31280.1	-0.906716012	-0.154634887	0.008537131	0.955473249	Potri.017G074200.1	AT3G63240.1
Potra2n17c31281.2	-0.369107249	0.226465104	0.043253196	0.847341104	Potri.004G101500.1	AT3G02530.1
Potra2n17c31282.1	-0.14232201	0.236814665	0.640735209	0.814981587	Potri.011G039800.2	AT1G11360.1
Potra2n17c31283.1	0.258160956	-0.055797671	0.695556199	0.986636967	Potri.004G101500.1	AT3G02530.1
Potra2n17c31284.5	1.331035409	0.026109153	0.107524789	0.997203914	Potri.012G054400.3	AT1G18370.1
Potra2n17c31285.1	5.320079947	NA	0.011625487	NA	Potri.012G054800.1	AT5G38260.1
Potra2n17c31286.1	-0.211176258	1.289749202	0.808376147	0.269832628	Potri.004G101500.1	AT3G02530.1
Potra2n17c31287.2	-0.041142879	-0.687354268	0.973384469	0.910477984	Potri.015G044600.1	AT1G18370.1
Potra2n17c31288.1	NA	NA	NA	NA	Potri.012G054550.1	AT5G38260.1
Potra2n17c31289.1	-1.092660106	-0.403075451	0.135770181	0.922011871	Potri.004G101600.1	
Potra2n17c31290.1	-0.770055085	0.032106751	0.0010638	0.987226805	Potri.017G074000.1	AT3G28345.1
Potra2n17c31291.1	0.569704413	0.216916706	2.05665E-11	0.442002381	Potri.017G073900.1	AT5G14950.1
Potra2n17c31292.1	-0.014111832	0.023216233	0.958205062	0.988065578	Potri.017G073800.1	AT5G40190.1
Potra2n17c31293.1	2.37575612	0.602783056	1.78274E-06	0.921099436	Potri.017G073700.1	AT3G28150.1
Potra2n17c31294.1	-1.328951947	-1.478536539	NA	NA	Potri.017G073300.1	AT3G27970.1
Potra2n17c31295.1	0.106199518	0.039617135	0.208824162	0.953108412	Potri.017G073200.2	AT5G40280.1
Potra2n17c31296.1	-0.212932084	0.151034141	0.223544233	0.910878765	Potri.017G073000.1	AT3G55250.1
Potra2n17c31297.1	NA	NA	NA	NA	Potri.004G029200.1	AT1G10740.1
Potra2n17c31298.1					Potri.010G225900.1	
Potra2n17c31299.1	1.784904996	-2.486725844	0.493984659	NA	Potri.017G072900.1	AT3G01490.1
Potra2n17c31300.1	-0.423267539	0.060565421	0.020425647	0.974194594	Potri.017G072800.5	AT3G01480.1
Potra2n17c31301.1	0.118661663	0.185973008	0.457802189	0.775519829	Potri.017G072600.1	AT3G01460.1
Potra2n17c31302.1	0.33052274	0.32759376	0.003289292	0.349324124	Potri.017G072600.1	AT3G01460.1
Potra2n17c31303.1	-0.102808722	0.132353655	0.529561859	0.903477395	Potri.017G072350.1	AT5G40250.1
Potra2n17c31304.4	0.262356693	0.244287588	0.009891439	0.788065047	Potri.017G072100.1	AT3G27700.2
Potra2n17c31305.1	0.751988009	0.417366716	4.03904E-09	0.296787774	Potri.017G071900.1	AT5G14530.1
Potra2n17c31306.1	0.28481479	-2.034618834	0.900247096	NA	Potri.017G071800.1	AT3G01570.1
Potra2n17c31307.1	0.106605812	0.223594114	0.408700994	0.725595998	Potri.017G071700.1	AT5G14680.1
Potra2n17c31308.3	0.131298529	0.295305368	0.641139353	0.817452127	Potri.017G071600.2	AT5G40400.1
Potra2n17c31309.1	-0.61910233	-0.767708831	NA	NA	Potri.017G071500.1	AT3G27810.1
Potra2n17c31310.1	-0.099148225	-0.059814693	0.294826582	0.953831244	Potri.017G071400.1	AT5G14610.2

Potra2n17c31311.1	-0.19044212	-0.116324764	0.767561706	0.977559521	Potri.001G344600.1	AT3G27580.2
Potra2n17c31312.1	-0.668284765	-0.196328198	6.11574E-06	0.712599153	Potri.017G071300.3	AT5G40500.1
Potra2n17c31313.1	-1.755252514	-0.239347746	0.002057921	0.909450222	Potri.017G071200.1	AT3G27440.1
Potra2n17c31314.1	0.034186864	0.007788248	0.841736751	0.996032145	Potri.017G071100.1	AT5G40580.2
Potra2n17c31315.1	-0.046213294	-0.408325596	0.938859078	0.859892315	Potri.017G070900.2	AT5G40610.1
Potra2n17c31316.1						
Potra2n17c31317.1	-0.536838267	-0.07631526	0.000422869	0.934755688	Potri.017G070800.1	AT3G46610.1
Potra2n17c31318.1	-0.729416196	-0.708349633	0.058463215	0.541196221	Potri.014G190600.1	AT3G07140.1
Potra2n17c31319.1	-0.637696467	-0.352218476	0.055893763	0.779077802	Potri.014G190600.1	AT3G07140.1
Potra2n17c31320.1	-0.0777576	-0.708205791	0.940052904	0.872233138		
Potra2n17c31321.1	NA	NA	NA	NA	Potri.009G155200.3	AT2G27170.1
Potra2n17c31322.1	0.218552133	-0.595508622	0.943691146	NA	Potri.001G172400.2	AT4G25970.1
Potra2n17c31323.1	-0.803430056	-0.145421111	0.79129592	NA		
Potra2n17c31324.1	0.041092219	-0.180863099	0.823328453	0.739555983	Potri.017G066000.1	AT3G01790.1
Potra2n17c31325.2	-0.093322486	0.095889998	0.546685915	0.903698951	Potri.017G065900.1	AT5G59710.1
Potra2n17c31326.1	0.01329229	-0.168319542	0.943195536	0.885628484	Potri.017G065800.2	AT5G40770.1
Potra2n17c31327.1	0.407613599	0.095562824	0.073540272	0.936549944	Potri.017G065500.1	AT5G50180.1
Potra2n17c31328.1	0.854673514	0.500290738	0.088282266	0.747657496		
Potra2n17c31329.1	0.031461667	0.122570607	0.873789146	0.935851032	Potri.017G065200.1	AT3G27470.2
Potra2n17c31330.1	-0.531605331	-0.170791699	0.00015511	0.907073354	Potri.017G069800.1	AT5G40470.1
Potra2n17c31331.1	1.664677056	2.676364087	NA	NA		
Potra2n17c31332.1	-0.734036898	1.788840384	0.319601159	0.028274622	Potri.002G235500.3	AT3G23670.1
Potra2n17c31333.1	-0.584982552	1.412510599	0.376531436	0.137620511	Potri.002G235500.3	AT3G23670.1
Potra2n17c31334.1	NA	2.509020267	NA	NA		
Potra2n17c31335.1	NA	NA	NA	NA	Potri.014G128200.1	AT4G02390.1
Potra2n17c31336.1	3.717804419	2.146414233	0.346881454	NA	Potri.T033800.1	AT5G14250.1
Potra2n17c31337.1	1.414035707	0.921280649	NA	NA	Potri.017G069900.1	
Potra2n17c31338.1	1.129598222	-1.405406747	0.108937684	0.550771689	Potri.017G070000.1	AT5G40460.1
Potra2n17c31339.1	2.340207562	NA	NA	NA		
Potra2n17c31340.1	-0.034877504	-0.079471215	0.805219284	0.896937427	Potri.017G070100.1	AT3G26020.1
Potra2n17c31341.1	0.169195992	-0.090439834	0.299401738	0.953831244	Potri.017G070200.1	AT5G40760.1
Potra2n17c31342.1	0.305732049	0.101476059	0.007661249	0.855199637	Potri.017G070300.1	AT5G40670.1

Potra2n17c31343.1						
Potra2n17c31344.1	0.415462089	0.231753791	3.68997E-05	0.660636854	Potri.017G070500.1	AT3G28030.1
Potra2n17c31345.1	0.023455515	-0.157783961	0.987410405	NA		
Potra2n17c31346.1	-0.225746609	-0.157783961	0.875830661	NA	Potri.T055866.1	
Potra2n17c31347.1	0.944005157	1.651442319	0.397092136	0.663037599	Potri.005G155100.1	AT4G05095.1
Potra2n17c31348.1	-1.703826942	-2.850094422	0.31750636	NA	Potri.017G067900.1	AT5G18610.2
Potra2n17c31349.1	2.673714858	-1.4579832	0.12750356	NA	Potri.T055866.1	
Potra2n17c31350.1	-0.332259522	-0.082360107	0.031935353	0.937276488	Potri.017G068600.1	AT4G17310.1
Potra2n17c31351.1	NA	NA	NA	NA	Potri.008G111700.1	AT1G26160.1
Potra2n17c31352.1	0.410254165	-0.239926668	0.190571599	0.898711301	Potri.017G067600.1	AT4G14605.1
Potra2n17c31353.1	-0.206289719	0.061237943	0.122723175	0.962661188	Potri.017G067400.1	AT5G40840.1
Potra2n17c31354.1	-0.351887534	-0.13820933	0.019497711	0.855432225	Potri.017G066900.2	AT1G64750.1
Potra2n17c31355.1	-0.757514746	-0.396404615	0.372780398	0.941855873	Potri.017G067000.1	AT3G48010.1
Potra2n17c31356.1					Potri.004G232250.1	AT5G49760.1
Potra2n17c31357.3	-0.077716503	0.019877784	0.592064567	0.982827151	Potri.017G066500.1	AT5G14260.2
Potra2n17c31358.1	-0.528072396	0.045440172	0.00629812	0.974275337	Potri.017G069100.1	AT3G27090.1
Potra2n17c31359.1	-0.287658965	0.076191163	0.077204659	0.956865545	Potri.T061800.1	AT3G27100.1
Potra2n17c31360.1	-0.053284693	0.045922626	0.726891481	0.950878725	Potri.017G069300.2	AT5G14170.1
Potra2n17c31361.1	-0.200456784	-0.022809896	0.128436316	0.978646026	Potri.017G069600.2	AT5G40870.1
Potra2n17c31362.1	NA	2.283425176	NA	NA	Potri.017G069700.1	AT5G14180.1
Potra2n17c31363.1	-0.008899312	0.160321933	0.957188488	0.828625141	Potri.T044200.2	AT1G24300.1
Potra2n17c31364.1	-1.142670683	0.096703158	0.000174313	0.971780393		
Potra2n17c31365.1	0.281158131	0.315974237	0.078448723	0.749144604	Potri.T044000.1	AT5G14100.1
Potra2n17c31366.1	0.195233826	0.354998278	0.568441444	0.70570456	Potri.001G327500.2	AT5G14090.1
Potra2n17c31367.3	0.263673856	0.032539717	0.344714065	0.987548286	Potri.T043900.1	AT3G01980.4
Potra2n17c31368.1	5.123022718	0.722879213	3.81227E-05	NA	Potri.T043800.1	AT5G13080.1
Potra2n17c31369.1	0.284672509	0.152639735	0.155988003	0.930871994	Potri.T043700.1	AT5G14105.1
Potra2n17c31370.1	NA	NA	NA	NA	Potri.T043600.1	
Potra2n17c31371.1	-2.630148806	0.569252569	0.321739637	NA		
Potra2n17c31372.1	-0.923229267	-0.454089962	0.150266754	0.896937427	Potri.017G064500.1	AT3G27030.1
Potra2n17c31373.1	-0.893646988	-0.555500752	0.679013098	NA	Potri.017G064600.1	AT3G27027.1
Potra2n17c31374.1	-0.820896749	-0.309597923	0.44773278	0.957872702		

Potra2n17c31375.1	-1.292371322	-0.448838388	0.066097498	0.88643982	Potri.017G064400.1	AT5G40960.1
Potra2n17c31376.1	4.711908821	2.32414027	0.054363798	NA	Potri.017G064300.1	AT5G08391.1
Potra2n17c31377.3	-0.089091249	0.17885356	0.703021897	0.834823243	Potri.001G329100.1	AT3G01930.2
Potra2n17c31378.1	0.855804853	0.099926337	0.005091695	0.983113084	Potri.017G064100.1	AT5G67400.1
Potra2n17c31379.1					Potri.T042800.1	AT4G24920.1
Potra2n17c31380.1	1.459962178	NA	NA	NA	Potri.012G109200.1	AT5G61840.1
Potra2n17c31381.1	0.030943345	-0.712472021	0.958743014	0.742962534		
Potra2n17c31382.1	-0.108110064	-0.694609931	0.832566887	0.679365931	Potri.T042700.1	AT1G01630.1
Potra2n17c31383.1	-0.15007638	-0.725493498	0.901329014	0.870614204		
Potra2n17c31384.1	0.022245296	-0.310361757	0.980326987	0.936890993		
Potra2n17c31385.1	-1.09555567	0.440971047	0.561096259	NA		
Potra2n17c31386.1	1.620153656	-0.195411254	2.81979E-05	0.972159498	Potri.T042700.1	AT1G01630.1
Potra2n17c31387.1					Potri.012G109300.1	AT5G50990.1
Potra2n17c31388.3	2.663568141	2.653300067	0.014611055	0.585361878		
Potra2n17c31389.1	0.912472875	-0.262257565	5.27019E-05	0.896937427		
Potra2n17c31390.5	-0.410011792	0.070835536	0.004725966	0.950706289	Potri.017G063800.1	AT3G26935.1
Potra2n17c31391.1	-0.140651261	-0.450989356	0.786806814	0.754292559	Potri.017G063700.1	AT5G41070.1
Potra2n17c31392.1	NA	1.363811008	NA	NA		
Potra2n17c31393.1	1.995523668	0.388871339	NA	0.903698951	Potri.017G063500.1	AT3G02040.1
Potra2n17c31394.1	3.71928908	1.028290015	1.23533E-22	0.466558048	Potri.017G063300.1	AT5G13180.1
Potra2n17c31395.1	0.2885834	0.691766608	0.925672737	NA	Potri.017G063200.1	
Potra2n17c31396.1	0.150502696	-0.032304459	0.23812668	0.966917036	Potri.017G063000.1	AT2G33490.1
Potra2n17c31397.1	-0.61910233	-0.767708831	NA	NA	Potri.017G062900.1	AT3G02645.1
Potra2n17c31398.1					Potri.017G062900.1	AT3G02645.1
Potra2n17c31399.1	NA	NA	NA	NA		
Potra2n17c31400.1	0.824497211	-0.767708831	NA	NA	Potri.017G062900.1	AT3G02645.1
Potra2n17c31401.4	-0.292068568	-0.143190459	0.10805822	0.896314971	Potri.017G062800.2	AT3G26900.1
Potra2n17c31402.1	0.82357019	NA	NA	NA	Potri.017G062500.1	AT5G14010.1
Potra2n17c31403.1	3.828949531	0.833620777	3.73589E-16	0.771552617	Potri.017G062400.1	AT4G18930.1
Potra2n17c31404.1	2.692001631	0.780907693	5.35865E-30	0.712127394	Potri.017G062100.1	AT4G25040.1
Potra2n17c31405.1	2.473373196	0.656474532	6.68133E-33	0.663037599	Potri.017G062000.1	AT5G14020.1
Potra2n17c31406.1	-0.974599008	-1.134518006	0.689279721	NA		

Potra2n17c31407.2	0.421674822	-0.023514397	0.000655308	0.983113084	Potri.017G061900.1	AT5G14030.3
Potra2n17c31408.2	-0.402682805	-0.315041097	0.006112908	0.50677056	Potri.017G061700.1	
Potra2n17c31409.1	-0.176006506	0.079911221	0.181207371	0.923413596	Potri.017G061600.1	AT3G26810.1
Potra2n17c31410.1						
Potra2n17c31411.1	-0.221836261	0.026441044	0.243593274	0.987288338	Potri.017G061400.1	AT1G12830.1
Potra2n17c31412.1	-2.218068851	0.156288299	0.502412469	NA	Potri.001G322700.1	AT3G26790.1
Potra2n17c31413.4	-0.083829126	-0.097430259	0.527452459	0.935474933	Potri.017G061100.1	AT1G12840.1
Potra2n17c31414.1	1.120277974	0.06197156	NA	NA		
Potra2n17c31415.1	-0.144464063	-0.223906035	0.275358914	0.699520904	Potri.017G060800.1	AT5G14040.1
Potra2n17c31416.1	-0.229231828	-0.343299607	0.560164887	0.834560473	Potri.001G322100.1	AT3G26782.1
Potra2n17c31417.1	-0.133605536	-0.162755768	0.808192391	0.966077009	Potri.001G322200.1	AT3G22790.1
Potra2n17c31418.1	0.237767837	-0.25282641	0.7504119	0.946464685	Potri.017G060300.1	AT3G23770.1
Potra2n17c31419.1	1.071148444	1.171597558	0.707923239	NA		
Potra2n17c31420.1	-0.021377818	0.031064771	0.895848138	0.98036348	Potri.017G060200.1	AT3G18790.1
Potra2n17c31421.1	0.663339123	0.258721836	1.05239E-09	0.590365286	Potri.017G060100.1	AT3G23790.1
Potra2n17c31422.1	0.384919625	0.535359746	0.287865966	0.704457584	Potri.017G060000.1	AT4G14030.1
Potra2n17c31423.1	2.714677912	0.235907114	2.94819E-09	0.960981446	Potri.017G059900.1	AT4G14010.1
Potra2n17c31424.1	0.235167201	-0.007849881	0.212155377	0.996281171	Potri.017G059800.1	AT4G14010.1
Potra2n17c31425.1	0.208280962	-0.616985887	0.927762795	NA	Potri.017G045950.1	
Potra2n17c31426.1	NA	NA	NA	NA	Potri.017G045950.1	
Potra2n17c31427.1	NA	NA	NA	NA	Potri.017G045950.1	
Potra2n17c31428.1	NA	NA	NA	NA	Potri.017G045950.1	
Potra2n17c31429.1						
Potra2n17c31430.1	NA	NA	NA	NA	Potri.017G045950.1	
Potra2n17c31431.1	0.172016003	0.096062462	0.322237815	0.917751882	Potri.017G059600.1	AT4G13980.1
Potra2n17c31432.1	-0.721895726	-0.486467148	0.026756177	0.71411664		
Potra2n17c31433.1	-0.716037737	-0.488443104	0.027435204	0.706822371	Potri.017G059500.1	AT4G13950.1
Potra2n17c31434.1	0.128170053	-0.34257095	0.655830438	0.817452127	Potri.017G059400.1	AT4G13940.1
Potra2n17c31435.1	-0.403545875	-0.370631773	0.187801862	0.857189843	Potri.017G059300.1	AT4G13930.1
Potra2n17c31436.1	0.567963787	0.229856409	0.004106941	0.759797728	Potri.017G059200.1	AT4G13870.2
Potra2n17c31437.1	0.697750747	-0.397831329	0.576525499	0.953608209		
Potra2n17c31438.1	-0.407317679	-0.5479461	0.242574354	0.738746172	Potri.017G059100.1	AT3G23820.1

Potra2n17c31439.1	0.1892092	-0.168420656	0.28736795	0.903477395	Potri.017G059000.1	AT4G13850.1
Potra2n17c31440.1	-4.726603003	-1.682749081	0.007933602	NA	Potri.017G058900.1	AT3G06240.1
Potra2n17c31441.1	NA	NA	NA	NA		
Potra2n17c31442.1	-2.881267416	-2.205463601	0.00497956	0.501154172	Potri.017G058900.1	AT3G06240.1
Potra2n17c31443.1	0.459367874	0.285064306	0.000111142	0.778013962	Potri.017G058600.2	AT5G43490.1
Potra2n17c31444.3	0.183702761	0.200246034	0.101427395	0.736149483	Potri.017G058500.1	AT5G43490.1
Potra2n17c31445.1	-0.592046465	-0.050536939	0.000385778	0.986190118	Potri.017G058400.1	AT4G13830.2
Potra2n17c31446.1	-0.202678765	0.166540171	0.210826844	0.869327705	Potri.017G058300.1	AT3G23900.3
Potra2n17c31447.1	-0.475477006	-0.175712328	0.001160101	0.885375133	Potri.017G058200.1	
Potra2n17c31448.1	-0.221288189	0.032103615	0.185877573	0.978203092	Potri.017G058200.1	
Potra2n17c31449.1	NA	2.253733581	NA	NA	Potri.017G058100.1	AT5G39820.1
Potra2n17c31450.1	0.12504478	0.21601509	0.952148786	NA	Potri.017G058000.1	AT3G06240.1
Potra2n17c31451.1	0.82357019	NA	NA	NA		
Potra2n17c31452.1	NA	0.674963689	NA	NA	Potri.017G058100.1	AT5G39820.1
Potra2n17c31453.1	-0.610863742	-0.205065919	0.052505789	0.924256651	Potri.017G058000.1	AT3G06240.1
Potra2n17c31454.1	-0.461205277	-0.033912293	0.011152018	0.9869326	Potri.017G057900.1	AT2G32810.1
Potra2n17c31455.1	-1.155245335	-2.068262644	0.174212033	0.361949049	Potri.017G057800.1	AT1G04920.1
Potra2n17c31456.1	NA	NA	NA	NA		
Potra2n17c31457.1	0.169663053	-0.092088429	0.169417841	0.924951347	Potri.017G057700.1	AT4G13780.1
Potra2n17c31458.1	-1.625617017	0.04976186	0.174457221	0.994692557	Potri.015G124466.1	AT2G35110.1
Potra2n17c31459.1	0.82357019	1.363811008	NA	NA		
Potra2n17c31460.1	-0.381677045	0.042562617	0.016925435	0.963993404	Potri.017G057500.1	AT4G22330.1
Potra2n17c31461.1	-0.174388602	-0.037899914	0.297563647	0.97064682	Potri.017G057400.2	AT4G14147.1
Potra2n17c31462.1	0.087065117	0.094179287	0.470833942	0.907073354	Potri.017G057300.2	AT1G04910.1
Potra2n17c31463.1	-0.11486668	-0.018527315	0.380901073	0.983113084	Potri.017G057100.1	AT4G13730.1
Potra2n17c31464.2	0.20663891	0.154955159	0.037129635	0.792511865	Potri.017G056900.2	AT1G04850.1
Potra2n17c31465.1	0.200885016	-0.083801075	0.15241262	0.91392603	Potri.017G056800.1	AT2G32760.1
Potra2n17c31466.1	0.252713304	0.373433384	0.063932315	0.389989789	Potri.017G056700.1	AT4G15570.1
Potra2n17c31467.1	2.632524198	2.676364087	NA	NA	Potri.017G056500.1	AT5G15020.1
Potra2n17c31468.3	-0.000269553	-0.101733418	0.999027927	0.917751882	Potri.017G056400.1	AT3G07550.1
Potra2n17c31469.5	1.487752203	-0.40597683	0.278911967	NA	Potri.001G316200.1	AT3G01320.1
Potra2n17c31470.1	2.063471427	5.265416228	NA	NA	Potri.017G056100.1	AT3G01320.2

Potra2n17c31471.1	0.82357019	4.630492604	NA	NA	Potri.017G056100.1	AT3G01320.2
Potra2n17c31472.1	NA	1.394613675	NA	NA	Potri.004G090000.1	AT1G24230.1
Potra2n17c31473.1	0.063103035	0.038615195	0.782216559	0.980475138	Potri.017G056000.1	AT3G25040.1
Potra2n17c31474.1						
Potra2n17c31475.1						
Potra2n17c31476.1	-0.972330897	0.140528203	0.003591703	0.956865545	Potri.017G055900.2	AT3G07130.1
Potra2n17c31477.1	1.705924504	0.365148662	8.76709E-12	0.779077802	Potri.017G055800.2	AT3G07130.1
Potra2n17c31478.1	-0.050251382	-0.911729566	0.94333778	0.860325259	Potri.017G055700.1	AT4G13600.1
Potra2n17c31479.1	0.050916337	-0.243396685	0.966690441	0.978203092		
Potra2n17c31480.2	0.008745287	-0.033275435	0.963012027	0.973347974	Potri.017G055600.1	AT1G04860.1
Potra2n17c31481.1	-0.397259785	0.178059531	0.596640011	0.966005919		
Potra2n17c31482.1	-0.009876772	-0.075854481	0.967732832	0.95840886		
Potra2n17c31483.1	NA	NA	NA	NA		
Potra2n17c31484.1	-0.3412058	-0.358299955	0.835981128	NA	Potri.017G055300.2	AT5G56150.1
Potra2n17c31485.1	0.01168382	0.409142122	0.986600095	0.898853929	Potri.017G055200.1	AT1G04880.1
Potra2n17c31486.1	2.282771052	NA	NA	NA		
Potra2n17c31487.1	2.437003803	0.885565801	2.35345E-15	0.354287698	Potri.017G055100.1	AT4G13630.1
Potra2n17c31488.1	-1.069909103	-0.334149125	3.5329E-05	0.667038977	Potri.017G055000.1	AT2G32800.1
Potra2n17c31489.1	-1.014670991	-0.323593671	0.000113159	0.716583657		
Potra2n17c31490.1	-0.089332871	-0.074472563	0.819064245	0.966005919	Potri.017G054800.1	AT4G13640.1
Potra2n17c31491.1	-2.074695157	-1.526434938	0.088162052	0.72838435		
Potra2n17c31492.1	0.65734257	-0.514247207	0.837653714	NA		
Potra2n17c31493.1	0.776470644	0.284273668	2.38075E-07	0.7696553	Potri.017G054700.2	AT3G24130.1
Potra2n17c31494.1	-0.851740293	-0.828335558	NA	NA		
Potra2n17c31495.1	0.82357019	NA	NA	NA	Potri.017G054500.1	AT3G24140.1
Potra2n17c31496.1	0.329563815	0.038699826	0.026298028	0.978203092		
Potra2n17c31497.1	0.294118386	0.050721738	0.021347841	0.966005919	Potri.017G054400.1	AT2G32730.1
Potra2n17c31498.1	0.705959184	0.05481317	1.77746E-07	0.941260209	Potri.017G054300.1	AT2G32720.1
Potra2n17c31499.1	-0.412183758	-0.169842718	0.012927702	0.84487051	Potri.017G054000.1	AT5G48820.1
Potra2n17c31500.1	-0.473597176	-0.391015781	0.000788329	0.354287698	Potri.017G053900.1	AT1G15800.1
Potra2n17c31501.1	0.429324766	-1.037254856	0.470668259	0.639292153	Potri.017G053700.1	AT1G80580.1
Potra2n17c31502.1	0.82357019	NA	NA	NA		

Potra2n17c31503.1	-0.00786958	-0.205818201	0.984438423	0.936890993	Potri.017G053600.1	AT1G69800.2
Potra2n17c31504.1	0.198502937	-0.140731491	0.051480945	0.855432225	Potri.017G053500.1	AT1G80600.1
Potra2n17c31505.1	-0.61910233	0.673278781	NA	NA	Potri.001G313100.1	AT5G09430.1
Potra2n17c31506.1	0.130785699	0.24969825	0.566306854	0.910477984	Potri.017G053100.1	AT1G02170.1
Potra2n17c31507.1	0.130667625	0.250998728	0.566626454	0.910477984	Potri.017G053200.1	AT5G64230.1
Potra2n17c31508.1	3.546352537	0.851266564	0.121114961	NA	Potri.017G053000.1	AT1G02170.1
Potra2n17c31509.1	1.274482656	0.110081123	NA	NA	Potri.017G052900.1	AT1G02170.1
Potra2n17c31510.1	NA	NA	NA	NA	Potri.017G052600.2	AT1G02170.1
Potra2n17c31511.1	NA	1.394613675	NA	NA	Potri.017G052600.2	AT1G02170.1
Potra2n17c31512.1	-1.14279598	0.329215777	NA	NA	Potri.017G052300.1	AT1G22360.1
Potra2n17c31513.1	0.476508333	-2.452100232	0.83084283	NA	Potri.017G052400.1	AT1G22360.1
Potra2n17c31514.1	1.114991597	1.356589801	0.761769272	NA	Potri.017G052300.1	AT1G22360.1
Potra2n17c31515.1	-0.143185816	-0.753530395	0.609215501	0.586851629	Potri.017G052000.1	AT1G22360.1
Potra2n17c31516.1	-1.11241194	-0.715023831	0.003365119	0.466145447	Potri.017G052400.1	AT1G22360.1
Potra2n17c31517.1	-0.552461285	-0.709103612	0.007024146	0.323147091	Potri.017G052400.1	AT1G22360.1
Potra2n17c31518.1	-0.914484221	0.196412486	0.846374508	NA	Potri.017G052300.1	AT1G22360.1
Potra2n17c31519.2	2.264824959	0.910023973	3.76596E-18	0.608568291	Potri.017G051800.1	AT5G64250.2
Potra2n17c31520.1	3.884734164	1.102128307	9.94101E-30	0.428801064	Potri.017G051700.1	AT5G64260.1
Potra2n17c31521.1	0.242921196	0.197859663	0.070817594	0.784407529	Potri.017G051600.1	AT5G65260.1
Potra2n17c31522.1	0.24736036	0.030666274	0.130286699	0.98177802	Potri.017G051500.1	AT3G21110.2
Potra2n17c31523.1	-2.006090126	-0.338691522	7.84749E-06	0.907073354	Potri.017G051300.1	AT1G17840.1
Potra2n17c31524.1	-1.330410476	0.633881332	0.701271777	0.971432539	Potri.017G051100.1	AT1G70840.1
Potra2n17c31525.3	2.048259273	-1.55593228	0.57069226	NA	Potri.017G051000.1	AT1G17840.1
Potra2n17c31526.2	-0.264523582	0.044807897	0.249659053	0.98177802	Potri.017G050900.1	AT1G60600.2
Potra2n17c31527.1	-0.170127117	-0.316525336	0.71220537	0.728883321	Potri.T105666.1	
Potra2n17c31528.1	-0.636020509	-0.116937301	8.6331E-06	0.886281281	Potri.017G136900.1	AT3G14460.1
Potra2n17c31529.1	NA	NA	NA	NA	Potri.017G137900.2	AT3G14460.1
Potra2n17c31530.1	-0.194108804	-0.006183454	0.356458679	0.996482488	Potri.017G050500.1	AT5G64290.1
Potra2n17c31531.1	-0.718866011	0.371420598	0.205401514	0.912295985		
Potra2n17c31532.1	3.809836344	-0.769132649	0.007274041	NA	Potri.017G050400.1	AT5G64300.1
Potra2n17c31533.1	-0.062086632	-0.620363876	0.797785175	0.627991232	Potri.017G050300.1	AT5G64310.1
Potra2n17c31534.1	6.677254582	3.458971917	1.1044E-43	0.708191066	Potri.017G050200.1	AT5G64310.1

Potra2n17c31535.1	-0.68114691	-0.188761567	1.95511E-08	0.672633803	Potri.017G050100.3	AT4G01020.1
Potra2n17c31536.1	-1.975173516	-0.339493164	3.3043E-07	0.804796567	Potri.017G050000.2	AT2G14095.1
Potra2n17c31537.1	1.25571712	0.33470726	2.37809E-05	0.733263164	Potri.017G049800.1	AT1G72220.1
Potra2n17c31538.1	-0.748349365	-0.305594526	3.57856E-06	0.560708883	Potri.017G049200.1	AT3G17380.1
Potra2n17c31539.2	0.738091213	0.755946181	0.016788745	0.633711902	Potri.017G048700.2	AT2G36690.1
Potra2n17c31540.1	0.181914737	0.051353938	0.499050671	0.98036348	Potri.017G048500.2	AT5G09450.1
Potra2n17c31541.2	-0.034158268	0.064035707	0.821393803	0.948002367	Potri.017G048400.1	AT1G12680.1
Potra2n17c31542.1	-4.175702674	-1.59281522	0.000163147	0.389056998	Potri.017G048300.1	AT4G33600.1
Potra2n17c31543.1	1.664677056	NA	NA	NA		
Potra2n17c31544.1	-0.078102854	0.178095098	0.80515872	0.874595634	Potri.017G048200.1	AT5G64330.1
Potra2n17c31545.1	-1.371268264	0.425350268	NA	NA	Potri.017G048100.1	AT3G23570.1
Potra2n17c31546.1	NA	NA	NA	NA		
Potra2n17c31547.1	-0.352245864	-0.167317629	0.045922199	0.900551258	Potri.017G048000.1	AT5G09460.1
Potra2n17c31548.1	-0.276113049	-0.184067454	0.183379744	0.846680547	Potri.017G047800.1	AT2G22500.1
Potra2n17c31549.1						
Potra2n17c31550.1	0.82357019	4.937615818	NA	NA	Potri.017G047700.1	AT1G58120.1
Potra2n17c31551.2	-0.242394854	0.024629708	0.127812631	0.981772014	Potri.017G047600.1	AT1G32750.1
Potra2n17c31552.1	NA	NA	NA	NA	Potri.017G047500.2	
Potra2n17c31553.1	2.104353734	-1.18902202	0.074409189	NA		
Potra2n17c31554.1	-0.011193176	-0.292824555	0.984976766	0.907073354	Potri.017G047300.1	AT5G60440.1
Potra2n17c31555.1	1.750487693	0.688770112	0.211033645	NA	Potri.017G046800.1	
Potra2n17c31556.1	0.603695388	-0.780334612	0.72501439	NA	Potri.017G047100.1	
Potra2n17c31557.1	0.294840473	-1.101868527	0.87361168	NA		
Potra2n17c31558.1	NA	NA	NA	NA		
Potra2n17c31559.1	NA	NA	NA	NA	Potri.017G045600.1	
Potra2n17c31560.1	NA	0.674963689	NA	NA	Potri.017G046500.1	
Potra2n17c31561.1	0.82357019	NA	NA	NA		
Potra2n17c31562.1					Potri.017G045600.1	
Potra2n17c31563.1						
Potra2n17c31564.2	-0.408270762	-0.047730556	0.010441318	0.974275337	Potri.017G045100.1	AT2G22500.1
Potra2n17c31565.1	0.669627431	-1.495886149	NA	NA		
Potra2n17c31566.1	1.223782556	0.112096151	2.72257E-07	0.960981446	Potri.004G076200.1	AT3G02720.1

Potra2n17c31567.1	-1.299718849	-0.657981308	0.131602903	0.818188978	Potri.010G120950.1	AT5G38260.1
Potra2n17c31568.1	-0.619601162	-0.084578288	1.92769E-09	0.910815461	Potri.017G044700.1	AT4G33580.1
Potra2n17c31569.1	1.64774763	NA	NA	NA		
Potra2n17c31570.2	0.047992731	0.036918228	0.813762178	0.981702181		
Potra2n17c31571.1	0.273415539	0.257162642	0.669627242	0.932537571		
Potra2n17c31572.2	-0.596988467	1.615196645	0.830836468	0.864223715	Potri.017G044500.1	AT2G22540.1
Potra2n17c31573.1	0.793942997	0.244002501	0.837727792	NA	Potri.017G044500.1	AT2G22540.1
Potra2n17c31574.1	1.664677056	NA	NA	NA	Potri.007G115300.1	AT5G09550.1
Potra2n17c31575.1	0.165730228	-0.121289807	0.295235276	0.920141992	Potri.017G044000.1	AT1G09730.2
Potra2n17c31576.1	0.509491157	-0.055551579	0.03946851	0.981772014	Potri.017G043900.1	AT2G14110.1
Potra2n17c31577.1	-1.409983009	-0.127619696	NA	NA	Potri.017G043600.1	AT1G29500.1
Potra2n17c31578.1	-1.864134742	-0.395641182	NA	NA	Potri.017G043500.1	AT5G27780.1
Potra2n17c31579.1	NA	1.571529889	NA	NA	Potri.017G043500.1	AT5G27780.1
Potra2n17c31580.1	NA	0.674963689	NA	NA	Potri.017G043600.1	AT1G29500.1
Potra2n17c31581.1	0.107288592	-0.164671003	0.693208673	0.911339141	Potri.017G043900.1	AT2G14110.1
Potra2n17c31582.1	NA	NA	NA	NA	Potri.017G043600.1	AT1G29500.1
Potra2n17c31583.1	-2.307432468	0.433983763	NA	NA	Potri.017G043500.1	AT5G27780.1
Potra2n17c31584.1	-0.741867778	-0.488554298	0.535882594	0.947969749	Potri.017G043400.1	AT5G27780.1
Potra2n17c31585.1	-0.255190316	-0.101041161	0.053300123	0.842247476	Potri.017G043200.6	AT4G33625.1
Potra2n17c31586.1	-0.126005259	0.013660741	0.931245816	0.999325699		
Potra2n17c31587.1						
Potra2n17c31588.5	-0.102817426	0.14212257	0.303654972	0.897389232	Potri.017G043100.4	AT4G33630.1
Potra2n17c31589.1	0.220328687	-0.134509087	0.366022209	0.930871994	Potri.017G043000.1	AT1G61000.1
Potra2n17c31590.1	-1.328951947	1.34958344	NA	NA		
Potra2n17c31591.1	-0.478686834	-0.129427382	0.036697828	0.867910364	Potri.017G042900.1	AT5G64380.1
Potra2n17c31592.1	-0.768639161	0.104418637	0.00293387	0.968407862	Potri.017G042800.1	AT5G49690.1
Potra2n17c31593.1	-2.295337707	-2.446267299	NA	NA	Potri.017G042600.1	AT2G22590.1
Potra2n17c31594.1	-1.08297085	-0.215664388	0.643354104	NA		
Potra2n17c31595.1	0.243814549	0.178591063	0.01153747	0.611349636	Potri.017G042500.1	AT5G64390.1
Potra2n17c31596.1	-0.05110959	-0.015841213	0.90253824	0.996032145	Potri.007G116400.1	AT4G33640.1
Potra2n17c31597.1	-0.085292087	-0.055392103	0.836439666	0.985393296		
Potra2n17c31598.1	-1.317292482	1.503467127	NA	NA		

Potra2n17c31599.2	0.520821628	0.100935404	8.09721E-06	0.864223715	Potri.017G042200.2	AT3G21175.2
Potra2n17c31600.1	0.357797151	-0.133404871	0.415340677	0.966005919	Potri.017G042100.1	AT4G32430.1
Potra2n17c31601.1					Potri.017G019600.1	AT5G44250.1
Potra2n17c31602.1	1.684296017	2.165539819	0.251986408	NA		
Potra2n17c31603.1	-0.201163112	0.262302355	0.566386073	0.857189843	Potri.007G132100.1	AT2G43465.1
Potra2n17c31604.1	-0.167698404	0.601790922	0.571109198	0.459379117	Potri.017G019900.1	AT4G37650.1
Potra2n17c31605.1	0.417544471	0.182868093	0.020813678	0.88661378	Potri.017G020000.1	AT3G59540.1
Potra2n17c31606.1	-0.181845629	-0.108252667	0.677484803	0.960113191	Potri.001G284000.1	
Potra2n17c31607.1	0.552857561	0.255956745	0.01880693	0.803684688	Potri.017G026100.2	AT3G20320.1
Potra2n17c31608.1	-0.882868756	-0.392404134	0.000186333	0.783421796	Potri.017G026200.1	AT1G08760.1
Potra2n17c31609.4	-0.57938813	-0.050011888	0.000110535	0.95172055	Potri.017G026300.1	AT1G08450.1
Potra2n17c31610.1	-0.394042291	0.13989549	0.014515116	0.912777843	Potri.017G026500.1	AT1G06130.2
Potra2n17c31611.3	-0.199066972	-0.07059881	0.056554168	0.917516356	Potri.017G026600.1	AT2G43410.2
Potra2n17c31612.1	0.074953888	0.022515183	0.567235052	0.978251387	Potri.017G027000.2	AT1G06110.1
Potra2n17c31613.1	-0.157390008	-0.35211139	0.130739801	0.269417208	Potri.017G027400.1	AT1G06070.1
Potra2n17c31614.1	-0.596800351	0.281699886	0.003968677	0.909675463	Potri.017G027500.1	AT2G43400.1
Potra2n17c31615.1	NA	NA	NA	NA	Potri.017G027600.1	AT3G59530.2
Potra2n17c31616.2	-0.555195873	-0.295926823	0.000740983	0.754074409	Potri.018G113100.3	AT5G35180.2
Potra2n17c31617.1	-0.554193756	-0.00932102	0.001473963	0.995579852	Potri.017G027800.1	AT3G59520.1
Potra2n17c31618.1	4.710446426	1.65098336	2.5721E-45	0.284420596	Potri.017G027900.1	AT3G13710.1
Potra2n17c31619.1	-1.399618989	-0.724230258	0.112152587	0.787760802	Potri.017G022466.1	AT3G59580.2
Potra2n17c31620.1	2.632524198	NA	NA	NA	Potri.017G022400.1	
Potra2n17c31621.1	-0.061645226	0.82823047	NA	NA	Potri.017G022300.2	AT3G59580.1
Potra2n17c31622.1	1.095534932	0.453720288	7.33239E-07	0.22901885	Potri.017G020600.1	AT2G43500.2
Potra2n17c31623.1	-1.902049286	-0.29679844	0.148212985	0.971752582	Potri.017G018800.1	AT4G15880.1
Potra2n17c31624.1	5.254516003	1.520234448	1.5129E-17	0.732708854	Potri.T078300.1	AT2G01570.1
Potra2n17c31625.1	6.704145344	3.985931312	5.9634E-41	0.006206365	Potri.017G027900.1	AT3G13710.1
Potra2n17c31626.1	4.747403561	1.030871966	2.10505E-16	0.825454445	Potri.017G027900.1	AT3G13710.1
Potra2n17c31627.1	2.85545712	0.707918106	2.18231E-17	0.583548322	Potri.017G028100.1	AT5G10610.1
Potra2n17c31628.1	-1.443545432	-0.064673975	4.37611E-07	0.980890614	Potri.017G028300.1	AT1G06040.1
Potra2n17c31629.1	0.08506767	-0.243718686	0.932394948	0.960981446	Potri.017G028400.1	AT3G59510.1
Potra2n17c31630.1	-0.067251003	0.05173355	0.664873958	0.958679295	Potri.017G028500.1	AT1G30890.2

Potra2n17c31631.2	-0.063130455	0.038862599	0.812717233	0.986840197	Potri.017G028800.2	AT4G17300.1
Potra2n17c31632.1	-0.073668836	0.032543058	0.798708053	0.9896961	Potri.017G029000.4	AT3G59480.1
Potra2n17c31633.1	-0.215562442	-0.056264098	0.363801012	0.953108412	Potri.014G176500.1	AT3G07500.1
Potra2n17c31634.1	0.235205997	0.192776424	0.217120382	0.874808359	Potri.017G029200.2	AT4G22720.1
Potra2n17c31635.1					Potri.T177200.1	AT5G42957.1
Potra2n17c31636.1	NA	0.674963689	NA	NA	Potri.T177200.1	AT5G42957.1
Potra2n17c31637.1					Potri.T177200.1	AT5G42957.1
Potra2n17c31638.1					Potri.T177200.1	AT5G42957.1
Potra2n17c31639.1	-0.181898638	0.160388039	0.521273067	0.930159505	Potri.017G029300.1	AT1G24430.1
Potra2n17c31640.1	NA	NA	NA	NA	Potri.005G172200.1	AT4G08500.1
Potra2n17c31641.1	3.550445216	1.381953902	0.4286819	NA	Potri.002G088900.1	AT4G08500.1
Potra2n17c31642.1	-2.138814248	-0.655858027	NA	NA	Potri.002G088900.1	AT4G08500.1
Potra2n17c31643.1	NA	1.911450358	NA	NA	Potri.T124700.1	AT3G07500.1
Potra2n17c31644.1	-1.979653451	-2.125673425	NA	NA	Potri.002G088900.1	AT4G08500.1
Potra2n17c31645.1	-0.595952689	-1.465929376	NA	NA	Potri.002G088900.1	AT4G08500.1
Potra2n17c31646.1	-0.239869095	0.380851491	0.499597436	0.71411664	Potri.014G176600.1	AT4G12850.1
Potra2n17c31647.1	2.115384252	2.307412514	NA	NA	Potri.005G172200.1	AT4G08500.1
Potra2n17c31648.1	-1.672332667	1.792073969	NA	NA	Potri.T124700.1	AT3G07500.1
Potra2n17c31649.1	3.761264091	-0.622352855	0.056980546	NA	Potri.002G088900.1	AT4G08500.1
Potra2n17c31650.1						
Potra2n17c31651.1						
Potra2n17c31652.1	3.517266058	1.121596789	0.103956027	NA	Potri.002G088900.1	AT4G08500.1
Potra2n17c31653.1	-1.362328201	-0.173907434	0.001955077	0.956865545		
Potra2n17c31654.1	-1.134803361	-0.370056443	0.000371534	0.874808359	Potri.017G029700.1	AT2G43290.1
Potra2n17c31655.1	0.933883504	0.02121573	0.000161632	0.990896655	Potri.017G029800.1	AT3G59430.2
Potra2n17c31656.1	-0.185100019	-0.221425259	0.480499026	0.823926359	Potri.017G030100.2	AT3G59400.1
Potra2n17c31657.1	-0.316722119	-0.20819703	0.243955871	0.864223715	Potri.017G030200.1	AT2G43310.1
Potra2n17c31658.1	-0.554466278	-0.058398262	0.020798049	0.97950444	Potri.017G030600.1	AT4G24220.1
Potra2n17c31659.1	-0.388323746	-0.416487723	0.790249378	NA	Potri.017G030600.1	AT4G24220.1
Potra2n17c31660.2					Potri.017G030832.1	AT1G05960.2
Potra2n17c31661.2	0.175310287	0.109019482	0.153338585	0.854608397	Potri.017G030900.2	AT1G05910.1
Potra2n17c31662.1	7.462917054	2.795259497	7.03536E-14	NA	Potri.017G031000.1	AT4G27410.2

Potra2n17c31663.1	2.005210896	0.452885217	0.003791258	0.948577865	Potri.017G030900.2	AT1G05910.1
Potra2n17c31664.1	7.636494599	3.702625739	7.28152E-09	0.241505788	Potri.017G031000.1	AT4G27410.2
Potra2n17c31665.1	1.616110904	-1.084465724	0.266639197	NA		
Potra2n17c31666.1	6.898786638	2.995930017	9.49782E-11	NA	Potri.017G031000.1	AT4G27410.2
Potra2n17c31667.1	-0.231089127	-0.286535584	0.747764731	0.932932656	Potri.017G031100.1	AT1G05910.1
Potra2n17c31668.1	-3.002531464	-0.772028905	NA	NA	Potri.017G030900.2	AT1G05910.1
Potra2n17c31669.1	NA	NA	NA	NA	Potri.T130100.1	
Potra2n17c31670.1	7.185462244	2.933387047	1.87459E-08	NA	Potri.017G031000.1	AT4G27410.2
Potra2n17c31671.1	2.576509932	1.693213065	0.195784052	NA		
Potra2n17c31672.1	7.386915016	2.901391285	1.02263E-11	NA	Potri.017G031000.1	AT4G27410.2
Potra2n17c31673.1	-0.226740892	0.038313024	0.176575904	0.98177802	Potri.017G041800.1	AT4G33650.1
Potra2n17c31674.2	-0.714777579	5.701435291	NA	NA	Potri.017G041700.7	AT3G25810.1
Potra2n17c31675.1	-0.549913322	-1.249806547	0.653780085	0.689693427	Potri.017G041600.1	AT3G22104.1
Potra2n17c31676.1					Potri.017G041500.1	AT3G49950.1
Potra2n17c31677.1	-0.627228079	0.172303807	0.703565679	0.987288338	Potri.017G041400.1	
Potra2n17c31678.1	0.058553998	-0.018525885	0.617768545	0.984373337	Potri.017G041300.1	AT1G11480.1
Potra2n17c31679.1	0.328930486	0.346555909	0.024540385	0.538284553	Potri.017G041100.1	AT4G15180.1
Potra2n17c31680.1	1.437334985	1.798459118	0.515830017	NA	Potri.017G040900.1	AT2G32280.1
Potra2n17c31681.1	-0.499958438	1.612009495	0.618276498	0.72680202	Potri.017G040800.1	AT4G15210.1
Potra2n17c31682.1	-0.467264842	1.586818615	0.624096651	0.720242695		
Potra2n17c31683.1	-2.230851398	-0.1390964	0.000346503	0.960981446	Potri.T101300.2	AT3G22070.1
Potra2n17c31684.1	-2.256074493	-0.170099143	0.000201879	0.953177702		
Potra2n17c31685.1	-1.413097245	0.08893627	0.595678759	NA	Potri.017G039800.1	AT5G60440.1
Potra2n17c31686.1	NA	NA	NA	NA	Potri.017G039900.1	AT3G22060.1
Potra2n17c31687.1	0.152462134	-1.55593228	NA	NA	Potri.T102001.1	AT3G22060.1
Potra2n17c31688.1					Potri.017G040200.1	AT3G22060.1
Potra2n17c31689.1	NA	NA	NA	NA	Potri.017G040200.1	AT3G22060.1
Potra2n17c31690.1						
Potra2n17c31691.1	NA	NA	NA	NA	Potri.017G040200.1	AT3G22060.1
Potra2n17c31692.1	1.459962178	NA	NA	NA	Potri.017G039900.1	AT3G22060.1
Potra2n17c31693.1	5.24437627	2.455013179	1.52299E-24	0.274923694	Potri.017G039600.2	AT1G05280.1
Potra2n17c31694.1	-0.037659229	0.075915447	0.847298953	0.935474933	Potri.017G039500.2	AT4G15240.1

Potra2n17c31695.1	0.682128857	0.368776256	0.076947717	0.79819686	Potri.017G039400.1	AT4G15248.1
Potra2n17c31696.1	0.955880348	0.032801061	0.077111976	0.995565508	Potri.017G039301.1	AT4G15250.1
Potra2n17c31697.1	0.785841314	1.008892807	0.019541105	0.252482202	Potri.017G039200.1	AT4G33090.1
Potra2n17c31698.1	0.450486617	1.845782831	0.862637044	0.820868072	Potri.T177200.1	AT5G42957.1
Potra2n17c31699.1	2.71835457	2.874079753	0.087236803	NA	Potri.T177200.1	AT5G42957.1
Potra2n17c31700.1	2.609755317	2.630320344	0.171192435	NA	Potri.T177200.1	AT5G42957.1
Potra2n17c31701.1	1.325662113	1.825643469	0.420002377	0.803948068	Potri.T177200.1	AT5G42957.1
Potra2n17c31702.4	0.110994525	0.288841037	0.5362227	0.797111689	Potri.017G039100.1	AT1G67120.1
Potra2n17c31703.2	0.048125829	0.160959814	0.781652376	0.900785351	Potri.017G039000.1	AT5G35730.1
Potra2n17c31704.1	0.232019702	0.085667297	0.009525136	0.912665563	Potri.017G038800.1	AT3G21865.1
Potra2n17c31705.1	0.698392126	0.168343207	0.000655178	0.903477395	Potri.017G038700.1	AT3G21820.1
Potra2n17c31706.1	-0.099931384	-0.153113192	0.590179383	0.882699919	Potri.017G038500.1	AT1G05270.1
Potra2n17c31707.1	0.824047125	0.061981213	0.001762134	0.977000726	Potri.017G038300.1	AT4G26400.1
Potra2n17c31708.1	-2.195971411	-0.395172103	0.001616918	0.692047169	Potri.017G038100.1	AT1G05260.1
Potra2n17c31709.1	-0.467024856	-0.302614533	0.081796988	0.792864576	Potri.017G037900.1	AT1G05260.1
Potra2n17c31710.4	0.360229808	-0.017282875	0.249225761	0.995261215	Potri.017G037800.1	
Potra2n17c31711.1					Potri.017G037600.1	AT2G32350.1
Potra2n17c31712.1	NA	NA	NA	NA	Potri.017G037500.1	AT2G32360.1
Potra2n17c31713.1	0.174356949	0.25983288	0.451716318	0.762567828	Potri.017G037200.1	AT3G21740.1
Potra2n17c31714.2	3.131766767	-0.046370319	0.3265062	NA	Potri.007G122700.1	AT4G27130.1
Potra2n17c31715.1	0.240004534	0.257462365	0.260748705	0.897789832	Potri.017G037000.1	AT2G46410.1
Potra2n17c31716.1	4.521823155	6.364481427	1.35682E-06	NA	Potri.017G036900.1	AT3G21720.1
Potra2n17c31717.1	0.92344177	0.593606098	5.4931E-10	0.522508444	Potri.001G418500.1	AT4G05320.2
Potra2n17c31718.2	0.927357205	0.574175039	4.70584E-10	0.52733716		
Potra2n17c31719.1	2.148165578	1.2604009	6.83823E-13	0.389989789	Potri.017G036700.1	AT5G40390.1
Potra2n17c31720.1	0.100082291	0.098077306	0.455581531	0.920141992	Potri.017G036600.1	AT2G43210.2
Potra2n17c31721.1	-0.201074995	0.598334606	0.689600964	0.715088274	Potri.017G036400.1	AT3G18110.1
Potra2n17c31722.1	-0.378255747	0.003746199	0.001519792	0.998412058	Potri.017G036300.1	AT3G59350.3
Potra2n17c31723.1	-0.300276576	-0.010176809	0.010683873	0.996032145	Potri.017G036200.1	AT1G54380.1
Potra2n17c31724.1	-0.014081848	0.330727084	0.970333727	0.817452127	Potri.017G036100.1	AT1G58210.1
Potra2n17c31725.2	-0.114263214	0.073691988	0.551772118	0.9632996	Potri.017G036000.1	AT3G49420.1
Potra2n17c31726.1	-0.12823336	-0.101188948	0.537936145	0.954419252	Potri.017G035800.1	AT3G59360.1

Potra2n17c31727.2	-0.092114789	0.120844014	0.587054445	0.901195443	Potri.017G035700.1	AT2G43250.1
Potra2n17c31728.1	0.593148744	0.265342817	0.078948584	0.882899048	Potri.017G035300.1	AT5G43740.2
Potra2n17c31729.1	1.060094867	-1.516780659	0.418926494	0.84487051		
Potra2n17c31730.1	2.990038189	NA	NA	NA		
Potra2n17c31731.1	NA	NA	NA	NA	Potri.014G114900.1	AT3G62240.1
Potra2n17c31732.1	0.789572344	0.325303779	3.10555E-05	0.689560382	Potri.T107066.1	AT5G43740.1
Potra2n17c31733.1	1.208242205	0.472297274	0.000117484	0.724604666	Potri.T107066.1	AT5G43740.1
Potra2n17c31734.1	1.333721182	0.765608578	0.001146364	0.436900046	Potri.017G033932.1	AT4G10780.1
Potra2n17c31735.1	1.869097582	0.850260361	0.033169221	0.824731243	Potri.017G035300.1	AT5G43740.2
Potra2n17c31736.1	NA	NA	NA	NA	Potri.017G035600.1	
Potra2n17c31737.1						
Potra2n17c31738.1	-0.227787585	-0.175814499	0.44096156	0.896089262	Potri.017G035700.1	AT2G43250.1
Potra2n17c31739.1	1.079009508	0.469243613	1.1612E-05	0.514638341	Potri.T107066.1	AT5G43740.1
Potra2n17c31740.1	0.481063612	0.368282227	0.040418682	0.621894291	Potri.T107066.1	AT5G43740.1
Potra2n17c31741.1	4.982327794	1.150641466	0.001536657	NA		
Potra2n17c31742.1	0.964276146	1.039921149	0.0041077	0.115675065	Potri.017G034300.1	
Potra2n17c31743.1	4.146505708	NA	0.132284724	NA		
Potra2n17c31744.1	-0.104131594	-0.035563107	0.428892206	0.974264269	Potri.017G035200.1	AT2G31600.1
Potra2n17c31745.1	-0.069085267	-0.137937608	0.93600412	0.95359265	Potri.012G053300.2	AT2G01050.1
Potra2n17c31746.1	-0.027086396	-0.19413604	0.979134095	0.917751882	Potri.017G033600.1	AT5G63380.1
Potra2n17c31747.2	-0.010431159	-0.161342175	0.992253987	0.940255038	Potri.017G033500.1	
Potra2n17c31748.1	-0.017881832	0.028188227	0.936621629	0.984292161	Potri.017G033400.1	AT2G43370.1
Potra2n17c31749.1	0.06514251	-0.356202218	0.761069366	0.507498694	Potri.017G033300.2	AT2G43360.1
Potra2n17c31750.1	0.265962089	0.117277422	0.014803479	0.80654978	Potri.017G033200.3	AT4G10320.1
Potra2n17c31751.1	0.643950144	0.151993677	NA	NA	Potri.007G126600.1	AT2G31570.1
Potra2n17c31752.1	0.028393815	0.080828904	0.93366676	0.972159498	Potri.017G033000.1	AT5G65560.1
Potra2n17c31753.1	0.140886466	0.682817226	0.786884657	NA	Potri.017G032800.1	AT1G05870.3
Potra2n17c31754.1	6.453231489	1.571529889	3.54662E-06	NA	Potri.017G032700.1	AT1G05680.1
Potra2n17c31755.1	0.573004318	0.445347814	0.00708736	0.802631112	Potri.017G032200.1	AT2G43330.1
Potra2n17c31756.1	-0.327038044	0.000369078	0.000406297	0.999937671	Potri.017G032000.1	AT2G31510.1
Potra2n17c31757.3	1.457431187	0.914649993	4.72166E-20	0.00010984	Potri.017G031800.1	AT2G47680.1
Potra2n17c31758.1	0.260928445	0.199233645	0.013509026	0.497543905	Potri.017G031700.1	AT2G43320.1

Potra2n17c31759.1	8.189216816	5.322020122	3.28355E-10	NA	Potri.017G031000.1	AT4G27410.2
Potra2n17c31760.1	1.657274424	-1.124317913	0.196468081	NA	Potri.010G030800.4	AT5G19070.1
Potra2n17c31761.1	0.851484339	0.51283957	0.242254613	0.905991243	Potri.017G030900.2	AT1G05910.1
Potra2n17c31762.1	4.749535439	1.578929132	0.002028928	NA	Potri.017G031000.1	AT4G27410.2
Potra2n17c31763.1	NA	1.571529889	NA	NA	Potri.007G127900.1	AT1G05910.1
Potra2n17c31764.1	-1.35927387	-1.507841271	NA	NA		
Potra2n17c31765.1	2.282771052	2.303900616	NA	NA	Potri.017G031400.2	AT1G05910.1
Potra2n17c31766.1					Potri.017G022400.1	
Potra2n17c31767.2	1.27463317	0.503100004	4.53932E-11	0.005615912	Potri.017G020600.1	AT2G43500.2
Potra2n17c31768.1	-0.645586038	0.222587143	0.226610112	0.910815461	Potri.017G021966.1	AT2G43500.1
Potra2n17c31769.1	0.49705051	0.146157854	0.507198843	0.974275337	Potri.013G084600.1	
Potra2n17c31770.1						
Potra2n17c31771.1	1.260580939	0.515651399	5.83624E-13	0.16018022	Potri.017G020600.1	AT2G43500.2
Potra2n17c31772.1	2.217130902	2.445678854	NA	NA	Potri.013G084600.1	
Potra2n17c31773.1					Potri.007G133200.1	
Potra2n17c31774.1	-0.158193111	0.093754587	0.620764938	0.970442612	Potri.017G021700.1	AT2G43500.1
Potra2n17c31775.1	0.534418741	0.406533485	0.553983731	0.951718113	Potri.013G084600.1	
Potra2n17c31776.1	0.681638429	0.282499143	0.00057589	0.72476666	Potri.T078200.1	AT2G43500.1
Potra2n17c31777.1	-0.208087298	0.076052563	0.08052	0.943483421	Potri.017G018400.1	AT3G07560.1
Potra2n17c31778.1	-0.19834845	-0.326494328	0.395449675	0.595538573	Potri.T078000.1	AT5G40520.2
Potra2n17c31779.1					Potri.T077800.1	
Potra2n17c31780.1	-0.346091672	0.148895838	0.050391292	0.921063801	Potri.T077700.1	AT2G30700.1
Potra2n17c31781.1						
Potra2n17c31782.1	10.79654528	23.26976681	3.85514E-10	NA	Potri.017G018100.1	
Potra2n17c31783.1	NA	NA	NA	NA	Potri.T077800.1	
Potra2n17c31784.1	-0.352308805	0.003536019	0.03606233	0.99819408	Potri.T077700.1	AT2G30700.1
Potra2n17c31785.1	-0.01712081	0.01123636	0.94614517	0.993191555	Potri.007G133900.1	AT5G62270.2
Potra2n17c31786.1	-0.101905311	-0.258072653	0.691954081	0.802631112	Potri.017G017800.1	AT4G04885.1
Potra2n17c31787.2	0.317233668	0.158763989	0.031513105	0.922011871	Potri.017G017700.1	AT1G06720.1
Potra2n17c31788.2	3.950593329	-0.785798209	2.13688E-08	NA	Potri.017G017600.1	AT1G17950.1
Potra2n17c31789.1	-0.17457554	-0.088883293	0.842554247	0.985393296	Potri.017G017500.1	AT2G43560.1
Potra2n17c31790.1	0.400154282	0.241578499	0.055831878	0.860325259	Potri.017G017400.2	AT2G20530.1

Potra2n17c31791.1	0.667093342	0.196980521	0.010511044	0.895460013	Potri.017G017200.1	AT5G44150.1
Potra2n17c31792.1	0.760967776	0.312371136	0.044805635	0.865611082	Potri.017G017300.1	AT1G28480.1
Potra2n17c31793.1	-0.138986286	-0.227759676	0.925672737	0.983113084	Potri.017G017100.1	AT1G03840.1
Potra2n17c31794.1	0.22893033	-0.008944729	0.63612409	0.996032145	Potri.017G017000.1	AT5G44170.1
Potra2n17c31795.1	1.754916895	-0.062945497	NA	0.99005311	Potri.017G016900.1	AT5G53980.1
Potra2n17c31796.1	0.110121541	0.028170319	0.365175403	0.98036348	Potri.017G016800.2	AT5G46070.1
Potra2n17c31797.1	-0.780606863	-0.329445814	2.95462E-05	0.818161208	Potri.017G016700.1	AT4G28500.1
Potra2n17c31798.1	2.635954721	0.648669972	1.12336E-29	0.663950227	Potri.017G016600.1	AT1G28440.1
Potra2n17c31799.1	1.64774763	NA	NA	NA		
Potra2n17c31800.1	0.628905532	0.697378594	0.635214512	0.900676614	Potri.017G016500.1	
Potra2n17c31801.1	0.82357019	NA	NA	NA	Potri.017G016300.1	AT4G18425.1
Potra2n17c31802.1	-0.46511576	-0.095178869	0.011788356	0.924104906	Potri.017G016200.1	AT5G44180.1
Potra2n17c31803.1	-0.107399146	-0.155709525	0.488115105	0.784036768	Potri.017G016100.1	AT2G20560.1
Potra2n17c31804.1	-0.299868457	0.094637126	0.153452444	0.949966783	Potri.017G016000.1	AT2G20560.1
Potra2n17c31805.1	-3.279216522	0.08893687	NA	NA	Potri.017G015900.1	AT2G20562.1
Potra2n17c31806.1	1.64774763	4.225123093	NA	NA	Potri.017G015800.1	AT2G20570.1
Potra2n17c31807.1	0.448283744	-0.182666091	0.118029918	0.923307655	Potri.017G015700.2	AT1G03820.1
Potra2n17c31808.1	0.226942557	0.076064676	0.038964529	0.940361195	Potri.017G014800.1	AT2G20580.1
Potra2n17c31809.1	-0.043988573	-0.037530103	0.819064245	0.974908697	Potri.017G014600.1	AT5G44200.1
Potra2n17c31810.1	0.615636962	0.155965192	2.27057E-06	0.895474078	Potri.017G014500.2	AT2G20585.1
Potra2n17c31811.2	0.633853794	0.168403408	9.84097E-07	0.872201371	Potri.017G014400.1	AT4G28440.1
Potra2n17c31812.3	3.05945276	0.899850362	1.85916E-30	0.503008551	Potri.017G014300.1	AT2G20590.1
Potra2n17c31813.2	-0.632803267	-0.178256369	0.028558828	0.930203195	Potri.017G014100.1	AT5G53970.1
Potra2n17c31814.1	-0.773759995	-0.350851886	0.000333152	0.788065047	Potri.017G013700.1	AT5G44210.1
Potra2n17c31815.1	-0.216919397	-0.383615322	0.43493204	0.699507809	Potri.001G465401.1	AT4G20900.1
Potra2n17c31816.1	0.003512934	-0.425007309	0.992773786	0.779077802		
Potra2n17c31817.1	0.428769809	0.108403189	0.000505753	0.862335622	Potri.017G013500.1	AT5G44240.2
Potra2n17c31818.1	-0.61910233	-0.767708831	NA	NA	Potri.017G013400.1	AT1G03790.1
Potra2n17c31819.1	0.788739972	-0.358069132	0.026656105	0.895560236	Potri.017G013300.2	AT4G28400.1
Potra2n17c31820.1					Potri.017G013200.1	AT5G44265.1
Potra2n17c31821.7	0.30704461	-0.003633197	0.10737037	0.998245864	Potri.007G138600.1	AT1G03780.2
Potra2n17c31822.1	-0.325131015	0.003502494	0.045677385	0.997923708	Potri.017G013000.1	AT5G44280.1

Potra2n17c31823.1					Potri.018G089600.1	AT2G01050.1
Potra2n17c31824.1	0.893180339	0.04196084	1.31165E-07	0.982422288	Potri.017G012800.1	AT4G28390.1
Potra2n17c31825.1	-0.507037562	-0.330258644	0.004919304	0.73777233		
Potra2n17c31826.1	-0.105547561	0.070106249	0.490198706	0.950491075	Potri.017G012500.1	AT1G03760.1
Potra2n17c31827.1	-0.213260943	-0.181600521	0.221101359	0.794596445	Potri.017G012400.1	AT1G03750.1
Potra2n17c31828.1	0.059605483	-0.048201088	0.981050115	NA	Potri.017G011200.1	AT4G28365.1
Potra2n17c31829.1	-0.937611701	-2.446267299	NA	NA	Potri.017G012200.3	AT4G12560.1
Potra2n17c31830.1	-0.595952689	0.151993677	NA	NA	Potri.017G010500.1	AT3G49190.1
Potra2n17c31831.2	4.041842832	1.363811008	0.137742642	NA	Potri.017G012100.1	AT1G19715.1
Potra2n17c31832.1	3.019853505	NA	NA	NA		
Potra2n17c31833.1	10.84431988	20.93368559	1.47494E-12	NA	Potri.017G010900.2	AT1G19715.1
Potra2n17c31834.1	9.105148413	5.826742624	1.29029E-06	NA	Potri.017G010900.2	AT1G19715.1
Potra2n17c31835.1	1.64774763	NA	NA	NA		
Potra2n17c31836.2	0.228224791	-1.465929376	NA	NA	Potri.003G041650.1	AT2G34400.1
Potra2n17c31837.1	6.526326204	3.668669004	5.50729E-28	0.077188177	Potri.003G041400.2	AT1G74170.1
Potra2n17c31838.1	1.664677056	1.363811008	NA	NA		
Potra2n17c31839.1	0.82357019	1.363811008	NA	NA	Potri.003G042000.1	
Potra2n17c31840.1	NA	1.394613675	NA	NA	Potri.003G041501.1	AT2G34400.1
Potra2n17c31841.1	7.444402899	5.390768431	5.4895E-09	NA	Potri.003G041600.2	AT1G54470.2
Potra2n17c31842.1	3.724941375	NA	0.198966381	NA	Potri.003G041700.2	AT1G58190.2
Potra2n17c31843.1	-1.317292482	-1.465929376	NA	NA		
Potra2n17c31844.1	2.409567423	NA	NA	NA	Potri.T096600.1	AT5G10610.1
Potra2n17c31845.1	NA	NA	NA	NA		
Potra2n17c31846.1						
Potra2n17c31847.1						
Potra2n17c31848.1	-0.405980349	0.230820522	0.511773293	0.905675187	Potri.017G019850.1	AT2G43465.1
Potra2n17c31849.1						
Potra2n17c31850.1	NA	NA	NA	NA	Potri.017G012200.3	AT4G12560.1
Potra2n17c31851.1	4.04176612	-0.744589582	0.010741706	NA	Potri.017G012100.1	AT1G19715.1
Potra2n17c31852.1						
Potra2n17c31853.1	NA	NA	NA	NA	Potri.017G012200.3	AT4G12560.1
Potra2n17c31854.1	5.197402909	2.32414027	0.001821937	NA	Potri.017G012000.1	

Potra2n17c31855.2	3.295749178	2.346460774	0.027723138	NA	Potri.017G010900.2	AT1G19715.1
Potra2n17c31856.1	0.328054513	0.313162985	0.000117605	0.221056825	Potri.017G011800.10	AT5G46470.1
Potra2n17c31857.1	NA	2.509020267	NA	NA		
Potra2n17c31858.1	1.495976022	4.200402999	0.464595808	NA	Potri.017G010700.1	AT3G49190.1
Potra2n17c31859.1	4.525890413	1.294611326	1.59408E-44	0.767368598	Potri.017G010600.1	AT3G26040.1
Potra2n17c31860.1	1.651310715	0.78108239	0.244842381	NA	Potri.017G010500.1	AT3G49190.1
Potra2n17c31861.1	0.732399841	-0.51126587	0.751461422	NA	Potri.017G010300.1	AT5G53390.1
Potra2n17c31862.1	0.159523674	0.124196884	0.341049384	0.913281585	Potri.017G010200.1	AT2G43790.1
Potra2n17c31863.1	0.090411863	0.109790523	0.607370616	0.943483421	Potri.017G010100.1	AT3G59800.1
Potra2n17c31864.1	-0.28648469	-0.222786907	0.304118263	0.906679354	Potri.017G009900.1	AT3G25500.1
Potra2n17c31865.1	-0.134630007	-0.111687286	0.593213242	0.954419252	Potri.017G009800.1	AT2G30720.1
Potra2n17c31866.1	0.024461792	0.149543472	0.890277693	0.816392132	Potri.017G009700.1	AT1G05690.1
Potra2n17c31867.1	1.219884824	1.046136803	6.11449E-08	0.217446264	Potri.017G009600.1	AT1G66910.1
Potra2n17c31868.1					Potri.017G009500.1	AT5G38260.1
Potra2n17c31869.3	5.403266011	2.134902029	0.000450433	NA	Potri.017G008600.2	AT1G67000.1
Potra2n17c31870.1	-0.025529276	0.205296616	0.919447697	0.848131816	Potri.017G008300.1	AT2G31800.1
Potra2n17c31871.1	3.908516794	0.674963689	0.073582572	NA	Potri.017G008200.1	AT1G66980.1
Potra2n17c31872.1	NA	1.363811008	NA	NA	Potri.012G108400.1	AT1G26880.1
Potra2n17c31873.1						
Potra2n17c31874.1	-0.175085378	-0.000942926	0.390253895	0.999536469	Potri.017G008300.1	AT2G31800.1
Potra2n17c31875.1	6.067539001	2.134902029	7.87622E-06	NA	Potri.017G009400.2	AT1G66920.2
Potra2n17c31876.1	3.930589719	0.674963689	0.160706465	NA	Potri.017G009300.1	
Potra2n17c31877.1	8.330998399	5.443360365	2.73481E-12	NA	Potri.017G007900.1	AT5G38260.1
Potra2n17c31878.1	-0.312236461	-0.033498473	0.021330944	0.975578749	Potri.017G007800.2	AT2G31800.1
Potra2n17c31879.1	-0.789201114	-0.458605324	1.15198E-06	0.185318547	Potri.017G007700.1	AT2G44065.1
Potra2n17c31880.2	4.106334441	0.884136698	5.75653E-08	NA	Potri.017G007600.1	AT5G48380.1
Potra2n17c31881.1	0.025312243	-0.00093153	0.90510171	0.999536469	Potri.017G007500.1	AT2G43980.1
Potra2n17c31882.1	-0.380425988	-0.080173308	0.033048592	0.966228173	Potri.017G007400.1	AT2G43970.1
Potra2n17c31883.1	0.401346231	0.13642735	0.036560746	0.936890993	Potri.017G007300.1	AT2G43950.1
Potra2n17c31884.1	-0.187006089	0.109219055	0.282425431	0.923397137	Potri.007G145000.2	AT5G24450.1
Potra2n17c31885.1	-0.03820071	0.517300894	0.922403842	0.639292153	Potri.017G007000.1	AT2G43940.1
Potra2n17c31886.2	0.325918216	0.147121135	0.097934993	0.922029226	Potri.017G006900.1	AT2G43900.1

Potra2n17c31887.1	0.318990801	0.146749247	0.106392223	0.92223649	Potri.017G006800.1	AT2G31820.1
Potra2n17c31888.1	NA	0.674963689	NA	NA	Potri.017G006700.1	AT2G43890.1
Potra2n17c31889.1	-0.059372787	-2.456974026	NA	NA	Potri.017G006700.1	AT2G43890.1
Potra2n17c31890.1	NA	NA	NA	NA	Potri.017G006400.1	AT1G05660.1
Potra2n17c31891.1	8.649526226	5.760721244	4.85785E-12	0.380665007	Potri.017G005800.1	AT3G59850.1
Potra2n17c31892.1	6.016088586	2.889284214	3.7661E-05	NA	Potri.017G006000.1	AT5G37980.1
Potra2n17c31893.1	2.286123637	0.686168905	0.014566488	0.930598202	Potri.017G005300.1	AT3G59850.1
Potra2n17c31894.1	7.284294014	4.294010224	7.627E-44	0.409656627	Potri.017G006200.2	AT3G59850.1
Potra2n17c31895.1	2.315713269	0.674963689	NA	NA	Potri.017G005400.1	AT5G37980.1
Potra2n17c31896.1	1.664677056	NA	NA	NA	Potri.017G005300.1	AT3G59850.1
Potra2n17c31897.1	7.341317846	4.954588618	2.49824E-22	0.004284785		
Potra2n17c31898.1	7.022671703	4.819522371	3.36354E-28	0.000370409	Potri.017G004900.1	AT5G48380.1
Potra2n17c31899.1	6.807615386	3.334968888	5.39312E-07	NA	Potri.017G003400.2	AT5G48380.1
Potra2n17c31900.1	8.518946496	5.39657804	4.63397E-13	0.660818471	Potri.T075000.1	AT5G48380.1
Potra2n17c31901.1	0.136654883	0.368694117	0.590011788	0.739520512	Potri.T075900.1	AT5G16970.1
Potra2n17c31902.1	-0.558688792	0.102596296	0.51111206	0.974756511	Potri.017G002800.1	AT2G01050.1
Potra2n17c31903.1	-0.240761454	0.408488651	0.306951048	0.679660122	Potri.017G002500.3	AT3G03080.1
Potra2n17c31904.1	-0.453773918	0.161415781	0.391261651	0.953768643	Potri.T076400.1	AT2G01050.1
Potra2n17c31905.1	-0.171329557	0.084112878	0.778765819	0.984292161	Potri.T076250.1	AT5G16980.2
Potra2n17c31906.1	-0.040796582	-0.388785791	0.964610993	0.922011871	Potri.017G002400.1	AT2G01050.1
Potra2n17c31907.1	0.065174368	0.298037466	0.768120033	0.824273962	Potri.T076700.1	AT1G26320.1
Potra2n17c31908.1	-0.382048017	-0.256182811	0.547694502	0.941855873	Potri.017G002200.1	AT2G43990.1
Potra2n17c31909.1	0.751775862	0.568572332	1.85324E-05	0.246133003	Potri.017G002000.1	AT2G31870.2
Potra2n17c31910.1	0.234180085	0.020466465	0.253582225	0.987226805	Potri.017G001900.1	AT3G59890.1
Potra2n17c31911.1	0.333749962	0.021804184	0.089305221	0.9869326	Potri.017G001700.1	AT1G05610.1
Potra2n17c31912.1	-0.238490838	0.044037091	0.185850118	0.971317865	Potri.017G001600.1	AT2G31890.1
Potra2n17c31913.1	-0.037141454	-0.093136507	0.937266812	0.963557834		
Potra2n17c31914.1	-0.324477674	-0.141090003	0.004970609	0.842247476	Potri.017G001300.3	AT3G59910.1
Potra2n17c31915.1	0.145556831	-0.079818046	0.218369568	0.937846405	Potri.017G001200.2	AT2G44100.1
Potra2n17c31916.2	0.870687559	0.189234851	6.84741E-07	0.722616537	Potri.017G001000.2	AT4G21065.1
Potra2n17c31917.4	-0.395788459	0.029872589	0.028671648	0.983113084	Potri.017G000800.1	AT4G02600.2
Potra2n17c31918.1	0.021422128	0.567229454	0.980326987	0.852273781	Potri.001G155750.1	AT1G21280.1

Potra2n17c31919.1	0.971682453	0.16907527	0.007386776	0.765390763	Potri.017G000900.1	AT4G02600.2
Potra2n17c31920.1	-1.752221408	-0.622796196	2.80941E-22	0.250129374	Potri.017G000700.1	AT3G59940.1
Potra2n17c31921.1	-0.280978658	-0.153269164	0.004386683	0.824317405	Potri.017G000500.1	AT2G44140.1
Potra2n17c31922.2	0.73787224	-0.449096384	0.107325266	0.832270846	Potri.017G000400.4	AT2G44150.1
Potra2n17c31923.1	1.489638499	-0.191668432	0.150351109	NA		
Potra2n17c31924.1	-0.252775299	-0.214617126	0.281903718	0.912777843	Potri.007G147300.1	AT2G44160.1
Potra2n17c31925.1	-0.305291732	-0.257320854	0.158104977	0.885628484		
Potra2n17c31926.1	-0.352104071	0.215124367	0.28736795	0.902847514	Potri.017G000200.1	AT3G59980.1
Potra2n17c31927.2	0.132895087	0.719803224	0.693449825	0.636664382	Potri.017G000100.1	AT5G10860.1
Potra2n17c31928.1	1.64774763	NA	NA	NA	Potri.017G003100.2	AT5G48380.1
Potra2n17c31929.1	-1.409983009	-1.55593228	NA	NA	Potri.T075400.2	AT5G48380.1
Potra2n17c31930.1	2.065771688	-0.046370319	NA	NA	Potri.017G004900.1	AT5G48380.1
Potra2n17c31931.1	-2.163225087	-0.659908307	0.009658091	0.860325259		
Potra2n17c31932.1	1.266042526	-1.03811561	0.405572062	NA	Potri.T074900.2	AT5G48380.1
Potra2n17c31933.1	0.963595307	-1.429847359	0.551343011	NA		
Potra2n180s34806.1	3.550995766	1.387398048	0.000395455	NA	Potri.010G012400.1	AT3G16660.1
Potra2n181s34807.1	2.386971288	0.620970461	3.31468E-07	0.544330018	Potri.001G386300.1	AT1G53440.1
Potra2n183s34808.1	NA	NA	NA	NA	Potri.013G100900.1	AT2G01050.1
Potra2n185s34809.1	3.241896319	2.118341335	6.97319E-08	0.148037164	Potri.015G025100.2	AT2G34930.1
Potra2n185s34810.1	5.38855565	1.895645992	2.27066E-12	NA	Potri.015G025100.2	AT2G34930.1
Potra2n185s34811.1	3.862051363	2.288120963	2.28826E-08	0.237361979	Potri.015G025100.2	AT2G34930.1
Potra2n185s34812.1	1.399690428	0.707886087	0.004747975	0.697578966	Potri.015G024500.2	AT2G34930.1
Potra2n185s34813.1	-0.463354164	0.05860585	0.101609768	0.98177802		
Potra2n185s34814.1	4.817562199	2.673140994	7.04394E-26	0.001910782	Potri.015G025100.2	AT2G34930.1
Potra2n186s34815.1	-0.844745849	0.296829027	0.158252119	0.943483421	Potri.009G024100.1	AT2G31955.1
Potra2n186s34816.1	NA	NA	NA	NA	Potri.001G230600.1	AT1G31350.1
Potra2n187s34817.1	-1.409983009	-1.55593228	NA	NA	Potri.011G027400.2	AT4G05220.1
Potra2n18c31934.1	-0.196733615	0.209849503	0.909413545	NA	Potri.017G155200.1	AT1G17830.1
Potra2n18c31935.1	-0.488969654	0.083952355	0.003401154	0.914162682	Potri.018G152400.1	AT1G17820.1
Potra2n18c31936.1	0.328612146	-0.473822882	0.278315895	0.590365286	Potri.018G152300.2	AT4G33990.1
Potra2n18c31937.1	5.567248787	1.554117716	8.53086E-71	0.340603139	Potri.018G152200.4	AT5G08370.1
Potra2n18c31938.1	1.095604851	0.862675244	NA	NA	Potri.018G152100.1	AT1G73190.1

Potra2n18c31939.1	1.59577253	0.58279435	1.56894E-05	0.767368598	Potri.018G152000.1	
Potra2n18c31940.1	-0.014239407	-0.159215978	0.943015356	0.869175327	Potri.018G151900.1	AT5G58210.2
Potra2n18c31941.1	NA	NA	NA	NA	Potri.006G081700.1	AT3G58970.1
Potra2n18c31942.2	-0.41648649	0.396800361	0.072717879	0.595538573	Potri.018G151600.1	AT3G13050.1
Potra2n18c31943.1	-0.816433825	0.897621515	0.212214	0.590087814	Potri.018G074600.2	AT1G03670.1
Potra2n18c31944.1					Potri.006G081700.1	AT3G58970.1
Potra2n18c31945.3	0.024206271	-0.17042169	0.932380832	0.872233138	Potri.018G151400.1	AT3G13040.2
Potra2n18c31946.1	0.641158456	0.295765119	1.14246E-11	0.461392125	Potri.018G151300.1	AT5G58130.1
Potra2n18c31947.1	0.020623614	0.056541074	0.893815425	0.957163493	Potri.018G150900.1	AT5G53530.1
Potra2n18c31948.1	2.074019598	0.729975245	3.65411E-22	0.324084615	Potri.018G150800.1	AT4G28400.1
Potra2n18c31949.1	NA	1.900652309	NA	NA	Potri.018G151600.1	AT3G13050.1
Potra2n18c31950.1	2.432738802	0.556577252	5.93924E-55	0.595538573	Potri.018G150700.3	AT3G13050.1
Potra2n18c31951.1	-0.029976163	0.186051416	0.843395084	0.784845657	Potri.018G150600.2	AT5G58100.1
Potra2n18c31952.1	-0.184786334	0.145043457	0.317074715	0.909025231	Potri.018G150400.1	AT5G58090.1
Potra2n18c31953.1	0.065533273	0.055199323	0.685175666	0.949960494	Potri.006G080400.1	AT2G26250.1
Potra2n18c31954.1	-0.674168651	0.148762035	0.016490247	0.956865545	Potri.018G150200.2	AT1G06330.1
Potra2n18c31955.1	-0.836891668	-0.112132808	0.023390116	0.966407607	Potri.018G150000.1	AT4G31040.1
Potra2n18c31956.3	0.335944062	0.09230051	0.065748317	0.927717809	Potri.018G149900.2	AT5G43270.1
Potra2n18c31957.1	-0.139549871	-0.035855533	0.215146172	0.974275337	Potri.018G149800.1	AT5G58190.2
Potra2n18c31958.1	1.17862389	0.773571697	0.01282401	0.755109612	Potri.010G055200.1	AT3G13080.1
Potra2n18c31959.1	1.88773863	0.499831698	0.384326292	NA	Potri.018G149600.1	AT3G13080.1
Potra2n18c31960.1	-0.445540561	0.216257354	4.82468E-05	0.688499606	Potri.018G149500.1	AT4G18240.1
Potra2n18c31961.1	-0.103023986	-0.048126458	0.609272269	0.971537796	Potri.018G149400.1	AT2G07360.1
Potra2n18c31962.1	2.310345434	1.809877165	3.73204E-15	0.351105764	Potri.018G149300.1	AT5G36110.1
Potra2n18c31963.1	-0.304934136	0.034777967	0.275125045	0.9869326	Potri.018G149201.1	AT2G07340.1
Potra2n18c31964.1	-0.221377091	-0.008377874	0.035830608	0.995409858	Potri.018G149100.2	AT2G07180.1
Potra2n18c31965.1	0.82357019	NA	NA	NA	Potri.018G149000.1	AT2G07170.1
Potra2n18c31966.1	NA	NA	NA	NA	Potri.018G148900.1	AT1G01490.1
Potra2n18c31967.1	-0.084667858	-0.226155068	0.798096601	0.879147863	Potri.018G148800.1	AT2G07050.1
Potra2n18c31968.1	-0.093686679	-0.535929217	0.857576477	0.689540835		
Potra2n18c31969.1					Potri.018G148700.1	AT1G04645.1
Potra2n18c31970.1	0.82357019	NA	NA	NA	Potri.T126500.1	AT1G16120.1

Potra2n18c31971.1	2.685015931	2.315796285	NA	NA	Potri.T126600.1	AT1G19390.1
Potra2n18c31972.1	1.545832463	NA	NA	NA	Potri.018G148400.1	AT1G69730.1
Potra2n18c31973.1	4.54347136	4.082055521	0.083677834	NA	Potri.T161800.1	AT1G58190.2
Potra2n18c31974.1	3.418021343	1.059555177	2.36741E-09	0.732556792	Potri.006G078800.1	AT2G26590.2
Potra2n18c31975.1	0.705352422	0.18113499	0.011074791	0.914903229	Potri.018G147400.2	AT5G35400.1
Potra2n18c31976.1					Potri.018G124266.1	AT2G26590.3
Potra2n18c31977.1	0.237198735	0.321523902	0.526488996	0.855432225	Potri.012G108000.1	AT2G34400.1
Potra2n18c31978.1	-1.987102176	-1.595207793	0.497259006	NA	Potri.018G147900.1	AT2G34400.1
Potra2n18c31979.1	2.445332871	0.480956292	0.00010003	0.923348116	Potri.006G077000.1	AT2G19090.1
Potra2n18c31980.1	0.255932676	0.328685159	0.533690284	0.824812052	Potri.T161600.1	AT2G19080.1
Potra2n18c31981.1	0.470973197	0.013615606	0.009302318	0.993589787	Potri.018G145200.1	AT3G53430.1
Potra2n18c31982.1	0.680448658	0.164496584	4.24345E-05	0.895474078	Potri.018G145200.1	AT3G53430.1
Potra2n18c31983.1	-0.280752732	-0.230090721	0.437763638	0.930203195	Potri.018G146500.1	AT5G41685.1
Potra2n18c31984.2	0.099432956	0.136972815	0.509546955	0.888518641	Potri.018G146700.1	AT5G35330.1
Potra2n18c31985.1	0.030322992	0.037851446	0.882757256	0.97601212	Potri.018G145600.1	
Potra2n18c31986.1	-0.134613943	-0.018195698	0.730836878	0.993815835		
Potra2n18c31987.1	0.199290375	0.190230275	0.471556652	0.894276674		
Potra2n18c31988.1	NA	NA	NA	NA		
Potra2n18c31989.1	0.988184768	1.102180974	0.348583867	0.816567218	Potri.018G145700.1	AT4G16130.1
Potra2n18c31990.2	-0.213564189	-0.061318354	0.210654715	0.955406656	Potri.018G145800.2	AT1G30330.2
Potra2n18c31991.1	-0.023117075	-0.039015615	0.845188404	0.965249957	Potri.018G145900.1	AT4G31300.3
Potra2n18c31992.1	0.000104022	0.208123637	0.999895833	0.948577865	Potri.006G078000.1	AT2G06990.1
Potra2n18c31993.1	3.472885412	0.019948791	2.41688E-05	0.998307362	Potri.018G146100.1	AT2G30490.1
Potra2n18c31994.1	NA	NA	NA	NA		
Potra2n18c31995.3	1.043404717	1.292755066	0.00670514	0.258140976	Potri.018G146300.1	AT5G35360.1
Potra2n18c31996.1					Potri.018G146400.1	
Potra2n18c31997.1	0.24871003	-0.110867954	0.304366177	0.971752582	Potri.018G146800.1	AT5G20700.1
Potra2n18c31998.1	-0.395008901	-0.138448548	0.060818195	0.878118098	Potri.018G146900.1	AT3G42630.1
Potra2n18c31999.1	-0.372018544	-0.114788349	0.241971376	0.936890993	Potri.006G078500.2	AT2G24370.1
Potra2n18c32000.1	0.388800799	-0.70912244	0.587793633	0.835256642	Potri.018G147100.1	AT2G32235.1
Potra2n18c32001.1	0.82357019	NA	NA	NA	Potri.018G147200.1	AT2G24370.1
Potra2n18c32002.1	0.82357019	NA	NA	NA	Potri.018G147300.1	AT5G35390.1

Potra2n18c32003.1	0.473588906	0.28491385	0.015368953	0.771247259	Potri.018G147400.2	AT5G35400.1
Potra2n18c32004.1	3.019853505	1.363811008	NA	NA	Potri.018G132200.1	AT4G10310.1
Potra2n18c32005.1					Potri.018G124200.1	AT3G10572.1
Potra2n18c32006.1	0.86053622	1.782575044	0.632819138	0.696913019	Potri.006G078800.1	AT2G26590.2
Potra2n18c32007.1	5.366796217	2.327470073	7.04434E-47	0.108619662		
Potra2n18c32008.1					Potri.018G124332.1	AT2G26590.3
Potra2n18c32009.1	0.836909988	0.590834573	3.0963E-06	0.560708883	Potri.006G078900.1	AT4G04940.1
Potra2n18c32010.1					Potri.017G108200.1	AT5G03340.1
Potra2n18c32011.1	2.135340983	-1.332046727	0.177724695	NA	Potri.017G108200.1	AT5G03340.1
Potra2n18c32012.1	-1.92220879	-2.071795494	NA	NA	Potri.017G108200.1	AT5G03340.1
Potra2n18c32013.1	3.520783827	NA	0.258176609	NA	Potri.002G075950.1	AT1G21270.1
Potra2n18c32014.1	4.234984965	1.621387239	4.02228E-28	0.526356391	Potri.T126700.1	AT1G16120.1
Potra2n18c32015.1	4.519720827	1.783952595	2.06283E-28	0.514425946		
Potra2n18c32016.1	0.82357019	NA	NA	NA	Potri.018G148400.1	AT1G69730.1
Potra2n18c32017.1					Potri.T126700.1	AT1G16120.1
Potra2n18c32018.1	-1.807992024	-0.890530955	0.238464541	0.820880672		
Potra2n18c32019.1	0.168759767	0.670154529	0.784006205	0.71424814	Potri.006G124000.1	
Potra2n18c32020.2	3.346890086	1.811624378	2.66027E-09	0.277578139	Potri.002G075950.1	AT1G21270.1
Potra2n18c32021.2					Potri.T126700.1	AT1G16120.1
Potra2n18c32022.1	1.958036813	0.487752082	0.463257237	NA		
Potra2n18c32023.1						
Potra2n18c32024.1	0.82357019	0.674963689	NA	NA	Potri.T126500.1	AT1G16120.1
Potra2n18c32025.1	0.82357019	2.900818469	NA	NA	Potri.018G148400.1	AT1G69730.1
Potra2n18c32026.1	4.392657839	1.7492118	6.16672E-23	0.557434221	Potri.018G148300.1	AT1G16120.1
Potra2n18c32027.1	1.664677056	1.381953902	NA	NA	Potri.019G096800.1	AT5G16250.1
Potra2n18c32028.1	-0.595952689	0.487799375	NA	NA	Potri.018G148400.1	AT1G69730.1
Potra2n18c32029.1	2.634099619	2.452196398	0.302448324	NA	Potri.T126600.1	AT1G19390.1
Potra2n18c32030.1	2.492428534	NA	NA	NA	Potri.T126300.1	AT1G04645.1
Potra2n18c32031.1	2.492428534	NA	NA	NA	Potri.001G207400.1	
Potra2n18c32032.1	0.82357019	NA	NA	NA	Potri.018G148700.1	AT1G04645.1
Potra2n18c32033.1	NA	NA	NA	NA	Potri.002G142700.1	AT3G20050.1
Potra2n18c32034.1					Potri.017G108200.1	AT5G03340.1

Potra2n18c32035.1					Potri.017G108200.1	AT5G03340.1
Potra2n18c32036.1	-1.807373693	-2.072423285	0.38961479	NA	Potri.006G123900.1	AT2G36910.1
Potra2n18c32037.1	3.528956139	1.019870759	5.21839E-12	0.79078676		
Potra2n18c32038.1	3.528956139	1.019870759	5.21839E-12	0.79078676	Potri.018G148300.1	AT1G16120.1
Potra2n18c32039.1	5.182980072	NA	0.017918255	NA	Potri.001G064900.1	AT1G58190.2
Potra2n18c32040.1	NA	NA	NA	NA	Potri.004G183300.1	AT5G60620.1
Potra2n18c32041.1					Potri.004G183300.1	AT5G60620.1
Potra2n18c32042.1	0.82357019	NA	NA	NA		
Potra2n18c32043.1	NA	NA	NA	NA	Potri.T161600.1	AT2G19080.1
Potra2n18c32044.1	4.44234491	2.390071623	0.02824841	NA	Potri.T161600.1	AT2G19080.1
Potra2n18c32045.1	0.796082317	0.474543647	0.029998266	0.833651746	Potri.012G108000.1	AT2G34400.1
Potra2n18c32046.1	8.156027702	4.79823279	5.79277E-23	0.066791025	Potri.003G041700.2	AT1G58190.2
Potra2n18c32047.1	0.074740787	0.011209673	0.66657096	0.993351655	Potri.018G143900.2	AT2G19160.1
Potra2n18c32048.2	-0.379369262	0.055653059	0.0277764	0.961820855		
Potra2n18c32049.1	1.888696032	0.728818068	0.000125581	0.814981587	Potri.018G143700.1	AT1G06980.1
Potra2n18c32050.3	-0.312659361	-0.173639522	0.017950824	0.888096054	Potri.018G143600.1	AT4G25850.1
Potra2n18c32051.1	-0.665376192	-0.226427422	0.00093937	0.890369631	Potri.018G143500.1	AT5G57230.1
Potra2n18c32052.1	-1.477483045	-0.479009643	1.50857E-09	0.579761554	Potri.018G143400.2	AT4G30020.1
Potra2n18c32053.2	-0.809881041	0.442079655	0.229272717	0.864019364	Potri.018G143300.1	AT3G29230.1
Potra2n18c32054.1	-3.45625203	-0.09355649	0.054563633	0.989304496	Potri.018G143300.1	AT3G29230.1
Potra2n18c32055.1	0.137997216	-0.051457356	0.148085581	0.926234768	Potri.018G143100.1	AT4G29950.1
Potra2n18c32056.1	-0.036508908	-0.149190796	0.809459732	0.803684688	Potri.018G143000.1	AT4G29960.1
Potra2n18c32057.1	-0.712613291	0.032942331	1.08316E-06	0.986671181	Potri.018G142800.1	AT5G57200.1
Potra2n18c32058.4	-1.649346048	-0.494677736	2.25428E-12	0.501154172	Potri.018G142700.2	AT1G22430.2
Potra2n18c32059.1	-0.12579667	-0.127206278	0.381577267	0.863768668	Potri.018G142500.1	AT4G30010.1
Potra2n18c32060.1	-0.416619431	-0.027048365	0.000132322	0.978066517	Potri.018G142400.1	AT4G29940.1
Potra2n18c32061.1	0.012213729	0.070069074	0.954034675	0.934439727	Potri.018G142300.1	AT2G19260.1
Potra2n18c32062.2	-0.88698122	-0.43741959	0.004098278	0.540010302	Potri.018G142100.1	AT5G57180.2
Potra2n18c32063.1	-0.298202353	-0.160089124	0.049400514	0.803444125	Potri.018G141900.1	AT5G57160.1
Potra2n18c32064.1	1.238867998	-1.620826217	0.447687639	NA	Potri.006G075000.1	AT2G19270.1
Potra2n18c32065.1	0.156926221	0.196615155	0.472915556	0.857189843	Potri.018G141800.1	AT5G57150.1
Potra2n18c32066.1	NA	NA	NA	NA		

Potra2n18c32067.1	2.618387201	0.821527142	7.07869E-06	0.661329087	Potri.018G141700.3	AT5G57150.1
Potra2n18c32068.1	0.027062693	0.217205242	0.929223797	0.937687311	Potri.018G141600.1	AT5G57150.1
Potra2n18c32069.1	-1.328951947	-1.478536539	NA	NA	Potri.018G141400.2	AT3G28345.1
Potra2n18c32070.1	-0.820258361	-0.179869773	0.091101778	0.956865545	Potri.018G141200.1	AT4G26020.1
Potra2n18c32071.1	1.433844468	0.309592406	3.72642E-20	0.779077802	Potri.018G141100.2	AT1G51590.1
Potra2n18c32072.1	-0.175990012	-0.201692717	0.563688429	0.855587395	Potri.018G141000.1	AT3G23750.1
Potra2n18c32073.1	-0.315048278	-0.495186046	0.625014584	0.805461106	Potri.018G140900.1	AT5G57130.1
Potra2n18c32074.1	-0.109215225	0.039534844	0.522872091	0.973347974	Potri.018G140800.1	AT3G28430.1
Potra2n18c32075.1	-0.136087787	-0.103840567	0.90746733	0.987801491	Potri.018G140700.2	AT3G19184.1
Potra2n18c32076.1	-0.816520892	0.127733191	0.000412044	0.939653927	Potri.018G140600.1	AT1G07400.1
Potra2n18c32077.1	-0.242382249	-0.350435692	0.700649348	0.91233528	Potri.018G140400.1	AT2G22590.1
Potra2n18c32078.1	0.847985217	0.203439069	2.73444E-06	0.873955419	Potri.006G073200.2	AT2G44120.2
Potra2n18c32079.1	0.117613355	-0.088712094	0.648257817	0.955322605	Potri.018G140200.1	AT5G57120.1
Potra2n18c32080.1	0.283248331	-0.001575819	0.129198455	0.999435636	Potri.T145700.1	AT5G62050.1
Potra2n18c32081.2	-0.890929236	0.068069318	0.000101695	0.966917036	Potri.006G072900.1	AT4G29900.1
Potra2n18c32082.1	-2.76942741	-0.957791833	0.000720217	0.799049555	Potri.018G139700.1	AT2G19330.1
Potra2n18c32083.1	-0.001018724	-0.295095759	0.996827447	0.72680202	Potri.018G139600.1	AT4G29870.1
Potra2n18c32084.2	-0.385730786	-0.086850816	0.059877492	0.953831244	Potri.018G139500.1	AT5G57100.1
Potra2n18c32085.1	NA	0.674963689	NA	NA	Potri.018G139400.1	AT5G57090.1
Potra2n18c32086.1	-0.502260244	-0.152992581	0.026839182	0.911339141	Potri.018G139300.1	AT4G31550.1
Potra2n18c32087.1	-1.525592111	-0.560084859	2.88204E-07	0.474295608	Potri.018G139200.1	
Potra2n18c32088.1	0.123170226	0.114892465	0.590493387	0.937146435	Potri.018G139000.1	AT2G17220.1
Potra2n18c32089.1	-0.036898423	-0.415521978	0.93427325	0.811742964	Potri.018G138700.1	AT2G17220.1
Potra2n18c32090.1	1.016544026	0.396780218	3.31468E-07	0.517110447	Potri.018G138500.2	AT1G50180.1
Potra2n18c32091.1	0.013829119	0.384249669	0.995109635	NA	Potri.018G135300.2	AT2G17220.1
Potra2n18c32092.2	1.743929287	0.786537727	2.24862E-09	0.389056998	Potri.018G134200.1	AT2G17220.1
Potra2n18c32093.1	0.61087997	0.340754119	0.000107527	0.641037344	Potri.018G134100.1	AT2G17220.2
Potra2n18c32094.1	0.53281011	0.343403882	0.000546971	0.585528204	Potri.018G134100.1	AT2G17220.2
Potra2n18c32095.1	2.492428534	0.674963689	NA	NA	Potri.018G134000.1	AT5G36110.1
Potra2n18c32096.1	0.129145686	-1.502484666	0.959312932	NA	Potri.T167900.2	AT5G36110.1
Potra2n18c32097.1	0.105273227	0.177970696	0.626702005	0.855587395	Potri.018G133700.1	AT3G55600.1
Potra2n18c32098.1					Potri.018G003100.1	AT4G16130.1

Potra2n18c32099.1	-0.622154575	-0.183772211	0.000227662	0.887496314	Potri.018G133500.1	AT4G29850.1
Potra2n18c32100.1	0.157412543	-0.112993479	0.354089247	0.918871056	Potri.018G133400.1	AT5G20500.1
Potra2n18c32101.1	3.975855975	1.381953902	0.1424982	NA		
Potra2n18c32102.1	0.031959287	-0.06363755	0.921112581	0.978917914	Potri.018G133300.1	
Potra2n18c32103.1	-1.35927387	-1.507841271	NA	NA	Potri.018G133200.1	AT5G38330.1
Potra2n18c32104.1	-0.214981255	-1.635229989	0.76352024	0.514425946	Potri.018G133100.1	AT5G06710.1
Potra2n18c32105.1	-0.079414337	-0.049192861	0.732273972	0.976302215	Potri.018G133000.1	AT5G20590.1
Potra2n18c32106.1	NA	NA	NA	NA	Potri.018G132950.1	
Potra2n18c32107.1	NA	NA	NA	NA	Potri.006G071400.1	
Potra2n18c32108.1					Potri.018G132900.1	AT1G56140.1
Potra2n18c32109.1	2.657266741	0.817428494	0.182120156	NA	Potri.018G132800.1	AT5G07610.1
Potra2n18c32110.1	-0.290060193	-0.059229074	0.010203302	0.937846405	Potri.018G132700.1	AT2G38670.1
Potra2n18c32111.1	-0.477840718	-0.124878844	0.007486853	0.903477395	Potri.018G132600.1	AT5G35320.1
Potra2n18c32112.3	-1.350289329	-0.418651868	1.29468E-07	0.728418847	Potri.018G132500.1	AT2G39930.1
Potra2n18c32113.1	0.136490028	0.047177383	0.780907958	0.9869326	Potri.006G070600.2	AT5G10990.1
Potra2n18c32114.1	-3.116646694	-0.562536829	0.001971123	0.898853929		
Potra2n18c32115.1	-1.660951924	0.031125469	6.31938E-05	0.988132773	Potri.018G132200.1	AT4G10310.1
Potra2n18c32116.1	0.798201739	0.163290647	0.00119056	0.901671593	Potri.018G132100.1	AT2G06925.1
Potra2n18c32117.1	0.746873732	0.358536461	7.02029E-11	0.322673812	Potri.018G132000.1	AT1G67940.1
Potra2n18c32118.1	3.479678994	1.708702784	9.86466E-05	0.586851629	Potri.018G131900.1	
Potra2n18c32119.1	-0.564846753	-0.214226855	1.94036E-05	0.865538396	Potri.018G131700.1	AT2G26670.1
Potra2n18c32120.1	-1.089395434	-2.203867199	0.523584623	NA	Potri.018G131600.1	AT2G41480.1
Potra2n18c32121.1	-1.830269323	0.227253654	1.40949E-26	0.924256651	Potri.018G131500.2	AT2G26660.1
Potra2n18c32122.1	NA	NA	NA	NA	Potri.018G131400.1	AT1G15360.1
Potra2n18c32123.1	-0.344215252	-0.192737575	0.059682158	0.860325259	Potri.018G131300.1	AT4G13730.1
Potra2n18c32124.1	0.437388015	-0.921631736	0.496142482	0.742685434	Potri.018G131200.1	AT1G52570.1
Potra2n18c32125.1	2.031729974	0.117532094	6.8575E-11	0.973347974	Potri.018G131100.1	AT3G15760.1
Potra2n18c32126.2	0.730541371	0.022996857	1.15513E-05	0.988354153	Potri.018G131000.1	AT5G20180.2
Potra2n18c32127.1	0.0082192	0.394687406	0.973253203	0.385550192	Potri.018G130900.1	AT5G25360.1
Potra2n18c32128.1	0.843004428	0.067768818	0.346390129	0.97950444	Potri.018G130800.1	AT2G25490.1
Potra2n18c32129.1	0.606004699	-0.044820351	0.436374334	0.986636967		
Potra2n18c32130.1	-3.334446166	-0.35328723	0.00095578	0.907271475	Potri.018G130700.1	AT1G80133.1

Potra2n18c32131.1	-0.446413409	-0.197436841	0.00052473	0.788065047	Potri.018G130500.6	AT5G35410.1
Potra2n18c32132.1	-3.671716513	-0.868039983	4.04821E-08	0.794820393	Potri.018G130300.1	AT1G80130.1
Potra2n18c32133.1	-6.550613112	-1.642102975	2.58459E-47	0.044004179	Potri.018G130000.1	AT1G02630.1
Potra2n18c32134.1	-2.238788732	-0.802862973	1.76772E-50	0.011701247	Potri.018G130100.1	AT1G80130.1
Potra2n18c32135.1	-2.443126322	-0.905726473	4.2183E-57	6.30416E-08	Potri.018G130000.1	AT1G02630.1
Potra2n18c32136.1	-2.195055365	-0.690348051	9.11444E-35	0.017908786		
Potra2n18c32137.1	-0.254079525	0.133926155	0.009662642	0.917516356	Potri.018G129900.2	AT5G20200.1
Potra2n18c32138.1	-1.317292482	-0.024939653	NA	NA		
Potra2n18c32139.2	1.911189818	-0.767708831	NA	NA	Potri.018G129800.1	AT2G26580.1
Potra2n18c32140.1	-0.233262634	0.102071381	0.072094163	0.93263929	Potri.018G129700.1	AT3G15790.1
Potra2n18c32141.1	-0.234022059	0.101702551	0.070961473	0.932984974	Potri.018G129600.1	AT4G02530.1
Potra2n18c32142.1	-0.354103361	-0.191359067	0.041341877	0.900015282	Potri.018G129500.2	AT2G26600.1
Potra2n18c32143.1	6.591086215	NA	5.85624E-07	NA	Potri.018G129400.1	AT5G20230.1
Potra2n18c32144.1	NA	1.571529889	NA	NA	Potri.018G129300.1	
Potra2n18c32145.1	2.795211372	1.280008676	1.29096E-22	0.002915668	Potri.006G067300.2	AT2G31050.1
Potra2n18c32146.1	1.864385786	-0.079512623	0.692960759	NA		
Potra2n18c32147.1	1.664677056	NA	NA	NA	Potri.018G129000.1	AT2G32300.1
Potra2n18c32148.1					Potri.018G129000.1	AT2G32300.1
Potra2n18c32149.1	-0.558188419	-0.190574256	0.072811211	0.92223649	Potri.006G067100.1	AT3G24150.1
Potra2n18c32150.1	4.514740228	2.0390154	7.81413E-14	0.31298304	Potri.018G128600.1	AT3G15810.1
Potra2n18c32151.1	0.621566523	-0.104497605	0.000185804	0.941855873	Potri.018G128400.1	AT3G33530.1
Potra2n18c32152.1	-0.099489714	0.014226235	0.467256544	0.9869326	Potri.018G128300.1	AT3G33520.1
Potra2n18c32153.1	0.387499598	0.090786761	0.008532793	0.919424623		
Potra2n18c32154.1	-0.011614073	-0.109351525	0.94936831	0.890446513	Potri.018G128100.1	AT2G42160.1
Potra2n18c32155.1	0.466591793	0.301956447	0.004750475	0.736000077	Potri.018G128000.1	AT3G15880.1
Potra2n18c32156.1	-0.410316732	-0.562243598	0.296727142	0.642417298		
Potra2n18c32157.1	0.274611125	-0.381977294	0.558867257	0.86212354	Potri.018G127800.1	AT1G04550.2
Potra2n18c32158.1	0.625145841	-0.30447432	0.276252481	0.931501341	Potri.018G127700.1	AT5G52600.1
Potra2n18c32159.2	-0.413364556	-0.079357	0.018264992	0.944116026	Potri.018G127500.1	AT5G52580.1
Potra2n18c32160.1	-0.450325546	-0.114595626	0.055550441	0.925184207		
Potra2n18c32161.1	0.049577091	0.669148082	0.975101485	NA		
Potra2n18c32162.1	0.037101549	0.656767195	0.980788309	0.886633917	Potri.018G127600.1	

Potra2n18c32163.1	-1.093939448	0.161487476	0.007209588	0.957064107	Potri.018G127400.1	AT4G32272.1
Potra2n18c32164.1	-0.889166957	0.20763816	4.38936E-06	0.895474078	Potri.018G127300.1	AT3G01300.1
Potra2n18c32165.1	-0.248514271	-0.118952373	0.000484216	0.834823243	Potri.018G127200.1	AT5G32440.3
Potra2n18c32166.1	1.017644929	-0.257296854	1.67674E-05	0.824905557	Potri.018G127100.1	AT2G43290.1
Potra2n18c32167.2	-0.972013866	-0.630339893	0.005421059	0.518528577	Potri.018G127000.2	
Potra2n18c32168.1	-0.072732221	0.025306724	0.662510526	0.983446961	Potri.012G059200.1	AT1G18560.1
Potra2n18c32169.1	-0.282467279	-0.12639664	0.733618637	0.982422288	Potri.012G059200.1	AT1G18560.1
Potra2n18c32170.1	-1.441234112	-0.843536399	2.19747E-05	0.498594799	Potri.018G126400.1	AT5G20250.4
Potra2n18c32171.3	-0.278186723	-0.140125742	0.016072246	0.833401866	Potri.018G126300.1	
Potra2n18c32172.1	-0.030825172	-0.070467484	0.873574213	0.943685779	Potri.018G126100.1	AT5G35430.1
Potra2n18c32173.1	2.099878214	-2.11085248	0.17460429	NA	Potri.018G126000.1	AT3G22142.1
Potra2n18c32174.1					Potri.018G125900.1	AT5G11150.1
Potra2n18c32175.2	-0.04811459	0.281112739	0.898477371	0.885375133	Potri.018G125500.2	AT2G25640.1
Potra2n18c32176.1	-0.457520685	-0.134274429	0.014535784	0.920141992	Potri.018G125400.1	AT2G10950.1
Potra2n18c32177.1	-0.246119227	-0.129378631	0.091789844	0.936509845	Potri.006G065100.1	AT5G35200.1
Potra2n18c32178.1	NA	NA	NA	NA		
Potra2n18c32179.1	0.49731474	0.078194482	0.001684819	0.936890993	Potri.006G065000.1	AT2G11000.1
Potra2n18c32180.1	-0.23343395	-0.062381536	0.09194299	0.977559521	Potri.018G125000.1	AT5G20260.1
Potra2n18c32181.1	-1.069966055	-0.29811066	4.1693E-05	0.752794139	Potri.018G124900.1	AT5G20260.1
Potra2n18c32182.1	-1.258393822	0.154902219	0.011108206	0.900015282	Potri.018G124832.1	AT3G42180.1
Potra2n18c32183.1	-0.596187428	0.045519744	0.00014958	0.974367223	Potri.018G124700.4	AT5G20280.1
Potra2n18c32184.2	0.734387007	0.317159695	0.000201438	0.29966352	Potri.018G124600.4	AT2G11520.1
Potra2n18c32185.1	6.271531554	3.081349306	3.82581E-07	NA		
Potra2n18c32186.1	5.85725565	3.022582998	1.29211E-46	0.028538645	Potri.T114300.1	AT1G54470.2
Potra2n18c32187.1	-0.349170431	0.14500808	0.01438619	0.887678043	Potri.006G078800.1	AT2G26590.2
Potra2n18c32188.1	2.125760842	-0.003010608	5.55091E-13	0.999325699	Potri.018G124100.1	AT5G33280.1
Potra2n18c32189.1	-0.308448163	-0.166519044	0.015196276	0.841527401	Potri.018G124000.2	AT1G05500.1
Potra2n18c32190.1					Potri.018G123900.1	AT5G10970.1
Potra2n18c32191.1	1.397073411	-1.721131216	0.417037975	NA	Potri.018G123800.1	AT5G25160.1
Potra2n18c32192.4	0.51106912	0.247065429	0.018045498	0.873823526	Potri.018G123700.1	AT5G34850.1
Potra2n18c32193.1	0.676155861	0.520574052	0.06208032	0.720242695		
Potra2n18c32194.1	0.105676983	0.10683863	0.724774409	0.966005919	Potri.018G123600.1	

Potra2n18c32195.1	0.687927148	0.309881978	2.4516E-05	0.682454638	Potri.018G123400.1	
Potra2n18c32196.1	0.046610992	0.009062201	0.794943736	0.995579852	Potri.018G123300.1	AT5G20300.1
Potra2n18c32197.1	-0.043199438	-0.062602425	0.802617534	0.94795983	Potri.018G123200.1	AT5G10790.1
Potra2n18c32198.1	-0.453901821	-0.166575052	0.009289878	0.94494655	Potri.018G123100.1	AT1G60430.2
Potra2n18c32199.1	-0.522124946	-0.200835153	0.017400129	0.893771277	Potri.018G123000.1	AT5G20410.1
Potra2n18c32200.1	0.118398065	0.087461006	0.387797836	0.923397137		
Potra2n18c32201.1	0.299473067	0.134187927	0.135106484	0.88643982	Potri.018G119600.1	AT4G39140.2
Potra2n18c32202.1	-0.10535325	0.639282788	0.713047142	0.677013336	Potri.018G119500.1	AT1G15690.1
Potra2n18c32203.1	-2.594669203	-0.397016562	0.333620572	NA		
Potra2n18c32204.1	0.479149226	0.406410015	5.1515E-05	0.459449783	Potri.018G119200.1	AT1G30270.1
Potra2n18c32205.1	8.899344176	5.067267536	4.74732E-14	0.233903684	Potri.T171300.1	AT5G15500.2
Potra2n18c32206.1	2.06007813	-0.05020672	0.553192663	NA	Potri.018G119000.1	
Potra2n18c32207.1	2.735991243	2.676364087	NA	NA	Potri.001G155750.1	AT1G21280.1
Potra2n18c32208.1						
Potra2n18c32209.1	3.346696182	1.602805887	0.164285163	NA	Potri.018G118900.1	
Potra2n18c32210.1	0.419879454	0.086401126	0.050102325	0.943819961	Potri.018G121900.1	AT4G20060.1
Potra2n18c32211.1	2.897568598	0.443917575	2.59451E-13	0.874507876	Potri.018G121800.1	AT5G54000.1
Potra2n18c32212.2	0.237750289	0.061656325	0.223809144	0.953831244	Potri.018G121600.2	AT5G20380.1
Potra2n18c32213.1	0.640640409	-0.218866876	0.094159283	0.8954569	Potri.018G121500.1	AT5G34940.2
Potra2n18c32214.1	0.188212629	-0.184920346	0.369167904	0.774820633		
Potra2n18c32215.3	-0.078575573	-0.069258954	0.635428459	0.935474933	Potri.018G121200.1	AT5G20350.1
Potra2n18c32216.1	0.002236053	-0.019444153	0.994577291	0.993054709	Potri.018G121100.1	AT2G25290.1
Potra2n18c32217.1	-0.199124173	0.257585465	0.197541018	0.769643258	Potri.018G118200.1	AT5G35080.1
Potra2n18c32218.1	0.482057766	0.157610368	4.23927E-11	0.829781359	Potri.T109100.1	AT2G12550.1
Potra2n18c32219.1	-0.004535721	0.004644362	0.987039542	0.998245864		
Potra2n18c32220.1	-0.595952689	-1.465929376	NA	NA	Potri.T109201.1	AT4G32060.1
Potra2n18c32221.1	NA	NA	NA	NA	Potri.T109600.1	AT4G32060.1
Potra2n18c32222.1	5.791412029	3.728893138	1.0636E-24	0.048463789	Potri.018G117600.1	AT1G07390.1
Potra2n18c32223.1	3.00203964	1.156279831	1.74528E-06	0.713032256	Potri.T109300.2	AT1G07390.3
Potra2n18c32224.1	0.82357019	2.307412514	NA	NA	Potri.018G120800.2	AT1G74190.1
Potra2n18c32225.1	3.06039544	1.925747501	3.44797E-05	0.498467227	Potri.018G117800.1	AT1G58190.2
Potra2n18c32226.4	0.385402266	0.209802544	0.004376675	0.814001428	Potri.018G116500.1	AT5G14600.1

Potra2n18c32227.1	-0.658589376	-0.101512025	0.001705617	0.953608209	Potri.018G116300.1	AT5G11090.1
Potra2n18c32228.1	-1.648312204	0.57104843	0.05977474	0.900551258	Potri.018G116200.2	AT5G11090.1
Potra2n18c32229.1	-0.520019199	-0.108853212	0.033087943	0.938967511	Potri.018G116100.1	AT2G12400.1
Potra2n18c32230.2	0.675193533	0.008579214	0.290192312	0.999325699	Potri.018G116000.1	AT2G12646.1
Potra2n18c32231.3	-1.386840714	-0.48310546	1.70395E-05	0.694769714	Potri.018G120000.1	AT2G41040.1
Potra2n18c32232.1	1.87191241	-1.029070229	0.367807934	NA	Potri.018G115601.1	
Potra2n18c32233.1	-4.567710683	-2.337802268	0.067754062	NA	Potri.006G059900.1	
Potra2n18c32234.2	0.027932198	0.158117902	0.824805896	0.828860751	Potri.018G115500.1	AT5G25270.1
Potra2n18c32235.1	0.391658128	-0.14421391	0.094855316	0.941260209	Potri.018G115400.1	AT5G23740.1
Potra2n18c32236.1	-0.843721649	-0.383880585	0.035737982	0.8693465		
Potra2n18c32237.2	0.24930033	-0.077508179	0.416153885	0.977227089	Potri.018G115100.5	AT2G25620.1
Potra2n18c32238.1	-0.311237804	-0.140537851	0.074807719	0.925184207	Potri.018G115000.1	AT2G13100.1
Potra2n18c32239.3	0.075832547	-0.104229773	0.602322155	0.906107926	Potri.018G114900.1	AT1G06220.1
Potra2n18c32240.1	2.978628956	0.464524225	5.20153E-17	0.917746944		
Potra2n18c32241.1	-0.522185515	-0.222374195	0.00021655	0.821348554	Potri.018G114700.10	AT5G45290.1
Potra2n18c32242.1	-0.377984568	-0.417554128	0.031826	0.49456927	Potri.018G114600.1	
Potra2n18c32243.1	1.687429564	0.422038166	2.41708E-09	0.74706342	Potri.018G114500.1	AT2G15580.1
Potra2n18c32244.1	0.358002511	0.16515196	0.009281012	0.860810425	Potri.018G114300.2	AT3G46920.1
Potra2n18c32245.1	0.999807541	-0.01406125	0.000583015	0.995268765	Potri.018G114200.1	AT5G62865.1
Potra2n18c32246.3	0.606787321	0.488331133	4.33596E-05	0.493313095	Potri.018G114100.1	AT5G25220.2
Potra2n18c32247.1	0.215988501	0.047279457	0.94137039	NA	Potri.018G113900.1	AT5G35110.1
Potra2n18c32248.1	NA	1.363811008	NA	NA		
Potra2n18c32249.1	2.271362449	0.32009853	0.001011913	0.958152146	Potri.018G113700.1	AT5G35110.1
Potra2n18c32250.1	-0.198601915	0.091194949	0.081289883	0.920763566	Potri.018G113600.1	AT3G09850.1
Potra2n18c32251.1	0.249155156	0.021998804	0.111326736	0.987226805	Potri.018G113500.1	AT5G35160.2
Potra2n18c32252.1	-1.017328678	-0.160802589	1.58412E-06	0.885628484	Potri.018G113400.1	AT5G35170.1
Potra2n18c32253.1	3.716146767	3.309656281	0.350807433	NA	Potri.018G113300.1	AT4G02050.1
Potra2n18c32254.1	0.560758051	0.015701349	0.002373597	0.994403137	Potri.018G113200.1	AT3G18760.1
Potra2n18c32255.1	-0.067027409	-0.071068041	0.70857821	0.931774514	Potri.018G113100.3	AT5G35180.2
Potra2n18c32256.1	-0.130199295	-0.2149925	0.527940029	0.79477084		
Potra2n18c32257.1	-0.844616633	0.081117324	0.017799387	0.957525726	Potri.018G113000.1	AT5G51350.1
Potra2n18c32258.1	-0.757555322	0.132671076	0.021591178	0.921099436	Potri.018G113000.1	AT5G51350.1

Potra2n18c32259.1	-0.406450134	-0.192654306	0.330323017	0.871075958	Potri.018G112900.1	
Potra2n18c32260.1	1.257881015	-0.291321053	0.002705546	0.935851032	Potri.018G112800.1	AT2G07690.1
Potra2n18c32261.1	-0.567683559	-0.131664456	0.773127715	NA		
Potra2n18c32262.1	0.851156957	-0.017187281	0.05151946	0.996032145	Potri.018G112600.1	AT2G22650.1
Potra2n18c32263.2	1.135778397	0.218454809	0.007543677	0.94728769	Potri.018G112500.1	AT1G80710.1
Potra2n18c32264.1	1.082807379	5.775001757	0.625608307	0.153859944	Potri.018G112400.1	AT3G42640.1
Potra2n18c32265.1	0.431245482	0.035594136	0.004362756	0.98177802	Potri.006G188500.1	AT4G31985.1
Potra2n18c32266.1	0.157846941	-0.098131449	0.567132545	0.948834049	Potri.018G112200.1	AT1G15860.1
Potra2n18c32267.1	-0.581650466	-0.066823883	0.099283896	0.975578749	Potri.018G112000.1	AT4G19810.1
Potra2n18c32268.1	2.246976267	1.613908599	0.000188303	0.437507219	Potri.018G149300.1	AT5G36110.1
Potra2n18c32269.1	0.254618018	0.029029025	0.479561446	0.989304496	Potri.018G111900.1	AT3G16030.1
Potra2n18c32270.1	3.309027936	0.908476427	2.02908E-19	0.554040943	Potri.018G111800.2	AT4G11530.1
Potra2n18c32271.1	-1.133457233	-0.180685767	0.00010126	0.948577865	Potri.018G111600.1	AT1G11330.2
Potra2n18c32272.1	NA	NA	NA	NA		
Potra2n18c32273.1	0.408339109	0.314937126	0.243572231	0.853281779	Potri.018G111900.1	AT3G16030.1
Potra2n18c32274.1	3.405606367	0.692133997	0.101658985	NA	Potri.018G111800.2	AT4G11530.1
Potra2n18c32275.1					Potri.018G111500.1	AT5G58200.1
Potra2n18c32276.1	NA	NA	NA	NA	Potri.018G111800.2	AT4G11530.1
Potra2n18c32277.1	1.474833013	-0.8309825	0.039517323	0.808901406	Potri.018G111600.1	AT1G11330.2
Potra2n18c32278.1					Potri.018G111500.1	AT5G58200.1
Potra2n18c32279.1	2.164853785	-0.023194019	0.011435249	0.997821888	Potri.018G111400.1	AT4G31020.1
Potra2n18c32280.2	-0.411318462	0.125682005	0.301632226	0.931774514	Potri.018G111300.1	AT2G25180.1
Potra2n18c32281.1	0.561258482	-0.038362608	0.011489638	0.973347974	Potri.018G111200.1	AT2G24300.2
Potra2n18c32282.1	0.09197814	0.155070813	0.598613074	0.864342685	Potri.018G111100.1	AT2G24290.1
Potra2n18c32283.1	-0.927872826	0.630212022	0.001451553	0.721810957	Potri.018G111000.1	AT2G24280.1
Potra2n18c32284.1	-1.345931437	-0.04693621	0.002774456	0.987891912	Potri.018G110900.1	
Potra2n18c32285.1	-0.672854857	-0.337886746	1.23043E-05	0.349324124	Potri.018G110800.1	AT5G58070.1
Potra2n18c32286.1	-0.495380809	-0.190242972	0.000105251	0.804796567	Potri.018G110700.1	AT5G58060.1
Potra2n18c32287.1	-0.401767278	0.297643048	0.33996422	0.903934429	Potri.018G110600.1	AT5G58050.1
Potra2n18c32288.1	-0.413648844	0.296095205	0.321585259	0.904709467		
Potra2n18c32289.1	-0.02834095	0.122035936	0.913936191	0.911339141	Potri.018G110300.1	AT4G30993.2
Potra2n18c32290.1	0.252370396	0.161633262	0.114932226	0.843667065	Potri.018G110200.1	AT1G63270.1

Potra2n18c32291.1	0.375919559	0.237071435	0.000139152	0.696755852	Potri.018G110100.1	AT5G58040.1
Potra2n18c32292.1	-0.031738691	-0.041023119	0.812915338	0.938501048	Potri.018G109900.1	AT2G19070.1
Potra2n18c32293.1	-0.640156933	-0.094791834	0.000122687	0.947290893	Potri.018G109800.1	AT5G58020.1
Potra2n18c32294.2	-0.430497535	-0.945811806	0.141289975	0.137620511	Potri.018G109700.1	AT2G24270.2
Potra2n18c32295.1	0.038928174	0.122618786	0.791442128	0.872570395	Potri.018G109600.10	AT4G31880.1
Potra2n18c32296.2	0.625963675	-0.07035876	0.004399914	0.933408221	Potri.018G109500.1	AT2G24260.1
Potra2n18c32297.1	-0.012093957	-0.23527432	0.946479747	0.611349636	Potri.006G186500.1	AT5G58005.2
Potra2n18c32298.4	-0.667433379	-0.030611323	0.000329211	0.989329144	Potri.018G109201.1	AT1G51120.1
Potra2n18c32299.1	-1.33263295	-1.788204938	0.312707525	0.720242695		
Potra2n18c32300.1	-0.668102657	-0.255144946	1.8494E-05	0.684244573	Potri.018G109100.2	AT4G30960.1
Potra2n18c32301.1	0.081756859	-0.763669453	NA	NA		
Potra2n18c32302.2	-0.255405633	0.101112132	0.177776766	0.943819961	Potri.018G108300.1	AT5G58003.1
Potra2n18c32303.2	-0.668311341	-0.24151344	0.000724087	0.812159187	Potri.018G108700.1	AT5G58000.1
Potra2n18c32304.1	-0.278868925	0.139962421	0.11136287	0.923766642	Potri.018G108000.2	AT5G58160.1
Potra2n18c32305.1	-1.169756475	-0.454728495	2.80976E-08	0.183934002	Potri.018G107800.1	AT5G46220.1
Potra2n18c32306.1	0.82357019	NA	NA	NA	Potri.018G107700.1	AT4G30950.1
Potra2n18c32307.1	1.459962178	2.141662216	NA	NA	Potri.018G107700.1	AT4G30950.1
Potra2n18c32308.1	NA	NA	NA	NA		
Potra2n18c32309.1	-0.555125381	-0.249320584	0.000963123	0.885900567	Potri.018G107600.1	AT2G24240.1
Potra2n18c32310.1	0.880890493	0.406626841	9.24777E-05	0.273841527	Potri.018G107500.1	AT5G57990.1
Potra2n18c32311.1	-0.370658496	-0.037506364	0.166211366	0.98534079	Potri.018G107400.1	AT2G24230.1
Potra2n18c32312.2	0.338797706	0.065522324	0.019647823	0.947969749	Potri.018G107300.1	AT1G13950.1
Potra2n18c32313.2	0.010639769	0.145036123	0.946958233	0.885233199	Potri.018G107000.5	AT4G30935.1
Potra2n18c32314.2	-0.468252275	-0.270351905	0.001188744	0.793944945	Potri.018G106900.3	AT5G57970.2
Potra2n18c32315.1	0.185661811	0.106607076	0.067415707	0.845627381	Potri.018G106800.2	AT4G30920.1
Potra2n18c32316.1	0.850852692	0.133761564	3.37744E-05	0.885375133	Potri.018G106700.1	AT4G30900.1
Potra2n18c32317.1	-0.22071913	-0.247759639	0.185900294	0.69862311	Potri.018G106600.1	AT5G10840.1
Potra2n18c32318.1	-0.61910233	-0.767708831	NA	NA	Potri.018G106500.1	AT2G23945.1
Potra2n18c32319.1	-0.227786662	0.022615738	0.233909533	0.986636967	Potri.018G106400.1	AT5G57930.2
Potra2n18c32320.1	0.119673858	0.088725513	0.269398481	0.885375133	Potri.018G106100.1	AT5G57940.2
Potra2n18c32321.1	0.943350674	-0.767708831	NA	NA	Potri.018G106000.1	AT3G43660.1
Potra2n18c32322.1	-0.214188962	0.019250918	0.038440357	0.985393296	Potri.018G105900.2	AT4G30600.1

Potra2n18c32323.1	2.335270396	0.752942177	2.6175E-09	0.812696706	Potri.018G105700.1	AT4G30610.1
Potra2n18c32324.1	-0.019433226	-0.056069847	0.927219167	0.957989526	Potri.018G105600.2	AT2G24020.1
Potra2n18c32325.1	-2.095956	-0.865807542	0.437834172	NA	Potri.018G105500.1	AT5G48930.1
Potra2n18c32326.1	-0.670765696	-0.193774399	1.27951E-05	0.813731863	Potri.006G183000.3	AT4G30630.1
Potra2n18c32327.1	0.396634919	0.146297533	0.00057827	0.871920317	Potri.018G105100.1	AT4G30660.2
Potra2n18c32328.3	0.398395054	0.148700263	0.000503949	0.868917644		
Potra2n18c32329.1	0.418528189	0.023699375	0.024596691	0.985313207	Potri.018G104900.1	AT5G57900.1
Potra2n18c32330.1	0.012264423	-0.174797957	0.981113169	0.84253168	Potri.018G104700.1	AT5G48930.1
Potra2n18c32331.1	-1.1129559	-0.961766075	0.032552681	0.395492956	Potri.018G104500.1	
Potra2n18c32332.2	-0.657093825	-0.339291646	0.019777203	0.816567218	Potri.018G104400.1	
Potra2n18c32333.1	0.080965301	-0.002166926	0.562735467	0.998785804	Potri.018G104100.1	AT5G57870.2
Potra2n18c32334.1	0.139471186	-0.41158746	0.777506388	0.862335622	Potri.018G104200.1	
Potra2n18c32335.1	-0.042990289	-0.051414238	0.819270805	0.953608209		
Potra2n18c32336.2	-0.577344133	-0.10617484	0.008237328	0.959881296	Potri.006G181900.1	AT5G17420.1
Potra2n18c32337.1	-0.562687002	-0.107795807	0.029173465	0.965215635		
Potra2n18c32338.1	-0.644396023	-0.312914446	0.000111895	0.788065047	Potri.018G103800.1	AT4G30710.1
Potra2n18c32339.1	-0.223323088	-0.220099113	0.150699419	0.687729258	Potri.018G103700.1	AT4G30720.1
Potra2n18c32340.1	-0.202524659	-0.257847254	0.380674808	0.753881059	Potri.018G103600.1	AT3G18430.1
Potra2n18c32341.1	-0.551481205	-0.038088764	0.0027896	0.9869326	Potri.018G103500.1	AT5G57830.1
Potra2n18c32342.1	-1.225104026	-0.603694156	0.137578533	0.88643982		
Potra2n18c32343.1	0.82357019	NA	NA	NA	Potri.018G103400.2	AT2G24130.1
Potra2n18c32344.1	-0.602828223	-0.162613467	1.60557E-05	0.902847514	Potri.018G103300.1	AT2G24120.1
Potra2n18c32345.1	-0.21927326	0.047549041	0.120301064	0.964160823	Potri.018G103200.2	AT4G30820.3
Potra2n18c32346.1	0.444044341	0.10321065	0.004665392	0.943819961	Potri.018G103100.1	AT4G30810.1
Potra2n18c32347.1	0.791110968	0.341752926	1.29874E-05	0.371384156	Potri.018G103000.1	AT4G30790.1
Potra2n18c32348.1					Potri.018G102900.1	AT3G26390.1
Potra2n18c32349.1	-0.079803674	-0.171190296	0.670819731	0.834823243	Potri.018G102800.1	AT2G24100.1
Potra2n18c32350.1	0.097213431	0.004725004	0.6711795	0.997821888	Potri.018G102700.1	AT2G24090.1
Potra2n18c32351.1	-0.66637769	0.342984837	0.05616217	0.865611082	Potri.018G102500.1	AT4G28080.1
Potra2n18c32352.1	-0.148114204	-0.002236315	0.719888837	0.999458638	Potri.018G102400.1	AT4G30850.2
Potra2n18c32353.2	-0.230585084	0.597435444	NA	NA	Potri.018G102200.1	AT1G16060.1
Potra2n18c32354.1	0.936656265	0.179624198	0.068004858	0.961703915	Potri.018G102100.1	AT4G30860.1

Potra2n18c32355.2	2.05723332	-3.454835369	0.142434166	NA	Potri.018G102000.1	AT2G23970.1
Potra2n18c32356.1	-1.182301504	-0.452247708	0.021306223	0.817452127	Potri.018G101800.1	
Potra2n18c32357.1	0.283137545	0.016234572	0.025427633	0.993589787	Potri.018G101700.1	AT1G17890.1
Potra2n18c32358.1	3.583257039	2.509020267	0.236067343	NA	Potri.018G101600.2	AT2G18660.1
Potra2n18c32359.1	NA	NA	NA	NA		
Potra2n18c32360.3	-0.207338665	-0.27065983	0.710352121	0.93466433	Potri.018G101500.1	AT3G02470.4
Potra2n18c32361.2	-0.076123203	-0.074656793	0.635942651	0.936890993	Potri.018G101200.1	AT4G30510.1
Potra2n18c32362.1	-0.063505462	-0.018351701	0.742220308	0.9896961	Potri.018G101100.2	AT2G23940.1
Potra2n18c32363.1	0.265882127	0.371441765	0.120940568	0.698483145	Potri.018G100900.2	AT4G28070.2
Potra2n18c32364.1					Potri.018G100800.1	
Potra2n18c32365.1	0.063558605	0.1213223	0.624522517	0.882123487	Potri.018G100700.5	AT4G30480.2
Potra2n18c32366.2	0.59457572	0.180101818	0.046856425	0.865591169	Potri.018G100600.4	AT2G23890.1
Potra2n18c32367.1	0.236716357	-0.093033637	0.772939684	0.976302215	Potri.018G100400.1	AT4G30440.1
Potra2n18c32368.1	-0.432709975	-0.068675443	0.18384069	0.98177802	Potri.018G100300.2	AT4G30460.1
Potra2n18c32369.1	-0.622706452	-0.061224464	0.017825498	0.985393296	Potri.018G100166.1	AT4G30460.1
Potra2n18c32370.1	1.64774763	NA	NA	NA	Potri.018G100000.1	AT5G57810.1
Potra2n18c32371.1	-0.226178076	-0.236536397	0.092172077	0.669123229	Potri.018G099900.1	AT1G22450.1
Potra2n18c32372.1	NA	0.674963689	NA	NA		
Potra2n18c32373.1	0.954992437	0.23188119	6.62587E-08	0.759797728	Potri.018G099800.1	AT2G23810.1
Potra2n18c32374.1	-0.76664119	-0.116807509	0.239077226	0.976302215	Potri.018G099700.1	AT3G28960.1
Potra2n18c32375.1	NA	NA	NA	NA	Potri.006G177800.2	AT4G30420.1
Potra2n18c32376.1	NA	NA	NA	NA	Potri.018G099500.1	AT4G30420.1
Potra2n18c32377.1	-0.22818007	-0.719718034	0.810662793	0.855754617	Potri.018G099400.7	AT5G57800.1
Potra2n18c32378.1	-0.751175768	-1.026596888	NA	NA	Potri.018G099300.1	
Potra2n18c32379.1	-2.295337707	-2.446267299	NA	NA	Potri.018G099201.1	
Potra2n18c32380.1	-1.158596097	-0.728348703	0.027862433	0.843179864	Potri.018G099100.1	AT4G30410.2
Potra2n18c32381.1	-0.907864191	-0.268703848	0.003223165	0.841653318	Potri.018G099000.3	AT5G57770.1
Potra2n18c32382.1	-0.384160453	-0.010897359	0.002332773	0.990896655	Potri.018G098700.1	AT5G14850.1
Potra2n18c32383.1	1.459962178	2.676364087	NA	NA	Potri.018G098600.1	AT3G44900.1
Potra2n18c32384.1	-0.430680919	-1.683031766	0.896859996	NA		
Potra2n18c32385.1	-0.99307977	-1.149473951	0.004492934	0.233130274	Potri.018G098500.1	AT4G30400.1
Potra2n18c32386.1	0.244654606	-0.048785738	0.217442411	0.978251387	Potri.018G098400.1	AT5G57740.1

Potra2n18c32387.1					Potri.018G098300.1	AT5G22900.1
Potra2n18c32388.1	8.811413378	3.084831731	2.1422E-09	0.830056077	Potri.018G098200.1	AT4G30380.1
Potra2n18c32389.1	0.425740475	-0.205387819	0.03369842	0.764957294	Potri.008G102800.1	AT3G06580.1
Potra2n18c32390.1	2.656708065	-0.090953061	1.03902E-08	0.985393296	Potri.018G098000.1	AT2G18670.1
Potra2n18c32391.1	-1.132478007	-0.316202075	3.51109E-05	0.804796567	Potri.018G097900.1	AT4G16850.1
Potra2n18c32392.1	2.113573973	1.056981347	8.254E-07	NA	Potri.018G097700.1	AT2G18700.1
Potra2n18c32393.2	0.450006313	0.052811892	5.53243E-05	0.953108412	Potri.018G097600.1	AT4G30360.1
Potra2n18c32394.1	-0.486155153	-0.025100619	0.326892818	0.993577196	Potri.018G097500.1	AT2G18710.1
Potra2n18c32395.1	1.578268123	2.253733581	NA	NA		
Potra2n18c32396.1	-1.336793218	1.051112472	NA	NA	Potri.018G097400.1	
Potra2n18c32397.1	-0.61910233	1.777057475	NA	NA		
Potra2n18c32398.1	-1.35927387	-1.507841271	NA	NA		
Potra2n18c32399.1	-0.400830356	-0.014535446	0.079476965	0.995139072	Potri.018G097300.1	AT5G57710.1
Potra2n18c32400.1						
Potra2n18c32401.1	0.210277448	0.067328261	0.108051526	0.943826727	Potri.018G097100.1	AT5G57700.3
Potra2n18c32402.1	-0.245788967	-0.054294694	0.327117987	0.971828097	Potri.018G097000.1	AT3G46550.1
Potra2n18c32403.1	0.308229105	0.24803516	0.211087047	0.79477084	Potri.018G096900.2	AT4G28020.1
Potra2n18c32404.1	NA	NA	NA	NA		
Potra2n18c32405.1	-0.293048732	-0.034649079	0.009259837	0.98036348	Potri.018G096700.1	AT2G18730.1
Potra2n18c32406.1	0.70413952	-0.822573091	0.444715018	NA	Potri.018G096600.1	AT1G23710.1
Potra2n18c32407.1	1.219766593	-1.309062094	0.597450105	NA		
Potra2n18c32408.1	3.908611111	1.363811008	0.297617706	NA	Potri.018G096500.1	AT2G29110.1
Potra2n18c32409.1	-0.086207665	-0.743519133	0.929831862	0.808901406	Potri.018G096600.1	AT1G23710.1
Potra2n18c32410.1	NA	NA	NA	NA	Potri.018G096300.1	AT2G29120.1
Potra2n18c32411.1	0.040780131	0.147274443	0.797009654	0.886059841	Potri.006G174000.1	AT2G18740.1
Potra2n18c32412.1	-1.505307924	-0.4054023	1.09269E-07	0.639797703	Potri.018G096100.1	AT5G57670.2
Potra2n18c32413.1	1.454221096	1.130086462	1.10525E-09	0.248778911	Potri.T094400.1	AT5G57660.1
Potra2n18c32414.1	0.304212756	-0.012179825	0.04161437	0.992508835	Potri.T094300.1	AT2G24765.1
Potra2n18c32415.1	0.49722621	0.5864537	0.347669476	0.833651746		
Potra2n18c32416.1	0.385634564	-0.018208738	0.082396887	0.992934677		
Potra2n18c32417.1						
Potra2n18c32418.1	8.622652136	4.629715948	6.05626E-13	0.422139068	Potri.T094100.1	AT4G11650.1

Potra2n18c32419.1	0.095548819	-1.853782611	0.947354962	NA	Potri.T094000.1	AT3G23760.1
Potra2n18c32420.1	8.611232648	4.470326869	1.84669E-09	0.590087814	Potri.T094100.1	AT4G11650.1
Potra2n18c32421.1	-1.756674733	-0.650121401	0.001870369	0.661005194	Potri.T094000.1	AT3G23760.1
Potra2n18c32422.1	0.563641761	1.009012843	2.26318E-05	NA	Potri.T093900.8	AT5G57655.2
Potra2n18c32423.1	7.522901353	3.444875688	3.26573E-19	0.070375978	Potri.T094100.1	AT4G11650.1
Potra2n18c32424.1	-1.434111928	-0.512728427	0.310001682	0.936890993	Potri.T094000.1	AT3G23760.1
Potra2n18c32425.1	0.799391225	1.094961068	1.43704E-10	NA	Potri.T093900.8	AT5G57655.2
Potra2n18c32426.1	4.421104777	0.422137442	3.05E-06	0.928335352	Potri.T094100.1	AT4G11650.1
Potra2n18c32427.1	0.268849594	0.059745503	0.174503714	0.966917036	Potri.T093800.1	AT5G57630.1
Potra2n18c32428.2	-0.025855647	0.038645604	0.814843623	0.952554996	Potri.T093700.3	AT4G25770.1
Potra2n18c32429.1	0.545759769	-0.213692709	0.142715929	0.900551258	Potri.T093600.1	AT4G25780.1
Potra2n18c32430.1	-0.969696678	-0.334237229	0.03596341	0.89438295	Potri.T093500.1	AT4G30320.1
Potra2n18c32431.1	2.685015931	2.676364087	NA	NA	Potri.018G096000.1	AT5G17050.1
Potra2n18c32432.1	NA	NA	NA	NA	Potri.018G095900.1	AT5G57620.1
Potra2n18c32433.4	-0.250097332	0.093332357	0.138257802	0.937846405	Potri.018G095800.1	AT5G57610.1
Potra2n18c32434.1	-0.012113812	0.291846146	0.953607736	0.82104194	Potri.018G095700.2	AT4G30310.2
Potra2n18c32435.1	0.463511197	0.103069666	0.559007526	0.985393296		
Potra2n18c32436.2	-0.082122841	-0.218535714	0.511925482	0.656082742	Potri.018G095300.2	AT5G57580.1
Potra2n18c32437.1	-0.006920037	-0.835252398	0.998515484	NA		
Potra2n18c32438.1	4.304156904	1.853470258	7.2527E-06	0.021874623	Potri.018G095200.1	AT4G25810.1
Potra2n18c32439.1	1.64774763	NA	NA	NA	Potri.018G095000.1	AT1G69490.1
Potra2n18c32440.1	2.44357036	1.046415631	NA	0.651818379	Potri.018G094900.1	AT4G25810.1
Potra2n18c32441.2	2.114576468	3.01106236	0.052584174	0.32064055	Potri.018G094800.1	AT5G57560.1
Potra2n18c32442.1	NA	1.571529889	NA	NA	Potri.018G094700.1	AT4G31920.1
Potra2n18c32443.1	-0.392923883	-0.507962115	0.660529278	0.900869139	Potri.004G193400.1	AT1G56430.1
Potra2n18c32444.1					Potri.018G094500.1	AT4G25830.1
Potra2n18c32445.1					Potri.018G094400.1	AT3G14240.1
Potra2n18c32446.1	-5.173762412	-1.347842223	7.04649E-08	0.451062617		
Potra2n18c32447.1	-4.620893051	-1.26502745	1.71354E-07	0.495345445	Potri.018G094300.1	AT5G57520.1
Potra2n18c32448.1	6.447759806	3.405958176	5.20465E-20	0.30801382	Potri.018G094000.1	AT5G57500.1
Potra2n18c32449.3	0.618103712	0.06141691	0.001449319	0.963878456	Potri.018G093900.1	AT5G57490.1
Potra2n18c32450.1	-0.292142212	-0.248431082	0.019257627	0.701494362	Potri.018G093800.1	AT2G18840.1

Potra2n18c32451.1	-0.085842916	-0.058038123	0.822175513	0.979719961	Potri.018G093700.1	AT2G27800.1
Potra2n18c32452.1	1.676694252	0.462278464	1.91265E-05	0.713237443	Potri.018G093600.2	AT5G57480.1
Potra2n18c32453.1	-0.028829116	0.21518086	0.870706708	0.861982498	Potri.018G093501.1	AT1G05205.1
Potra2n18c32454.1	-0.501518028	0.092360673	1.74489E-08	0.954419252	Potri.018G093400.2	AT4G30240.1
Potra2n18c32455.1	-0.373764532	0.009659542	0.428552921	0.99736971	Potri.018G093100.1	AT5G57450.2
Potra2n18c32456.1	-0.050860201	0.362886701	0.952019919	0.91647306		
Potra2n18c32457.1	-0.678415916	-0.199134802	0.001508474	0.836066102	Potri.005G115300.1	AT5G59690.1
Potra2n18c32458.1	-0.932536081	-0.408152302	0.002548987	0.798648673	Potri.005G115300.1	AT5G59690.1
Potra2n18c32459.1	-0.933616198	0.298969543	0.002023705	0.902676973	Potri.018G092800.1	AT4G30230.1
Potra2n18c32460.1	-1.011250075	-0.226979072	2.99249E-07	0.829862776	Potri.005G115300.1	AT5G59690.1
Potra2n18c32461.1	0.716939788	0.139909032	7.13957E-06	0.914238347	Potri.018G092500.1	AT5G57440.1
Potra2n18c32462.1	0.089304436	-0.184334782	0.797470267	0.938967511	Potri.018G092400.1	AT1G59720.1
Potra2n18c32463.1	0.450744878	-0.997871819	0.542627487	0.696755852	Potri.018G092300.1	AT5G57420.1
Potra2n18c32464.1	0.133651018	0.001657258	0.375059324	0.999325699		
Potra2n18c32465.1	0.451815432	0.36251569	3.29941E-07	0.715300081	Potri.018G092100.2	AT4G30210.2
Potra2n18c32466.1	-0.388228978	-0.200437535	0.069846862	0.848201665	Potri.018G092000.2	AT5G57410.1
Potra2n18c32467.1	1.545832463	NA	NA	NA	Potri.018G091900.1	AT4G25850.2
Potra2n18c32468.1					Potri.018G091800.1	AT4G25850.2
Potra2n18c32469.5	-0.147211073	-0.120156773	0.211196167	0.84435486	Potri.018G091700.1	AT3G02920.1
Potra2n18c32470.3	-1.078463543	-0.594508297	0.014529823	0.726986309	Potri.018G091600.1	AT5G57390.1
Potra2n18c32471.1	-0.337749118	-0.071503216	0.002464536	0.924951347		
Potra2n18c32472.1	-0.002664994	-0.058524234	0.991867476	0.98177802	Potri.018G091401.1	AT5G38420.1
Potra2n18c32473.1	-0.042744189	-0.160491858	0.809949193	0.865591169		
Potra2n18c32474.1	-1.132849227	-0.489816591	0.00344232	0.788065047	Potri.018G091200.1	AT2G18890.1
Potra2n18c32475.1	0.427747531	-0.01417473	0.007249068	0.994329048	Potri.018G091100.1	AT2G18900.1
Potra2n18c32476.1	4.383948577	2.435277079	8.36561E-12	NA	Potri.018G090966.1	AT5G38280.1
Potra2n18c32477.1	-0.419606332	-0.20676089	0.004428814	0.836066102	Potri.018G090900.2	AT2G18910.1
Potra2n18c32478.1	-0.157857177	-0.063224724	0.785800062	0.985393296		
Potra2n18c32479.1	-0.346081281	-0.118534566	0.139858152	0.947969749		
Potra2n18c32480.1	-0.411770766	-0.102979749	4.59398E-05	0.887496314	Potri.018G090800.1	AT5G57360.1
Potra2n18c32481.1	0.484986615	0.005418848	0.000101477	0.996292992	Potri.018G090700.1	AT2G18950.1
Potra2n18c32482.1	0.042708871	0.067954634	0.741868346	0.920729156	Potri.018G090400.1	AT3G11710.1

Potra2n18c32483.2	-0.181162129	-0.044095866	0.430078402	0.974275337	Potri.018G090300.1	AT2G18960.1
Potra2n18c32484.1	0.26457502	-1.332320361	0.936669792	NA	Potri.018G090200.1	AT2G18969.1
Potra2n18c32485.1	0.103256367	-0.160987735	0.627088124	0.901671593	Potri.018G090100.1	AT2G36460.1
Potra2n18c32486.1	-2.378690362	-0.422686991	0.072295272	0.941855873	Potri.018G090000.1	AT5G57340.2
Potra2n18c32487.1	1.459962178	1.381953902	NA	NA	Potri.018G089900.1	AT4G30170.1
Potra2n18c32488.1	2.492428534	NA	NA	NA	Potri.004G016300.1	
Potra2n18c32489.1						
Potra2n18c32490.2	-0.141467605	-0.007078039	0.397563683	0.99488781	Potri.018G089800.1	AT5G57330.1
Potra2n18c32491.3	-0.332220192	0.078914289	0.006561872	0.890417497	Potri.018G089700.3	AT4G30160.1
Potra2n18c32492.1					Potri.016G107700.1	AT2G35040.1
Potra2n18c32493.1	NA	1.571529889	NA	NA	Potri.018G088800.2	AT1G29660.1
Potra2n18c32494.1	-0.937611701	2.965185527	NA	0.645919795		
Potra2n18c32495.1	2.492428534	2.676364087	NA	NA	Potri.018G088800.2	AT1G29660.1
Potra2n18c32496.2	0.025926505	-0.050161329	0.914639235	0.971828097	Potri.018G088400.2	AT5G57300.2
Potra2n18c32497.1	1.664677056	1.363811008	NA	NA		
Potra2n18c32498.1	NA	3.390498064	NA	NA	Potri.018G088800.2	AT1G29660.1
Potra2n18c32499.1	NA	NA	NA	NA	Potri.018G088500.1	AT1G29670.1
Potra2n18c32500.1	-1.35927387	2.181003945	NA	NA		
Potra2n18c32501.1	NA	NA	NA	NA	Potri.018G089200.1	AT1G29670.1
Potra2n18c32502.1	-0.61910233	0.8502037	NA	NA		
Potra2n18c32503.2	0.148264091	-0.064825912	0.489392159	0.961670176	Potri.018G088400.2	AT5G57300.2
Potra2n18c32504.2	0.373648611	-0.030202197	0.00943736	0.98036348	Potri.018G088300.1	AT4G29130.1
Potra2n18c32505.1	2.784274487	0.889748472	1.57662E-21	0.231217782	Potri.018G088200.1	AT5G25930.1
Potra2n18c32506.1	3.821168431	0.834107428	1.45647E-10	0.87909151	Potri.018G088100.1	AT5G25930.1
Potra2n18c32507.1	4.001544529	3.020443752	0.27868309	NA		
Potra2n18c32508.1	0.353473543	0.035823622	0.026445579	0.98177802	Potri.018G088000.1	AT4G29120.1
Potra2n18c32509.1	0.515521821	-0.017361331	0.000479243	0.986636967	Potri.018G087900.1	AT2G37410.1
Potra2n18c32510.1	-0.066965785	-0.183495217	0.79985456	0.885649686	Potri.018G087800.2	AT2G19870.1
Potra2n18c32511.1	0.17607019	0.150239349	0.739560707	0.902676973	Potri.018G087700.1	AT2G19880.2
Potra2n18c32512.1	-0.210452228	-0.112961071	0.558432984	0.943483421	Potri.018G087600.1	AT1G60660.1
Potra2n18c32513.1	0.040699793	0.364482085	0.884135418	0.728418847	Potri.018G087500.2	AT4G15450.1
Potra2n18c32514.1	0.066465705	0.296955054	0.793430458	0.792511865		

Potra2n18c32515.1	1.545832463	NA	NA	NA		
Potra2n18c32516.1	1.182204217	-0.206328849	0.007492607	0.895474078	Potri.018G087400.1	AT2G44260.1
Potra2n18c32517.1	0.555256555	0.266855442	0.003148507	0.814981587	Potri.018G087300.1	AT2G40570.1
Potra2n18c32518.1	0.037603462	0.337401139	0.90253824	0.749682671	Potri.018G087100.1	AT3G28345.1
Potra2n18c32519.1	1.717350416	0.406390991	6.78246E-14	0.5691307	Potri.018G086900.1	AT1G35190.1
Potra2n18c32520.1	0.111299423	0.218125842	0.45576558	0.696743275	Potri.018G086800.1	AT1G35190.1
Potra2n18c32521.1	0.465133676	0.308784922	0.008248851	0.803721734	Potri.018G086700.1	AT1G79750.1
Potra2n18c32522.1	-0.499779698	-0.087512593	7.53284E-05	0.922011871	Potri.018G086600.1	AT2G19950.2
Potra2n18c32523.1	1.822190346	0.434672292	0.126888366	NA	Potri.018G086500.1	AT4G29110.1
Potra2n18c32524.1	2.97934182	1.633288622	0.152934842	NA		
Potra2n18c32525.1	-0.180638498	-0.110751995	0.438142065	0.920141992	Potri.018G086400.5	AT5G15770.1
Potra2n18c32526.5	-0.112296662	-0.08179542	0.529012076	0.903496619	Potri.018G086300.1	AT2G20000.1
Potra2n18c32527.1	0.085732497	0.063392224	0.436502759	0.943819961	Potri.018G086200.1	AT5G19680.1
Potra2n18c32528.1						
Potra2n18c32529.2	0.983584865	0.195057287	2.64961E-11	0.830056077	Potri.018G086000.1	AT5G19690.1
Potra2n18c32530.1	NA	NA	NA	NA		
Potra2n18c32531.1	-0.206082085	-0.214890515	0.117197164	0.64318167	Potri.018G085900.1	AT2G20010.2
Potra2n18c32532.1						
Potra2n18c32533.1	0.102734662	0.421586926	0.842902767	0.791589438	Potri.018G085800.2	AT2G27610.1
Potra2n18c32534.1	5.443460584	2.542176004	8.66499E-07	NA	Potri.018G085700.1	AT4G32800.1
Potra2n18c32535.1	0.192423951	0.581814483	0.379153797	0.742596528	Potri.018G085500.1	AT5G19670.1
Potra2n18c32536.1	0.032752783	0.126767965	0.874090109	0.886059841	Potri.018G085400.1	AT5G19670.1
Potra2n18c32537.1	-0.525976581	-1.669030477	0.799902001	NA	Potri.018G085300.1	AT2G20020.1
Potra2n18c32538.1	1.410399501	0.146909184	0.346052754	NA	Potri.018G085200.1	AT1G77210.2
Potra2n18c32539.1	0.031172762	-0.015475553	0.857273484	0.989878071	Potri.018G085100.2	AT4G28910.1
Potra2n18c32540.1	2.663240908	0.804701292	5.67517E-14	0.540010302	Potri.006G239500.1	AT2G20030.1
Potra2n18c32541.1	-0.170306609	0.033836717	0.243533346	0.979967506	Potri.018G084900.1	AT2G20050.1
Potra2n18c32542.2	-0.01496501	0.102561911	0.929646026	0.899845298	Potri.018G084800.1	AT1G67280.1
Potra2n18c32543.1	0.45003031	0.255019816	0.035669656	0.725595998	Potri.018G084700.1	AT5G51130.1
Potra2n18c32544.1	NA	NA	NA	NA		
Potra2n18c32545.1	-2.337106966	-2.486725844	NA	NA	Potri.018G084600.1	
Potra2n18c32546.3	0.490525831	0.15224107	0.005974958	0.767368598	Potri.018G084500.1	AT4G28880.1

Potra2n18c32547.1	0.73156768	0.472780619	0.045234522	0.692084498		
Potra2n18c32548.1	0.091974805	-0.012195643	0.670462142	0.993589787	Potri.018G084200.1	AT5G19580.1
Potra2n18c32549.1	-0.953641655	0.157183185	0.000447569	0.945254452	Potri.018G084100.1	AT2G20080.1
Potra2n18c32550.1	-0.067988634	0.093265861	0.735147089	0.930871994	Potri.018G083900.2	AT4G29060.1
Potra2n18c32551.1	0.130875958	0.185573254	0.45216351	0.824905557	Potri.018G083800.2	AT4G28830.1
Potra2n18c32552.1	-0.470631875	-0.017607909	0.002815069	0.983446961	Potri.018G083700.1	AT4G29100.1
Potra2n18c32553.1	-0.079826383	-0.161909654	0.788094477	0.921961043	Potri.018G083600.1	AT3G06880.2
Potra2n18c32554.1	0.042462544	0.173153534	0.866015522	0.911339141	Potri.001G423500.1	AT1G65980.1
Potra2n18c32555.1	-0.168318682	-0.015915975	0.474764024	0.993711199	Potri.018G083400.2	AT4G28950.1
Potra2n18c32556.1	0.760022003	-0.050534872	0.593070135	0.996032145		
Potra2n18c32557.1	-0.059632502	0.003813395	0.73487494	0.998027171	Potri.018G083300.1	AT4G28980.2
Potra2n18c32558.1	0.156149423	-0.021687314	0.094621383	0.983446961	Potri.018G083200.1	AT1G67300.1
Potra2n18c32559.1	-0.06506128	0.181509017	0.719888837	0.862143562	Potri.018G083100.1	AT4G28990.1
Potra2n18c32560.1	0.402063224	0.238090806	0.000283311	0.694611009	Potri.018G083000.1	AT4G29000.1
Potra2n18c32561.1	0.417208663	0.162404954	2.3253E-05	0.810712263	Potri.018G082900.4	AT4G29010.1
Potra2n18c32562.1	0.170657134	0.003237018	0.3780965	0.999315506	Potri.018G082800.1	AT1G55205.1
Potra2n18c32563.1	NA	NA	NA	NA	Potri.018G082700.2	
Potra2n18c32564.3	-0.122377505	-0.147573388	0.834953914	0.955473249	Potri.018G082600.1	AT2G46225.2
Potra2n18c32565.1	0.59988101	0.091608331	0.000733642	0.924251575	Potri.018G082500.1	AT5G19550.1
Potra2n18c32566.2	-0.878186123	-0.458723072	0.160876641	0.88643982	Potri.018G082400.5	AT3G24620.1
Potra2n18c32567.1	0.494319171	0.197847459	0.133762446	0.953768643	Potri.018G082300.2	AT3G24630.1
Potra2n18c32568.1	0.301141101	0.197222159	0.111804985	0.497568278	Potri.006G158100.1	AT2G20120.1
Potra2n18c32569.1	0.060357575	0.144223872	0.809967994	0.887496314	Potri.018G081800.2	AT3G24590.1
Potra2n18c32570.1	-0.5979192	0.275325302	0.000121178	0.874808359	Potri.018G081700.1	AT5G21222.1
Potra2n18c32571.1	0.20774816	0.090993523	0.254092589	0.93466433	Potri.018G081600.3	AT3G24570.1
Potra2n18c32572.2	0.067774157	0.030028766	0.775046561	0.981772014	Potri.018G081500.1	AT3G24560.2
Potra2n18c32573.1	0.136253111	0.072546614	0.307645377	0.937789963	Potri.018G081400.1	AT5G19660.1
Potra2n18c32574.1	-0.319016107	-0.136695261	0.013249737	0.926808461	Potri.018G081300.1	AT3G24550.1
Potra2n18c32575.1	-1.092827787	-0.404332279	1.01721E-05	0.229561501	Potri.018G081200.4	AT4G13250.1
Potra2n18c32576.1	NA	NA	NA	NA	Potri.001G378500.1	AT1G17120.1
Potra2n18c32577.1	-0.346258483	-0.212731665	0.013932519	0.818007496	Potri.018G081000.1	AT3G24530.1
Potra2n18c32578.1	-1.174049577	-2.438056518	0.326760694	0.211345761	Potri.018G080100.1	AT5G19650.1

Potra2n18c32579.1	-0.629302687	-0.388628957	0.508665705	0.935101372		
Potra2n18c32580.1	-0.785850751	-0.401594359	0.01168778	0.786548546		
Potra2n18c32581.1	-0.61895335	0.125168512	0.012963453	0.955322605	Potri.T137300.1	AT1G15260.1
Potra2n18c32582.1	-1.830353965	-0.407663449	0.005246196	0.871720501	Potri.T137400.1	AT3G24520.1
Potra2n18c32583.1	0.313330118	0.122629626	0.066491853	0.833667976	Potri.018G079800.1	AT3G16060.1
Potra2n18c32584.1	0.161559642	0.125508417	0.254380773	0.922681078	Potri.018G079700.1	AT3G24506.1
Potra2n18c32585.1	0.042844559	-0.03116186	0.813855949	0.983944804	Potri.018G079600.1	AT1G60770.1
Potra2n18c32586.3	-0.143960302	0.112068557	0.422620793	0.930203195	Potri.018G079500.1	AT4G32350.1
Potra2n18c32587.1	0.837269492	-0.791466197	0.00076408	0.148448694	Potri.018G079300.1	AT5G45130.1
Potra2n18c32588.1	0.60418211	0.792755827	0.036984506	0.428801064	Potri.018G079200.2	AT1G67910.1
Potra2n18c32589.1	NA	NA	NA	NA		
Potra2n18c32590.1	-1.884885581	-0.532060335	0.50158524	NA		
Potra2n18c32591.1	-1.674652401	-0.90082512	0.576461845	NA	Potri.018G078900.1	AT3G13227.1
Potra2n18c32592.1						
Potra2n18c32593.1	4.880470514	3.174708048	1.92066E-16	0.092522065	Potri.018G078801.1	AT5G53250.1
Potra2n18c32594.1	0.029104351	-1.6019E-05	0.881404236	0.999950004	Potri.018G078701.1	AT3G22440.1
Potra2n18c32595.1						
Potra2n18c32596.1	1.636899317	0.50448017	0.436004362	NA	Potri.018G078200.1	AT1G03670.1
Potra2n18c32597.1	-0.081113864	-0.179823562	0.802511069	0.900551258	Potri.018G078400.1	AT3G22440.1
Potra2n18c32598.1	0.860795175	0.556204421	0.654207994	NA	Potri.018G077250.1	AT1G03670.1
Potra2n18c32599.1	2.243903646	0.932802225	0.049910288	0.892986206		
Potra2n18c32600.1	0.739945825	0.105415049	0.533690284	0.99114586		
Potra2n18c32601.1	0.54816929	0.00182825	0.202724953	0.999937671	Potri.018G074600.2	AT1G03670.1
Potra2n18c32602.1	0.684110136	0.010289816	0.175184209	0.999121696	Potri.018G077300.1	
Potra2n18c32603.1	-0.014665327	0.532132088	0.98795791	0.82876584	Potri.018G078400.1	AT3G22440.1
Potra2n18c32604.1	0.32852136	-0.47525601	0.698477539	0.915488947	Potri.018G078400.1	AT3G22440.1
Potra2n18c32605.1	1.647772616	0.171715873	7.94547E-05	0.966917036	Potri.018G078200.1	AT1G03670.1
Potra2n18c32606.1	0.955536796	-0.38758803	0.046949366	0.914551931	Potri.018G077300.1	
Potra2n18c32607.1	-0.215664222	-0.101617338	NA	NA	Potri.018G074700.2	AT1G03670.1
Potra2n18c32608.1	0.614706868	-0.224935602	0.393224782	0.953608209	Potri.018G077250.1	AT1G03670.1
Potra2n18c32609.1	NA	0.674963689	NA	NA		
Potra2n18c32610.1	-0.588974985	0.06581036	0.32595125	0.98669034	Potri.018G075000.4	AT3G24503.1

Potra2n18c32611.1	NA	0.674963689	NA	NA		
Potra2n18c32612.1	0.322297001	0.234669485	0.304788813	0.881333174	Potri.018G075200.1	AT3G24500.1
Potra2n18c32613.1	-0.51125183	0.257332757	0.54894789	0.939644353	Potri.018G075300.1	AT5G19610.1
Potra2n18c32614.1	1.014786253	2.188680644	NA	NA		
Potra2n18c32615.1	-0.062754843	-0.040397357	0.645036087	0.954098985	Potri.018G075500.1	AT3G24490.1
Potra2n18c32616.1	-0.034711773	0.367218614	0.950066534	0.874808359		
Potra2n18c32617.1	-0.078621828	-0.059296631	0.535329174	0.930598202		
Potra2n18c32618.1	-0.139952561	-0.29040311	0.811933215	0.909715408	Potri.018G075800.1	
Potra2n18c32619.1	0.830048314	0.136999955	0.015853229	0.947969749		
Potra2n18c32620.1	0.745620722	-0.015801256	0.032084224	0.995579852	Potri.018G075900.1	AT4G18670.1
Potra2n18c32621.1	0.410957376	-0.472841589	0.400101907	0.759834612		
Potra2n18c32622.1	0.565146152	-0.200543139	0.102855194	0.90634987		
Potra2n18c32623.1	-0.443791124	-0.027639208	0.011634682	0.980890614	Potri.018G076000.1	AT3G24460.1
Potra2n18c32624.1	0.277625243	0.210591684	0.028945874	0.584187558	Potri.018G076200.1	AT4G13350.1
Potra2n18c32625.1	-1.328951947	-0.068349583	NA	NA		
Potra2n18c32626.1	0.82357019	0.674963689	NA	NA		
Potra2n18c32627.1	-1.35927387	-0.097655521	NA	NA		
Potra2n18c32628.1	NA	NA	NA	NA	Potri.018G076400.1	AT3G24450.1
Potra2n18c32629.1	0.827622564	-0.114971323	0.101798928	0.926234768	Potri.018G076500.1	AT3G24440.1
Potra2n18c32630.1	-1.435003209	-0.055600118	2.63563E-11	0.977530072	Potri.018G076600.3	AT3G24430.1
Potra2n18c32631.1	0.033037878	0.196605778	0.86880271	0.741833788	Potri.018G076700.3	AT4G13400.1
Potra2n18c32632.1	2.312596046	1.135744979	1.63426E-06	0.389989789	Potri.018G072101.1	AT4G13395.1
Potra2n18c32633.1	-0.295330323	-0.540230494	0.4914008	0.666699452		
Potra2n18c32634.1	0.546118085	0.267973164	0.000373167	0.694611009	Potri.018G076901.1	AT3G56120.1
Potra2n18c32635.1	1.211778948	1.374322399	0.489309057	NA	Potri.018G077000.1	AT3G24330.1
Potra2n18c32636.1	-0.7798611	-0.873002103	NA	NA		
Potra2n18c32637.1	-0.186819124	0.02592565	0.377421365	0.9896961	Potri.018G072400.1	AT3G24350.1
Potra2n18c32638.1	0.251380603	-0.580408971	0.646029377	0.859892315	Potri.018G072500.1	AT4G13370.1
Potra2n18c32639.1	0.078481921	0.11229013	0.604591477	0.871720501	Potri.018G072600.3	AT2G26890.1
Potra2n18c32640.1	-1.328951947	1.491399496	NA	NA	Potri.001G231400.3	AT2G44290.1
Potra2n18c32641.1	0.117466396	0.016668464	0.442632714	0.990874899	Potri.018G072700.1	AT1G08510.1
Potra2n18c32642.1	-0.24581205	-0.076323659	0.114545894	0.957533867	Potri.018G072900.1	AT5G25510.1

Potra2n18c32643.1	0.498434112	0.643066658	0.03829288	0.564195735	Potri.018G073000.1	AT5G19930.1
Potra2n18c32644.1	0.005022947	0.067381027	0.966899239	0.936549944	Potri.018G073100.2	AT5G19950.1
Potra2n18c32645.1	-0.13595682	-0.349827561	0.870012767	0.922011871	Potri.018G074500.1	AT2G26910.1
Potra2n18c32646.1	-0.436764921	-0.05343077	0.003066475	0.965515007	Potri.018G074400.1	AT2G26900.1
Potra2n18c32647.1	-0.559649494	-0.293228665	0.002499102	0.846499552	Potri.018G074300.1	AT2G26730.1
Potra2n18c32648.1	3.595214142	2.281598548	0.048253188	NA	Potri.018G074200.1	AT2G03500.1
Potra2n18c32649.1	-0.030195657	0.025487049	0.836610484	0.977559521	Potri.018G074100.2	AT5G19960.1
Potra2n18c32650.1	0.419784916	0.454443271	0.452848974	0.901195443	Potri.018G071900.1	AT5G19970.1
Potra2n18c32651.1	0.250873518	0.152338606	0.793802004	0.981772014	Potri.018G071800.1	AT1G08070.1
Potra2n18c32652.1	0.344466359	0.06354001	0.055477473	0.954419252	Potri.018G071700.1	AT5G19980.1
Potra2n18c32653.1	0.533763447	-0.186320896	0.025283041	0.902857376	Potri.018G071600.1	AT5G20140.1
Potra2n18c32654.1	0.336390287	0.034299012	0.005322011	0.974756511	Potri.018G073400.2	AT1G11020.1
Potra2n18c32655.1	0.919794998	0.252627211	1.53539E-06	0.842247476	Potri.018G071400.1	AT2G26650.1
Potra2n18c32656.1	0.528766128	0.242543688	0.000636283	0.813731863	Potri.018G071200.2	AT1G04870.2
Potra2n18c32657.1	1.931605898	NA	NA	NA	Potri.018G070900.1	AT2G26710.1
Potra2n18c32658.1	3.865828524	NA	0.168142507	NA	Potri.018G070800.1	AT5G20050.1
Potra2n18c32659.1	0.205446007	0.167861479	0.063748187	0.755109612	Potri.018G070700.1	AT5G20060.3
Potra2n18c32660.1	-0.947289408	-0.176309786	0.098829858	0.956865545		
Potra2n18c32661.1	-0.414883723	-0.141940209	0.000451686	0.850863374	Potri.018G070600.1	AT4G17486.1
Potra2n18c32662.1	0.185134327	0.198650798	0.020310898	0.820063065	Potri.018G070500.1	AT3G25910.1
Potra2n18c32663.1	0.596187421	0.013723375	5.89086E-05	0.993589787	Potri.018G070400.1	AT5G20070.1
Potra2n18c32664.2	0.273133684	-0.030690877	0.048272064	0.979724557	Potri.018G070200.1	AT1G72550.1
Potra2n18c32665.1	0.659522674	0.279931043	0.000153476	0.79819686	Potri.018G070100.1	AT2G19740.1
Potra2n18c32666.1	0.537573974	0.31272477	1.23177E-06	0.650005812	Potri.018G070000.1	AT5G56680.1
Potra2n18c32667.1	-0.339307233	-0.198593691	0.000371188	0.759797728	Potri.006G154000.3	AT4G29160.1
Potra2n18c32668.1	0.178120728	0.228008546	0.701617294	0.911993336	Potri.018G069800.1	AT4G29170.1
Potra2n18c32669.3	0.893596022	2.857240791	0.282258531	NA	Potri.018G069700.2	AT4G26260.2
Potra2n18c32670.1	-0.442911675	0.138385897	0.028647999	0.935851032	Potri.005G082800.1	AT4G39640.1
Potra2n18c32671.1	NA	1.381953902	NA	NA		
Potra2n18c32672.3	0.511979318	0.064447477	0.008397563	0.963878456	Potri.018G069200.3	AT4G26270.1
Potra2n18c32673.1	0.597911031	0.102281216	0.002056985	0.947317412		
Potra2n18c32674.1	-1.319555566	-1.225502441	0.150012753	0.335127138	Potri.018G069100.1	AT5G47240.1

Potra2n18c32675.1	0.082220488	0.476603546	0.850217509	0.805461106	Potri.018G068700.1	AT4G29230.1
Potra2n18c32676.1	-0.17723077	-0.07089259	0.315543764	0.953831244	Potri.018G068600.1	AT5G56590.1
Potra2n18c32677.1	0.549188877	-0.190510116	0.099266696	0.862824894	Potri.018G068500.1	AT5G56580.1
Potra2n18c32678.1	1.52354942	0.01620397	1.6689E-06	0.995579852	Potri.018G068400.1	AT5G19730.1
Potra2n18c32679.1	0.369219976	0.218241116	0.109726164	0.837070468	Potri.018G068300.1	AT3G47650.1
Potra2n18c32680.1	0.22204881	0.354123799	0.10856544	0.682369256	Potri.018G068200.2	AT2G19520.1
Potra2n18c32681.1	0.159152716	0.110661178	0.386122832	0.940771888	Potri.018G068100.10	AT5G56890.1
Potra2n18c32682.1	0.184448444	0.132341674	0.138339248	0.893265533	Potri.018G068000.1	AT3G47590.1
Potra2n18c32683.3	-0.084148163	-0.031487253	0.771609418	0.985393296	Potri.018G067900.1	AT2G19490.1
Potra2n18c32684.1	-0.260113265	0.220593932	0.62646703	0.94169165	Potri.018G067900.1	AT2G19490.1
Potra2n18c32685.1	0.061866733	0.062055222	0.65302283	0.932242797	Potri.018G067800.1	AT2G19480.3
Potra2n18c32686.1	-0.045414192	-0.390425376	0.87736007	0.560708883		
Potra2n18c32687.1	-0.375949148	-0.169955876	0.022986421	0.848131816	Potri.018G067700.1	AT5G56750.1
Potra2n18c32688.1	0.436492506	0.149427817	0.011831191	0.884463725	Potri.018G067300.1	AT4G29520.1
Potra2n18c32689.3	0.806778378	0.20193007	7.70655E-12	0.721007112	Potri.018G067200.1	AT4G29510.1
Potra2n18c32690.1						
Potra2n18c32691.1	NA	2.676364087	NA	NA	Potri.018G067000.1	AT4G26200.1
Potra2n18c32692.1	2.632524198	NA	NA	NA	Potri.018G066900.3	AT2G26695.1
Potra2n18c32693.1	0.077593206	0.141375167	0.571109198	0.824378527	Potri.018G066700.1	AT5G20120.1
Potra2n18c32694.1	0.37726281	0.045876011	0.00057827	0.966005919	Potri.018G066600.1	AT4G29680.1
Potra2n18c32695.1	-0.371822355	0.114341881	0.019821308	0.91525372	Potri.018G066500.1	AT4G29670.1
Potra2n18c32696.1	0.068704715	0.243672695	0.903186825	0.931774514	Potri.018G066400.1	AT2G19570.1
Potra2n18c32697.1	0.072241917	-0.273139326	0.825042107	0.764173811	Potri.018G066300.2	AT1G72740.2
Potra2n18c32698.1	0.660043735	-0.001968037	1.10714E-05	0.999325699		
Potra2n18c32699.2	0.577544692	0.113331742	1.42937E-07	0.858398895	Potri.018G066100.1	AT2G19450.1
Potra2n18c32700.1	-0.016700303	-0.01825058	0.89969699	0.98177802	Potri.018G065900.6	AT1G72710.1
Potra2n18c32701.1	-0.137049013	-0.058484605	0.559134368	0.957668459	Potri.018G065800.4	AT2G06010.1
Potra2n18c32702.1	-1.561600534	-0.921245278	0.000325876	0.454005567	Potri.018G065700.1	AT3G42725.1
Potra2n18c32703.1	0.207687971	0.022938008	0.61877592	0.995139072	Potri.018G065600.2	AT2G06050.1
Potra2n18c32704.1	-2.445459122	-0.576694343	0.177494359	0.950095151	Potri.018G065400.1	AT3G13960.1
Potra2n18c32705.1	0.047945185	-0.244186418	0.792629069	0.694769714	Potri.018G065300.1	AT2G06510.1
Potra2n18c32706.1	0.001972906	-0.520508151	0.995973022	0.353695439	Potri.018G065200.1	AT2G06520.1

Potra2n18c32707.1	-0.079129067	-0.00891561	0.575312677	0.995579852	Potri.018G065100.1	AT2G06530.1
Potra2n18c32708.1	-2.191501413	-0.776301758	1.19874E-05	0.435083833	Potri.018G065000.1	AT3G13980.1
Potra2n18c32709.2	-0.821716193	0.750016692	0.004766642	0.63682724	Potri.018G064900.2	AT5G20540.1
Potra2n18c32710.2	0.085391839	0.137940316	0.603589744	0.852534479	Potri.018G064800.1	AT5G20520.1
Potra2n18c32711.1	0.460286384	0.563218575	0.096334594	0.667038977	Potri.018G064700.1	AT1G72830.1
Potra2n18c32712.1					Potri.018G064600.1	AT3G51800.3
Potra2n18c32713.1	-0.168415181	0.055036529	0.157430682	0.948577865	Potri.018G064500.1	AT3G43240.1
Potra2n18c32714.1	NA	NA	NA	NA	Potri.003G058100.1	AT2G01050.1
Potra2n18c32715.2	-0.667568495	-0.068028985	4.43431E-06	0.954419252	Potri.018G064400.1	AT3G53690.1
Potra2n18c32716.1	-0.093131552	-0.020984152	0.46052349	0.985393296	Potri.018G064200.1	AT3G43230.1
Potra2n18c32717.1	-0.023014987	-0.034734791	0.841736751	0.959881296	Potri.018G064100.1	AT2G26460.1
Potra2n18c32718.1	-0.067959006	-0.015635415	0.550564107	0.989304496	Potri.018G063700.2	AT3G43220.1
Potra2n18c32719.1	0.065697632	0.072561813	0.638908075	0.924309478	Potri.018G063600.1	AT4G33250.1
Potra2n18c32720.1	-0.234493597	-0.126290872	0.481741536	0.966434509	Potri.018G063500.1	AT3G43190.1
Potra2n18c32721.1	-1.077521185	0.539511983	0.050744844	0.843683654	Potri.018G063400.1	AT5G20810.1
Potra2n18c32722.1	-0.841286868	-0.111895343	0.001792804	0.92978159	Potri.018G063300.1	AT3G26060.1
Potra2n18c32723.1	0.189187948	-0.033721852	0.381217354	0.985432044	Potri.018G063200.1	AT5G20720.1
Potra2n18c32724.1	-0.115223279	0.064076907	0.554591424	0.945454119	Potri.018G063000.1	AT1G19220.1
Potra2n18c32725.2	0.337829683	1.176076189	0.025886843	0.293583922	Potri.018G062900.2	AT1G74960.2
Potra2n18c32726.1	1.459962178	1.363811008	NA	NA	Potri.018G062800.1	AT5G20710.1
Potra2n18c32727.1	-0.367277186	-0.163678712	0.023130581	0.904788333	Potri.018G062700.2	AT1G19170.1
Potra2n18c32728.1	0.26351997	0.056342977	0.582889444	0.985268281	Potri.018G062600.1	AT1G23070.1
Potra2n18c32729.2	-0.107688807	0.084356492	0.496983305	0.943411075	Potri.018G062500.1	AT1G19110.1
Potra2n18c32730.1	1.459962178	NA	NA	NA	Potri.018G062400.1	AT5G20635.1
Potra2n18c32731.1	1.545832463	1.381953902	NA	NA	Potri.006G227100.1	AT5G56450.1
Potra2n18c32732.1	2.846495174	0.936496624	6.64023E-08	0.794596445	Potri.006G226900.1	AT4G33040.1
Potra2n18c32733.1	3.058853255	1.363811008	NA	NA		
Potra2n18c32734.2	-1.089424525	0.257901137	4.28101E-08	0.813731863	Potri.018G062100.1	AT2G26170.1
Potra2n18c32735.1	0.100857392	-1.081395443	0.928565967	0.71411664	Potri.006G226600.2	AT2G26180.1
Potra2n18c32736.1	2.122421893	0.896151529	0.003033359	0.727843918	Potri.018G061900.1	AT4G33050.3
Potra2n18c32737.1	0.012842713	0.094566371	0.932114476	0.864065517	Potri.018G061800.1	AT4G32960.1
Potra2n18c32738.3	-0.023129901	0.097943747	0.867614484	0.881894261	Potri.018G061701.1	AT4G33060.1

Potra2n18c32739.1	2.614817231	0.830490214	1.79802E-05	0.393597948		
Potra2n18c32740.1						
Potra2n18c32741.1	0.018457396	0.029561191	0.885152724	0.972470034	Potri.018G061300.1	AT3G13920.1
Potra2n18c32742.1	-0.272224333	0.101146899	0.01591481	0.902857376	Potri.018G056400.1	AT2G19390.1
Potra2n18c32743.2	-0.537057993	-0.126236474	1.35339E-08	0.87458517	Potri.018G056200.2	AT5G57040.1
Potra2n18c32744.1	-0.106690756	0.169607831	0.302933587	0.867960518	Potri.018G056100.2	AT2G25950.1
Potra2n18c32745.1	-0.149642723	-0.022074755	0.707972292	0.991407187	Potri.018G056000.1	AT5G11760.1
Potra2n18c32746.1	1.931624282	1.056397687	0.006793362	0.792511865	Potri.018G055900.1	
Potra2n18c32747.1	0.569086358	0.191382541	0.007510011	0.912168773	Potri.018G055832.1	AT5G26030.1
Potra2n18c32748.1	0.781987703	-0.046370319	NA	NA		
Potra2n18c32749.1	0.413840454	0.075700505	0.00093842	0.953768643	Potri.018G055700.1	AT4G26210.1
Potra2n18c32750.1	0.244885546	0.230910011	0.08686818	0.79477084	Potri.018G055300.1	AT2G25970.1
Potra2n18c32751.1	0.36503175	0.106427292	0.77487576	0.990390312	Potri.018G055100.1	AT1G23550.1
Potra2n18c32752.1	-0.006615604	0.16652026	0.993543918	0.962820058	Potri.018G055100.1	AT1G23550.1
Potra2n18c32753.1	-0.126759156	0.095094876	0.304976215	0.911749053	Potri.006G231000.1	AT1G17720.1
Potra2n18c32754.1	0.083873608	0.387002824	0.639038109	0.422139068	Potri.018G054900.2	AT5G56750.1
Potra2n18c32755.1	0.549538673	0.288131991	0.044827394	0.823926359	Potri.018G054800.1	AT1G17710.1
Potra2n18c32756.1	0.360617052	0.474900081	0.176774792	0.817799498	Potri.018G054700.5	AT4G32980.1
Potra2n18c32757.1					Potri.018G054400.1	AT2G26040.1
Potra2n18c32758.1	-0.255303082	-0.062186089	0.088648857	0.949847686	Potri.018G054100.2	AT5G11800.1
Potra2n18c32759.1						
Potra2n18c32760.1	0.391235847	0.428883048	0.320077574	0.802631112	Potri.006G229900.2	AT2G26070.1
Potra2n18c32761.1	-0.016760019	0.019518267	0.931625065	0.984292161	Potri.018G053800.1	AT5G11850.1
Potra2n18c32762.1	2.556135291	NA	NA	NA		
Potra2n18c32763.1	NA	NA	NA	NA		
Potra2n18c32764.1	1.64774763	3.145099921	NA	NA	Potri.018G061600.1	AT5G12000.1
Potra2n18c32765.1						
Potra2n18c32766.1	0.854045141	0.576389364	0.005096367	0.23556479		
Potra2n18c32767.1	-0.310045143	-0.048359286	0.003950065	0.962120332	Potri.018G060300.2	AT1G72770.1
Potra2n18c32768.1	-1.758332384	-0.918543577	0.629031067	NA	Potri.018G059800.1	AT1G79630.1
Potra2n18c32769.1					Potri.018G059700.1	AT3G20430.1
Potra2n18c32770.1	1.459962178	NA	NA	NA	Potri.018G059500.1	

Potra2n18c32771.1	-1.868550341	-0.376661088	1.40855E-09	0.87679119	Potri.018G059400.1	AT4G32940.1
Potra2n18c32772.1	-0.054464551	-0.0588759	0.720112602	0.947969749	Potri.006G233100.1	AT1G55340.1
Potra2n18c32773.1	-1.047470542	-0.220699603	4.35619E-06	0.873955419	Potri.018G059100.1	AT5G11730.1
Potra2n18c32774.1	-0.257297475	-0.009842216	0.218037425	0.995579852	Potri.006G234100.1	AT5G11710.1
Potra2n18c32775.1	0.158447136	0.168777703	0.154868413	0.829862776	Potri.018G058900.1	AT2G25910.1
Potra2n18c32776.1	2.077378129	-0.18996269	0.001978815	0.98177802	Potri.018G058800.2	AT3G49690.1
Potra2n18c32777.1	NA	NA	NA	NA	Potri.T153700.1	AT1G71820.2
Potra2n18c32778.1	-0.161136268	0.376320864	0.598740213	0.87909151	Potri.018G058600.1	AT4G29190.1
Potra2n18c32779.1	-2.988992255	0.637999461	NA	NA	Potri.018G058400.1	
Potra2n18c32780.1						
Potra2n18c32781.1	-0.161037931	0.038458878	0.139414297	0.977559521	Potri.018G058300.1	AT5G11700.2
Potra2n18c32782.1	0.232566283	0.259663491	0.148522047	0.829159711	Potri.018G058200.1	AT4G32915.1
Potra2n18c32783.1	0.894202407	-1.210882864	0.437609816	NA		
Potra2n18c32784.2	0.58673954	0.133700779	0.005358714	0.922029226	Potri.018G058000.1	AT5G27430.1
Potra2n18c32785.2	-0.066854543	0.164400969	0.590493387	0.790748053	Potri.018G057900.3	AT4G32820.1
Potra2n18c32786.1	1.651706702	3.311481645	NA	NA	Potri.006G234900.1	AT2G25890.1
Potra2n18c32787.1	0.015754168	-0.275982247	0.965441168	0.832979932	Potri.006G235000.1	AT4G32830.1
Potra2n18c32788.1	-0.23306937	-0.121442899	0.003311816	0.795173204	Potri.018G057600.10	AT2G19770.1
Potra2n18c32789.1	-0.272449693	-0.102421702	0.032047567	0.896306549	Potri.018G057500.2	AT2G25870.1
Potra2n18c32790.3	-0.289265502	0.009498427	0.052234367	0.992934677	Potri.018G057200.1	AT4G32850.5
Potra2n18c32791.1	0.140200646	0.060159983	0.257077011	0.925184207	Potri.018G057100.1	AT5G25930.1
Potra2n18c32792.1	-0.725721943	-0.070249641	0.064945169	0.98177802	Potri.018G057000.1	AT3G16500.1
Potra2n18c32793.1					Potri.018G053760.1	AT5G42955.1
Potra2n18c32794.1	4.410222053	3.495980907	0.029262828	NA	Potri.018G053760.1	AT5G42955.1
Potra2n18c32795.1	NA	0.674963689	NA	NA	Potri.018G053760.1	AT5G42955.1
Potra2n18c32796.1	NA	3.025856371	NA	NA	Potri.006G229300.1	AT4G33010.1
Potra2n18c32797.1	-2.979154818	0.042330885	0.022928135	0.995579852	Potri.018G053600.1	AT5G56860.1
Potra2n18c32798.1	0.501807237	0.010622948	0.002860496	0.993641684	Potri.018G053300.2	AT1G60960.1
Potra2n18c32799.2	0.503010175	-0.266104871	0.869516333	NA	Potri.006G228900.1	AT2G26100.1
Potra2n18c32800.1	-1.131200783	-0.413039554	4.92853E-06	0.769643258	Potri.018G053000.2	AT2G26110.1
Potra2n18c32801.1	0.01345439	-0.005494426	0.930760713	0.996032145	Potri.018G052900.1	AT5G11900.1
Potra2n18c32802.2	NA	NA	NA	NA	Potri.018G052700.2	AT3G13850.1

Potra2n18c32803.1	NA	NA	NA	NA	Potri.006G228400.1	AT1G68400.1
Potra2n18c32804.1	-0.404681757	-0.549417515	0.698893547	0.876272842		
Potra2n18c32805.1	-0.978914068	-0.562196274	5.37967E-05	0.669027738	Potri.018G052500.1	AT5G11890.1
Potra2n18c32806.1	-0.921856581	-0.411217238	0.118284453	0.912602562	Potri.010G220900.1	AT3G07950.1
Potra2n18c32807.1	0.243756421	0.208617524	0.292474314	0.831463341	Potri.018G052200.1	AT1G55110.1
Potra2n18c32808.1	2.440320822	0.151993677	0.340117101	NA		
Potra2n18c32809.1	-0.254779158	-0.015761406	0.110445525	0.987288338	Potri.018G051900.2	AT3G26850.2
Potra2n18c32810.1	-0.005454501	0.101645268	0.97835821	0.924256651	Potri.015G059700.1	AT5G39040.1
Potra2n18c32811.1	-0.028865809	0.071039414	0.890277693	0.953831244	Potri.018G051600.1	AT1G74120.1
Potra2n18c32812.1	0.114136424	0.263875159	0.581082655	0.753052725	Potri.018G051500.1	AT5G07950.1
Potra2n18c32813.1	-0.102290376	0.931333365	0.955822915	0.892570555	Potri.018G051400.1	AT3G14310.1
Potra2n18c32814.1	NA	NA	NA	NA		
Potra2n18c32815.1	3.256058854	0.065624825	3.94636E-05	0.992934677	Potri.018G051200.1	AT2G26450.1
Potra2n18c32816.1						
Potra2n18c32817.1	-0.140707105	-0.021873817	0.240443307	0.983113084	Potri.018G051100.2	AT5G35460.1
Potra2n18c32818.1	-0.377340582	0.049355977	0.002361852	0.963878456	Potri.018G050900.7	AT4G26060.1
Potra2n18c32819.2	1.423126673	0.421153274	2.86056E-16	0.689693427	Potri.018G050800.2	AT4G29810.1
Potra2n18c32820.1	NA	3.157019191	NA	NA		
Potra2n18c32821.1	0.037012451	0.010444349	0.817631101	0.994762886	Potri.018G050300.1	AT1G72820.1
Potra2n18c32822.1	NA	1.571529889	NA	NA		
Potra2n18c32823.1	-0.483011063	-0.004869992	0.000173082	0.99648431	Potri.018G050200.1	AT5G20510.1
Potra2n18c32824.1	NA	1.394613675	NA	NA	Potri.018G050100.1	
Potra2n18c32825.1	-1.212371984	-0.048294446	0.374271818	0.99488781	Potri.018G049900.1	AT3G13980.1
Potra2n18c32826.1	0.470001534	0.135169402	0.001923189	0.910815461	Potri.018G049800.3	AT5G57280.1
Potra2n18c32827.1	0.270498051	0.059491101	0.492780682	0.98177802	Potri.006G221400.1	AT1G33240.1
Potra2n18c32828.3	2.170337691	1.25258277	0.022268299	0.814981587	Potri.018G049600.1	AT2G16720.1
Potra2n18c32829.1	-1.672434581	-0.758448374	0.033631211	0.896627841	Potri.018G088600.1	AT1G29670.1
Potra2n18c32830.1	-0.7798611	-2.466612692	NA	NA	Potri.018G089500.1	AT1G29660.1
Potra2n18c32831.1	0.465172748	0.029324858	0.000764422	0.982665095	Potri.018G049100.1	AT5G35530.1
Potra2n18c32832.1	NA	NA	NA	NA	Potri.018G049000.1	AT5G14750.1
Potra2n18c32833.1	0.280906668	0.072981835	0.004142173	0.912666272	Potri.018G048700.3	AT4G33210.1
Potra2n18c32834.1	0.449121321	0.085514105	0.009518285	0.951752151		

Potra2n18c32835.1	0.282097837	-0.1130044	0.134038164	0.922029226	Potri.018G048600.1	AT2G26300.1
Potra2n18c32836.1	0.309174315	-0.067228612	0.280970647	0.967940508	Potri.018G048500.1	AT2G26310.1
Potra2n18c32837.1					Potri.006G219700.1	AT3G43860.1
Potra2n18c32838.1	0.282286788	1.217892958	0.549369387	0.734313982	Potri.018G048100.1	AT5G21940.1
Potra2n18c32839.1	-0.164586117	-0.399908646	0.951372889	NA		
Potra2n18c32840.1	2.735991243	NA	NA	NA	Potri.018G048000.1	AT2G26320.1
Potra2n18c32841.1	3.473556452	NA	0.406922964	NA	Potri.006G220100.1	AT2G26330.1
Potra2n18c32842.1	2.340207562	NA	NA	NA	Potri.006G220100.1	AT2G26330.1
Potra2n18c32843.1	1.664677056	0.674963689	NA	NA	Potri.018G047901.1	AT2G26330.1
Potra2n18c32844.1	1.552005842	0.560375132	4.17016E-16	0.496830954	Potri.018G047800.1	AT5G21930.1
Potra2n18c32845.2	0.142502254	0.20505988	0.191961954	0.846680547	Potri.018G047700.8	AT2G26280.1
Potra2n18c32846.1	-0.110162238	0.004972515	0.220198057	0.995579852	Potri.018G047500.1	AT3G43920.2
Potra2n18c32847.1	-0.05871583	0.095770764	0.702312056	0.930871994	Potri.018G047400.2	AT1G74530.3
Potra2n18c32848.1	1.999668923	0.871917256	0.001976829	0.817452127	Potri.018G047300.1	AT1G19210.1
Potra2n18c32849.1	2.343784188	-1.519215768	0.289709704	NA		
Potra2n18c32850.1	0.159054031	0.069025879	0.586228494	0.971317865	Potri.018G047100.1	AT5G20900.1
Potra2n18c32851.1	-0.160144145	-0.035244891	0.117095641	0.957437312	Potri.018G047000.1	AT2G26210.1
Potra2n18c32852.1	3.331628765	1.216048723	NA	0.682454638	Potri.018G046900.2	AT5G20885.1
Potra2n18c32853.1	-1.091066169	-0.577530221	0.253668683	0.910477984	Potri.018G046800.2	AT3G16360.2
Potra2n18c32854.1	0.109552839	-0.065653422	0.280097147	0.931774514	Potri.018G046700.2	AT2G25740.1
Potra2n18c32855.1	4.49871902	1.674321254	1.04198E-38	0.170845723	Potri.018G046600.1	AT5G25880.1
Potra2n18c32856.1	-0.28329105	-3.659844973	0.894705435	NA	Potri.018G046500.1	AT1G52140.1
Potra2n18c32857.1	-0.299575757	-0.11332321	0.000475945	0.849604439	Potri.018G046400.1	AT2G25760.1
Potra2n18c32858.1	-0.251731091	-0.087605773	0.43041104	0.949966783		
Potra2n18c32859.1	-0.693533035	-0.022592159	4.64493E-06	0.978917914	Potri.018G046200.1	AT1G15215.2
Potra2n18c32860.1	-1.47504672	-0.381117257	3.68175E-16	0.582021931	Potri.018G046100.1	AT2G25770.1
Potra2n18c32861.1	-5.636614247	-0.187873632	0.000509647	0.98177802	Potri.006G236800.1	AT4G32870.1
Potra2n18c32862.1	0.190967047	0.080037822	0.14968974	0.947969749	Potri.018G045800.1	AT5G11650.1
Potra2n18c32863.1	NA	NA	NA	NA	Potri.018G045600.1	AT5G25840.1
Potra2n18c32864.1	-1.872078583	-0.454785718	7.36671E-09	0.895460013	Potri.018G045500.1	AT2G25790.1
Potra2n18c32865.1					Potri.018G045250.1	AT5G42955.1
Potra2n18c32866.1	0.903569205	0.127764035	1.89169E-05	0.956865545	Potri.018G045100.1	AT4G32880.1

Potra2n18c32867.1	0.911943241	0.144034508	2.71398E-05	0.953831244	Potri.018G045100.1	AT4G32880.1
Potra2n18c32868.1	NA	1.363811008	NA	NA	Potri.007G089350.1	AT1G43760.1
Potra2n18c32869.1	-2.143270236	0.462124809	0.526577094	NA	Potri.018G044900.1	AT5G25830.1
Potra2n18c32870.1	-0.253945245	0.159121125	0.199337801	0.885375133	Potri.001G402800.1	AT3G15430.2
Potra2n18c32871.4	-0.186137934	-0.114405043	0.076494129	0.900637717	Potri.018G044300.1	AT4G32910.1
Potra2n18c32872.2	-0.67241868	-0.386882841	0.000170462	0.365730533	Potri.018G044200.1	AT2G25800.1
Potra2n18c32873.1	-2.473943521	-0.69324602	3.15705E-05	0.849446235	Potri.018G044100.1	AT4G32810.1
Potra2n18c32874.1					Potri.004G232250.1	AT5G49760.1
Potra2n18c32875.1	-1.133214636	-0.639319917	0.01074595	0.573339469	Potri.018G043900.1	AT5G11590.1
Potra2n18c32876.2	-0.059600721	0.132603542	0.812682099	0.910815461	Potri.018G043800.1	AT5G11600.1
Potra2n18c32877.2	-0.291152423	0.017324173	0.09731427	0.98876314	Potri.018G043700.1	AT5G25820.1
Potra2n18c32878.1	-0.331086256	-0.225140118	0.049198144	0.780946564	Potri.018G043600.1	AT3G46210.6
Potra2n18c32879.1	8.737176708	2.774467021	1.6497E-129	0.375012853	Potri.018G043500.1	AT3G63520.1
Potra2n18c32880.1	0.387313001	0.128478485	0.056706048	0.936549944	Potri.T167700.1	AT3G63520.1
Potra2n18c32881.1	0.400957711	0.158372909	0.032346313	0.910815461	Potri.018G042800.1	AT1G54690.1
Potra2n18c32882.1	10.83919187	6.936028347	3.70924E-21	0.468463678	Potri.018G042200.1	AT1G10340.1
Potra2n18c32883.1	-1.253510773	-0.38504554	0.000112873	0.903477395	Potri.018G042100.1	AT1G67280.1
Potra2n18c32884.1	-1.606949467	-0.471390289	1.24481E-06	0.84745523	Potri.018G042000.2	AT4G14740.1
Potra2n18c32885.1	-0.514905721	0.126782149	0.265293086	0.956865545	Potri.018G041000.2	AT4G28880.1
Potra2n18c32886.1	0.470940969	0.345387575	0.007117464	0.333353361	Potri.018G041800.1	AT1G52190.1
Potra2n18c32887.2	-1.339964097	0.624648695	0.015346752	0.790748053	Potri.018G040500.1	AT1G52190.1
Potra2n18c32888.1	2.039835169	3.721226497	0.56624232	NA	Potri.018G040500.1	AT1G52190.1
Potra2n18c32889.1	-0.719521158	1.749973466	0.749969479	0.525835282	Potri.018G040500.1	AT1G52190.1
Potra2n18c32890.1	NA	1.571529889	NA	NA	Potri.018G040400.1	AT3G16180.1
Potra2n18c32891.3	-0.007644135	0.18027187	0.988066205	0.953108412	Potri.018G040301.1	AT1G52190.1
Potra2n18c32892.1	0.139335357	0.107267463	0.30885722	0.876272842	Potri.018G040200.2	AT4G32770.1
Potra2n18c32893.1	-0.467187152	-0.23572158	0.177388662	0.921015963	Potri.018G040100.1	AT1G08070.1
Potra2n18c32894.1	1.545832463	NA	NA	NA	Potri.018G040000.1	
Potra2n18c32895.1	0.073321348	0.079698688	0.701508551	0.939143297	Potri.018G039800.1	AT5G11560.1
Potra2n18c32896.1	-0.721437079	-0.270391781	0.015297433	0.88643982	Potri.018G039600.1	AT5G11540.1
Potra2n18c32897.1	0.066293334	0.29814901	0.771155491	0.712599153	Potri.018G039500.1	AT5G11530.1
Potra2n18c32898.1	NA	2.86450839	NA	NA	Potri.018G039400.1	AT3G21360.1

Potra2n18c32899.1						
Potra2n18c32900.1	0.193666786	0.026323678	0.537982287	0.991909603	Potri.018G039300.1	AT4G29000.1
Potra2n18c32901.1	0.212227604	0.125166855	0.146950057	0.900551258	Potri.018G039200.2	AT5G25760.1
Potra2n18c32902.2	-0.251346194	-0.071376563	0.005599035	0.947290893	Potri.018G039100.1	AT4G32760.1
Potra2n18c32903.1	0.149044308	-0.009419983	0.34420219	0.992934677	Potri.018G039000.1	AT5G57850.1
Potra2n18c32904.1	-0.375367885	-0.227498582	0.06683077	0.815767573	Potri.018G038900.1	AT4G11175.1
Potra2n18c32905.1	0.276701436	0.248385007	0.431850644	0.884553748	Potri.018G038700.1	AT1G61470.1
Potra2n18c32906.1	0.007173405	-0.230288485	0.980180708	0.814981587	Potri.018G038600.1	AT4G32750.1
Potra2n18c32907.1	0.225595589	0.074258745	0.086441884	0.937789963	Potri.018G038500.1	AT5G25757.1
Potra2n18c32908.1	0.679869714	0.281796713	0.004267388	0.850010611	Potri.018G038400.1	AT2G20740.1
Potra2n18c32909.1	-0.106374334	-0.027239005	0.605113948	0.9869326	Potri.018G038300.1	AT1G12700.1
Potra2n18c32910.1	0.644420991	0.642522143	0.002585536	0.181966669	Potri.001G025400.1	AT3G14470.1
Potra2n18c32911.1	2.347465856	1.357936012	1.56314E-06	0.495345445	Potri.018G038100.1	AT5G21960.1
Potra2n18c32912.1	0.050326293	-0.08622775	0.809652372	0.928921164	Potri.018G038000.3	AT4G32730.2
Potra2n18c32913.1	0.227578576	0.169558271	0.173009307	0.863928049	Potri.018G037900.1	AT4G32720.2
Potra2n18c32914.1	-0.15417538	-0.103778149	0.275459986	0.934548357	Potri.006G241900.1	AT5G11500.1
Potra2n18c32915.1	0.184645729	-0.00645335	0.181034798	0.996032145	Potri.018G037700.1	AT1G56450.1
Potra2n18c32916.1	-0.9157813	-0.590337506	0.000647875	0.652931023	Potri.018G037500.2	AT2G25737.1
Potra2n18c32917.1	0.053466211	-0.359847314	0.897775066	0.821709751	Potri.018G037300.2	AT5G25752.1
Potra2n18c32918.1	-0.26108765	0.039099882	0.104663978	0.981772014	Potri.018G037200.3	AT5G25630.1
Potra2n18c32919.1	-0.254491258	0.03982442	0.127530798	0.981772014		
Potra2n18c32920.1	0.167603465	-0.119751534	0.361971317	0.910074792	Potri.018G037100.2	AT3G24570.1
Potra2n18c32921.1	0.888584383	-1.05128701	0.482476928	NA	Potri.005G111400.1	AT1G11000.1
Potra2n18c32922.1	0.275812283	-0.012629277	0.235094946	0.995579852	Potri.018G037000.1	AT5G11480.1
Potra2n18c32923.1	0.091225138	-0.142582902	0.861529485	0.953831244		
Potra2n18c32924.1	0.721233284	0.072376971	7.87529E-05	0.956865545	Potri.018G036900.1	AT1G15250.1
Potra2n18c32925.1	-1.249326435	-0.589790105	0.076756835	0.812521522	Potri.018G036800.1	AT5G25620.1
Potra2n18c32926.1	-0.998899995	-1.100786022	0.193112375	0.64562236	Potri.018G036700.1	AT5G25610.1
Potra2n18c32927.6	-0.19660083	0.145576296	0.253725227	0.875559093	Potri.018G036600.1	AT4G32700.2
Potra2n18c32928.1	-0.03070203	0.21838439	0.897829977	0.835819291	Potri.018G036500.4	AT5G11470.1
Potra2n18c32929.1	-0.406803409	-0.055024874	0.048364234	0.956865545	Potri.006G244100.1	AT5G25590.1
Potra2n18c32930.1	0.107937755	-1.704178604	0.973731563	NA	Potri.018G036300.1	AT2G25735.1

Potra2n18c32931.1	NA	NA	NA	NA	Potri.006G244300.2	AT4G32690.1
Potra2n18c32932.1	0.032119847	-0.13545915	0.924329422	0.95671968	Potri.018G036100.1	AT1G61470.1
Potra2n18c32933.1	0.366540124	-0.299685648	0.508965213	0.937687311	Potri.018G038700.1	AT1G61470.1
Potra2n18c32934.1	-0.181056903	-0.099834059	0.132817647	0.922011871	Potri.018G035800.1	AT4G32680.1
Potra2n18c32935.1	-0.2411999	-0.212309113	0.247854209	0.815419191	Potri.018G035700.1	AT1G18950.1
Potra2n18c32936.1	-1.409983009	-1.55593228	NA	NA	Potri.018G035600.1	AT2G25730.1
Potra2n18c32937.1	-0.471672278	-0.542027514	0.201520964	0.68015285	Potri.006G245000.1	AT4G32650.3
Potra2n18c32938.1	-0.471672278	-0.542027514	0.201520964	0.68015285		
Potra2n18c32939.1	-0.080526706	-0.421805252	0.750207128	0.6148597	Potri.018G035400.1	AT2G25690.1
Potra2n18c32940.1	-0.716526481	-1.929769913	0.51262906	NA		
Potra2n18c32941.1					Potri.018G035200.1	AT5G25560.2
Potra2n18c32942.1	-1.691594429	-1.183629511	0.014392541	0.378888039	Potri.018G035100.1	AT4G18670.1
Potra2n18c32943.1	0.347117631	-0.043134851	0.417772494	0.989304496	Potri.018G035000.1	AT4G32630.1
Potra2n18c32944.3	0.308384243	0.397643451	0.051154213	0.623566056	Potri.018G034900.1	AT4G32620.1
Potra2n18c32945.1	0.075334719	-0.064411462	0.456562033	0.936890993	Potri.006G246300.1	AT2G25670.2
Potra2n18c32946.1	-0.034309063	0.068380523	0.842022417	0.937846405	Potri.018G034700.1	AT2G25660.1
Potra2n18c32947.1	-0.462102085	-0.359635272	0.056527547	0.782444319	Potri.018G034600.1	AT5G25530.1
Potra2n18c32948.2	-0.308037695	-0.073608376	0.001207788	0.943483421	Potri.018G034500.1	AT5G25520.2
Potra2n18c32949.1	4.497526037	0.588598414	0.006727366	NA	Potri.006G175050.1	
Potra2n18c32950.1	0.298764908	-0.125940772	0.061907928	0.871720501	Potri.018G034400.2	AT4G32600.1
Potra2n18c32951.1	-0.561761673	-0.383173645	0.009363176	0.701494362	Potri.018G034200.1	AT5G25510.1
Potra2n18c32952.1	-0.855434586	-0.881896434	0.069457138	0.47454828		
Potra2n18c32953.1	-1.259334133	-0.120217921	0.005932871	0.976302215	Potri.018G034300.1	
Potra2n18c32954.3	0.080615451	-0.272632303	0.822754147	0.901070867	Potri.018G034100.1	AT5G11300.1
Potra2n18c32955.1	-0.50968593	-0.345568705	0.116498894	0.807661319	Potri.018G034000.1	
Potra2n18c32956.2	-0.601737036	-0.60170841	0.289189631	0.693459556	Potri.006G247400.1	AT5G11310.1
Potra2n18c32957.1	-0.615370587	-0.2299748	0.000301733	0.80340893	Potri.018G033700.1	AT4G32570.1
Potra2n18c32958.1	-1.903159261	-0.811838566	NA	NA	Potri.018G033600.1	AT1G15550.1
Potra2n18c32959.2	NA	NA	NA	NA	Potri.018G033500.1	AT2G38290.1
Potra2n18c32960.1	-0.781719426	0.262534624	0.06132295	0.897285619	Potri.006G247900.1	AT4G32560.1
Potra2n18c32961.1	NA	NA	NA	NA	Potri.018G033400.1	AT1G52820.1
Potra2n18c32962.1	-0.177836534	-0.021721049	0.191331239	0.984059716	Potri.018G033300.1	AT4G32551.1

Potra2n18c32963.1	-4.215521254	-1.827420245	0.229924406	NA	Potri.018G033200.1	AT5G11320.1
Potra2n18c32964.1					Potri.018G033100.1	
Potra2n18c32965.1	-0.385230924	0.059977718	0.302061768	0.979991162	Potri.018G033000.1	AT2G25620.1
Potra2n18c32966.2	-0.28159302	-0.337230638	0.401762281	0.869327705	Potri.018G032900.1	AT1G15520.1
Potra2n18c32967.1	NA	NA	NA	NA	Potri.018G032700.2	AT4G29250.1
Potra2n18c32968.1	0.059129065	-0.039122895	0.718344685	0.966005919	Potri.006G248700.1	AT4G32530.1
Potra2n18c32969.1	0.10441594	-0.027832048	0.508967654	0.976302215	Potri.018G032500.1	AT5G11330.1
Potra2n18c32970.1	0.42033095	0.179077743	0.000755576	0.851826887	Potri.018G032400.1	AT5G11340.1
Potra2n18c32971.1	0.019909047	0.194912063	0.880312043	0.736149483	Potri.018G032300.10	AT5G11350.1
Potra2n18c32972.1	NA	NA	NA	NA		
Potra2n18c32973.1	0.88588279	-0.280849509	0.012009077	0.813977047	Potri.018G032200.1	AT2G26640.1
Potra2n18c32974.1	-0.511780489	-0.209317733	6.92855E-10	0.367135531	Potri.018G032100.1	AT1G52740.1
Potra2n18c32975.1	-0.587456213	0.436103962	0.344649205	0.865591169	Potri.018G032000.1	AT1G52740.1
Potra2n18c32976.1	-1.939601152	-0.215504582	0.003375417	0.905323467	Potri.018G031901.1	AT4G30380.1
Potra2n18c32977.1	NA	1.363811008	NA	NA		
Potra2n18c32978.1	0.087807798	0.174713016	0.627463451	0.842247476	Potri.018G031800.1	AT5G11380.1
Potra2n18c32979.1	1.426930432	0.529169733	0.551722223	NA	Potri.018G031700.1	AT1G15460.1
Potra2n18c32980.1	1.991102042	-0.458402755	3.05061E-05	0.859813626	Potri.018G031600.1	AT2G25600.1
Potra2n18c32981.1	0.159495001	0.072296833	0.27821586	0.948577865	Potri.018G031400.1	AT5G25450.1
Potra2n18c32982.1	-0.169427809	0.103276827	0.802680794	0.981401592	Potri.018G031100.2	AT5G11420.1
Potra2n18c32983.1	-0.134432882	-0.182643622	0.280211816	0.762561685	Potri.018G030700.1	AT5G55190.1
Potra2n18c32984.1	-1.160340896	0.962923997	NA	NA	Potri.018G030800.1	
Potra2n18c32985.1	-0.230385395	0.221968221	0.169756848	0.833952007	Potri.018G030700.1	AT5G55190.1
Potra2n18c32986.1	-2.453099188	-2.59744079	NA	NA	Potri.018G030600.1	
Potra2n18c32987.1	-0.669246354	0.120227475	4.66801E-06	0.949048812	Potri.006G250600.1	AT4G32440.1
Potra2n18c32988.1	NA	1.363811008	NA	NA	Potri.018G030400.1	AT3G54980.1
Potra2n18c32989.1	0.252125809	0.080799058	0.083047566	0.949485515	Potri.018G030300.2	AT4G32420.1
Potra2n18c32990.1	-0.209745109	-0.308441008	0.433990605	0.79477084	Potri.018G030200.1	AT5G20060.2
Potra2n18c32991.1	0.981384136	-0.987028746	0.014366812	0.446808969	Potri.006G251200.2	AT3G22780.1
Potra2n18c32992.1	0.372656263	0.205792379	0.689349312	0.969421568	Potri.018G029900.1	AT3G15680.1
Potra2n18c32993.3	3.087181886	0.963517741	2.05083E-61	0.477594648	Potri.018G029700.1	AT5G11270.1
Potra2n18c32994.1	0.075038837	-0.214487527	0.796008676	0.932932656	Potri.018G029500.1	AT5G11260.1

Potra2n18c32995.3	0.906643232	0.030789298	0.001215997	0.983944804	Potri.018G029400.1	AT4G32410.1
Potra2n18c32996.1	0.232035921	0.079305125	0.112355254	0.940014069	Potri.018G029200.1	AT4G32400.1
Potra2n18c32997.1	2.497697449	2.238269715	0.124714069	0.72680202	Potri.018G029100.1	AT2G18660.1
Potra2n18c32998.1					Potri.001G155750.1	AT1G21280.1
Potra2n18c32999.1	1.268357002	1.46366011	0.266709036	0.68282906		
Potra2n18c33000.1	-0.943333911	-0.257985194	0.388056962	0.974756511		
Potra2n18c33001.1	-0.5407891	-0.244276523	0.683368412	0.971828097	Potri.018G029000.1	AT1G18485.1
Potra2n18c33002.1	0.344434636	-0.094251692	0.028291077	0.929239331	Potri.018G028900.1	AT4G32390.1
Potra2n18c33003.1	6.856648153	4.572620269	8.13687E-08	NA		
Potra2n18c33004.1	5.361516291	3.243512215	1.37299E-18	0.188881025	Potri.018G028800.1	AT5G06740.1
Potra2n18c33005.1	-0.338931136	-0.065128351	0.002858651	0.948834049	Potri.018G028600.1	AT5G11170.1
Potra2n18c33006.2	-0.438510619	-0.251894119	0.034836002	0.813731863	Potri.018G028500.2	AT3G47650.1
Potra2n18c33007.1	0.448385686	-0.23237024	0.386594539	0.954559968		
Potra2n18c33008.1	0.302863833	-0.257358445	0.55335908	0.946464685	Potri.018G028300.1	AT1G68620.1
Potra2n18c33009.1	-0.777372256	0.01086128	1.46088E-09	0.995579852	Potri.018G028200.1	AT2G26660.1
Potra2n18c33010.1	0.771892157	0.231001698	1.34739E-10	0.848005416	Potri.018G028100.1	AT3G49500.1
Potra2n18c33011.1	-1.245783249	-0.933524293	0.056428956	0.616130282	Potri.018G028000.1	AT5G25390.2
Potra2n18c33012.1	-2.479597942	-4.071023872	0.325450117	NA	Potri.018G027900.1	AT3G15730.1
Potra2n18c33013.1	NA	NA	NA	NA		
Potra2n18c33014.1	-0.15235798	-0.027639099	0.14003975	0.977227089	Potri.018G027800.1	AT5G25360.2
Potra2n18c33015.1	0.059577113	-0.023519087	0.865510472	0.979719961	Potri.018G027700.1	AT2G25490.1
Potra2n18c33016.1	0.013836887	-0.380344976	0.975847622	0.70570456	Potri.018G027600.2	AT1G30380.1
Potra2n18c33017.1	-0.253419417	0.015733262	0.027721116	0.992934677	Potri.018G027500.1	AT4G32330.1
Potra2n18c33018.1	-0.804663101	-0.03831885	0.071383984	0.993589888	Potri.018G027400.1	
Potra2n18c33019.1	0.658220277	0.036638271	0.004611261	0.988154814	Potri.018G027300.1	AT4G32300.1
Potra2n18c33020.1	1.627898524	0.202985854	NA	0.931774514	Potri.018G027200.1	AT3G11760.1
Potra2n18c33021.1	-1.04070216	-0.532658973	5.81968E-09	0.367131542	Potri.018G027100.1	AT4G32295.1
Potra2n18c33022.1	-0.319354089	-0.235549755	0.151159017	0.790748053	Potri.018G027001.1	AT3G19720.1
Potra2n18c33023.2	-0.569504589	-0.321706585	0.000420005	0.724504724	Potri.018G026900.1	AT2G25430.1
Potra2n18c33024.1	0.163036659	-0.13595106	0.71837676	0.949840005	Potri.018G026800.2	AT1G56130.1
Potra2n18c33025.1	-0.042694299	0.117746167	0.88854919	0.914554682	Potri.018G026700.1	AT4G35530.1
Potra2n18c33026.1	1.504515657	0.637664109	7.72448E-13	0.32064055	Potri.006G255400.3	AT4G32272.1

Potra2n18c33027.1	-1.154490013	-0.550062163	2.28476E-13	0.00826094	Potri.018G026500.1	AT4G32260.1
Potra2n18c33028.1	-0.563722366	-0.133072233	0.000119686	0.886633917	Potri.018G026400.2	AT4G32250.2
Potra2n18c33029.1	-0.588987024	-0.597943565	0.543266595	0.876740025	Potri.018G026300.1	AT4G32190.1
Potra2n18c33030.1	-0.421034177	-0.601285507	0.787191921	NA		
Potra2n18c33031.1	-0.621644743	-0.301900135	0.001036518	0.752192501	Potri.018G026200.2	AT5G11160.1
Potra2n18c33032.4	-0.318942508	-0.035192615	0.001842836	0.974367223	Potri.018G026000.1	AT4G32160.1
Potra2n18c33033.1	-0.063168796	-0.963388826	0.925338699	0.634943469	Potri.018G025900.1	AT2G45180.1
Potra2n18c33034.1	-0.253805405	-0.079828972	0.056627812	0.92978159	Potri.018G025800.1	AT5G11150.1
Potra2n18c33035.1	-2.252058597	-3.04653375	0.196311725	NA	Potri.018G025700.1	AT1G52520.1
Potra2n18c33036.1	0.357539755	0.182158144	6.248E-05	0.75939388		
Potra2n18c33037.1	-0.36997997	0.421536229	0.776537996	0.917746944	Potri.018G025400.2	AT5G25310.1
Potra2n18c33038.1						
Potra2n18c33039.1	NA	0.674963689	NA	NA	Potri.018G025200.2	AT5G11130.1
Potra2n18c33040.1	1.233058501	0.172328152	0.017200825	0.970442612	Potri.018G025100.1	AT5G20280.1
Potra2n18c33041.1	-0.718540042	0.193687796	0.012696273	0.930610374	Potri.018G025000.2	AT5G11100.1
Potra2n18c33042.1	NA	NA	NA	NA	Potri.T148400.2	AT2G28690.1
Potra2n18c33043.1	-0.447060174	-0.245102776	0.001094727	0.814001428	Potri.018G024800.16	AT5G34850.1
Potra2n18c33044.1	NA	NA	NA	NA	Potri.T076201.1	AT5G45400.1
Potra2n18c33045.1	NA	NA	NA	NA		
Potra2n18c33046.1	NA	NA	NA	NA	Potri.008G220300.1	
Potra2n18c33047.1	-0.556354042	0.041927842	0.00025961	0.978203092	Potri.018G024700.1	AT4G32140.1
Potra2n18c33048.1	0.022396949	0.083396166	0.882778783	0.903398621	Potri.018G024600.1	AT2G25310.1
Potra2n18c33049.1	-0.525599301	-0.229998371	0.039869974	0.88661378	Potri.018G024500.1	AT4G32120.1
Potra2n18c33050.3	0.186787074	0.178087845	0.649533468	0.954419252	Potri.018G024400.3	AT5G38690.1
Potra2n18c33051.1	0.48305545	0.072242406	0.006497892	0.966228173	Potri.018G024300.1	AT2G31170.2
Potra2n18c33052.1	1.063130581	0.421647233	2.53836E-06	0.765390763	Potri.018G024200.1	AT5G34940.2
Potra2n18c33053.1	-0.013936798	0.006073024	0.950127264	0.996281171	Potri.018G024000.1	
Potra2n18c33054.1	0.33783319	-0.015907457	0.267859949	0.995261215	Potri.018G023900.4	AT1G80490.1
Potra2n18c33055.1	0.59773819	0.230566948	0.006741761	0.837645998	Potri.018G023800.1	AT1G80490.2
Potra2n18c33056.1	-0.191449415	0.000451287	0.045933438	0.999737353	Potri.018G023700.1	AT2G25280.1
Potra2n18c33057.1	-0.002302487	0.121775939	0.994166747	0.953831244	Potri.018G023600.1	AT2G20142.1
Potra2n18c33058.1	-0.135075234	-0.105982219	0.65235741	0.948577865	Potri.018G023500.1	AT2G25290.3

Potra2n18c33059.1	0.198521441	0.170605932	0.01910382	0.736203664	Potri.018G023400.1	AT4G32060.1
Potra2n18c33060.1	-0.506335129	-0.156449001	0.108861751	0.938501048	Potri.018G023300.1	AT5G11090.1
Potra2n18c33061.4	-1.263516507	-0.48473498	5.60391E-06	0.747036979	Potri.018G023200.1	AT2G25270.1
Potra2n18c33062.1	-0.186236608	-0.107401121	0.372351239	0.912777843	Potri.018G023100.2	AT5G25265.1
Potra2n18c33063.1	0.082056263	0.399113675	0.761154945	0.636664382	Potri.018G023000.1	AT2G31650.1
Potra2n18c33064.1	4.263628288	0.468126709	0.000111499	NA	Potri.018G022900.1	AT5G25260.1
Potra2n18c33065.1	0.372613676	-0.016493105	0.0135189	0.989878071	Potri.018G022800.1	AT4G32050.1
Potra2n18c33066.1	-0.381844516	-0.416942999	0.029910969	0.692047169	Potri.018G022700.3	AT5G25220.1
Potra2n18c33067.1	-0.23285904	-0.155250666	0.766677609	0.981702181	Potri.018G022600.1	
Potra2n18c33068.1	-0.137949537	-0.051530308	0.397618609	0.945254452	Potri.018G022500.1	AT5G11040.1
Potra2n18c33069.1					Potri.018G019500.1	AT1G69440.1
Potra2n18c33070.1	-0.725434648	-0.328867145	3.18604E-07	0.277578139	Potri.018G022400.1	AT4G32020.1
Potra2n18c33071.1	0.283920745	-0.087542684	0.290388865	0.949847686	Potri.018G022300.1	AT4G32000.2
Potra2n18c33072.1	-0.437176912	-0.138308197	0.054063037	0.934579595	Potri.018G022200.2	AT4G31990.2
Potra2n18c33073.1	0.341790606	-0.076991963	0.085023142	0.963557834	Potri.006G188500.1	AT4G31985.1
Potra2n18c33074.1	-0.637934825	-1.507841271	NA	NA	Potri.018G022000.1	AT2G18950.1
Potra2n18c33075.1	1.446966732	-0.470411588	0.000585475	0.894019146	Potri.018G021900.1	AT5G25190.1
Potra2n18c33076.1	0.129289084	-0.071276138	0.734946434	0.974751051	Potri.018G021800.1	AT5G11000.1
Potra2n18c33077.1	-0.196055917	0.133831579	0.091753717	0.882920752	Potri.018G021700.1	AT5G25170.1
Potra2n18c33078.1	-0.039725701	-0.39079572	0.891764444	0.511337291	Potri.018G021600.1	AT4G31930.1
Potra2n18c33079.1	4.64235759	1.010865285	7.45562E-27	0.777674368	Potri.018G021400.1	AT5G10970.1
Potra2n18c33080.1	0.108894745	0.199774152	0.30408942	0.724604666	Potri.018G021300.3	AT2G25180.1
Potra2n18c33081.2	-0.074801185	0.180056187	0.488306481	0.776048341	Potri.018G021100.1	AT2G25170.1
Potra2n18c33082.1	0.133050577	-0.028899871	0.362058406	0.977033123	Potri.018G021000.1	AT5G54830.1
Potra2n18c33083.1	0.060809539	-0.01917622	0.749745908	0.983944804	Potri.018G020900.1	AT2G32070.1
Potra2n18c33084.1	2.082612078	0.932564434	0.005148444	0.820210443		
Potra2n18c33085.1	-0.610746198	0.190039172	0.033305497	0.919424623	Potri.018G020800.1	AT1G69360.1
Potra2n18c33086.1	0.717228184	-0.016606711	3.05519E-05	0.995005375	Potri.018G020700.2	AT2G25110.1
Potra2n18c33087.1	1.84585494	0.885181785	2.9372E-19	0.236854032	Potri.018G020600.1	AT2G24270.4
Potra2n18c33088.1	0.03196899	0.037358157	0.708872229	0.977559521	Potri.018G020400.2	AT4G31880.1
Potra2n18c33089.1	-0.031443481	0.508192982	0.918746302	0.742764478	Potri.018G020300.1	
Potra2n18c33090.1	-0.076580554	-0.033552001	0.604384146	0.973948905	Potri.018G020200.2	AT5G25150.1

Potra2n18c33091.1	-0.012355397	0.061130729	0.96818094	0.974628351	Potri.018G019900.1	AT5G10930.1
Potra2n18c33092.1	3.802897695	2.17756357	1.2292E-19	0.200085324	Potri.018G019800.1	AT1G80840.1
Potra2n18c33093.1	6.998752103	4.253300761	2.3817E-21	0.004284785	Potri.018G019700.1	AT1G80840.1
Potra2n18c33094.1	0.389504994	0.054608026	0.035532562	0.973948905	Potri.018G019600.1	AT2G25050.1
Potra2n18c33095.1	-0.423236734	-0.295561999	0.085832225	0.796890714	Potri.018G019400.1	AT4G31805.1
Potra2n18c33096.2	1.084541313	-0.017475318	0.020438049	0.995194098	Potri.018G019300.2	AT5G10870.1
Potra2n18c33097.1	0.743454261	-1.465929376	NA	NA	Potri.018G019200.1	AT3G23780.1
Potra2n18c33098.1	-0.208750393	0.042193751	0.072125563	0.974275337	Potri.018G019100.1	AT3G23780.1
Potra2n18c33099.2	0.157061152	0.394676388	0.794952803	0.886059841	Potri.018G019000.1	AT4G30935.1
Potra2n18c33100.1	-0.168657094	0.000677153	0.478836282	0.999771727	Potri.018G018900.1	AT5G66360.2
Potra2n18c33101.1	0.445715536	0.06497135	0.000803612	0.971537796	Potri.018G018800.1	AT4G31810.1
Potra2n18c33102.1	0.813045173	0.233283012	0.006211516	0.911339141	Potri.018G018700.1	
Potra2n18c33103.1	-1.209082536	-0.064663747	7.39169E-21	0.974275337	Potri.018G018600.2	AT4G31820.1
Potra2n18c33104.1	NA	NA	NA	NA	Potri.018G018500.1	AT5G22860.1
Potra2n18c33105.1	-0.142661825	-0.255032509	0.520027814	0.86267889	Potri.018G018400.1	AT5G10840.1
Potra2n18c33106.1	-0.074528266	-0.649974804	0.92224852	0.692084498	Potri.018G018300.1	AT4G31830.1
Potra2n18c33107.1	-0.519369222	-0.744088843	0.089328518	0.643173103	Potri.018G018200.1	AT2G25060.1
Potra2n18c33108.1	-0.09560167	-0.410089372	0.713254144	0.694675633	Potri.018G018100.1	AT5G25080.1
Potra2n18c33109.1					Potri.018G018000.1	AT5G42955.1
Potra2n18c33110.1					Potri.018G018000.1	AT5G42955.1
Potra2n18c33111.1	-0.328002602	-0.082911192	0.009900629	0.959881296	Potri.006G265100.1	AT4G31860.1
Potra2n18c33112.1	0.321857379	-0.083112282	0.020072405	0.935751704	Potri.018G017600.1	AT5G57870.2
Potra2n18c33113.1	0.34088355	0.208093949	6.49748E-06	0.639292153	Potri.018G017200.2	AT5G25060.1
Potra2n18c33114.1	-0.021049354	-0.081684768	0.905171457	0.943819961	Potri.018G017100.1	AT5G25050.1
Potra2n18c33115.1	0.174687453	-0.096969624	0.29632759	0.930598202	Potri.018G017000.1	AT5G10790.1
Potra2n18c33116.1	0.080215365	0.05486693	0.672522957	0.967129771	Potri.018G016800.1	AT5G10780.1
Potra2n18c33117.4	0.463756635	0.354245809	0.756060436	0.953831244	Potri.018G016700.1	
Potra2n18c33118.1	-0.271505435	0.009420121	0.339753101	0.996292992	Potri.018G016600.1	AT4G31780.2
Potra2n18c33119.1	-0.283295192	-0.432168263	0.298268189	0.637188449	Potri.018G016500.1	AT1G80450.1
Potra2n18c33120.1	-0.563438974	-0.271999508	0.064333296	0.797028313	Potri.018G016400.1	AT2G24860.1
Potra2n18c33121.1	-0.127873008	-0.178454622	0.579977309	0.855245045	Potri.018G016300.1	AT2G24970.1
Potra2n18c33122.1	0.250735439	0.14320514	0.024302532	0.844120859	Potri.018G016200.1	AT2G24830.1

Potra2n18c33123.1	0.888360024	0.493719799	6.75861E-13	0.407558142	Potri.018G016000.2	AT2G02250.1
Potra2n18c33124.1	-0.315306699	-0.076392534	0.057271043	0.953768643	Potri.018G015800.2	AT2G02240.1
Potra2n18c33125.1	-0.333658303	-0.076344896	0.0371859	0.953831244	Potri.018G015700.1	AT2G24820.1
Potra2n18c33126.1	0.540783138	0.208453527	4.54008E-05	0.689693427	Potri.018G015300.2	AT4G31770.1
Potra2n18c33127.1	0.418406539	0.150479372	0.000217026	0.706204031	Potri.018G015200.1	AT5G27640.1
Potra2n18c33128.1	-2.65051824	-1.208117824	NA	NA		
Potra2n18c33129.1	-0.037845394	0.092715861	0.919016397	0.973347974	Potri.018G015100.1	AT5G10770.1
Potra2n18c33130.1	-2.578176003	-0.763817967	0.336445189	NA	Potri.018G015000.1	AT5G61412.1
Potra2n18c33131.1	3.219950245	NA	NA	NA	Potri.006G250200.1	AT1G52360.1
Potra2n18c33132.1	-0.189593689	-0.164058537	0.60560543	0.924309478	Potri.018G014300.1	AT5G10750.1
Potra2n18c33133.1	-0.218495408	-0.212744069	0.186840469	0.676711874	Potri.018G014100.3	
Potra2n18c33134.2	-0.053041158	0.039719053	0.761069366	0.974275337	Potri.018G014000.1	AT5G24970.1
Potra2n18c33135.1	-0.024417876	-0.067700976	0.842429351	0.935474933	Potri.018G013900.1	AT4G31750.1
Potra2n18c33136.1	-0.300547191	-0.019917185	0.012604541	0.990927357	Potri.018G013800.1	AT5G24930.1
Potra2n18c33137.1	-0.328679592	-0.147061614	0.017629517	0.843683654	Potri.018G013700.1	AT2G24765.1
Potra2n18c33138.1	-1.948071914	-0.509344397	1.90131E-10	0.335923144	Potri.018G013600.1	AT5G57685.1
Potra2n18c33139.1	0.18901718	-0.037502853	0.19968902	0.975578749	Potri.018G013400.1	AT5G10730.1
Potra2n18c33140.1	0.393073537	0.025609452	0.010609185	0.98177802	Potri.018G013300.1	AT4G31720.1
Potra2n18c33141.1	-0.466641972	-0.281614885	0.399930986	0.885459527	Potri.018G013200.1	AT2G29110.1
Potra2n18c33142.1					Potri.018G013000.1	AT2G29120.1
Potra2n18c33143.1	NA	3.025856371	NA	NA	Potri.018G013000.1	AT2G29120.1
Potra2n18c33144.3	5.320674076	NA	0.002387224	NA	Potri.018G010700.1	AT2G29110.1
Potra2n18c33145.1	NA	NA	NA	NA	Potri.018G117100.1	
Potra2n18c33146.1	2.353578456	1.693898839	0.000721698	0.216891326	Potri.018G010700.1	AT2G29110.1
Potra2n18c33147.1	-1.336737103	0.123771946	NA	NA		
Potra2n18c33148.1	1.64774763	NA	NA	NA	Potri.018G013200.1	AT2G29110.1
Potra2n18c33149.1	0.943350674	-0.046370319	NA	NA		
Potra2n18c33150.1	4.88617619	3.079264881	0.032079771	NA	Potri.018G010700.1	AT2G29110.1
Potra2n18c33151.1	NA	2.490806657	NA	NA		
Potra2n18c33152.1	-0.476244491	0.458250215	0.637947989	0.91233528		
Potra2n18c33153.1	-1.35927387	2.398749263	NA	NA	Potri.018G010600.1	AT5G27100.1
Potra2n18c33154.1	-0.61910233	2.657660108	NA	NA		

Potra2n18c33155.1	NA	NA	NA	NA		
Potra2n18c33156.1	3.885939201	1.642687443	0.076955028	NA	Potri.018G010700.1	AT2G29110.1
Potra2n18c33157.2	-0.240028516	-0.119674267	0.823120935	0.987226805	Potri.018G010500.1	
Potra2n18c33158.1	1.568085224	1.022524372	0.009086635	0.663309616	Potri.018G012100.1	AT2G29120.1
Potra2n18c33159.1	NA	1.381953902	NA	NA		
Potra2n18c33160.1					Potri.018G012100.1	AT2G29120.1
Potra2n18c33161.1					Potri.018G009900.1	AT2G29110.1
Potra2n18c33162.1	2.063471427	NA	NA	NA	Potri.018G012000.1	AT2G29120.1
Potra2n18c33163.1	NA	NA	NA	NA	Potri.018G009900.1	AT2G29110.1
Potra2n18c33164.1	2.772423942	1.23073919	0.171433507	NA	Potri.018G012300.1	AT2G29110.1
Potra2n18c33165.1	3.453304894	1.231417778	3.24066E-05	0.872201371	Potri.018G011500.1	AT2G29110.1
Potra2n18c33166.4	-1.858700944	-0.099686453	0.625088023	NA	Potri.018G009700.1	AT5G10720.1
Potra2n18c33167.1	NA	0.674963689	NA	NA	Potri.018G009650.1	AT3G23280.1
Potra2n18c33168.1	-0.106281674	-0.161256342	0.461118341	0.848131816	Potri.018G009500.1	AT2G24640.1
Potra2n18c33169.1	-0.016894506	0.061338722	0.939152929	0.953608209	Potri.006G270700.2	AT4G31600.1
Potra2n18c33170.1	NA	1.363811008	NA	NA		
Potra2n18c33171.1	-0.217019691	0.071226042	0.350427618	0.971879168	Potri.018G009300.1	AT4G31590.1
Potra2n18c33172.1	0.345199316	0.678654441	0.379020968	0.591292872	Potri.018G009200.1	AT2G24610.1
Potra2n18c33173.1	0.010096008	0.08002978	0.961589514	0.948002367	Potri.018G009100.1	AT4G31580.2
Potra2n18c33174.1	-3.66284176	-0.437126459	0.102183245	NA	Potri.018G009000.1	AT2G15490.3
Potra2n18c33175.1	1.664677056	NA	NA	NA	Potri.018G008900.1	AT2G15490.3
Potra2n18c33176.2	0.346378816	0.37424324	0.042637385	0.360181553	Potri.018G008600.1	AT4G31570.1
Potra2n18c33177.2	0.998001826	0.467206081	5.16642E-10	0.381727866	Potri.018G008700.1	AT2G24580.1
Potra2n18c33178.1	3.610633119	1.74506435	1.66688E-05	0.260170161	Potri.018G008500.1	AT4G31550.2
Potra2n18c33179.1	-0.284972067	0.213058128	0.257517011	0.909450222	Potri.018G008400.1	AT4G31540.1
Potra2n18c33180.1	1.248512928	0.609877795	0.421341838	NA	Potri.018G004600.1	AT3G14470.1
Potra2n18c33181.1	0.675175123	-0.831474715	0.654696744	0.926235628	Potri.018G008300.1	AT1G13480.1
Potra2n18c33182.1	-0.258413118	0.048382747	0.153271486	0.983446961	Potri.018G008200.2	AT5G24890.1
Potra2n18c33183.1	1.459962178	NA	NA	NA	Potri.018G008100.1	AT1G29670.1
Potra2n18c33184.1	-0.132243515	0.224759343	0.46332565	0.829597923	Potri.018G008000.1	AT5G10700.1
Potra2n18c33185.1	NA	1.571529889	NA	NA	Potri.018G007900.1	AT2G04920.1
Potra2n18c33186.1	-0.181219637	-0.114972267	0.385101698	0.950453646	Potri.018G007400.1	AT4G31480.1

Potra2n18c33187.1	1.957415907	0.180973804	0.426547924	NA	Potri.018G007201.1	
Potra2n18c33188.1	-0.359588173	0.0976497	0.026969874	0.957525726	Potri.018G007100.1	AT2G24540.1
Potra2n18c33189.1	-0.356165796	0.477612459	0.81069053	0.940703488	Potri.018G007000.1	AT4G31470.1
Potra2n18c33190.1	0.072871751	-0.138899777	0.780907958	0.901251375	Potri.018G006900.1	AT4G31460.1
Potra2n18c33191.1	0.568262255	-0.717203965	0.441156098	0.862830524	Potri.018G006800.1	AT5G57510.1
Potra2n18c33192.1	NA	NA	NA	NA	Potri.018G006700.1	AT2G24490.2
Potra2n18c33193.2	0.82357019	NA	NA	NA	Potri.018G006600.1	AT4G31390.1
Potra2n18c33194.1	0.04924616	0.066486673	0.763707554	0.954559968	Potri.018G006500.1	AT5G10630.2
Potra2n18c33195.1	0.213932666	0.165837879	0.046301314	0.562306981	Potri.018G006400.3	AT4G31410.1
Potra2n18c33196.1	3.131409264	2.155927297	0.000824746	0.47454828	Potri.018G006300.1	AT4G31420.1
Potra2n18c33197.1	-0.216320589	0.072398554	0.103828899	0.95671968	Potri.018G006200.1	AT4G31430.2
Potra2n18c33198.1	-0.205812143	-0.084516641	0.09627427	0.937687311	Potri.018G006100.1	AT1G22920.1
Potra2n18c33199.1	-1.099462308	-0.167697902	1.83664E-05	0.938653363	Potri.006G275000.1	AT2G24520.1
Potra2n18c33200.2	-0.105131756	-0.090591604	0.503414875	0.896937427	Potri.018G005900.1	AT5G10710.2
Potra2n18c33201.1	0.521281141	0.317346908	0.000490989	0.41540836	Potri.018G005800.2	AT1G52600.1
Potra2n18c33202.1	-0.052578138	-0.182324554	0.808812326	0.840457474	Potri.018G005700.1	AT2G24530.1
Potra2n18c33203.1	0.015662361	-0.231710528	0.972909363	0.9045874		
Potra2n18c33204.1	-1.14279598	0.814677771	NA	NA		
Potra2n18c33205.1	-0.383122394	0.465625952	NA	NA		
Potra2n18c33206.2	-0.901941031	-0.303359219	0.000195568	0.743612955	Potri.018G005600.1	AT5G24870.1
Potra2n18c33207.1	-3.277145882	-0.789509679	0.158610221	NA	Potri.018G005500.1	AT5G10660.1
Potra2n18c33208.1	NA	NA	NA	NA	Potri.016G028900.2	AT2G40840.1
Potra2n18c33209.1	5.977265845	2.892658772	0.000116968	NA	Potri.018G005300.1	AT1G22640.1
Potra2n18c33210.1					Potri.018G005200.1	AT5G24860.1
Potra2n18c33211.1	4.58153377	1.166926133	0.011044651	NA		
Potra2n18c33212.1	4.252110639	1.190574929	0.01268836	NA	Potri.T130300.1	AT2G24450.1
Potra2n18c33213.1	0.502240604	-0.494759695	0.189267555	0.777553704	Potri.T130400.1	AT4G31360.1
Potra2n18c33214.1	-1.328951947	0.821573783	NA	NA	Potri.018G004600.1	AT3G14470.1
Potra2n18c33215.1	-0.052385634	0.107751766	0.906825988	0.976302215	Potri.006G272900.1	AT4G31540.1
Potra2n18c33216.1	1.517671607	1.394613675	NA	NA	Potri.018G008300.1	AT1G13480.1
Potra2n18c33217.1	-1.163205585	0.275784	0.325287932	0.972159498	Potri.018G003200.1	AT3G14470.1
Potra2n18c33218.1	-0.748235217	0.232532574	0.300133389	0.95671968		

Potra2n18c33219.1	1.664677056	2.490806657	NA	NA	Potri.018G003300.1	AT1G10330.1
Potra2n18c33220.1	-0.690761499	0.375483713	0.530401213	0.930059926	Potri.018G003400.1	AT5G24810.1
Potra2n18c33221.1	-0.15639143	-0.008771256	0.249622461	0.992934677	Potri.T130900.3	AT5G24830.1
Potra2n18c33222.6	-1.35927387	1.044491047	NA	NA	Potri.018G004100.1	AT5G65940.1
Potra2n18c33223.1	-0.232649476	-0.134542176	0.197279318	0.89438295	Potri.018G004000.1	AT4G26000.1
Potra2n18c33224.1	0.314643978	0.225622688	0.176774792	0.836066102	Potri.018G003900.1	AT5G24840.1
Potra2n18c33225.1	4.364993351	NA	0.028118873	NA	Potri.018G003800.1	AT2G24430.1
Potra2n18c33226.1	-0.174421928	-0.165646641	0.070003997	0.889106941	Potri.018G003700.10	AT4G31340.1
Potra2n18c33227.1	-4.495400603	-1.194164459	0.062105482	NA		
Potra2n18c33228.1	0.464484177	0.193529076	0.002569082	0.71703421	Potri.018G003400.1	AT5G24810.1
Potra2n18c33229.1	-1.099214089	-0.173267243	0.645036087	NA	Potri.018G004900.2	AT3G14470.1
Potra2n18c33230.1	-1.323133003	2.612948399	NA	NA		
Potra2n18c33231.1	NA	2.690879854	NA	NA		
Potra2n18c33232.1	-0.129666369	0.394909885	0.566640698	0.846680547	Potri.018G003100.1	AT4G16130.1
Potra2n18c33233.1	0.263794332	0.135714949	0.506679571	0.946464685	Potri.018G003000.1	AT2G24390.1
Potra2n18c33234.1	1.10339761	0.319584537	0.023092999	0.824905557		
Potra2n18c33235.2	1.141992022	0.376478817	0.009438313	0.706051231	Potri.006G279000.1	AT4G31290.1
Potra2n18c33236.1	0.132019321	-0.165189204	0.75881589	0.917751882	Potri.018G002801.1	AT4G31270.1
Potra2n18c33237.1	0.672710074	-0.428018778	0.400098031	0.936549944	Potri.018G002700.1	AT2G21080.1
Potra2n18c33238.1	1.664677056	2.906556465	NA	NA	Potri.018G002600.1	AT4G31250.1
Potra2n18c33239.1	2.873514836	-0.767708831	0.389224559	NA	Potri.018G002400.1	AT2G24370.1
Potra2n18c33240.1	0.170976644	0.080516598	0.1975943	0.926808461	Potri.018G002300.1	AT1G17370.1
Potra2n18c33241.1	0.058588307	-0.086263803	0.713492206	0.904812396	Potri.018G002000.1	AT4G31200.3
Potra2n18c33242.2	0.167869563	-0.036325211	0.055613417	0.953768643	Potri.018G001800.1	AT4G31170.1
Potra2n18c33243.1	3.142335757	1.044491047	0.101826195	NA	Potri.018G001700.1	
Potra2n18c33244.2	1.173783856	-0.227235873	0.002146673	0.951726178	Potri.018G001600.1	AT3G57560.1
Potra2n18c33245.1	4.137728496	4.471727657	0.043218547	NA	Potri.018G001550.1	AT5G27660.1
Potra2n18c33246.3	NA	1.363811008	NA	NA	Potri.018G001500.2	AT5G27660.1
Potra2n18c33247.1	-0.387060052	0.518448704	0.85488664	NA	Potri.018G001301.1	AT5G27660.1
Potra2n18c33248.1	NA	NA	NA	NA	Potri.018G001100.2	AT3G27925.1
Potra2n18c33249.1	-0.154805514	0.101201014	0.365344589	0.924309478	Potri.018G001000.3	AT4G31150.1
Potra2n18c33250.1	0.773754377	0.701130138	0.00014419	0.228343054	Potri.018G000900.1	AT4G31140.1

Potra2n18c33251.1	-0.140773268	0.022156243	0.473988737	0.985393296	Potri.003G221400.1	AT5G64150.1
Potra2n18c33252.1	0.852316007	-0.038753621	0.174183366	0.995005375	Potri.018G000700.1	AT3G53440.2
Potra2n18c33253.1	1.707348758	1.136849803	0.007939138	0.564195735	Potri.018G000700.1	AT3G53440.2
Potra2n18c33254.1	0.014558221	0.153505901	0.932322556	0.912777843	Potri.018G000600.1	AT4G31130.1
Potra2n18c33255.1	0.305838521	0.11597214	0.071469266	0.912666272	Potri.018G000500.2	AT4G31120.1
Potra2n18c33256.1	-0.389777901	0.140614428	0.056916163	0.949966783	Potri.005G002700.1	AT5G26610.2
Potra2n18c33257.1	1.64774763	0.674963689	NA	NA	Potri.008G001900.1	AT2G01050.1
Potra2n18c33258.1						
Potra2n191s34818.1	1.545832463	2.86450839	NA	NA	Potri.014G080100.3	AT1G01800.1
Potra2n191s34819.1	1.769290221	0.604980999	4.1358E-15	0.408298024		
Potra2n191s34820.1	0.751808417	0.166942239	2.66018E-07	0.907073354	Potri.014G079900.1	AT4G00570.1
Potra2n191s34821.1	0.157600283	0.121354701	0.247719922	0.917746944	Potri.002G155800.1	AT4G00585.1
Potra2n191s34822.1	-0.514325918	0.033231224	0.07578758	0.99114586	Potri.014G079700.1	AT4G00560.1
Potra2n192s34823.1	-0.057606867	0.010641045	0.866015522	0.996154416	Potri.012G054400.3	AT1G18370.1
Potra2n192s34824.1	-0.005317872	0.576944824	0.996365144	0.857189843	Potri.004G101600.1	
Potra2n192s34825.1	0.161205854	0.305423488	0.884055982	0.95671968	Potri.004G101500.1	AT3G02530.1
Potra2n192s34826.1	3.241532158	1.430544412	2.91342E-05	0.778013962	Potri.012G054550.1	AT5G38260.1
Potra2n192s34827.1	-0.973505359	0.220983908	0.427633197	0.978646026	Potri.004G101600.1	
Potra2n192s34828.1	-0.168586091	-0.451222665	0.90253824	0.927547456	Potri.012G054400.3	AT1G18370.1
Potra2n192s34829.1	0.196329423	1.563612841	0.931559055	0.849790859	Potri.004G101600.1	
Potra2n192s34830.1	0.083610351	0.378134033	0.9582386	0.947290893	Potri.004G101500.1	AT3G02530.1
Potra2n192s34831.1	-0.475904657	-0.654025288	0.858899442	NA	Potri.012G054400.3	AT1G18370.1
Potra2n192s34832.1	2.823874081	0.976091981	0.062105482	NA	Potri.012G054800.1	AT5G38260.1
Potra2n192s34833.4	0.173457727	1.143590275	0.870781733	0.576228147	Potri.004G101600.1	
Potra2n192s34834.1	1.888069917	-0.834601298	0.515544479	NA	Potri.012G054550.1	AT5G38260.1
Potra2n192s34835.1	2.360607259	0.471291684	7.18397E-08	0.896937427	Potri.012G054800.1	AT5G38260.1
Potra2n192s34836.1	-0.210274024	0.669792685	0.700087446	0.803948068	Potri.004G101600.1	
Potra2n193s34837.1	5.450655549	0.797607471	1.5073E-05	NA		
Potra2n193s34838.1	4.139533197	2.416788965	3.78363E-12	0.196885879	Potri.T065100.2	AT1G66920.2
Potra2n193s34839.1	4.752733045	2.888510034	9.22853E-18	0.109095357		
Potra2n193s34840.1	2.001261811	2.10346733	0.56624232	NA	Potri.017G118600.1	AT5G38200.1
Potra2n196s34841.1	1.110365483	1.575540292	0.640837297	NA	Potri.009G081500.1	AT5G64510.1

Potra2n196s34842.1	-1.317292482	1.084684214	NA	NA	Potri.001G287300.1	
Potra2n196s34843.1	1.525862217	0.012625168	0.008260834	0.996032145	Potri.001G287200.1	AT4G37850.1
Potra2n196s34844.1	0.581116685	-0.186527734	0.001347673	0.903496619	Potri.001G287100.1	AT4G33670.1
Potra2n197s34845.1	-0.340417141	-1.102152195	0.692408572	0.719104446	Potri.T040800.3	AT1G07390.3
Potra2n197s34846.1	-1.409983009	-1.55593228	NA	NA	Potri.001G063400.1	
Potra2n197s34847.1	0.82357019	1.871975723	NA	NA		
Potra2n197s34848.1	0.160817622	0.117481766	0.147772676	0.851395707	Potri.003G063500.2	AT1G72660.1
Potra2n197s34849.1	-0.322640618	0.00786779	0.005532349	0.995005375	Potri.003G063700.2	AT1G72650.1
Potra2n197s34850.1	-0.80857826	-1.0281993	0.03329391	0.117957555	Potri.003G063800.2	AT1G72640.1
Potra2n197s34851.1	2.556135291	NA	NA	NA		
Potra2n197s34852.1	NA	NA	NA	NA	Potri.008G220300.1	
Potra2n197s34853.1	0.043842385	0.02031549	0.718611108	0.983944804	Potri.003G064000.6	AT1G17450.2
Potra2n197s34854.1	-0.004699905	-0.369529824	0.986838707	0.694611009	Potri.003G063900.1	AT1G17455.1
Potra2n197s34855.1	0.346335852	0.179700045	0.002167481	0.720581396	Potri.003G064100.2	AT1G17440.2
Potra2n197s34856.1	-0.438099398	0.036467094	0.000573514	0.975578749	Potri.003G064300.1	AT1G54210.1
Potra2n197s34857.1	1.053820659	0.727897117	0.101661913	0.79477084	Potri.003G064600.1	AT5G14750.1
Potra2n197s34858.1	-1.328951947	0.139386737	NA	NA	Potri.003G064700.1	AT1G72570.1
Potra2n197s34859.1	-1.109883168	-0.814255175	4.56538E-05	0.175916011		
Potra2n197s34860.3	NA	4.178731694	NA	NA	Potri.003G065000.1	AT3G13960.1
Potra2n197s34861.1	NA	NA	NA	NA	Potri.013G141900.1	AT1G72610.1
Potra2n197s34862.1	0.467308857	1.839362807	0.740596912	0.540010302	Potri.003G065400.1	AT3G13840.1
Potra2n197s34863.1	0.245920112	0.068091083	0.155317228	0.948989658	Potri.003G065500.1	AT1G54150.1
Potra2n197s34864.1	-0.031735941	0.239966131	0.920036028	0.859892315	Potri.003G065800.2	
Potra2n197s34865.1	-0.110153226	-0.049708266	0.829826045	0.989329144	Potri.003G118000.1	AT1G12500.1
Potra2n197s34866.1	0.74958916	0.536627516	0.320038438	0.869327705		
Potra2n197s34867.1	-1.388616114	1.15786533	0.717847249	NA	Potri.001G168300.2	AT1G54140.1
Potra2n197s34868.1	-0.529829544	-0.1889189	1.12099E-05	0.70570456	Potri.003G066000.1	AT1G54130.1
Potra2n197s34869.1	0.183740373	0.160456253	0.205331388	0.824378527	Potri.003G066200.1	AT1G72560.2
Potra2n197s34870.1	0.590021812	0.031735303	0.005422321	0.991089517	Potri.003G066300.1	AT2G05940.1
Potra2n197s34871.1	3.38618284	3.021780406	NA	NA		
Potra2n197s34872.1	6.50520201	2.923254186	4.50835E-54	0.028004027	Potri.003G066400.1	AT5G07990.1
Potra2n197s34873.1	4.230920795	1.25396327	4.82661E-09	0.222841676	Potri.003G066600.1	AT5G07990.1

Potra2n197s34874.2	4.428734097	1.343458242	1.37543E-10	0.221840363	Potri.003G066600.1	AT5G07990.1
Potra2n197s34875.1	0.031395091	0.10488128	0.906996136	0.917751882	Potri.003G066900.1	AT1G54115.1
Potra2n197s34876.1	-0.10482187	0.099457112	0.611407972	0.947969749	Potri.003G067000.1	AT3G14067.1
Potra2n197s34877.1	-0.134021933	-0.078120283	0.108438893	0.914162682	Potri.003G067100.1	AT5G20570.1
Potra2n197s34878.1	-0.825321429	-0.035729554	0.722544727	NA	Potri.003G067200.1	AT3G14060.1
Potra2n197s34879.1	-0.024713131	0.08079954	0.933557154	0.945173238	Potri.003G067600.1	AT1G17420.1
Potra2n197s34880.2	0.783024205	0.379791734	1.85701E-09	0.523342013	Potri.003G067700.5	AT1G54100.1
Potra2n197s34881.1	0.259859932	0.104817074	0.512937734	0.974275337		
Potra2n197s34882.1	-0.058869164	0.038172504	0.723133495	0.974367223	Potri.003G067900.1	AT3G14075.2
Potra2n197s34883.1	0.713056257	0.0329513	1.32892E-05	0.979719961	Potri.003G068000.15	AT1G19110.1
Potra2n197s34884.1	1.462096298	1.099839894	0.129972983	0.848131816	Potri.003G068300.1	AT1G72490.1
Potra2n197s34885.1	-0.123625364	0.112940584	0.333620572	0.843765296	Potri.001G166500.1	AT1G72480.1
Potra2n197s34886.1	-0.246187362	-0.302216598	0.824186869	0.949847686		
Potra2n197s34887.1	-0.050446088	-0.198332398	0.763084328	0.677013336	Potri.003G068700.1	AT1G72470.1
Potra2n197s34888.1	0.165166225	0.101917747	0.873861326	0.984292161	Potri.003G068800.1	AT3G42880.1
Potra2n197s34889.1	7.576857726	3.828001209	1.36106E-19	0.298167012	Potri.003G068900.1	AT1G72450.1
Potra2n197s34890.1	-0.035387023	-0.05167131	0.795034777	0.948577865	Potri.003G069000.2	AT1G17370.1
Potra2n197s34891.4	0.131852828	0.091350654	0.304993577	0.903934429	Potri.003G069200.1	AT1G72390.1
Potra2n197s34892.1	0.085319781	0.055471515	0.518193253	0.948577865		
Potra2n197s34893.1	0.187189819	0.028709988	0.319063606	0.985393296		
Potra2n197s34894.1	1.380139286	0.40712649	3.6145E-10	0.725908641	Potri.003G069600.1	AT3G14130.1
Potra2n197s34895.1	0.117386614	-0.442662991	0.768410831	0.628821311	Potri.T055866.1	
Potra2n197s34896.1	NA	NA	NA	NA	Potri.T076901.1	
Potra2n197s34897.1	-0.035689151	-0.407488302	0.880582501	0.523403199	Potri.003G069700.1	AT5G66840.1
Potra2n197s34898.1	0.073882468	-0.028277404	0.635214512	0.981726976	Potri.003G069900.1	AT1G11890.1
Potra2n197s34899.5	-0.346327262	-0.104387179	0.000380654	0.857189843	Potri.003G070100.1	AT3G14160.1
Potra2n197s34900.1	-1.152536281	-0.370879865	6.03895E-07	0.769432732	Potri.003G070200.1	AT3G14170.1
Potra2n197s34901.7	0.321246388	0.138926118	0.019001438	0.798429987	Potri.003G070400.1	AT3G14172.1
Potra2n199s34902.1	2.525192055	3.00300511	NA	NA	Potri.010G072000.1	AT2G38940.1
Potra2n199s34903.1	NA	2.253733581	NA	NA		
Potra2n199s34904.1	1.459962178	3.859693997	NA	NA		
Potra2n199s34905.1	NA	2.253733581	NA	NA		

Potra2n199s34906.1						
Potra2n199s34907.1	0.92641307	2.966994084	NA	NA	Potri.010G071900.1	
Potra2n199s34908.1	0.82357019	NA	NA	NA		
Potra2n199s34909.1	0.038889329	-0.811937069	0.988230006	0.953831244	Potri.010G071600.1	AT2G38940.1
Potra2n199s34910.1	0.117005248	-0.329124319	0.668230757	0.786910796	Potri.005G223500.1	AT2G38940.1
Potra2n199s34911.1	-3.944351202	0.745401491	0.138354087	0.932622944	Potri.010G071400.1	AT1G08650.1
Potra2n199s34912.1	NA	1.394613675	NA	NA	Potri.010G071300.1	AT3G16210.1
Potra2n199s34913.1	-0.189565079	0.068752321	0.417043984	0.962661188	Potri.010G071200.2	AT1G04400.2
Potra2n199s34914.1	0.224209057	0.060387331	0.365703862	0.976302215	Potri.010G071000.1	AT5G43330.1
Potra2n199s34915.1	0.82357019	1.394613675	NA	NA		
Potra2n199s34916.1	4.05993522	1.042576208	6.20412E-65	0.492230064	Potri.010G070900.1	AT2G29420.1
Potra2n199s34917.1	-0.506604073	-0.102933854	0.136511255	0.967103157	Potri.010G070800.6	AT3G23280.2
Potra2n199s34918.1	-0.070991524	-0.025895547	0.42493086	0.984683464	Potri.010G070800.6	AT3G23280.2
Potra2n199s34919.1						
Potra2n199s34920.1	-0.251356147	-0.260970591	0.148952426	0.84801014	Potri.010G070700.1	AT1G07090.1
Potra2n19c33259.1	0.106734106	-0.042953861	0.384208116	0.966148753	Potri.019G133500.1	AT4G10360.2
Potra2n19c33260.1	0.272631942	0.291428597	0.039857312	0.63682724	Potri.019G133400.1	AT1G09575.1
Potra2n19c33261.1	0.586394698	0.159377495	0.00046551	0.900551258	Potri.019G133200.1	AT5G58240.1
Potra2n19c33262.2	-0.10977855	-0.033688834	0.378449631	0.971537796	Potri.019G133000.1	AT5G51340.1
Potra2n19c33263.1	0.389860727	0.346535095	0.083776134	0.63682724	Potri.019G132800.3	AT5G58270.1
Potra2n19c33264.1	NA	NA	NA	NA	Potri.002G176000.1	AT1G60170.1
Potra2n19c33265.1	-0.736958823	-0.954715291	0.40005044	0.862335622	Potri.019G132601.1	AT1G20670.1
Potra2n19c33266.1	-1.119283617	-0.016180357	0.00016426	0.995261215	Potri.019G132400.1	
Potra2n19c33267.1	-0.156976841	-0.021537308	0.237195486	0.983113084	Potri.019G132300.1	AT5G01970.1
Potra2n19c33268.1	1.70026788	1.666732563	0.003235773	0.323147091	Potri.013G160000.1	AT5G01960.1
Potra2n19c33269.1	0.092922645	0.292335229	0.701188143	0.812696706	Potri.019G132100.1	AT2G38610.1
Potra2n19c33270.1	0.449680304	-0.011697089	0.001849934	0.993939945	Potri.019G132000.1	AT5G05370.1
Potra2n19c33271.1	0.522366132	0.006967081	0.000194235	0.996032145	Potri.019G131900.1	AT1G26910.1
Potra2n19c33272.1	1.246104299	0.447036051	1.38032E-21	0.51059114	Potri.019G131800.3	AT1G06840.1
Potra2n19c33273.1	-2.295337707	-1.724928411	NA	NA	Potri.019G131700.1	AT1G17455.1
Potra2n19c33274.1	-0.431588701	-0.0822095	0.374423257	0.97815902	Potri.019G131600.1	AT2G30520.1
Potra2n19c33275.1	NA	NA	NA	NA		

Potra2n19c33276.1	-0.69332359	-0.370994238	0.000335422	0.744967605	Potri.019G131500.1	AT5G58300.2
Potra2n19c33277.1	-0.369668091	-0.147483383	4.23333E-06	0.679485917	Potri.019G131400.10	AT5G41700.1
Potra2n19c33278.1	2.40842434	1.139010218	5.69181E-12	0.152054556	Potri.019G131300.1	AT4G27950.1
Potra2n19c33279.1	0.364020653	0.006656843	0.041835158	0.996032145	Potri.013G158400.1	AT5G20920.3
Potra2n19c33280.1	-0.219787471	0.679473953	0.565568402	NA	Potri.019G131100.1	AT3G10870.1
Potra2n19c33281.1	3.63997188	3.843189633	0.241042918	NA		
Potra2n19c33282.1	0.539803795	0.448359263	0.646229687	0.941260209	Potri.019G131000.2	AT2G30500.2
Potra2n19c33283.1	-0.146972493	0.030890459	0.36887507	0.973347974	Potri.019G130900.1	AT1G55150.1
Potra2n19c33284.1	NA	NA	NA	NA		
Potra2n19c33285.1	0.021743048	0.098692589	0.940959005	0.953608209	Potri.019G130700.1	AT2G30490.1
Potra2n19c33286.1	1.898068119	-1.012072639	0.134794822	NA	Potri.T178900.1	AT1G17180.1
Potra2n19c33287.3	0.287060079	0.10739561	0.049642047	0.915488947	Potri.019G130300.1	AT4G32010.1
Potra2n19c33288.1	-1.825519125	-0.376610263	0.016676097	0.921615465	Potri.019G130200.1	AT4G34390.1
Potra2n19c33289.1	NA	1.381953902	NA	NA	Potri.019G130100.1	AT5G05280.1
Potra2n19c33290.1	-0.607435555	-0.390488243	0.000877549	0.72476666	Potri.019G130000.1	AT2G40620.1
Potra2n19c33291.1	-0.561327254	-0.529323774	0.301728653	0.809914834	Potri.019G129900.1	AT5G66750.1
Potra2n19c33292.1	-2.085200283	-0.830292766	4.83253E-05	0.684244573	Potri.019G129600.1	
Potra2n19c33293.1	0.187228148	-0.310942462	0.7324675	0.936549944	Potri.T119700.1	
Potra2n19c33294.1	0.707957817	-0.125720053	0.036091425	0.953831244	Potri.T119500.1	
Potra2n19c33295.1	1.062194695	-0.092342764	0.001195603	0.97601212	Potri.019G129600.1	
Potra2n19c33296.1	-1.728533998	-0.927408315	0.000356874	0.623736221	Potri.019G129600.1	
Potra2n19c33297.1	0.82357019	1.911450358	NA	NA	Potri.019G129000.1	AT5G05340.1
Potra2n19c33298.1	-0.270536423	-0.222961754	0.037829359	0.84231643	Potri.013G154800.1	AT1G06890.1
Potra2n19c33299.1	-0.282111522	-0.070585485	0.015136603	0.914903885	Potri.019G128800.1	AT3G51620.2
Potra2n19c33300.3	-0.387698183	0.228461357	0.061541616	0.884553748	Potri.019G128600.2	AT5G58350.1
Potra2n19c33301.1	-2.357078177	-2.009118585	0.000188536	0.089809981	Potri.019G128500.1	AT1G06920.1
Potra2n19c33302.1	-1.582661346	-0.916832112	0.082112663	0.716682524	Potri.019G128400.1	AT2G30395.1
Potra2n19c33303.1	-1.35927387	0.110081123	NA	NA	Potri.019G128200.1	AT2G30370.2
Potra2n19c33304.1	0.42287663	0.656849759	0.195001354	0.676711874	Potri.019G128100.1	AT2G30360.1
Potra2n19c33305.1	-0.092692568	0.19705037	0.700087446	0.879147863	Potri.019G128000.2	AT5G58370.2
Potra2n19c33306.1	0.37577252	-0.139283037	0.206466206	0.925035363	Potri.019G127900.1	AT5G58375.1
Potra2n19c33307.1	-0.286133972	0.221779901	0.330921978	0.888896953	Potri.019G127600.1	AT2G30350.2

Potra2n19c33308.1	-0.849091953	-0.154497702	2.70378E-09	0.854980698	Potri.019G127500.1	AT5G58380.1
Potra2n19c33309.1	-2.301250658	-1.108625061	5.64254E-05	0.520533674	Potri.019G127400.2	AT1G06930.1
Potra2n19c33310.1	-0.110991614	-0.742801941	0.872660055	0.837645998	Potri.019G127300.1	AT2G40470.1
Potra2n19c33311.1	0.189086406	-0.031001408	0.106699786	0.977061256	Potri.019G127000.2	AT1G06950.1
Potra2n19c33312.1	NA	NA	NA	NA	Potri.019G126900.1	AT2G30320.1
Potra2n19c33313.1	2.659097013	0.608748198	0.135604949	NA	Potri.019G126800.1	AT2G40435.1
Potra2n19c33314.1	NA	NA	NA	NA	Potri.019G126700.1	AT2G30300.1
Potra2n19c33315.1	-0.159209156	0.055036702	0.206106955	0.957877578	Potri.019G126500.2	AT2G30280.1
Potra2n19c33316.1	-0.592305522	-0.241432306	0.021984004	0.881105037	Potri.019G126400.1	AT2G25150.1
Potra2n19c33317.1	-0.236537195	-0.054868977	0.048451789	0.954400397	Potri.019G126300.1	AT2G30270.1
Potra2n19c33318.2	-0.069592556	0.045100061	0.536582013	0.939653927	Potri.019G126000.2	AT5G58410.1
Potra2n19c33319.1	-0.064178654	0.158998764	0.72419021	0.717365554		
Potra2n19c33320.1	0.438788854	-0.936302427	0.50158524	0.621894291	Potri.013G153900.1	AT1G16870.1
Potra2n19c33321.1	0.82357019	1.363811008	NA	NA	Potri.013G153800.2	AT3G10985.1
Potra2n19c33322.1	2.610910166	NA	NA	NA	Potri.019G125600.1	AT3G51680.1
Potra2n19c33323.4	0.908947316	0.111931196	0.012131065	0.971828097	Potri.016G092600.1	AT3G50950.1
Potra2n19c33324.1	-0.393308323	-0.305836146	0.046370536	0.685425442	Potri.019G125300.1	AT2G16030.1
Potra2n19c33325.1	1.829398776	1.961885577	0.303594909	NA		
Potra2n19c33326.1	-0.588463505	-4.824545133	0.634822256	NA	Potri.019G125100.1	AT3G09280.1
Potra2n19c33327.1	-1.953814271	-0.674513651	1.85327E-21	0.497568278	Potri.019G125000.1	AT4G03210.1
Potra2n19c33328.1	NA	NA	NA	NA	Potri.019G122300.1	AT3G51190.1
Potra2n19c33329.1	4.955328146	2.732288543	1.09425E-11	0.666490346	Potri.019G124500.1	AT1G73325.1
Potra2n19c33330.1	7.855977451	3.770124724	4.80504E-34	0.32068134		
Potra2n19c33331.1	8.134917932	4.182541428	1.91387E-63	0.027280415		
Potra2n19c33332.1	0.359701372	-0.082705296	0.027898122	0.953658357		
Potra2n19c33333.1	6.195694125	2.462908667	1.04804E-28	0.362237593		
Potra2n19c33334.1	0.580888332	-0.384637076	0.28832106	0.867960518	Potri.019G121700.1	AT2G30210.1
Potra2n19c33335.1	0.026513352	0.046405333	0.811630486	0.954419252	Potri.019G124200.1	AT5G07120.1
Potra2n19c33336.1	-0.588272628	-0.455569694	0.001494106	0.349324124	Potri.019G124100.2	AT2G38360.1
Potra2n19c33337.2	0.183148739	0.021156337	0.405567884	0.987454031	Potri.019G124000.1	AT5G58430.1
Potra2n19c33338.1	-0.296917253	0.066866351	0.452045773	0.98669034	Potri.019G123900.1	AT2G30230.1
Potra2n19c33339.1	NA	NA	NA	NA	Potri.019G123800.1	AT2G13620.1

Potra2n19c33340.1	2.160082706	NA	NA	NA	Potri.019G123700.1	AT2G13620.1
Potra2n19c33341.1	2.584445682	0.872672873	1.9416E-19	0.156102189	Potri.013G153400.1	AT2G38470.1
Potra2n19c33342.1	0.365490092	0.444017512	0.320102233	0.609779445	Potri.019G123400.1	AT5G01720.1
Potra2n19c33343.1	0.063680113	0.121765465	0.768454229	0.909675463	Potri.019G121400.1	AT2G30260.1
Potra2n19c33344.1	0.604553358	2.260550288	0.726545548	0.54752884	Potri.019G121300.1	AT5G03170.1
Potra2n19c33345.1	0.826085371	2.178204743	0.323464374	0.342512317		
Potra2n19c33346.1	-0.120830095	2.628993152	0.955261055	0.561272053	Potri.019G123100.1	AT5G03170.1
Potra2n19c33347.1	NA	NA	NA	NA		
Potra2n19c33348.1	4.213706679	2.055089933	0.000417145	0.772556533	Potri.019G120600.1	AT5G54490.1
Potra2n19c33349.1	-0.359829521	0.3342945	0.42811261	0.821704644	Potri.019G120300.1	AT4G21380.1
Potra2n19c33350.1	-0.237683542	-0.080732209	0.08548774	0.944323367	Potri.019G120200.1	
Potra2n19c33351.1	0.461497602	0.19493398	0.039864239	0.794641467	Potri.019G120000.1	AT4G03230.1
Potra2n19c33352.1	1.006178274	0.603245456	1.17339E-06	0.325703878	Potri.019G120000.1	AT4G03230.1
Potra2n19c33353.1	4.441713409	1.20816332	7.45666E-66	0.547799891	Potri.019G119600.3	AT4G03230.1
Potra2n19c33354.1	5.822104483	4.674305567	2.34374E-05	NA	Potri.019G120000.1	AT4G03230.1
Potra2n19c33355.1	4.827285802	1.832023167	4.73926E-16	0.712127394	Potri.019G119600.3	AT4G03230.1
Potra2n19c33356.1	4.535040047	1.330413309	7.79973E-60	0.456108303	Potri.019G119600.3	AT4G03230.1
Potra2n19c33357.1	0.43983019	0.187199139	0.042851657	0.853030738	Potri.019G119500.2	AT4G03260.1
Potra2n19c33358.1	0.484081616	0.270669268	0.054619085	0.724504724	Potri.019G119400.1	AT1G11360.3
Potra2n19c33359.1	0.172683174	-0.152152828	0.382226397	0.88080281	Potri.019G119300.1	AT4G03260.2
Potra2n19c33360.1	0.638497973	0.027767727	0.013074978	0.990874899	Potri.019G119200.1	AT2G19130.1
Potra2n19c33361.1	NA	NA	NA	NA	Potri.019G119100.1	AT1G15030.1
Potra2n19c33362.1	1.64774763	NA	NA	NA		
Potra2n19c33363.1	-1.35927387	0.446976274	NA	NA	Potri.019G118900.1	AT1G79180.1
Potra2n19c33364.1	6.370645776	3.010689165	3.47879E-05	NA	Potri.019G118800.1	AT5G16770.1
Potra2n19c33365.1	7.515796343	5.890769733	2.38711E-08	0.085896019	Potri.019G118700.1	AT5G16770.1
Potra2n19c33366.1	-1.091794275	-0.899952519	0.002825262	0.631733285	Potri.019G118600.1	AT4G03270.1
Potra2n19c33367.1	-0.530896619	-0.140125368	2.07008E-05	0.859892315	Potri.019G118500.1	AT4G03280.1
Potra2n19c33368.3	-0.237117624	-0.035680223	0.211764549	0.97950444	Potri.019G118400.1	AT4G05120.1
Potra2n19c33369.1	-0.80052956	-0.261568713	0.167046688	0.922681078	Potri.019G118300.1	AT4G28100.1
Potra2n19c33370.1	3.908516794	NA	0.073582572	NA	Potri.019G118200.1	AT4G21440.1
Potra2n19c33371.1	3.169748549	0.681663001	5.34143E-16	0.787866213	Potri.019G118000.1	AT1G03940.1

Potra2n19c33372.1						
Potra2n19c33373.1	6.530640329	3.99563954	4.8852E-06	0.258140976	Potri.019G118000.1	AT1G03940.1
Potra2n19c33374.1	-0.502098294	-0.265963852	0.001048696	0.633554324	Potri.019G117900.1	AT2G20890.1
Potra2n19c33375.1	-1.934126624	-2.0827378	NA	NA		
Potra2n19c33376.1	1.64774763	NA	NA	NA	Potri.013G147900.1	AT4G10270.1
Potra2n19c33377.1	-1.35715766	-0.341493081	2.77232E-09	0.762561685	Potri.019G116400.1	AT4G21450.1
Potra2n19c33378.1	-0.445453116	0.635757224	0.344557522	0.810232776	Potri.019G116300.1	AT4G28240.1
Potra2n19c33379.1	-0.084709623	-0.403287958	0.855703789	0.895140353	Potri.019G116200.1	AT4G28230.1
Potra2n19c33380.1	NA	NA	NA	NA	Potri.019G106500.1	AT1G06330.1
Potra2n19c33381.1	-0.462078007	0.009857294	0.022409369	0.996032145		
Potra2n19c33382.1	NA	NA	NA	NA	Potri.019G106500.1	AT1G06330.1
Potra2n19c33383.1	3.438871146	4.445095926	NA	NA	Potri.019G107700.1	AT4G03500.1
Potra2n19c33384.1					Potri.019G108600.1	
Potra2n19c33385.1						
Potra2n19c33386.1	NA	1.363811008	NA	NA	Potri.019G115733.1	AT5G51160.1
Potra2n19c33387.1					Potri.019G115101.1	AT5G51160.1
Potra2n19c33388.1	2.966442973	1.363811008	NA	NA		
Potra2n19c33389.1	NA	NA	NA	NA	Potri.019G106500.1	AT1G06330.1
Potra2n19c33390.1						
Potra2n19c33391.1	NA	NA	NA	NA	Potri.019G106500.1	AT1G06330.1
Potra2n19c33392.1	-0.013622513	0.074563132	0.956990155	0.973347974	Potri.019G106500.1	AT1G06330.1
Potra2n19c33393.1						
Potra2n19c33394.1					Potri.019G105600.1	
Potra2n19c33395.1					Potri.019G108300.1	
Potra2n19c33396.2	NA	NA	NA	NA	Potri.019G108400.1	AT3G50150.1
Potra2n19c33397.1	0.380390148	-0.089947964	0.887313212	NA	Potri.019G108700.1	
Potra2n19c33398.1					Potri.019G105600.1	
Potra2n19c33399.2					Potri.019G108600.1	
Potra2n19c33400.1	NA	NA	NA	NA	Potri.019G108400.1	AT3G50150.1
Potra2n19c33401.1	1.352793993	0.624801019	0.129114026	0.916192318	Potri.019G108700.1	
Potra2n19c33402.1	0.221108865	0.455483266	0.667147265	0.818007496	Potri.019G108800.1	AT4G03320.1
Potra2n19c33403.1	-0.147695666	0.012524424	0.501501622	0.994486196	Potri.019G105100.1	AT2G20810.1

Potra2n19c33404.1	NA	NA	NA	NA	Potri.013G146100.3	AT2G20815.1
Potra2n19c33405.1	0.087458448	-0.125855558	0.477133558	0.865611082	Potri.019G104900.1	AT2G20820.1
Potra2n19c33406.1	1.664677056	3.420502479	NA	NA	Potri.019G109200.1	
Potra2n19c33407.1	-1.689283465	-0.355045015	3.30538E-09	0.824317405	Potri.019G104700.1	AT2G20830.1
Potra2n19c33408.1	0.92641307	0.64247635	NA	NA		
Potra2n19c33409.2	-0.510324079	0.001084098	0.018508723	0.999582698	Potri.019G104600.1	AT4G03340.1
Potra2n19c33410.1	-0.419668877	-0.149413008	0.160101021	0.882991925	Potri.019G104500.1	AT4G28190.1
Potra2n19c33411.1	-0.968726936	-0.671265136	0.000210861	0.455054253	Potri.019G104400.1	AT1G03530.1
Potra2n19c33412.1	0.82357019	NA	NA	NA	Potri.002G263900.1	AT3G24060.1
Potra2n19c33413.1	1.64774763	NA	NA	NA	Potri.002G263900.1	AT3G24060.1
Potra2n19c33414.1	NA	3.145099921	NA	NA	Potri.012G106700.1	AT3G48880.1
Potra2n19c33415.1	-0.577352488	-0.264898476	0.00031078	0.633830624	Potri.019G104000.1	AT2G20840.1
Potra2n19c33416.2	-0.551536425	0.245981098	0.018049191	0.829862776	Potri.019G103900.1	AT4G03390.1
Potra2n19c33417.1	-2.478536027	-0.905412578	6.55016E-08	0.298167012	Potri.019G103800.1	AT1G11120.2
Potra2n19c33418.1	-1.409983009	-1.55593228	NA	NA		
Potra2n19c33419.1	3.125402034	3.408150094	NA	NA	Potri.019G103600.2	AT1G76810.1
Potra2n19c33420.1	2.685015931	NA	NA	NA	Potri.019G103500.1	AT4G03400.1
Potra2n19c33421.1	1.664677056	NA	NA	NA		
Potra2n19c33422.1	-0.019882081	0.031316067	0.936099329	0.985609221	Potri.019G103400.1	AT1G03560.1
Potra2n19c33423.2	0.079301404	-0.020259272	0.643976401	0.985393296	Potri.019G103200.1	AT4G03410.2
Potra2n19c33424.1	0.029370167	0.001731294	0.813116327	0.999325699	Potri.019G103100.3	AT4G03415.1
Potra2n19c33425.1	1.251060024	0.433549692	1.28826E-09	0.467563492	Potri.019G103000.1	AT4G03420.1
Potra2n19c33426.1	1.545832463	NA	NA	NA	Potri.004G183300.1	AT5G60620.1
Potra2n19c33427.1	-1.317292482	-0.744589582	NA	NA	Potri.019G102800.1	AT2G20875.1
Potra2n19c33428.2	0.244770655	0.010399322	0.137789168	0.995139072	Potri.019G102700.1	AT5G08415.1
Potra2n19c33429.1	4.084614497	NA	0.1425833	NA	Potri.019G102600.2	
Potra2n19c33430.1	1.459962178	2.0078969	NA	NA	Potri.019G102500.1	AT1G65690.1
Potra2n19c33431.1	NA	0.674963689	NA	NA	Potri.013G135700.1	AT2G14830.1
Potra2n19c33432.1	0.82357019	NA	NA	NA	Potri.019G102300.1	
Potra2n19c33433.1	-0.203719545	-0.383773122	0.601438697	0.489780897	Potri.019G102200.1	AT4G28140.1
Potra2n19c33434.1	-3.284408689	-1.238422223	0.048797371	0.84745523	Potri.019G102000.1	AT2G20760.1
Potra2n19c33435.1	-3.315319959	0.19968604	NA	NA		

Potra2n19c33436.1	-1.239634162	0.123140448	0.001327945	0.930203195	Potri.019G101900.1	AT4G28250.1
Potra2n19c33437.1	-0.150274904	-0.042657115	0.458192067	0.981772014	Potri.019G101800.1	AT2G20740.1
Potra2n19c33438.1	-0.644399791	0.070123134	0.456516069	0.989300076	Potri.019G101700.1	AT1G03670.1
Potra2n19c33439.2	0.004726549	0.080848668	0.994891611	0.984292161	Potri.019G101400.1	AT4G03500.1
Potra2n19c33440.1	0.520563676	0.652274048	0.685991821	0.908627223	Potri.019G101100.1	AT2G20725.1
Potra2n19c33441.1	-1.207418179	0.648080369	0.606501166	NA	Potri.019G047200.4	AT5G16760.1
Potra2n19c33442.1	-1.453442554	-0.979760739	0.473400994	NA		
Potra2n19c33443.1	0.448271578	0.028112897	0.408723183	0.994762886	Potri.019G101100.1	AT2G20725.1
Potra2n19c33444.1	-1.278182755	0.239099564	0.304993577	0.982827151	Potri.019G101000.1	AT4G28260.1
Potra2n19c33445.1	0.573503265	0.60582166	0.035491569	0.593648258	Potri.019G100800.1	AT4G03510.1
Potra2n19c33446.1	NA	NA	NA	NA	Potri.013G132000.2	AT5G56170.1
Potra2n19c33447.1	-0.703870087	-0.227005095	0.00168216	0.862097315	Potri.019G100601.1	AT4G28290.1
Potra2n19c33448.1	0.381796189	-0.071912263	0.014174852	0.950044491		
Potra2n19c33449.1	1.290777134	-0.728227935	0.003643292	0.690089431	Potri.019G100300.1	AT1G03700.1
Potra2n19c33450.1	NA	NA	NA	NA	Potri.019G100100.1	
Potra2n19c33451.1	NA	NA	NA	NA	Potri.019G100200.1	
Potra2n19c33452.1	0.941012205	-0.157119299	0.000414732	0.92223649	Potri.019G100001.1	AT3G09390.1
Potra2n19c33453.1	2.34224872	1.551035668	9.9055E-09	0.294386607	Potri.019G099800.1	AT5G24590.2
Potra2n19c33454.1					Potri.019G099700.1	AT1G62680.1
Potra2n19c33455.1	NA	NA	NA	NA	Potri.013G130400.1	AT2G20680.1
Potra2n19c33456.1	-0.756875934	1.844342023	0.579438548	0.84801014	Potri.019G099600.1	AT4G32480.1
Potra2n19c33457.1	1.566277346	0.372735594	1.35256E-10	0.88643982	Potri.019G099300.1	AT4G20970.1
Potra2n19c33458.1	0.647984085	0.07916577	0.010647712	0.9789664	Potri.019G099300.1	AT4G20970.1
Potra2n19c33459.1	0.490306944	0.120468081	0.000856378	0.886633917	Potri.019G099100.2	AT5G10460.1
Potra2n19c33460.1	0.679056323	0.168341871	1.6765E-05	0.900869139	Potri.019G099000.1	AT5G39740.2
Potra2n19c33461.1					Potri.019G097900.1	
Potra2n19c33462.1	3.488996875	2.388773718	1.11126E-29	0.07503536	Potri.019G114600.4	AT4G12010.1
Potra2n19c33463.1	-1.893280225	-0.087527652	NA	NA	Potri.013G128300.5	AT3G59780.1
Potra2n19c33464.1	3.913310545	2.536480704	1.0897E-41	0.107143374	Potri.019G095932.1	AT5G51630.1
Potra2n19c33465.1	1.659700231	-1.51555656	0.062388471	0.701494362		
Potra2n19c33466.1	4.516099252	0.708032374	9.56854E-05	NA	Potri.013G128300.5	AT3G59780.1
Potra2n19c33467.1	2.115384252	NA	NA	NA	Potri.019G114866.1	AT3G59780.1

Potra2n19c33468.1	4.089892233	3.109479177	3.57166E-05	0.200085324	Potri.019G098900.1	AT5G17680.1
Potra2n19c33469.1	0.552941959	0.305166378	0.740596912	NA	Potri.019G097300.1	AT4G16860.1
Potra2n19c33470.1	3.176292118	2.124058312	8.41461E-33	0.062524975	Potri.019G098900.1	AT5G17680.1
Potra2n19c33471.1	1.159127682	0.199516476	0.240956127	0.98177802	Potri.019G097300.1	AT4G16860.1
Potra2n19c33472.1	2.452935128	0.342822108	0.029271389	NA	Potri.019G098500.1	AT5G17680.1
Potra2n19c33473.1	2.987379103	-0.744589582	0.129953265	NA	Potri.019G095932.1	AT5G51630.1
Potra2n19c33474.1	1.359925609	-0.263700433	0.078382937	0.950734678	Potri.019G097000.1	AT5G49960.1
Potra2n19c33475.1	1.065880516	-1.519215768	NA	NA	Potri.019G096101.1	AT3G59780.1
Potra2n19c33476.1	0.224470821	1.53735798	NA	NA	Potri.013G128300.5	AT3G59780.1
Potra2n19c33477.1	5.424759226	3.346660825	0.000597647	NA	Potri.019G098900.1	AT5G17680.1
Potra2n19c33478.1	1.447246416	0.870814425	0.01085245	0.734766537	Potri.019G098800.1	AT4G16860.1
Potra2n19c33479.1	1.074650518	0.391543903	1.49265E-35	0.585361878	Potri.019G097000.1	AT5G49960.1
Potra2n19c33480.3	0.031062581	0.605491703	0.946167104	0.509990466	Potri.013G128300.5	AT3G59780.1
Potra2n19c33481.1	-0.011944714	-0.015172999	0.954155001	0.991036771	Potri.019G096900.1	AT2G43770.1
Potra2n19c33482.1	-0.160154692	-0.30333972	0.837164216	0.943819961	Potri.019G096800.1	AT5G16250.1
Potra2n19c33483.1	0.005254614	-0.025042962	0.98199381	0.988883632	Potri.019G096500.1	AT2G43780.2
Potra2n19c33484.1	NA	NA	NA	NA	Potri.019G095800.1	AT5G47530.1
Potra2n19c33485.1	NA	NA	NA	NA	Potri.013G118300.1	AT5G47530.1
Potra2n19c33486.1	-0.607045066	-0.127217763	2.77005E-10	0.885900567	Potri.019G112400.1	AT3G59690.1
Potra2n19c33487.1	0.82357019	NA	NA	NA	Potri.019G112500.1	
Potra2n19c33488.1	-1.761363711	-0.708505776	3.98314E-07	0.590797456	Potri.019G112300.2	AT3G59680.2
Potra2n19c33489.2	NA	0.674963689	NA	NA	Potri.019G112200.1	AT3G59670.1
Potra2n19c33490.1	0.476191469	-0.037742511	0.006405228	0.977559521	Potri.019G112100.1	AT1G05720.1
Potra2n19c33491.2	1.974149906	0.206511835	3.44685E-17	0.907073354	Potri.019G112000.1	AT1G05710.5
Potra2n19c33492.1	0.536582039	0.188778815	0.349232702	0.956865545	Potri.019G095600.2	AT5G60030.1
Potra2n19c33493.6	0.246080383	0.088215753	0.665474865	0.983113084	Potri.019G092000.1	AT1G14300.1
Potra2n19c33494.1	-0.915952827	-1.390697472	0.694526018	NA		
Potra2n19c33495.1	NA	NA	NA	NA		
Potra2n19c33496.1	-2.095321122	-0.267124606	0.242603078	0.972159498	Potri.013G124400.1	AT3G58120.1
Potra2n19c33497.1	0.605464768	-1.169772496	0.683368412	NA		
Potra2n19c33498.1	1.009429319	0.311557952	0.671394661	NA	Potri.019G091800.1	AT2G24720.1
Potra2n19c33499.1	0.0594946	0.207020624	0.702108903	0.857374454	Potri.019G091700.1	AT3G58110.1

Potra2n19c33500.1	0.82357019	NA	NA	NA		
Potra2n19c33501.1					Potri.013G119600.1	AT5G04530.1
Potra2n19c33502.1	NA	0.674963689	NA	NA	Potri.019G091600.1	AT5G04530.1
Potra2n19c33503.2	-0.346942026	-0.07466769	0.219632375	0.936549944	Potri.019G091500.1	AT3G58100.1
Potra2n19c33504.1	6.87888008	2.72539866	1.20066E-17	NA	Potri.019G091400.1	AT2G42360.1
Potra2n19c33505.1	-0.056520811	-0.425447334	0.917991175	0.886281281	Potri.019G091300.1	AT3G15030.3
Potra2n19c33506.1	1.64774763	NA	NA	NA	Potri.019G091100.1	AT2G31081.1
Potra2n19c33507.1	-0.073861923	-0.322633076	0.880280002	0.890076131	Potri.019G091000.1	AT3G58590.1
Potra2n19c33508.1					Potri.019G090900.1	
Potra2n19c33509.1	NA	0.674963689	NA	NA		
Potra2n19c33510.1					Potri.019G090800.1	AT2G31083.1
Potra2n19c33511.1					Potri.019G090800.1	AT2G31083.1
Potra2n19c33512.1	-0.425150059	0.101689232	0.003397502	0.921099436	Potri.019G090700.1	AT1G06230.1
Potra2n19c33513.1	-0.594043203	-0.34968384	0.001047184	0.481859302	Potri.019G090600.2	AT1G06240.1
Potra2n19c33514.1	-0.458170897	0.109746424	0.018314396	0.943819961	Potri.019G090500.1	AT2G31090.1
Potra2n19c33515.1	-2.396052862	-1.070911793	NA	NA		
Potra2n19c33516.1	-3.884969755	0.105587777	0.159058738	NA	Potri.019G090300.1	AT2G42560.1
Potra2n19c33517.2	-0.742715233	-0.255698194	0.003552154	0.88643982	Potri.019G090200.1	AT2G42570.1
Potra2n19c33518.1					Potri.019G090100.1	AT5G50260.1
Potra2n19c33519.1	0.82357019	NA	NA	NA	Potri.019G090000.1	AT3G12060.1
Potra2n19c33520.1					Potri.008G086700.3	AT2G02990.1
Potra2n19c33521.1	0.055912522	-0.899683761	0.976449721	NA	Potri.019G090000.1	AT3G12060.1
Potra2n19c33522.1	-0.046570213	0.144685971	0.834634932	0.886633917	Potri.019G089900.10	AT2G31130.1
Potra2n19c33523.1	0.068850621	0.033595249	0.67092962	0.975578749	Potri.019G089800.1	AT3G58600.1
Potra2n19c33524.1	-0.448529802	-0.070279531	0.039165417	0.983113084	Potri.019G089700.1	AT3G18000.1
Potra2n19c33525.1	NA	NA	NA	NA	Potri.019G089600.1	AT1G53270.1
Potra2n19c33526.1	2.115384252	0.674963689	NA	NA	Potri.019G089400.1	AT3G58610.1
Potra2n19c33527.1	1.608474934	0.741904669	0.100961086	0.906679354	Potri.019G089500.1	AT2G40200.1
Potra2n19c33528.1	NA	NA	NA	NA	Potri.019G089200.1	
Potra2n19c33529.1	0.601967062	-0.786502229	NA	NA	Potri.019G089400.1	AT3G58610.1
Potra2n19c33530.1	0.224057711	-1.049284157	0.813415246	0.676711874	Potri.019G089300.1	
Potra2n19c33531.1	NA	0.674963689	NA	NA	Potri.019G089200.1	

Potra2n19c33532.1	-0.762938866	-0.657713626	0.000125581	0.037004867	Potri.019G095500.6	AT2G02960.1
Potra2n19c33533.1	-1.925212245	-2.085345829	0.396417863	NA		
Potra2n19c33534.1	-0.226359448	-0.01178124	0.088520674	0.989304496	Potri.019G111600.1	AT4G28300.1
Potra2n19c33535.1	-0.776663149	-0.311697353	0.0027897	0.808212225	Potri.019G095300.2	AT4G28290.1
Potra2n19c33536.1	1.459962178	NA	NA	NA		
Potra2n19c33537.1	1.459962178	NA	NA	NA	Potri.019G095200.1	AT2G20700.1
Potra2n19c33538.1	-0.196260081	0.112582825	0.321227012	0.924309478	Potri.019G111200.1	AT4G03520.1
Potra2n19c33539.1	-0.893709013	-0.886902276	0.526240056	0.900551258	Potri.019G109600.1	AT4G29990.1
Potra2n19c33540.1	2.632524198	3.269258361	NA	NA		
Potra2n19c33541.1	1.41589929	0.294360213	8.06121E-06	0.885900567	Potri.019G093900.1	AT3G54420.1
Potra2n19c33542.1	2.685015931	NA	NA	NA		
Potra2n19c33543.1	8.579744203	7.221048188	6.52033E-10	0.381704566	Potri.019G093800.1	AT3G54420.1
Potra2n19c33544.1					Potri.013G148950.1	
Potra2n19c33545.1	8.932477452	7.497146976	4.59039E-07	0.162483245	Potri.019G093900.1	AT3G54420.1
Potra2n19c33546.1	0.82357019	NA	NA	NA		
Potra2n19c33547.3	-0.367576648	0.102878518	0.002028003	0.94169165	Potri.019G093600.1	AT3G53690.1
Potra2n19c33548.1	-1.852841885	-0.071730486	1.98301E-06	0.97950444	Potri.019G093400.1	AT4G19170.1
Potra2n19c33549.1	2.181935746	1.410631351	0.073765336	NA	Potri.019G093300.1	AT2G20520.1
Potra2n19c33550.1	-2.436586768	-0.493468212	2.12341E-07	0.788065047	Potri.019G093100.1	AT4G35160.1
Potra2n19c33551.1	4.162344144	0.085333823	0.000256077	NA	Potri.019G093000.1	AT4G35160.1
Potra2n19c33552.1	NA	NA	NA	NA		
Potra2n19c33553.1	-0.102050384	-0.086547772	0.395155427	0.909675463	Potri.019G092800.1	AT1G28520.2
Potra2n19c33554.1	1.641576237	0.600934745	NA	NA	Potri.019G092600.1	AT1G06290.1
Potra2n19c33555.1	-0.387759188	-0.311135779	0.00721593	0.589882481	Potri.019G092500.1	AT5G60860.1
Potra2n19c33556.1	NA	NA	NA	NA	Potri.019G092400.1	AT2G31060.3
Potra2n19c33557.1	-1.065237186	-0.16749698	1.78755E-05	0.940841616	Potri.019G092300.1	AT4G35020.2
Potra2n19c33558.1					Potri.019G092201.1	AT1G06280.1
Potra2n19c33559.1	0.960540194	0.3510297	3.13076E-11	0.572132405	Potri.019G092100.1	AT2G42390.1
Potra2n19c33560.1	0.801620682	0.304084016	5.45694E-09	0.674283226	Potri.019G092000.1	AT1G14300.1
Potra2n19c33561.1	-1.384024423	0.47538083	0.276386407	0.917751882	Potri.013G124400.1	AT3G58120.1
Potra2n19c33562.1	4.715674315	2.86450839	0.012972194	NA		
Potra2n19c33563.1	3.992063689	2.86450839	0.062220947	NA	Potri.019G091800.1	AT2G24720.1

Potra2n19c33564.1	NA	NA	NA	NA		
Potra2n19c33565.1	-0.157108277	0.046618328	0.411818626	0.978751709	Potri.019G091700.1	AT3G58110.1
Potra2n19c33566.1	3.159588006	1.871975723	NA	NA		
Potra2n19c33567.1	0.236685393	0.262145732	0.900563904	0.984292161	Potri.019G089500.1	AT2G40200.1
Potra2n19c33568.1	-0.26886453	-0.031953269	0.90044822	0.998802143		
Potra2n19c33569.1	0.82357019	NA	NA	NA		
Potra2n19c33570.1	1.578268123	NA	NA	NA	Potri.019G089400.1	AT3G58610.1
Potra2n19c33571.2	-0.52575348	-1.233292591	0.67670411	0.727446209		
Potra2n19c33572.1	NA	NA	NA	NA	Potri.019G089200.1	
Potra2n19c33573.1	1.461890778	0.838975343	0.000946258	0.672348168	Potri.019G089000.1	AT1G68810.1
Potra2n19c33574.1	NA	0.674963689	NA	NA	Potri.001G116100.1	AT1G12390.1
Potra2n19c33575.1	6.891664212	2.7429057	1.73537E-53	0.566750433	Potri.019G088600.1	AT5G09360.1
Potra2n19c33576.1						
Potra2n19c33577.1	2.867296901	1.117866802	0.011300887	0.907073354	Potri.019G088200.1	AT1G17860.1
Potra2n19c33578.4	-0.18198703	0.011497224	0.066184367	0.987226805	Potri.019G088100.2	AT1G71820.1
Potra2n19c33579.1	0.314438862	-2.456974026	0.940067098	NA		
Potra2n19c33580.1	-0.771369836	-2.456974026	NA	NA	Potri.019G088000.2	AT1G12890.1
Potra2n19c33581.1	-1.973940029	-0.800574427	4.49719E-09	0.294386607		
Potra2n19c33582.1	-2.002325604	-0.813645723	8.02421E-09	0.294386607	Potri.019G087800.2	
Potra2n19c33583.1	-0.360364432	-0.034378393	0.051736216	0.98036348	Potri.019G087700.1	AT1G71830.1
Potra2n19c33584.1	-0.306419053	-0.350010175	0.118758655	0.22901885	Potri.019G087400.2	AT1G34220.2
Potra2n19c33585.2	0.088699829	-0.122135195	0.473988737	0.784407529	Potri.019G087200.1	AT1G71840.1
Potra2n19c33586.1	0.706136951	-0.00214134	4.59478E-05	0.999458638	Potri.019G087100.1	AT4G12600.1
Potra2n19c33587.3	-0.293316045	-0.041409869	0.056544695	0.966240998	Potri.019G087000.1	AT1G34260.1
Potra2n19c33588.1	-0.579618141	-0.215386422	1.43983E-05	0.696869044	Potri.019G086800.1	AT1G34270.1
Potra2n19c33589.1	0.525222834	-0.032462077	0.002410036	0.985393296	Potri.019G086700.1	AT1G71865.1
Potra2n19c33590.1	2.881158588	NA	NA	NA		
Potra2n19c33591.1	3.583257039	0.674963689	0.236067343	NA	Potri.019G086600.1	AT5G54990.1
Potra2n19c33592.1	1.715604634	1.886276382	0.418590473	NA	Potri.019G086500.1	AT5G54990.1
Potra2n19c33593.1					Potri.013G115900.1	AT1G29670.1
Potra2n19c33594.1	0.804150518	0.036788531	0.137566713	0.985393296	Potri.019G086400.1	AT1G34300.1
Potra2n19c33595.1	-0.297141815	0.121519475	0.641298858	0.9761362	Potri.019G086300.1	AT1G34300.1

Potra2n19c33596.1	2.601778008	0.12280143	0.223254086	NA	Potri.019G086200.1	AT1G34300.1
Potra2n19c33597.1	-0.317776378	0.48989395	0.518483956	0.789817821	Potri.019G086100.1	AT1G71870.1
Potra2n19c33598.1	-1.328951947	-1.478536539	NA	NA		
Potra2n19c33599.1	NA	1.427144052	NA	NA	Potri.019G086000.1	AT1G71860.3
Potra2n19c33600.1	0.130359387	-0.011654175	0.321257353	0.990138406	Potri.019G085900.2	AT1G34320.1
Potra2n19c33601.1	-0.291163397	0.684038485	0.394028691	NA	Potri.019G085800.1	AT1G22710.1
Potra2n19c33602.1	-1.734758351	-3.25356595	0.563500071	NA	Potri.019G085700.1	AT1G34340.1
Potra2n19c33603.1	-1.245182133	-0.614769262	6.85049E-06	0.622985715	Potri.019G085600.1	AT2G04890.1
Potra2n19c33604.1	0.077171687	-0.055620959	0.765716188	0.971572661	Potri.019G085500.1	AT1G34350.1
Potra2n19c33605.1	-2.1941051	0.114924087	0.437988252	NA	Potri.008G135100.2	AT1G68060.1
Potra2n19c33606.1	1.025004642	0.231294638	4.45258E-09	0.922618347	Potri.019G085400.1	AT1G34370.2
Potra2n19c33607.1	NA	NA	NA	NA	Potri.019G085000.1	
Potra2n19c33608.1	0.82357019	NA	NA	NA	Potri.008G220300.1	
Potra2n19c33609.1	-0.264317138	-0.035516145	0.215364523	0.98177802	Potri.019G085200.1	AT1G22700.1
Potra2n19c33610.1	-0.098249627	0.279968478	0.85307658	0.91105993	Potri.019G085200.1	AT1G22700.1
Potra2n19c33611.3	0.595587417	0.359861516	1.56736E-05	0.361949049	Potri.019G085100.1	AT5G61330.1
Potra2n19c33612.1	NA	NA	NA	NA		
Potra2n19c33613.1	0.248038285	-0.082014393	0.102919287	0.950095151	Potri.019G084900.1	AT1G34430.1
Potra2n19c33614.1	-0.351722387	-0.253018921	0.417932633	0.907073354		
Potra2n19c33615.1	0.794483152	0.080210655	0.000427869	0.973347974	Potri.019G084800.1	AT1G34420.1
Potra2n19c33616.2	-0.788121346	-0.067457658	0.141629983	0.985393296	Potri.019G084600.2	AT1G71900.1
Potra2n19c33617.1	4.626284655	1.381953902	0.009511728	NA	Potri.019G084500.1	AT1G71910.1
Potra2n19c33618.1	NA	NA	NA	NA	Potri.019G084400.1	AT1G77855.1
Potra2n19c33619.1	-0.529046646	-0.08390536	0.013216021	0.967122333	Potri.019G084100.1	AT1G34550.1
Potra2n19c33620.1	-0.580362221	-0.198734199	0.001048643	0.895460013	Potri.019G084200.1	
Potra2n19c33621.1	-2.674746828	0.742840959	0.424137674	NA	Potri.019G083900.1	AT1G22690.3
Potra2n19c33622.1	-0.285389302	0.246857808	0.425913766	0.876272842	Potri.019G083800.1	AT5G56150.2
Potra2n19c33623.1	-0.073855605	-0.051189649	0.477054451	0.941424651	Potri.019G083700.1	AT1G65280.1
Potra2n19c33624.2	0.568794293	-0.494975597	0.168850631	0.814981587	Potri.019G083600.1	AT1G71930.1
Potra2n19c33625.1	-0.012960183	-0.01131385	0.941427977	0.994403137	Potri.019G083500.1	AT1G71940.1
Potra2n19c33626.1	0.123298582	0.060914103	0.411106837	0.956865545	Potri.019G083400.1	AT1G35680.1
Potra2n19c33627.1	0.066620579	0.278620204	0.788413371	0.775618506	Potri.019G083300.1	AT1G71950.1

Potra2n19c33628.1	-0.509677582	-0.376903823	0.199990504	0.813731863	Potri.019G083200.1	AT4G09570.1
Potra2n19c33629.2	0.281142706	0.436556562	0.020203357	0.312622509	Potri.019G083100.1	AT1G35660.1
Potra2n19c33630.1	-0.325574271	-0.171744925	0.533388923	0.954400397	Potri.019G083000.1	AT1G71960.1
Potra2n19c33631.1	4.533195235	2.868663647	0.014711467	NA	Potri.019G082900.1	AT1G22680.1
Potra2n19c33632.1	1.675368699	0.095165554	0.000497479	0.985393296	Potri.019G082600.1	AT5G09520.1
Potra2n19c33633.1	1.830640851	0.145882025	0.000155856	0.978203092	Potri.019G082700.1	
Potra2n19c33634.1	-0.044418769	0.099982395	0.763557205	0.857374454	Potri.019G082500.1	AT1G71980.1
Potra2n19c33635.1	0.181637342	0.120737028	0.206313802	0.873955419	Potri.019G082400.1	AT1G35620.1
Potra2n19c33636.1	-0.122117189	-0.030569558	0.946777968	0.997811695	Potri.019G082300.1	
Potra2n19c33637.1	0.49823801	-0.039909261	0.000669015	0.978203092	Potri.019G082200.1	AT1G71990.1
Potra2n19c33638.1	NA	NA	NA	NA	Potri.019G082100.1	AT4G09530.1
Potra2n19c33639.1	0.24161038	0.066375894	0.117404738	0.941115972	Potri.019G082000.1	AT4G09510.1
Potra2n19c33640.1	0.042927282	0.195453154	0.853415584	0.824905557	Potri.019G081800.1	AT1G72010.1
Potra2n19c33641.1	-0.044397353	-0.093645536	0.802763301	0.924309478	Potri.013G110600.2	AT1G72020.1
Potra2n19c33642.1	NA	NA	NA	NA		
Potra2n19c33643.1	-0.342937482	-0.000914186	0.001056404	0.999458638	Potri.019G081600.1	AT1G72040.1
Potra2n19c33644.1						
Potra2n19c33645.1	0.387100319	-0.469770286	0.383255733	0.779077802	Potri.019G081500.1	AT1G22640.1
Potra2n19c33646.1	0.82357019	NA	NA	NA	Potri.005G203700.1	AT1G48120.1
Potra2n19c33647.1	0.208991034	-0.111556751	0.612545168	0.971593102	Potri.013G109100.1	AT1G14060.1
Potra2n19c33648.1	NA	1.871975723	NA	NA	Potri.019G081300.2	AT1G75240.1
Potra2n19c33649.1	0.09145813	0.144747941	0.78765889	0.943819961	Potri.019G081200.1	AT1G07400.1
Potra2n19c33650.1	0.475306401	0.135578298	0.016260588	0.8689387	Potri.019G081100.1	AT1G72050.2
Potra2n19c33651.1	-1.062536771	-0.054204649	8.44205E-05	0.976302215	Potri.019G081000.1	AT3G20230.1
Potra2n19c33652.1	-0.092754093	0.065653386	0.451807665	0.945769042	Potri.010G111500.1	AT2G01480.1
Potra2n19c33653.1	-0.042072735	0.043960154	0.717542214	0.965445938	Potri.019G080800.2	AT1G35470.2
Potra2n19c33654.1	5.061986558	1.363811008	0.003721642	NA	Potri.019G080700.1	AT4G25950.1
Potra2n19c33655.1	2.898255651	1.044262535	0.010550635	0.817452127	Potri.019G080600.1	AT1G72060.1
Potra2n19c33656.1	-1.843768208	0.768624386	0.000968014	0.776478926	Potri.019G080500.1	AT5G36110.1
Potra2n19c33657.1	-0.170569238	0.056199794	0.123624152	0.954476684	Potri.019G080300.2	AT1G22620.1
Potra2n19c33658.1	-0.385263557	0.735843497	0.554182919	0.828625141	Potri.013G107800.1	AT1G72090.1
Potra2n19c33659.1	-0.290296836	0.831330832	0.625608307	0.779493734	Potri.013G107800.1	AT1G72090.1

Potra2n19c33660.1	-0.31336791	0.778557596	0.626230676	0.817452127	Potri.019G080100.1	AT1G72090.1
Potra2n19c33661.1	-0.047628022	-0.124236633	0.902149403	0.956865545	Potri.019G080000.1	AT1G22610.1
Potra2n19c33662.1	-0.979381066	-0.531417665	NA	NA	Potri.019G109925.1	AT1G05700.1
Potra2n19c33663.1	-0.968747451	-0.108407382	2.3662E-06	0.935474933	Potri.019G079900.4	AT1G35460.1
Potra2n19c33664.1	-0.29972302	0.228280219	0.193169008	0.920174237	Potri.019G079800.1	AT1G35430.1
Potra2n19c33665.4	-1.242637817	0.229970944	0.020416815	0.953108412	Potri.019G079700.1	AT1G22540.1
Potra2n19c33666.1	-0.761098389	-0.303528108	5.17254E-06	0.595538573	Potri.019G079500.1	AT1G22530.1
Potra2n19c33667.1	NA	0.674963689	NA	NA	Potri.019G079400.1	
Potra2n19c33668.1	-0.173212286	-0.038018641	0.296262199	0.981772014	Potri.019G079300.1	AT1G72160.1
Potra2n19c33669.1	-0.158147499	-0.001773867	0.405495693	0.999458638		
Potra2n19c33670.1	1.841896402	1.013786481	4.25598E-08	0.626019376	Potri.014G001500.1	AT1G78955.1
Potra2n19c33671.1	1.651706702	1.363811008	NA	NA		
Potra2n19c33672.1	0.178157299	0.095254594	0.1159008	0.907073354	Potri.019G079101.1	AT1G22520.2
Potra2n19c33673.1	-2.531039333	-0.514586181	1.90848E-12	0.797861066	Potri.019G079000.1	AT1G35420.2
Potra2n19c33674.1	-0.232722256	0.024360003	0.283960507	0.986636967	Potri.019G078800.5	AT1G72175.1
Potra2n19c33675.5	0.05547462	0.335829285	0.774215098	0.66599469	Potri.019G078700.2	AT4G09140.1
Potra2n19c33676.1	1.545832463	2.032498096	NA	NA	Potri.019G078600.1	AT5G36110.1
Potra2n19c33677.2	-0.905015661	-0.034299079	0.001518044	0.974756511	Potri.019G078400.1	AT1G72180.1
Potra2n19c33678.1	2.684428911	1.802542659	3.11053E-13	0.225576181	Potri.019G078300.1	AT5G47850.1
Potra2n19c33679.1	-0.019141693	-0.174598157	0.940160916	0.848005416	Potri.019G078200.4	AT5G47840.1
Potra2n19c33680.1						
Potra2n19c33681.1	-0.076637187	1.005757528	0.910554063	0.48698575	Potri.013G103900.1	AT1G75280.1
Potra2n19c33682.1	-0.877595159	-0.25491101	0.005697386	0.917516356	Potri.019G078000.1	
Potra2n19c33683.1	NA	0.674963689	NA	NA	Potri.019G077900.1	AT5G49910.1
Potra2n19c33684.1	1.59753422	1.196020749	0.639738704	NA	Potri.004G224400.1	AT5G49910.1
Potra2n19c33685.2	-0.607801515	-0.169501562	0.000105217	0.799049555	Potri.019G077700.1	AT1G58350.2
Potra2n19c33686.1	0.078622511	0.618798819	0.809337753	0.779201817	Potri.019G077600.1	AT5G49650.1
Potra2n19c33687.2	-0.043020637	0.120525445	0.720768989	0.824905557	Potri.019G077500.1	AT1G33980.1
Potra2n19c33688.2	0.036938704	0.132464396	0.801525362	0.798429987	Potri.019G077400.2	AT4G09900.1
Potra2n19c33689.1	-0.434963106	-0.054389398	0.000462671	0.965249957	Potri.019G077300.2	AT1G33970.1
Potra2n19c33690.1	NA	3.97626828	NA	NA	Potri.019G077200.1	AT4G09960.1
Potra2n19c33691.1	-0.279880222	-0.292793594	0.134398486	0.778396015	Potri.019G077100.1	AT1G09645.1

Potra2n19c33692.1	-0.311613358	-0.171606377	0.004282226	0.792511865	Potri.019G077000.1	AT1G09640.1
Potra2n19c33693.1	3.29094823	NA	NA	NA	Potri.019G076800.1	AT1G71692.1
Potra2n19c33694.1					Potri.019G076700.1	AT5G04885.1
Potra2n19c33695.1	-1.286180515	-1.271270068	0.313968673	0.846680547	Potri.019G076600.1	AT1G71691.2
Potra2n19c33696.1	1.849368488	0.253070875	0.0543677	0.97754057		
Potra2n19c33697.1	0.164471563	0.04617832	0.268166237	0.971317865	Potri.019G076400.1	AT1G33810.1
Potra2n19c33698.1	-0.763508362	-0.304087744	0.004569967	0.864223715	Potri.019G076300.1	AT1G33800.1
Potra2n19c33699.1					Potri.T153700.1	AT1G71820.2
Potra2n19c33700.1	0.419005595	0.204029419	0.023867563	0.848131816	Potri.019G076200.1	
Potra2n19c33701.1	-0.000569395	-0.334368623	0.999189233	0.814981587	Potri.T112601.1	AT1G22840.1
Potra2n19c33702.1	0.458565261	-0.004115182	0.004359958	0.998245864	Potri.T056301.1	AT1G22840.1
Potra2n19c33703.1	0.158357707	0.227926642	0.336661492	0.767368598	Potri.019G075900.1	AT1G05670.1
Potra2n19c33704.1	-0.754536784	0.020784189	0.008988346	0.992934677	Potri.019G075700.2	AT1G24160.1
Potra2n19c33705.1	5.054958943	2.316428477	1.26923E-07	0.798429987	Potri.019G075600.1	AT1G33760.1
Potra2n19c33706.1	-0.460444772	0.973599345	0.519303071	0.642417298	Potri.019G075500.1	AT1G71450.1
Potra2n19c33707.1	-0.20544866	-0.333378795	0.631578648	0.79522599	Potri.019G075400.1	AT1G71460.1
Potra2n19c33708.1	NA	NA	NA	NA	Potri.011G087400.1	
Potra2n19c33709.1	-4.074830118	-4.945815962	0.257659775	NA		
Potra2n19c33710.1	-1.748787037	-2.72278699	0.709868393	NA	Potri.019G075300.1	AT3G44540.1
Potra2n19c33711.1						
Potra2n19c33712.1						
Potra2n19c33713.1	-1.881679658	-2.032537384	NA	NA	Potri.019G075300.1	AT3G44540.1
Potra2n19c33714.1	NA	1.571529889	NA	NA		
Potra2n19c33715.1	NA	1.363811008	NA	NA		
Potra2n19c33716.1	NA	2.676364087	NA	NA	Potri.019G075300.1	AT3G44540.1
Potra2n19c33717.1	NA	NA	NA	NA		
Potra2n19c33718.1	-3.751849916	-3.181689592	0.397618609	NA	Potri.019G075200.1	AT3G44540.1
Potra2n19c33719.1	1.038614066	0.128703453	0.002941898	0.972159498	Potri.019G074600.1	AT4G10030.1
Potra2n19c33720.1	0.086143822	0.035575496	0.703021897	0.986636967	Potri.019G074700.1	AT1G71490.1
Potra2n19c33721.1	0.90483635	0.157471326	0.201001213	0.977559521		
Potra2n19c33722.1	1.439425065	0.108773427	0.046856425	0.988210253	Potri.019G074400.1	AT4G32480.1
Potra2n19c33723.1	1.818485101	0.675427124	7.11425E-06	0.543814189	Potri.019G069500.1	AT5G17680.1

Potra2n19c33724.1	1.693695949	0.692152796	0.091483056	0.890446513	Potri.019G069500.1	AT5G17680.1
Potra2n19c33725.1	0.228268995	0.21778491	0.81753104	0.966006233	Potri.019G070700.2	AT5G17680.1
Potra2n19c33726.1	0.033600089	0.805829526	NA	NA	Potri.019G070350.1	AT5G45230.1
Potra2n19c33727.1	1.104576413	0.450885439	2.97184E-05	0.420046656	Potri.T077100.1	AT5G17680.1
Potra2n19c33728.1	0.631842689	0.395933628	0.030528589	0.74044311	Potri.T077100.1	AT5G17680.1
Potra2n19c33729.1	2.215492267	1.270601279	8.34588E-12	0.145028933		
Potra2n19c33730.1	0.227937259	0.776426801	0.743855747	0.605100478	Potri.019G069901.1	AT4G19470.1
Potra2n19c33731.1	-0.843409032	1.483278708	0.725817643	NA	Potri.019G069901.1	AT4G19470.1
Potra2n19c33732.1	-0.099796177	0.325381458	0.875519592	0.87458517	Potri.T077000.3	AT5G17680.1
Potra2n19c33733.1	-1.317292482	-0.037599366	NA	NA		
Potra2n19c33734.1	0.79951374	0.647884793	0.00071857	0.158136359	Potri.019G069600.2	AT4G12010.1
Potra2n19c33735.1	0.488485734	-0.748597007	0.758244159	NA	Potri.T077600.1	AT4G12010.1
Potra2n19c33736.1	NA	NA	NA	NA	Potri.019G070900.3	AT5G53390.1
Potra2n19c33737.1	-0.129072728	0.101538669	0.412359866	0.940376196	Potri.T076900.2	AT4G10100.1
Potra2n19c33738.1						
Potra2n19c33739.1	1.458549989	-0.747177386	0.021653402	0.816392132	Potri.019G070700.2	AT5G17680.1
Potra2n19c33740.2	0.861000855	0.059483396	0.001198502	0.984556994	Potri.019G074600.1	AT4G10030.1
Potra2n19c33741.1	1.144747926	-0.123176433	0.02403351	0.982422288	Potri.019G074400.1	AT4G32480.1
Potra2n19c33742.1	1.330781223	0.743490331	0.370859797	0.91392603	Potri.019G074000.1	AT3G14460.1
Potra2n19c33743.1	-0.651608883	-0.229082909	0.032180784	0.877460254	Potri.019G073900.2	AT1G71500.1
Potra2n19c33744.1	1.772378913	-0.05574223	0.546591712	NA	Potri.019G075000.1	AT3G44540.1
Potra2n19c33745.1	3.559718771	1.082182836	5.62692E-34	0.216627903	Potri.019G073800.1	AT1G70170.1
Potra2n19c33746.1	1.818715821	0.027550167	1.13336E-09	0.992934677	Potri.019G073700.1	AT1G70170.1
Potra2n19c33747.1	1.03856094	-0.274093941	1.95868E-06	0.825454445	Potri.019G073800.1	AT1G70170.1
Potra2n19c33748.1	1.333268373	-0.157438957	1.55606E-08	0.937687311	Potri.019G073800.1	AT1G70170.1
Potra2n19c33749.1	2.807104776	1.97520653	0.110838543	0.792511865	Potri.019G073300.1	AT1G22810.1
Potra2n19c33750.1	-0.585369118	0.072708032	0.789261195	NA	Potri.019G073100.1	AT1G71530.1
Potra2n19c33751.5	0.104143329	0.355244993	0.632493589	0.749144604	Potri.019G073000.1	AT1G33780.1
Potra2n19c33752.1	0.23489337	0.038533041	0.053674274	0.966148753	Potri.019G072900.1	AT4G10050.1
Potra2n19c33753.1	-0.707768779	-0.179861772	9.11585E-05	0.919424623	Potri.019G072800.1	AT5G49900.1
Potra2n19c33754.3	0.094162358	0.217964649	0.581391081	0.741833788	Potri.019G072700.1	AT1G71440.1
Potra2n19c33755.1	NA	NA	NA	NA		

Potra2n19c33756.1	-0.034641135	0.011048227	0.800428102	0.991909603	Potri.019G072500.1	AT4G10070.1
Potra2n19c33757.1	-0.159101307	0.044683321	0.381641517	0.976888173	Potri.019G072300.10	AT1G31480.1
Potra2n19c33758.2	-0.345945607	-0.10988293	0.041854072	0.905025315	Potri.019G071900.1	AT1G22850.1
Potra2n19c33759.1	-0.652232146	-0.284969662	0.039337993	0.851986664	Potri.019G072100.1	
Potra2n19c33760.1	-0.084950347	0.075126732	0.551813492	0.930203195	Potri.019G071700.1	AT4G19710.2
Potra2n19c33761.1	0.199212279	-1.491523487	0.938098085	NA	Potri.019G071600.2	AT1G07160.1
Potra2n19c33762.1	0.416863648	0.082924876	5.80382E-05	0.914238347	Potri.019G071400.2	AT3G02760.1
Potra2n19c33763.1	0.074024054	-0.052889812	0.631188274	0.953608209	Potri.019G071300.1	AT4G13530.1
Potra2n19c33764.1	2.978495374	0.275445517	0.004787655	NA	Potri.019G071200.2	AT2G26710.1
Potra2n19c33765.1	NA	NA	NA	NA		
Potra2n19c33766.1	1.722865303	0.306809969	3.3767E-06	0.902676973		
Potra2n19c33767.1					Potri.019G071000.1	AT5G53390.1
Potra2n19c33768.1	2.426648654	1.474374821	0.002532521	0.276620704	Potri.019G071000.1	AT5G53390.1
Potra2n19c33769.1	0.82357019	1.388295429	NA	NA	Potri.019G070900.3	AT5G53390.1
Potra2n19c33770.1	-0.158741586	0.071194889	0.232458753	0.956610193	Potri.T076900.2	AT4G10100.1
Potra2n19c33771.1						
Potra2n19c33772.1	NA	NA	NA	NA	Potri.019G070900.3	AT5G53390.1
Potra2n19c33773.1	0.068689459	0.21525406	0.728901367	0.848131816	Potri.T076900.2	AT4G10100.1
Potra2n19c33774.1	0.82357019	NA	NA	NA		
Potra2n19c33775.1	1.054470742	-0.031123687	0.175184209	0.996032145	Potri.T077000.3	AT5G17680.1
Potra2n19c33776.1	-2.93141954	-0.716054677	NA	NA	Potri.T077100.1	AT5G17680.1
Potra2n19c33777.1					Potri.019G070460.1	AT5G17680.1
Potra2n19c33778.1	0.404399537	1.47714047	0.923652819	NA	Potri.T077100.1	AT5G17680.1
Potra2n19c33779.1	0.669422433	0.370143754	0.019366791	0.653581422	Potri.019G070460.1	AT5G17680.1
Potra2n19c33780.1	2.256397311	0.240411228	0.217263035	NA	Potri.T077100.1	AT5G17680.1
Potra2n19c33781.1	0.329923679	0.233801259	0.153171178	0.852284301	Potri.T077100.1	AT5G17680.1
Potra2n19c33782.1	-0.075791616	0.233221676	0.895509603	0.918311446	Potri.019G070350.1	AT5G45230.1
Potra2n19c33783.1	-0.433338736	0.555172856	0.505574057	0.747657496	Potri.019G069500.1	AT5G17680.1
Potra2n19c33784.1	1.327121681	0.454335289	0.019957176	0.919722977	Potri.019G070700.2	AT5G17680.1
Potra2n19c33785.1	0.382207391	0.191264662	0.097655241	0.881137749	Potri.T077100.1	AT5G17680.1
Potra2n19c33786.1	0.985429534	0.818162309	0.068535645	0.663037599	Potri.019G069901.1	AT4G19470.1
Potra2n19c33787.1	1.251375303	0.385066407	0.530785202	NA	Potri.T077400.2	AT4G12010.1

Potra2n19c33788.1	0.68827825	0.549992268	0.000355851	0.253052485	Potri.019G069500.1	AT5G17680.1
Potra2n19c33789.1	0.608211503	0.384227728	0.025414775	0.577490326		
Potra2n19c33790.1	-0.40733804	-0.388311536	0.219362184	0.779077802	Potri.019G069300.1	AT1G71380.1
Potra2n19c33791.1	0.532247458	0.26452012	0.076214452	0.720242695	Potri.019G069200.1	AT5G17680.1
Potra2n19c33792.1	-0.061893318	0.02534539	0.743452998	0.976302215	Potri.019G069000.1	AT4G10110.1
Potra2n19c33793.1	0.209113791	-0.050103501	0.683005675	0.974275337	Potri.019G068900.5	AT1G71360.1
Potra2n19c33794.1	2.900414336	NA	NA	NA	Potri.019G068801.1	AT5G66770.1
Potra2n19c33795.1	NA	NA	NA	NA	Potri.019G068801.1	AT5G66770.1
Potra2n19c33796.3	0.088234742	0.037448586	0.405950731	0.979094708	Potri.019G068700.1	AT1G59610.1
Potra2n19c33797.1	4.021878885	1.571529889	0.136062833	NA	Potri.019G068600.1	AT2G05250.1
Potra2n19c33798.1	-0.42305433	-0.088916307	0.110581732	0.94795983	Potri.019G068500.1	AT1G71310.2
Potra2n19c33799.1	-0.142837013	-0.079992152	0.190191976	0.912777843	Potri.019G068400.1	AT1G33470.2
Potra2n19c33800.3	0.096621747	0.192071775	0.729987948	0.850863374	Potri.019G068200.1	AT5G17680.1
Potra2n19c33801.1	-0.360301925	0.037130244	0.328832674	0.987226805		
Potra2n19c33802.1	2.340207562	6.43705385	NA	0.075045852	Potri.019G068100.1	AT5G16370.1
Potra2n19c33803.1	-0.533086718	-0.765034843	0.605601715	0.874808359	Potri.019G067800.1	AT5G16370.1
Potra2n19c33804.1	-0.036965825	0.730596622	0.947767069	0.68015285	Potri.019G067800.1	AT5G16370.1
Potra2n19c33805.1	-3.279216522	-1.850018649	NA	NA	Potri.019G067600.1	AT1G71250.1
Potra2n19c33806.1	-1.819483623	-0.292932651	9.62299E-11	0.813575895	Potri.019G067500.1	AT1G59740.1
Potra2n19c33807.1	-0.829578334	-0.433571308	0.321257353	0.918687733		
Potra2n19c33808.1	-0.802651208	-0.459620323	0.314796706	0.915580711	Potri.019G067400.1	AT5G25810.1
Potra2n19c33809.1	3.515418547	4.08181845	0.395777094	NA	Potri.019G067200.1	AT3G07820.1
Potra2n19c33810.1	1.545832463	NA	NA	NA	Potri.019G066700.1	AT3G07820.1
Potra2n19c33811.1	-1.92220879	-1.350456732	NA	NA	Potri.019G067200.1	AT3G07820.1
Potra2n19c33812.1	2.476290371	NA	NA	NA	Potri.019G066700.1	AT3G07820.1
Potra2n19c33813.1	NA	0.674963689	NA	NA		
Potra2n19c33814.1	NA	0.674963689	NA	NA	Potri.019G067200.1	AT3G07820.1
Potra2n19c33815.1	0.699096783	0.005199667	0.711505172	NA		
Potra2n19c33816.1	-1.409983009	-0.114960142	NA	NA	Potri.008G157700.1	AT1G04200.1
Potra2n19c33817.1	-0.6019575	0.933578569	0.376992636	0.805461106	Potri.010G011600.1	AT2G35040.1
Potra2n19c33818.1	0.608083563	0.559439894	NA	NA	Potri.019G066700.1	AT3G07820.1
Potra2n19c33819.2	0.371821288	0.043302494	0.001073602	0.960981446	Potri.019G066600.2	AT1G77350.1

Potra2n19c33820.1	0.031658963	0.320525278	0.856757383	0.672348168	Potri.019G066500.1	AT1G21651.1
Potra2n19c33821.1	-0.06743985	0.209947731	0.769204039	0.833651746		
Potra2n19c33822.1	0.597721075	0.031256347	0.004030314	0.989304496	Potri.019G066200.2	AT3G19770.1
Potra2n19c33823.1	-1.57254816	-1.21988859	0.034430674	0.755774501	Potri.019G066100.1	AT3G27090.1
Potra2n19c33824.1					Potri.019G066000.1	AT4G10350.1
Potra2n19c33825.1	NA	NA	NA	NA	Potri.019G065900.1	AT1G17680.2
Potra2n19c33826.1	4.008528813	1.381953902	0.273384631	NA	Potri.019G065800.1	AT2G24550.1
Potra2n19c33827.1	0.603255962	0.201295344	1.38341E-07	0.605100478	Potri.019G065700.2	AT3G56460.1
Potra2n19c33828.3	0.523932007	0.320803861	1.08063E-06	0.55464068	Potri.019G065500.1	AT2G40815.1
Potra2n19c33829.2	0.252540008	0.130065219	0.119670507	0.886633917	Potri.019G065300.1	AT3G09720.1
Potra2n19c33830.1	3.7871296	-0.685368309	1.45133E-08	NA	Potri.019G065200.1	AT1G03220.1
Potra2n19c33831.1	-0.625584793	-1.495886149	NA	NA		
Potra2n19c33832.1	3.705566943	0.28032209	1.63165E-13	0.96478728	Potri.019G065000.1	AT1G03220.1
Potra2n19c33833.1	2.448730605	-1.495886149	0.1976689	NA		
Potra2n19c33834.1	2.939012119	0.651571454	0.236616206	NA	Potri.019G065000.1	AT1G03220.1
Potra2n19c33835.1	2.442813499	0.274580593	1.5871E-05	0.948002367	Potri.019G065100.1	AT1G03220.1
Potra2n19c33836.1	2.55080631	0.674963689	NA	NA		
Potra2n19c33837.1	NA	NA	NA	NA	Potri.019G064300.1	AT1G03220.1
Potra2n19c33838.1	-0.514493367	0.287833241	0.152703755	0.886265374		
Potra2n19c33839.1	-0.498454352	0.341506528	0.157186416	0.843683654	Potri.019G064200.1	AT5G24910.1
Potra2n19c33840.1	0.22422677	0.351869877	0.546119324	0.794641467	Potri.019G064300.1	AT1G03220.1
Potra2n19c33841.1	0.261102522	0.294529675	0.722936626	0.935099709	Potri.019G064200.1	AT5G24910.1
Potra2n19c33842.2	-0.255361977	-0.090922307	0.016953718	0.916403589	Potri.019G064100.1	AT3G54190.1
Potra2n19c33843.1	0.154778904	0.787922346	0.936772151	NA		
Potra2n19c33844.1	-0.125378028	-0.028135643	0.346194857	0.985393296	Potri.019G064000.2	AT3G14172.1
Potra2n19c33845.2	0.391854528	0.185601015	0.155599531	0.910477984	Potri.019G063900.2	AT5G38520.1
Potra2n19c33846.1	0.598521164	0.453977903	0.021506345	0.84487051	Potri.019G063800.1	AT1G33330.1
Potra2n19c33847.1	-0.513101262	-1.761581511	0.707772375	NA	Potri.019G063700.1	AT1G33340.1
Potra2n19c33848.1	-1.650139182	-1.135591845	0.000407002	0.34511071	Potri.019G063600.2	AT4G10260.1
Potra2n19c33849.1	0.166756183	0.279710949	0.374037783	0.701745625		
Potra2n19c33850.1	0.524535519	-0.0961316	0.303523245	0.98177802	Potri.015G113800.2	AT5G45470.1
Potra2n19c33851.1	3.083389401	0.227103762	1.34877E-40	0.896593025	Potri.019G063500.2	AT3G26590.1

Potra2n19c33852.1	0.516843496	1.141652887	0.051033529	0.208715733	Potri.019G063400.1	AT5G44400.1
Potra2n19c33853.1	-0.618669512	0.038048666	0.001411187	0.985393296	Potri.019G063300.1	AT4G10330.1
Potra2n19c33854.1					Potri.T099300.1	AT3G01190.1
Potra2n19c33855.1	-0.489011618	-0.092606055	0.0144865	0.960656795	Potri.019G063100.1	AT4G10340.1
Potra2n19c33856.1	1.545832463	NA	NA	NA	Potri.019G063000.1	AT1G79580.2
Potra2n19c33857.1						
Potra2n19c33858.1	1.459962178	NA	NA	NA	Potri.019G063000.1	AT1G79580.2
Potra2n19c33859.1	-1.947761941	-1.163060825	0.240858562	NA	Potri.019G133600.3	AT3G56380.1
Potra2n19c33860.1	-0.097529946	0.118824221	0.550423589	0.911094206	Potri.019G062800.2	AT2G40810.1
Potra2n19c33861.1	0.454107785	0.501433938	0.000627115	0.534000676	Potri.019G062600.1	AT3G10850.1
Potra2n19c33862.3	0.264259387	0.050808699	0.008400297	0.949966783	Potri.T099700.5	AT1G18070.2
Potra2n19c33863.1	2.15406803	1.592819577	0.012269848	0.664564719		
Potra2n19c33864.1	0.173728247	0.469806925	0.835549608	0.857374454	Potri.006G242500.1	AT1G12700.1
Potra2n19c33865.3	-0.323751494	-0.143432922	0.036189218	0.887496314	Potri.019G062100.1	AT5G58300.1
Potra2n19c33866.1	-3.367109651	-0.998565406	0.051164474	0.83521351	Potri.019G062000.1	AT3G08710.2
Potra2n19c33867.1	1.416515192	-1.613018931	0.455221088	NA	Potri.019G061900.1	AT5G43360.1
Potra2n19c33868.1	-2.337106966	-2.486725844	NA	NA	Potri.016G138400.1	AT3G08720.1
Potra2n19c33869.1	-0.267624764	-0.189582018	0.44969219	0.831463341	Potri.019G061700.1	AT3G10870.1
Potra2n19c33870.1	1.459962178	2.315796285	NA	NA		
Potra2n19c33871.1	0.69306754	0.07874651	0.130667418	0.958152146		
Potra2n19c33872.1	1.400185291	1.004262996	1.93596E-19	0.000870939	Potri.019G061400.1	AT5G05190.1
Potra2n19c33873.1	-0.350514169	-0.068679879	0.016769754	0.949485515	Potri.019G061300.1	AT5G05200.1
Potra2n19c33874.1	-0.105028463	-0.132719971	0.532549229	0.874507876	Potri.019G061200.1	AT2G40780.1
Potra2n19c33875.1	-0.064837404	0.251161803	0.646100787	0.605100478	Potri.019G060900.1	AT2G40770.1
Potra2n19c33876.1						
Potra2n19c33877.1	-0.168189346	0.000460912	0.8444881	0.999937671	Potri.002G086300.1	AT3G42150.1
Potra2n19c33878.1	4.395628623	2.283425176	0.027063626	NA	Potri.013G090400.1	AT2G40740.1
Potra2n19c33879.1	-0.016344016	0.022753982	0.922088811	0.987226805	Potri.019G059200.1	AT2G40730.1
Potra2n19c33880.1	0.142007116	0.02312486	0.435809195	0.985313207	Potri.019G059100.1	AT2G40700.1
Potra2n19c33881.1	1.946957916	0.544350645	0.013488596	0.813731863	Potri.019G059000.1	
Potra2n19c33882.1	1.956012318	0.544350645	NA	0.813731863	Potri.019G058900.1	AT3G56380.1
Potra2n19c33883.1	-0.053679876	-0.068031155	0.702333587	0.93466433	Potri.019G058700.1	AT2G40660.1

Potra2n19c33884.1	-0.201033584	-0.119499184	0.537228981	0.940014069	Potri.019G058600.1	AT3G56370.1
Potra2n19c33885.1	0.035488111	0.06620268	0.931400783	0.971828097		
Potra2n19c33886.1						
Potra2n19c33887.1	1.0984032	0.397716175	7.38426E-09	0.485694701	Potri.019G058400.1	AT3G51580.1
Potra2n19c33888.1	-0.390373147	-0.031285825	0.000646775	0.966434509	Potri.019G058300.1	AT5G05230.1
Potra2n19c33889.1	0.688245137	-0.023733206	0.08481778	0.995005375	Potri.019G058200.1	AT5G05240.1
Potra2n19c33890.1	-2.24328228	-0.28247307	8.41881E-16	0.917814867	Potri.001G422600.1	AT3G56360.1
Potra2n19c33891.2	3.662802866	3.029124375	0.36293887	NA	Potri.006G131100.1	AT5G03040.3
Potra2n19c33892.1	9.007552081	4.513815074	2.12891E-34	0.365910084	Potri.019G057900.1	AT5G36110.1
Potra2n19c33893.1	0.82357019	NA	NA	NA	Potri.T134800.1	AT5G59480.1
Potra2n19c33894.1	1.850667051	0.666318448	6.32116E-18	0.408090023	Potri.T134750.1	AT5G05270.2
Potra2n19c33895.3	-1.118051585	-0.210164799	3.34737E-10	0.779077802	Potri.019G057700.1	AT3G10910.1
Potra2n19c33896.4	-0.332160652	0.067878834	0.713971333	0.988601759	Potri.013G091400.1	AT2G40620.1
Potra2n19c33897.1	-0.524384415	-0.341272181	0.596640011	0.953831244	Potri.019G057550.1	AT2G40620.1
Potra2n19c33898.1	3.250110679	0.637020957	1.57702E-19	0.813977047	Potri.019G057500.3	AT2G40610.1
Potra2n19c33899.1	0.373787635	0.341189192	2.16235E-05	0.293583922	Potri.019G057400.1	AT3G10915.2
Potra2n19c33900.1	0.944368454	-0.021312922	1.45739E-05	0.989304496	Potri.019G057300.1	AT3G10920.1
Potra2n19c33901.1	0.415044485	0.071300388	0.02107939	0.972159498	Potri.019G057200.1	AT1G09795.1
Potra2n19c33902.1	2.409567423	NA	NA	NA	Potri.019G057100.1	AT5G47540.1
Potra2n19c33903.2	0.712327297	0.065855864	3.25446E-05	0.967940508	Potri.019G057000.1	AT2G40510.1
Potra2n19c33904.1	-0.142669626	0.266918139	0.253745914	0.719713755	Potri.019G056800.1	AT3G56320.1
Potra2n19c33905.1	-0.131526353	0.132607952	0.400959251	0.935474933	Potri.019G056700.1	AT3G56310.1
Potra2n19c33906.1	0.379072912	-0.266336198	0.496725742	0.948834049		
Potra2n19c33907.1	2.315713269	1.871975723	NA	NA		
Potra2n19c33908.1	-0.41979971	0.044194372	0.205331388	0.98177802	Potri.019G056500.2	AT2G40540.2
Potra2n19c33909.1	-1.367374485	-0.346795779	0.242631361	0.941677175	Potri.019G056400.2	AT3G56290.1
Potra2n19c33910.1	0.568552398	0.180339054	0.00011426	0.809914834	Potri.019G056300.4	AT5G01230.1
Potra2n19c33911.1	0.623477597	0.028724317	0.029876255	0.987577016	Potri.019G056200.1	AT2G40550.1
Potra2n19c33912.1	6.358706155	-0.623066104	2.23452E-09	0.958883625	Potri.019G056000.1	AT3G05550.1
Potra2n19c33913.1	-0.448432861	-0.167557943	0.001578334	0.862824894	Potri.019G055900.1	AT3G56250.1
Potra2n19c33914.1	-1.026437078	-0.135365532	4.19775E-08	0.760398319	Potri.019G055800.1	AT2G40460.1
Potra2n19c33915.1	NA	1.871975723	NA	NA	Potri.018G019500.1	AT1G69440.1

Potra2n19c33916.1	0.136782346	-0.037577755	0.157046364	0.974275337	Potri.019G055600.1	AT5G17010.3
Potra2n19c33917.1	-0.860527511	-0.071054832	0.006084294	0.973347974	Potri.019G055500.1	AT5G65660.1
Potra2n19c33918.1	-0.09499615	-0.121423292	0.571614947	0.835819291		
Potra2n19c33919.1	0.595608351	0.190744175	0.000108862	0.851395707	Potri.019G055300.1	AT1G65290.1
Potra2n19c33920.1	3.884084757	1.990136471	4.92845E-77	0.147107193	Potri.019G055200.2	AT1G08450.1
Potra2n19c33921.1	-0.118322035	-0.047785409	0.426686831	0.964971544	Potri.019G055100.1	AT1G65270.2
Potra2n19c33922.5	-0.025654456	0.122227484	0.86168005	0.894138111	Potri.019G055000.1	AT3G03340.1
Potra2n19c33923.1	-0.178857857	-0.034138865	0.181229333	0.985912891	Potri.019G054900.1	AT3G02740.1
Potra2n19c33924.1	-0.451844046	0.012360707	0.018043711	0.995005375	Potri.019G054800.1	AT3G02730.1
Potra2n19c33925.2	-0.090641314	0.194382716	0.520755457	0.773063484	Potri.019G054600.1	AT1G24706.1
Potra2n19c33926.1	-2.379490373	-0.529066348	NA	NA	Potri.019G045900.1	AT1G08810.1
Potra2n19c33927.1	0.421454023	-0.018722869	0.000601502	0.989300076	Potri.019G054500.3	AT3G24090.1
Potra2n19c33928.2	0.709863592	0.08043381	0.000974866	0.945254452	Potri.019G054400.2	AT5G16390.1
Potra2n19c33929.1	NA	NA	NA	NA	Potri.001G193800.1	AT2G01050.1
Potra2n19c33930.1					Potri.T083200.1	AT2G01050.1
Potra2n19c33931.1	0.173621047	0.064966344	0.413158087	0.974367223	Potri.019G054300.2	AT4G12910.1
Potra2n19c33932.1	-0.06908804	-0.073054048	0.591750072	0.951752151	Potri.019G054200.3	AT3G02750.1
Potra2n19c33933.1	-0.045468058	0.097487379	0.737294388	0.926808461	Potri.019G054000.1	AT3G47390.2
Potra2n19c33934.1	0.189253386	0.16731913	0.158610989	0.720242695	Potri.019G053800.1	AT5G16450.1
Potra2n19c33935.1	1.225849984	0.33429145	2.79397E-18	0.519527448	Potri.019G053700.1	AT3G02780.1
Potra2n19c33936.1	0.065171526	0.066285671	0.582305085	0.937846405	Potri.019G053600.1	AT5G36230.1
Potra2n19c33937.1	-0.152467301	-0.034107375	0.243135941	0.977559521	Potri.019G053500.1	AT3G02790.1
Potra2n19c33938.3	-0.172331503	0.037268789	0.314848954	0.974275337	Potri.019G053400.1	AT5G36290.2
Potra2n19c33939.1	-0.541969631	-0.040263343	0.002546257	0.987226805	Potri.019G053300.1	AT5G16490.1
Potra2n19c33940.1	1.13457761	-1.465929376	NA	NA		
Potra2n19c33941.1	0.046593575	0.027206315	0.612647819	0.98177802	Potri.019G053200.1	AT3G02830.1
Potra2n19c33942.1	-0.407579252	-0.126201858	0.099236479	0.895717156	Potri.019G053100.1	AT1G65295.1
Potra2n19c33943.1	-0.123009605	0.149211405	0.407351403	0.84487051	Potri.019G053000.1	AT4G18530.1
Potra2n19c33944.1	0.82357019	1.363811008	NA	NA	Potri.004G054700.1	
Potra2n19c33945.1	0.040437313	-1.465929376	NA	NA	Potri.019G052800.1	AT5G16530.1
Potra2n19c33946.3	0.038152672	0.053677689	0.804112853	0.967103157	Potri.019G052600.1	AT1G65320.1
Potra2n19c33947.1	0.963339549	-0.089973309	0.003659459	0.946457495	Potri.019G052500.2	AT1G50240.2

Potra2n19c33948.1	0.701991712	0.387389662	9.70118E-06	0.713032256	Potri.T058000.1	AT1G50240.2
Potra2n19c33949.1	-0.051698223	-0.077300895	0.640974799	0.898711301	Potri.019G052400.1	AT5G17070.1
Potra2n19c33950.1	-0.22296374	-0.352010024	0.799981734	0.930871994	Potri.019G052300.1	AT5G36100.1
Potra2n19c33951.1	0.282827905	0.115651851	0.029876255	0.89257034	Potri.019G052200.1	AT5G17060.1
Potra2n19c33952.1	-0.023621376	-0.047948541	0.845735739	0.966434509	Potri.019G052100.1	AT5G17020.1
Potra2n19c33953.1	0.011639431	-0.073975834	0.952443548	0.953831244		
Potra2n19c33954.1	-0.22316474	-0.013329217	0.001115397	0.989304496	Potri.019G051700.1	AT5G16510.1
Potra2n19c33955.1	-0.636311025	-0.659514525	0.602358553	0.903496619	Potri.019G051600.1	AT3G07070.1
Potra2n19c33956.1	-0.044776893	-0.005052599	0.69224244	0.996032145	Potri.019G051500.1	AT3G16090.1
Potra2n19c33957.1	0.138042269	0.001327413	0.315411266	0.999458638	Potri.019G051400.2	AT3G03100.1
Potra2n19c33958.1	-0.330709756	0.285487004	0.006951284	0.584487746	Potri.019G051300.1	AT3G59390.2
Potra2n19c33959.1	-1.148718369	-0.052180619	2.99109E-05	0.969020904	Potri.019G051200.1	AT4G17900.1
Potra2n19c33960.1	-0.386311595	0.044980073	0.002219829	0.978410516	Potri.019G048600.1	AT3G10940.1
Potra2n19c33961.1	-0.192887449	0.096994309	0.028399895	0.921015963	Potri.019G048700.3	AT5G05310.2
Potra2n19c33962.1						
Potra2n19c33963.1	-0.917110207	-0.305969515	1.53274E-10	0.71231959		
Potra2n19c33964.1	0.105876057	-0.124457582	0.644475994	0.947317412	Potri.019G048900.2	AT2G40490.1
Potra2n19c33965.1	-0.015507317	0.071958443	0.928672397	0.949485515	Potri.T071100.1	AT3G10970.1
Potra2n19c33966.1	-1.447266966	-0.947054714	0.001617319	0.611349996	Potri.019G049100.1	AT5G05330.1
Potra2n19c33967.1	-1.940251548	-0.644625654	4.65129E-10	0.576091828	Potri.T071300.1	AT2G40480.1
Potra2n19c33968.1	0.33418733	-0.034209952	0.05829479	0.983446961	Potri.T071400.1	AT1G08580.1
Potra2n19c33969.1	-0.067393489	0.14046743	0.659358209	0.827804213	Potri.T071500.1	AT5G27270.1
Potra2n19c33970.1	3.5673075	0.848956433	6.61328E-18	0.805461106	Potri.019G049500.2	AT1G65060.1
Potra2n19c33971.1	NA	0.674963689	NA	NA	Potri.019G049600.1	AT5G16920.1
Potra2n19c33972.1	0.591150092	0.126613831	0.022611216	0.853147297	Potri.019G049700.1	AT3G03050.1
Potra2n19c33973.1	-0.040498171	0.061267488	0.784365987	0.930871994	Potri.019G049800.1	AT5G16890.1
Potra2n19c33974.1	-0.11465315	-0.04351898	0.20777547	0.95671968	Potri.019G050000.1	AT5G16880.1
Potra2n19c33975.1	NA	2.676364087	NA	NA	Potri.019G097640.1	AT5G46470.1
Potra2n19c33976.1	-0.009430302	-0.045878787	0.961725555	0.959881296	Potri.019G048100.1	AT3G03140.1
Potra2n19c33977.1	0.691552921	0.260876465	1.09229E-05	0.619954942	Potri.019G048200.1	AT2G16790.1
Potra2n19c33978.1					Potri.005G133200.1	
Potra2n19c33979.1	1.64774763	NA	NA	NA		

Potra2n19c33980.1	0.82357019	NA	NA	NA	Potri.014G004200.2	
Potra2n19c33981.1	-0.33852436	-0.033712861	0.141729549	0.985964146	Potri.019G048300.1	AT5G17210.1
Potra2n19c33982.1	-0.447517338	0.002017098	0.102779903	0.999458638		
Potra2n19c33983.1	3.338937571	3.207114396	2.58759E-05	0.289079975	Potri.013G136730.1	ATCG00750.1
Potra2n19c33984.1	2.955745568	2.952753425	0.000218266	0.368305629	Potri.013G140700.1	ATCG00770.1
Potra2n19c33985.1	2.832500446	2.825055487	0.002809937	0.49278277	Potri.009G095100.1	AT4G15480.1
Potra2n19c33986.1	-0.607612345	1.449267194	NA	NA		
Potra2n19c33987.1	-0.027847045	0.101238903	0.911424294	0.933996725	Potri.019G046400.1	AT1G03250.1
Potra2n19c33988.1	-2.901582317	0.486910521	NA	NA	Potri.019G045900.1	AT1G08810.1
Potra2n19c33989.1	-0.512063768	-0.260735376	0.251210928	0.911749053	Potri.019G045800.1	AT3G03630.1
Potra2n19c33990.1	5.158825113	3.840849509	2.08455E-28	0.002915668	Potri.019G043600.1	AT5G17540.1
Potra2n19c33991.1	NA	NA	NA	NA		
Potra2n19c33992.1	-0.185865752	-0.00196317	0.487249193	0.999325699	Potri.019G044600.1	AT3G45400.1
Potra2n19c33993.1	-0.634781408	0.135466733	0.722872444	0.985393296		
Potra2n19c33994.1	-0.746038835	-0.130443159	0.625029229	0.986636967	Potri.013G067300.2	AT5G17770.1
Potra2n19c33995.3	1.168797704	0.969849256	0.603700101	NA	Potri.019G044100.1	AT5G17620.1
Potra2n19c33996.1	-0.338870251	0.093096453	0.076606774	0.953831244	Potri.019G044200.1	AT1G08540.1
Potra2n19c33997.2	-0.097096015	0.24733793	0.572363404	0.586851629	Potri.019G043800.1	AT5G35930.1
Potra2n19c33998.1					Potri.019G043700.1	AT5G17590.1
Potra2n19c33999.1	5.10881347	3.92833973	3.15775E-48	1.15031E-06	Potri.019G043600.1	AT5G17540.1
Potra2n19c34000.1	0.512839291	0.279620663	0.000360365	0.560708883	Potri.019G043400.6	AT5G35980.1
Potra2n19c34001.1	-1.948378959	-0.903108239	6.93776E-23	0.016502768	Potri.019G043300.1	AT1G64860.1
Potra2n19c34002.1	-0.087916596	0.227718896	0.805991647	0.824905557	Potri.019G043101.1	AT5G14770.1
Potra2n19c34003.1	-0.004737637	0.101298671	0.978887696	0.871025364	Potri.019G043000.1	AT1G64890.1
Potra2n19c34004.1	-0.214236485	0.437620353	0.512242836	0.784063198	Potri.019G042900.1	AT1G09815.1
Potra2n19c34005.1	-0.221558459	-0.277734505	0.220184202	0.679660122		
Potra2n19c34006.1	-0.045997509	-0.095674599	0.718956335	0.885375133	Potri.019G042800.1	AT5G17510.1
Potra2n19c34007.1	-0.118159461	-0.022700595	0.390124548	0.983446961	Potri.019G042700.1	AT4G27610.2
Potra2n19c34008.1	-0.436905734	-0.024924088	0.011897932	0.989304496	Potri.019G042600.1	AT5G17170.1
Potra2n19c34009.1	0.808658235	-0.098705911	0.054360555	0.952344644	Potri.019G042500.1	AT1G64980.1
Potra2n19c34010.1	3.255719224	3.399920631	NA	NA	Potri.019G042300.1	AT3G13960.1
Potra2n19c34011.2	-0.493232803	0.032903135	0.002573692	0.978203092	Potri.013G077700.1	AT4G27630.2

Potra2n19c34012.1	0.026394834	-0.046512893	0.859813396	0.96332588	Potri.019G042100.1	AT5G60570.1
Potra2n19c34013.1	1.664677056	NA	NA	NA		
Potra2n19c34014.3	0.100914103	0.271434806	0.36120152	0.636664382	Potri.019G041900.3	AT5G16680.1
Potra2n19c34015.1	-0.068180235	0.0252034	0.593296721	0.981772014	Potri.019G041700.2	AT5G16660.1
Potra2n19c34016.1	0.250233513	0.46628449	0.415809463	0.80848715	Potri.019G041600.1	AT1G22430.1
Potra2n19c34017.1	-0.173452058	0.147735528	0.454161158	0.895460013	Potri.019G041400.1	AT5G16650.1
Potra2n19c34018.1	0.046970004	-0.041017082	0.766650815	0.958874237	Potri.019G041300.1	AT5G62030.1
Potra2n19c34019.1	0.09738138	-0.157698621	0.727526633	0.849446235	Potri.019G041100.1	AT3G02910.1
Potra2n19c34020.1	8.219392075	2.939174151	2.05636E-11	NA	Potri.T080900.1	AT1G16260.2
Potra2n19c34021.2	0.650262938	0.319594133	0.003861042	0.790362046	Potri.003G185600.1	AT1G79690.1
Potra2n19c34022.1	2.275054632	1.203413662	0.007150226	0.794641467	Potri.019G040900.1	AT1G69560.1
Potra2n19c34023.1	NA	0.674963689	NA	NA	Potri.019G040800.2	AT3G03660.2
Potra2n19c34024.1	-1.49342874	0.089000059	0.000241606	0.974782554	Potri.019G040700.1	AT3G47500.1
Potra2n19c34025.1	1.220477444	-0.007718237	1.52826E-08	0.996032145	Potri.019G040500.1	AT3G04440.1
Potra2n19c34026.1	0.309369486	-0.153984842	0.140568594	0.898853929	Potri.019G040400.1	AT5G17840.1
Potra2n19c34027.1					Potri.007G056100.1	AT5G39850.1
Potra2n19c34028.1	3.48490206	0.91443671	1.60143E-24	0.663950227	Potri.019G040500.1	AT3G04440.1
Potra2n19c34029.1	0.116215396	0.521119785	0.570831101	0.108800306	Potri.013G065900.2	AT5G17850.1
Potra2n19c34030.1	2.235464379	0.31003855	0.016720822	0.962696825	Potri.019G040200.1	AT5G17860.1
Potra2n19c34031.1	-0.86719665	-0.211520638	0.001222053	0.769714337	Potri.019G040100.10	AT3G03710.1
Potra2n19c34032.1	0.774243812	0.200272764	0.000891066	0.895460013	Potri.019G040000.1	AT5G27740.1
Potra2n19c34033.1	0.131692254	-0.08930838	0.259349238	0.903934429	Potri.019G039900.1	AT5G17900.1
Potra2n19c34034.1	-0.379685137	-0.016883398	0.090531929	0.992934677	Potri.019G039700.1	AT1G58030.1
Potra2n19c34035.1	-0.13150939	0.17818112	0.55420194	0.813731863	Potri.019G039600.1	AT3G03720.1
Potra2n19c34036.1	-0.169741399	0.263431722	0.462913928	0.679365931		
Potra2n19c34037.1	-0.077024238	0.15156755	0.829666474	0.924256651		
Potra2n19c34038.1	-0.041643871	-0.005375856	0.839745279	0.996154416	Potri.019G039500.1	AT3G03740.1
Potra2n19c34039.5	-0.238172347	0.435034172	0.194766639	0.341783585	Potri.019G039300.6	AT1G28760.1
Potra2n19c34040.1	-2.888796463	0.400643049	0.047067263	0.937846405		
Potra2n19c34041.1	-0.102003097	-0.062718473	0.331675801	0.943819961	Potri.019G039200.4	AT2G02760.1
Potra2n19c34042.1	1.006160613	1.933314724	0.393267139	0.338903894	Potri.019G039000.1	AT3G03770.2
Potra2n19c34043.1	4.160842927	-0.608157407	2.64228E-18	0.881062472	Potri.T069600.1	AT5G39890.1

Potra2n19c34044.1	3.893055691	NA	0.161877718	NA		
Potra2n19c34045.1	0.219378139	0.018203262	0.071130999	0.990874899	Potri.T069700.1	AT5G35910.1
Potra2n19c34046.1	0.32314651	0.190856421	0.184031738	0.865611082	Potri.T069800.1	AT3G03773.1
Potra2n19c34047.1	-0.354645455	-1.140505961	0.88232345	NA		
Potra2n19c34048.1	-0.084709611	0.088455913	0.423402018	0.884463725	Potri.019G038400.2	AT5G55000.2
Potra2n19c34049.1	-0.138256397	0.419519885	0.710059734	NA		
Potra2n19c34050.2	-0.069559424	-0.224187761	0.80041397	0.910815461	Potri.019G038200.7	AT5G17920.1
Potra2n19c34051.1	1.106290349	0.441278461	1.36502E-05	0.635323636	Potri.T070300.1	AT3G62800.2
Potra2n19c34052.1	0.71481202	0.907239084	0.85777295	NA		
Potra2n19c34053.1	1.803932143	1.965357877	3.03476E-06	0.175898575	Potri.T070300.1	AT3G62800.2
Potra2n19c34054.1	0.764607286	0.483026752	0.458388461	0.922618347	Potri.T070300.1	AT3G62800.2
Potra2n19c34055.1	0.41757007	1.094398514	0.780907958	0.83433596	Potri.019G038000.1	
Potra2n19c34056.2	-0.089119435	0.154966576	0.490416661	0.786005488	Potri.T070800.1	AT3G62800.1
Potra2n19c34057.1					Potri.001G155750.1	AT1G21280.1
Potra2n19c34058.1						
Potra2n19c34059.1					Potri.019G037800.1	AT3G17675.1
Potra2n19c34060.1	NA	NA	NA	NA	Potri.019G037700.1	AT1G54400.1
Potra2n19c34061.2	-0.093994168	-0.966136087	0.855140511	0.18388493	Potri.001G105300.2	AT1G63450.1
Potra2n19c34062.1	-0.107964741	-0.330273086	0.678769155	0.825493963	Potri.019G037600.2	AT5G32450.1
Potra2n19c34063.1	1.523772449	0.139386737	NA	NA	Potri.019G037500.1	
Potra2n19c34064.1	-0.172527806	0.444423005	0.777562457	0.920028955	Potri.019G037400.1	AT5G20950.1
Potra2n19c34065.2	-0.718051982	0.573479027	0.181694266	0.848005416	Potri.019G037300.5	
Potra2n19c34066.1					Potri.019G037100.1	AT3G14470.1
Potra2n19c34067.1	NA	NA	NA	NA	Potri.003G026900.1	AT3G14460.1
Potra2n19c34068.1	-0.126462128	0.021446004	0.350966574	0.986636967	Potri.019G036900.1	AT5G45520.1
Potra2n19c34069.1	-0.241796314	0.050876012	0.101440531	0.977530072	Potri.019G036700.1	AT3G03800.1
Potra2n19c34070.1	-0.224781329	0.039474937	0.076455226	0.977323635	Potri.019G036600.1	AT3G03810.1
Potra2n19c34071.1	0.655550238	0.070757016	2.16377E-05	0.957668459	Potri.019G036500.1	AT4G38040.1
Potra2n19c34072.1						
Potra2n19c34073.1	4.577257002	1.698501518	0.000675254	NA	Potri.019G036400.1	AT3G13540.1
Potra2n19c34074.2	-3.162023981	-3.310868886	NA	NA	Potri.019G036200.1	AT5G19820.1
Potra2n19c34075.1	NA	NA	NA	NA	Potri.019G036200.1	AT5G19820.1

Potra2n19c34076.1	0.253038968	-2.42671106	0.961890434	NA		
Potra2n19c34077.1	-2.146127372	-1.139036885	0.208579697	0.918722297	Potri.019G036100.1	AT5G19790.1
Potra2n19c34078.1	2.406357145	2.177393172	0.252241702	NA		
Potra2n19c34079.2	-0.576675106	0.213692795	0.001055434	0.900514809	Potri.019G036000.1	AT5G19780.1
Potra2n19c34080.1	-0.339633222	0.066478288	0.026656105	0.947547562	Potri.019G035800.2	AT5G18100.1
Potra2n19c34081.2	-0.424153036	-0.185680171	0.276252481	0.930598202		
Potra2n19c34082.2	-0.107010624	0.04865934	0.48019861	0.961670176	Potri.019G035700.1	AT3G03860.1
Potra2n19c34083.1	-0.34906691	0.033330097	0.180932417	0.982453292	Potri.019G035600.1	AT5G18130.1
Potra2n19c34084.1	-0.272686778	-0.032575462	0.216539107	0.977559521	Potri.019G035300.1	AT1G55340.1
Potra2n19c34085.1	0.000228234	-0.220109573	0.999346752	0.657855643	Potri.019G035200.1	AT3G03890.1
Potra2n19c34086.1	NA	NA	NA	NA		
Potra2n19c34087.1	-0.395429219	0.012043185	0.605332844	0.998084737	Potri.019G035100.1	AT5G18140.1
Potra2n19c34088.1	0.454419149	0.056253745	0.239594769	0.972336961	Potri.019G035000.1	AT5G18150.1
Potra2n19c34089.1	-2.007422625	-0.495540436	1.9936E-07	0.779493734	Potri.019G034800.10	AT5G18170.1
Potra2n19c34090.1	0.829560236	0.1054071	0.001552595	0.954476684	Potri.019G034900.1	AT3G03920.1
Potra2n19c34091.1	1.555601084	1.911098625	0.022290401	0.531719758	Potri.019G034700.2	AT4G20970.1
Potra2n19c34092.1	3.325083945	2.994874146	1.4354E-07	0.399733072	Potri.019G034700.2	AT4G20970.1
Potra2n19c34093.1	0.157893588	-0.065267803	0.152223338	0.943483421	Potri.019G034500.2	AT3G03940.1
Potra2n19c34094.1	NA	1.363811008	NA	NA	Potri.019G034600.1	AT4G04220.1
Potra2n19c34095.1	-0.105485267	-0.297083994	0.774770581	0.800037157		
Potra2n19c34096.1	0.113167252	-0.186311577	0.563526298	0.842247476	Potri.019G034400.1	AT5G18200.1
Potra2n19c34097.1	0.608665232	0.13607289	0.00011004	0.895740429	Potri.019G034300.2	AT3G13460.1
Potra2n19c34098.1	-1.328951947	0.139386737	NA	NA		
Potra2n19c34099.1	0.157356277	-0.042512408	0.231901753	0.959881296	Potri.019G034200.1	AT3G03960.1
Potra2n19c34100.1	0.359354568	-0.854496809	0.624676953	0.807871531	Potri.019G034100.1	AT1G64770.1
Potra2n19c34101.1	-2.181158351	-0.807500731	0.083450804	0.842247476	Potri.019G034000.1	AT1G55370.2
Potra2n19c34102.2	0.831099022	-0.456734583	0.209958527	0.897285619	Potri.019G033900.1	AT1G54385.2
Potra2n19c34103.1	-0.382855768	-0.305391971	0.009814413	0.472351144	Potri.019G033800.1	AT1G54390.4
Potra2n19c34104.1	0.338906734	0.192798894	0.015698672	0.767368598	Potri.019G033600.1	AT3G03980.1
Potra2n19c34105.1	0.147346386	0.23453274	0.374670872	0.767368598	Potri.019G033500.1	AT3G03980.1
Potra2n19c34106.1						
Potra2n19c34107.2	-0.268795864	-0.049121379	0.044827394	0.953608209		

Potra2n19c34108.1	-0.136539451	0.562425092	0.785717275	0.657878257	Potri.019G033200.1	AT2G23840.1
Potra2n19c34109.1	-0.733841219	-0.4361287	0.086307752	0.714411171	Potri.019G033000.1	AT4G32890.1
Potra2n19c34110.1	-1.160943246	-0.197740153	3.7027E-06	0.914903229	Potri.019G032900.2	AT1G64760.1
Potra2n19c34111.1	0.048117371	-0.02959637	0.677879086	0.977165266	Potri.019G032800.4	AT5G18230.1
Potra2n19c34112.1	0.389009162	0.074837459	0.842389964	0.994283386	Potri.019G032700.1	AT3G04030.3
Potra2n19c34113.1	NA	NA	NA	NA		
Potra2n19c34114.1	-0.060349706	2.134625936	0.954540191	0.371998415	Potri.019G032600.1	AT5G56350.1
Potra2n19c34115.3	-0.077811026	2.11404709	0.940854462	0.377476554	Potri.019G032500.3	AT5G56340.1
Potra2n19c34116.1	0.496575529	0.728687866	0.022841269	0.420332337	Potri.019G032400.1	
Potra2n19c34117.1	-0.25708864	-0.01390298	0.084440997	0.9896961	Potri.019G032300.2	AT5G18260.1
Potra2n19c34118.1					Potri.019G032200.1	
Potra2n19c34119.1	NA	NA	NA	NA	Potri.013G061100.2	AT2G32080.1
Potra2n19c34120.1	0.036240443	0.477479979	0.928387777	0.835627384	Potri.019G031900.1	AT1G08570.1
Potra2n19c34121.1	0.364374565	0.885739215	0.29454942	0.297164129	Potri.019G031800.1	AT2G33260.1
Potra2n19c34122.1	-1.313668028	-0.077990725	0.000128626	0.983113084	Potri.013G054300.2	AT1G09230.1
Potra2n19c34123.1	NA	NA	NA	NA	Potri.T001800.1	AT1G21980.1
Potra2n19c34124.1	1.64774763	NA	NA	NA	Potri.T003100.1	AT1G21980.1
Potra2n19c34125.1	3.493688714	0.827724002	2.51304E-06	NA	Potri.019G097580.1	AT4G11170.1
Potra2n19c34126.1	-0.523346857	-0.420646022	0.124859905	0.673884433	Potri.019G031600.1	AT5G18270.1
Potra2n19c34127.1	-0.344613161	-0.284002664	0.165494809	0.817452127	Potri.019G031500.1	AT2G39050.1
Potra2n19c34128.1	2.489984876	1.047992946	3.54783E-05	0.49278277	Potri.019G031400.1	AT3G04070.2
Potra2n19c34129.1	-0.234504303	-0.040418394	0.133954174	0.98197198	Potri.019G031000.2	AT5G18280.1
Potra2n19c34130.2	1.373467012	-0.21755432	4.42121E-05	0.921099436	Potri.019G030900.1	AT3G04090.1
Potra2n19c34131.1	-0.809611557	0.008743381	0.078271457	0.996851113	Potri.019G030700.1	AT1G08550.2
Potra2n19c34132.1	-0.474300789	0.118453169	0.317285831	0.95172055		
Potra2n19c34133.1	-0.489261189	0.32589409	0.257037095	0.857189843	Potri.013G053100.2	AT1G08550.2
Potra2n19c34134.1	-0.670853053	-0.27404887	0.28276905	0.896516863	Potri.013G053100.2	AT1G08550.2
Potra2n19c34135.1	-0.635907907	-0.158295951	0.281903718	0.954559968	Potri.013G053100.2	AT1G08550.2
Potra2n19c34136.1	-0.088161498	0.246094118	0.908725546	0.917751882	Potri.013G053100.2	AT1G08550.2
Potra2n19c34137.1	-0.725622857	0.087218191	0.397344419	0.978827428	Potri.013G053100.2	AT1G08550.2
Potra2n19c34138.1	-0.052862951	0.304984275	0.830029064	0.605100478	Potri.014G003600.3	AT3G14460.1
Potra2n19c34139.1	0.26719382	0.289149046	0.545618023	0.865611082	Potri.019G030400.1	AT1G08530.1

Potra2n19c34140.1	0.289441395	0.483223556	0.747677927	0.902847514		
Potra2n19c34141.1	-0.026059895	0.229036624	0.925593883	0.784407529	Potri.014G005400.3	AT3G14460.1
Potra2n19c34142.1	0.037972105	0.272404184	0.936605582	0.860810425	Potri.019G030400.1	AT1G08530.1
Potra2n19c34143.1	-0.290523952	0.039775901	0.146013946	0.974367223	Potri.019G030400.1	AT1G08530.1
Potra2n19c34144.1	-0.800140434	-0.11112009	4.52818E-11	0.936549944	Potri.019G030300.2	AT2G38430.1
Potra2n19c34145.1	-0.288266158	-0.07522809	0.414615417	0.977227089	Potri.012G123550.1	AT1G16470.2
Potra2n19c34146.1	0.120541376	0.010121334	0.546074051	0.995565508	Potri.012G122900.1	AT3G14470.1
Potra2n19c34147.1						
Potra2n19c34148.1	-0.439443041	0.022749223	0.212868806	0.992934677		
Potra2n19c34149.1	-0.246095694	-0.517730606	0.631639946	0.725595998	Potri.019G029800.1	AT2G38430.1
Potra2n19c34150.1	-0.70761609	-0.279126513	0.015434824	0.817452127	Potri.019G029800.1	AT2G38430.1
Potra2n19c34151.1	-0.755067758	-0.121183916	0.070322926	0.957668459	Potri.019G029800.1	AT2G38430.1
Potra2n19c34152.1	-1.013110191	-0.097933136	0.070635718	0.975887373	Potri.019G030300.2	AT2G38430.1
Potra2n19c34153.1	-1.062882323	-0.102995867	0.019813573	0.974275337	Potri.019G029800.1	AT2G38430.1
Potra2n19c34154.1	0.67390036	-0.606383497	0.099059608	0.715585895	Potri.019G029800.1	AT2G38430.1
Potra2n19c34155.1	0.746383887	-0.111249543	1.75436E-05	0.897389232	Potri.019G029800.1	AT2G38430.1
Potra2n19c34156.2	1.825172094	0.004099396	1.09916E-16	0.999315506	Potri.019G029600.2	AT5G18310.2
Potra2n19c34157.1	0.121089445	0.045313052	0.678013661	0.971992625	Potri.019G029500.1	AT3G07360.1
Potra2n19c34158.1	-0.37045013	0.086272576	0.08769822	0.937146435		
Potra2n19c34159.1	-0.409683297	0.071326077	0.024150085	0.940503323	Potri.019G029400.4	AT3G04160.1
Potra2n19c34160.1					Potri.013G051700.1	AT3G05950.1
Potra2n19c34161.1	-0.295002307	-0.101676439	0.086673215	0.953608209	Potri.019G029200.1	AT1G64200.1
Potra2n19c34162.1	6.695082961	3.308335477	1.99639E-31	5.66957E-05	Potri.019G028800.1	AT4G13440.1
Potra2n19c34163.1	4.300045931	0.674963689	0.093324563	NA	Potri.T056000.1	AT4G13440.1
Potra2n19c34164.1	1.545832463	0.674963689	NA	NA		
Potra2n19c34165.1	NA	NA	NA	NA	Potri.001G155750.1	AT1G21280.1
Potra2n19c34166.1					Potri.T056100.1	AT4G13440.1
Potra2n19c34167.1	0.259397108	-0.884892609	0.945128599	NA	Potri.019G026400.2	AT3G05950.1
Potra2n19c34168.1	0.263845789	-0.777708568	0.926862433	NA	Potri.019G026100.1	AT3G05950.1
Potra2n19c34169.1	-0.402116162	-0.107635955	0.087292825	0.932447954	Potri.019G025700.1	AT5G18390.1
Potra2n19c34170.1	-0.32823181	-0.066946435	0.001330879	0.909715408	Potri.019G025600.2	AT3G04240.1
Potra2n19c34171.1	1.711635273	0.409112336	1.90969E-15	0.802631112	Potri.019G025500.1	AT5G63930.1

Potra2n19c34172.1	-0.269946015	0.115656263	0.146658765	0.921099436	Potri.019G025200.2	AT5G18410.2
Potra2n19c34173.1	-0.313688485	-0.033663673	0.056826347	0.982422288		
Potra2n19c34174.1	-1.107359594	-0.437089758	6.88938E-13	0.221840363	Potri.019G025100.1	AT3G04260.1
Potra2n19c34175.1					Potri.019G025000.1	AT5G26594.1
Potra2n19c34176.1					Potri.019G024900.1	AT5G26594.1
Potra2n19c34177.1	NA	NA	NA	NA	Potri.019G024800.1	AT5G33370.1
Potra2n19c34178.1	NA	1.571529889	NA	NA	Potri.019G024700.1	AT5G33370.1
Potra2n19c34179.1	NA	NA	NA	NA	Potri.019G024600.1	AT5G33370.1
Potra2n19c34180.1	-1.35927387	-1.507841271	NA	NA	Potri.013G051000.1	AT3G04290.1
Potra2n19c34181.2	0.349082417	0.209093716	0.002703304	0.556125138	Potri.019G024200.1	AT1G64790.1
Potra2n19c34182.3	-1.468142485	0.06288878	0.179794309	0.99114586	Potri.019G024100.1	AT5G18460.1
Potra2n19c34183.2	-0.074594864	-0.161769549	0.608500788	0.844277327	Potri.019G024000.1	AT3G06050.1
Potra2n19c34184.1	0.812745358	0.211356702	0.015464452	0.90405476	Potri.019G023900.2	AT1G54870.1
Potra2n19c34185.1	-0.527260543	-0.095423734	0.017192847	0.948002367	Potri.019G023700.1	AT5G37360.1
Potra2n19c34186.1					Potri.019G023600.1	AT1G64780.1
Potra2n19c34187.1	NA	NA	NA	NA	Potri.019G023600.1	AT1G64780.1
Potra2n19c34188.1	-0.238215185	0.178189062	0.499499861	0.943819961	Potri.001G018100.1	AT2G39725.2
Potra2n19c34189.1	-0.251905621	-0.047160126	0.12633743	0.974367223	Potri.019G023400.1	AT3G04310.1
Potra2n19c34190.1	-0.331797516	-0.065637655	0.00574194	0.938713299	Potri.019G022700.1	AT5G32470.1
Potra2n19c34191.1	-1.160019514	-0.233337211	7.14518E-07	0.915339763	Potri.019G022000.1	AT5G04370.1
Potra2n19c34192.1	NA	NA	NA	NA	Potri.019G021900.5	AT5G06450.1
Potra2n19c34193.1	-0.233177907	-0.661174246	0.586077264	0.621109642	Potri.019G021800.1	AT4G33270.1
Potra2n19c34194.1	NA	NA	NA	NA	Potri.019G021700.1	AT1G08590.1
Potra2n19c34195.1	-0.219985884	0.062029793	0.21841219	0.956865545	Potri.019G021500.1	AT1G08600.4
Potra2n19c34196.1	1.980225385	0.164869539	3.2355E-06	0.95999889	Potri.019G021400.1	AT2G02540.1
Potra2n19c34197.1	-0.145286136	-0.018387909	0.542173157	0.987402575	Potri.019G021200.1	AT1G12700.1
Potra2n19c34198.1	-0.053440603	0.136459636	0.761378137	0.916192318	Potri.019G020900.10	AT5G29000.2
Potra2n19c34199.1	5.188100452	1.738724289	4.43476E-48	0.607523318	Potri.019G020700.1	AT5G40010.1
Potra2n19c34200.1	5.356190522	3.145099921	0.003106037	NA	Potri.019G020100.1	AT2G21840.1
Potra2n19c34201.3	-0.223092732	0.03355748	0.488537403	0.989304496	Potri.019G020000.1	AT3G04460.1
Potra2n19c34202.1	1.454374458	1.035386122	2.8842E-08	0.118866455	Potri.019G020100.1	AT2G21840.1
Potra2n19c34203.1	0.476458832	0.51321256	0.04371363	0.432051484	Potri.019G020000.1	AT3G04460.1

Potra2n19c34204.1	0.275332681	0.054974528	0.211045835	0.972464243	Potri.019G020100.1	AT2G21840.1
Potra2n19c34205.1	1.937977155	0.441010242	0.000229732	0.914162682	Potri.019G020000.1	AT3G04460.1
Potra2n19c34206.1	0.614294508	0.860490119	0.08364112	0.294386607		
Potra2n19c34207.1	1.311857699	0.887591461	3.41658E-14	0.178681813		
Potra2n19c34208.1	4.907720841	2.780026555	0.001056534	NA	Potri.011G087400.1	
Potra2n19c34209.2	-0.157834171	0.02951522	0.739267202	0.992934677	Potri.019G020100.1	AT2G21840.1
Potra2n19c34210.2	0.082881747	0.192370698	0.737212924	0.895474078	Potri.019G020000.1	AT3G04460.1
Potra2n19c34211.1						
Potra2n19c34212.1	-0.246133907	0.050412178	0.004781769	0.954476684	Potri.019G019800.1	AT5G28910.2
Potra2n19c34213.1	-0.151105882	0.099386223	0.067968958	0.910815461	Potri.019G019700.1	AT3G04470.1
Potra2n19c34214.1	0.256853175	0.077899347	0.011123767	0.935099709	Potri.019G019600.2	AT3G04480.1
Potra2n19c34215.1	-0.138356183	-0.080479055	0.141353324	0.937276488	Potri.019G019500.1	AT5G28850.2
Potra2n19c34216.1	2.249249553	3.514704656	NA	NA	Potri.013G047100.1	AT1G10095.1
Potra2n19c34217.1	-0.643865584	0.468377737	0.167507503	0.810799242	Potri.019G019300.1	AT3G04760.1
Potra2n19c34218.1	0.122078167	-0.040071114	0.584418234	0.974194594		
Potra2n19c34219.1	0.371389962	0.130542264	0.000727244	0.782245062	Potri.019G019100.1	AT3G04490.1
Potra2n19c34220.1	-0.295099819	-0.135367294	0.088162084	0.848005416	Potri.019G018900.1	AT3G04500.1
Potra2n19c34221.1	1.22331563	0.536783646	5.89106E-11	0.302692927	Potri.019G018800.1	AT5G36930.2
Potra2n19c34222.1	1.761615252	0.602635423	3.54965E-13	0.51571418	Potri.001G028700.1	AT5G36930.2
Potra2n19c34223.1	1.824910109	0.703984256	1.14798E-20	0.234967741		
Potra2n19c34224.1	-0.172381972	0.050373121	0.157923732	0.960981446	Potri.019G018700.1	AT1G08620.1
Potra2n19c34225.1	1.64774763	NA	NA	NA		
Potra2n19c34226.1	-0.634728261	-0.285562259	6.86128E-07	0.666699452	Potri.019G018600.1	AT5G28490.1
Potra2n19c34227.1	NA	NA	NA	NA		
Potra2n19c34228.1	1.003825539	0.553579153	4.43086E-09	0.592872379	Potri.019G018500.1	AT1G08630.4
Potra2n19c34229.1	0.369608092	0.239319339	8.00228E-06	0.739192712	Potri.019G018400.2	AT1G09770.1
Potra2n19c34230.1	0.711584097	0.25633375	1.07695E-06	0.70570456	Potri.019G018300.1	AT1G08640.1
Potra2n19c34231.1	8.170399279	NA	1.07939E-11	NA	Potri.019G018200.2	AT3G23250.1
Potra2n19c34232.1	-0.046241607	-0.240631531	0.855394969	0.849489745	Potri.019G018100.1	AT3G04530.1
Potra2n19c34233.1	NA	NA	NA	NA		
Potra2n19c34234.1	-0.059963104	0.09445243	0.646626545	0.896306549	Potri.019G018000.1	AT2G39840.1
Potra2n19c34235.1	0.997974138	0.264865744	6.87195E-05	0.802942736	Potri.019G017900.1	AT2G20410.1

Potra2n19c34236.1	8.805557136	5.944505948	1.2822E-13	NA		
Potra2n19c34237.1						
Potra2n19c34238.1	7.669094571	4.443769731	6.79725E-10	NA	Potri.019G017850.1	
Potra2n19c34239.1					Potri.017G052700.1	ATCG00340.1
Potra2n19c34240.1	2.735991243	NA	NA	NA		
Potra2n19c34241.1	0.173461831	0.030582809	0.376258173	0.985393296	Potri.019G017900.1	AT2G20410.1
Potra2n19c34242.1	9.953852979	7.199192051	7.12305E-19	0.432156259		
Potra2n19c34243.3	0.82357019	3.215633166	NA	NA		
Potra2n19c34244.1	-1.903049284	0.114325273	NA	NA	Potri.019G016500.1	AT5G23960.2
Potra2n19c34245.1	NA	0.674963689	NA	NA	Potri.T072400.1	AT1G68040.1
Potra2n19c34246.1						
Potra2n19c34247.1						
Potra2n19c34248.2	1.459962178	NA	NA	NA	Potri.019G045100.1	AT5G23960.1
Potra2n19c34249.1	-0.652183226	-0.855183578	0.007932632	0.003507774	Potri.017G044300.1	
Potra2n19c34250.1	0.533832767	0.165184796	0.013844618	0.911106947	Potri.019G017900.1	AT2G20410.1
Potra2n19c34251.1	8.836534258	6.002054352	2.91059E-12	0.305728522	Potri.019G017600.1	
Potra2n19c34252.1	2.250728707	-0.191585755	0.168071866	NA	Potri.019G017150.1	
Potra2n19c34253.1	3.219950245	3.404223726	NA	NA		
Potra2n19c34254.1	2.340207562	2.253733581	NA	NA	Potri.019G016500.1	AT5G23960.2
Potra2n19c34255.1	NA	NA	NA	NA		
Potra2n19c34256.1	2.632524198	2.872807905	NA	NA	Potri.T139200.1	AT1G68040.1
Potra2n19c34257.1					Potri.T138900.1	
Potra2n19c34258.1					Potri.T138950.1	
Potra2n19c34259.1	-2.983195238	-2.405857034	NA	NA	Potri.T139000.1	
Potra2n19c34260.1	4.713370065	1.111503671	3.14658E-23	NA	Potri.T139100.1	
Potra2n19c34261.1	0.781440944	0.371937707	0.79011766	NA		
Potra2n19c34262.1	6.09158583	2.631066719	6.79336E-31	0.489780897	Potri.019G015600.1	
Potra2n19c34263.1	-0.541206402	0.650003991	0.790321042	NA	Potri.019G015500.1	AT3G23240.1
Potra2n19c34264.1	0.82357019	NA	NA	NA	Potri.T050700.1	AT2G26710.1
Potra2n19c34265.1	-0.659282106	0.519749458	0.441420111	0.900637717	Potri.T050800.1	AT2G26560.1
Potra2n19c34266.1	-0.229187329	0.048316578	0.300623271	0.978203092	Potri.T051000.5	AT2G14050.1
Potra2n19c34267.1	3.996089094	1.502265498	0.009905164	NA	Potri.019G014900.1	AT4G37060.1

Potra2n19c34268.1	-0.720485257	-0.483319749	0.640809858	0.960981446	Potri.T051200.3	AT4G37070.2
Potra2n19c34269.1	3.385008575	-0.052371924	0.003423193	NA	Potri.T051100.1	AT5G43590.1
Potra2n19c34270.2	3.403908387	0.674963689	NA	NA	Potri.T051200.3	AT4G37070.2
Potra2n19c34271.1	0.91145363	0.064817419	9.59969E-09	0.971828097	Potri.T051300.1	AT3G56070.1
Potra2n19c34272.1	0.335230729	0.771995929	0.312456238	0.679660122	Potri.T051800.2	AT5G43590.1
Potra2n19c34273.1	-0.177431499	0.040552459	0.397618609	0.977559521	Potri.T052100.2	AT1G58440.1
Potra2n19c34274.1						
Potra2n19c34275.1	0.088331643	0.331643021	0.666165709	0.332648024		
Potra2n19c34276.1	2.215123189	1.911450358	NA	NA	Potri.T052100.2	AT1G58440.1
Potra2n19c34277.1	-0.470909219	-0.054777364	0.01823748	0.979719961	Potri.T052100.2	AT1G58440.1
Potra2n19c34278.1	4.035930261	2.898762544	2.86171E-05	0.19180022		
Potra2n19c34279.1	1.403799325	0.919864607	0.009455635	0.49278277	Potri.T053000.2	AT4G27220.1
Potra2n19c34280.1	1.173431886	0.66910919	0.000405856	0.360126886	Potri.T052900.1	
Potra2n19c34281.4	0.853667494	0.274283933	0.001388529	0.70570456	Potri.T053000.2	AT4G27220.1
Potra2n19c34282.1	0.828544489	0.39921921	0.003531655	0.472700176	Potri.T053000.2	AT4G27220.1
Potra2n19c34283.4	1.069121108	0.466535311	0.00029321	0.513712531	Potri.T028301.1	AT4G27220.1
Potra2n19c34284.1	0.739796229	0.939166839	0.42330442	0.72101822	Potri.T025032.1	AT4G27220.1
Potra2n19c34285.1	1.664677056	1.89838425	NA	NA	Potri.T027700.1	AT4G27220.1
Potra2n19c34286.1					Potri.004G232250.1	AT5G49760.1
Potra2n19c34287.1	0.197229	0.150777439	0.834969794	0.973347974	Potri.T053000.2	AT4G27220.1
Potra2n19c34288.1	0.634904004	0.012235616	0.07188583	0.996032145	Potri.T025230.1	AT4G27190.1
Potra2n19c34289.1	0.102236182	1.173895664	NA	NA	Potri.T024600.1	AT5G23530.1
Potra2n19c34290.1	1.660328678	0.425334694	0.002879475	0.913466025	Potri.T025230.1	AT4G27190.1
Potra2n19c34291.1	0.347277755	0.350740764	0.617679881	0.924418763	Potri.016G059800.2	AT4G12570.1
Potra2n19c34292.2	0.572498123	0.000949795	0.141051452	0.999937671	Potri.T053000.2	AT4G27220.1
Potra2n19c34293.1	0.379018661	-0.204368981	0.845169441	NA	Potri.016G012900.4	AT4G12570.1
Potra2n19c34294.5	0.859683181	0.099661157	0.065129831	0.970432679	Potri.T025230.1	AT4G27190.1
Potra2n19c34295.1	1.1506204	0.3414135	0.013745387	0.817452127	Potri.T028301.1	AT4G27220.1
Potra2n19c34296.1	1.241260382	0.297205149	0.11473239	0.950878725	Potri.T052675.1	
Potra2n19c34297.1	-0.013325344	-1.395004375	0.997305141	NA	Potri.019G014400.1	AT5G23530.1
Potra2n19c34298.1	-0.163346318	0.092250835	0.367316789	0.917891908	Potri.019G014300.1	AT3G04580.1
Potra2n19c34299.1	-0.316207977	0.017393019	0.026476401	0.985393296	Potri.019G014200.1	AT3G04590.2

Potra2n19c34300.1	-0.6073247	-0.284167245	0.000427613	0.71411664	Potri.019G014100.1	AT2G14045.2
Potra2n19c34301.1	-0.153301856	-0.132683462	0.481492854	0.869327705	Potri.019G014000.1	AT1G08750.1
Potra2n19c34302.1	-0.031940414	0.022268125	0.893428297	0.987226805	Potri.019G013900.1	AT5G03970.1
Potra2n19c34303.1	2.827346164	-0.843576241	0.039058828	NA	Potri.019G013800.2	AT1G54560.1
Potra2n19c34304.1	5.444203802	2.944463136	1.62283E-14	0.372363623	Potri.019G013750.1	AT3G56710.1
Potra2n19c34305.1	NA	1.571529889	NA	NA	Potri.013G138300.1	ATCG00020.1
Potra2n19c34306.1	3.328370362	1.076613362	0.000596022	0.903934429	Potri.019G013750.1	AT3G56710.1
Potra2n19c34307.1	0.027499098	0.226563779	0.945572916	0.912602562	Potri.019G013100.1	AT5G28640.1
Potra2n19c34308.1						
Potra2n19c34309.1	1.294888502	1.075977407	0.345739117	NA	Potri.019G012400.1	
Potra2n19c34310.1	3.114341532	NA	NA	NA	Potri.019G012800.1	
Potra2n19c34311.1	-0.018586512	-0.034146057	0.912977413	0.977559521	Potri.019G012300.3	AT2G37690.1
Potra2n19c34312.1	0.028769653	0.372462716	0.849362987	0.3699319	Potri.019G012200.1	AT1G08800.1
Potra2n19c34313.1	-0.316806679	-0.160645056	0.003226545	0.759941508		
Potra2n19c34314.1	1.621239618	1.958105756	0.563140734	NA	Potri.019G012000.1	AT4G05430.1
Potra2n19c34315.1	0.253587214	-0.006468404	0.353062104	0.996943725	Potri.019G011400.1	AT5G60900.1
Potra2n19c34316.1	-0.899710826	-0.342337439	0.051667392	0.873823526	Potri.019G007300.1	AT5G60900.1
Potra2n19c34317.1	0.454278358	0.058351499	0.171467935	0.982221696	Potri.019G011400.1	AT5G60900.1
Potra2n19c34318.1	0.00255125	0.371603769	0.99822887	0.941855873	Potri.019G007700.1	AT5G60900.1
Potra2n19c34319.1	-0.356827613	-0.460616253	0.687554355	0.926234768	Potri.019G007300.1	AT5G60900.1
Potra2n19c34320.1	-0.068138884	-1.781050713	0.97584493	0.836633315	Potri.019G011750.1	AT5G60900.1
Potra2n19c34321.1	-0.575403655	-0.077878484	6.52501E-05	0.936360532	Potri.019G011600.1	AT1G49180.1
Potra2n19c34322.1	-0.257698083	-0.262473101	0.564534702	0.900869139	Potri.019G011400.1	AT5G60900.1
Potra2n19c34323.1	-0.13188556	0.11144869	0.400723086	0.931774514	Potri.019G007200.2	AT1G49180.1
Potra2n19c34324.1	-0.61910233	-0.767708831	NA	NA	Potri.019G007000.1	AT5G56530.1
Potra2n19c34325.1	2.10218636	-0.54512121	0.383937384	NA	Potri.019G011000.1	AT1G73260.1
Potra2n19c34326.1	0.788511749	-0.024763445	0.002235593	0.992934677	Potri.019G011400.1	AT5G60900.1
Potra2n19c34327.1	-0.149508977	0.185654547	0.309957518	0.849446235	Potri.019G011300.3	AT1G49180.1
Potra2n19c34328.1	1.64774763	NA	NA	NA	Potri.019G007000.1	AT5G56530.1
Potra2n19c34329.1	4.155015926	1.895229922	0.245715978	NA	Potri.019G006900.1	AT1G73260.1
Potra2n19c34330.1	-0.687850488	1.05123756	0.79675235	NA	Potri.019G010900.1	AT1G73260.1
Potra2n19c34331.1	-0.007462356	0.087837429	0.973296382	0.954559968	Potri.019G010700.1	AT3G18770.1

Potra2n19c34332.1	1.054298388	0.237200215	0.052970841	0.949966783	Potri.019G010600.1	AT1G49230.1
Potra2n19c34333.1	0.212144563	-1.411890438	0.948199824	NA		
Potra2n19c34334.1	1.64774763	NA	NA	NA		
Potra2n19c34335.1	-1.092577101	0.516043226	0.054623636	0.755585068	Potri.019G010500.1	AT1G49230.1
Potra2n19c34336.1	-0.111781489	-0.012661217	0.440533398	0.990927357	Potri.019G010400.1	AT5G09810.1
Potra2n19c34337.1	0.048542694	-0.024149093	0.842022417	0.986671181	Potri.019G010200.1	AT1G76890.2
Potra2n19c34338.1	-0.934299931	-0.589573438	0.180283098	0.88080281	Potri.019G006500.1	AT3G18800.1
Potra2n19c34339.1	0.000757389	-0.213266511	0.997734801	0.830149692	Potri.019G010000.2	AT2G18465.1
Potra2n19c34340.1	-0.449498961	0.036634498	0.003744675	0.9869326	Potri.019G006300.2	
Potra2n19c34341.2	-0.506420825	-0.004031196	0.003902727	0.999325699		
Potra2n19c34342.1	1.238197702	1.081759267	1.99636E-10	0.138040185	Potri.019G009700.1	AT3G14840.2
Potra2n19c34343.1	NA	1.571529889	NA	NA	Potri.019G008300.2	AT1G29670.1
Potra2n19c34344.1	NA	NA	NA	NA	Potri.019G008000.2	AT1G29670.1
Potra2n19c34345.1	4.130885783	1.690282711	2.27539E-17	0.353847713	Potri.019G009800.1	AT3G14840.2
Potra2n19c34346.1	NA	NA	NA	NA	Potri.007G111200.1	AT5G60900.1
Potra2n19c34347.1					Potri.019G004801.1	AT3G14840.2
Potra2n19c34348.1	0.067425975	0.257873286	0.680693254	0.775343511	Potri.019G004000.1	AT5G09840.1
Potra2n19c34349.1	-0.067251198	0.307621715	0.571527115	0.79078676	Potri.019G003900.1	AT5G09850.1
Potra2n19c34350.1	0.138107385	-0.088761825	0.219347607	0.904097715	Potri.019G003600.1	AT5G05320.1
Potra2n19c34351.1	-0.0185652	-0.035112402	0.902084442	0.97601212	Potri.019G003100.1	AT1G05520.1
Potra2n19c34352.1	0.946216174	0.448414748	0.455581531	0.966077009	Potri.019G002900.1	AT5G17540.1
Potra2n19c34353.2	1.617351281	-0.094110278	1.02357E-08	0.960656795	Potri.019G002500.1	AT5G17680.1
Potra2n19c34354.1	3.086414629	-0.561517328	0.004208199	NA	Potri.019G002500.1	AT5G17680.1
Potra2n19c34355.1	1.563078741	0.144653598	1.29033E-06	0.936364479	Potri.019G002500.1	AT5G17680.1
Potra2n19c34356.1					Potri.019G002400.1	
Potra2n19c34357.1	NA	NA	NA	NA	Potri.019G002300.1	AT1G15190.1
Potra2n19c34358.1	-1.207303508	-0.326884826	2.27416E-08	0.153430901	Potri.019G002201.1	AT5G53590.1
Potra2n19c34359.1	NA	1.381953902	NA	NA	Potri.019G002000.1	
Potra2n19c34360.1	-0.239137595	-0.12655276	0.082467538	0.903698951	Potri.019G001900.1	AT5G13340.1
Potra2n19c34361.1	-1.054631847	-0.049191422	4.5967E-06	0.982933413	Potri.019G001800.1	AT5G63710.1
Potra2n19c34362.1	0.501006348	0.126130718	0.002934073	0.918630346	Potri.T128200.1	AT5G36930.2
Potra2n19c34363.1						

Potra2n19c34364.1	0.629498959	0.330464036	0.006775526	0.586851629	Potri.T128200.1	AT5G36930.2
Potra2n19c34365.1	0.663644514	0.270717547	0.004038146	0.803948068	Potri.T127900.1	AT5G36930.1
Potra2n19c34366.1	1.481510672	1.186223665	0.433434296	NA	Potri.T128200.1	AT5G36930.2
Potra2n19c34367.1	2.728578922	0.857388755	4.87157E-08	0.472351144		
Potra2n19c34368.1	2.772050473	1.020386602	3.97976E-11	0.04665809	Potri.019G001500.2	AT1G34320.1
Potra2n19c34369.1	2.151033683	1.296241025	0.391165348	NA	Potri.018G104700.1	AT5G48930.1
Potra2n19c34370.1	NA	NA	NA	NA	Potri.019G001200.1	AT3G26040.1
Potra2n19c34371.1	-1.978930457	-1.451659946	0.037720624	0.704368358	Potri.019G001400.1	AT3G26040.1
Potra2n19c34372.1	-1.23758649	-2.225941838	0.536963918	NA	Potri.019G001200.1	AT3G26040.1
Potra2n19c34373.1	0.623227745	0.390410766	8.47589E-05	0.507997681	Potri.019G001100.1	AT2G21120.1
Potra2n19c34374.1	0.068312461	-0.22255822	0.63516121	0.601577719	Potri.019G001000.1	AT4G24290.2
Potra2n19c34375.2	-0.151838221	-0.178560694	0.213685484	0.642417298	Potri.019G000900.1	AT3G21060.1
Potra2n19c34376.1	0.457364305	-0.206155279	0.393267139	0.948577865	Potri.019G000800.1	AT2G38290.1
Potra2n19c34377.1	0.685414582	0.068194135	0.181735104	0.986636967	Potri.T000200.1	AT2G38290.1
Potra2n19c34378.1	0.245170784	1.256810202	NA	NA	Potri.T000300.1	AT3G25830.1
Potra2n19c34379.1	0.384067764	0.299028674	0.705524545	0.959881296	Potri.T000200.1	AT2G38290.1
Potra2n19c34380.1	6.544714695	0.110081123	7.51874E-06	NA	Potri.T000300.1	AT3G25830.1
Potra2n19c34381.1	-1.247190502	-1.288126628	0.590349718	NA	Potri.019G000400.1	AT5G23960.2
Potra2n19c34382.1	-0.240201375	-2.993456346	0.865220902	0.409505482	Potri.019G023000.1	AT3G25830.1
Potra2n19c34383.1					Potri.019G045100.1	AT5G23960.1
Potra2n19c34384.1	NA	NA	NA	NA	Potri.019G000400.1	AT5G23960.2
Potra2n19c34385.1	-0.61910233	-0.767708831	NA	NA	Potri.019G023000.1	AT3G25830.1
Potra2n19c34386.1	2.367304019	-0.065664892	0.003308296	0.993815835	Potri.T001933.1	AT5G36930.2
Potra2n19c34387.1	1.907218289	-0.225140307	0.000265526	0.948577865	Potri.T003001.1	AT5G36930.2
Potra2n19c34388.1	NA	1.394613675	NA	NA	Potri.T003100.1	AT1G21980.1
Potra2n19c34389.1	0.990557503	0.235922784	0.000111873	0.87062349	Potri.T003200.1	AT4G08180.1
Potra2n19c34390.1	-1.727032036	-2.828111881	0.611059385	NA	Potri.001G007501.1	AT2G20142.1
Potra2n19c34391.1	2.508839591	0.011656414	8.21497E-14	0.997011178	Potri.T003001.1	AT5G36930.2
Potra2n19c34392.1	3.275136327	0.475678052	0.10989986	NA	Potri.T003001.1	AT5G36930.2
Potra2n19c34393.1	2.196235887	NA	NA	NA	Potri.T001800.1	AT1G21980.1
Potra2n19c34394.1	NA	NA	NA	NA	Potri.T003100.1	AT1G21980.1
Potra2n19c34395.1	4.044457874	4.022981072	0.279056329	NA	Potri.T003100.1	AT1G21980.1

Potra2n19c34396.1	0.775833672	0.137062733	2.14824E-08	0.871720501	Potri.T003200.1	AT4G08180.1
Potra2n19c34397.1	2.632524198	2.02236598	NA	NA	Potri.T003300.1	AT5G08640.1
Potra2n19c34398.1	3.333810282	1.442369288	8.62876E-09	0.560708883	Potri.T003400.1	AT5G35370.1
Potra2n19c34399.1	NA	NA	NA	NA	Potri.T003500.1	
Potra2n19c34400.1					Potri.011G011700.1	AT3G17770.1
Potra2n19c34401.1	0.226690787	0.224135904	0.565437769	0.901535658		
Potra2n19c34402.1	NA	0.674963689	NA	NA	Potri.T003500.1	
Potra2n19c34403.1	-0.431148402	1.441925113	0.820249001	0.514425946	Potri.T003500.1	
Potra2n19c34404.1	0.97075192	0.889271655	0.000725735	0.558624809		
Potra2n19c34405.1	1.701154169	1.624585496	8.51589E-06	0.240947787		
Potra2n19c34406.1	0.499722473	0.18991453	0.004247029	0.876272842	Potri.T004400.1	AT5G08630.1
Potra2n19c34407.1	2.935256048	0.386565031	1.43302E-08	0.947969749	Potri.T004600.1	AT5G14930.2
Potra2n19c34408.1					Potri.T004700.1	AT1G04110.1
Potra2n19c34409.1	7.873377742	4.860351378	2.88518E-11	0.408808106	Potri.T004800.1	AT5G14930.2
Potra2n19c34410.1	3.801300754	NA	0.192048523	NA		
Potra2n19c34411.1	2.610910166	2.283425176	NA	NA		
Potra2n19c34412.1	NA	NA	NA	NA		
Potra2n19c34413.1	1.64774763	NA	NA	NA		
Potra2n19c34414.2	0.478519721	0.181459329	0.13522386	0.915339763	Potri.T010600.1	AT5G63620.1
Potra2n19c34415.1	0.359024298	0.128385577	0.375059324	0.910815461	Potri.T010700.2	AT5G63640.1
Potra2n19c34416.2	-0.195738958	0.073010875	0.302551614	0.957203914	Potri.004G138400.1	AT2G30390.1
Potra2n19c34417.1	0.399435368	-0.318451275	0.243468429	0.902847514	Potri.T011000.1	AT1G10940.1
Potra2n19c34418.1	-1.882753361	-0.576758454	3.77997E-10	0.66742133	Potri.T011100.1	AT1G72160.1
Potra2n19c34419.2	0.185495425	-0.341151471	0.580896375	0.767368598		
Potra2n19c34420.1	-0.684866113	-0.1925525	0.012177608	0.930598202	Potri.T011400.1	AT4G38620.1
Potra2n19c34421.1	4.978174091	3.493973493	0.006714011	NA	Potri.T011750.1	AT5G36930.1
Potra2n19c34422.1	1.64774763	0.674963689	NA	NA	Potri.T011851.1	
Potra2n19c34423.1	-0.473174032	0.314466722	0.435876015	0.900514809		
Potra2n19c34424.1	5.696453707	NA	0.000305026	NA		
Potra2n19c34425.1						
Potra2n19c34426.1						
Potra2n19c34427.1	4.406648316	0.674963689	0.02708552	NA	Potri.T011750.1	AT5G36930.1

Potra2n19c34428.1	0.174276244	-0.04185142	0.264796651	0.974628351	Potri.T012200.1	AT5G23630.1
Potra2n19c34429.1	-0.060437391	-0.017386242	0.77755084	0.992934677	Potri.T012300.1	AT5G23610.2
Potra2n19c34430.1	-0.622933167	0.091365484	0.083558446	0.961820855		
Potra2n19c34431.1	0.372849448	0.382375795	0.659152731	0.953108412	Potri.T012500.1	AT5G25610.1
Potra2n19c34432.1	-0.740428114	0.107797193	0.045072124	0.956865545	Potri.T012500.1	AT5G25610.1
Potra2n19c34433.1	1.052714355	-0.532452005	8.39047E-06	0.05006716	Potri.T012500.1	AT5G25610.1
Potra2n19c34434.1	0.740716364	-0.261295696	0.298048873	0.967122333	Potri.T012666.1	
Potra2n19c34435.1	0.681542587	-0.339736538	0.000337113	0.617980199	Potri.T012500.1	AT5G25610.1
Potra2n19c34436.1	0.644367032	0.155237564	6.53034E-07	0.837241494	Potri.T012800.1	AT1G35670.1
Potra2n19c34437.1	0.467826584	0.34248877	0.57527346	0.948577865		
Potra2n19c34438.1	0.37892084	0.194865189	0.011172723	0.749682671	Potri.T013000.1	AT5G23520.1
Potra2n19c34439.1	-2.327038515	-0.707680593	0.062413503	0.853768491	Potri.T013100.1	
Potra2n19c34440.2	-0.286862228	0.16462651	0.030718982	0.909675463	Potri.004G135901.1	AT3G11310.1
Potra2n19c34441.1	0.767431875	0.53774812	0.000264423	0.024085877		
Potra2n19c34442.1	1.051460013	-0.00620326	0.458483099	NA		
Potra2n19c34443.1	0.089952087	0.134905227	0.820249001	0.953108412	Potri.T015100.4	AT5G08450.1
Potra2n19c34444.1	0.71834538	-1.098716064	0.713810952	NA	Potri.T015666.1	AT5G08450.1
Potra2n19c34445.1	1.545832463	NA	NA	NA		
Potra2n19c34446.1	0.005572642	0.123874711	0.975716143	0.890446513	Potri.T044700.1	AT5G08450.3
Potra2n19c34447.1	-0.546378493	-0.150930925	0.223691807	0.954247482	Potri.T045400.1	AT4G15093.1
Potra2n19c34448.1	NA	NA	NA	NA	Potri.T014000.1	
Potra2n19c34449.1						
Potra2n19c34450.1	NA	NA	NA	NA	Potri.T013950.1	AT4G36760.1
Potra2n19c34451.1	-1.92220879	1.584719654	NA	NA	Potri.007G029700.1	AT4G36760.1
Potra2n19c34452.1	-0.434388848	0.176945758	0.033918958	0.896878863	Potri.T045200.1	AT4G10780.1
Potra2n19c34453.1	-0.638140032	-0.283079816	0.679107558	0.965801986	Potri.007G000900.1	AT5G23420.1
Potra2n19c34454.1	-0.471515322	-0.343412955	0.069818348	0.79078676	Potri.T045500.2	AT4G33420.1
Potra2n19c34455.1	1.329734851	-0.914353649	0.070181205	NA		
Potra2n19c34456.1	-1.731920422	-0.071528407	0.171369129	0.99114586	Potri.018G135800.1	AT3G14460.1
Potra2n19c34457.1	-0.844541275	-1.733747168	NA	NA	Potri.018G136100.1	AT3G14470.1
Potra2n19c34458.1	-0.394239481	-0.245506488	0.243282018	0.930598202	Potri.T045500.2	AT4G33420.1
Potra2n19c34459.1	0.388022981	0.911702589	0.763629047	0.797111689	Potri.018G135800.1	AT3G14460.1

Potra2n19c34460.1	NA	NA	NA	NA	Potri.T015000.1	
Potra2n19c34461.1	-0.374045838	-0.334645174	0.089483042	0.667038977	Potri.T045500.2	AT4G33420.1
Potra2n19c34462.1	-0.616832011	0.241211094	0.415094365	0.934627699	Potri.018G135800.1	AT3G14460.1
Potra2n19c34463.1	-0.227841233	0.237966721	0.565437769	0.900823365	Potri.018G136100.1	AT3G14470.1
Potra2n19c34464.1	-0.625992197	-0.472678098	0.011614212	0.598566355	Potri.T045500.2	AT4G33420.1
Potra2n19c34465.1	-0.276753299	-0.003803348	0.007492607	0.99812851	Potri.T045600.1	AT1G33420.1
Potra2n19c34466.1					Potri.018G137100.1	AT1G22910.1
Potra2n19c34467.2	0.149162028	0.041697105	0.182585214	0.954559968	Potri.018G137400.1	AT5G23570.1
Potra2n19c34468.1	-0.024947263	0.040992008	0.870012767	0.959881296	Potri.T045800.1	AT5G08500.1
Potra2n19c34469.1	2.917555842	2.591458059	0.1189615	NA	Potri.018G137700.2	
Potra2n19c34470.1	0.771905207	0.908483368	0.002822459	0.229802222	Potri.011G000100.1	AT4G04880.1
Potra2n19c34471.3	1.094240156	0.249912022	0.386690904	0.974893501	Potri.011G000500.1	AT3G20530.1
Potra2n19c34472.3	-0.022865111	-0.104591447	0.866911704	0.88221216	Potri.018G138200.1	AT5G26360.1
Potra2n19c34473.1	-0.576366144	0.311997573	0.034941956	0.865611082	Potri.T046200.1	AT5G49890.1
Potra2n19c34474.3	0.793134633	0.334399743	5.69601E-07	0.429397101	Potri.018G138000.1	AT5G49160.1
Potra2n1c1.1	0.364570751	0.104443387	0.002931119	0.857189843	Potri.001G000400.1	AT2G30942.1
Potra2n1c10.1	0.271919267	-0.695697894	NA	NA	Potri.001G001000.1	
Potra2n1c100.1	-0.435457038	-0.023659229	0.007479294	0.986636967	Potri.001G009501.1	AT2G20480.1
Potra2n1c1000.1	-0.621929641	0.030596298	2.07644E-07	0.97521505	Potri.001G116800.1	AT1G12370.2
Potra2n1c1001.4	-0.358740756	0.026729852	0.001476707	0.978203092	Potri.001G116900.1	AT1G12360.1
Potra2n1c1002.1	-1.208315305	-0.114562295	0.009553617	0.972159498	Potri.001G117000.1	AT1G12330.1
Potra2n1c1003.1	-0.61910233	-0.767708831	NA	NA	Potri.001G117100.1	AT3G60800.1
Potra2n1c1004.1	-0.002202525	0.004347042	0.9881779	0.996032145	Potri.001G117200.1	AT4G02570.4
Potra2n1c1005.1	-0.199783468	-0.107946273	0.004846818	0.777674368	Potri.001G117400.2	AT4G22740.2
Potra2n1c1006.1	0.212157319	0.201827539	0.376280775	0.870889425	Potri.001G117500.1	AT1G62850.2
Potra2n1c1007.1	0.101145187	0.091122203	0.764922664	0.971537796		
Potra2n1c1008.1	3.841098408	0.844188086	1.4977E-06	0.868573085	Potri.001G117600.1	AT3G60780.1
Potra2n1c1009.1	-0.174303244	-0.052764099	0.377684585	0.96245401	Potri.001G117700.1	AT4G12130.1
Potra2n1c101.1	1.016069769	-0.20320822	0.015767062	0.951394085	Potri.001G009600.1	AT1G69770.1
Potra2n1c1010.1	-0.28954804	-0.309148759	0.558642631	0.874295231	Potri.001G117800.1	AT4G22730.1
Potra2n1c1011.1	-0.441653222	-0.276301325	9.53617E-05	0.562306981	Potri.001G117900.1	AT1G12310.1
Potra2n1c1012.1	0.135973106	0.190223177	0.333620572	0.805052871	Potri.001G118000.1	AT4G12230.1

Potra2n1c1013.1	0.502721914	0.038800637	0.002640865	0.975578749	Potri.001G118100.2	AT1G07770.1
Potra2n1c1014.3	0.966420169	0.53324011	3.179E-10	0.560708883	Potri.001G118200.6	AT1G62810.1
Potra2n1c1015.1	-0.319656142	-0.130888311	0.020866731	0.833952007	Potri.001G118300.1	AT4G12290.1
Potra2n1c1016.1	-1.017622589	-0.365134991	0.088202567	0.883391101	Potri.001G118400.1	AT4G12300.1
Potra2n1c1017.1	-0.720581088	0.009873469	0.009752621	0.996292992	Potri.005G045800.1	AT4G12300.1
Potra2n1c1018.1	-0.291144064	0.215570057	0.041363373	0.815767573		
Potra2n1c1019.1	0.073507912	0.082090408	0.613414301	0.926235628	Potri.001G118700.2	AT4G12340.1
Potra2n1c102.1	0.133301369	0.139395642	0.060396313	0.596719268	Potri.001G009700.1	AT3G13670.1
Potra2n1c1020.1	-0.769048323	0.027343464	2.02441E-05	0.9896961	Potri.001G118800.1	AT4G12350.1
Potra2n1c1021.1	-0.07187666	-0.05141235	0.585028087	0.971317865	Potri.001G118900.1	AT4G22670.1
Potra2n1c1022.2	3.572142086	1.23935598	1.41828E-66	0.009688919	Potri.001G119000.1	AT1G62790.2
Potra2n1c1023.1	0.100342346	-0.072780494	0.735164195	0.953768643	Potri.001G119100.12	AT1G62780.1
Potra2n1c1024.1	NA	2.722516656	NA	NA	Potri.003G113700.1	AT4G12390.1
Potra2n1c1025.1	0.296647984	-2.486725844	0.954980235	NA	Potri.001G119300.1	AT1G62760.1
Potra2n1c1026.1	-0.053413631	0.107867421	0.64606483	0.894138111	Potri.001G119500.1	AT4G12400.2
Potra2n1c1027.1	-0.536640487	1.382929204	0.761069366	0.715585895		
Potra2n1c1028.1	-0.199846821	-0.281274225	0.379544769	0.635349767	Potri.001G119700.1	AT1G62730.1
Potra2n1c1029.1	-1.303142957	-0.590596481	1.54398E-13	0.002051352	Potri.003G113300.1	AT1G62710.1
Potra2n1c103.1	0.59719274	0.002849684	0.002499897	0.999458638	Potri.001G010300.1	AT3G58060.1
Potra2n1c1030.1	0.690056034	0.276528841	0.374365482	0.965249957	Potri.001G119900.1	AT4G12410.1
Potra2n1c1031.1	-1.39596337	-0.561114763	9.51744E-06	0.480580998	Potri.001G120000.1	AT1G12260.1
Potra2n1c1032.1	-1.545348754	-0.613455108	7.06239E-06	0.584489518		
Potra2n1c1033.1	-3.118199136	-0.765561931	0.06112026	0.923478213		
Potra2n1c1034.1	NA	NA	NA	NA	Potri.003G112900.4	AT1G12250.2
Potra2n1c1035.1	-0.135140573	0.041444871	0.172085606	0.956865545	Potri.001G120400.1	AT1G12230.1
Potra2n1c1036.1	1.735952942	0.871260721	0.000731105	0.754074409	Potri.001G120500.1	AT4G22590.1
Potra2n1c1037.1	-0.769219319	-0.359738847	0.290181989	0.930598202	Potri.001G120700.2	AT4G22600.1
Potra2n1c1038.2	0.010986968	-0.067021407	0.949281334	0.961820855	Potri.001G120900.1	AT1G62640.1
Potra2n1c1039.1	0.39292344	-0.083642236	0.05767486	0.95981322	Potri.001G120800.1	AT4G22580.1
Potra2n1c104.1	0.011950309	0.23848565	0.926186477	0.824905557	Potri.001G010400.2	AT4G10620.1
Potra2n1c1040.1	0.361644207	0.253621419	0.035094141	0.723658895	Potri.001G121000.1	AT1G62620.1
Potra2n1c1041.1	-0.172881675	-0.116459341	0.311912913	0.935469038	Potri.001G121200.1	AT1G62530.1

Potra2n1c1042.1	-1.735190235	-0.409260434	1.83758E-08	0.422139068	Potri.001G121300.1	AT2G30590.1
Potra2n1c1043.1	-1.112447564	-0.290708298	6.18328E-10	0.779645942	Potri.001G121400.2	AT4G22570.1
Potra2n1c1044.1	-0.688830519	-0.245020687	6.34029E-07	0.843683654	Potri.001G121600.1	AT4G22560.1
Potra2n1c1045.1	NA	NA	NA	NA		
Potra2n1c1046.1	NA	NA	NA	NA	Potri.001G039600.1	
Potra2n1c1047.1	-0.448621992	0.406389115	0.053478708	0.812373531	Potri.001G121700.10	AT4G22540.1
Potra2n1c1048.1	1.459962178	NA	NA	NA	Potri.001G121800.1	AT1G12100.1
Potra2n1c1049.1	0.561797534	-0.413431791	0.060356463	0.655979264	Potri.001G121900.1	AT4G12510.1
Potra2n1c105.1	-0.454867671	0.033806007	0.211989372	0.986190118	Potri.001G010700.1	AT3G45240.1
Potra2n1c1050.1	3.129406048	3.440702788	0.088023269	NA		
Potra2n1c1051.1	NA	NA	NA	NA	Potri.001G122100.1	AT3G22142.1
Potra2n1c1052.1	0.193721351	0.05988463	0.103158009	0.937846405	Potri.001G122200.2	AT5G47570.1
Potra2n1c1053.1	1.210848526	0.030420802	1.91248E-09	0.984376543	Potri.001G122400.1	AT4G17260.1
Potra2n1c1054.1	1.318444116	0.056009758	0.00517865	0.9869326	Potri.001G122400.1	AT4G17260.1
Potra2n1c1055.2	0.213404524	0.236859403	0.223932402	0.792544431		
Potra2n1c1056.1	0.419594988	0.280740737	0.060766502	0.852220353		
Potra2n1c1057.1	-2.295337707	0.085253599	NA	NA	Potri.001G122600.1	
Potra2n1c1058.1	-0.464814227	-0.127262671	0.022735724	0.894276674	Potri.001G122700.1	AT4G16520.2
Potra2n1c1059.2	1.013717953	0.383448411	4.54008E-05	0.597141087	Potri.001G122800.2	AT4G00150.1
Potra2n1c106.1	-0.726654653	0.169082584	0.173725455	0.940152186	Potri.001G010600.1	AT2G31945.1
Potra2n1c1060.2	0.098227475	0.146380682	0.740596912	0.935751704		
Potra2n1c1061.1	-0.306656083	0.078630969	0.163551382	0.968950851	Potri.001G123000.1	AT2G35736.1
Potra2n1c1062.1	-0.146948592	-1.255035048	0.910242475	NA		
Potra2n1c1063.1	0.854118292	0.305724087	6.7139E-07	0.801458793	Potri.003G110700.1	AT4G17250.1
Potra2n1c1064.1	-0.213950556	-0.039483577	0.100936402	0.963878456	Potri.001G123200.2	AT4G16530.1
Potra2n1c1065.1	-1.409983009	-1.55593228	NA	NA	Potri.001G123300.1	AT4G19980.1
Potra2n1c1066.1	0.37466767	0.446512332	0.007150226	0.330629242	Potri.001G123400.4	AT5G44800.1
Potra2n1c1067.1	0.0251552	0.091918903	0.831549431	0.865611082	Potri.001G123500.2	AT1G79570.1
Potra2n1c1068.1	0.234567065	0.090314123	0.082137905	0.922011871	Potri.001G123600.1	AT1G45332.1
Potra2n1c1069.1	-0.716672082	0.065409388	0.000307864	0.960530719	Potri.001G123700.1	AT2G35060.1
Potra2n1c107.1	0.3713981	-0.096789807	0.021702505	0.943483421	Potri.001G010800.1	AT1G50480.1
Potra2n1c1070.1	0.92641307	-0.767708831	NA	NA	Potri.001G123900.1	

Potra2n1c1071.2	0.028941	-0.049522159	0.846317508	0.972159498	Potri.001G124000.1	AT4G25230.2
Potra2n1c1072.1	-0.016303987	-0.132444586	0.945572916	0.914321114	Potri.001G124100.1	AT4G25225.1
Potra2n1c1073.1	-1.045559716	0.484271284	0.000102571	0.83404888	Potri.001G124200.1	AT3G47420.1
Potra2n1c1074.1	-0.2358117	-0.053105585	0.045200691	0.953608209	Potri.001G124300.1	AT5G51410.3
Potra2n1c1075.1	0.078840448	-0.068042479	0.526092844	0.953831244	Potri.001G124500.1	AT2G45010.1
Potra2n1c1076.1	1.545832463	NA	NA	NA	Potri.001G124700.2	AT5G20110.1
Potra2n1c1077.1					Potri.003G108601.1	
Potra2n1c1078.1					Potri.001G124800.1	AT3G47440.1
Potra2n1c1079.1	-0.509165106	-0.177376698	0.03567484	0.910878765	Potri.001G124900.1	AT3G47450.2
Potra2n1c108.1	NA	NA	NA	NA	Potri.001G010900.2	AT5G06700.1
Potra2n1c1080.2	0.12914277	-0.469276072	0.722958746	0.784845657	Potri.001G125000.1	AT5G62410.1
Potra2n1c1081.1	NA	3.152096249	NA	NA	Potri.001G125100.1	
Potra2n1c1082.1	0.585338443	0.291106292	1.38356E-05	0.64562236	Potri.001G125200.1	AT3G24080.1
Potra2n1c1083.1					Potri.001G125300.1	AT1G02080.1
Potra2n1c1084.1	3.125402034	NA	NA	NA	Potri.001G125400.1	AT5G62420.1
Potra2n1c1085.1	0.82357019	2.930270544	NA	NA	Potri.003G108100.1	AT1G76520.2
Potra2n1c1086.1	-0.147865819	0.006504818	0.409958387	0.996032145	Potri.001G125600.1	AT2G42590.3
Potra2n1c1087.1	-0.137832175	0.025051046	0.229890768	0.973347974	Potri.001G125700.1	AT5G61450.1
Potra2n1c1088.1	0.593523315	0.233971536	0.000255846	0.759797728	Potri.001G125800.1	AT5G07670.1
Potra2n1c1089.1	1.563265709	-0.48453614	0.321464312	NA	Potri.001G125900.2	AT3G60580.1
Potra2n1c109.1	8.168921468	2.143173882	1.47342E-10	NA	Potri.001G011000.1	AT4G08780.1
Potra2n1c1090.1	3.114341532	2.303900616	NA	NA	Potri.001G126000.1	AT4G25190.1
Potra2n1c1091.1	-1.221904389	-0.368443696	2.84192E-06	0.784830006	Potri.001G126100.1	AT5G61480.1
Potra2n1c1092.2	-0.277473507	-0.143456661	0.038269562	0.814981587	Potri.001G126200.1	AT5G07630.1
Potra2n1c1093.1	-1.363374583	-0.715919892	3.81322E-10	0.064873104	Potri.001G126300.1	AT4G25170.1
Potra2n1c1094.1	-0.266573397	-0.109741873	0.078485073	0.887678043	Potri.001G126400.1	AT5G51300.2
Potra2n1c1095.1	-0.136277196	-0.014397048	0.203589256	0.992934677	Potri.001G126500.1	AT5G61500.1
Potra2n1c1096.1	0.015747078	-0.137577575	0.94178621	0.909675463	Potri.001G126700.1	AT5G04620.2
Potra2n1c1097.1	0.972714208	1.136863019	0.666526112	0.881090429	Potri.003G106800.1	AT5G51330.1
Potra2n1c1098.1	-0.882839857	-0.476427394	1.14161E-06	0.590087814	Potri.001G127100.2	AT5G45290.1
Potra2n1c1099.3	0.290342705	0.300220526	0.066101575	0.477196168	Potri.001G127300.1	AT1G31650.1
Potra2n1c11.1	NA	NA	NA	NA	Potri.001G001100.1	AT2G03090.1

Potra2n1c110.1	NA	NA	NA	NA	Potri.018G117100.1	
Potra2n1c1100.1					Potri.001G127400.1	AT5G17540.1
Potra2n1c1101.1	2.492428534	1.363811008	NA	NA	Potri.001G127600.1	AT5G17540.1
Potra2n1c1102.1	6.213745503	3.15388062	2.58037E-10	0.346132193	Potri.001G127700.1	AT5G47650.1
Potra2n1c1103.1	-2.049851982	-1.13615537	0.177589494	0.817452127	Potri.001G127900.2	AT4G27870.1
Potra2n1c1104.1	0.754394281	-0.107300204	0.690172858	NA	Potri.017G011101.1	
Potra2n1c1105.1	-1.012012902	-0.107571669	4.93978E-08	0.946322185	Potri.001G128100.1	AT1G31490.1
Potra2n1c1106.1	-1.437762822	0.44069969	0.64267651	NA		
Potra2n1c1107.1	-0.610733963	-0.226804209	0.015968612	0.887678043	Potri.001G128200.1	AT5G45275.1
Potra2n1c1108.2	4.527822387	1.626137074	3.35037E-17	0.526938837	Potri.001G128300.1	AT4G19460.1
Potra2n1c1109.1	-0.749823207	-0.10215251	NA	NA	Potri.001G128500.1	AT1G61590.1
Potra2n1c111.1	6.309711792	1.793508148	2.44966E-07	0.824905557	Potri.001G011000.1	AT4G08780.1
Potra2n1c1110.1	-0.883133697	-0.287323236	0.003187108	0.788065047	Potri.001G128600.1	AT1G31460.1
Potra2n1c1111.1	0.055291843	0.000713869	0.688389693	0.999458638	Potri.001G128700.1	AT2G03670.1
Potra2n1c1112.1	-0.173545176	-0.060852048	0.122439869	0.941855873	Potri.001G128800.1	AT4G34660.1
Potra2n1c1113.1	0.245170784	-1.465929376	NA	NA	Potri.001G128900.1	AT1G31410.1
Potra2n1c1114.3	-0.024704466	0.077444303	0.903870776	0.937802986	Potri.001G129000.1	AT1G31420.2
Potra2n1c1115.1	0.498319887	0.016382381	0.001222503	0.989329144	Potri.001G129100.1	AT1G04170.1
Potra2n1c1116.1	NA	3.213195509	NA	NA	Potri.001G129200.2	AT3G07870.1
Potra2n1c1117.1	NA	NA	NA	NA	Potri.001G129300.1	AT4G28050.1
Potra2n1c1118.1	-0.447300138	-0.434294005	0.562882746	0.890446513	Potri.001G129400.1	AT5G61320.1
Potra2n1c1119.1	-0.319103801	-0.060830658	0.049935141	0.970930149	Potri.001G129500.1	
Potra2n1c112.1	4.911448338	3.860295133	0.005414048	NA	Potri.001G011500.1	AT5G06720.1
Potra2n1c1120.1	0.186237464	0.209487272	0.104735579	0.64562236	Potri.001G129600.1	AT3G23640.2
Potra2n1c1121.1	-0.023392407	0.040926583	0.876339132	0.950944141	Potri.001G129800.1	AT5G61230.1
Potra2n1c1122.1	0.150775664	0.128975161	0.293932163	0.885375133	Potri.001G129900.1	AT2G38250.1
Potra2n1c1123.1	-0.096637917	0.075883232	0.681666327	0.953338546	Potri.001G130100.1	AT3G45900.1
Potra2n1c1124.1	-0.106587924	-0.013171536	0.476697957	0.99114586	Potri.001G130200.1	AT1G31812.1
Potra2n1c1125.3	-0.277637775	-0.122871041	0.252983431	0.910815461	Potri.001G130400.1	AT3G61360.1
Potra2n1c1126.1	-0.009800637	0.039830946	0.952409141	0.973347974	Potri.001G130500.1	AT4G19150.1
Potra2n1c1127.1	NA	NA	NA	NA	Potri.003G103300.1	AT5G45390.1
Potra2n1c1128.1	2.227693419	-1.452060329	0.136579231	NA	Potri.001G130700.1	AT3G17380.1

Potra2n1c1129.1	-0.615650819	0.002817789	2.41987E-07	0.999121696	Potri.003G103100.1	
Potra2n1c113.1	6.020506587	5.08010697	0.000273164	NA	Potri.001G011500.1	AT5G06720.1
Potra2n1c1130.1	-0.065236706	0.154310774	0.761240998	0.905991243	Potri.001G130900.1	AT4G19140.1
Potra2n1c1131.4	0.744885172	0.068667914	2.58659E-05	0.957163493	Potri.003G102700.1	AT5G45400.1
Potra2n1c1132.1	0.257180429	0.109038623	0.203430882	0.930598202	Potri.001G131300.1	AT4G25030.2
Potra2n1c1133.1	0.186257122	-0.232792465	0.454236781	0.860901249	Potri.001G131400.2	AT1G31850.2
Potra2n1c1134.1	3.796763363	0.760087236	4.16267E-43	0.719713755	Potri.001G131500.2	
Potra2n1c1135.3	5.779971548	3.036922374	7.61127E-92	0.000175439	Potri.001G131600.1	
Potra2n1c1136.1	0.187579618	1.750793482	0.927536662	0.63454842	Potri.001G131800.1	AT1G31880.1
Potra2n1c1137.1	-0.82043979	-0.104450161	0.022500136	0.972159498	Potri.001G132001.1	AT4G19100.1
Potra2n1c1138.1	-1.795995878	-0.719376946	0.003827027	0.704368358	Potri.001G132100.1	AT3G01430.1
Potra2n1c1139.2	-0.391560722	0.248716795	0.828351677	NA	Potri.T172200.1	
Potra2n1c114.2	-1.3421759	-0.307978444	1.20746E-08	0.519348125	Potri.001G011700.1	AT1G51110.1
Potra2n1c1140.1	NA	NA	NA	NA	Potri.001G132300.1	AT1G75950.1
Potra2n1c1141.2	-0.119838095	0.128705052	0.380722329	0.873823526	Potri.001G132400.1	AT4G19070.1
Potra2n1c1142.1	-0.551207873	0.534258605	0.621091344	0.860810425	Potri.001G132500.1	AT4G27890.1
Potra2n1c1143.1	-0.121277565	-0.086851418	0.596762987	0.957686558	Potri.001G132600.1	AT4G37740.1
Potra2n1c1144.1	0.050022291	0.244229214	0.802617534	0.792864576	Potri.001G132700.1	AT4G19045.1
Potra2n1c1145.1	0.9792012	0.092658095	1.06972E-06	0.953831244	Potri.001G132800.1	AT1G32120.1
Potra2n1c1146.1	-0.01567537	0.015458087	0.905346526	0.990128003	Potri.001G132900.1	AT4G19040.1
Potra2n1c1147.1	1.858841966	0.895636497	0.000381896	0.605100478	Potri.001G133000.1	AT4G34135.1
Potra2n1c1148.1	-0.492508808	-0.425896618	0.781916057	0.959881296	Potri.001G133100.1	AT1G78270.1
Potra2n1c1149.1	-1.015492553	-0.637165756	0.011111982	0.871720501	Potri.001G133200.1	AT1G32100.1
Potra2n1c115.1	-0.213010416	-0.053773853	0.074445362	0.965801986		
Potra2n1c1150.1	0.812670397	1.338528029	0.241548244	0.444720646	Potri.001G133300.1	AT1G32100.1
Potra2n1c1151.1	-1.088088054	-0.488059391	0.001539985	0.678727836	Potri.001G133400.1	AT5G45580.1
Potra2n1c1152.1	-0.278401248	-0.019317879	0.083441958	0.987226805	Potri.001G133500.2	AT1G80480.1
Potra2n1c1153.2	-0.065416798	-0.017902918	0.758888544	0.990128003	Potri.001G133600.1	AT5G45590.1
Potra2n1c1154.2	-0.712811953	-0.25056691	0.018100269	0.763747409	Potri.001G133800.1	AT1G32090.1
Potra2n1c1155.1	-1.396974339	-0.294173274	0.002133921	0.919926422	Potri.001G133900.1	AT2G35550.1
Potra2n1c1156.1	-0.319929366	-0.342325562	0.055572451	0.775618506	Potri.001G134000.1	AT1G32060.1
Potra2n1c1157.1	0.078977038	0.112470765	0.840215881	0.953831244	Potri.001G134800.2	AT5G45610.1

Potra2n1c1158.1	0.009476198	0.170458614	0.961890434	0.83405494	Potri.001G135900.1	AT1G32130.1
Potra2n1c1159.1	0.049653386	-0.003345332	0.624568741	0.996032145	Potri.001G136000.1	AT2G35530.1
Potra2n1c116.1	-0.390140324	-0.002092079	0.005420659	0.999325699	Potri.001G011800.1	AT5G19900.1
Potra2n1c1160.1	0.205836627	0.512940789	0.554799549	0.834759753	Potri.001G136100.1	AT1G32170.1
Potra2n1c1161.1	-0.204139244	-0.307562459	0.244058769	0.8324377	Potri.001G136200.1	AT3G03050.1
Potra2n1c1162.1	-0.16970258	-0.227719124	0.351468802	0.812373531	Potri.001G136300.1	AT5G61910.3
Potra2n1c1163.2	-0.567676637	-0.329157903	0.011081046	0.888896953	Potri.001G136400.1	AT1G32190.1
Potra2n1c1164.3	-0.155274464	0.137217013	0.48019861	0.937687311	Potri.001G136600.1	AT1G32200.1
Potra2n1c1165.1	1.664677056	NA	NA	NA	Potri.001G136700.1	AT3G12890.1
Potra2n1c1166.1	0.130958625	-0.136024845	0.532258996	0.912777843	Potri.003G096800.1	AT1G32210.1
Potra2n1c1167.1	0.120363133	0.073801781	0.42493086	0.941677175	Potri.001G136900.2	AT1G32220.1
Potra2n1c1168.1	0.575343299	0.208348529	0.000156024	0.720242695	Potri.001G137000.1	
Potra2n1c1169.1	1.944023666	0.53392427	0.000683012	0.859892315	Potri.001G137100.1	
Potra2n1c117.1	0.006845522	0.124225839	0.969123106	0.885375133	Potri.001G011900.2	AT3G07160.1
Potra2n1c1170.1	0.107683925	0.221855762	0.357768528	0.779077802	Potri.001G137200.1	AT1G32230.1
Potra2n1c1171.2	1.129465341	0.7359347	0.013350827	0.787760802	Potri.010G022800.1	AT5G46160.2
Potra2n1c1172.1	4.462842944	NA	0.079153468	NA	Potri.001G137600.1	AT1G32240.1
Potra2n1c1173.1	-0.136099314	-0.124891837	0.694116184	0.940376196	Potri.001G137700.1	AT5G46910.1
Potra2n1c1174.3	-0.453128506	-0.715061572	0.778931092	NA	Potri.001G137800.1	AT5G46880.1
Potra2n1c1175.1	0.122424431	0.005183078	0.431050899	0.996154416	Potri.001G137900.1	AT2G35490.1
Potra2n1c1176.1	-0.89422916	-0.441252658	0.00388883	0.759797728	Potri.001G138000.1	AT1G32250.1
Potra2n1c1177.1	0.010161773	0.005597349	0.956381318	0.996032145	Potri.001G138100.2	AT1G74340.1
Potra2n1c1178.1	-0.515329435	-0.228923609	0.268896968	0.943685779	Potri.001G138200.1	AT1G32260.1
Potra2n1c1179.1	-0.20153394	-0.067237016	0.153015195	0.963878456		
Potra2n1c118.6	-0.532392009	-0.235275208	0.05100253	0.920028955	Potri.001G012200.1	AT5G13000.1
Potra2n1c1180.1	-0.176620127	-0.041155014	0.188210685	0.980063549	Potri.001G138400.2	AT4G17720.1
Potra2n1c1181.1	-0.333089192	0.091485184	0.02883758	0.951539476	Potri.001G138500.2	AT5G46860.1
Potra2n1c1182.1	0.213665225	-0.461774425	0.877431343	0.936549944	Potri.003G095200.2	AT5G46850.3
Potra2n1c1183.1	-0.123862104	-0.772836944	0.9309217	0.910878765		
Potra2n1c1184.3	-0.342191691	0.01896394	0.135288008	0.992934677	Potri.001G138700.2	AT1G32310.1
Potra2n1c1185.2	0.268086366	0.14714061	0.10765778	0.869327705	Potri.001G138900.2	AT1G32330.1
Potra2n1c1186.1	0.977041316	0.333449967	0.018100269	0.915488947	Potri.001G139100.1	AT4G17760.1

Potra2n1c1187.1	0.15307118	0.080512735	0.554112878	0.958152146	Potri.001G139200.1	AT1G52620.1
Potra2n1c1188.1	-0.135501794	0.062013865	0.613775798	0.968554995	Potri.001G139300.1	AT5G01110.1
Potra2n1c1189.2	0.452532823	0.179442957	0.004048584	0.755355354	Potri.001G139400.2	AT1G32340.1
Potra2n1c119.3	-1.537321739	0.107698312	1.29971E-07	0.966005919	Potri.001G012300.1	AT5G42340.1
Potra2n1c1190.1	NA	NA	NA	NA	Potri.015G069300.1	AT2G01050.1
Potra2n1c1191.1	-1.440100375	-0.554614819	3.80785E-18	0.561272053	Potri.001G139500.1	AT4G17770.1
Potra2n1c1192.1	0.200481127	-1.217319016	0.820463714	0.540010302	Potri.001G139600.2	AT4G25960.1
Potra2n1c1193.1	3.404798739	0.627277983	0.243284992	NA	Potri.001G139700.1	AT4G25960.1
Potra2n1c1194.1	0.803887427	0.542891454	6.51556E-05	0.454422475	Potri.001G139800.2	AT3G57280.1
Potra2n1c1195.1	0.264663135	-0.078165449	0.625049247	0.981772014	Potri.001G139900.1	AT5G16770.1
Potra2n1c1196.1	-0.294120389	-0.042738783	0.186902489	0.98177802	Potri.001G140000.1	AT5G16760.1
Potra2n1c1197.1	1.275253983	0.52412271	0.000200324	0.372755083	Potri.001G140200.1	AT4G17790.1
Potra2n1c1198.1	0.383449203	0.022607428	6.53E-05	0.974756511	Potri.001G140300.1	AT5G46840.1
Potra2n1c1199.1	NA	NA	NA	NA	Potri.001G140400.1	AT2G35880.1
Potra2n1c12.1	-0.142276533	-0.621670864	0.650577818	0.455054253	Potri.003G223400.3	AT1G55540.1
Potra2n1c120.1	1.249781912	-0.431090218	1.76498E-08	0.766714848	Potri.T180100.1	AT3G12120.1
Potra2n1c1200.1	0.818297826	0.280680893	0.000120967	0.874507876	Potri.001G140500.1	AT4G17940.1
Potra2n1c1201.2	0.479789699	0.035521748	0.001847551	0.983446961	Potri.001G140600.1	AT5G61510.1
Potra2n1c1202.1	4.061394909	0.39044265	8.20216E-24	0.937687311	Potri.001G140700.1	AT2G23910.1
Potra2n1c1203.1	0.055706772	0.088000124	0.600200849	0.926808461	Potri.001G140800.1	AT1G32360.1
Potra2n1c1204.1	0.02089444	-0.619206972	0.988779878	0.938458121	Potri.001G141100.1	AT5G46690.1
Potra2n1c1205.2	-1.139787166	-0.357328111	0.001784184	0.706051231	Potri.001G141200.1	AT3G03550.1
Potra2n1c1206.1	0.060042298	0.482244086	0.885208399	0.814981587	Potri.001G141300.1	AT2G35410.1
Potra2n1c1207.1	-0.348237745	-0.010133911	0.238195063	0.996032145	Potri.001G141400.1	AT5G46700.1
Potra2n1c1208.1	0.25525337	0.44863809	0.230760373	0.595667201	Potri.003G092800.1	AT4G17900.1
Potra2n1c1209.1	-0.289192876	-0.068037258	0.45668094	0.967229803		
Potra2n1c121.1	1.174943095	-0.501260543	1.0068E-09	0.568587629	Potri.T180100.1	AT3G12120.1
Potra2n1c1210.1	-0.336390309	-0.073123146	0.256073482	0.953768643	Potri.001G141600.1	AT1G32690.1
Potra2n1c1211.1					Potri.016G020300.1	AT4G34200.1
Potra2n1c1212.3	-1.245326483	-0.327423559	2.32923E-05	0.813731863		
Potra2n1c1213.2	0.425995995	0.689482456	0.355898795	0.626455563		
Potra2n1c1214.1	-0.61910233	1.586078131	NA	NA	Potri.001G141900.1	AT1G55200.1

Potra2n1c1215.1	0.318911972	-0.565616235	0.403093693	0.778013962	Potri.001G142000.1	AT5G46740.1
Potra2n1c1216.2	-0.096482283	0.02479201	0.558562855	0.987824571	Potri.001G142100.1	AT4G17890.1
Potra2n1c1217.2	1.054983749	0.301769254	0.000623283	0.759797728	Potri.001G142200.1	AT1G32640.1
Potra2n1c1218.3	0.215924552	0.084196117	0.550522833	0.963878456	Potri.001G142300.10	
Potra2n1c1219.1	0.21073473	-0.128296256	0.072140412	0.859892315	Potri.001G142400.1	AT2G35230.1
Potra2n1c122.1	-0.765440978	-0.054889607	0.071724666	0.983113084	Potri.T180100.1	AT3G12120.1
Potra2n1c1220.1	-1.021893024	0.247753558	0.042082679	0.949048812	Potri.001G142500.2	AT4G17870.1
Potra2n1c1221.1	-0.631292306	-0.196710134	0.005377977	0.859892315	Potri.001G142600.1	AT5G46800.1
Potra2n1c1222.1	3.467582626	0.22646714	4.28246E-05	NA	Potri.001G142700.1	AT2G35290.1
Potra2n1c1223.1	0.040437313	-0.037599366	NA	NA	Potri.001G142800.1	AT4G17800.1
Potra2n1c1224.1	0.82357019	NA	NA	NA		
Potra2n1c1225.1					Potri.001G142900.1	AT4G17810.1
Potra2n1c1226.1	-0.61910233	-0.767708831	NA	NA		
Potra2n1c1227.1	NA	NA	NA	NA	Potri.001G155750.1	AT1G21280.1
Potra2n1c1228.1	0.504031045	0.059407686	0.000868803	0.961077436	Potri.001G143200.1	AT4G17830.1
Potra2n1c1229.1	-0.702537299	-0.371805834	7.66455E-06	0.288650898	Potri.001G143400.1	AT2G35260.1
Potra2n1c123.1	NA	2.32414027	NA	NA	Potri.001G012600.1	
Potra2n1c1230.1	0.460550199	-0.156741851	0.007352361	0.900869139	Potri.001G143500.1	AT4G17950.1
Potra2n1c1231.1	-0.140665312	-0.138762884	0.232429024	0.874507876	Potri.001G143600.1	AT4G17960.1
Potra2n1c1232.1	-0.932782463	-1.542156768	0.770269079	NA	Potri.001G143700.1	AT1G32560.1
Potra2n1c1233.4	0.187440783	0.05229411	0.074468886	0.959881296	Potri.001G144000.1	AT2G35320.1
Potra2n1c1234.1	6.858361547	3.010689165	1.89137E-07	NA	Potri.001G144100.1	AT4G35470.1
Potra2n1c1235.1	8.041984232	4.366002939	5.908E-12	NA	Potri.001G144100.1	AT4G35470.1
Potra2n1c1236.5	0.035652275	0.210062875	0.845188404	0.777553704	Potri.001G144200.1	AT2G35330.1
Potra2n1c1237.1	NA	1.363811008	NA	NA	Potri.001G144300.1	AT4G17970.1
Potra2n1c1238.1	0.025121814	-0.195132129	0.980496703	0.956292878	Potri.001G144400.1	AT5G46590.1
Potra2n1c1239.1	-0.239358979	-0.16995399	0.223691905	0.849815276	Potri.001G144500.1	AT1G32500.1
Potra2n1c124.1	3.751325214	0.722662614	4.73469E-24	0.842247476	Potri.001G015500.1	AT3G45140.1
Potra2n1c1240.1	0.346151926	-0.064699186	0.10856544	0.969717058	Potri.001G144700.1	AT2G35350.1
Potra2n1c1241.1	-2.934181405	-3.053210574	0.11268868	NA	Potri.001G144800.1	AT1G32470.1
Potra2n1c1242.1	-0.505675629	-0.130455028	0.002876343	0.924951347	Potri.001G145066.1	
Potra2n1c1243.1	0.168055915	0.232485066	0.800435111	0.930598202	Potri.001G145200.1	AT1G32450.1

Potra2n1c1244.1	0.032345401	-0.272195468	0.892888016	0.815119099	Potri.001G145300.1	AT1G32440.1
Potra2n1c1245.1	-0.115835129	-0.701130386	0.767174175	0.605100478		
Potra2n1c1246.1	0.003506866	-0.339069608	0.990603442	0.71703421		
Potra2n1c1247.1	1.459962178	NA	NA	NA		
Potra2n1c1248.1	NA	2.134902029	NA	NA	Potri.001G337200.1	AT5G22660.2
Potra2n1c1249.1	-0.785636943	-0.091352674	9.50743E-09	0.923397137	Potri.001G145400.1	AT3G57990.1
Potra2n1c125.1	0.82357019	NA	NA	NA		
Potra2n1c1250.1	0.178813727	0.236469789	0.219818192	0.676711874	Potri.001G145500.1	AT1G32370.2
Potra2n1c1251.1	1.022807933	0.348357145	0.039542155	0.893394174	Potri.001G145700.1	AT2G35390.2
Potra2n1c1252.4	0.410223789	-0.133672095	0.483020002	0.900884796	Potri.001G145900.6	AT4G18010.2
Potra2n1c1253.1	2.249249553	2.695774987	NA	NA		
Potra2n1c1254.2	1.613084118	0.119128619	4.62756E-09	0.953608209		
Potra2n1c1255.8	0.291156198	0.00141777	0.026521023	0.999325699	Potri.001G181000.2	AT5G07610.1
Potra2n1c1256.1	2.549191961	NA	NA	NA		
Potra2n1c1257.1	-0.07997724	0.236156718	0.896048808	0.941855873		
Potra2n1c1258.1	0.411990792	-0.063747064	0.098204684	0.9761362	Potri.001G146200.18	AT4G18020.6
Potra2n1c1259.1	0.436028691	-0.137796247	0.128980616	0.943819961		
Potra2n1c126.1	-0.188157562	-0.374408062	0.570277968	0.865611082	Potri.001G015700.1	AT3G57880.1
Potra2n1c1260.1	-0.040771019	-0.074663676	0.771847901	0.921015963	Potri.001G146400.1	AT4G18030.1
Potra2n1c1261.1	0.075421808	0.146525902	0.565706099	0.777400198	Potri.001G146500.1	AT5G46560.1
Potra2n1c1262.1	-0.279424897	0.100542616	0.173196571	0.918630346	Potri.001G146600.2	AT1G32410.3
Potra2n1c1263.1	0.177982345	0.077409796	0.465748646	0.974788153	Potri.001G146700.1	AT3G59840.1
Potra2n1c1264.2	0.777382184	0.135228566	3.98937E-07	0.894138111	Potri.001G146900.1	AT5G18070.1
Potra2n1c1265.1	-0.060346445	-0.118816039	0.81510772	0.941681215	Potri.001G147000.1	AT5G45420.1
Potra2n1c1266.6	0.084381869	-0.181397369	0.834953914	0.878330363	Potri.001G147100.1	AT1G31870.1
Potra2n1c1267.1	0.990503312	0.010014668	0.018118403	0.996719899	Potri.001G147200.2	AT4G17030.1
Potra2n1c1268.1	2.76051815	0.885335095	5.97648E-29	0.377730381	Potri.001G147300.1	AT2G38740.1
Potra2n1c1269.1	0.648548481	0.080295752	0.002924666	0.959881296	Potri.001G147400.1	AT2G38740.1
Potra2n1c127.1	0.418293818	0.087527844	3.56724E-06	0.922993749	Potri.001G015900.1	AT1G73730.1
Potra2n1c1270.1	0.17901546	-0.018242312	0.625460033	0.991089517	Potri.001G147500.2	AT3G60190.1
Potra2n1c1271.1	3.560331047	1.496950567	1.28649E-24	0.293583922	Potri.001G147600.1	AT4G18210.1
Potra2n1c1272.1	-1.075903413	-0.164891068	2.12082E-05	0.938501048	Potri.001G147700.1	AT1G14390.1

Potra2n1c1273.1	1.569612549	0.889487623	0.595404949	NA		
Potra2n1c1274.1	-0.078545452	0.200975893	0.936621629	0.966917036	Potri.001G147800.1	
Potra2n1c1275.1	-0.056110694	0.047249111	0.836700536	0.982422288		
Potra2n1c1276.1	0.013683845	-0.078842737	0.931559055	0.948577865	Potri.003G086700.1	AT2G44610.1
Potra2n1c1277.1	0.270091307	0.089373552	0.004888669	0.935474933	Potri.001G148000.1	AT2G28910.1
Potra2n1c1278.1	0.101168169	0.093802344	0.40072564	0.892986206	Potri.001G148100.1	AT5G47010.1
Potra2n1c1279.1	0.936828474	-0.016714075	0.011081046	0.996292992	Potri.001G148200.1	AT1G73320.1
Potra2n1c128.1	NA	2.32414027	NA	NA	Potri.001G016100.2	
Potra2n1c1280.1	0.058475525	0.344073936	0.746344707	0.310353725	Potri.001G148300.1	AT5G47020.1
Potra2n1c1281.1	0.395032429	0.0192415	0.000497718	0.985393296	Potri.001G148400.2	AT5G47040.1
Potra2n1c1282.1	2.746663603	-0.757196931	0.25995169	NA		
Potra2n1c1283.1	-0.415388897	-0.177213708	0.050539515	0.851087483	Potri.001G148500.1	AT1G32730.1
Potra2n1c1284.1	-0.493693048	-0.181528648	0.035157354	0.88066693	Potri.001G148600.1	AT1G32740.1
Potra2n1c1285.1	-0.871510048	-0.530498154	4.16833E-05	0.599849196	Potri.001G148700.1	AT4G17670.1
Potra2n1c1286.1					Potri.010G022900.1	
Potra2n1c1287.1						
Potra2n1c1288.1	3.241012097	0.660619236	0.181173766	NA	Potri.T170600.1	AT1G37113.1
Potra2n1c1289.1	0.038169466	0.244987752	0.819330054	0.616163505	Potri.001G148800.1	AT4G17140.3
Potra2n1c129.2					Potri.T076201.1	AT5G45400.1
Potra2n1c1290.1	-0.385723706	0.354546404	0.04841862	0.812677788	Potri.001G148900.1	AT4G17090.1
Potra2n1c1291.1	-0.377357869	0.363082138	0.055653511	0.804796567		
Potra2n1c1292.1	0.808371269	-0.020245896	0.003641567	0.99226031	Potri.001G149100.1	AT5G47070.1
Potra2n1c1293.1	-0.176641849	-0.137579017	0.170581104	0.911339141	Potri.001G149200.1	AT2G35190.1
Potra2n1c1294.2	0.256827386	0.249514864	0.024620074	0.886059841	Potri.001G149300.1	AT3G10950.1
Potra2n1c1295.1	0.31963427	0.279360034	0.006238047	0.875559093	Potri.001G149400.1	AT4G17080.1
Potra2n1c1296.1	-0.746720299	-0.319748376	0.148589165	0.910815461	Potri.001G149500.1	
Potra2n1c1297.1	-1.423132358	-0.854962567	0.070554751	0.749684992		
Potra2n1c1298.2	-0.229304112	-0.052100457	0.015030239	0.954476684	Potri.001G149600.1	AT5G47080.1
Potra2n1c1299.1	-2.277249482	0.121846537	NA	NA	Potri.010G027100.2	AT3G21510.1
Potra2n1c13.1	0.349675432	0.228420008	0.022317834	0.719104446	Potri.001G001300.1	AT4G26410.1
Potra2n1c130.1						
Potra2n1c1300.1	-0.338392357	0.062720608	0.047594845	0.974275337	Potri.001G149800.1	AT5G47090.1

Potra2n1c1301.1	-2.578176003	-0.683372352	0.336445189	NA	Potri.001G149700.1	
Potra2n1c1302.1	0.343854335	0.116714354	0.000440909	0.811018642	Potri.001G149900.1	AT4G17620.1
Potra2n1c1303.1	NA	NA	NA	NA	Potri.001G150100.1	AT4G17616.1
Potra2n1c1304.1	0.158129409	0.009705958	0.313499524	0.992934677	Potri.001G150200.1	AT5G47100.1
Potra2n1c1305.1	1.275549539	0.482553877	1.22936E-08	0.563956825	Potri.001G150300.1	AT4G17070.1
Potra2n1c1306.1	0.224618809	-0.075249238	0.223672906	0.948577865	Potri.001G150400.1	AT5G41150.1
Potra2n1c1307.1	0.065226789	0.003320618	0.74226081	0.999276367	Potri.001G150500.2	AT4G33510.1
Potra2n1c1308.1	-0.614881507	-0.115553092	0.005796648	0.948577865	Potri.001G150600.1	AT2G44730.1
Potra2n1c1309.1	-2.721450667	0.828281449	0.067232203	0.862335622	Potri.001G150700.1	AT5G04770.1
Potra2n1c131.1	2.018195243	0.718197208	0.28736795	NA		
Potra2n1c1310.1	-0.990801761	-0.06919107	0.000633099	0.966005919	Potri.003G083600.1	AT4G17050.1
Potra2n1c1311.1	-0.045647021	0.091869404	0.767102601	0.917751882	Potri.001G150900.1	AT4G17610.1
Potra2n1c1312.1	2.632524198	2.307412514	NA	NA	Potri.001G151100.1	AT2G19170.1
Potra2n1c1313.1	NA	NA	NA	NA	Potri.001G151100.1	AT2G19170.1
Potra2n1c1314.1	-0.61910233	0.660619236	NA	NA	Potri.001G151100.1	AT2G19170.1
Potra2n1c1315.1	NA	NA	NA	NA	Potri.001G151150.1	
Potra2n1c1316.1	0.796826484	-0.046370319	NA	NA	Potri.001G151200.1	AT5G07440.3
Potra2n1c1317.1	-0.023446033	-0.273749695	0.936605582	0.832750076	Potri.001G151300.1	AT4G17600.1
Potra2n1c1318.1	1.64774763	NA	NA	NA	Potri.001G151500.1	AT4G17030.1
Potra2n1c1319.1	2.020430669	-0.948622962	0.102904614	NA	Potri.003G082900.1	
Potra2n1c132.1	-0.907742683	-0.224701638	4.93436E-07	0.935099709	Potri.001G016200.1	AT3G20810.1
Potra2n1c1320.1	0.10123246	0.191317061	0.728492402	0.895575326	Potri.001G151700.2	AT4G17570.3
Potra2n1c1321.2	-0.045154764	-0.062150431	0.733379529	0.965535524	Potri.001G151800.2	AT5G47120.1
Potra2n1c1322.1	1.117300403	0.209269915	0.005040577	0.937276488	Potri.001G151900.1	AT4G16970.1
Potra2n1c1323.1	-0.109935957	0.058329503	0.5097101	0.956865545	Potri.001G152000.1	AT3G60600.1
Potra2n1c1324.2	-0.266776097	0.026470316	0.031688287	0.983113084	Potri.001G152100.2	AT5G47180.1
Potra2n1c1325.1	-0.350162824	-0.137464996	0.037282027	0.895474078	Potri.001G152200.1	AT4G17560.1
Potra2n1c1326.1	-0.431076269	-0.198403492	0.177584593	0.912666272	Potri.001G152300.1	AT4G17550.1
Potra2n1c1327.1	0.82357019	0.674963689	NA	NA		
Potra2n1c1328.1	-0.97725024	-0.196900096	0.309525496	0.975578749		
Potra2n1c1329.1	-0.595756871	0.548896882	0.553962342	0.912665563	Potri.003G082200.2	AT4G17540.1
Potra2n1c133.1	3.287977261	0.829672509	3.52533E-25	0.776094736	Potri.001G016300.1	AT5G12890.1

Potra2n1c1330.1	-1.294721784	-0.649653503	1.2606E-10	0.23623457	Potri.001G152500.1	AT5G02890.1
Potra2n1c1331.1	-0.637934825	-1.507841271	NA	NA		
Potra2n1c1332.1	-1.345217457	-0.342071574	1.09297E-10	0.773113207	Potri.001G152600.1	AT2G35660.1
Potra2n1c1333.1	-0.267560834	-0.110559382	0.561096259	0.95946978	Potri.001G152700.2	AT2G35658.2
Potra2n1c1334.1	-0.113749048	0.020298161	0.498935475	0.991089517	Potri.003G081800.1	AT1G02130.1
Potra2n1c1335.1	0.318992525	-0.031708926	0.0188043	0.981702181	Potri.001G153000.1	AT4G17520.1
Potra2n1c1336.1	6.938341952	3.858211306	0.00031584	NA	Potri.001G153100.1	AT4G16820.1
Potra2n1c1337.1	NA	NA	NA	NA	Potri.001G153200.1	AT4G14920.1
Potra2n1c1338.1	-0.12908135	0.0359752	0.476895538	0.983446961	Potri.001G153300.3	AT4G16800.1
Potra2n1c1339.4	0.197682025	0.176709957	0.280866211	0.865091851	Potri.001G153400.1	AT3G27740.1
Potra2n1c134.1						
Potra2n1c1340.1	2.126821066	1.596933797	0.35576261	NA		
Potra2n1c1341.1	2.881158588	1.885226318	NA	NA		
Potra2n1c1342.1	1.639301402	0.454738346	1.33849E-05	0.869327705	Potri.001G153800.1	AT5G60900.1
Potra2n1c1343.1	2.920063888	NA	NA	NA		
Potra2n1c1344.1	2.051068002	0.826462056	1.30459E-05	0.72476666	Potri.001G153800.1	AT5G60900.1
Potra2n1c1345.1	3.918214356	1.356313551	0.002553251	NA	Potri.001G154100.1	AT4G17500.1
Potra2n1c1346.1	1.9008476	1.722044448	1.37783E-10	0.006185055	Potri.001G154200.1	AT5G47230.1
Potra2n1c1347.1	-0.260093082	0.268846252	0.34812748	0.900551258	Potri.001G154300.1	AT5G47240.1
Potra2n1c1348.1	0.002111153	-0.009585249	0.989294206	0.99324929	Potri.001G154400.1	AT5G47310.1
Potra2n1c1349.1	0.998889602	-0.082043223	1.20821E-05	0.963272912	Potri.001G154500.1	AT2G25410.1
Potra2n1c135.1	NA	1.571529889	NA	NA	Potri.003G210400.1	AT5G12890.1
Potra2n1c1350.1	0.030848742	0.099224859	0.830398508	0.907073354	Potri.001G154600.1	AT3G60340.1
Potra2n1c1351.3	-0.172426309	0.01672116	0.130617795	0.990128003	Potri.001G154700.1	AT2G35680.1
Potra2n1c1352.1	-3.655162895	-2.382957004	0.102919287	NA		
Potra2n1c1353.1	-0.159042595	0.034677383	0.481061071	0.983446961	Potri.001G154901.1	AT5G47360.1
Potra2n1c1354.1	-1.562926806	-0.491219919	0.060795301	0.889258723	Potri.001G155000.1	AT4G16790.1
Potra2n1c1355.1	-1.381595193	-0.342278745	0.000200449	0.778013962	Potri.001G155100.1	AT5G47370.1
Potra2n1c1356.1	0.048927806	0.040085339	0.749848389	0.969421568	Potri.001G155200.1	AT2G39810.1
Potra2n1c1357.1	NA	NA	NA	NA		
Potra2n1c1358.1	-0.033006053	-0.059100949	0.849362987	0.962696825	Potri.001G155300.1	AT5G47390.1
Potra2n1c1359.1	-0.61910233	1.411159022	NA	NA	Potri.001G155400.1	AT4G17430.1

Potra2n1c136.1	1.368307583	1.226705359	1.42558E-05	0.651818379	Potri.001G016500.1	AT5G12890.1
Potra2n1c1360.1	0.397697895	-0.013703278	0.000114069	0.990390312	Potri.001G155500.1	AT4G16760.1
Potra2n1c1361.1					Potri.001G155600.1	AT5G13620.1
Potra2n1c1362.1	0.3189412	0.545489309	0.721089714	0.885649686	Potri.001G155700.1	AT2G44940.1
Potra2n1c1363.1	0.368454217	0.031552269	0.004146477	0.976302215	Potri.001G155800.1	AT5G47400.1
Potra2n1c1364.1	0.11354176	0.006493611	0.487910523	0.995203254	Potri.001G155900.1	AT5G47420.1
Potra2n1c1365.2	0.209241604	0.092685887	0.093223608	0.845803338	Potri.001G156000.1	AT5G47430.1
Potra2n1c1366.2	0.766941451	0.089767286	7.06589E-05	0.953608209	Potri.001G156100.1	AT4G16720.1
Potra2n1c1367.1	0.718605914	0.05160231	0.000876287	0.977786506	Potri.001G156200.2	AT4G17380.1
Potra2n1c1368.1	NA	NA	NA	NA	Potri.T076201.1	AT5G45400.1
Potra2n1c1369.1	1.212999622	-1.465929376	NA	NA		
Potra2n1c137.1	0.503865808	0.018408541	0.012696273	0.993617124	Potri.001G016600.1	AT5G12900.1
Potra2n1c1370.1	NA	NA	NA	NA	Potri.019G096800.1	AT5G16250.1
Potra2n1c1371.1	1.64774763	2.407052702	NA	NA	Potri.008G220300.1	
Potra2n1c1372.1	-0.056420285	0.218892878	0.953436176	0.953731124	Potri.002G079400.1	AT1G77380.1
Potra2n1c1373.1	-0.050804671	-0.042985529	0.744544599	0.967103157	Potri.001G156600.1	AT5G47435.2
Potra2n1c1374.1	1.600576518	0.647906335	0.007779144	0.825511903	Potri.001G156700.4	AT4G17350.1
Potra2n1c1375.2	-1.328951947	0.87420356	NA	NA	Potri.001G156900.1	AT3G60510.1
Potra2n1c1376.1	NA	3.320235483	NA	NA	Potri.001G157000.1	AT5G47450.1
Potra2n1c1377.1	NA	NA	NA	NA	Potri.001G157100.1	AT5G13910.1
Potra2n1c1378.1	1.459962178	NA	NA	NA		
Potra2n1c1379.1	-0.215490869	0.532011991	0.919016397	NA	Potri.001G157200.3	AT5G47380.1
Potra2n1c138.1	0.47118098	3.330908145	0.809676507	0.335923144		
Potra2n1c1380.3	-0.111632675	0.226330648	0.426222384	0.71102669	Potri.001G157300.1	AT4G17330.1
Potra2n1c1381.1	0.050135263	0.082832176	0.777168504	0.937276488	Potri.001G157400.1	AT4G16650.1
Potra2n1c1382.1	-1.363135115	-0.609543964	7.60883E-06	0.453004317	Potri.001G157500.1	AT4G16640.1
Potra2n1c1383.1	2.677565659	0.192284761	9.98972E-11	0.965249957	Potri.001G157600.1	AT5G47470.1
Potra2n1c1384.1	-0.69501899	-0.159823617	0.001606287	0.929248075	Potri.001G157700.1	AT3G60580.1
Potra2n1c1385.1	-0.05243091	-0.016697277	0.732115285	0.986636967	Potri.001G157800.1	AT5G47490.1
Potra2n1c1386.1	0.020981814	-0.147247582	0.921959326	0.879379661		
Potra2n1c1387.2	0.486317436	0.165016092	0.022484112	0.896314971	Potri.001G157900.1	AT3G03490.1
Potra2n1c1388.1	-0.013553905	-0.012420456	0.967960093	0.995268765	Potri.001G158000.1	AT2G35710.1

Potra2n1c1389.1	-0.374298682	-0.281465574	0.600413831	0.946322185	Potri.001G158100.1	
Potra2n1c139.1	-0.051795334	0.120026819	0.81059783	0.929052703	Potri.001G016800.2	AT5G12920.1
Potra2n1c1390.1	0.427680027	0.195671409	0.103686272	0.824378527	Potri.001G158200.1	AT2G35720.1
Potra2n1c1391.1	-0.284600446	0.451425272	NA	NA		
Potra2n1c1392.1	1.951129464	0.207920826	0.336887292	NA	Potri.001G158400.1	AT4G12520.1
Potra2n1c1393.2	-0.637934825	-0.097655521	NA	NA	Potri.001G158500.1	AT4G16570.1
Potra2n1c1394.1	5.755008694	2.063008321	NA	NA	Potri.001G158600.1	AT4G16563.1
Potra2n1c1395.1	NA	0.674963689	NA	NA		
Potra2n1c1396.1	2.340207562	4.777218018	NA	NA	Potri.001G158700.1	AT5G47510.1
Potra2n1c1397.1	4.486717729	2.155079178	8.79137E-18	0.024748697	Potri.001G158800.1	AT4G39720.1
Potra2n1c1398.1	-0.351447456	-0.185349323	0.088900791	0.924309478	Potri.001G158900.1	AT5G44790.1
Potra2n1c1399.1	-0.100312073	0.152039447	0.61702876	0.921099436	Potri.001G159000.1	AT2G35010.1
Potra2n1c14.1	0.029588617	0.023935938	0.892624746	0.986636967	Potri.001G001400.1	AT1G55535.1
Potra2n1c140.1	-0.171954956	0.262146824	0.606501166	0.89438295	Potri.001G016900.1	AT5G10400.1
Potra2n1c1400.1	-3.519013884	-0.411504191	2.7649E-08	0.600720177	Potri.001G159200.1	AT1G31040.1
Potra2n1c1401.1	-2.626062229	-0.350026918	6.582E-09	0.748560495	Potri.001G159300.1	AT5G05280.1
Potra2n1c1402.1	-0.602047637	-0.055646903	1.42165E-07	0.950095151	Potri.001G159400.1	AT2G32260.1
Potra2n1c1403.2	0.350016028	0.251497528	0.009003581	0.758676613	Potri.001G159600.1	AT2G35035.1
Potra2n1c1404.1	-0.025376157	-0.107861175	0.763025203	0.798218026	Potri.001G159700.1	AT2G35020.1
Potra2n1c1405.1	1.878241999	0.078621699	5.69137E-07	0.984292161	Potri.001G159900.1	AT4G20050.2
Potra2n1c1406.1	0.530141702	0.375463402	0.003213365	0.590087814	Potri.001G160100.1	AT4G20070.1
Potra2n1c1407.1	-0.555417142	-0.10091144	0.005507735	0.905231727	Potri.001G160300.2	AT1G52310.1
Potra2n1c1408.1	1.512143901	1.406622907	0.592797668	NA	Potri.001G160400.1	AT3G12750.1
Potra2n1c1409.1	0.115613017	0.012070043	0.36611179	0.990138406	Potri.001G160500.1	AT1G05780.1
Potra2n1c141.1	0.977858086	0.362670432	0.008854347	0.813769073	Potri.001G017000.1	AT4G12070.1
Potra2n1c1410.1	-0.096877259	0.04521035	0.405047846	0.966228173	Potri.001G160700.1	AT1G30970.1
Potra2n1c1411.2	-0.065087919	-0.139291739	0.800435111	0.903698951	Potri.001G160800.1	AT1G30960.1
Potra2n1c1412.1	-1.594535097	-1.329585374	0.668785127	NA	Potri.001G160900.1	AT1G30950.1
Potra2n1c1413.1	-2.137186149	-0.767942194	3.28021E-14	0.119705213		
Potra2n1c1414.1	-0.508944921	-0.121793412	0.046611067	0.905145891	Potri.001G161000.1	AT1G72300.1
Potra2n1c1415.1	0.086998741	-0.041243555	0.581732972	0.973006135	Potri.001G161100.1	AT1G53710.1
Potra2n1c1416.2	2.906841419	0.52117121	1.92475E-51	0.779493734	Potri.001G161400.1	AT1G17260.1

Potra2n1c1417.1	0.127559815	0.135246091	0.324740178	0.859813626	Potri.001G161500.1	AT3G14360.1
Potra2n1c1418.2	-0.020123183	0.03141863	0.92667941	0.983113084	Potri.001G161600.1	AT1G53730.1
Potra2n1c1419.2	0.417120965	0.264350322	0.000245306	0.71719508	Potri.001G161700.1	AT1G53750.1
Potra2n1c142.1	0.319910694	-0.182059514	0.608630587	0.954559968		
Potra2n1c1420.1	-0.244918632	-0.255435194	0.163895205	0.663037599	Potri.001G161800.1	AT1G53760.1
Potra2n1c1421.1	-0.294390032	-0.073471489	0.007596411	0.948002367	Potri.001G161900.1	AT1G53800.1
Potra2n1c1422.1	-0.41932629	-0.123239159	9.0234E-05	0.865611082	Potri.001G162200.2	AT5G50430.1
Potra2n1c1423.1	0.939936769	-0.266637518	3.22789E-05	0.909675463	Potri.001G162000.1	AT1G72310.1
Potra2n1c1424.1	0.87504857	-0.034748131	0.228167606	0.995621291	Potri.001G162300.2	
Potra2n1c1425.1	0.406839153	-0.343172656	0.077758122	0.859892315	Potri.001G162400.1	AT3G14310.1
Potra2n1c1426.1	2.870322969	0.370058585	0.001606261	0.949966783	Potri.001G162500.1	AT3G14310.1
Potra2n1c1427.1	3.037928972	0.33814703	0.000412118	0.956865545	Potri.001G162500.1	AT3G14310.1
Potra2n1c1428.1	0.748891154	0.106624442	0.121458027	0.974367223	Potri.001G162800.1	AT1G72330.1
Potra2n1c1429.1	0.075174492	-0.012165229	0.656863311	0.992934677	Potri.001G162900.1	AT3G14290.1
Potra2n1c143.1	-0.367171048	-0.331325616	0.113507513	0.60449179	Potri.001G017300.4	AT1G51140.1
Potra2n1c1430.1	-0.61910233	-0.767708831	NA	NA		
Potra2n1c1431.2	-0.054222141	-0.063959777	0.924054565	0.983113084	Potri.001G163000.1	AT1G53860.1
Potra2n1c1432.1	-0.377402182	-0.041997501	0.001884093	0.9761362	Potri.001G163100.1	AT3G14280.1
Potra2n1c1433.3	-0.085248757	0.103072341	0.46876636	0.910878765	Potri.001G163200.1	AT3G14270.1
Potra2n1c1434.1	0.655294432	0.07576808	1.59144E-06	0.935474933	Potri.001G163300.1	AT1G53880.1
Potra2n1c1435.1	-0.196909319	-1.401985753	0.800598724	0.380665007	Potri.001G163400.1	AT1G53885.1
Potra2n1c1436.1	-1.797071313	-0.552629031	0.175184209	0.909675463	Potri.002G192300.10	AT1G26540.1
Potra2n1c1437.1	0.641045241	0.359408689	0.000211511	0.874507876	Potri.001G163600.1	AT3G14240.1
Potra2n1c1438.1	0.127839995	-0.058214515	0.39627044	0.972159498	Potri.001G163700.1	AT1G53910.3
Potra2n1c1439.1	2.340207562	3.399920631	NA	NA	Potri.001G163800.2	AT2G01080.1
Potra2n1c144.1	NA	NA	NA	NA	Potri.001G017400.1	AT1G76320.1
Potra2n1c1440.1						
Potra2n1c1441.3	0.265957926	0.265034435	0.026832895	0.699520904	Potri.001G163900.10	AT3G14205.1
Potra2n1c1442.1	0.525032825	0.376144322	0.000371807	0.557595248	Potri.001G164000.1	AT1G72370.1
Potra2n1c1443.1	0.582218402	0.26748303	6.93421E-06	0.741833788	Potri.001G164100.1	AT1G72440.1
Potra2n1c1444.1	-1.35927387	-1.507841271	NA	NA	Potri.001G164200.1	AT1G54050.1
Potra2n1c1445.1	-1.497581316	-0.420705505	1.07164E-05	0.79819686	Potri.001G164300.1	AT1G72430.1

Potra2n1c1446.1					Potri.001G164400.1	
Potra2n1c1447.1	NA	2.676364087	NA	NA	Potri.001G164500.1	AT1G17350.2
Potra2n1c1448.2	-0.246816626	1.732860244	0.628672236	NA	Potri.001G164800.2	AT1G54070.1
Potra2n1c1449.4	-0.205511733	0.248174719	0.350564241	0.729625623	Potri.001G164900.2	AT1G72410.1
Potra2n1c145.1	4.405087853	2.384917139	0.000461054	0.715585895	Potri.001G017500.1	AT3G20820.1
Potra2n1c1450.1					Potri.001G165000.1	AT3G28860.1
Potra2n1c1451.1	1.64774763	2.676364087	NA	NA	Potri.001G165000.1	AT3G28860.1
Potra2n1c1452.1	NA	NA	NA	NA	Potri.001G165200.1	AT3G54620.1
Potra2n1c1453.1	-0.075641798	-0.053980119	0.636131754	0.95671968	Potri.001G165600.1	AT1G11890.1
Potra2n1c1454.1	NA	NA	NA	NA		
Potra2n1c1455.1	NA	0.674963689	NA	NA		
Potra2n1c1456.3	0.222920009	0.101151899	0.049620775	0.897285619	Potri.001G165800.1	AT1G72390.1
Potra2n1c1457.1	0.28397909	0.104894118	0.001780662	0.865591169	Potri.001G165900.1	AT3G14120.2
Potra2n1c1458.1	0.088934181	0.793968447	0.737946952	0.375012853	Potri.001G166000.1	AT3G14110.1
Potra2n1c1459.2	-0.011798304	0.007274185	0.93600412	0.996032145	Potri.001G166100.4	AT1G17370.1
Potra2n1c146.1	0.523772406	0.32158696	0.590786307	0.953831244		
Potra2n1c1460.1	-0.067571896	0.07950618	0.759846246	0.94795983		
Potra2n1c1461.1	6.878312201	3.189476289	4.65093E-11	0.058708336	Potri.001G166200.2	AT1G17380.1
Potra2n1c1462.1	NA	1.363811008	NA	NA	Potri.001G166300.1	AT3G42880.1
Potra2n1c1463.2	0.034037017	0.035728707	0.847259627	0.973347974	Potri.001G166500.1	AT1G72480.1
Potra2n1c1464.1	-0.187634563	0.01050983	0.254106179	0.993577196	Potri.001G166400.4	AT1G72470.1
Potra2n1c1465.1	0.833905992	0.17979645	0.015643739	0.962410508		
Potra2n1c1466.1	-0.22256855	-0.097112837	0.060405767	0.910815461	Potri.001G166600.1	AT3G14080.1
Potra2n1c1467.2	-1.35927387	-1.507841271	NA	NA	Potri.001G166700.1	AT1G72490.1
Potra2n1c1468.1	0.784357994	-0.275013871	0.000869385	0.885375133	Potri.001G166800.1	AT1G19110.1
Potra2n1c1469.1	1.434208122	2.181985985	NA	NA		
Potra2n1c147.1	0.855620312	0.204889818	0.078920145	0.936890993		
Potra2n1c1470.1	1.426609633	0.064045114	0.001187433	0.985393296	Potri.001G166900.1	AT1G72510.1
Potra2n1c1471.1	0.075419248	0.490883934	0.80381175	0.72968165	Potri.001G167100.3	AT1G54100.2
Potra2n1c1472.1	-0.106826179	-0.027144557	0.572055161	0.983446961	Potri.001G167200.1	AT1G54115.1
Potra2n1c1473.1	-0.233274517	-0.20865559	0.392650018	0.872201371	Potri.001G167300.1	AT3G14067.1
Potra2n1c1474.1	0.095611268	0.087212117	0.30298705	0.922993749	Potri.001G167400.1	AT5G20570.1

Potra2n1c1475.1						
Potra2n1c1476.1	2.152629095	0.740696661	3.80037E-12	0.554040943	Potri.001G167700.1	AT1G17420.1
Potra2n1c1477.1	1.675906683	-0.312208374	2.86077E-05	0.915580711	Potri.001G167800.1	AT5G07990.1
Potra2n1c1478.1	5.317469812	2.447087471	1.72373E-34	0.003507774	Potri.001G167900.1	AT5G07990.1
Potra2n1c1479.1	6.787334476	2.653585531	1.8553E-48	0.425573107	Potri.001G168000.1	AT2G05940.1
Potra2n1c148.1	-0.781302253	-0.464226126	0.008781757	0.728883321	Potri.001G018700.1	AT3G20860.1
Potra2n1c1480.3	0.388865972	0.240632307	0.011643717	0.720242695	Potri.001G168100.1	AT1G72560.1
Potra2n1c1481.1	-0.05561286	-0.033743731	0.796540559	0.9761362	Potri.001G168200.1	AT1G54130.1
Potra2n1c1482.1	-0.017183518	0.052654693	0.899597478	0.943377754	Potri.001G168300.2	AT1G54140.1
Potra2n1c1483.1	0.126419704	0.301477455	0.863953054	0.940687904	Potri.001G168400.1	
Potra2n1c1484.1	-1.549958593	0.05189972	0.035316348	0.98177802	Potri.001G168600.1	AT1G17430.1
Potra2n1c1485.2	-0.599859266	-0.194018717	0.018392935	0.859892315	Potri.001G168700.1	AT1G54150.1
Potra2n1c1486.1	-0.749207536	-0.484084826	0.168520961	0.842247476	Potri.001G168800.1	AT3G13840.1
Potra2n1c1487.1	-0.418408071	-0.672200177	0.595365696	0.859892315		
Potra2n1c1488.1	-1.523269385	-0.002493252	0.185060757	0.999937671		
Potra2n1c1489.1	-1.317292482	-1.465929376	NA	NA		
Potra2n1c149.1	0.350062895	0.140610531	0.027578046	0.894276674	Potri.001G018800.1	AT3G20870.1
Potra2n1c1490.1	NA	NA	NA	NA	Potri.001G169000.2	AT1G72610.1
Potra2n1c1491.1	NA	3.145099921	NA	NA	Potri.001G229400.1	
Potra2n1c1492.1	NA	1.363811008	NA	NA	Potri.001G169100.1	AT3G13960.1
Potra2n1c1493.1	-0.899387973	-2.207602935	0.306951048	0.514425946	Potri.001G169500.1	AT1G72570.1
Potra2n1c1494.1	-0.048007929	-1.751598208	0.980234048	NA	Potri.001G169600.1	AT5G40330.1
Potra2n1c1495.2	-0.72097533	-0.16762963	5.15332E-05	0.886281281	Potri.001G169700.1	AT1G54210.1
Potra2n1c1496.1	-0.251556843	0.087364562	0.643242595	0.981772014		
Potra2n1c1497.1	0.254837252	0.114303503	0.040699434	0.897789832	Potri.001G169900.1	AT1G17440.1
Potra2n1c1498.1	1.020032691	0.4716517	0.05726738	0.852534479	Potri.001G170000.1	
Potra2n1c1499.1	1.031559877	0.657367875	0.084336805	0.775519829	Potri.003G064000.6	AT1G17450.2
Potra2n1c15.1	-0.09133479	-0.039354874	0.597840156	0.9761362	Potri.001G001500.1	AT5G56340.1
Potra2n1c150.1	-1.89486399	-1.107644382	0.145792888	0.79819686	Potri.001G018900.1	AT1G51220.1
Potra2n1c1500.1	-0.250370144	0.018441403	0.220971429	0.989878071		
Potra2n1c1501.2	-0.372667908	0.066006774	0.004274063	0.948002367	Potri.001G170200.1	AT1G72650.2
Potra2n1c1502.1	0.267497738	0.155584767	0.074463837	0.80069536	Potri.001G170300.3	AT1G72660.2

Potra2n1c1503.1	-0.130985446	-0.012948724	0.49919606	0.994762886	Potri.001G170400.1	AT2G06530.1
Potra2n1c1504.2	-1.52953027	-0.192173898	1.44921E-07	0.860325259	Potri.003G063400.1	AT3G13980.1
Potra2n1c1505.1	0.133247479	0.079327177	0.316748512	0.941115972	Potri.001G170700.1	AT3G13990.2
Potra2n1c1506.1	-0.610556873	-0.01951836	0.003980272	0.992934677	Potri.001G170800.1	AT3G14000.1
Potra2n1c1507.1	-0.255899768	-0.023563142	0.009318419	0.981401592	Potri.001G171000.1	AT5G13640.1
Potra2n1c1508.2	-0.050866393	-0.04336623	0.676079176	0.950878725	Potri.001G171100.1	AT1G54170.1
Potra2n1c1509.1	-0.034436743	-0.017417612	0.841736751	0.989300076	Potri.001G171200.1	AT1G52600.1
Potra2n1c151.1	-0.125124136	0.196358821	0.478048019	0.862830524	Potri.001G019000.1	AT5G19620.1
Potra2n1c1510.1	-0.303296541	0.432986048	0.708027527	0.86212354	Potri.001G171300.1	AT1G80150.1
Potra2n1c1511.1	-0.708423754	-0.006901683	0.004544463	0.99819408	Potri.001G171300.1	AT1G80150.1
Potra2n1c1512.1	1.195291534	1.41919648	0.021019117	0.605100478	Potri.001G171600.1	AT1G80160.1
Potra2n1c1513.1	0.546161216	0.33676052	7.98881E-05	0.583309532	Potri.001G171800.1	AT1G15390.1
Potra2n1c1514.1	0.473108797	0.03267595	0.173729118	0.988065578	Potri.001G171900.3	AT1G80170.1
Potra2n1c1515.1	0.794247217	-0.032305226	0.011292845	0.990896655	Potri.001G172100.1	AT1G15400.3
Potra2n1c1516.1	0.709326881	0.032737555	0.004853546	0.989304496	Potri.001G172300.1	AT1G12280.1
Potra2n1c1517.1	-0.135101139	0.053731538	0.143809517	0.930203195	Potri.001G172400.2	AT4G25970.1
Potra2n1c1518.1	1.708775008	1.937106897	0.72215261	NA		
Potra2n1c1519.1	0.364416537	-0.179189277	0.118957846	0.94728769	Potri.001G172500.1	AT3G49890.1
Potra2n1c152.1	-0.264743849	0.281692166	0.259349238	0.837332339	Potri.001G019100.1	AT1G63440.1
Potra2n1c1520.1	0.215517243	0.09584846	0.102813261	0.903934429	Potri.001G172600.2	AT3G15690.2
Potra2n1c1521.1	-0.328470158	-0.760589837	0.717181035	0.828847087	Potri.001G172700.1	AT3G15680.1
Potra2n1c1522.1	-0.107516382	0.258252024	0.496725742	0.772556533	Potri.001G172800.1	AT1G80210.1
Potra2n1c1523.1	0.48181636	-0.097625916	0.882359232	NA	Potri.001G172900.1	AT1G52680.1
Potra2n1c1524.1	NA	NA	NA	NA	Potri.001G173000.1	AT1G52690.1
Potra2n1c1525.1	0.542440742	0.173668644	0.002110682	0.892986206	Potri.001G173100.1	AT1G15420.1
Potra2n1c1526.1	0.322001214	-0.049921881	0.014584552	0.978203092	Potri.001G173500.1	AT1G15440.1
Potra2n1c1527.2	0.05022222	0.161919222	0.978246302	NA	Potri.001G173600.2	AT3G22780.1
Potra2n1c1528.3	0.958568401	0.251816078	0.141289975	0.952831766	Potri.001G173700.1	AT1G52700.1
Potra2n1c1529.1	0.061874799	0.145900035	0.787550129	0.885375133	Potri.001G173800.1	AT1G80230.1
Potra2n1c153.2	-0.239431526	0.123570454	0.090949562	0.914903229	Potri.001G019200.1	AT1G76870.1
Potra2n1c1530.1	-0.25818537	0.128598339	0.074714037	0.88643982	Potri.001G173900.1	AT4G32440.1
Potra2n1c1531.3	0.474516031	0.299002965	9.04591E-07	0.270576747	Potri.001G174100.4	AT5G08470.1

Potra2n1c1532.1	1.517671607	NA	NA	NA	Potri.007G128300.1	AT3G59410.1
Potra2n1c1533.1	-2.060272237	-1.778788983	0.22244331	NA		
Potra2n1c1534.1	-2.137148715	-1.266735241	0.419791748	NA		
Potra2n1c1535.1	-0.276218874	-0.0643074	0.238707927	0.983113084	Potri.001G174300.1	AT1G52720.1
Potra2n1c1536.1	-1.451968267	-3.135330602	0.703505858	NA	Potri.001G174400.1	AT5G25460.1
Potra2n1c1537.1	-2.580869438	-2.160808786	0.017277963	0.428801064	Potri.001G174500.2	AT1G15460.1
Potra2n1c1538.1	0.238681285	0.010545374	0.086381527	0.991407187	Potri.001G174600.1	AT1G52730.1
Potra2n1c1539.4	1.339444557	0.433348048	0.54896104	NA	Potri.001G174700.1	AT5G11470.1
Potra2n1c154.1	0.35002096	-0.238972868	0.156569544	0.885375133	Potri.001G019400.1	AT5G13490.2
Potra2n1c1540.1	-0.205796582	-0.215368437	0.332359589	0.751428922	Potri.001G174900.1	AT1G80280.1
Potra2n1c1541.1	-0.061794108	-0.233805988	0.807477168	0.862830524	Potri.001G175000.1	AT1G52760.1
Potra2n1c1542.1	-0.088854337	-0.311564619	0.752392237	0.787393135		
Potra2n1c1543.1	0.941618527	0.318065524	0.00040496	0.828625141	Potri.001G175200.1	AT1G80300.1
Potra2n1c1544.1	-0.343469804	-0.657205267	0.320296174	0.441009549	Potri.001G175400.1	AT1G52770.1
Potra2n1c1545.1	-0.257175182	-0.721740163	0.60642145	0.598566355		
Potra2n1c1546.1	0.51692761	0.042318304	0.335126284	0.992934677	Potri.001G175500.1	AT3G15550.1
Potra2n1c1547.2	-0.784137062	-1.068844997	0.474450227	0.881526761	Potri.001G175700.1	AT1G15520.1
Potra2n1c1548.1						
Potra2n1c1549.1	3.241544177	1.394613675	NA	NA	Potri.001G175900.2	AT1G52820.1
Potra2n1c155.2	-0.114063914	-0.095943559	0.634636047	0.953831244	Potri.001G019500.1	AT2G22410.1
Potra2n1c1550.1	7.356176489	4.90072246	6.13423E-09	NA	Potri.001G176100.1	AT1G52800.1
Potra2n1c1551.1	6.017229813	3.277480885	0.00307382	NA	Potri.001G176100.1	AT1G52800.1
Potra2n1c1552.1	NA	NA	NA	NA	Potri.001G176200.1	AT1G52820.1
Potra2n1c1553.1						
Potra2n1c1554.2	NA	NA	NA	NA	Potri.001G176500.1	AT1G52800.1
Potra2n1c1555.1	-4.039211861	-0.532040467	9.06302E-06	0.859892315	Potri.001G176600.1	AT1G15550.1
Potra2n1c1556.1	0.23351152	-1.478536539	NA	NA	Potri.001G176800.1	AT4G14746.1
Potra2n1c1557.1	0.090465314	0.058645851	0.536074777	0.951394085	Potri.001G176900.1	AT1G80350.1
Potra2n1c1558.1	0.080296883	-0.081110802	0.605927278	0.947969749	Potri.001G177000.2	AT1G80360.1
Potra2n1c1559.2	-0.082449327	-0.23778203	0.869978373	0.935099709	Potri.001G177100.1	AT1G80370.1
Potra2n1c156.1	6.042139202	3.774788531	1.57368E-06	NA		
Potra2n1c1560.1	0.057647334	-0.083331978	0.868335116	0.972336961	Potri.003G058300.1	AT2G15690.1

Potra2n1c1561.1	0.098011234	0.4456682	0.906796841	0.829862776	Potri.001G177200.1	AT2G15690.1
Potra2n1c1562.1	0.275884016	0.184561883	0.285264823	0.930159505	Potri.001G177300.4	AT1G80380.2
Potra2n1c1563.1	1.398872204	-1.293661617	0.138608357	0.821709751	Potri.001G177350.1	AT1G52827.1
Potra2n1c1564.1	0.335639481	0.00453906	0.290190554	0.999325699	Potri.001G177400.1	AT1G04250.1
Potra2n1c1565.1	0.726045291	-0.565652206	0.090272844	0.871720501	Potri.001G177500.1	AT3G15540.1
Potra2n1c1566.1	-1.673977449	-0.098888051	0.226409323	0.989864467		
Potra2n1c1567.1	4.053661696	0.975293868	0.001531977	NA	Potri.001G177600.1	AT1G08510.1
Potra2n1c1568.1	3.840837094	1.021008015	1.55193E-09	0.828405846	Potri.001G177600.1	AT1G08510.1
Potra2n1c1569.1	0.148849685	0.052086267	0.181086486	0.953768643	Potri.001G178000.2	AT1G80410.1
Potra2n1c157.1	0.82357019	NA	NA	NA		
Potra2n1c1570.1	-0.004536237	0.141307537	0.981929744	0.874808359	Potri.001G178100.4	AT1G80420.1
Potra2n1c1571.1	0.04920948	0.186953631	0.819160313	0.857189843	Potri.001G178200.1	AT1G15660.1
Potra2n1c1572.1	0.315134595	0.242560652	0.665049283	0.95981322	Potri.001G178300.1	AT1G80440.1
Potra2n1c1573.1	-1.19535525	-0.552811255	0.250639102	0.895560236	Potri.001G178500.1	AT1G15670.1
Potra2n1c1574.10	-0.145126247	0.091069196	0.357885813	0.922993749		
Potra2n1c1575.1	-0.326354004	-0.059390976	0.020304302	0.957872702	Potri.001G179000.7	AT3G15940.2
Potra2n1c1576.1	0.188085637	0.119194981	0.081420465	0.884463725	Potri.001G179100.1	AT1G52380.1
Potra2n1c1577.1	0.096360731	-0.012717212	0.467808783	0.993191555	Potri.001G179200.2	AT1G52360.1
Potra2n1c1578.1	-0.976659729	-0.370103986	0.011479445	0.903698951	Potri.001G179400.2	AT3G15990.1
Potra2n1c1579.1	-0.209192852	-0.096080719	0.562677144	0.967076029		
Potra2n1c158.1	6.86879941	5.027713723	5.38768E-06	NA		
Potra2n1c1580.2	-0.24095466	-0.172038425	0.424446745	0.922011871	Potri.001G179500.2	AT1G79990.1
Potra2n1c1581.2	-0.207642627	0.160588866	0.240443307	0.832240074	Potri.001G179600.1	AT3G16000.1
Potra2n1c1582.2	-0.177935443	0.072658371	0.360189689	0.930610374	Potri.001G179700.1	AT5G11460.1
Potra2n1c1583.1	-0.299928998	0.156708422	0.10856544	0.911094206	Potri.001G179800.2	AT3G24490.1
Potra2n1c1584.1	NA	NA	NA	NA	Potri.001G179900.1	
Potra2n1c1585.1	1.545832463	2.303900616	NA	NA	Potri.019G057100.1	AT5G47540.1
Potra2n1c1586.3	0.288932024	0.202292585	0.083880284	0.769631196	Potri.001G180000.1	AT3G16010.1
Potra2n1c1587.1	0.146384098	-0.017238722	0.182647361	0.987226805	Potri.001G180100.1	AT1G79920.1
Potra2n1c1588.1	0.973873422	0.095578728	6.13921E-06	0.97137556	Potri.001G180200.1	AT1G79950.1
Potra2n1c1589.1	7.070059345	2.676364087	1.18716E-07	NA	Potri.001G180400.1	AT1G79960.1
Potra2n1c159.1	1.011457215	0.189796083	0.004212271	0.937687311		

Potra2n1c1590.1	6.445418833	NA	1.32907E-06	NA	Potri.001G180500.1	AT2G25735.1
Potra2n1c1591.1	-1.297720463	-0.290675206	0.000122986	0.833952007	Potri.001G180600.3	AT1G52330.1
Potra2n1c1592.1	-1.846569106	-0.433470714	1.58694E-05	0.71411664		
Potra2n1c1593.1	3.372556152	0.79828902	1.09657E-10	0.824905557	Potri.001G181000.2	AT5G07610.1
Potra2n1c1594.1	NA	NA	NA	NA	Potri.005G021850.1	
Potra2n1c1595.1	0.30147256	-0.025451079	0.353599204	0.987226805	Potri.001G181100.1	AT1G52320.2
Potra2n1c1596.1	-0.363808945	-0.086232165	0.037198802	0.946404907	Potri.001G181200.1	AT1G79915.1
Potra2n1c1597.1	-0.786963704	-0.302144912	0.041225451	0.831738681	Potri.001G181300.1	AT1G79910.1
Potra2n1c1598.1	5.049274269	2.10421535	0.000204454	NA	Potri.001G181500.1	AT1G71450.1
Potra2n1c1599.1	2.140962713	0.205770759	4.09223E-05	0.978203092		
Potra2n1c16.1	0.521077095	-0.057949981	6.21578E-05	0.966005919	Potri.001G001600.1	AT5G56350.1
Potra2n1c160.1	0.767297199	0.183295837	0.096380958	0.917788336	Potri.001G019600.5	AT3G63530.2
Potra2n1c1600.1	0.096443259	-0.024501315	0.575165022	0.984292161	Potri.001G181800.2	AT1G15270.1
Potra2n1c1601.1	-0.416130477	-0.004100104	0.032114076	0.998245864	Potri.001G182000.1	AT1G79900.1
Potra2n1c1602.1	-0.194751044	0.007384036	0.388345082	0.996032145	Potri.001G182100.1	AT3G16050.1
Potra2n1c1603.1	0.12285167	-0.036874671	0.628662945	0.98534079	Potri.001G182300.2	AT3G16060.1
Potra2n1c1604.1	0.852303956	2.240762318	0.617322714	0.70570456	Potri.001G182400.2	AT4G33870.1
Potra2n1c1605.1	0.66807857	0.021089725	0.066915062	0.994403137	Potri.001G182600.1	AT1G79890.1
Potra2n1c1606.1	-0.255123958	-0.320456809	0.144345522	0.843443324	Potri.001G182700.2	AT2G25737.1
Potra2n1c1607.1	-0.977370144	0.308806634	0.500774082	0.957989526		
Potra2n1c1608.1					Potri.003G053500.1	AT1G15230.1
Potra2n1c1609.1	0.627898874	0.287116216	0.000528989	0.87458517	Potri.001G182900.1	AT3G16100.1
Potra2n1c161.1	0.753266622	0.155137923	0.238747726	0.97318201	Potri.001G019600.5	AT3G63530.2
Potra2n1c1610.2	0.102930505	0.001222667	0.575165022	0.999458638	Potri.001G183000.2	AT3G24550.1
Potra2n1c1611.1	0.058910592	0.184270202	0.723815711	0.798648673	Potri.001G183100.1	AT1G15240.2
Potra2n1c1612.1	0.455268999	0.182655885	0.043717655	0.886633917	Potri.001G183200.1	AT3G16080.1
Potra2n1c1613.1	1.664677056	1.89838425	NA	NA	Potri.001G183300.2	AT2G25737.1
Potra2n1c1614.1	1.545832463	NA	NA	NA	Potri.001G183400.1	AT2G25737.1
Potra2n1c1615.1	NA	1.571529889	NA	NA	Potri.008G220300.1	
Potra2n1c1616.1	0.10016308	0.116281169	NA	NA		
Potra2n1c1617.1	0.009802841	0.096585302	0.965441168	0.927423678	Potri.001G183500.1	AT3G16110.1
Potra2n1c1618.1	-0.176955741	-0.153098823	0.09361978	0.855587395	Potri.001G183600.1	AT1G45688.1

Potra2n1c1619.1	0.03034002	-0.231646697	0.93113768	0.790362046	Potri.001G183700.1	AT1G79870.1
Potra2n1c162.1	6.12098905	2.363763957	3.60488E-15	NA		
Potra2n1c1620.1	-1.009394123	-0.372864835	0.124682485	0.908294078	Potri.001G183800.2	AT1G79860.1
Potra2n1c1621.1	-0.174656216	-0.205208288	0.168657348	0.817489719	Potri.001G184100.3	AT1G79840.1
Potra2n1c1622.1	-0.334555918	-0.295235227	0.047655976	0.511337291	Potri.001G184000.1	AT1G79850.1
Potra2n1c1623.1	0.018525724	0.105681174	0.908973708	0.917751882	Potri.001G184700.2	AT1G52220.1
Potra2n1c1624.2	0.046064963	0.075676658	0.816783549	0.951726178	Potri.001G184800.1	AT4G29000.1
Potra2n1c1625.4	-0.079949811	0.155543132	0.675413832	0.894276674	Potri.001G185050.1	AT1G79830.1
Potra2n1c1626.1	-0.826526977	-0.718661638	0.354276883	0.898381609	Potri.001G185200.1	AT3G15740.1
Potra2n1c1627.1	-0.217210184	-0.015802878	0.187028905	0.99005311	Potri.001G185300.1	AT1G79820.1
Potra2n1c1628.1	0.229578778	-0.042433486	0.151085672	0.977559521	Potri.001G185500.1	AT3G16170.1
Potra2n1c1629.1	-0.031403609	-0.007210691	0.843058943	0.995579852	Potri.001G185600.4	AT1G80560.1
Potra2n1c163.1	4.80746492	2.774484352	0.043813721	NA	Potri.003G204428.1	
Potra2n1c1630.1	-1.167433715	-0.204420299	2.92618E-14	0.903477395	Potri.001G185700.1	AT1G52190.1
Potra2n1c1631.1	-1.833034929	0.087566127	3.38367E-08	0.965595755	Potri.001G185900.1	AT4G29100.1
Potra2n1c1632.2	-0.011791053	0.136704108	0.964610993	0.862830524	Potri.001G186000.1	AT1G79810.1
Potra2n1c1633.1	NA	NA	NA	NA	Potri.006G261100.1	AT1G79620.1
Potra2n1c1634.1	-0.61910233	1.766779644	NA	NA	Potri.001G186050.1	
Potra2n1c1635.1	-1.271005838	-0.340515891	2.07944E-15	0.714538735	Potri.001G186100.1	AT4G29080.1
Potra2n1c1636.1	-0.144622984	-0.198231013	0.641542016	0.886059841	Potri.001G186200.1	AT3G16200.1
Potra2n1c1637.2	0.089582856	-0.034126103	0.825611899	0.989304496	Potri.001G186500.1	AT3G23330.1
Potra2n1c1638.1	-0.058747537	0.039253526	0.680397013	0.958152146	Potri.001G186600.2	AT3G16230.2
Potra2n1c1639.1	-0.757013498	-0.123006468	0.000640566	0.947969749	Potri.001G186700.1	AT3G16240.1
Potra2n1c164.1	5.050936625	3.7769567	0.027933273	NA	Potri.001G019900.1	
Potra2n1c1640.1	-2.800039036	-0.686913047	0.000202447	0.812629287	Potri.001G186800.2	AT3G16250.1
Potra2n1c1641.2	0.211821054	-0.056211455	0.162005415	0.953177702	Potri.001G186900.1	AT3G16260.1
Potra2n1c1642.1	0.003488877	-0.003886251	0.980496703	0.996851113	Potri.001G187000.1	AT3G16270.1
Potra2n1c1643.1	0.096549163	0.210126417	0.746894426	0.930598202	Potri.001G187100.2	AT5G06220.2
Potra2n1c1644.1	0.212776732	0.041351238	0.234418426	0.973347974	Potri.001G187200.1	AT1G69750.1
Potra2n1c1645.1	-0.451458418	0.010670709	0.105209551	0.996209574	Potri.001G187300.1	AT1G52155.1
Potra2n1c1646.1					Potri.010G012200.1	
Potra2n1c1647.1	-1.129395439	0.150066873	0.221758242	0.975578749	Potri.001G187400.1	

Potra2n1c1648.1	2.29057797	0.20501707	0.002419142	0.98177802	Potri.001G187500.1	AT5G11590.1
Potra2n1c1649.1	3.234302517	2.394600968	1.61911E-05	0.541287579	Potri.001G187600.3	AT1G10340.2
Potra2n1c165.1	0.949877256	-0.868793663	0.752079773	NA	Potri.003G204428.1	
Potra2n1c1650.1	NA	0.674963689	NA	NA	Potri.001G187700.1	AT1G79800.1
Potra2n1c1651.1	2.602314828	0.447005381	3.47227E-17	0.922011871	Potri.001G188000.1	AT3G16300.1
Potra2n1c1652.1	0.300305531	0.087166333	0.007080676	0.926808461	Potri.001G188300.1	AT3G16310.1
Potra2n1c1653.1	-0.513592391	0.018519286	0.111429	0.995920419	Potri.015G007700.1	AT4G27680.1
Potra2n1c1654.1	NA	NA	NA	NA	Potri.001G188400.1	
Potra2n1c1655.1	-1.592337539	-1.981174223	0.205783847	0.50317213	Potri.001G188500.2	AT4G32890.1
Potra2n1c1656.1	-2.318472378	-0.805968296	0.250850701	NA	Potri.001G188700.1	AT1G79770.1
Potra2n1c1657.1	-0.268594166	-0.132284882	0.035227504	0.830056077	Potri.001G188800.1	AT1G52150.1
Potra2n1c1658.1	-0.112548082	-0.213161711	0.954299611	NA	Potri.001G188900.1	AT1G52140.1
Potra2n1c1659.1	-0.225969317	-0.006643679	0.014587638	0.994762886	Potri.001G189100.2	AT2G25760.1
Potra2n1c166.1	1.115458809	-0.868793663	0.700339633	NA	Potri.001G019900.1	
Potra2n1c1660.1	-0.127113175	0.054653208	0.565437769	0.974275337	Potri.001G189200.1	AT1G15215.2
Potra2n1c1661.1	-2.035147001	-0.797812384	1.36639E-10	0.637165039	Potri.001G189400.1	AT1G79760.1
Potra2n1c1662.1	-0.144657032	-0.123258442	0.486883426	0.903398621		
Potra2n1c1663.1	-0.030755504	-0.040277288	0.805584451	0.948577865	Potri.001G190000.2	AT1G79730.1
Potra2n1c1664.1	-0.115167283	-0.180034446	0.702333587	0.922873058	Potri.001G190100.1	AT3G21360.1
Potra2n1c1665.1	0.338931223	-0.03112041	0.670462142	0.98177802	Potri.001G190200.1	AT1G51940.1
Potra2n1c1666.1	-0.786927449	0.459525499	0.012970454	0.819353048	Potri.001G190300.1	AT3G16500.1
Potra2n1c1667.1	-0.262383575	-0.022236372	0.344649205	0.992934677	Potri.001G190400.1	AT2G19860.1
Potra2n1c1668.1	2.735991243	NA	NA	NA	Potri.002G203000.1	
Potra2n1c1669.1	NA	NA	NA	NA		
Potra2n1c167.1	-0.422034116	0.185670177	0.221120316	0.93154079	Potri.001G020200.1	AT4G35040.1
Potra2n1c1670.1	-1.146042498	-0.105281244	0.004630066	0.973948905	Potri.001G190500.1	AT3G16490.1
Potra2n1c1671.1	1.471474423	3.190912229	0.023514254	NA	Potri.001G190600.1	
Potra2n1c1672.1	1.479818665	3.213354293	0.030194629	NA	Potri.001G190700.3	AT5G56550.1
Potra2n1c1673.2	1.096118202	2.003933854	0.270228941	0.517743314	Potri.006G132300.1	AT3G45630.1
Potra2n1c1674.1	-0.519127529	-0.353087552	0.000636275	0.759797728	Potri.001G190800.2	AT2G19770.1
Potra2n1c1675.1	1.012003399	-0.373772352	0.002882806	0.734057255	Potri.001G191000.1	AT4G25150.1
Potra2n1c1676.1	0.361213618	0.154965153	0.00289807	0.774820633	Potri.001G191100.1	AT1G51980.1

Potra2n1c1677.1	-0.064174484	0.046603677	0.67670411	0.960981446	Potri.001G191200.2	AT5G50340.1
Potra2n1c1678.1	-0.152919396	-0.132160853	0.388205125	0.917892599	Potri.001G191300.2	AT1G52080.1
Potra2n1c1679.1	4.727253523	NA	0.010111673	NA	Potri.001G192100.1	AT5G20230.1
Potra2n1c168.1	0.09919477	-0.029459228	0.197307209	0.975578749	Potri.001G020300.13	AT3G51950.1
Potra2n1c1680.1	-0.760149745	0.111443882	0.0122938	0.961820855	Potri.001G192200.1	AT3G15800.1
Potra2n1c1681.1	-0.576512054	0.237778237	0.044079958	0.841517891	Potri.001G192400.1	AT1G15340.1
Potra2n1c1682.1	-0.082028981	-0.075083586	0.761069366	0.972026118	Potri.001G192500.1	AT3G15780.1
Potra2n1c1683.1	-0.011911504	-0.006134917	0.957238887	0.996032145	Potri.001G192700.2	AT5G25360.1
Potra2n1c1684.1	3.406013973	1.070750972	3.85225E-18	0.544461626	Potri.001G192800.1	AT1G52565.1
Potra2n1c1685.1	0.105634649	-0.04922042	0.347211484	0.937276488	Potri.001G193000.1	AT1G52570.1
Potra2n1c1686.2	0.090358161	-0.107397153	0.613600734	0.926106039	Potri.001G193100.1	AT1G15370.1
Potra2n1c1687.1	0.303507116	0.328635668	0.055068176	0.586594557	Potri.001G193300.1	AT3G45890.1
Potra2n1c1688.1	0.210004276	0.122981131	0.467081008	0.950796801		
Potra2n1c1689.1	0.327617275	0.538400444	0.265462734	0.709241338	Potri.001G193400.1	AT4G19160.2
Potra2n1c169.1	NA	2.295459777	NA	NA	Potri.001G020400.1	AT2G03200.1
Potra2n1c1690.1	0.076020109	-0.179012801	0.720594906	0.817452127	Potri.001G193500.1	AT5G62200.1
Potra2n1c1691.1	1.105097857	0.354193544	2.91106E-05	0.817799498	Potri.001G199600.1	AT1G51720.1
Potra2n1c1692.1	0.214471068	0.125593299	0.082349701	0.759797728	Potri.001G193700.1	AT1G51730.1
Potra2n1c1693.1	-0.194233939	0.001560804	0.0875405	0.999325699	Potri.001G193900.1	AT1G51745.1
Potra2n1c1694.1	0.514676301	-0.017917631	0.000789781	0.990128003	Potri.001G194000.1	AT2G19730.1
Potra2n1c1695.1	1.22158228	-1.588078961	0.198244874	0.341455002	Potri.001G194100.1	
Potra2n1c1696.1	-0.205194721	0.031896563	0.605036284	0.991257512		
Potra2n1c1697.1	0.558821751	0.337474312	0.017575538	0.805461106	Potri.001G195900.1	AT5G11730.1
Potra2n1c1698.1	3.065778315	NA	NA	NA		
Potra2n1c1699.1	-0.153117177	-0.137560195	0.322975474	0.813575895	Potri.001G196500.1	AT1G17640.1
Potra2n1c17.1	-0.291844469	0.082520521	0.012772103	0.910815461	Potri.001G001700.1	AT5G56360.1
Potra2n1c170.1	-0.19039634	-0.035941301	0.341981023	0.985393296	Potri.001G020600.1	AT2G41560.1
Potra2n1c1700.1	-0.726098437	0.623674624	0.811428955	NA	Potri.013G100900.1	AT2G01050.1
Potra2n1c1701.1	0.82357019	NA	NA	NA		
Potra2n1c1702.1	-0.273566125	0.000658096	0.077655703	0.999771727	Potri.001G196600.1	AT5G10900.1
Potra2n1c1703.1	-0.274499932	-0.010379643	0.108623543	0.995579852	Potri.001G196700.1	AT3G13780.1
Potra2n1c1704.1	3.134876421	1.571529889	NA	NA	Potri.T055866.1	

Potra2n1c1705.1	-0.198620477	-0.015660245	0.03145957	0.987226805	Potri.001G196800.1	AT1G72880.2
Potra2n1c1706.1	0.5044394	0.017109188	0.191441197	0.996032145		
Potra2n1c1707.1	0.722296758	0.893134555	0.512762239	0.840251436	Potri.001G197000.1	AT3G13890.2
Potra2n1c1708.1	-1.409983009	0.06197156	NA	NA	Potri.001G197100.1	AT1G72670.1
Potra2n1c1709.1						
Potra2n1c171.1	-0.332983474	-0.58762903	0.748538206	0.870614204	Potri.003G204100.1	AT1G07740.1
Potra2n1c1710.2	-0.032639312	-0.067203009	0.847852805	0.935474933	Potri.003G043200.1	AT1G60970.1
Potra2n1c1711.1	0.988140784	0.945725772	NA	NA		
Potra2n1c1712.1	-0.167311669	0.027999691	0.197836675	0.98177802	Potri.001G197300.1	AT1G17720.2
Potra2n1c1713.2	0.003538442	0.064251626	0.980739176	0.948577865	Potri.001G197400.1	AT3G21280.1
Potra2n1c1714.1	NA	NA	NA	NA		
Potra2n1c1715.1	-0.158167761	-0.059493589	0.287286297	0.963257831	Potri.001G197500.1	AT1G17500.1
Potra2n1c1716.1	-0.08574182	0.030407195	0.429516068	0.972159498	Potri.001G197600.1	AT1G72710.1
Potra2n1c1717.1	-0.070281375	0.039807428	0.761100936	0.976302215		
Potra2n1c1718.1	0.230591946	0.045370831	0.293835001	0.974275337	Potri.001G197700.1	AT2G19460.1
Potra2n1c1719.2	0.594869983	0.573225023	0.002016586	0.457390923	Potri.001G197900.1	AT3G13920.1
Potra2n1c172.1	0.227706671	0.036077798	0.200124142	0.98177802	Potri.001G020700.3	AT5G13470.1
Potra2n1c1720.1	NA	NA	NA	NA	Potri.001G198000.3	AT3G13930.1
Potra2n1c1721.1	0.638881403	0.820821994	0.83918994	NA	Potri.001G198000.3	AT3G13930.1
Potra2n1c1722.1	-0.151194349	-0.210045171	0.345670918	0.836066102	Potri.001G198100.1	AT1G17530.1
Potra2n1c1723.1	-0.018786044	1.240800219	0.986519559	0.621109642	Potri.001G198300.2	AT5G26150.1
Potra2n1c1724.2	-0.676599613	0.06916588	0.070390454	0.974275337	Potri.003G044200.1	AT1G72770.1
Potra2n1c1725.1	0.28101541	-0.099625219	0.061220644	0.935474933	Potri.001G198600.1	AT3G13940.1
Potra2n1c1726.1	-0.05277964	0.044446127	0.740749106	0.960670343	Potri.001G198700.1	AT4G26060.1
Potra2n1c1727.1	-0.093516183	0.035858539	0.501212199	0.969421568		
Potra2n1c1728.1	-0.110983971	-0.170239116	0.822521143	0.923348116	Potri.001G198900.1	AT1G72790.1
Potra2n1c1729.1	0.246973266	0.256852387	0.09980717	0.65293511	Potri.001G199100.1	AT1G72810.1
Potra2n1c173.1	0.174565983	0.269583495	0.304993577	0.586851629	Potri.001G020800.1	AT2G41540.3
Potra2n1c1730.2	-0.418053895	0.148741545	0.011635851	0.859892315	Potri.001G199200.1	AT5G20490.1
Potra2n1c1731.1	0.074251734	-0.051697121	0.700649348	0.962661188	Potri.001G199500.1	AT1G51730.1
Potra2n1c1732.1	-0.350229459	0.008489548	0.000833366	0.992934677	Potri.001G199600.1	AT1G51720.1
Potra2n1c1733.1	-0.711004159	-0.352105114	0.005101167	0.751428922	Potri.001G200000.1	AT3G13690.1

Potra2n1c1734.1	0.630933407	1.654475822	0.051152862	0.156405333	Potri.001G200100.1	AT3G13730.1
Potra2n1c1735.1	-0.326229897	-0.087113288	0.042201381	0.948577865	Potri.001G200200.2	AT1G55160.1
Potra2n1c1736.1	-0.345601781	0.227718036	0.285493197	0.895575326	Potri.001G200300.1	AT1G55140.1
Potra2n1c1737.1	0.595589221	-0.154082897	0.419701509	0.921099436	Potri.001G200400.1	AT3G13750.1
Potra2n1c1738.1	0.050119409	-0.16358799	0.892934343	0.927547456		
Potra2n1c1739.2	-0.075160752	0.039452781	0.592912871	0.95946978	Potri.001G200500.2	AT1G15750.3
Potra2n1c174.1	0.338812712	0.212928517	0.023791204	0.7730851	Potri.001G020900.1	AT3G63440.1
Potra2n1c1740.2	-0.056945471	0.049764665	0.681917379	0.950095151	Potri.001G200500.2	AT1G15750.3
Potra2n1c1741.1	-0.153375142	-0.026750512	0.072648889	0.978203092		
Potra2n1c1742.1	3.176046142	1.376818946	1.7748E-07	0.534000676	Potri.001G200700.1	AT1G17620.1
Potra2n1c1743.1	-4.517404127	-0.756933154	0.001564718	0.885375133	Potri.001G200800.1	AT1G72970.1
Potra2n1c1744.2	0.07701323	0.020839494	0.571336669	0.977559521	Potri.001G201000.1	AT1G72960.1
Potra2n1c1745.1	0.233840198	0.005190988	0.732383981	0.999427005		
Potra2n1c1746.1	2.685015931	2.644565975	NA	NA		
Potra2n1c1747.1	1.523347315	0.308976925	2.99927E-05	0.900869139	Potri.001G201300.2	
Potra2n1c1748.2	0.011939136	0.227467174	0.946748295	0.551744322	Potri.001G201400.5	AT1G80480.1
Potra2n1c1749.1	0.713940387	-0.106541471	0.004231513	0.949966783	Potri.001G201500.2	AT1G52510.1
Potra2n1c175.1	0.008546465	0.254258263	0.977696823	0.753881059		
Potra2n1c1750.1	-0.145613301	-0.006156291	0.488724673	0.996032145	Potri.001G201600.3	AT1G52520.1
Potra2n1c1751.1	0.684312201	0.327430839	6.83074E-07	0.72680202	Potri.001G201700.1	AT1G52530.1
Potra2n1c1752.1	-0.00740851	0.058995802	0.967806429	0.955628098	Potri.012G059200.1	AT1G18560.1
Potra2n1c1753.1	-0.201274976	0.090137845	0.044061322	0.88661378	Potri.001G201900.1	AT3G15920.1
Potra2n1c1754.1	0.422967366	0.275269546	0.660529278	0.960981446		
Potra2n1c1755.1	-0.240903479	0.013166917	0.058727055	0.99112251		
Potra2n1c1756.1	-0.259876078	-0.093063765	0.107251497	0.92978159		
Potra2n1c1757.1	-0.358170807	-0.193703256	0.095453696	0.871920317		
Potra2n1c1758.1	-0.096055645	-0.130507841	0.959312932	0.987288338	Potri.001G202100.1	AT1G80080.1
Potra2n1c1759.1					Potri.001G202200.1	AT1G80030.2
Potra2n1c176.4	1.556716724	0.093828494	4.51282E-10	0.966917036	Potri.001G021000.1	AT5G13460.1
Potra2n1c1760.1	5.705440972	2.747655538	6.26887E-09	NA		
Potra2n1c1761.1	-0.044079028	0.022790908	0.865857539	0.989329144	Potri.017G113300.1	AT3G02520.1
Potra2n1c1762.1	1.664677056	NA	NA	NA		

Potra2n1c1763.1	-0.613631697	-0.465852143	0.000646949	0.180635806		
Potra2n1c1764.1	-0.591224311	-0.281694366	1.25448E-05	0.356355995	Potri.001G204000.1	AT3G15890.1
Potra2n1c1765.3	-0.097330118	0.231268254	0.588920571	0.688752768	Potri.001G204900.10	AT1G15780.1
Potra2n1c1766.1	NA	1.363811008	NA	NA	Potri.001G205100.1	AT3G02645.1
Potra2n1c1767.1	NA	NA	NA	NA	Potri.001G205200.1	AT1G23080.1
Potra2n1c1768.1	0.195797531	0.076746657	0.22186156	0.958071483	Potri.001G205300.1	AT5G09420.1
Potra2n1c1769.1	2.561195336	0.811760101	2.2469E-26	0.569995068	Potri.001G205400.2	AT4G33520.2
Potra2n1c177.1	0.023081028	0.061321959	0.92931414	0.965535524	Potri.001G021100.1	AT3G51940.1
Potra2n1c1770.2	-0.025099106	0.129094454	0.859969551	0.821140081	Potri.001G205500.1	AT5G09400.1
Potra2n1c1771.1	-0.068227654	0.186380757	0.655150637	0.817191104	Potri.001G205700.10	AT5G09390.1
Potra2n1c1772.1	-0.091360498	0.26341169	0.374365482	0.865591169	Potri.001G205800.1	AT5G64170.2
Potra2n1c1773.1	NA	NA	NA	NA	Potri.003G023600.1	AT5G64160.1
Potra2n1c1774.1	1.000812763	0.462459753	1.09455E-10	0.088940474	Potri.001G206100.1	AT1G36050.2
Potra2n1c1775.1	0.82357019	1.571529889	NA	NA	Potri.001G206200.1	AT5G09360.1
Potra2n1c1776.1	0.391681634	0.165926523	0.031648665	0.885375133	Potri.001G206400.1	AT2G13840.1
Potra2n1c1777.1	-0.238938222	-0.070776464	0.049249082	0.922029226	Potri.001G206600.1	AT5G64550.1
Potra2n1c1778.1	0.448669295	0.125525445	0.000583015	0.911339141	Potri.001G206700.1	AT4G33430.1
Potra2n1c1779.2	0.478229366	0.221169163	0.088245723	0.930059926	Potri.001G206800.2	AT5G64570.1
Potra2n1c178.1	NA	NA	NA	NA	Potri.019G023500.1	AT5G46170.1
Potra2n1c1780.1	-1.947103562	-0.715892105	0.105694499	0.854980698	Potri.001G206900.1	AT5G64530.1
Potra2n1c1781.1	0.624884047	0.438228828	0.011707767	0.745183467	Potri.001G207000.1	AT3G26410.1
Potra2n1c1782.1	0.450836478	0.333969621	0.001177327	0.724504724	Potri.001G207100.1	AT3G26420.1
Potra2n1c1783.2	-0.254552583	-0.030525983	0.389959228	0.983446961	Potri.001G207500.1	AT5G16850.1
Potra2n1c1784.1	-2.062071908	-1.146828668	0.000434167	0.60449179	Potri.001G207600.1	
Potra2n1c1785.1	0.038136723	0.211670232	0.80041397	0.824731243	Potri.001G207700.1	AT3G12810.1
Potra2n1c1786.1					Potri.T172100.1	AT1G37113.1
Potra2n1c1787.1	0.82357019	NA	NA	NA	Potri.T170600.1	AT1G37113.1
Potra2n1c1788.1	1.545832463	NA	NA	NA	Potri.T170600.1	AT1G37113.1
Potra2n1c1789.1					Potri.T172100.1	AT1G37113.1
Potra2n1c179.1	-0.655381485	-0.21143732	0.526598791	0.971537796	Potri.001G021300.1	
Potra2n1c1790.1	NA	NA	NA	NA	Potri.009G003600.1	
Potra2n1c1791.1	0.099412664	0.091031298	0.445603386	0.910815461	Potri.001G207900.1	AT4G14110.1

Potra2n1c1792.1	0.062095819	0.200415152	0.746409079	0.901137019	Potri.001G208000.1	AT2G01080.1
Potra2n1c1793.1						
Potra2n1c1794.1	0.017815817	0.178569817	0.91479607	0.811159921	Potri.001G208200.1	AT1G15200.1
Potra2n1c1795.1	-0.420536789	-1.292229428	NA	NA	Potri.001G208600.1	AT1G68150.1
Potra2n1c1796.1	0.079957699	-0.185647457	0.673707457	0.826906752	Potri.001G208700.1	AT3G54630.1
Potra2n1c1797.1	-0.80031808	-0.245817383	1.09856E-06	0.786615729	Potri.001G208800.2	AT1G68140.1
Potra2n1c1798.1	-0.88408486	-0.296471537	5.70163E-08	0.674022701		
Potra2n1c1799.1	0.395702342	0.184290578	0.008457543	0.775618506	Potri.011G095700.4	AT5G06350.1
Potra2n1c18.1	0.943183178	0.156090966	0.000176177	0.951394085		
Potra2n1c180.1	-1.192536467	-0.432557968	0.601236857	NA	Potri.001G021400.1	
Potra2n1c1800.1					Potri.001G209000.1	AT4G33230.1
Potra2n1c1801.1	0.018557837	0.178135152	0.922636206	0.79477084	Potri.001G209100.1	AT1G11400.3
Potra2n1c1802.1	1.911189818	-0.767708831	NA	NA	Potri.001G209200.1	AT5G49180.1
Potra2n1c1803.2	NA	1.394613675	NA	NA		
Potra2n1c1804.1	1.875929171	-0.335858081	1.88112E-09	0.922029226	Potri.001G209300.1	AT2G02850.1
Potra2n1c1805.1	-0.370393813	-0.063713944	0.103526327	0.973695414	Potri.001G209400.2	AT2G01070.1
Potra2n1c1806.1	0.040736163	0.051163476	0.847834486	0.957668459	Potri.001G209500.3	AT1G33270.1
Potra2n1c1807.1	-0.732555463	-0.061639785	7.46571E-05	0.95671968	Potri.001G209600.4	AT3G26070.1
Potra2n1c1808.1	-0.493379026	-0.025899496	0.397581402	0.994861477	Potri.003G020600.1	AT2G26730.1
Potra2n1c1809.1	0.455046729	0.129230273	1.22203E-05	0.857189843	Potri.001G209800.1	AT5G64090.1
Potra2n1c181.1	-0.154333674	0.269813082	0.456562033	0.583548322	Potri.001G021500.1	AT1G78100.1
Potra2n1c1810.1					Potri.006G172300.1	
Potra2n1c1811.1	-0.574819819	-0.432609312	0.006302905	0.257634895	Potri.001G210000.1	AT1G15820.1
Potra2n1c1812.1	0.27914614	-0.054390252	0.049652794	0.97521505	Potri.001G210100.2	AT5G64080.1
Potra2n1c1813.1	0.076889986	0.253079851	0.670411799	0.579008759	Potri.001G210200.1	AT1G71240.2
Potra2n1c1814.1	0.147036429	0.339484059	0.426306418	0.573203325	Potri.001G210400.1	AT1G77700.1
Potra2n1c1815.1	1.459962178	NA	NA	NA	Potri.003G019900.1	AT5G17540.1
Potra2n1c1816.1	0.282746489	0.645281476	0.361621096	0.638657271	Potri.001G210500.1	AT5G24690.1
Potra2n1c1817.1	-0.060715037	0.108870706	0.867752477	0.92812284	Potri.001G210600.1	AT1G10790.1
Potra2n1c1818.2	1.042472468	0.857160949	0.05614349	0.701745625	Potri.001G210800.1	AT1G10780.1
Potra2n1c1819.1	-0.16484608	0.037861886	0.379078778	0.979719961	Potri.001G210900.1	AT1G09580.1
Potra2n1c182.1	0.643451849	0.159712774	0.00046551	0.898701658	Potri.001G021600.2	AT1G22220.1

Potra2n1c1820.1	-0.24640704	0.076737894	0.074333879	0.945454119		
Potra2n1c1821.5	-0.26752972	0.223227419	0.130286699	0.749682671	Potri.001G211200.1	AT1G60890.2
Potra2n1c1822.1	0.747304018	0.742334968	0.000307331	0.568587629	Potri.001G211300.1	AT1G10910.1
Potra2n1c1823.1	-0.684041974	-0.511069906	0.654170985	0.943483421	Potri.001G211400.1	AT1G71160.1
Potra2n1c1824.1	-0.486778553	-0.013551063	0.0149952	0.995920419	Potri.001G211500.1	AT1G71180.1
Potra2n1c1825.1	-0.141647214	0.012726807	0.377879679	0.992934677	Potri.001G211600.2	AT1G71190.1
Potra2n1c1826.1	-0.078316663	-0.062724026	0.572448002	0.935751704	Potri.001G211700.1	AT1G60995.1
Potra2n1c1827.1	-0.012725714	-0.130637125	0.949299816	0.821709751		
Potra2n1c1828.1	-3.385705305	-1.186868255	0.276218348	NA	Potri.001G211800.1	AT1G11040.1
Potra2n1c1829.1	1.664677056	1.571529889	NA	NA	Potri.001G211800.1	AT1G11040.1
Potra2n1c183.1	NA	NA	NA	NA	Potri.001G022000.1	
Potra2n1c1830.1	0.028777894	0.139386737	NA	NA	Potri.009G073300.1	AT3G11130.1
Potra2n1c1831.1						
Potra2n1c1832.1	0.796852865	0.16803977	0.747849763	NA		
Potra2n1c1833.1	0.885888257	0.197498581	0.562557172	NA	Potri.001G211900.1	AT1G22030.1
Potra2n1c1834.1	-0.067441439	-0.012986444	0.522299735	0.991089517	Potri.001G212000.1	AT1G22020.1
Potra2n1c1835.1	-1.232788532	0.246957197	0.023920481	0.949966783	Potri.001G212100.1	AT3G16720.1
Potra2n1c1836.1						
Potra2n1c1837.1					Potri.001G212200.1	AT1G08170.1
Potra2n1c1838.1						
Potra2n1c1839.1	1.274482656	0.110081123	NA	NA	Potri.019G028100.1	ATCG00350.1
Potra2n1c184.1	0.82357019	NA	NA	NA	Potri.001G022100.1	
Potra2n1c1840.1	1.664677056	1.571529889	NA	NA	Potri.003G066700.1	ATCG00340.1
Potra2n1c1841.1					Potri.013G141500.1	ATCG00380.1
Potra2n1c1842.1	-1.358878226	-0.61124345	0.001046917	0.683816919	Potri.001G212400.1	AT2G27930.1
Potra2n1c1843.1						
Potra2n1c1844.1	-0.108458123	0.029122827	0.464274485	0.981726976	Potri.001G212600.3	AT2G27900.1
Potra2n1c1845.1	-0.394760388	0.028161604	0.011625487	0.985393296	Potri.001G212700.1	AT1G08190.1
Potra2n1c1846.2	-0.135535795	0.221978056	0.601478995	0.839016145	Potri.009G002300.1	AT5G22860.1
Potra2n1c1847.1	0.00016408	0.18394701	0.999895833	0.973948905	Potri.001G212900.1	AT5G22860.1
Potra2n1c1848.1	-1.312821482	-0.161868126	0.000104296	0.912083068	Potri.001G213400.2	AT5G22860.1
Potra2n1c1849.1	-0.533796873	-0.033435023	0.013021602	0.989878071	Potri.001G213300.1	AT5G22860.1

Potra2n1c185.1	0.92641307	1.562090794	NA	NA	Potri.001G022200.1	
Potra2n1c1850.1	-0.146087181	-0.205930443	0.296473564	0.798673448	Potri.001G213600.2	AT5G22850.1
Potra2n1c1851.1						
Potra2n1c1852.1	3.467831475	1.476921257	1.02726E-40	0.000353566	Potri.001G213700.1	AT2G27880.1
Potra2n1c1853.1	0.115000385	0.176493731	0.237093694	0.742962534	Potri.001G213800.2	AT5G22000.3
Potra2n1c1854.1	-0.246810464	0.050312217	0.012873233	0.948577865	Potri.001G213900.10	AT5G22010.1
Potra2n1c1855.1	0.041423567	-0.183946942	0.850530623	0.890369631	Potri.001G214500.1	AT1G08470.1
Potra2n1c1856.1	8.075411816	4.513756598	1.13795E-18	NA	Potri.001G214600.1	AT2G21100.1
Potra2n1c1857.2	1.172874637	0.258915757	2.9332E-06	0.954419252	Potri.001G214700.2	AT1G08465.1
Potra2n1c1858.1	-0.129956971	0.049387297	0.633460328	0.965215635	Potri.001G214800.2	AT5G22030.2
Potra2n1c1859.1						
Potra2n1c186.3	-0.163965014	0.688267852	0.601295809	0.731800878	Potri.001G022400.1	AT1G22220.1
Potra2n1c1860.1	-0.891715222	-0.294388976	1.06586E-08	0.641037344	Potri.001G215000.1	AT5G22050.2
Potra2n1c1861.1	-0.915097475	-0.096588781	0.218001832	0.981772014	Potri.009G015700.1	AT5G22060.1
Potra2n1c1862.1	-0.196512986	-0.001267961	0.195560993	0.999458638	Potri.001G215200.1	AT3G22190.1
Potra2n1c1863.1	0.061241128	-0.139215499	0.759828758	0.917516356	Potri.001G215400.1	AT5G22070.1
Potra2n1c1864.1	0.094884448	0.004487816	0.654813825	0.99812851	Potri.001G215500.1	AT3G44160.1
Potra2n1c1865.1	NA	0.674963689	NA	NA		
Potra2n1c1866.1	0.001225407	0.127838577	0.99358175	0.738352588	Potri.001G215700.1	AT1G08420.1
Potra2n1c1867.1	-0.06050079	0.005532618	0.571155077	0.995005375	Potri.001G215800.1	AT5G22080.1
Potra2n1c1868.1	-1.821091964	-0.230352832	1.15265E-09	0.924951347	Potri.001G216000.1	AT5G22090.1
Potra2n1c1869.1	-3.234575038	-4.104947783	0.126019931	NA		
Potra2n1c187.1	-0.424265966	0.027183261	0.024340655	0.981423914	Potri.001G022500.2	AT4G31560.1
Potra2n1c1870.1	5.344823169	3.608771122	0.004207298	NA	Potri.018G090966.1	AT5G38280.1
Potra2n1c1871.1					Potri.001G216100.1	AT2G35930.1
Potra2n1c1872.1	1.545832463	NA	NA	NA		
Potra2n1c1873.1					Potri.001G216300.1	AT3G11840.1
Potra2n1c1874.1	-0.073362394	0.209300939	0.690571417	0.771626158	Potri.001G216400.1	AT5G22100.1
Potra2n1c1875.1	-0.077398492	0.020342683	0.720112602	0.988065578	Potri.001G216500.2	AT5G22120.1
Potra2n1c1876.1	-0.232595422	0.218287496	0.153350385	0.83433596	Potri.001G216600.1	AT2G35940.3
Potra2n1c1877.1						
Potra2n1c1878.1	-0.457566461	0.156392023	0.041758498	0.897789832	Potri.001G216900.2	AT2G27230.2

Potra2n1c1879.1	-0.506727897	-0.624510826	0.868816435	0.978917914	Potri.001G217200.1	AT1G08440.1
Potra2n1c188.1	-0.264270868	-0.254446919	0.115926479	0.845132414	Potri.001G022700.1	AT3G08550.1
Potra2n1c1880.1	-3.658736849	-2.398090752	0.411499344	NA	Potri.001G217200.1	AT1G08440.1
Potra2n1c1881.1	NA	NA	NA	NA	Potri.001G217200.1	AT1G08440.1
Potra2n1c1882.1					Potri.001G217500.1	
Potra2n1c1883.1	0.242343669	-0.299135233	0.673233262	0.895474078		
Potra2n1c1884.1	-0.052871135	-0.056560221	0.904979054	0.983446961	Potri.001G217600.1	AT1G05750.1
Potra2n1c1885.2	0.56782882	0.00586221	0.021806474	0.99819408	Potri.001G217700.1	AT3G23750.1
Potra2n1c1886.1	0.223500085	0.025455664	0.177622591	0.985393296	Potri.001G217800.3	AT3G44190.1
Potra2n1c1887.1						
Potra2n1c1888.1	-0.235383758	-0.274490481	0.129427398	0.753052725	Potri.001G218100.1	AT3G44200.1
Potra2n1c1889.1	2.735991243	NA	NA	NA	Potri.001G218200.1	AT2G27260.1
Potra2n1c189.1	1.64774763	NA	NA	NA	Potri.004G196100.1	AT3G14470.1
Potra2n1c1890.1	NA	NA	NA	NA	Potri.009G019900.2	AT2G35980.1
Potra2n1c1891.1	-0.738314185	-0.303809087	1.16469E-06	0.602446338	Potri.001G218300.1	AT2G27260.1
Potra2n1c1892.2	-1.106115623	-0.129752003	5.23583E-11	0.922618347	Potri.001G218400.1	AT3G44380.1
Potra2n1c1893.1	NA	1.571529889	NA	NA	Potri.001G218500.1	AT5G22410.1
Potra2n1c1894.1	NA	0.674963689	NA	NA	Potri.001G218500.1	AT5G22410.1
Potra2n1c1895.1	NA	1.871975723	NA	NA	Potri.001G218500.1	AT5G22410.1
Potra2n1c1896.1	0.895071893	0.322824519	4.98559E-07	0.769631196	Potri.009G019400.2	AT3G52580.1
Potra2n1c1897.1	1.366253619	1.018411562	0.001413408	0.207333286	Potri.001G218800.1	AT3G44350.2
Potra2n1c1898.1	1.354354454	1.037812938	0.001443337	0.191991847	Potri.001G218900.1	
Potra2n1c1899.1	NA	1.394613675	NA	NA		
Potra2n1c19.1	0.518358142	0.777188124	0.165795145	0.624854364	Potri.001G002000.1	AT3G13460.1
Potra2n1c190.1	-1.864134742	-1.292229428	NA	NA	Potri.001G022800.1	AT3G14460.1
Potra2n1c1900.1					Potri.001G189600.1	
Potra2n1c1901.1	2.474316084	0.747755582	0.01950413	0.711204954	Potri.001G219100.1	AT5G04760.1
Potra2n1c1902.1	0.221027134	0.393671244	0.22152159	0.766240139	Potri.001G219200.1	AT5G22360.1
Potra2n1c1903.1	2.857394777	0.804297389	2.54163E-11	0.741833788	Potri.001G219300.1	AT5G21105.1
Potra2n1c1904.1	0.001697346	0.063710748	0.992844277	0.941855873	Potri.001G219400.2	AT2G25570.3
Potra2n1c1905.1	-0.058148192	0.070254349	0.518511753	0.935851032	Potri.001G219500.2	AT5G21090.1
Potra2n1c1906.1	-0.285223114	0.146135196	0.670337528	0.95671968		

Potra2n1c1907.2	0.147574632	0.463765102	0.141011282	0.3699319	Potri.001G219700.2	AT2G27040.2
Potra2n1c1908.1	1.808164029	2.159659662	0.234184249	0.542400894	Potri.001G219800.1	AT2G27035.1
Potra2n1c1909.1	NA	1.381953902	NA	NA	Potri.001G219900.1	AT2G27035.1
Potra2n1c191.1	0.82357019	2.676364087	NA	NA	Potri.001G022800.1	AT3G14460.1
Potra2n1c1910.1	NA	0.674963689	NA	NA		
Potra2n1c1911.1	0.25382741	0.096798368	0.085800493	0.936549944	Potri.001G220100.1	AT5G21160.3
Potra2n1c1912.1	0.397604806	0.246228928	0.015460844	0.68015285		
Potra2n1c1913.1	-0.178136036	-0.041453733	0.462782218	0.977227089	Potri.001G220300.1	AT3G59040.1
Potra2n1c1914.1	0.091869602	-1.461434148	0.959104737	NA	Potri.001G220400.1	
Potra2n1c1915.1	NA	2.676364087	NA	NA	Potri.001G220500.3	AT1G77450.1
Potra2n1c1916.1	0.26984807	0.000283311	0.058593115	0.999937671	Potri.001G220600.1	AT2G41060.2
Potra2n1c1917.1	-0.296641253	0.161722765	0.026032869	0.893240619	Potri.001G220700.2	AT3G56850.1
Potra2n1c1918.1	0.286219724	0.75231896	0.506520431	NA	Potri.001G220800.1	AT5G21170.1
Potra2n1c1919.1	0.668191313	-0.12746277	0.147824451	0.974275337		
Potra2n1c192.1	0.515609968	-0.758679239	0.387141442	0.774693915	Potri.001G022900.1	AT3G51930.1
Potra2n1c1920.1	0.346459487	0.056987628	0.001841251	0.940376196	Potri.001G221000.1	AT2G26060.1
Potra2n1c1921.1	0.186242356	0.110081123	NA	NA		
Potra2n1c1922.1	1.708775008	1.166773781	0.72215261	NA	Potri.007G089300.1	AT2G01050.1
Potra2n1c1923.1	NA	NA	NA	NA		
Potra2n1c1924.1						
Potra2n1c1925.1	1.545832463	1.363811008	NA	NA		
Potra2n1c1926.1					Potri.001G224000.2	AT3G60120.1
Potra2n1c1927.1	0.121854237	0.057067163	0.320749862	0.949966783	Potri.001G224100.1	AT2G27020.1
Potra2n1c1928.1	1.664677056	NA	NA	NA	Potri.001G224200.1	AT3G07870.1
Potra2n1c1929.1	0.82357019	NA	NA	NA	Potri.001G224300.1	AT3G06240.1
Potra2n1c193.1	0.253094448	0.139542617	0.198328902	0.879221016	Potri.001G023000.1	AT3G04040.1
Potra2n1c1930.1	1.64774763	NA	NA	NA		
Potra2n1c1931.1	1.127510973	0.313300704	1.2396E-08	0.771626158		
Potra2n1c1932.1	-0.187314403	0.147960335	0.392770654	0.936402128	Potri.001G224500.2	AT3G11440.1
Potra2n1c1933.1	0.643217014	0.288466581	2.68084E-06	0.724604666	Potri.001G224700.1	AT5G12110.1
Potra2n1c1934.1	0.02216492	0.862883113	0.978751757	0.645351835	Potri.001G224800.1	AT5G56790.1
Potra2n1c1935.1	-0.917424379	0.017887711	0.001319374	0.995579852	Potri.001G224950.1	AT5G19500.1

Potra2n1c1936.1	0.35218923	-0.006719893	0.080307073	0.996154416	Potri.001G225100.2	AT2G26930.1
Potra2n1c1937.2	-1.010615451	0.004333191	0.021418894	0.999458638	Potri.001G224950.1	AT5G19500.1
Potra2n1c1938.1	-0.043668147	-0.114361456	0.776537996	0.865611082	Potri.001G225200.1	AT2G26920.1
Potra2n1c1939.1	-0.164293492	0.045956048	0.359127556	0.961820855	Potri.001G225300.1	AT5G37290.1
Potra2n1c194.1	0.281962394	0.183371863	0.301522096	0.863768668		
Potra2n1c1940.1	0.346139634	0.185672346	0.393753282	0.926235628	Potri.001G225400.1	AT3G12480.1
Potra2n1c1941.1	-0.120121244	-0.16254976	0.455073955	0.865611082	Potri.001G225500.1	AT5G42850.2
Potra2n1c1942.2	1.082931971	-0.108295836	0.126904261	0.979719961	Potri.001G225600.2	AT3G59550.1
Potra2n1c1943.1	-0.277078529	0.85753757	0.670157663	0.677274011	Potri.001G225700.1	AT2G01290.1
Potra2n1c1944.1	7.865087266	-2.348219758	7.10985E-37	NA	Potri.001G225800.1	AT3G12490.2
Potra2n1c1945.1						
Potra2n1c1946.1	1.459962178	3.010689165	NA	NA	Potri.T055866.1	
Potra2n1c1947.1	2.658032118	NA	NA	NA		
Potra2n1c1948.1	NA	NA	NA	NA	Potri.001G225900.1	AT3G60130.1
Potra2n1c1949.1						
Potra2n1c195.1	-0.259976033	0.015181568	0.14887624	0.994220827	Potri.001G023100.1	AT2G41430.1
Potra2n1c1950.1	3.421838676	0.837905211	0.040357152	NA	Potri.001G226500.1	
Potra2n1c1951.2					Potri.001G226000.1	AT5G24550.1
Potra2n1c1952.1					Potri.006G019900.1	
Potra2n1c1953.1	-0.085460216	0.052204358	0.482743283	0.960981446	Potri.001G222700.1	AT2G26990.1
Potra2n1c1954.3	-0.142178147	0.152511133	0.686752681	0.902857376	Potri.001G222600.14	AT2G26980.4
Potra2n1c1955.1	-0.276715133	-0.024409843	0.007829186	0.974275337	Potri.001G222500.1	AT5G53530.1
Potra2n1c1956.2	0.427318178	-0.037619955	0.014199329	0.98534079	Potri.001G222400.1	AT4G15010.3
Potra2n1c1957.1	-0.050550192	0.161397293	0.689138288	0.807968523	Potri.001G222300.1	AT2G26970.1
Potra2n1c1958.1	-0.380811029	-0.205600438	1.21626E-05	0.663037599	Potri.001G222200.2	AT2G27030.3
Potra2n1c1959.1	2.685015931	NA	NA	NA	Potri.001G227200.1	AT2G44480.1
Potra2n1c196.1	NA	NA	NA	NA	Potri.001G023200.1	AT4G30880.1
Potra2n1c1960.1	0.943350674	-0.767708831	NA	NA	Potri.001G227300.1	AT2G44480.1
Potra2n1c1961.1	-0.937611701	-2.446267299	NA	NA	Potri.001G227300.1	AT2G44480.1
Potra2n1c1962.1	0.750097292	0.187510503	0.020597483	0.943819961	Potri.001G227700.1	AT1G32400.3
Potra2n1c1963.1	0.393064703	0.394472204	0.488124683	0.862335622	Potri.001G227800.1	
Potra2n1c1964.1	-0.040949408	-0.269589546	0.785484582	0.859892315	Potri.001G228000.1	AT1G04945.2

Potra2n1c1965.1	-0.734348855	-0.507461092	3.46347E-07	0.590905253	Potri.001G228100.1	AT2G32850.2
Potra2n1c1966.1	4.801135412	2.86450839	0.008711491	NA	Potri.001G228200.1	AT5G60900.1
Potra2n1c1967.1	-0.270310671	0.109770463	0.287405396	0.948577865	Potri.001G228200.1	AT5G60900.1
Potra2n1c1968.1	-0.747209412	-0.499593352	1.21505E-08	0.548741057	Potri.001G228100.1	AT2G32850.2
Potra2n1c1969.1	2.961676152	NA	NA	NA	Potri.001G228200.1	AT5G60900.1
Potra2n1c197.1					Potri.001G023300.2	AT4G30880.1
Potra2n1c1970.1	NA	NA	NA	NA	Potri.001G228300.2	AT5G60900.1
Potra2n1c1971.1	0.302016424	0.839278886	0.796540559	0.842247476	Potri.001G228300.2	AT5G60900.1
Potra2n1c1972.1	-0.038518683	-0.678426319	0.97499749	0.912777843	Potri.001G228300.2	AT5G60900.1
Potra2n1c1973.1	NA	NA	NA	NA	Potri.001G228300.2	AT5G60900.1
Potra2n1c1974.1	0.413625689	-0.855710623	0.697549587	0.869186333	Potri.001G228300.2	AT5G60900.1
Potra2n1c1975.1					Potri.001G228500.2	AT5G06800.1
Potra2n1c1976.1					Potri.001G228500.2	AT5G06800.1
Potra2n1c1977.2	-0.328798934	0.074361064	0.035550328	0.924951347	Potri.001G229000.1	AT5G13020.1
Potra2n1c1978.1	-0.085964619	0.222556745	0.710447347	0.812677788	Potri.001G229100.1	AT5G67030.1
Potra2n1c1979.1	-0.601119587	0.240439567	0.001816801	0.911749053	Potri.001G229300.1	AT2G44430.1
Potra2n1c198.1	-1.934126624	-2.0827378	NA	NA	Potri.001G023400.1	AT5G50400.1
Potra2n1c1980.1					Potri.001G229400.1	
Potra2n1c1981.1	5.333510078	2.316585279	3.84738E-16	0.534852254	Potri.001G229500.1	AT2G27660.1
Potra2n1c1982.1	-0.262424149	-0.059186275	0.050471358	0.966407607	Potri.001G229600.1	AT2G44410.1
Potra2n1c1983.1	-0.774208231	-2.317513346	0.552383199	NA		
Potra2n1c1984.1	0.202751117	-1.341934515	0.942058875	NA		
Potra2n1c1985.1	-0.186558022	0.174566443	0.478113666	0.926808461	Potri.001G229700.1	AT5G06710.1
Potra2n1c1986.1	0.050549633	-0.265781404	0.765890871	0.623736221	Potri.001G229800.1	AT2G32840.1
Potra2n1c1987.1	2.397417972	1.09960016	0.052505789	NA	Potri.001G229900.1	AT2G28270.1
Potra2n1c1988.1	0.349521387	0.316784958	0.007591561	0.387987556	Potri.001G230000.1	AT1G05570.1
Potra2n1c1989.1	0.09533361	0.00864565	0.582972775	0.994236852	Potri.001G230100.1	AT2G44360.1
Potra2n1c199.1	-0.260850866	0.087199043	0.049094148	0.93017274	Potri.001G024100.1	AT2G38800.1
Potra2n1c1990.2	-0.158576938	0.188017858	0.593246593	0.905274942	Potri.001G230200.1	
Potra2n1c1991.1	-0.651615025	-0.261308729	0.269997045	0.937846405	Potri.001G230300.1	
Potra2n1c1992.1	-0.836306781	-2.523469902	NA	NA	Potri.001G230400.1	
Potra2n1c1993.1	1.127986619	0.31217162	5.07522E-09	0.743612955	Potri.001G230500.2	AT2G44350.2

Potra2n1c1994.1	-1.933486224	-0.201487512	5.60253E-05	0.926106039	Potri.001G230900.1	AT2G31945.1
Potra2n1c1995.1	-0.919704797	-0.016014482	0.000770248	0.995581218	Potri.006G112100.1	AT5G01980.1
Potra2n1c1996.1	-0.967159512	0.003541531	0.001684766	0.999325699		
Potra2n1c1997.1	0.171688446	-0.055864677	0.461804318	0.974275337	Potri.001G231100.1	AT2G44310.1
Potra2n1c1998.1	0.030946701	-0.868352943	0.977071219	0.812118278	Potri.001G231200.1	AT4G25835.1
Potra2n1c1999.1	-0.17421854	-0.916430803	0.865220902	0.824905557		
Potra2n1c2.1					Potri.014G151100.1	
Potra2n1c20.1	-1.051562043	-0.099552886	3.42317E-09	0.953831244	Potri.001G002300.10	AT5G56460.1
Potra2n1c200.1	-0.562947644	0.096726093	1.5966E-05	0.892986206	Potri.001G024200.1	AT2G38780.1
Potra2n1c2000.1	-0.040459913	0.998003196	0.988066205	NA	Potri.001G231300.1	AT2G31940.1
Potra2n1c2001.1	-0.664566686	-0.480324417	8.80224E-05	0.373510649	Potri.001G231400.3	AT2G44290.1
Potra2n1c2002.1	0.221821605	0.023647713	0.1828863	0.987226805	Potri.013G129600.1	AT5G47320.1
Potra2n1c2003.1	-0.783902335	-0.5448516	0.006107585	0.679125998	Potri.001G231500.1	AT2G44280.1
Potra2n1c2004.1	-2.102156303	-0.743946682	1.81694E-11	0.140770927	Potri.001G231600.1	AT2G31930.1
Potra2n1c2005.1	1.459962178	1.381953902	NA	NA	Potri.001G231700.2	AT1G05577.1
Potra2n1c2006.1	-0.282901868	-0.022161837	0.0244568	0.978485478	Potri.001G231800.1	AT5G23880.1
Potra2n1c2007.1	-1.676999892	-0.352539804	1.05485E-07	0.718629174	Potri.001G231900.2	AT2G44260.1
Potra2n1c2008.1	0.249814302	0.021587447	0.127072548	0.985313207	Potri.001G232600.1	AT1G05590.1
Potra2n1c2009.1	-2.299005217	-1.700685647	8.86306E-06	0.284581679	Potri.001G232900.1	AT2G18370.1
Potra2n1c201.1	0.412045634	-0.009364521	0.016527449	0.996061609	Potri.001G024500.1	AT1G63780.1
Potra2n1c2010.1	-1.121896946	-0.250727845	1.08997E-10	0.53266226	Potri.001G233000.1	AT3G13510.1
Potra2n1c2011.1	0.01258614	0.036595643	0.97078897	0.985016122	Potri.001G233100.1	AT2G31900.1
Potra2n1c2012.1						
Potra2n1c2013.1						
Potra2n1c2014.1	-0.109223714	-0.008068171	0.445253656	0.995409858	Potri.001G233200.1	AT3G45880.1
Potra2n1c2015.1	-0.012395009	0.157028452	0.940160916	0.862830524	Potri.001G233300.1	AT3G45770.1
Potra2n1c2016.1	0.194848342	0.189380693	0.19028471	0.775343511	Potri.001G233400.3	AT1G07670.1
Potra2n1c2017.1	-4.180599859	-0.314866507	1.80574E-06	0.936170918	Potri.001G233600.3	AT5G59990.1
Potra2n1c2018.2	-0.600319952	-0.300098577	0.006951284	0.817452127	Potri.001G233700.2	AT3G45850.2
Potra2n1c2019.1	0.167089422	0.214177183	0.130957299	0.804641567	Potri.001G233800.1	AT3G45830.1
Potra2n1c202.1	1.231699115	1.12123936	0.252469942	0.814981587	Potri.001G024600.1	AT1G76390.2
Potra2n1c2020.1	-0.448343959	-0.063066349	0.000275317	0.945227748	Potri.001G233900.1	AT5G60050.1

Potra2n1c2021.1	2.251628932	-0.767708831	NA	NA	Potri.001G233950.1	AT1G60800.1
Potra2n1c2022.1	NA	NA	NA	NA	Potri.009G026300.1	AT1G07850.1
Potra2n1c2023.1	0.191160691	-0.00031763	0.283776193	0.999937671	Potri.001G234100.1	AT1G07870.2
Potra2n1c2024.1	-0.854919298	-0.265845349	2.46339E-07	0.803948068	Potri.001G234200.1	AT3G46290.1
Potra2n1c2025.1	-0.447789923	-0.071100343	0.190354307	0.9761362	Potri.001G234200.1	AT3G46290.1
Potra2n1c2026.1	0.043248766	0.106610318	0.760162099	0.862830524	Potri.001G234300.4	AT3G46220.1
Potra2n1c2027.1	-0.637502356	1.332494942	0.661398986	0.82876584	Potri.001G234500.1	AT2G28630.1
Potra2n1c2028.1	3.243472491	1.383515824	7.92768E-09	0.607885566	Potri.001G234600.1	AT2G39380.1
Potra2n1c2029.1	2.666517184	0.245622993	7.84272E-05	0.961372512	Potri.001G234700.1	AT5G02600.2
Potra2n1c203.1	4.288691151	1.782974462	9.58854E-10	0.788065047	Potri.001G024800.1	AT2G38760.1
Potra2n1c2030.1	0.385446331	0.221388136	0.011917197	0.71248439	Potri.001G234800.1	AT5G59740.1
Potra2n1c2031.1	0.786437886	1.781190399	0.739230411	NA	Potri.001G234900.1	AT5G59750.1
Potra2n1c2032.1	-0.080626234	-0.252408836	0.837225663	0.875559093		
Potra2n1c2033.1	2.124657703	-0.898821406	0.269839529	NA	Potri.001G235200.1	AT2G28690.1
Potra2n1c2034.1	-1.707162982	0.0379316	3.41997E-05	0.994403137	Potri.001G235300.1	AT4G01470.1
Potra2n1c2035.1	0.26352615	-0.036235342	0.066493682	0.978203092	Potri.001G235400.1	AT5G59770.1
Potra2n1c2036.2	-1.258102695	0.309589643	0.000106354	0.817452127	Potri.001G235500.1	AT3G46130.1
Potra2n1c2037.1	1.025577003	0.778222439	1.24081E-05	0.138040185	Potri.001G235700.1	AT5G59790.1
Potra2n1c2038.1	2.501613031	1.565418428	0.000123351	0.333044935	Potri.001G235800.1	AT2G37430.1
Potra2n1c2039.1	0.82357019	NA	NA	NA	Potri.001G344400.1	AT5G40510.1
Potra2n1c204.1	5.007546781	-0.767708831	0.001077249	NA	Potri.001G024900.1	AT2G38750.1
Potra2n1c2040.1	NA	NA	NA	NA	Potri.008G220300.1	
Potra2n1c2041.1	0.075778453	0.162856221	0.51585112	0.601577719	Potri.001G236000.1	AT5G59830.2
Potra2n1c2042.1	-0.887554117	-0.348192016	1.25277E-07	0.717960391	Potri.001G236100.1	AT5G59840.1
Potra2n1c2043.1	1.664677056	NA	NA	NA		
Potra2n1c2044.2	-0.045207346	-0.111298734	0.892558039	0.948577865	Potri.001G236200.1	AT3G46020.1
Potra2n1c2045.1	-0.185818957	-0.06340607	0.649968243	0.980890614	Potri.001G236300.1	AT3G23830.2
Potra2n1c2046.2	-0.124058882	-0.185379405	0.520340787	0.851986664	Potri.001G236400.2	AT3G46010.1
Potra2n1c2047.1	-0.044600652	-0.145988114	0.713254144	0.824351791	Potri.001G236500.1	AT5G59890.1
Potra2n1c2048.1	-0.071419127	0.167579033	0.699368447	0.804796567	Potri.001G236800.1	AT5G59900.1
Potra2n1c2049.1	-0.039340244	0.109264286	0.834186723	0.850010611	Potri.001G236900.1	AT5G59950.5
Potra2n1c205.1	-0.631179097	0.009788054	9.51341E-08	0.996032145	Potri.001G025100.12	AT3G54500.1

Potra2n1c2050.1	0.081483319	0.080991669	0.535593521	0.921065381		
Potra2n1c2051.1	-0.081259447	-0.02241428	0.457334239	0.98177802	Potri.001G237000.2	AT5G59960.1
Potra2n1c2052.1	-0.28609969	0.182136362	0.058647619	0.899212956	Potri.001G237100.1	AT2G39570.1
Potra2n1c2053.1	-0.510983819	-0.345444078	0.008602301	0.814981587	Potri.001G237300.1	AT2G28780.1
Potra2n1c2054.1	0.125159612	0.182486148	0.387853878	0.661611864	Potri.001G237400.1	AT3G46450.1
Potra2n1c2055.1					Potri.001G237500.1	AT1G07645.1
Potra2n1c2056.1	-0.686114606	-1.03992478	0.380613546	0.695756198	Potri.001G237600.1	AT2G28790.1
Potra2n1c2057.1	-1.286356226	-0.486913865	1.31747E-09	0.235153493	Potri.001G237800.1	AT2G28800.1
Potra2n1c2058.1	-1.572293341	-0.48267602	3.65159E-08	0.513411699	Potri.001G237700.1	AT5G59305.1
Potra2n1c2059.1	NA	NA	NA	NA	Potri.001G237900.1	AT5G59340.1
Potra2n1c206.1	1.675416526	0.251292179	0.212862417	0.97064682	Potri.001G109300.1	AT1G33720.1
Potra2n1c2060.2	-1.024621714	-0.296850286	5.12754E-08	0.701494362	Potri.001G238300.1	AT5G59350.1
Potra2n1c2061.1	-0.61910233	-0.767708831	NA	NA		
Potra2n1c2062.1	-0.459137926	-0.955807784	0.662596602	0.756757704	Potri.001G238400.1	AT3G55370.1
Potra2n1c2063.1	NA	NA	NA	NA		
Potra2n1c2064.1	0.474061856	0.120443893	0.000113725	0.849446235	Potri.001G238500.1	AT3G46510.1
Potra2n1c2065.1	0.370267857	-2.578469326	0.846691383	NA	Potri.001G238700.1	AT1G53540.1
Potra2n1c2066.1	-0.44074426	0.345926919	0.040188889	0.842247476	Potri.001G238800.1	AT2G28840.1
Potra2n1c2067.1	0.6593771	-0.134640277	0.016040399	0.95671968	Potri.001G238900.1	AT5G02440.1
Potra2n1c2068.1	-1.385950247	-1.377093514	0.003167635	0.371384156	Potri.001G239000.1	AT5G02420.1
Potra2n1c2069.1	1.522999228	0.511108345	1.14628E-13	0.50924822	Potri.001G239100.2	AT2G45910.1
Potra2n1c207.1	1.093272551	0.153998766	2.24043E-10	0.878118098	Potri.001G025400.1	AT3G14470.1
Potra2n1c2070.1	1.687356717	0.5656794	5.71771E-13	0.549719769	Potri.001G239200.1	AT3G46540.1
Potra2n1c2071.1	0.119683713	0.157670377	0.506199591	0.842247476	Potri.001G239300.1	AT2G28890.1
Potra2n1c2072.1	-0.520974293	-0.611972768	0.441199034	0.791002527	Potri.001G239500.1	AT5G02390.1
Potra2n1c2073.2	-0.137791578	-0.092180264	0.62841279	0.953831244	Potri.001G239600.1	AT1G07615.1
Potra2n1c2074.1	-2.880997526	0.030623078	NA	NA		
Potra2n1c2075.1	0.817698871	0.069534136	0.052027834	0.966228173	Potri.001G239900.1	AT2G28900.1
Potra2n1c2076.1	0.043943434	-0.450043	0.882608295	0.830470159	Potri.001G240000.1	AT5G24318.1
Potra2n1c2077.1	0.24456223	-0.003274259	0.29702673	0.998307362	Potri.001G240100.1	AT3G46580.1
Potra2n1c2078.1	0.114887748	0.06416396	0.412571583	0.936890993	Potri.001G240200.1	AT3G22480.2
Potra2n1c2079.1	2.880122302	2.879652232	0.000976024	0.416170423	Potri.001G409700.1	ATCG00770.1

Potra2n1c208.1	0.955107615	0.243621248	0.161174396	0.956865545	Potri.001G025500.1	AT3G54490.1
Potra2n1c2080.1	3.107804038	2.938256283	8.9259E-08	0.360229665	Potri.013G136730.1	ATCG00750.1
Potra2n1c2081.1	-0.473656555	-1.258122047	0.429437469	0.517743314	Potri.001G240300.1	AT3G55440.1
Potra2n1c2082.1	-0.05857751	0.197973631	0.79369432	0.790362046	Potri.001G240400.4	AT1G07570.1
Potra2n1c2083.1	0.312819398	-0.141120768	0.117961991	0.842247476	Potri.001G240500.1	AT2G28940.2
Potra2n1c2084.1	NA	NA	NA	NA	Potri.001G240600.1	AT1G03230.1
Potra2n1c2085.1	NA	NA	NA	NA	Potri.001G240600.1	AT1G03230.1
Potra2n1c2086.4	-1.012290687	0.155124674	0.116410621	0.960656795	Potri.001G240666.1	AT1G13790.1
Potra2n1c2087.1	-0.623770684	-0.599978242	0.001741331	0.386834221	Potri.001G240900.1	AT2G39700.1
Potra2n1c2088.1						
Potra2n1c2089.2	-1.115241841	-0.196577799	5.18618E-05	0.910477984	Potri.001G241300.1	AT2G29050.1
Potra2n1c209.1	-0.208263144	-0.000814416	0.053805758	0.999458638	Potri.T153900.1	AT3G54480.1
Potra2n1c2090.1	-0.90487161	-0.959842299	0.540481294	0.881526761	Potri.003G000900.2	AT5G49880.1
Potra2n1c2091.1	-0.486451462	-0.013841397	0.02377427	0.991407187	Potri.001G241400.1	AT5G59400.1
Potra2n1c2092.2	0.182502003	-0.007637197	0.203304772	0.995005375	Potri.001G241500.1	AT5G59410.1
Potra2n1c2093.2	-0.322726231	-0.20220669	0.00024415	0.764333974	Potri.001G241600.1	AT1G07540.1
Potra2n1c2094.1	-0.475827606	0.017075894	0.011806188	0.986190118	Potri.001G241700.1	AT1G07530.1
Potra2n1c2095.1	0.760001902	0.510976579	1.00969E-11	0.088654706	Potri.001G241800.1	AT1G07530.1
Potra2n1c2096.2	-1.792518177	-3.486535826	0.701189843	NA	Potri.001G241900.1	AT1G07530.1
Potra2n1c2097.1	2.214110689	0.474144114	1.6715E-08	0.860001323	Potri.001G242000.1	AT1G07530.1
Potra2n1c2098.1	2.110605521	0.506226171	3.02964E-07	0.849749156	Potri.001G242100.1	AT1G07530.1
Potra2n1c2099.1	0.692962158	0.232406944	2.89531E-07	0.600720177	Potri.001G242200.1	AT2G29070.2
Potra2n1c21.1	-1.11547823	-0.124571098	9.81108E-09	0.943819961	Potri.001G002200.2	AT4G26330.1
Potra2n1c210.1	9.273072219	5.647363339	3.68175E-16	0.06359123	Potri.T154000.1	AT5G20710.1
Potra2n1c2100.1	-0.564661254	-0.193674803	3.26517E-08	0.925035363	Potri.001G242300.2	AT5G59480.1
Potra2n1c2101.1	-0.184194793	-0.186361703	0.419853037	0.895474078	Potri.001G242400.1	AT5G59500.1
Potra2n1c2102.1	3.694185431	1.926635273	8.31203E-05	0.720242695	Potri.001G242600.5	AT2G29090.1
Potra2n1c2103.1	-2.221978699	-1.774857434	0.120953387	NA	Potri.001G242700.1	
Potra2n1c2104.1	0.976829728	0.680914762	0.152444561	0.853030738	Potri.001G242900.2	AT1G03910.2
Potra2n1c2105.1	NA	2.32414027	NA	NA	Potri.001G243000.1	AT3G27320.1
Potra2n1c2106.1	0.642176954	0.178244282	0.164945843	0.920028955		
Potra2n1c2107.2	0.642176954	0.178244282	0.164945843	0.920028955	Potri.001G243100.1	AT1G76440.3

Potra2n1c2108.1	7.539327953	3.495980907	5.65945E-09	NA	Potri.009G034500.1	AT2G29130.1
Potra2n1c2109.1	0.271491592	-0.128208792	0.133762446	0.890369631	Potri.001G243300.1	AT3G46620.1
Potra2n1c211.1	-1.35927387	0.110081123	NA	NA	Potri.001G025800.1	AT5G20710.1
Potra2n1c2110.1	0.216184893	0.040993415	0.04619187	0.961703915	Potri.001G243400.1	AT2G29190.1
Potra2n1c2111.1	0.14050917	-0.020913059	0.219424021	0.987288338	Potri.001G243500.1	AT2G29180.1
Potra2n1c2112.1	-0.417015902	-0.261878424	0.00416348	0.867910364	Potri.001G243600.1	AT3G46640.1
Potra2n1c2113.1	-0.427340228	-0.273938524	0.095467952	0.788065047	Potri.001G243800.1	AT5G59600.1
Potra2n1c2114.1	NA	NA	NA	NA		
Potra2n1c2115.1						
Potra2n1c2116.1	-0.065205832	0.021432643	0.630952309	0.985393296	Potri.001G244100.7	AT5G59250.1
Potra2n1c2117.1	-0.04613139	0.057638617	0.631369408	0.935099709	Potri.009G036700.1	AT1G07480.2
Potra2n1c2118.3	-0.085333846	0.059897804	0.550305628	0.954098985	Potri.001G244600.1	AT2G29210.1
Potra2n1c2119.1	0.216399732	-0.212175343	0.485979447	0.833651746	Potri.001G244800.1	AT3G46780.1
Potra2n1c212.1	0.053854315	-0.066828964	0.950842821	0.990389888		
Potra2n1c2120.1	-0.074632473	-0.154623639	0.797470267	0.936549944	Potri.001G244900.1	AT2G29260.1
Potra2n1c2121.1	2.043050195	0.69054994	2.77919E-31	0.371384156	Potri.001G245000.1	AT1G07440.1
Potra2n1c2122.1	NA	1.571529889	NA	NA	Potri.001G245100.1	
Potra2n1c2123.1	0.708338785	-0.075469832	0.001780678	0.956622854	Potri.001G245200.1	AT1G07430.1
Potra2n1c2124.1	-0.762200103	-0.084727211	0.019483881	0.974275337	Potri.001G245300.1	AT1G07420.1
Potra2n1c2125.1	-1.916791236	-0.70890975	2.22876E-19	0.294386607	Potri.001G245400.1	
Potra2n1c2126.2	-0.277762881	-0.036785886	0.006467441	0.974275337	Potri.001G245500.1	AT5G59210.1
Potra2n1c2127.1	1.545832463	4.122709331	NA	NA	Potri.009G037600.1	AT3G46790.1
Potra2n1c2128.1	-0.179558084	0.06890021	0.03494645	0.890978957	Potri.001G245700.1	AT2G29400.1
Potra2n1c2129.1	0.212113407	0.11429926	0.340485545	0.921099436	Potri.001G245800.1	AT2G29410.1
Potra2n1c213.1	-0.206065023	-0.012324802	0.179617074	0.995005375	Potri.003G198800.1	AT3G14180.1
Potra2n1c2130.1	0.334573449	0.151222144	0.285080805	0.932683676	Potri.001G245900.1	AT5G59590.1
Potra2n1c2131.1	3.645912453	1.273966461	3.36458E-09	0.725595998	Potri.001G246000.1	AT5G59030.1
Potra2n1c2132.1	-0.000985816	0.324891043	0.994385722	0.583548322	Potri.001G246100.1	AT3G09360.1
Potra2n1c2133.1	-1.188127927	-0.256422376	3.63352E-11	0.713696872	Potri.001G246200.1	AT5G59020.1
Potra2n1c2134.1	-0.732429751	-0.004510257	0.072564258	0.99905083	Potri.001G246300.1	AT3G54020.1
Potra2n1c2135.1	-1.270577311	-0.504750211	5.21344E-14	0.156572927	Potri.001G246200.1	AT5G59020.1
Potra2n1c2136.1	-0.570222817	0.011107682	0.090388062	0.996032145	Potri.001G246300.1	AT3G54020.1

Potra2n1c2137.2	-1.232645553	-0.340243765	2.03191E-16	0.386834221	Potri.001G246300.1	AT3G54020.1
Potra2n1c2138.4	0.052865604	0.101130744	0.88592903	0.910815461	Potri.001G246400.1	AT5G59010.1
Potra2n1c2139.2	-0.004023843	-0.000706908	0.980871647	0.999536469	Potri.001G246700.1	AT5G05830.1
Potra2n1c214.1	0.112580417	0.317108171	0.210811596	0.60926347	Potri.001G026100.1	AT3G07300.2
Potra2n1c2140.1	-1.180961667	-2.951091287	0.461577759	NA	Potri.009G039900.1	AT3G54040.1
Potra2n1c2141.1	0.106899818	-0.125998093	0.518897539	0.863324416	Potri.001G246800.1	AT5G52370.1
Potra2n1c2142.1	0.263354604	-0.151426195	0.173456301	0.923348116	Potri.001G246900.1	AT5G62690.1
Potra2n1c2143.1	-0.391367586	0.118346128	0.014828434	0.902579655	Potri.001G247100.1	
Potra2n1c2144.1	NA	NA	NA	NA		
Potra2n1c2145.2	-0.566736831	0.826363809	0.033337954	0.151351582	Potri.001G247200.1	AT5G01120.1
Potra2n1c2146.1	-1.070838001	0.900128593	0.028046503	0.650005812	Potri.001G247200.1	AT5G01120.1
Potra2n1c2147.1	NA	NA	NA	NA		
Potra2n1c2148.1	-0.619084132	0.818801705	0.249384494	0.660456866	Potri.001G247200.1	AT5G01120.1
Potra2n1c2149.1	0.223065399	0.187010774	0.110581732	0.643558659	Potri.001G247400.1	AT1G07380.1
Potra2n1c215.1	0.533797239	-0.006361555	0.00075717	0.996307365	Potri.001G026200.1	AT3G61790.1
Potra2n1c2150.1	0.188479105	1.364364794	0.45280097	0.139318601	Potri.001G247600.1	AT3G11320.1
Potra2n1c2151.1	0.139330601	0.824079646	0.460937477	0.305102088	Potri.001G247700.1	AT2G29570.1
Potra2n1c2152.1	-0.389901579	-0.020581586	0.032228019	0.98876314	Potri.001G247800.1	AT5G58970.1
Potra2n1c2153.1	NA	NA	NA	NA	Potri.009G041000.1	AT5G58960.1
Potra2n1c2154.1	-0.173096989	0.220002009	0.36443289	0.872366576	Potri.001G248100.1	AT1G07350.1
Potra2n1c2155.2	0.082611936	0.083444799	0.522975186	0.912044974	Potri.001G248200.1	AT3G46980.3
Potra2n1c2156.1	0.006358734	0.022708299	0.998166972	NA		
Potra2n1c2157.1	-0.630748765	-0.549255529	0.000573011	0.428801064	Potri.001G248400.1	AT5G58930.1
Potra2n1c2158.4	-0.775348056	-0.616909395	0.170904238	0.824905557	Potri.001G248600.1	AT3G54140.1
Potra2n1c2159.1	1.461270025	-0.007775729	0.000704612	0.999315506	Potri.001G248700.1	AT2G38080.1
Potra2n1c216.1	0.265227117	-0.059435518	0.050441143	0.956865545	Potri.001G026300.1	AT3G54470.1
Potra2n1c2160.1	NA	NA	NA	NA	Potri.004G117600.1	
Potra2n1c2161.1	-2.241240059	-0.749181942	7.45409E-15	0.001940588	Potri.001G248800.1	AT2G38090.1
Potra2n1c2162.1	-2.758413533	-0.09242364	0.282658403	NA		
Potra2n1c2163.1	-1.409983009	-0.834601298	NA	NA		
Potra2n1c2164.1	1.545832463	2.490806657	NA	NA	Potri.001G248900.1	AT5G26250.1
Potra2n1c2165.1	NA	2.134902029	NA	NA	Potri.001G249000.1	AT5G58880.1

Potra2n1c2166.1	-0.181829271	0.060195691	0.174623698	0.95652089	Potri.001G249100.1	AT3G47060.1
Potra2n1c2167.1	-0.406459096	-0.315215078	0.176774792	0.779135176	Potri.001G249200.1	AT2G29630.2
Potra2n1c2168.1	0.174460841	0.110223433	0.426336626	0.910815461	Potri.001G249400.2	AT2G29640.1
Potra2n1c2169.1	-1.927968462	-1.080839386	0.593296721	NA	Potri.001G249500.1	AT1G07300.1
Potra2n1c217.1	-0.891416436	-0.588753115	0.010740573	0.427906987	Potri.001G026400.1	AT3G07940.1
Potra2n1c2170.1	-1.221739768	-0.440868478	1.35625E-06	0.714411171	Potri.001G249600.1	AT3G47070.1
Potra2n1c2171.1	-0.856460408	-0.428391184	0.000164794	0.775519829	Potri.001G249800.1	AT2G29650.1
Potra2n1c2172.1	2.111680128	-0.511467705	1.22629E-05	0.920028955	Potri.001G249700.1	AT5G58860.1
Potra2n1c2173.1	0.357163417	0.121043434	0.190532395	0.953108412	Potri.001G249900.1	AT2G29660.1
Potra2n1c2174.1	1.459962178	1.363811008	NA	NA	Potri.001G250000.1	AT5G58850.1
Potra2n1c2175.1	-0.388882366	-0.432879173	0.098837283	0.794521408	Potri.001G250100.4	AT2G29670.1
Potra2n1c2176.3	-0.072497384	-0.373976957	0.91454696	0.888248505	Potri.001G250200.1	AT1G07270.1
Potra2n1c2177.2	-0.30821549	0.162827412	0.046771597	0.824905557	Potri.001G250400.1	AT2G29700.1
Potra2n1c2178.1	-1.081335305	-0.231479753	2.99182E-06	0.903769998	Potri.001G250500.1	AT1G07230.1
Potra2n1c2179.3	0.491661596	0.096886512	0.01874461	0.956865545	Potri.001G250700.1	AT3G18600.1
Potra2n1c218.1	-1.04109041	-0.514635213	0.000699933	0.716548554	Potri.001G026500.1	AT2G11890.1
Potra2n1c2180.3	-0.568790903	-0.159170802	0.057880732	0.917751882	Potri.001G250800.1	AT3G47160.1
Potra2n1c2181.1	2.658032118	NA	NA	NA	Potri.001G250900.1	AT5G58770.1
Potra2n1c2182.1	1.931605898	NA	NA	NA	Potri.001G250900.1	AT5G58770.1
Potra2n1c2183.1	-0.020715424	0.0233181	0.875834813	0.978203092	Potri.001G251000.1	AT5G58760.1
Potra2n1c2184.1	-0.952173069	-0.099797432	0.765437768	NA	Potri.001G251100.2	AT2G29940.1
Potra2n1c2185.1	2.084264711	-0.815428204	0.07276627	0.941260209	Potri.001G251200.1	AT2G38170.1
Potra2n1c2186.1	-0.317039678	-0.09258945	0.006748193	0.910878765	Potri.001G251300.1	AT5G58740.1
Potra2n1c2187.1	NA	NA	NA	NA	Potri.001G251400.1	AT5G60440.1
Potra2n1c2188.1	0.130520459	0.287385812	0.59634253	0.561435202	Potri.001G251500.1	AT5G58730.1
Potra2n1c2189.1	0.59203528	0.323026356	0.161182086	0.940361195	Potri.001G251600.4	AT2G29950.1
Potra2n1c219.1	-0.660596061	-0.421852735	6.49286E-12	0.573590153	Potri.001G026600.1	AT5G13810.1
Potra2n1c2190.1	-0.075126727	0.073100183	0.637433541	0.947547562	Potri.001G251700.1	AT2G29960.1
Potra2n1c2191.1	-0.192253572	0.151599396	0.423918958	0.88661378	Potri.001G251900.1	AT5G01450.1
Potra2n1c2192.2	-0.641108452	-0.338157914	0.02481949	0.833667976	Potri.001G252000.1	AT1G07220.1
Potra2n1c2193.1	-0.079155991	-0.1885041	0.717473485	0.885649686	Potri.001G252100.1	AT5G58700.1
Potra2n1c2194.1	-1.104493921	-0.552214978	0.142723243	0.897285619	Potri.001G252400.1	AT3G55950.1

Potra2n1c2195.1	-0.329585459	0.265018769	0.072454451	0.751694102	Potri.001G252500.1	AT1G07200.2
Potra2n1c2196.1	1.099912626	-0.016980925	3.54094E-13	0.995005375	Potri.001G252600.1	AT5G58620.1
Potra2n1c2197.1	NA	NA	NA	NA		
Potra2n1c2198.1	-1.382528724	-0.284835267	5.66607E-16	0.912777843	Potri.001G252900.1	AT5G05580.1
Potra2n1c2199.1	0.363222979	0.009519953	4.17029E-05	0.993819913	Potri.001G253000.1	AT3G47300.1
Potra2n1c22.1	-0.725780823	0.096462564	0.000419386	0.906545272	Potri.001G002300.10	AT5G56460.1
Potra2n1c220.1	0.355552981	-0.046821674	0.031638469	0.974992932	Potri.001G026700.1	AT5G59560.2
Potra2n1c2200.1	0.031755656	0.235029059	0.826845319	0.55991545	Potri.001G253100.1	AT1G34470.1
Potra2n1c2201.2	-0.26056458	-0.031287855	0.196262862	0.986636967	Potri.001G253200.1	AT3G60500.3
Potra2n1c2202.1	-0.080887703	0.684962637	0.797470267	0.551699675	Potri.001G253300.13	AT2G13360.2
Potra2n1c2203.1	0.205841911	0.31672008	0.091346537	0.503119002	Potri.001G253400.10	AT2G13370.1
Potra2n1c2204.2	-0.026316966	-0.028073894	0.831447081	0.980475138	Potri.001G253500.1	AT1G48900.1
Potra2n1c2205.1	-0.194635023	0.01016319	0.321209465	0.995005375	Potri.001G253700.1	AT3G09150.2
Potra2n1c2206.1	0.572437193	0.257897864	0.002121026	0.857418365	Potri.001G253800.1	AT1G77210.2
Potra2n1c2207.1	0.186440825	0.091009537	0.338933515	0.953768643	Potri.001G253900.1	AT4G31530.1
Potra2n1c2208.1	0.44089725	0.126586946	0.000252188	0.902857376	Potri.001G254001.1	AT1G50510.1
Potra2n1c2209.1	-4.357203299	-0.443258169	0.198638707	NA	Potri.001G254100.2	AT1G74670.1
Potra2n1c221.4	-0.10375909	-0.0503807	0.556293318	0.957609353	Potri.003G197900.1	AT5G13480.1
Potra2n1c2210.2	-2.966360761	1.141403458	0.110310821	0.759797728	Potri.009G049200.1	AT2G13540.1
Potra2n1c2211.1					Potri.017G081800.1	
Potra2n1c2212.1	NA	NA	NA	NA	Potri.017G081800.1	
Potra2n1c2213.1	0.299328843	0.291517683	0.005340224	0.596772738	Potri.001G254300.1	AT5G12400.1
Potra2n1c2214.1	0.580130571	-0.257576677	0.208094236	0.898711301	Potri.001G254500.1	AT1G50490.1
Potra2n1c2215.1	0.487667697	0.245996707	0.01762924	0.814981587	Potri.001G254600.1	AT5G12410.1
Potra2n1c2216.1	0.120306842	-0.210734889	0.708004871	0.918687733	Potri.001G254800.1	AT1G50460.1
Potra2n1c2217.1	NA	1.571529889	NA	NA	Potri.001G257300.1	AT2G22720.3
Potra2n1c2218.1	-0.069879441	0.011266471	0.746123165	0.994403137	Potri.001G255000.1	AT5G12430.1
Potra2n1c2219.1	-0.082437922	0.101848371	0.657062601	0.903698951	Potri.001G255000.1	AT5G12430.1
Potra2n1c222.2	0.029198835	0.042510323	0.865106271	0.97950444	Potri.001G026900.1	AT5G19420.2
Potra2n1c2220.1	NA	NA	NA	NA		
Potra2n1c2221.1	8.715717453	5.578334914	3.42766E-19	0.420046656	Potri.001G255100.1	AT4G16260.1
Potra2n1c2222.1	2.612816361	-0.046370319	0.24723013	NA		

Potra2n1c2223.3	0.23351152	1.795748251	NA	NA	Potri.001G255300.1	
Potra2n1c2224.1	-0.891109413	-0.211867149	0.004302359	0.939285988	Potri.001G255400.1	AT3G21100.2
Potra2n1c2225.1	0.303270518	0.507378738	0.748391792	0.907073354	Potri.001G255532.1	AT3G51950.1
Potra2n1c2226.1	0.172933881	0.049924451	0.122020796	0.953831244	Potri.001G255600.2	AT1G51510.1
Potra2n1c2227.2	0.412465087	0.237972221	0.104432294	0.830056077	Potri.001G255700.2	
Potra2n1c2228.1	-0.457929371	-0.476802888	0.574647746	0.922993749	Potri.001G255800.1	AT3G21090.1
Potra2n1c2229.1	-0.144185164	0.082068621	0.790622174	0.977559521	Potri.001G255900.1	AT3G21090.1
Potra2n1c223.1	0.147435106	0.106743148	0.565627623	0.956865545	Potri.001G027000.1	AT3G57040.1
Potra2n1c2230.1	0.239533187	0.031850521	0.470046175	0.989300076	Potri.001G256000.2	AT1G51450.1
Potra2n1c2231.1	0.072924639	0.043230613	0.629528274	0.968407862	Potri.001G256100.2	AT3G21070.2
Potra2n1c2232.1	NA	NA	NA	NA	Potri.001G256200.1	AT5G12460.1
Potra2n1c2233.1	1.8343316	0.8502037	NA	NA		
Potra2n1c2234.1	1.491309611	0.350262264	3.28281E-14	0.673884433	Potri.001G256400.1	AT5G19440.1
Potra2n1c2235.1					Potri.001G256600.2	AT3G04070.2
Potra2n1c2236.1	0.084325835	0.862675244	NA	NA	Potri.001G256700.1	AT1G51405.1
Potra2n1c2237.1	0.743075178	0.050973005	1.6084E-05	0.966077009	Potri.001G257000.2	AT5G04590.1
Potra2n1c2238.1	-1.023641288	-0.395911555	3.9973E-09	0.66992341	Potri.001G256900.1	AT1G51400.1
Potra2n1c2239.1	-0.333748921	-0.223982663	0.079709063	0.835819291	Potri.001G257100.1	AT5G12480.1
Potra2n1c224.2	-1.13022431	0.002259581	0.001561997	0.999458638	Potri.001G027100.1	AT3G54450.1
Potra2n1c2240.1	-0.857777197	-0.235654183	0.036285643	0.909675463	Potri.001G257400.1	AT2G01870.1
Potra2n1c2241.2	-0.113039571	-0.16390889	0.373449777	0.813944514	Potri.001G257500.1	AT3G20920.1
Potra2n1c2242.1	-0.062707965	0.063009467	0.728589373	0.953108412	Potri.001G257600.1	AT5G12840.2
Potra2n1c2243.1	-0.01330131	-0.437433	0.977318556	0.738164159	Potri.001G257700.1	AT3G20898.1
Potra2n1c2244.1	-0.171207698	0.063441162	0.186587791	0.945173238	Potri.001G258000.1	AT3G20890.1
Potra2n1c2245.1	1.360989519	0.381632226	0.039096336	0.885375133	Potri.001G258100.1	AT5G19855.1
Potra2n1c2246.1	0.040030601	0.313464455	0.91714837	0.862607943	Potri.001G258400.2	AT5G12850.1
Potra2n1c2247.1	-0.369311719	-0.231924468	0.31469284	0.913281585	Potri.001G258500.1	AT4G13650.1
Potra2n1c2248.1	0.399708795	-0.012137319	0.007112399	0.995005375	Potri.001G258600.1	AT5G12860.1
Potra2n1c2249.1	0.247943084	-0.089454355	0.198900169	0.951731753		
Potra2n1c225.1	-1.54862513	-0.377503309	9.58413E-07	0.850863374	Potri.003G196900.1	AT2G40030.1
Potra2n1c2250.1	-0.967901666	-0.358120839	1.46426E-06	0.633830624	Potri.001G258700.1	AT5G12870.1
Potra2n1c2251.1	0.870321629	0.288891927	5.06284E-07	0.759797728	Potri.009G054000.3	AT1G50920.1

Potra2n1c2252.1	0.01995529	0.126009579	0.905702423	0.907073354	Potri.001G259000.1	AT1G51310.1
Potra2n1c2253.1	0.63582624	0.769459564	0.797627653	NA	Potri.001G259100.7	AT4G36250.1
Potra2n1c2254.1	-0.468467863	0.297718444	0.030501504	0.833952007	Potri.001G259200.3	AT1G51260.1
Potra2n1c2255.1	0.650094169	2.993160496	0.670763699	0.323147091		
Potra2n1c2256.1	0.87770358	4.185055065	0.604750662	0.418644133	Potri.001G260200.1	
Potra2n1c2257.2	1.737527072	3.971790056	0.639205375	0.64562236		
Potra2n1c2258.1	0.121860292	-0.093720788	0.708927082	0.957525726	Potri.001G259900.1	AT5G43210.1
Potra2n1c2259.1	-0.593571325	-0.077657761	0.431735437	0.987226805	Potri.001G260700.1	AT3G51970.1
Potra2n1c226.1	-0.037702827	0.491381162	0.842022417	0.725682228	Potri.001G027400.1	AT3G54440.1
Potra2n1c2260.1	0.637834327	-0.224080617	0.000323528	0.860901249	Potri.001G260800.1	AT3G51990.1
Potra2n1c2261.1	-0.10072323	-0.021444273	0.569595352	0.988415	Potri.001G260900.1	AT3G63460.1
Potra2n1c2262.1						
Potra2n1c2263.1	1.64774763	NA	NA	NA	Potri.001G261201.1	AT3G63470.1
Potra2n1c2264.1	0.040809526	0.101969939	0.844618981	0.921063801	Potri.001G261300.1	AT3G14460.1
Potra2n1c2265.1	-0.428952126	0.01454568	0.017288938	0.992934677	Potri.001G261300.1	AT3G14460.1
Potra2n1c2266.1	2.142767311	0.811314573	0.026662488	0.895474078	Potri.001G261366.1	
Potra2n1c2267.1	-0.086750902	0.101928046	0.671887165	0.931774514	Potri.001G261300.1	AT3G14460.1
Potra2n1c2268.1	-0.02781774	0.093330597	0.899624647	0.936549944		
Potra2n1c2269.1	0.007471234	0.091723279	0.976262397	0.939149035	Potri.001G261300.1	AT3G14460.1
Potra2n1c227.1	0.349030003	0.126776256	0.813360459	0.987226805	Potri.001G027500.1	AT1G08590.1
Potra2n1c2270.1	-0.61910233	-0.767708831	NA	NA	Potri.004G016200.1	AT3G60840.1
Potra2n1c2271.3	-0.256743633	0.079689554	0.252579225	0.943819961	Potri.001G261600.1	AT3G14470.1
Potra2n1c2272.1	-0.232117883	0.014193636	0.110005102	0.992934677	Potri.001G261600.1	AT3G14470.1
Potra2n1c2273.3	0.498245387	0.189315341	9.67107E-06	0.833952007	Potri.001G261800.1	AT5G13780.1
Potra2n1c2274.1	0.267170882	-0.061382845	0.266657766	0.9761362	Potri.001G261900.1	AT5G13760.1
Potra2n1c2275.1	-0.160494335	-0.080221856	0.657862799	0.960981446	Potri.001G262000.1	AT3G52040.1
Potra2n1c2276.1	0.859677716	0.191688876	9.56175E-09	0.818284569	Potri.001G262100.1	AT5G59240.1
Potra2n1c2277.1	0.959905756	0.241152939	2.57194E-08	0.799601397	Potri.001G262200.5	AT5G12470.1
Potra2n1c2278.1	-0.551271448	-0.027200986	4.37611E-07	0.982629254	Potri.001G262400.6	AT3G63490.1
Potra2n1c2279.1	-0.020176522	0.113376158	0.841736751	0.888366602	Potri.001G262600.1	AT5G15920.1
Potra2n1c228.1	-0.452386983	-0.247691462	0.001024274	0.534852254	Potri.001G027600.2	
Potra2n1c2280.1					Potri.009G057300.1	AT5G13720.1

Potra2n1c2281.1	1.459962178	NA	NA	NA	Potri.001G262800.1	AT2G34930.1
Potra2n1c2282.1	3.556456493	1.363811008	0.380439637	NA	Potri.001G263200.1	AT1G30370.1
Potra2n1c2283.1	1.532277385	-0.119338036	0.144745569	0.989304496	Potri.001G263400.1	AT5G13700.1
Potra2n1c2284.1	NA	NA	NA	NA	Potri.009G058300.1	AT3G53170.1
Potra2n1c2285.1	-0.428535775	-0.326430447	0.369772821	0.922011871	Potri.001G263700.1	AT5G13710.1
Potra2n1c2286.1	0.403025463	0.089640967	0.030503388	0.943483421	Potri.001G263800.1	AT5G09680.2
Potra2n1c2287.1	0.487094379	-0.26321518	0.268862834	0.829862776	Potri.001G263900.1	AT3G08030.1
Potra2n1c2288.2	0.931171965	-0.343685479	0.010186441	0.895474078	Potri.001G264000.1	AT3G23890.2
Potra2n1c2289.1	0.390992999	0.382863157	0.000220701	0.459449783	Potri.001G264100.1	AT3G63500.2
Potra2n1c229.1	-2.678204181	-1.385466389	0.029731742	0.669027738	Potri.001G027900.1	AT3G07960.1
Potra2n1c2290.1	0.312547061	0.074463283	0.049517838	0.948577865	Potri.001G264400.1	AT3G21640.1
Potra2n1c2291.1	-0.053713306	0.132830039	0.753501305	0.910477984	Potri.001G264500.1	AT3G27310.1
Potra2n1c2292.1	-0.855504734	1.478018672	NA	NA	Potri.009G059400.1	AT3G52080.1
Potra2n1c2293.1	-0.16155035	0.007604564	0.069835278	0.992934677	Potri.001G264800.1	AT3G08020.1
Potra2n1c2294.1	-0.221681505	-0.101973379	0.095684278	0.88643982	Potri.001G265100.1	AT5G47940.1
Potra2n1c2295.2	-0.574338625	-0.162676615	0.0009464	0.855373532	Potri.001G265200.1	AT3G60830.1
Potra2n1c2296.1	-1.755744694	-0.268269236	6.88256E-06	0.899990252	Potri.001G265300.1	AT2G28305.1
Potra2n1c2297.1	-0.234015462	0.11723582	0.267261324	0.936890993	Potri.001G265400.1	AT3G63520.1
Potra2n1c2298.1	-0.146309698	0.311328193	0.716910736	0.846399928	Potri.001G266000.4	AT5G12840.2
Potra2n1c2299.2	-0.203206108	0.013649463	0.220854749	0.990138406	Potri.001G266200.2	AT4G15030.2
Potra2n1c23.1	0.590802252	0.278754068	5.80774E-06	0.677589151	Potri.001G002500.1	AT3G13470.1
Potra2n1c230.1	-0.727645993	-0.141886124	0.00071857	0.930871994	Potri.001G028000.1	AT3G27890.1
Potra2n1c2300.1	0.480280427	0.15379428	0.176765221	0.870614204	Potri.001G266400.3	AT5G05170.1
Potra2n1c2301.1	4.692322162	1.363811008	0.055252373	NA	Potri.001G266500.1	AT1G07985.1
Potra2n1c2302.2	0.066733195	-0.079921908	0.634216506	0.947969749	Potri.001G266700.1	AT2G41900.1
Potra2n1c2303.1	-1.761105459	0.044634279	0.000530546	0.983113084	Potri.001G266900.1	AT3G08040.1
Potra2n1c2304.1	-1.605373156	-0.80022156	1.29055E-07	0.272381508	Potri.001G267200.1	AT3G08490.1
Potra2n1c2305.1	-0.410805708	0.010677256	0.050964097	0.997079482	Potri.001G267300.1	AT3G08500.1
Potra2n1c2306.1	1.138406543	0.355430548	1.02172E-09	0.736000077	Potri.001G267400.1	AT4G26900.1
Potra2n1c2307.1	0.655600807	-0.370268603	0.000269841	0.696755852	Potri.001G267501.1	AT3G52105.1
Potra2n1c2308.2	0.631405647	-0.297096236	0.00026567	0.802631112	Potri.001G267600.1	AT3G52110.2
Potra2n1c2309.1	1.212999622	2.927830513	NA	NA	Potri.001G267700.1	AT3G63530.2

Potra2n1c231.1	-0.92443247	0.02606953	0.002138183	0.992934677	Potri.001G028100.1	AT5G13500.2
Potra2n1c2310.1	0.252032459	0.113273402	0.075102556	0.905991243	Potri.001G267800.1	AT5G13490.2
Potra2n1c2311.1	4.084614497	4.011859777	0.1425833	NA	Potri.001G267900.2	AT3G57670.1
Potra2n1c2312.1	-0.526605336	-0.208687761	0.264581363	0.948577865	Potri.001G268000.1	
Potra2n1c2313.1	0.124708906	0.227422463	0.322237815	0.788065047	Potri.001G268200.1	AT3G08505.1
Potra2n1c2314.1	3.219950245	1.381953902	NA	NA	Potri.001G268400.1	
Potra2n1c2315.1	NA	NA	NA	NA		
Potra2n1c2316.1	1.478301766	0.02168417	0.005845459	0.992934677	Potri.001G268500.1	
Potra2n1c2317.1	3.896384281	1.731097546	5.43129E-31	0.375012853	Potri.001G268600.1	AT4G37980.1
Potra2n1c2318.1	NA	0.674963689	NA	NA	Potri.001G268801.1	AT2G41905.1
Potra2n1c2319.1	0.080588615	-0.371089339	0.855703789	0.849815276	Potri.001G269001.1	AT3G12650.1
Potra2n1c232.1	-0.496066813	-0.399127592	0.017288938	0.802631112	Potri.001G028200.1	AT3G54400.1
Potra2n1c2320.1	0.116774702	0.02350883	0.311889045	0.978646026	Potri.001G269200.1	AT3G12640.1
Potra2n1c2321.1	-0.61910233	-0.767708831	NA	NA		
Potra2n1c2322.1	0.10911831	0.123866018	0.464640289	0.943438281	Potri.001G269400.1	AT3G12630.1
Potra2n1c2323.1	0.771357893	0.226908903	0.014118647	0.903477395		
Potra2n1c2324.1	0.236140154	0.04168892	0.195885252	0.978646026	Potri.001G269500.5	AT2G03870.1
Potra2n1c2325.1	0.463440817	-0.048690783	0.00485347	0.965445938	Potri.001G269600.1	AT2G19740.1
Potra2n1c2326.1	0.752047579	0.426954888	0.000380665	0.433369183	Potri.001G269700.1	AT3G52115.1
Potra2n1c2327.1	-2.203117643	-1.660854098	0.010069974	0.327435845	Potri.001G269800.1	AT3G20820.1
Potra2n1c2328.1	1.459962178	NA	NA	NA	Potri.001G269850.1	
Potra2n1c2329.1	-0.18765066	-0.01468277	0.051243972	0.989300076	Potri.001G269900.2	AT3G52120.1
Potra2n1c233.1	0.052765581	-0.092341431	0.722493591	0.887678043	Potri.001G028300.1	AT1G08315.1
Potra2n1c2330.3	-0.249437083	-0.173150462	0.170581104	0.837874974	Potri.001G270000.1	AT2G42280.1
Potra2n1c2331.1	0.025024854	0.02986934	0.8706219	0.974264269	Potri.001G270100.1	AT3G12160.1
Potra2n1c2332.1	7.285693499	3.846202676	2.55808E-07	0.064817571	Potri.001G270300.1	AT1G12740.1
Potra2n1c2333.1	6.136121245	3.258956542	2.49783E-48	3.26803E-06	Potri.001G270500.1	AT1G12740.1
Potra2n1c2334.1	-1.903049284	-2.05258594	NA	NA	Potri.001G274600.1	AT5G07990.1
Potra2n1c2335.1	NA	NA	NA	NA	Potri.001G274700.1	AT5G12180.1
Potra2n1c2336.1					Potri.001G274800.2	AT5G19370.1
Potra2n1c2337.1	0.160518648	0.146859313	0.017916881	0.662390414	Potri.001G274900.2	AT5G13530.1
Potra2n1c2338.1	0.028826161	0.080168036	0.906701886	0.9574223	Potri.001G275000.1	AT1G62350.1

Potra2n1c2339.1	1.64774763	NA	NA	NA	Potri.001G275100.1	AT4G28010.1
Potra2n1c234.1	0.149411001	-0.866977801	0.949070453	NA		
Potra2n1c2340.1	NA	NA	NA	NA		
Potra2n1c2341.1	-0.223548047	-0.027711949	0.100408859	0.981772014	Potri.001G275200.1	AT5G19390.1
Potra2n1c2342.1	-0.316186884	-0.167788532	0.012892069	0.814981587	Potri.001G275300.1	AT4G29070.2
Potra2n1c2343.1	0.27693393	0.167658521	0.002750515	0.700160628	Potri.001G275400.2	AT5G19400.1
Potra2n1c2344.1	1.686315318	0.250226566	1.83488E-07	0.92651938	Potri.001G275500.1	AT2G26870.1
Potra2n1c2345.1	0.684415937	0.130291768	2.97278E-05	0.910815461	Potri.001G275600.1	AT5G13500.3
Potra2n1c2346.1	1.664677056	2.676364087	NA	NA	Potri.001G275700.1	AT5G49700.1
Potra2n1c2347.1	-0.083519318	-0.337389128	0.767339996	0.844277327	Potri.001G275800.1	AT5G12300.1
Potra2n1c2348.2	0.158954162	0.22518904	0.295369479	0.802942736	Potri.009G070600.1	AT5G19430.1
Potra2n1c2349.1	-0.25562426	-0.041668409	0.223860161	0.976302215	Potri.001G276100.1	AT5G12320.1
Potra2n1c235.1	-0.159062425	-0.133825608	0.555255076	0.829455551	Potri.001G028400.1	AT3G54390.1
Potra2n1c2350.1	1.600901948	0.5505707	7.9135E-12	0.788065047	Potri.001G276200.2	AT5G12330.4
Potra2n1c2351.1						
Potra2n1c2352.1	0.457623814	0.62370366	0.203111024	0.560682869	Potri.014G192200.1	AT5G44010.1
Potra2n1c2353.1	-0.552112368	-0.18761375	0.002056985	0.857189843	Potri.014G192300.1	AT5G44000.1
Potra2n1c2354.1	-0.497618661	0.083395723	0.005697386	0.956865545	Potri.014G192500.2	AT5G43990.5
Potra2n1c2355.1	0.105997571	0.085776895	0.669804536	0.965445938	Potri.014G192601.1	AT5G43970.1
Potra2n1c2356.1	-0.973292779	-1.715664642	0.605899061	NA		
Potra2n1c2357.7	-0.076519146	-0.009039176	0.65511746	0.994329048	Potri.014G192800.1	AT1G04080.1
Potra2n1c2358.1	-0.225291389	0.139388887	0.314647157	0.897973296	Potri.014G192900.1	AT5G43960.1
Potra2n1c2359.1	-0.600078873	-0.065716624	0.000329239	0.950095151	Potri.014G193100.1	AT5G43950.1
Potra2n1c236.1	-0.097936705	-0.128156308	0.771147637	0.957609353	Potri.001G028500.1	AT3G06730.1
Potra2n1c2360.2	0.198676814	0.028197361	0.004828549	0.966148753	Potri.014G193500.1	AT2G20330.1
Potra2n1c2361.1	3.960629566	1.410518579	1.01896E-73	0.089235664	Potri.002G254900.6	AT5G43940.1
Potra2n1c2362.1	-0.035266903	-0.031749147	0.811114005	0.974165061	Potri.014G193900.2	AT1G04140.1
Potra2n1c2363.1	-0.857475353	-0.130172933	2.40628E-06	0.862335622	Potri.014G194100.1	AT5G43920.1
Potra2n1c2364.1	1.138455458	-0.783222569	0.029863942	0.560708883	Potri.001G270800.1	AT1G12740.1
Potra2n1c2365.1	4.39874425	2.86450839	0.199412897	NA	Potri.001G270900.1	AT3G20950.1
Potra2n1c2366.1	0.82357019	1.394613675	NA	NA	Potri.001G271000.2	AT3G52130.1
Potra2n1c2367.1	-0.417707835	-0.148520021	0.013164934	0.813731863	Potri.001G271100.1	AT5G48020.1

Potra2n1c2368.2	0.277197115	0.133233327	0.04161437	0.865591169	Potri.001G271200.2	AT3G52140.1
Potra2n1c2369.1	-0.037330156	0.05854334	0.81753104	0.966917036	Potri.001G271300.1	AT5G48030.1
Potra2n1c237.1	0.032049456	0.126006039	0.937379086	0.959881296	Potri.001G028800.1	
Potra2n1c2370.1	1.664677056	2.32414027	NA	NA	Potri.001G271400.1	AT5G48060.1
Potra2n1c2371.1	0.807927754	0.194370618	1.79052E-06	0.888518641	Potri.001G271500.1	AT1G74050.1
Potra2n1c2372.1	-1.64473862	-1.044528074	NA	NA	Potri.009G065900.1	AT3G52150.2
Potra2n1c2373.1	3.365036301	1.060459683	2.73964E-13	0.540010302	Potri.001G271700.1	AT3G45640.1
Potra2n1c2374.1	NA	NA	NA	NA	Potri.001G006800.2	AT3G13610.1
Potra2n1c2375.1	0.444501238	0.152342225	0.00055969	0.842247476	Potri.001G271800.2	AT5G19485.1
Potra2n1c2376.1	0.129555011	-0.019551276	0.464174327	0.987226805	Potri.001G272000.1	AT2G26760.1
Potra2n1c2377.1	0.141135023	0.091529149	0.402823147	0.92560649	Potri.001G272100.1	AT2G43760.3
Potra2n1c2378.1	-0.243745075	0.270485263	0.064490292	0.728883321	Potri.001G272300.1	AT2G26770.1
Potra2n1c2379.1	-0.295665637	-0.242676317	0.082490198	0.842247476	Potri.001G272400.1	AT5G12260.1
Potra2n1c238.1	-1.146813382	-0.996677254	0.254607879	0.734313982		
Potra2n1c2380.1	NA	NA	NA	NA		
Potra2n1c2381.1	0.102236182	1.177743307	NA	NA	Potri.016G070500.1	AT2G01820.1
Potra2n1c2382.1	-0.724708563	-0.197547495	0.002674337	0.910815461	Potri.001G272800.1	AT5G62690.1
Potra2n1c2383.1	-0.399187219	0.083922412	0.115198033	0.975244691	Potri.001G272800.1	AT5G62690.1
Potra2n1c2384.1	3.485007864	2.868603373	0.25473881	NA	Potri.001G272900.1	AT1G59740.1
Potra2n1c2385.1	-0.702079098	-0.267726301	0.000105977	0.725595998	Potri.001G273000.1	AT1G08500.1
Potra2n1c2386.1	-0.773679584	-0.289812204	0.004815736	0.798096728		
Potra2n1c2387.2	-0.046068176	0.131669049	0.870388369	0.92812284	Potri.001G273100.1	AT1G11900.1
Potra2n1c2388.1	-0.300769405	0.099062004	0.815549368	0.987959275		
Potra2n1c2389.3	3.957040005	1.691679265	5.83497E-38	0.059891583	Potri.004G025900.1	AT4G05200.1
Potra2n1c239.1	-0.007685482	-0.076260276	0.976449721	0.95999889	Potri.001G029400.1	AT5G07900.1
Potra2n1c2390.1	-0.238040904	-0.111378929	0.038612176	0.869186333	Potri.001G273200.1	AT5G12190.1
Potra2n1c2391.1	1.20589458	0.551885988	2.75683E-26	0.099597414	Potri.009G067700.1	AT5G12200.1
Potra2n1c2392.1						
Potra2n1c2393.1	-0.231464328	-0.054325966	0.552383199	0.976302215	Potri.001G273600.2	AT5G12220.1
Potra2n1c2394.1	-0.61155656	0.020168011	0.480836177	0.996032145	Potri.001G273700.1	AT1G46264.1
Potra2n1c2395.1	-0.998883168	-0.252750228	4.18802E-08	0.859510169	Potri.001G273900.1	AT2G32560.1
Potra2n1c2396.1	-0.875660495	-0.284047512	0.000150585	0.829716187	Potri.001G274000.1	AT2G26530.1

Potra2n1c2397.1	-0.3035622	0.009045667	0.059285165	0.995579852	Potri.009G068600.1	AT2G26800.1
Potra2n1c2398.1	-3.378190375	-0.99908366	0.000222564	0.715585895	Potri.001G274200.1	AT5G12235.1
Potra2n1c2399.1	0.294586509	-0.002838684	0.062357522	0.998245864	Potri.001G274400.1	AT5G12240.1
Potra2n1c24.1	0.193389107	-0.184311387	0.327763052	0.895575326	Potri.001G002600.2	AT5G56510.1
Potra2n1c240.1	0.116373679	-0.003194232	0.347108446	0.996851113	Potri.001G029500.1	AT3G12600.1
Potra2n1c2400.1	0.172221684	0.186339586	0.039085657	0.516379084	Potri.001G274500.2	AT2G26780.1
Potra2n1c2401.1	NA	1.363811008	NA	NA	Potri.009G071100.1	AT5G12340.1
Potra2n1c2402.1	NA	NA	NA	NA		
Potra2n1c2403.1	0.149577166	0.299231794	0.430847938	0.588516072	Potri.001G276500.10	AT5G01110.1
Potra2n1c2404.1	2.48791875	-0.111642176	0.00014442	0.991407187	Potri.001G276600.1	AT1G70530.1
Potra2n1c2405.1	0.079360162	-0.030517982	0.6775155	0.982422288	Potri.001G276700.2	AT2G03120.1
Potra2n1c2406.1	-0.220849868	0.040290623	0.059550873	0.960981446	Potri.001G276800.2	AT4G03250.1
Potra2n1c2407.4	0.069175975	0.019528059	0.588952256	0.983446961	Potri.001G276900.1	AT5G19420.1
Potra2n1c2408.1	0.824497211	-0.767708831	NA	NA	Potri.001G006600.1	AT1G55320.1
Potra2n1c2409.1	0.236957312	-0.199672992	0.750554952	0.966917036	Potri.001G006700.1	AT1G55320.1
Potra2n1c241.2	0.064952145	0.009337019	0.455076603	0.989304496	Potri.001G029600.1	AT5G13530.1
Potra2n1c2410.1					Potri.T170500.1	AT3G13610.1
Potra2n1c2411.1	NA	0.674963689	NA	NA	Potri.001G277000.1	AT1G72300.1
Potra2n1c2412.5	-0.011149468	0.079694086	0.927937907	0.893135869	Potri.001G277200.1	AT4G14590.1
Potra2n1c2413.1	0.580573914	0.291264051	0.015700049	0.797111689	Potri.016G031800.1	AT1G34540.1
Potra2n1c2414.1	-0.247279077	0.172556411	0.422581595	0.900015282	Potri.001G277400.1	AT2G17030.1
Potra2n1c2415.1						
Potra2n1c2416.1	2.476290371	NA	NA	NA	Potri.001G277500.1	AT5G12380.1
Potra2n1c2417.1	-0.37596343	-0.323746582	0.6640269	0.890978957	Potri.001G277600.1	AT1G07175.1
Potra2n1c2418.1	1.635044354	0.67282749	6.5511E-12	0.454099783	Potri.001G277800.1	AT1G07170.2
Potra2n1c2419.1	0.120867861	-0.012643552	0.362381671	0.989304496	Potri.001G278000.1	AT1G65700.1
Potra2n1c242.1	NA	NA	NA	NA		
Potra2n1c2420.1	2.366186576	0.128056069	0.000314384	0.983446961	Potri.001G278200.1	AT5G58660.1
Potra2n1c2421.1	0.109544591	-0.230206628	0.780710842	0.902080412	Potri.001G278300.1	AT5G58600.1
Potra2n1c2422.1	-1.954596188	0.965889907	0.211088658	0.850863374	Potri.009G072900.2	AT3G47340.1
Potra2n1c2423.1	NA	NA	NA	NA	Potri.008G220300.1	
Potra2n1c2424.1	1.64774763	NA	NA	NA	Potri.008G220300.1	

Potra2n1c2425.1	NA	NA	NA	NA	Potri.001G344400.1	AT5G40510.1
Potra2n1c2426.1	NA	NA	NA	NA		
Potra2n1c2427.1	NA	NA	NA	NA	Potri.T076201.1	AT5G45400.1
Potra2n1c2428.1	1.236590282	0.135192705	0.273326495	NA		
Potra2n1c2429.1	0.90545151	0.395455303	1.48851E-05	0.70570456	Potri.001G278500.1	AT2G30020.1
Potra2n1c243.1	6.395986152	3.546442195	5.91194E-06	NA	Potri.001G029700.1	AT3G56710.1
Potra2n1c2430.1						
Potra2n1c2431.4	-0.060928422	-0.005991579	0.681278441	0.996032145	Potri.001G278800.2	AT3G11130.1
Potra2n1c2432.1	-0.065874184	-0.119435275	0.565604163	0.832750076	Potri.001G278900.1	AT2G30060.1
Potra2n1c2433.3	-0.726758842	0.025224216	5.33675E-05	0.981772014	Potri.001G279000.1	AT1G07120.1
Potra2n1c2434.1	-0.0953297	0.006395856	0.400101907	0.995565508	Potri.001G279100.1	AT1G07110.1
Potra2n1c2435.1	0.692880687	0.212037177	3.05203E-06	0.726986309	Potri.001G279300.1	AT2G30080.1
Potra2n1c2436.1	-1.009728413	0.251830423	0.136381762	0.950549007	Potri.001G279400.1	AT2G30090.1
Potra2n1c2437.1	-1.008770035	-0.345270579	1.44527E-13	0.749986406	Potri.001G279500.1	AT3G51780.1
Potra2n1c2438.1	-0.145851477	0.071909193	0.367596544	0.943685779	Potri.001G279700.1	AT5G58575.1
Potra2n1c2439.1	1.310718164	0.959578502	0.605739608	NA	Potri.001G279800.1	AT5G58570.1
Potra2n1c244.2	0.318259105	0.001436953	0.000963188	0.999325699	Potri.001G029800.11	AT3G12250.2
Potra2n1c2440.1	0.263569494	0.063238513	0.039189529	0.939653927	Potri.001G279900.1	AT5G58560.1
Potra2n1c2441.1	0.921450698	0.226336815	0.599807475	NA	Potri.001G280000.1	AT2G38300.1
Potra2n1c2442.1	-0.357503975	0.090458117	0.044387129	0.963993404	Potri.009G075300.1	AT3G51770.2
Potra2n1c2443.1	-0.553222826	0.216600127	0.027435204	0.929556032	Potri.001G280200.1	AT5G45840.2
Potra2n1c2444.2	0.802170114	0.499760292	0.171923046	0.817452127	Potri.001G280266.1	
Potra2n1c2445.1	0.813920203	0.223244367	0.328832674	0.953608209	Potri.017G038300.1	AT4G26400.1
Potra2n1c2446.1	0.940033715	0.941112323	0.431623411	0.759797728	Potri.017G038300.1	AT4G26400.1
Potra2n1c2447.1	1.760318459	1.69109406	0.181674281	0.544461626	Potri.017G038300.1	AT4G26400.1
Potra2n1c2448.1	-0.783512	-0.392705129	3.8204E-05	0.64781796	Potri.001G280400.1	AT5G58530.1
Potra2n1c2449.1	0.210309846	0.105168839	0.055002723	0.902676973	Potri.001G280500.2	AT2G30105.1
Potra2n1c245.1	-0.018313797	-0.042543758	0.897107376	0.965249957	Potri.001G029900.1	AT3G12260.1
Potra2n1c2450.1	-0.07832136	-0.064754952	0.631194504	0.964333742	Potri.001G280600.1	AT2G30110.1
Potra2n1c2451.1	-1.162622974	-0.416629139	7.73894E-07	0.739555983	Potri.001G280700.1	AT5G58520.1
Potra2n1c2452.1	-0.61910233	-0.767708831	NA	NA	Potri.009G076000.1	AT5G58510.1
Potra2n1c2453.1	NA	1.571529889	NA	NA	Potri.009G076000.1	AT5G58510.1

Potra2n1c2454.1	-0.61910233	3.068889115	NA	NA		
Potra2n1c2455.1	-0.144820969	0.932643748	0.791752929	0.560708883	Potri.001G281101.1	
Potra2n1c2456.1	1.004423551	0.890981192	2.78083E-06	0.404632832	Potri.001G281200.2	AT3G10110.1
Potra2n1c2457.2	0.081420638	-0.067638679	0.584227794	0.920141992	Potri.001G281300.1	AT5G58470.2
Potra2n1c2458.1	0.655480574	0.319541753	9.70246E-05	0.754074409	Potri.001G281400.1	AT3G14750.1
Potra2n1c2459.1	0.120895928	-0.561773953	0.856025962	0.845379753	Potri.001G281500.2	AT1G07060.1
Potra2n1c246.1	0.1322556	-0.147542484	0.350577551	0.821709751	Potri.001G030000.1	AT2G38730.1
Potra2n1c2460.1	2.02054234	1.381953902	NA	NA		
Potra2n1c2461.1	4.962362553	1.571529889	0.03914091	NA	Potri.001G281600.1	AT2G30130.1
Potra2n1c2462.1	1.291265159	-0.192413152	0.001135009	0.965215635	Potri.001G281700.1	AT1G07050.1
Potra2n1c2463.1	3.135581987	-0.219273341	2.05863E-18	0.963557834	Potri.001G281800.1	AT2G30150.1
Potra2n1c2464.1	3.086821978	0.376382431	1.9589E-21	0.844250405	Potri.001G281900.1	AT2G30140.1
Potra2n1c2465.1	3.670824429	1.987858534	1.99636E-10	0.312546315		
Potra2n1c2466.1	7.777089022	3.605725291	9.74847E-11	NA	Potri.001G282100.1	AT2G30140.2
Potra2n1c2467.1	NA	NA	NA	NA		
Potra2n1c2468.1	NA	NA	NA	NA		
Potra2n1c2469.1	-0.066674186	-0.089044866	0.677081449	0.915488947	Potri.001G282500.1	AT2G30170.1
Potra2n1c247.1	-0.458961307	-0.209871057	0.004948398	0.845920228	Potri.001G030100.1	AT5G06970.1
Potra2n1c2470.1	0.015905201	-0.101762718	0.933698062	0.90634987	Potri.001G282400.1	AT1G07030.1
Potra2n1c2471.1	0.961729784	0.737936133	0.450236304	0.911339141	Potri.009G077800.2	AT2G30200.1
Potra2n1c2472.1	-0.001545106	-1.507841271	NA	NA	Potri.009G077900.1	AT1G07010.1
Potra2n1c2473.1					Potri.003G087350.1	
Potra2n1c2474.1	-2.40976443	-0.333029891	0.001897876	0.912777843		
Potra2n1c2475.1	NA	NA	NA	NA	Potri.001G282800.1	AT5G37060.1
Potra2n1c2476.1	0.652237859	-0.007673707	0.000773447	0.996748906	Potri.001G282900.1	AT5G09570.1
Potra2n1c2477.1	-0.091105235	-0.122084996	0.590373522	0.918687733	Potri.001G283000.2	AT1G74520.1
Potra2n1c2478.1	2.167895245	2.649462788	0.133946497	0.674283226	Potri.013G138400.1	ATCG00830.1
Potra2n1c2479.1	2.872754102	2.870257266	0.001694423	0.457213513	Potri.013G138584.1	ATCG00830.1
Potra2n1c248.2	0.24341276	0.101229892	0.290491003	0.951726178	Potri.001G030200.3	AT3G12270.1
Potra2n1c2480.1	-0.61910233	-0.767708831	NA	NA	Potri.013G138500.1	ATCG01280.1
Potra2n1c2481.1	4.888742564	1.606252893	2.96076E-18	0.415978723	Potri.001G283400.1	AT5G10530.1
Potra2n1c2482.1	NA	1.571529889	NA	NA	Potri.004G232000.1	

Potra2n1c2483.3	-1.113454008	0.000522265	4.93511E-10	0.999937671	Potri.001G284301.1	AT4G37940.1
Potra2n1c2484.2	-1.206902435	-0.020628482	1.63133E-12	0.991032223	Potri.001G284800.1	
Potra2n1c2485.1	-0.173801628	-0.061353573	0.308206189	0.946464685	Potri.001G285000.1	AT5G09580.1
Potra2n1c2486.1	0.499584701	0.072652115	0.000175001	0.953608209	Potri.001G285500.1	AT5G09590.1
Potra2n1c2487.1	0.102236182	-0.767708831	NA	NA		
Potra2n1c2488.3	0.223143936	0.235940493	0.132663111	0.784407529	Potri.009G079900.1	AT5G64420.1
Potra2n1c2489.1	0.029133259	-0.084074839	0.846958004	0.896937427	Potri.001G285700.1	AT1G73150.1
Potra2n1c249.1	-0.155359015	-0.054990993	0.656514348	0.972159498	Potri.001G030300.1	AT5G07900.1
Potra2n1c2490.1	-0.10472369	-0.435222205	0.962347275	NA		
Potra2n1c2491.1	0.043095232	0.140156689	0.732383981	0.829837204	Potri.001G285900.1	AT5G64440.1
Potra2n1c2492.1	-0.079624187	0.132723564	0.534315748	0.846926485	Potri.001G286000.2	AT5G64460.8
Potra2n1c2493.1	0.034724438	0.006850464	0.89294464	0.996748906	Potri.001G286100.1	AT5G64470.2
Potra2n1c2494.2	-0.293981311	-0.011525887	0.053277135	0.993589787	Potri.001G286300.1	AT5G09630.1
Potra2n1c2495.1	-0.336555401	-0.40117945	0.757092983	0.945454119	Potri.001G286200.1	AT5G64480.1
Potra2n1c2496.1	0.417476902	-0.011541409	0.015761273	0.993579699	Potri.001G286400.1	AT1G58290.1
Potra2n1c2497.1	-2.239013547	-1.671008871	NA	NA		
Potra2n1c2498.1	0.709695327	0.021266118	3.9072E-06	0.985912891	Potri.001G286500.1	AT4G21860.3
Potra2n1c2499.1	-0.517495662	-0.187818603	0.000170564	0.834759753	Potri.001G286600.5	AT5G64500.1
Potra2n1c25.1	0.924413359	0.632158571	0.000356543	0.475175307	Potri.001G003100.1	AT5G36110.1
Potra2n1c250.1	NA	NA	NA	NA	Potri.001G030401.1	AT4G37210.1
Potra2n1c2500.1	0.742352326	0.307645393	1.13102E-07	0.788065047	Potri.001G286700.1	AT5G56000.1
Potra2n1c2501.1	0.617102966	0.049890468	0.000173661	0.981014593	Potri.001G286700.1	AT5G56000.1
Potra2n1c2502.1	2.002514212	-0.228146829	0.000173605	0.936549944	Potri.009G081150.1	
Potra2n1c2503.1	-0.152794744	-0.148241722	0.370446	0.812373531	Potri.001G286900.2	AT5G09650.1
Potra2n1c2504.1	0.329898897	0.207743539	0.004787738	0.751694102	Potri.001G287000.1	AT2G14255.1
Potra2n1c2505.1	0.767070627	-0.170595191	1.67088E-10	0.935474933	Potri.001G287100.1	AT4G33670.1
Potra2n1c2506.1	1.244096636	-0.016910971	0.023563529	0.996032145	Potri.001G287200.1	AT4G37850.1
Potra2n1c2507.1	NA	2.509020267	NA	NA	Potri.001G287300.1	
Potra2n1c2508.1	-0.10315804	0.863492416	0.864043389	0.657139582	Potri.001G287400.2	AT2G22780.1
Potra2n1c2509.3	-0.199826485	-0.088500952	0.105513836	0.907073354	Potri.001G287600.1	AT3G24730.1
Potra2n1c251.1	0.102236182	-0.767708831	NA	NA	Potri.001G030500.1	AT4G37210.1
Potra2n1c2510.1	0.153940321	0.018286147	0.268625192	0.98669034	Potri.001G287700.1	AT2G14260.1

Potra2n1c2511.1	0.850739684	-0.088763242	0.052134496	0.982422288	Potri.001G287800.1	AT4G17000.1
Potra2n1c2512.1	-0.031114731	0.152190127	0.884628951	0.888018559	Potri.001G287900.10	AT4G33690.1
Potra2n1c2513.2	-0.032960287	0.143620597	0.911386547	0.903140487	Potri.001G288000.2	AT2G14520.1
Potra2n1c2514.1	0.126911226	0.127566571	0.186486146	0.918630346	Potri.001G288200.1	AT3G49720.1
Potra2n1c2515.1	0.139940829	0.11118485	0.384553165	0.946668278		
Potra2n1c2516.1	-0.614739916	0.258172761	0.462000102	0.926808461		
Potra2n1c2517.1					Potri.T131500.1	AT2G14580.1
Potra2n1c2518.1	8.8435024	3.992527838	6.9276E-08	0.786005488	Potri.T131500.1	AT2G14580.1
Potra2n1c2519.1	9.234052007	3.987071846	4.87579E-06	0.760408803	Potri.001G288600.1	AT2G14610.1
Potra2n1c252.1	2.389523423	0.188522005	1.16814E-08	0.966005919	Potri.001G030600.1	AT2G22590.1
Potra2n1c2520.1	1.545832463	NA	NA	NA		
Potra2n1c2521.1	1.459962178	NA	NA	NA	Potri.001G288700.1	AT1G77180.2
Potra2n1c2522.1					Potri.001G288700.1	AT1G77180.2
Potra2n1c2523.1	0.464232578	0.224831341	0.275308565	0.742596528	Potri.001G288800.1	AT4G33740.3
Potra2n1c2524.1	0.464165636	0.221360974	0.27633089	0.752192501	Potri.001G288900.1	AT1G10020.1
Potra2n1c2525.1	2.963977803	2.787468129	NA	NA	Potri.004G039000.1	AT4G21660.1
Potra2n1c2526.1	-0.295483085	-0.192129499	0.313373896	0.910815461	Potri.T131100.1	AT4G33760.1
Potra2n1c2527.1	0.037838464	-0.661088458	0.959312932	0.733169379	Potri.001G289100.1	AT2G16270.1
Potra2n1c2528.1	-0.258049128	0.041118116	0.097934993	0.966228173	Potri.001G289200.1	AT1G50030.1
Potra2n1c2529.1	-0.491990332	0.079190639	0.007893161	0.953768643	Potri.001G289400.11	AT1G50020.1
Potra2n1c253.1	0.265233161	0.007913299	0.027722111	0.996292992	Potri.001G030750.1	AT1G29940.1
Potra2n1c2530.1	-0.536010235	-0.405789765	0.003062377	0.70570456	Potri.001G289500.1	AT4G14960.2
Potra2n1c2531.1	0.303350534	0.051183233	0.108091501	0.966917036	Potri.001G289600.1	AT4G33780.1
Potra2n1c2532.1	-1.018271781	-0.06415744	1.43267E-06	0.956889095	Potri.001G289700.1	AT1G17870.1
Potra2n1c2533.6	-0.351057213	0.125482258	0.014613781	0.918666656	Potri.001G289800.1	AT1G44910.1
Potra2n1c2534.1	-3.293078443	-3.444126063	NA	NA	Potri.001G290000.1	
Potra2n1c2535.1	-0.61910233	-0.767708831	NA	NA	Potri.009G085600.1	AT3G49780.1
Potra2n1c2536.1	-0.06562523	0.024163324	0.85186544	0.989328048	Potri.001G290100.1	AT1G50000.1
Potra2n1c2537.1	1.64774763	NA	NA	NA	Potri.001G290300.1	
Potra2n1c2538.2	-0.131777072	0.041801536	0.438673607	0.974367223	Potri.009G086100.4	AT5G14930.2
Potra2n1c2539.1	-0.649274751	1.202545617	0.414885015	0.403189027	Potri.001G291800.1	AT1G33540.1
Potra2n1c254.1	-0.164997439	-0.204393372	0.288713918	0.84487051	Potri.001G030900.3	AT3G15140.1

Potra2n1c2540.2	0.414480708	0.974322081	0.660436171	0.720242695	Potri.001G291300.1	AT1G33540.1
Potra2n1c2541.1	0.489417931	-0.045549215	0.588575358	0.994762886	Potri.001G291700.1	AT5G36180.1
Potra2n1c2542.3	-0.613093168	-0.048403916	1.53539E-06	0.971880993	Potri.009G086300.3	AT3G19640.1
Potra2n1c2543.1	2.685015931	3.269258361	NA	NA	Potri.001G292100.1	AT4G33800.1
Potra2n1c2544.1						
Potra2n1c2545.3	-0.081857642	-2.446267299	NA	NA		
Potra2n1c2546.1	1.461680898	-0.108076853	0.000287259	0.980890614	Potri.001G292300.1	AT1G70210.1
Potra2n1c2547.1	-0.752643248	0.059765851	8.63858E-05	0.971828097	Potri.009G086800.1	AT1G49960.1
Potra2n1c2548.1	0.020657029	0.086208987	0.885186807	0.923348116	Potri.001G292700.3	AT1G49950.1
Potra2n1c2549.1	NA	NA	NA	NA		
Potra2n1c255.1	1.64774763	2.134902029	NA	NA	Potri.001G031200.1	
Potra2n1c2550.1	-0.468736964	0.321333734	0.125844273	0.875861859	Potri.001G292800.1	AT2G14680.1
Potra2n1c2551.1	-0.549722346	0.697768206	0.44955441	0.839016145	Potri.009G087200.1	AT1G10200.1
Potra2n1c2552.1	-1.16555538	-0.099732262	0.051609501	0.975887373		
Potra2n1c2553.1	-0.000559106	-0.159190865	0.998299297	0.794916198	Potri.001G293100.1	AT3G49870.1
Potra2n1c2554.2	-1.251711777	0.148812648	0.005633038	0.953831244	Potri.001G293150.1	AT5G05010.1
Potra2n1c2555.1						
Potra2n1c2556.1	-0.019510321	-1.352220342	0.995536939	NA	Potri.001G293200.1	
Potra2n1c2557.1	-0.647395407	-0.181696498	0.097541517	0.941260209	Potri.001G293300.1	AT2G17150.1
Potra2n1c2558.2	1.838789024	-0.852811267	0.000605676	0.696743275	Potri.001G293400.1	AT2G14750.1
Potra2n1c2559.1	0.015600312	-0.006654217	0.968888152	0.99819408	Potri.001G293600.1	AT1G49980.1
Potra2n1c256.1	-2.161613341	0.969112335	NA	NA	Potri.001G031200.1	
Potra2n1c2560.1	-0.206209353	-0.057217502	0.208545078	0.965215635	Potri.001G293900.2	AT4G25210.1
Potra2n1c2561.1	1.170570274	0.280391119	0.358213093	0.958159215	Potri.001G294000.1	AT5G67500.1
Potra2n1c2562.1	NA	NA	NA	NA		
Potra2n1c2563.1	5.021324764	1.460816052	3.28312E-59	0.453118701	Potri.001G294100.1	AT5G67500.1
Potra2n1c2564.1	-0.258798584	-0.115020641	0.053568813	0.89108297	Potri.001G294200.1	AT2G14835.1
Potra2n1c2565.1	-0.001545106	-1.507841271	NA	NA	Potri.001G294300.2	AT4G33880.1
Potra2n1c2566.1	0.078809678	0.024538445	0.582938659	0.985313207	Potri.001G294400.1	AT2G14740.2
Potra2n1c2567.1	-2.872622301	-0.313464871	NA	NA	Potri.001G294600.1	AT3G19610.1
Potra2n1c2568.1					Potri.001G294600.1	AT3G19610.1
Potra2n1c2569.1	NA	NA	NA	NA	Potri.001G294700.1	AT3G19620.1

Potra2n1c257.1	-0.273390746	-0.00727321	0.076955028	0.996032145	Potri.001G031400.1	AT3G12280.2
Potra2n1c2570.1	NA	NA	NA	NA	Potri.001G294700.1	AT3G19620.1
Potra2n1c2571.1	-1.300423173	0.275114469	0.244988254	0.954419252	Potri.001G294800.1	AT3G19615.1
Potra2n1c2572.1	0.001307793	-0.123067231	0.994385722	0.874808359	Potri.001G294900.1	AT3G19630.1
Potra2n1c2573.1	-0.070916183	-0.160611188	0.705135334	0.859892315	Potri.001G295100.1	AT1G49910.1
Potra2n1c2574.1	-0.403156106	0.059081029	0.022389037	0.966917036	Potri.001G295200.1	AT5G40160.1
Potra2n1c2575.1	-0.21053731	-0.094328174	0.466163081	0.953831244		
Potra2n1c2576.1	0.069189834	0.368454669	0.788698509	0.762561685	Potri.009G089300.1	AT1G49970.1
Potra2n1c2577.1	0.576055457	0.049896108	0.105836398	0.985393296	Potri.001G295500.1	AT1G27730.1
Potra2n1c2578.1	1.492264199	0.89484065	0.529859516	NA		
Potra2n1c2579.1	-1.961363351	-0.773753519	7.06537E-06	0.587011639	Potri.001G295600.1	AT2G14820.1
Potra2n1c258.1	0.156561548	0.029865872	0.469955749	0.98177802	Potri.001G031500.1	AT5G22130.1
Potra2n1c2580.1	-0.650476056	-0.327509964	0.00109423	0.765464081	Potri.001G295800.1	AT1G49890.1
Potra2n1c2581.1	-2.252889997	-1.805944712	0.390847168	NA	Potri.009G089900.2	AT5G01075.1
Potra2n1c2582.1	-1.356626639	-0.366516623	0.000128986	0.830056077		
Potra2n1c2583.1	0.777330419	0.252894184	5.72384E-07	0.756757704	Potri.001G295901.1	
Potra2n1c2584.1	-0.61910233	1.185624326	NA	NA	Potri.001G296000.1	AT1G49880.1
Potra2n1c2585.2	0.97782711	0.193100223	7.77416E-14	0.814981587	Potri.001G296100.1	AT3G19553.1
Potra2n1c2586.1	0.505366049	0.099596044	0.007092583	0.937846405	Potri.001G296200.1	AT4G33890.1
Potra2n1c2587.1	0.09978252	-0.089360517	0.665049283	0.940856252	Potri.001G296400.1	AT2G14860.1
Potra2n1c2588.1	1.846296783	0.610630053	9.87661E-17	0.50924822	Potri.001G296433.1	
Potra2n1c2589.1	1.748884296	0.561365783	2.42204E-17	0.574820121	Potri.001G296500.1	AT3G43740.1
Potra2n1c259.1	0.27570871	0.211754491	0.653270834	0.955853443	Potri.001G031600.1	AT5G47530.1
Potra2n1c2590.1	-0.226295981	-0.086811968	0.669612068	0.973347974		
Potra2n1c2591.1	-0.361615158	-0.243118219	0.52104096	0.957163493	Potri.001G296700.2	AT1G49870.1
Potra2n1c2592.1	0.892634065	0.247723424	4.61151E-07	0.773157146	Potri.001G296800.1	AT4G33910.1
Potra2n1c2593.1	1.459962178	NA	NA	NA	Potri.001G296900.1	AT3G19550.1
Potra2n1c2594.2	1.263876144	0.097063137	5.51279E-06	0.966005919	Potri.001G297000.1	AT1G49850.1
Potra2n1c2595.1	0.787574825	0.06226725	0.00068918	0.938501048	Potri.001G297200.1	AT4G33920.1
Potra2n1c2596.1	-0.617697223	-0.127883491	4.7069E-05	0.903934429	Potri.001G297300.1	AT5G02860.1
Potra2n1c2597.1	-0.031333168	-0.251811906	0.921381662	0.886281281	Potri.001G297400.1	AT2G14880.1
Potra2n1c2598.1	-0.571007192	-0.537251693	0.061934432	0.349381798	Potri.001G297466.1	

Potra2n1c2599.1	0.261693757	-0.029540361	0.300720098	0.988601759	Potri.001G297500.1	AT3G19540.1
Potra2n1c26.1	1.01843835	0.334779193	2.81652E-05	0.724604666	Potri.001G003300.1	AT3G13480.1
Potra2n1c260.1	-0.002883526	0.194664819	0.983167098	0.797111689	Potri.001G031700.1	AT5G09860.1
Potra2n1c2600.1					Potri.017G109200.1	AT3G02910.1
Potra2n1c2601.1	0.632378648	0.502675471	8.33799E-06	0.231944696	Potri.001G297600.2	AT4G34280.1
Potra2n1c2602.2	-0.028775976	-0.239091731	0.921381662	0.743874478	Potri.001G297900.1	AT2G18990.1
Potra2n1c2603.1					Potri.001G298000.1	AT5G56510.1
Potra2n1c2604.1	-0.22244825	0.585509232	0.334843093	0.652831518	Potri.001G298200.1	AT2G14910.1
Potra2n1c2605.1	4.016660273	1.029459253	3.5288E-08	0.852534479	Potri.001G298300.1	AT2G14960.1
Potra2n1c2606.1	-0.226179886	0.129305779	0.040560138	0.802631112	Potri.001G298400.1	AT3G19510.1
Potra2n1c2607.1	0.062268627	-0.074488224	0.746409079	0.930203195	Potri.001G298500.1	AT4G34270.1
Potra2n1c2608.1	-0.465312424	0.303821331	0.038118689	0.846680547	Potri.001G298700.1	AT1G24625.1
Potra2n1c2609.1	-0.072085076	-0.130784717	0.670687692	0.860325259	Potri.001G298900.1	AT4G34260.1
Potra2n1c261.3	0.269444944	0.888627396	0.359681176	0.560708883	Potri.001G031800.1	AT5G06980.4
Potra2n1c2610.1	-0.845787514	-0.295484364	0.06245876	0.812696706	Potri.001G299000.1	AT4G34260.1
Potra2n1c2611.1	1.189918282	-0.635400372	0.702140076	NA	Potri.009G093900.1	AT4G34260.1
Potra2n1c2612.1	0.786176575	0.243359676	0.057030421	0.941855873	Potri.001G299200.1	AT3G19508.1
Potra2n1c2613.1	-0.269909808	-0.078271498	0.845692855	0.993589787	Potri.001G299300.1	AT3G19500.1
Potra2n1c2614.1	-0.193362311	-0.090646272	0.4907867	0.971828097	Potri.001G299500.1	AT2G15220.1
Potra2n1c2615.1	1.826701862	-0.046370319	NA	NA	Potri.001G299700.1	AT1G49820.1
Potra2n1c2616.1	0.73862675	-0.046370319	NA	NA	Potri.001G299600.1	AT2G15220.1
Potra2n1c2617.1	NA	NA	NA	NA	Potri.001G299800.1	AT1G49800.1
Potra2n1c2618.1					Potri.001G299900.2	AT1G49800.1
Potra2n1c2619.1	-3.507256421	-2.225653174	0.26418413	NA	Potri.001G300000.1	AT4G34230.1
Potra2n1c262.1					Potri.001G031900.2	AT5G06990.1
Potra2n1c2620.1	-0.001229198	-0.039426221	0.995126097	0.974785573	Potri.001G300100.1	AT2G15240.1
Potra2n1c2621.1	1.249347447	-0.739732158	0.187663018	NA	Potri.001G300200.1	AT4G37260.1
Potra2n1c2622.2	0.207986322	-0.035808505	0.135909976	0.964971544	Potri.001G300400.2	AT3G19460.1
Potra2n1c2623.1	0.545886083	-0.094456954	0.132688132	0.971828097		
Potra2n1c2624.2	0.062879194	-0.023809968	0.774498028	0.985393296	Potri.001G300500.1	AT2G15290.1
Potra2n1c2625.1	-0.690771472	-0.165255223	0.04985019	0.945254452	Potri.001G300700.1	AT4G34220.1
Potra2n1c2626.1	-0.932466494	-0.161354557	0.000423395	0.936170918	Potri.001G300800.2	AT4G34215.2

Potra2n1c2627.1	0.048985988	-0.24689792	0.869082187	0.860810425	Potri.001G300900.1	AT4G34200.1
Potra2n1c2628.1	1.784833123	0.073693582	5.22381E-06	0.984373337	Potri.001G301000.1	AT3G19490.1
Potra2n1c2629.1	0.108671628	0.19541309	0.633215224	0.877832902	Potri.001G301200.1	AT3G62290.1
Potra2n1c263.1	-0.07778475	0.130169799	0.646088151	0.848131816	Potri.001G032000.1	AT1G74320.1
Potra2n1c2630.1	-2.345090547	-0.482293721	0.114279783	0.935851032	Potri.009G097000.1	AT2G01640.2
Potra2n1c2631.1	-0.182358282	0.134080364	0.538765922	0.919424623	Potri.001G301400.1	AT2G15320.1
Potra2n1c2632.2	0.063743874	0.133944395	0.655175107	0.814001428	Potri.001G301600.2	AT4G35220.1
Potra2n1c2633.1	-1.032559653	-0.496883457	0.011364328	0.779493734	Potri.001G301800.1	AT4G34160.1
Potra2n1c2634.1	1.30990827	0.337371596	8.32521E-18	0.595446346	Potri.001G301900.1	AT4G34150.1
Potra2n1c2635.1	-0.056016933	-0.026943294	0.534400849	0.978251387	Potri.001G302000.1	AT3G19420.1
Potra2n1c2636.1	-0.461796795	-0.123246591	0.006394277	0.937846405	Potri.001G302100.2	AT1G47128.1
Potra2n1c2637.1	0.48750283	-0.090928635	0.275088418	0.978428876	Potri.001G302500.1	AT4G06744.1
Potra2n1c2638.1	-0.807441599	-0.058151444	0.000768046	0.966917036	Potri.001G302600.1	AT5G67210.1
Potra2n1c2639.1	-1.515776215	0.399965935	0.001043379	0.861203076		
Potra2n1c264.1	0.505452924	2.303565945	NA	NA	Potri.001G032100.1	AT3G28050.1
Potra2n1c2640.1	0.007821869	-2.514137983	NA	NA	Potri.005G009000.1	
Potra2n1c2641.1	0.22162612	0.371927016	0.517152101	0.613589951		
Potra2n1c2642.2	-3.322256847	0.061492668	NA	NA	Potri.019G012300.3	AT2G37690.1
Potra2n1c2643.1					Potri.001G155750.1	AT1G21280.1
Potra2n1c2644.1	-0.124740298	-0.09520327	0.537936145	0.923397137	Potri.001G303900.1	AT4G36910.1
Potra2n1c2645.1	0.3969114	0.086297563	0.002579989	0.935474933	Potri.001G304000.1	AT1G49760.1
Potra2n1c2646.1	0.304214576	0.219160912	0.007412685	0.704368358	Potri.001G304100.1	AT4G34100.2
Potra2n1c2647.1	-0.733408913	-0.228924718	0.01949949	0.911094206	Potri.001G304200.1	AT3G19370.3
Potra2n1c2648.1	0.236146059	-0.072725844	0.111385628	0.950878725	Potri.001G304600.1	AT5G53390.1
Potra2n1c2649.1	-0.079030703	-0.31539449	0.777618386	0.836066102	Potri.001G304800.1	AT4G34050.1
Potra2n1c265.1	-1.864134742	-0.572581169	NA	NA	Potri.001G032300.2	AT5G07040.1
Potra2n1c2650.1	0.42181006	-0.02918328	0.005209317	0.986636967	Potri.001G304700.1	AT5G18380.1
Potra2n1c2651.1	0.008433812	-0.008214595	0.978672661	0.996354253	Potri.009G100000.2	
Potra2n1c2652.1	-0.112756126	0.099641351	0.560110009	0.937146435	Potri.001G304900.1	AT4G34040.1
Potra2n1c2653.1	0.08750578	0.228566581	0.38540213	0.775519829	Potri.001G304900.1	AT4G34040.1
Potra2n1c2654.1	2.735991243	1.879759176	NA	NA	Potri.001G305100.2	AT1G49770.1
Potra2n1c2655.3	-0.409564622	-0.085947849	0.000243386	0.912777843	Potri.001G305300.1	AT5G08650.1

Potra2n1c2656.1	0.901078828	1.24575515	0.813360459	NA	Potri.001G305400.1	AT2G38290.1
Potra2n1c2657.1	-0.565318691	-0.283808694	0.014094397	0.733975197	Potri.001G305500.1	AT3G21060.1
Potra2n1c2658.1	0.057856275	-0.276163963	0.769479019	0.77013803	Potri.001G305600.1	AT4G24290.2
Potra2n1c2659.1	0.051308824	0.033405924	0.708927082	0.9761362	Potri.001G305700.2	AT2G21120.1
Potra2n1c266.1	9.383653991	6.478691931	3.8679E-70	0.035841202	Potri.001G032400.1	AT5G16080.1
Potra2n1c2660.1	0.210573465	0.143238576	0.0445824	0.901195443	Potri.001G305800.1	AT1G34320.1
Potra2n1c2661.1	-0.424748085	0.02496539	0.003437676	0.98838468	Potri.001G305900.1	AT5G63700.1
Potra2n1c2662.1	-1.430564854	-0.196541394	3.96924E-06	0.895460013	Potri.001G306000.1	AT5G63710.1
Potra2n1c2663.1	-0.386157199	-0.00232509	0.000404945	0.999325699	Potri.001G306100.1	AT5G13340.1
Potra2n1c2664.1	-0.777338818	-0.296833593	0.031003111	0.895474078	Potri.001G306200.1	AT2G03200.1
Potra2n1c2665.1	-1.915623002	-0.33937932	2.203E-09	0.817452127	Potri.001G306300.1	AT5G53590.1
Potra2n1c2666.1	0.218932908	0.442723317	0.377818528	0.727425783	Potri.001G306500.1	AT5G64730.1
Potra2n1c2667.1	NA	NA	NA	NA	Potri.001G306600.1	AT4G39340.1
Potra2n1c2668.1	-1.473602576	-1.367645553	0.00541152	0.077114891		
Potra2n1c2669.1	-1.182325483	-0.932519303	0.001385318	0.583548322	Potri.001G306866.1	
Potra2n1c267.1	-1.928889824	-0.308992457	1.63582E-20	0.624602712	Potri.001G032500.1	AT5G07050.1
Potra2n1c2670.1	-1.489525167	-0.767948744	3.63459E-10	0.186273003	Potri.001G307200.1	AT4G39330.1
Potra2n1c2671.1	-0.368755151	-1.421664775	0.51851035	0.34684536	Potri.001G307300.1	AT5G17680.1
Potra2n1c2672.1	0.035411388	0.000284779	0.798056147	0.999937671	Potri.001G307400.1	AT1G05520.1
Potra2n1c2673.3	1.060613831	0.505362698	0.204782479	0.912079684	Potri.001G307500.1	AT5G05320.1
Potra2n1c2674.1	0.081227893	0.315627317	0.703552858	0.802631112	Potri.001G307600.1	AT5G09850.1
Potra2n1c2675.1	0.267596607	-0.007572744	0.111839875	0.997065289	Potri.001G307700.1	AT5G09840.1
Potra2n1c2676.1	2.002083092	0.79548721	0.012183514	0.910815461	Potri.001G308200.1	AT3G25830.1
Potra2n1c2677.1	2.492428534	2.86450839	NA	NA	Potri.001G308300.1	AT3G25820.1
Potra2n1c2678.1	1.832252342	0.21603331	9.05165E-27	0.828625141	Potri.001G308600.1	AT1G07650.1
Potra2n1c2679.1	2.131232428	0.409600241	5.14392E-18	0.759797728		
Potra2n1c268.1	-0.817690551	-0.121597566	0.000197895	0.939653927	Potri.003G192400.2	AT2G38720.1
Potra2n1c2680.1	-1.492077085	-0.217529029	0.139219394	0.962820058	Potri.019G008000.2	AT1G29670.1
Potra2n1c2681.1	0.365606037	-1.642075045	0.847469672	0.78423153	Potri.001G308800.1	
Potra2n1c2682.1	0.272563051	0.327244554	0.017133407	0.32064055	Potri.001G308900.1	AT2G18465.1
Potra2n1c2683.1	-2.67131154	-0.739393912	0.007724898	0.788065047	Potri.001G309000.1	AT3G18800.1
Potra2n1c2684.1	0.538515443	0.229232365	0.084352003	0.874496903	Potri.001G309100.1	AT1G76890.2

Potra2n1c2685.1	-0.01442377	0.033702406	0.935481419	0.970442612	Potri.001G309200.1	AT5G09830.1
Potra2n1c2686.1	-0.315278987	0.06522507	0.383937384	0.974275337	Potri.001G309300.1	AT5G09820.2
Potra2n1c2687.1	-0.389463989	-0.053036637	6.13798E-05	0.948486101	Potri.001G309500.1	AT5G09810.1
Potra2n1c2688.1	-0.325299346	0.196090534	0.716665968	0.951734262	Potri.001G309600.1	AT1G49230.1
Potra2n1c2689.1	-1.301135313	0.055735295	0.006778468	0.983113084	Potri.001G309700.1	AT1G49230.1
Potra2n1c269.1	0.274000978	0.265758199	0.413296857	0.811823756	Potri.001G032800.1	AT2G26710.1
Potra2n1c2690.1	0.090237747	0.166059772	0.661185747	0.895560236	Potri.001G309800.1	AT3G18770.1
Potra2n1c2691.1	-1.922767854	-0.641794843	NA	NA	Potri.001G309900.1	AT1G17860.1
Potra2n1c2692.1	-0.92514695	-0.327612558	6.05686E-08	0.719713755	Potri.001G310000.1	AT3G26040.1
Potra2n1c2693.1	0.484826835	0.007603298	0.22342749	0.997164503	Potri.017G050100.3	AT4G01020.1
Potra2n1c2694.1	2.492428534	NA	NA	NA	Potri.001G310300.1	
Potra2n1c2695.1	1.92172847	0.395460996	7.11612E-10	0.724604666	Potri.001G310500.1	AT5G64300.1
Potra2n1c2696.1	3.085103319	-0.47878274	0.183534304	NA	Potri.001G310600.1	AT5G64290.1
Potra2n1c2697.1	0.573628451	-2.446267299	0.885186807	NA	Potri.001G310700.1	AT2G02220.1
Potra2n1c2698.1	-0.056296736	-0.014222848	0.599877986	0.989304496	Potri.001G310900.1	AT5G64270.1
Potra2n1c2699.1	0.469548184	0.136288323	0.00212327	0.896306549	Potri.001G311000.1	AT5G10360.1
Potra2n1c27.1	1.467534728	0.347794194	2.09362E-05	0.855121771	Potri.001G003200.1	AT4G04220.1
Potra2n1c270.1	-0.277771785	-0.014285503	0.083719418	0.990927357	Potri.001G032900.1	AT3G12490.2
Potra2n1c2700.1	NA	NA	NA	NA	Potri.001G311100.1	AT2G44710.1
Potra2n1c2701.1	1.459962178	2.676364087	NA	NA	Potri.001G311201.1	AT2G44710.1
Potra2n1c2702.1						
Potra2n1c2703.1	NA	NA	NA	NA	Potri.001G311400.1	AT1G60600.2
Potra2n1c2704.1	5.462871787	3.039104336	0.000215358	0.240141349	Potri.001G311300.1	AT1G17840.1
Potra2n1c2705.1	NA	NA	NA	NA	Potri.008G022500.1	
Potra2n1c2706.1	6.431511286	4.506136326	6.06533E-12	0.012563109	Potri.001G311300.1	AT1G17840.1
Potra2n1c2707.1					Potri.001G311300.1	AT1G17840.1
Potra2n1c2708.1	0.75992438	0.317080482	0.001013601	0.595538573	Potri.001G311600.1	AT5G65260.1
Potra2n1c2709.1	1.364512079	0.119928708	7.02281E-05	0.937276488	Potri.001G311700.1	AT5G64260.1
Potra2n1c271.1	0.074817178	0.078783296	0.71837676	0.930871994	Potri.001G033200.1	AT3G12480.1
Potra2n1c2710.1					Potri.003G087350.1	
Potra2n1c2711.1					Potri.001G312500.1	AT1G22360.1
Potra2n1c2712.1					Potri.017G052300.1	AT1G22360.1

Potra2n1c2713.1					Potri.001G312600.1	AT1G22360.1
Potra2n1c2714.1	NA	1.571529889	NA	NA	Potri.001G312500.1	AT1G22360.1
Potra2n1c2715.1	NA	NA	NA	NA	Potri.006G172300.1	
Potra2n1c2716.1					Potri.001G312600.1	AT1G22360.1
Potra2n1c2717.1	3.172094397	1.071418418	8.33673E-12	0.742848888	Potri.001G312800.1	AT1G33540.1
Potra2n1c2718.1	-0.10146472	-0.064734307	0.389034834	0.940735643	Potri.001G312900.1	AT1G22310.2
Potra2n1c2719.1	6.414181184	1.571529889	0.000545566	NA	Potri.001G313000.1	AT1G22360.1
Potra2n1c272.1	-0.850025554	-0.937131104	0.539186072	0.857374454	Potri.001G033400.1	AT1G15780.1
Potra2n1c2720.1	2.492463366	1.355796624	0.000118123	0.606364174	Potri.017G053200.1	AT5G64230.1
Potra2n1c2721.1	-0.500087671	0.595954135	0.71974224	0.930448915		
Potra2n1c2722.1					Potri.001G313100.1	AT5G09430.1
Potra2n1c2723.1	0.086870267	0.108846992	0.751704529	0.937687311	Potri.001G313301.1	AT5G63020.1
Potra2n1c2724.1					Potri.009G039800.2	AT3G54040.1
Potra2n1c2725.1	-1.35927387	2.815161303	NA	NA	Potri.001G313500.1	AT1G80580.1
Potra2n1c2726.1	-0.038162429	-0.069656041	0.861509285	0.958255161	Potri.001G313900.1	AT1G15800.1
Potra2n1c2727.1	-0.014935145	-0.078553513	0.90253824	0.902626948	Potri.001G314000.4	AT5G48820.1
Potra2n1c2728.1	1.121579529	0.499801797	8.3487E-08	0.61651277	Potri.001G314200.1	AT2G32720.1
Potra2n1c2729.1	0.05192979	-0.007049554	0.623114753	0.993332869	Potri.001G314300.1	AT2G32730.1
Potra2n1c273.1	-0.301877343	0.429186715	0.447300884	0.673131445	Potri.001G033500.3	
Potra2n1c2730.1	NA	NA	NA	NA	Potri.001G314400.1	AT3G24140.1
Potra2n1c2731.1	NA	NA	NA	NA		
Potra2n1c2732.1	0.717836742	0.201785103	6.9638E-05	0.874808359	Potri.001G314500.1	AT5G48760.2
Potra2n1c2733.1	-0.065955695	0.101626122	0.845296664	0.942409925	Potri.001G314700.1	AT1G04900.1
Potra2n1c2734.3	-0.839709776	-0.175389636	2.68439E-09	0.736429196	Potri.001G314800.1	AT4G13640.1
Potra2n1c2735.1	0.414139022	-0.082375287	0.643809959	0.98876314	Potri.017G055000.1	AT2G32800.1
Potra2n1c2736.1	0.691310946	-0.185400787	0.181244203	0.956631493	Potri.001G315200.10	AT1G04880.1
Potra2n1c2737.1	NA	NA	NA	NA	Potri.001G315300.1	AT4G13620.1
Potra2n1c2738.1	-0.524478602	-0.145228972	0.038968501	0.922011871		
Potra2n1c2739.1	0.203188763	0.821960029	NA	NA	Potri.001G315500.1	AT2G30810.1
Potra2n1c274.1	-0.490426742	-0.255747413	1.31342E-05	0.607487588	Potri.001G033600.1	AT2G26920.1
Potra2n1c2740.1	0.001750192	0.002098012	0.991867476	0.998438878	Potri.001G315600.1	AT1G04860.1
Potra2n1c2741.1	0.245170784	-1.465929376	NA	NA	Potri.001G315700.1	AT4G13600.1

Potra2n1c2742.1	0.015034235	-0.040787045	0.930760713	0.9761362	Potri.001G315800.1	AT3G25040.1
Potra2n1c2743.1	0.104497474	0.0619903	0.850530623	0.985393296		
Potra2n1c2744.1	0.398176923	0.096454075	0.033629867	0.941424651	Potri.001G315950.1	AT2G04235.1
Potra2n1c2745.1	1.64774763	1.571529889	NA	NA	Potri.001G316200.1	AT3G01320.1
Potra2n1c2746.1	2.075688017	1.321685333	0.001112373	0.349381798	Potri.001G316300.1	AT3G01320.1
Potra2n1c2747.1	0.119460866	0.139373485	0.486717428	0.895474078	Potri.001G316400.1	AT1G04850.1
Potra2n1c2748.1	0.169022472	-0.219133808	0.628262763	0.899757743	Potri.001G316500.1	AT1G04840.1
Potra2n1c2749.1	0.227606429	0.112199425	0.121855406	0.855193394	Potri.001G316700.2	AT4G13730.1
Potra2n1c275.1	2.270603348	0.261351246	2.95497E-07	0.97318201	Potri.001G033800.1	AT2G03220.1
Potra2n1c2750.1	-0.124972791	-0.108399339	0.275245348	0.874496903	Potri.001G316800.1	AT1G04910.1
Potra2n1c2751.2	2.905002388	-0.042515609	0.003213226	NA	Potri.001G317001.1	AT4G04980.1
Potra2n1c2752.1	0.329441422	0.242814716	0.003384646	0.843683654	Potri.001G317200.1	AT4G13750.1
Potra2n1c2753.1	0.102809272	0.013808088	0.522501041	0.989708171	Potri.001G317400.1	AT4G22330.1
Potra2n1c2754.1	0.290799062	0.005173148	0.144265854	0.99702095	Potri.001G317300.1	AT5G49210.2
Potra2n1c2755.2	0.036708052	0.042425848	0.765710478	0.970011758	Potri.001G317500.2	AT4G13780.1
Potra2n1c2756.2	-1.298029077	-0.218405186	0.58149704	0.978646026	Potri.001G317600.1	AT1G04920.1
Potra2n1c2757.1	-0.189857123	-0.172591224	0.171583222	0.922993749	Potri.001G317700.1	AT2G31740.1
Potra2n1c2758.1	0.373297354	0.40355965	0.054088661	0.319291077	Potri.001G317900.1	AT3G23910.1
Potra2n1c2759.1	-0.107767493	0.313925381	0.645108257	0.660818471	Potri.001G318100.1	AT3G42860.1
Potra2n1c276.1	-0.181799552	0.109888461	0.248028279	0.953831244	Potri.001G033900.3	AT2G43190.2
Potra2n1c2760.1	1.894788419	-1.507841271	0.502097378	NA	Potri.001G318300.1	AT3G06240.1
Potra2n1c2761.1	7.815495402	3.34472007	2.00686E-11	NA	Potri.001G318500.1	AT2G32830.1
Potra2n1c2762.1					Potri.001G318600.1	
Potra2n1c2763.1						
Potra2n1c2764.2	0.064851921	0.226705393	0.700189778	0.788065047	Potri.001G318700.1	AT1G71900.1
Potra2n1c2765.1	0.091041334	0.29228792	0.629177801	0.681738793	Potri.017G058300.1	AT3G23900.3
Potra2n1c2766.1	0.210057758	0.200267533	0.042076329	0.86915032	Potri.001G318800.2	AT4G30990.1
Potra2n1c2767.1	0.122949023	0.249116707	0.318839646	0.704224102	Potri.017G058300.1	AT3G23900.3
Potra2n1c2768.1	-0.692034428	-0.326381409	4.31002E-05	0.727843918	Potri.001G319100.1	AT4G13830.2
Potra2n1c2769.1	-2.660319131	-0.379491343	0.029919796	0.959881296	Potri.001G319200.1	AT4G13840.1
Potra2n1c277.1	NA	NA	NA	NA	Potri.001G034000.1	AT1G14110.1
Potra2n1c2770.1	NA	2.146414233	NA	NA		

Potra2n1c2771.1	-1.956134842	-3.320201509	0.314696144	NA		
Potra2n1c2772.1	-0.016081815	0.121974111	0.944383978	0.886633917	Potri.001G319500.1	AT5G43490.1
Potra2n1c2773.1	0.319488398	0.181107523	0.046025353	0.874808359	Potri.001G319600.1	AT5G43490.1
Potra2n1c2774.1	0.493963334	0.041984928	0.002072365	0.98177802	Potri.T162000.1	AT4G13850.1
Potra2n1c2775.1	1.137718363	0.516751135	0.001025508	0.684835668	Potri.001G320000.1	AT3G23820.1
Potra2n1c2776.1	0.259391177	0.13899535	0.229600152	0.900551258	Potri.001G320201.1	AT4G13870.2
Potra2n1c2777.1	-0.15787417	-0.256204273	0.490952944	0.859892315	Potri.001G320300.1	AT2G31970.1
Potra2n1c2778.2	-0.164838149	-0.261724842	0.472915556	0.857189843	Potri.001G320400.2	AT4G13930.1
Potra2n1c2779.1	-0.279913211	-0.487342312	0.265251049	0.675169873	Potri.001G320500.1	AT4G13940.1
Potra2n1c278.1	2.735991243	4.666003791	NA	NA	Potri.001G034000.1	AT1G14110.1
Potra2n1c2780.1	0.193435037	0.198935032	0.920914494	NA		
Potra2n1c2781.1	-1.022866933	-0.531447885	0.031229354	0.815119099	Potri.001G320700.1	AT3G23805.1
Potra2n1c2782.1	-1.350365396	-0.858738529	1.34115E-09	0.326889386	Potri.001G320900.1	AT4G13980.1
Potra2n1c2783.1	0.340522075	-0.106234417	0.1678122	0.966501596	Potri.001G321000.1	AT4G13990.1
Potra2n1c2784.1	0.647277433	0.210383858	0.000592713	0.922011871	Potri.001G321100.2	AT4G14000.1
Potra2n1c2785.1	NA	NA	NA	NA	Potri.001G321200.1	
Potra2n1c2786.1	-0.430451117	-0.208049676	0.119400391	0.857374454	Potri.001G321300.2	AT4G14010.1
Potra2n1c2787.1	-0.804628519	-0.580232372	0.179788473	0.82104194	Potri.001G321500.1	AT4G14010.1
Potra2n1c2788.1	-0.148368526	-0.104669107	0.692960759	0.959881296	Potri.001G321600.1	AT4G14030.1
Potra2n1c2789.1	-0.067490721	-0.460018013	0.967316784	NA		
Potra2n1c279.1	6.391077966	3.021780406	1.14713E-06	NA	Potri.001G034100.1	AT2G03220.1
Potra2n1c2790.1	-1.149375663	0.020641838	0.0001218	0.995005375	Potri.001G321800.1	AT3G23790.1
Potra2n1c2791.1					Potri.001G321900.1	AT3G23770.1
Potra2n1c2792.1	0.317175135	0.09457246	0.313151713	0.97601212	Potri.001G322100.1	AT3G26782.1
Potra2n1c2793.1	0.221119222	0.371471471	0.815531558	0.937789963	Potri.001G322200.1	AT3G22790.1
Potra2n1c2794.1	0.488727819	0.080106856	0.000102352	0.943483421	Potri.001G322300.1	AT5G14040.1
Potra2n1c2795.1	-0.030443917	-0.059467119	0.862888888	0.956865545	Potri.001G322500.1	AT1G12840.1
Potra2n1c2796.1	-1.429639781	-0.231088898	8.60365E-09	0.901671593	Potri.001G322700.1	AT3G26790.1
Potra2n1c2797.1					Potri.001G322800.1	
Potra2n1c2798.1	-0.003089114	0.109300547	0.98838652	0.874808359	Potri.001G322900.1	AT3G18150.1
Potra2n1c2799.1	0.149249512	0.049816245	0.567655035	0.975578749	Potri.001G323000.1	AT1G12830.1
Potra2n1c28.2	-0.211868339	0.173862648	0.16670248	0.788065047	Potri.001G003400.1	AT4G09830.1

Potra2n1c280.1	-0.148725668	-0.134049708	0.272038261	0.852534479	Potri.001G034200.1	AT3G12400.1
Potra2n1c2800.2	-0.073520267	0.07937419	0.667605677	0.953608209	Potri.001G323100.1	AT3G26810.1
Potra2n1c2801.2	-0.37982179	-0.052003475	0.045845887	0.978725627	Potri.017G061700.1	
Potra2n1c2802.1	2.652345515	-1.765386957	0.101734763	NA	Potri.001G323300.1	AT5G13200.1
Potra2n1c2803.1	0.001175441	0.079402948	0.993503802	0.953831244	Potri.001G323400.4	AT5G14030.3
Potra2n1c2804.1						
Potra2n1c2805.1	5.605782114	3.255194335	NA	NA	Potri.001G323500.2	AT2G33500.2
Potra2n1c2806.1	-0.350201681	-0.731120019	0.715023574	0.870321018	Potri.001G323600.1	AT5G14020.1
Potra2n1c2807.1	-1.041972662	-0.723532816	6.34029E-07	0.394823746	Potri.001G323700.1	AT4G15620.1
Potra2n1c2808.1	NA	NA	NA	NA	Potri.016G106400.1	
Potra2n1c2809.1					Potri.016G106400.1	
Potra2n1c281.1	0.157151316	0.056997451	0.260579805	0.959881296	Potri.001G034300.1	AT1G65440.2
Potra2n1c2810.1	NA	NA	NA	NA		
Potra2n1c2811.1	-1.555910594	2.43111789	NA	NA	Potri.001G324000.1	AT5G41130.1
Potra2n1c2812.1	1.834017095	4.401354684	0.478894762	0.205878227	Potri.001G324000.1	AT5G41130.1
Potra2n1c2813.1	2.409567423	NA	NA	NA	Potri.004G170400.1	AT3G14470.1
Potra2n1c2814.1	-0.18486223	0.907912793	0.559454053	0.47454828	Potri.001G324200.1	AT5G41120.1
Potra2n1c2815.1	-0.161870694	-0.08894084	0.417865981	0.962120332	Potri.001G324300.1	AT5G64670.1
Potra2n1c2816.1	NA	NA	NA	NA	Potri.010G022900.1	
Potra2n1c2817.1	0.502150163	-0.013776204	0.013882232	0.991407187	Potri.001G324400.1	AT5G41130.1
Potra2n1c2818.1	NA	1.571529889	NA	NA	Potri.004G072100.1	AT5G62650.1
Potra2n1c2819.1	7.205440549	3.281041337	1.59992E-59	0.033225381	Potri.001G324500.1	AT4G18930.1
Potra2n1c282.1	0.204666414	-0.11519986	0.184725366	0.894138111	Potri.003G190800.2	AT3G12390.1
Potra2n1c2820.1	-2.056249835	-4.465146349	0.512939662	NA	Potri.001G324600.1	AT5G14010.1
Potra2n1c2821.1	-0.270498016	0.654384211	0.144614143	0.526356391	Potri.001G324700.2	AT3G26890.3
Potra2n1c2822.1	0.161382993	0.118851053	0.224690972	0.821704644	Potri.001G324800.1	AT2G33490.1
Potra2n1c2823.1	NA	1.381953902	NA	NA	Potri.001G324900.3	
Potra2n1c2824.2	0.017266671	0.017385829	0.938859078	0.987891912		
Potra2n1c2825.1	1.991169479	0.543581199	8.01233E-15	0.346327696	Potri.001G325100.1	AT5G13180.1
Potra2n1c2826.1	0.009683892	0.451452308	0.987935873	0.841517891	Potri.001G325200.1	AT3G02040.1
Potra2n1c2827.1	-0.449608919	-0.167471494	0.000108496	0.749684992	Potri.001G325300.1	AT5G41070.1
Potra2n1c2828.1	NA	1.571529889	NA	NA	Potri.017G067900.1	AT5G18610.2

Potra2n1c2829.1	0.203339306	-0.111466826	0.321534763	0.936549944	Potri.001G325500.1	AT3G26950.1
Potra2n1c283.1	-0.619468414	-0.303634132	0.033053962	0.817452127	Potri.001G034500.1	AT5G07900.1
Potra2n1c2830.3	-0.414525891	-0.142606556	0.00441583	0.806746126	Potri.001G325700.1	AT3G26990.1
Potra2n1c2831.1						
Potra2n1c2832.1	-1.991638968	-1.285278257	0.000188536	0.202843914	Potri.001G325800.1	AT3G02000.1
Potra2n1c2833.1	NA	0.674963689	NA	NA		
Potra2n1c2834.1	-0.348366742	-0.124856794	0.000369465	0.830470159	Potri.001G325900.2	AT3G26980.1
Potra2n1c2835.1	-2.286316826	-0.997099362	NA	NA	Potri.001G326000.1	AT1G66350.1
Potra2n1c2836.1	1.307202636	2.215583023	NA	NA	Potri.001G326100.1	AT4G23882.1
Potra2n1c2837.1	-0.115709801	-0.054330709	0.724878777	0.9761362	Potri.001G326200.1	AT5G41050.1
Potra2n1c2838.1	-0.06834094	0.164884481	0.902084442	0.924251575	Potri.001G326300.1	AT5G41040.1
Potra2n1c2839.1	0.303758409	-0.045262355	0.193407786	0.981772014	Potri.001G326400.1	AT3G62870.1
Potra2n1c284.1	0.661401372	0.566392972	0.000717866	0.428801064	Potri.001G034600.1	AT5G07900.1
Potra2n1c2840.1	-0.131504016	0.004546946	0.238152481	0.996032145	Potri.001G326500.2	AT1G24300.1
Potra2n1c2841.1	0.00867362	-0.029472765	0.952148786	0.981726976	Potri.001G326600.2	AT3G27000.1
Potra2n1c2842.1	-0.439563747	-0.284392084	0.754076017	0.966759357		
Potra2n1c2843.1	-0.092084103	0.087895512	0.43041104	0.87679119	Potri.001G326700.2	AT1G24300.1
Potra2n1c2844.1	-0.210973622	0.067859598	0.629177801	0.974367223	Potri.001G326900.1	AT5G22900.1
Potra2n1c2845.2	-0.163866074	-0.100618614	0.660565091	0.956610193	Potri.001G326800.1	AT5G14080.1
Potra2n1c2846.1	-0.713159286	0.193875962	0.000629852	0.909771714	Potri.001G327000.2	AT3G01990.1
Potra2n1c2847.2	-0.473027473	0.170686065	0.003348132	0.821709751	Potri.001G327100.1	AT3G27010.1
Potra2n1c2848.1	-0.540934832	-0.137556295	0.000469678	0.900869139	Potri.001G327300.1	AT3G27020.1
Potra2n1c2849.1	0.3400803	0.608194181	0.002012562	0.316032926	Potri.001G327400.1	
Potra2n1c285.1	0.162208499	-0.152897223	0.468384075	0.912777843	Potri.001G034800.1	
Potra2n1c2850.1	0.470115288	0.620204522	0.023510249	0.443657809		
Potra2n1c2851.2	0.269238818	0.65013499	0.345814223	0.306363984	Potri.001G327500.2	AT5G14090.1
Potra2n1c2852.1					Potri.001G327700.1	AT1G30890.2
Potra2n1c2853.1	0.442002157	-0.255507406	0.04817367	0.860810425	Potri.001G327800.1	AT3G01980.4
Potra2n1c2854.1	6.821450029	2.461401969	1.07662E-11	0.79078676	Potri.001G328000.2	AT5G13080.1
Potra2n1c2855.1	0.20813834	-0.028397867	0.137070176	0.981772014	Potri.T043700.1	AT5G14105.1
Potra2n1c2856.1	NA	NA	NA	NA		
Potra2n1c2857.1	0.73862675	-0.767708831	NA	NA		

Potra2n1c2858.1	-0.61910233	-0.767708831	NA	NA	Potri.001G328200.1	
Potra2n1c2859.1	-3.264817922	0.2181339	NA	NA	Potri.001G328300.1	AT3G01960.1
Potra2n1c286.1	-0.013336755	-0.158055173	0.947244323	0.850863374	Potri.001G034700.1	AT5G07900.1
Potra2n1c2860.1	-0.423703931	0.615568082	0.107030494	0.549295993	Potri.001G328600.2	AT5G60910.1
Potra2n1c2861.2	-0.490212458	0.530957459	0.066345452	0.636664382		
Potra2n1c2862.1	-2.522655216	0.325944567	0.012316155	0.943685779		
Potra2n1c2863.1	NA	0.674963689	NA	NA		
Potra2n1c2864.1	-2.983195238	-0.841569523	NA	NA		
Potra2n1c2865.1	0.350327772	0.693466656	0.70260292	0.832664059	Potri.001G328700.1	AT3G27027.1
Potra2n1c2866.1	0.149138598	0.322091089	0.576593343	0.791848264	Potri.001G328800.1	AT3G27030.1
Potra2n1c2867.1	0.145128431	0.342150657	0.576461845	0.758098467		
Potra2n1c2868.1	0.328276701	0.555112349	0.266608039	0.724604666	Potri.001G329000.1	AT5G08391.1
Potra2n1c2869.1	0.328276701	0.555112349	0.266608039	0.724604666	Potri.017G064400.1	AT5G40960.1
Potra2n1c287.1	0.521684674	0.585359721	0.138354087	0.333044935	Potri.001G034900.2	AT1G21150.1
Potra2n1c2870.1	-1.336737103	-1.4850079	NA	NA		
Potra2n1c2871.1	0.159674829	-0.144869742	0.486184629	0.921099436	Potri.001G329100.1	AT3G01930.2
Potra2n1c2872.1	NA	NA	NA	NA	Potri.001G329200.1	AT5G14130.1
Potra2n1c2873.1	0.118031559	-0.068076959	0.396307806	0.937687311	Potri.001G329300.1	AT5G14140.1
Potra2n1c2874.1	0.006953401	-0.113130898	0.966893576	0.869365831	Potri.001G329500.1	AT5G40950.1
Potra2n1c2875.1	-0.072106917	-0.009870689	0.765890871	0.995565508	Potri.001G329600.1	AT3G27050.1
Potra2n1c2876.1	-0.069015722	-0.18836192	0.946496496	0.975578749		
Potra2n1c2877.1	-0.043690593	-0.22475825	0.823625679	0.811742964	Potri.001G329700.1	AT3G27060.1
Potra2n1c2878.1	NA	0.674963689	NA	NA	Potri.001G329800.1	AT5G55690.1
Potra2n1c2879.1	0.032019757	-0.133091667	0.925672737	0.915524068	Potri.001G329900.1	AT1G60550.1
Potra2n1c288.1	0.420808247	0.198939911	0.022348758	0.885610409	Potri.003G190400.1	AT5G07900.1
Potra2n1c2880.5	-0.281537499	-0.001447363	0.027435204	0.999325699	Potri.001G330000.1	AT3G48200.1
Potra2n1c2881.1	-0.02982535	-0.042738785	0.84868287	0.966228173	Potri.001G330200.1	AT1G27390.1
Potra2n1c2882.1	0.063765159	0.075915202	0.591892316	0.939915707	Potri.001G330300.2	AT3G27090.1
Potra2n1c2883.1	0.049146739	0.047256168	0.690293198	0.953831244	Potri.001G330400.1	AT3G27100.1
Potra2n1c2884.1	-0.453573839	-0.241794039	1.62777E-07	0.71703421	Potri.001G330501.1	AT1G64940.1
Potra2n1c2885.1					Potri.001G330600.1	AT1G15690.2
Potra2n1c2886.1	-0.525649109	-0.018869493	0.005959064	0.987288338	Potri.001G330800.1	AT3G27110.2

Potra2n1c2887.1	-0.026175922	0.031471539	0.83776794	0.96820892	Potri.001G330900.2	AT3G01910.1
Potra2n1c2888.1	-0.002369505	-0.548665243	0.996783495	0.663037599	Potri.001G331200.1	AT3G27120.1
Potra2n1c2889.1	-1.348357988	-1.265272616	0.006669918	0.063996679	Potri.001G331100.1	AT3G01900.1
Potra2n1c289.1	0.10508184	-0.109472353	0.728660054	0.947969749	Potri.001G034900.2	AT1G21150.1
Potra2n1c2890.1	1.64774763	NA	NA	NA	Potri.001G331300.2	AT3G27120.1
Potra2n1c2891.1	-0.089841934	-0.025285154	0.392650018	0.979719961	Potri.001G331400.1	AT5G14170.1
Potra2n1c2892.3	NA	0.674963689	NA	NA	Potri.008G175500.1	AT1G67430.1
Potra2n1c2893.1	0.517927592	0.05475713	3.49049E-05	0.941291229	Potri.001G331500.1	AT3G27150.1
Potra2n1c2894.1	-0.506306515	-0.288719391	0.001695774	0.664635201	Potri.001G331600.3	AT3G27160.1
Potra2n1c2895.1	-0.690626305	0.212534897	0.231901753	0.947290893	Potri.001G331700.1	AT3G27170.1
Potra2n1c2896.1	-0.183230458	-0.072512381	0.026006237	0.914903229	Potri.001G331800.2	AT3G58730.1
Potra2n1c2897.1	-0.175284635	-0.078838964	0.036180657	0.908764369		
Potra2n1c2898.1	-0.483078916	-0.257141483	0.00513976	0.758090459	Potri.001G331900.1	AT3G27180.1
Potra2n1c2899.1	0.299330021	-0.056554507	0.101999378	0.972336961	Potri.001G332100.1	AT5G40870.1
Potra2n1c29.1	NA	NA	NA	NA		
Potra2n1c290.1	0.681803076	0.186155768	0.001120814	0.871720501	Potri.001G035000.1	AT5G07900.1
Potra2n1c2900.1	-1.088232824	-0.100075844	0.016068081	0.978251387	Potri.001G332200.2	AT3G27200.1
Potra2n1c2901.1	-0.183964965	-0.128060777	0.655352594	0.972336961	Potri.001G332300.1	AT5G14180.1
Potra2n1c2902.1	-0.140493655	0.085661809	0.312634579	0.924251575	Potri.001G332500.1	AT3G01860.1
Potra2n1c2903.1	-0.397326728	-0.028317721	0.08532689	0.985016122		
Potra2n1c2904.1	0.476031203	0.368638149	0.635323555	0.943483421	Potri.001G332600.1	
Potra2n1c2905.1	-0.214798101	-0.087696978	0.107251497	0.932346161	Potri.001G332700.1	AT3G01850.1
Potra2n1c2906.3	0.314172289	1.214191225	0.740749106	0.679365931	Potri.T114600.1	AT3G50160.1
Potra2n1c2907.1	-0.446067263	-0.230162109	0.002823358	0.817407708	Potri.001G333100.1	AT3G01820.1
Potra2n1c2908.1	-0.764662778	-0.310770635	7.45893E-05	0.794641467	Potri.001G333200.1	AT3G01810.1
Potra2n1c2909.1	-1.130540298	-0.334232135	8.0655E-07	0.738935769	Potri.001G333300.1	AT5G14210.1
Potra2n1c291.1	-0.461855539	-0.130006065	0.199106098	0.948577865	Potri.001G035200.1	AT5G07900.1
Potra2n1c2910.1	-1.064268222	-0.282966787	1.86119E-06	0.824905557		
Potra2n1c2911.1					Potri.014G133200.1	AT5G07900.1
Potra2n1c2912.1	NA	NA	NA	NA	Potri.001G333400.1	AT4G39640.2
Potra2n1c2913.1	0.98420731	0.259291353	1.80085E-08	0.774545343	Potri.001G333500.1	AT5G14220.1
Potra2n1c2914.1	NA	1.571529889	NA	NA		

Potra2n1c2915.1	-0.846679892	-0.446173503	1.22003E-10	0.185836749	Potri.001G333600.1	AT5G14230.1
Potra2n1c2916.1	0.212953359	-0.061462347	0.272032812	0.959009636	Potri.001G333700.3	AT3G01800.1
Potra2n1c2917.1	0.200196992	-0.106199424	0.46399972	0.947969749	Potri.001G333800.1	AT4G17310.2
Potra2n1c2918.3	-2.921912828	0.286964786	NA	NA	Potri.001G333901.1	AT5G20090.1
Potra2n1c2919.1	-0.084260633	-0.014731184	0.706896643	0.994403137	Potri.001G334000.1	AT3G01790.2
Potra2n1c292.1	-0.553144116	-0.162300548	0.00167852	0.885192012	Potri.001G035300.1	AT5G07900.1
Potra2n1c2920.1	-0.343296399	-0.022638719	0.001648644	0.975578749	Potri.001G334100.1	AT5G14240.1
Potra2n1c2921.1	-0.40039069	-0.060555321	0.033715845	0.954419252		
Potra2n1c2922.1	0.001815254	-0.017021605	0.989416581	0.984373337	Potri.001G334200.2	AT5G14250.1
Potra2n1c2923.1	-0.162118232	0.003138742	0.231265618	0.998245864	Potri.001G334300.2	AT3G01780.1
Potra2n1c2924.1	NA	NA	NA	NA	Potri.017G033600.1	AT5G63380.1
Potra2n1c2925.1	-2.167220118	2.140785973	0.435298021	0.677013336	Potri.001G334500.1	AT5G50360.1
Potra2n1c2926.1	-0.246451424	-0.101361022	0.09957117	0.907073354	Potri.001G334600.1	AT5G45010.1
Potra2n1c2927.1	3.939328007	1.720733518	6.70229E-86	0.017464098	Potri.001G334700.1	AT4G31940.1
Potra2n1c2928.1	3.666794322	-0.376863699	1.67103E-07	0.960981446	Potri.001G334800.1	AT3G27220.1
Potra2n1c2929.1	0.207296054	-0.287505345	0.254748163	0.517110447	Potri.001G334900.1	AT5G59440.3
Potra2n1c293.1	0.199079622	-0.039161273	0.190979298	0.976279533		
Potra2n1c2930.1	0.014211436	0.058624407	0.896605074	0.943483421	Potri.001G335000.1	AT3G27260.1
Potra2n1c2931.1	-0.235534798	-0.202248782	0.37014286	0.910477984	Potri.001G335100.1	AT5G14280.1
Potra2n1c2932.1	1.459962178	2.60306301	NA	NA	Potri.001G335200.1	AT5G40780.1
Potra2n1c2933.1	1.299488076	0.556574777	0.203224042	0.915358268		
Potra2n1c2934.3	4.987352079	3.285431493	2.4007E-101	0.008641552	Potri.001G335300.1	AT5G40780.1
Potra2n1c2935.1	-0.414413474	-0.09253622	0.000194347	0.893945378	Potri.001G335400.3	AT3G01750.1
Potra2n1c2936.1	0.554467715	0.12739394	0.005214501	0.936402128	Potri.001G335600.1	AT5G14290.1
Potra2n1c2937.1	0.62100455	0.061056218	0.000866942	0.974275337	Potri.001G335700.1	AT5G40770.1
Potra2n1c2938.1	0.023062428	0.13142132	0.95683306	0.965595755	Potri.001G335900.1	AT1G19250.1
Potra2n1c2939.1	-0.514567542	-1.814911445	0.499001636	0.427304019	Potri.001G335900.1	AT1G19250.1
Potra2n1c294.1	0.1978692	-0.04012932	0.190576197	0.975578749	Potri.001G035500.1	AT4G12560.2
Potra2n1c2940.1						
Potra2n1c2941.1	-0.833286862	0.070614247	0.12828991	0.9869326	Potri.001G336200.1	AT5G40690.1
Potra2n1c2942.1	-0.661481131	-0.750970111	0.121286423	0.707861355	Potri.001G336300.1	AT3G01710.2
Potra2n1c2943.1	0.50804607	0.157925842	0.000273436	0.804796567	Potri.001G336400.1	AT3G01720.1

Potra2n1c2944.1	-0.549276176	-0.323857972	0.088598155	0.688733073	Potri.012G081000.1	AT1G70420.1
Potra2n1c2945.1	-0.517371169	-0.359261729	0.197541018	0.709432167		
Potra2n1c2946.1	-0.34726993	0.045537535	0.098848598	0.974275337	Potri.001G336500.1	AT5G40710.1
Potra2n1c2947.1	0.088627554	0.175933411	0.704501169	0.846680547	Potri.001G336600.1	AT3G27340.1
Potra2n1c2948.1	-0.782837462	-0.345950076	0.05581706	0.856141735	Potri.001G336700.1	AT5G14340.1
Potra2n1c2949.1	-0.272309419	0.989832605	0.544746781	0.773641026	Potri.001G336832.1	AT3G27330.1
Potra2n1c295.1	0.210407314	0.452341548	0.854903321	0.943819961	Potri.001G034700.1	AT5G07900.1
Potra2n1c2950.1	-0.20677994	-0.187053701	0.037244759	0.72618389	Potri.001G336900.1	AT5G50960.1
Potra2n1c2951.1	-0.305772799	-0.055083782	0.677653331	0.9896961		
Potra2n1c2952.1	-1.122129238	-0.251284483	1.38358E-06	0.854980698	Potri.001G337100.1	AT3G28050.1
Potra2n1c2953.1	0.265743846	-0.010957045	0.336776666	0.996032145	Potri.001G337200.1	AT5G22660.2
Potra2n1c2954.1	0.081733494	0.016280719	0.646461789	0.9896961	Potri.001G337300.1	AT3G26000.1
Potra2n1c2955.1	0.057886876	0.050502077	0.748214164	0.959899756	Potri.001G337400.2	AT5G40760.1
Potra2n1c2956.1						
Potra2n1c2957.1	NA	NA	NA	NA	Potri.001G182200.1	
Potra2n1c2958.1	-0.329762591	0.154944561	0.005918595	0.849446235	Potri.001G337600.1	AT5G40740.1
Potra2n1c2959.1	NA	NA	NA	NA		
Potra2n1c296.1	0.321649764	0.319343795	0.002468945	0.365620154	Potri.001G035700.1	AT3G54380.1
Potra2n1c2960.1	1.517671607	1.571529889	NA	NA	Potri.001G337900.1	AT3G02645.1
Potra2n1c2961.1	3.076036896	NA	NA	NA	Potri.001G337900.1	AT3G02645.1
Potra2n1c2962.1	0.584910023	0.808023943	NA	NA		
Potra2n1c2963.1						
Potra2n1c2964.1	-0.093096089	-0.034966322	0.549617752	0.977530072	Potri.001G338000.1	AT3G27325.2
Potra2n1c2965.1	4.280935185	3.279271581	0.003043622	NA	Potri.001G338100.1	AT5G40690.1
Potra2n1c2966.1	0.228932627	0.314110616	0.123219413	0.716548554	Potri.001G338200.2	AT5G40660.1
Potra2n1c2967.1	NA	NA	NA	NA	Potri.001G338300.1	
Potra2n1c2968.1	0.213575349	-0.002121183	0.089799204	0.999276367		
Potra2n1c2969.1	-0.12927455	-0.262957671	0.915664997	0.966917036		
Potra2n1c297.1	0.89807706	0.230067519	6.44445E-08	0.829716187	Potri.001G035800.1	AT3G11450.1
Potra2n1c2970.1	-0.933193129	0.022342568	0.02909345	0.995039528	Potri.001G338500.2	AT5G40645.1
Potra2n1c2971.1	0.446058749	0.105897374	0.000341395	0.921063801	Potri.001G338700.1	AT5G57280.1
Potra2n1c2972.1	NA	0.674963689	NA	NA	Potri.001G338800.1	AT5G14345.1

Potra2n1c2973.3	-0.328789441	-0.067031035	0.000577488	0.907073354	Potri.001G338900.1	AT5G40640.1
Potra2n1c2974.1	-2.115746751	0.204702253	0.210688004	0.977559521	Potri.017G070800.1	AT3G46610.1
Potra2n1c2975.1	0.228791894	-0.507823185	0.800598724	0.923478213	Potri.001G339100.1	AT5G14360.1
Potra2n1c2976.1	-1.629350185	-0.28222422	0.201520964	0.973428178	Potri.T125900.1	AT1G53540.1
Potra2n1c2977.1	-0.276033557	-0.183848878	0.293411455	0.763747409	Potri.001G339200.3	AT5G14370.1
Potra2n1c2978.1	NA	NA	NA	NA	Potri.004G041900.1	AT1G65070.2
Potra2n1c2979.1	NA	NA	NA	NA		
Potra2n1c298.1	2.685015931	NA	NA	NA		
Potra2n1c2980.1	3.73874856	1.561996384	1.25587E-52	0.076328052	Potri.001G339500.2	AT3G27400.1
Potra2n1c2981.2	1.64774763	NA	NA	NA	Potri.001G339600.1	AT1G44900.2
Potra2n1c2982.1	1.931605898	3.594836778	NA	NA	Potri.013G138500.1	ATCG01280.1
Potra2n1c2983.1	0.011929649	-0.033150379	0.957932829	0.981057849	Potri.001G339900.1	AT3G03420.1
Potra2n1c2984.1	0.397354652	-0.031933521	0.784207765	NA		
Potra2n1c2985.2	-0.69656793	-0.213395919	3.7499E-05	0.731818379	Potri.001G340000.1	AT5G14390.1
Potra2n1c2986.1	0.098875962	-0.114689201	0.77628232	0.953831244	Potri.001G340100.1	
Potra2n1c2987.1	-0.358742463	0.016285194	0.33626337	0.995581218		
Potra2n1c2988.1	-0.074968295	0.206422636	0.856655327	0.911339141		
Potra2n1c2989.1	NA	1.89838425	NA	NA	Potri.001G039600.1	
Potra2n1c299.1	3.073325943	1.571529889	NA	NA	Potri.001G035900.2	AT3G13690.1
Potra2n1c2990.1	NA	1.394613675	NA	NA	Potri.015G047300.1	AT5G60410.4
Potra2n1c2991.1	-1.614894497	-0.567977495	0.449099672	NA	Potri.001G341000.1	AT5G14400.1
Potra2n1c2992.1	0.538510898	0.068199393	0.000487735	0.953831244	Potri.001G341100.1	AT5G20890.1
Potra2n1c2993.1	2.353595065	-0.145762333	0.373081088	NA		
Potra2n1c2994.1	0.373253338	0.22383248	0.001693921	0.683816919	Potri.001G341300.1	AT3G56510.2
Potra2n1c2995.1	0.318345432	0.091863259	0.029653019	0.945019616	Potri.001G341400.1	AT4G35850.1
Potra2n1c2996.1	1.664677056	NA	NA	NA	Potri.001G341600.1	AT5G60020.1
Potra2n1c2997.1	0.958792987	0.172626068	0.276377507	0.979719961	Potri.001G341700.1	
Potra2n1c2998.1						
Potra2n1c2999.1						
Potra2n1c3.1	1.459962178	NA	NA	NA	Potri.001G000500.1	AT1G55570.1
Potra2n1c30.1	0.068605873	-0.025726757	0.853445815	0.99005311	Potri.001G003500.1	AT5G56520.1
Potra2n1c300.1	-0.25021455	-0.063683621	0.168607719	0.956865545	Potri.001G036000.1	AT3G11440.1

Potra2n1c3000.1	-0.008824099	0.257101127	0.967316784	0.8043603	Potri.001G341900.1	AT5G60040.1
Potra2n1c3001.1	-0.012343457	0.214378398	0.974763364	0.895460013		
Potra2n1c3002.1	1.254300712	1.077247295	0.003088057	0.349558394	Potri.001G342000.1	AT3G45780.2
Potra2n1c3003.1	-0.698248982	-0.33830921	1.43986E-07	0.351105764	Potri.001G342200.1	AT5G14420.4
Potra2n1c3004.1	0.110961275	-0.079761821	0.399725304	0.944480065	Potri.001G342300.1	AT5G14430.1
Potra2n1c3005.1	0.82357019	2.253733581	NA	NA	Potri.017G071200.1	AT3G27440.1
Potra2n1c3006.1	0.365736556	0.109612501	0.028714806	0.921516343	Potri.001G342400.1	AT5G40570.1
Potra2n1c3007.1	0.500115799	0.051708182	0.004024654	0.977559521	Potri.001G342500.1	AT4G15000.1
Potra2n1c3008.1	-0.061618108	0.028143043	0.822580695	0.97521505	Potri.001G342600.1	AT5G14450.1
Potra2n1c3009.1	-0.453002691	-0.015306999	1.05385E-05	0.989878071	Potri.001G342700.1	AT3G27460.1
Potra2n1c301.1	-0.059789029	-0.004340994	0.725002503	0.99702095	Potri.001G036100.3	AT5G06120.3
Potra2n1c3010.1	-0.350198041	-0.061514362	0.001870518	0.941855873	Potri.001G342800.1	AT4G13010.1
Potra2n1c3011.2	-0.136350456	-0.091321489	0.190641112	0.921099436	Potri.001G343100.1	AT5G14460.1
Potra2n1c3012.1	-0.282377217	0.085141325	0.158974577	0.941855873	Potri.001G343200.1	AT3G27520.1
Potra2n1c3013.1	-0.199444106	-0.139995784	0.268129672	0.914903229	Potri.001G343400.1	AT3G01640.1
Potra2n1c3014.1	-0.175531731	0.224096716	0.153657459	0.842247476	Potri.001G343500.1	AT3G27530.1
Potra2n1c3015.1	0.107889193	-0.02542401	0.532003386	0.983113084	Potri.001G343600.1	AT3G27540.1
Potra2n1c3016.1	0.404481576	0.232645276	0.080073353	0.899205262	Potri.001G343700.1	AT3G27550.1
Potra2n1c3017.1	2.010637778	0.220026594	1.46088E-09	0.946137117	Potri.001G343800.1	AT3G01600.1
Potra2n1c3018.1	0.420868411	0.043023962	0.049148067	0.959881296	Potri.001G343900.1	AT5G50180.1
Potra2n1c3019.3	-0.902622323	-0.220309697	1.77586E-13	0.784845657	Potri.001G344100.4	AT5G14500.1
Potra2n1c302.1	-0.047827815	0.071426482	0.728589373	0.930203195	Potri.001G036400.1	AT3G54360.1
Potra2n1c3020.1	-0.90757993	0.338222884	NA	NA	Potri.001G344200.1	AT5G14510.1
Potra2n1c3021.1	0.82357019	NA	NA	NA	Potri.001G344300.1	AT3G28007.1
Potra2n1c3022.1	0.098099973	0.103211802	0.333968655	0.906545272	Potri.001G344400.1	AT5G40510.1
Potra2n1c3023.1	-0.77129019	-0.391644959	2.59661E-07	0.522372765	Potri.001G344600.1	AT3G27580.2
Potra2n1c3024.1	-1.071658633	-0.181825626	7.16237E-06	0.892986206	Potri.001G344700.2	AT5G40500.2
Potra2n1c3025.1	-0.011210766	-0.034386727	0.925739565	0.972159498	Potri.001G344900.2	AT5G40490.1
Potra2n1c3026.1	-1.662160358	-0.346771329	0.000235849	0.846680547		
Potra2n1c3027.1	0.05668439	0.180411768	0.753887957	0.773641026	Potri.001G345100.1	AT5G40480.1
Potra2n1c3028.1	0.141896498	0.174831404	0.276969295	0.844001073	Potri.001G345200.1	AT5G19130.1
Potra2n1c3029.1	-0.071676506	0.815923144	0.769884551	0.298167012	Potri.001G345300.1	AT5G40450.1

Potra2n1c303.1	3.252589452	1.796301909	8.67017E-11	0.116967248	Potri.001G036600.1	AT5G07010.1
Potra2n1c3030.1	0.25549142	-0.2966384	0.741674522	0.956610193	Potri.001G345400.1	AT3G27640.1
Potra2n1c3031.1	-0.039304236	-0.038740054	0.736327815	0.968101851	Potri.001G345500.1	AT5G40440.1
Potra2n1c3032.1	0.127968738	-0.553203856	0.929136015	0.962696825	Potri.001G345700.1	AT5G63090.3
Potra2n1c3033.1	-1.970460481	-3.841734588	0.458592523	NA	Potri.001G345800.2	AT3G01570.1
Potra2n1c3034.1	0.102918014	-0.060187431	0.529988964	0.96820892	Potri.001G345900.2	AT5G14530.1
Potra2n1c3035.3	0.153994303	0.226689486	0.249669616	0.562306981	Potri.001G346000.1	AT3G27670.1
Potra2n1c3036.1	0.257539842	0.481097174	0.854612009	NA	Potri.017G071600.2	AT5G40400.1
Potra2n1c3037.1	-0.519351091	-0.301203325	0.000336821	0.442809314	Potri.001G346100.1	AT3G27830.1
Potra2n1c3038.1	-0.188472077	-0.112399644	0.139219394	0.869175327	Potri.001G346200.1	AT3G27820.1
Potra2n1c3039.1	-2.394942576	0.120885407	0.189687624	0.987548286	Potri.001G322100.1	AT3G26782.1
Potra2n1c304.1	2.207592799	0.499402265	0.283517505	NA	Potri.001G036600.1	AT5G07010.1
Potra2n1c3040.1	-1.345253658	1.06963283	0.476895538	0.830056077	Potri.001G346500.1	AT1G05700.1
Potra2n1c3041.2	0.824497211	0.660619236	NA	NA	Potri.017G071500.1	AT3G27810.1
Potra2n1c3042.1	0.188274461	0.076435773	0.206103076	0.922993749	Potri.001G346700.1	AT5G14660.2
Potra2n1c3043.1	6.40424661	2.512595291	3.31849E-13	NA	Potri.001G346800.1	AT5G14650.1
Potra2n1c3044.1	0.718210752	-0.06617226	0.00225744	0.973948905	Potri.001G346900.1	AT2G44580.1
Potra2n1c3045.1					Potri.006G152300.1	
Potra2n1c3046.1	-0.108906033	-0.01765843	0.313640819	0.982422288	Potri.001G347000.10	AT5G15380.1
Potra2n1c3047.3	0.206187893	0.084151649	0.077036549	0.900551258	Potri.001G347100.1	AT5G14610.2
Potra2n1c3048.1	1.664677056	NA	NA	NA	Potri.001G347200.1	AT3G27785.1
Potra2n1c3049.1	-0.039061824	0.762369162	0.984165229	0.879339642	Potri.001G347300.2	AT3G01550.1
Potra2n1c305.1	-0.61910233	-0.767708831	NA	NA	Potri.001G036700.1	AT5G07010.1
Potra2n1c3050.1	1.63896333	0.423521468	7.36938E-14	0.817407708	Potri.001G347400.2	AT3G27770.1
Potra2n1c3051.1	-1.936986977	0.378352127	0.136516267	0.945254452	Potri.001G347600.1	AT5G23240.1
Potra2n1c3052.1	0.118231562	-0.042078961	0.345839884	0.972159498	Potri.001G347700.2	AT5G40370.1
Potra2n1c3053.1	0.075784978	0.041859319	0.659682966	0.97318201	Potri.001G347800.1	AT5G14590.1
Potra2n1c3054.1	0.214638129	-0.13701528	0.343116105	0.941855873	Potri.001G347900.1	AT5G14580.1
Potra2n1c3055.1	-1.905574497	-0.209767103	0.223111491	0.977559521		
Potra2n1c3056.1	0.821899975	-0.022417553	0.20495551	0.996154416	Potri.001G348000.1	AT5G40380.1
Potra2n1c3057.1	1.349342802	0.155898299	0.379998718	NA	Potri.001G348100.1	AT2G16890.2
Potra2n1c3058.3	-0.006994696	-0.255365454	0.99016258	0.927423678	Potri.001G348200.2	AT3G27730.1

Potra2n1c3059.1	0.551575695	-0.172402266	0.08586167	0.900551258	Potri.001G348300.1	AT5G14570.1
Potra2n1c306.1	-0.185667413	0.041887801	0.262988809	0.973948905	Potri.001G036800.1	AT1G73100.1
Potra2n1c3060.1	-0.377177567	-0.112389759	0.044945822	0.936364479	Potri.001G348400.2	AT5G14550.1
Potra2n1c3061.1	-0.12037035	-0.153882435	0.395397155	0.835819291	Potri.001G348500.1	AT3G01560.1
Potra2n1c3062.1	2.197840931	0.404991698	1.33792E-18	0.720714044	Potri.001G348600.1	AT5G14760.1
Potra2n1c3063.1	-0.078825195	0.110468575	0.57527346	0.880870972	Potri.001G348700.2	AT3G27930.1
Potra2n1c3064.1	-0.746674529	-0.225413136	0.001787862	0.788065047	Potri.001G348800.2	AT3G27925.1
Potra2n1c3065.1	-1.409983009	-0.834601298	NA	NA	Potri.001G348900.11	AT3G01500.2
Potra2n1c3066.1	0.232113441	0.274551065	0.124893932	0.646294977	Potri.001G349000.1	AT3G01510.1
Potra2n1c3067.1	NA	0.674963689	NA	NA		
Potra2n1c3068.1	-1.592456044	-0.317805802	1.8807E-06	0.677013336	Potri.001G349100.3	AT3G27870.1
Potra2n1c3069.1	-0.00840621	-0.011397833	0.940160916	0.989878071	Potri.001G349300.1	AT5G14720.1
Potra2n1c307.1	-0.982681264	-0.837964761	8.96022E-05	0.216891326	Potri.001G036900.1	AT3G21240.1
Potra2n1c3070.1	0.08611499	-0.031953651	0.487326074	0.974756511	Potri.001G349400.1	AT5G59710.1
Potra2n1c3071.1	0.487652285	0.16216692	0.00201562	0.879434349	Potri.001G349500.1	AT5G14710.1
Potra2n1c3072.1	2.187115295	0.396650862	2.02794E-11	0.827798439	Potri.001G349600.1	AT5G14700.1
Potra2n1c3073.1	-2.427590862	-0.120343673	0.23264872	NA		
Potra2n1c3074.1	-2.151048126	-1.671345499	0.094539505	0.781101394	Potri.001G349700.1	AT5G14690.1
Potra2n1c3075.1	-0.180017219	-0.011134529	0.23866235	0.992934677	Potri.001G349800.1	AT3G28460.1
Potra2n1c3076.1	0.38562439	0.198902916	0.000891448	0.293583922	Potri.001G349900.1	AT3G28450.1
Potra2n1c3077.1	0.34903218	0.204990984	0.003216892	0.680030952	Potri.001G350000.1	AT1G74860.1
Potra2n1c3078.1	-0.630470326	-0.175525188	0.004656842	0.922011871	Potri.001G350100.2	AT3G28370.1
Potra2n1c3079.1	0.116293233	0.23495739	0.252257085	0.693906898	Potri.001G350200.1	AT5G15020.2
Potra2n1c308.1	1.847831248	0.747229276	5.55801E-20	0.175898575	Potri.001G037000.1	AT5G23570.1
Potra2n1c3080.1	-0.316574779	-0.359530095	0.125844273	0.660636854	Potri.001G350300.1	AT5G40190.1
Potra2n1c3081.1	0.667924336	0.247550919	1.58688E-10	0.477503371	Potri.001G350400.1	AT5G14950.1
Potra2n1c3082.1	0.156721623	0.026023141	0.405915956	0.985393296	Potri.001G350500.3	AT5G14910.1
Potra2n1c3083.1	3.048038743	2.257947819	0.001587086	0.766668365	Potri.009G001800.1	AT2G01050.1
Potra2n1c3084.1	NA	NA	NA	NA	Potri.001G350600.1	AT5G14920.1
Potra2n1c3085.1	-0.006829979	0.093846413	0.970103579	0.895474078	Potri.001G350700.1	AT3G01380.1
Potra2n1c3086.1	1.545832463	NA	NA	NA		
Potra2n1c3087.1	0.509363397	-0.466960841	0.067474717	0.556125138	Potri.T058000.1	AT1G50240.2

Potra2n1c3088.1	-0.196886304	0.093710502	0.08572261	0.856891638	Potri.001G350800.1	AT3G01370.1
Potra2n1c3089.1	-0.454940231	-0.187977275	2.94256E-05	0.752523551	Potri.001G350900.2	AT2G04700.1
Potra2n1c309.4	-0.228952566	-0.001862317	0.009972298	0.998823528	Potri.001G037200.1	AT3G14250.1
Potra2n1c3090.1	-0.132852729	-0.289624952	0.711044134	0.865538396	Potri.001G351000.1	AT3G28200.1
Potra2n1c3091.1	4.649185416	3.157019191	0.048194666	NA	Potri.017G077000.1	AT3G28700.1
Potra2n1c3092.1	4.60397395	1.88036188	1.5164E-19	0.17564584	Potri.001G351200.2	AT5G62680.1
Potra2n1c3093.1	0.513660381	0.49892963	0.836618484	NA	Potri.001G351300.1	AT3G01490.1
Potra2n1c3094.1	-0.089769652	0.027443724	0.795364518	0.991110595	Potri.017G072800.5	AT3G01480.1
Potra2n1c3095.1	-0.049460396	0.106759836	0.775046561	0.91753191	Potri.015G073600.1	AT5G40250.1
Potra2n1c3096.1					Potri.015G049700.1	AT5G61280.1
Potra2n1c3097.1	-0.179755461	-0.011780009	0.081242366	0.991732307	Potri.017G072100.1	AT3G27700.2
Potra2n1c3098.1	-0.39172244	0.109337538	0.007377329	0.920141992	Potri.001G351800.1	AT4G18240.1
Potra2n1c3099.1	NA	NA	NA	NA	Potri.001G351600.2	AT1G78520.1
Potra2n1c31.1	0.871267362	0.446337783	0.003262641	0.683816919	Potri.001G003700.2	AT5G23710.1
Potra2n1c310.1	-1.085201922	-0.354934422	0.001170219	0.623736221	Potri.001G037400.1	AT5G43150.1
Potra2n1c3100.2	0.278359315	0.134487212	0.017707459	0.813575895	Potri.001G352000.1	AT4G18230.1
Potra2n1c3101.1	-0.192478034	0.132135515	0.812056369	0.938967511	Potri.001G352100.1	AT4G18210.1
Potra2n1c3102.1	0.456788105	0.263356554	0.159916321	0.860810425	Potri.001G352000.1	AT4G18230.1
Potra2n1c3103.1	0.252285509	0.405551394	0.710954244	0.679125998	Potri.001G352100.1	AT4G18210.1
Potra2n1c3104.1	-0.051854421	0.294845101	0.929646026	0.874648591	Potri.001G352200.1	AT4G18210.1
Potra2n1c3105.2	-0.660120899	-0.268487389	0.006550883	0.9045874	Potri.001G352300.2	AT2G34410.1
Potra2n1c3106.1	4.653240382	1.687244032	7.76188E-37	0.305895513	Potri.001G352400.1	AT4G18170.1
Potra2n1c3107.1	0.700589899	-0.151124466	0.193585416	0.949966783	Potri.001G352500.1	AT4G18160.1
Potra2n1c3108.7	-0.235474338	-0.12655099	0.010825243	0.814001428	Potri.001G352600.1	AT1G29850.1
Potra2n1c3109.1	-0.218604827	-0.155303289	0.357549908	0.922687399	Potri.001G352700.1	AT1G29790.2
Potra2n1c311.1	-0.272857706	-0.190414436	0.221088236	0.858481608	Potri.001G037500.1	AT2G24530.1
Potra2n1c3110.1	0.293247482	0.374712507	0.10967367	0.305539181	Potri.001G352800.1	AT1G29810.1
Potra2n1c3111.1	-0.109027971	-0.01402192	0.516924839	0.989300076	Potri.001G352900.2	AT1G60970.1
Potra2n1c3112.2	0.126156304	-0.032649666	0.423908607	0.974756511	Potri.001G353000.1	AT1G29820.1
Potra2n1c3113.1	0.019848929	0.132888084	0.907309627	0.885375133	Potri.001G353100.1	AT1G29350.1
Potra2n1c3114.1	-0.108674431	0.275834379	0.752408441	0.854980698	Potri.001G353200.1	
Potra2n1c3115.2	0.141151806	-0.146157802	0.698340144	0.953608209	Potri.001G353400.1	AT1G29380.1

Potra2n1c3116.2	-0.635606127	0.020438079	0.002676051	0.992934677	Potri.001G353500.1	AT1G29390.1
Potra2n1c3117.1					Potri.001G353600.1	AT1G76750.1
Potra2n1c3118.3	1.066653467	0.38606303	5.68633E-16	0.163343066	Potri.001G353700.1	AT5G46410.2
Potra2n1c3119.1	-0.558560364	0.018292633	3.71498E-05	0.988154814	Potri.001G353800.1	AT5G46420.1
Potra2n1c312.1	-0.813553695	-0.279392676	0.001519374	0.79477084	Potri.001G037700.1	AT5G57330.1
Potra2n1c3120.1	0.40167518	0.0539451	0.005515417	0.955790664	Potri.001G353900.1	AT4G18100.1
Potra2n1c3121.1	-0.128214717	-0.026247095	0.280316061	0.977227089	Potri.001G354000.1	AT3G47810.2
Potra2n1c3122.6	0.273579729	0.266393502	0.471448956	0.900551258	Potri.001G354400.1	AT5G46460.1
Potra2n1c3123.1	-0.751175768	-0.065104401	NA	NA	Potri.001G354500.1	AT1G29520.1
Potra2n1c3124.1	0.228224791	-0.744589582	NA	NA		
Potra2n1c3125.1	-0.102642187	0.070752388	0.416533814	0.943685779	Potri.001G354600.1	AT1G29530.1
Potra2n1c3126.1	-0.190768725	-0.021204672	0.249505865	0.988210253	Potri.011G077500.1	AT4G18060.1
Potra2n1c3127.1	-0.927033731	-0.202529989	0.000359855	0.929630614	Potri.001G354900.1	AT4G18050.1
Potra2n1c3128.2	2.03991299	0.381019122	6.18595E-07	0.856141735		
Potra2n1c3129.1	2.58269132	0.349689099	1.76857E-05	0.917014822	Potri.001G355100.1	AT1G17020.1
Potra2n1c313.1	0.028777894	-1.478536539	NA	NA	Potri.001G037800.2	AT3G12660.1
Potra2n1c3130.1	8.013336739	2.798547252	7.46577E-32	0.544330018	Potri.001G355200.1	AT1G17020.1
Potra2n1c3131.2	0.180267288	0.079425995	0.382226397	0.939915707	Potri.001G355300.1	AT5G46550.1
Potra2n1c3132.1	-1.316917544	0.566646426	0.227113577	0.753615154	Potri.001G355500.1	AT3G14770.1
Potra2n1c3133.1	-0.036452887	0.058138569	0.898711515	0.973347974	Potri.001G355600.1	AT4G26850.1
Potra2n1c3134.1	NA	NA	NA	NA		
Potra2n1c3135.1	0.076233065	0.003396763	0.639157348	0.997821888	Potri.001G355800.1	AT5G55130.1
Potra2n1c3136.2	1.459962178	NA	NA	NA	Potri.001G356000.1	AT2G32990.1
Potra2n1c3137.1					Potri.006G161300.1	AT5G66680.1
Potra2n1c3138.1	-0.193153952	-0.110087609	0.146534617	0.935099709	Potri.001G356100.1	AT1G50640.1
Potra2n1c3139.1	-0.21377743	-0.307858815	0.637049315	0.900551258		
Potra2n1c314.1	-0.801635776	-0.906114321	0.211045835	0.691615807	Potri.001G037900.10	AT1G79640.1
Potra2n1c3140.1	-0.881639751	-0.551781359	0.055098041	0.743252386	Potri.001G356200.1	AT3G20300.1
Potra2n1c3141.1	-0.107502054	-0.058777715	0.29238133	0.937687311	Potri.001G356300.1	AT3G20290.1
Potra2n1c3142.1	-0.699694929	-0.087502255	0.000223982	0.960981446	Potri.001G356400.3	AT4G22320.1
Potra2n1c3143.1	NA	NA	NA	NA	Potri.003G144900.2	AT1G64625.2
Potra2n1c3144.1	-0.087738503	-0.011656283	0.667612553	0.996032145	Potri.001G356500.1	AT5G55230.1

Potra2n1c3145.1	-0.184802598	-0.403496959	0.965441168	NA	Potri.001G356600.2	AT1G50720.1
Potra2n1c3146.1	-0.15428553	-0.008310587	0.207772465	0.994692557	Potri.001G356700.1	AT1G50730.1
Potra2n1c3147.4	-0.032913493	0.078297669	0.870568632	0.948577865	Potri.001G356800.4	AT5G55100.2
Potra2n1c3148.1	0.153131088	0.16130306	0.276252481	0.852284301	Potri.001G356900.2	AT1G11910.1
Potra2n1c3149.1	1.64774763	NA	NA	NA	Potri.014G072800.1	AT3G61050.2
Potra2n1c315.1	-0.675186094	-0.361813254	0.009114182	0.767368598	Potri.001G037900.10	AT1G79640.1
Potra2n1c3150.1	0.722845317	0.046825198	6.45214E-07	0.972159498	Potri.001G357000.1	AT4G26910.1
Potra2n1c3151.1	-0.113631845	-0.180922076	0.577053331	0.855587395	Potri.001G357100.1	AT1G30440.1
Potra2n1c3152.1	-0.138185343	-0.07185124	0.435826406	0.953108412	Potri.001G357200.1	AT3G20330.1
Potra2n1c3153.1	-0.291245295	-0.113228829	0.089762699	0.911339141	Potri.001G357600.2	AT1G30450.2
Potra2n1c3154.1	0.432126579	0.025789213	0.000313388	0.989300076	Potri.001G357700.1	AT2G34680.1
Potra2n1c3155.1	3.544969546	1.363811008	0.389034834	NA	Potri.011G090100.1	AT1G50660.1
Potra2n1c3156.1	0.104754287	-0.06348041	0.404309533	0.954419252	Potri.001G357800.1	AT1G30460.1
Potra2n1c3157.1					Potri.005G151900.1	
Potra2n1c3158.1						
Potra2n1c3159.1	0.131094684	0.065468244	0.139409412	0.930270138	Potri.001G357900.1	AT4G05420.1
Potra2n1c316.1	0.079635494	0.029753912	0.551167967	0.973347974	Potri.001G038000.1	AT1G79650.1
Potra2n1c3160.1	1.664677056	NA	NA	NA	Potri.001G358000.1	AT4G03620.1
Potra2n1c3161.1	0.804292952	-0.080636047	0.109513165	0.983446961	Potri.001G358100.1	AT4G21070.1
Potra2n1c3162.1	-0.574683788	-0.235691807	0.008112437	0.880316339	Potri.001G358200.1	AT5G07220.1
Potra2n1c3163.2	-0.425825527	0.025441952	0.058829159	0.987226805	Potri.001G358300.1	AT1G30360.1
Potra2n1c3164.2	0.325924783	0.284457822	0.108444022	0.814001428	Potri.001G358400.2	AT2G34640.1
Potra2n1c3165.1	0.274610676	0.279388982	0.328271781	0.846048947		
Potra2n1c3166.1	-0.050730734	-0.064796283	0.746558624	0.957668459	Potri.001G358500.1	AT5G37020.1
Potra2n1c3167.6	2.109676378	0.465477564	5.35645E-18	0.737914109	Potri.001G358600.1	AT1G30320.1
Potra2n1c3168.1	-0.254361227	-0.030257309	0.012875426	0.966228173	Potri.001G358700.1	AT5G55260.1
Potra2n1c3169.1	0.510557317	0.218986753	0.000286811	0.69862311	Potri.001G358800.1	AT1G04970.1
Potra2n1c317.1	0.100818435	0.11923454	0.370247222	0.860325259	Potri.001G038100.1	AT1G16180.2
Potra2n1c3170.1	-0.219418446	0.025811436	0.070011679	0.981772014	Potri.001G358900.6	AT1G50620.1
Potra2n1c3171.1	-2.215582392	-0.587969265	0.000172659	0.80340893	Potri.001G359000.1	AT5G55250.1
Potra2n1c3172.1	-0.127786146	0.128994755	0.307096012	0.914554682	Potri.001G359200.3	AT3G20440.2
Potra2n1c3173.3	-0.053233833	0.018381238	0.707192121	0.986636967	Potri.001G359300.2	AT3G20800.1

Potra2n1c3174.7	-0.034239748	-0.027456929	0.748640242	0.977227089	Potri.001G359400.1	AT1G04950.2
Potra2n1c3175.1	-0.165028867	-0.09126798	0.118421187	0.936402128	Potri.001G359500.1	AT5G55280.1
Potra2n1c3176.2	-0.157446922	-0.047924773	0.277276446	0.968979184		
Potra2n1c3177.1	0.19288728	0.223686333	0.002794007	0.521565921	Potri.001G359700.1	AT5G55310.1
Potra2n1c3178.1	0.2128388	0.179008406	0.0493694	0.754727624	Potri.001G359900.1	AT5G55390.2
Potra2n1c3179.1	-0.138437676	0.049770489	0.453620188	0.973948905	Potri.001G360100.1	AT4G26700.2
Potra2n1c318.1	-0.200302606	-0.000830842	0.482234419	0.999937671	Potri.001G040300.1	AT1G79660.1
Potra2n1c3180.2	0.468796754	-0.61225647	0.30397275	0.731968019	Potri.001G360200.3	AT3G20150.1
Potra2n1c3181.2	0.018842588	-0.147580475	0.908552805	0.833952007	Potri.001G360300.1	AT5G55510.1
Potra2n1c3182.1	1.13457761	-1.465929376	NA	NA	Potri.017G110000.1	AT5G59240.1
Potra2n1c3183.1	0.331085886	0.043808344	0.012389315	0.971880993	Potri.001G360600.1	AT5G55500.1
Potra2n1c3184.1	-0.120343543	-0.24101521	0.821694085	0.872698255	Potri.001G360800.1	AT5G55480.1
Potra2n1c3185.1	-0.054110551	-0.142583203	0.913010398	0.937846405	Potri.001G360700.1	AT4G26690.1
Potra2n1c3186.1	-0.121694699	0.303048896	0.600434692	0.80654978	Potri.001G361100.1	AT5G55530.1
Potra2n1c3187.1	0.877886026	0.355460878	8.36378E-15	0.837852092	Potri.001G361200.2	AT5G55540.1
Potra2n1c3188.1	0.125582788	-0.081537208	0.282407622	0.917516356	Potri.001G361300.1	AT3G07810.1
Potra2n1c3189.1					Potri.001G361400.1	AT2G01050.1
Potra2n1c319.1	6.27835913	3.89997123	9.03354E-05	NA	Potri.T132900.1	AT1G16130.1
Potra2n1c3190.1	0.418605896	0.098252909	0.007873321	0.925933968	Potri.001G361500.1	AT3G02650.1
Potra2n1c3191.2	-0.134502014	0.235990859	0.678923333	0.922011871	Potri.001G361600.1	AT4G26640.2
Potra2n1c3192.1	-0.088712454	-0.146570291	0.581630207	0.880553952		
Potra2n1c3193.1	-0.630645104	-0.104715272	0.011821508	0.940255038	Potri.001G361800.1	AT5G55580.1
Potra2n1c3194.1	0.066618091	0.037635122	0.687687483	0.963355476	Potri.001G361900.1	AT4G37190.1
Potra2n1c3195.1	-0.044324241	0.021084742	0.797686424	0.986636967	Potri.001G362100.1	AT1G55930.1
Potra2n1c3196.1	1.168194147	0.663465872	3.76418E-13	0.064873104	Potri.001G364000.1	AT3G13110.1
Potra2n1c3197.1	-0.290797106	-0.110886686	0.044345045	0.868145269	Potri.001G364100.1	AT3G13120.1
Potra2n1c3198.1	-0.637934825	0.67621918	NA	NA	Potri.001G364200.1	AT5G55590.1
Potra2n1c3199.1	-0.121835614	0.016486286	0.268862834	0.985393296	Potri.001G365800.3	AT5G55600.1
Potra2n1c32.1	1.087879158	1.026695244	NA	NA		
Potra2n1c320.1	8.411140252	5.787273143	3.20299E-13	0.361307731	Potri.T166600.1	AT1G16260.1
Potra2n1c3200.1	0.437077466	0.002830551	0.006861705	0.999325699	Potri.001G366000.1	AT5G55610.1
Potra2n1c3201.1	0.549746865	0.048958417	0.032782105	0.985393296	Potri.001G366100.1	AT1G55910.1

Potra2n1c3202.1	0.102124129	0.078326582	0.497846546	0.925184207	Potri.001G364500.1	AT1G55900.1
Potra2n1c3203.1	NA	NA	NA	NA		
Potra2n1c3204.1	-1.059370142	-0.223652797	1.5932E-05	0.903698951	Potri.001G366500.1	AT3G13130.1
Potra2n1c3205.5	0.205834808	0.236015741	0.353060603	0.854980698	Potri.001G366700.1	AT3G13170.1
Potra2n1c3206.1	-0.312471436	-0.198532569	0.067752208	0.798429987	Potri.001G366800.1	AT5G55630.1
Potra2n1c3207.1	-0.250220149	-0.030129257	0.063680983	0.979822671		
Potra2n1c3208.1	-0.64617404	0.120922953	0.033238188	0.945173238	Potri.001G367000.1	AT1G55880.1
Potra2n1c3209.1	-0.412186069	-0.1181497	3.5198E-07	0.741833788	Potri.001G367200.1	AT5G55640.1
Potra2n1c321.2	1.139923869	0.111103943	1.61699E-05	0.943819961	Potri.T166600.1	AT1G16260.1
Potra2n1c3210.3	-0.081653265	0.179582232	0.542005268	0.726986309	Potri.001G367400.2	AT4G26630.1
Potra2n1c3211.1	-1.218504145	0.226588841	7.6442E-05	0.936549944	Potri.001G367500.1	AT4G14540.1
Potra2n1c3212.1	0.178726347	-0.622373406	0.683913191	0.387395572	Potri.001G367700.1	
Potra2n1c3213.1	3.219950245	1.381953902	NA	NA	Potri.011G093400.2	AT5G63180.1
Potra2n1c3214.1	0.786341265	-0.009570262	0.108177428	0.996154416	Potri.001G367900.1	AT5G55730.1
Potra2n1c3215.1	1.651242191	0.088852518	0.556752258	NA		
Potra2n1c3216.1	1.880524222	-0.812879229	NA	0.856337524		
Potra2n1c3217.1	-0.318767761	0.38573633	0.202724953	0.723658895	Potri.001G368000.17	AT5G47650.1
Potra2n1c3218.1	-2.16854629	-1.52302938	0.156945391	0.701287572		
Potra2n1c3219.2	-0.28391282	-0.179458043	0.108491414	0.724604666	Potri.001G368100.1	AT5G55760.1
Potra2n1c322.1	-0.662662248	-0.087404533	0.066950828	0.974367223	Potri.001G038200.1	AT1G79660.1
Potra2n1c3220.1	0.77039726	0.013904297	0.002970051	0.996032145	Potri.001G368200.1	AT5G55820.1
Potra2n1c3221.1	-0.523747528	-0.44950867	0.097741206	0.549979426	Potri.001G368300.2	AT5G55830.1
Potra2n1c3222.1	-0.276105099	-0.259531635	0.163113536	0.83433596	Potri.001G368400.1	AT1G55870.2
Potra2n1c3223.1	-0.284969831	0.1348779	0.062943172	0.911339141	Potri.001G368500.1	AT3G13180.1
Potra2n1c3224.1	0.316795178	0.247643383	0.009153726	0.408808106	Potri.001G368600.1	AT1G55860.2
Potra2n1c3225.4	-0.286981409	0.252553025	0.047700934	0.731968019	Potri.001G368700.1	AT5G55840.1
Potra2n1c3226.1	-0.22552072	0.272958213	0.137635408	0.83405494	Potri.001G368900.1	AT5G55850.3
Potra2n1c3227.2	0.097838843	0.032797365	0.455925969	0.978203092	Potri.001G369000.1	AT3G07680.1
Potra2n1c3228.1	5.495546015	0.298369552	2.2019E-22	NA	Potri.001G369100.1	AT1G55850.1
Potra2n1c3229.1	0.224601318	0.345979115	0.045677363	0.435083833	Potri.001G369200.1	AT5G55860.1
Potra2n1c323.1	7.821431463	5.873978566	1.60098E-09	0.073093992	Potri.001G039200.1	AT1G16130.1
Potra2n1c3230.1	-0.284868875	-0.075345075	0.121832129	0.952773082	Potri.001G369300.1	AT4G26620.1

Potra2n1c3231.1	0.256580362	0.064580584	0.006858099	0.915339763	Potri.001G369400.1	AT1G55840.1
Potra2n1c3232.1	0.17296601	0.262624607	0.843058943	0.964107641		
Potra2n1c3233.1	0.356472212	-0.058195479	0.066355621	0.977754991	Potri.001G369500.1	AT1G60620.1
Potra2n1c3234.1	0.582673286	0.021400493	5.72656E-06	0.99114586	Potri.001G369600.1	AT4G26600.1
Potra2n1c3235.1	2.186450731	1.214896031	0.40792529	NA	Potri.001G369700.1	AT4G26590.1
Potra2n1c3236.1	-0.016597417	0.092408199	0.958058111	0.951394085	Potri.001G369900.1	AT5G66520.1
Potra2n1c3237.1	1.64774763	1.394613675	NA	NA		
Potra2n1c3238.1					Potri.016G096100.1	
Potra2n1c3239.1	0.304973866	0.06894472	0.053985359	0.956865545	Potri.001G370000.1	AT5G55940.1
Potra2n1c324.1	4.106550299	1.775280914	0.001118279	NA	Potri.001G039200.1	AT1G16130.1
Potra2n1c3240.1					Potri.001G369700.1	AT4G26590.1
Potra2n1c3241.1	1.931605898	1.394613675	NA	NA	Potri.001G370100.1	AT4G26590.1
Potra2n1c3242.1						
Potra2n1c3243.1	-0.122482009	0.020739251	0.402112155	0.987288338	Potri.001G370200.2	AT5G55940.1
Potra2n1c3244.1	0.140271315	-0.077667954	0.162522089	0.934439727	Potri.001G370300.2	AT3G13224.2
Potra2n1c3245.1	1.545832463	NA	NA	NA		
Potra2n1c3246.2	-0.208519257	-0.135489432	0.053671465	0.89108297	Potri.001G370400.4	AT3G13200.1
Potra2n1c3247.1	-0.088922894	-0.113609285	0.488306481	0.896878863	Potri.001G370500.1	AT5G55960.1
Potra2n1c3248.1	1.545832463	0.674963689	NA	NA	Potri.001G370600.1	AT3G13220.1
Potra2n1c3249.1	-0.208666122	0.076222524	0.210458757	0.943041425	Potri.001G370700.2	AT1G55830.2
Potra2n1c325.1	3.065319844	1.363811008	NA	NA	Potri.T132900.1	AT1G16130.1
Potra2n1c3250.1	0.078885065	-0.049530686	0.409894544	0.959881296	Potri.001G370900.1	AT3G07660.1
Potra2n1c3251.1	-1.336793218	0.122898849	NA	NA	Potri.001G371000.2	
Potra2n1c3252.1	-0.841837462	-0.138091165	0.000686487	0.953768643	Potri.001G371200.4	AT5G55970.1
Potra2n1c3253.1	NA	0.674963689	NA	NA	Potri.001G371300.1	AT1G45249.1
Potra2n1c3254.1	NA	NA	NA	NA	Potri.001G371400.1	AT5G55980.1
Potra2n1c3255.1	-0.302844374	-0.002465488	0.018702633	0.999325699	Potri.001G371500.2	AT1G30475.1
Potra2n1c3256.1	0.158016267	0.15813925	0.34312899	0.860325259	Potri.001G371600.1	AT3G13225.2
Potra2n1c3257.1	0.099055275	0.086873106	0.485356483	0.896937427	Potri.001G371700.3	AT5G55990.1
Potra2n1c3258.1	0.879072973	-0.061836014	0.041685115	0.968101851	Potri.001G371800.1	AT4G21740.1
Potra2n1c3259.1	0.092852695	0.207138824	0.546581074	0.794641467	Potri.001G371900.1	AT4G05390.1
Potra2n1c326.1	NA	NA	NA	NA		

Potra2n1c3260.1	-0.454935331	0.033608271	0.00289807	0.985964146	Potri.001G372000.1	AT4G21160.4
Potra2n1c3261.1	0.027786192	0.098087812	0.8383069	0.90927817	Potri.001G372100.1	AT1G30500.2
Potra2n1c3262.1						
Potra2n1c3263.1	-0.79770084	-0.03255663	9.24122E-07	0.974275337	Potri.001G372300.1	AT2G34710.1
Potra2n1c3264.1	NA	NA	NA	NA	Potri.001G372400.1	AT4G39330.1
Potra2n1c3265.1	-0.451140478	-0.216443082	0.030842351	0.84801014	Potri.001G372500.1	AT2G45880.1
Potra2n1c3266.1	-0.068051632	-0.083170472	0.588575358	0.896306549	Potri.001G372600.1	AT1G30480.1
Potra2n1c3267.1	NA	2.878256876	NA	NA	Potri.001G372700.1	
Potra2n1c3268.1	-1.503710917	-1.598249918	0.364112579	NA	Potri.010G071200.2	AT1G04400.2
Potra2n1c3269.1	0.102236182	-0.767708831	NA	NA		
Potra2n1c327.1	-1.71555682	-1.995286198	0.716382347	NA	Potri.001G015400.1	AT3G45140.1
Potra2n1c3270.1	0.477948832	0.790073748	0.46863479	0.679485917	Potri.001G372800.1	AT3G15115.1
Potra2n1c3271.1	-1.767831098	-0.870190715	0.2994452	NA	Potri.001G373000.1	
Potra2n1c3272.1	-0.013598001	-0.091055923	0.977481423	0.9789664	Potri.001G373100.1	AT3G15095.1
Potra2n1c3273.1	0.027771592	0.03107217	0.808629155	0.972159498	Potri.001G373300.1	AT3G18380.1
Potra2n1c3274.4	0.1023665	0.001413688	0.457922185	0.999325699	Potri.001G373400.1	AT5G46030.1
Potra2n1c3275.1	0.282132267	0.116857192	0.029712105	0.886988579	Potri.001G373500.2	AT3G15080.1
Potra2n1c3276.1					Potri.002G132800.1	AT2G33580.1
Potra2n1c3277.2	0.474272866	0.206550139	0.564974642	0.966917036	Potri.004G051100.1	AT5G46020.1
Potra2n1c3278.1	0.82357019	NA	NA	NA		
Potra2n1c3279.1						
Potra2n1c328.1	NA	NA	NA	NA	Potri.001G015300.1	AT3G45140.1
Potra2n1c3280.1	-0.176909374	0.001001085	0.084795374	0.999458638	Potri.001G373700.1	AT1G53190.2
Potra2n1c3281.1	0.236854088	-0.038837574	0.069138162	0.974367223	Potri.001G374000.1	AT5G60860.1
Potra2n1c3282.1	1.266533162	0.720990685	0.000106014	0.368305629	Potri.001G373900.1	AT3G48880.2
Potra2n1c3283.1	-0.087107142	0.149555349	0.668654956	0.94494655	Potri.001G374100.2	AT5G54080.1
Potra2n1c3284.1	-1.581222983	-0.458530219	0.017692571	0.908294078	Potri.001G374200.1	AT3G58120.1
Potra2n1c3285.1						
Potra2n1c3286.1	1.366833895	1.947552496	0.627052725	NA	Potri.001G374300.2	AT2G29120.1
Potra2n1c3287.2	-0.087738469	2.083597761	NA	NA	Potri.001G375300.2	AT1G53210.1
Potra2n1c3288.1	-0.527555939	-0.024655633	0.045597133	0.991110595	Potri.001G375500.1	AT1G53210.1
Potra2n1c3289.3	-0.483161044	-1.537084684	0.67662623	NA	Potri.001G375600.1	AT4G27330.1

Potra2n1c329.1	7.635760353	5.376941252	3.61552E-17	0.007318585	Potri.001G039900.1	AT1G16130.1
Potra2n1c3290.1	-0.633293878	-0.00248322	0.004105112	0.999458638	Potri.001G375700.1	AT3G15050.1
Potra2n1c3291.1	-0.363668807	0.175931579	0.096313278	0.802631112	Potri.001G375900.1	AT1G73170.1
Potra2n1c3292.1	-0.192167785	0.207862602	0.151588876	0.766668365	Potri.001G377500.1	AT3G14450.1
Potra2n1c3293.1	NA	NA	NA	NA	Potri.001G377700.1	AT1G78400.1
Potra2n1c3294.1	0.056194757	0.003973066	0.689600964	0.997164503	Potri.001G377800.1	AT1G17130.1
Potra2n1c3295.1	-1.418815977	-0.496586924	2.31765E-11	0.276009559	Potri.001G377900.1	AT1G78430.1
Potra2n1c3296.1	-2.505314564	-0.386808641	0.173342827	NA		
Potra2n1c3297.1	-0.311524131	-0.114965076	0.007419374	0.871720501	Potri.001G378000.2	AT1G78420.2
Potra2n1c3298.1					Potri.001G378100.1	AT2G19520.1
Potra2n1c3299.1						
Potra2n1c33.1	0.261161966	0.127905797	0.128751559	0.833651746	Potri.001G003800.1	AT1G55360.1
Potra2n1c330.1					Potri.001G040400.1	AT5G22250.1
Potra2n1c3300.1	NA	NA	NA	NA		
Potra2n1c3301.1	NA	NA	NA	NA		
Potra2n1c3302.1	NA	NA	NA	NA	Potri.001G378300.1	AT1G17147.1
Potra2n1c3303.1	2.312318886	0.729780895	0.012260486	0.675955768	Potri.001G378400.1	AT1G78440.1
Potra2n1c3304.1	-0.413379002	-0.142276068	0.048186841	0.924951347	Potri.001G378500.1	AT1G17120.1
Potra2n1c3305.1	-0.61910233	-0.767708831	NA	NA	Potri.001G378500.1	AT1G17120.1
Potra2n1c3306.1	7.502157643	3.390498064	5.46486E-09	NA	Potri.001G378700.2	AT3G20180.1
Potra2n1c3307.1	-0.111542387	0.037273906	0.343177133	0.956865545	Potri.001G378900.1	AT1G17110.1
Potra2n1c3308.1	-0.080264701	0.215017838	0.799201718	0.921432293	Potri.001G379000.1	AT1G17100.1
Potra2n1c3309.1	0.386244672	0.390626733	0.05509071	0.825493963	Potri.001G379100.1	
Potra2n1c331.1	-0.120926598	0.251393969	0.626392313	0.816392132	Potri.001G041200.1	AT1G16080.1
Potra2n1c3310.1	-1.185898075	0.122422959	0.032862289	0.974367223	Potri.001G379300.2	
Potra2n1c3311.1	-1.954357138	-0.620032556	1.82229E-07	0.684244573	Potri.001G379400.2	
Potra2n1c3312.1	-1.84567096	-0.530194888	5.85338E-07	0.779077802	Potri.001G379400.2	
Potra2n1c3313.1	0.27554464	0.109148125	0.246727482	0.945150162	Potri.001G379500.1	AT3G14580.1
Potra2n1c3314.1	0.858574539	0.238832466	0.028156293	0.759797728	Potri.001G379800.2	AT3G14590.2
Potra2n1c3315.1	4.406366499	2.676364087	0.078090623	NA	Potri.001G380000.1	AT3G62730.1
Potra2n1c3316.1	NA	2.31063453	NA	NA	Potri.001G380000.1	AT3G62730.1
Potra2n1c3317.2	-0.10838057	-0.025355363	0.340998432	0.977323635	Potri.003G023600.1	AT5G64160.1

Potra2n1c3318.2	0.228792735	0.304071923	0.028477076	0.22650515	Potri.003G023500.1	AT1G36050.2
Potra2n1c3319.1	0.635522341	0.645754437	0.559007526	0.903769998	Potri.006G120900.1	
Potra2n1c332.1	1.932658089	1.763177905	3.56877E-18	NA		
Potra2n1c3320.1	0.158443363	0.082865632	0.963184973	NA	Potri.003G023400.2	
Potra2n1c3321.1	-1.42531687	-0.339309554	0.52687245	NA	Potri.001G380000.1	AT3G62730.1
Potra2n1c3322.1	-0.261703678	-0.114859079	0.003833617	0.85266401	Potri.001G380100.1	AT1G53580.1
Potra2n1c3323.1	-0.98126408	0.045691545	4.15564E-06	0.9761362		
Potra2n1c3324.1	0.193383073	-0.132524265	0.183304536	0.843683654	Potri.001G380500.1	AT1G78510.1
Potra2n1c3325.1	0.974346634	1.094555228	1.46226E-05	0.282619136		
Potra2n1c3326.1	0.914743867	1.085035548	4.8639E-05	0.289079975	Potri.001G380600.2	AT1G78520.1
Potra2n1c3327.1	2.853474997	0.897776182	0.000321353	0.869175327	Potri.001G380700.2	AT1G53520.1
Potra2n1c3328.1	2.533366429	0.439743072	0.000308793	0.945454119	Potri.001G380800.1	AT1G78530.1
Potra2n1c3329.1	-0.273035497	0.013438629	0.664682657	0.99702095		
Potra2n1c333.1	1.933890384	1.756975182	3.08159E-18	NA	Potri.001G041300.1	AT3G09390.1
Potra2n1c3330.1	-0.448643606	-0.007787059	0.028083079	0.996032145	Potri.001G380900.1	AT1G78540.1
Potra2n1c3331.1	1.873287167	0.978003873	0.000250125	0.475435867	Potri.001G381000.1	AT4G16580.1
Potra2n1c3332.1	-2.337106966	-2.486725844	NA	NA	Potri.001G381100.1	
Potra2n1c3333.1	-0.031212071	0.292363124	0.823328453	0.776440978	Potri.001G381300.1	AT3G14720.1
Potra2n1c3334.1	-0.949293169	-0.690497904	0.473988737	0.921065381	Potri.001G381400.2	AT1G17030.1
Potra2n1c3335.1	-1.908334414	-0.641097897	0.000388832	0.751295646	Potri.001G381500.2	AT1G79450.1
Potra2n1c3336.1	-1.544730242	-1.725740889	0.5866381	NA	Potri.001G381600.1	AT1G17030.1
Potra2n1c3337.1	1.309491819	0.385101824	2.64935E-12	0.561435202	Potri.001G381700.1	AT1G17020.1
Potra2n1c3338.1	6.515688584	1.791860013	4.37177E-07	NA	Potri.001G381900.1	AT1G68470.1
Potra2n1c3339.1	-1.951691612	-3.465481486	0.582233419	NA	Potri.002G221800.1	
Potra2n1c334.1	-0.358738052	-0.696716475	0.522679717	0.693793454	Potri.001G041400.1	AT1G16070.1
Potra2n1c3340.1	4.959657335	1.563789464	5.20418E-48	0.564019611	Potri.001G382400.1	AT1G17020.1
Potra2n1c3341.1	0.152462134	-1.55593228	NA	NA		
Potra2n1c3342.1	0.152462134	-1.55593228	NA	NA		
Potra2n1c3343.1	1.65646305	0.248901791	0.00344232	0.962696825	Potri.001G382500.1	AT3G14740.2
Potra2n1c3344.2	0.074323879	0.062301569	0.485802886	0.943819961	Potri.001G382700.1	AT3G14750.1
Potra2n1c3345.1	1.329624442	-0.066852884	NA	NA	Potri.001G382800.1	AT3G14760.1
Potra2n1c3346.1	0.610420983	0.106788688	0.004641083	0.926234768	Potri.001G383000.11	AT3G14770.1

Potra2n1c3347.1	0.272930597	-0.153768476	0.013782639	0.885375133	Potri.001G383500.1	AT1G78570.1
Potra2n1c3348.1	0.279975556	-0.367217704	0.336050156	0.704971683	Potri.001G383600.1	AT1G78580.1
Potra2n1c3349.1	1.851247165	0.597995405	6.22744E-05	0.849446235	Potri.001G383700.1	
Potra2n1c335.1	1.585878641	1.029671467	0.09083642	0.788065047	Potri.001G041500.1	AT1G79700.2
Potra2n1c3350.3	-0.101398318	0.194586065	0.497688903	0.824378527	Potri.012G059200.1	AT1G18560.1
Potra2n1c3351.3	0.830384606	0.235806098	0.088520674	0.935130474	Potri.001G383900.10	AT1G53490.1
Potra2n1c3352.1	-0.319351933	-0.170617301	0.043431909	0.798673448	Potri.001G384000.1	AT1G78600.1
Potra2n1c3353.1	-0.144689222	-0.13666108	0.449932433	0.895129305		
Potra2n1c3354.1	-1.357728288	-0.463511952	0.000462307	0.775391304	Potri.001G384100.1	AT1G78610.1
Potra2n1c3355.1	0.471694871	0.036197158	0.000898833	0.978646026	Potri.001G384300.1	AT1G78620.1
Potra2n1c3356.1	-2.907673959	-1.133336615	NA	NA	Potri.001G384200.1	AT3G49180.1
Potra2n1c3357.1	0.588881474	0.127115994	0.049457342	0.952554996		
Potra2n1c3358.1	0.878702723	0.110081123	NA	NA	Potri.004G232100.1	
Potra2n1c3359.1					Potri.001G038800.1	
Potra2n1c336.1	NA	NA	NA	NA	Potri.008G209800.1	AT3G16510.1
Potra2n1c3360.1	0.228314081	-0.066229438	0.367014334	0.97064682	Potri.001G384600.1	AT1G78630.1
Potra2n1c3361.1	-0.388229828	1.245400238	0.861465919	NA		
Potra2n1c3362.1	1.714354145	0.602608125	0.031662592	0.911503812	Potri.001G385200.1	AT3G14840.2
Potra2n1c3363.1	NA	NA	NA	NA		
Potra2n1c3364.1	-0.149931388	0.110322284	0.677843426	0.953108412	Potri.001G385200.1	AT3G14840.2
Potra2n1c3365.1	0.891761716	0.168479509	0.001046917	0.910815461	Potri.001G385300.1	AT1G53440.1
Potra2n1c3366.1	6.554188972	0.488753927	1.18605E-11	NA	Potri.001G385400.1	AT1G53430.1
Potra2n1c3367.2	1.509209413	0.807905652	0.005133895	0.767368598	Potri.001G385300.1	AT1G53440.1
Potra2n1c3368.1	-1.328951947	-1.478536539	NA	NA	Potri.001G385200.1	AT3G14840.2
Potra2n1c3369.1	-0.327744244	0.352298316	0.567834436	0.849446235	Potri.001G384700.2	AT3G14840.2
Potra2n1c337.1	0.579176839	0.143078699	0.004366578	0.813731863	Potri.001G041600.1	AT1G79710.1
Potra2n1c3370.1						
Potra2n1c3371.5	6.2881968	1.881728551	8.72056E-07	NA	Potri.001G386500.2	AT1G53440.1
Potra2n1c3372.1	-0.549771379	-0.147753938	9.80496E-05	0.899757743	Potri.003G026500.1	AT3G14830.1
Potra2n1c3373.1	-1.240635441	-0.647259157	2.36723E-05	0.637188449	Potri.003G026600.1	AT1G78700.1
Potra2n1c3374.1	1.277582105	1.417778081	0.011561448	0.417067084	Potri.001G387000.3	
Potra2n1c3375.2	0.085475255	-0.019403622	0.590715424	0.985393296	Potri.003G026800.4	AT1G78680.1

Potra2n1c3376.1	0.147883306	0.105539316	0.346194857	0.908764369		
Potra2n1c3377.1	0.725075385	-0.132661597	0.40832384	0.983446961	Potri.001G387200.1	
Potra2n1c3378.1	-0.392175728	-0.107854021	0.000389183	0.92267921	Potri.001G387300.1	AT1G78650.1
Potra2n1c3379.1	-1.599149547	-0.407794694	0.320642673	0.956773505		
Potra2n1c338.1	0.616414182	0.11592193	0.006590141	0.864110603	Potri.001G041700.1	AT1G79720.1
Potra2n1c3380.2	-0.439273561	-0.111038528	0.000129197	0.936890993	Potri.001G387400.1	AT1G53400.1
Potra2n1c3381.1	-0.944618153	0.479778171	0.765326999	NA		
Potra2n1c3382.1	-0.126031732	-0.008693838	0.562680743	0.996032145	Potri.001G387500.1	AT3G14860.2
Potra2n1c3383.1					Potri.003G087350.1	
Potra2n1c3384.1	0.258445108	0.02372145	0.056218994	0.987288338	Potri.001G387600.2	AT2G34250.1
Potra2n1c3385.1	-1.570507997	-0.787828378	1.72864E-07	0.524846781	Potri.001G387700.1	AT1G53380.1
Potra2n1c3386.1	-0.224001548	-0.081773921	0.834959771	0.99114586	Potri.T062200.1	
Potra2n1c3387.5	0.183768543	0.295417366	0.664413424	0.835819291	Potri.001G385200.1	AT3G14840.2
Potra2n1c3388.5	2.050245314	0.78238896	1.77359E-07	0.663037599	Potri.001G385300.1	AT1G53440.1
Potra2n1c3389.1	0.703884283	-0.273027579	0.513341638	NA		
Potra2n1c339.1	0.02218504	0.34401308	0.942265874	0.691708217	Potri.001G041800.10	AT2G24100.1
Potra2n1c3390.1	-0.607612345	2.027935684	NA	NA	Potri.001G384700.2	AT3G14840.2
Potra2n1c3391.1	0.258621115	0.693362004	0.800435111	0.84801014	Potri.001G384700.2	AT3G14840.2
Potra2n1c3392.1	1.322450216	0.584614429	3.72414E-08	0.554040943	Potri.001G388800.1	
Potra2n1c3393.1	2.682479554	0.607082727	6.24559E-26	0.808630999	Potri.001G388900.1	
Potra2n1c3394.1	2.451780877	2.166529345	0.049400514	0.523342013	Potri.001G389200.1	AT1G05680.1
Potra2n1c3395.1	-0.734866214	-0.48368777	0.725313168	NA	Potri.001G389300.1	AT1G78770.1
Potra2n1c3396.1	0.603845448	0.310844265	0.011806188	0.692544813	Potri.001G389300.1	AT1G78770.1
Potra2n1c3397.1	0.904574977	0.371429089	0.026753465	0.887099891	Potri.001G389800.1	AT1G78780.2
Potra2n1c3398.1	NA	2.134902029	NA	NA	Potri.001G389400.1	AT1G78780.2
Potra2n1c3399.1					Potri.001G390000.1	AT1G78800.1
Potra2n1c34.1	-0.142086898	-0.010270281	0.2540614	0.992934677	Potri.001G003900.3	AT1G55350.3
Potra2n1c340.1	-0.041240334	0.141178513	0.874562823	0.897642615	Potri.001G041900.1	AT1G80940.1
Potra2n1c3400.1						
Potra2n1c3401.1	NA	NA	NA	NA	Potri.001G390100.1	AT3G14880.2
Potra2n1c3402.1	0.620324595	0.1009748	1.16817E-06	0.900551258	Potri.001G390200.1	AT1G53320.1
Potra2n1c3403.1	0.06717958	-0.082245948	0.618660032	0.920763566	Potri.001G390400.2	AT3G58680.1

Potra2n1c3404.1	0.584509709	0.437537735	0.000131747	0.204120601	Potri.001G390500.2	AT3G14890.1
Potra2n1c3405.1	-0.041838927	-0.249936553	0.909353922	0.882657052	Potri.001G390600.1	AT3G14900.1
Potra2n1c3406.1	-0.013246892	0.037703428	0.94466355	0.974275337	Potri.001G390700.3	AT3G14910.1
Potra2n1c3407.1	0.002361678	-0.006492586	0.993258041	0.996032145	Potri.001G390800.1	AT3G14930.1
Potra2n1c3408.1	-2.337106966	-1.07654148	NA	NA		
Potra2n1c3409.1						
Potra2n1c341.1	-0.173115854	-0.393973517	0.618041385	0.779493734	Potri.001G042000.1	AT5G57820.1
Potra2n1c3410.1	-0.270508981	0.098649122	0.338940203	0.956856444	Potri.001G390900.1	AT1G78815.1
Potra2n1c3411.2	NA	1.381953902	NA	NA		
Potra2n1c3412.1	-1.738519292	-0.578963632	0.045300942	0.888248505	Potri.001G391000.1	AT1G78850.1
Potra2n1c3413.1	NA	1.394613675	NA	NA	Potri.001G391100.1	AT4G13010.1
Potra2n1c3414.1	NA	2.86450839	NA	NA		
Potra2n1c3415.1	0.940084169	-3.620742322	0.68310118	NA	Potri.001G391300.1	AT1G78860.1
Potra2n1c3416.1	0.917001607	0.095511821	5.2884E-05	0.959009636	Potri.001G391400.1	AT1G31860.1
Potra2n1c3417.2	0.020135691	0.152044736	0.906021949	0.771626158	Potri.001G391900.6	AT3G14940.1
Potra2n1c3418.1	-1.117740273	-0.078595862	0.07303791	0.981772014	Potri.011G110800.1	AT1G16900.1
Potra2n1c3419.1	-1.702685582	0.503391561	0.149177737	0.903698951		
Potra2n1c342.1	-0.592903734	-0.422773106	0.810833681	NA	Potri.001G042100.1	
Potra2n1c3420.4	-0.04200607	-0.090882108	0.807009658	0.937036471	Potri.001G392200.1	AT2G42590.2
Potra2n1c3421.1	-0.529080615	-0.312345641	0.388742036	0.941855873	Potri.001G392300.2	AT3G50940.1
Potra2n1c3422.1	NA	2.906556465	NA	NA	Potri.001G392400.1	AT1G29140.1
Potra2n1c3423.1	-0.032485339	-0.004320532	0.771458017	0.996134356	Potri.011G111400.1	AT1G78870.2
Potra2n1c3424.1						
Potra2n1c3425.1	NA	1.571529889	NA	NA		
Potra2n1c3426.1	-0.096567999	0.006125152	0.590493387	0.99736971	Potri.001G392600.2	AT1G53300.1
Potra2n1c3427.1	-0.79736527	1.616567551	NA	NA		
Potra2n1c3428.1	-0.630370019	-0.153618191	0.001709818	0.915358268	Potri.001G392700.1	AT1G53290.1
Potra2n1c3429.1	-0.364126694	0.021605823	0.017341136	0.98177802	Potri.001G392801.1	AT3G14980.1
Potra2n1c343.1	-2.117215685	-0.179911384	0.026801459	0.982313228	Potri.001G042200.1	
Potra2n1c3430.1	1.245744892	1.244202865	NA	NA		
Potra2n1c3431.1	-1.409983009	-1.55593228	NA	NA		
Potra2n1c3432.1	2.549191961	NA	NA	NA	Potri.001G393101.1	

Potra2n1c3433.1	0.957594022	-0.05501709	0.18308184	0.99114586	Potri.001G393200.3	AT1G16670.1
Potra2n1c3434.2	2.101189164	0.06197156	0.444032936	NA	Potri.001G393300.1	AT1G53270.1
Potra2n1c3435.1	0.147041076	-0.187701742	0.422087479	0.874595634	Potri.001G393400.4	AT3G15000.1
Potra2n1c3436.1	-0.832565521	-0.241233932	0.035008885	0.9045874	Potri.001G393700.2	AT1G53250.1
Potra2n1c3437.1	0.792184919	-0.035785987	0.374614248	0.996032145	Potri.001G393800.1	AT3G14440.1
Potra2n1c3438.1					Potri.001G393900.1	
Potra2n1c3439.1	0.182047036	0.041737795	0.283786867	0.972159498	Potri.001G394000.1	AT1G31660.1
Potra2n1c344.1	NA	NA	NA	NA	Potri.001G042300.1	AT3G50310.1
Potra2n1c3440.1	-0.423446627	-0.490485156	0.074549976	0.564019611	Potri.001G394200.1	AT4G20010.1
Potra2n1c3441.1	-0.194749456	-0.482206592	0.507455964	0.642174814		
Potra2n1c3442.1	0.096188416	0.130843489	0.418254764	0.779493734	Potri.001G394300.2	AT1G16570.1
Potra2n1c3443.1	-2.939214643	-0.812015471	5.12942E-10	0.624602712	Potri.001G394400.1	AT3G14420.2
Potra2n1c3444.1	0.160856991	0.02876676	0.519491103	0.984292161	Potri.001G394500.1	AT3G14410.1
Potra2n1c3445.1	-0.017535941	0.026521036	0.89939066	0.966407607	Potri.001G394600.1	AT3G14400.1
Potra2n1c3446.1	NA	1.900652309	NA	NA	Potri.001G394800.1	AT1G17180.1
Potra2n1c3447.1	NA	NA	NA	NA		
Potra2n1c3448.1	-1.94743302	-0.380781957	0.120518728	0.953831244	Potri.014G138700.1	AT2G48160.1
Potra2n1c3449.1						
Potra2n1c345.1	-1.538966482	-0.680566024	4.13883E-10	0.131157963	Potri.003G184200.1	AT4G36950.1
Potra2n1c3450.1	8.071041727	4.39332719	5.86556E-11	NA		
Potra2n1c3451.1	-3.45935264	-1.030797372	0.016095298	0.823250234	Potri.001G395100.1	AT5G53980.1
Potra2n1c3452.1	-1.433400911	-0.81479364	7.50674E-05	0.269417208	Potri.001G395200.1	AT2G20562.1
Potra2n1c3453.1						
Potra2n1c3454.1	1.060351844	-2.033943134	0.696074928	NA	Potri.001G395700.2	AT5G39080.1
Potra2n1c3455.1					Potri.011G121800.1	AT3G15420.1
Potra2n1c3456.1						
Potra2n1c3457.1	1.522965574	-0.767708831	NA	NA		
Potra2n1c3458.1	-0.595952689	-1.465929376	NA	NA	Potri.001G395700.2	AT5G39080.1
Potra2n1c3459.1	2.047788537	1.434369124	0.000639404	0.401650443	Potri.001G395700.2	AT5G39080.1
Potra2n1c346.1	-2.083483469	-0.487534714	1.1311E-09	0.137620511		
Potra2n1c3460.1	3.548357597	1.807896028	0.024626694	0.824448251	Potri.001G395700.2	AT5G39080.1
Potra2n1c3461.1	2.802167814	1.743944294	8.47128E-13	0.116771876	Potri.001G397200.1	AT3G15210.1

Potra2n1c3462.1	1.931605898	NA	NA	NA		
Potra2n1c3463.1	-0.017666032	-0.050145929	0.947989862	0.977559521	Potri.001G396900.1	AT4G27540.1
Potra2n1c3464.1	1.134987255	0.62156784	0.178756203	0.84487051	Potri.001G396800.1	AT3G07870.1
Potra2n1c3465.1	-0.9919942	0.053382273	0.029697644	0.985393296	Potri.001G396700.1	AT5G18790.1
Potra2n1c3466.1	-0.218313428	-0.257016092	0.309162008	0.736149483	Potri.001G396600.3	AT3G15190.1
Potra2n1c3467.1	0.45903635	0.19185345	1.5303E-06	0.732556792	Potri.001G396500.2	AT3G15180.1
Potra2n1c3468.1	NA	NA	NA	NA	Potri.001G396300.1	AT5G53950.1
Potra2n1c3469.1	-0.110661545	-0.311639911	0.602551082	0.562306981	Potri.001G396100.1	AT2G33845.1
Potra2n1c347.1	-2.096434009	-0.488456253	9.17825E-10	0.135796543	Potri.001G042500.1	AT2G24130.1
Potra2n1c3470.1	NA	2.676364087	NA	NA	Potri.001G396800.1	AT3G07870.1
Potra2n1c3471.1	NA	0.674963689	NA	NA		
Potra2n1c3472.1	-0.444735917	-0.204711602	0.003632127	0.802942736	Potri.001G397600.2	AT3G15220.1
Potra2n1c3473.1	1.744227452	0.099476425	9.25713E-06	0.978251387	Potri.001G397800.1	AT5G53900.2
Potra2n1c3474.1	-3.017449429	-1.126339717	0.185567249	NA	Potri.001G398000.1	AT1G53163.1
Potra2n1c3475.1	-2.289908956	-0.997471886	0.372538924	NA		
Potra2n1c3476.1	0.393417289	-0.057475078	0.001153248	0.951754672	Potri.001G398100.1	AT3G15260.2
Potra2n1c3477.1	-3.570204057	-0.379646001	0.044179969	0.972402328	Potri.001G398200.1	AT3G15270.1
Potra2n1c3478.1	0.357536474	0.369396886	0.226294325	0.653123964		
Potra2n1c3479.1	-1.116918551	-0.156266234	0.002581502	0.960981446		
Potra2n1c348.1	-1.042408623	0.377279755	0.000686255	0.701494362	Potri.001G042600.1	AT3G12580.1
Potra2n1c3480.2	-0.413307301	-0.14969683	0.003132688	0.821396711	Potri.001G398600.1	AT3G15290.1
Potra2n1c3481.1	3.243794422	1.027956142	8.84295E-27	0.098336438	Potri.001G398800.1	AT5G53870.1
Potra2n1c3482.1						
Potra2n1c3483.1	-0.20242489	0.064017537	0.322953267	0.954098985	Potri.001G398900.1	AT5G53860.2
Potra2n1c3484.1	0.150866429	0.113380638	0.156899436	0.81916677	Potri.001G399000.1	AT5G53850.2
Potra2n1c3485.1	-0.508551945	0.298538975	0.052877579	0.870875305	Potri.001G399100.1	AT1G28280.2
Potra2n1c3486.1	NA	NA	NA	NA	Potri.010G225900.1	
Potra2n1c3487.1	NA	NA	NA	NA		
Potra2n1c3488.1	NA	NA	NA	NA		
Potra2n1c3489.1	0.151463785	0.245113019	0.126093741	0.220559393	Potri.001G399400.2	AT4G27500.1
Potra2n1c349.1	-0.72794979	0.607857945	0.245244623	0.83404888	Potri.001G042700.1	AT1G16030.1
Potra2n1c3490.1	-0.323318856	0.044761285	0.118305493	0.978251387	Potri.001G399500.1	AT3G15350.2

Potra2n1c3491.2	-0.244183434	0.087025411	0.008038945	0.926478129	Potri.001G399600.10	AT3G15354.1
Potra2n1c3492.1	-0.310386847	-0.082271247	0.017347776	0.90226843	Potri.001G399700.1	AT1G53050.1
Potra2n1c3493.1	-0.199727056	0.001117511	0.314515247	0.999536469	Potri.001G399800.1	AT1G53040.2
Potra2n1c3494.1	0.088025195	-0.149366195	0.675595198	0.881552217	Potri.001G400000.2	AT3G15352.1
Potra2n1c3495.1	-1.934338976	0.191486014	2.68512E-11	0.947969749	Potri.001G400100.5	AT3G15353.1
Potra2n1c3496.1					Potri.015G080600.1	AT5G03340.1
Potra2n1c3497.1	0.211740551	0.170374267	0.013991376	0.743612955	Potri.001G400300.3	AT1G53025.1
Potra2n1c3498.1	0.801881481	-0.060135345	0.010441222	0.987226805	Potri.001G400401.1	
Potra2n1c3499.1					Potri.001G400700.1	
Potra2n1c35.3	-0.478341447	-0.233972711	5.9654E-05	0.534000676	Potri.001G004000.1	AT1G55340.1
Potra2n1c350.1	0.216883388	-0.181684835	0.365703862	0.922993749	Potri.001G042800.1	
Potra2n1c3500.1	-0.110688514	0.053233349	0.450218204	0.953108412	Potri.001G400800.1	AT5G53770.1
Potra2n1c3501.1	-0.089450857	0.020109478	0.555449023	0.991482109	Potri.001G400900.5	AT1G53000.1
Potra2n1c3502.1	-0.823687201	-0.039440571	0.052505789	0.991398092	Potri.001G401300.1	AT5G60020.1
Potra2n1c3503.1	6.188857146	3.59813173	0.000118107	NA	Potri.001G401400.1	AT1G23220.1
Potra2n1c3504.1	0.143355228	-0.01089036	0.279824893	0.992934677	Potri.001G401500.3	AT3G15360.1
Potra2n1c3505.1					Potri.001G401700.1	AT3G15370.1
Potra2n1c3506.1	-5.231703482	-1.139458565	0.010738528	0.907073354	Potri.001G401800.1	AT4G29280.1
Potra2n1c3507.1	0.254016092	-0.013950548	0.090828916	0.992934677	Potri.001G402000.1	AT3G15395.3
Potra2n1c3508.1	-0.587051704	0.543142966	0.694786364	0.903477395	Potri.001G402100.1	
Potra2n1c3509.1	0.200639425	0.088003728	0.410593118	0.948002367	Potri.001G402400.1	AT5G53760.1
Potra2n1c351.1	0.200161553	-0.063882342	0.333155273	0.97950444	Potri.001G042900.1	AT5G48930.1
Potra2n1c3510.1	0.568097974	0.229670538	7.22114E-12	0.565491408	Potri.001G402500.1	AT1G52980.1
Potra2n1c3511.1	-1.488761069	-0.168028327	0.006648409	0.953831244	Potri.001G402600.2	AT1G07750.1
Potra2n1c3512.1	-0.33587431	-0.070283298	0.003220396	0.930598202	Potri.001G402800.1	AT3G15430.2
Potra2n1c3513.1	-0.17896622	-0.085986506	0.52594133	0.966917036		
Potra2n1c3514.1	-0.092439301	0.138074096	0.723629442	0.921065381	Potri.001G403000.1	AT4G27450.1
Potra2n1c3515.1	0.407483668	0.212231068	0.001278493	0.79393535	Potri.001G403100.1	AT3G15460.1
Potra2n1c3516.1	0.057198923	0.142128934	0.800435111	0.900629215	Potri.001G403200.1	AT1G52920.1
Potra2n1c3517.1	-1.021928254	-0.562127294	0.000197055	0.454005567	Potri.001G403300.2	AT5G54190.1
Potra2n1c3518.1	-0.232429633	0.058234117	0.04745786	0.936549944	Potri.001G403400.2	AT5G54200.1
Potra2n1c3519.1	1.664677056	0.674963689	NA	NA		

Potra2n1c352.1	-0.295268837	0.286156184	0.060108144	0.837085163	Potri.001G043000.2	AT4G31860.1
Potra2n1c3520.2	-0.011242084	-0.040383777	0.941219977	0.9761362	Potri.001G403500.1	AT3G15480.1
Potra2n1c3521.1	-0.834839741	-0.354471794	3.24653E-05	0.760398319	Potri.001G403600.1	AT3G15480.1
Potra2n1c3522.1	-0.36559398	-0.147157631	0.045859831	0.886633917	Potri.001G403700.1	AT4G27430.2
Potra2n1c3523.1	2.632524198	NA	NA	NA	Potri.001G403800.1	AT1G52900.1
Potra2n1c3524.1	2.918246155	0.565085725	1.68131E-08	0.930871994		
Potra2n1c3525.1	-1.145910299	0.04212685	0.107041188	0.992934677	Potri.001G402600.2	AT1G07750.1
Potra2n1c3526.2	1.16841784	0.528934131	1.29465E-17	0.297164129	Potri.001G403900.1	AT1G61810.3
Potra2n1c3527.1	-1.321532124	-0.693487637	NA	NA	Potri.001G404000.1	AT4G27420.1
Potra2n1c3528.1	1.217139367	0.316368677	0.222170923	0.947969749	Potri.001G404100.1	AT4G27410.2
Potra2n1c3529.1	0.82357019	NA	NA	NA		
Potra2n1c353.1	0.197329597	1.381564062	0.640757564	0.723658895	Potri.001G043100.1	AT1G80920.1
Potra2n1c3530.1	0.193726908	-0.139756407	0.544287373	0.930203195	Potri.001G404400.1	AT3G15510.1
Potra2n1c3531.1	-0.353938859	-0.331885731	0.472159116	0.854980698	Potri.001G404500.1	AT3G15518.1
Potra2n1c3532.2	0.696609816	0.53742486	0.000368892	0.660456866	Potri.001G404600.1	AT1G52870.2
Potra2n1c3533.1	0.53228086	0.312522188	0.201520964	0.857189843	Potri.001G404700.1	AT3G15520.1
Potra2n1c3534.1	0.007824806	-0.131688889	0.980963321	0.939387548	Potri.001G405200.1	AT5G54400.1
Potra2n1c3535.1					Potri.001G308500.1	
Potra2n1c3536.1	-0.354623744	-0.546579063	0.590342093	0.829742539	Potri.001G405500.1	AT5G54380.1
Potra2n1c3537.1	NA	NA	NA	NA	Potri.T072800.1	
Potra2n1c3538.1	-0.181296889	-0.000279972	0.884628951	0.999937671	Potri.001G405500.1	AT5G54380.1
Potra2n1c3539.1	NA	NA	NA	NA	Potri.001G405600.1	
Potra2n1c354.5	0.088160382	-0.116273462	0.588920571	0.921099436	Potri.001G043200.1	AT1G16010.2
Potra2n1c3540.1	-0.198016718	0.27041285	0.904382252	0.9761362	Potri.011G127900.1	AT5G54370.1
Potra2n1c3541.3	0.109812578	0.19184855	0.888521791	0.953177702	Potri.001G405700.1	AT4G21570.1
Potra2n1c3542.1	0.517283011	0.295075322	0.001044655	0.520533674	Potri.001G405800.2	AT4G27390.1
Potra2n1c3543.1	1.982186483	1.02857824	0.103904126	NA		
Potra2n1c3544.1	NA	0.674963689	NA	NA		
Potra2n1c3545.1	-0.496610056	-0.128027532	0.038872892	0.88221216	Potri.001G406200.3	AT3G13677.2
Potra2n1c3546.4	0.13888403	0.273099944	0.472944964	0.733263164	Potri.001G406300.1	AT5G54310.1
Potra2n1c3547.1	0.460935001	0.476702435	0.068004858	0.597163621	Potri.017G135500.1	AT3G02860.2
Potra2n1c3548.1	-0.442170236	-0.085909463	0.000365857	0.936890993	Potri.001G406500.1	AT5G03610.1

Potra2n1c3549.1	-0.246458073	0.00716101	0.464008401	0.997932292	Potri.001G406600.1	AT1G61260.1
Potra2n1c355.1	-0.013590549	-0.009090183	0.965315424	0.995579852	Potri.001G043300.1	AT1G16000.1
Potra2n1c3550.1	-0.28816741	-0.144851157	0.160505429	0.894513758	Potri.001G406700.1	AT5G54290.2
Potra2n1c3551.1	0.658072135	0.255024923	0.546560872	0.97137556		
Potra2n1c3552.1	0.28353632	-0.121988798	0.053795114	0.912777843	Potri.001G406900.1	AT4G27380.1
Potra2n1c3553.1	0.25389111	-0.126401655	0.102929424	0.912777843		
Potra2n1c3554.5	-0.738371184	0.196492034	6.98802E-05	0.871585666	Potri.001G407000.1	AT5G54280.2
Potra2n1c3555.1	-0.78353047	-0.087065301	6.898E-08	0.941260209	Potri.001G407100.1	AT5G54270.1
Potra2n1c3556.1	-0.204619156	-0.288946662	0.470251054	0.833401866	Potri.001G407200.3	
Potra2n1c3557.1	-0.18530514	0.082934542	0.316083818	0.922993749	Potri.001G407300.1	AT5G54260.1
Potra2n1c3558.1	1.675247424	1.688262039	0.000483146	0.013017322	Potri.001G407400.1	
Potra2n1c3559.1	0.125015879	0.57655546	0.76499063	0.647178693	Potri.001G407400.1	
Potra2n1c356.1	0.370266673	0.153286415	0.018636826	0.856645555	Potri.001G043400.5	AT1G80500.1
Potra2n1c3560.1	0.443265114	0.046330902	0.487492302	0.992934677	Potri.001G407800.1	AT5G54250.1
Potra2n1c3561.1	-2.396052862	-0.167632542	NA	NA	Potri.001G407900.1	AT4G27360.1
Potra2n1c3562.1	NA	2.283425176	NA	NA	Potri.001G408000.1	AT5G54240.1
Potra2n1c3563.1	0.474667154	0.286209318	0.003049671	0.497568278	Potri.001G408200.1	AT5G54110.1
Potra2n1c3564.1	1.707515872	0.269133536	9.66411E-08	0.900869139	Potri.001G408500.1	AT2G20900.2
Potra2n1c3565.1	1.450131076	-1.303823209	0.264293163	NA	Potri.001G408700.1	AT4G21440.1
Potra2n1c3566.1	-0.230390426	-0.10299898	0.03583115	0.88661378	Potri.001G408800.1	AT3G20630.1
Potra2n1c3567.1	-0.325693285	-0.074877914	0.517492937	0.985136323	Potri.001G408900.1	AT5G08240.1
Potra2n1c3568.1	1.664677056	NA	NA	NA	Potri.001G414900.1	
Potra2n1c3569.2	-0.445574039	-0.225296637	0.001452718	0.759797728	Potri.001G409100.1	AT1G11360.3
Potra2n1c357.1	-0.418472165	-0.120631693	0.034550696	0.935851032	Potri.001G043500.1	AT1G80880.1
Potra2n1c3570.1	-0.47023136	0.073229686	0.312797527	0.985393296	Potri.001G414700.1	AT5G54470.1
Potra2n1c3571.1	0.258166882	0.53741771	0.935011146	NA	Potri.001G414600.1	AT4G27280.1
Potra2n1c3572.1	-0.906948893	-3.508399403	0.644154811	NA	Potri.001G409300.1	AT4G21380.1
Potra2n1c3573.1	0.188087229	-2.626448277	0.935011146	NA		
Potra2n1c3574.1	3.980948459	-0.472712432	1.27498E-12	0.947290893	Potri.001G409300.1	AT4G21380.1
Potra2n1c3575.1	4.219423593	0.259811907	3.52876E-12	0.978251387	Potri.001G414200.2	AT4G21380.1
Potra2n1c3576.1					Potri.001G414100.1	
Potra2n1c3577.1	0.963323479	1.137398747	0.210241965	0.643558659	Potri.001G414000.1	AT4G27290.1

Potra2n1c3578.1	-1.908795789	-1.337627464	NA	NA	Potri.003G176000.1	AT4G13700.1
Potra2n1c3579.1	-1.43721255	0.258085735	0.117434088	0.952517602	Potri.003G176000.1	AT4G13700.1
Potra2n1c358.1	-0.433758026	-0.074285055	0.096925002	0.972159498	Potri.001G043700.1	AT1G80870.1
Potra2n1c3580.1	NA	NA	NA	NA	Potri.001G413866.1	AT4G27300.1
Potra2n1c3581.1	5.350766736	NA	0.00092474	NA	Potri.001G413800.1	AT4G27290.1
Potra2n1c3582.1	1.926062597	0.38676615	1.45352E-11	0.867540758		
Potra2n1c3583.1	1.988974681	0.388165519	1.03216E-15	0.805052871	Potri.001G413700.1	AT4G27290.1
Potra2n1c3584.1	0.066757379	-0.196619845	0.698991314	0.752940067	Potri.001G413500.1	AT2G31670.1
Potra2n1c3585.1	-1.044850582	-0.347456577	1.53762E-11	0.522777488	Potri.001G412900.1	AT4G34240.1
Potra2n1c3586.1	0.04632435	0.17201513	0.781315993	0.810712263	Potri.001G412800.1	AT2G36740.1
Potra2n1c3587.1	1.664677056	NA	NA	NA		
Potra2n1c3588.1	0.863720788	0.301018172	0.213316475	0.943411075		
Potra2n1c3589.1	0.761973973	0.253004411	1.09263E-06	0.79819686	Potri.001G412000.1	AT4G27290.1
Potra2n1c359.1	2.701721146	0.806281629	3.11693E-07	0.896937427	Potri.001G043900.1	AT1G19780.1
Potra2n1c3590.1	-1.24589361	-0.018705848	0.068751173	0.996164153		
Potra2n1c3591.1	1.189470801	0.949094008	9.39735E-08	0.182404592	Potri.001G411800.1	AT4G27280.1
Potra2n1c3592.1	1.031806614	0.429000422	0.000191116	0.704224102		
Potra2n1c3593.1	2.888299833	1.128337703	4.29372E-06	0.756757704	Potri.001G411300.1	AT4G27290.1
Potra2n1c3594.1	0.997276799	0.231151823	8.19263E-05	0.908294078	Potri.001G411000.1	AT4G27290.1
Potra2n1c3595.2	-0.775317707	-0.54157288	0.036714947	0.886633917	Potri.001G410800.2	AT4G27290.1
Potra2n1c3596.2	-0.56246499	-0.433009335	0.002361852	0.570154529	Potri.001G410700.1	AT4G27270.1
Potra2n1c3597.3	-1.676293277	0.130751356	1.74826E-08	0.943483421	Potri.001G410600.1	AT3G20640.1
Potra2n1c3598.2	-0.51320282	-0.083620715	0.004896672	0.960981446	Potri.001G410500.1	AT5G54520.1
Potra2n1c3599.1	-1.714956999	-1.034162	0.000250012	0.595538573	Potri.001G410400.1	AT5G54510.1
Potra2n1c36.1	-0.515212015	-0.387155472	8.61645E-05	0.496666963		
Potra2n1c360.1	0.520977134	0.571988914	0.633236181	0.936890993		
Potra2n1c3600.1	-2.5843661	-2.734541843	0.452176315	NA		
Potra2n1c3601.1	-0.681456661	-0.257220644	0.000451975	0.802942736	Potri.001G410300.1	AT5G54530.1
Potra2n1c3602.1	-0.282041775	0.139968866	0.029828357	0.910815461	Potri.001G410100.1	AT5G54540.1
Potra2n1c3603.2	-0.266769992	0.125418134	0.054966858	0.916192318	Potri.011G130100.7	AT3G20650.1
Potra2n1c3604.1	-1.36687853	-0.357732152	5.86496E-10	0.798429987		
Potra2n1c3605.1	-1.43084295	-0.342313491	1.80967E-09	0.786910796	Potri.001G409900.1	AT5G54570.1

Potra2n1c3606.1	0.321238152	-0.19045555	0.123954516	0.908294078	Potri.001G409800.1	AT5G54580.1
Potra2n1c3607.1	-0.748499818	-0.279951361	2.33704E-05	0.723580731	Potri.001G415400.1	AT5G54600.1
Potra2n1c3608.2	-1.407082396	-0.531930397	3.90217E-14	0.310657002	Potri.001G415500.1	AT4G27250.1
Potra2n1c3609.1	-1.400303451	-0.531158835	6.69456E-14	0.306363984	Potri.001G415600.1	AT5G54630.1
Potra2n1c361.1	NA	NA	NA	NA		
Potra2n1c3610.1	-0.583437391	-0.301331473	4.22733E-06	0.458127962	Potri.001G415700.1	AT1G51060.1
Potra2n1c3611.2	0.164385099	-0.269015114	0.752190722	0.912777843	Potri.001G415800.2	AT4G38070.1
Potra2n1c3612.1	-0.143903416	-0.015361863	0.196381289	0.987288338	Potri.011G131700.1	AT5G54650.1
Potra2n1c3613.1	0.091353509	-0.129527485	0.715386112	0.91998377	Potri.001G416000.1	
Potra2n1c3614.1	-0.406791157	-0.109253807	0.317826514	0.972159498	Potri.001G416200.1	AT5G54660.1
Potra2n1c3615.1	-0.910262001	-0.30065008	1.11262E-07	0.721438964	Potri.001G416300.1	AT5G54670.1
Potra2n1c3616.1	-0.939033086	-0.685832807	0.048744735	0.619615765	Potri.001G416400.1	AT3G01440.1
Potra2n1c3617.1	-1.824847398	0.82155371	3.64882E-06	NA	Potri.001G416500.1	AT1G11530.1
Potra2n1c3618.1	-0.395484898	-0.137260786	0.007796358	0.881624318	Potri.001G416600.1	AT5G54680.1
Potra2n1c3619.1	-0.680220285	-0.398065967	0.000723168	0.786910796	Potri.001G416800.1	AT5G54690.1
Potra2n1c362.1	4.339777754	-0.296075425	9.19704E-10	NA	Potri.001G043800.1	AT2G34930.1
Potra2n1c3620.1	1.124429249	-0.133022124	0.760162099	NA	Potri.001G416900.1	AT4G27190.1
Potra2n1c3621.1	-0.253976856	0.033894793	0.156302723	0.98177802	Potri.001G417000.1	AT5G54730.1
Potra2n1c3622.1	0.295745673	0.191811589	0.144265854	0.786348406	Potri.001G417200.3	AT3G20720.2
Potra2n1c3623.1	0.177776799	0.057518805	0.198358035	0.954419252	Potri.001G417300.1	AT3G20740.1
Potra2n1c3624.2	0.096289727	0.117429053	0.594357883	0.918687733	Potri.001G417400.1	AT4G04790.1
Potra2n1c3625.1	-1.003581	-0.625326015	0.122903642	0.824265661	Potri.001G417600.1	AT3G28345.1
Potra2n1c3626.1	0.027649578	0.286089393	0.993308491	NA	Potri.011G133800.1	AT3G28390.1
Potra2n1c3627.1	0.058937112	-0.586350402	NA	NA	Potri.001G417700.1	AT3G28345.1
Potra2n1c3628.1	1.318197499	0.266416157	4.7676E-10	0.874648591	Potri.001G418000.1	AT3G20600.1
Potra2n1c3629.1						
Potra2n1c363.1	0.361587069	-0.08090317	0.042729713	0.966005919	Potri.001G044200.1	AT1G80860.2
Potra2n1c3630.1	0.856452355	-0.84292892	0.055838996	0.602446338	Potri.001G411400.1	AT4G27290.1
Potra2n1c3631.1	-0.615574496	-0.763669795	NA	NA	Potri.001G418200.1	AT4G21200.1
Potra2n1c3632.1					Potri.001G418300.1	AT1G50960.1
Potra2n1c3633.1	0.266728978	-0.005818594	0.051514845	0.995920419	Potri.001G418400.1	AT5G54750.1
Potra2n1c3634.1	-0.073055201	-0.23256132	0.558642631	0.621109642	Potri.001G418500.1	AT4G05320.2

Potra2n1c3635.1	-0.072987047	-0.232367666	0.558642631	0.621109642		
Potra2n1c3636.1	0.458087142	0.214184564	0.000306557	0.65293511	Potri.011G134700.1	AT4G27130.1
Potra2n1c3637.1					Potri.004G020500.3	AT5G54770.1
Potra2n1c3638.2	0.108040373	0.223874806	0.498450056	0.784845657	Potri.001G418800.1	AT3G20480.1
Potra2n1c3639.5	-0.041691116	0.069535587	0.788433396	0.957766214	Potri.001G419000.1	AT3G20580.1
Potra2n1c364.1	-0.128613533	-0.110982331	0.473016837	0.900869139	Potri.001G044300.1	AT1G15980.1
Potra2n1c3640.2	-0.234612707	-0.069842495	0.316881369	0.972068777	Potri.001G419100.2	AT4G27100.2
Potra2n1c3641.1	-0.057108094	-0.002436195	0.735847062	0.999315506	Potri.001G419300.1	AT3G20560.1
Potra2n1c3642.1	1.186001962	0.856005088	0.225643859	0.796459872	Potri.001G419200.1	AT3G20570.1
Potra2n1c3643.1	4.161712025	1.641855976	8.04859E-26	0.371597978	Potri.001G419400.1	AT3G21710.2
Potra2n1c3644.1	1.664677056	1.363811008	NA	NA	Potri.001G419500.2	AT4G04890.1
Potra2n1c3645.1	NA	1.363811008	NA	NA		
Potra2n1c3646.1					Potri.001G419600.1	AT3G15160.1
Potra2n1c3647.1	-0.174445048	0.307474878	0.662558472	0.910485505	Potri.001G427300.1	AT4G27220.1
Potra2n1c3648.1	0.890155017	0.208798463	0.249917406	0.974275337	Potri.T176000.2	AT4G27190.1
Potra2n1c3649.1	-1.811199684	-0.604260546	0.006957293	0.810712263	Potri.T176000.2	AT4G27190.1
Potra2n1c365.2	0.618704819	-0.287051091	0.681434235	0.968706951	Potri.001G044401.1	AT5G57970.1
Potra2n1c3650.1						
Potra2n1c3651.1	NA	NA	NA	NA		
Potra2n1c3652.1					Potri.001G419600.1	AT3G15160.1
Potra2n1c3653.1	NA	1.571529889	NA	NA	Potri.001G422700.1	
Potra2n1c3654.1	-1.913580399	-1.984429736	0.676797964	NA	Potri.001G419800.1	AT4G27220.1
Potra2n1c3655.1	-0.169738467	-0.18485781	0.376758694	0.887678043	Potri.001G420200.2	AT5G54800.1
Potra2n1c3656.1	0.688034387	0.070945416	0.001684819	0.958952027	Potri.001G420300.1	AT4G27070.1
Potra2n1c3657.2	-0.035472605	0.198833968	0.831082213	0.81583604	Potri.001G420400.1	AT3G20550.1
Potra2n1c3658.1	1.459962178	NA	NA	NA	Potri.001G420500.1	AT3G62230.1
Potra2n1c3659.1	0.496995958	-0.04003568	0.012176533	0.974367223	Potri.001G420700.1	AT5G54840.1
Potra2n1c366.1	3.905056905	1.238914284	0.001508586	0.788065047	Potri.001G044500.1	AT1G80840.1
Potra2n1c3660.1	-0.121482753	-0.44813557	0.657141098	0.47454828	Potri.001G420800.1	AT3G47860.1
Potra2n1c3661.1	-0.644170935	-0.228376342	0.001886704	0.787393135	Potri.001G420900.1	AT1G50900.1
Potra2n1c3662.1	-0.46812452	-0.288798901	0.028651347	0.738746172	Potri.001G421000.1	AT5G54850.1
Potra2n1c3663.1	-0.339426674	-0.12111878	0.006762935	0.87679119	Potri.001G421200.1	AT4G27060.1

Potra2n1c3664.1	-0.310877515	-0.09577166	0.00746704	0.906107926	Potri.001G421300.1	AT1G50840.1
Potra2n1c3665.1	2.556135291	1.381953902	NA	NA	Potri.001G421400.1	AT3G20530.1
Potra2n1c3666.3	-0.127247292	0.379470169	0.733434296	0.824448251		
Potra2n1c3667.1	0.136858829	0.562730221	0.72215261	0.422139068	Potri.001G207400.1	
Potra2n1c3668.1	0.139065777	0.530359385	0.707972292	0.483645683		
Potra2n1c3669.1	0.183159244	-0.272732367	0.846317508	0.966005919		
Potra2n1c367.1	0.202813251	0.101421104	0.396932575	0.936402128	Potri.001G044700.2	AT4G21090.1
Potra2n1c3670.1	0.403044893	-0.393405599	0.372194504	0.902847514	Potri.001G422301.1	
Potra2n1c3671.1	1.475657766	0.54699473	0.406457899	NA		
Potra2n1c3672.1	0.475085893	-0.005592783	0.149106333	0.999325699	Potri.001G422000.2	
Potra2n1c3673.1	-0.61783378	0.25033223	0.077806874	0.950109239	Potri.001G422301.1	
Potra2n1c3674.1	-2.396052862	-2.542948491	NA	NA		
Potra2n1c3675.1	0.729155555	0.312537438	1.75699E-06	0.663037599	Potri.001G424950.1	AT2G34520.1
Potra2n1c3676.1	NA	NA	NA	NA	Potri.011G137400.1	AT5G54860.1
Potra2n1c3677.1	0.251759091	0.113330776	0.140004912	0.880477186	Potri.001G424600.1	AT4G27040.1
Potra2n1c3678.1	-2.518737085	-0.199743038	0.449436587	NA	Potri.001G424700.1	AT4G27030.1
Potra2n1c3679.3	-0.198162939	-0.142424516	0.087839194	0.845014308	Potri.001G424500.1	AT4G27020.1
Potra2n1c368.1	3.201146609	0.674963689	NA	NA	Potri.001G044600.1	
Potra2n1c3680.1	0.413408747	0.22410602	0.011310812	0.724604666	Potri.011G137800.1	AT5G36110.1
Potra2n1c3681.1	0.090495137	0.123671362	0.789232383	0.926808461	Potri.001G423800.1	AT5G54890.1
Potra2n1c3682.1	0.170327885	0.472458348	0.659506392	0.745178903	Potri.001G423700.1	AT3G20500.1
Potra2n1c3683.3	0.198330041	0.060053324	0.171362648	0.974628351	Potri.001G423600.1	AT4G27010.1
Potra2n1c3684.2	-0.055052544	0.105054973	0.779316971	0.943819961	Potri.001G423500.1	AT1G65980.1
Potra2n1c3685.3	0.00252891	-0.083562031	0.984251832	0.872233138	Potri.001G423400.1	AT4G27000.1
Potra2n1c3686.1	0.185425517	0.037787726	0.347211484	0.983446961	Potri.001G423300.1	AT5G54910.1
Potra2n1c3687.1	-0.111028749	-0.138868313	0.711994172	0.923348116	Potri.001G423200.1	AT5G54920.1
Potra2n1c3688.1	-0.00980584	0.524182828	0.997584497	NA	Potri.T066500.2	AT5G54930.1
Potra2n1c3689.1	0.80159622	1.409620769	0.792354286	NA		
Potra2n1c369.1					Potri.001G044800.1	AT4G29280.1
Potra2n1c3690.1	2.170051646	2.164154664	0.012889054	0.727326968	Potri.001G432900.1	
Potra2n1c3691.1	NA	0.674963689	NA	NA	Potri.001G432900.1	
Potra2n1c3692.1	0.060527488	0.164887995	0.848805122	0.932447954	Potri.T176000.2	AT4G27190.1

Potra2n1c3693.1	NA	NA	NA	NA		
Potra2n1c3694.2	-0.068154857	-0.000806283	0.761069366	0.999536469		
Potra2n1c3695.1	0.037097825	0.042134706	0.918948225	0.976302215	Potri.001G430000.1	AT2G38130.2
Potra2n1c3696.1	-1.573587552	-0.04252368	5.38677E-07	0.987226805	Potri.001G435400.1	AT3G20340.1
Potra2n1c3697.1	-0.538473783	0.022716119	2.01514E-05	0.986636967	Potri.001G435500.1	AT1G50670.1
Potra2n1c3698.1	0.519039823	0.119281228	0.000394008	0.936549944	Potri.001G435700.1	AT3G20390.1
Potra2n1c3699.1	3.627428647	2.501508747	0.380971308	NA	Potri.001G435800.1	AT3G20395.1
Potra2n1c37.1	-0.311998625	0.034798073	0.008443028	0.969898621	Potri.001G004200.1	AT1G55325.2
Potra2n1c370.1	-0.630068697	-0.398929754	0.012032712	0.744962075	Potri.001G044900.1	AT1G15960.1
Potra2n1c3700.2	3.459722542	0.657945785	0.13654842	NA	Potri.011G139700.1	AT1G17230.1
Potra2n1c3701.1	1.222048413	1.552411619	0.002310297	0.344206454	Potri.001G430500.1	AT1G17230.1
Potra2n1c3702.1	2.400418299	0.870997373	0.043815128	0.912665563	Potri.001G430600.1	AT3G14370.1
Potra2n1c3703.1	1.499989415	0.138981965	4.543E-07	0.930598202	Potri.001G430700.1	AT1G72280.1
Potra2n1c3704.1	1.021599004	0.94620841	0.437554204	0.821709751	Potri.001G430600.1	AT3G14370.1
Potra2n1c3705.6	1.610843751	0.187626922	8.1937E-10	0.885375133	Potri.001G430700.1	AT1G72280.1
Potra2n1c3706.1	-0.354145534	-0.045563089	0.363655541	0.985393296	Potri.001G436200.10	AT1G72250.2
Potra2n1c3707.2	-0.474058468	-0.092760672	0.001593421	0.909675463	Potri.001G436300.1	AT1G17220.1
Potra2n1c3708.1	-0.750134265	-0.204876375	0.00344232	0.93125845	Potri.001G436400.1	AT1G17200.1
Potra2n1c3709.1	0.049602664	-0.041807764	0.824773856	0.978725627	Potri.001G431200.1	AT1G78380.1
Potra2n1c371.4	3.322216551	0.896212309	3.55019E-14	0.728883321	Potri.001G045100.1	AT1G15950.1
Potra2n1c3710.1	2.687817169	1.27318558	7.5107E-19	0.012853651	Potri.001G436600.1	AT1G78380.1
Potra2n1c3711.1	-0.159340242	1.32774363	0.738212772	0.146565149	Potri.001G431400.2	AT1G78380.1
Potra2n1c3712.1					Potri.001G437500.1	AT1G78370.1
Potra2n1c3713.1	1.098134893	0.766768407	0.316743536	0.88661378	Potri.001G437100.1	AT1G78380.1
Potra2n1c3714.1	1.853206702	1.262132037	9.82053E-12	0.001454466	Potri.001G437200.1	AT1G78380.1
Potra2n1c3715.1	-0.225225854	0.652898976	0.853562656	0.253058618	Potri.001G437400.1	AT1G78380.1
Potra2n1c3716.1	-0.144922404	0.737430049	0.908299948	0.175916011	Potri.001G437400.1	AT1G78380.1
Potra2n1c3717.1	3.069173756	1.769621302	5.21665E-08	0.289406217	Potri.001G437600.1	AT2G33060.1
Potra2n1c3718.1	6.468171245	3.054220049	3.7971E-36	NA	Potri.001G437700.1	AT1G47890.1
Potra2n1c3719.1	5.772597271	3.277480885	0.004216363	NA	Potri.T098200.1	AT4G21390.1
Potra2n1c372.1	-0.152331889	-1.333940512	0.940160916	NA	Potri.001G046000.2	AT3G50160.1
Potra2n1c3720.3	2.79586706	1.235566522	3.65902E-16	0.684244573	Potri.001G438000.1	AT2G33080.1

Potra2n1c3721.1						
Potra2n1c3722.1	-0.264628614	0.052145207	0.028938387	0.965880015	Potri.001G438300.1	AT1G79030.1
Potra2n1c3723.2	1.46901196	0.518638888	2.94935E-05	0.547624419	Potri.001G438400.1	AT1G16670.1
Potra2n1c3724.1	-0.32790518	-0.126146469	0.183795476	0.962288867	Potri.001G438500.2	AT1G56000.1
Potra2n1c3725.3	-0.021143662	-0.001723622	0.964480362	0.999536469	Potri.001G438700.2	AT1G79040.1
Potra2n1c3726.1	-0.058144175	-0.025982024	0.85498028	0.9896961	Potri.001G438900.1	AT1G79050.1
Potra2n1c3727.1	-0.350188552	0.023831313	0.005214545	0.983008617	Potri.001G439000.1	AT1G16590.1
Potra2n1c3728.1	0.008179008	-0.040107866	0.978724998	0.986088609	Potri.001G439100.1	AT1G79120.1
Potra2n1c3729.1	-0.308328914	-0.285381809	0.187700663	0.833067606	Potri.001G439600.1	AT3G12920.1
Potra2n1c373.1	-0.872348025	-2.235379122	0.722091803	NA	Potri.001G355800.1	AT5G55130.1
Potra2n1c3730.1	8.112974148	4.579818572	3.04046E-46	0.004892292	Potri.001G439401.1	AT2G32210.1
Potra2n1c3731.1	1.184103301	1.576872579	0.692137138	NA		
Potra2n1c3732.1	0.82357019	NA	NA	NA	Potri.001G440000.1	
Potra2n1c3733.2	0.016683926	-0.151929359	0.9280008	0.88643982	Potri.001G440100.1	AT5G44500.1
Potra2n1c3734.1	NA	NA	NA	NA	Potri.001G207400.1	
Potra2n1c3735.1	9.494538451	4.244642008	4.50366E-17	NA	Potri.001G440200.1	AT5G36110.1
Potra2n1c3736.1	-0.888529334	-0.366963972	0.026998335	0.889258723	Potri.001G440300.1	AT4G20430.1
Potra2n1c3737.1					Potri.001G440400.1	
Potra2n1c3738.1	NA	NA	NA	NA	Potri.001G440500.1	
Potra2n1c3739.1	-0.081989803	-0.099148235	0.565627623	0.926478129	Potri.001G440600.1	AT4G20410.1
Potra2n1c374.1	4.866784423	2.059770004	4.66975E-33	0.085896019	Potri.001G046100.1	AT1G15950.1
Potra2n1c3740.1	NA	NA	NA	NA	Potri.001G440800.1	AT1G30800.1
Potra2n1c3741.1	-0.09785951	0.029582022	0.610131563	0.982368449	Potri.001G440900.1	AT1G30810.2
Potra2n1c3742.1	2.858999506	0.885772804	0.144945918	NA	Potri.001G441400.1	AT4G21390.1
Potra2n1c3743.1	4.412409236	1.879759176	0.029619245	NA	Potri.001G441400.1	AT4G21390.1
Potra2n1c3744.1	5.658388781	0.103001015	3.05151E-08	NA	Potri.001G442200.1	AT4G21390.1
Potra2n1c3745.1	2.463418979	-1.745799514	0.09968161	NA	Potri.001G442300.1	AT5G44550.1
Potra2n1c3746.1	-0.298003194	-0.060763633	0.094352711	0.969421568	Potri.001G442400.1	AT4G20380.7
Potra2n1c3747.2	-0.3022707	0.201349209	0.239581875	0.902857376		
Potra2n1c3748.2	0.112869988	0.665902827	0.627166734	0.579724936	Potri.001G442600.1	AT2G36840.1
Potra2n1c3749.1	1.459962178	NA	NA	NA	Potri.001G442700.1	AT5G11720.1
Potra2n1c375.1	NA	NA	NA	NA		

Potra2n1c3750.1	2.079832605	0.669662732	6.49418E-20	0.432051484	Potri.001G442800.1	AT5G11720.1
Potra2n1c3751.1	0.033891531	-0.000756594	0.826528636	0.999536469	Potri.001G442900.10	AT4G19490.2
Potra2n1c3752.1	-0.661429045	0.204518182	0.70743584	0.971317865	Potri.011G153800.3	
Potra2n1c3753.4	-0.248488541	0.105610713	0.120395512	0.915339763	Potri.001G443200.2	AT2G35630.1
Potra2n1c3754.1	NA	NA	NA	NA	Potri.001G446200.1	
Potra2n1c3755.1	1.664677056	NA	NA	NA	Potri.001G444400.1	
Potra2n1c3756.1	0.824497211	0.8502037	NA	NA		
Potra2n1c3757.1	-0.312325821	-0.092105675	0.333725907	0.970432679	Potri.001G450800.1	AT1G32990.1
Potra2n1c3758.1	1.091395283	0.276807201	0.000970771	0.911509355	Potri.001G450700.1	AT3G61460.1
Potra2n1c3759.3	-0.215063643	-0.036147253	0.505363811	0.975578749	Potri.011G151200.1	AT1G32960.1
Potra2n1c376.1	4.482733114	1.817192132	1.00849E-11	0.216627903	Potri.001G046100.1	AT1G15950.1
Potra2n1c3760.1	-1.35927387	-1.507841271	NA	NA	Potri.001G450300.1	AT5G14230.1
Potra2n1c3761.1	-0.61910233	-0.767708831	NA	NA	Potri.001G450300.1	AT5G14230.1
Potra2n1c3762.1	-0.152299216	1.772192929	0.689158051	0.34109377	Potri.001G450200.1	AT1G32930.1
Potra2n1c3763.1	0.82357019	NA	NA	NA	Potri.004G232250.1	AT5G49760.1
Potra2n1c3764.1	-0.13840537	0.228994656	0.450890165	0.865591169	Potri.001G450100.1	AT1G64720.1
Potra2n1c3765.1	0.004377488	0.463459775	0.996266078	0.901671593	Potri.001G449800.1	AT1G32928.1
Potra2n1c3766.1	-0.368702172	0.054195352	0.603900314	0.991147673	Potri.001G449800.1	AT1G32928.1
Potra2n1c3767.1	-0.037672829	-0.035629502	0.961654639	0.995261215	Potri.001G449800.1	AT1G32928.1
Potra2n1c3768.1	0.189439185	0.380024627	0.859516535	0.947547562	Potri.001G449800.1	AT1G32928.1
Potra2n1c3769.1	NA	NA	NA	NA	Potri.001G449700.2	
Potra2n1c377.1	0.898603537	0.147224379	1.12869E-06	0.92223649	Potri.003G181200.1	AT2G32060.2
Potra2n1c3770.1	-0.386010706	-0.088440363	0.062100256	0.954559968	Potri.001G449600.1	AT5G42110.1
Potra2n1c3771.1	-0.938758376	-0.214935722	1.76597E-09	0.788065047	Potri.011G152200.1	AT1G32900.1
Potra2n1c3772.1	-1.459663722	-0.513814297	1.20885E-07	0.568587629	Potri.001G449100.2	AT1G32860.1
Potra2n1c3773.1	0.123882889	0.044961086	0.249182893	0.956865545	Potri.001G449000.2	AT4G10570.1
Potra2n1c3774.1	-0.041000491	0.097620108	0.766650815	0.930203195	Potri.001G448900.1	AT1G32810.2
Potra2n1c3775.1	-0.249516462	0.056389043	0.006100907	0.953608209	Potri.004G201600.1	AT5G22400.1
Potra2n1c3776.1	0.113336774	-0.11808038	0.517071995	0.902991033	Potri.001G448800.3	AT1G32790.1
Potra2n1c3777.1						
Potra2n1c3778.1	1.64774763	1.394613675	NA	NA	Potri.008G022500.1	
Potra2n1c3779.2	2.37806727	1.664097707	0.133816188	NA	Potri.T098200.1	AT4G21390.1

Potra2n1c378.1	-0.463299843	-0.352205168	0.001933903	0.522715829	Potri.001G046700.1	AT2G32070.1
Potra2n1c3780.1	1.64774763	0.674963689	NA	NA	Potri.001G441500.1	AT5G18470.1
Potra2n1c3781.1						
Potra2n1c3782.2	3.295047393	1.680487398	0.11276734	NA	Potri.T098200.1	AT4G21390.1
Potra2n1c3783.1	2.632524198	1.363811008	NA	NA		
Potra2n1c3784.1	3.083722284	2.256014376	0.10804152	NA	Potri.004G232250.1	AT5G49760.1
Potra2n1c3785.1	-0.231898791	0.527581534	0.570958857	0.751093503		
Potra2n1c3786.1	NA	NA	NA	NA		
Potra2n1c3787.1	-1.294879587	1.377685121	0.246301942	0.805461106	Potri.001G447500.1	AT5G17540.1
Potra2n1c3788.1	1.459962178	NA	NA	NA	Potri.013G112000.1	
Potra2n1c3789.1	-1.048170809	1.472770418	0.26349845	0.751694102	Potri.001G447400.1	AT5G17540.1
Potra2n1c379.1	0.106066903	-0.300506472	0.740596912	0.647623607	Potri.001G046800.1	AT1G80760.1
Potra2n1c3790.1	-1.148551658	1.492538887	0.185636756	0.821709751	Potri.001G447500.1	AT5G17540.1
Potra2n1c3791.1	0.102236182	0.660619236	NA	NA	Potri.001G447400.1	AT5G17540.1
Potra2n1c3792.1	NA	NA	NA	NA	Potri.001G447400.1	AT5G17540.1
Potra2n1c3793.1	NA	NA	NA	NA		
Potra2n1c3794.1	-1.211930276	-0.091698357	1.4379E-05	0.953831244	Potri.001G448000.1	AT5G17540.1
Potra2n1c3795.1	-0.729009418	-3.439023268	0.813762465	NA	Potri.001G448100.2	AT4G00820.1
Potra2n1c3796.1						
Potra2n1c3797.1	-0.176305702	-0.087086078	0.276191227	0.948577865	Potri.001G448400.1	AT2G46770.1
Potra2n1c3798.1	1.392953023	1.105019748	0.093452706	0.79549388	Potri.001G448500.1	AT1G64700.1
Potra2n1c3799.1	NA	NA	NA	NA	Potri.001G448600.1	
Potra2n1c38.1	-0.169537311	0.087339558	0.290267817	0.925184207	Potri.001G004300.2	AT4G26310.1
Potra2n1c380.1	0.047939686	-0.248359142	0.855140511	0.760408803	Potri.001G046900.1	AT1G15900.1
Potra2n1c3800.1	0.363905016	-0.030794993	0.010888201	0.979755286	Potri.001G448700.1	AT1G32780.1
Potra2n1c3801.1	NA	NA	NA	NA	Potri.001G448700.1	AT1G32780.1
Potra2n1c3802.1	-1.098801042	1.951131439	0.252718657	0.737914109	Potri.001G447400.1	AT5G17540.1
Potra2n1c3803.1	-0.202457699	-3.465481486	0.958696742	NA	Potri.001G451500.2	AT4G10500.1
Potra2n1c3804.1	3.56475484	1.363811008	0.384454894	NA	Potri.001G451600.1	AT4G10500.1
Potra2n1c3805.1	3.852909421	0.837287472	8.91884E-15	0.872570395	Potri.001G451900.1	AT4G10490.1
Potra2n1c3806.1	1.119324361	-0.003475678	0.00422109	0.999244673	Potri.001G452100.1	AT1G61570.1
Potra2n1c3807.1	3.340156477	0.463716251	NA	0.937846405	Potri.001G452000.1	AT2G36090.1

Potra2n1c3808.1	5.807868172	1.471908575	2.21593E-16	NA	Potri.001G452200.1	AT2G46870.1
Potra2n1c3809.1	0.293051239	0.242119442	0.00215265	0.453952326	Potri.001G452300.2	AT1G33050.4
Potra2n1c381.1	-0.240752495	-0.035021444	0.111337985	0.985912891	Potri.001G047100.1	AT1G15880.1
Potra2n1c3810.1	2.580497546	1.320507593	0.266621115	NA	Potri.001G452400.1	AT2G18196.1
Potra2n1c3811.1	4.924385104	3.135996653	1.1729E-14	0.244991632	Potri.001G452600.1	AT4G13010.1
Potra2n1c3812.1	0.098032281	0.112841648	0.633436336	0.93017274	Potri.001G452700.1	AT1G33060.1
Potra2n1c3813.1	-0.253192779	0.028495934	0.165033583	0.988862007	Potri.001G452800.1	AT2G45530.1
Potra2n1c3814.1	1.305750782	0.655675622	1.32144E-08	0.284282353	Potri.001G453000.1	AT3G30340.1
Potra2n1c3815.1	-1.966603852	-1.63487921	0.558306726	NA	Potri.001G452900.1	AT1G63310.1
Potra2n1c3816.1					Potri.001G453100.1	AT5G19790.1
Potra2n1c3817.12	-0.208751747	0.007177345	0.04356718	0.994236852	Potri.001G453200.1	AT1G10390.2
Potra2n1c3818.1	-0.135073323	0.038827048	0.636530223	0.979273211		
Potra2n1c3819.1	-0.096347521	-0.036816728	0.63759069	0.974367223	Potri.001G453400.1	AT1G73940.1
Potra2n1c382.2	0.196390322	0.028513119	0.215845395	0.981772014	Potri.001G047200.1	AT1G76280.3
Potra2n1c3820.1	-0.145596786	0.211033119	0.498221726	0.824905557	Potri.001G453500.1	AT5G15940.1
Potra2n1c3821.4	-0.147956506	-0.032452017	0.436364182	0.99114586	Potri.001G453600.2	AT5G09810.1
Potra2n1c3822.1	-3.472943819	-2.903157078	0.39976348	NA		
Potra2n1c3823.1	-0.455307201	1.252134835	0.809382597	0.819608939	Potri.001G453700.1	AT5G09810.1
Potra2n1c3824.1	-0.116546436	0.14424344	0.835102817	0.977559521	Potri.001G453800.1	AT5G47610.1
Potra2n1c3825.1	0.361350938	-0.030390537	0.009837613	0.985393296	Potri.001G453900.1	AT1G33140.1
Potra2n1c3826.1	0.360154209	-0.038868021	0.018114299	0.983113084	Potri.001G453900.1	AT1G33140.1
Potra2n1c3827.1	0.322057194	-0.020484813	0.074409189	0.992486563	Potri.001G453900.1	AT1G33140.1
Potra2n1c3828.1	-0.131038927	-0.085146641	0.432875384	0.935851032	Potri.001G454200.1	AT4G38120.1
Potra2n1c3829.1	-1.488405943	-0.496092848	6.76381E-07	0.477503371	Potri.001G454300.1	AT1G33170.1
Potra2n1c383.1	0.27031478	-0.106903442	0.078357772	0.900551258	Potri.001G047400.1	AT1G80750.1
Potra2n1c3830.1	0.083719806	0.072315861	0.613997556	0.948455569	Potri.001G454400.1	AT1G33230.1
Potra2n1c3831.1	-0.677685863	-0.138370675	0.009780457	0.901671593	Potri.001G454500.1	AT1G76890.2
Potra2n1c3832.1	-0.241858154	-0.161452532	0.253582225	0.922011871	Potri.001G454600.1	AT1G33250.1
Potra2n1c3833.1	-0.31526722	-0.316602689	0.331403877	0.8954569	Potri.001G454800.1	AT1G33260.2
Potra2n1c3834.1	-0.205467619	-0.094099878	0.447626891	0.92893808	Potri.001G454900.1	AT1G33265.1
Potra2n1c3835.1	-0.047412653	-0.074849765	0.887685045	0.96551968	Potri.001G455000.1	AT4G10380.1
Potra2n1c3836.1	-2.367361922	0.488608458	NA	NA		

Potra2n1c3837.1	-1.650393094	0.627792096	0.521873915	NA	Potri.001G455200.1	AT5G39050.1
Potra2n1c3838.1	-0.270378411	-0.070125378	0.198253905	0.960981446	Potri.001G455300.1	AT5G65930.2
Potra2n1c3839.1	0.35378958	-0.673457139	0.538899105	0.739520512	Potri.001G455400.1	AT1G14130.1
Potra2n1c384.1	-0.258035333	-0.380814328	0.212157767	0.277054321	Potri.001G047500.1	AT5G10970.1
Potra2n1c3840.2	0.197546674	-0.306948786	0.453348907	0.817489719	Potri.001G455500.1	AT5G06740.1
Potra2n1c3841.1	-0.11325179	0.146197642	0.463014544	0.824448251	Potri.001G455600.1	AT5G24740.1
Potra2n1c3842.1	-0.83667936	0.015900614	0.003261848	0.995579852	Potri.001G455800.1	AT4G10550.1
Potra2n1c3843.1	-0.563355065	-0.180786047	0.027751708	0.810712263	Potri.001G455900.1	AT3G51510.1
Potra2n1c3844.1	-0.511993601	-0.571539765	0.190519477	0.864223715	Potri.001G456400.1	AT1G76520.1
Potra2n1c3845.1	-0.054481459	-0.042287433	0.563669516	0.936364479	Potri.001G456500.1	AT2G17410.2
Potra2n1c3846.1	-0.079388591	-0.049945746	0.646978348	0.974275337	Potri.001G456600.3	AT5G63870.2
Potra2n1c3847.1	-0.481465337	-0.743294168	0.452176315	0.679365931	Potri.001G456700.1	
Potra2n1c3848.1	0.322368269	-0.159557732	0.014483845	0.854980698	Potri.001G456800.1	AT2G17420.1
Potra2n1c3849.1	1.517251188	-2.514137983	0.50663087	NA	Potri.001G457000.1	AT1G76490.1
Potra2n1c385.1	0.022323388	-0.14764842	0.925904848	0.918687733	Potri.001G047600.1	AT1G15870.1
Potra2n1c3850.1	0.52055466	-0.052770495	0.002143931	0.974275337	Potri.001G457201.1	AT5G65950.1
Potra2n1c3851.1	-0.755561203	-0.423917483	0.076051874	0.663950227	Potri.001G457300.1	AT1G79080.1
Potra2n1c3852.1	-0.064685408	0.052215537	0.590536746	0.941855873	Potri.001G457550.1	AT1G04390.1
Potra2n1c3853.1	0.664665428	1.250687949	0.71974224	NA		
Potra2n1c3854.1	-5.129982162	-0.991361164	6.88915E-07	0.32064055	Potri.001G457700.1	AT1G22240.1
Potra2n1c3855.1	-0.174425815	-0.089780745	0.136712395	0.900869139	Potri.001G457800.1	AT1G79090.1
Potra2n1c3856.1	0.20701581	-0.072973893	0.446212748	0.975578749	Potri.001G457900.1	AT1G56050.1
Potra2n1c3857.1	-1.099789941	-0.260690962	1.32549E-06	0.888366602	Potri.001G458000.1	AT3G12955.1
Potra2n1c3858.1	0.029666685	-0.11189361	0.896048808	0.930598202	Potri.001G458200.2	AT5G16930.1
Potra2n1c3859.1	NA	NA	NA	NA		
Potra2n1c386.1	-1.191749394	-0.551236009	0.000138641	0.654454883	Potri.001G047800.1	AT1G80690.1
Potra2n1c3860.1	1.545832463	NA	NA	NA	Potri.001G458400.1	AT3G06240.1
Potra2n1c3861.1	-0.471941534	-0.156134511	0.044827394	0.899205262	Potri.001G458600.2	AT3G12930.1
Potra2n1c3862.1						
Potra2n1c3863.1	2.735991243	NA	NA	NA		
Potra2n1c3864.1	0.241141276	0.428172659	0.635428459	0.842960488	Potri.T176000.2	AT4G27190.1
Potra2n1c3865.1						

Potra2n1c3866.1						
Potra2n1c3867.1	NA	NA	NA	NA		
Potra2n1c3868.2	-0.443539953	0.021607162	0.051358311	0.992934677	Potri.011G155800.1	AT1G30630.1
Potra2n1c3869.1	1.459962178	NA	NA	NA	Potri.018G074600.2	AT1G03670.1
Potra2n1c387.1	-0.198955433	0.083813486	0.140513692	0.91392603	Potri.001G047900.6	AT1G15860.1
Potra2n1c3870.1	NA	NA	NA	NA		
Potra2n1c3871.1	-0.365660874	0.10039351	0.016060393	0.922618347	Potri.011G155800.1	AT1G30630.1
Potra2n1c3872.1						
Potra2n1c3873.1	-0.190683316	0.640858194	0.797470267	0.829455551	Potri.001G459400.1	AT5G23530.1
Potra2n1c3874.1	0.526456555	0.225430513	0.016567265	0.820966257	Potri.001G459700.1	AT1G30620.1
Potra2n1c3875.1	-3.293078443	1.9466407	NA	0.849848068		
Potra2n1c3876.2	0.966635713	0.136984965	0.438386527	NA	Potri.001G459900.1	AT1G30610.2
Potra2n1c3877.1	-0.001336993	-0.10085755	0.999196947	0.988883632	Potri.014G010900.1	AT3G14470.1
Potra2n1c3878.1	1.809051307	0.860391749	0.171155196	NA	Potri.014G011400.2	AT3G14470.1
Potra2n1c3879.1	0.358759228	0.127213634	0.625608307	0.978203092	Potri.004G169800.1	AT3G14460.1
Potra2n1c388.1					Potri.001G048000.1	AT1G80680.1
Potra2n1c3880.2	-0.310536328	0.213778344	0.212157767	0.88661378	Potri.001G459900.1	AT1G30610.2
Potra2n1c3881.1	-1.407243375	-0.662560971	NA	NA	Potri.014G003600.3	AT3G14460.1
Potra2n1c3882.1	NA	NA	NA	NA		
Potra2n1c3883.2	2.330171789	0.963876493	5.29891E-16	0.227212532	Potri.001G462600.2	AT1G01980.1
Potra2n1c3884.1	NA	NA	NA	NA		
Potra2n1c3885.1	-0.720346426	-0.281923972	9.73731E-05	0.712599153	Potri.001G462800.1	AT2G34790.1
Potra2n1c3886.1	-0.6122007	-0.876609742	0.450941836	0.797111689		
Potra2n1c3887.1	2.098804896	0.670673788	3.11594E-12	0.485057514	Potri.001G462600.2	AT1G01980.1
Potra2n1c3888.2	0.230463181	-0.071214278	0.562625242	0.978646026	Potri.001G460000.1	AT3G44680.1
Potra2n1c3889.1	-0.916440389	-1.09721607	0.458628844	0.82876584	Potri.001G459900.1	AT1G30610.2
Potra2n1c389.1	0.085511367	-0.105230188	0.729987948	0.932328711	Potri.001G048100.1	AT1G80670.1
Potra2n1c3890.1	0.289576405	0.111274012	0.447176732	0.949881967	Potri.004G169800.1	AT3G14460.1
Potra2n1c3891.1	0.796814844	-0.046370319	NA	NA		
Potra2n1c3892.2	-0.154770806	0.169497369	0.64864553	0.922778027	Potri.001G459900.1	AT1G30610.2
Potra2n1c3893.1	0.466348654	0.292370102	0.474439689	0.916192318	Potri.004G169800.1	AT3G14460.1
Potra2n1c3894.1	0.011465939	-0.042890441	0.952019317	0.960981446	Potri.001G460000.1	AT3G44680.1

Potra2n1c3895.1	0.247852217	-0.129849252	0.131436476	0.932242797	Potri.001G460100.2	AT1G25260.1
Potra2n1c3896.1	-0.187264637	-0.047201136	0.126340099	0.963355476	Potri.011G156400.1	AT4G21105.1
Potra2n1c3897.1	-0.219180242	-0.10310367	0.097741206	0.920141992		
Potra2n1c3898.1	0.291400965	-0.024414854	0.506602648	0.994403137	Potri.001G460300.1	AT3G25690.1
Potra2n1c3899.1	-0.050061336	0.273488198	0.866798179	0.825064676	Potri.001G460400.1	AT1G07090.1
Potra2n1c39.1	-0.748667691	-2.518530379	0.627313403	NA	Potri.001G004432.1	
Potra2n1c390.1	-1.104179667	-0.830401065	5.37436E-05	0.065793866	Potri.001G048200.1	AT5G25190.1
Potra2n1c3900.2	-0.297604509	0.300041339	0.364362464	0.830056077	Potri.011G156900.1	AT1G73720.1
Potra2n1c3901.1	-0.98107548	0.014642114	3.02207E-05	0.996154416	Potri.001G460600.1	AT1G30650.1
Potra2n1c3902.1						
Potra2n1c3903.1	0.824497211	-0.767708831	NA	NA		
Potra2n1c3904.1	-0.769324788	-0.675590602	0.586100132	0.903698951	Potri.001G460800.1	
Potra2n1c3905.1	0.82357019	NA	NA	NA		
Potra2n1c3906.1	NA	NA	NA	NA	Potri.001G461000.1	AT2G34820.1
Potra2n1c3907.1	0.289423579	0.110571736	0.217442411	0.943483421	Potri.001G461100.2	AT2G44510.1
Potra2n1c3908.3	-0.047735972	0.121362634	0.822238296	0.922993749	Potri.001G461200.1	AT1G30680.1
Potra2n1c3909.1	-0.258713829	-0.102084126	0.026419057	0.922681078	Potri.001G461300.1	AT1G30680.1
Potra2n1c391.4	-0.325510569	-0.455499269	0.63715777	0.907073354	Potri.001G048300.1	AT1G80660.1
Potra2n1c3910.1	-0.75145514	-0.444126207	0.001283556	0.776094736	Potri.001G461400.1	AT1G30690.1
Potra2n1c3911.1	4.494538431	2.249665367	1.45604E-05	NA	Potri.001G461500.1	AT4G20820.1
Potra2n1c3912.1					Potri.011G158100.1	AT5G44390.1
Potra2n1c3913.1	8.675117734	1.413466566	1.51079E-12	NA	Potri.001G461900.1	AT4G20820.1
Potra2n1c3914.1	2.340207562	NA	NA	NA		
Potra2n1c3915.1	NA	NA	NA	NA		
Potra2n1c3916.1	2.073352726	0.686202322	1.93576E-16	0.560452893	Potri.001G462600.2	AT1G01980.1
Potra2n1c3917.1	-0.830432759	-0.266739342	3.36711E-11	0.719850204	Potri.001G462800.1	AT2G34790.1
Potra2n1c3918.1	-1.343734386	-0.046423919	8.83547E-06	0.986636967		
Potra2n1c3919.1	-1.33323178	-0.19846094	5.39451E-11	0.895560236	Potri.001G463000.1	AT1G70500.1
Potra2n1c392.3	-0.045481194	-0.865850476	0.892558039	0.176054801	Potri.001G048500.1	AT2G25220.1
Potra2n1c3920.1	-0.320416619	-1.200126903	0.821063023	NA	Potri.001G463100.1	AT2G34790.1
Potra2n1c3921.2	-0.258979273	0.108487746	0.332442853	0.950182516	Potri.001G463200.2	AT3G26710.1
Potra2n1c3922.1	0.70462915	-1.356235503	0.72070137	NA	Potri.001G463300.1	AT2G34790.1

Potra2n1c3923.1	2.484531581	-0.1853952	0.08099148	NA	Potri.001G463300.1	AT2G34790.1
Potra2n1c3924.1	-0.970015859	-0.696808038	0.048436123	0.588177782	Potri.001G463400.1	AT5G44400.1
Potra2n1c3925.1	0.260982286	0.357220398	0.042864969	0.779493734	Potri.011G161901.1	AT2G34780.1
Potra2n1c3926.1	0.435372292	-0.172445486	0.44032934	0.930203195	Potri.001G463800.1	AT2G34770.1
Potra2n1c3927.1	NA	NA	NA	NA	Potri.001G463900.1	AT1G28600.1
Potra2n1c3928.1	0.049813597	0.209618123	0.844396096	0.857704467	Potri.001G464200.1	AT2G29760.1
Potra2n1c3929.1	0.128695837	0.162755485	0.671634241	0.895474078	Potri.001G464300.1	AT4G20880.1
Potra2n1c393.1	-0.72423096	0.638371635	0.289391024	0.830470159		
Potra2n1c3930.1	NA	NA	NA	NA		
Potra2n1c3931.1	-0.33506433	0.051930967	0.051163084	0.974264269	Potri.001G464400.1	AT5G62690.1
Potra2n1c3932.1	NA	NA	NA	NA	Potri.001G464500.2	AT5G39190.1
Potra2n1c3933.1	NA	NA	NA	NA		
Potra2n1c3934.1	0.111813926	-2.42671106	NA	NA	Potri.001G464900.1	AT3G16360.2
Potra2n1c3935.1	NA	NA	NA	NA	Potri.001G465000.1	AT3G16360.2
Potra2n1c3936.1	1.263411437	0.268727928	2.03711E-11	0.874507876	Potri.001G465300.1	AT5G63890.2
Potra2n1c3937.1	-0.974198305	-0.299309051	0.030220899	0.935751704	Potri.001G465401.1	AT4G20900.1
Potra2n1c3938.1	0.089133471	-0.142347233	0.724375722	0.937276488	Potri.001G465500.1	AT4G20910.1
Potra2n1c3939.3	0.250399225	0.577901554	0.28842451	0.566863304	Potri.001G465600.1	AT4G20930.1
Potra2n1c394.1	0.051442609	0.250697596	0.8539485	0.788065047	Potri.001G048700.1	AT1G15520.1
Potra2n1c3940.1	-0.977295495	-0.481218275	0.004809057	0.733006577	Potri.001G465800.1	AT4G20940.1
Potra2n1c3941.1	0.350332089	-0.188876758	0.543437707	0.953831244	Potri.001G466000.2	AT5G56000.1
Potra2n1c3942.2	0.34816676	0.037712223	0.007279813	0.981772014	Potri.001G465900.1	AT1G30580.1
Potra2n1c3943.1	0.37262812	0.022222634	0.004162029	0.9896961	Potri.001G466000.2	AT5G56000.1
Potra2n1c3944.1	-0.519472661	-0.115917603	0.101535428	0.954419252	Potri.001G466066.1	AT4G33990.1
Potra2n1c3945.1	2.379695424	-0.767708831	NA	NA	Potri.001G466200.1	AT2G45600.1
Potra2n1c3946.1	NA	2.283425176	NA	NA		
Potra2n1c3947.1	NA	NA	NA	NA	Potri.001G466300.1	AT2G45600.1
Potra2n1c3948.1	-1.317292482	-1.465929376	NA	NA	Potri.001G466300.1	AT2G45600.1
Potra2n1c3949.1	-0.060740385	0.138034374	0.592495752	0.897285619	Potri.001G466500.1	
Potra2n1c395.3	1.304035688	-0.230428561	3.46095E-14	0.903769998	Potri.001G048700.1	AT1G15520.1
Potra2n1c3950.1	0.128420152	0.056207268	0.835266007	0.993589787	Potri.001G466700.1	AT5G14980.1
Potra2n1c3951.1	-0.200103485	0.130943463	0.148085581	0.910878765	Potri.001G466800.1	AT2G34730.1

Potra2n1c3952.1	-0.23820696	-0.088716525	0.234808074	0.940856252	Potri.001G466900.1	AT1G30540.1
Potra2n1c3953.1	-0.257267518	0.217780017	0.359320216	0.919424623	Potri.001G467000.1	AT1G30570.1
Potra2n1c3954.1	-0.332653144	0.158452707	0.552354842	0.963194733	Potri.001G467100.1	AT4G26555.1
Potra2n1c3955.1	-1.342639153	-0.039105454	9.06914E-11	0.986636967	Potri.001G467300.1	AT5G56040.2
Potra2n1c3956.1	-0.541949113	-0.132442136	0.009085698	0.922011871	Potri.001G467400.1	AT3G13226.1
Potra2n1c3957.1	0.535348269	1.379694776	NA	NA		
Potra2n1c3958.1	-1.463525756	-0.487035018	5.82943E-07	0.787990612	Potri.001G467600.1	AT3G44730.1
Potra2n1c3959.1	-0.224439352	0.026135741	0.070339572	0.987288338	Potri.001G467800.1	AT1G55810.3
Potra2n1c396.1	6.652062767	1.911450358	9.59351E-07	NA	Potri.001G048900.2	AT1G15520.1
Potra2n1c3960.1	-0.622086915	-0.269398736	0.000949321	0.549127296	Potri.001G468300.1	AT4G26500.1
Potra2n1c3961.1	-0.573761044	-0.500493343	0.442064748	0.821709751	Potri.011G165500.1	AT4G26490.1
Potra2n1c3962.1	0.063591739	0.503703596	0.982639181	NA		
Potra2n1c3963.1	1.545832463	NA	NA	NA	Potri.001G468500.1	AT4G05030.1
Potra2n1c3964.1	-0.711628339	-0.200948125	0.000131239	0.857189843	Potri.001G468700.1	AT1G55760.1
Potra2n1c3965.1	-0.696904418	-0.233731114	0.000529428	0.824905557		
Potra2n1c3966.1	-0.703557221	0.219156493	0.477189082	0.974367223	Potri.001G468800.1	AT5G59810.1
Potra2n1c3967.1					Potri.001G468900.1	AT5G59810.1
Potra2n1c3968.1	5.353484594	1.271293918	8.83875E-67	0.530383957	Potri.001G469000.1	AT5G59810.1
Potra2n1c3969.1	4.209749742	0.06197156	0.007962165	NA	Potri.012G108000.1	AT2G34400.1
Potra2n1c397.3	0.455652614	0.144890109	0.009010848	0.949048812	Potri.001G049100.1	AT1G78955.1
Potra2n1c3970.1	9.88995633	5.949208464	4.29933E-41	0.149897924	Potri.001G064900.1	AT1G58190.2
Potra2n1c3971.1	6.490096094	1.571529889	3.5327E-06	NA	Potri.018G147900.1	AT2G34400.1
Potra2n1c3972.1	10.13612366	6.411991558	3.04046E-46	0.386015987	Potri.003G041700.2	AT1G58190.2
Potra2n1c3973.1	6.522872181	2.149378023	7.85601E-55	0.377857883	Potri.001G469000.1	AT5G59810.1
Potra2n1c3974.1	6.588557844	1.571529889	2.9923E-06	NA	Potri.012G108000.1	AT2G34400.1
Potra2n1c3975.1	9.499508616	5.162523564	4.64897E-25	0.585428557	Potri.001G065300.1	AT5G49290.1
Potra2n1c3976.1					Potri.T094701.1	AT2G19080.1
Potra2n1c3977.1	1.568582118	1.332851691	0.582247463	NA	Potri.001G063200.1	AT4G37070.1
Potra2n1c3978.1	10.05694936	6.077625836	6.95206E-18	0.518597946	Potri.001G064900.1	AT1G58190.2
Potra2n1c3979.1	6.51790089	1.50738503	3.97758E-08	NA	Potri.001G065300.1	AT5G49290.1
Potra2n1c398.2	-0.226950098	-0.174028563	0.444210789	0.902857376	Potri.003G178700.1	AT3G24800.1
Potra2n1c3980.1	-1.317292482	0.897912335	NA	NA	Potri.001G155750.1	AT1G21280.1

Potra2n1c3981.1	NA	NA	NA	NA	Potri.T094701.1	AT2G19080.1
Potra2n1c3982.1	1.419815199	1.080671404	0.407969852	NA	Potri.001G063200.1	AT4G37070.1
Potra2n1c3983.1	9.805068924	5.649747265	1.20453E-16	0.225576181	Potri.018G148000.1	AT1G58190.2
Potra2n1c3984.1	-1.35927387	-1.507841271	NA	NA	Potri.001G064200.1	AT4G30110.1
Potra2n1c3985.1	10.11047486	5.973902534	5.81162E-48	0.113016317	Potri.012G108000.1	AT2G34400.1
Potra2n1c3986.1	0.82357019	NA	NA	NA	Potri.018G144400.1	AT4G30110.1
Potra2n1c3987.1	1.179133951	0.160811036	3.06594E-09	0.930598202	Potri.001G469200.1	AT3G13230.1
Potra2n1c3988.1	0.062431205	0.056380725	0.726348567	0.974367223	Potri.001G469300.1	AT3G13235.3
Potra2n1c3989.1	-0.700834716	0.268821593	0.03496897	0.88661378	Potri.001G469401.1	AT3G13275.1
Potra2n1c399.6	-0.106595421	0.031957219	0.30396109	0.972159498	Potri.001G049400.3	AT2G32700.6
Potra2n1c3990.1	-0.079949333	-0.028520972	0.477455741	0.971750134	Potri.001G469500.1	AT3G13300.2
Potra2n1c3991.1	-0.715994485	-0.373134048	2.83383E-09	0.64495179	Potri.001G469600.1	AT3G13310.1
Potra2n1c3992.4	-0.81925743	-0.295956584	3.64272E-08	0.776048341	Potri.011G166800.1	AT5G56090.1
Potra2n1c3993.1	-0.098599047	-0.065969223	0.499597436	0.966077009	Potri.001G470000.1	AT5G23810.1
Potra2n1c3994.1	-0.723592029	-3.257886644	0.796092317	NA		
Potra2n1c3995.1	0.602254657	0.026481503	0.158222939	0.992934677	Potri.011G167200.1	AT5G23810.1
Potra2n1c3996.1	-0.21745037	0.140888383	0.135738341	0.901816804	Potri.001G470500.1	AT5G56110.1
Potra2n1c3997.1	0.366376231	0.221830024	0.014366812	0.841487301	Potri.001G470600.2	AT3G13330.1
Potra2n1c3998.1	-2.234028292	-0.646959052	8.84767E-06	0.709241338	Potri.001G470700.1	AT1G60950.1
Potra2n1c3999.1	0.203991539	-0.119581553	0.250425271	0.911339141	Potri.001G470900.1	AT5G56130.1
Potra2n1c4.1	NA	1.571529889	NA	NA	Potri.001G000600.1	AT1G55570.1
Potra2n1c40.1	0.296304535	0.100180687	0.009894305	0.90634987	Potri.001G004500.1	AT5G19760.1
Potra2n1c400.2	-0.076411607	-0.698840274	0.984974293	NA		
Potra2n1c4000.1	-0.263950612	-0.746435273	0.439309747	0.605100478	Potri.001G470800.1	AT5G56120.1
Potra2n1c4001.2	0.377705804	0.176396421	0.000204923	0.797111689	Potri.001G471000.1	AT5G56140.1
Potra2n1c4002.1	-0.744257763	0.070898527	3.47892E-06	0.966005919	Potri.001G471100.2	AT4G26470.1
Potra2n1c4003.1	0.46271252	0.220178011	2.43129E-05	0.517743314	Potri.001G471200.1	AT5G53300.4
Potra2n1c4004.1	0.82357019	NA	NA	NA		
Potra2n1c4005.5	0.406951925	0.230753511	0.001508474	0.652931023	Potri.001G471300.5	AT1G55690.1
Potra2n1c4006.1	0.387223965	0.185153172	0.005765237	0.643288421	Potri.001G471700.2	AT5G56180.1
Potra2n1c4007.1	0.422487386	0.094660446	0.042491341	0.939149035	Potri.001G471600.1	AT5G66520.1
Potra2n1c4008.1	-0.428727311	-0.212454039	0.000981641	0.560708883	Potri.001G471800.6	AT3G13340.2

Potra2n1c4009.3	-0.395855014	-0.072014081	4.55389E-06	0.93263929	Potri.001G472000.3	AT4G26455.1
Potra2n1c401.1	NA	NA	NA	NA	Potri.001G108600.1	AT1G25270.1
Potra2n1c4010.1	NA	NA	NA	NA		
Potra2n1c4011.1	-1.93768197	0.065388169	0.296135026	NA	Potri.001G472200.2	AT5G56220.1
Potra2n1c4012.1	NA	NA	NA	NA		
Potra2n1c4013.1	0.309175683	0.260810604	0.009837505	0.692084498	Potri.001G472400.1	AT5G56240.1
Potra2n1c4014.1	0.053987583	0.089603904	0.72311831	0.921099436	Potri.001G472600.10	AT4G26450.1
Potra2n1c4015.1	-0.045582659	0.06660032	0.812682099	0.956865545	Potri.001G472700.1	AT1G55620.2
Potra2n1c4016.1	-0.953072831	-0.249286494	6.14794E-05	0.851395707	Potri.001G472800.1	AT5G56270.1
Potra2n1c4017.3	0.304381807	0.102912857	0.089791792	0.95359265	Potri.001G472900.1	AT1G55610.1
Potra2n1c4018.1	0.154280983	0.307564445	0.265539181	0.702001326	Potri.001G473100.2	AT5G56290.1
Potra2n1c4019.1	-1.280254539	-0.470203342	0.370851982	NA	Potri.001G473200.1	AT1G55580.1
Potra2n1c402.1	1.664677056	NA	NA	NA	Potri.008G135300.1	AT1G74590.1
Potra2n1c4020.1	-0.153974398	-0.242526315	0.390890763	0.469019862	Potri.001G473300.1	AT1G16220.1
Potra2n1c4021.1	-0.057361903	1.460421947	NA	NA		
Potra2n1c403.1	-1.818458736	-1.290729063	0.110779786	0.710721635	Potri.001G049700.1	AT4G13195.1
Potra2n1c404.2	-0.213133265	-0.149804815	0.039807312	0.874808359	Potri.001G049800.1	AT3G24160.1
Potra2n1c405.1	0.18980754	-0.019067013	0.172177343	0.98177802	Potri.001G050000.1	AT3G24170.1
Potra2n1c406.1	0.092382651	-0.085478254	0.601478995	0.936890993	Potri.001G049900.1	AT4G13670.1
Potra2n1c407.1	5.159438443	1.956047938	1.61776E-61	0.016840421	Potri.001G050100.1	AT2G02230.1
Potra2n1c408.1	-1.317292482	-0.024939653	NA	NA	Potri.001G050200.1	AT2G33100.1
Potra2n1c409.1	0.798758999	0.915053636	0.022675443	0.540010302	Potri.001G050300.2	AT1G04730.1
Potra2n1c41.1	-0.454487162	-0.242657405	0.01419598	0.850010611	Potri.001G004600.1	AT5G19770.1
Potra2n1c410.1	-0.03687332	0.085076385	0.877382559	0.95671968	Potri.001G050400.1	AT1G04760.1
Potra2n1c411.1	-0.024758205	0.036263597	0.947082574	0.987440949	Potri.001G050600.1	AT1G04770.1
Potra2n1c412.2	0.279601322	0.064855333	0.007909975	0.927423678	Potri.001G050700.5	AT3G24190.1
Potra2n1c413.1	-0.331175326	-2.348152951	0.815712622	NA	Potri.001G050900.1	AT3G04280.3
Potra2n1c414.1	-0.545916287	0.150364886	0.129347878	0.936402128	Potri.001G051000.1	AT5G26594.1
Potra2n1c415.1	0.292272705	0.444146576	0.021278577	0.646171083	Potri.001G051200.1	AT3G24200.1
Potra2n1c416.1	-0.936404385	-0.26331619	0.007752097	0.83433596	Potri.001G051300.1	AT1G34010.1
Potra2n1c417.1	-0.216729328	-0.256854474	0.037151258	0.615720834	Potri.001G051400.2	AT1G04780.1
Potra2n1c418.1	6.104232338	2.644566563	5.65205E-20	0.386834221	Potri.001G051500.1	AT5G13930.1

Potra2n1c419.1	5.950739684	2.000519583	5.48192E-11	0.677013336	Potri.001G051600.1	AT5G13930.1
Potra2n1c42.1	-2.153240635	-0.859992458	0.023822636	0.83433596	Potri.001G004700.1	AT5G19790.1
Potra2n1c420.1	-0.013974123	-0.444018321	0.972757998	0.70570456	Potri.001G051700.1	AT3G23940.1
Potra2n1c421.1	0.2140034	1.082153239	0.837006058	0.759797728	Potri.003G176500.1	AT1G04790.1
Potra2n1c422.4	0.601827413	0.112196024	4.59337E-12	0.903934429	Potri.001G051900.1	AT2G33150.1
Potra2n1c423.1	-0.506305883	0.181937286	0.592034464	0.976302215	Potri.001G052000.1	AT1G04700.1
Potra2n1c424.1	0.2228916	0.01471535	0.091178803	0.985393296	Potri.001G052100.3	AT1G04690.1
Potra2n1c425.2	-0.335013227	-0.439659747	0.217105349	0.590087814	Potri.001G052300.1	AT4G13710.1
Potra2n1c426.1	0.47726564	0.305067757	0.017981282	0.70570456	Potri.001G052400.1	AT4G13720.1
Potra2n1c427.1	-0.62729303	0.035824139	0.269398481	0.991089517	Potri.001G052500.1	AT3G24240.1
Potra2n1c428.1	-0.98303538	-0.304824864	0.073173804	0.911094206	Potri.001G052500.1	AT3G24240.1
Potra2n1c429.1	1.64774763	NA	NA	NA	Potri.001G052600.1	AT1G04670.1
Potra2n1c43.1	1.459962178	1.381953902	NA	NA		
Potra2n1c430.1					Potri.001G052700.1	
Potra2n1c431.1					Potri.001G052800.1	
Potra2n1c432.1						
Potra2n1c433.1	-0.560504775	-0.355589337	0.033314175	0.8689387	Potri.003G175300.1	AT1G04650.1
Potra2n1c434.1	3.133303066	0.523274248	0.002932396	NA	Potri.001G053100.1	AT3G24060.1
Potra2n1c435.1	NA	NA	NA	NA	Potri.001G053300.1	AT3G24060.1
Potra2n1c436.1	-0.35373894	-0.075046197	0.003598637	0.919424623	Potri.001G053400.1	AT2G33170.1
Potra2n1c437.3	0.528998656	0.136789842	0.102966989	0.895474078	Potri.001G053500.1	AT3G24050.1
Potra2n1c438.1	0.2177871	0.235539217	0.257274402	0.61887385	Potri.001G053600.1	AT4G13590.1
Potra2n1c439.1	-0.145661717	0.017678109	0.284685717	0.987226805	Potri.001G053800.1	AT3G24040.1
Potra2n1c44.1	0.353743563	0.105256978	0.005456758	0.875861859	Potri.001G004800.1	AT5G19820.1
Potra2n1c440.1	0.174366043	0.125845072	0.527485393	0.930059926	Potri.003G174600.1	AT4G13590.1
Potra2n1c441.1	-1.512824867	0.063577624	1.88396E-08	0.982933413	Potri.001G053700.1	AT2G33180.1
Potra2n1c442.1	-0.130045118	2.911677531	0.924326514	NA	Potri.001G054000.1	AT3G24020.1
Potra2n1c443.1	NA	NA	NA	NA	Potri.001G054100.1	AT4G13580.1
Potra2n1c444.1	-1.323133003	0.481489453	NA	NA	Potri.001G054200.1	
Potra2n1c445.2	0.732004276	0.067770292	0.000189929	0.976302215	Potri.001G054400.1	AT3G23990.1
Potra2n1c446.1	0.097499946	-0.05679529	0.432538015	0.951731753	Potri.001G054500.1	AT1G04630.1
Potra2n1c447.1	-0.312661179	0.029625507	0.53691241	0.99488781	Potri.001G054600.1	AT5G60020.1

Potra2n1c448.1	-0.062019789	0.001210324	0.761551081	0.999458638	Potri.001G054700.10	AT5G11860.3
Potra2n1c449.1	-0.463068276	-0.609246849	0.430334823	0.528681203	Potri.001G054800.1	AT1G74110.1
Potra2n1c45.1					Potri.001G004900.1	
Potra2n1c450.1	0.645608205	0.057488409	3.61104E-05	0.976888173	Potri.001G054900.1	AT1G27390.1
Potra2n1c451.3	-0.680744996	0.411775966	0.026935297	0.847341104	Potri.001G055100.1	AT1G27920.1
Potra2n1c452.1	0.76873407	-0.44129605	0.156604071	0.857374454	Potri.001G055000.1	AT5G43250.1
Potra2n1c453.1	-0.745511071	0.450485923	0.051954284	0.833667976	Potri.003G173200.1	AT1G67370.1
Potra2n1c454.1	0.34594021	0.242897286	0.020934636	0.693906898	Potri.001G055300.1	AT1G67360.1
Potra2n1c455.1	0.051131938	0.097089253	0.739556471	0.894032078	Potri.001G055400.1	AT1G67350.1
Potra2n1c456.1	1.911189818	-0.767708831	NA	NA	Potri.001G055500.1	AT3G04280.1
Potra2n1c457.1					Potri.001G055600.1	AT5G26594.1
Potra2n1c458.1	2.235338028	0.674963689	NA	NA	Potri.001G055700.1	AT1G62940.1
Potra2n1c459.1	-0.042651717	0.063998935	0.809459732	0.945121757	Potri.001G055900.1	AT5G43270.1
Potra2n1c46.1	0.20713548	0.265120275	0.499050671	0.802942736	Potri.001G005000.1	AT5G19830.1
Potra2n1c460.1	-0.084341811	0.003316696	0.449418162	0.996292992	Potri.001G056000.3	AT1G67325.1
Potra2n1c461.2	0.172914827	0.168423694	0.086247492	0.809914834	Potri.001G056100.1	AT3G13060.2
Potra2n1c462.1	-0.372084178	-0.127592436	0.844284587	NA	Potri.001G056200.3	AT1G27950.1
Potra2n1c463.1	0.403130338	2.722342159	0.537640922	0.331800699	Potri.001G056300.1	AT1G67330.1
Potra2n1c464.1	-0.575447551	-0.3103542	0.001224214	0.64781796	Potri.T147500.1	AT1G27385.1
Potra2n1c465.2	-0.61910233	-0.767708831	NA	NA	Potri.T147401.1	AT1G11780.1
Potra2n1c466.1	-0.183543564	0.037416604	0.44920486	0.985964146	Potri.001G056600.1	AT5G43260.1
Potra2n1c467.1	-1.109570601	-0.45910968	8.17603E-09	0.441009549	Potri.001G056700.3	AT3G61470.1
Potra2n1c468.1	NA	NA	NA	NA		
Potra2n1c469.1	-0.445946843	-0.090544914	0.007555313	0.947969749	Potri.001G056800.1	AT1G67340.1
Potra2n1c47.1	0.913249588	0.339465334	0.000242708	0.895575326		
Potra2n1c470.1	-0.555854032	-0.310009662	0.000725378	0.735844641	Potri.001G057100.2	AT1G27340.1
Potra2n1c471.2	0.482054074	0.222210309	0.000919995	0.617980199	Potri.001G057200.4	AT2G25560.1
Potra2n1c472.1	1.104287945	0.435922515	0.715179775	NA	Potri.001G057350.1	
Potra2n1c473.1	-0.068511053	0.239704256	0.79596155	0.865591169	Potri.001G057400.1	AT1G27320.1
Potra2n1c474.1	-0.042569494	-0.011113089	0.657862799	0.988862007	Potri.001G057700.1	AT1G27300.1
Potra2n1c475.1	-0.042688491	-0.011113089	0.657062601	0.988862007	Potri.001G057500.1	AT1G27970.1
Potra2n1c476.1	0.377194759	0.143288565	0.000192492	0.857374454	Potri.001G057800.1	AT1G67310.1

Potra2n1c477.1					Potri.003G170500.1	AT5G08391.1
Potra2n1c478.2	-1.161992466	0.147393433	0.000103259	0.966148753	Potri.001G058100.3	AT1G27290.1
Potra2n1c479.1	-0.61910233	-0.767708831	NA	NA		
Potra2n1c48.1	0.85852888	0.281971813	0.001077312	0.918674657	Potri.001G005100.1	AT3G13540.1
Potra2n1c480.1	-0.12056231	0.534328799	0.640339543	0.766554688		
Potra2n1c481.1	-0.044892498	0.583436999	0.872571062	0.70570456	Potri.001G058200.2	AT2G03710.3
Potra2n1c482.1	-0.65902503	-1.43637599	0.750360192	NA	Potri.001G058400.1	AT1G24260.2
Potra2n1c483.1	1.64774763	1.394613675	NA	NA	Potri.001G058500.1	
Potra2n1c484.1	-0.436091143	-0.019714046	0.002839328	0.987440949	Potri.001G058600.1	AT5G50570.2
Potra2n1c485.2	-0.101724065	0.160283604	0.59967634	0.824905557	Potri.001G058700.1	AT1G24265.1
Potra2n1c486.1	2.089478315	-0.963347719	8.40588E-05	0.726091974	Potri.001G058800.1	AT5G13080.1
Potra2n1c487.1	-0.929387951	-0.13720968	0.370912465	0.982827151	Potri.001G058900.1	AT5G60740.1
Potra2n1c488.4	-0.008905667	0.103281051	0.971761938	0.937846405	Potri.001G059000.1	AT1G27980.1
Potra2n1c489.1	-1.737119063	-0.532579716	0.034579238	0.875559093	Potri.001G059100.1	AT5G14090.1
Potra2n1c49.1	1.664613756	1.742803637	0.565194614	NA		
Potra2n1c490.1	-0.566145129	-0.023690934	0.002812365	0.992986441	Potri.001G059400.1	AT5G13090.1
Potra2n1c491.1	2.432694306	0.648055103	0.012067083	0.923766642	Potri.001G059500.2	AT1G27990.1
Potra2n1c492.1	-0.167027722	-0.094395596	0.059919489	0.926030989	Potri.001G059700.2	AT5G13100.1
Potra2n1c493.1	-0.549380625	0.404460989	0.851256252	0.974790553	Potri.001G059800.1	AT5G50710.1
Potra2n1c494.1	-0.866532338	-0.07692914	1.67337E-06	0.959881296	Potri.001G059900.1	AT5G13110.1
Potra2n1c495.1	0.25609434	-0.122165008	0.142482607	0.939824856		
Potra2n1c496.1	1.157462771	-0.021315227	0.000361156	0.988132773	Potri.001G060100.2	AT1G69040.2
Potra2n1c497.1	0.10711651	0.008492357	0.555526671	0.995579852	Potri.001G060200.1	AT5G13120.1
Potra2n1c498.1	0.736200704	0.413243132	6.54392E-07	0.517463209	Potri.001G060300.1	
Potra2n1c499.1	0.528902336	0.202450107	0.823202663	NA	Potri.001G060400.1	AT5G50760.1
Potra2n1c5.1					Potri.T006500.1	AT2G01050.1
Potra2n1c50.1	0.138838983	0.309507246	0.62771271	0.679975189	Potri.001G005300.1	AT5G19850.1
Potra2n1c500.1	0.564543472	-0.060007968	0.275553466	0.987226805	Potri.001G060500.1	AT5G13140.1
Potra2n1c501.1	-1.74077592	-0.980782242	0.008345109	0.630517286	Potri.001G060600.1	AT5G14070.1
Potra2n1c502.1	-0.7798611	-1.02632046	NA	NA	Potri.001G060700.1	AT5G13150.1
Potra2n1c503.1	-0.012278291	0.00478711	0.949047511	0.996185763	Potri.001G060800.1	AT5G13160.1
Potra2n1c504.1	-1.35927387	-1.507841271	NA	NA	Potri.001G060900.1	AT5G13170.1

Potra2n1c505.1	0.241769718	-0.022666496	0.146743627	0.983446961	Potri.001G061000.1	AT5G43280.1
Potra2n1c506.1	6.215259604	3.47211971	1.015E-10	0.170208245	Potri.001G061100.1	AT3G02040.1
Potra2n1c507.1	-0.061932005	0.072904117	0.8691235	0.956865545	Potri.001G061200.1	AT5G13180.1
Potra2n1c508.1	-0.010049882	-0.137950232	0.959084559	0.859478649	Potri.001G061400.1	AT5G50850.1
Potra2n1c509.1	0.377792561	0.228252702	0.044359178	0.742848888	Potri.001G061500.1	AT2G33490.1
Potra2n1c51.1	-0.484821742	-0.072171946	0.027102628	0.975919374	Potri.001G005400.1	AT5G11950.2
Potra2n1c510.1	-1.605001128	-0.661854451	0.000572281	0.743657005	Potri.001G061600.1	AT5G21090.1
Potra2n1c511.1	4.88523479	3.152096249	0.038482579	NA	Potri.001G061700.1	AT1G71830.1
Potra2n1c512.1	-0.590660811	-0.339248715	0.002248714	0.842247476	Potri.001G061800.1	AT1G28050.1
Potra2n1c513.1	1.117449951	0.628111108	0.009395221	0.805461106	Potri.001G061900.1	AT5G13190.1
Potra2n1c514.1	-0.637406366	-0.321359557	1.62172E-05	0.403434416	Potri.001G062000.1	AT2G33510.1
Potra2n1c515.1	3.63909099	0.404167575	1.22748E-27	0.943041425		
Potra2n1c516.1						
Potra2n1c517.1	6.636554213	3.182639428	2.98417E-18	0.439101966	Potri.001G062500.2	AT5G13220.1
Potra2n1c518.1	-0.374463997	-0.172905173	0.325470146	0.919424623	Potri.003G164900.1	AT5G13230.1
Potra2n1c519.1	-0.402649697	0.020079293	0.003149691	0.987226805	Potri.001G062700.1	AT5G13250.1
Potra2n1c52.1	-0.519742766	-0.075207879	0.022645296	0.975128355	Potri.001G005500.1	AT4G08810.1
Potra2n1c520.1	-0.203718786	-0.058196155	0.237925006	0.956865545	Potri.001G062800.1	AT3G48800.1
Potra2n1c521.1	3.524778498	3.277480885	0.247207403	NA	Potri.001G062900.3	AT5G50915.1
Potra2n1c522.2	0.115371559	0.279915367	0.473245569	0.687847759	Potri.001G065600.1	AT2G33540.1
Potra2n1c523.1	2.515219162	1.777279516	NA	NA		
Potra2n1c524.1	2.747280685	1.077351297	0.167661184	NA		
Potra2n1c525.1	0.771289603	-0.744589582	NA	NA	Potri.001G065700.1	
Potra2n1c526.1	0.125199326	-0.198561009	0.344748054	0.836633315	Potri.001G065800.3	AT5G13260.1
Potra2n1c527.1	-0.267887234	0.704654249	0.551694446	0.619575855	Potri.001G065900.1	AT1G28110.1
Potra2n1c528.1	-0.694000108	-0.107898439	0.004378208	0.937846405	Potri.001G066000.2	AT1G28100.2
Potra2n1c529.1	-0.306062361	-0.227881094	0.33996422	0.885845309	Potri.001G066100.1	AT5G13270.1
Potra2n1c53.1	3.512195089	1.575584627	3.26637E-25	0.007569467	Potri.001G005600.2	AT3G13600.1
Potra2n1c530.1	0.413064002	0.281188364	0.000200159	0.492230064	Potri.001G066200.10	AT5G62000.1
Potra2n1c531.1	-0.652006953	0.358120848	0.082516176	0.911339141	Potri.001G066400.1	AT3G26740.1
Potra2n1c532.1	2.053634494	2.056553484	0.002220527	0.056247855	Potri.001G066500.1	AT5G36930.2
Potra2n1c533.1	-0.713437408	0.546102794	0.038346119	0.525835282	Potri.001G066700.1	AT5G13290.2

Potra2n1c534.1	-0.052447387	-0.007800951	0.743103706	0.995579852	Potri.001G066800.1	AT5G13300.1
Potra2n1c535.1	-0.483285127	0.014024853	0.004987158	0.994403137	Potri.001G066900.1	AT2G33550.1
Potra2n1c536.1	-0.486409457	0.635274975	0.833411184	0.943819961	Potri.001G067000.1	AT1G17100.1
Potra2n1c537.1	-0.345973404	0.064123961	0.050633971	0.964620043	Potri.001G067300.1	AT5G61960.2
Potra2n1c538.1	-0.092198418	0.370274857	0.833364409	0.811742964		
Potra2n1c539.2	0.04219301	0.055864128	0.776696891	0.953428955	Potri.001G067400.2	AT1G28120.1
Potra2n1c54.1	4.091740062	2.382045622	7.12864E-12	0.073093992		
Potra2n1c540.1	0.088794457	-2.436516334	NA	NA	Potri.001G067500.1	
Potra2n1c541.1	4.855486416	0.199240022	0.001776092	0.985964146	Potri.001G067600.1	AT5G13330.1
Potra2n1c542.1	NA	NA	NA	NA	Potri.001G067700.2	AT1G57820.1
Potra2n1c543.2	0.372684202	0.064152945	0.009622579	0.954559968	Potri.001G067800.1	AT1G29880.1
Potra2n1c544.1	0.196211269	-0.014166938	0.013949685	0.986190118	Potri.001G068000.1	AT1G01630.1
Potra2n1c545.1	0.152159183	-0.046712368	0.170581104	0.955322605	Potri.001G067900.1	AT5G13430.1
Potra2n1c546.1	-0.525014836	-0.170658828	0.031130855	0.936890993	Potri.001G068100.2	AT1G27440.1
Potra2n1c547.2	-0.009963399	-0.168276594	0.961890434	0.908764369	Potri.001G068200.1	AT5G13420.1
Potra2n1c548.3	0.148474736	-0.010851274	0.243389559	0.990927357	Potri.001G068300.1	AT5G13410.1
Potra2n1c549.1	0.089282895	-0.184578788	0.630570619	0.88513886	Potri.001G068400.1	AT1G11680.1
Potra2n1c55.1	9.081936555	5.799243349	3.75072E-15	0.017817938	Potri.001G005600.2	AT3G13600.1
Potra2n1c550.1	0.360862552	-0.152520513	0.104326996	0.930598202	Potri.001G068500.1	AT5G64680.2
Potra2n1c551.1	0.856258065	-0.047303088	1.8494E-05	0.979719961	Potri.001G068600.1	AT5G13400.1
Potra2n1c552.1	1.000463572	0.052837925	6.85807E-10	0.973695414	Potri.001G068700.1	AT5G38895.3
Potra2n1c553.1	0.112484125	0.063463631	0.358324655	0.926808461	Potri.001G068900.1	AT5G13390.1
Potra2n1c554.1	-0.845584531	1.461666503	NA	NA	Potri.001G069000.1	AT1G28130.1
Potra2n1c555.1	0.683870512	0.285954831	4.22904E-06	0.779077802	Potri.001G069100.1	AT3G61110.1
Potra2n1c556.1	5.204245578	1.381953902	0.030370342	NA	Potri.003G161100.1	AT1G12880.1
Potra2n1c557.1	4.084614497	NA	0.1425833	NA		
Potra2n1c558.1	-1.317292482	-1.465929376	NA	NA	Potri.001G069300.1	AT5G13910.1
Potra2n1c559.1	2.557916408	0.675211345	2.60521E-39	0.724604666	Potri.001G069501.1	AT1G76390.1
Potra2n1c56.2	0.12674019	0.001596246	0.482296576	0.999325699	Potri.001G005900.2	AT4G25650.1
Potra2n1c560.1	NA	2.303900616	NA	NA	Potri.T134000.1	AT4G13420.1
Potra2n1c561.1	1.308063044	1.360700123	0.019594813	0.459556934	Potri.T133800.2	AT4G13420.1
Potra2n1c562.1	NA	2.315796285	NA	NA	Potri.001G069900.1	AT3G50700.1

Potra2n1c563.1	0.707223956	0.000721656	0.002236807	0.999937671	Potri.001G070100.1	AT3G26935.1
Potra2n1c564.1	-0.121600613	0.231126123	0.872790851	0.949966783	Potri.001G070200.1	AT5G32450.1
Potra2n1c565.1	-0.270480918	-0.53189966	0.459446133	0.169320478	Potri.003G160500.1	AT1G20050.1
Potra2n1c566.1	1.405212746	0.370602564	1.12657E-07	0.843359254	Potri.001G070400.1	AT4G15755.1
Potra2n1c567.2	1.293214381	-0.204758608	0.001831779	0.963878456	Potri.001G070500.1	AT1G44900.1
Potra2n1c568.1	1.106124138	-0.083867721	0.009978813	0.98177802	Potri.001G070700.1	AT1G28190.1
Potra2n1c569.1	0.660584268	0.135123407	0.000331772	0.915358268	Potri.001G070800.1	AT1G44960.1
Potra2n1c57.1	-1.181392639	-0.537533975	0.002817086	0.804536645	Potri.001G006100.1	AT2G41890.1
Potra2n1c570.1	1.563963142	0.572639337	2.53458E-07	0.605897158	Potri.001G070900.1	AT5G47910.1
Potra2n1c571.1	1.096092522	-0.453278673	NA	0.794641467	Potri.001G071000.1	AT5G13870.1
Potra2n1c572.1	0.470792101	0.077536711	9.3673E-05	0.924869625	Potri.001G071100.1	AT1G09690.1
Potra2n1c573.1	1.981475243	1.213641224	0.000914199	0.605140419	Potri.001G071300.1	AT3G50120.1
Potra2n1c574.1	1.934107996	1.324068166	0.000470109	0.512736781	Potri.001G071500.1	AT5G47900.1
Potra2n1c575.1	0.058266122	-0.062630342	0.627998686	0.935851032	Potri.001G071900.1	AT5G47890.1
Potra2n1c576.1	0.289646113	-0.134402386	0.000910296	0.906679354	Potri.001G072000.1	AT3G26618.1
Potra2n1c577.1	0.008417838	-0.173719512	0.964972279	0.84801014	Potri.001G072100.1	AT1G09760.1
Potra2n1c578.1	0.223151956	-0.000848304	0.109880553	0.999536469	Potri.001G072200.1	AT3G51980.1
Potra2n1c579.1	0.081297382	-0.295818668	0.752763056	0.684244573	Potri.001G072300.1	AT5G47870.1
Potra2n1c58.1	1.140523377	0.738311901	0.380674808	0.885375133	Potri.001G006200.1	AT3G13600.1
Potra2n1c580.1	-0.181715089	-0.020268372	0.343137903	0.989329144	Potri.001G072400.4	AT5G47860.1
Potra2n1c581.1	-0.234005922	-0.039744711	0.050200965	0.978251387	Potri.001G072600.1	AT5G11680.1
Potra2n1c582.1	-0.206035573	-0.037869356	0.097260529	0.980592639	Potri.001G072500.1	AT4G38040.1
Potra2n1c583.1	0.024987706	0.051959991	0.916382654	0.96670549	Potri.001G072700.1	AT5G59610.1
Potra2n1c584.2	0.25640839	-0.037638958	0.083917447	0.97950444	Potri.001G072800.1	AT3G46740.1
Potra2n1c585.1	0.083648161	-0.280133054	0.779073559	0.71703421		
Potra2n1c586.1	1.548705256	-0.206998353	1.27784E-08	0.881191525	Potri.001G073000.8	AT5G44600.1
Potra2n1c587.1	3.022867684	0.103348632	6.72323E-29	0.970442612	Potri.001G073100.2	AT1G30820.1
Potra2n1c588.1	-0.068476402	0.107077533	0.638779693	0.872570395	Potri.001G073300.1	AT1G30825.1
Potra2n1c589.1	0.215125196	-0.053942766	0.063795867	0.948002367	Potri.001G073400.1	AT4G20300.2
Potra2n1c59.1	0.196809506	-0.02639071	0.226976702	0.985393296	Potri.001G006300.4	AT1G55300.1
Potra2n1c590.1	1.545832463	0.674963689	NA	NA	Potri.001G073350.1	
Potra2n1c591.1	0.113646354	0.156453118	0.481595018	0.812373531	Potri.001G073500.1	AT4G20280.1

Potra2n1c592.1	0.050937322	0.136138633	0.804833047	0.879538011		
Potra2n1c593.1	-1.696903441	-0.734814721	3.16609E-05	0.100242924	Potri.001G073600.1	AT4G20270.1
Potra2n1c594.2	-0.000503135	-0.041314546	0.998322779	0.9761362	Potri.001G073700.1	AT3G13440.1
Potra2n1c595.2	-0.139290272	-0.055487793	0.576461845	0.973347974	Potri.001G073800.1	AT2G34900.1
Potra2n1c596.1	-2.074294075	-0.428010767	2.80762E-14	0.820880672	Potri.001G073900.2	AT4G20260.1
Potra2n1c597.1	0.692713324	-0.605161991	0.044073295	0.774957538	Potri.001G074000.2	AT5G44635.1
Potra2n1c598.1	-1.85793613	0.099202572	0.022167954	0.9789664	Potri.001G074100.2	AT1G30840.2
Potra2n1c599.1	-1.066012964	-0.489942391	3.67479E-05	0.278663901	Potri.001G074200.2	AT5G44650.1
Potra2n1c6.1	0.271468579	0.056535943	0.004458885	0.936549944	Potri.001G000700.2	AT2G32080.2
Potra2n1c60.1	0.050967978	-0.047035606	0.714474252	0.956292878	Potri.001G006500.1	AT3G13560.2
Potra2n1c600.1	0.134861098	0.044365667	0.416120867	0.956622854	Potri.001G074400.1	AT3G26430.1
Potra2n1c601.1	-0.360476809	0.406300852	0.274683036	0.846926485	Potri.001G074600.1	AT5G44670.1
Potra2n1c602.1	-3.370461399	-0.955947336	0.001344737	0.67090514	Potri.001G074700.1	AT5G44680.1
Potra2n1c603.1	-0.065376575	0.081861512	0.95834966	0.99114586	Potri.001G074800.1	AT2G34920.1
Potra2n1c604.1	-0.08018058	-0.061988922	0.596762987	0.953831244	Potri.001G074901.1	AT4G20150.1
Potra2n1c605.1	1.664677056	NA	NA	NA	Potri.001G075201.1	
Potra2n1c606.1	-0.069469743	-0.03311994	0.699396895	0.979896883	Potri.001G075300.1	AT5G17370.1
Potra2n1c607.1	0.084325835	1.437653285	NA	NA	Potri.001G075400.1	AT3G12720.1
Potra2n1c608.1	-0.329716602	0.391562964	0.304769638	0.590365286	Potri.001G075500.1	AT5G45680.1
Potra2n1c609.1	0.39196261	-0.155715154	0.012026593	0.925582271	Potri.001G075600.2	AT1G30900.1
Potra2n1c61.2	0.831024803	-2.070444007	0.76224535	NA	Potri.001G006600.1	AT1G55320.1
Potra2n1c610.1	-0.742177606	-0.353321037	1.43916E-05	0.475695381	Potri.001G075700.1	AT4G20090.1
Potra2n1c611.1	-0.319237141	0.064463159	0.264475681	0.976302215	Potri.001G075800.1	AT3G57430.1
Potra2n1c612.1	-0.070027824	-0.004945442	0.789866582	0.998438878	Potri.001G075900.1	AT4G20090.1
Potra2n1c613.2	-0.062883711	0.051492073	0.788698509	0.983446961	Potri.001G076100.1	AT2G34960.1
Potra2n1c614.1	0.331744796	-0.030163899	0.231434212	0.987226805	Potri.001G076200.1	AT3G47630.2
Potra2n1c615.8	1.187469698	0.111493729	1.8378E-09	0.947317412	Potri.001G076400.10	
Potra2n1c616.1	2.054777381	0.628378278	3.60812E-14	0.428801064	Potri.001G076500.4	
Potra2n1c617.1	0.739976606	0.915379366	0.001571568	0.131671023	Potri.001G077100.10	
Potra2n1c618.1	7.320916166	3.876179701	7.92768E-09	NA	Potri.T154400.1	
Potra2n1c619.1	7.020760544	3.567709814	1.04694E-16	0.655979264	Potri.001G076800.1	
Potra2n1c62.1	-0.235585304	-0.154748402	0.26341027	0.937789963	Potri.001G006700.1	AT1G55320.1

Potra2n1c620.1	0.543684666	-0.13595151	0.806908209	NA	Potri.001G076800.1	
Potra2n1c621.1	0.057546739	0.111269229	0.874308743	0.948577865	Potri.003G152950.1	
Potra2n1c622.6	0.354627028	0.078259858	0.022934997	0.939644353	Potri.T154500.3	
Potra2n1c623.1	6.340711814	3.131288844	3.14658E-23	0.267223581	Potri.001G077200.1	
Potra2n1c624.1	1.506414836	-0.09124112	5.97555E-10	0.963878456	Potri.001G077300.1	
Potra2n1c625.1	0.598400471	0.308047747	0.000112049	0.389989789	Potri.001G077400.10	
Potra2n1c626.1	-0.759283886	-0.121991446	2.10025E-06	0.917014822	Potri.001G077600.1	
Potra2n1c627.1	8.621143179	4.292925362	7.8137E-08	0.507546032	Potri.001G077900.1	
Potra2n1c628.1	-0.408763708	-0.200111472	0.006297426	0.659942944	Potri.003G152100.1	
Potra2n1c629.2	3.329564962	NA	NA	NA	Potri.001G078200.1	
Potra2n1c63.1	0.047463204	-0.006008948	0.887408947	0.998561549	Potri.T170500.1	AT3G13610.1
Potra2n1c630.2	3.48666361	0.687974895	0.000165502	0.921063801	Potri.001G078300.2	
Potra2n1c631.1	3.867304109	0.188014075	1.12677E-47	0.970930149	Potri.001G078400.1	
Potra2n1c632.1	0.318836442	0.006795198	0.012904423	0.996032145	Potri.001G078500.1	AT1G02410.1
Potra2n1c633.1	0.369155768	0.216609402	0.002060616	0.72101822	Potri.001G078600.1	AT1G33410.2
Potra2n1c634.1	0.132241142	0.397107106	0.451930548	0.65293511	Potri.001G078700.1	AT2G02370.1
Potra2n1c635.1	-0.278732259	0.097711556	0.075960208	0.952773082	Potri.001G078800.1	AT4G24250.1
Potra2n1c636.1	-0.467897484	-0.029348859	0.003384195	0.987454031	Potri.001G078900.1	AT5G49720.1
Potra2n1c637.1	-0.669335939	-0.102341775	2.65876E-06	0.903934429	Potri.001G079000.1	AT5G49730.1
Potra2n1c638.1	-0.692019783	-0.185877843	3.81231E-05	0.833401866		
Potra2n1c639.1	-0.362888465	-0.18271507	0.028902126	0.744452143	Potri.001G079100.1	AT5G07590.1
Potra2n1c64.1					Potri.003G218300.1	AT4G30380.1
Potra2n1c640.1	-0.529179562	-0.170445622	0.293835001	0.946617249	Potri.001G079201.1	
Potra2n1c641.1	-0.030760288	0.058975199	0.866843077	0.959881296	Potri.001G079300.1	AT2G44900.1
Potra2n1c642.1	-0.497041483	0.045851378	0.001300398	0.975138549	Potri.001G079400.4	AT5G51180.1
Potra2n1c643.1	0.503466569	-2.033943134	0.894705435	NA	Potri.001G079600.1	AT5G07580.1
Potra2n1c644.1	2.804043026	1.084699694	1.04244E-10	0.52733716	Potri.001G079800.1	AT5G51190.1
Potra2n1c645.1	2.764864229	1.059555119	8.98175E-11	0.536358516		
Potra2n1c646.1	2.158511185	0.133323783	0.00077058	0.980155789	Potri.001G079900.1	AT4G17500.1
Potra2n1c647.1	2.282771052	2.490806657	NA	NA	Potri.001G080000.1	AT4G25140.1
Potra2n1c648.1	-1.35927387	-1.507841271	NA	NA	Potri.001G080100.1	AT5G07500.1
Potra2n1c649.1	-0.251576957	-0.033295129	0.074571491	0.95981322	Potri.001G080200.1	AT5G51230.1

Potra2n1c65.1	-0.514536551	0.224329887	0.148651302	0.927536125	Potri.001G007100.1	AT1G55290.1
Potra2n1c650.1	1.545832463	0.674963689	NA	NA	Potri.001G080300.1	AT5G61620.1
Potra2n1c651.1	-0.308994298	-0.110755823	0.023193084	0.924309478	Potri.001G080400.1	AT1G02130.1
Potra2n1c652.1					Potri.001G080500.1	AT5G61630.1
Potra2n1c653.1	NA	NA	NA	NA	Potri.001G080600.1	AT5G07480.1
Potra2n1c654.1	3.090168383	1.361687302	0.007151469	NA	Potri.001G080700.1	AT5G07475.1
Potra2n1c655.1	NA	NA	NA	NA		
Potra2n1c656.1	0.878702723	-0.097655521	NA	NA	Potri.001G080900.2	AT2G43000.1
Potra2n1c657.1	-1.35927387	-1.507841271	NA	NA	Potri.003G149600.1	AT4G19610.1
Potra2n1c658.1	1.238538357	0.607646884	5.28629E-14	0.180908519	Potri.001G081100.1	AT3G06260.1
Potra2n1c659.1	4.557413534	2.283425176	0.019852064	NA	Potri.001G081200.1	AT1G72700.1
Potra2n1c66.1	0.152522647	0.116410171	0.64077262	0.948577865	Potri.001G007300.1	AT3G13620.1
Potra2n1c660.1	-0.112747482	-0.284889634	0.570831101	0.534184071	Potri.001G081300.1	AT1G80245.1
Potra2n1c661.1	0.5090496	-0.420843737	0.051856115	0.824378527	Potri.001G081400.1	AT1G31320.1
Potra2n1c662.1	-0.748244395	-0.487641323	5.34269E-07	0.105402179	Potri.001G081500.1	AT1G31330.1
Potra2n1c663.2	0.1196816	0.14629952	0.252428346	0.777047113	Potri.001G081600.1	AT5G45190.1
Potra2n1c664.1	0.152349566	0.066415097	0.174733317	0.912665563	Potri.001G081900.1	AT1G22860.1
Potra2n1c665.1	0.150602791	0.161918925	0.283240166	0.833667976	Potri.001G082000.3	AT5G16950.1
Potra2n1c666.1	0.334842068	0.363792143	9.30496E-05	0.367890074	Potri.001G082100.1	AT5G16780.1
Potra2n1c667.1	0.625231459	0.239920034	0.000312768	0.818971264	Potri.001G082150.1	AT3G54780.1
Potra2n1c668.1	2.14699796	1.030475003	0.104619882	NA	Potri.001G082200.1	AT5G48655.2
Potra2n1c669.1	2.250683731	1.410554108	3.54592E-05	0.524600623	Potri.003G148200.1	AT2G30070.1
Potra2n1c67.1	0.847525688	0.254335267	0.006615913	0.663950227	Potri.001G007300.1	AT3G13620.1
Potra2n1c670.1	2.055644822	1.053929606	0.001846708	0.773063484		
Potra2n1c671.1	-0.652611726	-0.626156666	0.150735098	0.694477853	Potri.001G082332.1	AT1G31335.1
Potra2n1c672.1	-1.323671972	-0.788306862	0.000723087	0.314225571	Potri.001G082400.3	AT4G24120.1
Potra2n1c673.1	1.543212559	1.004199754	0.000463782	0.788065047	Potri.001G082500.1	AT4G24110.1
Potra2n1c674.1	0.729404685	-0.765978345	0.564531952	NA	Potri.001G082600.1	AT1G64690.1
Potra2n1c675.1	-0.643926487	0.015359483	0.870899261	NA	Potri.001G082700.1	AT4G24150.1
Potra2n1c676.1	0.272499563	0.102337388	0.246792762	0.926817497	Potri.001G082800.2	AT4G19550.1
Potra2n1c677.1	0.852829116	-0.291494747	0.057522933	0.914551931	Potri.001G082900.1	AT1G56140.1
Potra2n1c678.1	0.980086571	0.063992414	0.034380468	0.989708171	Potri.001G082900.1	AT1G56140.1

Potra2n1c679.1	2.321797603	0.077092338	1.22108E-09	0.991110595	Potri.001G082900.1	AT1G56140.1
Potra2n1c68.1	-0.256297426	-1.743484761	0.849970044	NA	Potri.001G007400.1	AT5G09280.1
Potra2n1c680.1	-0.060497937	-0.060996562	0.77491083	0.966917036	Potri.001G083100.1	AT5G42090.1
Potra2n1c681.1	9.870643318	7.257781601	4.00981E-18	0.192841261	Potri.001G083200.1	AT1G02800.1
Potra2n1c682.1	-1.743405462	-0.782995419	3.69249E-09	0.180635806	Potri.001G083300.1	AT1G64680.1
Potra2n1c683.1	1.943019694	0.393199678	0.174128414	NA	Potri.001G083400.1	AT4G24140.1
Potra2n1c684.1						
Potra2n1c685.1	0.916706502	-0.007373616	0.270124027	0.999325699	Potri.001G083500.1	AT1G32640.1
Potra2n1c686.1	3.53785401	2.513821984	0.00081727	0.582190571	Potri.001G083600.1	AT1G14430.1
Potra2n1c687.1	-1.460583247	0.252595636	0.128799754	0.965215635	Potri.001G083700.1	AT2G46680.1
Potra2n1c688.3	0.240573464	0.228669445	0.086995036	0.630774341	Potri.001G083800.5	AT1G26665.1
Potra2n1c689.1	0.906188328	0.599012062	0.473257598	0.881624318	Potri.001G083900.1	AT2G46660.1
Potra2n1c69.1						
Potra2n1c690.1	1.124385687	0.232541185	0.014104053	0.936170918	Potri.001G084000.1	AT1G64660.1
Potra2n1c691.1	-1.077226084	-0.093499854	5.24997E-05	0.978646026	Potri.001G084200.1	AT4G24130.1
Potra2n1c692.1	-0.395920803	-0.101340991	0.007223494	0.906985723	Potri.001G084300.1	AT5G41940.1
Potra2n1c693.3	1.464808341	0.413945483	6.99145E-07	0.70570456	Potri.001G084400.1	AT4G24120.1
Potra2n1c694.1	-1.640490994	-0.347289516	8.38276E-09	0.765480128	Potri.001G084500.2	AT4G24090.1
Potra2n1c695.1	0.011773975	0.169856477	0.920224147	0.811104553	Potri.001G084700.1	AT4G10730.1
Potra2n1c696.1	0.92641307	-0.767708831	NA	NA	Potri.003G145900.1	AT5G41970.1
Potra2n1c697.1	1.325633114	-0.801499921	0.622254568	NA	Potri.001G084800.1	
Potra2n1c698.1	0.12988246	-0.006418377	0.804085636	0.999121696	Potri.003G145700.1	AT4G10750.1
Potra2n1c699.1	0.130529726	-0.022417171	0.23495522	0.983113084	Potri.001G085200.1	AT4G10760.1
Potra2n1c7.1	0.283198191	0.029880977	0.018933009	0.975578749		
Potra2n1c70.1	1.070250682	0.529229505	0.038487861	0.811159921	Potri.001G007501.1	AT2G20142.1
Potra2n1c700.1	-0.777187643	-0.491898223	0.024596691	0.855432225	Potri.001G085100.1	AT1G64640.1
Potra2n1c701.1	-0.393449832	0.159762329	0.004948208	0.831463341	Potri.001G085400.1	AT4G27745.1
Potra2n1c702.1	-0.07295353	-0.038456396	0.62837642	0.971752582	Potri.001G085500.1	AT5G41990.1
Potra2n1c703.1	0.001896434	-0.047905502	0.988586154	0.947414934	Potri.001G085600.1	AT4G10790.1
Potra2n1c704.1	0.82357019	NA	NA	NA	Potri.003G145100.1	AT5G63830.1
Potra2n1c705.1	-2.318502538	0.000494837	0.004267684	0.999937671	Potri.001G085900.1	AT4G00910.1
Potra2n1c706.1	-1.656716411	-0.220013868	4.37845E-14	0.885375133	Potri.001G086000.1	AT1G64625.2

Potra2n1c707.1	0.1976014	0.051972688	0.485733338	0.9761362		
Potra2n1c708.1	0.034705924	-0.042209373	0.833001913	0.960981446	Potri.001G086100.1	AT3G05675.2
Potra2n1c709.1	0.050585971	0.073503107	0.827214623	0.949048812	Potri.001G086300.1	AT5G42000.1
Potra2n1c71.1	1.059461181	0.142117906	0.526463362	NA	Potri.003G218000.1	AT5G17230.1
Potra2n1c710.1	-0.236437161	-0.270857545	0.082867403	0.567971082	Potri.001G086400.4	AT4G24060.1
Potra2n1c711.1	0.260702522	-0.077350746	0.059561302	0.948577865	Potri.001G086600.1	AT5G24320.1
Potra2n1c712.1	NA	NA	NA	NA	Potri.001G086700.1	AT5G49330.1
Potra2n1c713.1	0.164600133	0.108431647	0.332550059	0.937846405	Potri.001G086800.1	AT1G64600.1
Potra2n1c714.1	-3.297168727	-1.492888154	NA	NA	Potri.001G086900.1	AT4G24050.1
Potra2n1c715.1	1.042343664	0.132649306	9.94998E-08	0.903698951	Potri.001G087100.1	AT4G24040.1
Potra2n1c716.1	-0.37743422	-0.071958421	0.01606506	0.967969623	Potri.001G087200.3	AT4G10810.1
Potra2n1c717.1	0.564796835	0.257036339	2.70678E-07	0.473068322	Potri.001G087300.2	AT1G64550.1
Potra2n1c718.1	-0.285822177	-0.211347984	0.084223798	0.720488481	Potri.001G087400.1	AT1G55090.1
Potra2n1c719.1	0.315396226	0.08995687	0.069387851	0.949485515	Potri.001G087500.1	AT5G42020.1
Potra2n1c72.1	1.178991223	0.047619576	0.517128212	NA	Potri.001G007600.2	AT5G17230.3
Potra2n1c720.1	-0.1900847	-0.118096636	0.065910188	0.827421725	Potri.001G087600.1	AT3G23920.1
Potra2n1c721.1	-0.185402077	-0.452087042	0.585548079	0.832270846	Potri.001G087701.1	
Potra2n1c722.1	-0.631286068	-0.200505154	0.000184114	0.842247476	Potri.001G087800.1	AT4G10840.1
Potra2n1c723.1	-1.126912143	-0.230286478	3.18594E-11	0.773063484	Potri.001G087900.1	AT4G24020.1
Potra2n1c724.1	-0.374181441	-0.048495137	0.00185318	0.957686558	Potri.001G088000.1	AT5G63610.1
Potra2n1c725.1	1.503933991	0.544709869	0.000768888	0.696847835	Potri.001G088100.1	AT3G49290.1
Potra2n1c726.1	0.044766161	-0.001791184	0.712154051	0.999315506	Potri.001G088200.1	AT1G64520.1
Potra2n1c727.1	-0.72376935	0.011329507	0.001324165	0.996032145	Potri.001G088300.1	AT1G64510.1
Potra2n1c728.1	NA	NA	NA	NA	Potri.003G142400.1	AT4G23990.1
Potra2n1c729.1	-0.481159643	0.06799287	0.019128687	0.972470034	Potri.001G088600.3	AT4G23980.1
Potra2n1c73.1	NA	NA	NA	NA	Potri.001G007700.1	AT5G17230.2
Potra2n1c730.3	0.203188763	0.843292434	NA	NA	Potri.001G088700.1	AT5G46290.1
Potra2n1c731.1	0.903732989	-0.31309881	9.1806E-06	0.755261745	Potri.001G088800.3	AT5G42050.1
Potra2n1c732.1	-1.236959908	-1.828154868	0.589678164	NA	Potri.003G141800.1	AT1G64500.1
Potra2n1c733.2	-0.087956314	-0.157671181	0.655175107	0.849532804	Potri.001G089100.1	AT5G10560.1
Potra2n1c734.1	-1.616660676	-1.057835228	0.012229647	0.585361878	Potri.001G089000.1	AT3G61920.1
Potra2n1c735.1	0.717059267	0.587539476	0.581954627	0.914551931	Potri.001G089201.1	AT4G16580.1

Potra2n1c736.1	-0.128302583	-0.012447966	0.488482865	0.989329144	Potri.001G089300.1	AT3G13550.1
Potra2n1c737.1	0.161107788	-0.015656779	0.386231469	0.989878071	Potri.001G089400.1	AT4G10920.2
Potra2n1c738.2	-0.784645153	0.29504742	0.000264654	0.89372314	Potri.001G089500.5	AT5G42070.1
Potra2n1c739.2	-0.166059986	-0.129312595	0.255538129	0.87909151	Potri.001G089600.1	AT4G10925.3
Potra2n1c74.1	2.492428534	1.571529889	NA	NA	Potri.001G207400.1	
Potra2n1c740.1	0.198160942	0.27106951	0.245823097	0.741833788	Potri.001G089700.1	AT4G23950.1
Potra2n1c741.1	0.154052821	0.085173185	0.269839529	0.928154618	Potri.001G089800.2	AT4G23940.1
Potra2n1c742.2	0.041274797	0.235008393	0.711373724	0.544330018	Potri.001G089900.2	AT4G10930.1
Potra2n1c743.1	-0.158273026	0.149629496	0.468013221	0.880553952	Potri.001G090000.1	AT2G19900.1
Potra2n1c744.1	1.712516638	1.297743307	0.06721147	0.606364174	Potri.001G090200.10	AT5G24270.2
Potra2n1c745.1	-0.887690087	-0.255345227	8.1937E-10	0.29966352	Potri.001G090300.1	AT2G46500.2
Potra2n1c746.1	-0.731360706	-0.663841658	0.011313762	0.177918242	Potri.003G141200.1	AT1G64450.1
Potra2n1c747.1	-0.768466471	-0.204034892	0.000289957	0.859892315	Potri.001G090500.1	AT4G10955.1
Potra2n1c748.1	-0.104606998	-0.102620481	0.545740621	0.940449094	Potri.001G090600.2	AT5G42080.1
Potra2n1c749.1	-0.806086183	-0.347967731	3.48999E-07	0.563971296	Potri.001G090700.1	AT4G10960.1
Potra2n1c75.2	0.260073612	-0.250977015	0.603589744	0.903698951		
Potra2n1c750.1	-0.62181051	-0.435468693	0.036727667	0.72101822	Potri.001G090800.1	AT1G64430.2
Potra2n1c751.1	0.112436675	0.032559993	0.592495752	0.976302215	Potri.001G090900.1	AT4G10970.3
Potra2n1c752.1	0.367589162	0.120662506	0.13026365	0.955473249	Potri.003G140600.1	AT4G23900.1
Potra2n1c753.1	0.291658424	0.011765702	0.00178904	0.988862007	Potri.001G091100.1	AT4G23895.3
Potra2n1c754.1	-0.593553385	0.245521376	0.041504472	0.922029226	Potri.001G091300.1	AT4G23890.1
Potra2n1c755.1	4.155908013	1.82613075	9.749E-12	0.565491408	Potri.001G091400.2	AT4G23880.1
Potra2n1c756.1	9.619483495	3.818459843	5.0634E-18	NA	Potri.001G091500.1	AT1G64405.1
Potra2n1c757.1	0.222601909	0.050106735	0.177600169	0.967122333	Potri.001G091600.1	AT4G23860.2
Potra2n1c758.1	0.624525774	-0.594236602	0.338084764	0.888571801	Potri.001G091700.1	AT5G53110.1
Potra2n1c759.1	0.449516584	0.148702559	0.002639802	0.872698255	Potri.001G091800.2	AT5G62050.1
Potra2n1c76.1	1.670381921	0.674310143	0.022375718	0.849446235	Potri.001G007501.1	AT2G20142.1
Potra2n1c760.1	0.285256547	0.0430587	0.005484344	0.969542045	Potri.001G091900.2	AT4G23850.1
Potra2n1c761.1	0.292972621	0.554623921	0.020662851	0.579724936	Potri.001G092000.1	AT4G23840.1
Potra2n1c762.1	1.316651904	0.593891562	0.000406297	0.820966257	Potri.001G092100.1	AT2G29380.1
Potra2n1c763.2	1.45490246	0.323877893	1.33296E-05	0.937789963	Potri.001G092200.1	AT1G64390.1
Potra2n1c764.1	1.82697449	-0.107273711	0.186607206	NA		

Potra2n1c765.1	-0.1294519	-0.026498789	0.61702876	0.987226805	Potri.001G092300.1	AT1G64385.1
Potra2n1c766.1	-1.828127997	0.203862772	0.517117305	NA	Potri.001G092400.1	AT1G64380.1
Potra2n1c767.1	0.255917489	0.301383386	0.116914938	0.723658895	Potri.001G092500.1	AT5G53160.2
Potra2n1c768.1	1.580085058	0.516563686	2.14929E-11	0.700153847	Potri.001G092600.1	AT5G41860.1
Potra2n1c769.1	-1.74684456	-0.539068246	0.001051804	0.802942736	Potri.001G092700.1	AT2G19170.1
Potra2n1c77.1	0.902780416	0.477057472	0.423177387	0.939824856	Potri.001G007700.1	AT5G17230.2
Potra2n1c770.1	0.081727961	-0.271494139	0.777122002	0.835819291	Potri.001G092800.1	AT5G41850.1
Potra2n1c771.1	NA	NA	NA	NA	Potri.001G093100.1	
Potra2n1c772.1	2.1748422	1.306501289	6.73172E-10	0.016522993	Potri.001G092900.1	AT4G11070.1
Potra2n1c773.1	0.962986086	0.853440799	0.002732535	0.031255998	Potri.003G138500.1	AT1G64355.1
Potra2n1c774.1	NA	NA	NA	NA	Potri.001G093100.1	
Potra2n1c775.1	-4.117669484	0.144656138	0.118957846	NA	Potri.003G138400.1	AT5G42800.1
Potra2n1c776.1	-0.2755208	-0.858251464	0.846630807	0.937846405	Potri.001G093500.1	AT5G41810.2
Potra2n1c777.5	-0.221531255	-0.032925313	0.161028354	0.986636967	Potri.001G093700.1	AT4G11080.1
Potra2n1c778.1	0.76078425	-0.552609398	0.113102943	0.832750076		
Potra2n1c779.1	-0.442604753	-0.290639382	0.101193939	0.888896953	Potri.001G093800.1	AT4G11090.1
Potra2n1c78.1	-0.193259533	-0.108497093	0.099684996	0.889106941	Potri.001G007800.1	AT2G42500.1
Potra2n1c780.1	-0.245597204	0.165130318	0.296821018	0.914903229	Potri.001G093900.1	AT3G61670.1
Potra2n1c781.1	0.432316008	0.08172557	0.846920956	NA	Potri.001G094000.1	AT3G61660.1
Potra2n1c782.1	-0.057190736	0.052949462	0.5530665	0.956865545	Potri.001G094100.1	AT4G01100.1
Potra2n1c783.1	0.952375982	-0.424655395	0.169849985	0.88643982	Potri.001G094200.2	AT5G41790.1
Potra2n1c784.1	-0.136925065	-0.071723524	0.245813945	0.907073354	Potri.001G094400.1	AT5G41770.1
Potra2n1c785.2	-0.456427298	-0.070106866	9.21956E-07	0.917539064	Potri.001G094500.1	AT4G11110.1
Potra2n1c786.2	-0.131692976	-0.145102329	0.42967252	0.875559093	Potri.001G094600.1	AT5G41760.1
Potra2n1c787.1	-0.198449441	-0.456862525	0.729468854	0.833952007		
Potra2n1c788.1	1.697260093	0.453527632	0.404928883	NA	Potri.001G094800.1	AT4G23750.2
Potra2n1c789.1	-0.003878737	-0.032847024	0.968678999	0.959881296	Potri.001G094900.1	AT1G64230.2
Potra2n1c79.1	-0.253464566	-0.096287983	0.015110014	0.924309478	Potri.001G007900.2	AT2G42520.1
Potra2n1c790.1	-1.087430673	-0.534695204	5.92248E-06	0.586463643	Potri.001G095200.1	AT4G23740.1
Potra2n1c791.1	-0.575361031	-0.41929711	0.154791824	0.848005416		
Potra2n1c792.1	0.445441176	0.418212404	0.067985208	0.416378557	Potri.001G095300.1	AT3G61610.1
Potra2n1c793.1	-0.61910233	-0.767708831	NA	NA	Potri.001G095400.1	AT5G53340.1

Potra2n1c794.1	0.241962727	-0.098618793	0.020553711	0.930610374	Potri.001G095500.1	AT4G11160.1
Potra2n1c795.1	0.767361742	0.130867594	0.61702876	NA	Potri.001G095600.1	AT3G59140.1
Potra2n1c796.1	0.136345775	-0.052204611	0.423402018	0.972159498	Potri.001G095800.1	AT5G41670.2
Potra2n1c797.1	-0.138266091	-0.221972975	0.843382869	0.944081499	Potri.001G095900.1	AT1G60500.1
Potra2n1c798.1	-0.090338498	-0.095374391	0.911663036	0.983586241	Potri.001G096000.1	AT4G23720.1
Potra2n1c799.1	0.04393211	-0.065453404	0.76176637	0.936549944	Potri.001G096100.1	AT3G61600.1
Potra2n1c8.1	-1.261759122	-0.350234261	1.49428E-06	0.70570456	Potri.001G000800.1	AT1G55550.1
Potra2n1c80.1	-0.632455371	-0.314761926	0.006632161	0.777553704	Potri.001G008000.1	AT1G55270.1
Potra2n1c800.1	-0.249007299	-0.0103207	0.335126284	0.996032145	Potri.001G096200.1	AT5G41620.1
Potra2n1c801.1	-0.327142159	-0.084824007	0.254093728	0.95671968	Potri.001G096400.1	AT5G41610.1
Potra2n1c802.1	-0.943349112	-0.060358475	0.008305544	0.985393296	Potri.001G096900.1	AT4G23690.1
Potra2n1c803.1	-2.142972408	0.17135493	0.000604371	0.957163493	Potri.001G097100.1	AT1G64150.1
Potra2n1c804.1	-0.206676089	-0.206063548	0.078920145	0.802942736	Potri.001G097200.3	AT1G64140.1
Potra2n1c805.1	-1.409983009	-1.55593228	NA	NA	Potri.001G097300.1	AT3G18440.1
Potra2n1c806.1	-0.016236543	-0.024739527	0.900292979	0.978251387	Potri.001G097400.1	AT4G23650.1
Potra2n1c807.1	-0.125424267	-0.029153465	0.258248719	0.974275337	Potri.001G097500.1	AT5G60750.1
Potra2n1c808.1	-0.267484992	0.280853712	0.408752972	0.810712263	Potri.001G097600.2	AT1G64110.2
Potra2n1c809.1	-0.023838071	0.002927488	0.81547267	0.996307365	Potri.001G097700.1	AT1G64090.1
Potra2n1c81.1	1.459962178	NA	NA	NA	Potri.003G217600.1	
Potra2n1c810.1	0.261423838	-0.094721504	0.152316719	0.948834049	Potri.001G097800.1	AT4G23620.1
Potra2n1c811.1	-1.246325551	-0.523208586	0.094159283	0.881090429	Potri.T144300.1	AT1G13130.1
Potra2n1c812.1	-0.007846921	-0.177349512	0.996872848	0.986636967	Potri.001G098000.1	AT1G64080.1
Potra2n1c813.1	4.919849051	3.041958117	8.11007E-06	0.576228147	Potri.001G098100.1	AT2G46150.1
Potra2n1c814.1	-0.763740661	-0.908917948	0.749848389	NA	Potri.001G098200.1	AT1G64065.1
Potra2n1c815.1	1.263085808	0.022066328	1.95667E-09	0.994692557	Potri.001G098300.1	AT1G64060.1
Potra2n1c816.1	1.387160845	-0.653476958	0.049114429	0.931774514	Potri.001G098400.1	AT5G41590.1
Potra2n1c817.1	0.318027	0.158102882	0.004220849	0.768280439	Potri.001G098500.1	AT1G64050.1
Potra2n1c818.2	0.103387735	-0.072311885	0.469384155	0.930203195	Potri.001G098600.1	AT4G11240.1
Potra2n1c819.1	-0.122350171	0.048731151	0.458409658	0.949176188	Potri.001G098700.1	AT4G11270.1
Potra2n1c82.1	NA	NA	NA	NA	Potri.001G008100.1	AT4G26130.1
Potra2n1c820.1	-1.686086308	-0.455074372	2.93726E-05	0.701431845	Potri.001G098900.1	
Potra2n1c821.1	1.664677056	0.674963689	NA	NA	Potri.001G099001.1	AT4G01250.1

Potra2n1c822.1	0.159656482	-0.16712051	0.922729627	0.9869326	Potri.001G099100.1	AT1G63990.1
Potra2n1c823.1	0.657626847	0.249251594	6.68798E-06	0.582021931	Potri.001G099200.1	AT5G41560.1
Potra2n1c824.1	0.508414278	0.1418493	0.073655534	0.926428079	Potri.001G099300.1	AT1G63970.1
Potra2n1c825.1	3.358470778	1.233629859	1.59859E-25	0.038179555	Potri.001G099400.1	AT4G11280.1
Potra2n1c826.1	-0.851740293	-2.446267299	NA	NA	Potri.001G099500.2	AT1G01490.1
Potra2n1c827.1	0.256579722	0.136645477	0.144675121	0.89321847	Potri.001G099600.1	AT1G63940.2
Potra2n1c828.1	-0.318999648	-0.098035325	0.118333046	0.948834049		
Potra2n1c829.1	-0.96942616	-0.214249708	1.97483E-05	0.918687733	Potri.001G099800.1	AT1G63910.1
Potra2n1c83.1	NA	NA	NA	NA		
Potra2n1c830.1	0.559403575	0.192410448	0.00051152	0.825833782	Potri.002G162500.1	AT4G01370.1
Potra2n1c831.1	0.631805749	0.261918903	0.000314361	0.792511865	Potri.001G100000.1	AT4G23500.1
Potra2n1c832.1	-0.784408637	-0.04050519	1.37443E-07	0.976043496	Potri.001G100100.1	AT1G69230.1
Potra2n1c833.1	0.163020139	-0.132541383	0.411948508	0.936890993	Potri.001G100200.1	AT4G25700.1
Potra2n1c834.1	-0.185208082	-0.081000413	0.438128167	0.947547562	Potri.001G100300.1	AT1G28140.1
Potra2n1c835.1	1.545832463	3.970049737	NA	NA	Potri.001G100500.1	AT5G41470.1
Potra2n1c836.1	0.323468568	0.025048617	0.366620808	0.993589787	Potri.001G100600.1	AT4G13650.1
Potra2n1c837.1	0.960555207	0.886004889	0.570831101	NA		
Potra2n1c838.1	-0.956336045	-0.149516873	2.74959E-05	0.949966783	Potri.001G100700.1	AT5G41460.1
Potra2n1c839.1	NA	NA	NA	NA		
Potra2n1c84.1						
Potra2n1c840.1	3.078394927	0.908323476	6.24605E-08	0.862335622	Potri.001G100800.2	AT5G41410.1
Potra2n1c841.1	0.124120933	0.013209936	0.60733085	0.996032145	Potri.001G100900.1	AT1G63850.1
Potra2n1c842.3	1.104227317	0.217199855	2.93084E-15	0.71411664	Potri.001G101100.2	AT1G63830.2
Potra2n1c843.1	1.545832463	NA	NA	NA	Potri.001G101200.1	AT5G41380.1
Potra2n1c844.1	0.27062527	0.067131057	0.034072415	0.936890993	Potri.001G101300.2	AT5G41370.1
Potra2n1c845.1	0.26791169	0.041487537	0.038161234	0.974367223	Potri.001G101400.1	AT4G23460.1
Potra2n1c846.4	-1.018415483	-2.287247387	0.044867048	0.021753094		
Potra2n1c847.1	-0.174603509	0.160762939	0.205546161	0.865591169	Potri.001G101600.1	AT1G75060.2
Potra2n1c848.1	0.524120753	0.324892837	1.71437E-06	0.202545031	Potri.001G101700.6	AT5G41350.1
Potra2n1c849.1	-0.353809225	0.202541335	0.024776267	0.804796567	Potri.001G101800.1	AT4G11400.1
Potra2n1c85.1					Potri.T038300.2	AT5G36930.2
Potra2n1c850.1	-0.393692863	0.202776832	0.010108572	0.788066587		

Potra2n1c851.2	-0.096553705	-0.052350841	0.552481165	0.950182516	Potri.001G101900.1	AT1G63800.1
Potra2n1c852.1	0.389160683	0.082960125	0.001797988	0.935474933	Potri.001G102000.3	AT1G63770.3
Potra2n1c853.1	0.713888148	0.437901181	0.008727915	0.699892892	Potri.001G102200.1	AT5G52430.1
Potra2n1c854.1	0.763442535	0.186616894	0.057202153	0.915020432	Potri.001G102300.1	AT5G41330.1
Potra2n1c855.1	8.349805211	0.394326816	4.6104E-31	0.974756511	Potri.001G102400.1	AT4G11650.1
Potra2n1c856.1	8.561343676	0.908979409	4.44205E-20	0.93125845	Potri.001G102400.1	AT4G11650.1
Potra2n1c857.1	1.559005433	0.581151994	3.96373E-10	0.185836749	Potri.003G123600.1	AT1G12770.1
Potra2n1c858.1	-0.323499563	0.586213652	0.641233803	0.809914834		
Potra2n1c859.1	1.876952302	0.427632322	2.10545E-10	0.660636854	Potri.001G102600.1	AT4G11655.1
Potra2n1c86.1	NA	1.571529889	NA	NA		
Potra2n1c860.1	1.904961694	0.623730903	1.72137E-11	0.163343066	Potri.001G102600.1	AT4G11655.1
Potra2n1c861.1	-1.328951947	-1.478536539	NA	NA	Potri.001G102700.1	AT1G12780.1
Potra2n1c862.1	0.46988108	0.233082162	1.2543E-05	0.701494362	Potri.001G102900.10	AT1G63700.1
Potra2n1c863.1	-2.286316826	-1.716336752	NA	NA	Potri.001G103000.1	AT4G11410.1
Potra2n1c864.1	-0.097181436	-0.032034935	0.492081113	0.974367223	Potri.001G103100.1	AT1G63690.1
Potra2n1c865.1	0.82357019	1.394613675	NA	NA	Potri.001G103200.2	
Potra2n1c866.1	0.82357019	NA	NA	NA		
Potra2n1c867.1	-0.698841756	-0.331283166	0.049866957	0.876272842	Potri.001G103300.1	AT1G63680.1
Potra2n1c868.1	0.259100723	0.29297347	0.6492367	0.915358268	Potri.001G103400.3	AT1G63670.3
Potra2n1c869.1	1.916638423	0.490491595	5.6261E-17	0.704663803	Potri.001G103600.2	AT5G41315.1
Potra2n1c87.1	-0.390362023	-0.469763947	0.141696901	0.316032926	Potri.T157100.1	AT1G55265.1
Potra2n1c870.1	0.201302255	-0.04152473	0.131226421	0.968554995	Potri.001G103800.1	AT4G23330.2
Potra2n1c871.1	-1.328951947	1.674078742	NA	NA	Potri.001G103900.1	AT4G26400.1
Potra2n1c872.2	0.269085648	0.076769968	0.128358836	0.949966783	Potri.001G104000.1	AT1G63640.1
Potra2n1c873.1	-1.901113498	0.99706042	0.605332844	NA		
Potra2n1c874.1	-1.901113498	0.321444593	0.605332844	NA	Potri.003G127600.1	AT4G11440.1
Potra2n1c875.1	-0.355121834	-0.038046018	0.014366959	0.9761362	Potri.001G104200.1	AT4G11450.1
Potra2n1c876.1	-0.172191272	0.199604521	0.158222939	0.750123244	Potri.001G104300.1	AT4G11560.1
Potra2n1c877.1	5.449725335	1.394613675	0.010887157	NA	Potri.003G127300.1	AT4G11560.1
Potra2n1c878.1	0.15949647	-0.173239139	0.485356483	0.909675463	Potri.T159100.1	AT4G11570.2
Potra2n1c879.1	0.641906548	0.070730838	0.012824228	0.925184207	Potri.001G104500.1	AT4G23100.1
Potra2n1c88.1	-0.133956096	-0.129887867	0.339378256	0.886059841	Potri.001G008300.1	AT1G55265.1

Potra2n1c880.1	-0.941650478	0.177768217	0.000878134	0.941855873	Potri.001G104600.1	AT5G12250.1
Potra2n1c881.1	0.26735135	0.130602804	0.007315325	0.775284329	Potri.001G104700.1	AT1G63500.1
Potra2n1c882.1	0.010124142	-0.084692635	0.974398049	0.954823693	Potri.001G104800.10	AT3G52905.1
Potra2n1c883.1	0.564618649	0.196852533	0.001593368	0.903477395	Potri.001G104900.5	AT1G63470.1
Potra2n1c884.1	0.725714806	0.320244231	0.000324778	0.874648591	Potri.001G105100.5	AT1G63460.1
Potra2n1c885.1	1.367504818	0.242042223	2.10506E-19	0.70986976	Potri.001G105200.1	AT4G11600.1
Potra2n1c886.1	3.0527749	3.037269708	0.034737965	0.645919795	Potri.001G105400.1	AT4G11610.1
Potra2n1c887.1	0.251506181	0.799380627	0.066504719	0.432156259	Potri.001G105600.1	AT5G41240.1
Potra2n1c888.1	0.033600089	1.396966852	NA	NA	Potri.003G125700.1	AT1G63440.1
Potra2n1c889.1	-0.751865663	0.214577176	0.086353315	0.907073354	Potri.001G105800.1	AT1G63440.1
Potra2n1c89.1	-0.235364858	0.150537914	0.109293739	0.935474933	Potri.001G008400.1	AT5G19860.1
Potra2n1c890.1	1.119584195	0.659945406	1.16567E-06	0.149735105	Potri.001G105900.1	AT3G48990.1
Potra2n1c891.2	0.19256469	0.140455251	0.148409821	0.860325259	Potri.001G106000.1	AT4G11640.1
Potra2n1c892.1	-1.383548794	0.238666829	0.042864969	0.948577865	Potri.001G106100.1	AT5G23860.2
Potra2n1c893.1	0.258445838	4.328467891	0.93392193	0.497568278	Potri.001G106200.1	AT4G00680.1
Potra2n1c894.1					Potri.001G106300.1	AT1G63410.1
Potra2n1c895.1					Potri.001G106400.1	AT1G63410.1
Potra2n1c896.1	-0.96993596	-0.275294315	1.33036E-06	0.821709751	Potri.001G106600.1	AT1G63430.1
Potra2n1c897.1	1.134647142	0.972121298	0.027959475	0.425472871	Potri.001G106700.1	AT1G63300.1
Potra2n1c898.1	NA	NA	NA	NA	Potri.001G106800.1	AT3G51970.1
Potra2n1c899.1	-1.183712747	-0.219086831	9.32605E-06	0.927879595	Potri.001G107000.1	AT5G23750.1
Potra2n1c9.1	-0.211835517	-0.05762441	0.110581732	0.961573158	Potri.001G000900.2	AT3G13410.1
Potra2n1c90.2	0.488666024	0.38803688	0.001548916	0.646274123	Potri.001G008500.1	AT2G44290.1
Potra2n1c900.1	NA	4.370782683	NA	NA	Potri.001G107100.3	AT3G61230.1
Potra2n1c901.1	-0.225604508	0.025889839	0.132849851	0.989406722	Potri.001G107200.1	AT1G63260.2
Potra2n1c902.1	0.639543788	-0.002751129	0.000621736	0.999458638	Potri.001G107300.1	AT1G63250.1
Potra2n1c903.1	3.488491071	2.383329394	0.010050213	NA	Potri.001G107400.1	
Potra2n1c904.1	0.443467187	0.084425731	0.001309169	0.887678043	Potri.001G107500.1	AT4G20330.1
Potra2n1c905.1					Potri.001G107700.1	AT4G11650.1
Potra2n1c906.1	5.617964965	5.594235119	0.008106723	NA	Potri.001G107800.1	AT4G11650.1
Potra2n1c907.1	-1.463943102	-0.493454627	4.13378E-05	0.593648258	Potri.003G123600.1	AT1G12770.1
Potra2n1c908.1	-0.420294059	-0.146782754	0.001739873	0.88066693	Potri.001G108100.1	AT4G11660.1

Potra2n1c909.1	-0.426384222	-0.400114852	0.019218544	0.616547434	Potri.001G108200.1	AT3G47670.1
Potra2n1c91.1	-0.329230588	0.719899806	0.866683948	0.895474078	Potri.001G008600.1	AT1G11880.1
Potra2n1c910.1	4.849871768	2.101539572	9.78842E-16	0.435083833	Potri.001G108300.1	AT2G45760.1
Potra2n1c911.1	0.05428408	0.10177798	0.741041431	0.867540758	Potri.001G108400.1	AT4G11670.1
Potra2n1c912.1	NA	NA	NA	NA		
Potra2n1c913.1	NA	NA	NA	NA	Potri.003G123101.1	AT2G36620.1
Potra2n1c914.1	-0.480641053	0.142281002	0.452015227	0.973347974	Potri.001G108500.1	AT5G66520.1
Potra2n1c915.1	2.632524198	1.571529889	NA	NA	Potri.001G108600.1	AT1G25270.1
Potra2n1c916.1	-1.053304685	-0.253161011	0.000245306	0.896937427	Potri.001G108700.1	AT1G63120.1
Potra2n1c917.1	3.106726791	0.961136433	4.00981E-18	0.344692295	Potri.001G108800.1	AT4G23060.1
Potra2n1c918.1	NA	NA	NA	NA		
Potra2n1c919.1	0.2651136	-0.199864195	0.536373609	0.936549944	Potri.001G109000.10	AT4G11720.1
Potra2n1c92.1	0.189548946	0.10022534	0.129822585	0.932984974	Potri.001G008700.2	AT3G32940.1
Potra2n1c920.1	1.761059743	-1.050206259	0.374264015	NA	Potri.001G109100.1	AT1G12740.1
Potra2n1c921.1	0.278566554	0.28909732	0.009073163	0.566942081	Potri.001G109200.1	AT1G63110.1
Potra2n1c922.1	0.485500168	3.368845378	0.753922519	0.29966352	Potri.001G109300.1	AT1G33720.1
Potra2n1c923.1	-0.155992176	-0.652847974	0.834132228	0.816567218	Potri.003G122400.1	AT1G63100.1
Potra2n1c924.1	0.143361441	0.005430569	0.1144736	0.996032145	Potri.001G109500.1	AT4G11740.1
Potra2n1c925.1	0.01723116	-0.018506363	0.952409141	0.991036771	Potri.001G109600.1	AT4G26965.1
Potra2n1c926.1	-0.770435067	-0.258380581	0.058179095	0.912777843	Potri.001G109700.1	AT5G64620.1
Potra2n1c927.1						
Potra2n1c928.1	-0.861007643	-0.423652543	1.80702E-07	0.227304165	Potri.001G109800.3	AT1G12710.1
Potra2n1c929.1	-1.104185938	0.490070367	0.769758043	NA	Potri.001G109901.1	AT4G25520.1
Potra2n1c93.1	0.061329007	0.084515568	0.566676185	0.913459614	Potri.001G008800.3	AT1G55250.3
Potra2n1c930.1	0.13309908	0.087710818	0.264600043	0.892756772	Potri.001G110000.1	AT1G12650.1
Potra2n1c931.1	-0.696952282	-0.221420856	3.51781E-06	0.716548554	Potri.001G110100.1	AT1G12640.1
Potra2n1c932.1	-0.479650036	-0.325664625	3.70206E-08	0.475175307	Potri.001G110200.1	AT4G20360.1
Potra2n1c933.1	1.056769652	0.897912335	NA	NA	Potri.001G110400.1	AT1G11260.1
Potra2n1c934.1	3.37510751	-1.55593228	0.181878487	NA	Potri.001G110500.1	AT5G52020.1
Potra2n1c935.1	5.617996028	1.833798897	NA	NA	Potri.001G110700.1	AT5G51990.1
Potra2n1c936.1	6.441875031	2.611445458	NA	NA	Potri.001G110700.1	AT5G51990.1
Potra2n1c937.1	3.753133876	-0.304505358	NA	NA	Potri.001G110800.1	AT1G12610.1

Potra2n1c938.1	-0.61910233	-0.767708831	NA	NA		
Potra2n1c939.1	-1.663838342	-0.404940198	0.058681423	0.915358268	Potri.001G110900.1	AT4G23020.2
Potra2n1c94.1	2.790522217	1.898061817	9.87213E-06	0.354287698	Potri.001G008900.1	AT1G55230.1
Potra2n1c940.1	1.24694055	0.260377703	2.31271E-07	0.68291168	Potri.001G111000.2	AT4G23010.3
Potra2n1c941.1	0.322695555	0.175454122	0.001149164	0.659126444	Potri.001G111100.1	AT4G11790.1
Potra2n1c942.3	0.360358785	0.403344129	0.044436182	0.62849354	Potri.001G111300.1	AT1G63020.2
Potra2n1c943.1	0.420716266	0.160446223	0.002219933	0.836066102	Potri.001G111400.1	AT4G22990.1
Potra2n1c944.1	3.065778315	NA	NA	NA	Potri.001G111500.1	AT1G12570.1
Potra2n1c945.1	2.923441662	1.142427783	4.31506E-11	0.477196168		
Potra2n1c946.1	2.9731661	1.182350881	7.11598E-12	0.427640327	Potri.001G111600.1	AT5G51920.1
Potra2n1c947.1	0.670508339	1.131446746	6.45911E-05	2.08394E-06	Potri.001G111700.1	AT4G11820.2
Potra2n1c948.1	0.331251836	-0.120094098	0.244058769	0.961695543	Potri.001G111800.1	AT5G51910.2
Potra2n1c949.1	-1.317292482	-1.465929376	NA	NA	Potri.001G111900.1	AT5G59090.1
Potra2n1c95.1	NA	NA	NA	NA	Potri.001G009000.1	AT1G55230.1
Potra2n1c950.1	NA	NA	NA	NA	Potri.001G111900.1	AT5G59090.1
Potra2n1c951.1	-0.079881616	-0.247812447	0.649469817	0.814001428	Potri.001G112000.1	AT1G63000.1
Potra2n1c952.1	2.121419853	0.535511918	1.38445E-31	0.379734246	Potri.014G074700.1	AT2G42010.1
Potra2n1c953.1	-0.52973901	0.021584438	0.012379643	0.993485167	Potri.001G112200.1	AT1G62990.1
Potra2n1c954.1	0.030663877	0.128614071	0.790597083	0.70570456	Potri.001G112300.1	AT4G11860.1
Potra2n1c955.3	0.000251742	0.159519833	0.999451097	0.915358268	Potri.001G112400.1	AT4G22950.1
Potra2n1c956.1	-0.298228723	-0.050166033	0.707312337	0.993589787	Potri.001G112500.1	AT4G22920.1
Potra2n1c957.1	-0.780941834	-0.209073021	0.009358967	0.87679119	Potri.001G112600.1	AT4G22920.1
Potra2n1c958.1	-0.550391437	-0.172507238	0.005764614	0.856337524	Potri.001G112700.1	AT4G22910.1
Potra2n1c959.1	-1.280913422	-0.557673225	0.000256052	0.744763949	Potri.001G112800.1	AT4G22900.1
Potra2n1c96.1	-0.503197983	-0.474728567	0.077037628	0.758676613	Potri.001G009100.1	AT1G58170.1
Potra2n1c960.1	-2.67571617	-2.836900478	0.272357034	NA	Potri.008G057100.1	AT2G39700.1
Potra2n1c961.1	-1.466188721	-0.725556398	0.038465322	0.70570456	Potri.001G113000.3	AT4G22890.2
Potra2n1c962.1	6.545557402	2.577493599	4.89619E-22	0.116967248	Potri.001G113100.1	AT4G22880.1
Potra2n1c963.1	0.992506765	0.314417829	1.25071E-22	0.674175338	Potri.001G113250.1	AT2G45630.2
Potra2n1c964.1	NA	NA	NA	NA	Potri.001G113400.1	AT5G51790.1
Potra2n1c965.1	-0.261251894	0.095421633	0.019459947	0.881171061	Potri.001G113500.1	AT4G11970.2
Potra2n1c966.1	0.039413296	-0.213997349	0.923779017	0.910815461	Potri.003G118700.1	AT1G04110.1

Potra2n1c967.1	-0.310287424	0.04707244	0.011931338	0.975578749	Potri.001G113600.1	AT3G58630.1
Potra2n1c968.1	NA	NA	NA	NA	Potri.001G113700.1	AT1G04110.1
Potra2n1c969.1	0.045267015	-0.226195349	0.910537729	0.907073354	Potri.001G113800.1	AT1G12520.1
Potra2n1c97.1	-1.615768078	-0.551480762	NA	NA	Potri.001G009200.1	AT5G19875.1
Potra2n1c970.1	0.307608637	0.488701979	0.517595859	0.915358268	Potri.001G113900.1	AT2G45550.1
Potra2n1c971.1	-0.001545106	0.110081123	NA	NA	Potri.001G114000.1	AT4G37740.1
Potra2n1c972.1	-1.083641909	-0.248445472	6.92362E-05	0.903496619	Potri.001G114100.1	AT1G12500.1
Potra2n1c973.1	NA	1.363811008	NA	NA	Potri.013G030000.1	AT2G32300.1
Potra2n1c974.4	-0.487898025	-0.166107106	0.093739745	0.896937427	Potri.001G114400.2	AT4G22860.1
Potra2n1c975.1	-0.31239788	-0.030086627	0.221797313	0.987288338	Potri.001G114500.1	AT1G12460.1
Potra2n1c976.1	NA	1.363811008	NA	NA		
Potra2n1c977.1	0.050811908	-0.113868336	0.876811813	0.908757873	Potri.001G114600.1	AT1G12450.1
Potra2n1c978.1	-2.058731619	-1.702389371	0.322853042	NA	Potri.003G117300.1	AT4G22840.1
Potra2n1c979.1	-1.078280105	-0.348424433	3.58401E-06	0.479233295	Potri.001G114800.4	AT4G22830.1
Potra2n1c98.1	0.82357019	1.381953902	NA	NA	Potri.001G009300.1	AT5G19875.1
Potra2n1c980.1	-1.092637084	-0.353153887	5.31774E-06	0.466145447	Potri.001G114900.1	AT3G60890.2
Potra2n1c981.1	-0.436309178	-0.122132179	0.01168778	0.889258723	Potri.001G115000.1	AT4G12040.2
Potra2n1c982.1	0.158590518	0.158858727	0.354266191	0.885375133	Potri.003G117000.2	AT1G12430.1
Potra2n1c983.1	NA	NA	NA	NA	Potri.001G115200.1	AT4G22810.1
Potra2n1c984.1	1.70339271	0.997415246	0.646496724	NA	Potri.001G115300.1	
Potra2n1c985.1	-0.402841489	-0.157499028	0.000247614	0.803948068	Potri.001G115500.1	AT4G12060.1
Potra2n1c986.1	-2.424149374	-0.592683993	1.5851E-11	0.779330106	Potri.001G115400.1	AT4G22790.1
Potra2n1c987.1	-0.856155125	-0.047105047	3.24707E-06	0.983046384	Potri.001G115600.1	AT1G12420.1
Potra2n1c988.1	-1.369878352	-0.409408263	0.031299413	0.88661378		
Potra2n1c989.1	-0.07791112	0.102901763	0.566488304	0.88643982	Potri.001G115800.1	AT4G12080.1
Potra2n1c99.1	1.64774763	NA	NA	NA	Potri.003G215900.1	AT5G07610.1
Potra2n1c990.1	0.190454353	0.272528718	0.819330054	0.936890993		
Potra2n1c991.1	0.36585228	0.241723423	0.204895761	0.835819291		
Potra2n1c992.1	0.345191336	0.312542276	0.066567208	0.593018714		
Potra2n1c993.1	-0.117090547	-0.03314933	0.353085798	0.974782554	Potri.001G115900.2	AT1G12410.1
Potra2n1c994.1	0.200726574	0.229750865	0.282406299	0.723658895	Potri.001G116000.1	AT1G12400.1
Potra2n1c995.1	0.032036691	0.10300183	0.829598862	0.887678043	Potri.001G116100.1	AT1G12390.1

Potra2n1c996.1	-0.375627702	0.688605434	0.848972997	0.917746944	Potri.001G116200.1	AT4G22758.1
Potra2n1c997.1	-0.559870835	-1.282403538	0.744516193	NA	Potri.001G116300.1	AT4G22760.1
Potra2n1c998.1	-0.510584385	-0.252808413	0.005864616	0.855587395	Potri.001G116500.1	AT4G12110.1
Potra2n1c999.1	-0.697992013	-0.219639598	3.47566E-06	0.726986309		
Potra2n200s34921.1	-0.11806968	0.126276678	0.842022417	0.971537796		
Potra2n200s34922.1	-0.041719871	-0.198523946	0.833935275	0.753615154	Potri.019G120700.2	AT1G35710.1
Potra2n200s34923.1	-0.088025121	-0.177981716	0.644635572	0.833401866	Potri.019G059500.1	AT2G40765.1
Potra2n200s34924.1	0.0988566	-0.110116028	0.478048019	0.893071612	Potri.019G059400.1	AT2G40760.1
Potra2n200s34925.1	3.131069512	2.283425176	NA	NA	Potri.019G059300.1	AT2G40740.1
Potra2n202s34926.3	0.600006904	0.280878021	0.028738514	0.773596862	Potri.001G053500.1	AT3G24050.1
Potra2n202s34927.1	-0.386456631	-0.149329524	0.001375582	0.817452127	Potri.001G053400.1	AT2G33170.1
Potra2n202s34928.1	NA	NA	NA	NA	Potri.001G053300.1	AT3G24060.1
Potra2n203s34929.1	2.063471427	NA	NA	NA	Potri.007G037200.1	AT2G23620.1
Potra2n203s34930.1	NA	NA	NA	NA	Potri.007G037200.1	AT2G23620.1
Potra2n203s34931.1	NA	NA	NA	NA	Potri.007G037000.1	AT2G23620.1
Potra2n203s34932.1	NA	NA	NA	NA		
Potra2n203s34933.1	4.183061823	0.963725757	0.000307575	NA	Potri.007G037700.1	AT2G23620.1
Potra2n203s34934.1	2.476290371	NA	NA	NA	Potri.007G037200.1	AT2G23620.1
Potra2n203s34935.1	0.677127226	-0.891748855	0.53862701	0.873889603	Potri.007G037700.1	AT2G23620.1
Potra2n203s34936.1	-0.060781619	0.048145228	0.596762987	0.945254452	Potri.007G037800.1	AT5G66930.2
Potra2n203s34937.1	-2.893310937	-1.615704867	NA	NA	Potri.007G038000.1	
Potra2n203s34938.1	0.95578761	-0.260263985	0.022841269	0.918722297	Potri.007G038100.1	AT5G65590.1
Potra2n203s34939.1	0.245170784	-0.744589582	NA	NA	Potri.007G038200.1	AT4G37160.1
Potra2n203s34940.1	-1.376962975	-0.400277947	2.69684E-07	0.828073327	Potri.007G038300.2	AT5G66920.1
Potra2n203s34941.1	-1.382326512	-0.416157246	4.63697E-08	0.802631112		
Potra2n203s34942.2	-1.497697097	-0.372910241	5.23779E-08	0.855587395	Potri.007G038300.2	AT5G66920.1
Potra2n203s34943.1	NA	0.674963689	NA	NA	Potri.007G038400.1	
Potra2n203s34944.1	1.35506908	0.989424552	4.79674E-15	0.017817938	Potri.007G038500.1	AT2G23640.1
Potra2n203s34945.1	NA	NA	NA	NA	Potri.007G038600.1	AT3G50470.1
Potra2n203s34946.1	NA	NA	NA	NA	Potri.007G038701.1	AT5G66900.1
Potra2n203s34947.1	-3.1732318	-0.812333635	0.054300495	0.88643982	Potri.007G039000.1	AT5G66900.1
Potra2n203s34948.1	NA	1.363811008	NA	NA	Potri.013G089700.1	

Potra2n203s34949.1	6.932459665	3.938306536	6.5036E-08	NA	Potri.007G039100.1	AT5G66900.1
Potra2n203s34950.2	4.038271059	2.266827928	2.61639E-42	0.11440223	Potri.007G039100.1	AT5G66900.1
Potra2n203s34951.1	3.745948676	1.862658583	7.55657E-50	0.043212438	Potri.007G039100.1	AT5G66900.1
Potra2n203s34952.1	7.999589087	3.937220576	4.35393E-20	0.343290193	Potri.007G039300.1	AT5G66900.1
Potra2n203s34953.1	-1.35927387	1.555436866	NA	NA	Potri.005G134400.1	AT5G66880.1
Potra2n203s34954.1	0.92641307	1.576243557	NA	NA	Potri.005G134500.1	AT3G19080.1
Potra2n203s34955.1	0.92641307	0.64247635	NA	NA		
Potra2n203s34956.1	0.906698059	0.599592521	0.100199985	0.843443324	Potri.007G039400.1	AT4G37180.1
Potra2n203s34957.3	-0.741230933	-0.416782737	0.000887598	0.741026208	Potri.007G039500.1	AT5G66870.1
Potra2n203s34958.1	-0.055884665	-0.323916971	0.839765464	0.817452127	Potri.007G039700.1	AT5G66860.1
Potra2n203s34959.1	0.114797905	0.056830471	0.36730591	0.970817579	Potri.007G039800.1	AT5G66850.1
Potra2n203s34960.1	-0.228897511	0.74028447	0.939278415	NA		
Potra2n203s34961.1	0.383306082	0.174201354	0.009297095	0.874808359	Potri.007G040000.1	AT4G28200.1
Potra2n203s34962.1	0.550531764	-0.30869913	0.001665495	0.720652439	Potri.005G135500.1	AT2G15790.1
Potra2n206s34963.1	2.340207562	NA	NA	NA	Potri.011G017400.2	AT3G18670.1
Potra2n206s34964.1	NA	NA	NA	NA	Potri.011G020250.1	AT3G18670.1
Potra2n207s34965.1	3.49234276	2.90365882	0.000771227	0.362601699		
Potra2n207s34966.1	0.105434794	-0.13633343	0.479717671	0.863705586	Potri.001G194400.1	AT1G17730.1
Potra2n210s34967.1					Potri.007G062400.1	
Potra2n210s34968.1	0.027658617	0.062574555	0.829720025	0.951394085	Potri.002G207200.2	AT2G47940.1
Potra2n210s34969.1	0.481240612	0.000380596	0.027530791	0.999937671	Potri.002G207400.2	AT1G48910.1
Potra2n210s34970.1	-1.928216252	-0.577871339	0.000108503	0.776094736	Potri.002G207500.1	AT2G47930.1
Potra2n210s34971.1					Potri.017G072700.1	AT5G05800.1
Potra2n210s34972.1	1.761132086	-0.2568593	0.020819214	0.953831244	Potri.002G207700.1	AT2G47920.1
Potra2n210s34973.1					Potri.003G125600.1	AT1G63440.1
Potra2n211s34974.1	NA	NA	NA	NA	Potri.018G010700.1	AT2G29110.1
Potra2n211s34975.1	0.996150907	0.651314553	0.055941732	0.773574963		
Potra2n211s34976.1	4.905312579	1.849701723	0.000494369	NA	Potri.018G010700.1	AT2G29110.1
Potra2n211s34977.1	4.223171243	NA	0.056537004	NA	Potri.018G010700.1	AT2G29110.1
Potra2n211s34978.2	0.216565131	0.139386737	NA	NA		
Potra2n211s34979.1	1.64774763	NA	NA	NA	Potri.018G012100.1	AT2G29120.1
Potra2n211s34980.1	3.382762599	1.381953902	NA	NA	Potri.018G010700.1	AT2G29110.1

Potra2n211s34981.1	2.577017714	1.339446293	0.304029015	NA	Potri.018G010700.1	AT2G29110.1
Potra2n211s34982.1	NA	NA	NA	NA	Potri.T038300.2	AT5G36930.2
Potra2n211s34983.1	-1.068059507	-1.309954012	0.723815711	NA	Potri.018G010700.1	AT2G29110.1
Potra2n211s34984.1	-2.911779158	-1.472381608	NA	NA		
Potra2n212s34985.1	NA	NA	NA	NA	Potri.013G037400.1	
Potra2n212s34986.1						
Potra2n212s34987.1	8.446203732	4.819729399	3.85089E-12	NA		
Potra2n215s34988.1					Potri.T092600.3	AT4G27190.1
Potra2n215s34989.1	-0.819559175	0.038120654	0.72215261	0.99736971	Potri.013G100900.1	AT2G01050.1
Potra2n216s34990.1	NA	NA	NA	NA	Potri.017G025700.1	AT5G44250.1
Potra2n216s34991.1						
Potra2n219s34992.1					Potri.019G066700.1	AT3G07820.1
Potra2n219s34993.1					Potri.019G067200.1	AT3G07820.1
Potra2n220s34994.1	0.18837245	-0.328174488	0.785198487	0.924951347	Potri.004G101500.1	AT3G02530.1
Potra2n220s34995.1	-1.317292482	-1.465929376	NA	NA	Potri.004G101600.1	
Potra2n220s34996.1	0.034451617	-0.421731052	0.96028878	0.835819291	Potri.004G101500.1	AT3G02530.1
Potra2n220s34997.1	-0.679091443	-0.3522709	0.179567224	0.874808359	Potri.004G101500.1	AT3G02530.1
Potra2n220s34998.1	-0.798654507	-0.481422287	0.097009309	0.82876584	Potri.015G044600.1	AT1G18370.1
Potra2n221s34999.1	NA	NA	NA	NA		
Potra2n221s35000.1	-1.328951947	-1.478536539	NA	NA		
Potra2n221s35001.1	1.077609047	1.173696693	0.4648546	NA	Potri.017G152600.2	AT1G03610.1
Potra2n221s35002.1	0.184789554	0.421737331	0.945572916	NA		
Potra2n222s35003.1	-0.643083462	-0.710830104	0.742050423	NA	Potri.006G132000.1	AT5G02880.1
Potra2n222s35004.1	-1.328951947	1.072538092	NA	NA		
Potra2n222s35005.1	NA	1.394613675	NA	NA	Potri.004G135500.1	AT1G07650.1
Potra2n222s35006.1	NA	NA	NA	NA	Potri.T015000.1	
Potra2n222s35007.1						
Potra2n222s35008.1	-0.476538647	-1.012567065	0.801539088	NA	Potri.018G136100.1	AT3G14470.1
Potra2n222s35009.1	0.40869553	1.354058062	0.877460839	NA	Potri.018G136100.1	AT3G14470.1
Potra2n222s35010.1	0.733322468	0.438996721	0.033667344	0.813575895	Potri.T045000.1	AT4G10780.1
Potra2n222s35011.1	-0.333582784	0.432611849	0.662558472	0.862830524		
Potra2n222s35012.1	-1.941514238	-0.916560915	0.590249369	NA	Potri.004G135300.1	AT4G15093.1

Potra2n222s35013.1	0.871938416	0.223913449	0.001901876	0.913622521	Potri.T013800.1	AT4G27220.1
Potra2n222s35014.1					Potri.T013950.1	AT4G36760.1
Potra2n225s35015.1	1.740349964	0.325502414	1.62819E-09	0.855754617	Potri.T042700.1	AT1G01630.1
Potra2n225s35016.1					Potri.001G155750.1	AT1G21280.1
Potra2n227s35017.1	-0.408338867	-0.068505429	0.005458537	0.936890993	Potri.001G018600.1	AT1G51200.3
Potra2n227s35018.1	0.745150569	0.378442452	2.63907E-06	0.52891249	Potri.001G018500.1	AT5G13070.1
Potra2n227s35019.1	NA	NA	NA	NA	Potri.001G018400.1	AT3G20840.1
Potra2n227s35020.1	-1.311347697	-0.138053958	6.49962E-10	0.880316339	Potri.001G018300.1	AT3G20830.1
Potra2n227s35021.1	-1.016062618	-0.428115169	1.22127E-07	0.314259515	Potri.001G018200.1	AT5G12950.1
Potra2n227s35022.1	-0.104281797	0.203943802	0.81705237	0.93017274	Potri.001G018100.1	AT2G39725.2
Potra2n227s35023.1						
Potra2n228s35024.1	-0.113470741	-0.033689991	0.372399505	0.978203092	Potri.018G003700.10	AT4G31340.1
Potra2n228s35025.1	5.777595733	1.394613675	0.000174305	NA	Potri.018G003800.1	AT2G24430.1
Potra2n228s35026.1	0.247087642	0.158677876	0.332722634	0.902676973	Potri.018G003900.1	AT5G24840.1
Potra2n229s35027.1	NA	0.674963689	NA	NA	Potri.006G270000.1	AT5G36930.2
Potra2n229s35028.1	0.532470333	-0.134343738	0.146984016	0.94608223	Potri.011G015400.3	AT1G27170.1
Potra2n229s35029.1	0.942747724	0.189042847	3.36612E-05	0.91820026	Potri.011G012000.1	AT5G36930.2
Potra2n229s35030.1	2.479697454	2.088818767	0.116402755	NA	Potri.T067800.1	AT5G36930.2
Potra2n229s35031.1	-1.34256587	-0.04371145	0.573514431	NA	Potri.T068200.1	
Potra2n229s35032.1	0.093267355	-0.982082032	0.949733145	0.826134955	Potri.011G012300.1	AT5G13950.3
Potra2n229s35033.1					Potri.012G117100.1	AT5G45160.1
Potra2n230s35034.1	0.242826737	-0.151956216	0.578633684	0.945742637	Potri.T051300.1	AT3G56070.1
Potra2n230s35035.2	-1.400006915	-0.196868057	0.408086486	0.986236808	Potri.T051200.3	AT4G37070.2
Potra2n232s35036.1	2.315713269	NA	NA	NA	Potri.T032966.1	AT1G29730.1
Potra2n232s35037.1	-1.409983009	-1.55593228	NA	NA	Potri.011G072732.1	AT1G29730.1
Potra2n232s35038.2	3.061176985	NA	NA	NA	Potri.T032966.1	AT1G29730.1
Potra2n233s35039.1	0.167408791	0.405045772	0.913662884	0.950878725		
Potra2n233s35040.1	5.284739215	-2.071795494	1.03597E-06	NA		
Potra2n233s35041.1	-0.577530148	0.086612347	0.003506117	0.956865545	Potri.017G110700.1	AT5G15860.1
Potra2n233s35042.1	4.870800075	-2.049121849	5.61089E-10	NA	Potri.017G110800.1	
Potra2n233s35043.1	-0.734412173	0.278106022	0.004346685	0.808901406	Potri.017G110700.1	AT5G15860.1
Potra2n234s35044.1	2.685015931	3.87354687	NA	NA	Potri.012G095100.1	AT1G29320.1

Potra2n234s35045.1	0.054397297	0.27147243	0.788899691	0.830056077	Potri.012G095000.1	AT5G63380.1
Potra2n234s35046.1	0.690657563	0.055243768	0.000669015	0.953831244	Potri.012G094800.1	AT5G63380.1
Potra2n234s35047.1	0.622110021	0.169942551	0.008387892	0.917746944	Potri.012G094700.1	AT1G67570.1
Potra2n234s35048.1	0.494003235	0.22296742	0.005580168	0.571096094	Potri.012G094600.1	AT1G67580.2
Potra2n234s35049.1	0.015082258	-0.258413623	0.983593635	0.939915707	Potri.015G092000.1	AT5G03860.1
Potra2n234s35050.1	0.115481008	-0.290333624	0.899299032	0.947547562		
Potra2n234s35051.1	NA	1.892950973	NA	NA	Potri.012G094400.1	AT5G50360.1
Potra2n234s35052.2	-0.136141963	0.092900047	0.384542256	0.945254452	Potri.012G094300.1	AT5G63320.1
Potra2n234s35053.1	0.235618268	0.076229189	0.09957117	0.953608209	Potri.012G094200.1	AT5G50350.1
Potra2n234s35054.1	0.372797425	-0.014117473	0.017526335	0.991407187	Potri.012G094100.1	AT1G13440.1
Potra2n234s35055.1	2.650430111	0.923064381	0.076212755	NA	Potri.012G094000.1	AT5G50335.1
Potra2n234s35056.1					Potri.012G093900.1	AT1G66350.1
Potra2n234s35057.1	0.208731828	-0.081902094	0.294078407	0.960981446	Potri.012G093800.1	AT5G63310.1
Potra2n234s35058.2	-1.174211116	-0.251390852	1.43948E-06	0.70570456	Potri.012G093600.1	AT4G24810.2
Potra2n234s35059.1	0.067090132	0.050157143	0.692744032	0.976172519	Potri.015G090900.1	AT4G24820.2
Potra2n234s35060.1	1.815358055	-1.55593228	0.701189843	NA	Potri.012G093300.1	AT1G24440.1
Potra2n234s35061.1	-0.162594901	-0.218781296	0.556259814	0.822998494	Potri.012G093200.1	AT3G18960.1
Potra2n234s35062.2	0.1633833	-0.093146286	0.555402114	0.89370478	Potri.012G093000.2	AT5G62640.1
Potra2n234s35063.1	0.143382964	-0.217702115	0.635949207	0.849446235	Potri.012G092900.1	AT5G63290.1
Potra2n234s35064.1	0.000957512	-0.089102372	0.995303032	0.901251375	Potri.015G090500.1	AT5G50320.1
Potra2n234s35065.1	0.289368571	0.15929526	0.018307423	0.829433032	Potri.012G092500.2	AT3G48440.1
Potra2n234s35066.1	-0.027721828	0.280491137	0.909353922	0.857189843	Potri.012G092400.1	AT3G48430.1
Potra2n235s35067.1	1.456238393	0.233326578	0.007891735	0.921399056	Potri.010G240800.1	AT5G04760.1
Potra2n235s35068.1	1.64774763	NA	NA	NA	Potri.010G240700.1	AT1G02335.1
Potra2n235s35069.1	1.64774763	NA	NA	NA	Potri.010G240600.1	AT3G62020.2
Potra2n235s35070.1	-0.344086535	0.216687641	0.187692734	0.874808359	Potri.010G240401.1	AT5G04750.1
Potra2n235s35071.1	0.808913022	0.897912335	NA	NA	Potri.010G240500.1	AT3G04200.1
Potra2n235s35072.1	-0.064370824	0.125998915	0.696093942	0.909675463	Potri.010G240300.1	AT3G52220.1
Potra2n235s35073.1	0.989904959	-0.036428675	8.00572E-07	0.985393296	Potri.010G240200.1	AT3G19330.1
Potra2n235s35074.1	-0.087917996	-0.175200798	0.713220061	0.889258723	Potri.010G240000.2	AT2G38710.2
Potra2n235s35075.4	0.156120749	0.041368994	0.258702515	0.963557834	Potri.010G239900.1	AT3G54300.2
Potra2n238s35076.1	1.484821983	0.089475393	0.396344846	NA		

Potra2n241s35077.1	-0.400754946	-0.06536804	0.089501438	0.974756511	Potri.004G193000.2	AT5G20930.1
Potra2n241s35078.1	-0.2451255	0.274781375	0.439089673	0.883647605	Potri.009G155200.3	AT2G27170.1
Potra2n241s35079.1	-0.091815106	0.154574761	0.637275271	0.924251575		
Potra2n241s35080.1	-0.141476639	-0.028465382	0.46368212	0.982827151	Potri.011G130000.1	AT5G54540.1
Potra2n241s35081.1	-0.417088722	-0.221133324	0.062202199	0.859892315	Potri.009G155200.3	AT2G27170.1
Potra2n243s35082.1	0.081381604	0.039406017	0.666612307	0.978203092	Potri.016G127200.1	AT4G38495.1
Potra2n243s35083.1	0.720221959	0.603828337	0.158646385	0.752252096	Potri.T092400.1	AT3G51660.1
Potra2n243s35084.1	NA	1.571529889	NA	NA	Potri.T092300.1	AT1G47890.1
Potra2n243s35085.1	0.82357019	0.674963689	NA	NA	Potri.T092200.1	AT1G47890.1
Potra2n243s35086.1	NA	NA	NA	NA	Potri.T092300.1	AT1G47890.1
Potra2n243s35087.1	0.82357019	0.674963689	NA	NA	Potri.T092200.1	AT1G47890.1
Potra2n243s35088.1	-0.429000881	-0.188462227	0.772282076	0.978646026	Potri.016G127200.1	AT4G38495.1
Potra2n243s35089.1	-0.846358543	-1.115710276	0.744911287	NA	Potri.T091900.1	AT3G05660.1
Potra2n243s35090.1	0.562698574	-2.523469902	0.858771724	NA	Potri.T092000.1	AT3G05660.1
Potra2n243s35091.1	0.82357019	NA	NA	NA	Potri.T092200.1	AT1G47890.1
Potra2n245s35092.1	1.992520214	0.849750035	0.000160749	0.642417298	Potri.005G008901.1	AT1G58190.2
Potra2n245s35093.1	NA	NA	NA	NA		
Potra2n245s35094.1	NA	NA	NA	NA	Potri.003G010800.1	
Potra2n245s35095.1	0.087182329	0.200539578	0.491929626	0.704663803	Potri.003G029600.1	AT1G15780.1
Potra2n245s35096.1	1.811995413	1.642139187	0.375519514	0.828625141		
Potra2n245s35097.1	4.021458735	1.489542157	6.4503E-14	0.468208385	Potri.003G013600.1	
Potra2n245s35098.1	1.625549312	0.909989926	0.001010032	0.663037599	Potri.003G027300.1	AT1G15780.1
Potra2n245s35099.1	1.545832463	NA	NA	NA	Potri.003G013400.1	
Potra2n245s35100.1	-0.886998084	-1.702723216	0.607969324	0.817407708	Potri.003G026900.1	AT3G14460.1
Potra2n245s35101.1	0.274380139	1.033895241	0.896155321	NA	Potri.003G026900.1	AT3G14460.1
Potra2n245s35102.1	0.2584121	-0.105012571	0.864942132	0.9869326	Potri.003G026900.1	AT3G14460.1
Potra2n245s35103.1	-0.056749171	0.132941492	0.728503884	0.821709751	Potri.003G027400.10	AT1G15780.1
Potra2n245s35104.1	-0.05673888	0.814394159	0.935252261	0.727843918	Potri.009G102900.1	AT3G19190.1
Potra2n245s35105.1	0.419898506	1.331353731	0.781117616	0.834560473	Potri.009G098966.1	AT4G34131.1
Potra2n245s35106.1	NA	1.394613675	NA	NA	Potri.001G214100.1	AT4G19130.1
Potra2n245s35107.1	NA	1.887510518	NA	NA	Potri.003G027300.1	AT1G15780.1
Potra2n246s35108.1	4.351035205	NA	0.210191109	NA	Potri.007G037200.1	AT2G23620.1

Potra2n246s35109.1	-0.357548418	0.762665525	0.676991319	0.64562236	Potri.002G261000.1	AT1G75170.1
Potra2n246s35110.1	0.238415533	0.381580627	0.363719904	0.805461106	Potri.007G037700.1	AT2G23620.1
Potra2n246s35111.1	-4.482972683	-1.123278118	0.045984185	NA	Potri.007G016400.1	AT4G36220.1
Potra2n246s35112.1	-0.379637484	0.694748918	0.624792472	0.895474078	Potri.007G037200.1	AT2G23620.1
Potra2n246s35113.1	3.636265227	1.911450358	0.41570728	NA	Potri.007G037200.1	AT2G23620.1
Potra2n246s35114.1	3.219950245	0.674963689	NA	NA	Potri.007G037200.1	AT2G23620.1
Potra2n249s35115.1	-0.61910233	-0.046370319	NA	NA		
Potra2n250s35116.1	-0.474058799	-0.223067426	0.766677609	0.97950444		
Potra2n250s35117.2	1.275332741	0.576961081	9.13202E-16	0.027226177	Potri.017G018800.1	AT4G15880.1
Potra2n250s35118.1	0.976890905	0.627051567	1.45333E-06	0.010990669	Potri.017G020600.1	AT2G43500.2
Potra2n250s35119.1	-2.61033313	-1.320258884	NA	NA	Potri.017G022532.1	AT2G43500.1
Potra2n251s35120.1						
Potra2n251s35121.1	NA	NA	NA	NA	Potri.T098200.1	AT4G21390.1
Potra2n251s35122.1	5.684805504	-0.828335558	2.65267E-09	NA	Potri.001G442200.1	AT4G21390.1
Potra2n251s35123.1	2.166817139	-3.043858706	0.123067005	NA	Potri.001G442300.1	AT5G44550.1
Potra2n251s35124.1	-0.279272259	-0.073188985	0.358924973	0.971828097	Potri.001G442400.1	AT4G20380.7
Potra2n252s35125.1	-3.554279518	-0.319092125	0.012016161	0.967129771	Potri.006G114200.1	AT3G54220.1
Potra2n252s35126.2	-0.116298197	0.078369009	0.428855286	0.931214679	Potri.006G114300.4	AT2G38650.1
Potra2n253s35127.1	1.761251355	0.891605925	0.630042446	NA		
Potra2n254s35128.1	2.066865005	1.011968904	1.41181E-11	0.095938059	Potri.T047500.2	AT5G17680.1
Potra2n254s35129.1	1.150480364	0.872367223	1.75012E-06	0.000343925	Potri.T048266.1	AT1G69550.1
Potra2n255s35130.1	-0.037913129	0.096331925	0.801757301	0.936509845	Potri.012G064200.1	AT5G15740.1
Potra2n255s35131.1	0.285382223	0.158088643	0.104512588	0.870293488	Potri.016G095750.1	AT5G03730.1
Potra2n256s35132.1					Potri.002G231200.2	AT4G35830.1
Potra2n256s35133.1					Potri.014G151200.1	AT3G59280.1
Potra2n256s35134.1	0.19107579	-0.187198529	0.565023009	0.911339141	Potri.018G078400.1	AT3G22440.1
Potra2n257s35135.1	0.472951079	0.184077589	0.013129065	0.924828248	Potri.T085300.1	AT1G78590.1
Potra2n257s35136.1					Potri.011G104500.1	AT1G53490.1
Potra2n257s35137.1	8.435703544	6.551436085	1.86227E-11	0.004329954	Potri.T085500.2	AT3G11010.1
Potra2n257s35138.1						
Potra2n258s35139.1					Potri.002G110500.1	AT1G70320.1
Potra2n258s35140.1	1.664677056	NA	NA	NA	Potri.003G024400.1	AT2G35040.1

Potra2n258s35141.3	1.626967754	0.173836997	3.21935E-12	0.954098985	Potri.003G024200.2	AT1G60500.1
Potra2n262s35142.1	0.114648624	0.139386737	NA	NA	Potri.T092300.1	AT1G47890.1
Potra2n262s35143.1	0.216565131	2.341978469	NA	NA	Potri.T092300.1	AT1G47890.1
Potra2n262s35144.1	NA	NA	NA	NA	Potri.T091900.1	AT3G05660.1
Potra2n262s35145.1	1.64774763	NA	NA	NA	Potri.T092000.1	AT3G05660.1
Potra2n264s35146.2	-0.381969519	-0.102017876	0.021018239	0.948577865	Potri.011G111700.1	AT1G53290.1
Potra2n264s35147.2	-0.226325395	-0.292620622	0.168071866	0.809914834	Potri.011G111500.1	AT1G53300.1
Potra2n266s35148.1	1.459962178	NA	NA	NA	Potri.006G170800.1	AT5G57620.1
Potra2n266s35149.1	1.875925012	0.522261909	9.79237E-27	0.687729258	Potri.006G170900.1	AT5G52450.1
Potra2n266s35150.1	1.152586145	-0.003966708	0.00405217	0.999325699	Potri.006G171100.1	AT5G17050.1
Potra2n266s35151.1						
Potra2n266s35152.1	-2.295337707	1.005351428	NA	NA	Potri.006G171300.1	AT4G30320.1
Potra2n266s35153.2	2.323846567	0.752434306	5.30198E-12	0.70570456	Potri.006G171400.6	AT5G57630.1
Potra2n266s35154.1					Potri.T094100.1	AT4G11650.1
Potra2n266s35155.1	-0.184489017	-0.076105596	0.291276746	0.970817579	Potri.T094300.1	AT2G24765.1
Potra2n266s35156.1	3.266247063	0.318681587	3.76E-14	0.937276488	Potri.006G171700.1	AT4G15560.1
Potra2n266s35157.1						
Potra2n266s35158.1	0.06072378	0.458786634	0.947527277	0.875559093	Potri.006G173600.1	AT5G57660.1
Potra2n267s35159.1						
Potra2n267s35160.1	-0.034712282	0.013348349	0.804972576	0.992934677	Potri.008G209100.2	AT4G15780.1
Potra2n267s35161.1	-0.309800243	-0.085632456	0.094146268	0.956631493	Potri.008G208000.1	AT4G02500.1
Potra2n267s35162.1	-1.173664772	-0.475108812	1.89793E-11	0.081661619	Potri.008G208200.1	AT4G15800.1
Potra2n267s35163.1	-0.447448182	-0.291741967	0.000869607	0.583631586	Potri.008G207700.2	AT3G16560.1
Potra2n267s35164.1	0.237512935	0.025814178	0.402823147	0.988601759	Potri.008G207500.1	AT4G15790.1
Potra2n267s35165.1					Potri.014G189100.1	AT2G01050.1
Potra2n267s35166.1	NA	3.641321801	NA	NA	Potri.011G161500.1	AT2G34790.1
Potra2n267s35167.1	5.561831849	2.484201661	3.87174E-05	NA	Potri.011G161400.1	AT1G30760.1
Potra2n267s35168.1	-0.28464817	2.999120448	0.834819726	0.449772714	Potri.011G161500.1	AT2G34790.1
Potra2n267s35169.1	4.275383012	0.619680655	5.01318E-12	0.948577865	Potri.011G161400.1	AT1G30760.1
Potra2n267s35170.1	8.707340807	1.363811008	4.13299E-12	NA	Potri.011G159600.1	AT1G30760.1
Potra2n268s35171.4	-1.22456578	-0.179844413	2.54817E-06	0.923348672	Potri.007G056400.1	AT2G17820.1
Potra2n270s35172.1	-0.927948047	0.008956972	0.00277464	0.995203254	Potri.009G155200.3	AT2G27170.1

Potra2n270s35173.1	NA	NA	NA	NA			
Potra2n270s35174.1	-1.339104484	-0.127638718	9.37818E-13	0.926697188	Potri.019G047200.4	AT5G16760.1
Potra2n272s35175.1	-1.80959137	-2.283435837	NA	0.597141087	Potri.011G085501.1	AT5G55570.1
Potra2n272s35176.1	-2.497337776	-2.549585638	0.203339015	0.586851629	Potri.011G085501.1	AT5G55570.1
Potra2n274s35177.1					Potri.016G129500.1	AT5G01740.1
Potra2n274s35178.1	NA	NA	NA	NA	Potri.016G129600.1	AT5G01740.1
Potra2n276s35179.1	-0.044818209	0.203538805	0.753976122	0.733263164	Potri.013G068400.10	AT3G54280.1
Potra2n276s35180.1	-1.35927387	-0.097655521	NA	NA	Potri.015G137800.2	AT5G05800.1
Potra2n276s35181.1						
Potra2n278s35182.1	0.602912703	-0.029524695	0.000106832	0.987226805	Potri.009G119300.1	AT5G11880.1
Potra2n278s35183.1	-0.142775709	0.267728582	0.706654124	0.912666272	Potri.009G119200.1	AT2G16370.1
Potra2n278s35184.1	3.641711699	1.325288224	5.14479E-75	0.105402179	Potri.T172000.1	AT3G50930.1
Potra2n278s35185.1	1.65191645	0.493932374	7.14247E-17	0.507784389	Potri.T172000.1	AT3G50930.1
Potra2n278s35186.1	-0.511296276	-1.494238734	0.616808506	0.531649858	Potri.009G119000.1	AT4G34560.1
Potra2n278s35187.1	0.534877251	0.029106576	0.001489038	0.986671181	Potri.004G157200.1	AT4G39200.1
Potra2n278s35188.1	-5.344795901	-0.669190465	2.38926E-16	0.759797728	Potri.009G118800.1	AT4G39210.1
Potra2n278s35189.1	-2.367361922	-2.514137983	NA	NA	Potri.009G118700.2	AT3G48530.1
Potra2n278s35190.1	0.190460675	0.511481971	0.540822708	0.727843918	Potri.009G118651.1	AT2G16365.2
Potra2n280s35191.1	0.744782164	0.279109957	1.98365E-07	0.773063484	Potri.009G018100.1	AT5G21160.1
Potra2n280s35192.1	3.3227211	-1.114199967	NA	NA	Potri.009G017900.1	AT1G08440.1
Potra2n280s35193.1	-0.299865963	-0.119070482	0.02441779	0.936549944	Potri.009G017700.1	AT2G27230.2
Potra2n282s35194.1					Potri.002G029800.1	AT1G09850.1
Potra2n284s35195.1	0.309313708	0.047328366	0.322802944	0.982827151	Potri.011G072566.1	AT1G29750.2
Potra2n286s35196.1	0.353026714	0.072200132	0.000696724	0.950878725	Potri.001G220100.1	AT5G21160.3
Potra2n287s35197.1	NA	NA	NA	NA	Potri.001G202900.1	AT1G80070.1
Potra2n287s35198.1	-0.596846192	-0.387495347	0.008123793	0.576374432	Potri.001G204000.1	AT3G15890.1
Potra2n287s35199.1	0.620264518	-0.276834947	0.009066843	0.802631112	Potri.001G204100.1	AT3G15820.1
Potra2n287s35200.1	0.102236182	-0.767708831	NA	NA	Potri.T007700.1	
Potra2n287s35201.1	0.229431664	-0.046320528	0.092469038	0.966407607	Potri.001G204200.1	AT1G66340.1
Potra2n287s35202.1	-1.061849892	-0.208702352	0.000155731	0.923475785	Potri.001G204300.2	AT3G15840.1
Potra2n287s35203.1	1.289540989	0.013643999	2.67795E-09	0.995268765	Potri.001G204400.1	AT1G80510.1
Potra2n287s35204.1	1.827540519	1.683123897	0.222332684	0.475175307	Potri.001G204500.1	AT4G18840.1

Potra2n287s35205.1	-0.735856726	-0.416503715	0.011735471	0.626455563	Potri.001G204600.1	AT1G15760.1
Potra2n287s35206.1	-0.025302059	0.19117779	0.912281829	0.89257034	Potri.001G204700.1	AT1G80530.1
Potra2n288s35207.1	1.553383153	-0.117607424	7.71443E-08	0.957872702	Potri.001G189500.1	AT1G59870.1
Potra2n288s35208.1	NA	NA	NA	NA	Potri.004G232250.1	AT5G49760.1
Potra2n288s35209.2	NA	NA	NA	NA	Potri.001G189600.1	
Potra2n288s35210.1	1.362030475	0.548185625	1.2397E-21	0.226817729	Potri.001G189700.5	AT1G79750.1
Potra2n288s35211.1	0.373515158	0.392344235	0.007515331	0.769643258	Potri.001G189800.1	AT3G16350.1
Potra2n288s35212.1	NA	NA	NA	NA		
Potra2n288s35213.1	NA	NA	NA	NA		
Potra2n288s35214.1	0.277444109	0.622132858	0.947082574	NA	Potri.015G118500.1	AT1G10180.1
Potra2n288s35215.1	2.618317767	3.892828506	0.424878883	NA	Potri.015G118500.1	AT1G10180.1
Potra2n288s35216.1	NA	NA	NA	NA	Potri.001G189900.2	AT3G16360.2
Potra2n290s35217.1	4.975041524	NA	0.005212472	NA	Potri.007G141900.2	AT2G31750.1
Potra2n290s35218.1	5.385625887	2.211102242	1.64949E-16	0.566863304		
Potra2n290s35219.1	6.589496281	2.302111319	1.85892E-15	0.411601435	Potri.007G141000.3	AT5G38280.1
Potra2n290s35220.1	0.156114591	0.586757891	NA	NA	Potri.007G140900.1	AT1G66910.1
Potra2n290s35221.1	-1.328951947	-1.478536539	NA	NA	Potri.007G140300.1	AT1G05680.1
Potra2n290s35222.1	0.237838373	-0.889711674	0.927567758	NA	Potri.007G117200.1	AT2G43840.2
Potra2n293s35223.1	-0.247735736	-0.085024758	0.508947021	0.964333742	Potri.017G131700.1	AT3G19940.1
Potra2n293s35224.1	0.638305421	0.431384728	0.440095961	0.925035363		
Potra2n293s35225.1	-0.780299078	-0.047501913	0.033125603	0.985016122	Potri.017G131800.1	AT1G66180.1
Potra2n293s35226.1	0.678851754	0.166240976	0.000965973	0.930610374	Potri.017G131900.1	AT5G16940.2
Potra2n293s35227.1	1.70380896	0.289187239	2.68474E-18	0.880553952	Potri.017G132000.1	AT5G16940.2
Potra2n293s35228.1	1.980858192	0.435494184	4.30695E-20	0.811159921	Potri.017G132000.1	AT5G16940.2
Potra2n293s35229.1	2.431399976	0.620379218	1.61789E-09	0.849790859	Potri.017G132100.1	AT5G36930.2
Potra2n293s35230.1	0.738240578	0.265362599	0.004729586	0.831463341	Potri.017G133000.1	AT1G11050.1
Potra2n293s35231.1	-0.03368653	-0.851879591	0.969067024	0.590365286		
Potra2n293s35232.1	0.229513209	-0.028686078	0.1717377	0.986636967	Potri.017G133000.1	AT1G11050.1
Potra2n293s35233.1	0.08496006	-0.091799098	0.585160416	0.914162682	Potri.017G133000.1	AT1G11050.1
Potra2n293s35234.1	-0.025389921	-0.070073658	0.910242475	0.956865545	Potri.017G133200.1	AT3G59990.3
Potra2n295s35235.1	1.459962178	NA	NA	NA	Potri.001G193800.1	AT2G01050.1
Potra2n296s35236.1	4.323949398	1.698378683	0.000397541	NA		

Potra2n296s35237.1	2.685015931	NA	NA	NA	Potri.T103000.2	AT4G08850.1
Potra2n296s35238.1	NA	1.571529889	NA	NA	Potri.002G008200.1	AT4G08850.2
Potra2n296s35239.1	3.580290532	0.64247635	0.056932998	NA	Potri.T102800.1	AT4G08850.1
Potra2n296s35240.1	6.23615918	0.674963689	3.57852E-05	NA	Potri.T103200.2	AT4G08850.1
Potra2n296s35241.1	5.818403505	NA	0.003145259	NA	Potri.T102800.1	AT4G08850.1
Potra2n296s35242.1	0.091350711	0.109924485	0.839765464	0.966407607	Potri.002G008600.1	AT1G20760.1
Potra2n296s35243.1	5.984250147	3.787800616	1.88873E-05	NA	Potri.T103000.2	AT4G08850.1
Potra2n296s35244.1	0.122124769	0.05642756	0.797724392	0.985393296	Potri.002G008600.1	AT1G20760.1
Potra2n297s35245.1	1.651706702	NA	NA	NA	Potri.019G002601.1	AT5G36930.2
Potra2n297s35246.1	1.600455502	-0.059941009	2.42681E-09	0.977215798	Potri.019G002500.1	AT5G17680.1
Potra2n297s35247.1	1.713803697	0.05599351	0.200608455	0.996032145	Potri.001G307300.1	AT5G17680.1
Potra2n298s35248.1	-0.873676548	-0.282684531	5.68935E-05	0.794641467	Potri.001G227000.2	AT2G44500.1
Potra2n298s35249.1	-0.723872575	-0.837104146	0.095541548	0.319291077	Potri.009G022400.1	AT3G45140.1
Potra2n299s35250.1	3.004903989	0.677855795	0.03112474	NA	Potri.008G131100.1	AT1G70890.1
Potra2n299s35251.1	0.377824947	1.868982257	0.528916617	0.291399558	Potri.010G111100.1	
Potra2n299s35252.1					Potri.008G130800.1	AT2G01505.1
Potra2n299s35253.1	1.81315497	-0.496657496	NA	0.554040943	Potri.008G130700.1	AT1G23110.1
Potra2n299s35254.1	2.25843811	-1.383488146	NA	NA	Potri.010G111400.1	AT3G18010.1
Potra2n299s35255.1	0.037421403	0.12694752	0.836838235	0.88661378	Potri.008G130600.1	AT2G01480.1
Potra2n299s35256.1	0.236302356	0.047899515	0.150899061	0.97726831	Potri.008G130500.1	AT1G23100.1
Potra2n299s35257.1	1.664677056	1.871975723	NA	NA		
Potra2n299s35258.1	-0.599634483	0.599225939	0.022167954	0.694477853	Potri.008G130400.1	AT1G23090.1
Potra2n299s35259.1	0.089559862	-0.137608922	0.541904038	0.88221216	Potri.008G130300.1	AT2G01470.1
Potra2n299s35260.1	-0.008270239	0.188936532	0.97123367	0.849041117	Potri.008G130200.3	AT2G01460.1
Potra2n2c4022.1	NA	NA	NA	NA	Potri.010G138600.1	AT1G69040.2
Potra2n2c4023.1					Potri.008G001900.1	AT2G01050.1
Potra2n2c4024.1	0.069791939	-0.0070914	0.826363381	0.99819408	Potri.015G000100.1	AT4G27870.1
Potra2n2c4025.1	-1.102017172	-0.160807293	1.47746E-13	0.885459527	Potri.002G263800.1	AT1G75130.1
Potra2n2c4026.1					Potri.019G052000.1	AT5G36930.1
Potra2n2c4027.1	NA	NA	NA	NA	Potri.019G023000.1	AT3G25830.1
Potra2n2c4028.1	NA	NA	NA	NA		
Potra2n2c4029.1					Potri.002G263700.1	

Potra2n2c4030.1	-0.679014337	0.243170493	0.004134457	0.900551258	Potri.002G263600.1	AT3G50700.1
Potra2n2c4031.1	NA	1.571529889	NA	NA		
Potra2n2c4032.1	-0.731734949	-0.063699811	NA	NA	Potri.002G263300.1	AT2G30942.1
Potra2n2c4033.1	-0.091152091	0.041722487	0.395777094	0.956865545	Potri.002G263200.1	AT5G42970.1
Potra2n2c4034.1	-0.004987007	0.10057424	0.983288653	0.927547456	Potri.002G263100.2	AT2G30942.1
Potra2n2c4035.1	-0.35328882	0.110082097	0.009722489	0.909675463	Potri.002G263000.2	AT1G45160.2
Potra2n2c4036.1	0.253235023	-0.058730045	0.128922637	0.95999889	Potri.002G262900.1	AT1G75140.1
Potra2n2c4037.1	0.113126193	-0.257830975	0.678924547	0.824448251	Potri.002G262800.1	AT1G75150.1
Potra2n2c4038.1	0.395081502	0.014934438	0.017790934	0.993630002	Potri.002G262700.1	AT1G45170.1
Potra2n2c4039.1	-0.587857158	-0.620674352	0.755597154	0.940449094	Potri.014G181200.1	AT3G56870.1
Potra2n2c4040.1	NA	1.363811008	NA	NA	Potri.002G262500.1	AT5G19650.1
Potra2n2c4041.2	0.521728367	0.271086269	0.257713244	0.932097833	Potri.002G262400.1	AT1G75160.1
Potra2n2c4042.1	0.183924499	0.144329293	0.201360818	0.880553952	Potri.002G262300.2	AT5G42950.1
Potra2n2c4043.1	-0.20583889	-0.426766586	0.604658292	0.643558659	Potri.002G261900.1	AT3G46940.1
Potra2n2c4044.1	1.712397949	0.483829321	0.035094171	0.936890993	Potri.002G261800.1	AT2G16230.1
Potra2n2c4045.1	-0.256190391	0.055292972	0.267341958	0.976632015	Potri.002G261700.1	AT5G42710.1
Potra2n2c4046.1	-0.071530915	0.154397556	0.652177664	0.849063549	Potri.002G261500.1	
Potra2n2c4047.1	0.742522824	0.858064228	2.7433E-05	0.125665612		
Potra2n2c4048.2	1.851377061	0.617815292	4.92407E-13	0.375012853	Potri.002G261000.1	AT1G75170.1
Potra2n2c4049.1	-0.077236608	0.37385625	0.804617162	0.819608939	Potri.002G260900.1	AT1G75180.1
[truncated: 437,787 more chars]
